# Plasma Oxylipins and Their Precursors Are Strongly Associated with COVID-19 Severity and with Immune Response Markers

**DOI:** 10.3390/metabo12070619

**Published:** 2022-07-04

**Authors:** Naama Karu, Alida Kindt, Lieke Lamont, Adriaan J. van Gammeren, Anton A. M. Ermens, Amy C. Harms, Lutzen Portengen, Roel C. H. Vermeulen, Willem A. Dik, Anton W. Langerak, Vincent H. J. van der Velden, Thomas Hankemeier

**Affiliations:** 1 Metabolomics and Analytics Centre, Leiden Academic Centre for Drug Research, Leiden University, 2333 CC Leiden, The Netherlands; a.s.d.kindt@lacdr.leidenuniv.nl (A.K.); l.lamont@lacdr.leidenuniv.nl (L.L.); a.c.harms@lacdr.leidenuniv.nl (A.C.H.); 2 Department of Clinical Chemistry and Hematology, Amphia Hospital, 4818 CK Breda, The Netherlands; avangammeren@amphia.nl (A.J.v.G.); aamermens@gmail.com (A.A.M.E.); 3 Department of Population Health Sciences, Institute for Risk Assessment Sciences, University Utrecht, 3584 CK Utrecht, The Netherlands; l.portengen@uu.nl (L.P.); r.c.h.vermeulen@uu.nl (R.C.H.V.); 4 Laboratory Medical Immunology, Department of Immunology, Erasmus MC University Medical Center Rotterdam, 3015 GD Rotterdam, The Netherlands; w.dik@erasmusmc.nl (W.A.D.); a.langerak@erasmusmc.nl (A.W.L.); v.h.j.vandervelden@erasmusmc.nl (V.H.J.v.d.V.)

**Keywords:** SARS-CoV-2, COVID-19, lipid, eicosanoid, oxylipin, metabolomics, cytokine, inflammation

## Abstract

COVID-19 is characterised by a dysregulated immune response, that involves signalling lipids acting as mediators of the inflammatory process along the innate and adaptive phases. To promote understanding of the disease biochemistry and provide targets for intervention, we applied a range of LC-MS platforms to analyse over 100 plasma samples from patients with varying COVID-19 severity and with detailed clinical information on inflammatory responses (>30 immune markers). The second publication in a series reports the results of quantitative LC-MS/MS profiling of 63 small lipids including oxylipins, free fatty acids, and endocannabinoids. Compared to samples taken from ward patients, intensive care unit (ICU) patients had 2–4-fold lower levels of arachidonic acid (AA) and its cyclooxygenase-derived prostanoids, as well as lipoxygenase derivatives, exhibiting negative correlations with inflammation markers. The same derivatives showed 2–5-fold increases in recovering ward patients, in paired comparison to early hospitalisation. In contrast, ICU patients showed elevated levels of oxylipins derived from poly-unsaturated fatty acids (PUFA) by non-enzymatic peroxidation or activity of soluble epoxide hydrolase (sEH), and these oxylipins positively correlated with markers of macrophage activation. The deficiency in AA enzymatic products and the lack of elevated intermediates of pro-resolving mediating lipids may result from the preference of alternative metabolic conversions rather than diminished stores of PUFA precursors. Supporting this, ICU patients showed 2-to-11-fold higher levels of linoleic acid (LA) and the corresponding fatty acyl glycerols of AA and LA, all strongly correlated with multiple markers of excessive immune response. Our results suggest that the altered oxylipin metabolism disrupts the expected shift from innate immune response to resolution of inflammation.

## 1. Introduction

The COVID-19 pandemic raised an urgent need to characterise the SARS-CoV-2 pathogenicity and host response. A persistent and excessive innate immune response contributes to COVID-19 severity. Various lipids, some acting as immune modulators, are dysregulated along the course of the disease [1,2,3,4,5,6], yet obtaining an exact metabolic picture of small lipids involvement is still work in progress [5,7,8,9,10]. Low molecular weight lipids (<500 Da) play diverse biochemical roles, as they are embedded in cell membranes and take part in cell signalling, energy production and storage, among other endogenous processes. Lipid perturbations measured in the host can reflect endogenous processes as well as metabolic remodelling by coronaviruses [11], including SARS-CoV-2 [12]. Both the host and the virus can alter the expression and activity of key enzymes in lipid synthesis and metabolism [1,2,13,14,15]. For example, as part of the host innate immune response, pro-inflammatory cytokines upregulate phospholipase A2 (PLA2) to release long chain fatty acids from glycerophospholipids in the cell membrane. PLA2 upregulation is also induced by coronaviruses to support their propagation via the formation of double-membrane vesicles [11,13,15,16]. Likewise, the metabolism of polyunsaturated fatty acids (PUFA) was modulated by induced expression of cyclooxygenase-2 (COX-2), in coronavirus infected cells and in animal models [17,18]. There is a large group of inflammation mediating oxylipins which are metabolised from PUFA by various COX, LOX (lipoxygenases), CYP (cytochrome P-450), epoxygenases and hydroxylases, or non-enzymatic peroxidation [14,19]. The prostanoids produced from arachidonic acid (AA) by COX and the hydroxyoctadecadienoic acids (HODEs) derived from linoleic acid (LA) exhibit dual inflammatory mediating activity, depending on the phase of the immune response, the producing cells, and the activated receptors. In the initial phases of the innate immune response, the above metabolites induce the synthesis of pro-inflammatory cytokines and chemokines [20], acting alongside the pro-inflammatory leukotrienes derived from AA by LOX, which induce bronchoconstriction, production of mucus, and increase vascular permeability. Later in the immune response, HODEs [20,21,22], PGD2 [23,24], and PGE2 [25,26] can have anti-inflammatory effects, especially in the lungs [27]. In comparison, a consistent anti-inflammatory activity is attributed to AA-derived lipoxins, and to protectins, resolvins, and maresins produced from the omega-3 PUFAs eicosapentaenoic acid (EPA) and docosahexaenoic acid (DHA). These specialised pro-resolving mediators (SPM) suppress microbial proliferation, inhibit inflammation, reduce organ fibrosis, and promote wound healing [6,9,28,29,30].

To try and elucidate the dynamic role of signalling lipids in COVID-19, the presented study focused on circulating free fatty acids, oxylipins and their intermediates, and the immuno-active endocannabinoids that can serve as precursors for free fatty acids and oxylipins. Overall, 63 signalling lipids were analysed by targeted metabolomics in 103 plasma samples taken in March–April 2020 from COVID-19 patients at varying disease states. The quantified metabolites underwent differential analysis based on disease severity, and were also correlated with over 30 immune response markers available from the same cohort [31]. The results of the current study can direct future research into the biochemistry of COVID-19 progression.

## 2. Results

### 2.1. Unsupervised Multivariate Analysis

**Figure 1:**
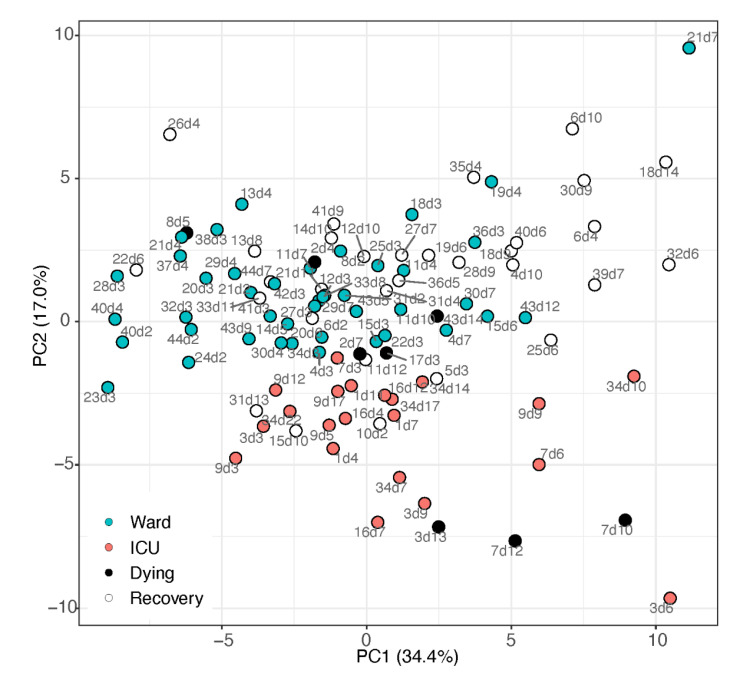
PCA scores plot of samples from patients admitted to ward (blue markers) or ICU (red markers), based on all metabolite data (cube-root transformed and Pareto-scaled). Data points of samples taken within a day of release from hospital (“recovery”) are depicted by open circles, and samples taken within 4 days of death are in black. Each data point is tagged with the patient ID and sample day (corresponding with Table S2). ICU patients #7 and #17 appeared close to the ward patients, both on day 3 when transferred from ward to ICU due to health deterioration.

The signalling lipids profile was obtained from the COVID-19 patient cohort summarised in Table 1. Utilising all 63 lipids that passed the quality control process, principal component analysis (PCA, Figure 1) demonstrated a separation between samples taken from patients in the ward (suffering from pneumonia) and patients in the ICU (suffering from ARDS and other complications). The loadings of the PCA (Table S7; Figure S1) show that the ICU sample cluster was directed by elevated levels of the free fatty acids alpha-linolenic acid (aLA; omega-3) and LA, with oxidation products of LA, and three fatty acylglycerol esters (endocannabinoids). In contrast, the ward cluster had elevated levels of AA and its metabolites. These observations indicate interesting metabolic findings, and are further explored via univariate analysis.

### 2.2. Signalling Lipids Associated with Severe COVID-19

**Figure 2:**
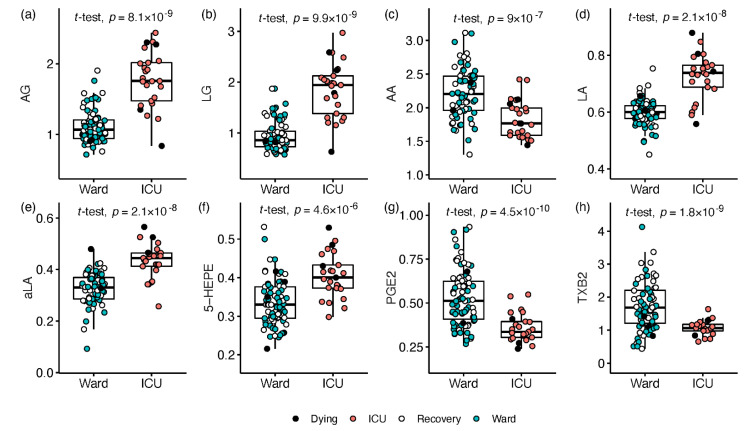
Box and whisker and scatter plots of metabolites differentiating between hospitalisation status: ICU (red) vs. ward (blue; open markers for recovering patients within 24 h of release). Black markers represent patients who died within 4 days. Prior to plotting, metabolite peak area ratios with internal standards were cuberoot-transformed. Metabolites: (**a**) arachidonoyl glycerol (sum of AG isomers); (**b**) linoleyl glycerol (sum of LG isomers); (**c**) arachidonic acid (AA); (**d**) linoleic acid (LA); (**e**) alpha-linolenic acid (aLA); (**f**) 5-hydroxy EPA (5-HEPE); (**g**) prostaglandin E2 (PGE2); (**h**) thromboxane B2 (TXB2). All results are in Table S8.

Disease severity at time of sampling was defined as the hospitalisation status (ICU or ward), and univariate regression analysis was conducted utilising all 103 plasma samples, adjusting for age, sex, BMI, and count of samples per patient. Of the 63 measured signalling lipids, ICU patients had elevated levels of 22 metabolites compared with ward patients, while 12 metabolites were decreased (≥20% median fold change, and Q < 0.05; Table S8; Document S2). The ICU-increased lipids were led by strong increase (2.5–11) fold in the endocannabinoids arachidonoyl-, linoleoyl-, and oleoyl-glycerol esters (AG, LG, and OG, respectively; Figure 2a,b). Moderate 25–60% increases in ICU patients were recorded for other endocannabinoids, such as the ethanolamides of DHA (DHEA), LA (LEA), and aLA (aLEA). Four of the eight measured free fatty acids changed significantly in ICU. AA decreased by almost 2-fold in ICU (Figure 2c), and ~2-fold increases were recorded for LA and aLA (Figure 2d,e), while five of their derivatives showed smaller increases (Table S8). The omega-3 EPA showed a modest increase together with five derivatives (1.5–2 FC; see 5-HEPE in Figure 2f). Five prostanoids derived by COX activity on AA, showed the most noticeable decrease in ICU patients compared with ward (1.8–4 FC; see PGE2 and TXB2 in Figure 2g,h), while decreases of 30–70% were observed in four CYP and LOX derivatives of AA (HETEs). Conversely, the only ICU-elevated AA derivative was a non-enzymatic peroxidation product (8,12-iPF2a IV; 1.6 FC).

### 2.3. Paired Analysis in Non-Critical Patients

**Figure 3:**
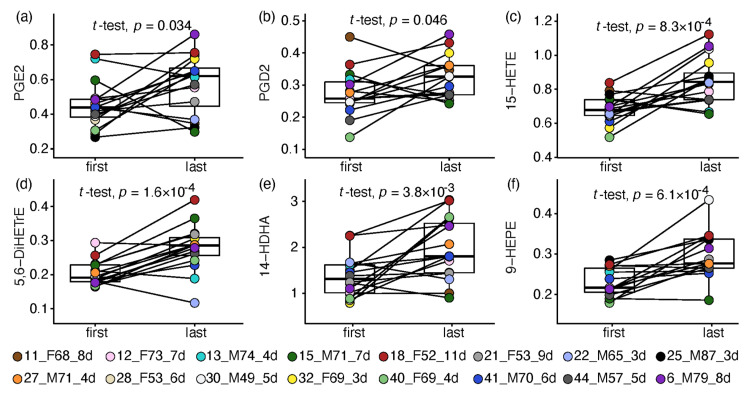
Box and whisker and scatter plots of paired changes of metabolite levels in COVID-19 ward patients. A line connects each patient’s paired samples, with the first time point being not more than 4 days from admission, and last time point during the 24 h before release from hospital. Metabolite peak area ratios with internal standards were cuberoot-transformed. Metabolites: (**a**) PGE2; (**b**) PGD2; (**c**) 15-HETE; (**d**) 5,6-diHETrE; (**e**) 14-HDHA; (**f**) 9-HEPE. The legend shows the individual patient by marker colour, with indication of patient number, sex, age, and number of days between time points. Patient information is provided in Table S2. FDR-corrected paired *t*-tests, gender differences and fold changes are provided in Table S9.

Next, the metabolic changes during recovery from COVID-19 were assessed in 16 ward patients who complied with the following criteria. The patient-paired analysis compared one sample taken at the start of hospitalisation (days 1–4 since admission) and one at the recovery stage (up to a day before release from the hospital) with no less than 3 days in between. Significant results were obtained for 41 signalling lipids, all increased towards the patients’ recovery, with a median magnitude ranging from 20% to 5-fold (*n* = 27 with Q < 0.05). Owing to the small sample size and the impact of within-group variance (especially in male patients), most of the significant changes were statistically driven by females, and 10 alterations were observed only in females. Resonating with the strong reduction in prostanoids in ICU patients, the same COX-derived AA metabolites were increased by 2–5 fold towards recovery of ward patients (see PGE2 and PGD2 in Figure 3a,b), yet without change in their precursor AA. AA and LA metabolites derived by LOX and CYP activity followed suit, with increases between 30% and 3-fold (e.g., Figure 3c,d). Omega-3 fatty acid metabolism was also altered, exhibiting a consistent increase (1.5–2.5 FC) in eight hydroxy-DHAs (HDHA; e.g., Figure 3e), while four EPA derivatives increased by 1.4–2.1 fold (e.g., Figure 3f). Seven ethanolamides increased towards recovery by 1.5–2 fold; however, most were significant in females only. 

### 2.4. Correlation between Metabolites and Immune Response Markers

**Figure 4:**
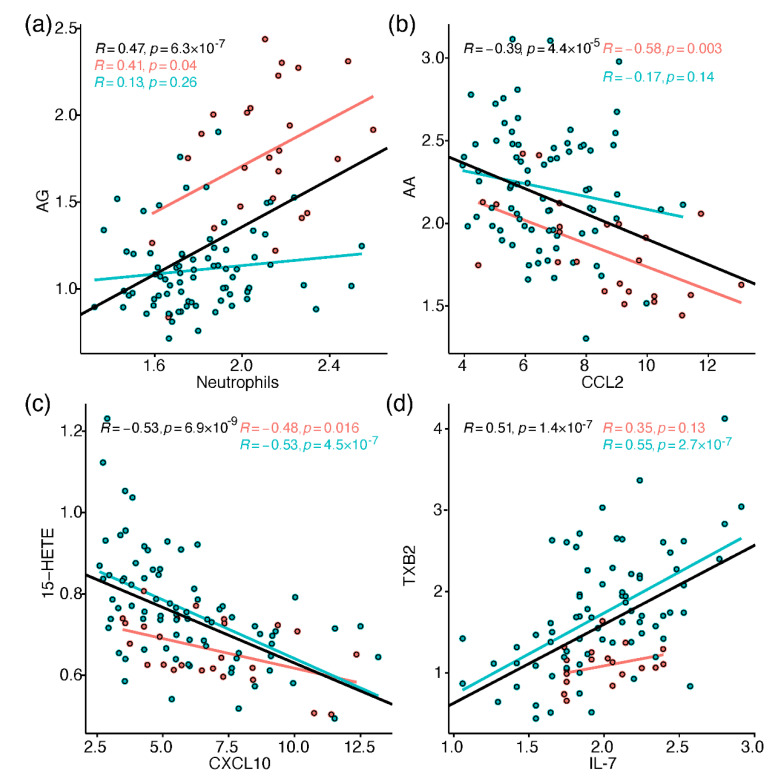
Selected Pearson correlation scatter plots between metabolites and immune response markers (cuberoot-transformed). Red markers are samples from ICU patients, and blue markers are from ward patients. The regression lines and Pearson R values and *p* values (uncorrected) are in black for all samples, red for ICU, and blue for ward. Metabolites: (**a**) AG vs. neutrophils; (**b**) AA vs. CCL2; (**c**) 15-HETE vs. CXCL10; (**d**) TXB2 vs. IL-7. The full correlation results are in Tables S10–S12 and Document S4.

To link the metabolic perturbations to relevant immune processes, Pearson correlations were calculated between all metabolites and 37 immune response markers, including different leukocytes, chemokines, cytokines, and others (heatmap in Figure S2). These results were incorporated in the biochemical discussion section, and some interesting correlations were individually plotted in Figure 4. Within the limitations of the sample size, many correlations had similar trend between ICU and ward samples, while some correlations were found only in one group, or showed reversed trends. To follow up such cases, separate correlation networks were produced for ICU and ward samples, as demonstrated for arachidonic acid metabolism in Figure S3. The immune markers that showed the most consistent correlations with multiple signalling lipids (|R| > 0.3; FDR Q < 0.05) included leukocytes (neutrophils; T-cells); the pro-inflammatory cytokines TNF-alpha, IL-6, GMCSF (promotes differentiation of granulocytes), IL-7, IL-8, and IL-18; macrophage-activation markers (soluble (s) CD206 and CD163); the immune cell-attracting chemokines CCL2 (MCP1), CXCL10 (IP10), and CCL17; the IL-6 receptor alpha (IL-6Ra); the acute phase protein C-reactive protein (CRP); and ferritin.

## 3. Discussion

**Figure 5:**
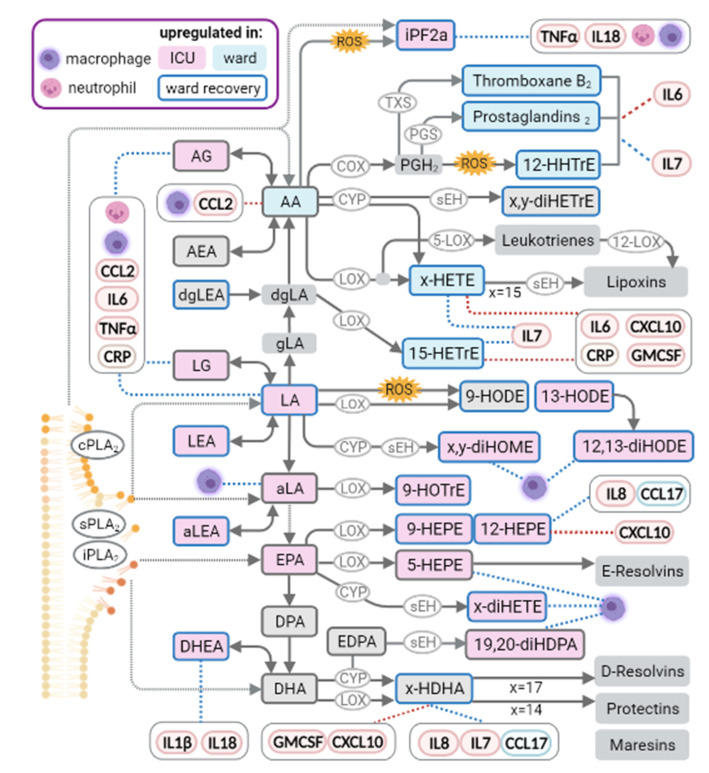
Biochemical pathway map incorporating differential analysis of metabolites (in boxes), combining the results of ICU vs. ward analysis (background colour) with the paired analysis of ward patients’ recovery status (frame colour). Correlation of metabolites with immune parameters (in ovals) is indicated by blue (positive) or red (negative) dashed lines. In a metabolite name, x and y denote various isomers, as depicted in the results of the study (see supplementary tables for complete results). Enzymes are in ovals over reaction arrows. Acetylated COX acts similarly to the corresponding LOX. COX, cyclooxygenase; cPLA2, cytosolic calcium-dependent PLA2 (high selectivity to AA-containing phospholipids); iPF2a, 8,12-iPF2-alpha IV; iPLA2, cytosolic calcium-independent PLA2; PGS, prostaglandin synthases; sEH, soluble epoxide hydrolase; sPLA2, secretory PLA2; TXS, thromboxane synthases. The figure was created with BioRender.com.

The main findings of the study are summarised in Figure 5, illustrating the main metabolic perturbations along the pathways from endocannabinoids (left column) via free fatty acids (middle) to oxylipins (right), as well as highlighting associations with immune parameters.

### 3.1. Endocannabinoids

Beyond their role as CNS modulators, endocannabinoids are peripheral immune mediators, with anti- or pro-inflammatory activity, depending on their chemistry, the cell type (immune cells [32,33]; endothelial cells [34]), and receptor binding (CB_1_; CB_2_; etc.) [35,36]. For example, in some immune cells, the activation of CB2 receptors by the binding of 2-AG and AEA [37,38] can decrease cytokines production, reduce the production and mobilisation of neutrophils and M1 macrophages, and subsequently lead to lower levels of ROS [39]. In our study, samples from ICU patients showed elevated levels of 6 out of 11 detected endocannabinoids, without any specific preference for fatty acyl chain length or double bonds. The acylglycerols AG, LG and OG exhibited the most dramatic increases in ICU (>4-fold, compared to mild increases in acylethanolamides), without change towards recovery in ward patients. Mainly owing to the high levels in ICU patients, these acylglycerols positively correlated with various markers of hyper-inflammation (see AG vs. neutrophils in Figure 4a), and proposed ratio markers of COVID-19, namely neutrophil/leukocyte ratio and CD4/CD8 T-cell ratio. As illustrated in Figure S3, some correlations of acylglycerols were dissimilar between ICU and ward, including the weak inverse association with CRP, and the ward-unique correlation with IL-6 receptor-alpha (produced by CD4 T cells [40] as part of the adaptive immune response). The gathered observations led us to hypothesise that ICU patients exhibited higher levels of endocannabinoids due to accelerated catabolism of lipid precursors in various tissues (which react differently to inflammation), to meet an increased demand for free fatty acids and oxylipin synthesis [38,41,42]. Due to the indiscriminate elevation in endocannabinoids in ICU patients, we found it less plausible that it reflected specific activity to promote inflammation resolution. A shift towards endocannabinoid synthesis from free fatty acids can occur without medication [27] or via treatment with corticosteroids (that inhibit PLA2 and COX) or NSAIDs (inhibit COX) [18,43,44], both scarcely applied in this cohort. This hypothesis is the basis to the proposed treatment of COVID-19 patients with endocannabinoid agonists (AEA; 2-AG), to divert the immune response towards recovery [37].

### 3.2. Free Fatty Acids

Perturbations in circulating fatty acids are harder to interpret partly due to the multifaceted processes they are involved in. Apart from the bidirectional conversion to acylethanolamides and acylglycerolesters [41] and the role of their derivatives as signalling molecules, long-chain fatty acids are utilised as energy source via mitochondrial beta-oxidation, which is impaired by SARS-CoV-2 infection [45]. We recorded approximately double the levels of LA and aLA in patients in the ICU compared to ward (Figure 5). In a similar manner to the elevated endocannabinoids, the high levels in ICU patients led to correlations between LA and markers of excessive innate immune response: TNF-alpha, CRP, neutrophils, and markers of macrophage activation. COVID-19 severity was associated with several free fatty acids such as LA and aLA [46], and the increases in LA were related to elevated sPLA2 enzymes [16]. Sourcing PUFA from immune cell membrane phospholipids through cleavage by various PLA2 enzymes is common during infection and inflammation, and can be induced by coronaviruses [2,11,13,15]. In contrast, PLA2 enzymes are non-selectively inhibited by chloroquine [47] (received by 80% of cohort patients) and corticosteroids (only 5% of patients). Our results could suggest different degree of alterations in various PLA2, specifically in cPLA2 that has higher affinity to AA-containing phospholipids. Unlike LA, ICU patients showed the opposite results for AA, with decreases of ~2-fold compared to ward patients, leading to negative correlations with activation markers of leukocytes and macrophages (Figure 4b). Decreased levels of AA were reported in severe COVID-19 patients [48] and also in hospitalised COVID-19 patients compared to recovered (with ongoing lipid dysregulation) [49]. AA deficiency was suggested to contribute to COVID-19 severity, due to interruption in immune modulation by its derivatives (discussed next), and also owing to a proposed antiviral activity of AA [14,28,50].

### 3.3. Arachidonic Acid Derived Oxylipins

The health deterioration of people with COVID-19 is commonly characterised by hypercytokinaemia accompanied by an eicosanoid storm. However, we could not see evidence for a “classic” eicosanoid storm (AA-derivatives) along the hospitalisation period. The only AA derivative that increased in ICU patients was the isoprostane 8,12-iPF2a IV, a product of non-enzymatic AA peroxidation induced by ROS activity [14,19]. The isoprostane can be also produced from peroxidation of esterified AA within the cell membrane, and then be released by PLA2 [51]. We found a strong correlation between the isoprostane, TNF-alpha, and macrophage activation markers. Several isoprostanoids were elevated in COVID-19 ICU patients in another study [5], potentially indicating the oxidative stress that accompanies inflammation in critically ill patients, especially those who suffer ARDS [52]. ROS dysregulation can further contribute to endothelial dysfunction, since redox reactions affect cell adhesion, platelet aggregation, vasoconstriction, inflammatory gene expression, and more [53]. It is likely that enhanced ROS activity affects the overall profile of oxylipins, occurring on the account of controlled enzymatic conversions of the substrates.

Consistently along the hospitalisation period, ICU patients showed a strong reduction in AA oxylipins (excluding diHETrEs), while most oxylipins exhibited dramatic increases towards recovery of ward patients. The prostanoids, five of which led the oxylipin changes in ICU patients, negatively correlated with IL-6. HETEs negatively correlated with markers of acute immune response, apart from the terminal 20-HETE (see 15-HETE in Figure 4c and Figure S3). The elevated HETEs and three prostanoids also correlated with IL-7 (e.g., TXB2 in Figure 4d), possibly reflecting increased blood cell count. COVID-19 studies found that compared to patients in the ward, ICU patients had lower levels of the same prostanoids as we recorded [7], or of PGE2, PGD2, and several HETEs [5]. In comparable conditions, ICU sepsis non-survivors showed reduced PGE2, PGD2, and TXB2 compared to survivors [54]. The association of lower metabolite levels with worse health status seem to contradict the detrimental effects of AA derivatives, such as vascular leakage by prostanoids, platelet adhesion in endothelial cells by HETEs [55], and activation of the Leukotriene B4 receptor 2 (BLT2) by the potent 12-HHTrE. COVID-19 severity was also hypothesised to be linked to specific CYP over-production of mid-chain and terminal HETEs that were considered pro-inflammatory [56]. Nevertheless, the results we gathered did not reflect these activities of AA oxylipins, perhaps due to the discrepancy between circulating levels and lung metabolism, or the study design (studies reported high levels mainly compared to healthy controls, or measured in serum and not plasma). More likely, we observed a deficiency in AA-derived immune mediators, impeding the normal course of immune response. In animal models of inflammation, during the acute response phase a decrease was recorded in prostaglandin synthases [23] and a variety of AA eicosanoids [57], followed by an increase during the resolution phase. Therefore, we could expect that in our study, lower levels of prostanoids may hinder a shift from production of pro-inflammatory M1 macrophages towards the anti-inflammatory M2 macrophages, facilitated by prostanoids [6]. In addition, lower HETEs levels could hamper their conversion into the pro-resolving lipoxins [30,42]. Altered levels of oxylipins (not only AA-derived) may also mirror reduced activity of specific enzymes, perhaps due to dysfunctional immune cells, which were reported in COVID-19 patients [58]. Although the metabolic picture in plasma is far more complex, this suggestion is based on the specialised enzymatic activity in isolated immune cells (e.g., 15-LOX, COX-2 primarily expressed in endothelial cells, 5-LOX in neutrophils, and 12-LOX in platelets) [27]. While we observed mostly class-related alterations, a study of serum lipidome in COVID-19 patients reported similarity between metabolites produced by the same enzyme across the pathways [7].

### 3.4. Linoleic Acid Derivatives

Of the seven detected LA derivatives, five were elevated in ICU patients compared to ward, including 2–5-fold increases in 12,13-diHOME, 9,10-diHOME, and 12,13-diHODE. Compared to healthy controls, a small pilot study recorded increases in COVID-19 patients in the same diHOMEs and their epoxy intermediates [59]. The diHOMEs are termed “leukotoxin-diols” and considered mitochondrial toxins that induce vascular leakage and associated with ARDS [60]. We found correlations between the diHOMEs and diHODE with markers of macrophage activation. This possibly reflects the severe inflammation in ICU patients, accompanied by increased activity of ROS and sEH (soluble epoxide hydrolase) that can metabolise the abundant LA. While various LA derivatives increased towards recovery of ward patients, the highest increases were recorded in 9- and 13-HODE that are produced via enzymatic or non-enzymatic peroxidation of LA. Although HODEs can display cellular pro-inflammatory activity [20,61], like other oxylipins, they act as agonists of the transcription factor PPAR (peroxisome proliferator-activated receptor), which promotes the resolution phase of inflammation [20,22,62,63]. Moreover, 13-HODE inhibits platelet adhesion [55], while both 13-HODE and 9-HODE promote apoptosis and clearance of macrophages [20]. Our results for ward patients may agree with a pro-resolving activity, also due to weak positive correlations with IL-6Ra, and negative correlations with CXCL10 in ward patients (0.3 ≤ |R| ≤ 0.4).

### 3.5. Oxylipins Derived from Omega-3 Fatty Acids

Figure 5 concisely illustrates the metabolism of omega-3 fatty acids into SPMs and their intermediates, all expected to be rapidly recruited to inflammation cites to promote resolution and prevent tissue damage [6,30,64]. This recruitment, alongside high oxidative stress, may explain the elevated aLA, EPA, and peroxidation derivatives in the plasma of ICU patients. In contrast, neither DHA nor its HDHA derivatives, which are precursors of many SPMs, were higher in ICU patients. Other COVID-19 studies reported severity-dependent downregulation of DHA-derived SPMs [5,9], together with a shift in the expression of LOX enzymes [9]. In contrast, SPMs increased in the serum of COVID-19 patients compared to healthy controls [10]. Possibly reflecting pro-resolving role, we observed multiple increases in HDHAs towards recovery of ward patients, as well as negative correlations with CXCL10 and GMCSF. Moreover, this class of oxylipins was the only one to consistently exhibit positive correlations with the chemokine CCL17, a T cell development inducer (part of the adaptive immune response) that is downregulated in COVID-19 [65]. As we described for AA derivatives, the DHA metabolism results also suggest disruption in the production of pro-resolving signalling lipids in severe COVID-19.

### 3.6. Additional Aspects and Study Limitations

When interpreting any metabolic perturbations and specifically those of small lipids, it is important to consider that people who are predisposed to develop severe COVID-19 may already be suffering from dysregulation of lipid metabolism due to underlying health conditions [66,67]. Moreover, medication prescribed for those conditions can alter the baseline lipid profile, adding to the existing high impact of population-varying dietary fats and gut microbiota composition. Host–microbe co-metabolism affects the ingested, absorbed, and transformed lipids (such as endocannabinoids), while microbes also alter the expression of related receptors and enzymes, with further implications on the host immune signalling [68]. Dietary supplementation of omega-3 PUFA was proposed as a treatment of COVID-19, and showed a potential in improving survival rates and several parameters of lung function [69,70,71]. The rationale behind this approach included the increased production of SPMs, and also a reduction in omega-6/omega-3 ratio in cell membranes, to lower prostanoids levels and decrease viral replication [69,72,73,74]. Although an eicosanoid storm can be expected early in the immune response, the goal of lowering the omega-6 PUFA products does not take into account the shift in their action along the inflammation process and their importance in promoting resolution. The latter seemed to characterise ICU patients compared to ward patients; however, a non-disease-specific bias is introduced through the ICU treatment, due to the special feeding regimens and the application of strong antibiotics that diminish the gut bacteria. Nevertheless, strong overall correlations between lipids and immune markers suggest a true disease-related context rather than an ICU bias. To isolate these effects, it is advised to recruit a control group of ICU patients who do not have COVID-19, (e.g., ARDS due to other pulmonary infections [75]). The lack of an appropriate control group is a limitation of our study, in addition to an imbalanced cohort across the disease stages and along the hospitalisation period. However, we refrained from selecting control samples from a separate cohort, as conducted in some COVID-19 studies, commonly incorporating healthy controls. Combining cohorts can lead to technical bias due to varying blood collection protocols and processing conditions, specifically affecting the less-stable endocannabinoids and oxylipins [76]. The aspects of collection, storage, and processing of blood samples are thoroughly discussed elsewhere [77,78,79,80]. Briefly, since circulating signalling lipids may fluctuate diurnally, special attention should be given to the time of blood collection, preferably in the early morning following overnight fasting [79]. Another source of differences between studies is the choice of blood product, which is also affected to a varying degree by pre-processing temperature and duration of storage. For lipidomics analysis, blood plasma is preferred over serum, since it prevents a skewed profiling of oxylipins and lysophospholipids, among other compounds [80]. To inspect the biochemical reproducibility of our findings, a follow-up study with a larger cohort is warranted. This may prove medically complicated due to the ongoing changes in treatment procedures, and the evolving SARS-CoV-2 variants.

## 4. Materials and Methods

### 4.1. Cohort

The cohort consisted of 44 adults admitted to the regional Amphia hospital in Breda, the Netherlands, on 24 March 2020–14 April 2020. Table 1 summarises key characteristics of the 44 patients and 103 collected blood samples (a more detailed summary is in Table S1). Table S2 provides background information and hospitalisation details per patient, such as comorbidities, treatment, and outcome. All patients reported COVID-19-related complaints and tested positive for the SARS-CoV-2 by a PCR.

### 4.2. Samples

EDTA blood samples were collected in intervals of 3–4 days throughout the study, as detailed in Table S2 per patient. A small aliquot of the collected blood was immediately taken for flow cytometric immune profiling. Plasma was isolated from the remaining blood, aliquoted, and stored at −20 °C until serological analysis, or until transportation to the analytical chemistry laboratory, where kept at −80 °C until sub-aliquoting and LC-MS analysis.

### 4.3. Haematological and Serological Analysis

Flowcytometric leukocyte analysis and serological analysis of cytokines and soluble cell surface molecules have been reported previously by Schrijver et al. [31]. All assays were performed according to manufacturer’s protocol. The measured parameters, values, and units are detailed in supplementary Table S3.

### 4.4. Plasma Lipids Analysis 

The complete details of sample preparation, metabolic coverage, analytical method, and performance are provided in Supplementary Document S1. Plasma samples were prepared by liquid-liquid extraction using butanol:MTBE (1:1, *v*/*v*), and analysed by two different RPLC-MS/MS methods (high and low pH). The chromatography was conducted on a Shimadzu Nexera X2 UHPLC (Shimadzu Corporation, Kyoto, Japan). For the high pH method, a Kinetex EVO C18 column was utilised (2.1 × 50 mm, 1.7 μm; Phenomenex Inc., Torrance, CA, USA). The low pH method used a Waters BEH C18 column (2.1 × 50 mm, 1.7 µm; Waters Corporation, Milford, MA, USA). Mass Spectrometry was conducted using a Shimadzu 8050 system in the high pH method, and a Sciex QTRAP 6500 MS (Sciex, Framingham, MA, USA) in the low pH method. ESI-MS was performed with polarity switching and multiple-reaction-monitoring (MRM). The acquired LC-MS data were processed using the vendor software (Sciex MultiQuant v3.0.2; Shimadzu Labsolutions v3.3), integrating the assigned MRM peaks and further correcting according to the peak areas of matched internal standards. In-house quality-control software (mzQuality) was utilised to assess and correct the analytical performance based on study QC replicates, blank samples, and internal standards. A total of 69 metabolites were measured by the two platforms, of which 63 passed the strict quality rules as examined by a data analysis expert, and utilised in the statistical analysis (see Document S1). The processed peak areas per metabolite and sample are deposited in Tables S4 and S5.

### 4.5. Statistical Analysis

All statistical analyses were performed in R, and graphs were plotted using the packages ggpubr and stats. Overall, 49 metabolites presented zero missingness and 14 had a maximum of 11% missingness (below LOD); therefore, no imputations were performed. Cytokine and immune marker data (*n* = 37) were analysed as provided (Table S3 [31]). All variables were cuberoot-transformed prior to statistical analyses. We could not identify clear outliers; therefore, no samples were removed from the dataset. Differential analysis between ICU and ward patients incorporating all samples was performed using linear regression correcting for age, sex, and BMI, grouped by patient, and weighted by the inverse number of observations per patient. The correlation between age, sex, and BMI and all variables is detailed in Table S6. Paired analyses between two time points of the same patient were performed using a paired t-test assuming unequal variances. This approach enabled patient-corrected analysis of metabolic changes, and a more meaningful metabolite fold-change than when calculated in non-paired analysis. Metabolite fold-change values were calculated based on the untransformed data, per patient in the paired t-test analysis, or by dividing the medians of experimental classes, in non-paired analysis. Pearson correlation analyses between metabolites and immune markers were conducted for all samples together and per hospitalisation status (ICU or ward), plotted as three regression lines to provide complementary information. The *p*-values obtained in all tests were adjusted for multiple testing using the Benjamini–Hochberg method implemented in the p.adjust R function (v.4.0.3), and termed Q-values. Significance levels were defined as Q < 0.1. The corrections were for either the number of variables in univariate tests (*n* = 63) or for the number of unique correlations in the Pearson correlation tests (*n* = 2394).

## 5. Conclusions

In this study, we demonstrated substantial differences between the signalling lipid profiles of COVID-19 patients at varying disease stages. The overall metabolic picture in severe COVID-19 was correlated with persistent inflammation and showed disruption in the balance of signalling lipids, potentially preventing an effective shift into resolution of inflammation. With the goal of increasing the levels of anti-inflammatory and pro-resolving lipid mediators, these observations can promote the fine tuning of pharmaceutical treatment, for example by cytokine inhibitors vs. corticosteroid and other inhibitors of lipid metabolism enzymes. Together with other studies, we showcased the importance of metabolomics and lipidomics approaches to expend the biochemical knowledge and advance the research of COVID-19 progression, effects, and treatment.

## Figures and Tables

**Table 1:** Selected characteristics of the COVID-19 patients in the metabolomics study *. Values are *n* (%) or median [full range]. See Tables S1 and S2 for further information.

	Patients (*n* = 44)	Samples (*n* = 103)
Age, years	73 [49–87]	71 [49–87]
Male (%)	30 (68%)	65 (63%)
BMI	27 [19–42]	
Diabetes and/or cardiovascular disease (CVD)	14 (32%)	
Chronic obstructive pulmonary disease (COPD)	8 (18%)	
Days with symptoms until hospitalisation	8 [1–19]	
Total hospitalisation days	7 [2–62]	
Admitted to ward	37 (84%)	78 (76%)
Admitted to ICU	7 (16%)	25 (24%)
Deceased	9 (20%)	
Treatment with chloroquine	35 (80%)	
Treatment with antibiotics	38 (86%)	
Treatment with corticosteroids	2 (5%)	
CRP, mg/L (normal <10)		104.5 [3–577]
Lymphocytes, 109/L (normal 1.0–2.8)		0.95 [0.26–3.15]
Neutrophils, 109/L (normal 1.7–6.5)		6.36 [2.3–17.5]

* Information about comorbidities and medication (4 weeks pre-admission) is missing for 25% of patients (*n* = 12).

## Data Availability

All data utilised in the statistical analyses are available as part of supplementary files.

## Supplementary Materials

The following supporting information can be downloaded at: https://www.mdpi.com/article/10.3390/metabo12070619/s1, Figure S1: PCA loadings of all study samples, based on all metabolites; Figure S2: Heatmap of Pearson correlations between metabolites and immune markers; Figure S3: Correlation networks between immune markers and arachidonic acid pathway metabolites, produced separately for ward and ICU patient samples; Table S1: Demographics, health and treatment overview of the cohort patients; Table S2: Description of the cohort patients; Table S3: Immune markers measurement results; Table S4: Processed relative peak areas in the signalling lipids high pH platform; Table S5: Processed relative peak areas in the signalling lipids low pH platform; Table S6: Pearson correlation analysis between confounders and analysed variables; Table S7: Loading values of the PCA plot in Figure 1 and the loading plot in Figure S1; Table S8: Significance values for metabolites differentiating between ICU and ward patients; Table S9: Paired t-tests comparing a first and last time point of selected ward patients; Table S10: Pearson correlation coefficients between all variables; Table S11: Pearson correlation *p* values between all variables; Table S12: Pearson correlation FDR Q values between metabolites and immune markers; Table S13: Pearson correlations between metabolites and immune markers—ICU patients; Table S14: Pearson correlations between metabolites and immune markers—ward patients; Document S1: Signalling lipids platform metabolic coverage, method, and performance; Document S2: Box and scatter plots for all comparisons between ICU and ward patients; Document S3: Box and scatter plots for all paired comparisons between ward patients; Document S4: Pearson correlation plots between metabolites and immune markers.
