# Supplementary material for: Plasma Oxylipins and Their Precursors Are Strongly Associated with COVID-19 Severity and with Immune Response Markers
Source: Metabolites. 2022 Jul 4;12(7):619. doi: 10.3390/metabo12070619 (PMC9319897; doi:10.3390/metabo12070619)
Supplement: Supplementary file 1 [file metabolites-12-00619-s001.zip › Document S3.pdf]

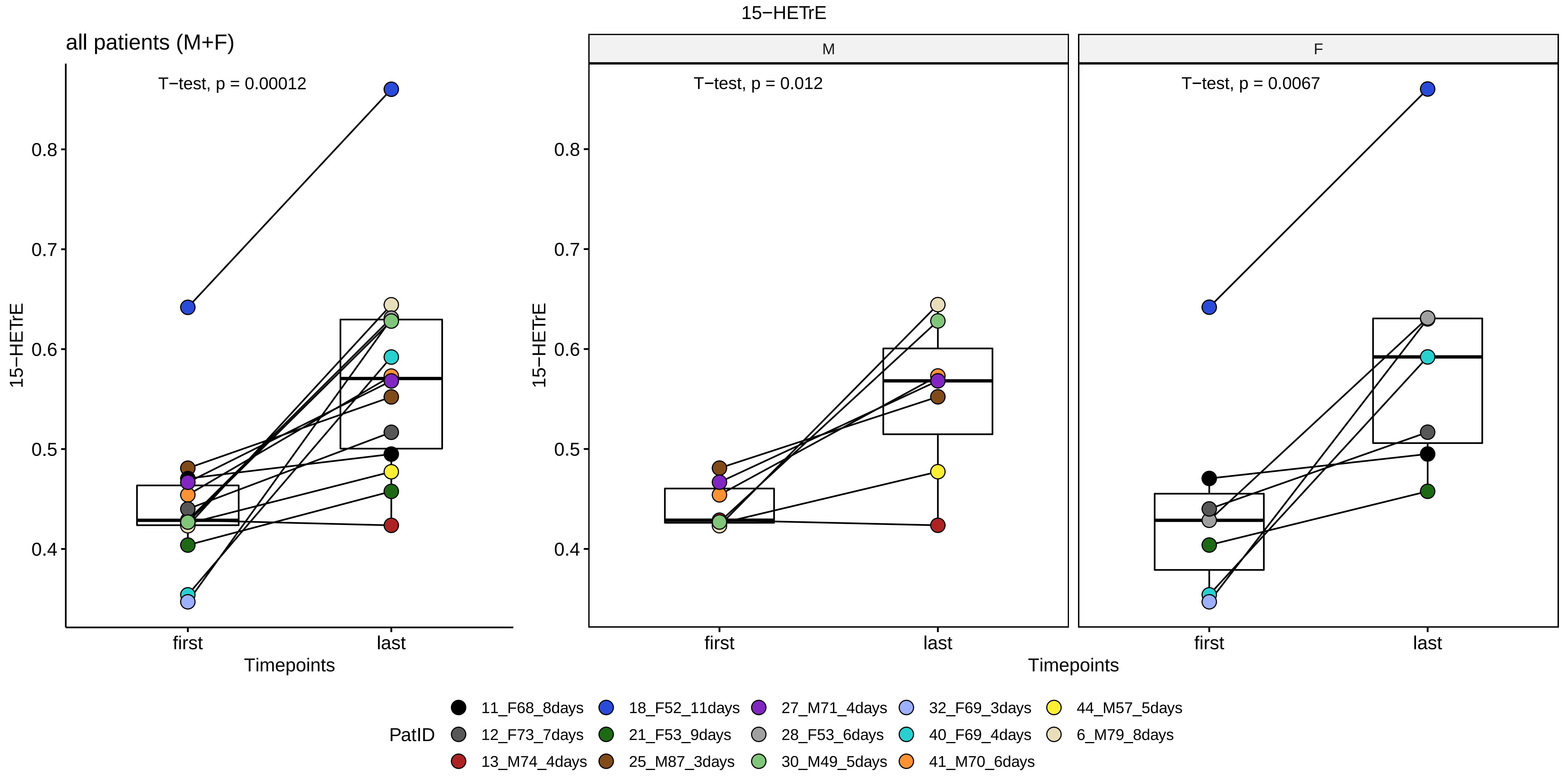

15−HETrE
all patients (M+F)
T−test, p = 0.00012
M
T−test, p = 0.012
F
T−test, p = 0.0067
15−HETrE
0.4
0.5
0.6
0.7
0.8
first
last
Timepoints
PatID
11_F68_8days
12_F73_7days
13_M74_4days
18_F52_11days
21_F53_9days
25_M87_3days
27_M71_4days
28_F53_6days
30_M49_5days
32_F69_3days
40_F69_4days
41_M70_6days
44_M57_5days
6_M79_8days

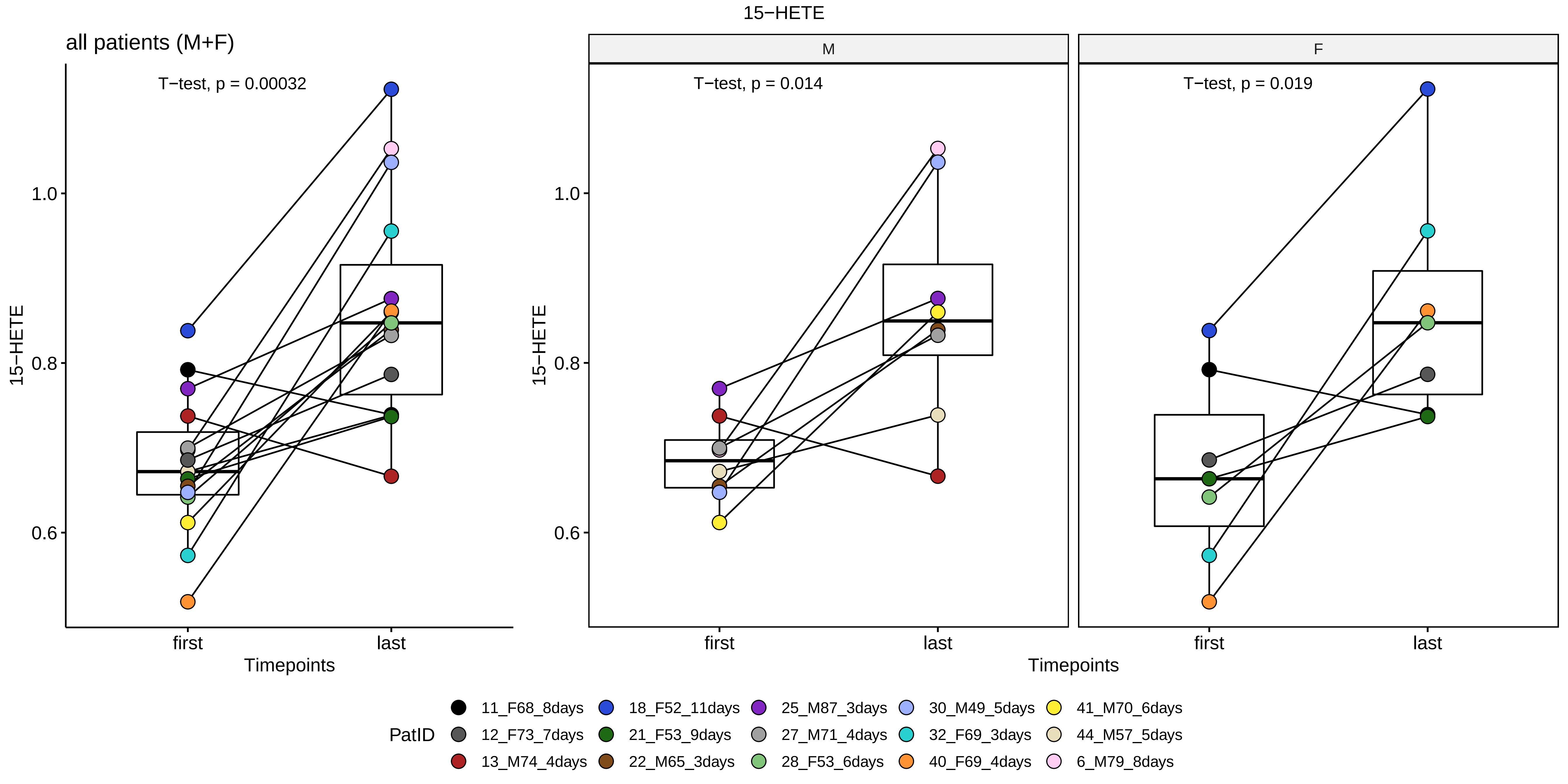
15−HETE
all patients (M+F)
T−test, p = 0.00032
M
T−test, p = 0.014
F
T−test, p = 0.019
15−HETE
1.0
0.8
0.6
first
last
Timepoints
PatID
11_F68_8days
12_F73_7days
13_M74_4days
18_F52_11days
21_F53_9days
22_M65_3days
25_M87_3days
27_M71_4days
28_F53_6days
30_M49_5days
32_F69_3days
40_F69_4days
41_M70_6days
44_M57_5days
6_M79_8days

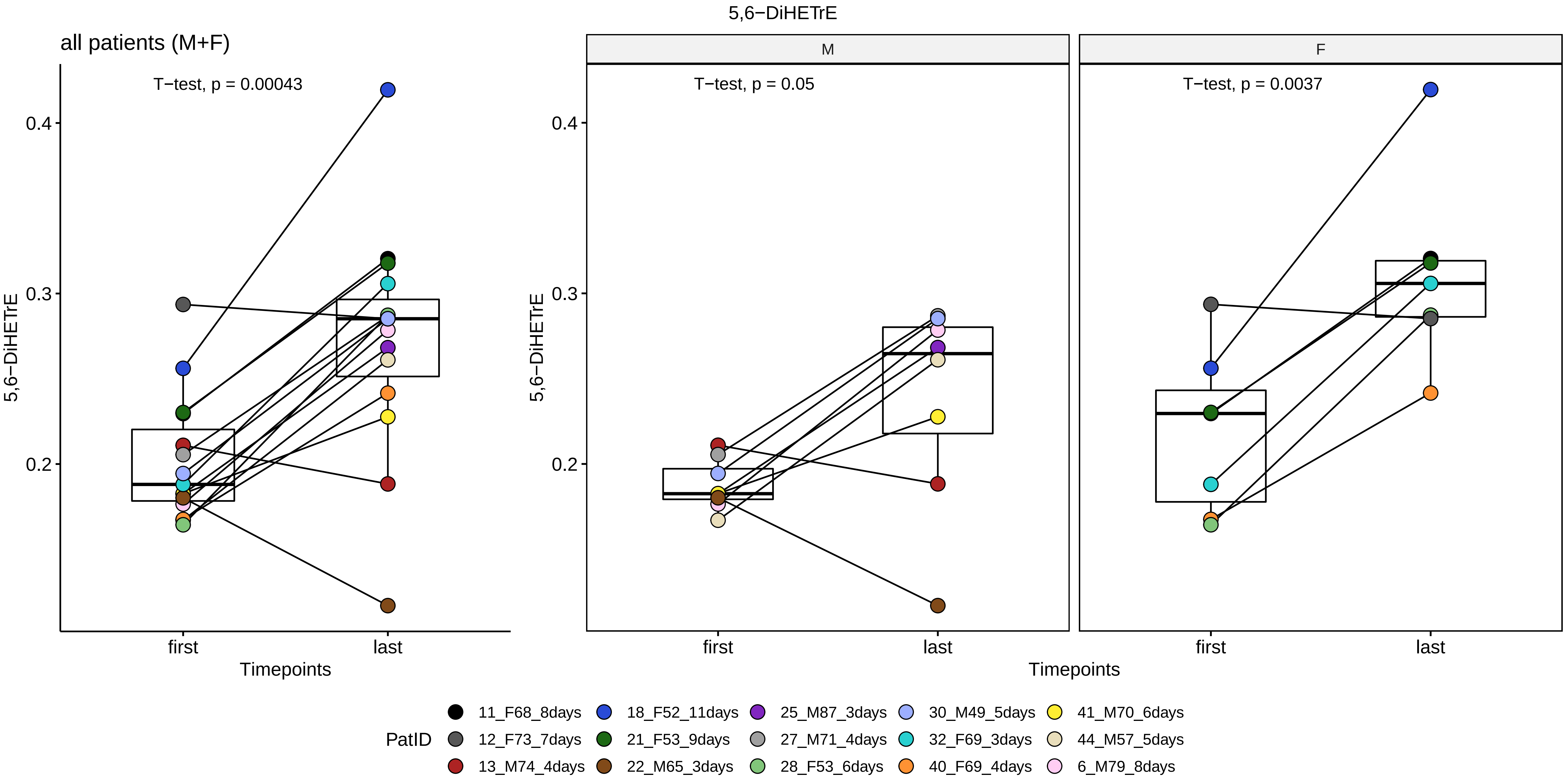
5,6−DiHETrE
all patients (M+F)
T−test, p = 0.00043
M
T−test, p = 0.05
F
T−test, p = 0.0037
5,6−DiHETrE
0.4
0.3
0.2
first
last
Timepoints
PatID
11_F68_8days
12_F73_7days
13_M74_4days
18_F52_11days
21_F53_9days
22_M65_3days
25_M87_3days
27_M71_4days
28_F53_6days
30_M49_5days
32_F69_3days
40_F69_4days
41_M70_6days
44_M57_5days
6_M79_8days

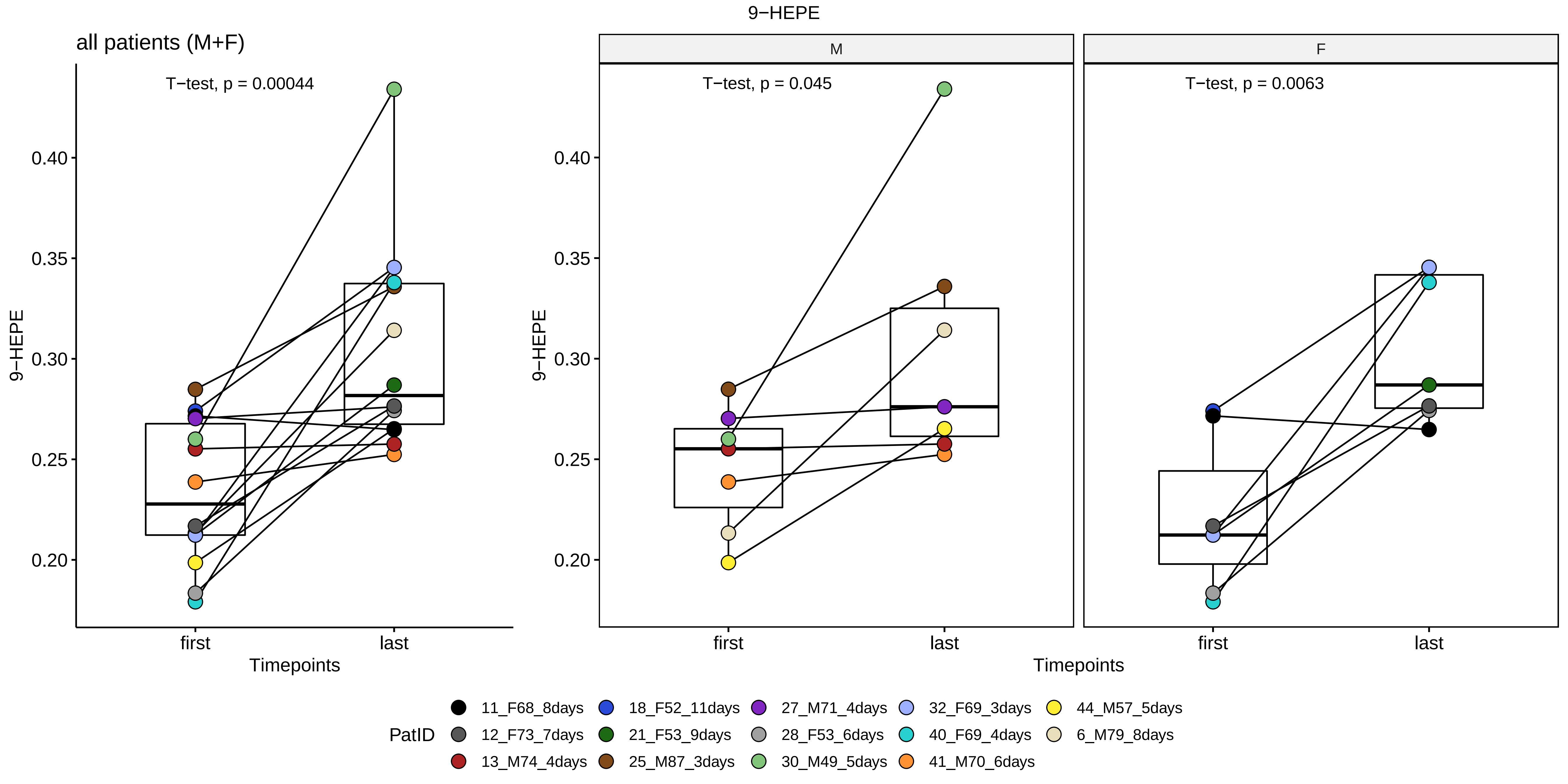
9−HEPE
all patients (M+F)
T−test, p = 0.00044
M
T−test, p = 0.045
F
T−test, p = 0.0063
9−HEPE
0.20
0.25
0.30
0.35
0.40
first
last
Timepoints
PatID
11_F68_8days
12_F73_7days
13_M74_4days
18_F52_11days
21_F53_9days
25_M87_3days
27_M71_4days
28_F53_6days
30_M49_5days
32_F69_3days
40_F69_4days
41_M70_6days
44_M57_5days
6_M79_8days

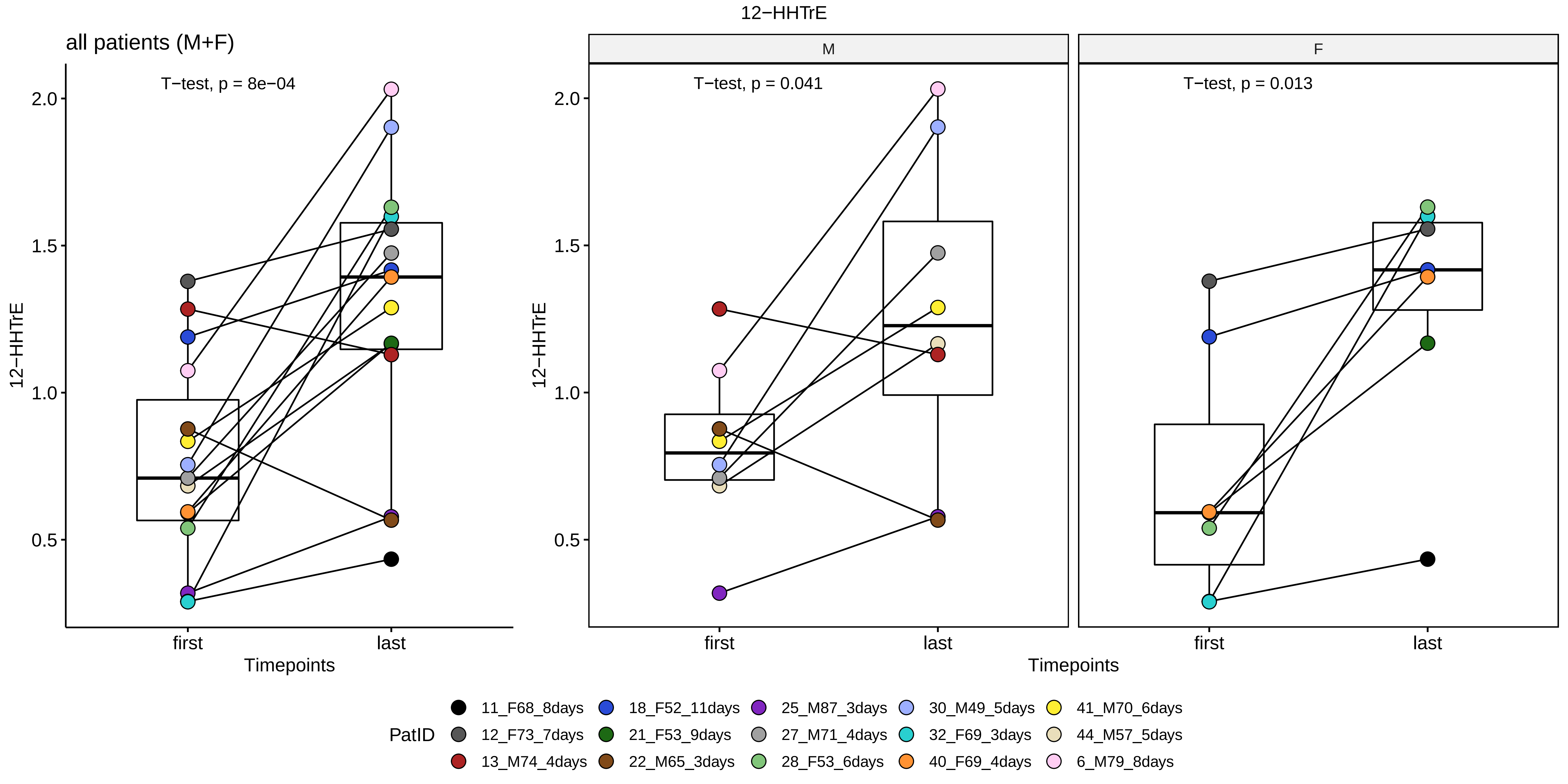
12−HHTrE
all patients (M+F)
T−test, p = 8e−04
M
T−test, p = 0.041
F
T−test, p = 0.013
12−HHTrE
2.0
1.5
1.0
0.5
first
last
Timepoints
PatID
11_F68_8days
12_F73_7days
13_M74_4days
18_F52_11days
21_F53_9days
22_M65_3days
25_M87_3days
27_M71_4days
28_F53_6days
30_M49_5days
32_F69_3days
40_F69_4days
41_M70_6days
44_M57_5days
6_M79_8days

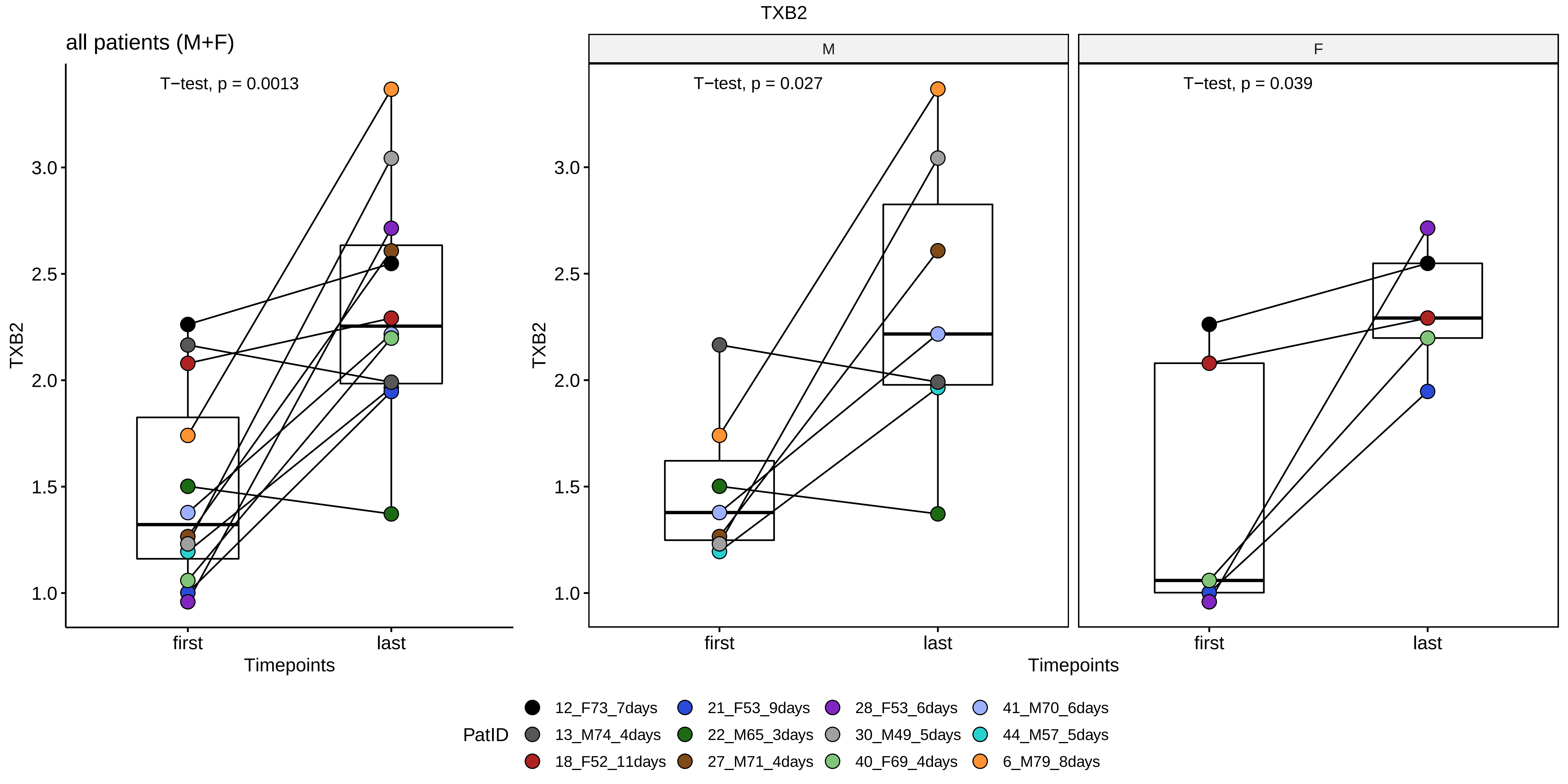
TXB2
all patients (M+F)
T−test, p = 0.0013
M
T−test, p = 0.027
F
T−test, p = 0.039
TXB2
3.0
2.5
2.0
1.5
1.0
first
last
Timepoints
PatID
12_F73_7days
13_M74_4days
18_F52_11days
21_F53_9days
22_M65_3days
27_M71_4days
28_F53_6days
30_M49_5days
40_F69_4days
41_M70_6days
44_M57_5days
6_M79_8days

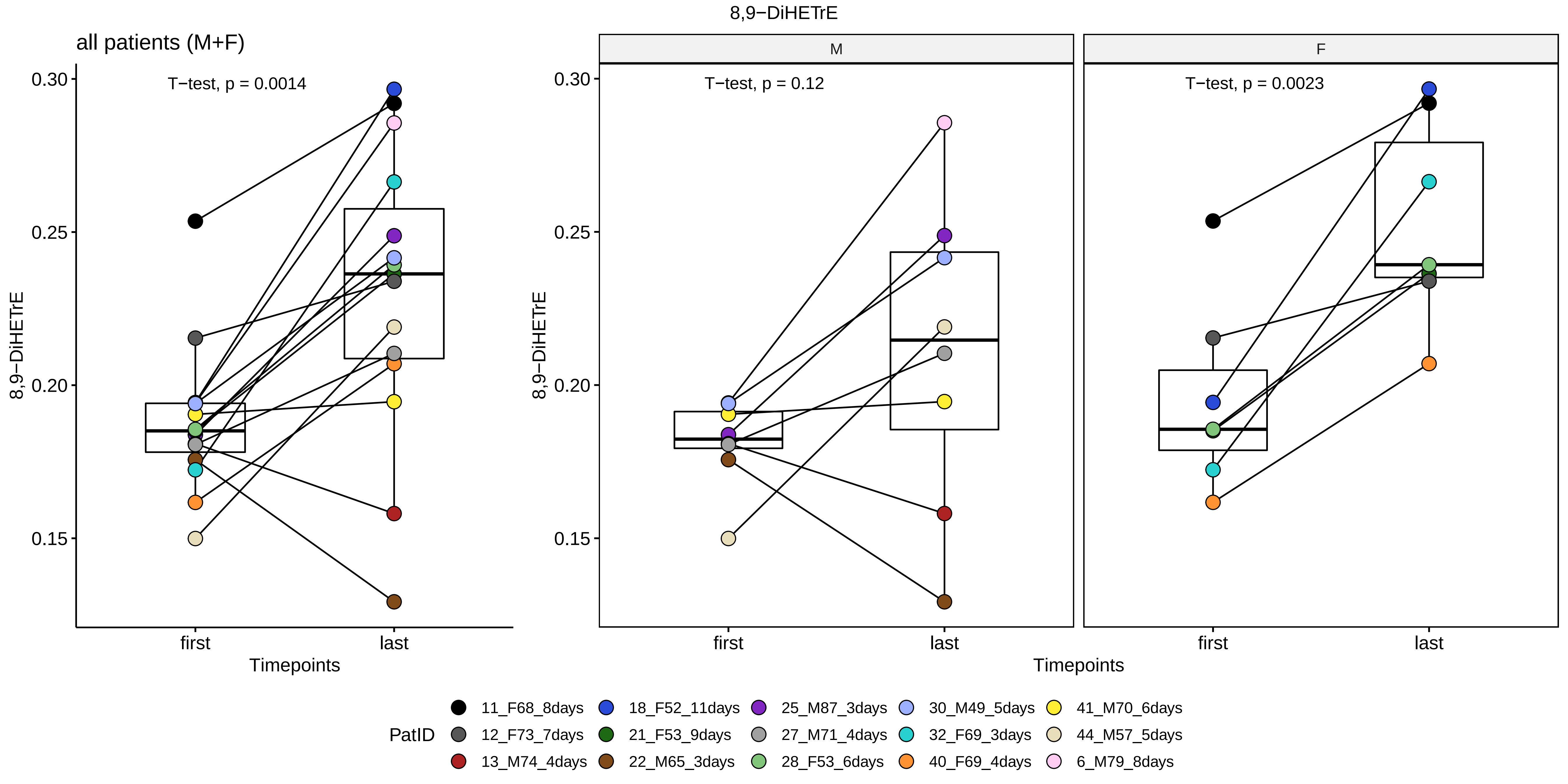

8,9−DiHETrE
all patients (M+F)
T−test, p = 0.0014
M
T−test, p = 0.12
F
T−test, p = 0.0023
0.30
0.25
0.20
0.15
8,9−DiHETrE
first
last
Timepoints
PatID
11_F68_8days
12_F73_7days
13_M74_4days
18_F52_11days
21_F53_9days
22_M65_3days
25_M87_3days
27_M71_4days
28_F53_6days
30_M49_5days
32_F69_3days
40_F69_4days
41_M70_6days
44_M57_5days
6_M79_8days

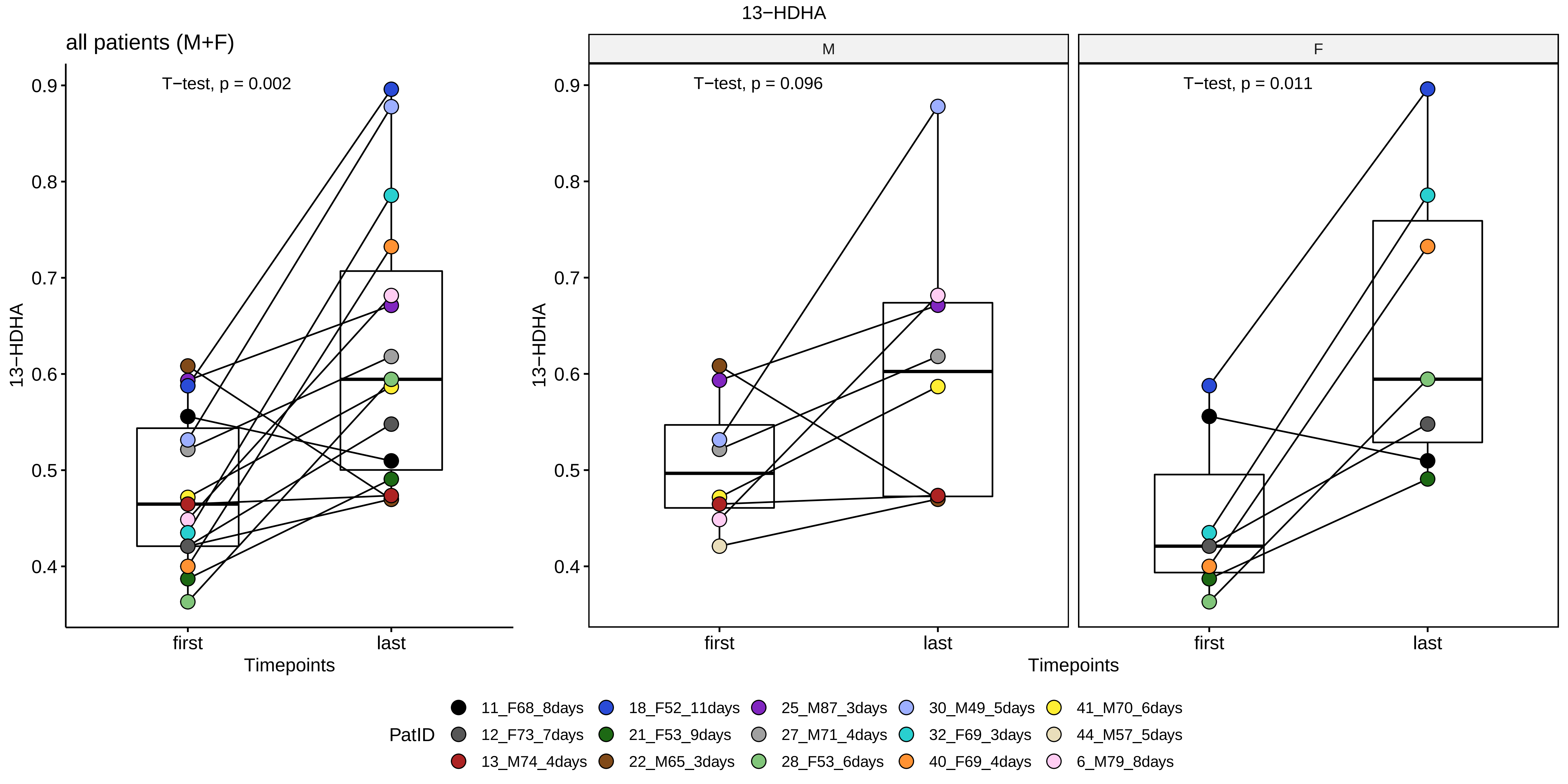

13−HDHA
all patients (M+F)
T−test, p = 0.002
M
T−test, p = 0.096
F
T−test, p = 0.011
13−HDHA
0.9
0.8
0.7
0.6
0.5
0.4
first
last
Timepoints
PatID
11_F68_8days
12_F73_7days
13_M74_4days
18_F52_11days
21_F53_9days
22_M65_3days
25_M87_3days
27_M71_4days
28_F53_6days
30_M49_5days
32_F69_3days
40_F69_4days
41_M70_6days
44_M57_5days
6_M79_8days

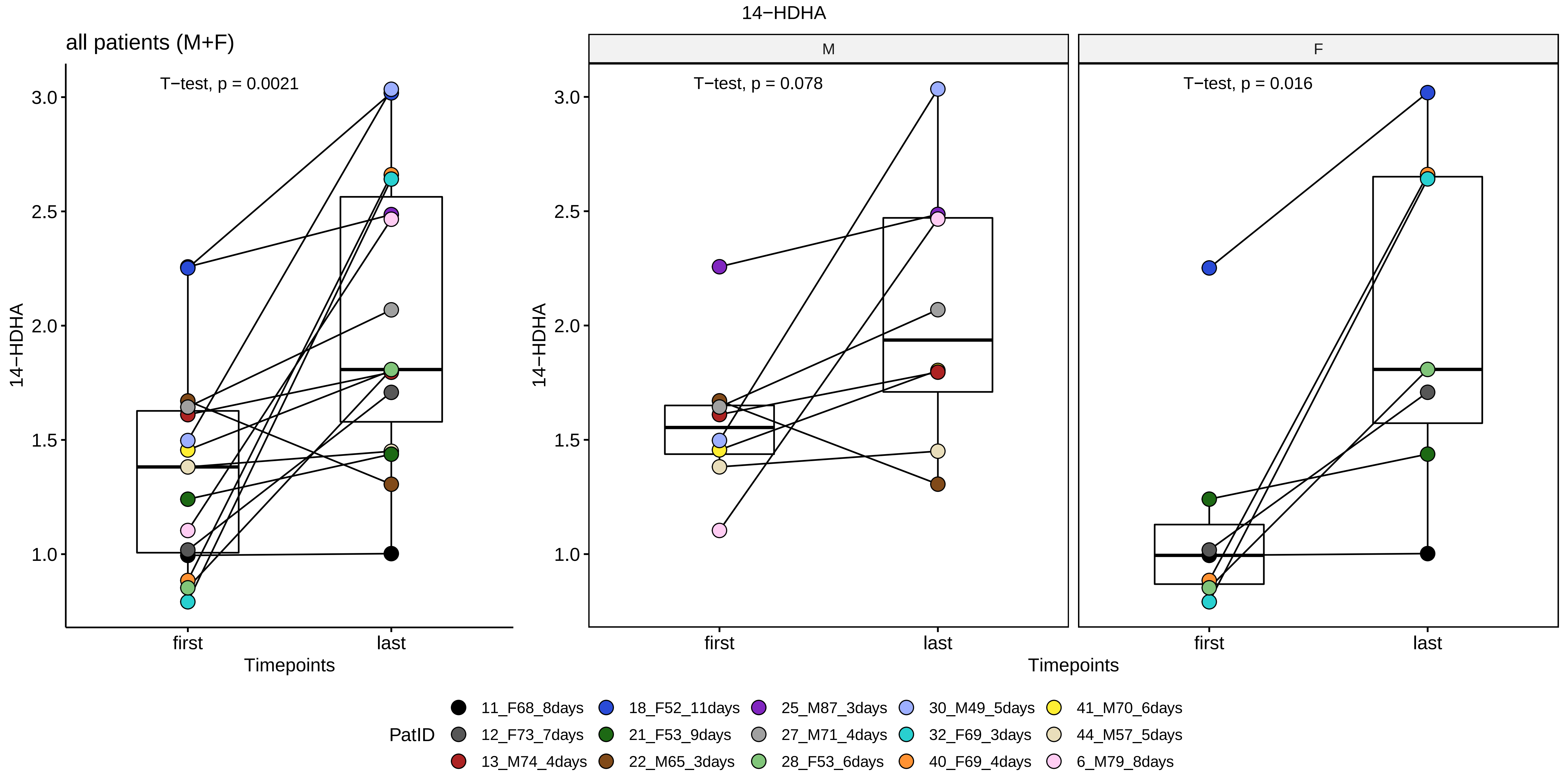

14−HDHA
all patients (M+F)
T−test, p = 0.0021
M
T−test, p = 0.078
F
T−test, p = 0.016
14−HDHA
3.0
2.5
2.0
1.5
1.0
first
last
Timepoints
PatID
11_F68_8days
12_F73_7days
13_M74_4days
18_F52_11days
21_F53_9days
22_M65_3days
25_M87_3days
27_M71_4days
28_F53_6days
30_M49_5days
32_F69_3days
40_F69_4days
41_M70_6days
44_M57_5days
6_M79_8days

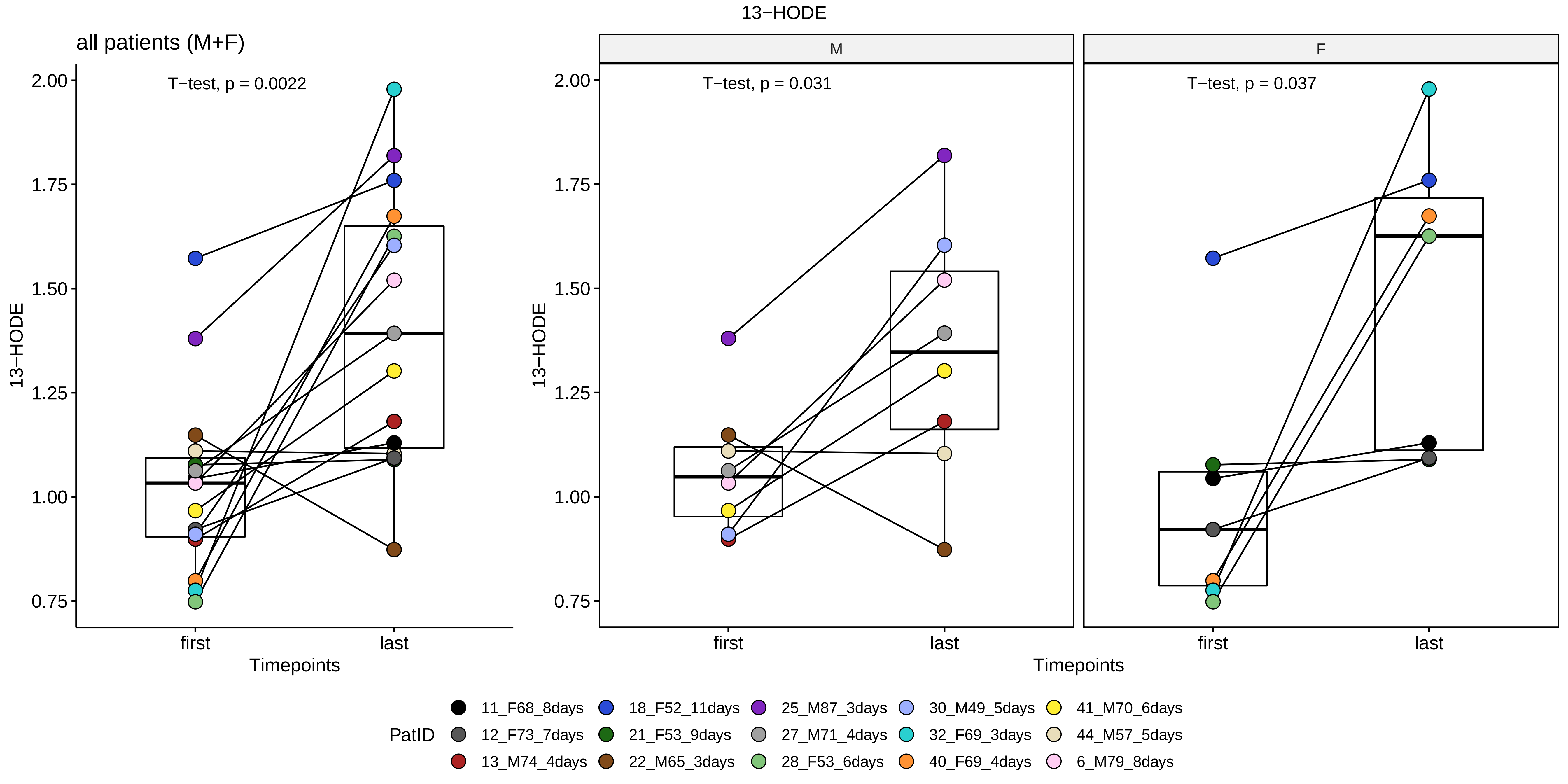

13−HODE
all patients (M+F)
T−test, p = 0.0022
M
T−test, p = 0.031
F
T−test, p = 0.037
13−HODE
2.00
1.75
1.50
1.25
1.00
0.75
first
last
Timepoints
PatID
11_F68_8days
12_F73_7days
13_M74_4days
18_F52_11days
21_F53_9days
22_M65_3days
25_M87_3days
27_M71_4days
28_F53_6days
30_M49_5days
32_F69_3days
40_F69_4days
41_M70_6days
44_M57_5days
6_M79_8days

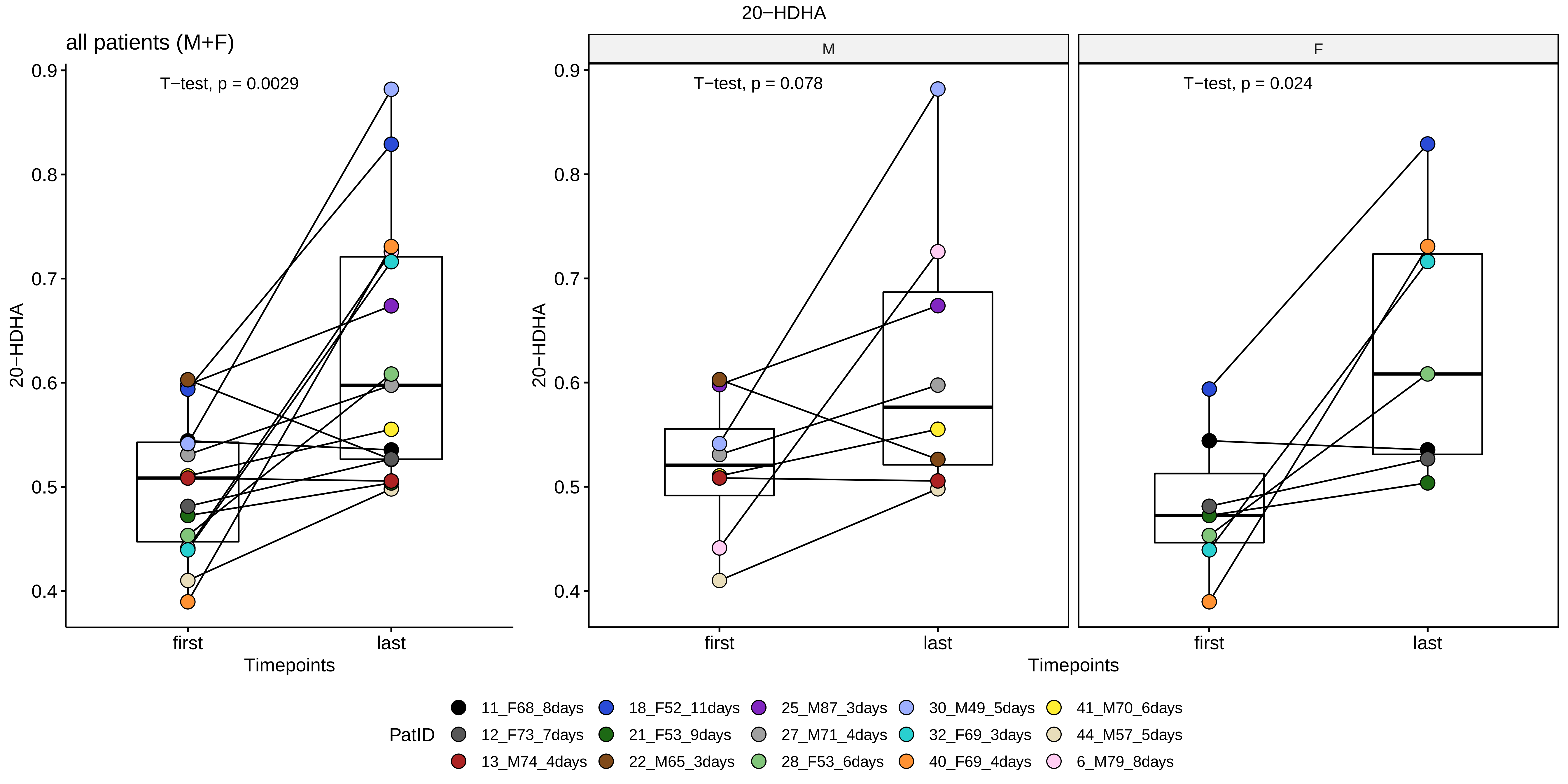
20−HDHA
all patients (M+F)
T−test, p = 0.0029
M
T−test, p = 0.078
F
T−test, p = 0.024
20−HDHA
0.4
0.5
0.6
0.7
0.8
0.9
first
last
Timepoints
PatID
11_F68_8days
12_F73_7days
13_M74_4days
18_F52_11days
21_F53_9days
22_M65_3days
25_M87_3days
27_M71_4days
28_F53_6days
30_M49_5days
32_F69_3days
40_F69_4days
41_M70_6days
44_M57_5days
6_M79_8days

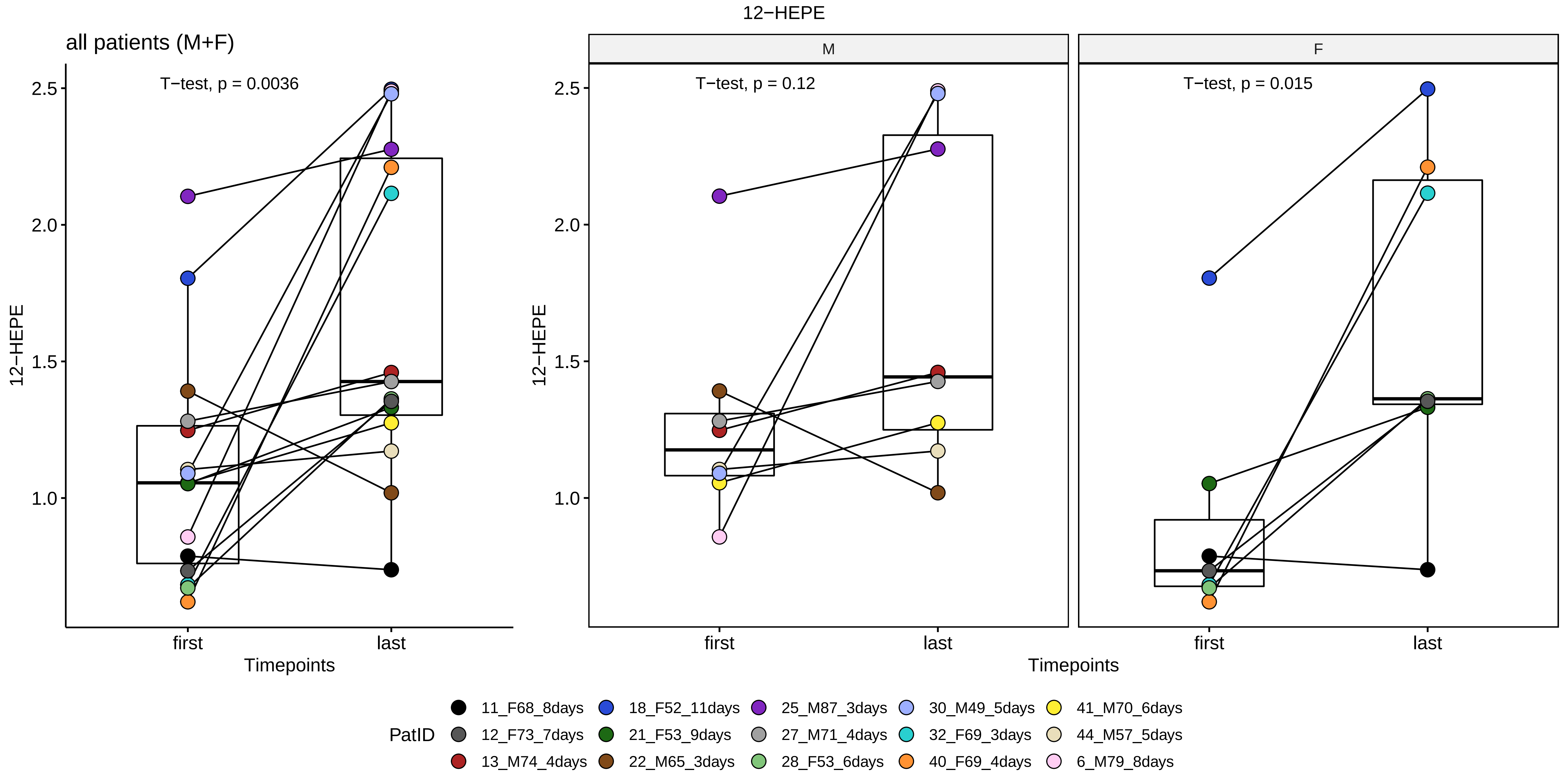

12−HEPE
all patients (M+F)
T−test, p = 0.0036
M
T−test, p = 0.12
F
T−test, p = 0.015
2.5
2.0
1.5
1.0
12−HEPE
first
last
Timepoints
PatID
11_F68_8days
12_F73_7days
13_M74_4days
18_F52_11days
21_F53_9days
22_M65_3days
25_M87_3days
27_M71_4days
28_F53_6days
30_M49_5days
32_F69_3days
40_F69_4days
41_M70_6days
44_M57_5days
6_M79_8days

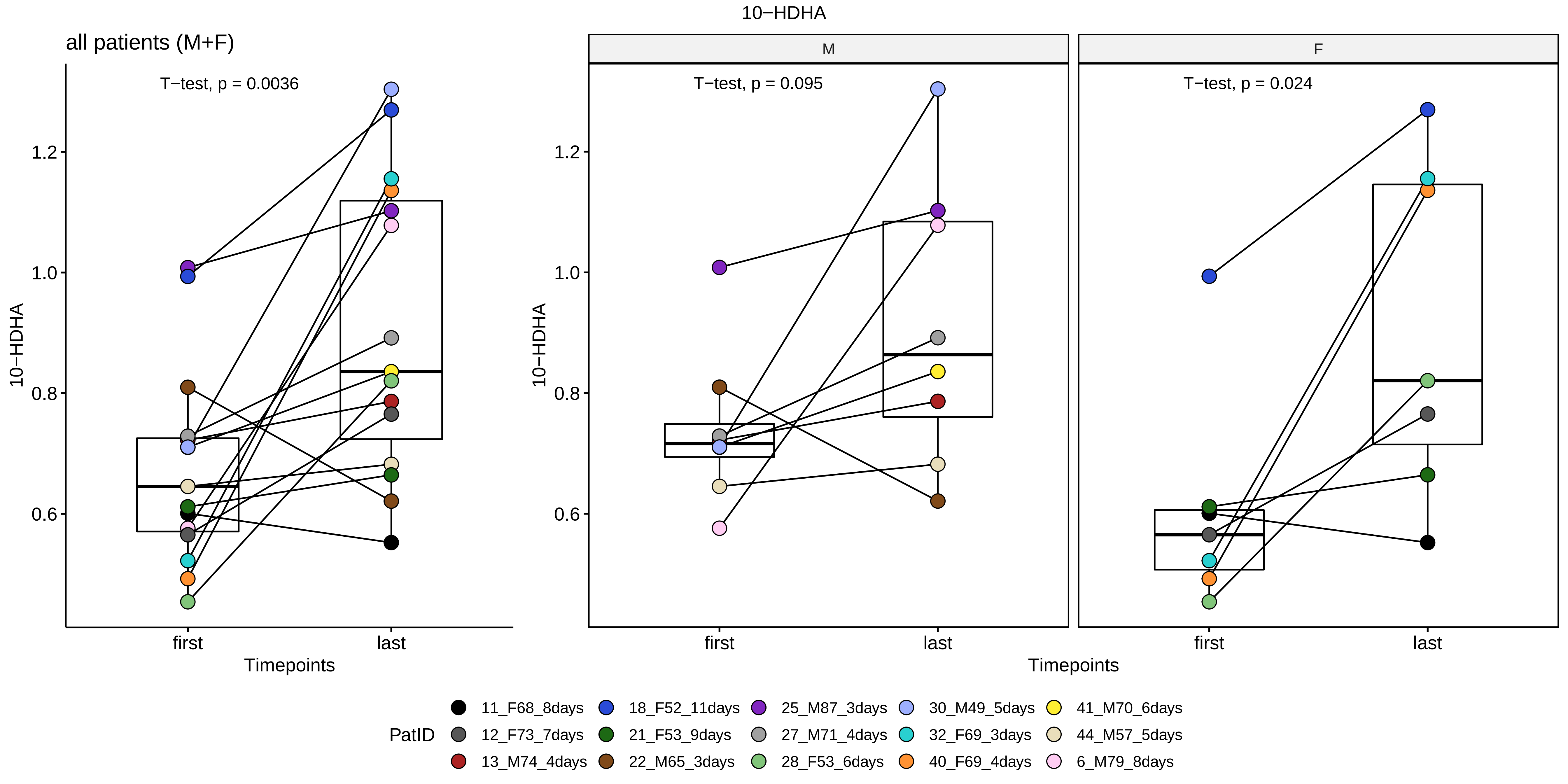

10−HDHA
all patients (M+F)
T−test, p = 0.0036
M
T−test, p = 0.095
F
T−test, p = 0.024
1.2
1.0
0.8
0.6
10−HDHA
first
last
Timepoints
PatID
11_F68_8days
12_F73_7days
13_M74_4days
18_F52_11days
21_F53_9days
22_M65_3days
25_M87_3days
27_M71_4days
28_F53_6days
30_M49_5days
32_F69_3days
40_F69_4days
41_M70_6days
44_M57_5days
6_M79_8days

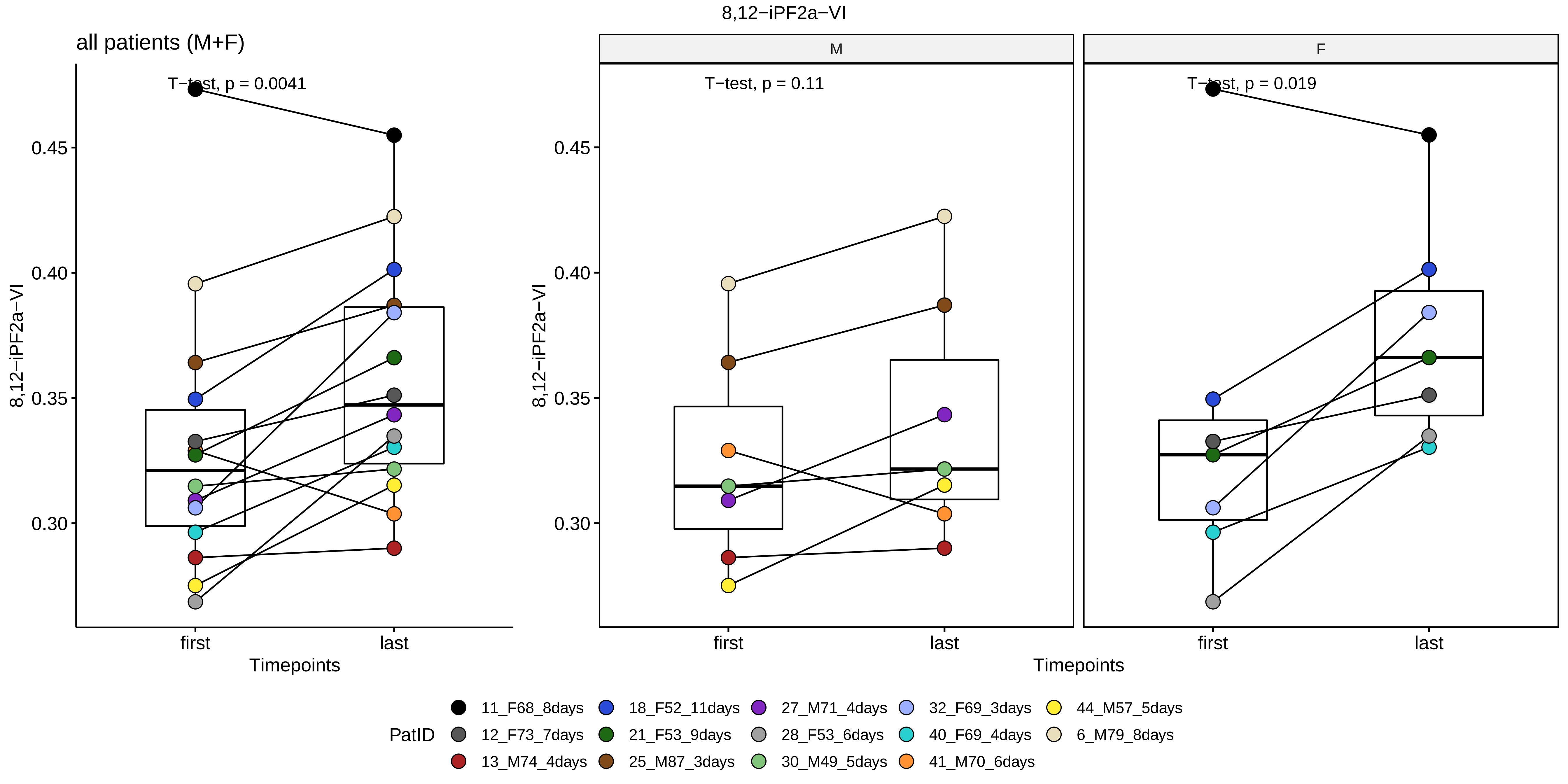

8,12−iPF2a−VI
all patients (M+F)
T−test, p = 0.0041
M
T−test, p = 0.11
F
T−test, p = 0.019
8,12−iPF2a−VI
0.45
0.40
0.35
0.30
first
last
Timepoints
PatID
11_F68_8days
12_F73_7days
13_M74_4days
18_F52_11days
21_F53_9days
25_M87_3days
27_M71_4days
28_F53_6days
30_M49_5days
32_F69_3days
40_F69_4days
41_M70_6days
44_M57_5days
6_M79_8days

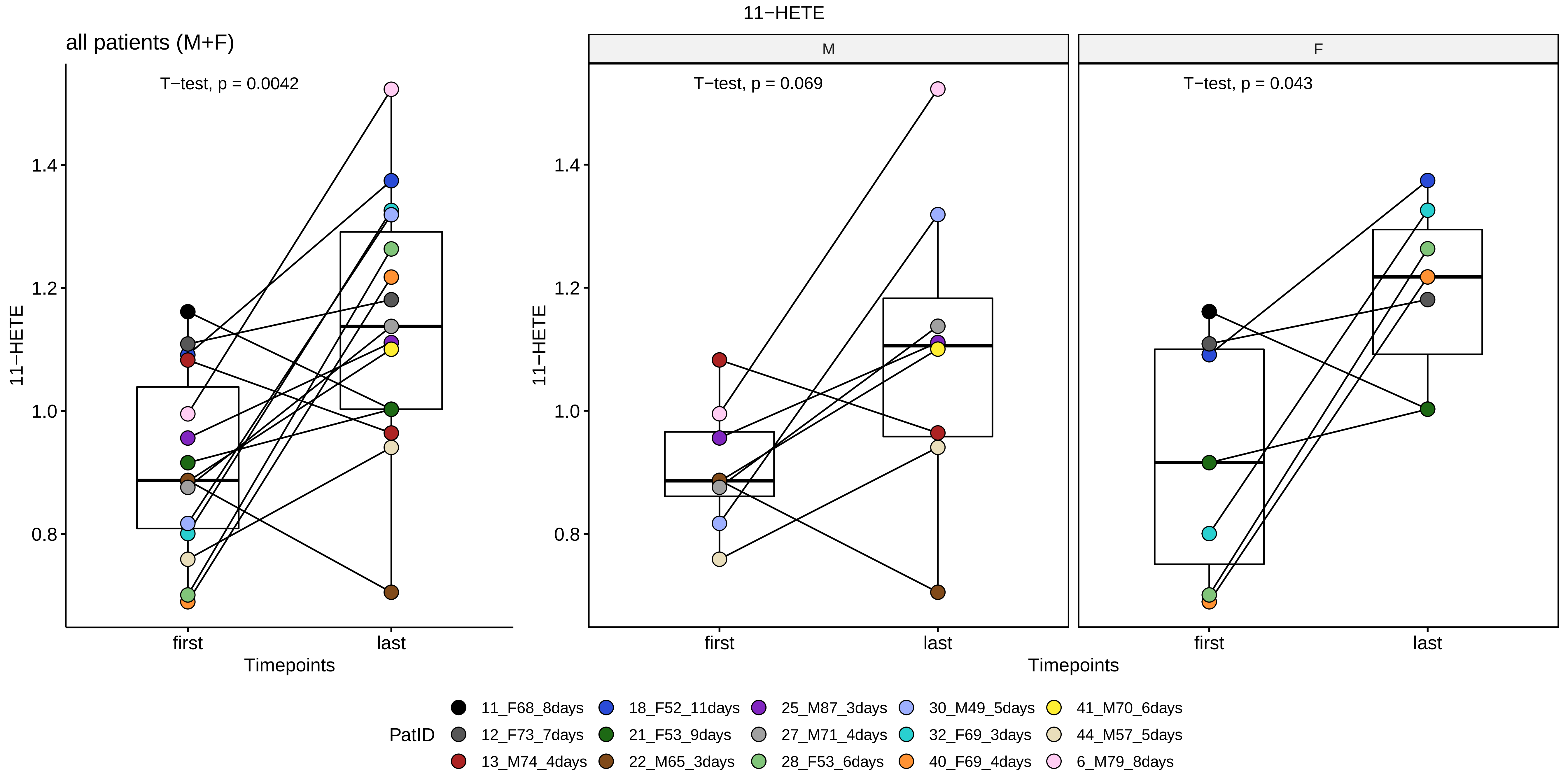

11−HETE
all patients (M+F)
T−test, p = 0.0042
M
T−test, p = 0.069
F
T−test, p = 0.043
11−HETE
1.4
1.2
1.0
0.8
first
last
Timepoints
PatID
11_F68_8days
12_F73_7days
13_M74_4days
18_F52_11days
21_F53_9days
22_M65_3days
25_M87_3days
27_M71_4days
28_F53_6days
30_M49_5days
32_F69_3days
40_F69_4days
41_M70_6days
44_M57_5days
6_M79_8days

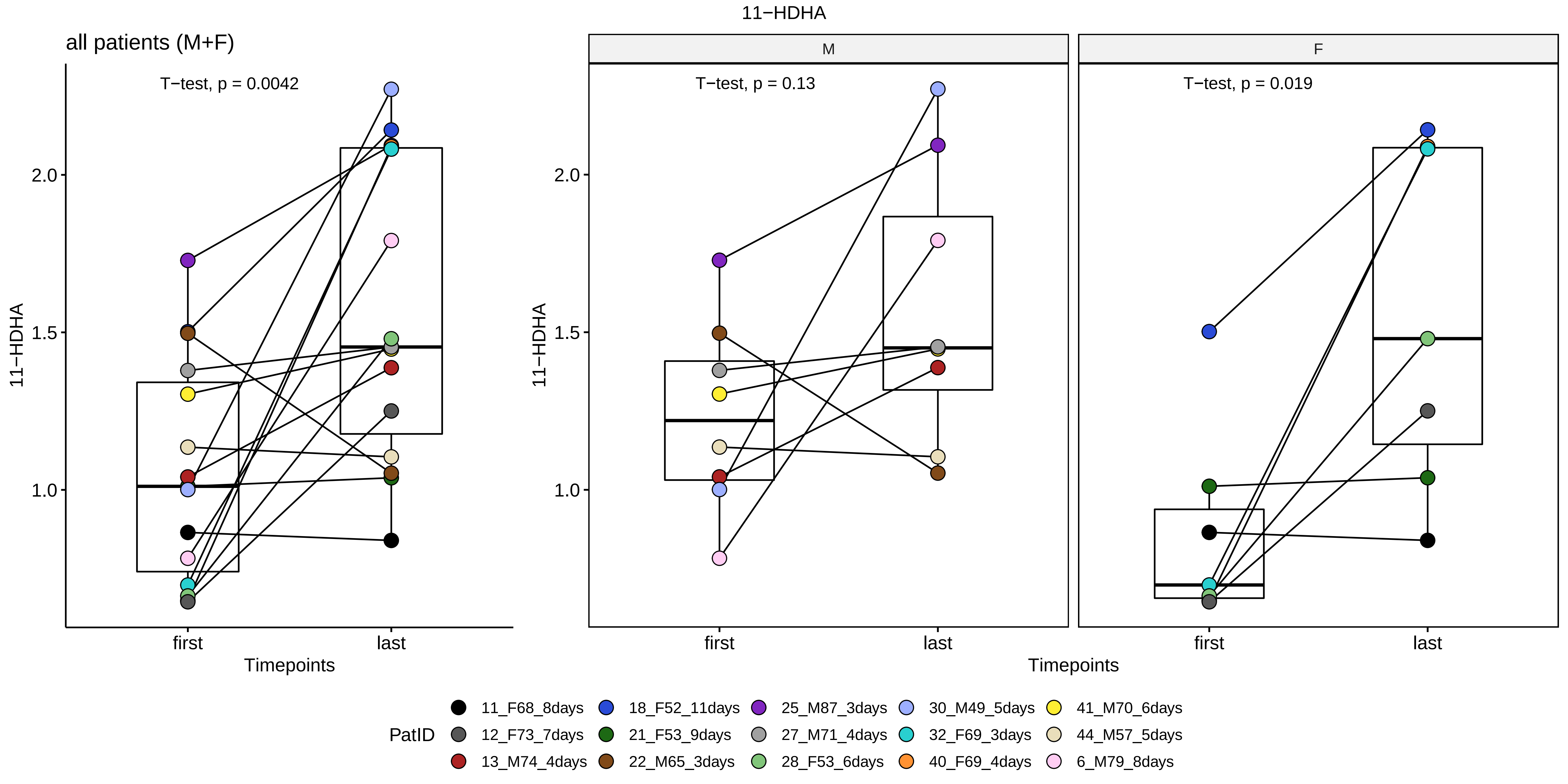

11−HDHA
all patients (M+F)
T−test, p = 0.0042
M
T−test, p = 0.13
F
T−test, p = 0.019
11−HDHA
2.0
1.5
1.0
first
last
Timepoints
PatID
11_F68_8days
12_F73_7days
13_M74_4days
18_F52_11days
21_F53_9days
22_M65_3days
25_M87_3days
27_M71_4days
28_F53_6days
30_M49_5days
32_F69_3days
40_F69_4days
41_M70_6days
44_M57_5days
6_M79_8days

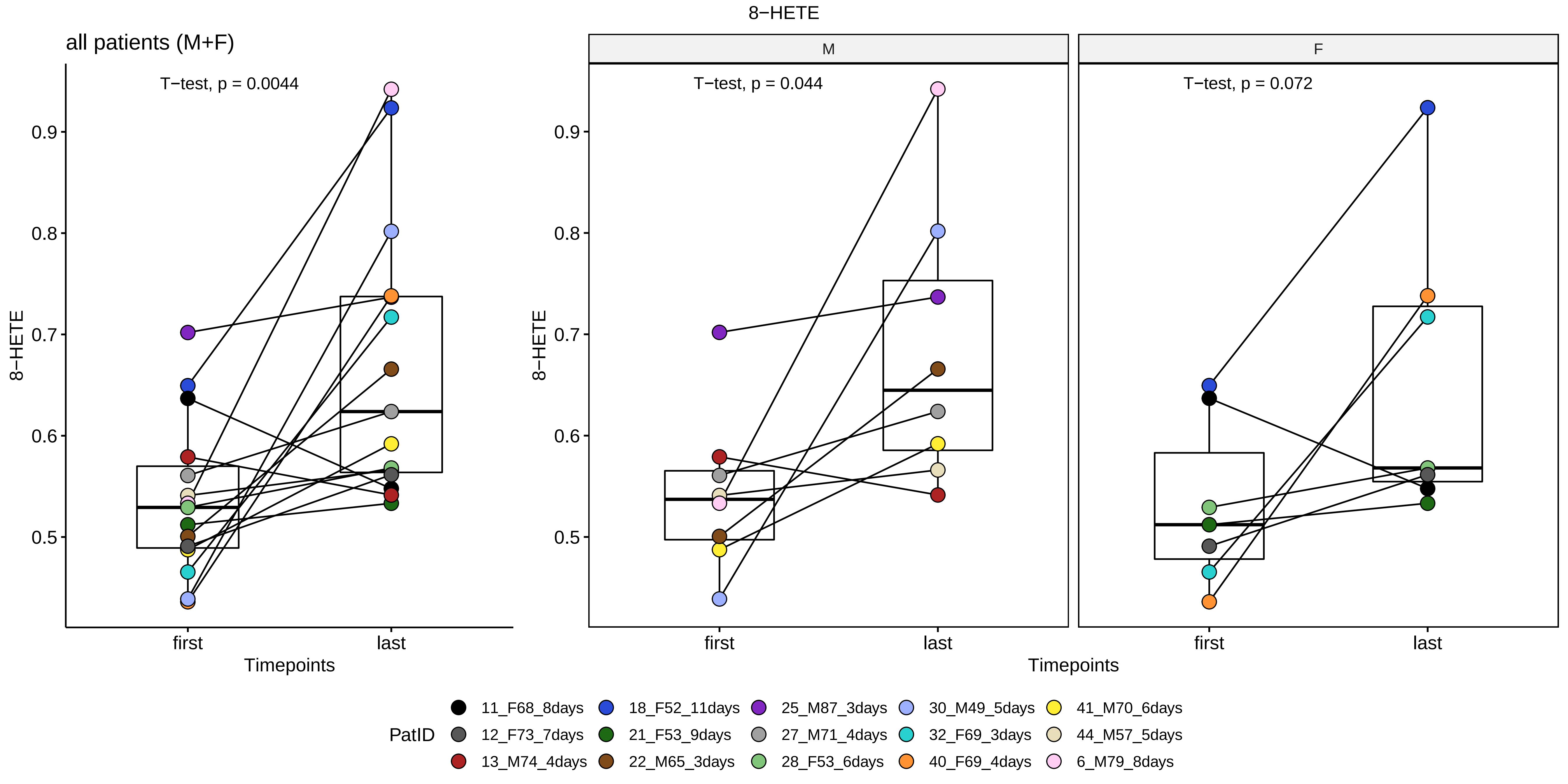
8−HETE
all patients (M+F)
T−test, p = 0.0044
M
T−test, p = 0.044
F
T−test, p = 0.072
8−HETE
0.5
0.6
0.7
0.8
0.9
first
last
Timepoints
PatID
11_F68_8days
12_F73_7days
13_M74_4days
18_F52_11days
21_F53_9days
22_M65_3days
25_M87_3days
27_M71_4days
28_F53_6days
30_M49_5days
32_F69_3days
40_F69_4days
41_M70_6days
44_M57_5days
6_M79_8days

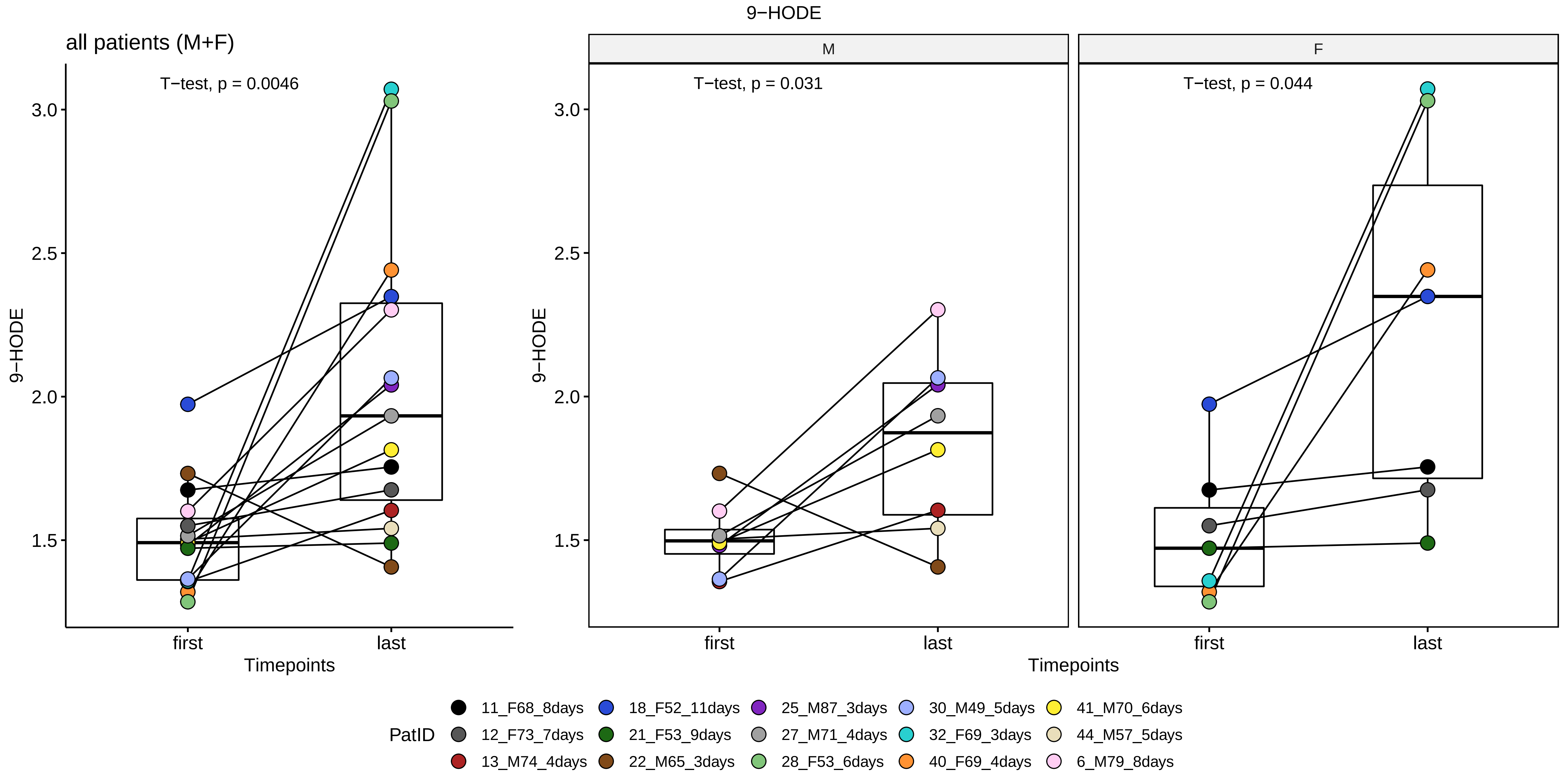
9−HODE
all patients (M+F)
T−test, p = 0.0046
9−HODE
3.0
2.5
2.0
1.5
first
last
Timepoints
M
T−test, p = 0.031
F
T−test, p = 0.044
PatID
11_F68_8days
12_F73_7days
13_M74_4days
18_F52_11days
21_F53_9days
22_M65_3days
25_M87_3days
27_M71_4days
28_F53_6days
30_M49_5days
32_F69_3days
40_F69_4days
41_M70_6days
44_M57_5days
6_M79_8days

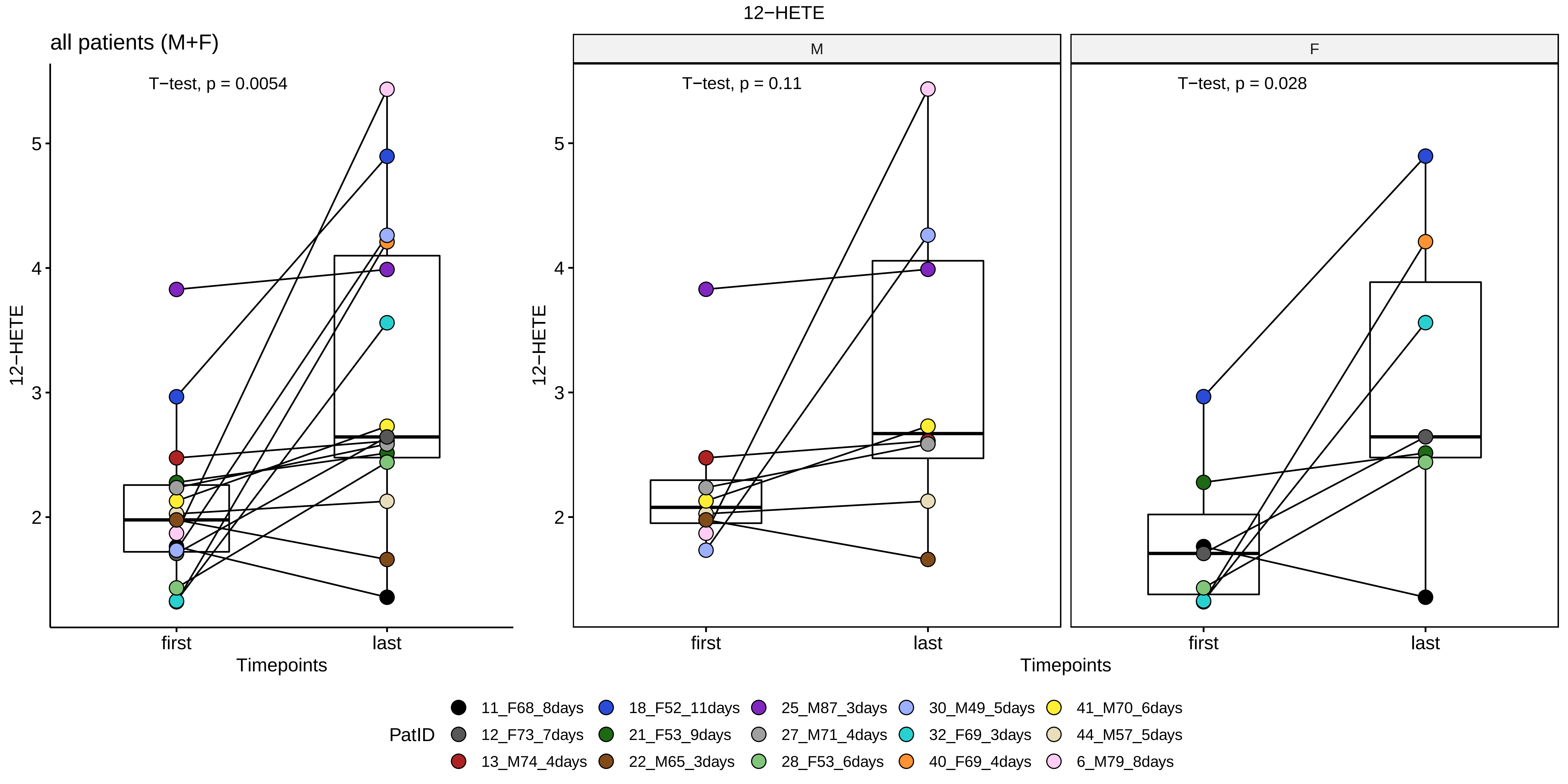
12−HETE
all patients (M+F)
T−test, p = 0.0054
M
T−test, p = 0.11
F
T−test, p = 0.028
12−HETE
5
4
3
2
first
last
Timepoints
PatID
11_F68_8days
12_F73_7days
13_M74_4days
18_F52_11days
21_F53_9days
22_M65_3days
25_M87_3days
27_M71_4days
28_F53_6days
30_M49_5days
32_F69_3days
40_F69_4days
41_M70_6days
44_M57_5days
6_M79_8days

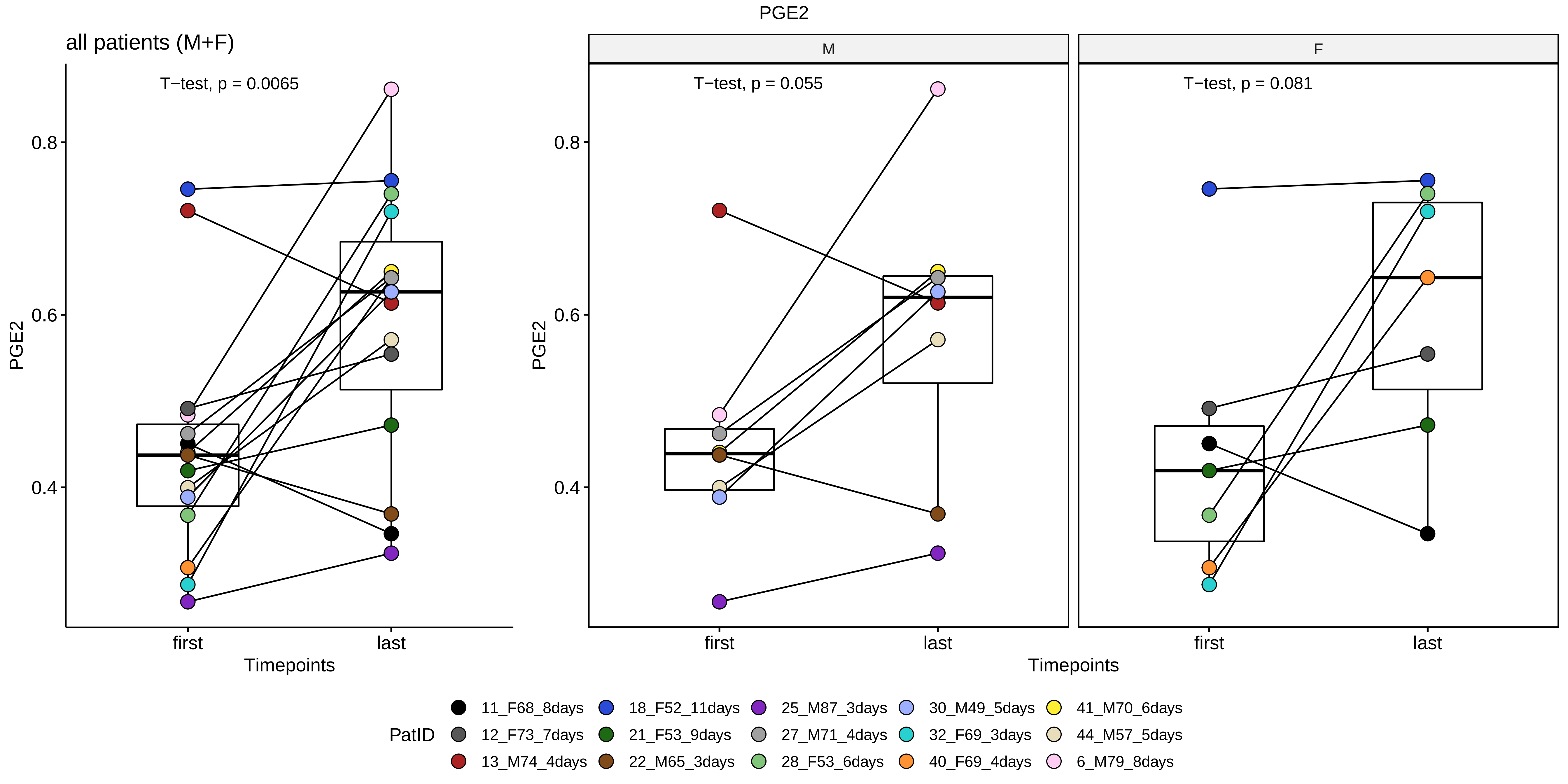
PGE2
all patients (M+F)
T−test, p = 0.0065
M
T−test, p = 0.055
F
T−test, p = 0.081
PGE2
0.8
0.6
0.4
first
last
Timepoints
PatID
11_F68_8days
12_F73_7days
13_M74_4days
18_F52_11days
21_F53_9days
22_M65_3days
25_M87_3days
27_M71_4days
28_F53_6days
30_M49_5days
32_F69_3days
40_F69_4days
41_M70_6days
44_M57_5days
6_M79_8days

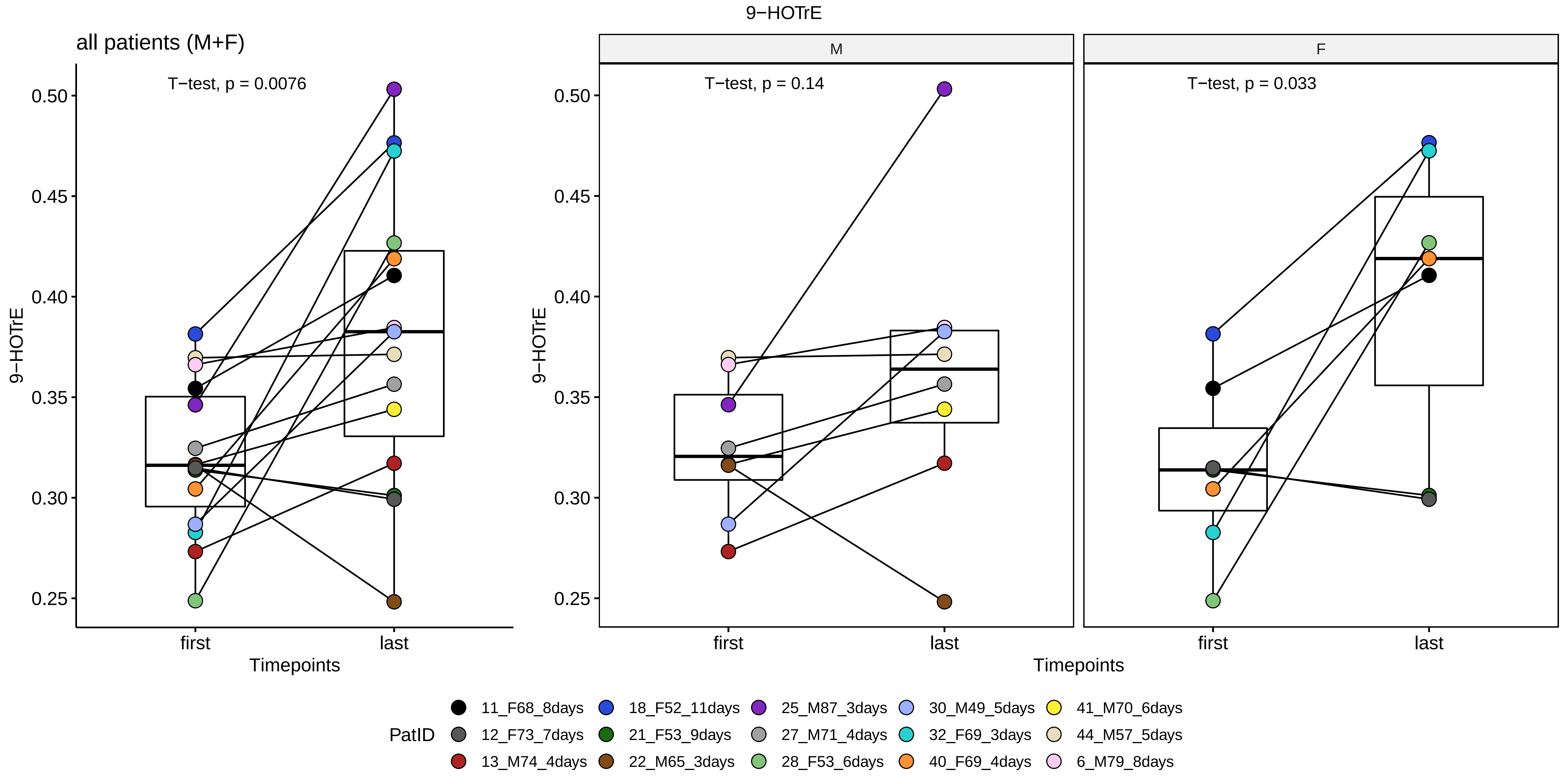

9−HOTrE
all patients (M+F)
T−test, p = 0.0076
M
T−test, p = 0.14
F
T−test, p = 0.033
0.50
0.45
0.40
0.35
0.30
0.25
9−HOTrE
first
last
Timepoints
PatID
11_F68_8days
12_F73_7days
13_M74_4days
18_F52_11days
21_F53_9days
22_M65_3days
25_M87_3days
27_M71_4days
28_F53_6days
30_M49_5days
32_F69_3days
40_F69_4days
41_M70_6days
44_M57_5days
6_M79_8days

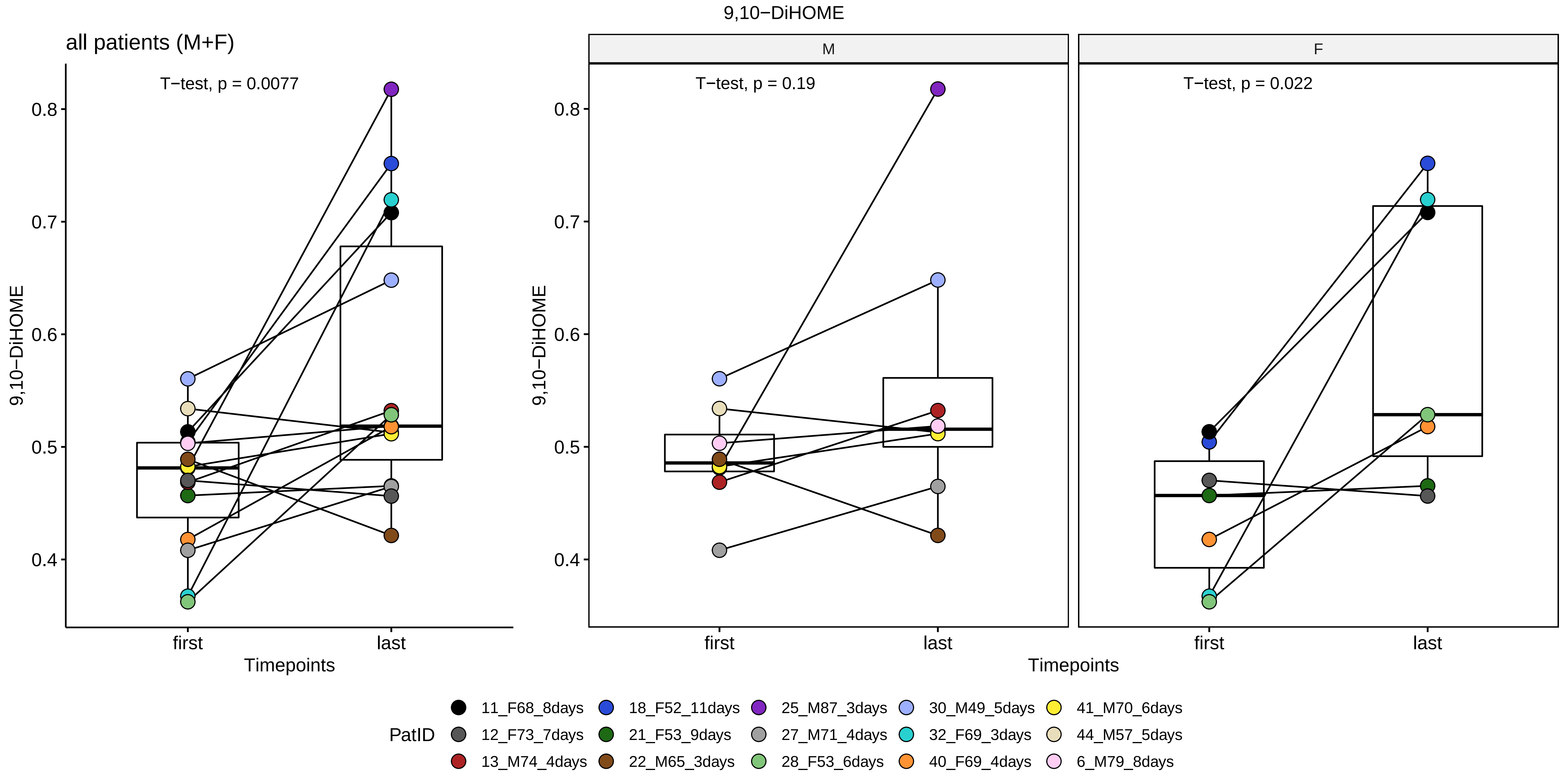
9,10−DiHOME
all patients (M+F)
T−test, p = 0.0077
M
T−test, p = 0.19
F
T−test, p = 0.022
9,10−DiHOME
0.4
0.5
0.6
0.7
0.8
first
last
Timepoints
PatID
11_F68_8days
12_F73_7days
13_M74_4days
18_F52_11days
21_F53_9days
22_M65_3days
25_M87_3days
27_M71_4days
28_F53_6days
30_M49_5days
32_F69_3days
40_F69_4days
41_M70_6days
44_M57_5days
6_M79_8days

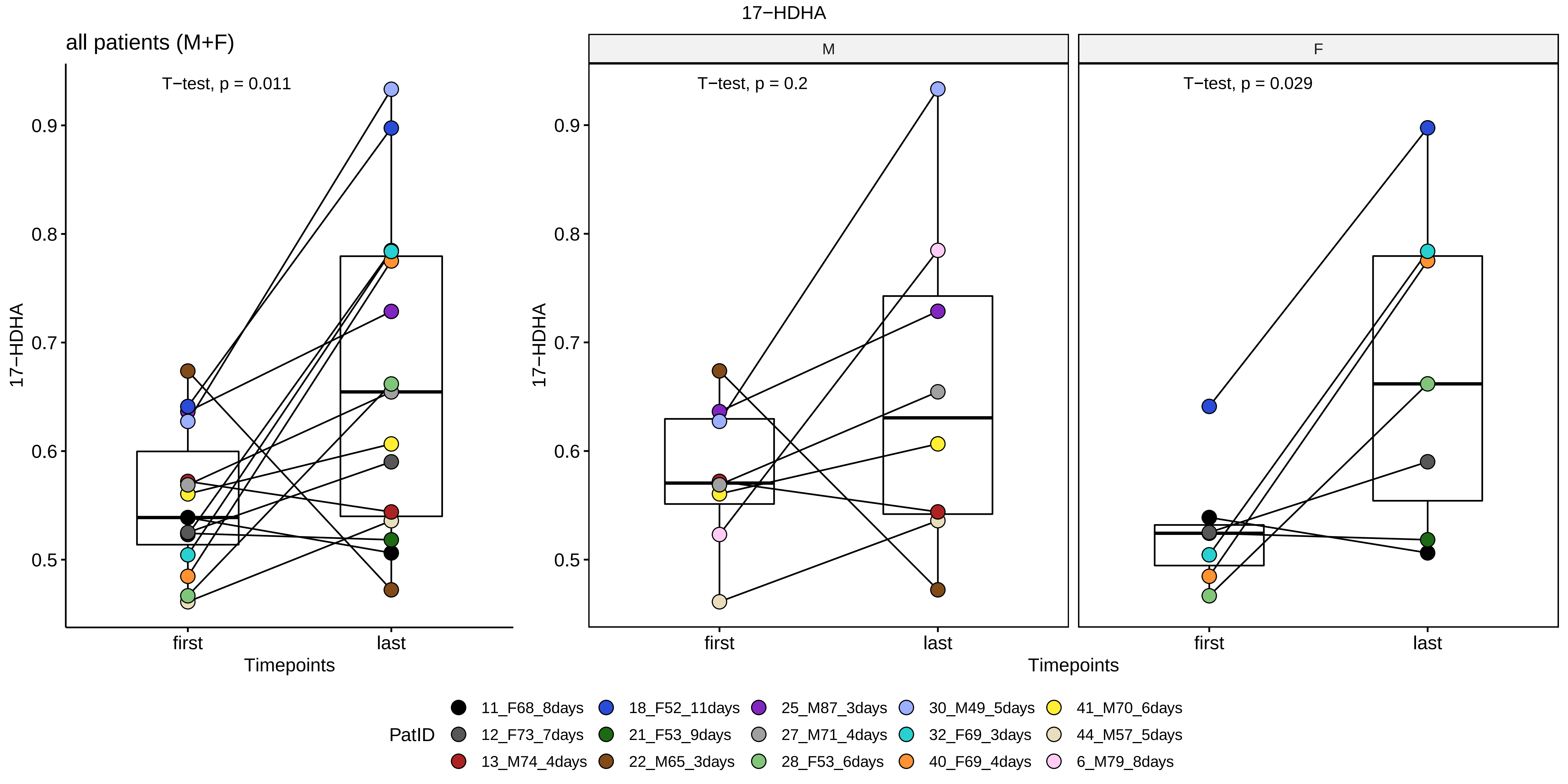

17−HDHA
all patients (M+F)
T−test, p = 0.011
M
T−test, p = 0.2
F
T−test, p = 0.029
17−HDHA
0.5
0.6
0.7
0.8
0.9
first
last
Timepoints
PatID
11_F68_8days
12_F73_7days
13_M74_4days
18_F52_11days
21_F53_9days
22_M65_3days
25_M87_3days
27_M71_4days
28_F53_6days
30_M49_5days
32_F69_3days
40_F69_4days
41_M70_6days
44_M57_5days
6_M79_8days

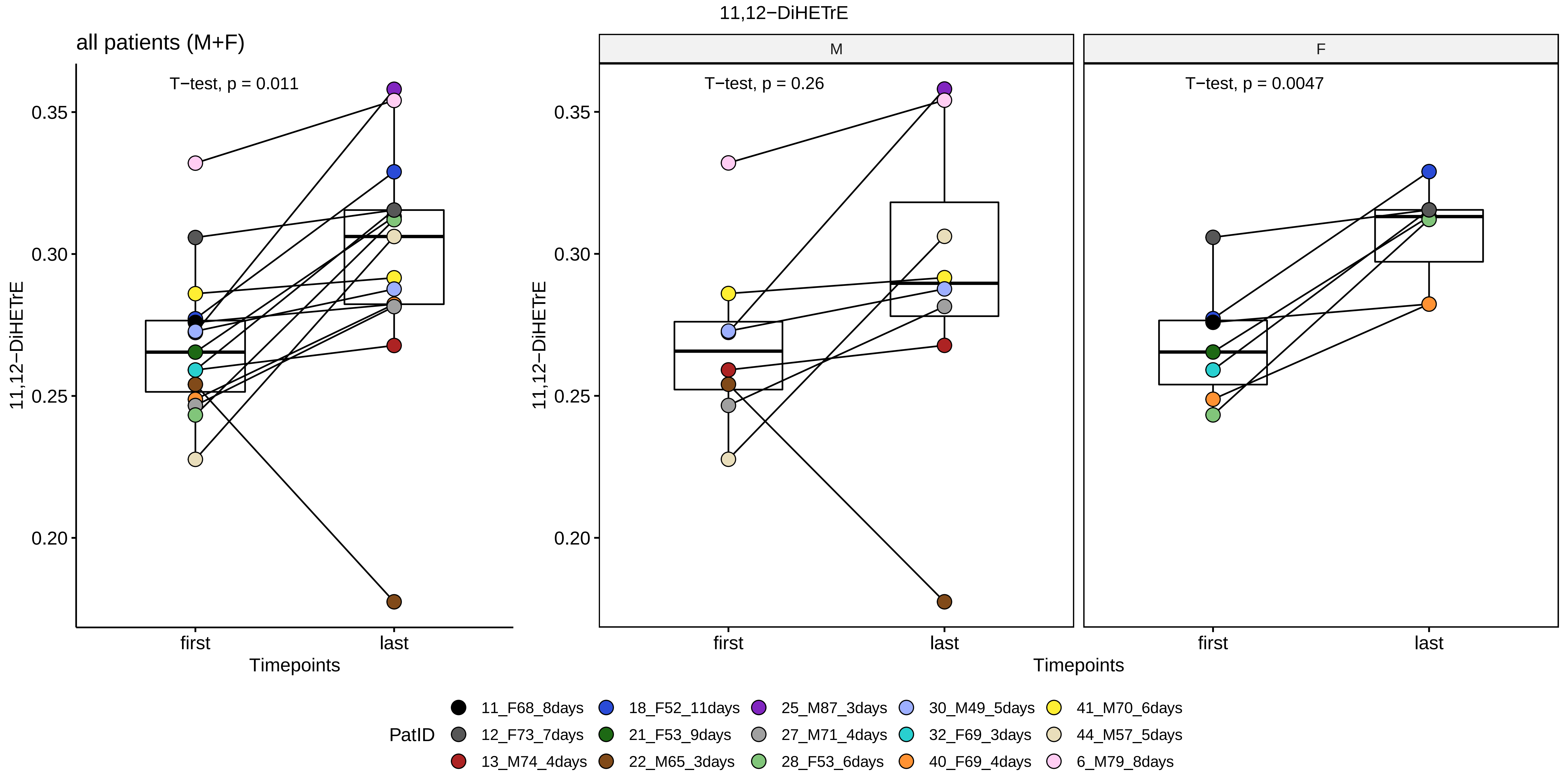

11,12−DiHETrE
all patients (M+F)
T−test, p = 0.011
M
T−test, p = 0.26
F
T−test, p = 0.0047
11,12−DiHETrE
0.35
0.30
0.25
0.20
first
last
Timepoints
PatID
11_F68_8days
12_F73_7days
13_M74_4days
18_F52_11days
21_F53_9days
22_M65_3days
25_M87_3days
27_M71_4days
28_F53_6days
30_M49_5days
32_F69_3days
40_F69_4days
41_M70_6days
44_M57_5days
6_M79_8days

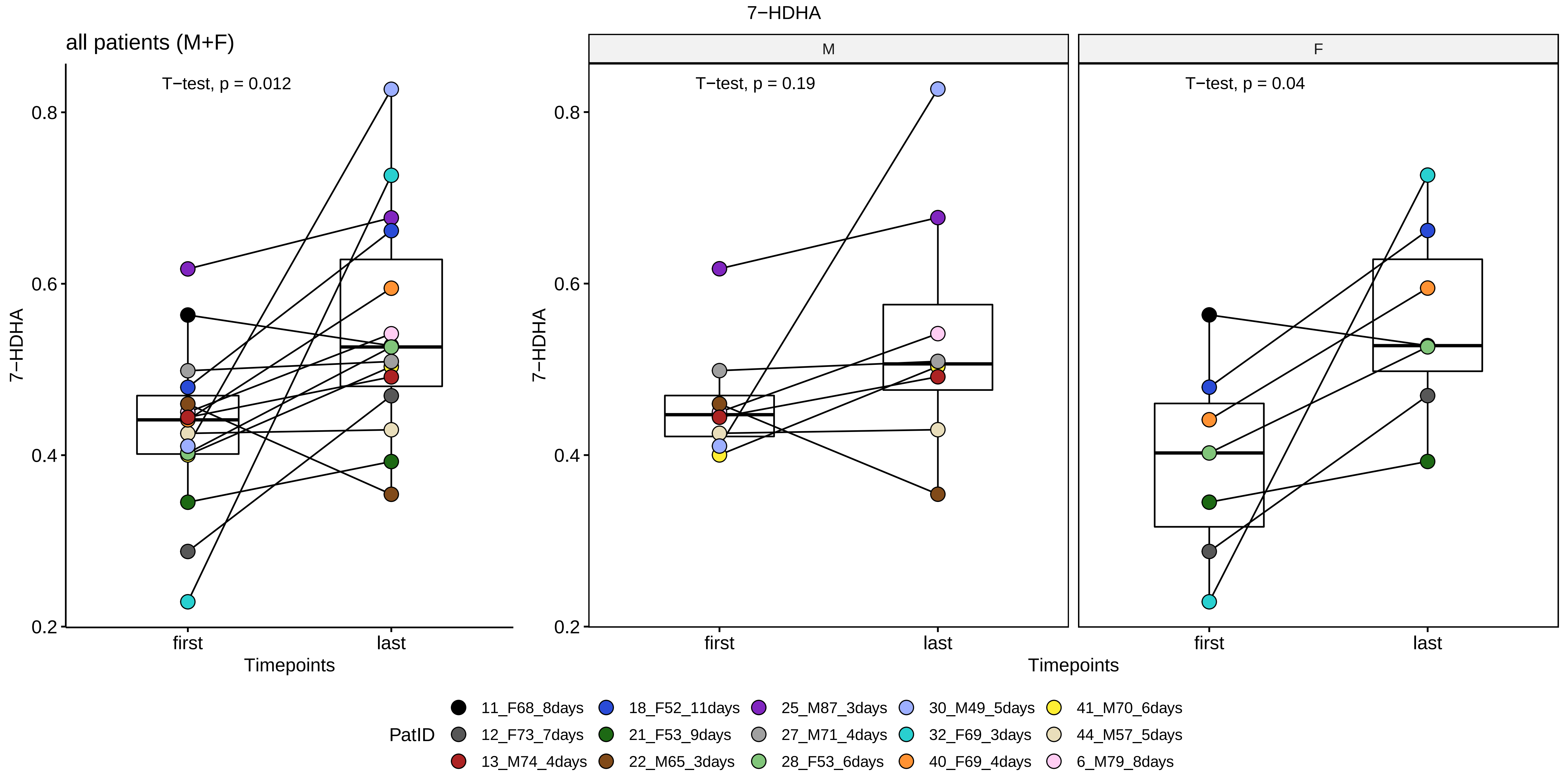
7−HDHA
all patients (M+F)
T−test, p = 0.012
M
T−test, p = 0.19
F
T−test, p = 0.04
7−HDHA
0.8
0.6
0.4
0.2
first
last
Timepoints
PatID
11_F68_8days
12_F73_7days
13_M74_4days
18_F52_11days
21_F53_9days
22_M65_3days
25_M87_3days
27_M71_4days
28_F53_6days
30_M49_5days
32_F69_3days
40_F69_4days
41_M70_6days
44_M57_5days
6_M79_8days

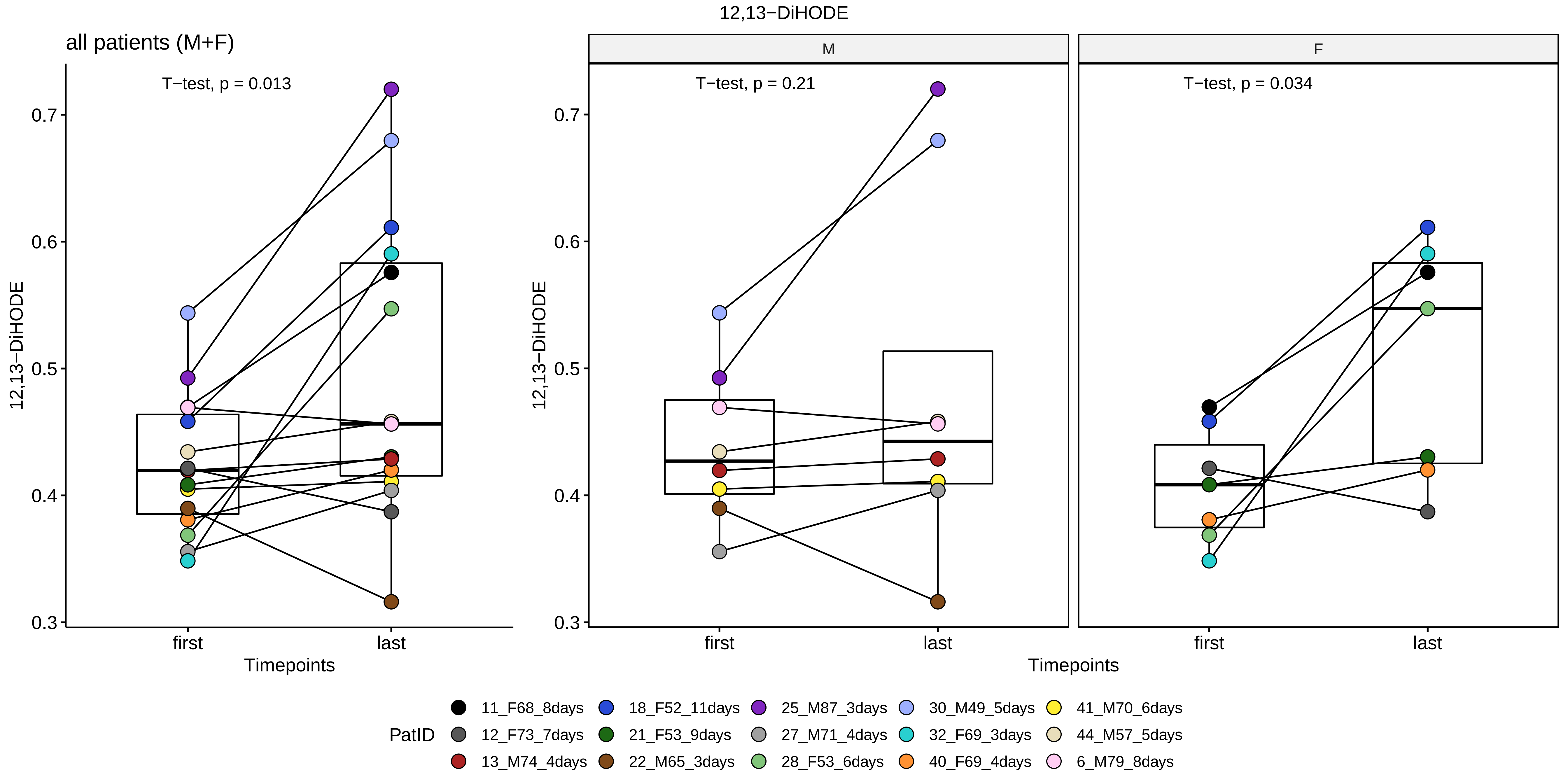
12,13−DiHODE
all patients (M+F)
T−test, p = 0.013
M
T−test, p = 0.21
F
T−test, p = 0.034
12,13−DiHODE
0.3
0.4
0.5
0.6
0.7
first
last
Timepoints
PatID
11_F68_8days
12_F73_7days
13_M74_4days
18_F52_11days
21_F53_9days
22_M65_3days
25_M87_3days
27_M71_4days
28_F53_6days
30_M49_5days
32_F69_3days
40_F69_4days
41_M70_6days
44_M57_5days
6_M79_8days

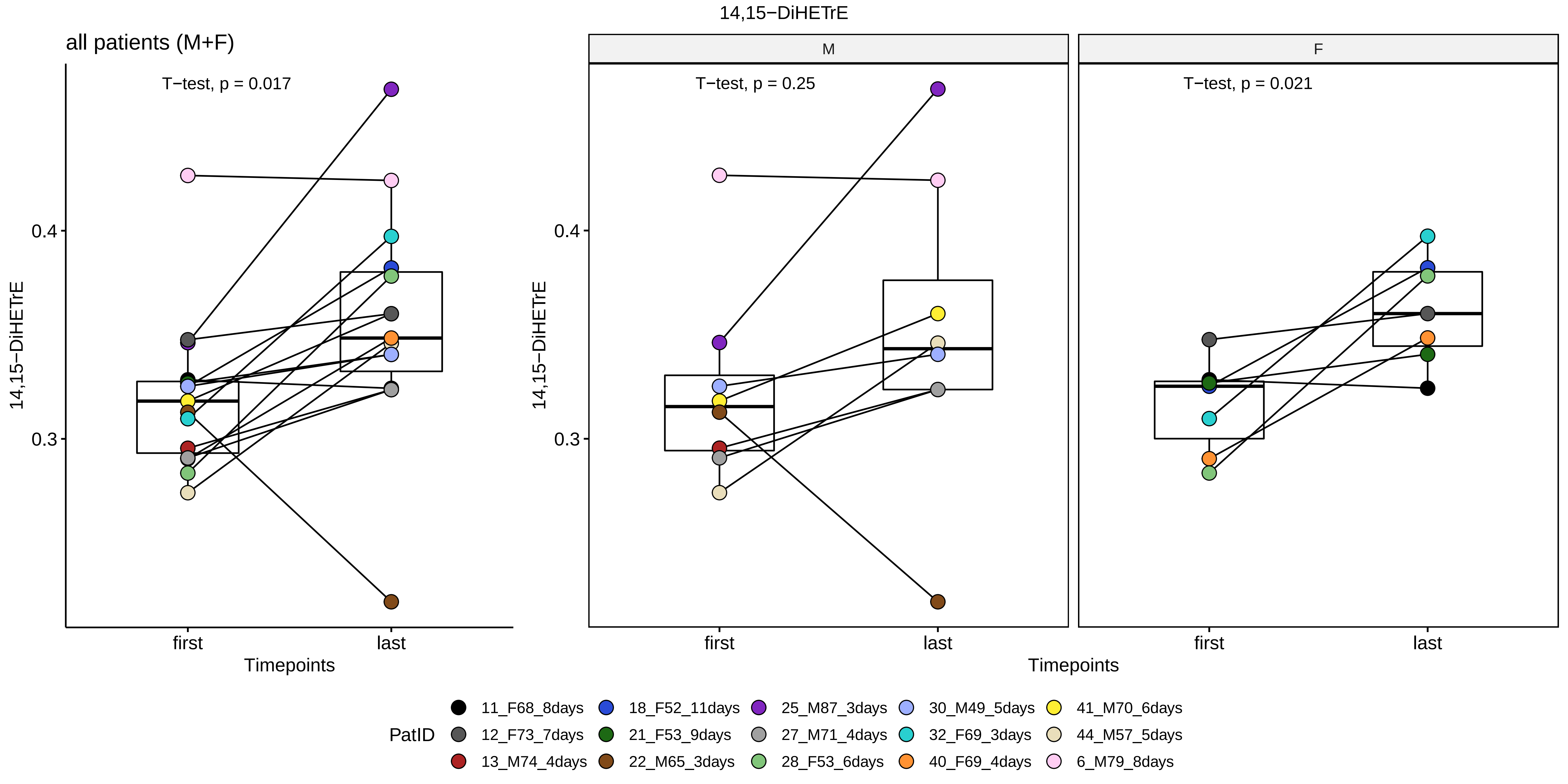

14,15−DiHETrE
all patients (M+F)
T−test, p = 0.017
M
T−test, p = 0.25
F
T−test, p = 0.021
14,15−DiHETrE
0.4
0.3
first
last
Timepoints
PatID
11_F68_8days
12_F73_7days
13_M74_4days
18_F52_11days
21_F53_9days
22_M65_3days
25_M87_3days
27_M71_4days
28_F53_6days
30_M49_5days
32_F69_3days
40_F69_4days
41_M70_6days
44_M57_5days
6_M79_8days

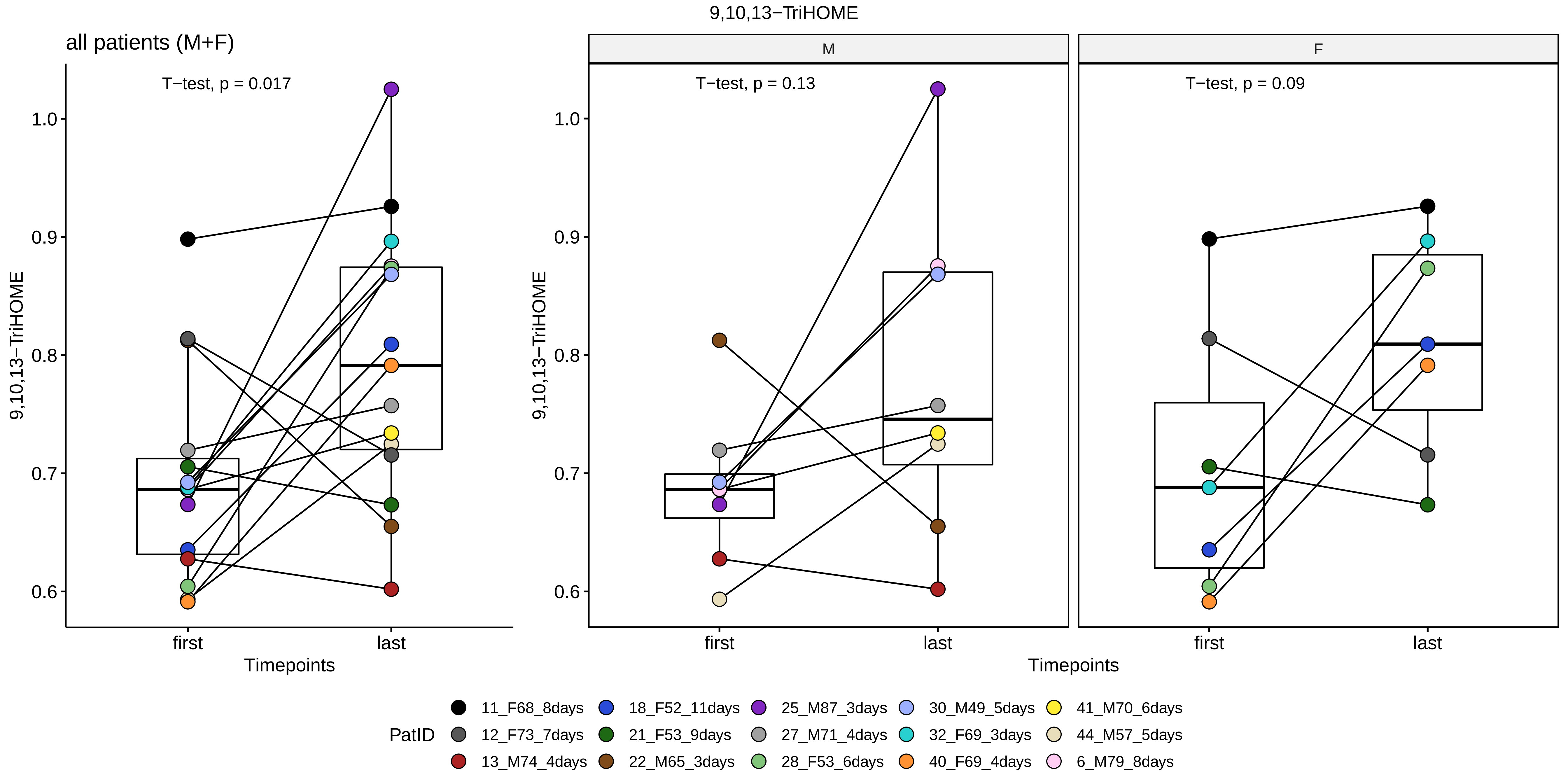

9,10,13−TriHOME
all patients (M+F)
T−test, p = 0.017
M
T−test, p = 0.13
F
T−test, p = 0.09
1.0
0.9
0.8
0.7
0.6
9,10,13−TriHOME
first
last
Timepoints
PatID
11_F68_8days
12_F73_7days
13_M74_4days
18_F52_11days
21_F53_9days
22_M65_3days
25_M87_3days
27_M71_4days
28_F53_6days
30_M49_5days
32_F69_3days
40_F69_4days
41_M70_6days
44_M57_5days
6_M79_8days

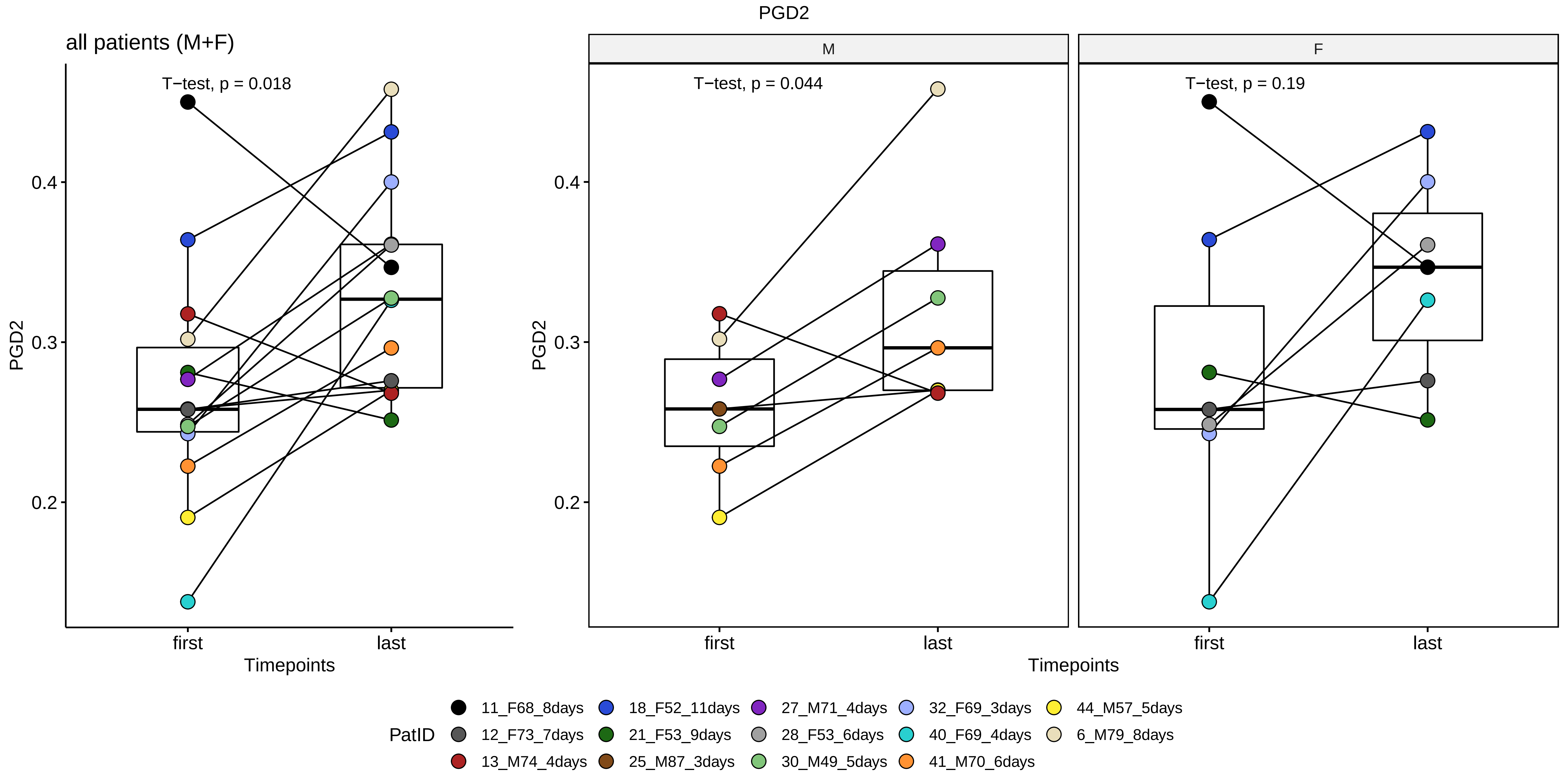

PGD2
all patients (M+F)
T−test, p = 0.018
M
T−test, p = 0.044
F
T−test, p = 0.19
0.4
0.3
0.2
PGD2
first
last
Timepoints
PatID
11_F68_8days
12_F73_7days
13_M74_4days
18_F52_11days
21_F53_9days
25_M87_3days
27_M71_4days
28_F53_6days
30_M49_5days
32_F69_3days
40_F69_4days
41_M70_6days
44_M57_5days
6_M79_8days

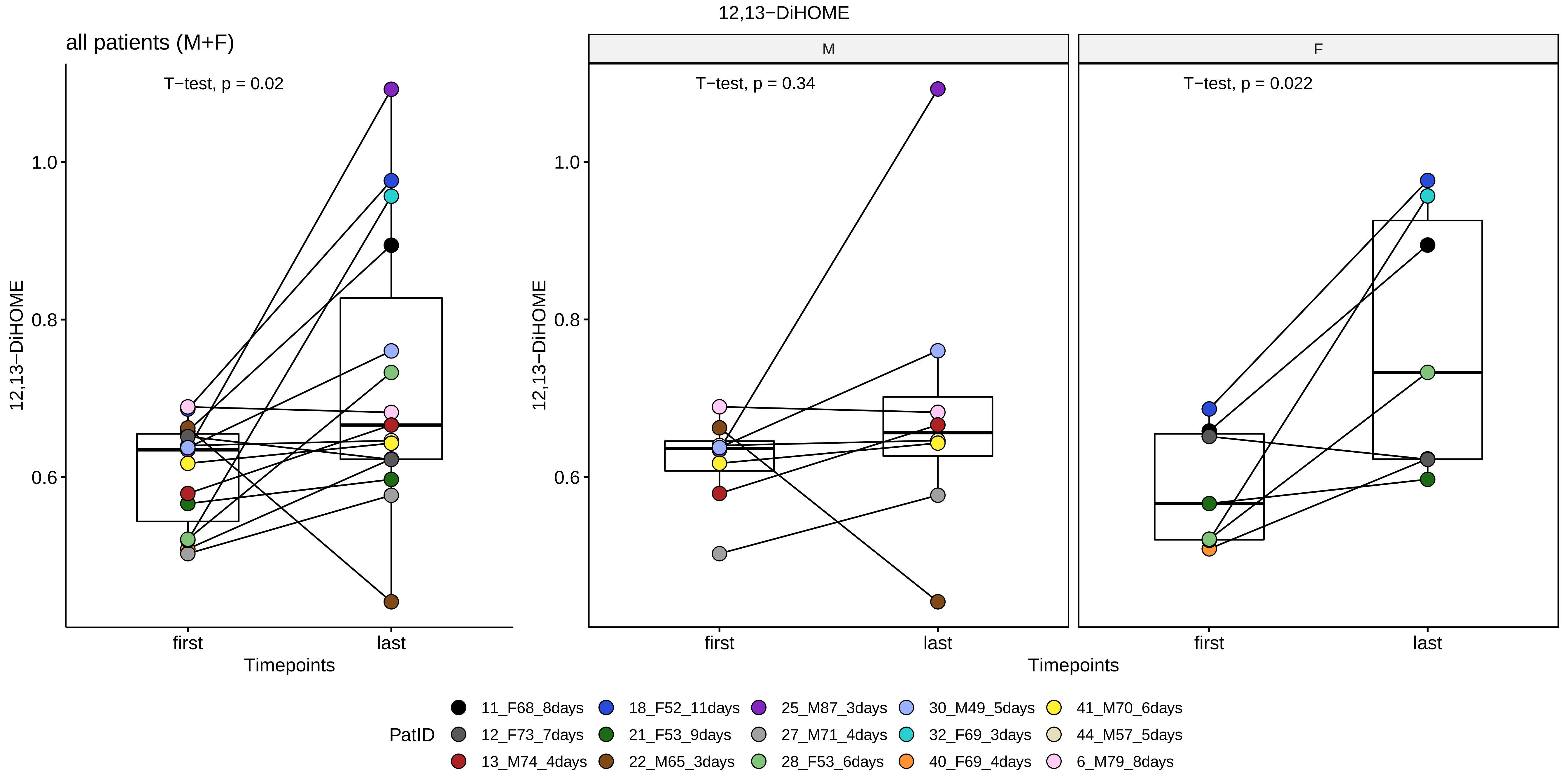
12,13−DiHOME
all patients (M+F)
T−test, p = 0.02
12,13−DiHOME
1.0
0.8
0.6
first
last
Timepoints
M
T−test, p = 0.34
F
T−test, p = 0.022
PatID
11_F68_8days
12_F73_7days
13_M74_4days
18_F52_11days
21_F53_9days
22_M65_3days
25_M87_3days
27_M71_4days
28_F53_6days
30_M49_5days
32_F69_3days
40_F69_4days
41_M70_6days
44_M57_5days
6_M79_8days

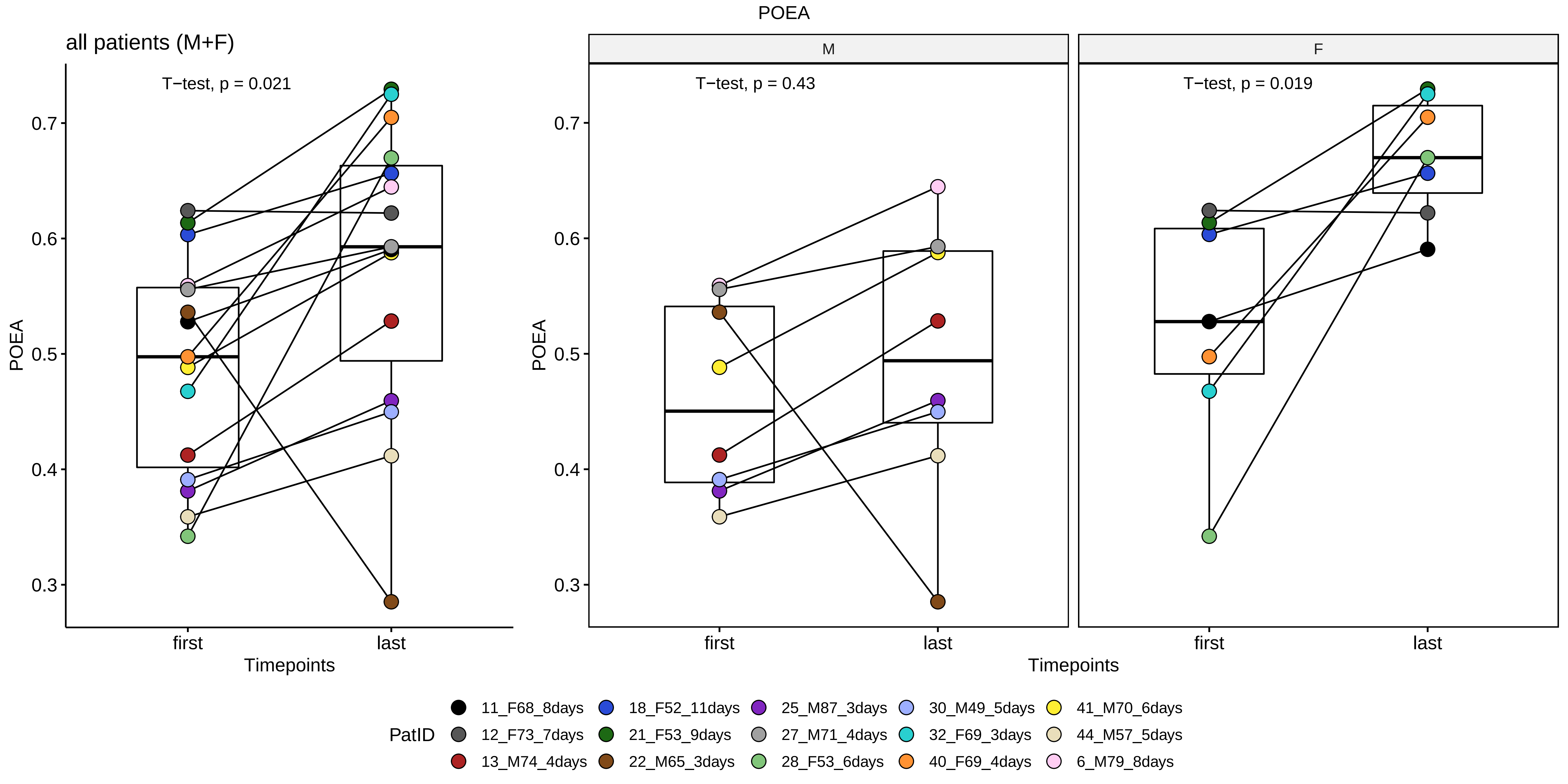
POEA
all patients (M+F)
T−test, p = 0.021
M
T−test, p = 0.43
F
T−test, p = 0.019
POEA
0.3
0.4
0.5
0.6
0.7
first
last
Timepoints
PatID
11_F68_8days
12_F73_7days
13_M74_4days
18_F52_11days
21_F53_9days
22_M65_3days
25_M87_3days
27_M71_4days
28_F53_6days
30_M49_5days
32_F69_3days
40_F69_4days
41_M70_6days
44_M57_5days
6_M79_8days

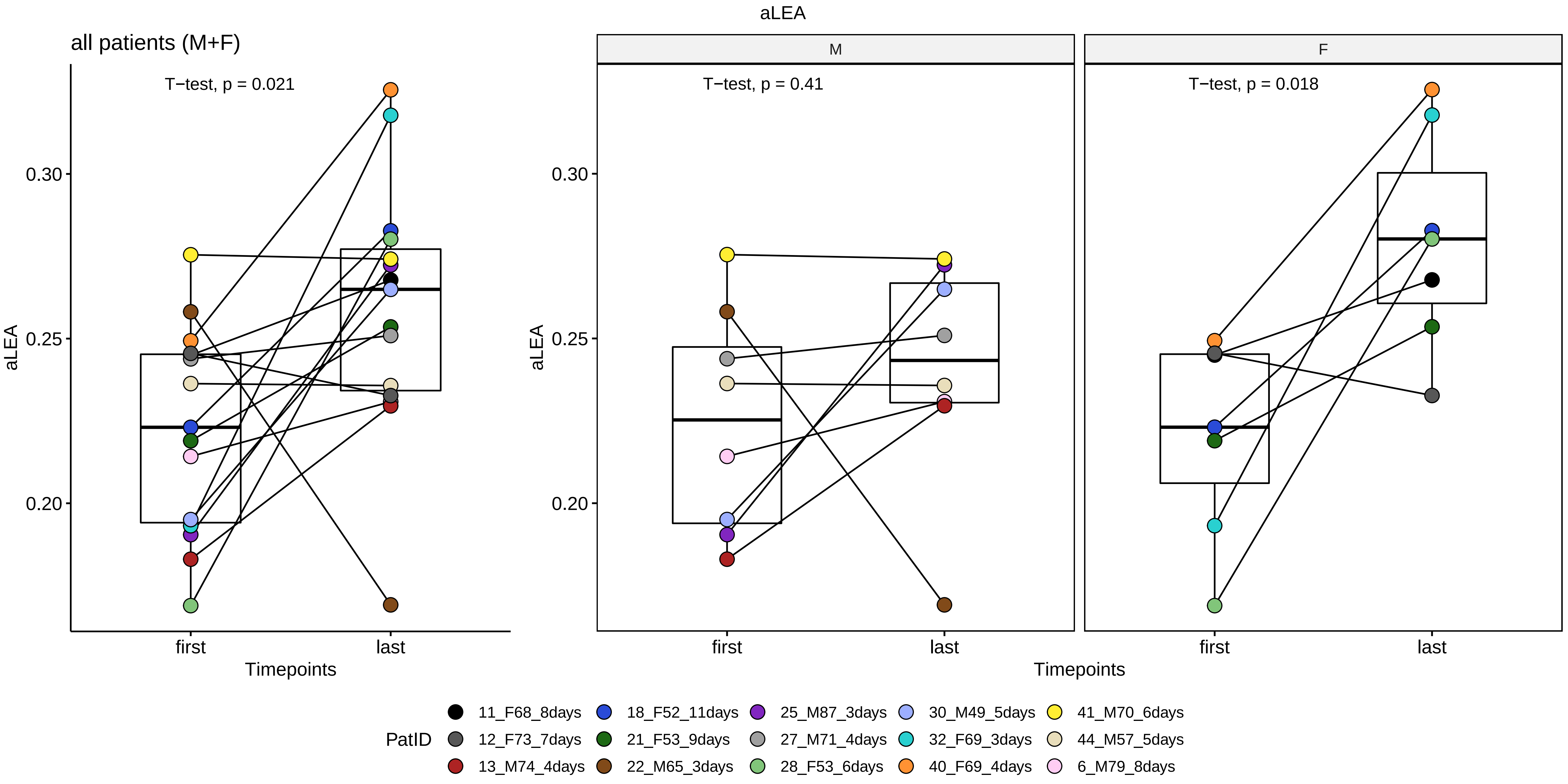
aLEA
all patients (M+F)
T−test, p = 0.021
M
T−test, p = 0.41
F
T−test, p = 0.018
aLEA
0.30
0.25
0.20
first
last
Timepoints
PatID
11_F68_8days
12_F73_7days
13_M74_4days
18_F52_11days
21_F53_9days
22_M65_3days
25_M87_3days
27_M71_4days
28_F53_6days
30_M49_5days
32_F69_3days
40_F69_4days
41_M70_6days
44_M57_5days
6_M79_8days

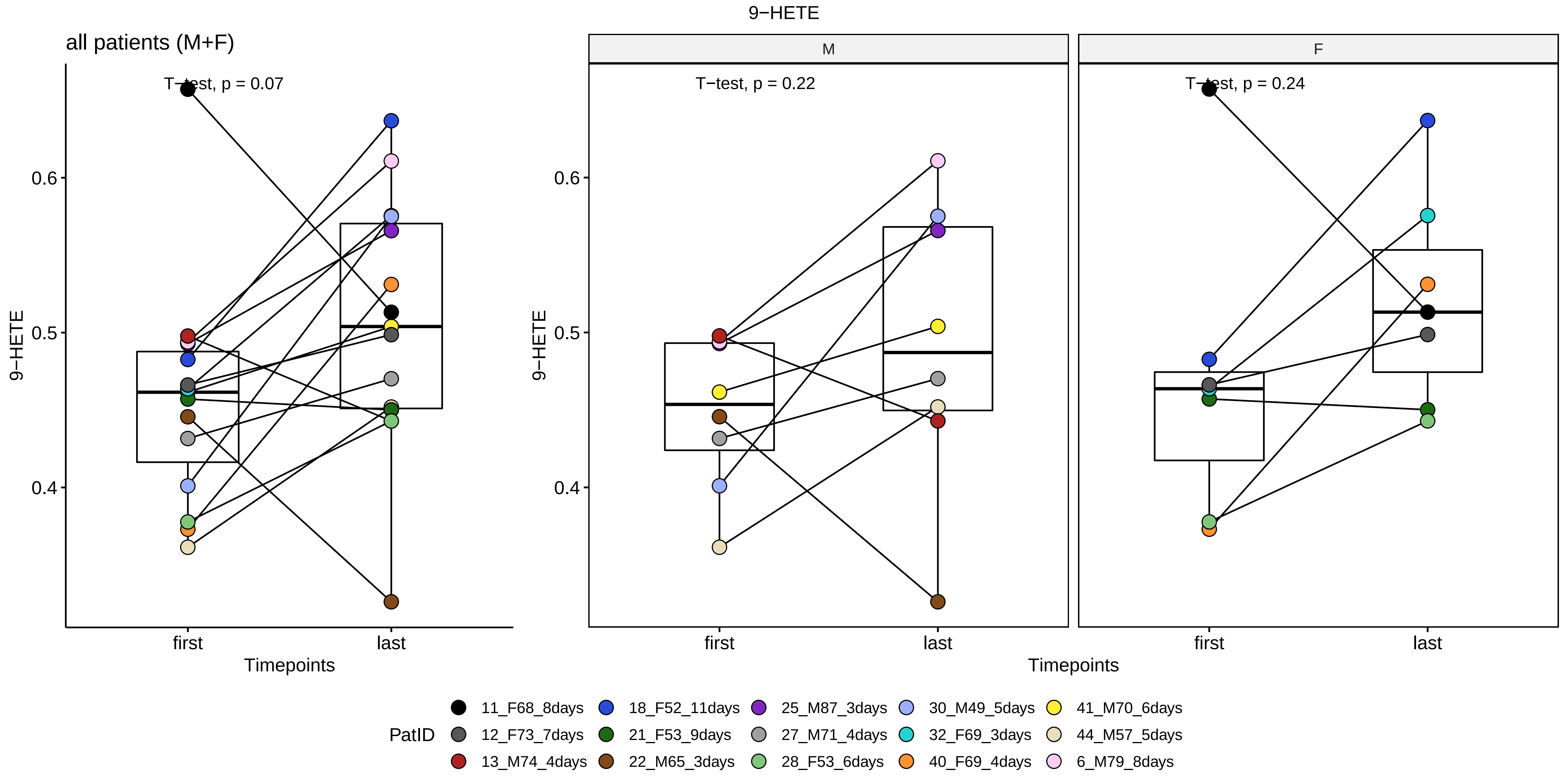

9−HETE
all patients (M+F)
T−test, p = 0.07
M
T−test, p = 0.22
F
T−test, p = 0.24
0.6
0.5
0.4
9−HETE
first
last
Timepoints
PatID
11_F68_8days
12_F73_7days
13_M74_4days
18_F52_11days
21_F53_9days
22_M65_3days
25_M87_3days
27_M71_4days
28_F53_6days
30_M49_5days
32_F69_3days
40_F69_4days
41_M70_6days
44_M57_5days
6_M79_8days

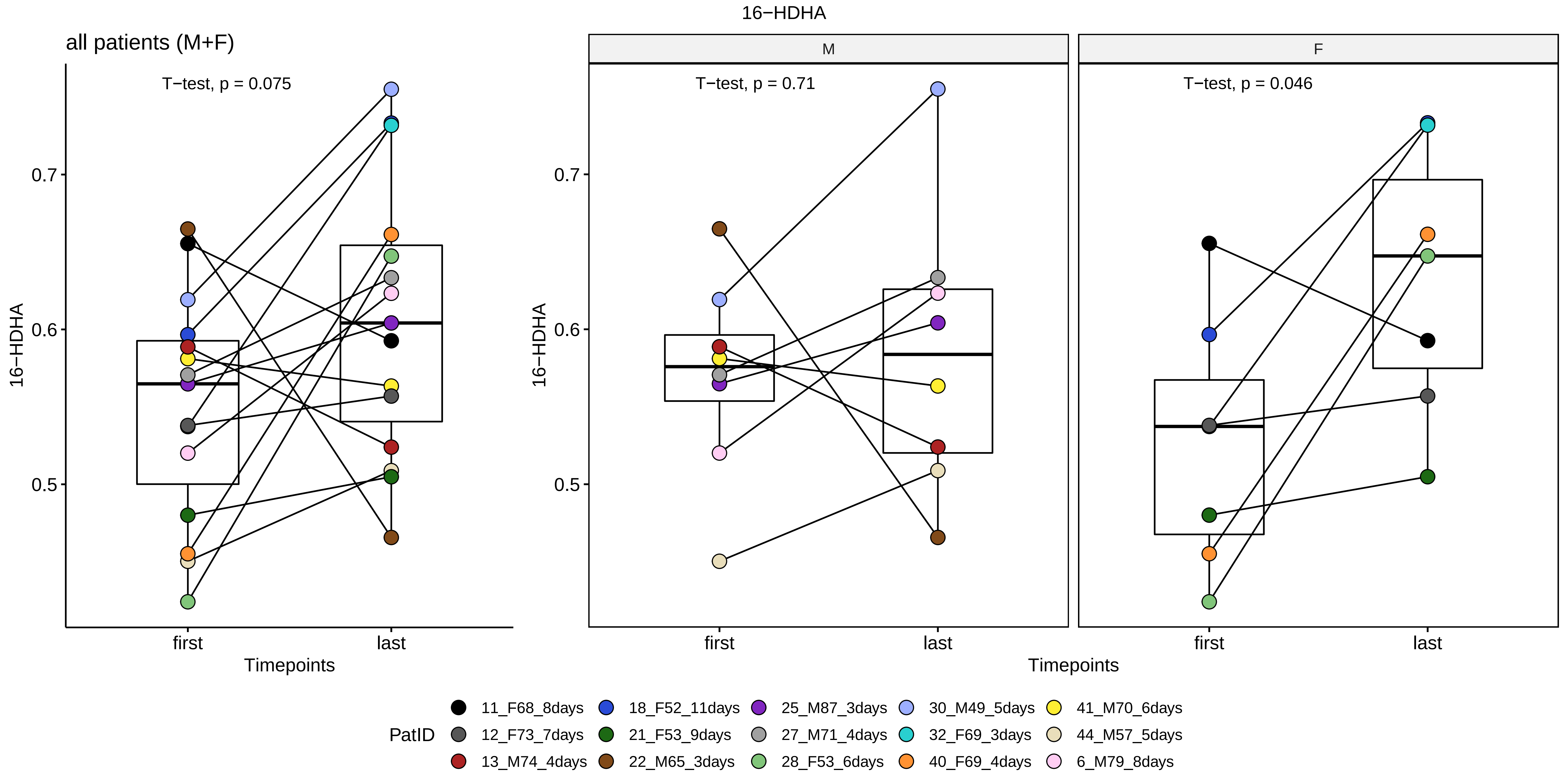

16−HDHA
all patients (M+F)
T−test, p = 0.075
0.7
0.6
0.5
16−HDHA
first
last
Timepoints
M
T−test, p = 0.71
F
T−test, p = 0.046
PatID
11_F68_8days
12_F73_7days
13_M74_4days
18_F52_11days
21_F53_9days
22_M65_3days
25_M87_3days
27_M71_4days
28_F53_6days
30_M49_5days
32_F69_3days
40_F69_4days
41_M70_6days
44_M57_5days
6_M79_8days

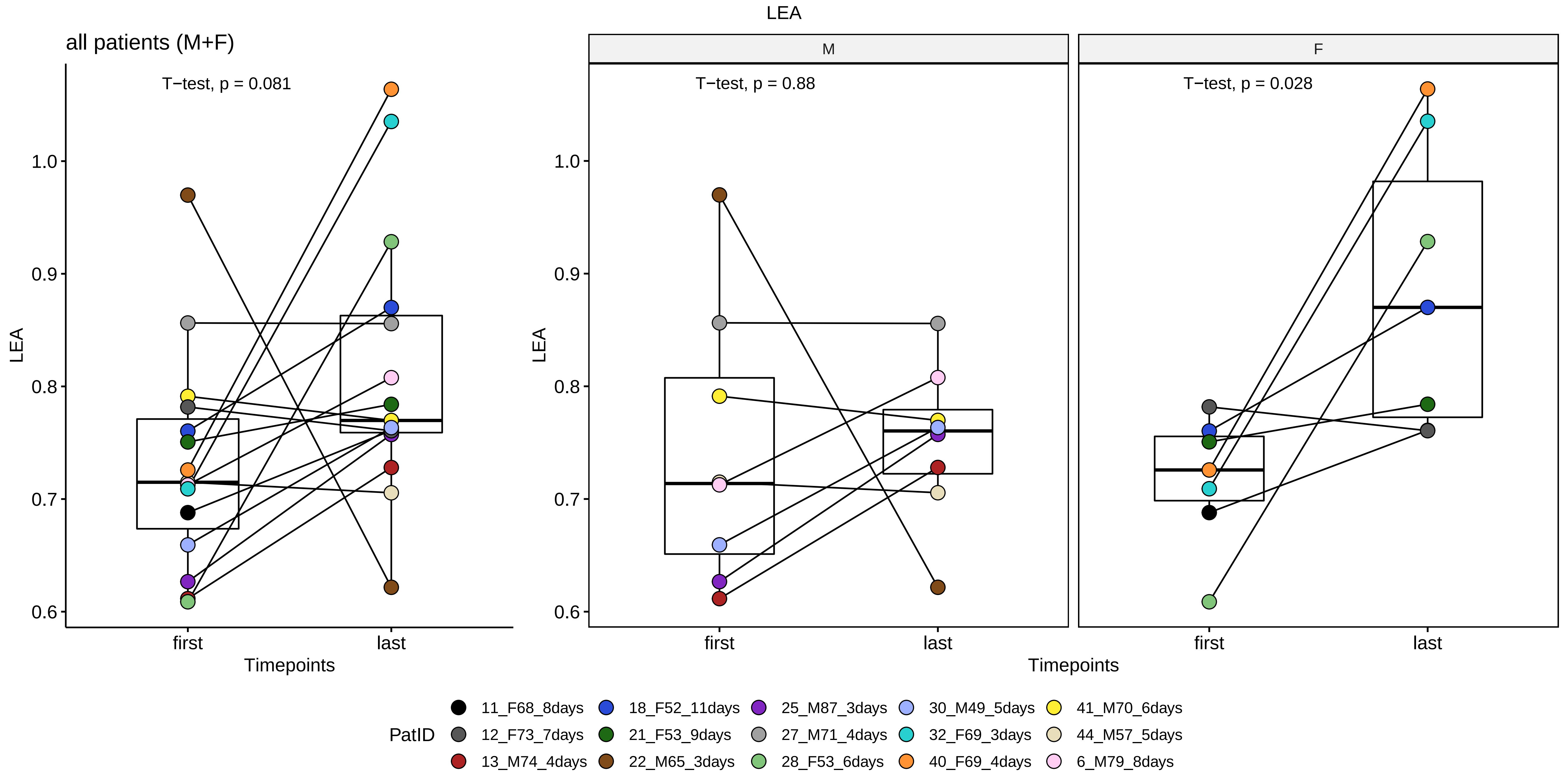
LEA
all patients (M+F)
T−test, p = 0.081
M
T−test, p = 0.88
F
T−test, p = 0.028
LEA
1.0
0.9
0.8
0.7
0.6
first
last
Timepoints
PatID
11_F68_8days
12_F73_7days
13_M74_4days
18_F52_11days
21_F53_9days
22_M65_3days
25_M87_3days
27_M71_4days
28_F53_6days
30_M49_5days
32_F69_3days
40_F69_4days
41_M70_6days
44_M57_5days
6_M79_8days

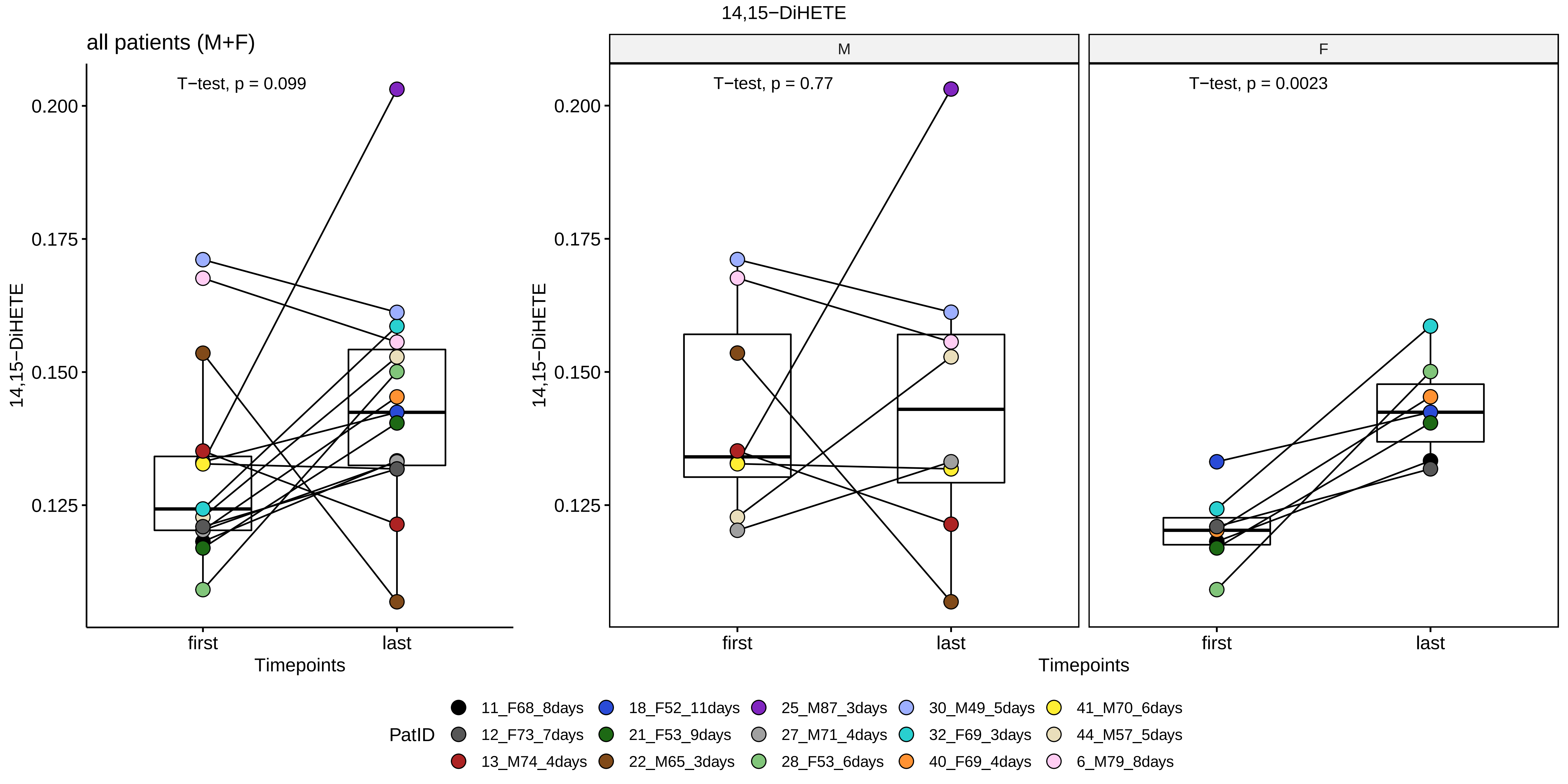
14,15−DiHETE
all patients (M+F)
T−test, p = 0.099
M
T−test, p = 0.77
F
T−test, p = 0.0023
0.200
0.175
0.150
0.125
14,15−DiHETE
first
last
Timepoints
PatID
11_F68_8days
12_F73_7days
13_M74_4days
18_F52_11days
21_F53_9days
22_M65_3days
25_M87_3days
27_M71_4days
28_F53_6days
30_M49_5days
32_F69_3days
40_F69_4days
41_M70_6days
44_M57_5days
6_M79_8days

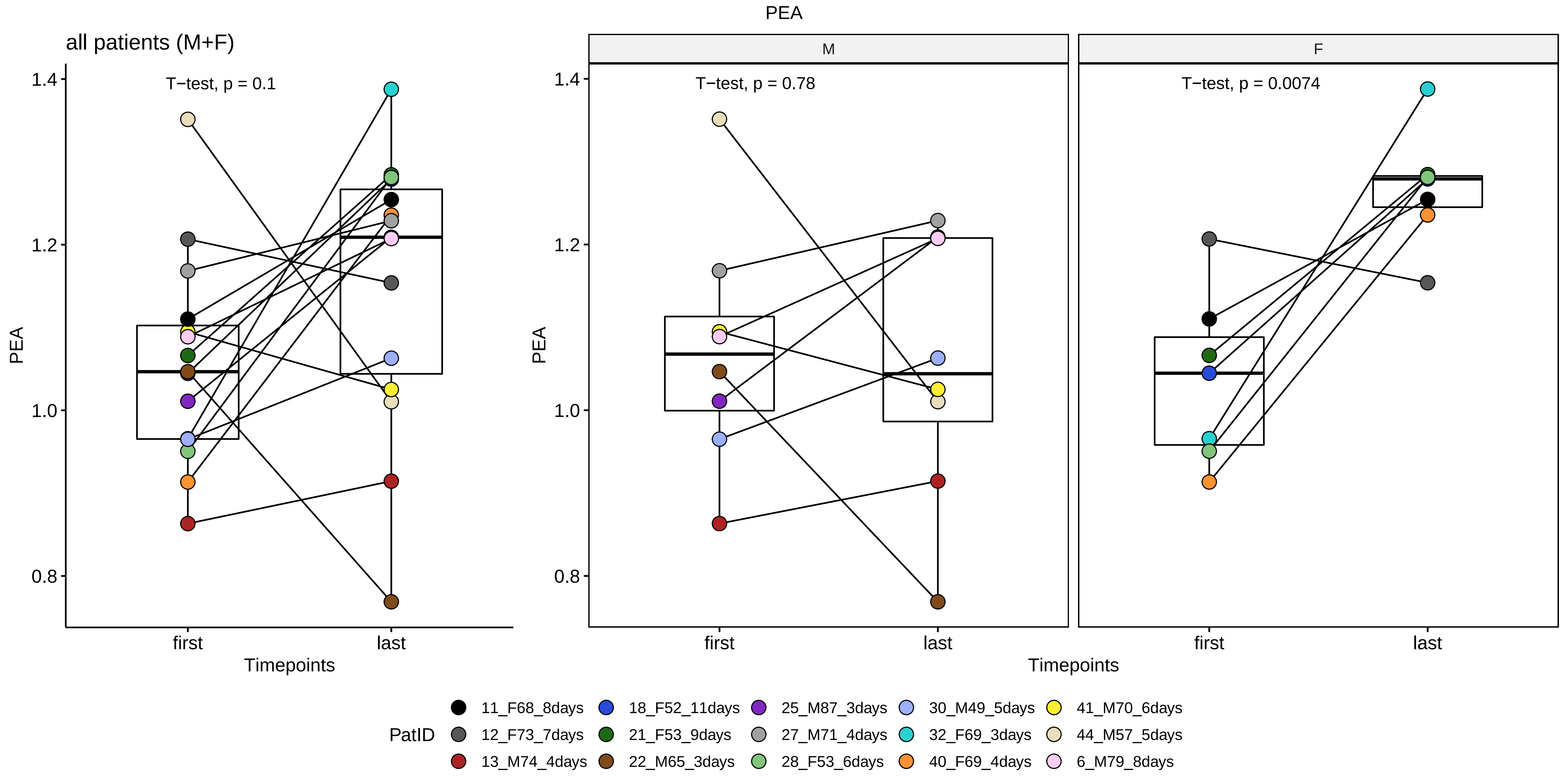
PEA
all patients (M+F)
T−test, p = 0.1
M
T−test, p = 0.78
F
T−test, p = 0.0074
1.4
1.2
1.0
0.8
PEA
first
last
Timepoints
PatID
11_F68_8days
12_F73_7days
13_M74_4days
18_F52_11days
21_F53_9days
22_M65_3days
25_M87_3days
27_M71_4days
28_F53_6days
30_M49_5days
32_F69_3days
40_F69_4days
41_M70_6days
44_M57_5days
6_M79_8days

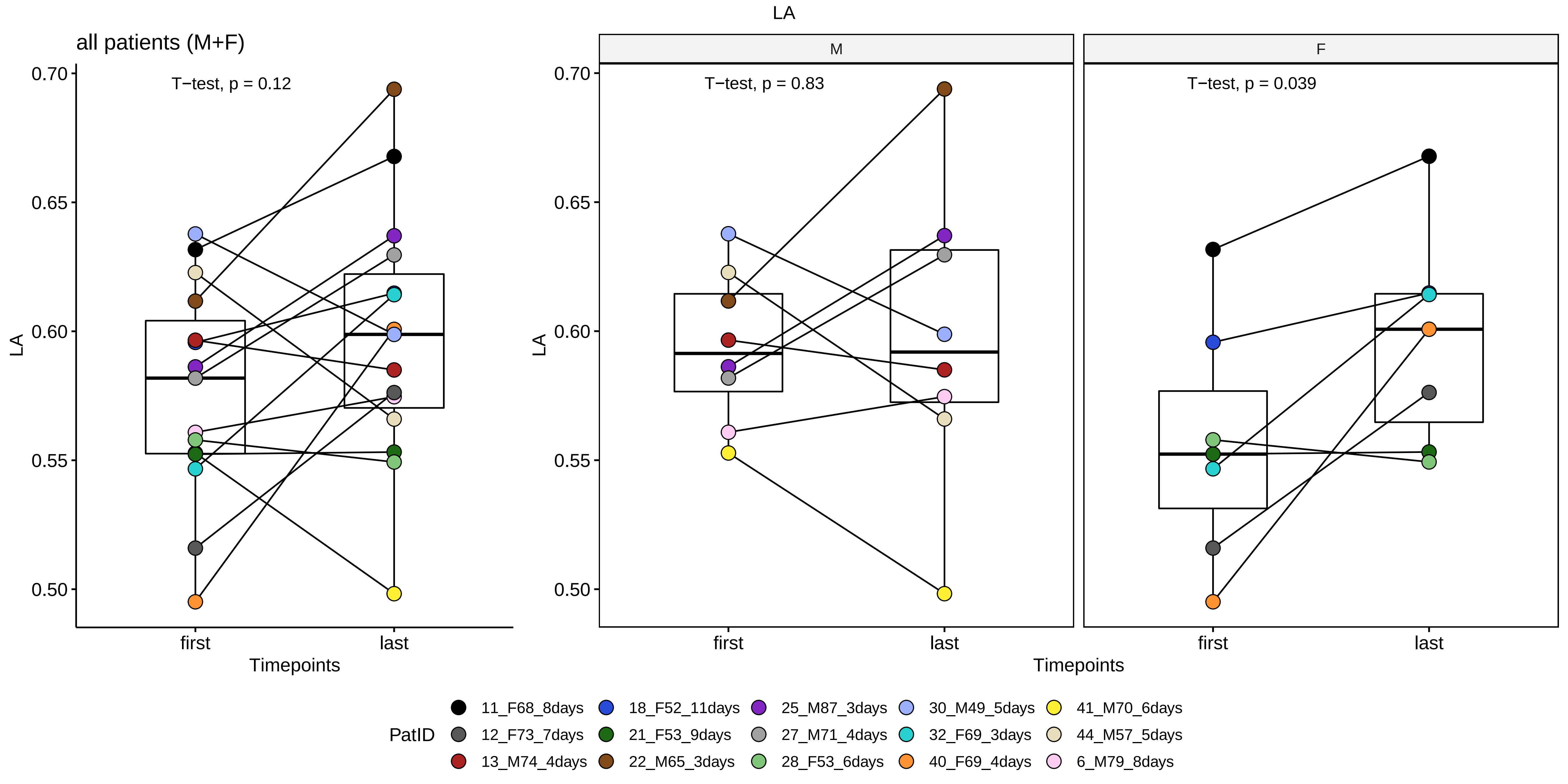

LA
all patients (M+F)
T−test, p = 0.12
M
T−test, p = 0.83
F
T−test, p = 0.039
0.70
0.65
0.60
0.55
0.50
LA
first
last
Timepoints
PatID
11_F68_8days
12_F73_7days
13_M74_4days
18_F52_11days
21_F53_9days
22_M65_3days
25_M87_3days
27_M71_4days
28_F53_6days
30_M49_5days
32_F69_3days
40_F69_4days
41_M70_6days
44_M57_5days
6_M79_8days

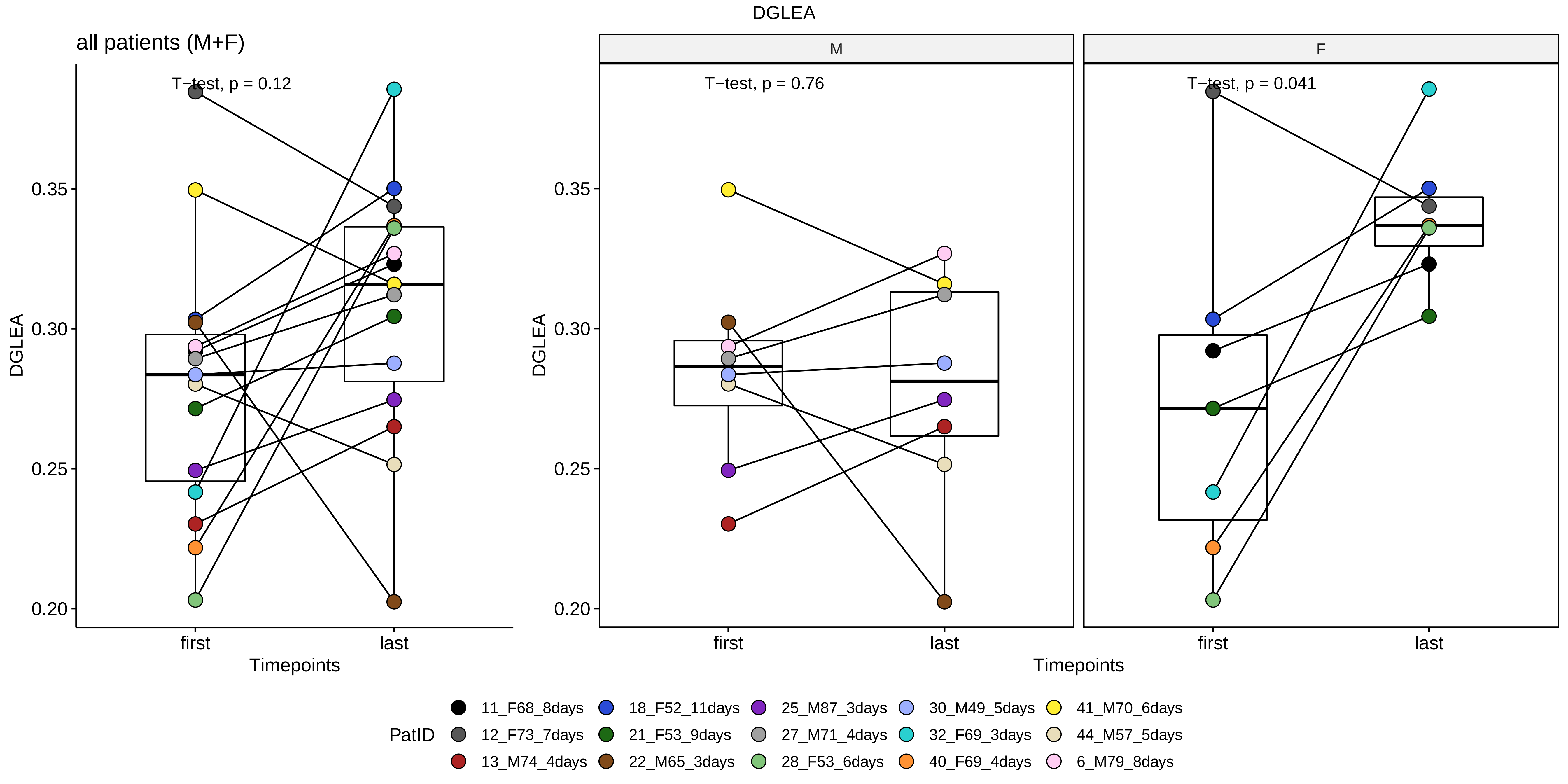

DGLEA
all patients (M+F)
T−test, p = 0.12
M
T−test, p = 0.76
F
T−test, p = 0.041
0.35
0.30
0.25
0.20
DGLEA
first
last
Timepoints
PatID
11_F68_8days
12_F73_7days
13_M74_4days
18_F52_11days
21_F53_9days
22_M65_3days
25_M87_3days
27_M71_4days
28_F53_6days
30_M49_5days
32_F69_3days
40_F69_4days
41_M70_6days
44_M57_5days
6_M79_8days

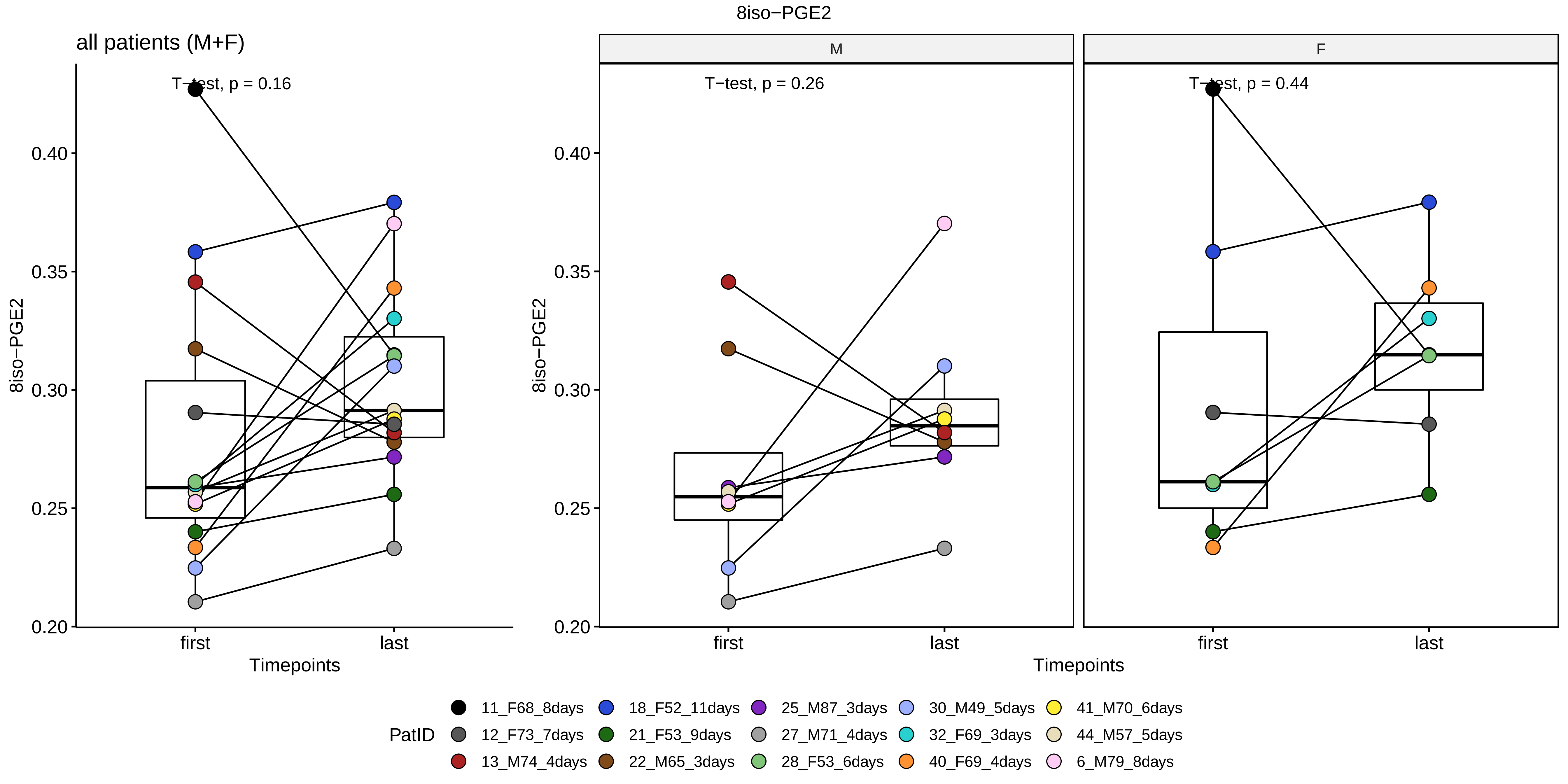

8iso−PGE2
all patients (M+F)
M
F
T−test, p = 0.16
T−test, p = 0.26
T−test, p = 0.44
8iso−PGE2
0.20
0.25
0.30
0.35
0.40
first
last
Timepoints
PatID
11_F68_8days
12_F73_7days
13_M74_4days
18_F52_11days
21_F53_9days
22_M65_3days
25_M87_3days
27_M71_4days
28_F53_6days
30_M49_5days
32_F69_3days
40_F69_4days
41_M70_6days
44_M57_5days
6_M79_8days

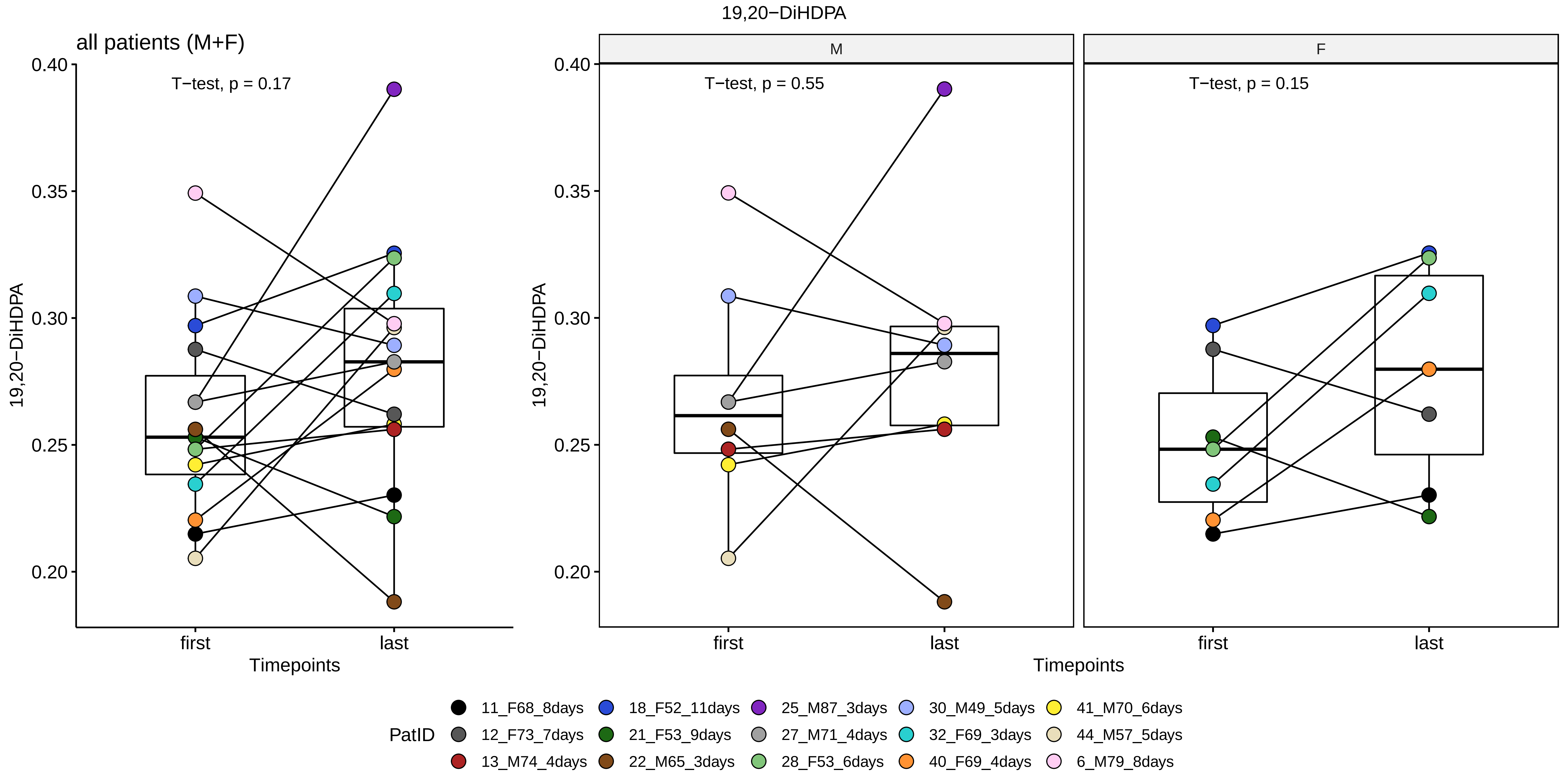

19,20−DiHDPA
all patients (M+F)
T−test, p = 0.17
M
F
T−test, p = 0.55
T−test, p = 0.15
19,20−DiHDPA
0.40
0.35
0.30
0.25
0.20
first
last
Timepoints
PatID
11_F68_8days
12_F73_7days
13_M74_4days
18_F52_11days
21_F53_9days
22_M65_3days
25_M87_3days
27_M71_4days
28_F53_6days
30_M49_5days
32_F69_3days
40_F69_4days
41_M70_6days
44_M57_5days
6_M79_8days

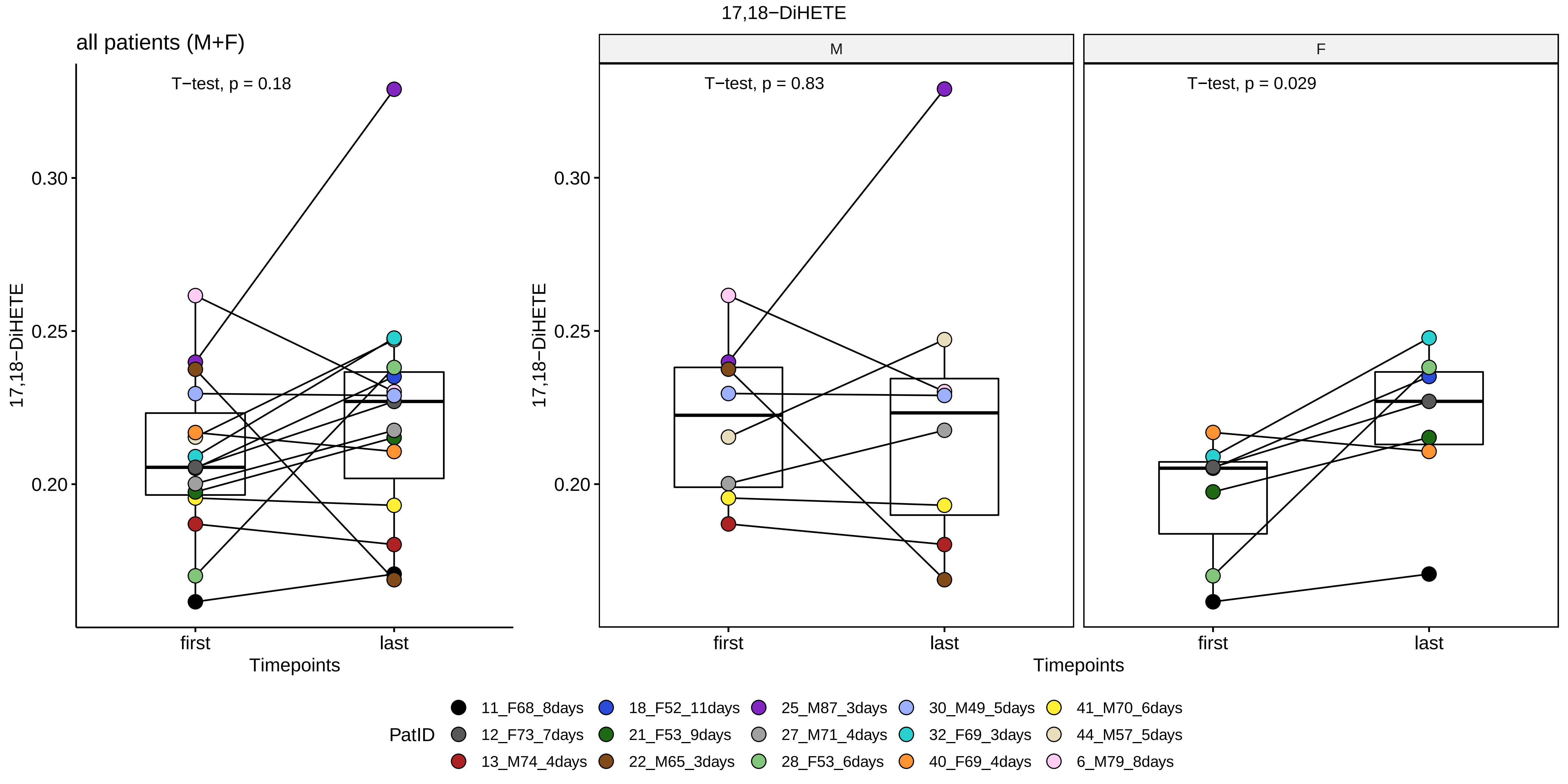

17,18−DiHETE
all patients (M+F)
T−test, p = 0.18
M
T−test, p = 0.83
F
T−test, p = 0.029
17,18−DiHETE
0.30
0.25
0.20
first
last
Timepoints
PatID
11_F68_8days
12_F73_7days
13_M74_4days
18_F52_11days
21_F53_9days
22_M65_3days
25_M87_3days
27_M71_4days
28_F53_6days
30_M49_5days
32_F69_3days
40_F69_4days
41_M70_6days
44_M57_5days
6_M79_8days

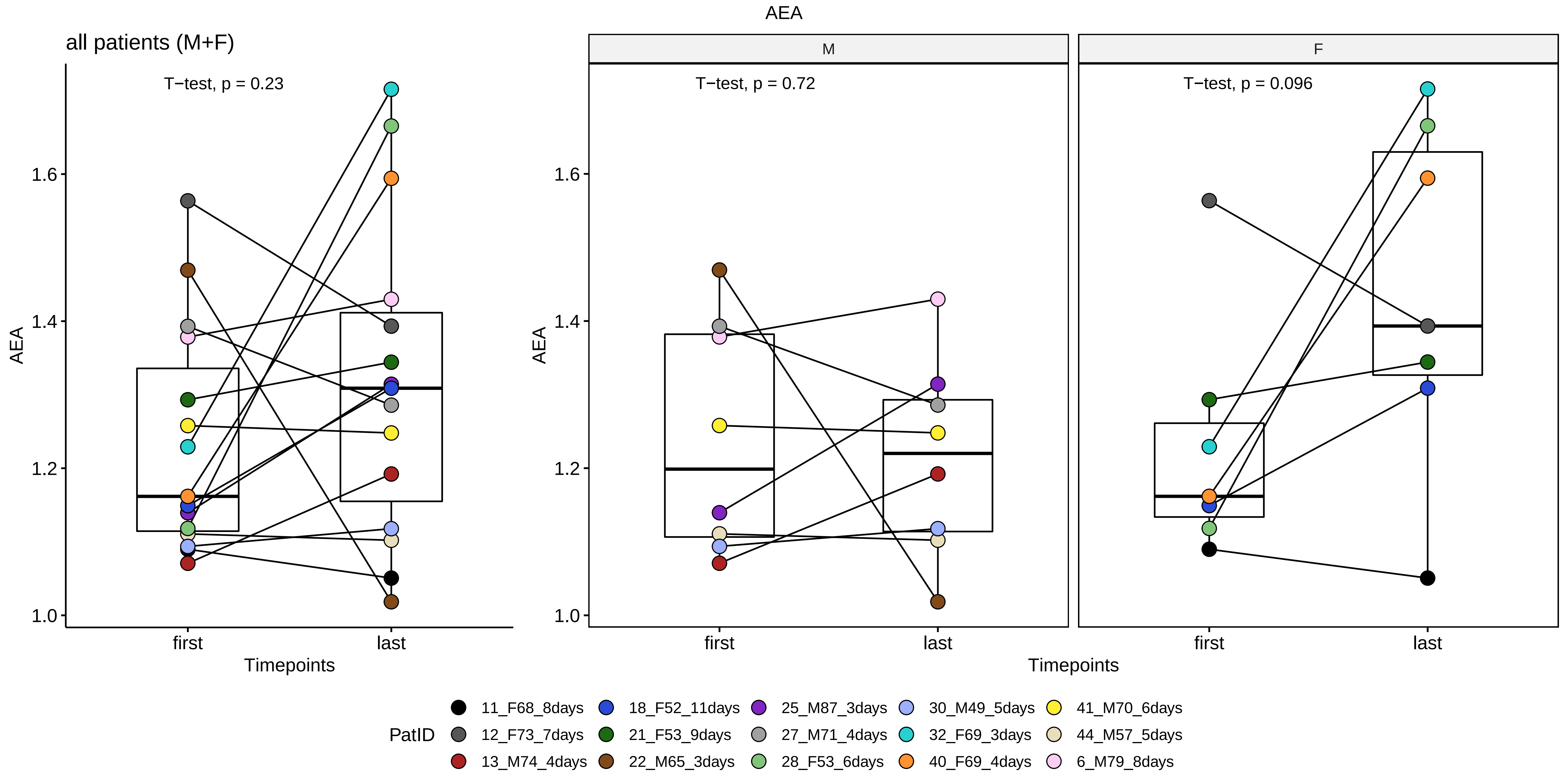

AEA
all patients (M+F)
T−test, p = 0.23
M
T−test, p = 0.72
F
T−test, p = 0.096
AEA
1.0
1.2
1.4
1.6
first
last
Timepoints
PatID
11_F68_8days
12_F73_7days
13_M74_4days
18_F52_11days
21_F53_9days
22_M65_3days
25_M87_3days
27_M71_4days
28_F53_6days
30_M49_5days
32_F69_3days
40_F69_4days
41_M70_6days
44_M57_5days
6_M79_8days

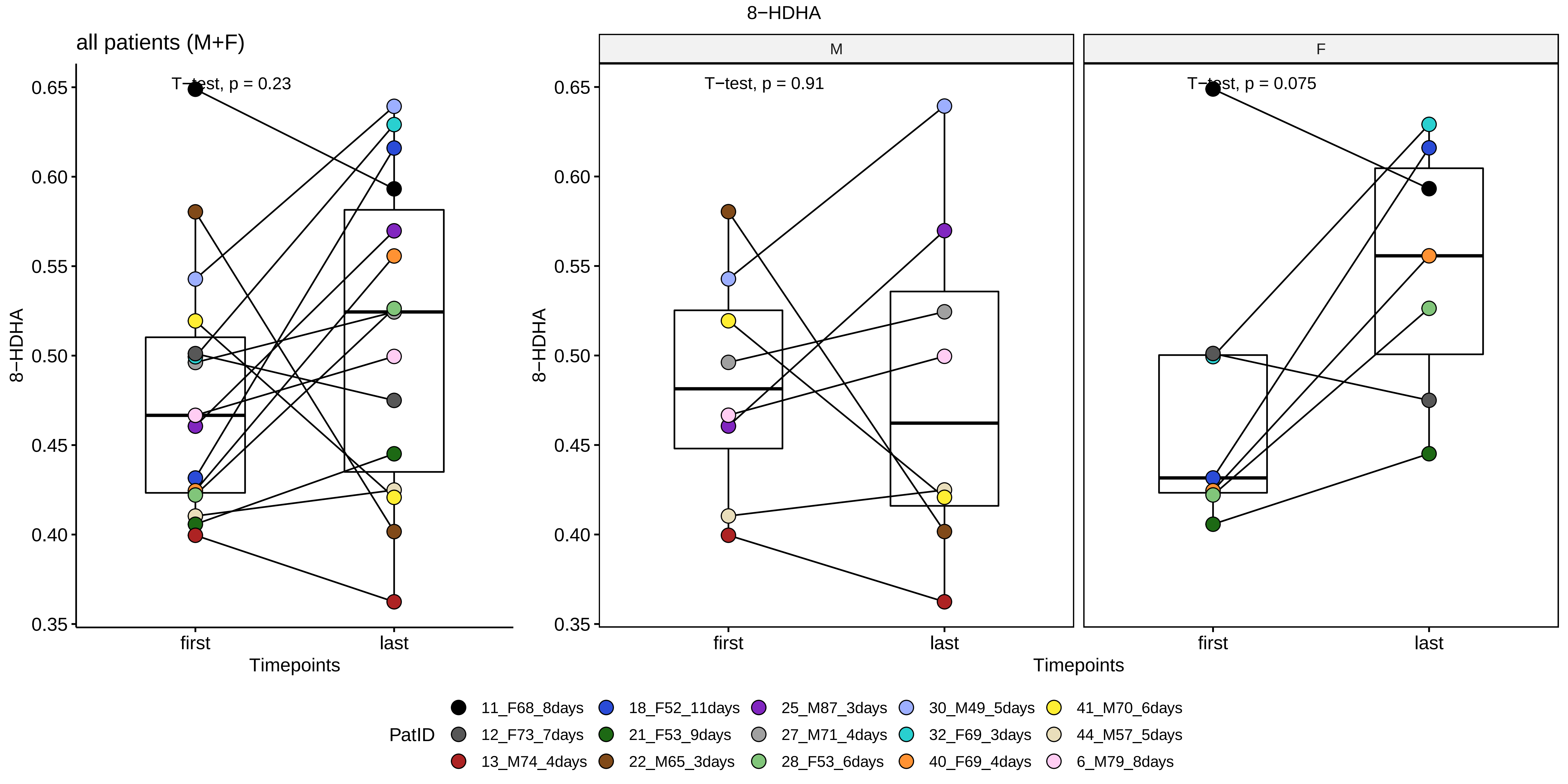

8−HDHA
all patients (M+F)
T−test, p = 0.23
M
T−test, p = 0.91
F
T−test, p = 0.075
8−HDHA
0.65
0.60
0.55
0.50
0.45
0.40
0.35
first
last
Timepoints
PatID
11_F68_8days
12_F73_7days
13_M74_4days
18_F52_11days
21_F53_9days
22_M65_3days
25_M87_3days
27_M71_4days
28_F53_6days
30_M49_5days
32_F69_3days
40_F69_4days
41_M70_6days
44_M57_5days
6_M79_8days

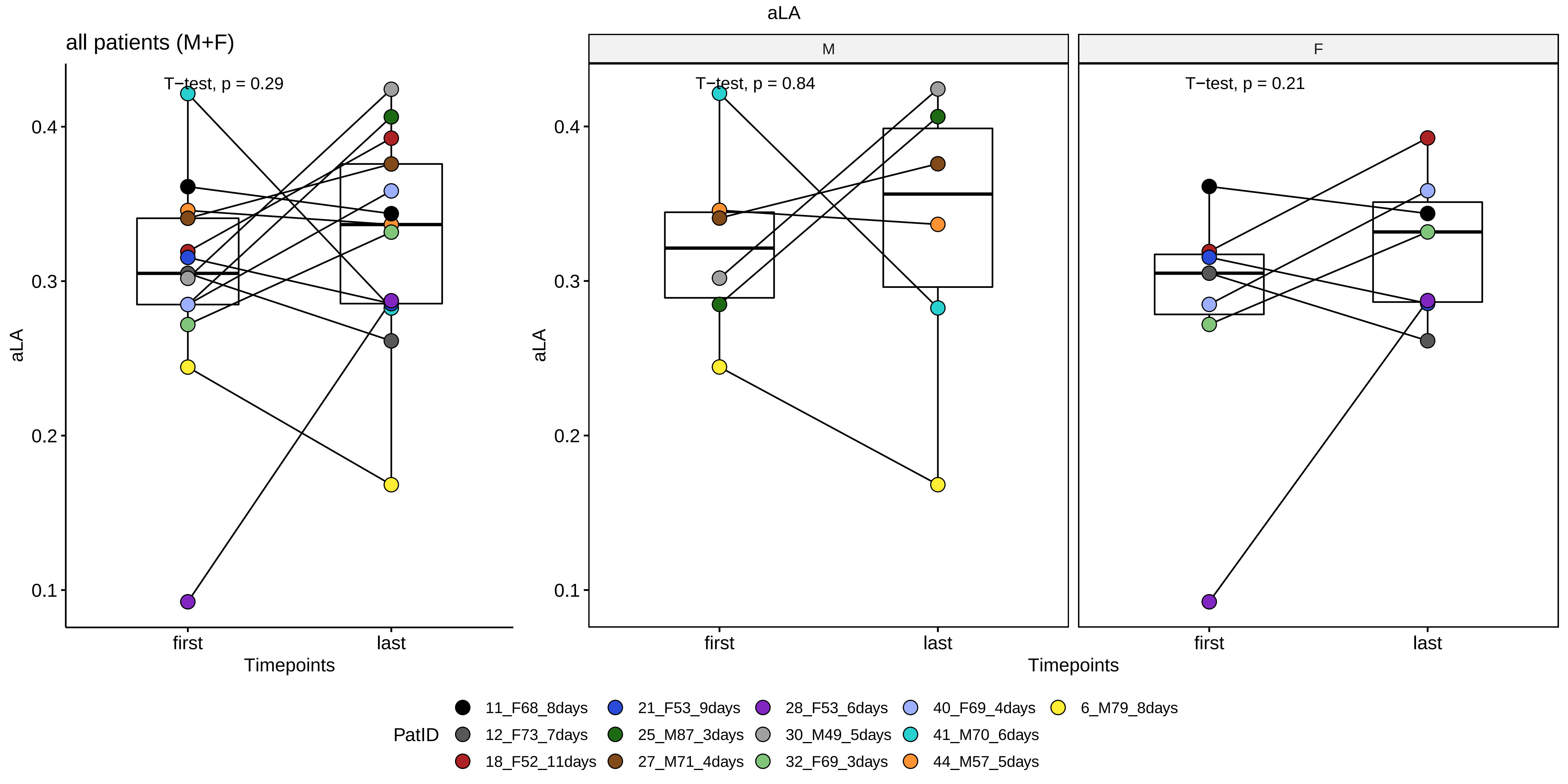
aLA
all patients (M+F)
T−test, p = 0.29
M
T−test, p = 0.84
F
T−test, p = 0.21
aLA
0.1
0.2
0.3
0.4
first
last
Timepoints
PatID
11_F68_8days
12_F73_7days
18_F52_11days
21_F53_9days
25_M87_3days
27_M71_4days
28_F53_6days
30_M49_5days
32_F69_3days
40_F69_4days
41_M70_6days
44_M57_5days
6_M79_8days

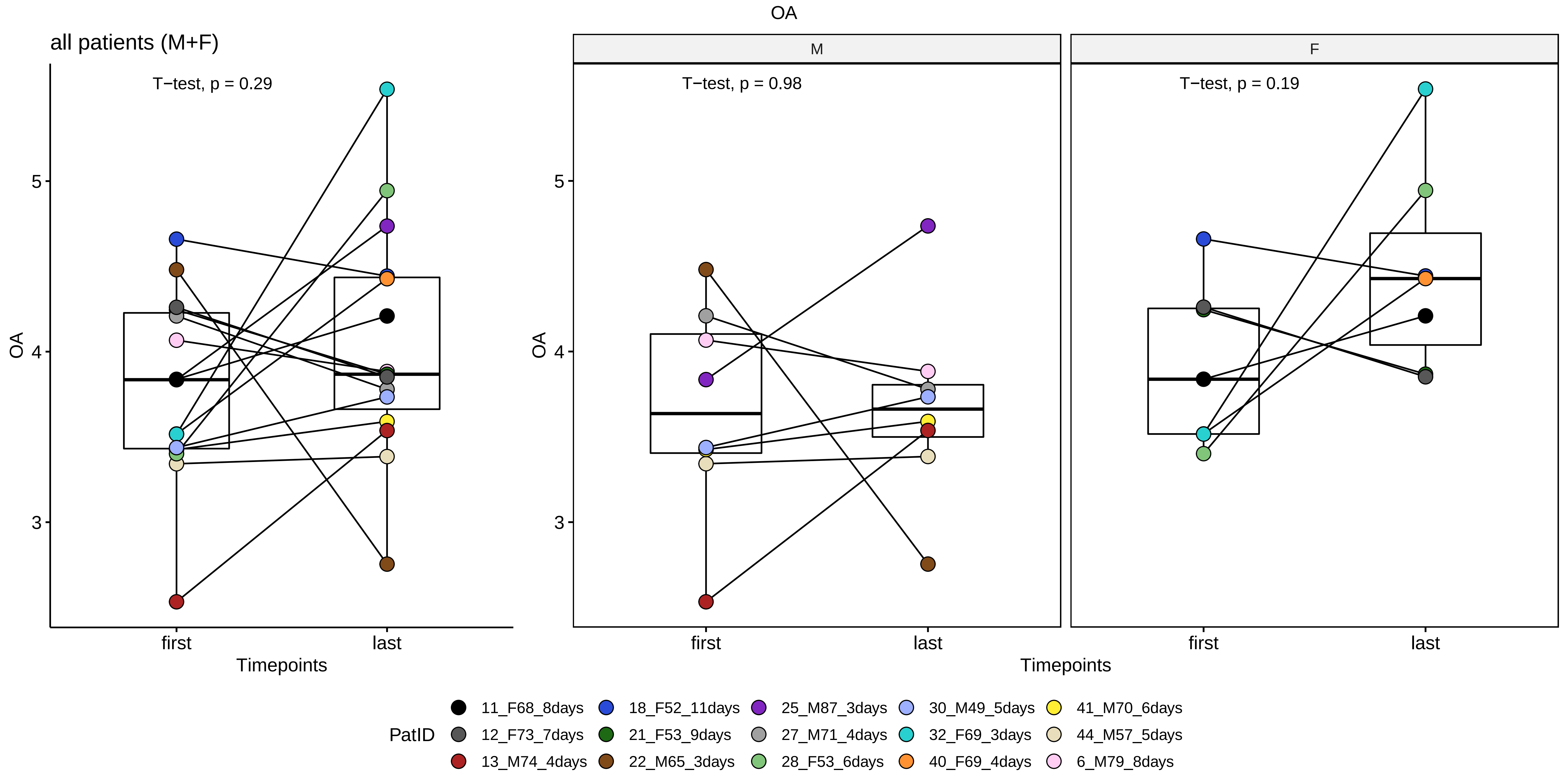

OA
all patients (M+F)
T−test, p = 0.29
M
T−test, p = 0.98
F
T−test, p = 0.19
5
4
3
OA
first
last
Timepoints
PatID
11_F68_8days
12_F73_7days
13_M74_4days
18_F52_11days
21_F53_9days
22_M65_3days
25_M87_3days
27_M71_4days
28_F53_6days
30_M49_5days
32_F69_3days
40_F69_4days
41_M70_6days
44_M57_5days
6_M79_8days

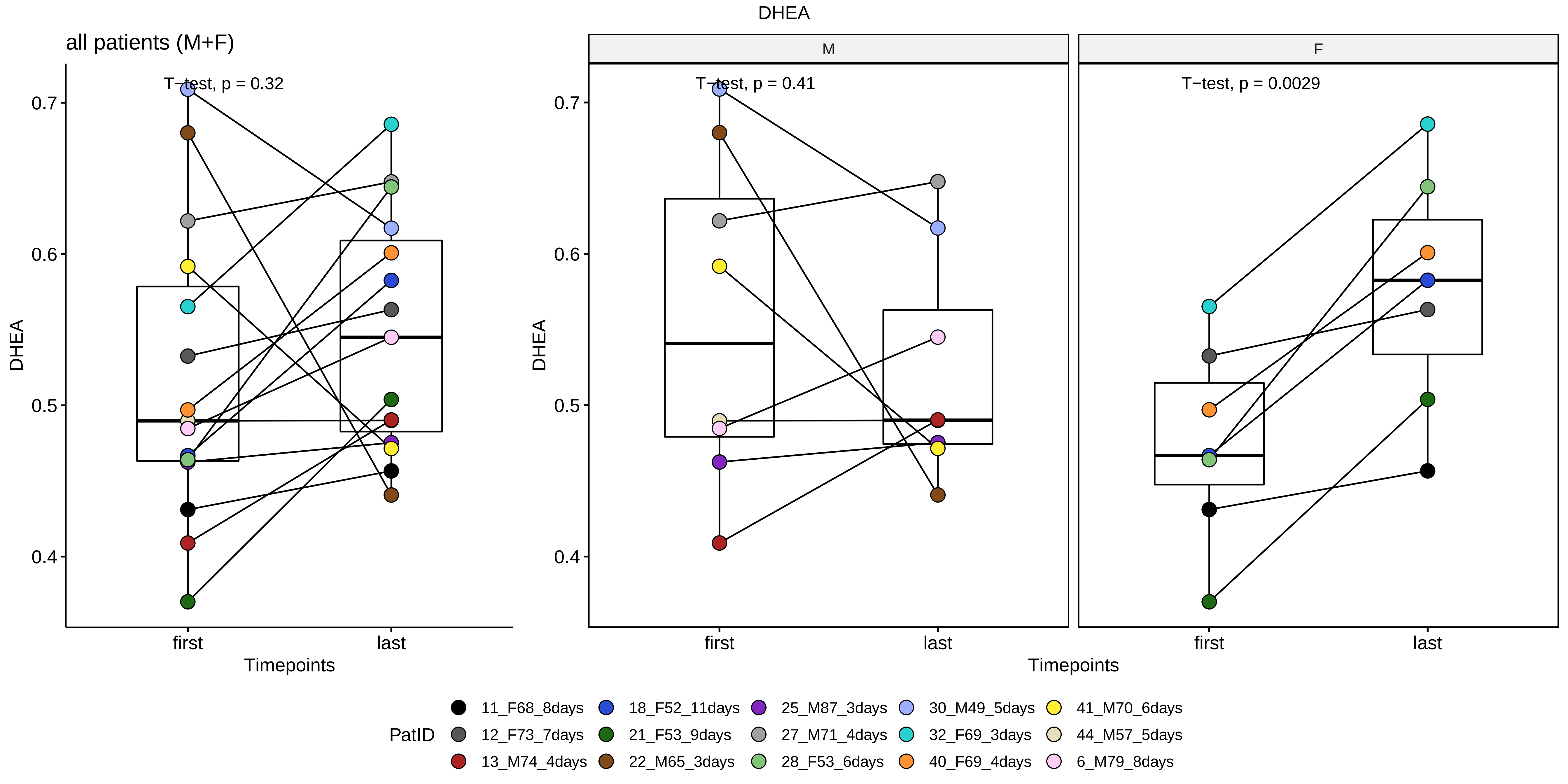

DHEA
all patients (M+F)
T−test, p = 0.32
M
T−test, p = 0.41
F
T−test, p = 0.0029
0.7
0.6
0.5
0.4
DHEA
first
last
Timepoints
PatID
11_F68_8days
12_F73_7days
13_M74_4days
18_F52_11days
21_F53_9days
22_M65_3days
25_M87_3days
27_M71_4days
28_F53_6days
30_M49_5days
32_F69_3days
40_F69_4days
41_M70_6days
44_M57_5days
6_M79_8days

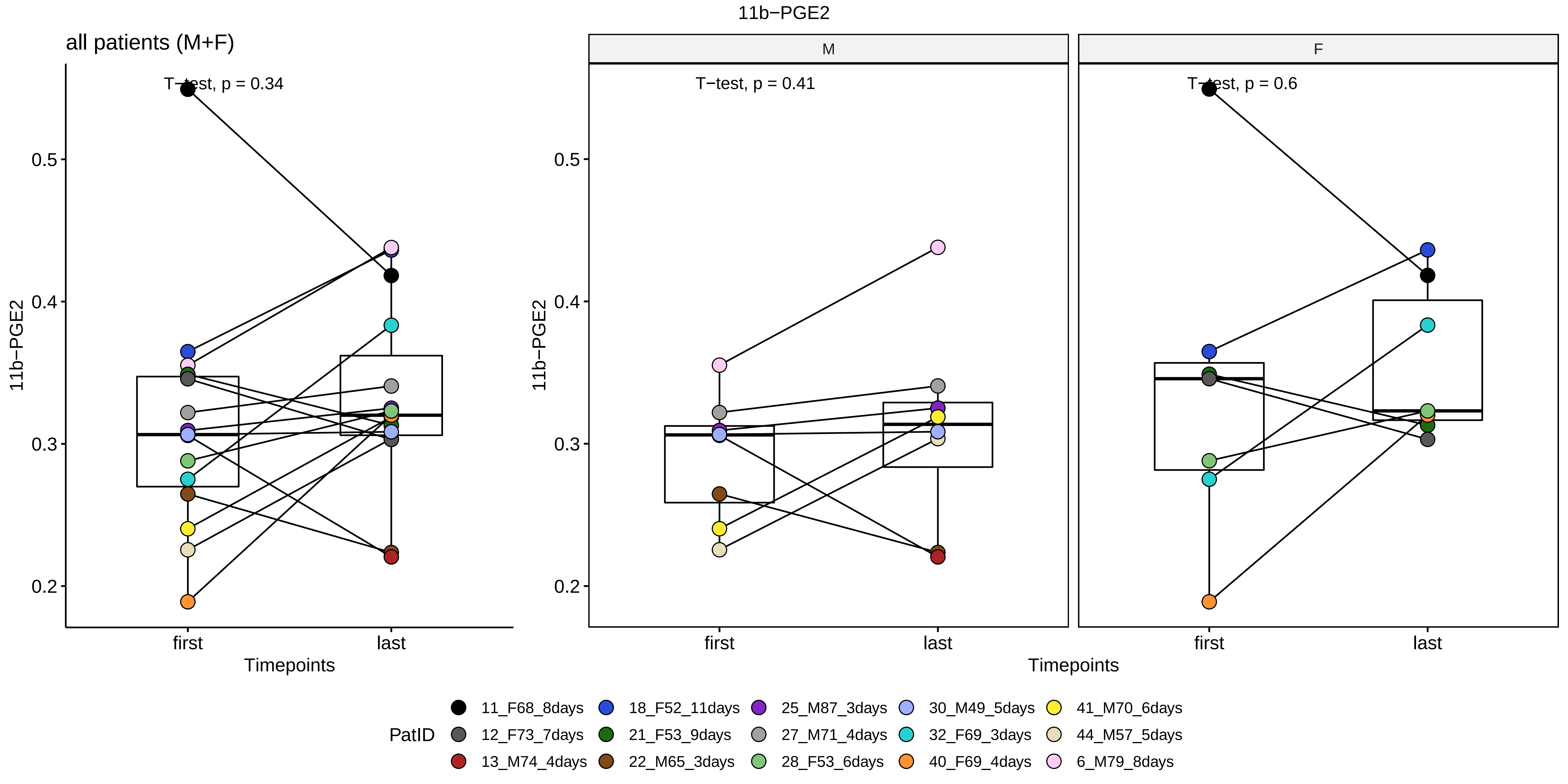

11b−PGE2
all patients (M+F)
T−test, p = 0.34
M
T−test, p = 0.41
F
T−test, p = 0.6
11b−PGE2
0.2
0.3
0.4
0.5
first
last
Timepoints
PatID
11_F68_8days
12_F73_7days
13_M74_4days
18_F52_11days
21_F53_9days
22_M65_3days
25_M87_3days
27_M71_4days
28_F53_6days
30_M49_5days
32_F69_3days
40_F69_4days
41_M70_6days
44_M57_5days
6_M79_8days

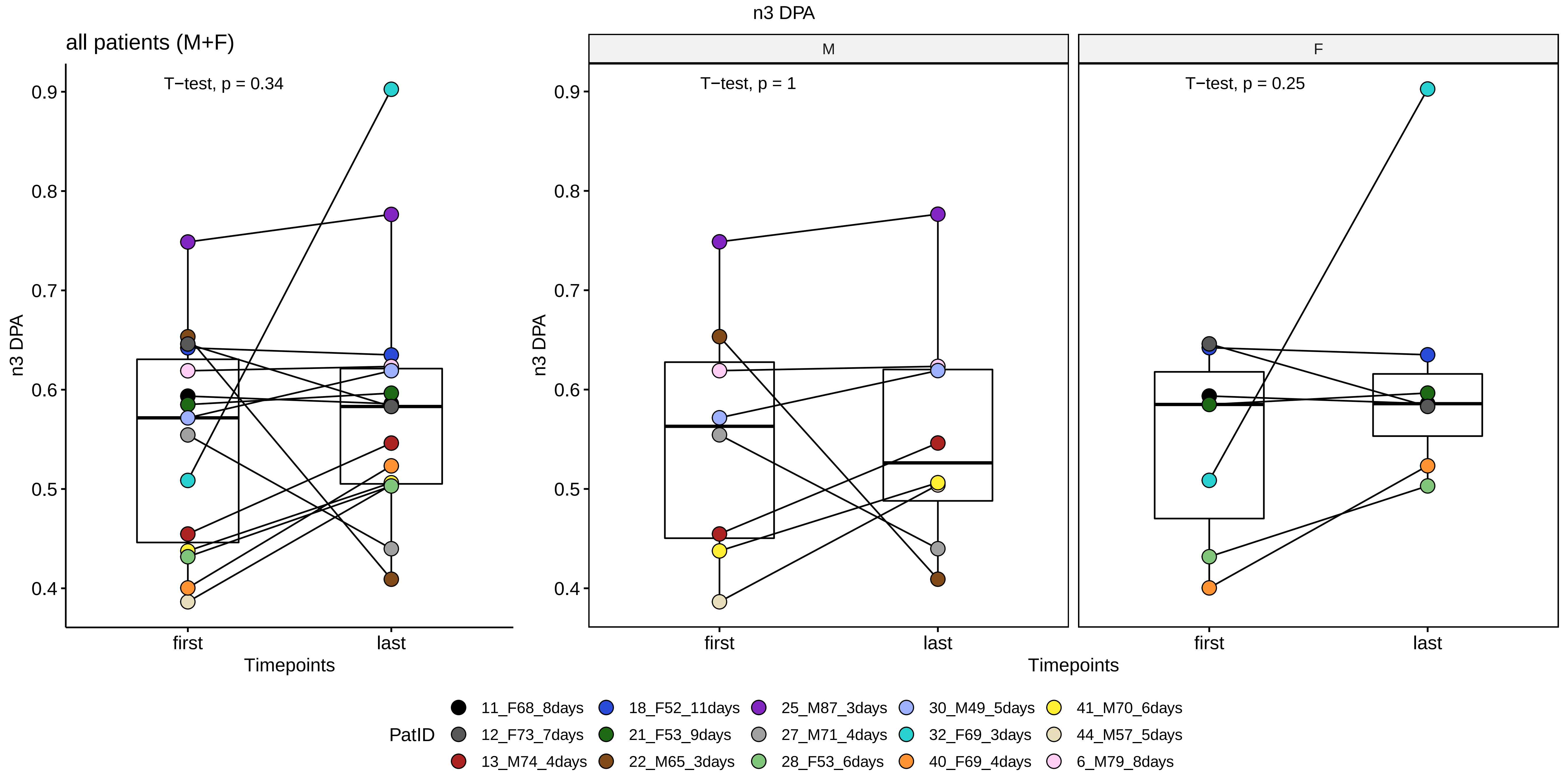

n3 DPA
all patients (M+F)
T−test, p = 0.34
M
T−test, p = 1
F
T−test, p = 0.25
n3 DPA
0.9
0.8
0.7
0.6
0.5
0.4
first
last
Timepoints
PatID
11_F68_8days
12_F73_7days
13_M74_4days
18_F52_11days
21_F53_9days
22_M65_3days
25_M87_3days
27_M71_4days
28_F53_6days
30_M49_5days
32_F69_3days
40_F69_4days
41_M70_6days
44_M57_5days
6_M79_8days

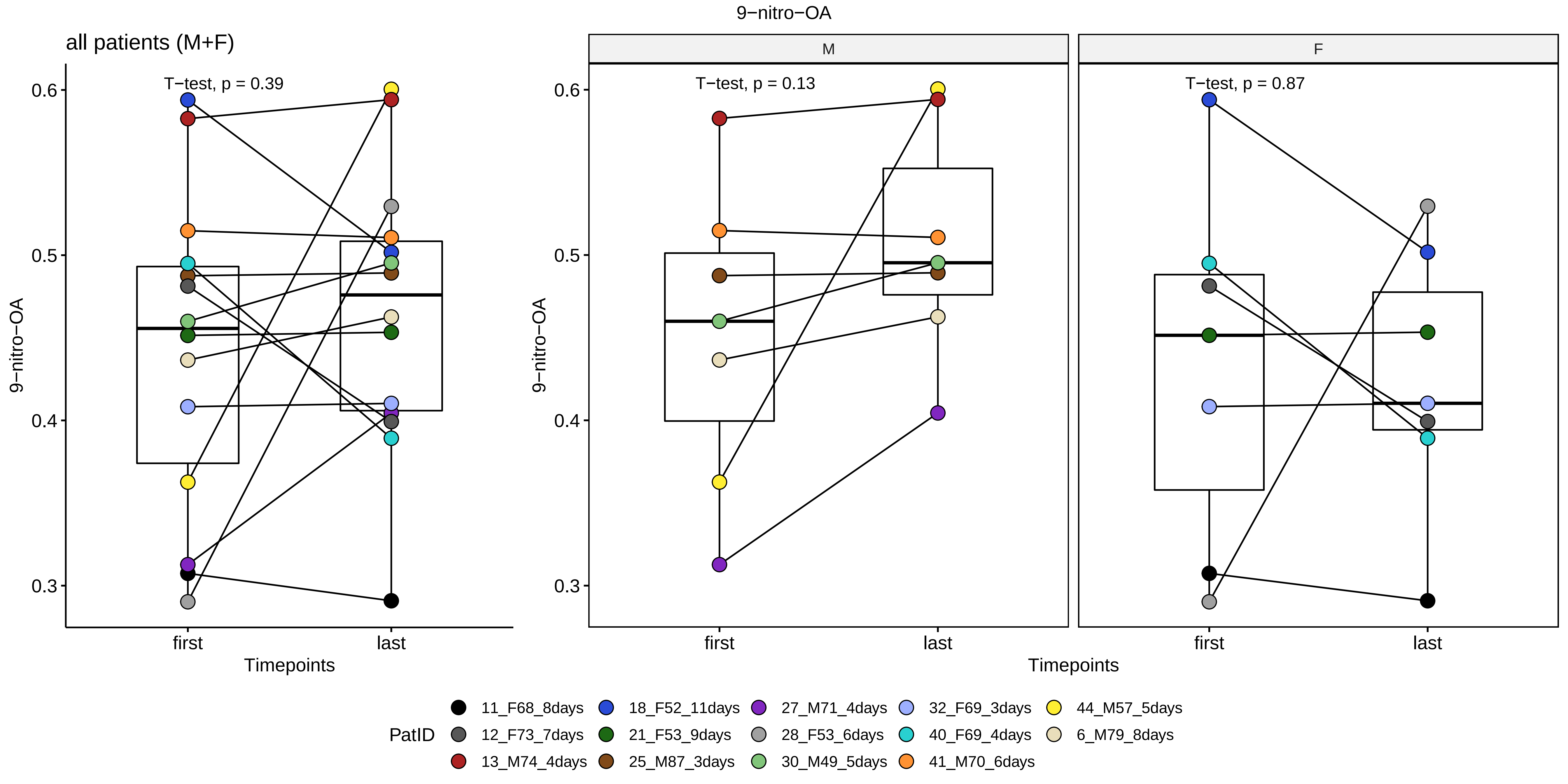

9−nitro−OA
all patients (M+F)
T−test, p = 0.39
M
T−test, p = 0.13
F
T−test, p = 0.87
9−nitro−OA
0.6
0.5
0.4
0.3
first
last
Timepoints
PatID
11_F68_8days
12_F73_7days
13_M74_4days
18_F52_11days
21_F53_9days
25_M87_3days
27_M71_4days
28_F53_6days
30_M49_5days
32_F69_3days
40_F69_4days
41_M70_6days
44_M57_5days
6_M79_8days

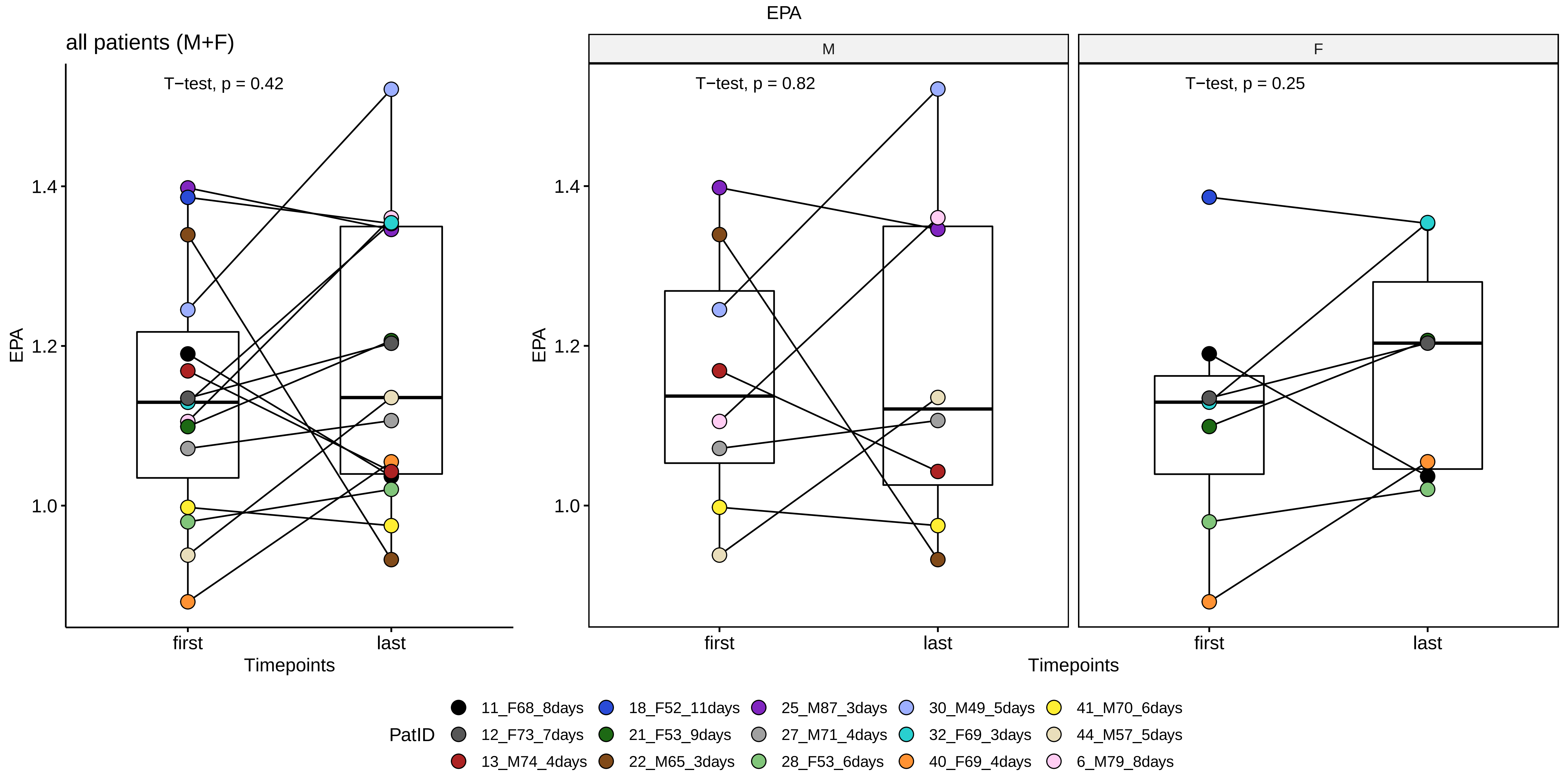

EPA
all patients (M+F)
T−test, p = 0.42
M
T−test, p = 0.82
F
T−test, p = 0.25
1.4
1.2
1.0
EPA
first
last
Timepoints
PatID
11_F68_8days
12_F73_7days
13_M74_4days
18_F52_11days
21_F53_9days
22_M65_3days
25_M87_3days
27_M71_4days
28_F53_6days
30_M49_5days
32_F69_3days
40_F69_4days
41_M70_6days
44_M57_5days
6_M79_8days

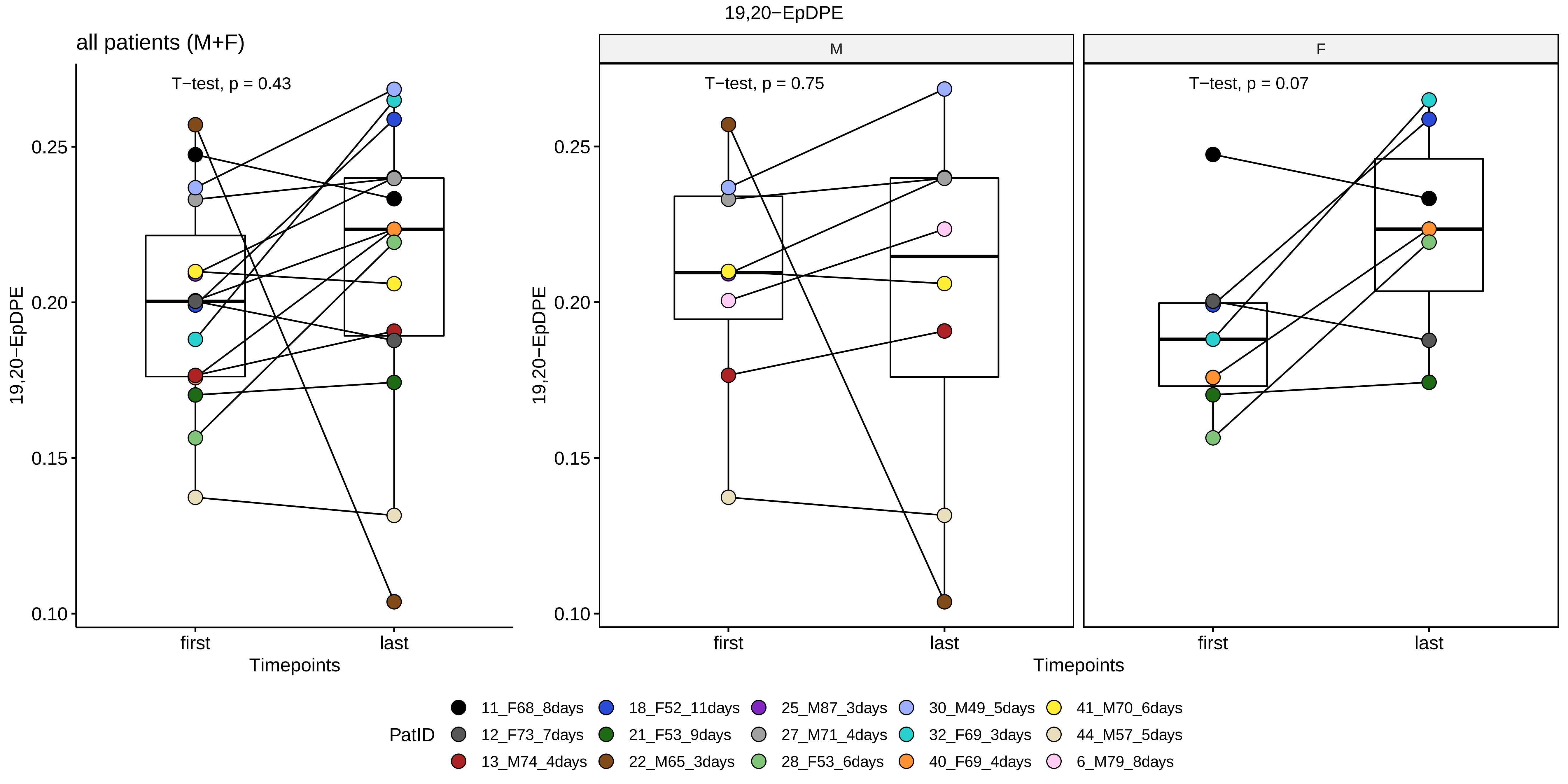

19,20−EpDPE
all patients (M+F)
T−test, p = 0.43
M
T−test, p = 0.75
F
T−test, p = 0.07
19,20−EpDPE
0.25
0.20
0.15
0.10
first
last
Timepoints
PatID
11_F68_8days
12_F73_7days
13_M74_4days
18_F52_11days
21_F53_9days
22_M65_3days
25_M87_3days
27_M71_4days
28_F53_6days
30_M49_5days
32_F69_3days
40_F69_4days
41_M70_6days
44_M57_5days
6_M79_8days

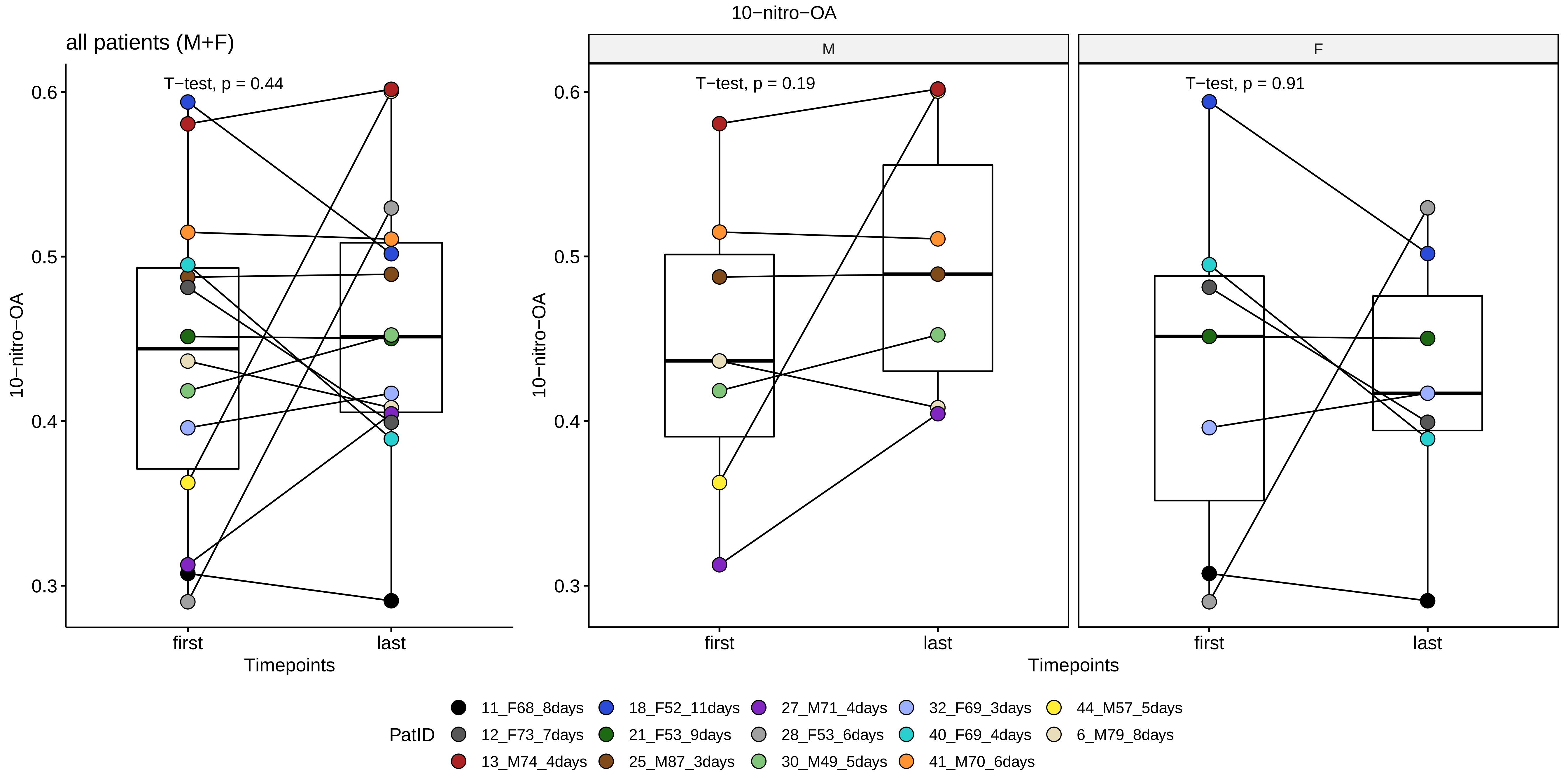

10−nitro−OA
all patients (M+F)
T−test, p = 0.44
M
T−test, p = 0.19
F
T−test, p = 0.91
10−nitro−OA
0.6
0.5
0.4
0.3
first
last
Timepoints
PatID
11_F68_8days
12_F73_7days
13_M74_4days
18_F52_11days
21_F53_9days
25_M87_3days
27_M71_4days
28_F53_6days
30_M49_5days
32_F69_3days
40_F69_4days
41_M70_6days
44_M57_5days
6_M79_8days

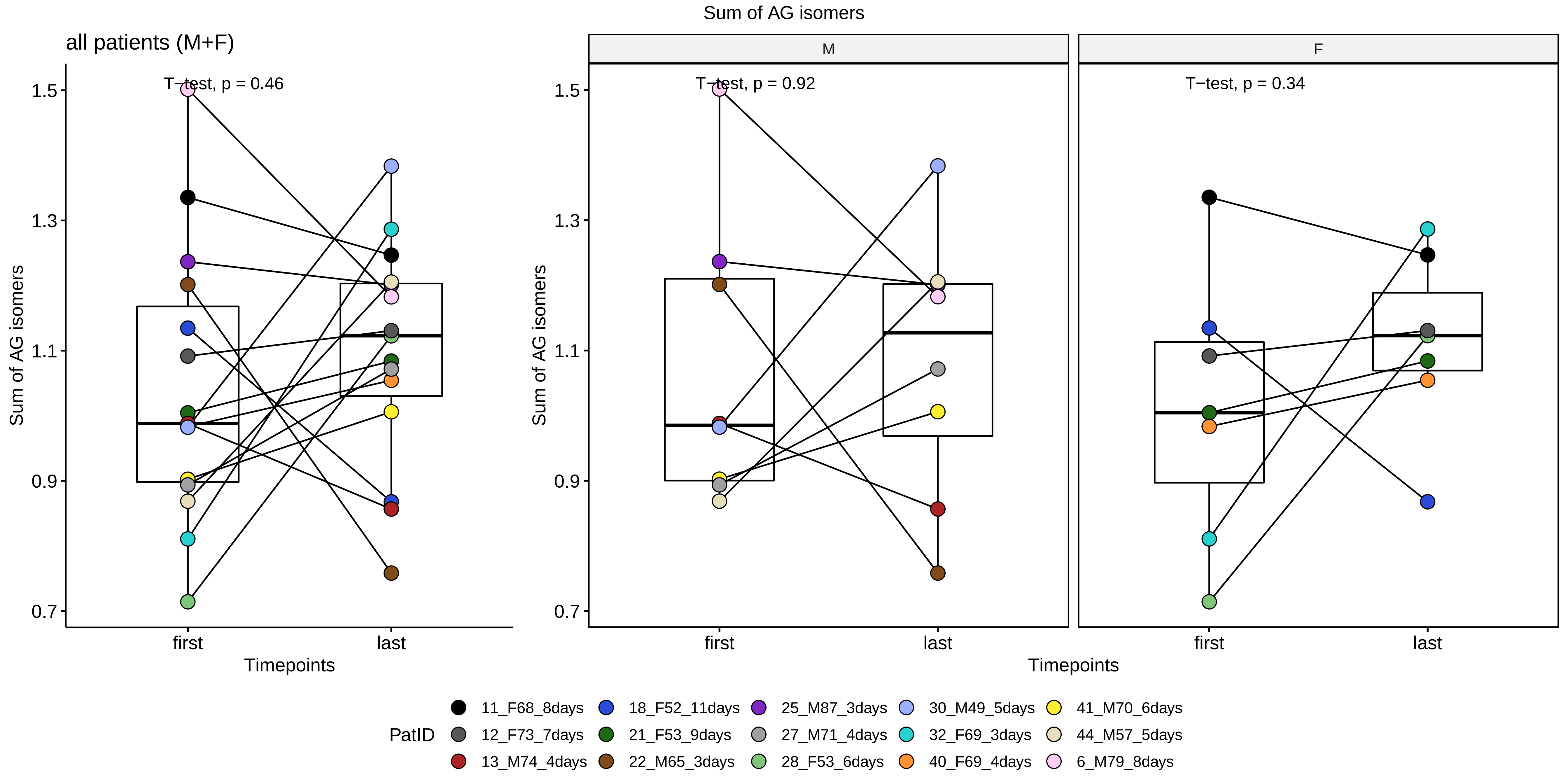

Sum of AG isomers
all patients (M+F)
T−test, p = 0.46
M
T−test, p = 0.92
F
T−test, p = 0.34
Sum of AG isomers
0.7
0.9
1.1
1.3
1.5
first
last
Timepoints
PatID
11_F68_8days
12_F73_7days
13_M74_4days
18_F52_11days
21_F53_9days
22_M65_3days
25_M87_3days
27_M71_4days
28_F53_6days
30_M49_5days
32_F69_3days
40_F69_4days
41_M70_6days
44_M57_5days
6_M79_8days

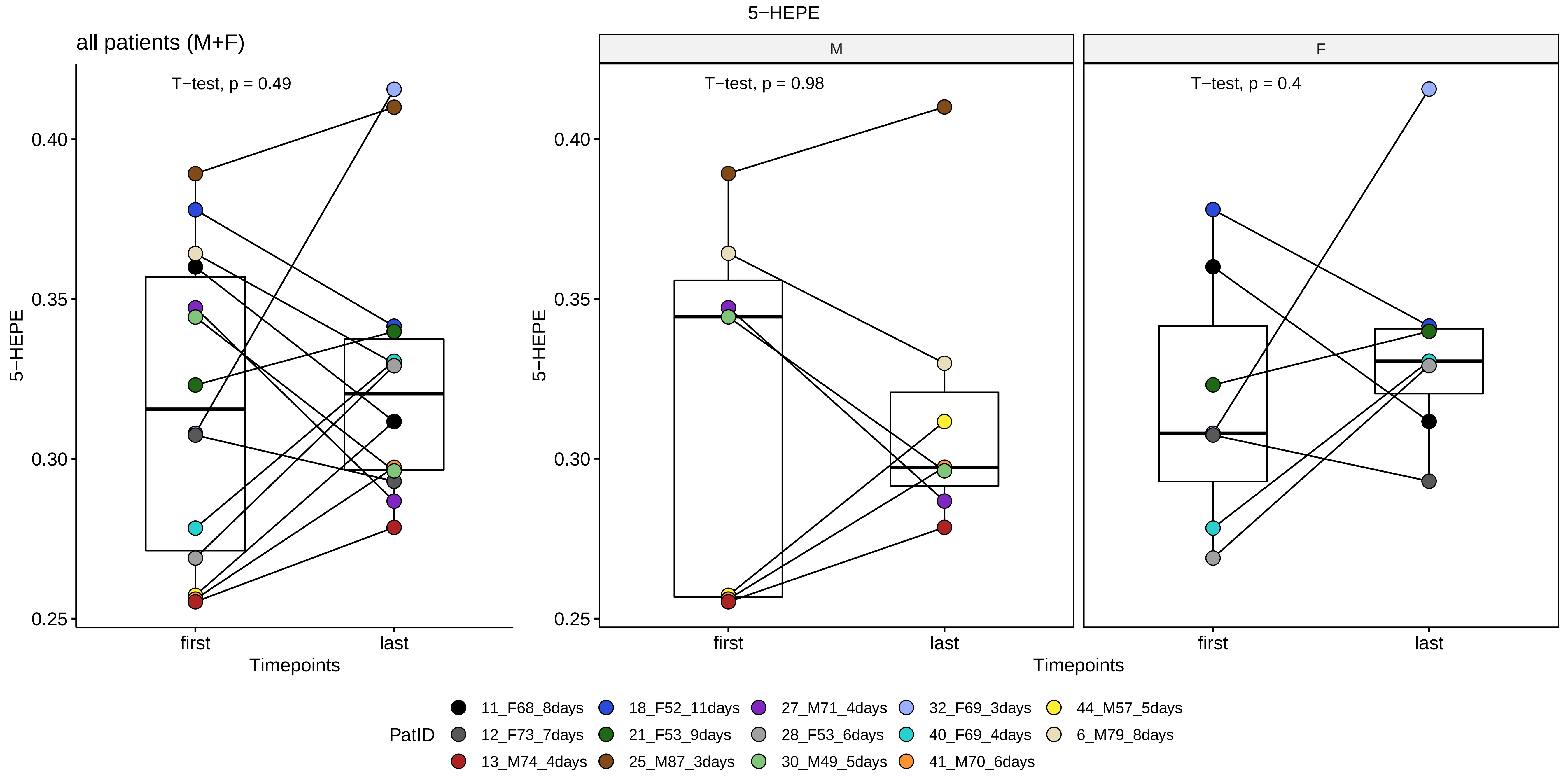
5−HEPE
all patients (M+F)
T−test, p = 0.49
M
T−test, p = 0.98
F
T−test, p = 0.4
5−HEPE
0.25
0.30
0.35
0.40
first
last
Timepoints
PatID
11_F68_8days
12_F73_7days
13_M74_4days
18_F52_11days
21_F53_9days
25_M87_3days
27_M71_4days
28_F53_6days
30_M49_5days
32_F69_3days
40_F69_4days
41_M70_6days
44_M57_5days
6_M79_8days

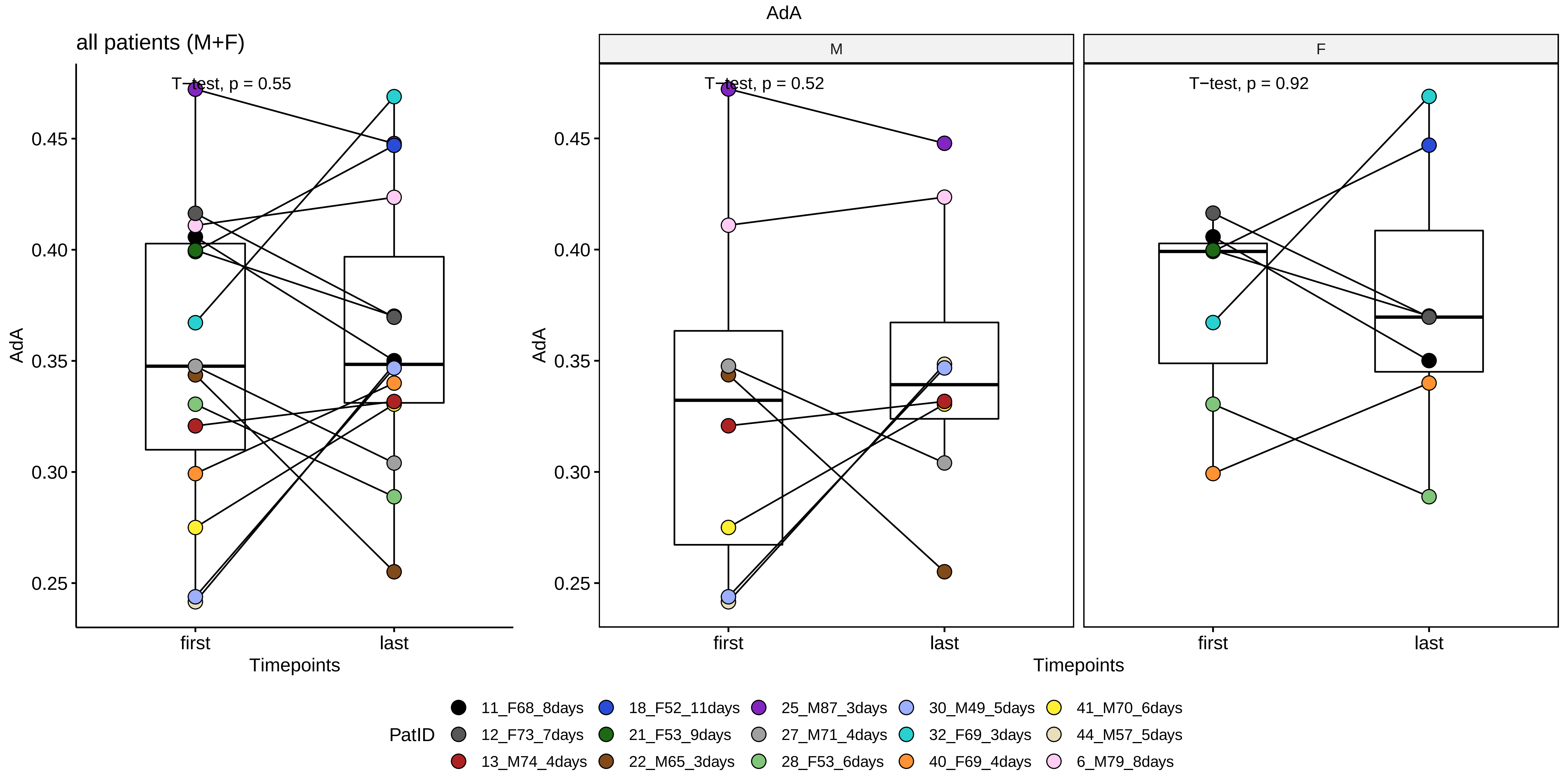
AdA
all patients (M+F)
T−test, p = 0.55
M
T−test, p = 0.52
F
T−test, p = 0.92
AdA
0.45
0.40
0.35
0.30
0.25
first
last
Timepoints
PatID
11_F68_8days
12_F73_7days
13_M74_4days
18_F52_11days
21_F53_9days
22_M65_3days
25_M87_3days
27_M71_4days
28_F53_6days
30_M49_5days
32_F69_3days
40_F69_4days
41_M70_6days
44_M57_5days
6_M79_8days

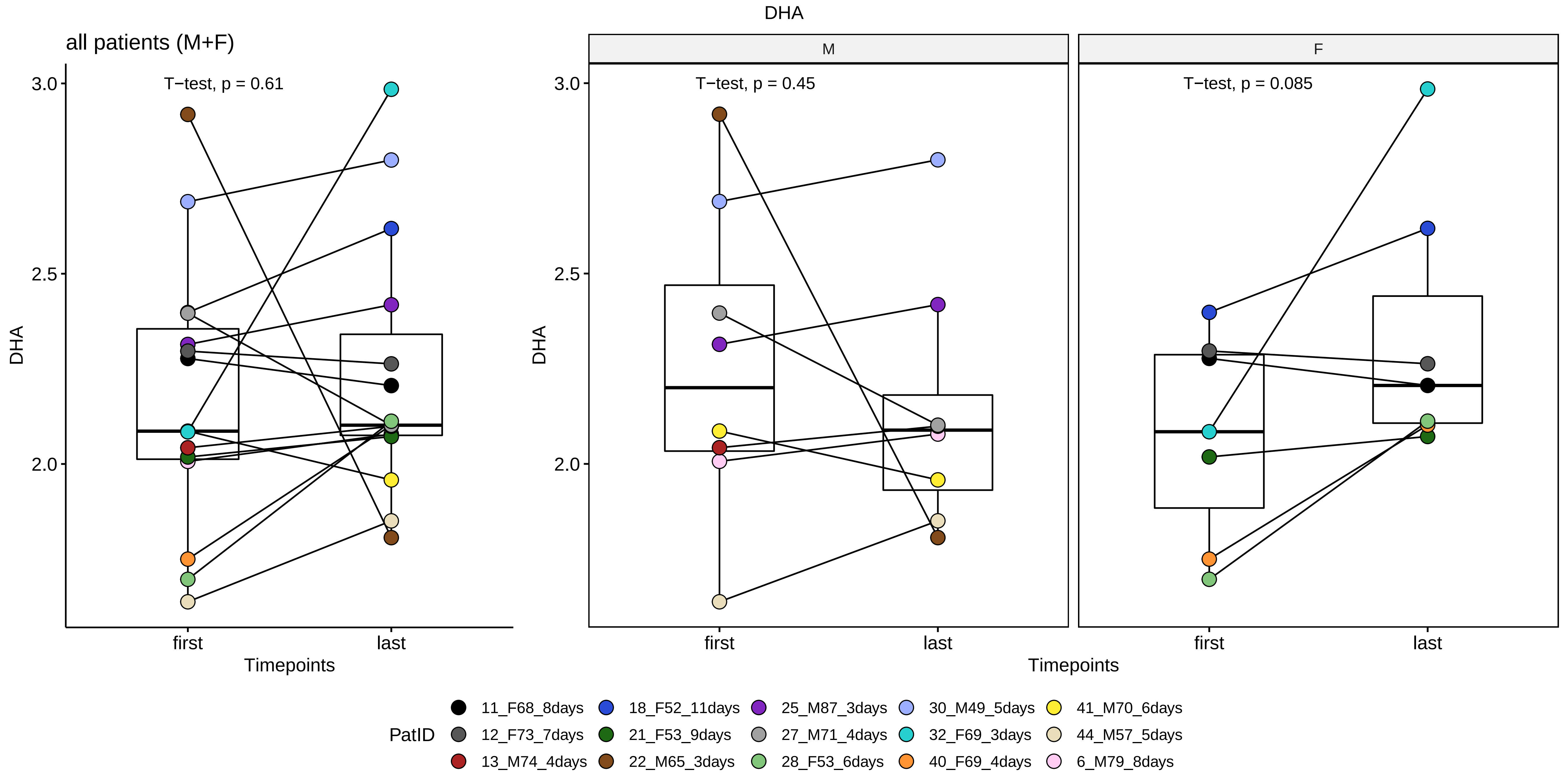
DHA
all patients (M+F)
T−test, p = 0.61
M
T−test, p = 0.45
F
T−test, p = 0.085
3.0
2.5
2.0
DHA
first
last
Timepoints
PatID
11_F68_8days
12_F73_7days
13_M74_4days
18_F52_11days
21_F53_9days
22_M65_3days
25_M87_3days
27_M71_4days
28_F53_6days
30_M49_5days
32_F69_3days
40_F69_4days
41_M70_6days
44_M57_5days
6_M79_8days

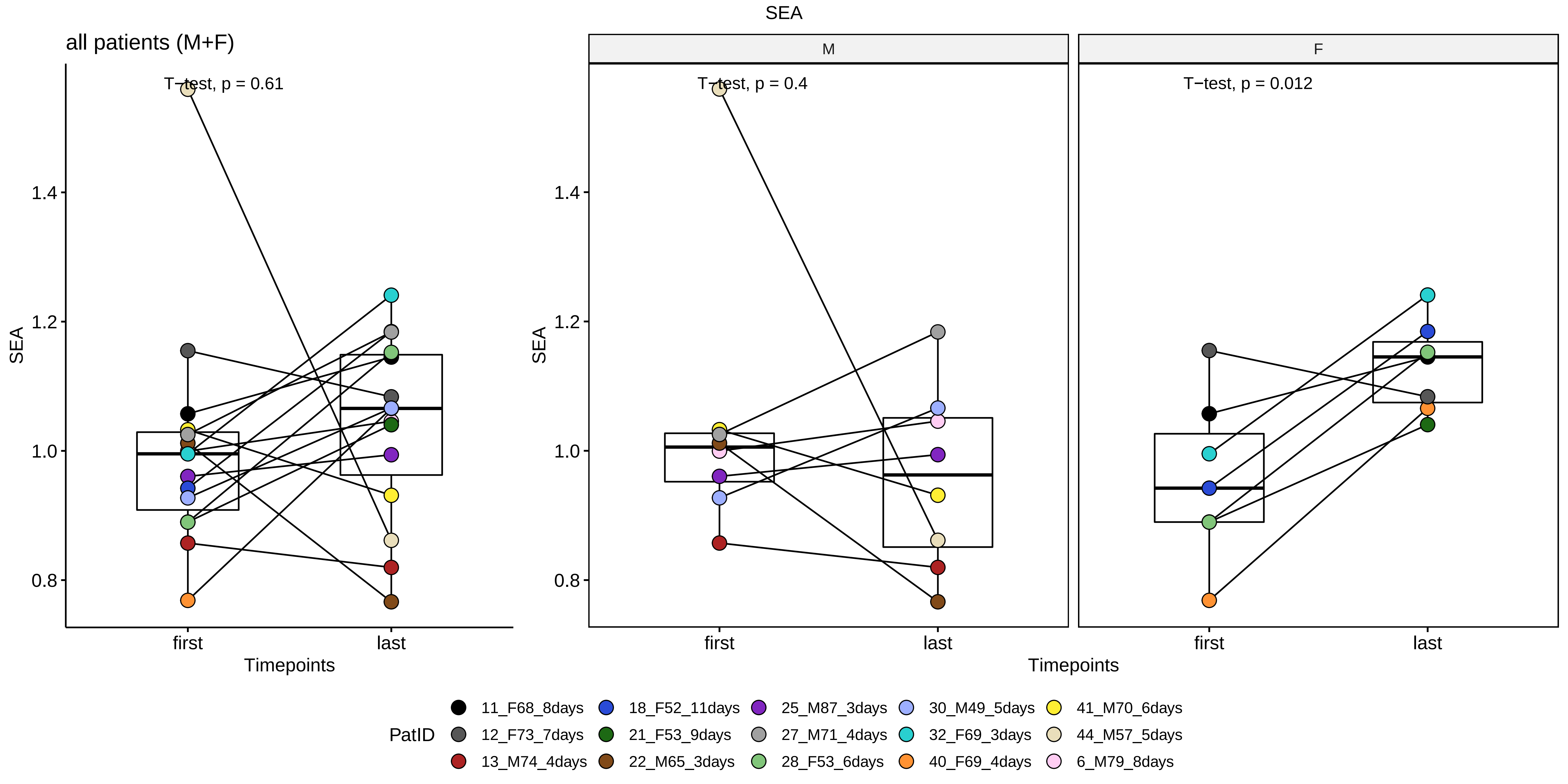
SEA
all patients (M+F)
T−test, p = 0.61
M
T−test, p = 0.4
F
T−test, p = 0.012
SEA
1.4
1.2
1.0
0.8
first
last
Timepoints
PatID
11_F68_8days
12_F73_7days
13_M74_4days
18_F52_11days
21_F53_9days
22_M65_3days
25_M87_3days
27_M71_4days
28_F53_6days
30_M49_5days
32_F69_3days
40_F69_4days
41_M70_6days
44_M57_5days
6_M79_8days

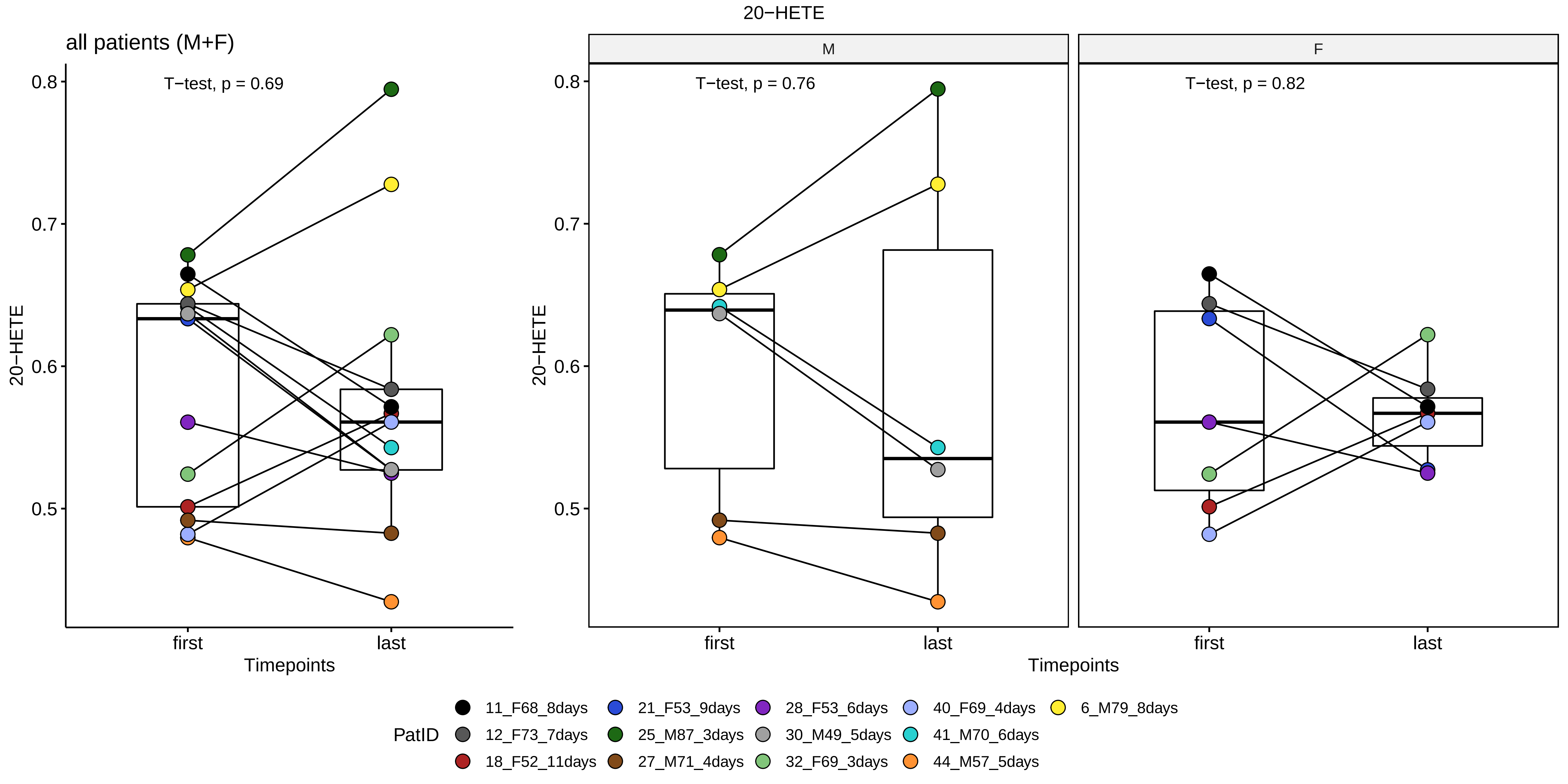

20−HETE
all patients (M+F)
T−test, p = 0.69
M
T−test, p = 0.76
F
T−test, p = 0.82
0.8
0.7
0.6
0.5
20−HETE
first
last
Timepoints
PatID
11_F68_8days
12_F73_7days
18_F52_11days
21_F53_9days
25_M87_3days
27_M71_4days
28_F53_6days
30_M49_5days
32_F69_3days
40_F69_4days
41_M70_6days
44_M57_5days
6_M79_8days

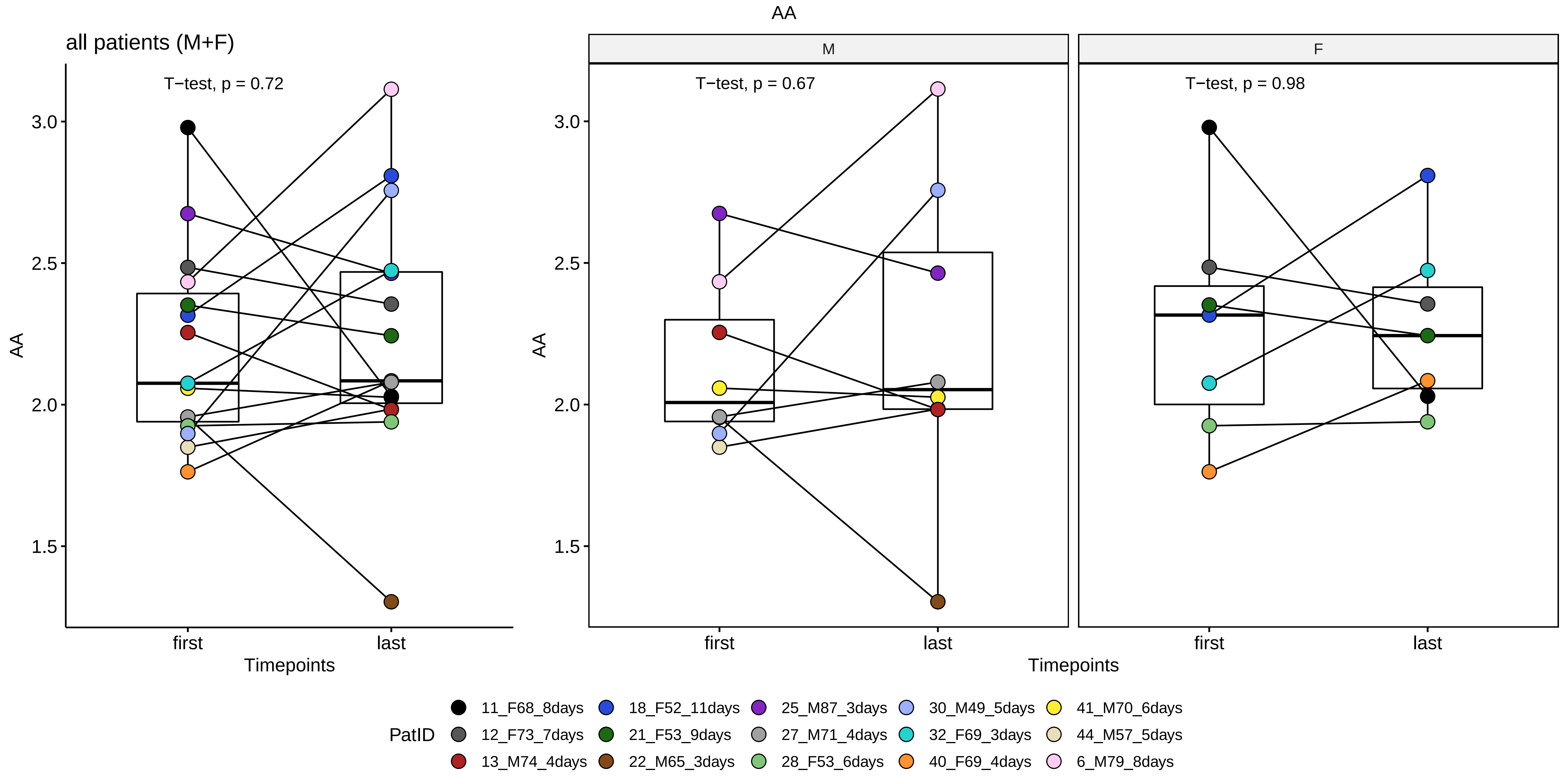
AA
all patients (M+F)
T−test, p = 0.72
M
T−test, p = 0.67
F
T−test, p = 0.98
AA
3.0
2.5
2.0
1.5
first
last
Timepoints
PatID
11_F68_8days
12_F73_7days
13_M74_4days
18_F52_11days
21_F53_9days
22_M65_3days
25_M87_3days
27_M71_4days
28_F53_6days
30_M49_5days
32_F69_3days
40_F69_4days
41_M70_6days
44_M57_5days
6_M79_8days

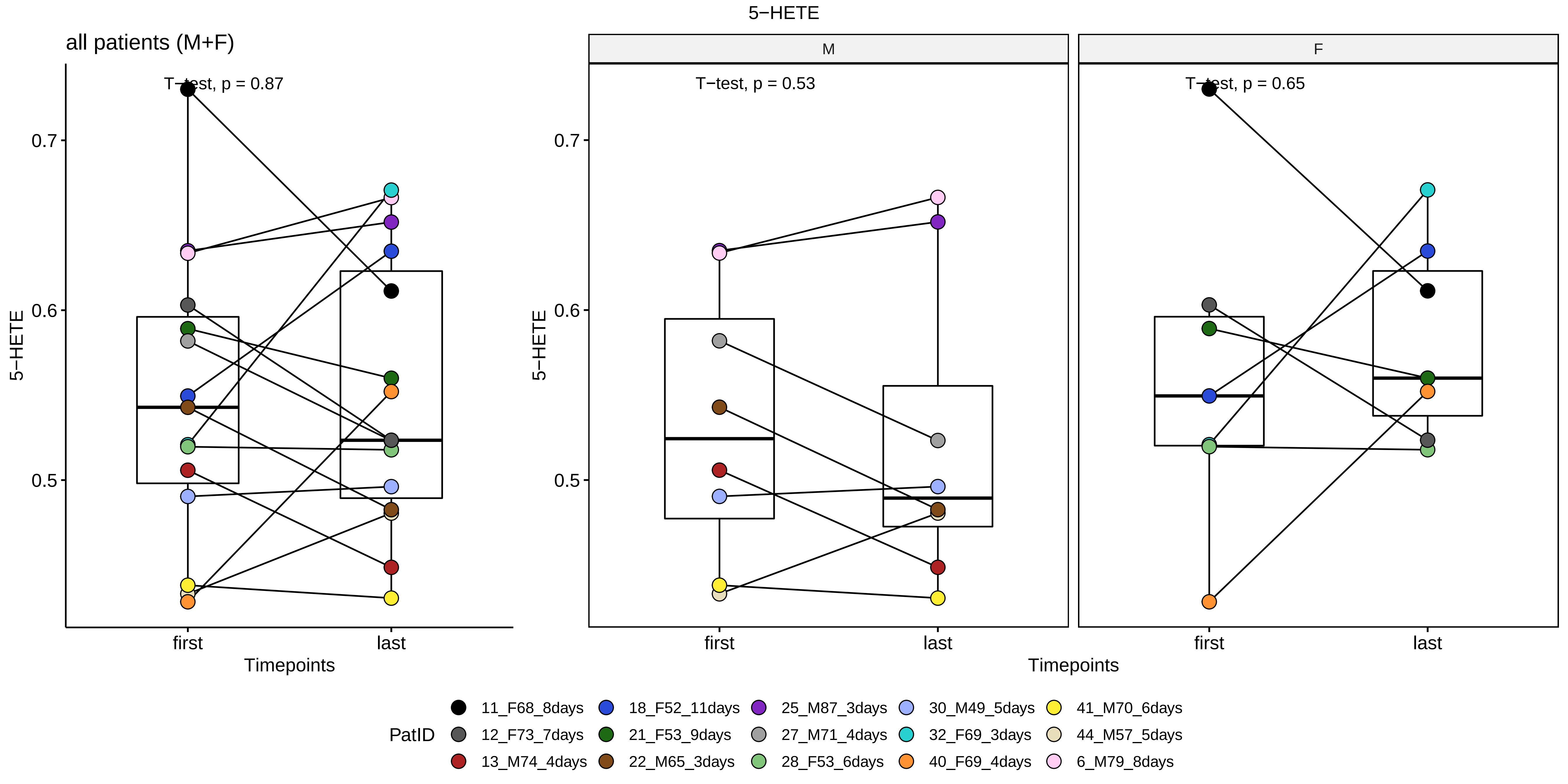

5−HETE
all patients (M+F)
T−test, p = 0.87
M
T−test, p = 0.53
F
T−test, p = 0.65
0.7
0.6
0.5
5−HETE
first
last
Timepoints
PatID
11_F68_8days
12_F73_7days
13_M74_4days
18_F52_11days
21_F53_9days
22_M65_3days
25_M87_3days
27_M71_4days
28_F53_6days
30_M49_5days
32_F69_3days
40_F69_4days
41_M70_6days
44_M57_5days
6_M79_8days

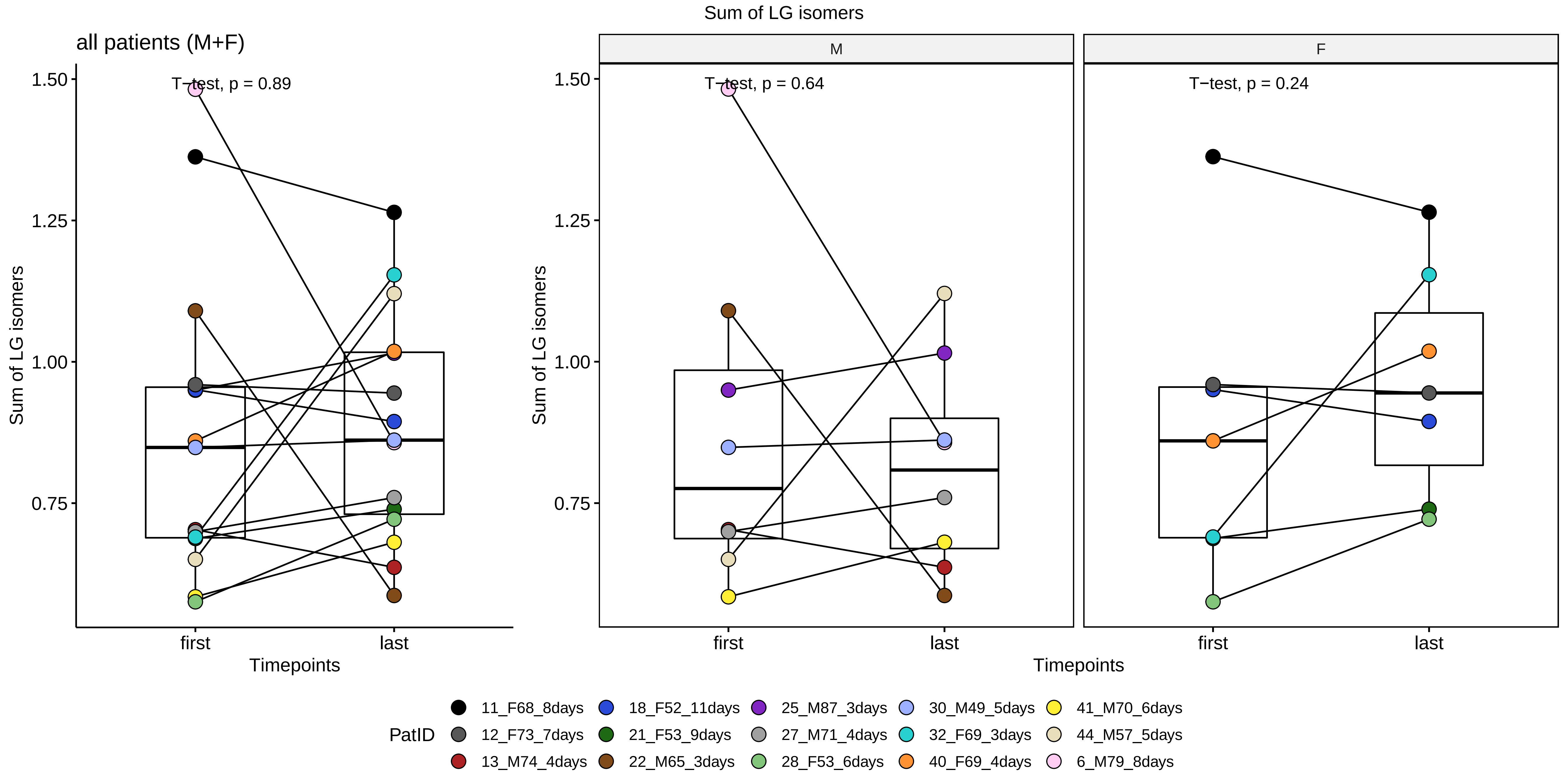

Sum of LG isomers
all patients (M+F)
T−test, p = 0.89
1.50
1.25
1.00
0.75
Sum of LG isomers
first
last
Timepoints
M
F
T−test, p = 0.64
T−test, p = 0.24
PatID
11_F68_8days
12_F73_7days
13_M74_4days
18_F52_11days
21_F53_9days
22_M65_3days
25_M87_3days
27_M71_4days
28_F53_6days
30_M49_5days
32_F69_3days
40_F69_4days
41_M70_6days
44_M57_5days
6_M79_8days

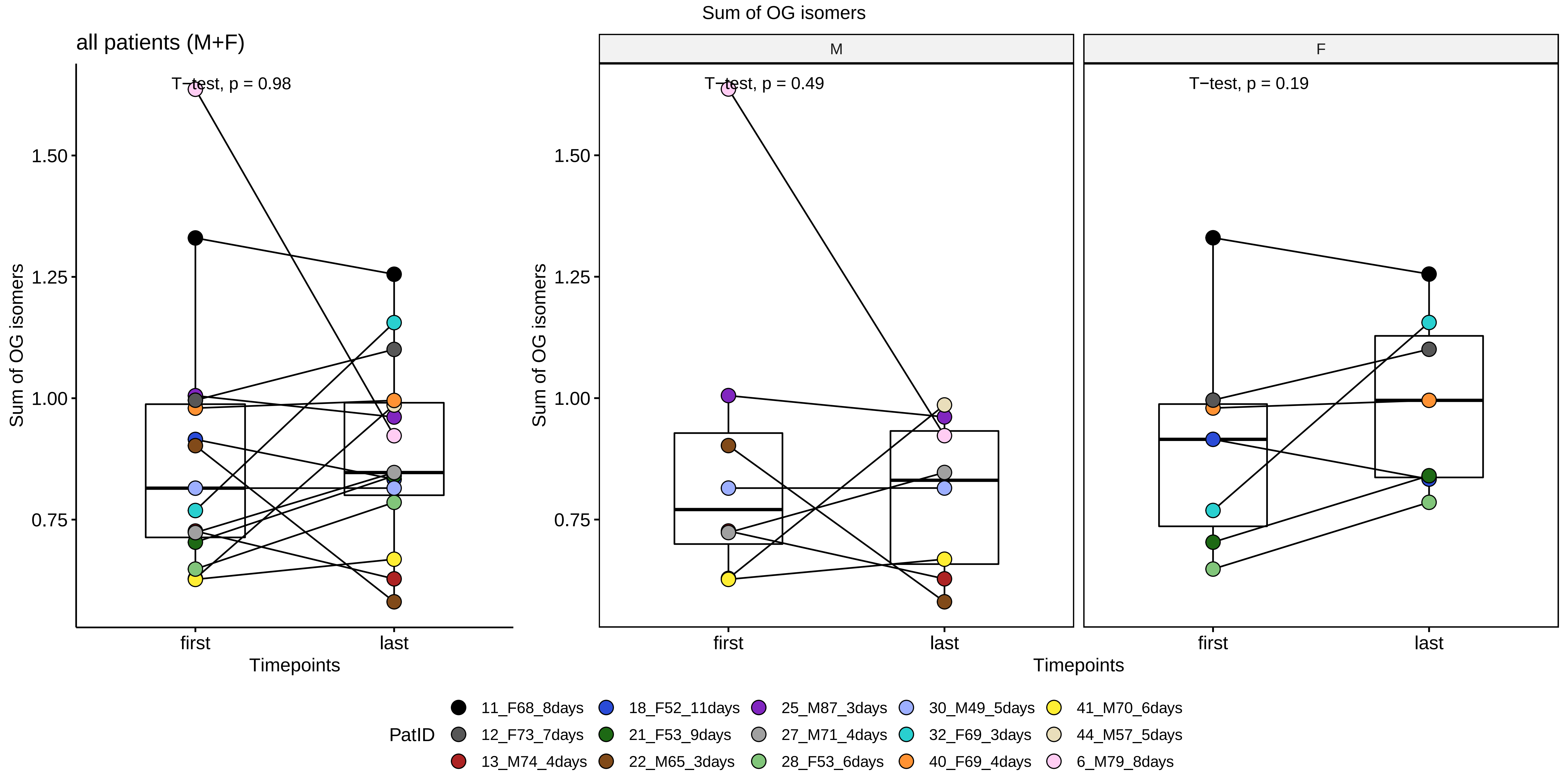

Sum of OG isomers
all patients (M+F)
M
F
T−test, p = 0.98
T−test, p = 0.49
T−test, p = 0.19
Sum of OG isomers
1.50
1.25
1.00
0.75
first
last
Timepoints
PatID
11_F68_8days
12_F73_7days
13_M74_4days
18_F52_11days
21_F53_9days
22_M65_3days
25_M87_3days
27_M71_4days
28_F53_6days
30_M49_5days
32_F69_3days
40_F69_4days
41_M70_6days
44_M57_5days
6_M79_8days
