# Supplementary material for: Plasma Oxylipins and Their Precursors Are Strongly Associated with COVID-19 Severity and with Immune Response Markers
Source: Metabolites. 2022 Jul 4;12(7):619. doi: 10.3390/metabo12070619 (PMC9319897; doi:10.3390/metabo12070619)
Supplement: Supplementary file 1 [file metabolites-12-00619-s001.zip › Supplementary figures.pdf]

Plasma oxylipins and their precursors are strongly associated with COVID-19 severity and with immune response markers

Naama Karu [1,*], Alida Kindt [1], Lieke Lamont [1], Adriaan J. van Gammeren [2], Anton A.M. Ermens [2], Amy C. Harms [1], Lutzen Portengen [3], Roel C.H. Vermeulen [3], Willem A. Dik [4], Anton W. Langerak [4], Vincent H.J. van der Velden [4] and Thomas Hankemeier [1,*]

[1] Metabolomics and Analytics Centre, Leiden Academic Centre for Drug Research, Leiden University, 2333 CC Leiden, the Netherlands
[2] Department of Clinical Chemistry and Hematology, Amphia Hospital, 4818 CK Breda, The Netherlands
[3] Department of Population Health Sciences, Institute for Risk Assessment Sciences, University Utrecht, 3584 CK Utrecht, the Netherlands
[4] Laboratory Medical Immunology, Department of Immunology, Erasmus MC University Medical Center Rotterdam, 3015 GD Rotterdam, the Netherlands

Supplementary figures

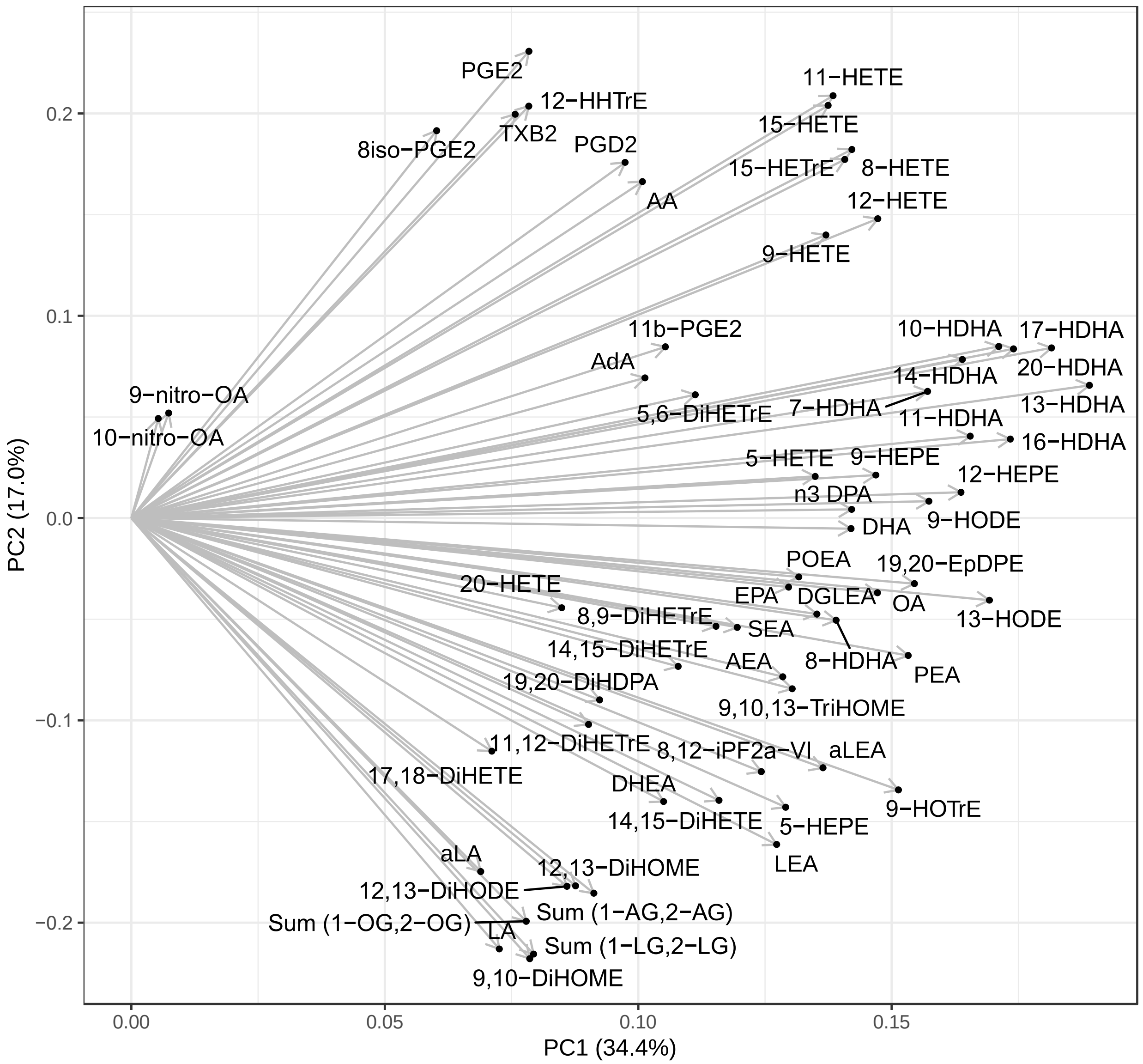

Figure S1. PCA loadings of all study samples, based on all metabolites, as described in Figure 1.

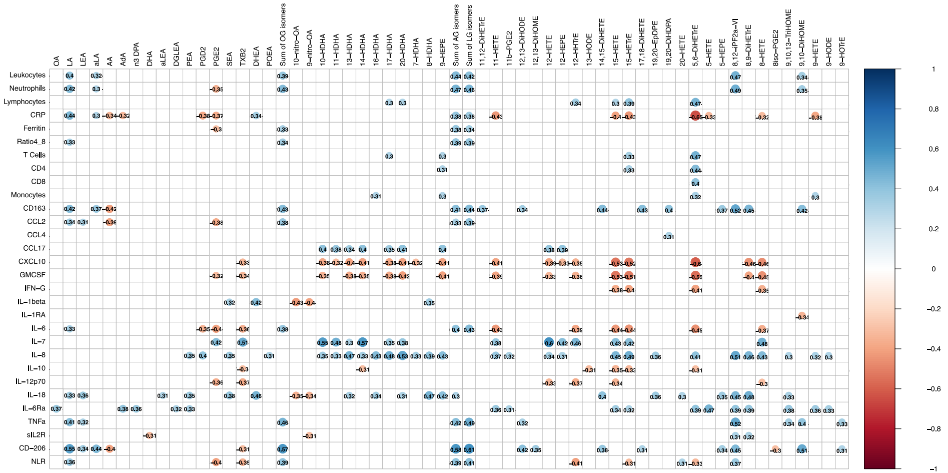

Figure S2. Heatmap of Pearson correlation results between selected metabolites and immune response markers (all cuberoot-transformed). Complete correlation matrices (with R, P, Q values) are provided in Tables S10-12.

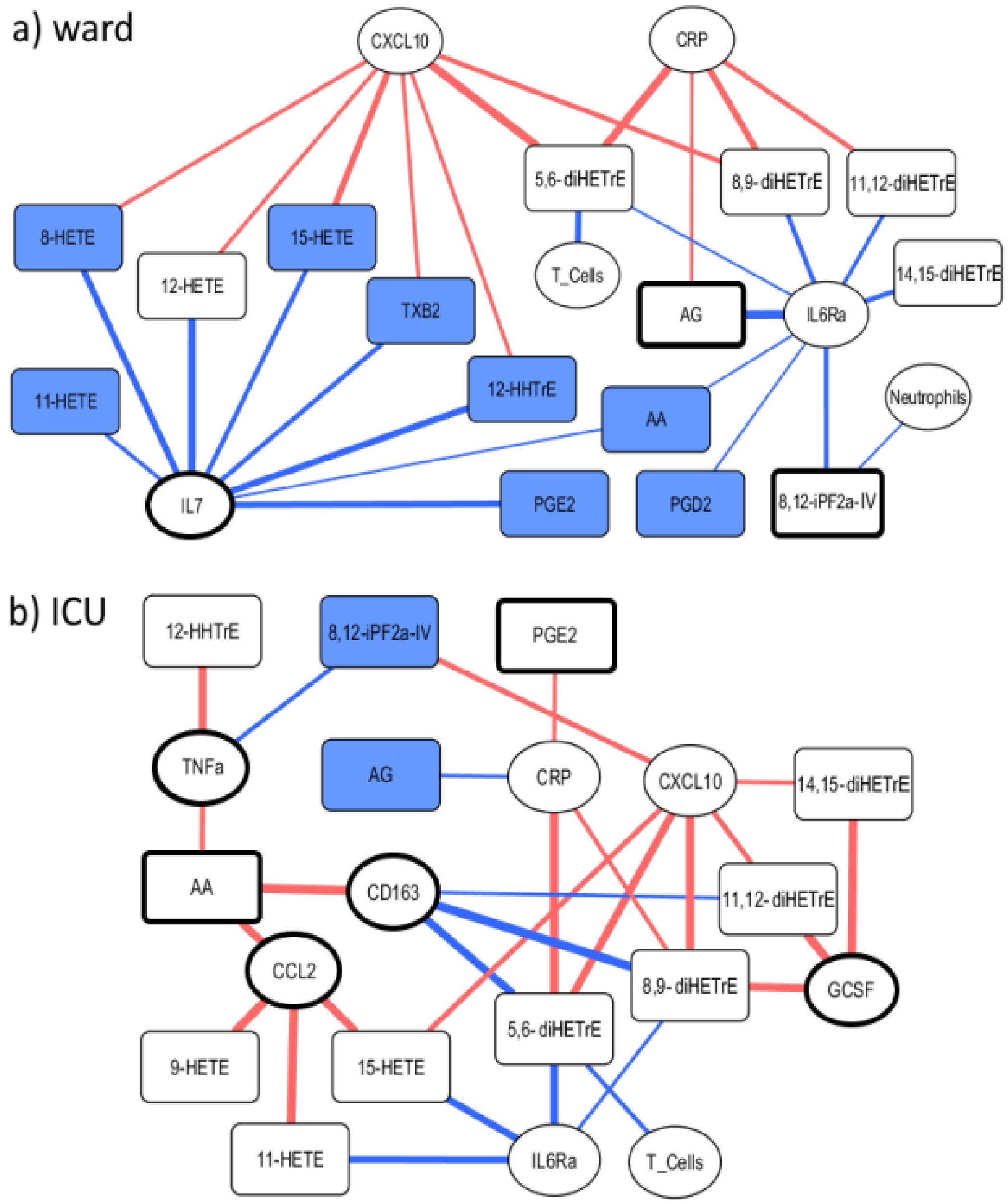

Figure S3. Correlation network between immune markers and the arachidonic acid pathway metabolites, in ward samples (a) and ICU samples (b). Circular nodes are immune markers, and square nodes are metabolites (in blue - elevated in that subset). Blue edges depict positive correlation, and red negative correlation, with line width corresponding with |R| (between 0.3 and 0.7). Nodes with thick frame are of special interest due to differences between the two groups. The correlation matrices are provided in Tables S13-14.
