# Supplementary figures and images for: Plasma Oxylipins and Their Precursors Are Strongly Associated with COVID-19 Severity and with Immune Response Markers
Source: Metabolites. 2022 Jul 4;12(7):619. doi: 10.3390/metabo12070619 (PMC9319897; doi:10.3390/metabo12070619)

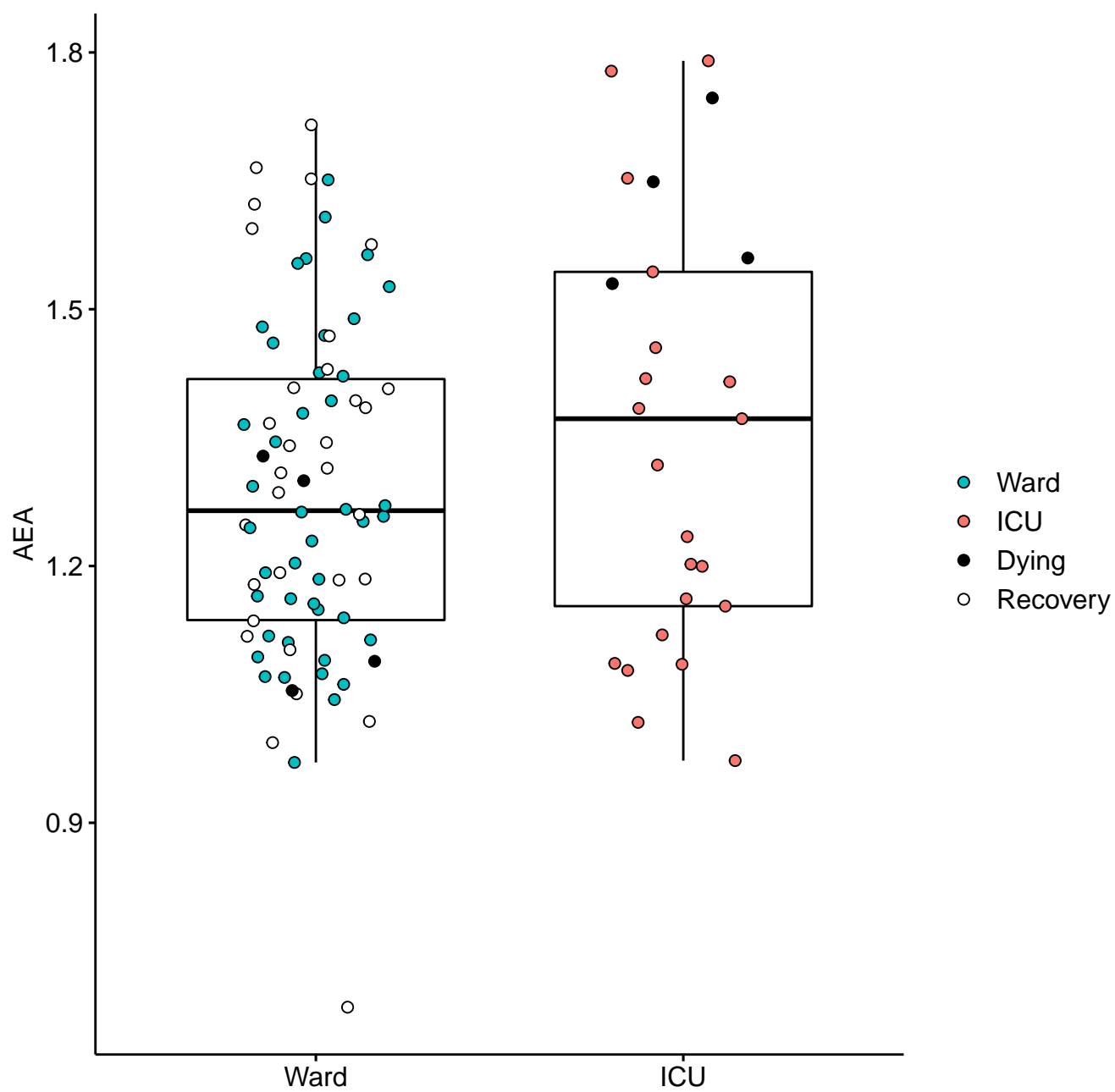

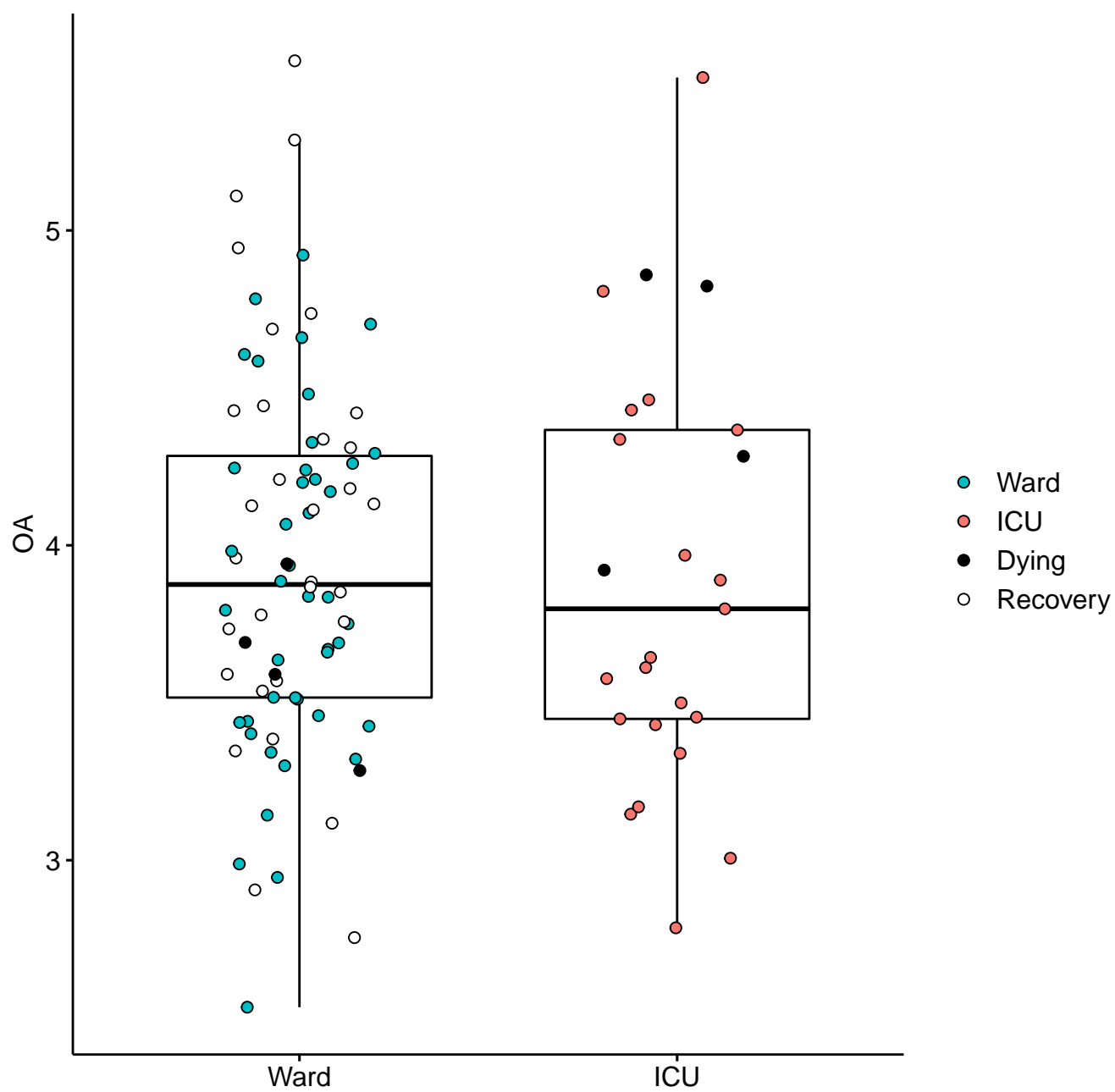

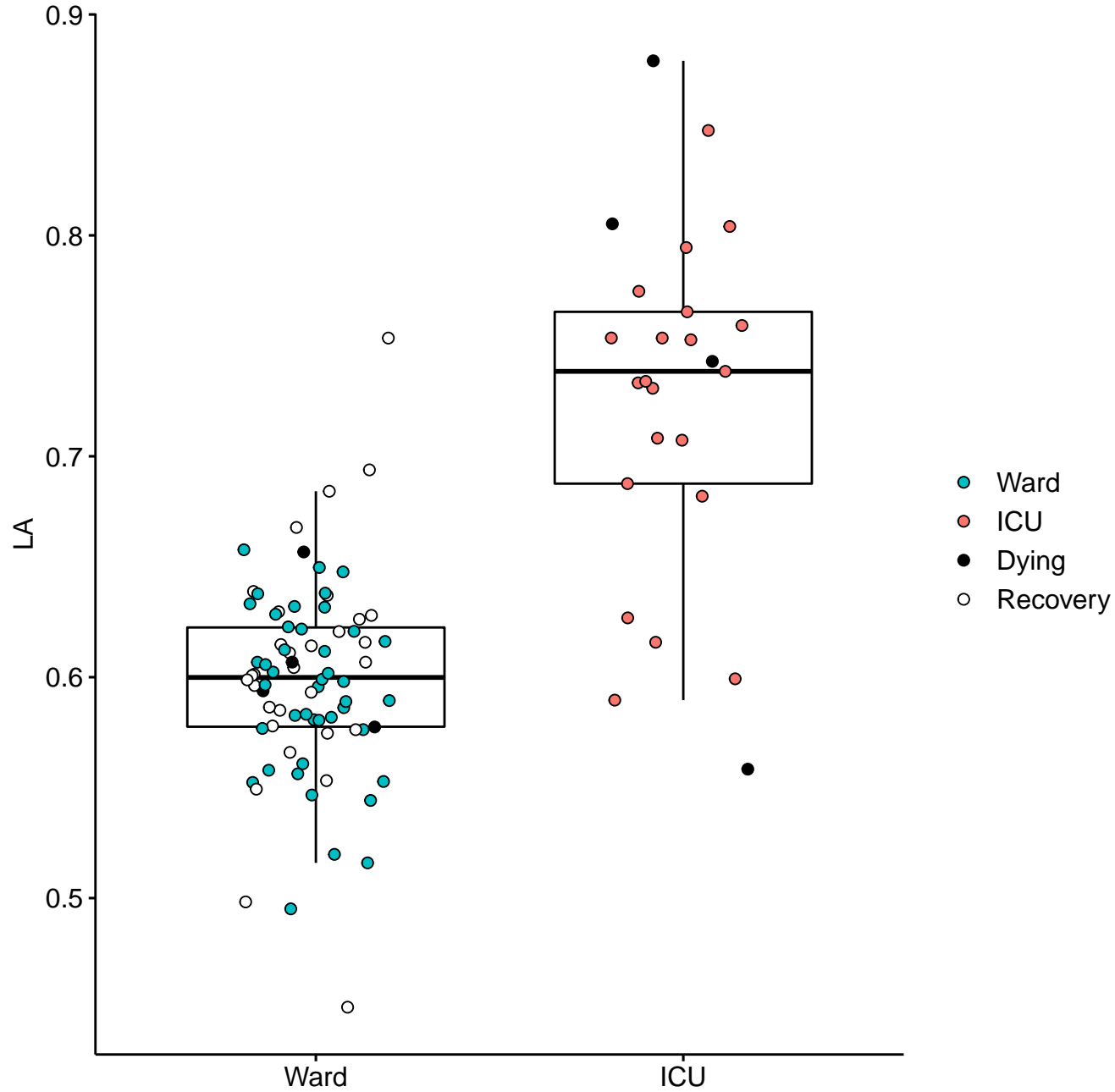

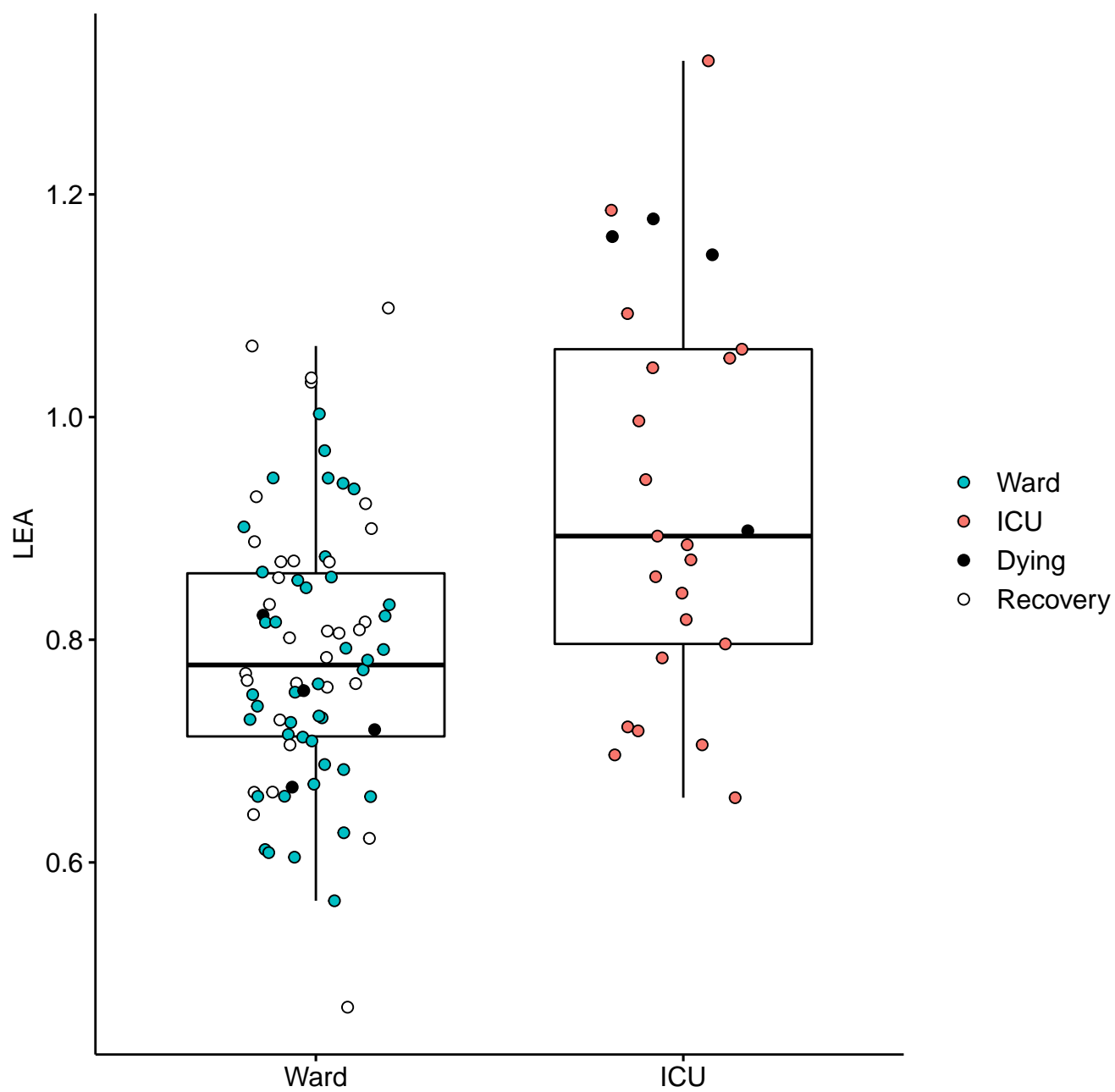

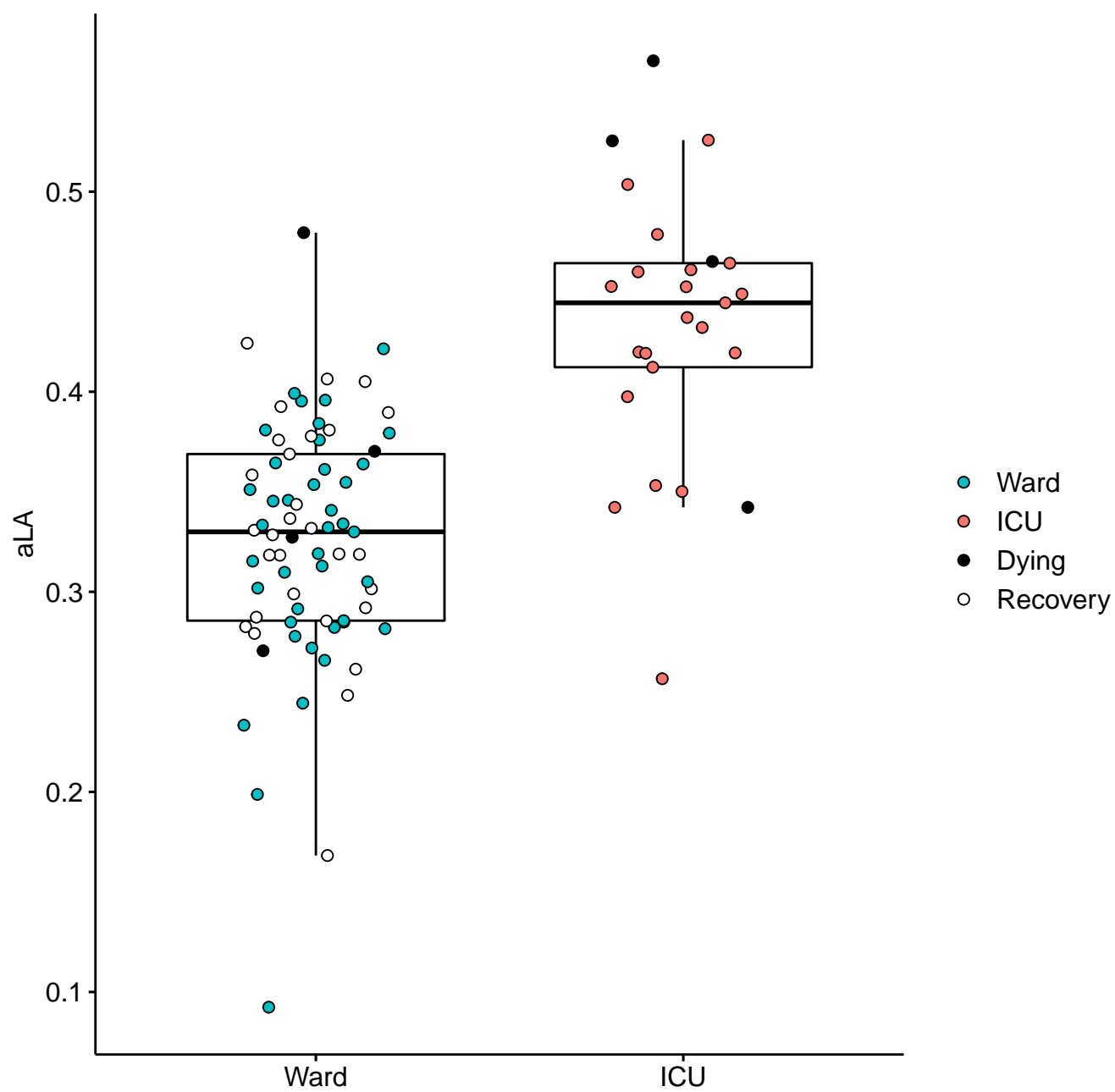

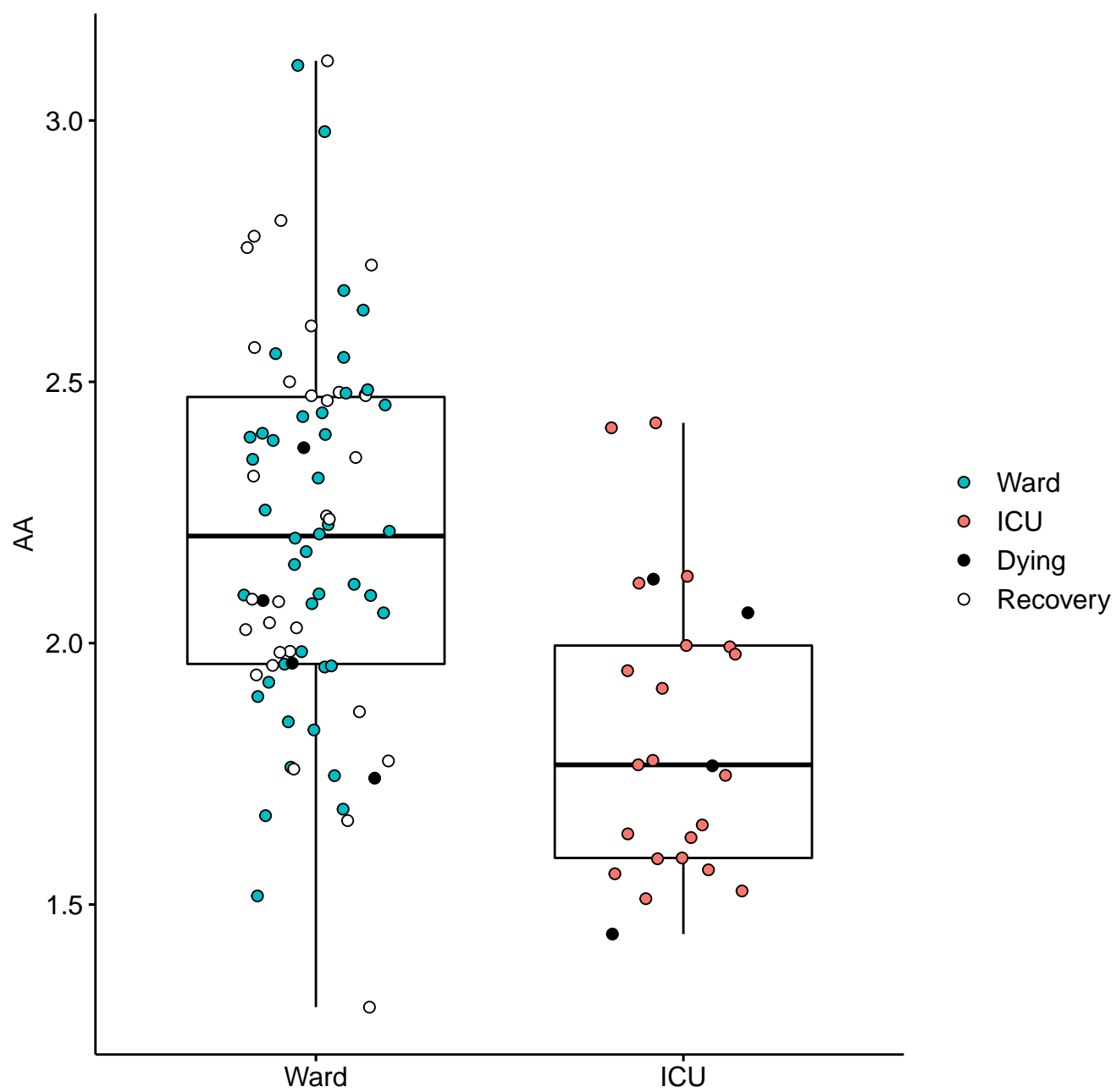

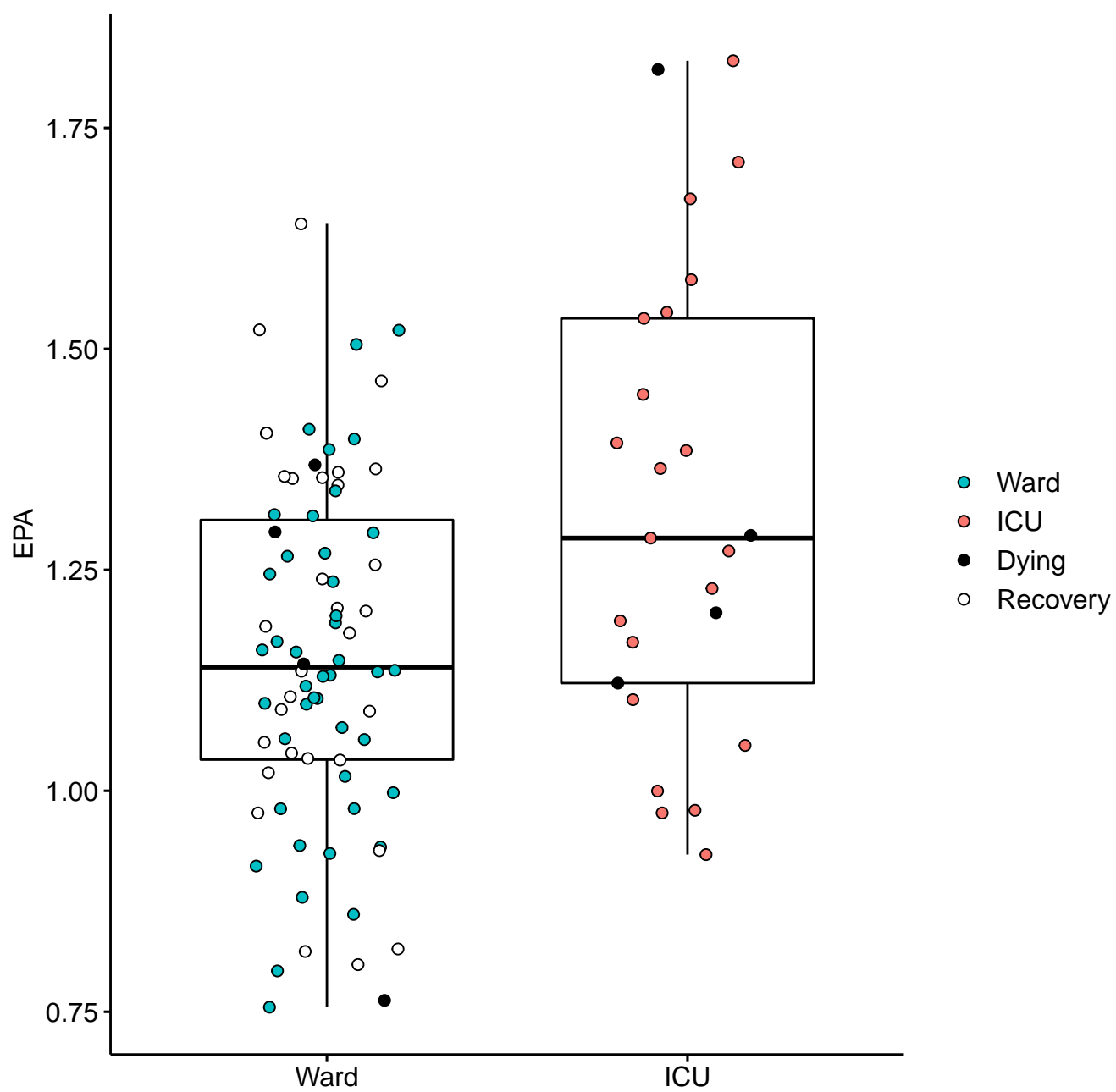

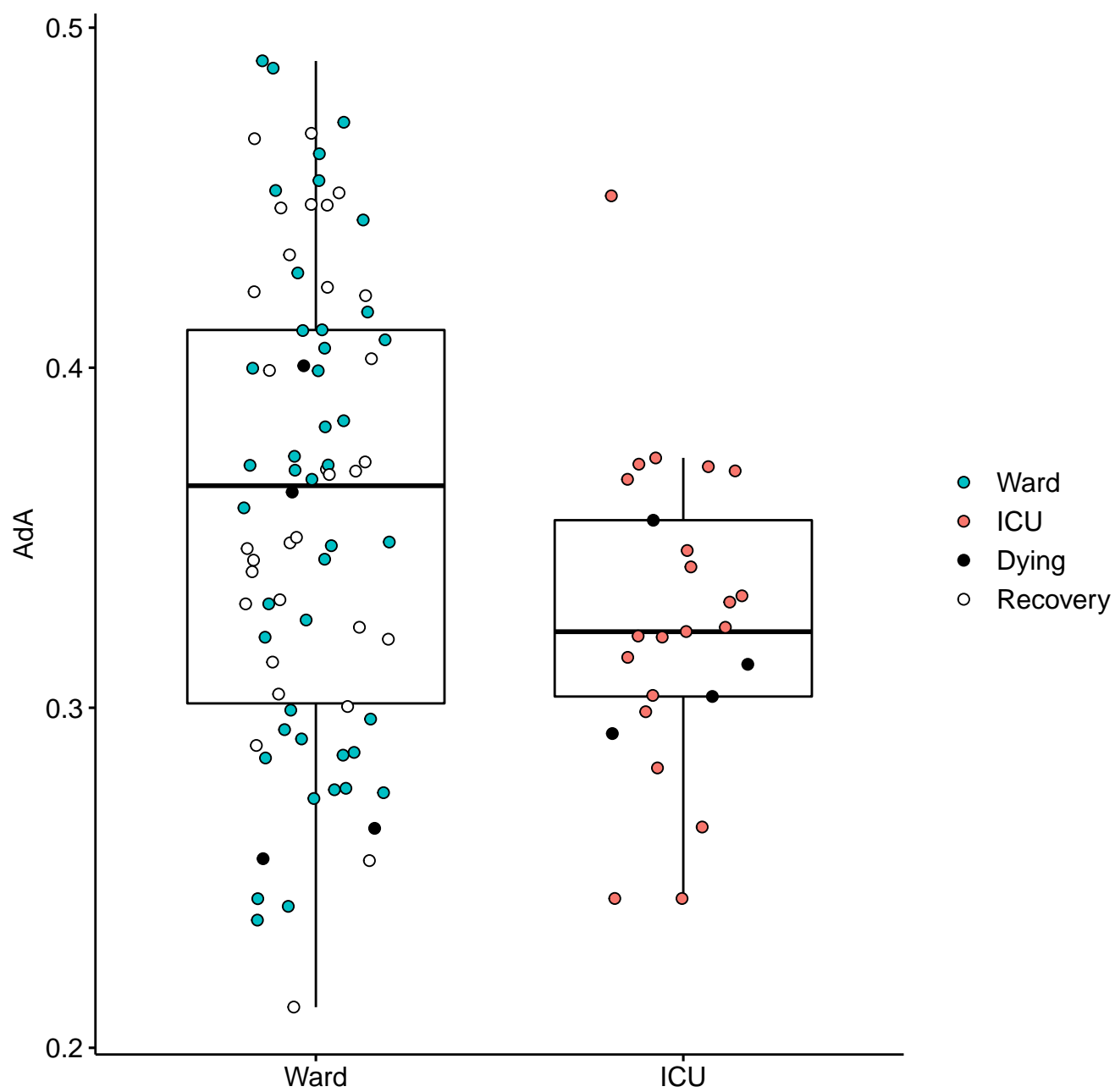

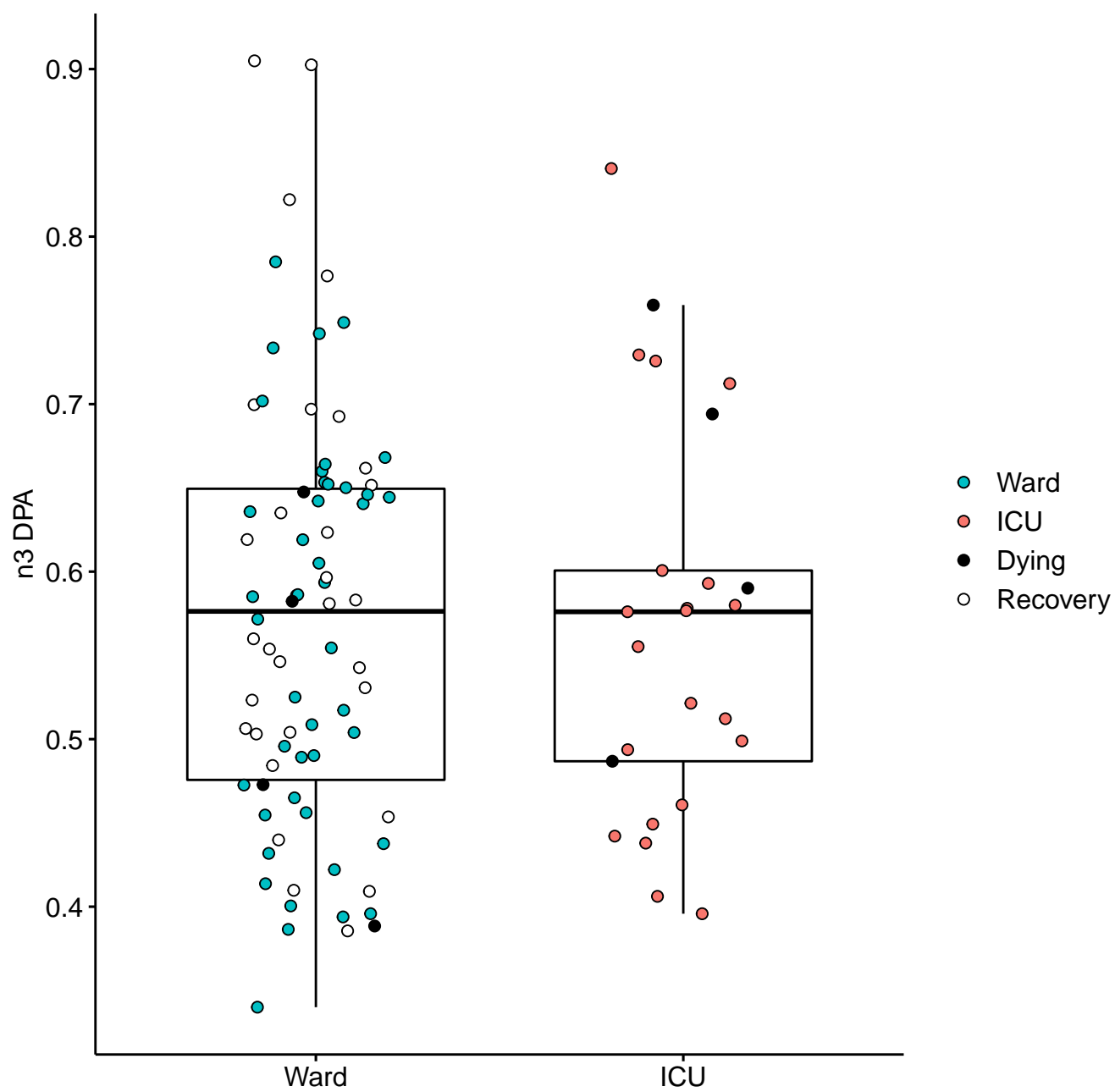

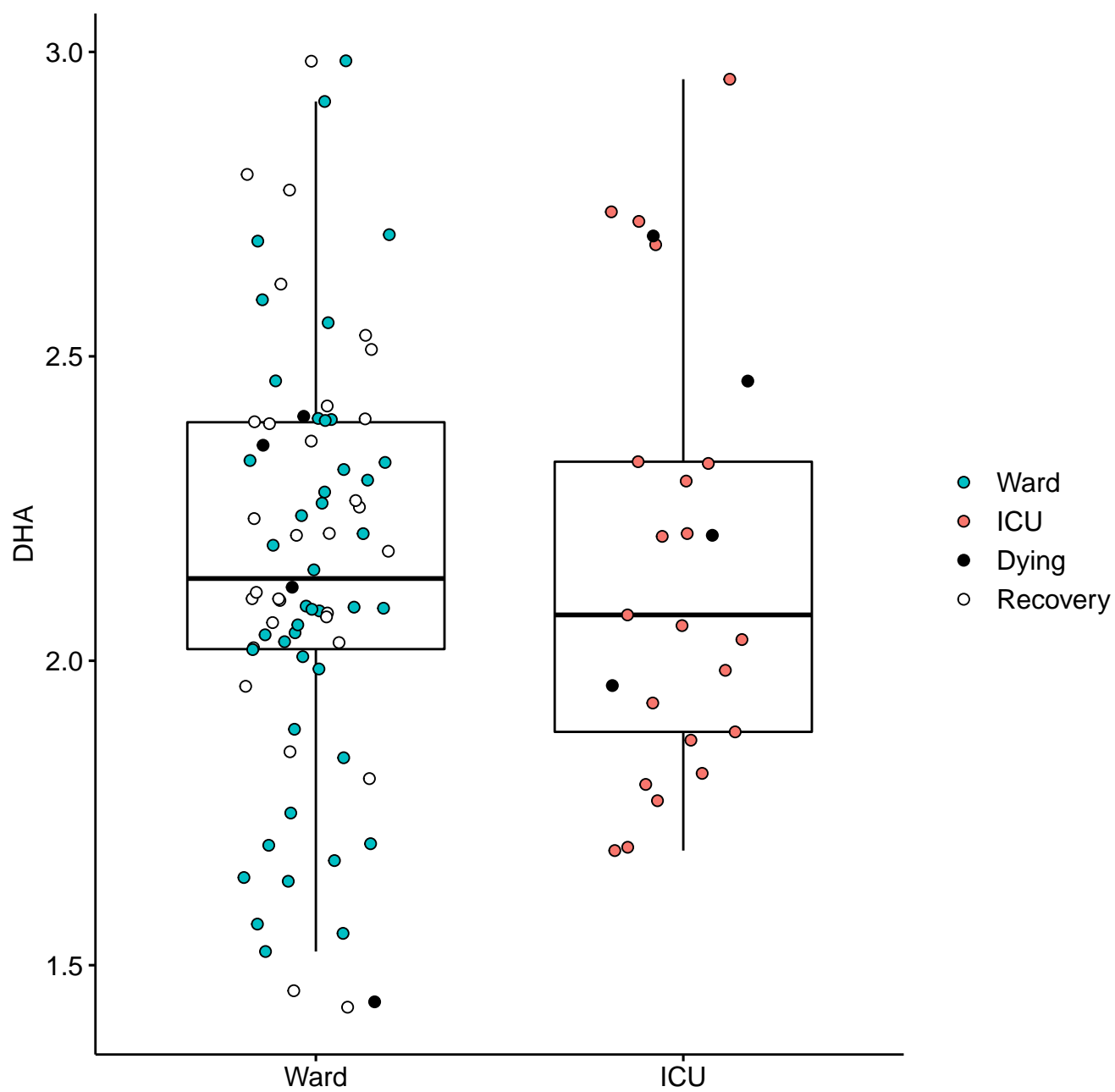

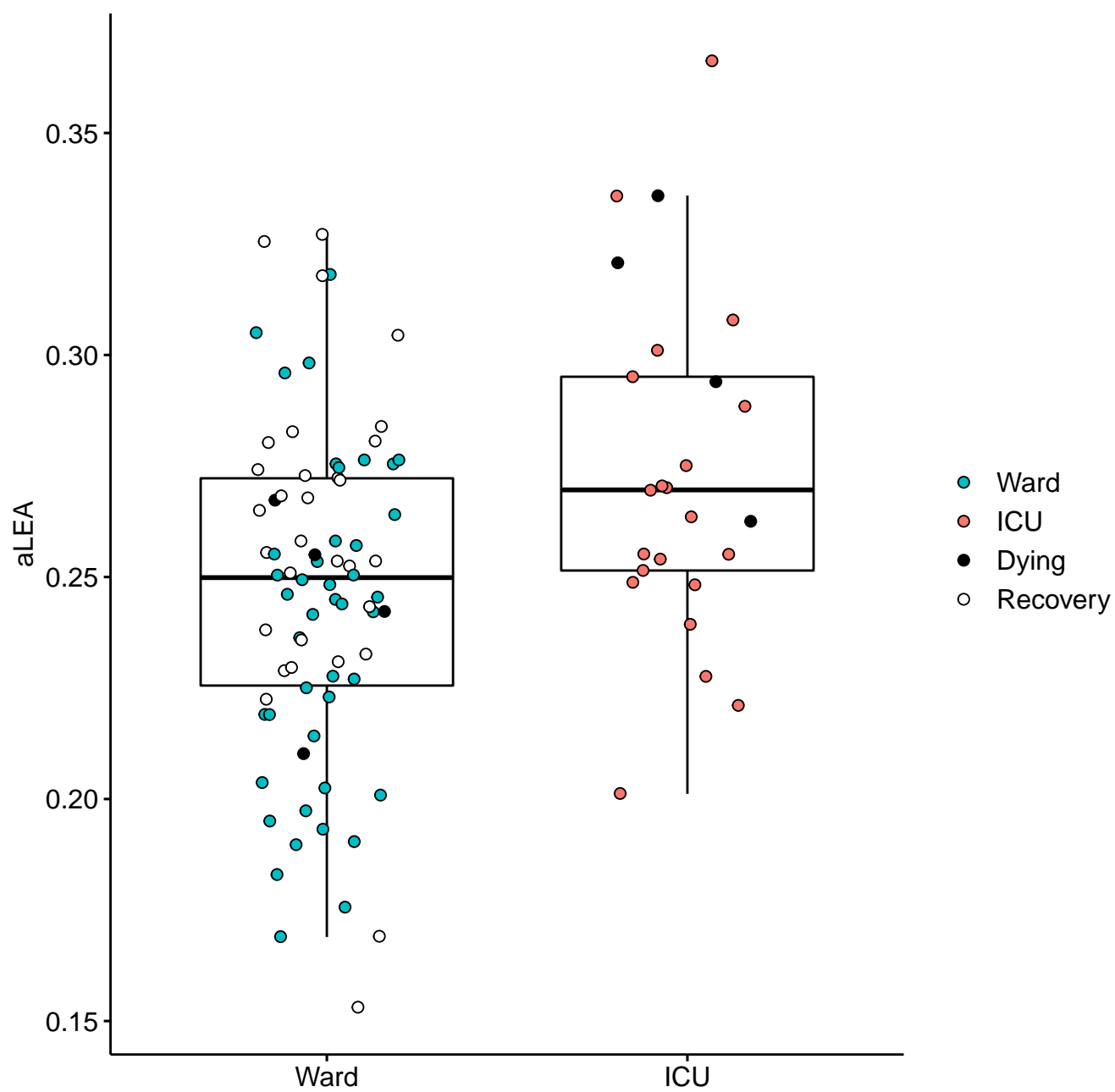

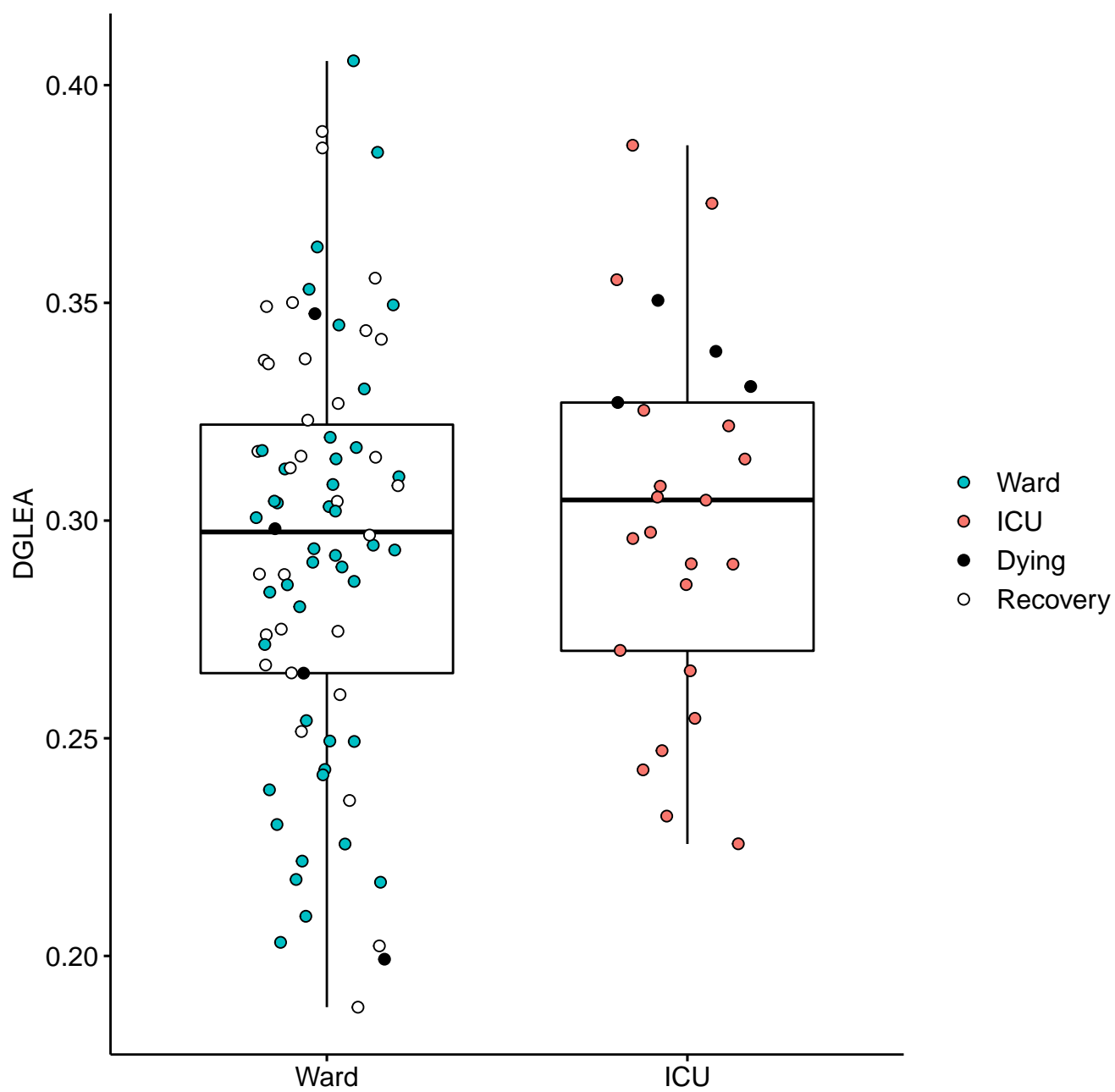

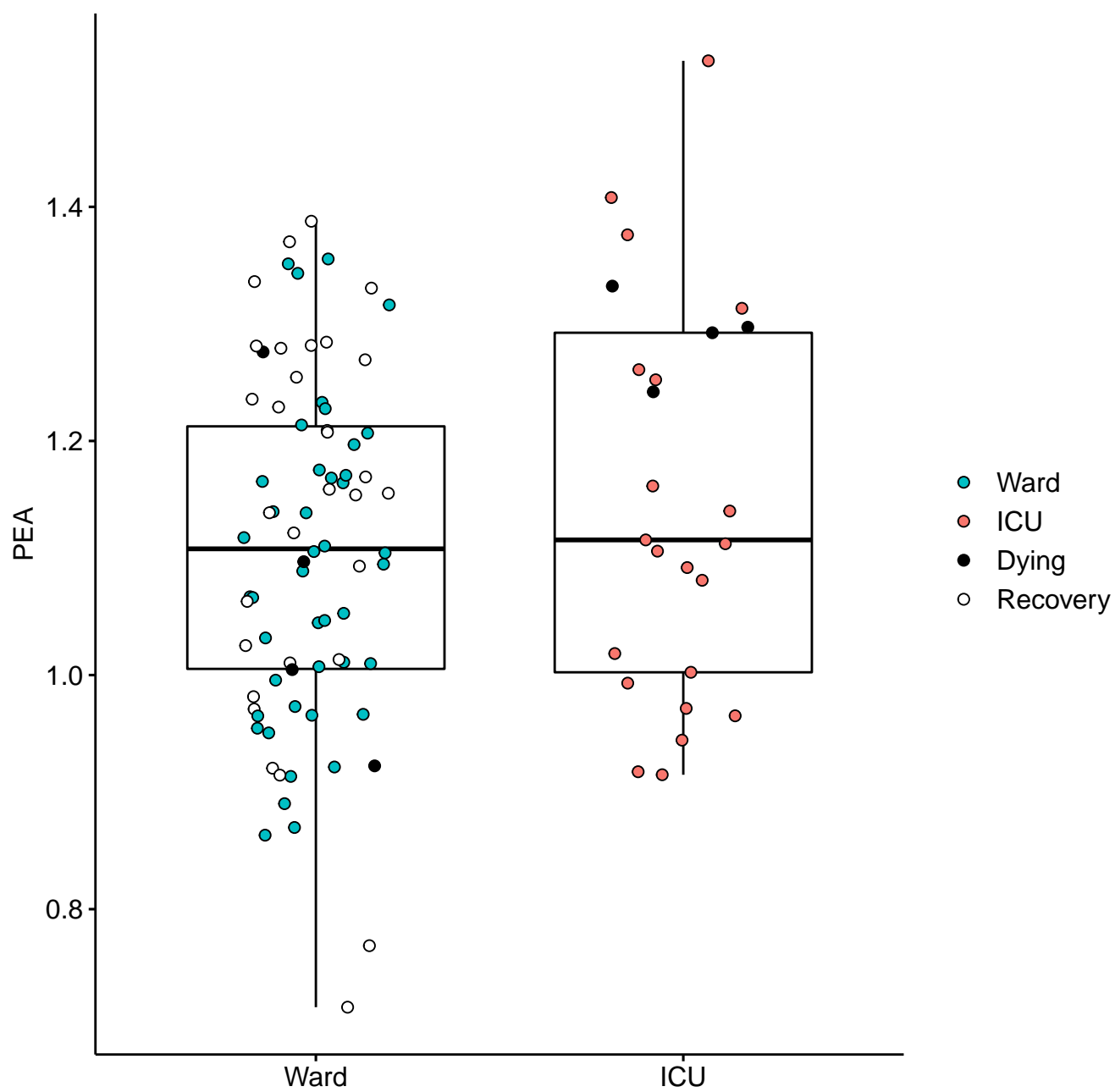

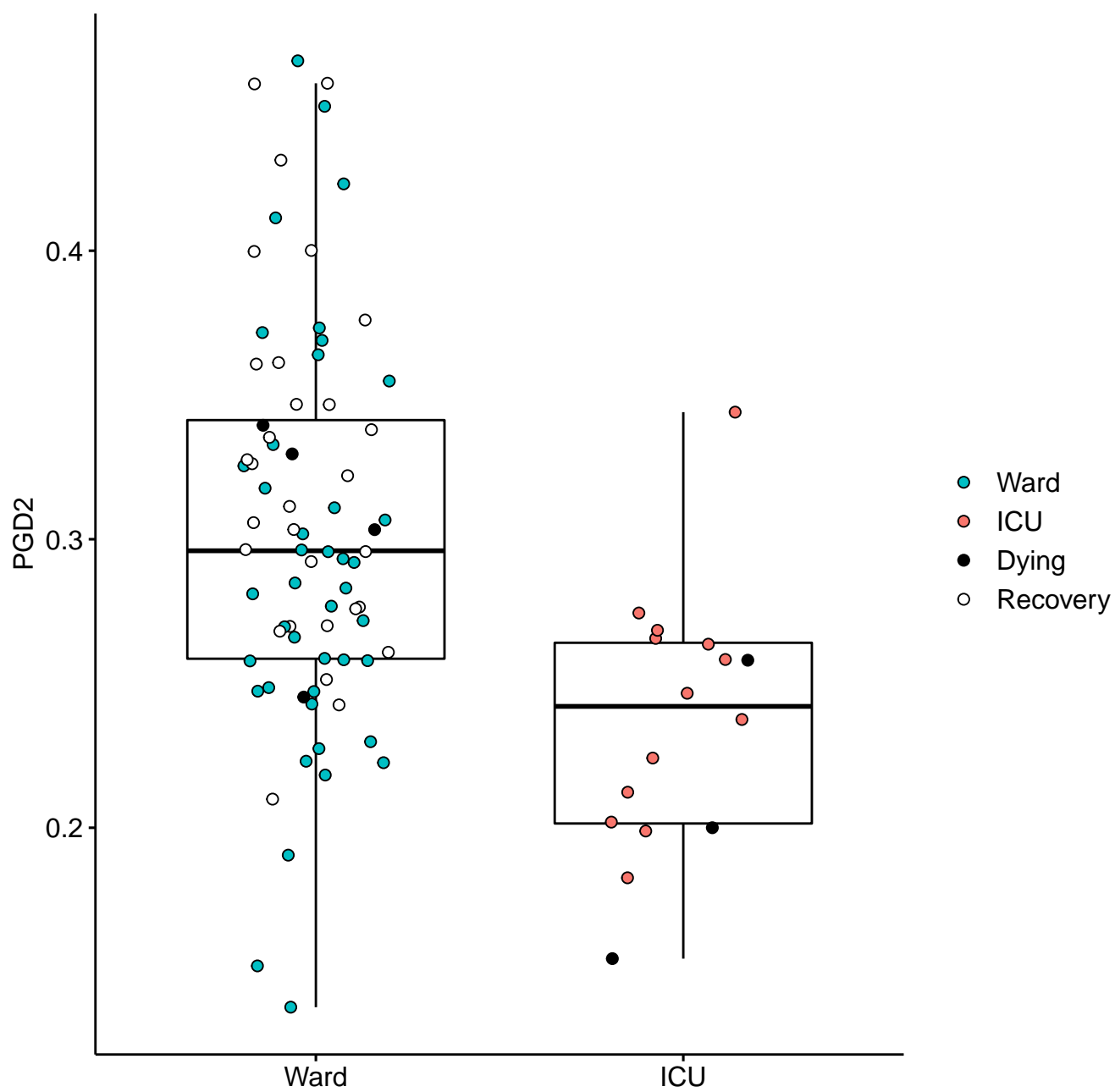

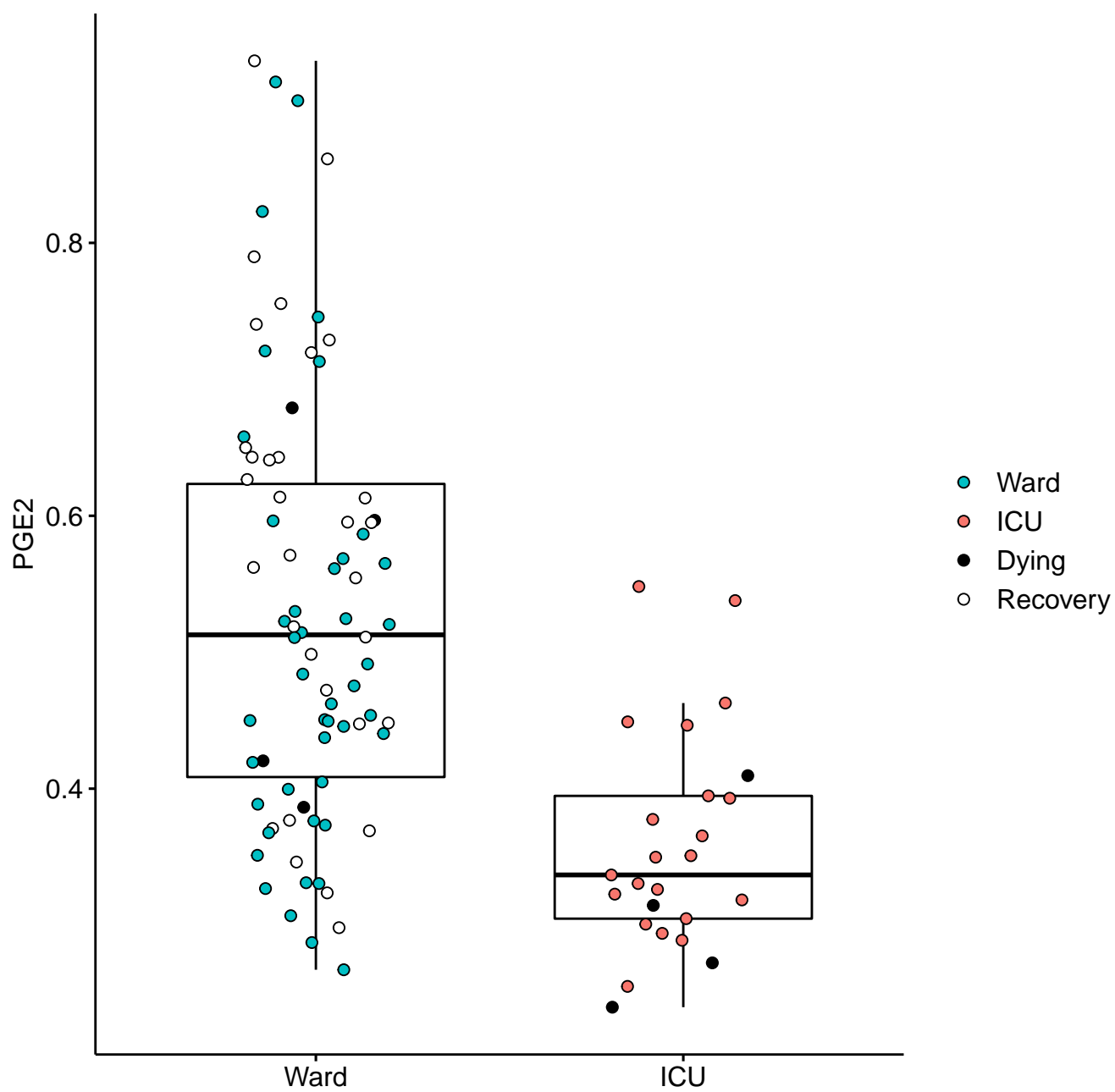

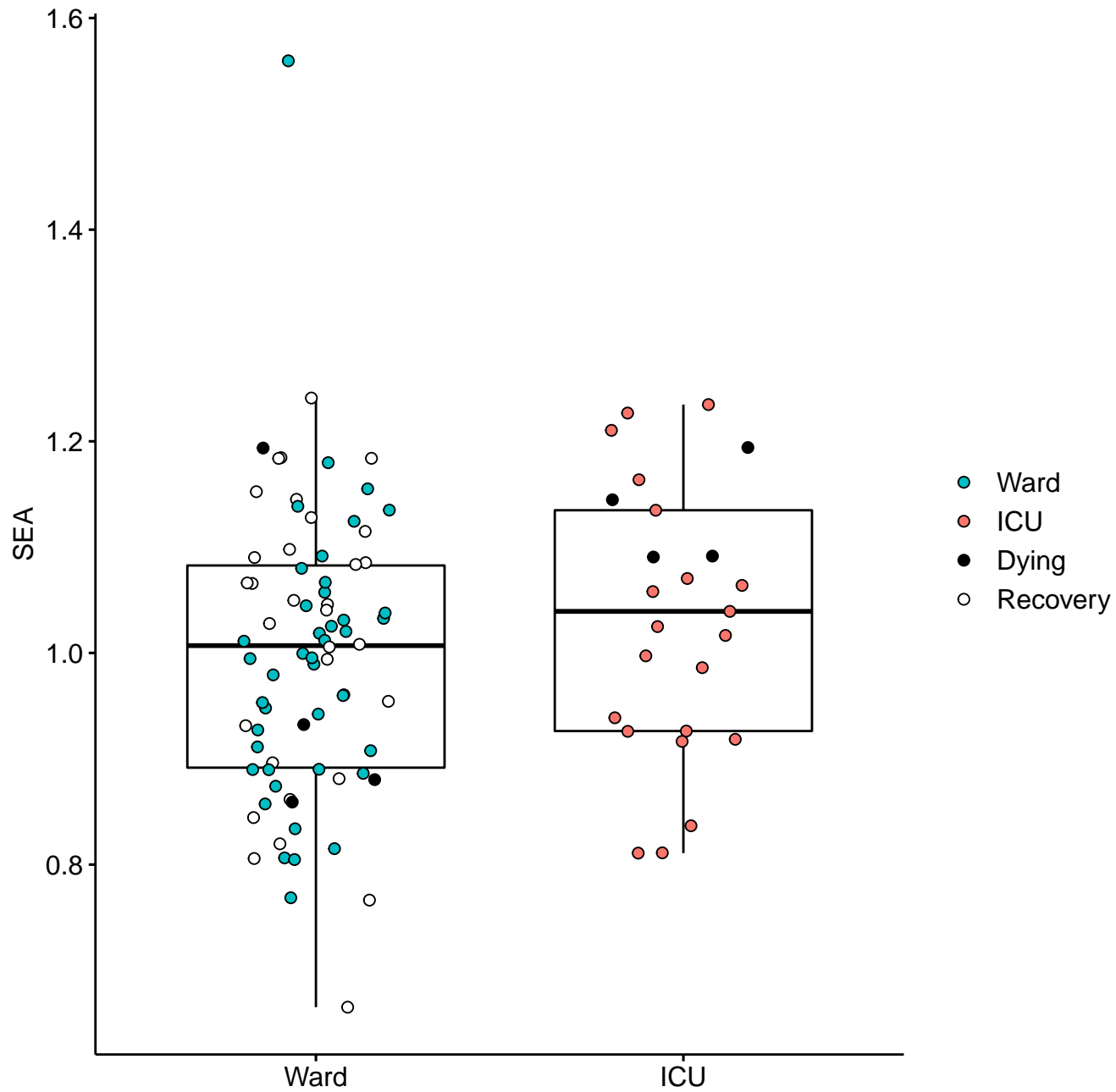

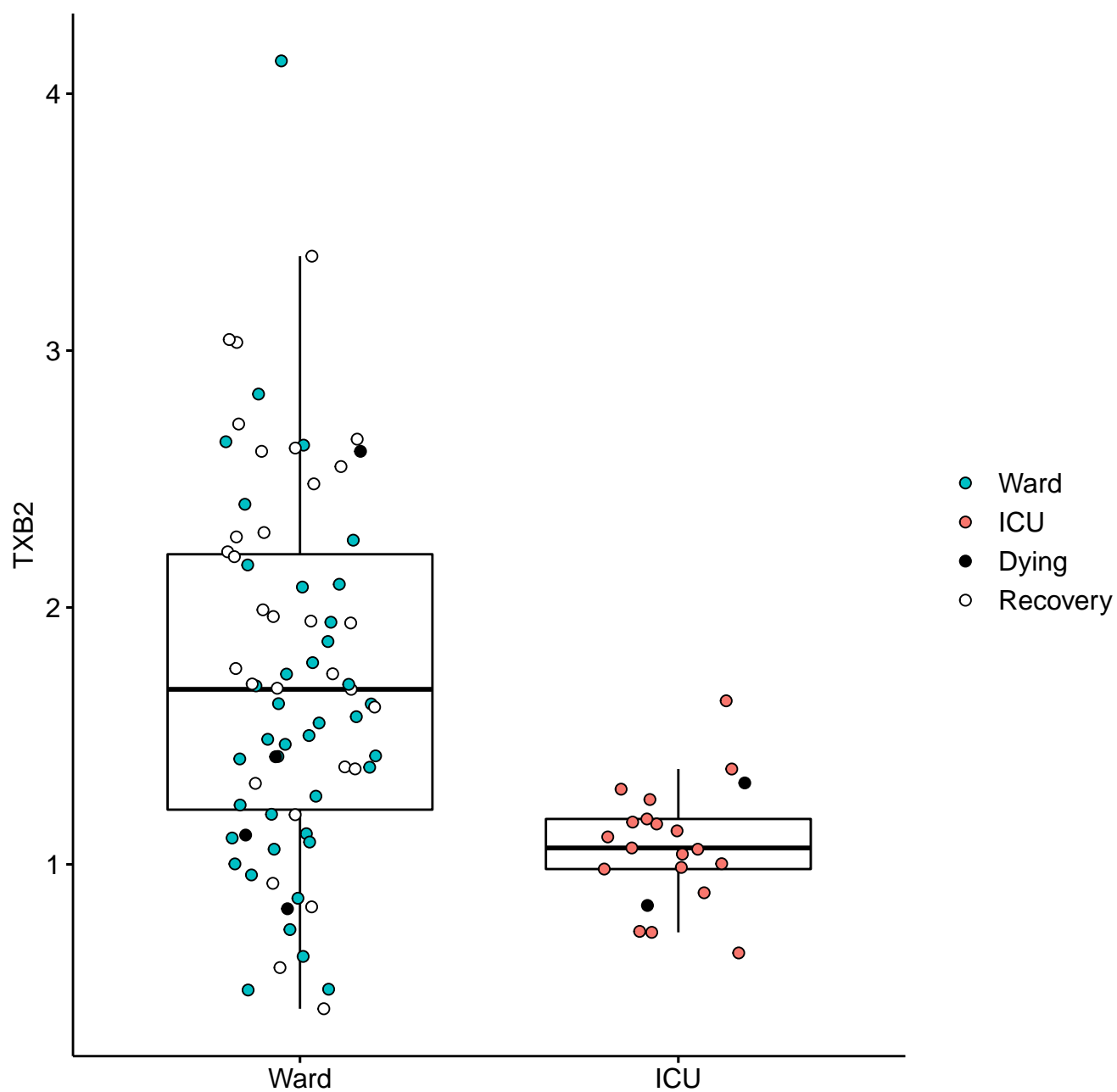

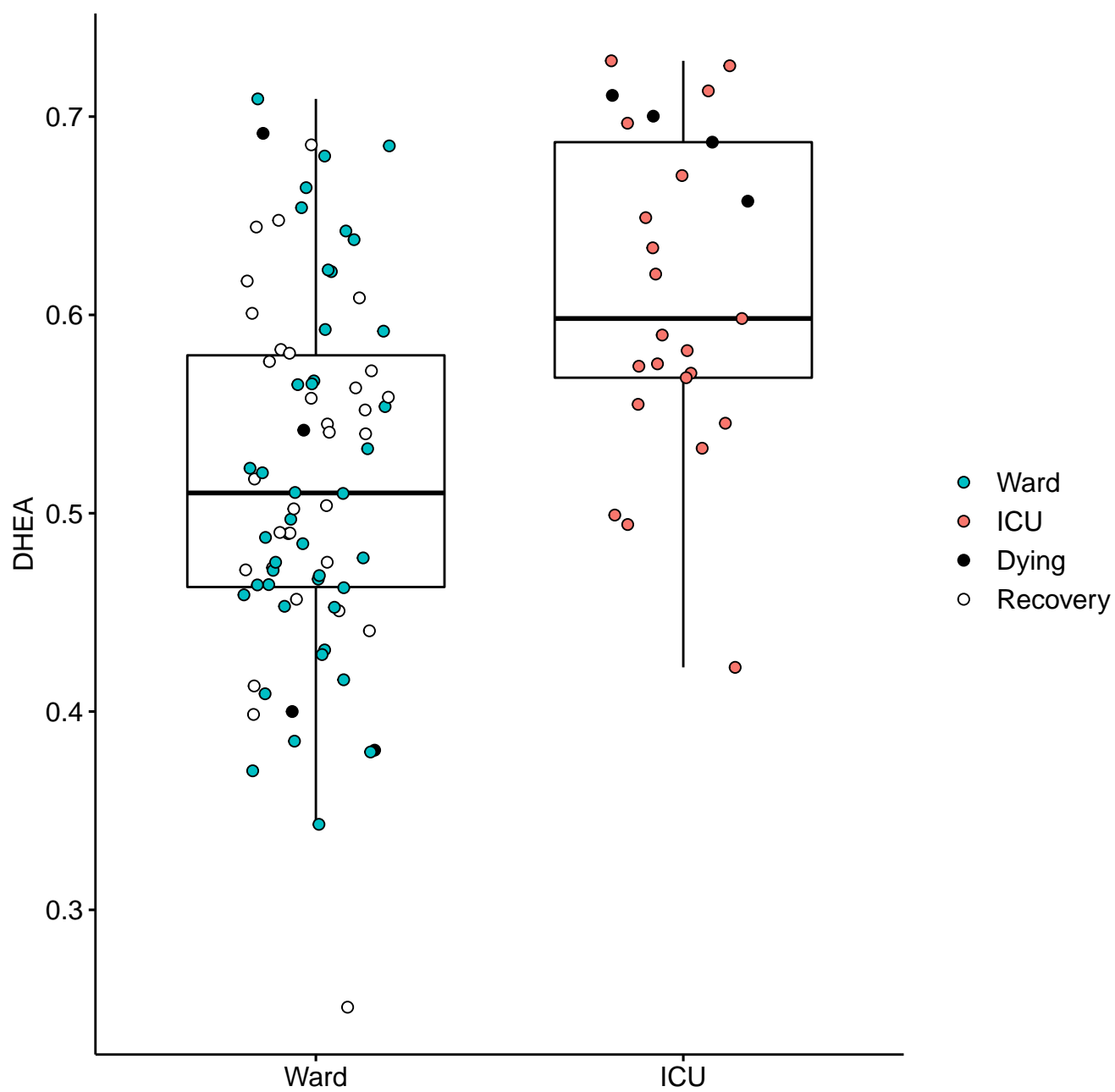

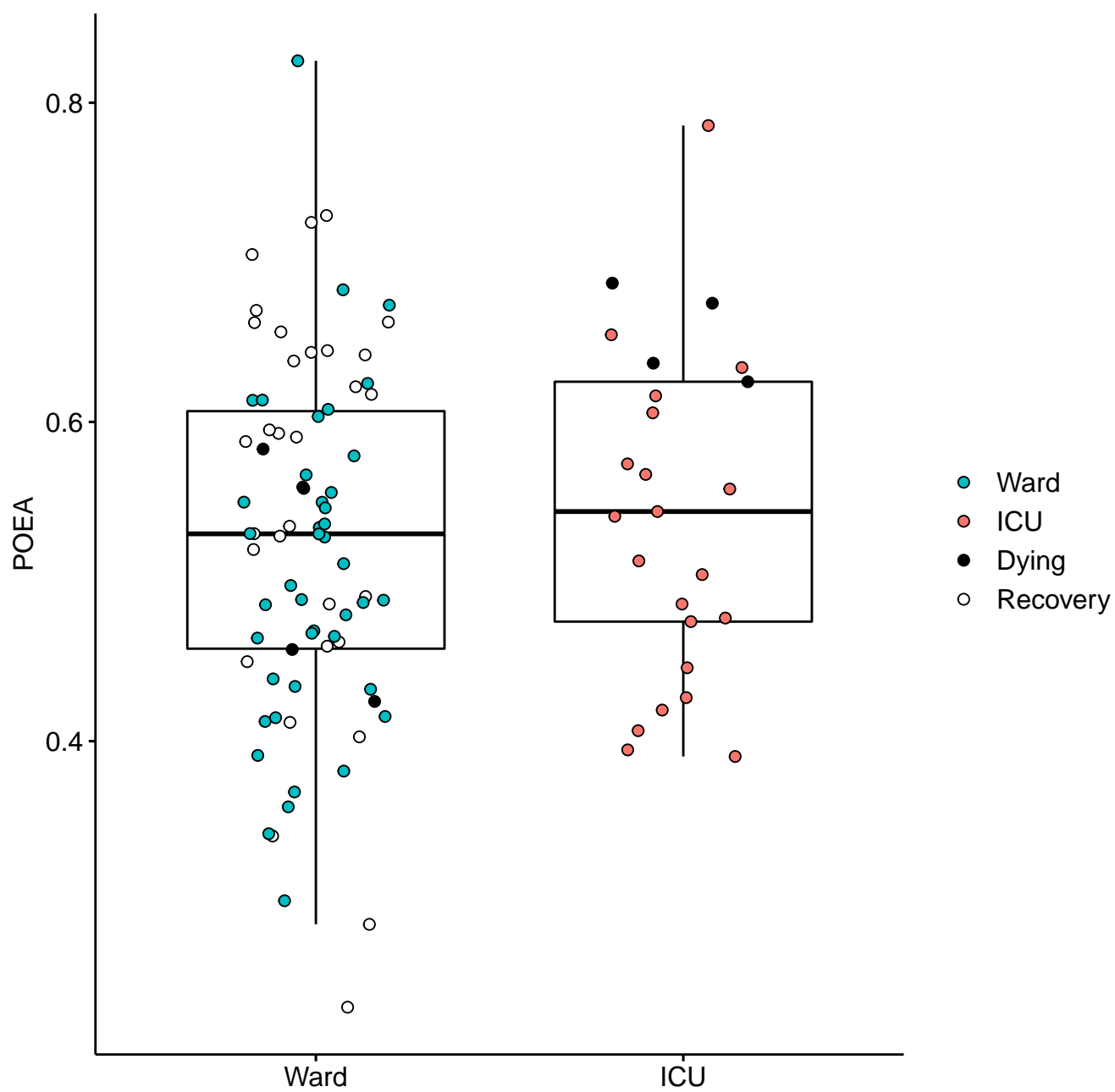

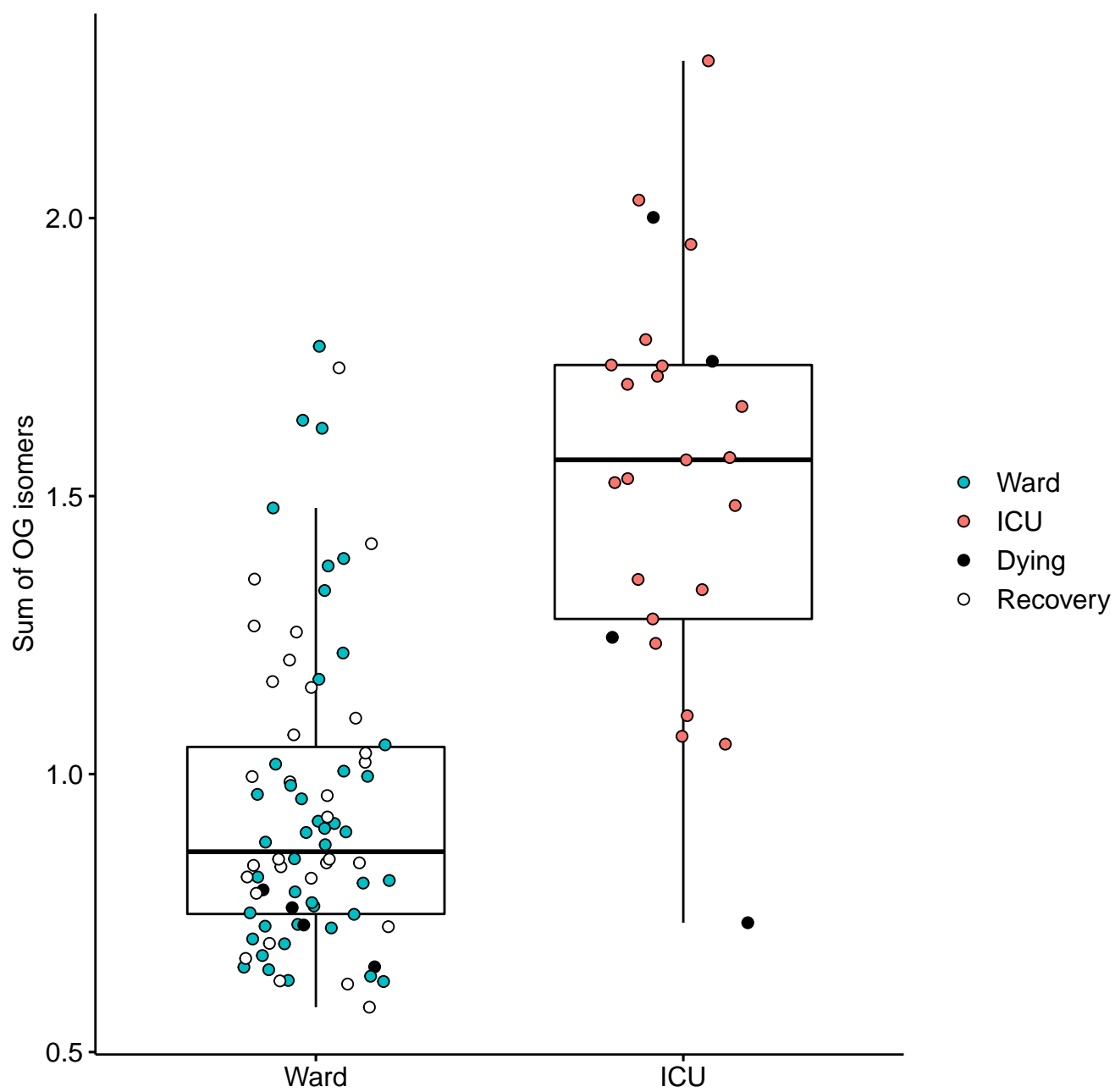

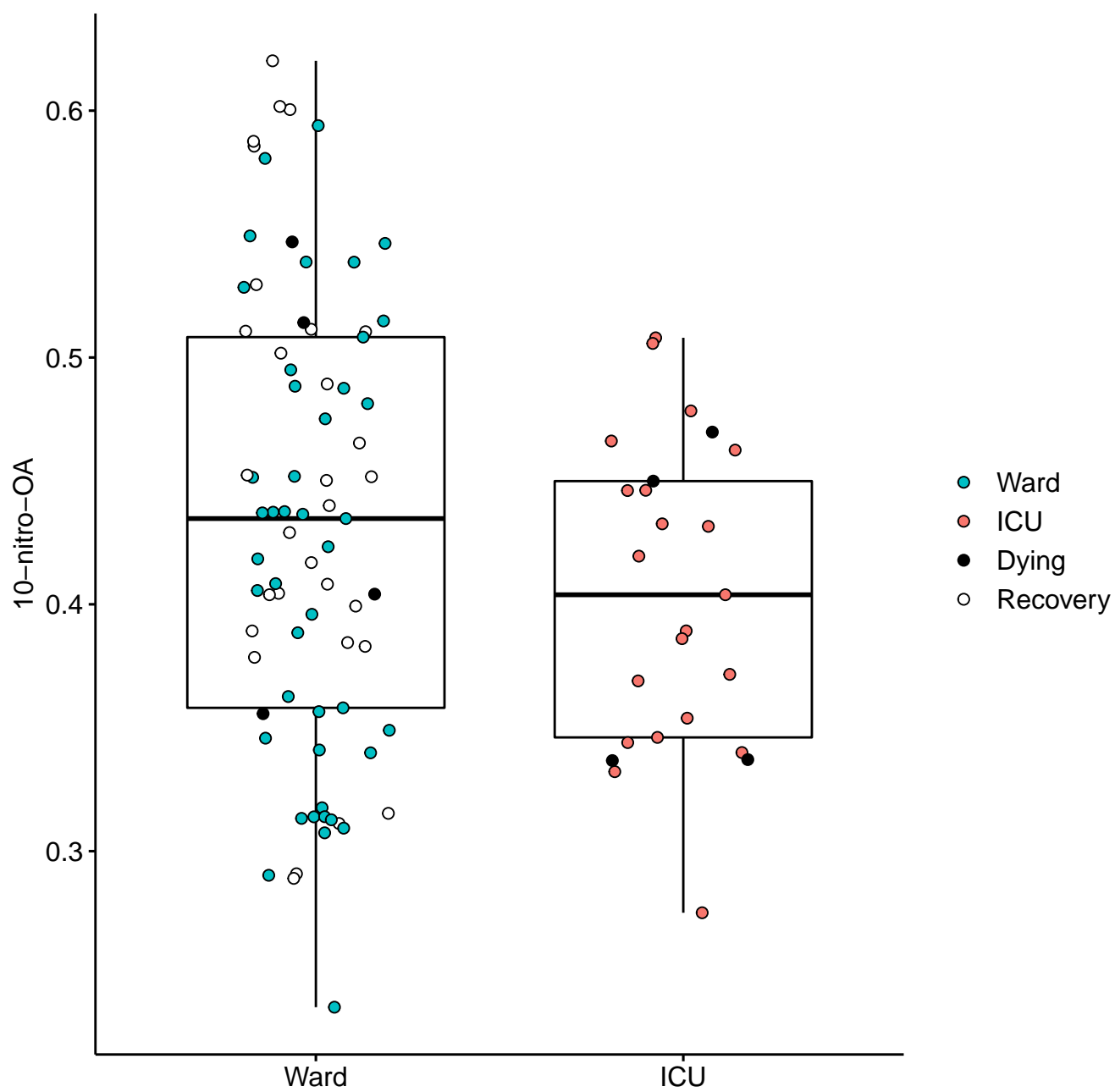

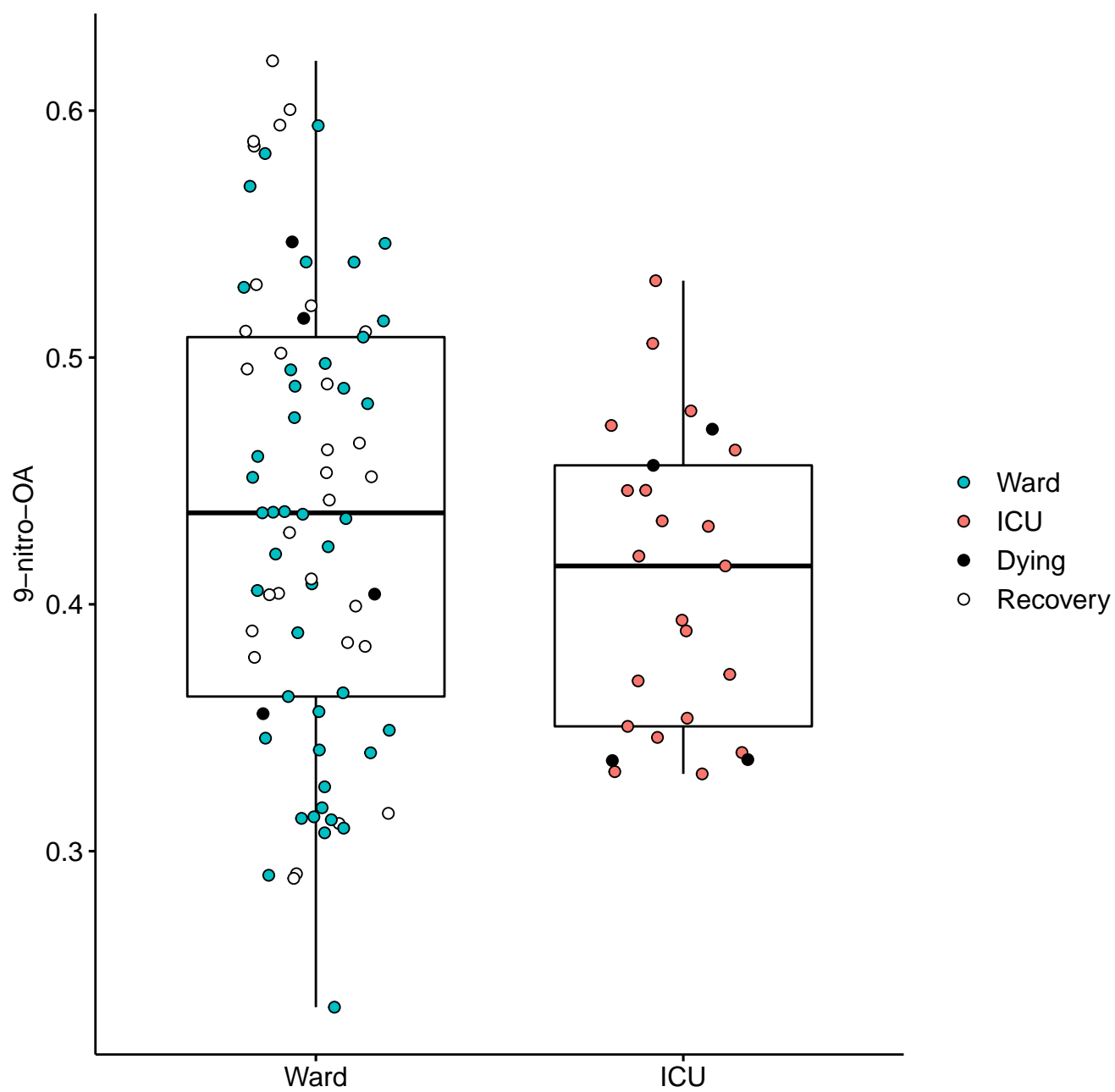

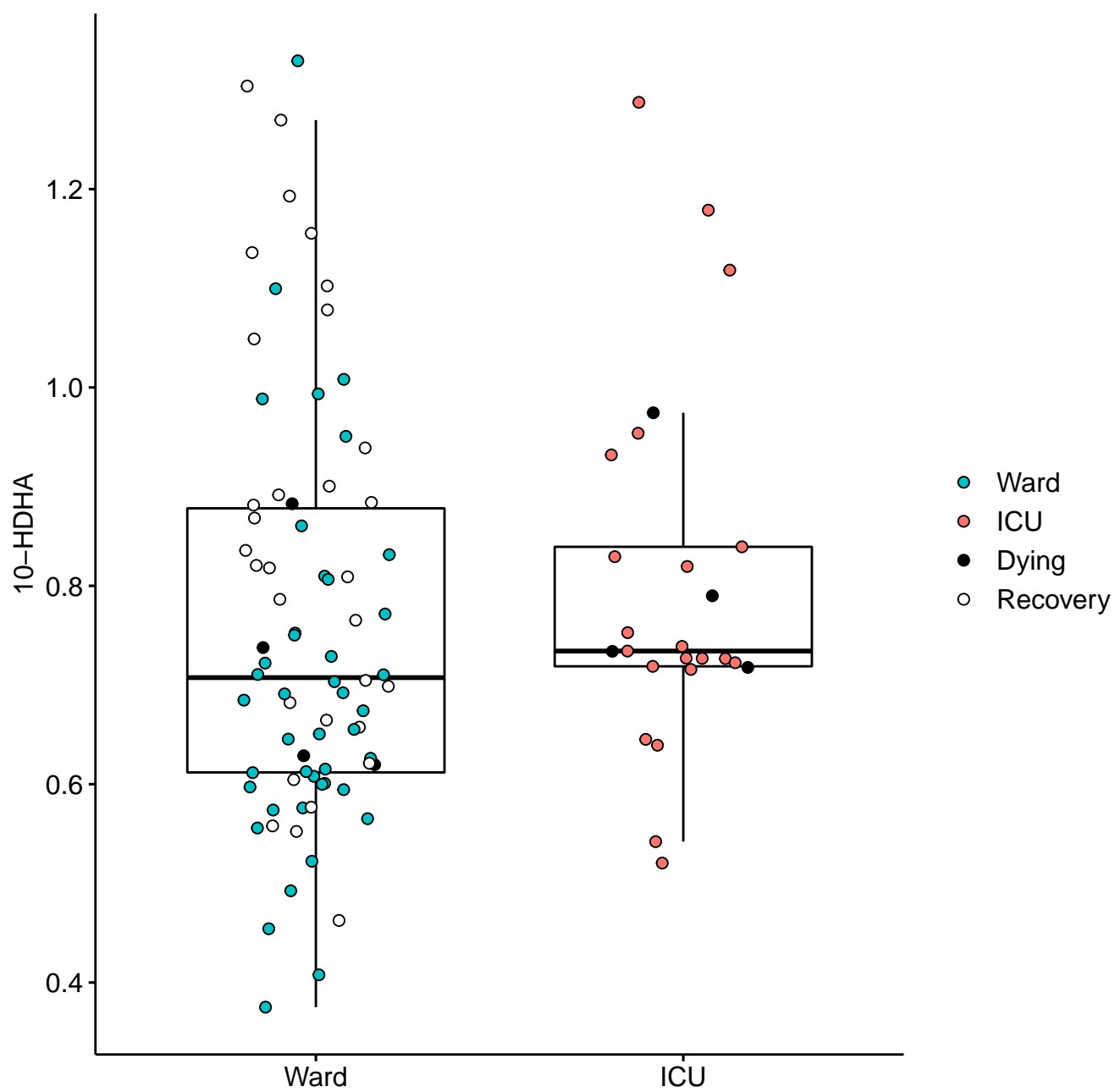

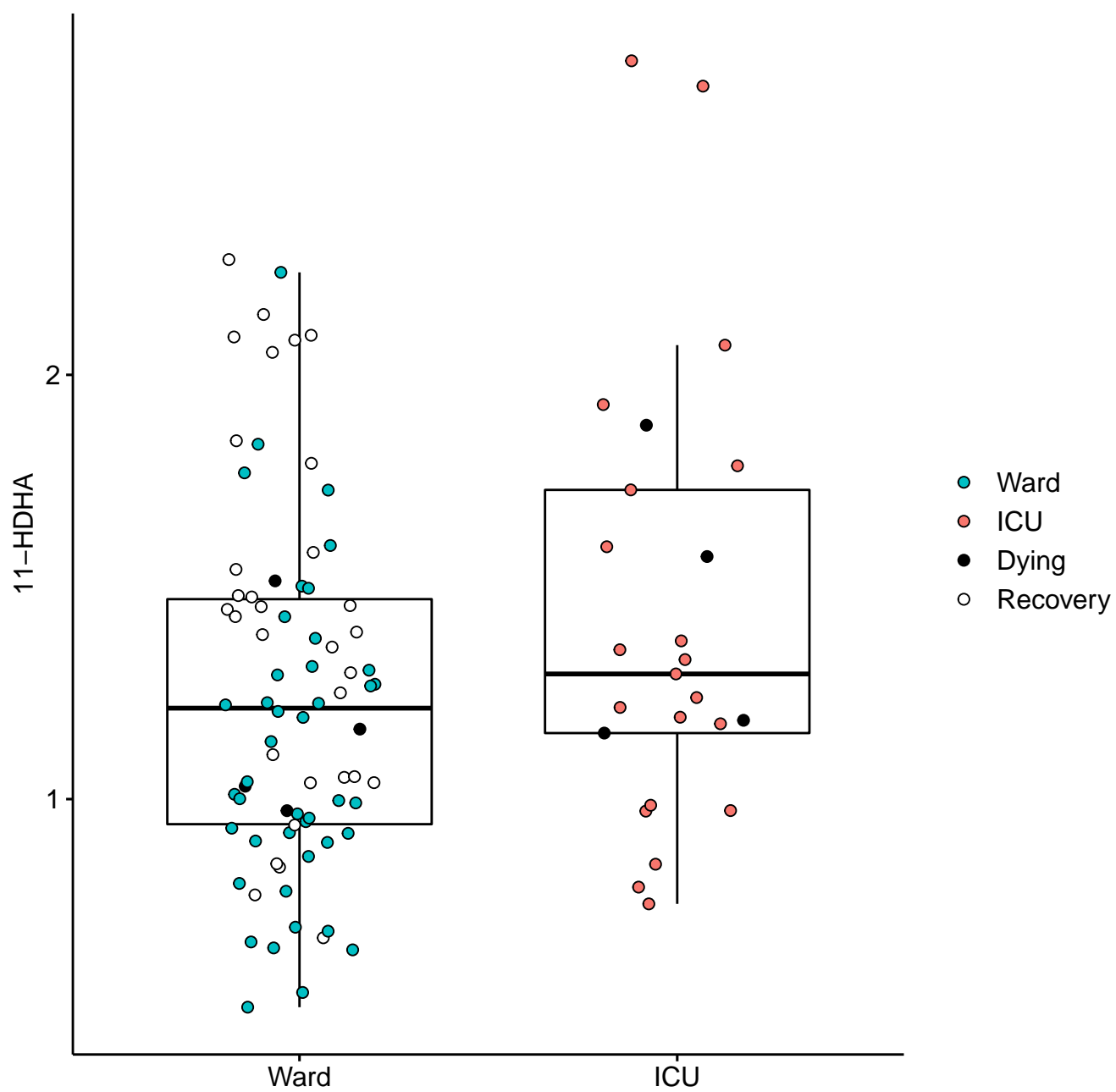

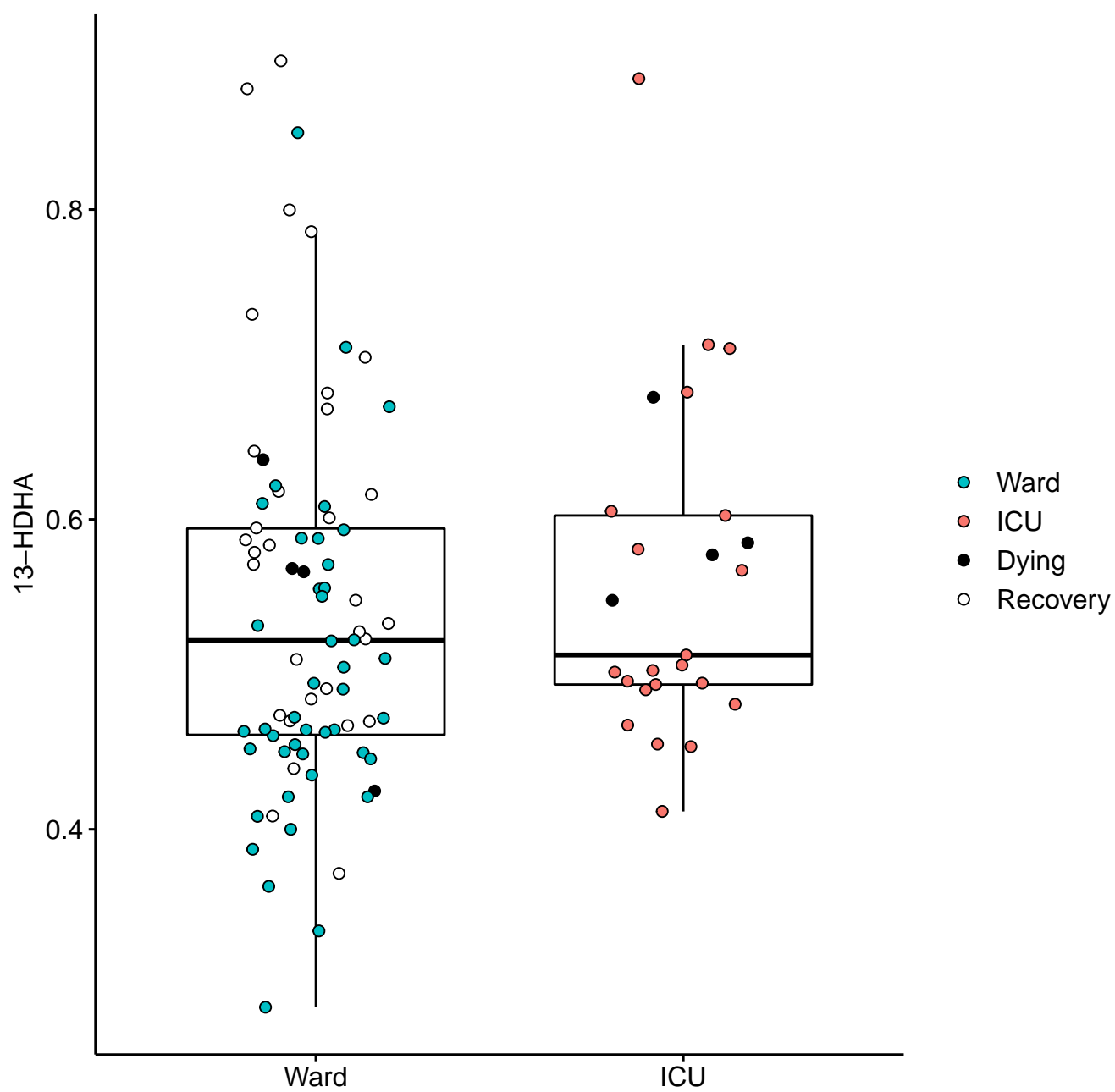

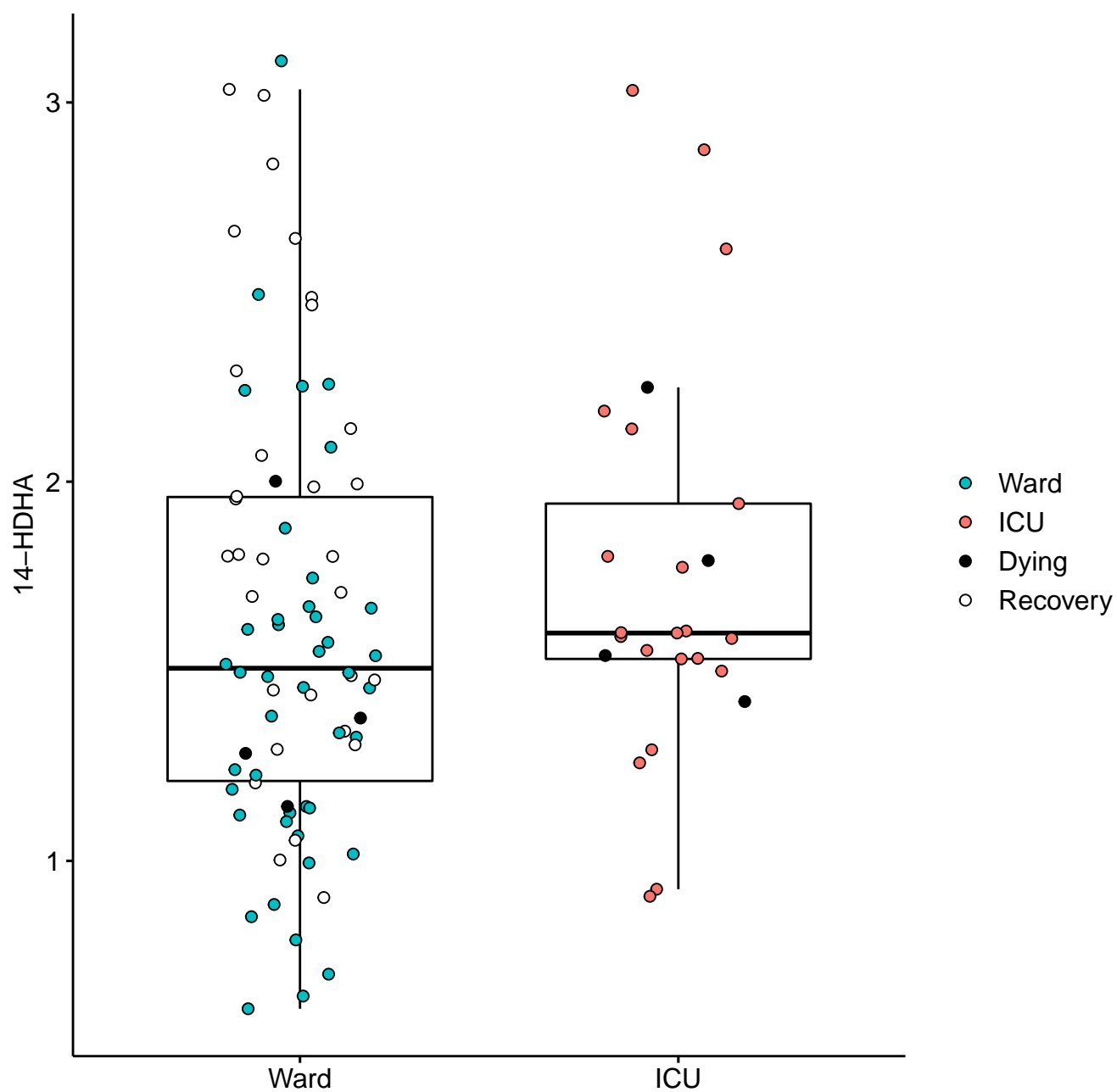

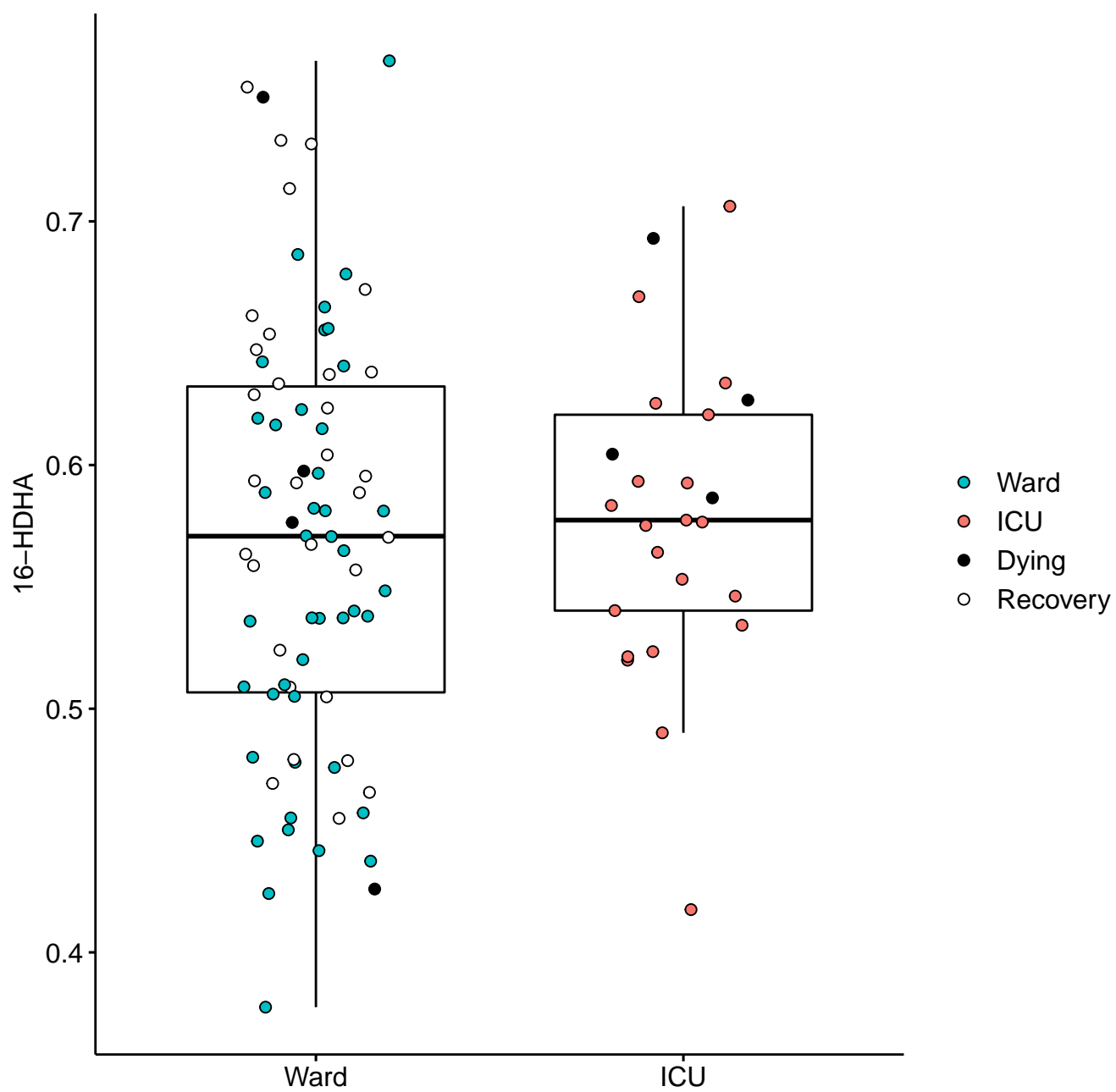

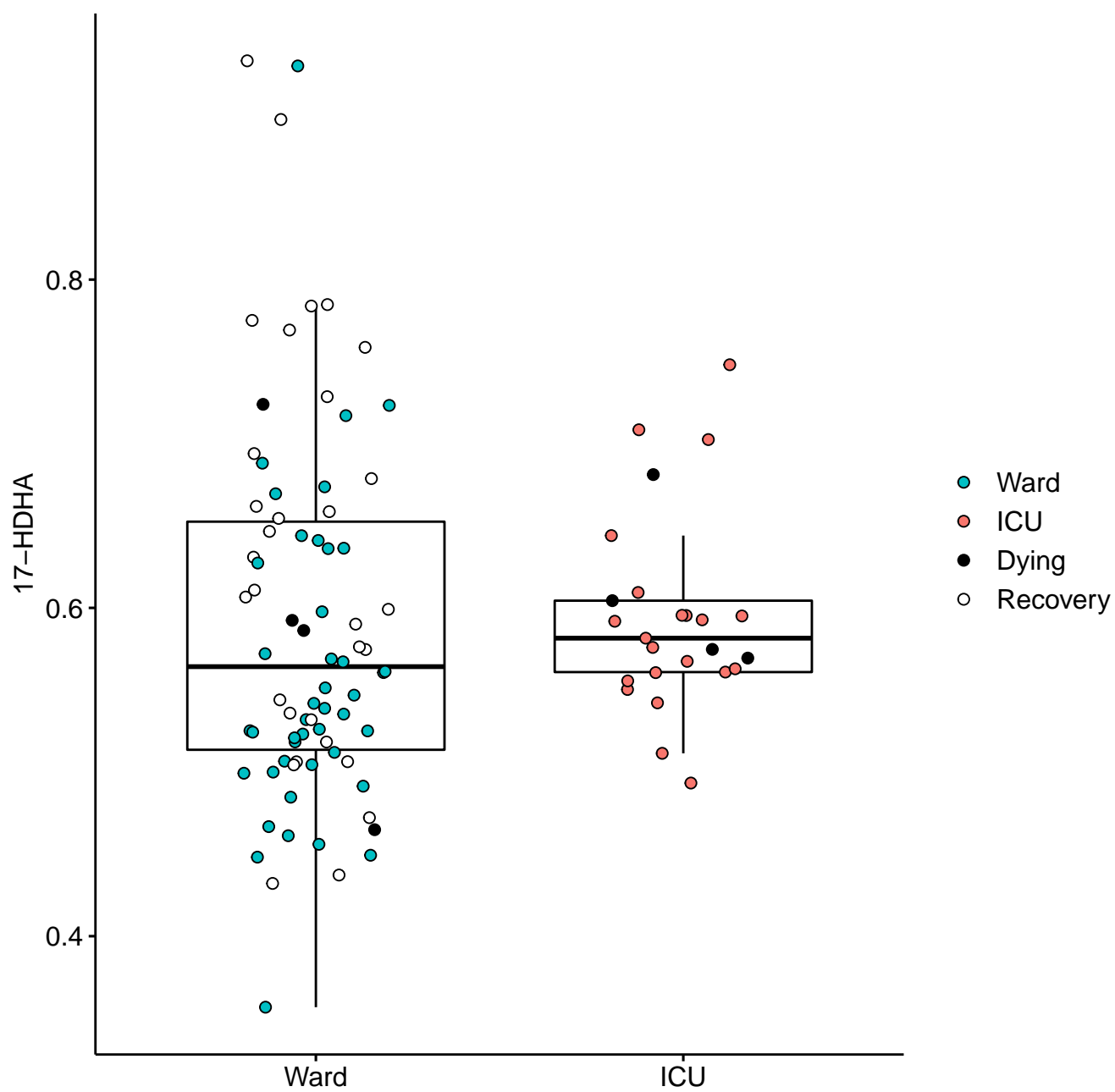

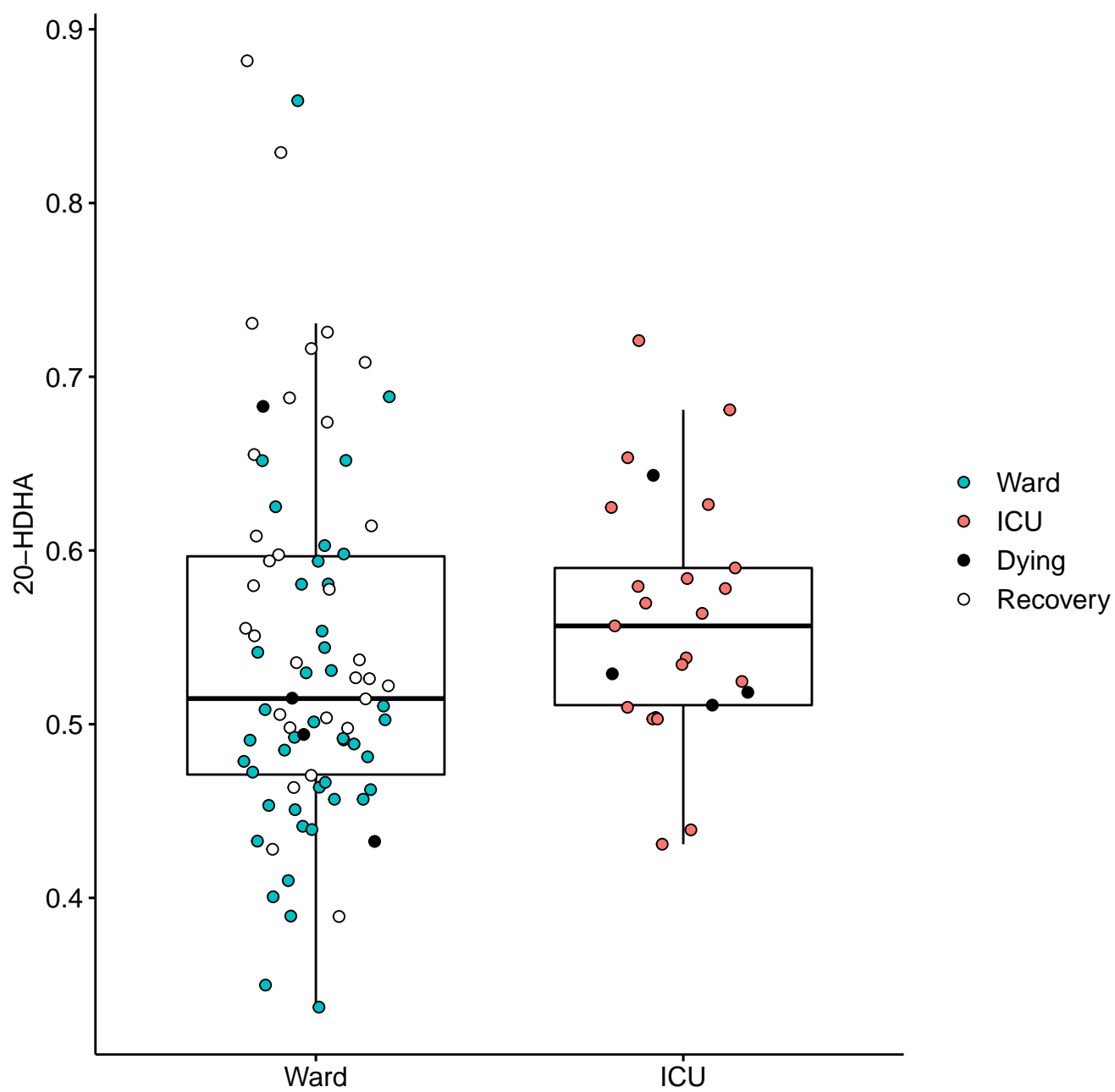

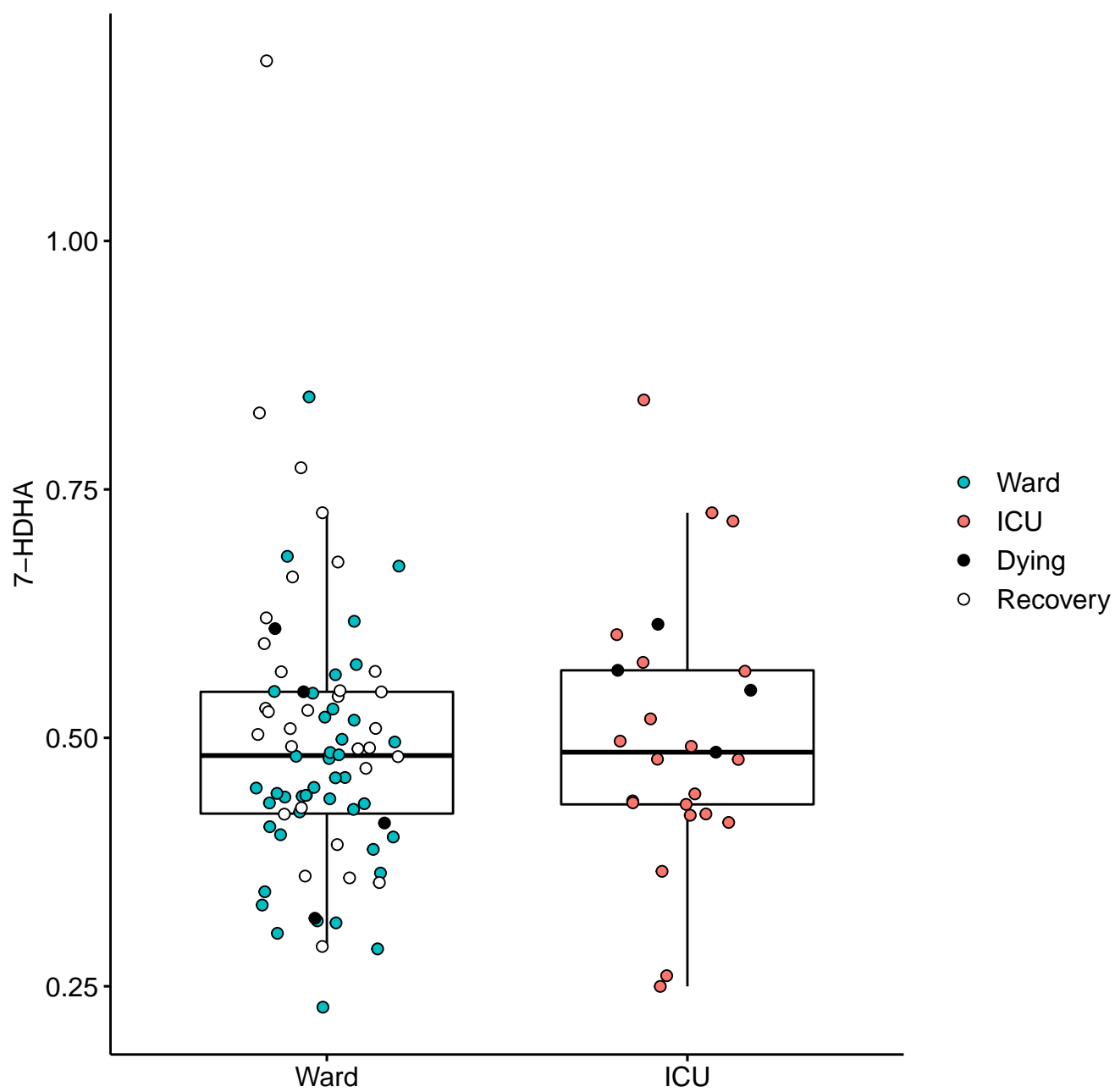

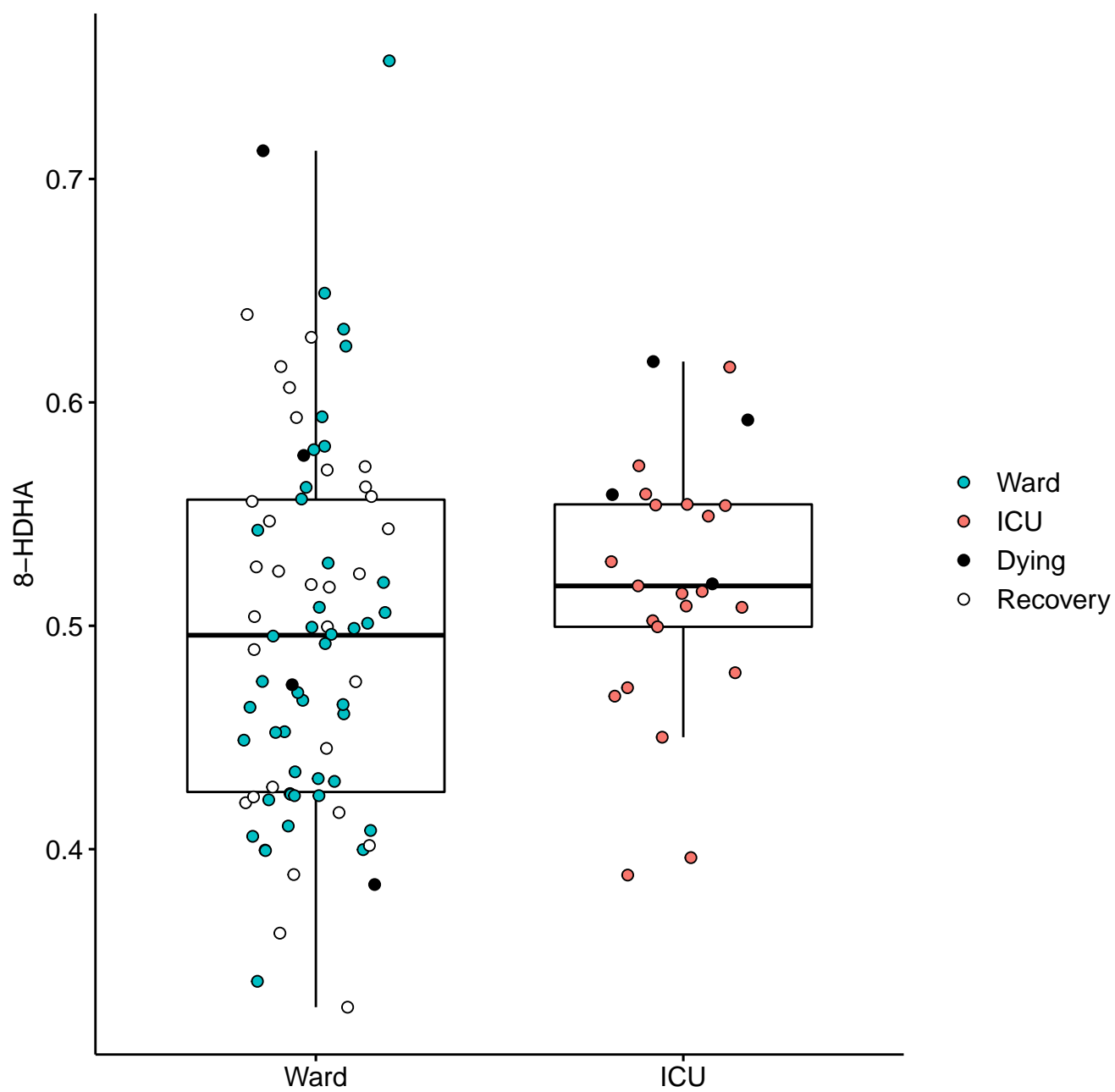

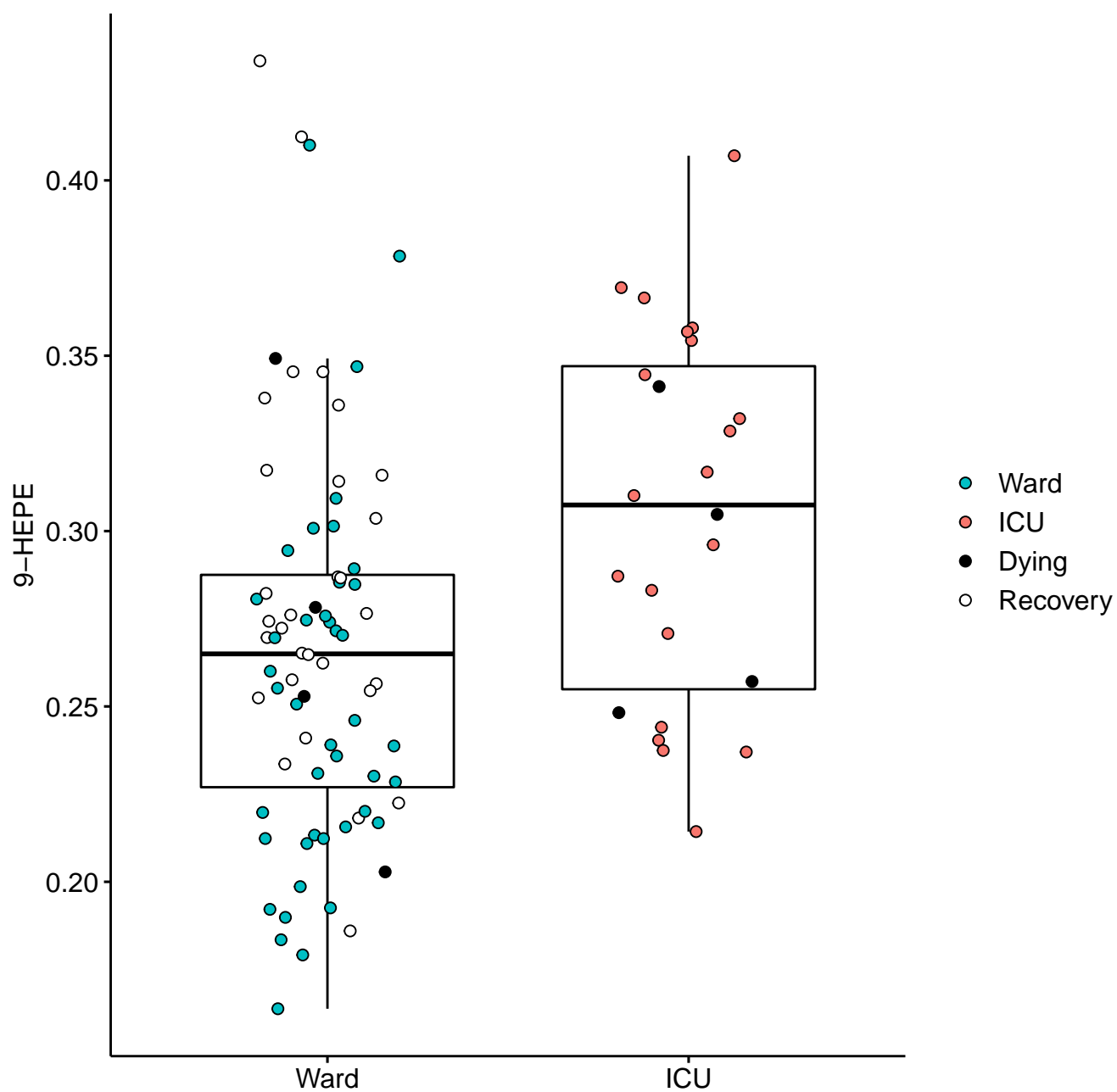



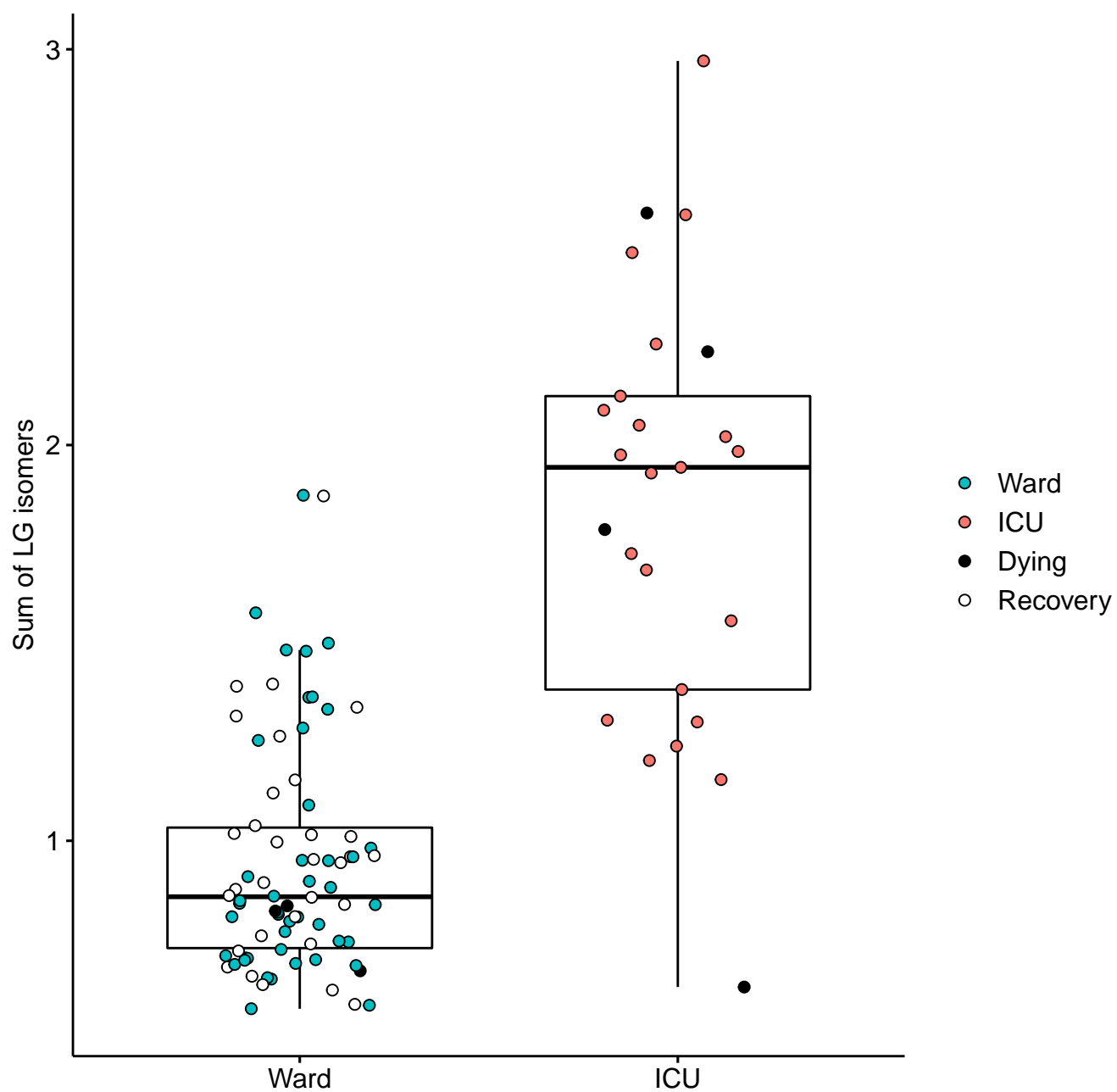

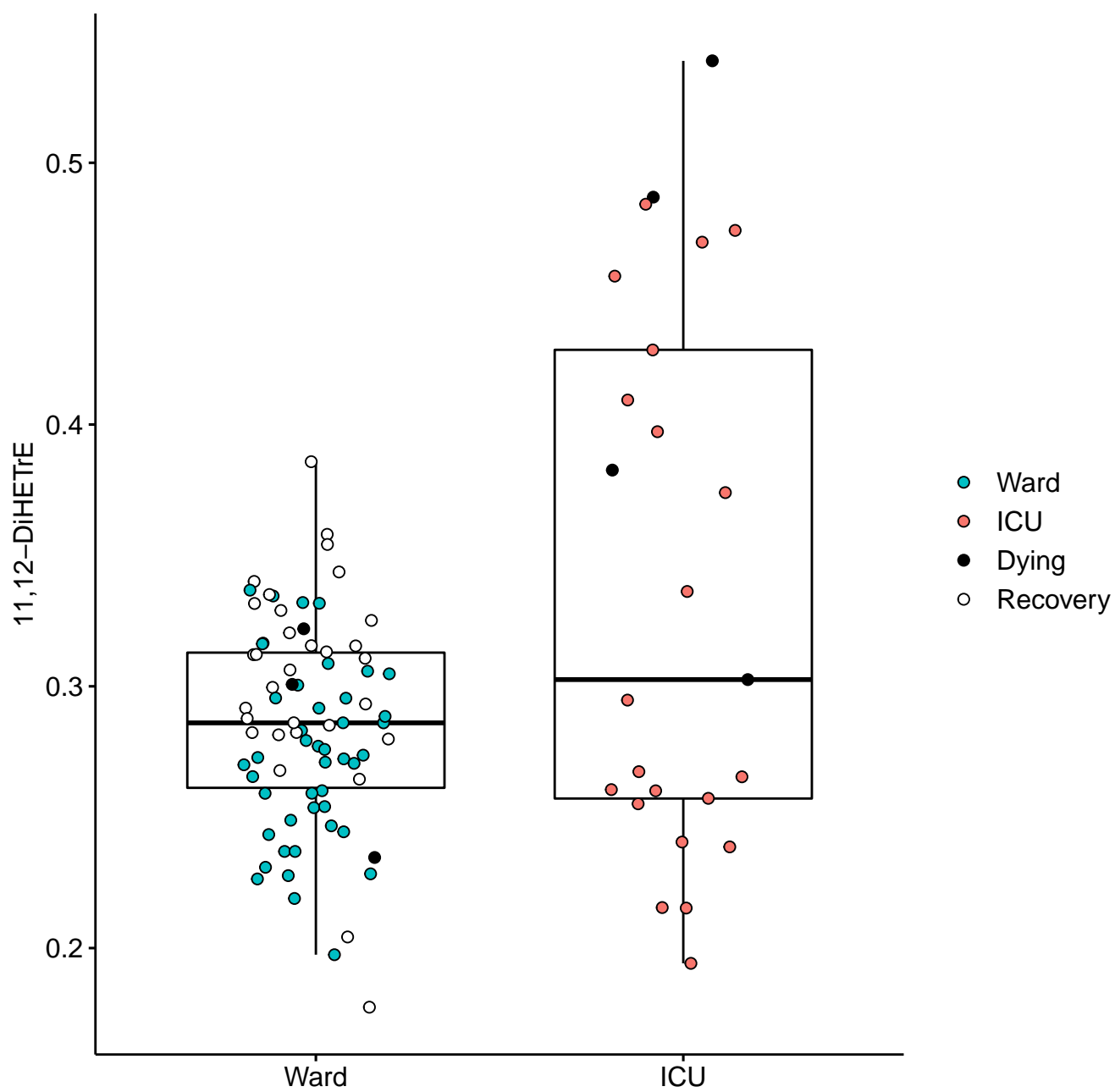

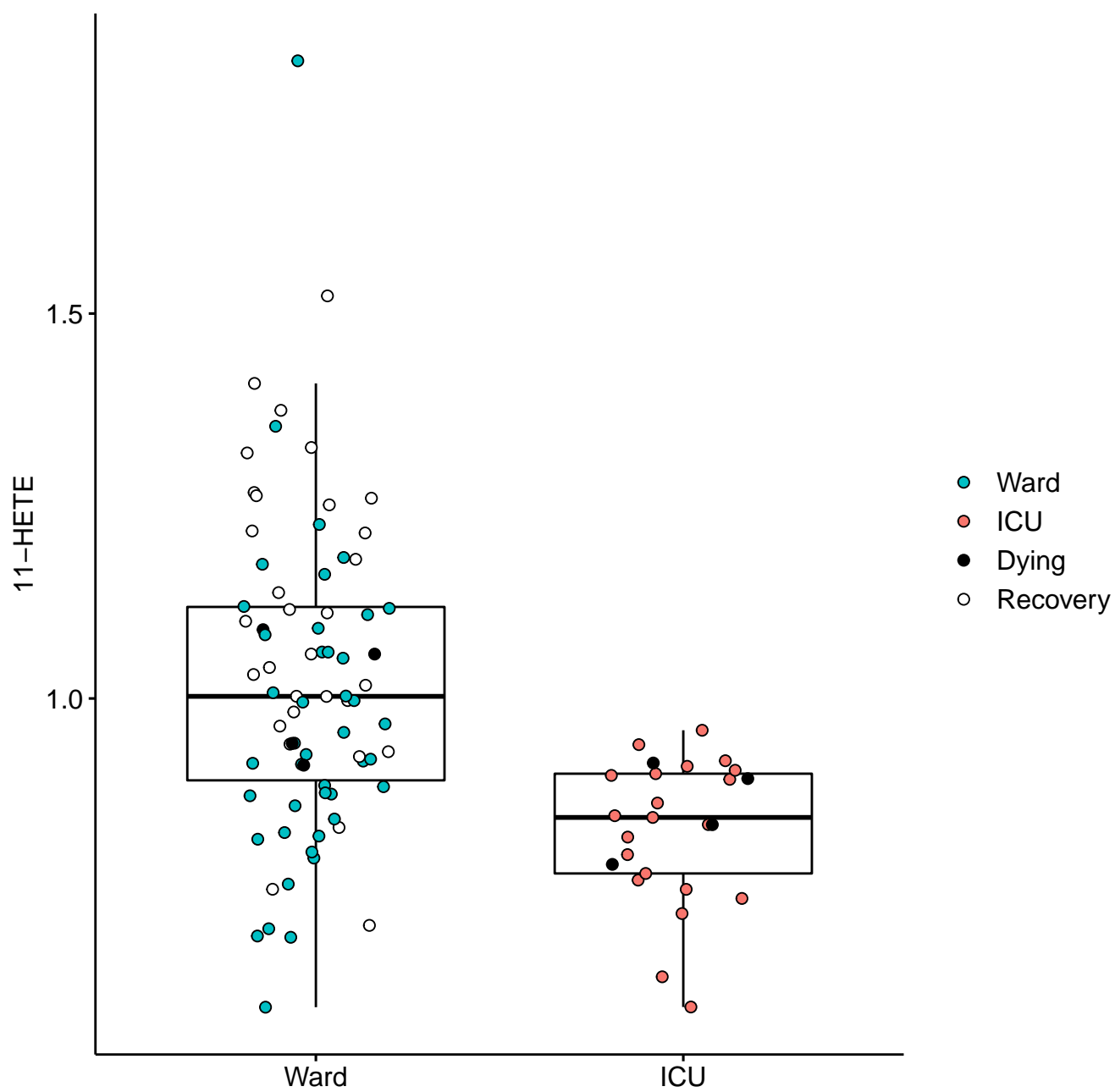

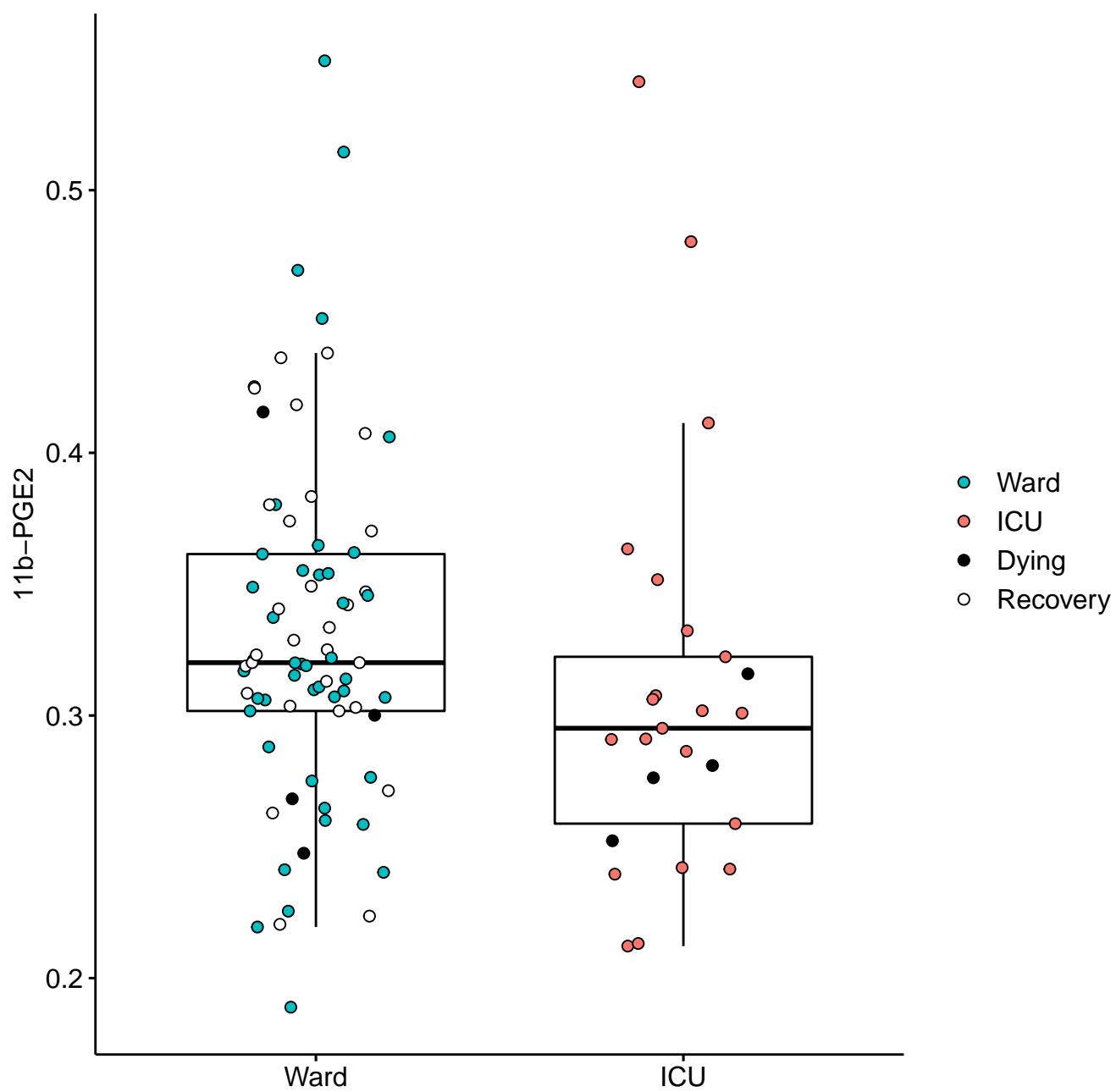

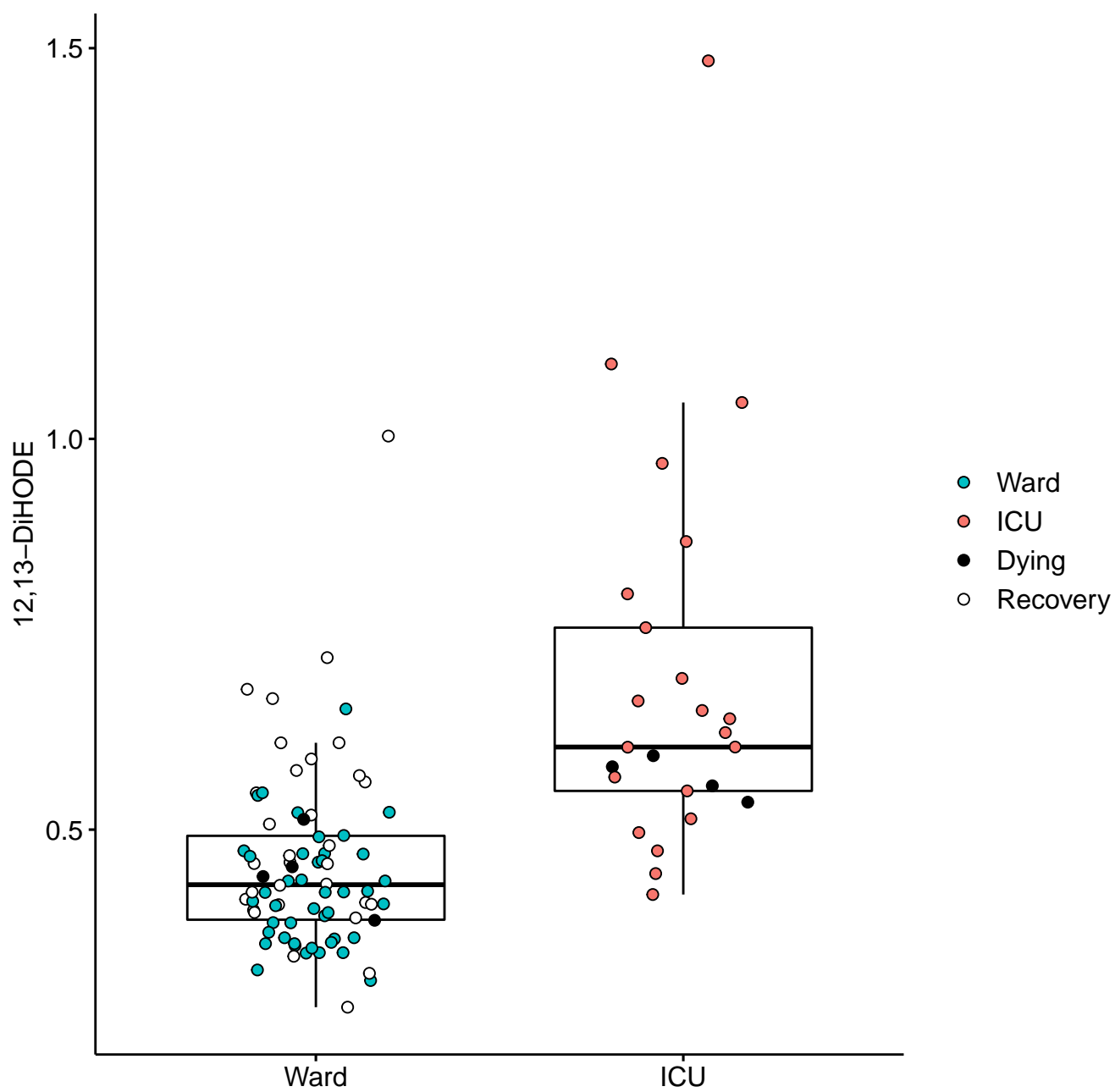

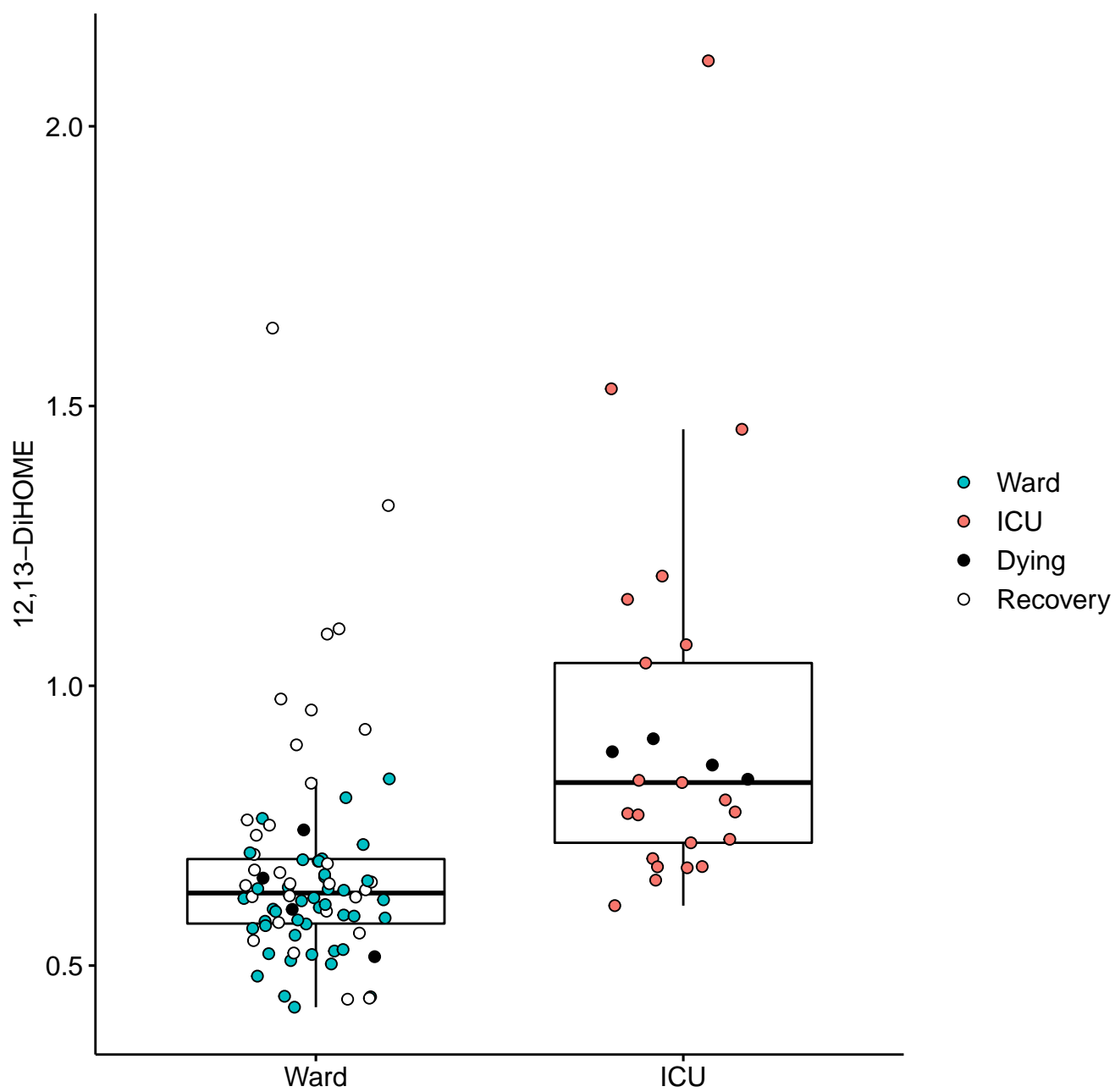

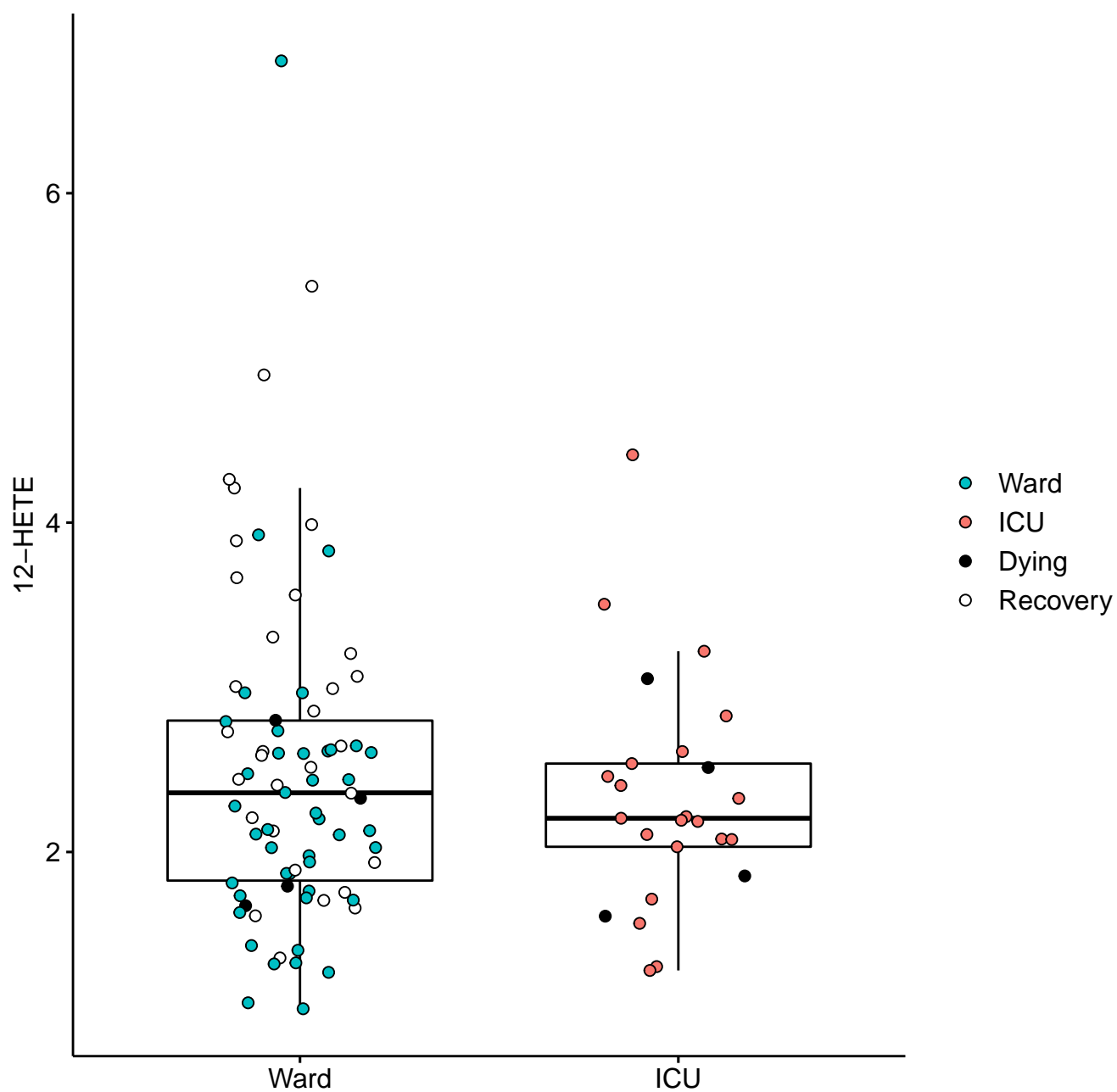

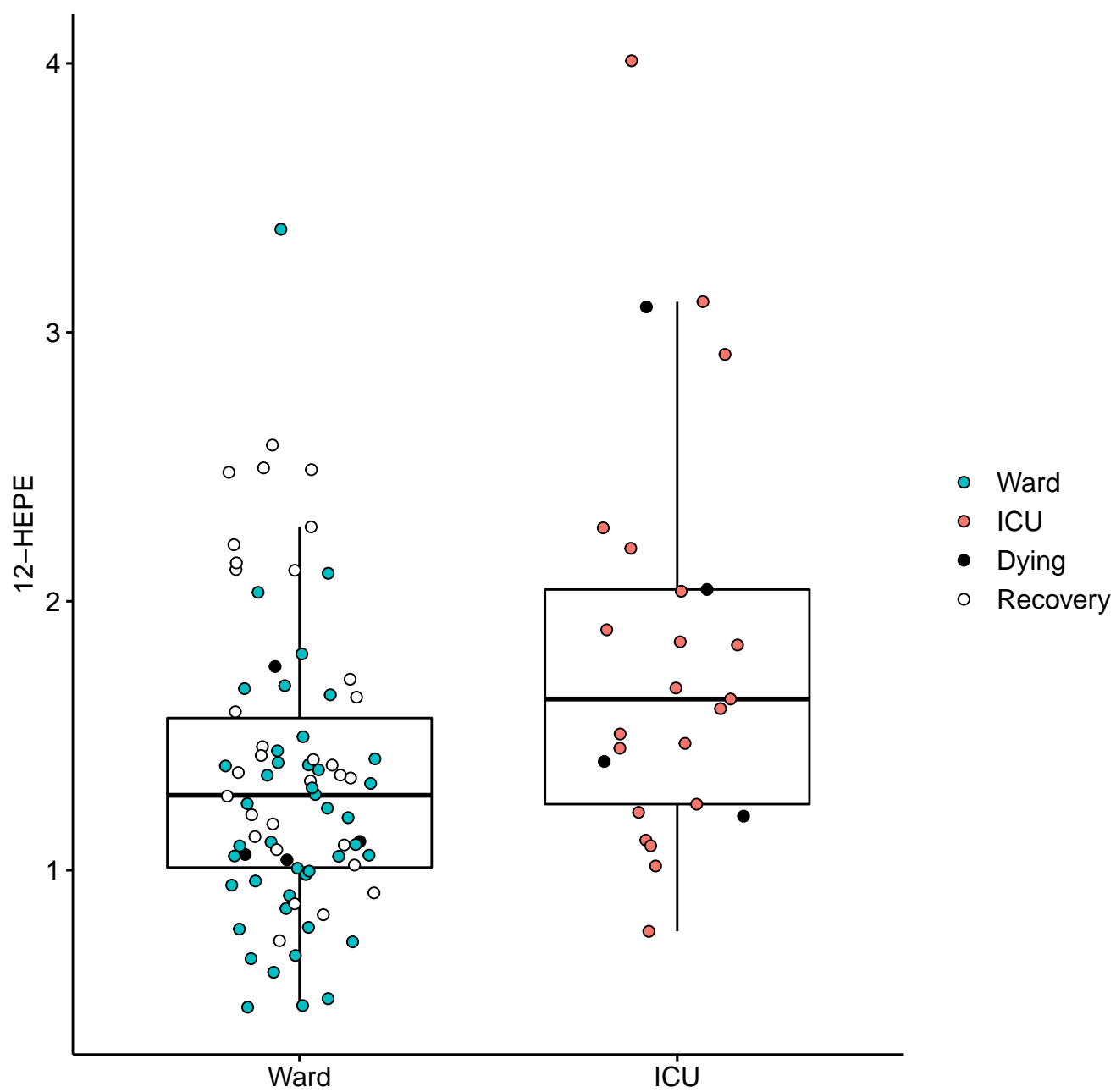

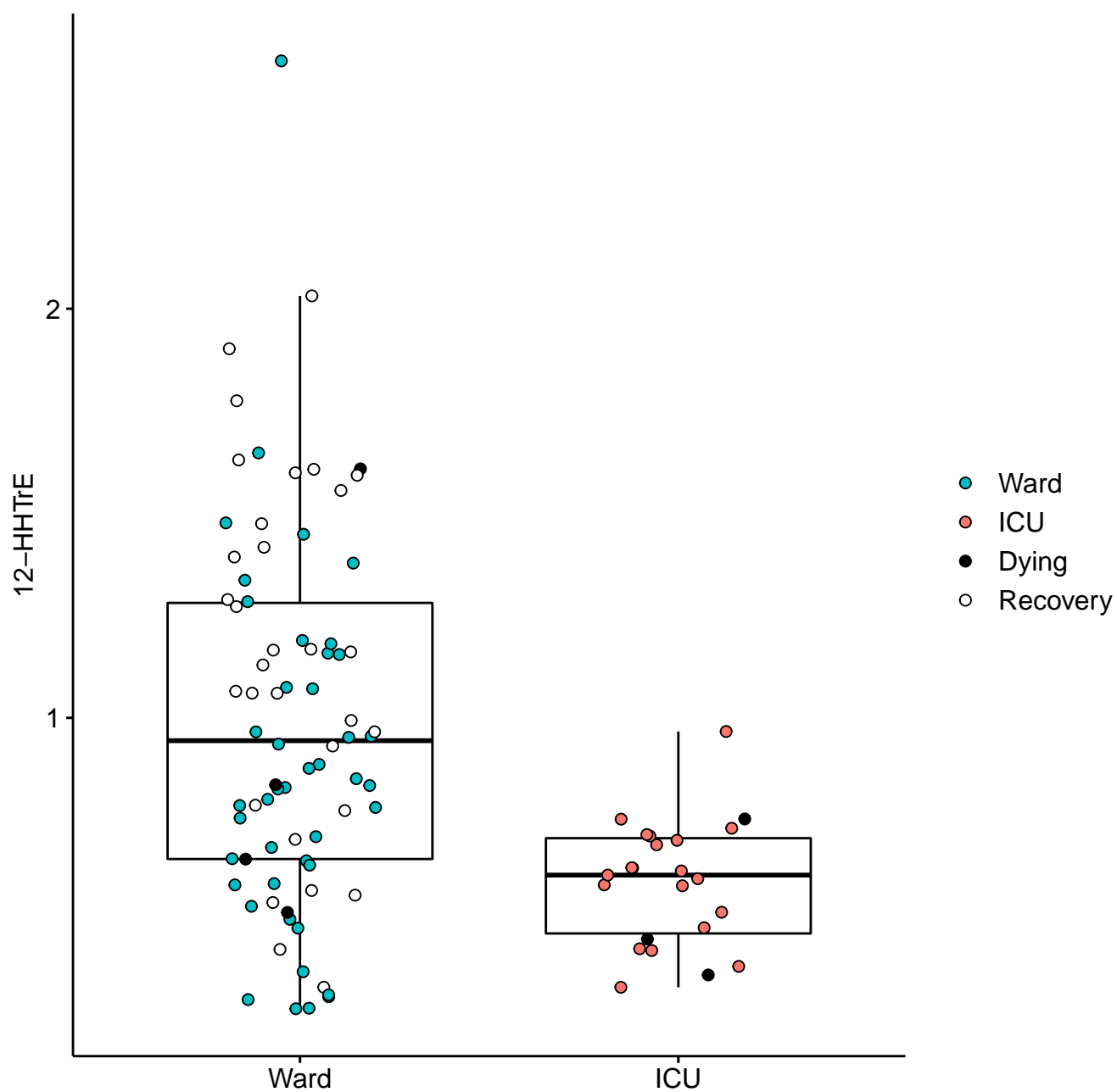

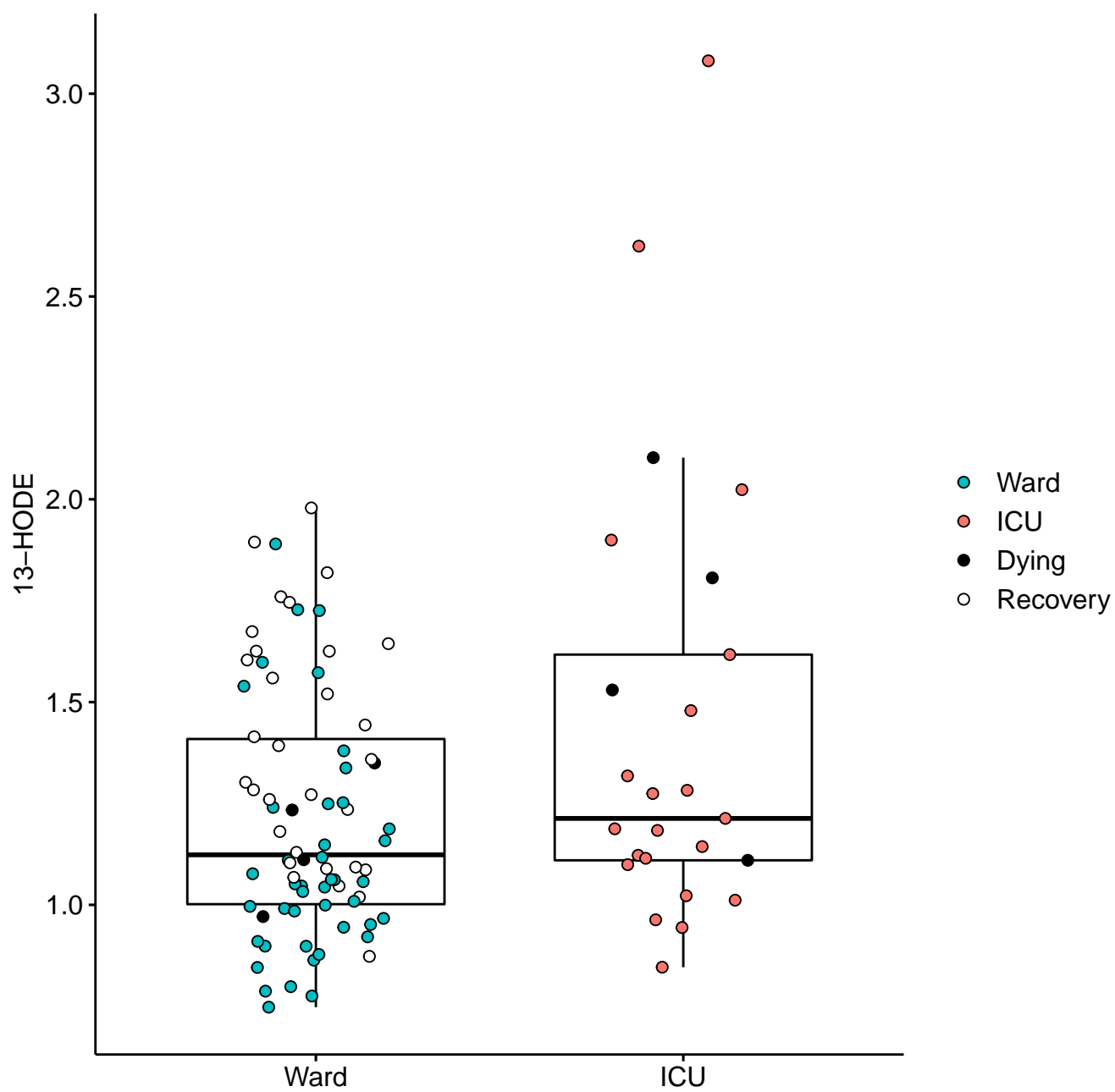

14,15-DiHETE

0.25  
0.20  
0.15  
0.10

Ward

ICU

- Ward
- ICU
- Dying
- Recovery

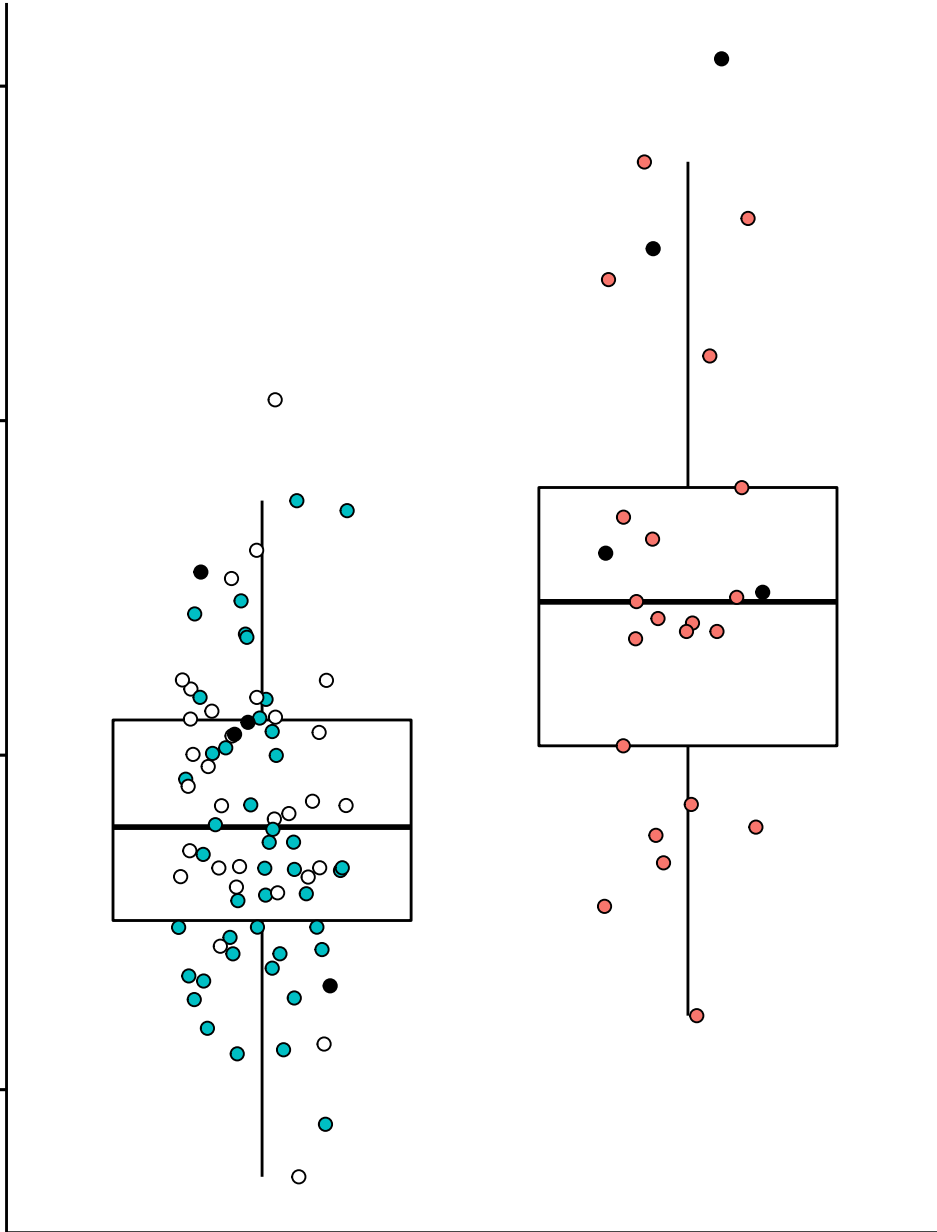

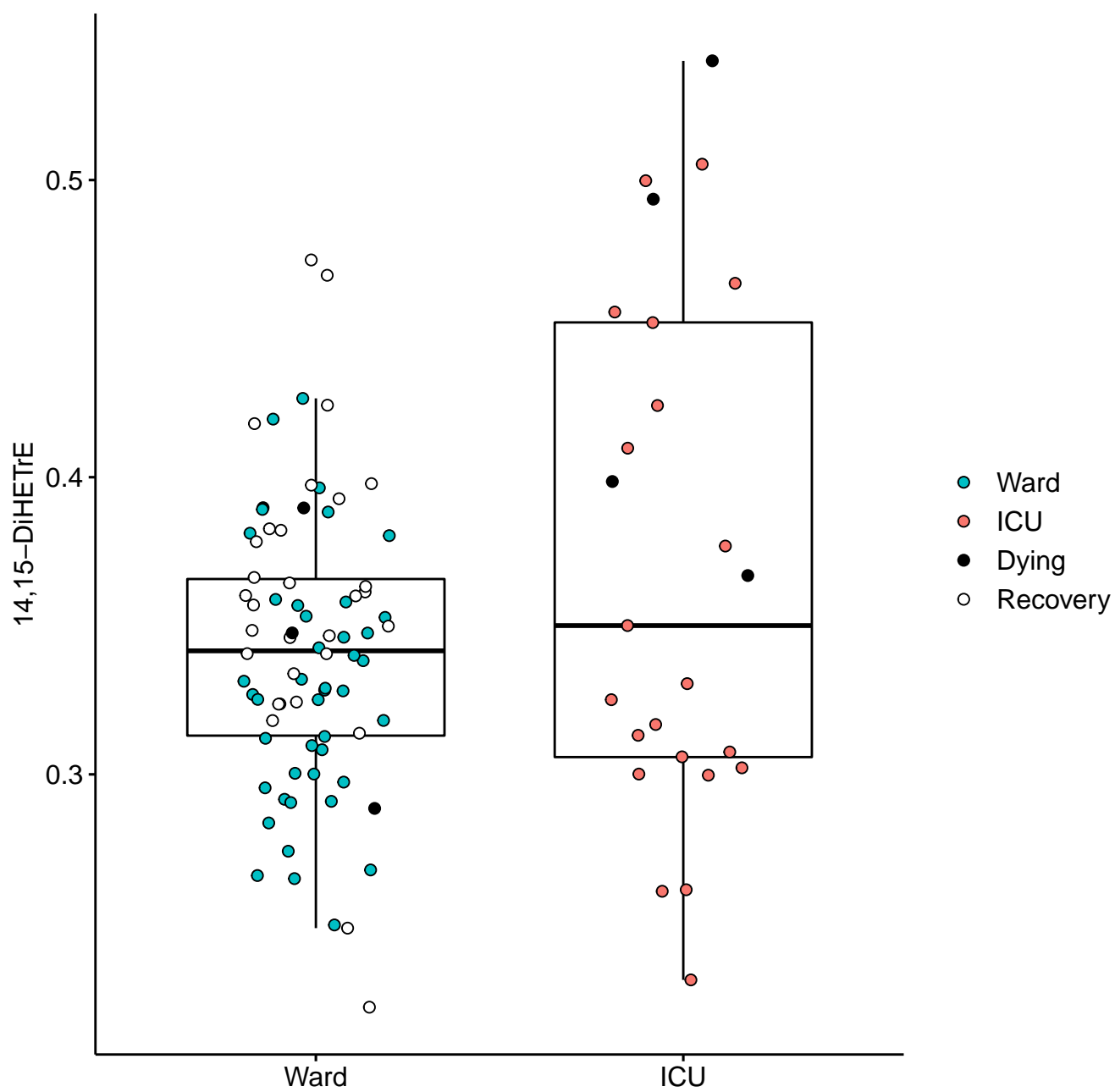

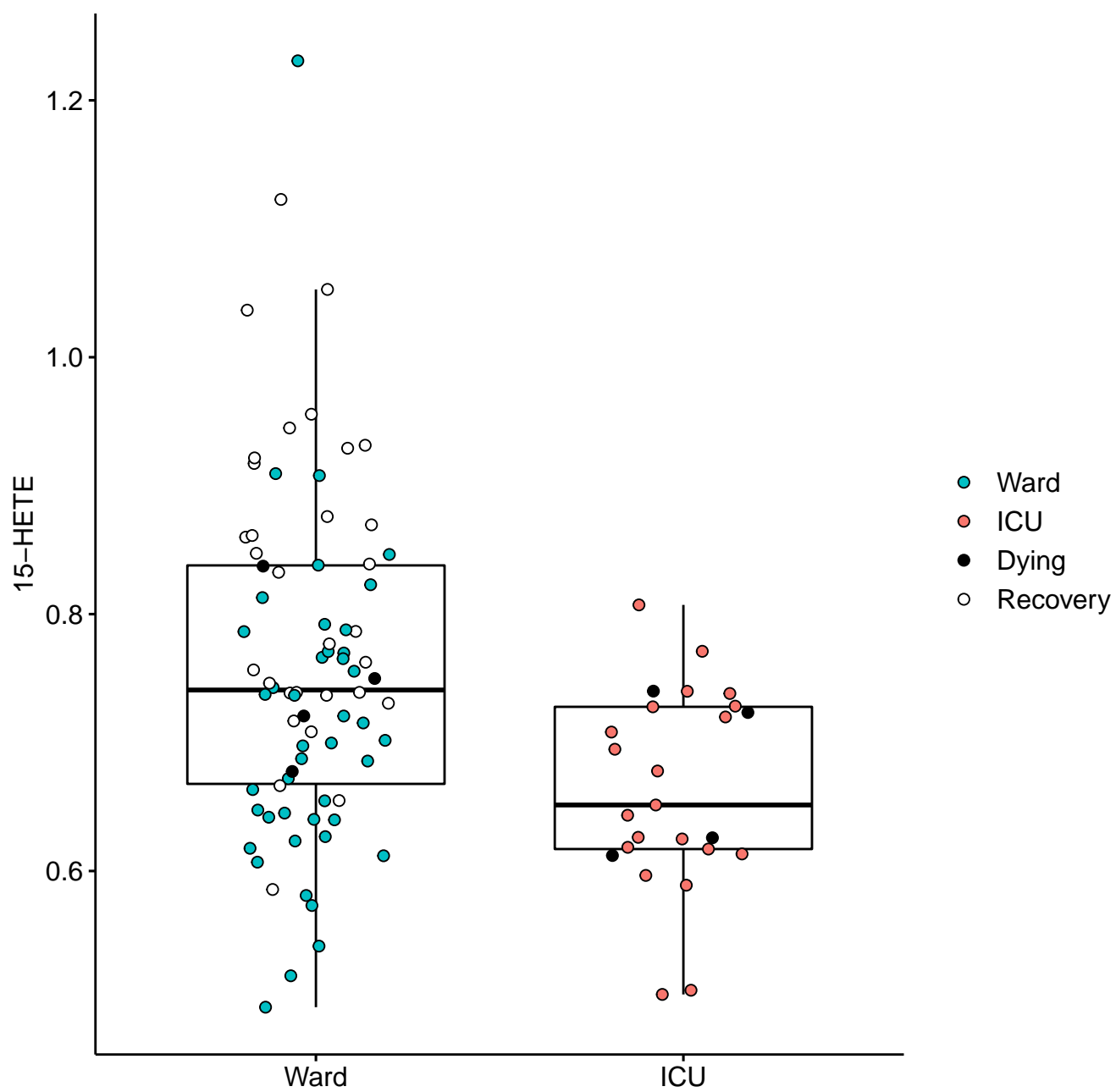

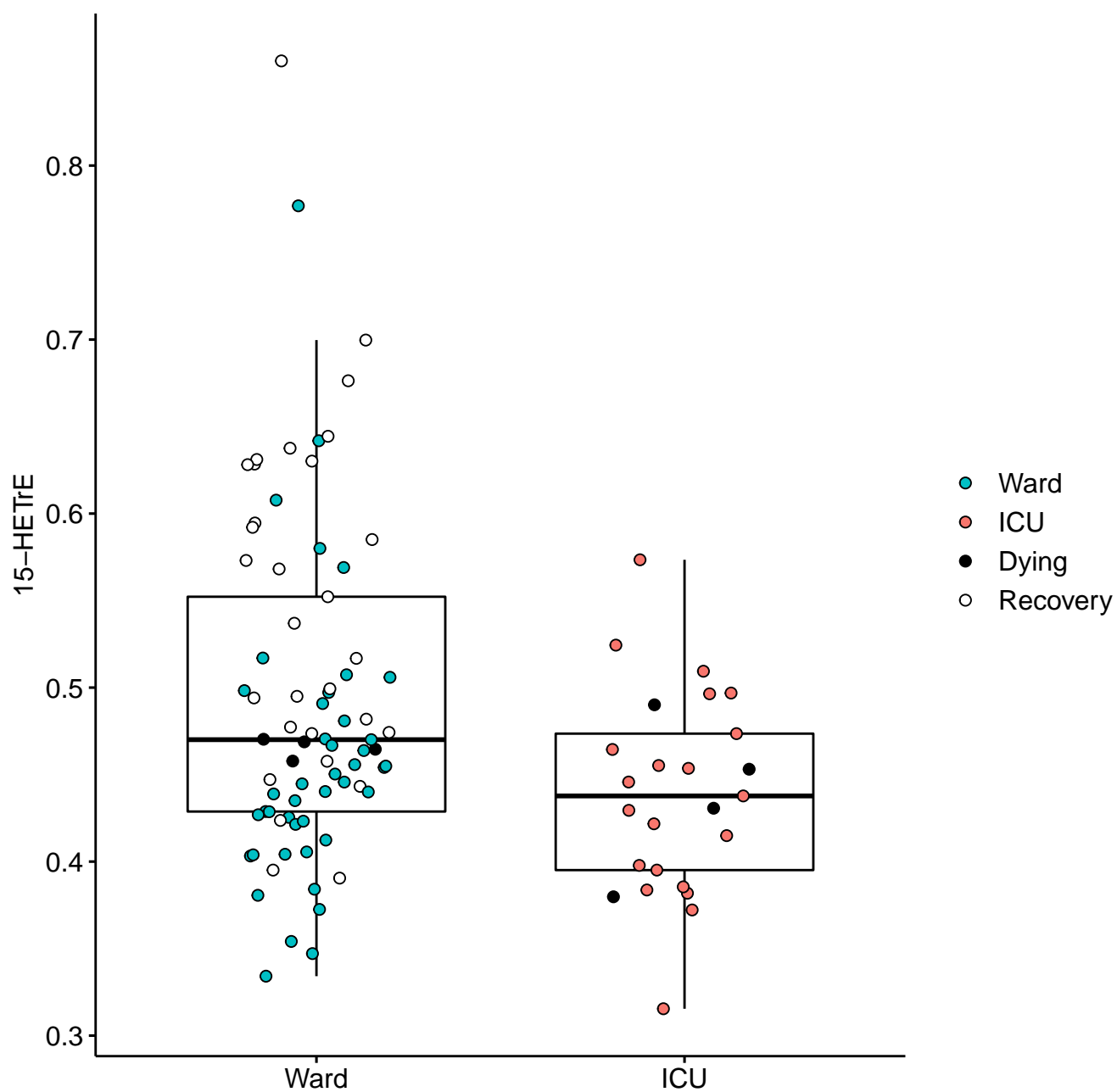

17,18-DiHETE

0.4

0.3

0.2

Ward

ICU

- Ward
- ICU
- Dying
- Recovery

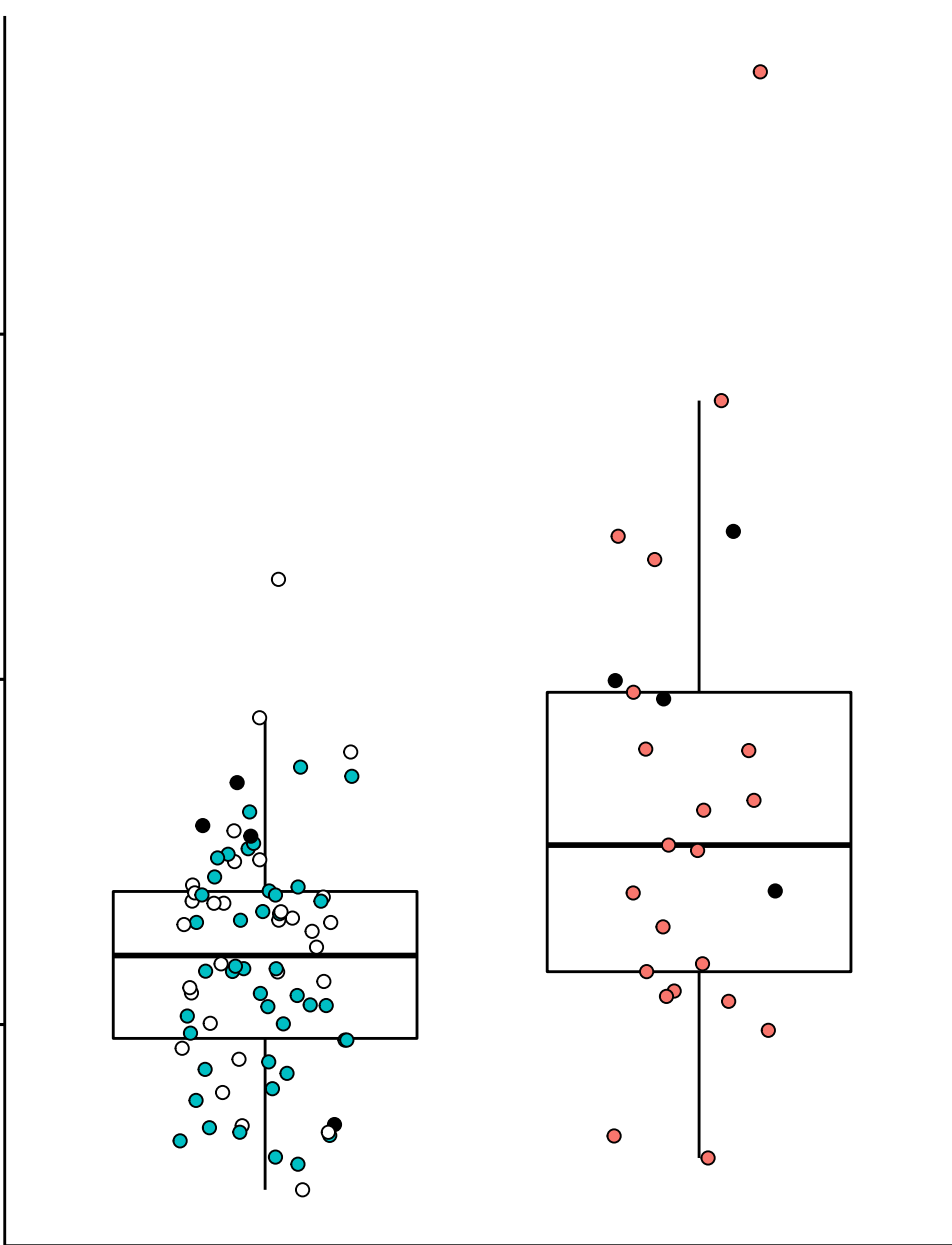

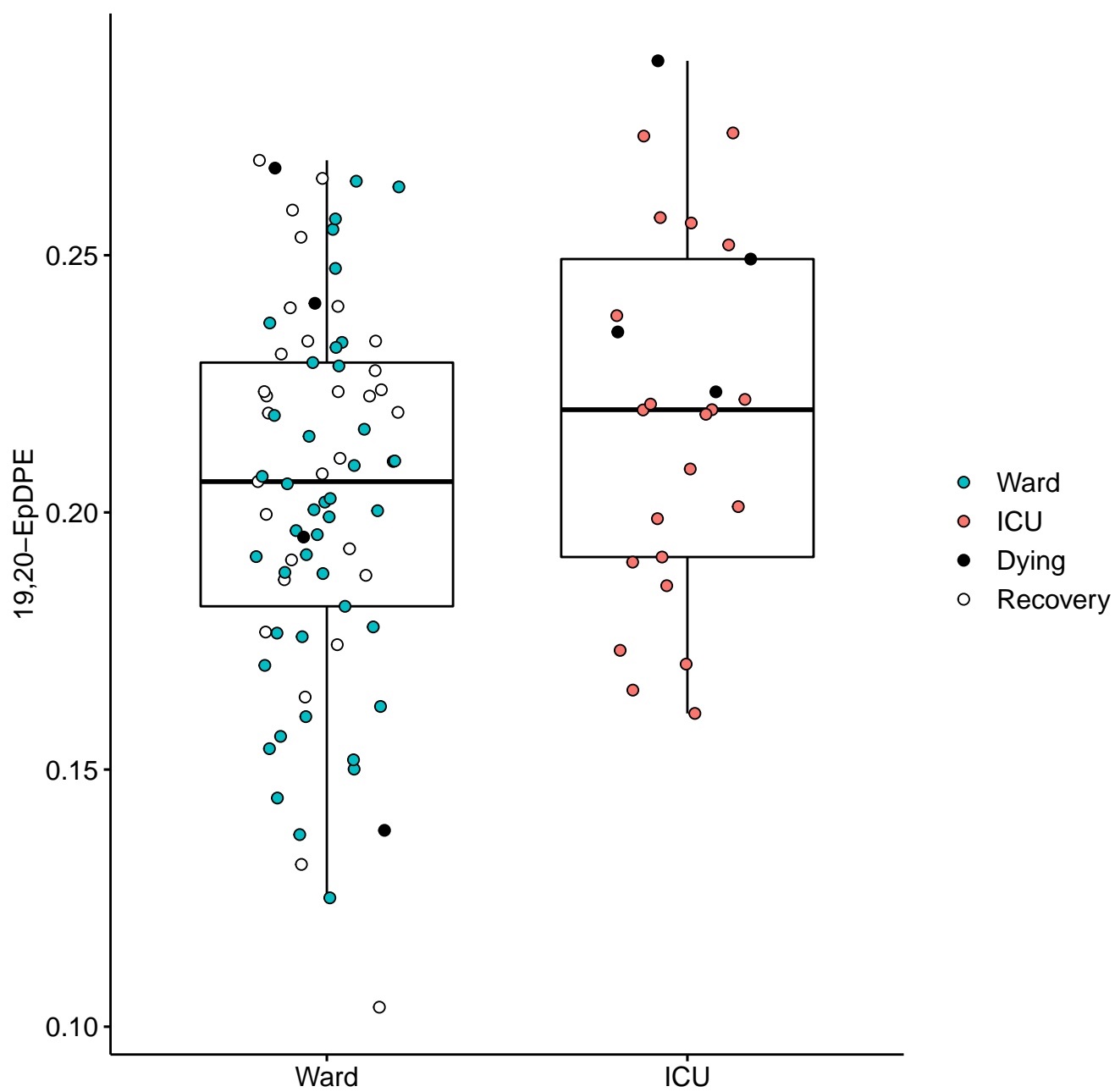

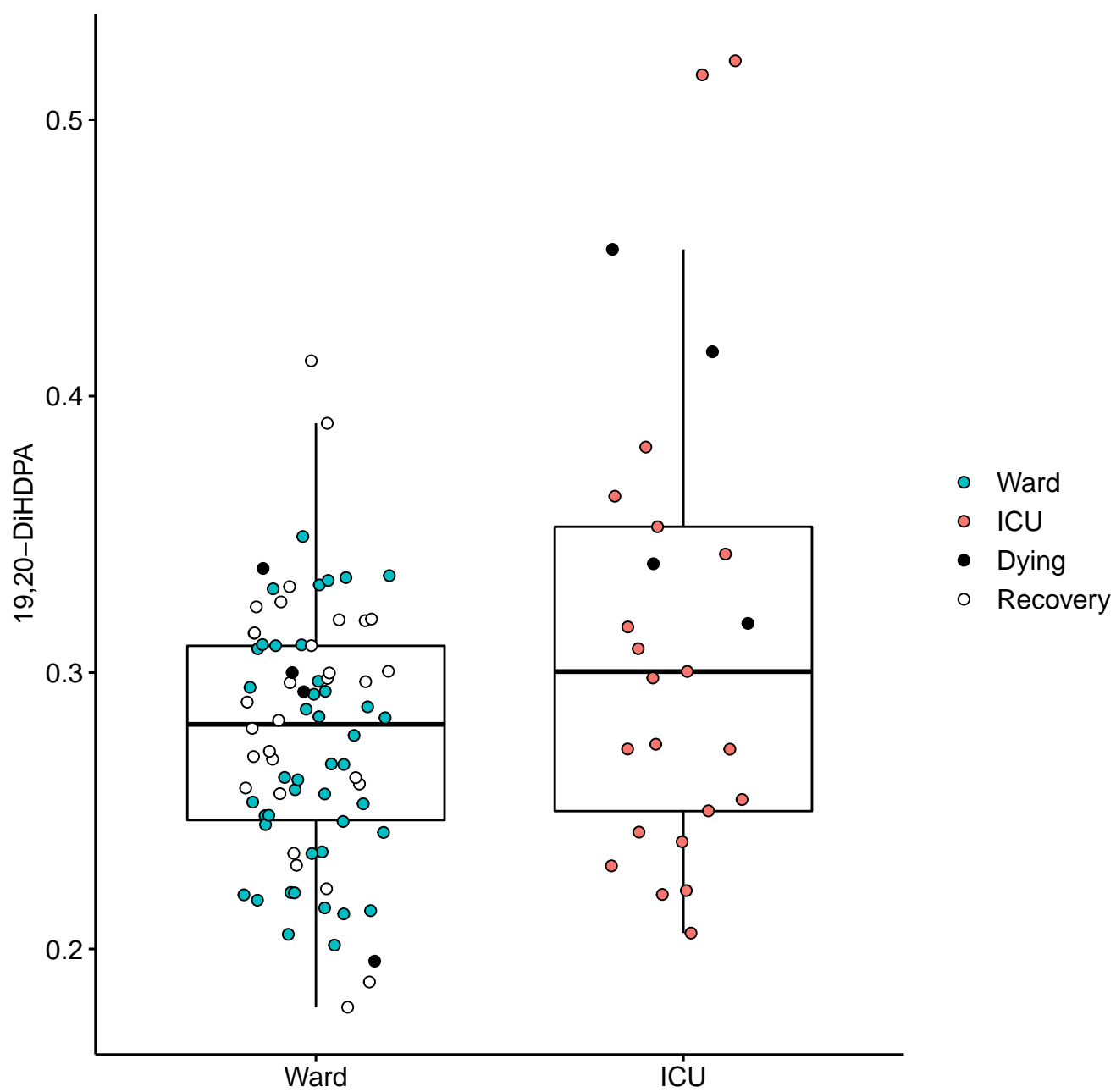

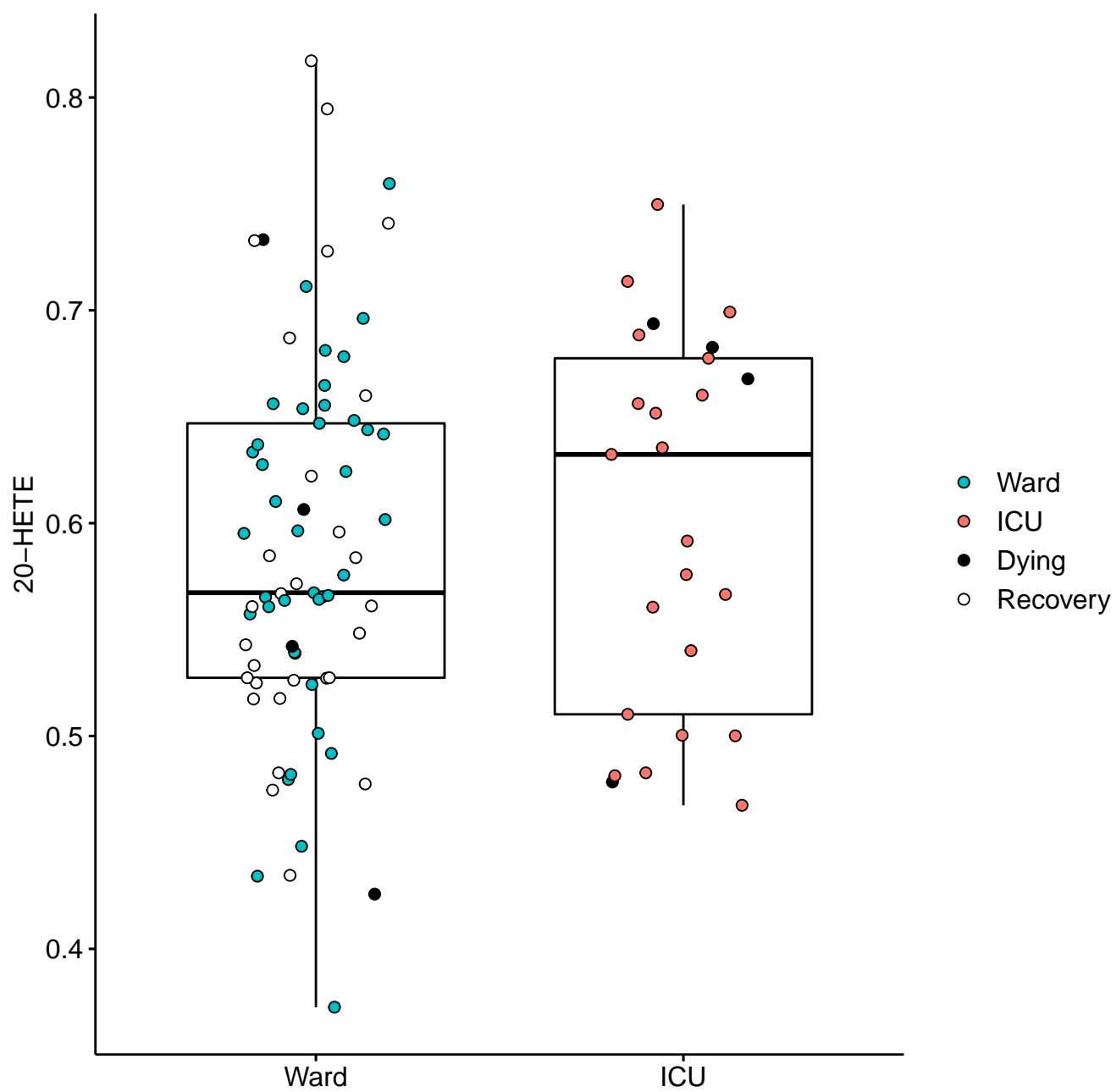

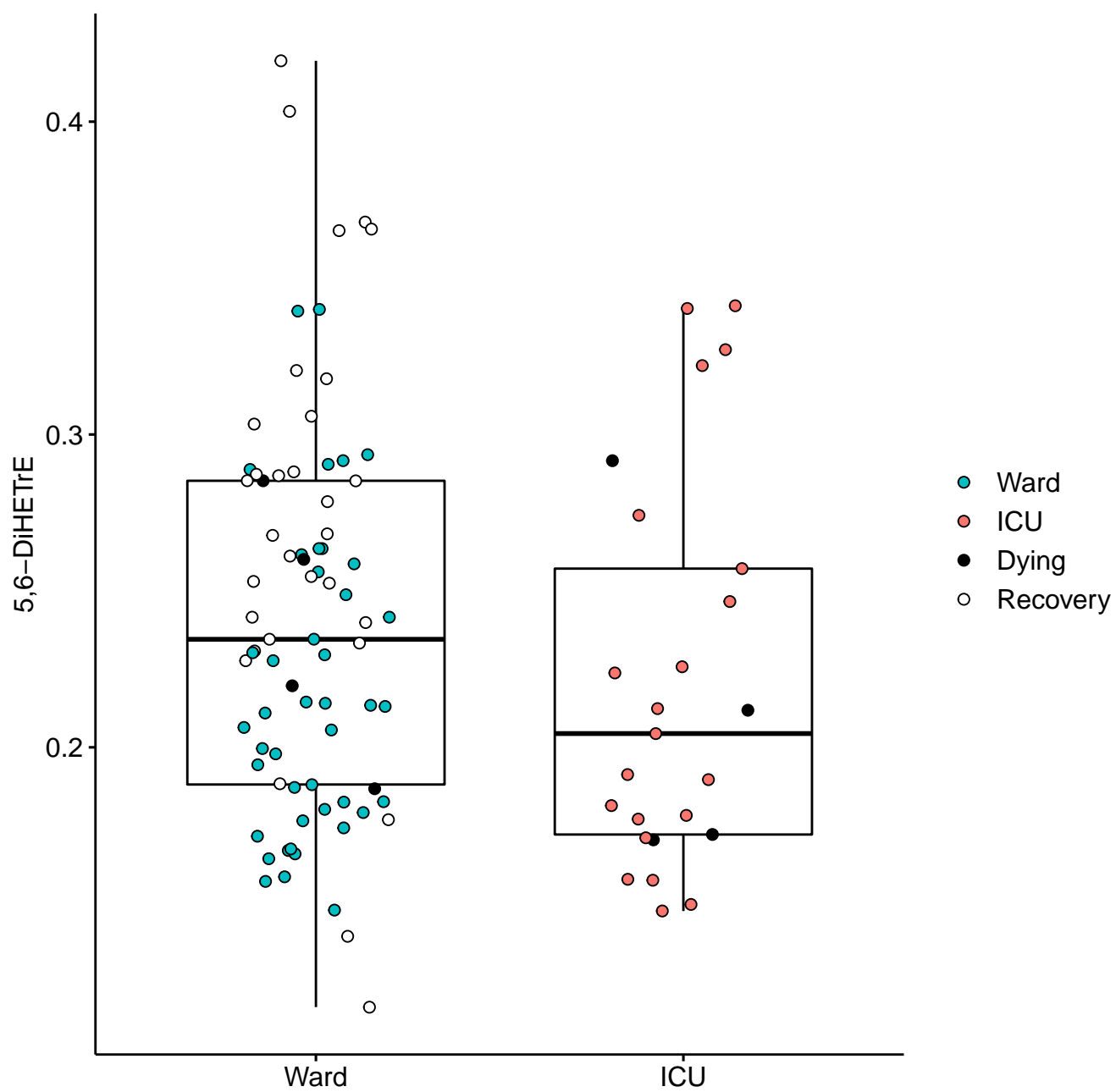

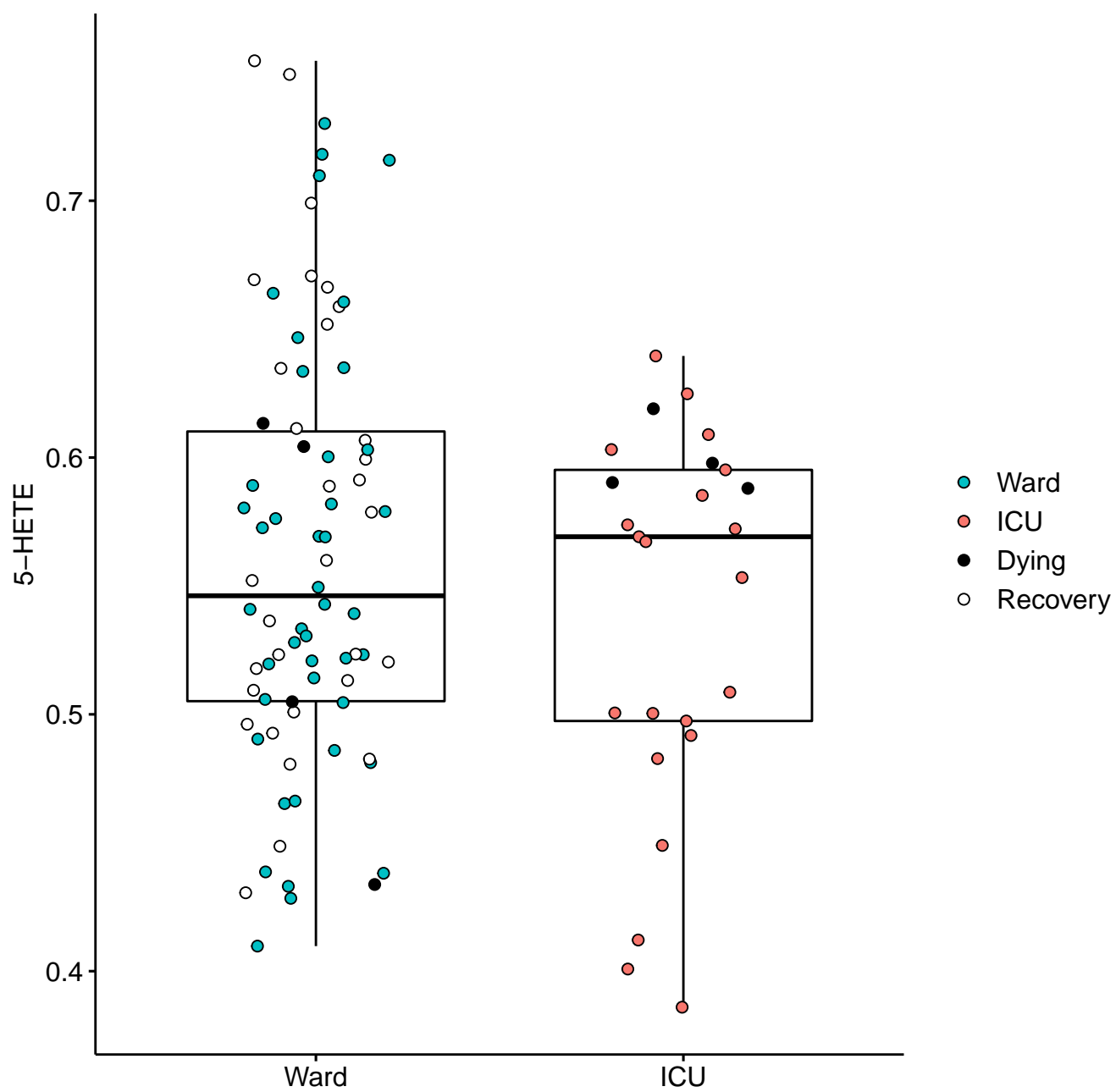

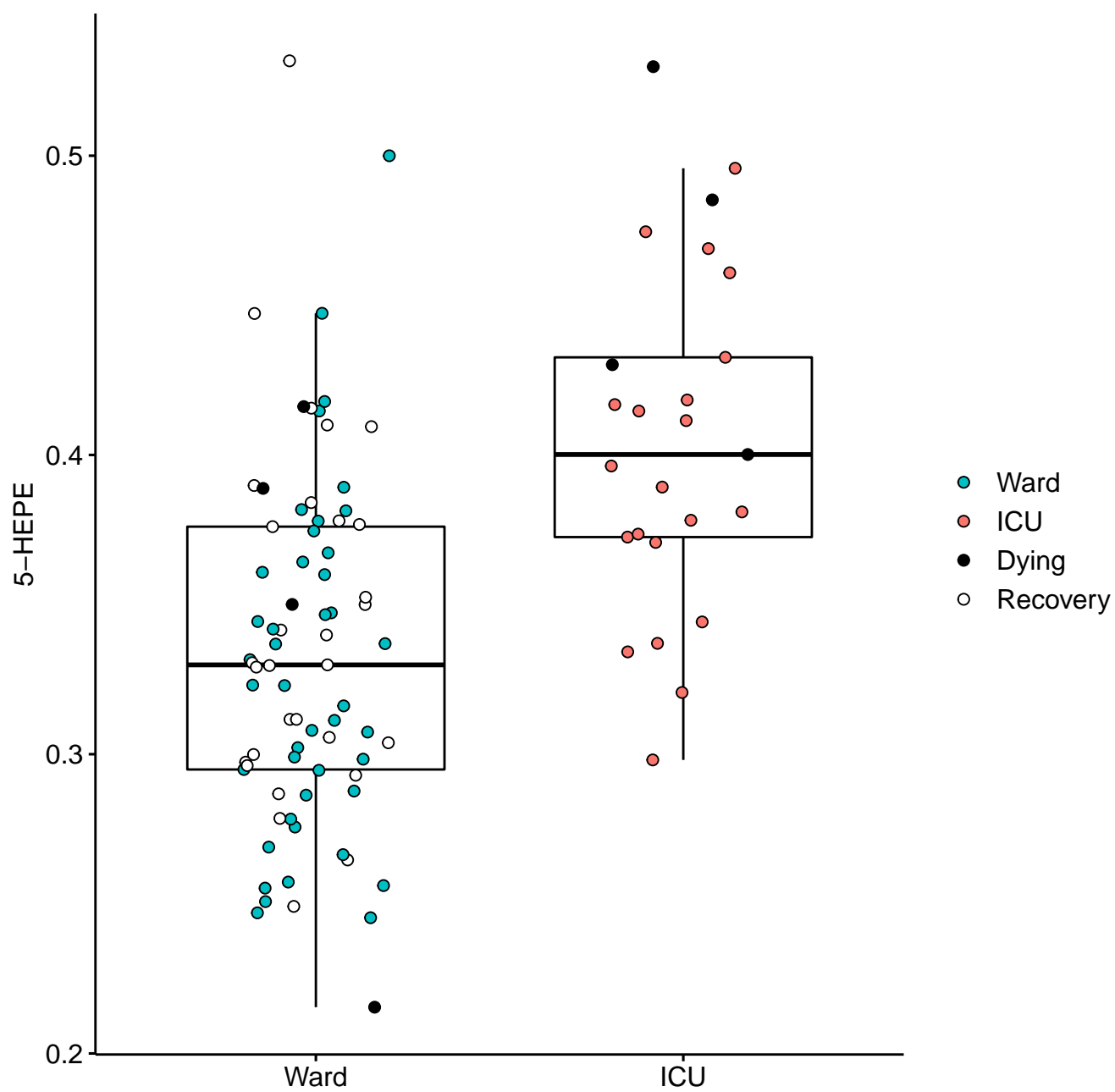

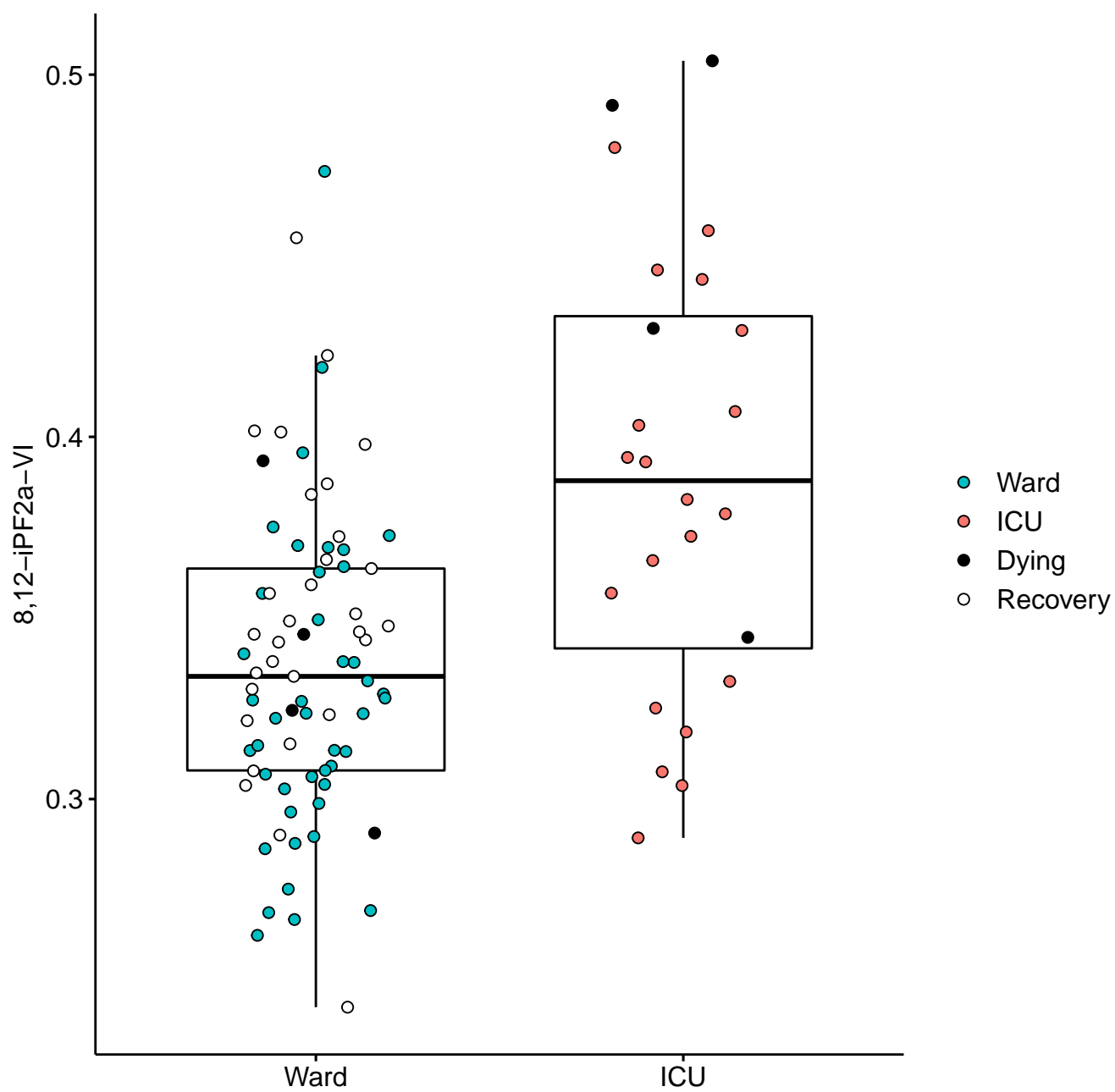

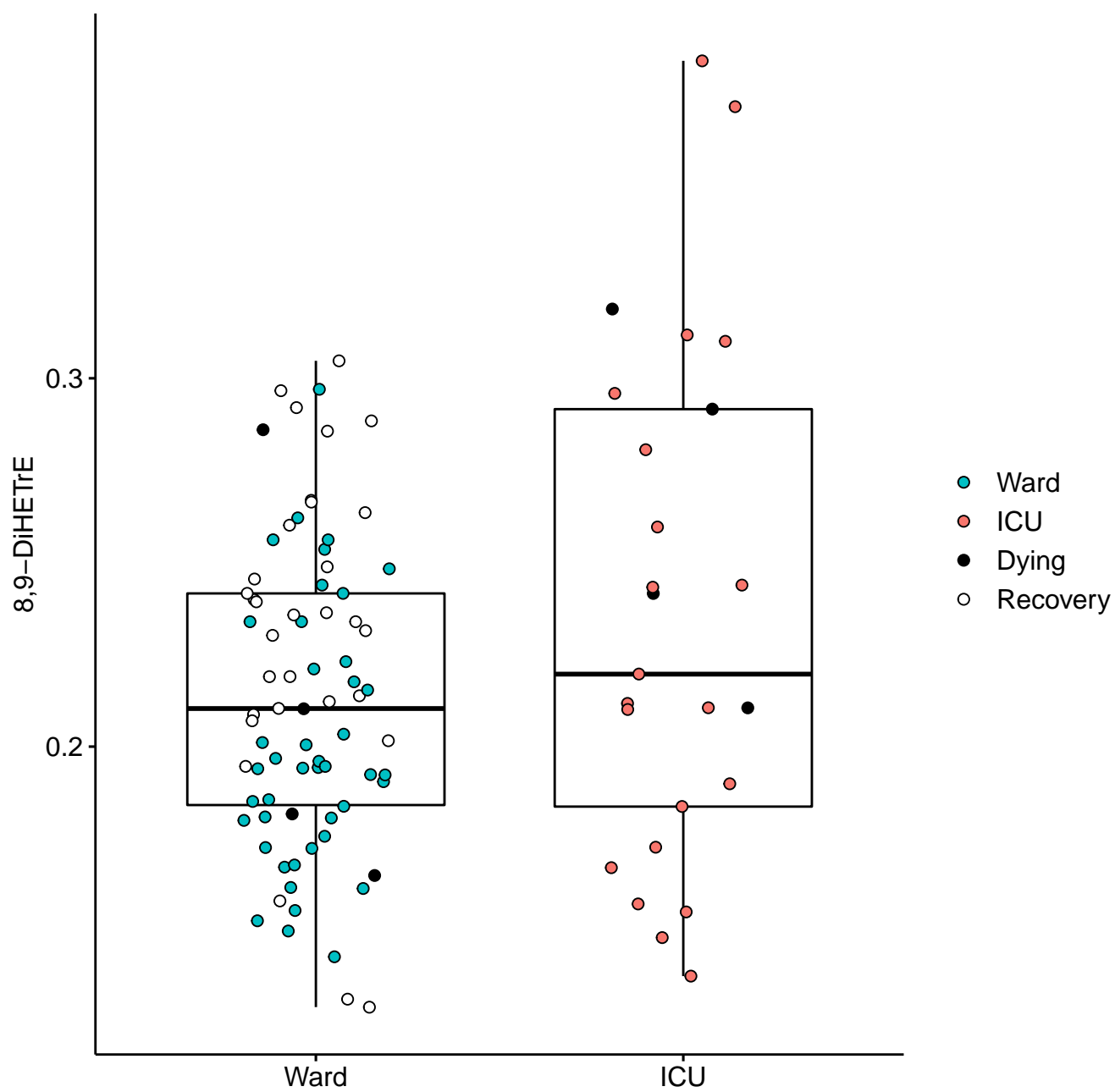

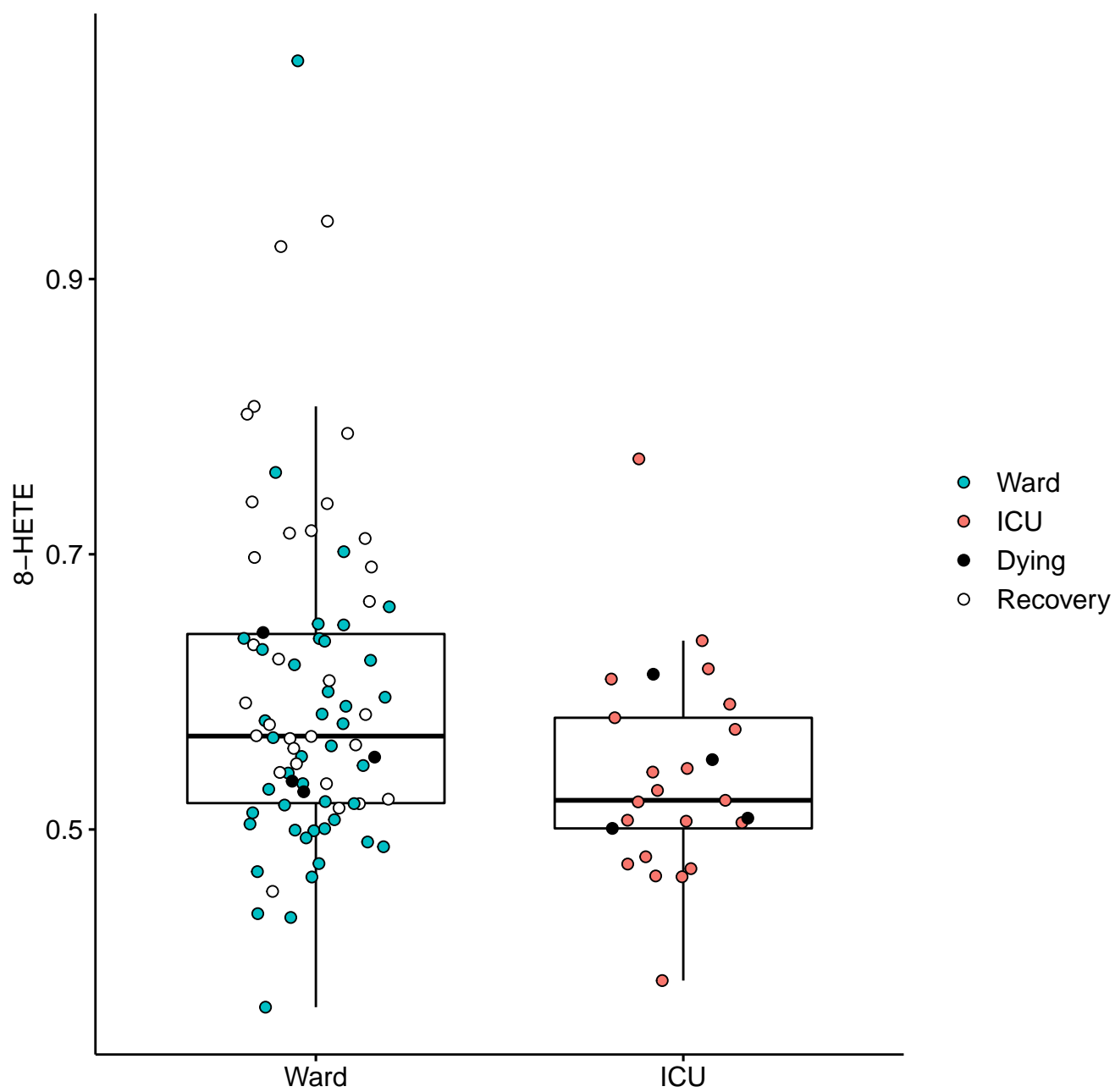

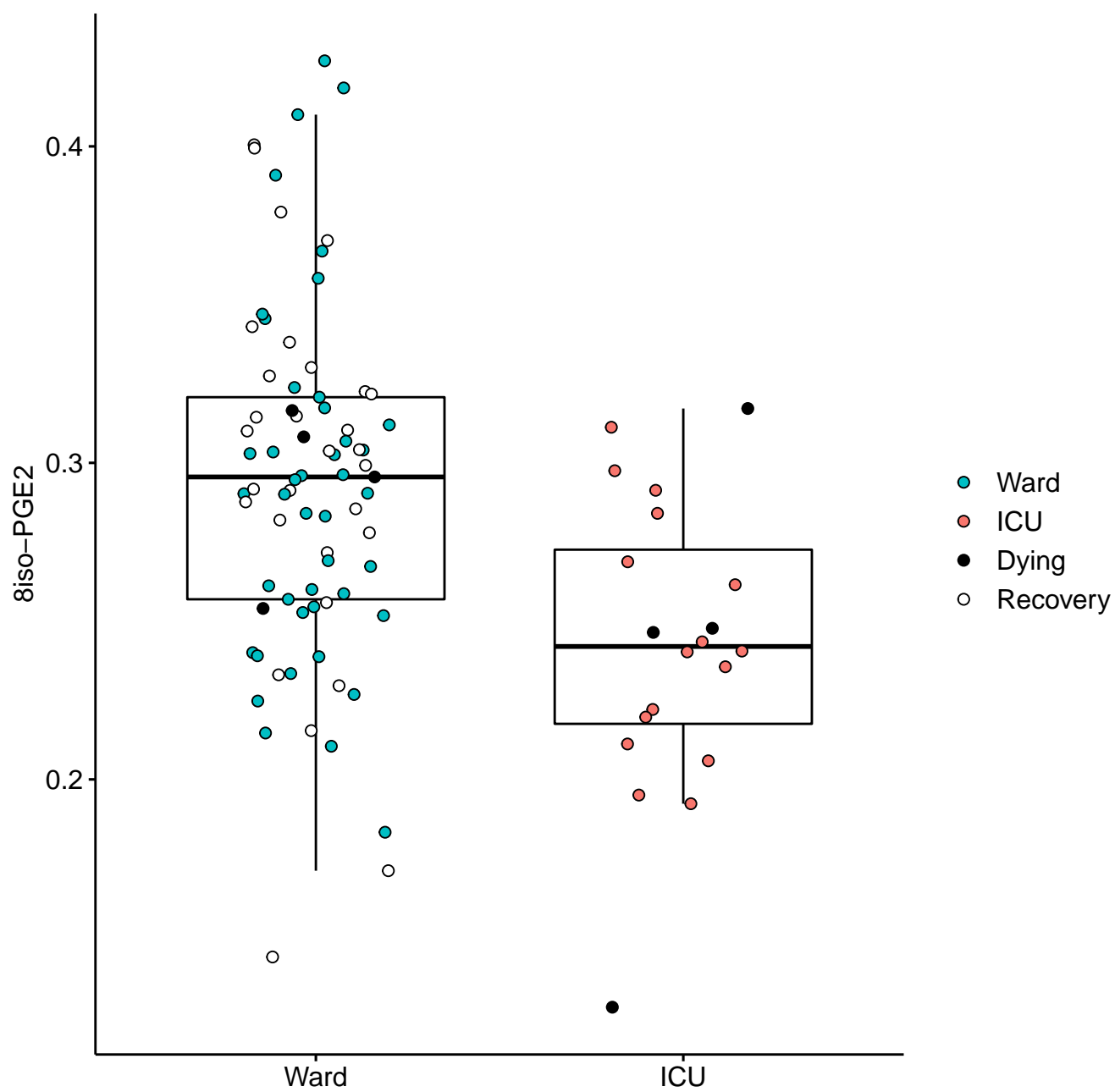

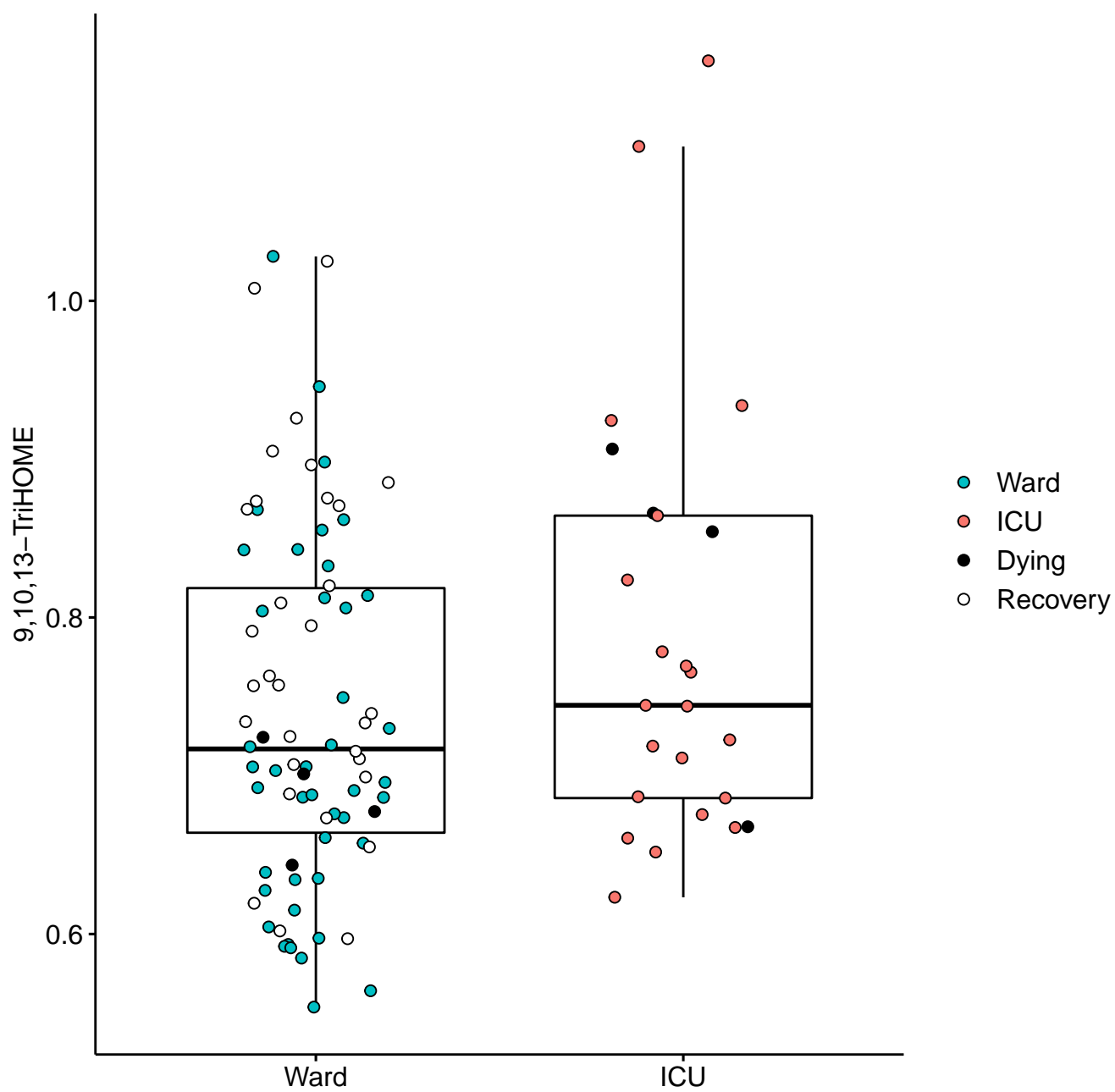

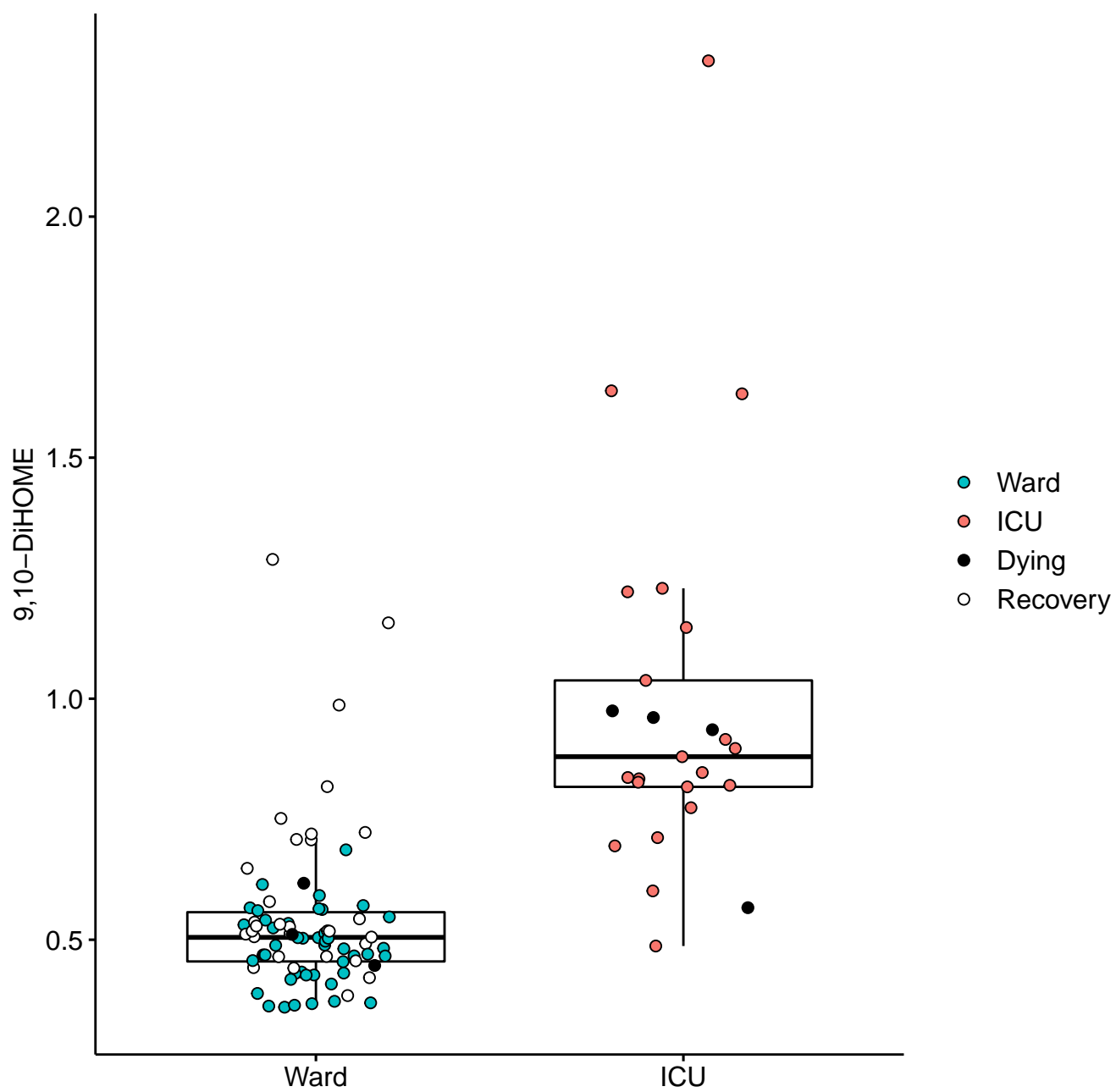

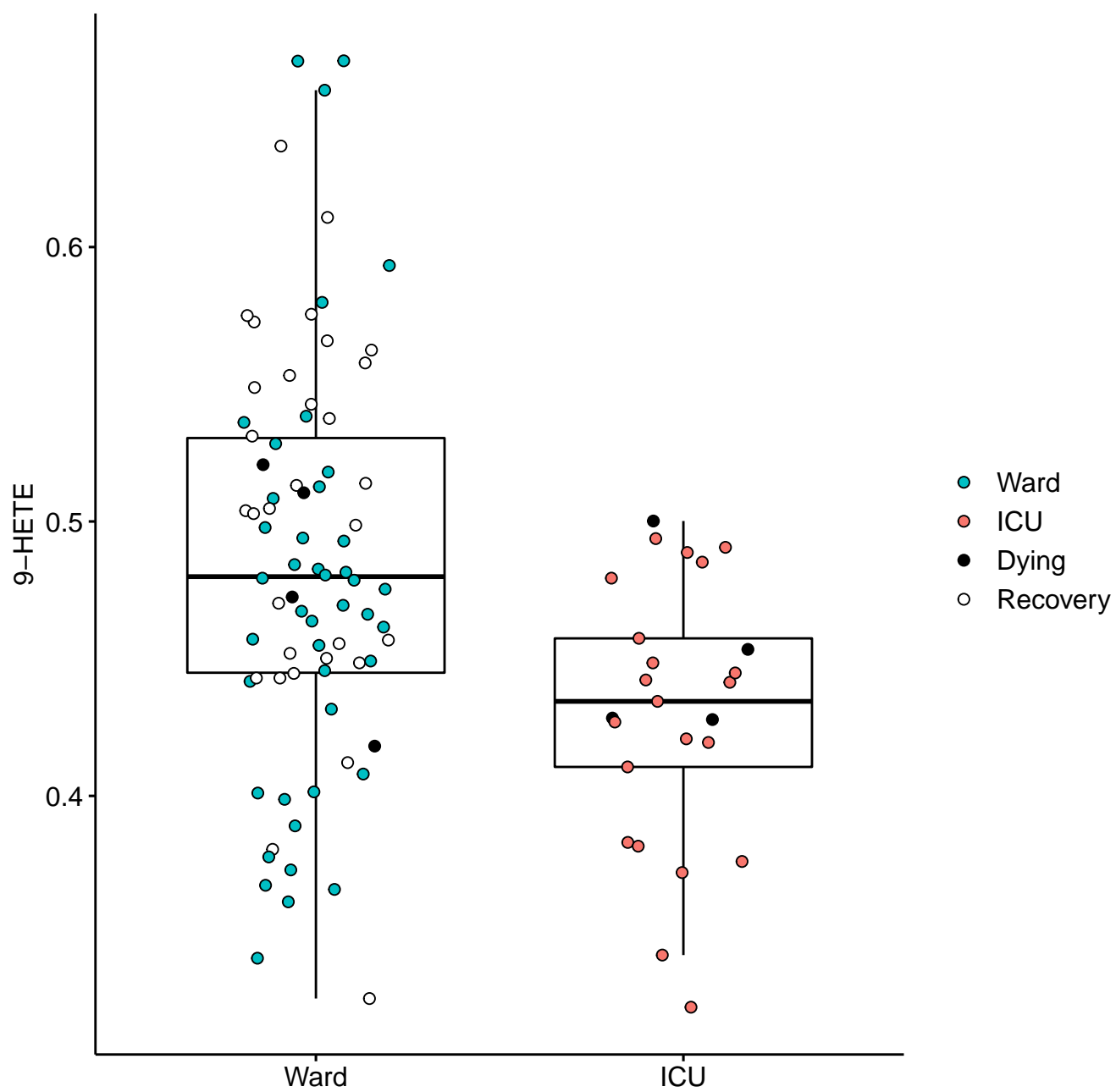

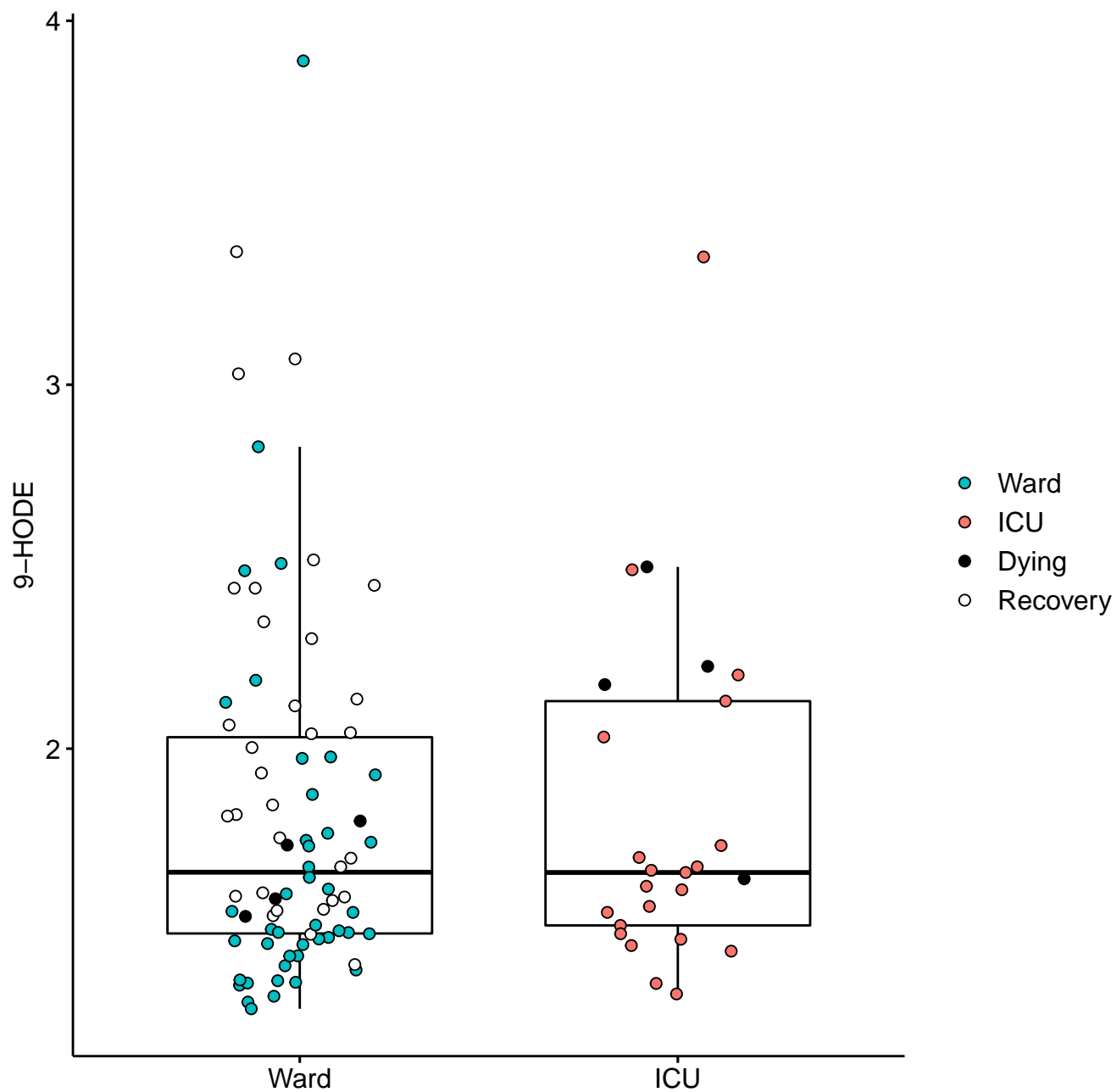

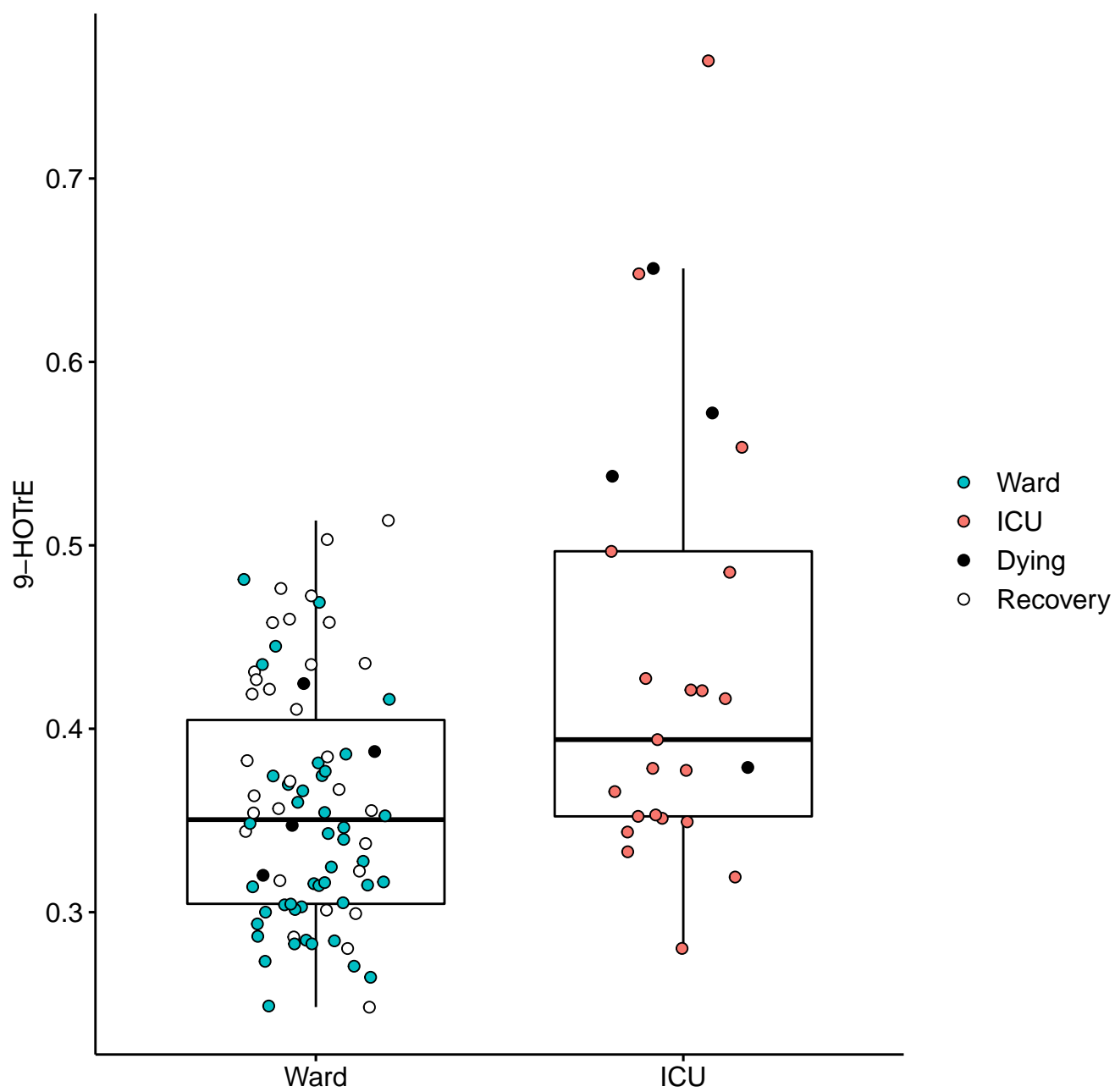

Supplement: Supplementary file 1 [file metabolites-12-00619-s001.zip › Document S2.pdf]

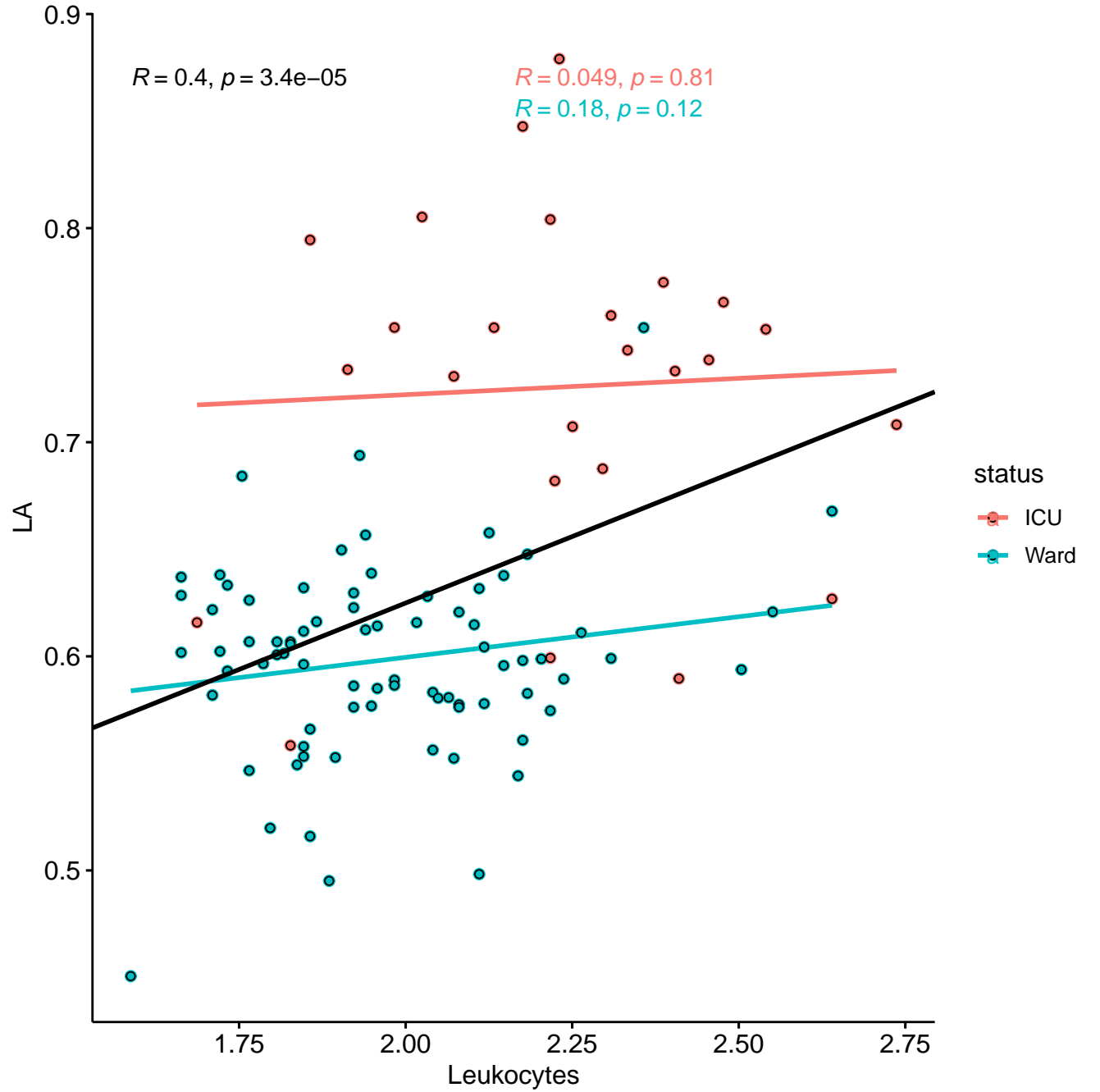

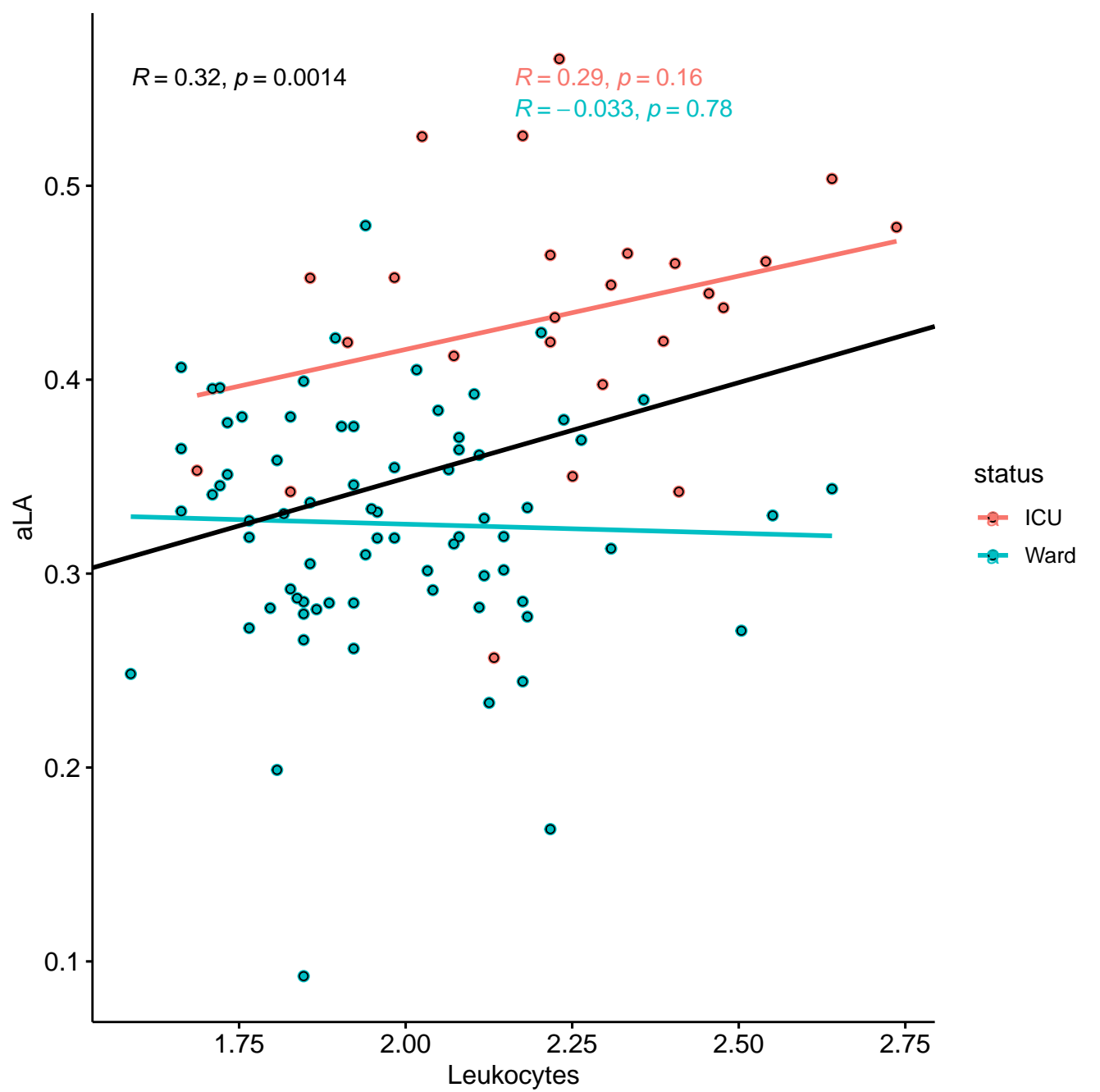

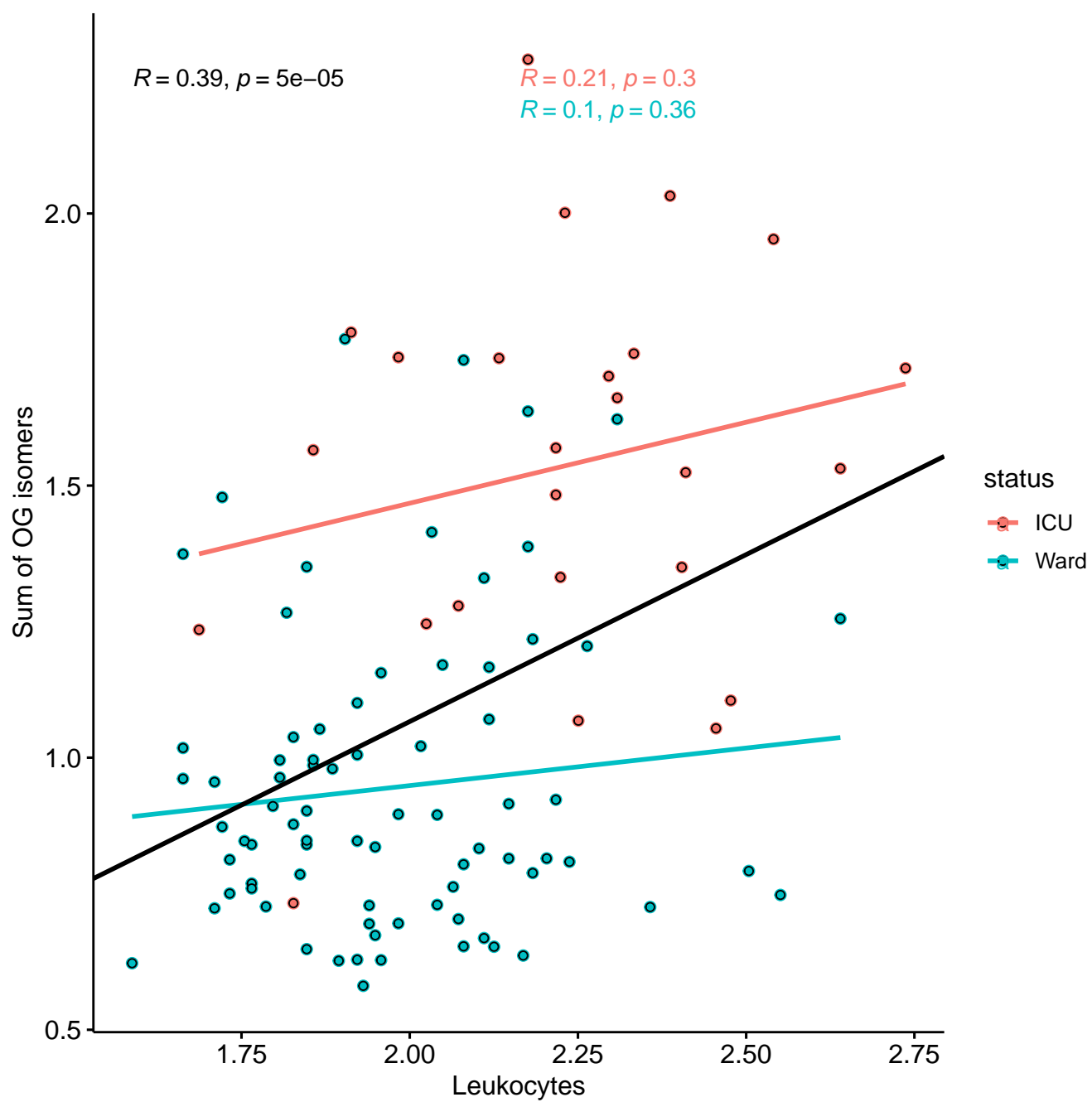

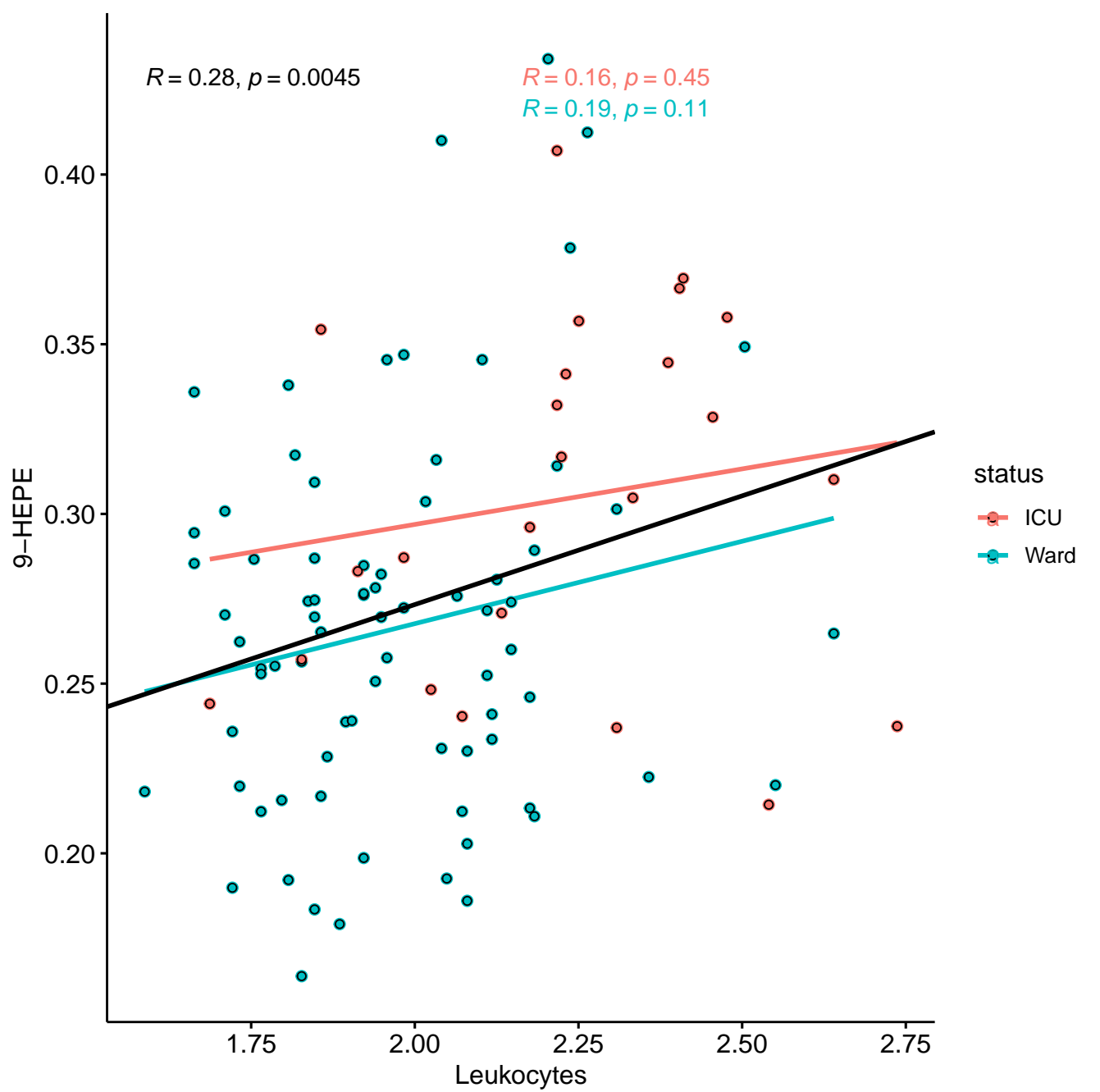

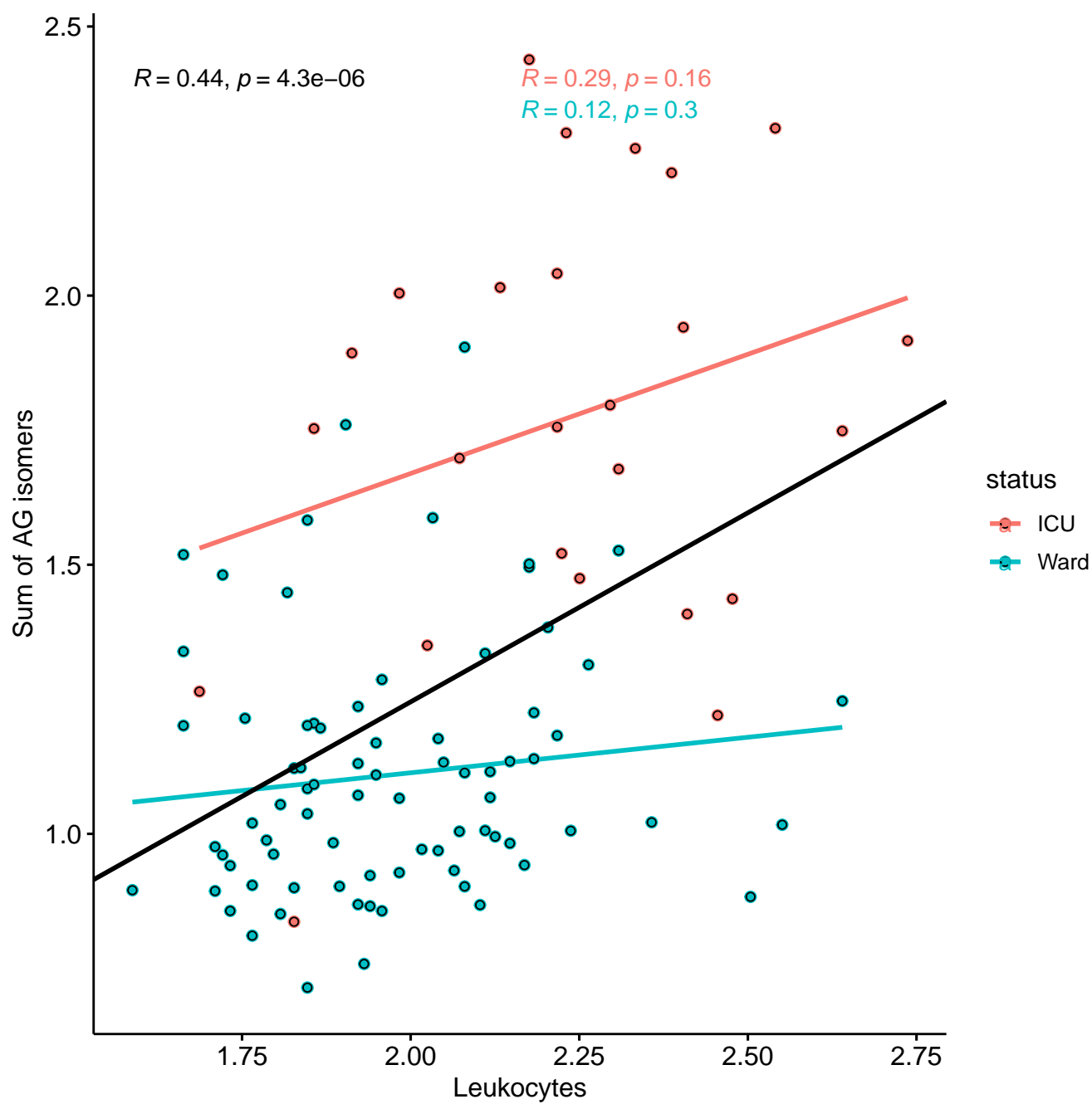

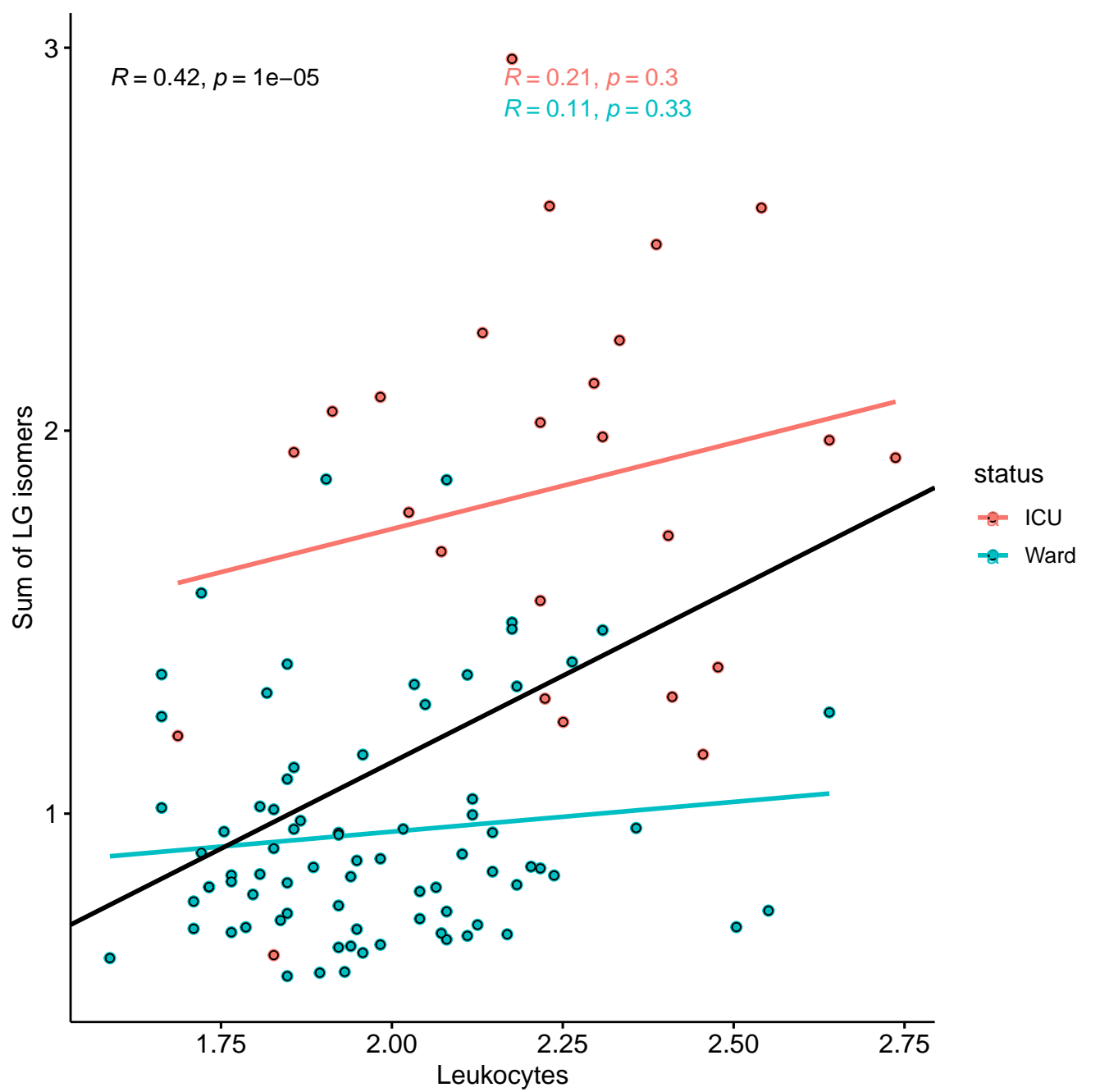

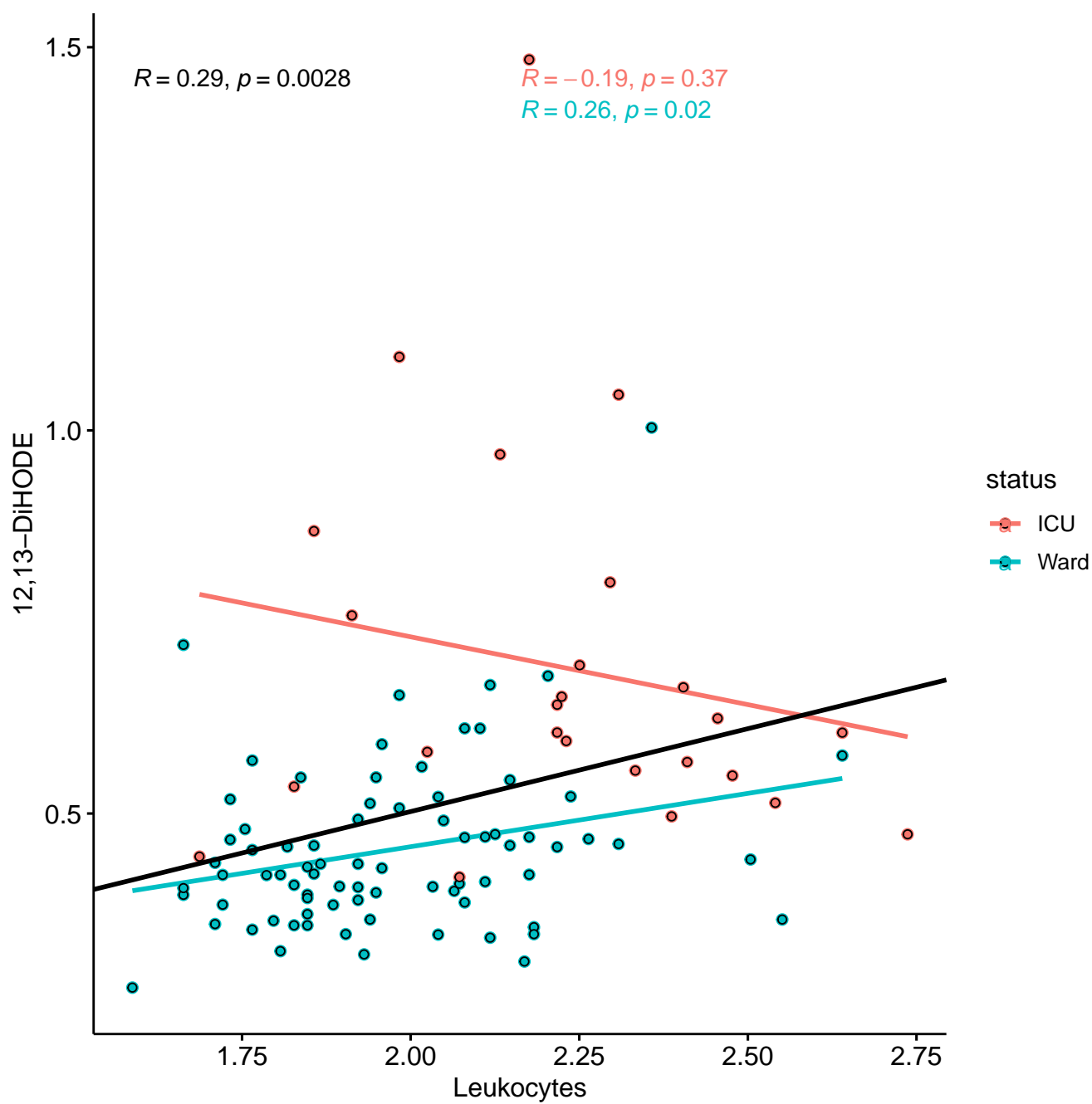

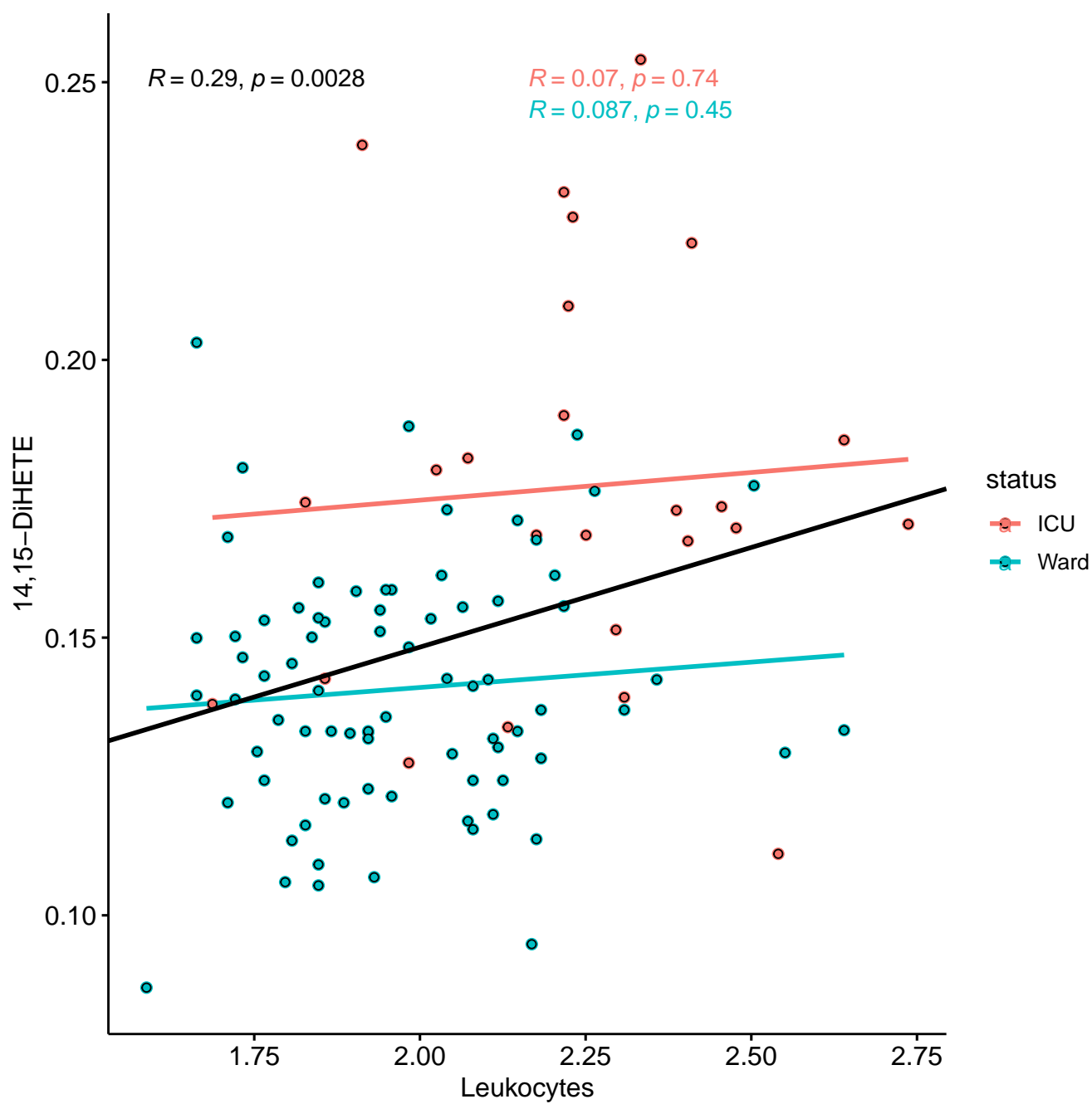

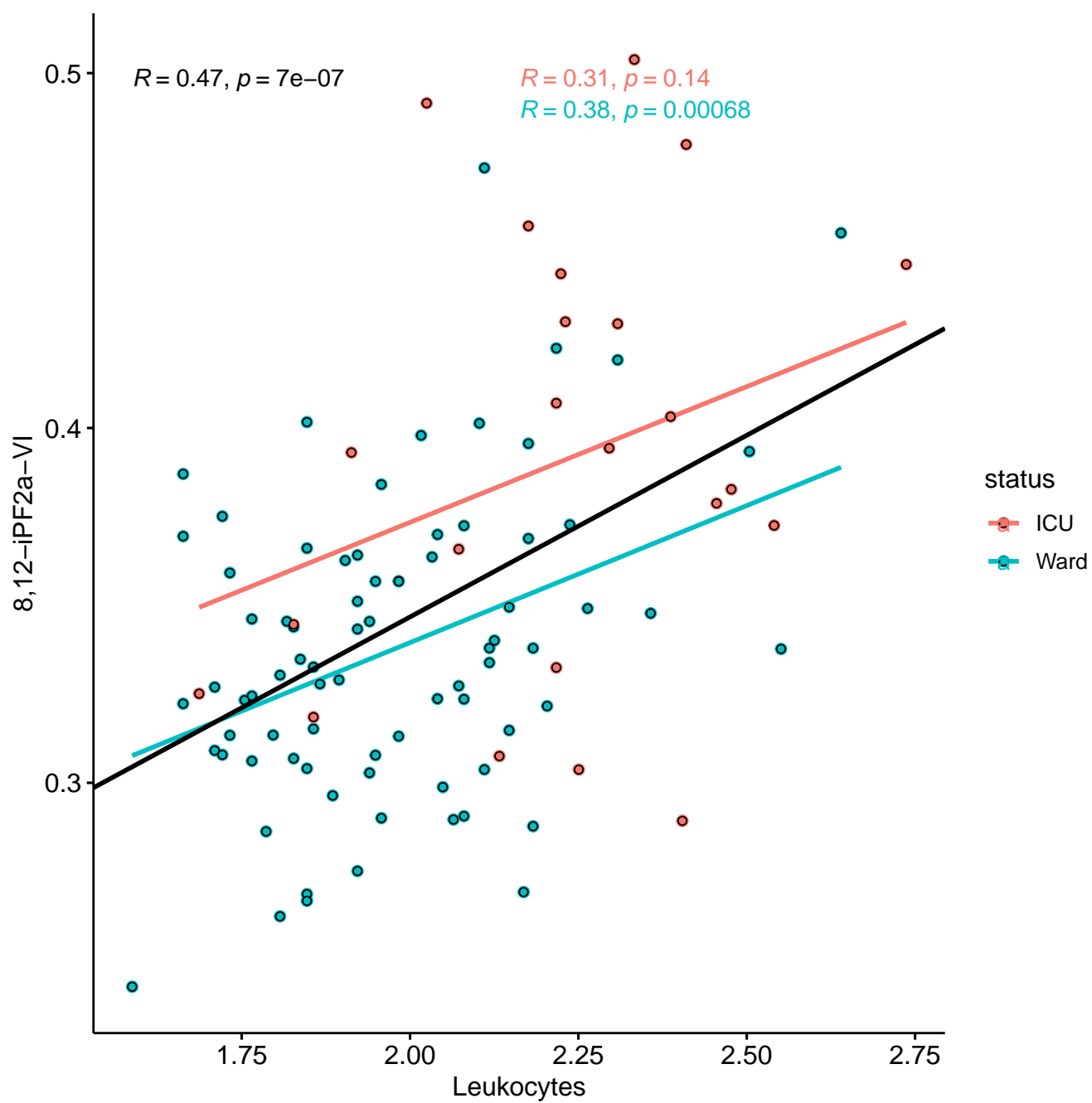

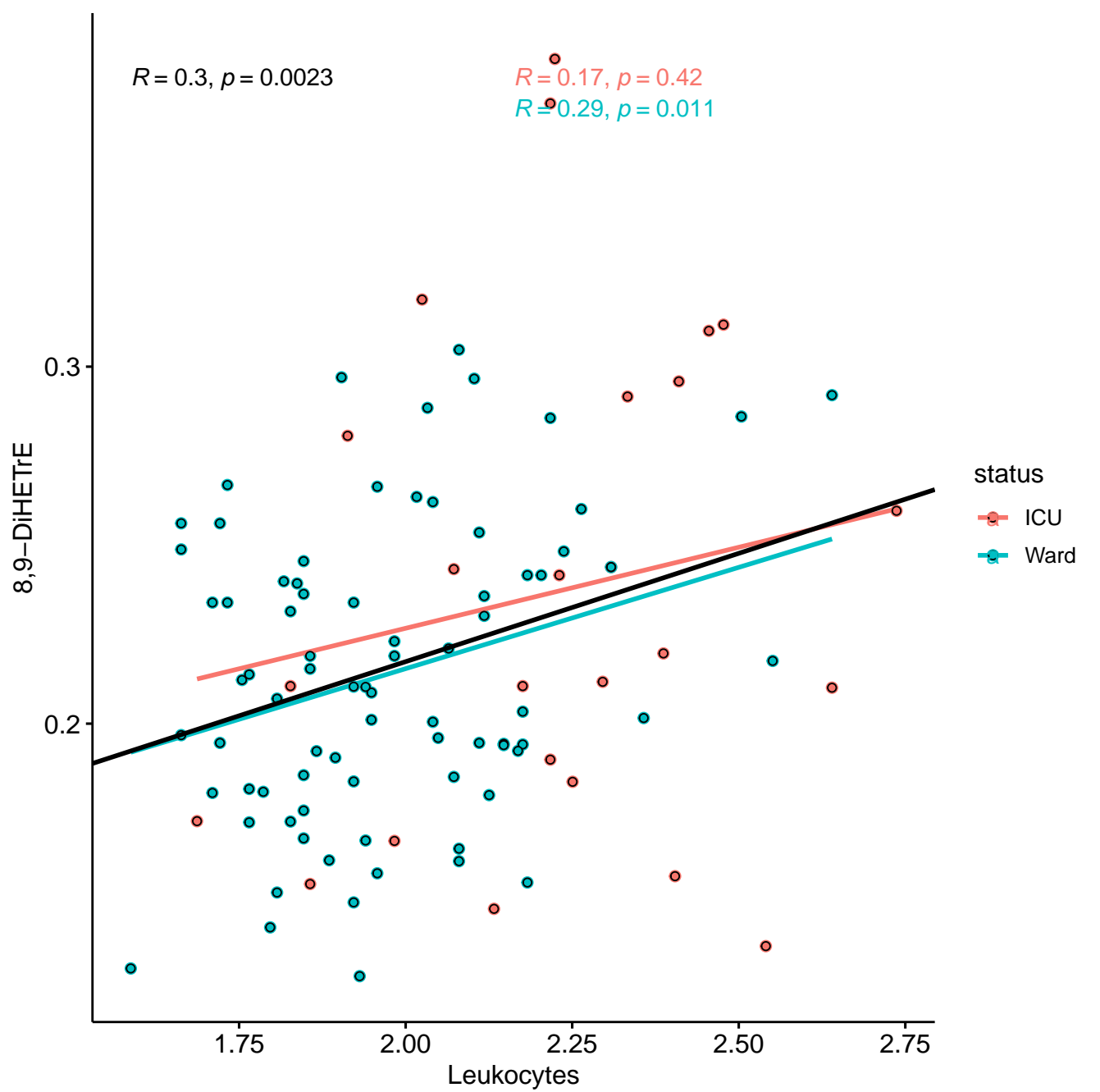

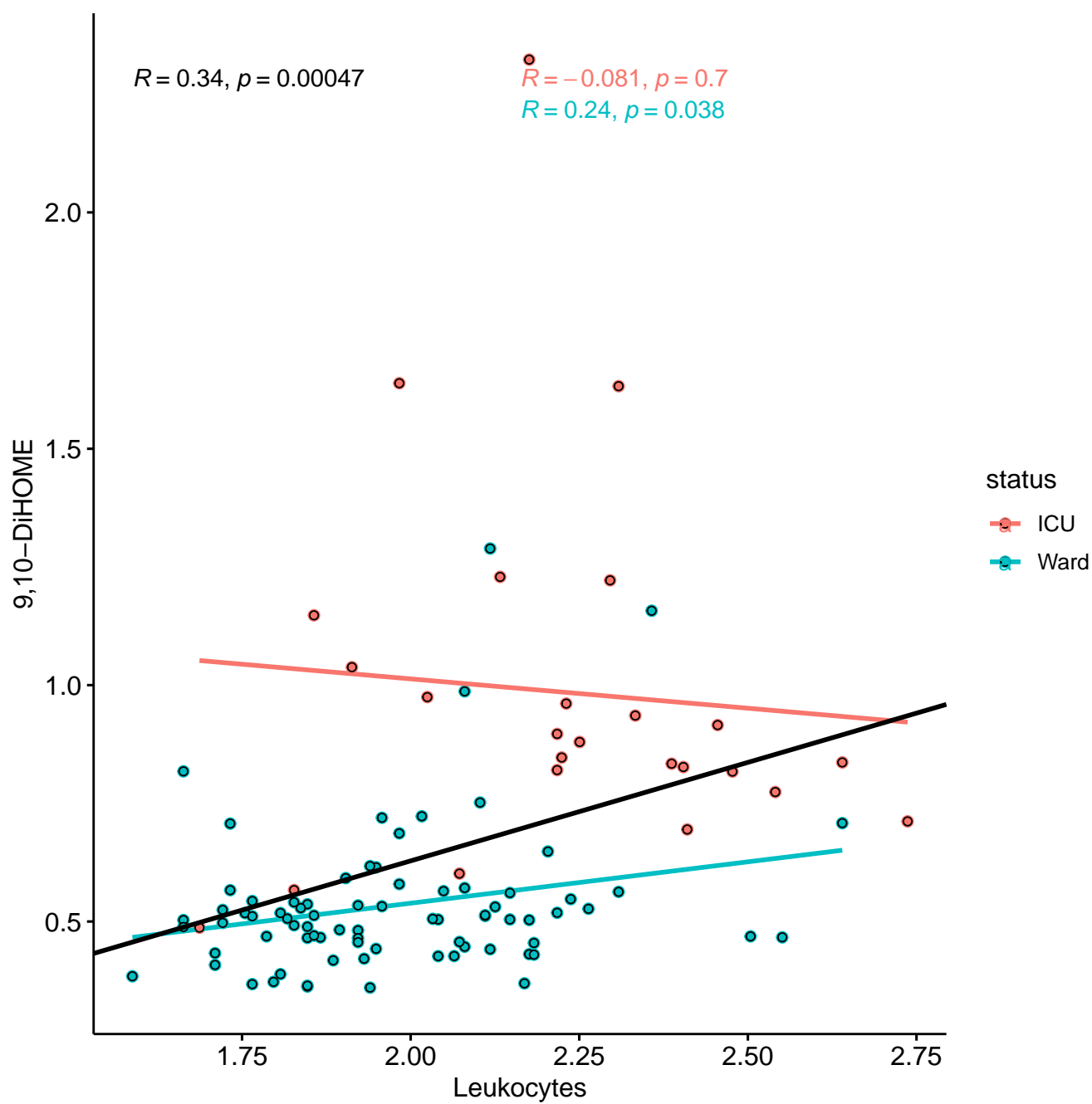

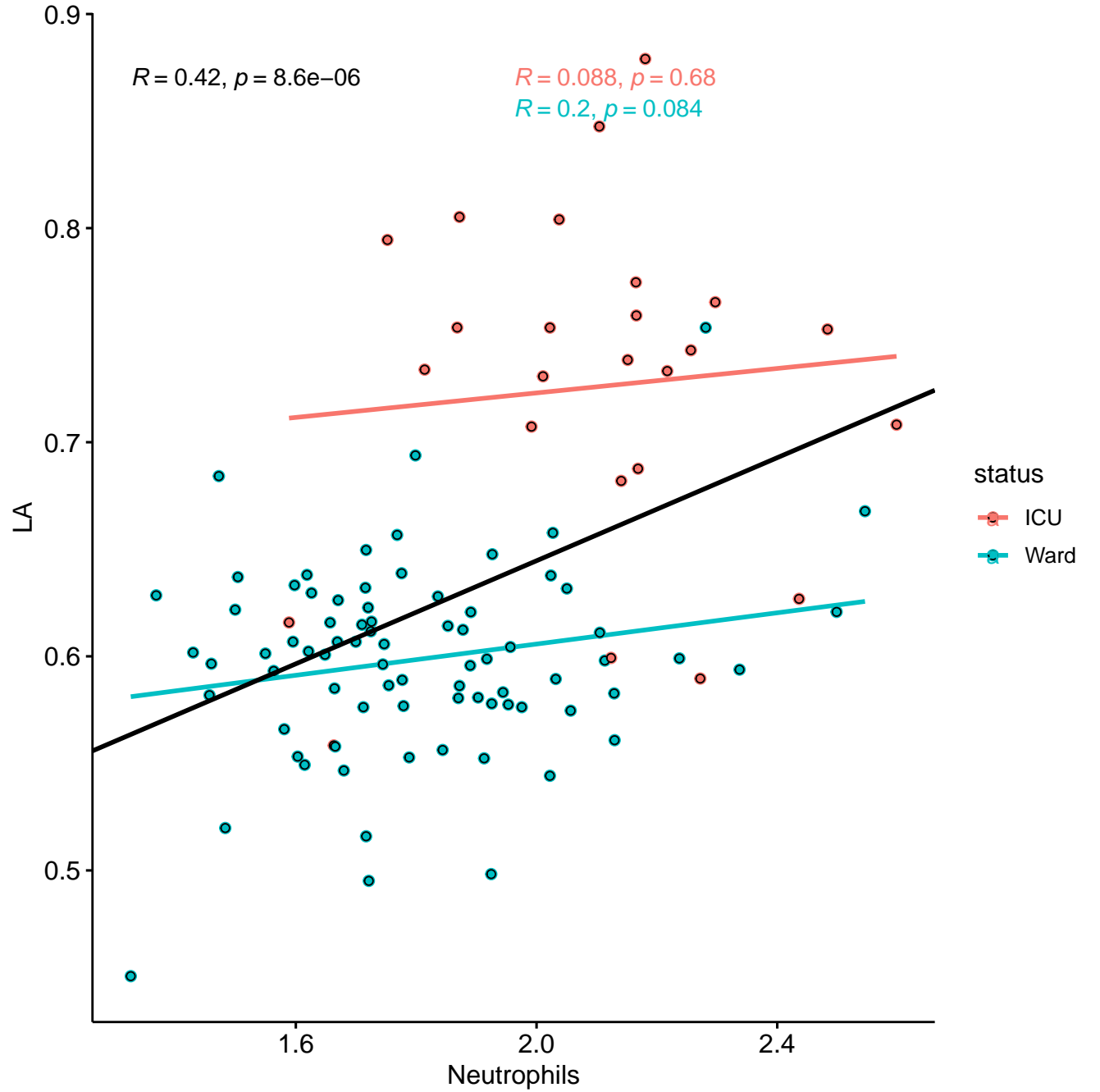

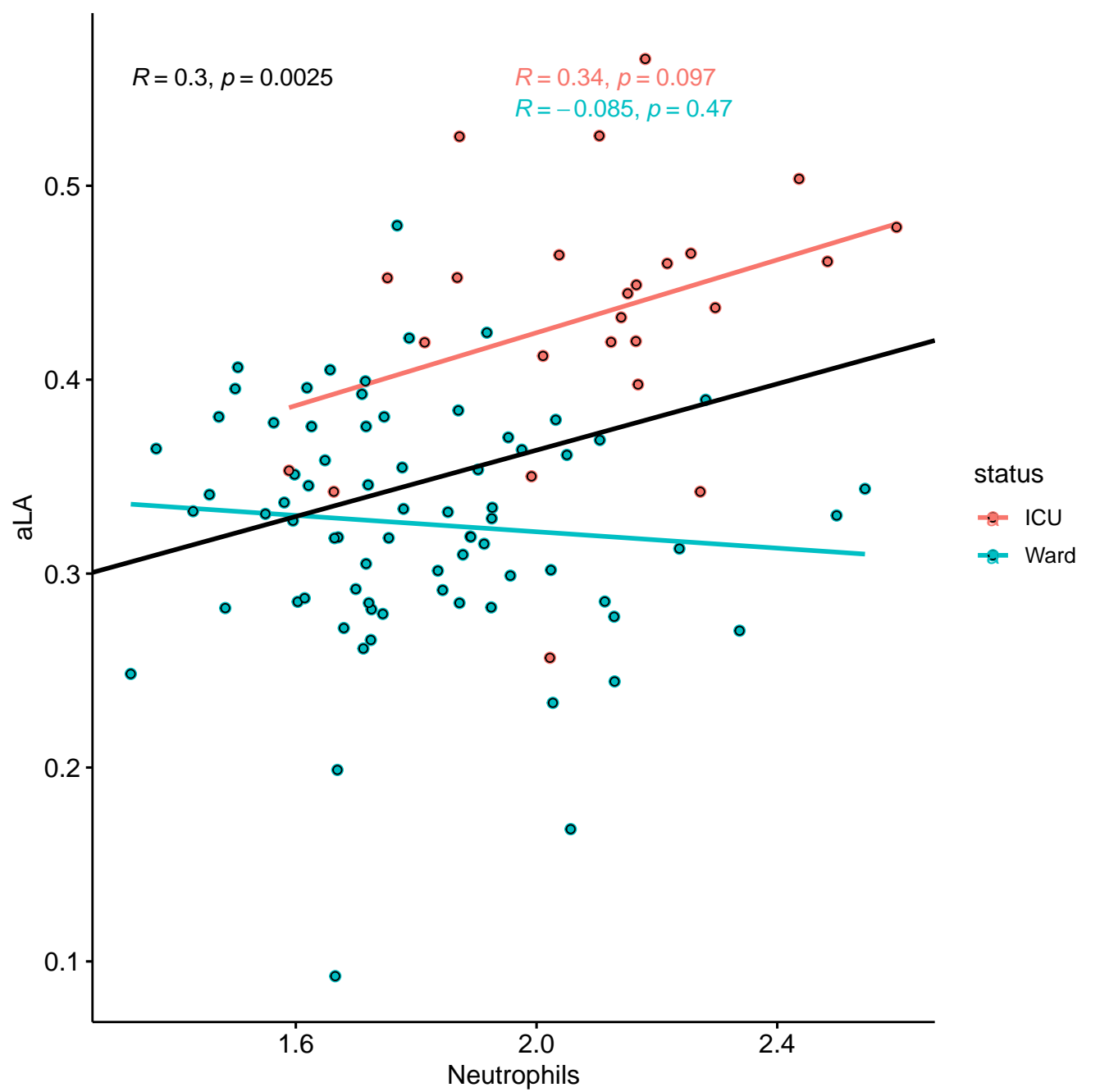

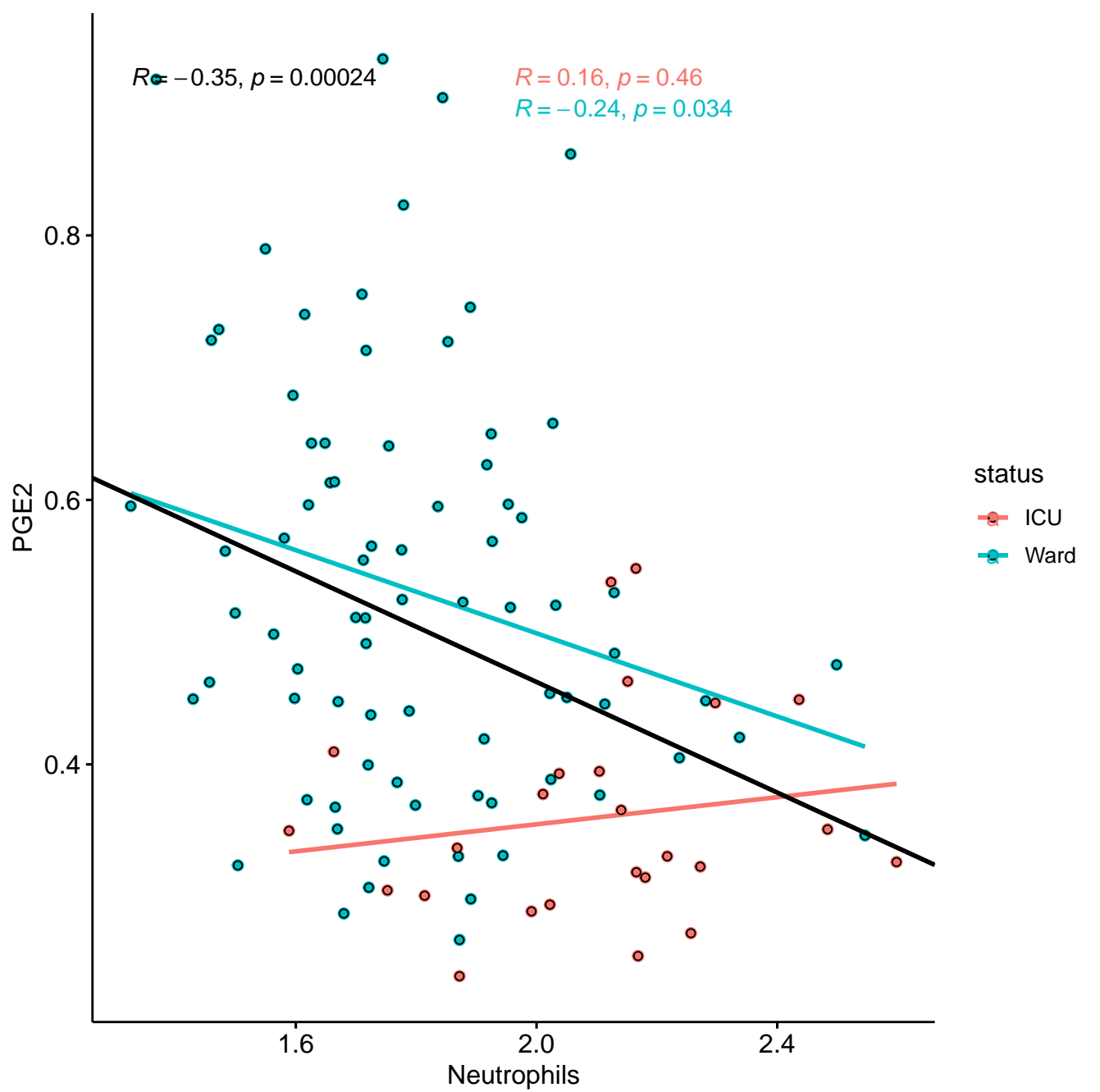

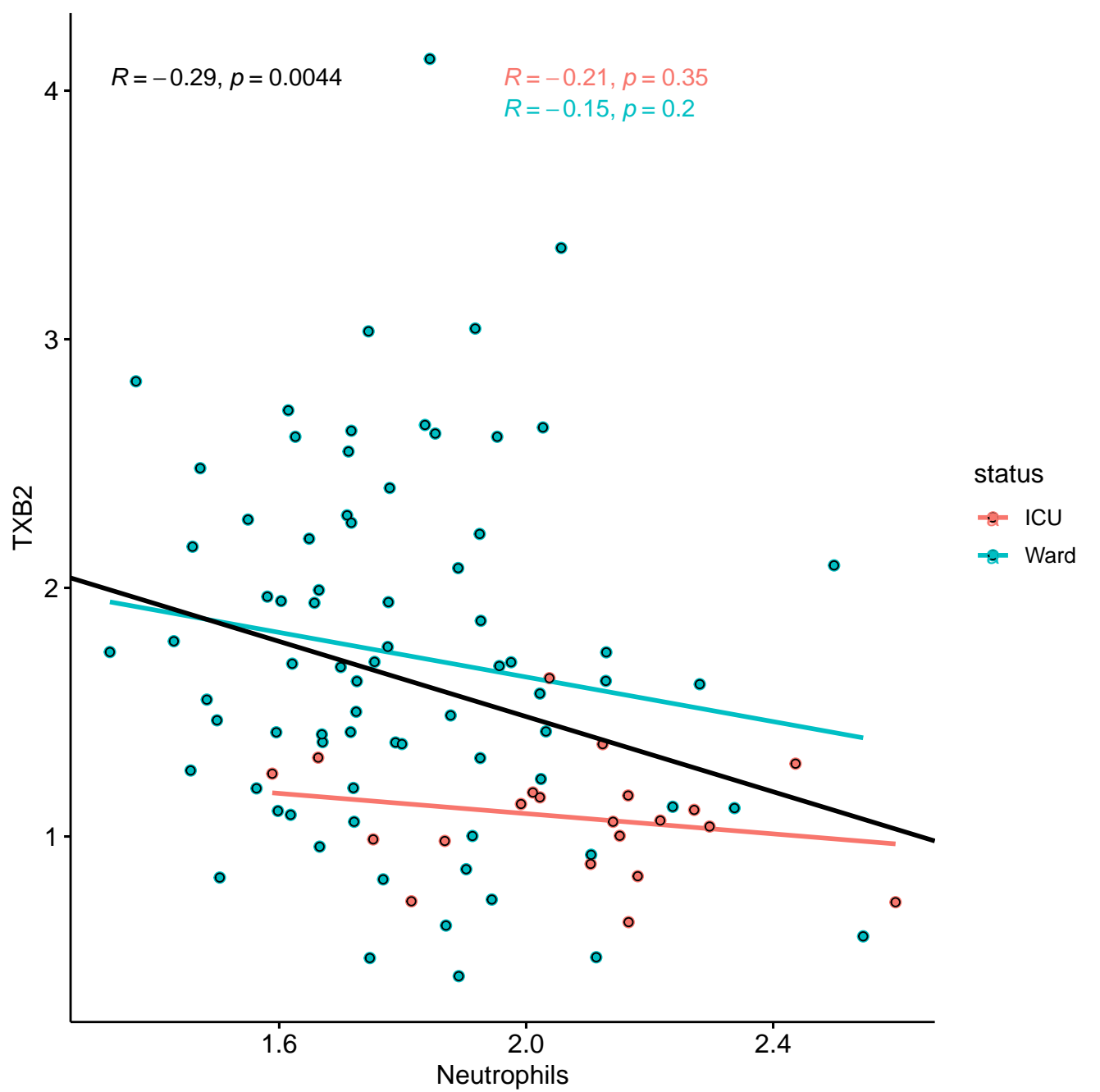

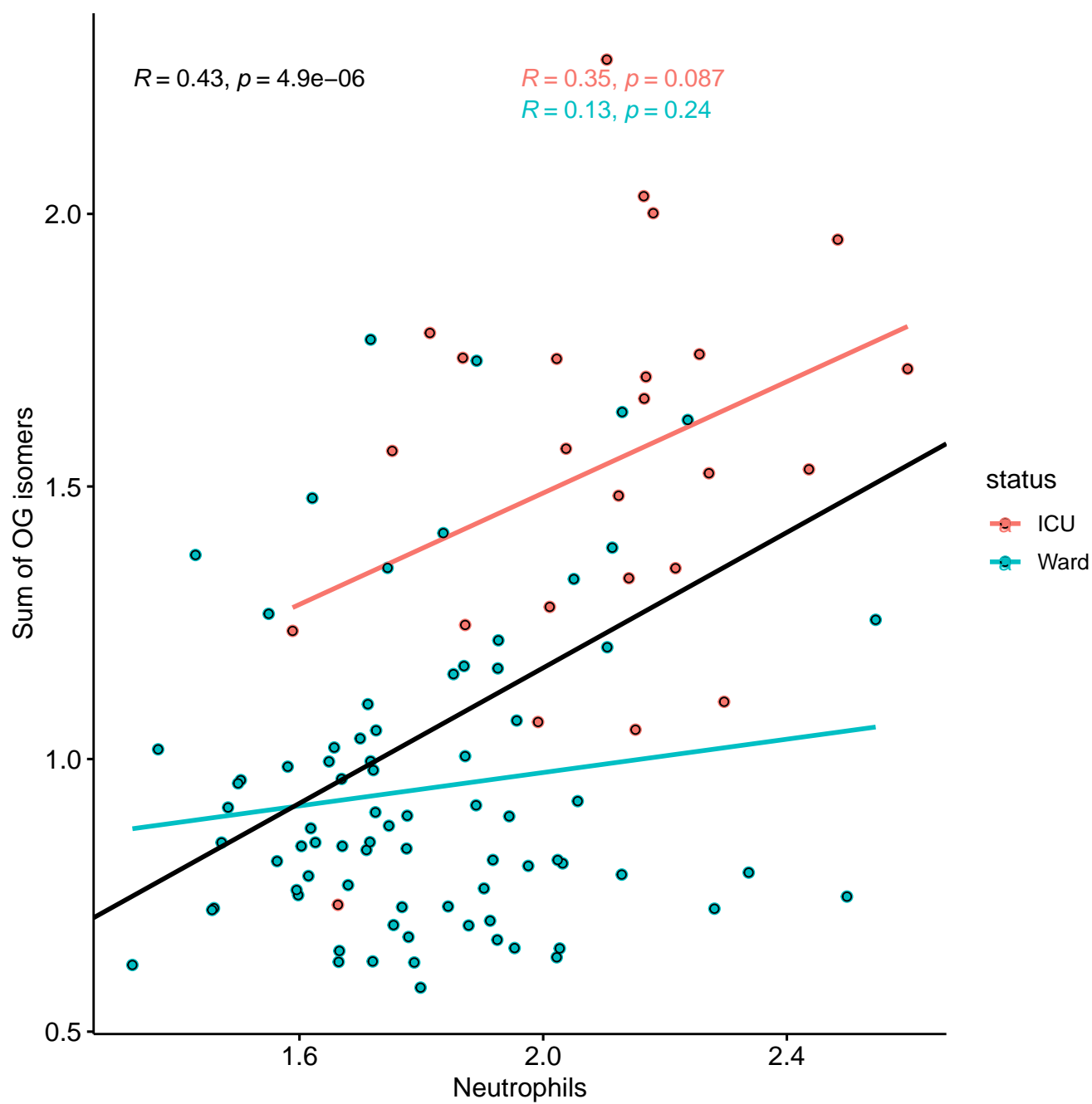

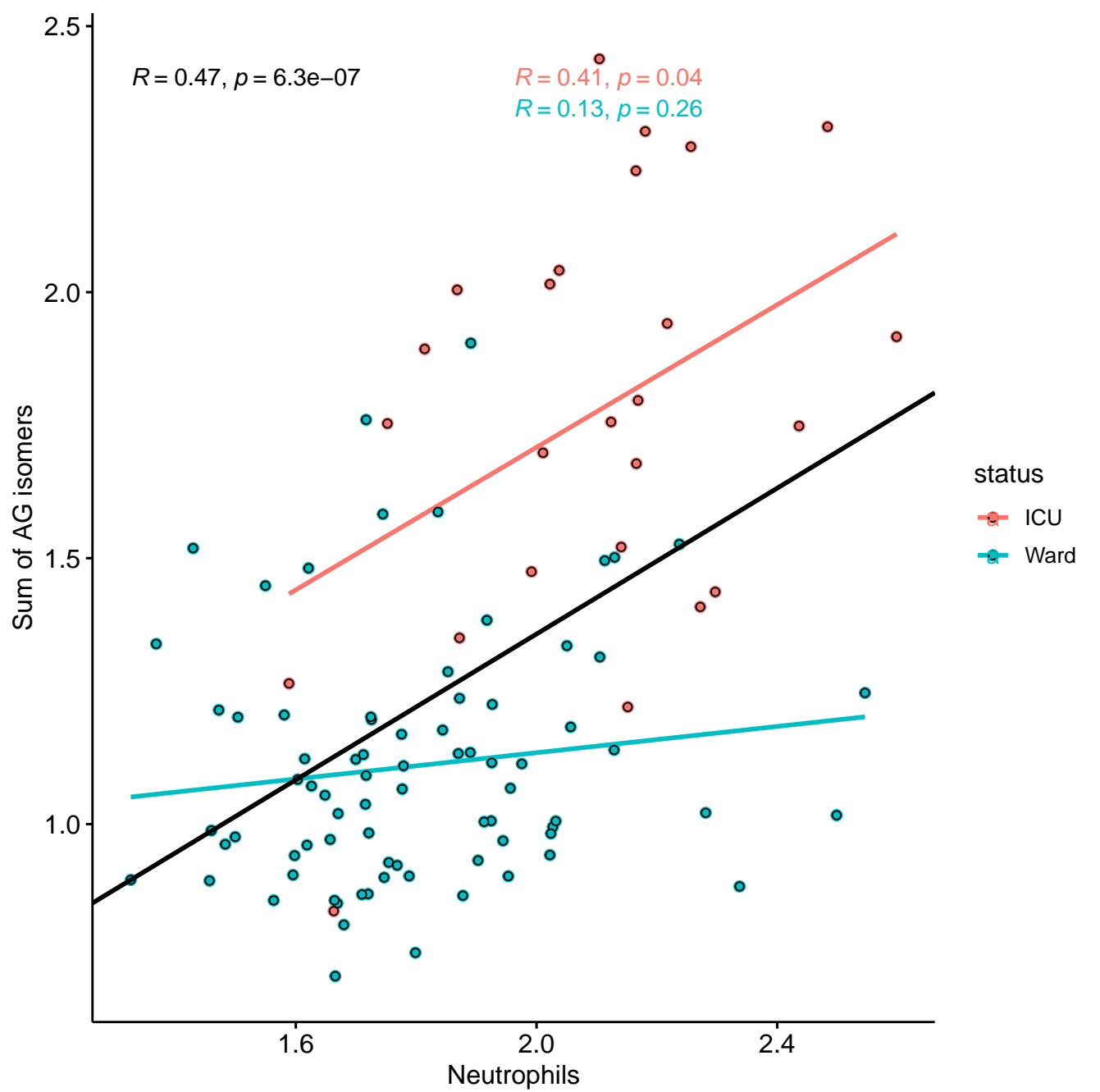

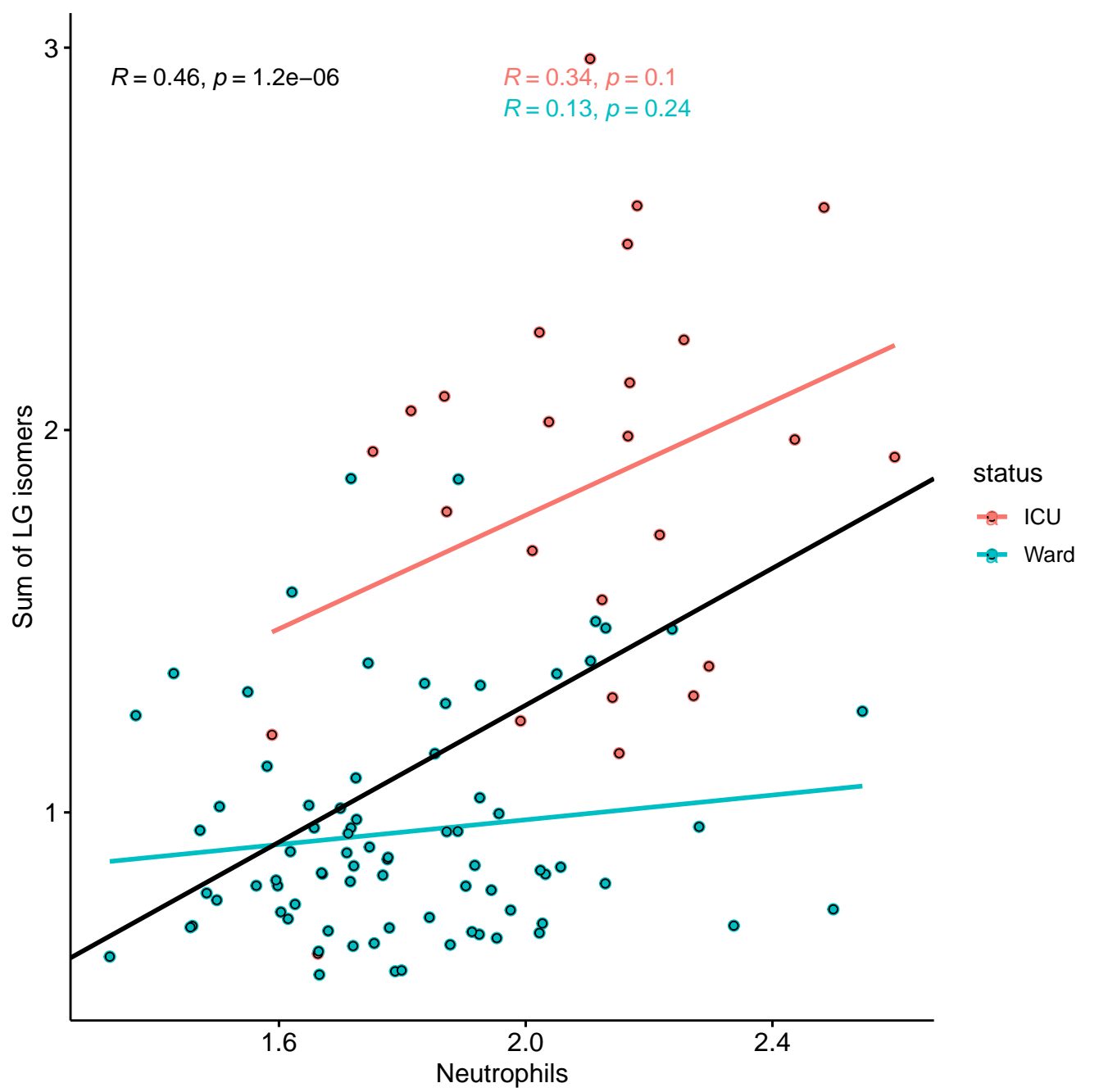

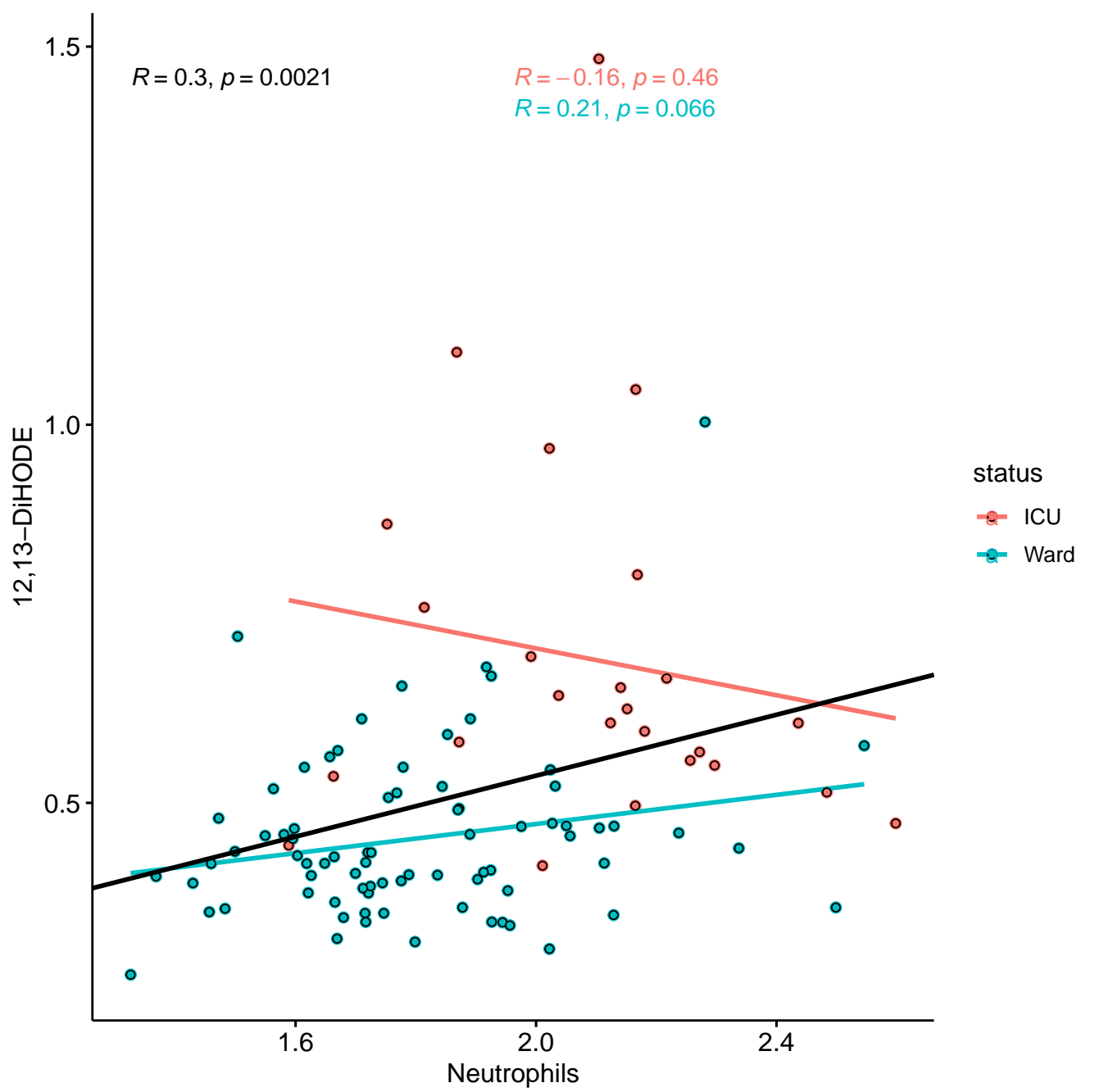

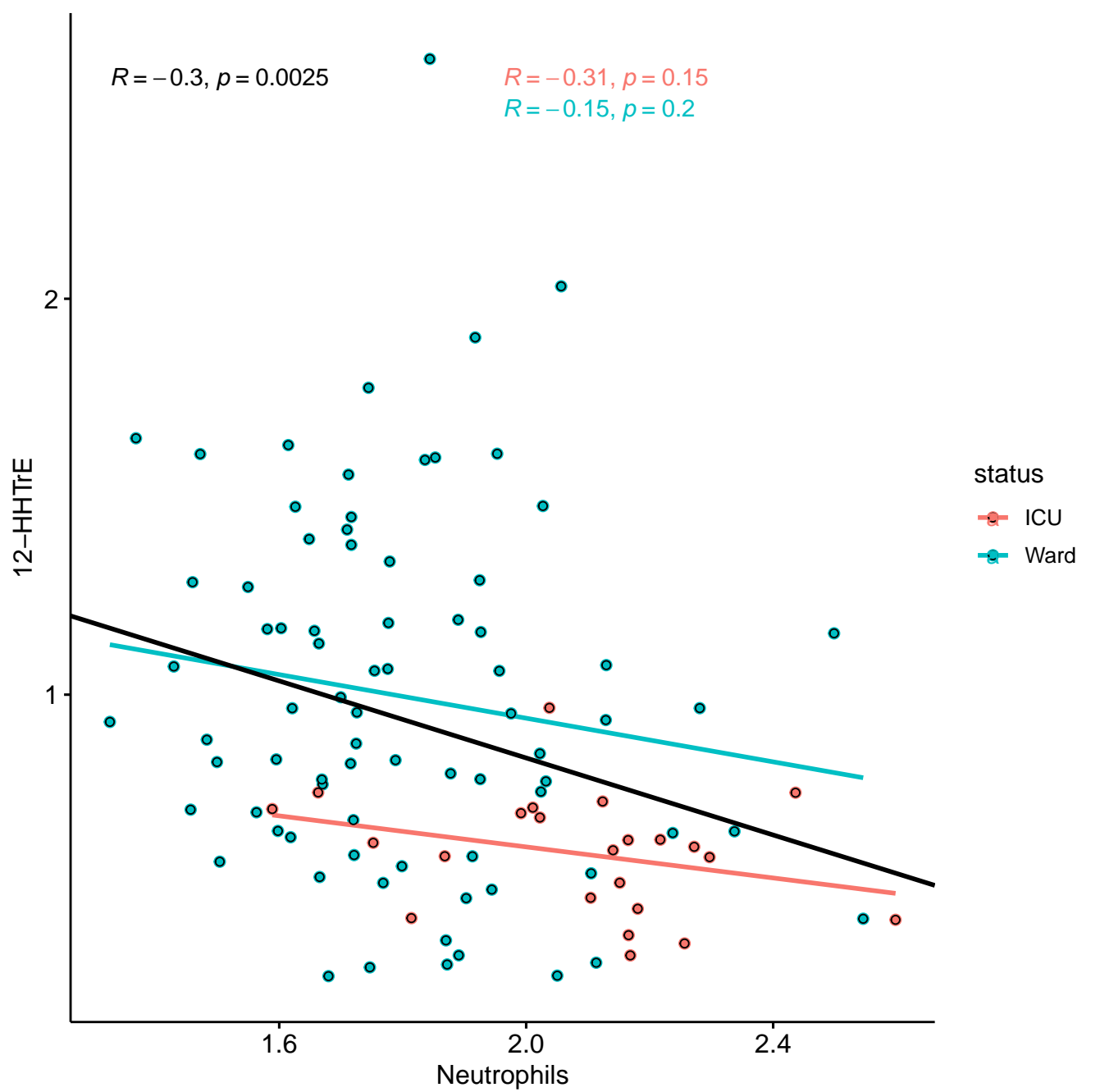

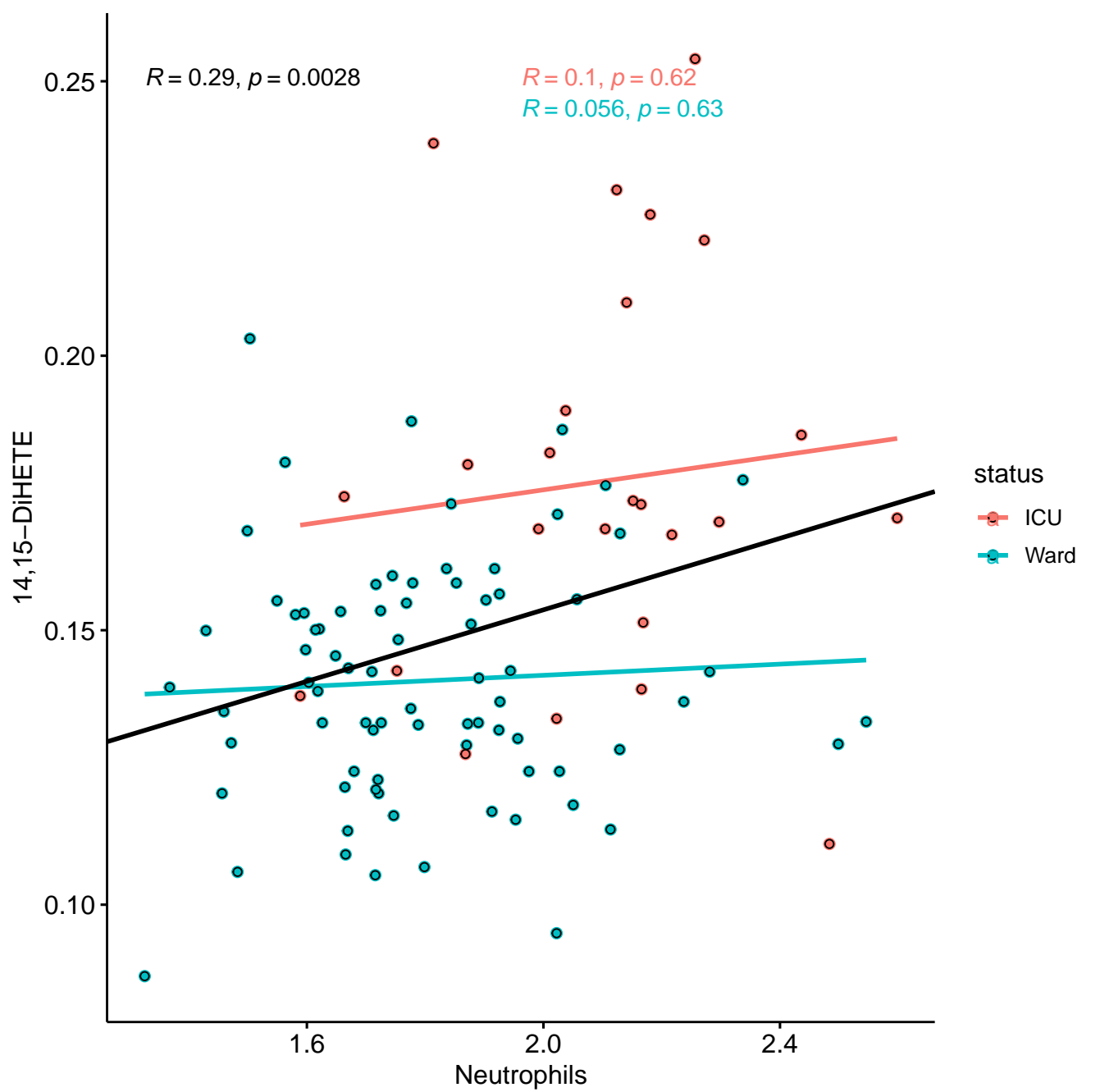

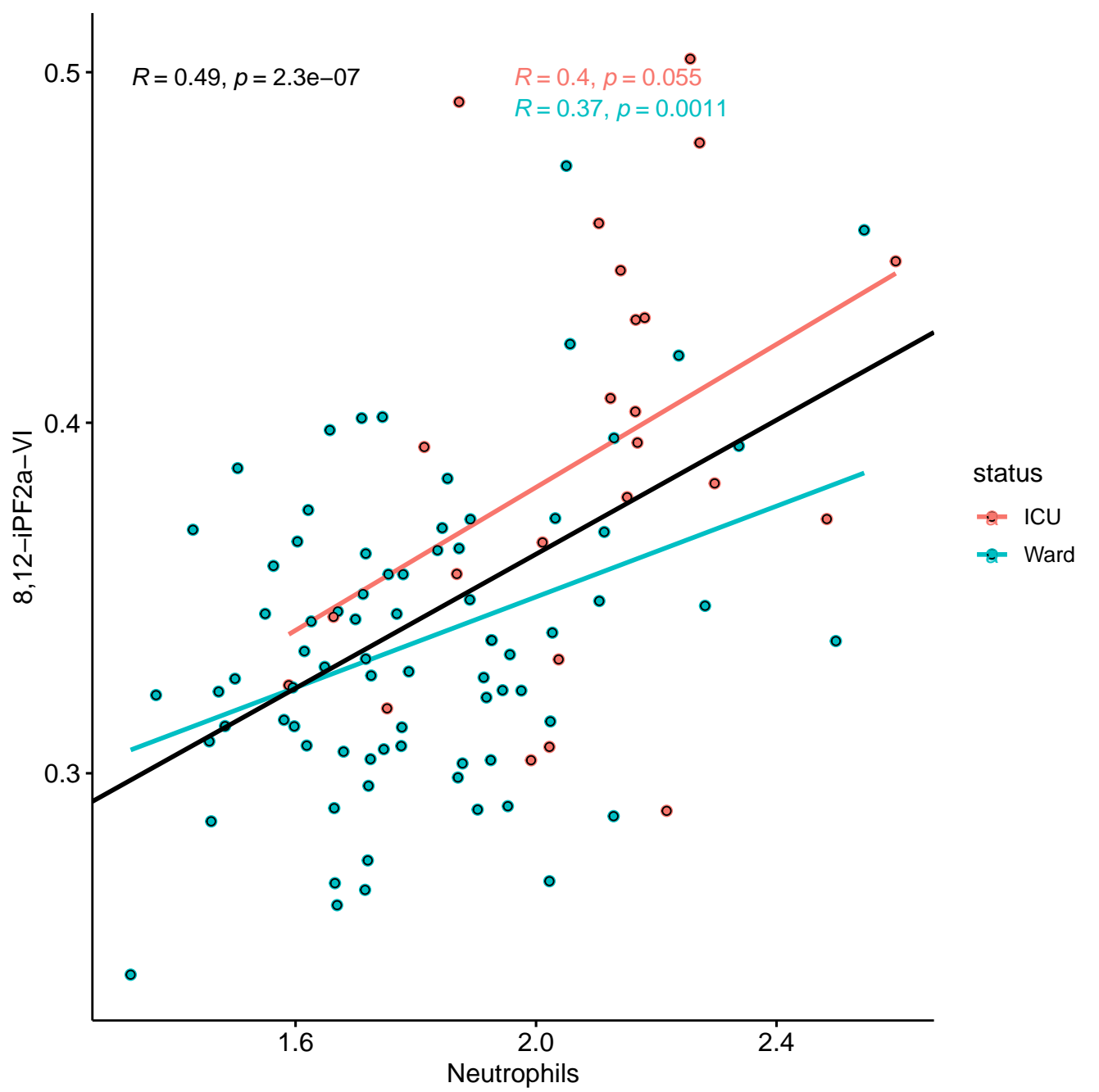

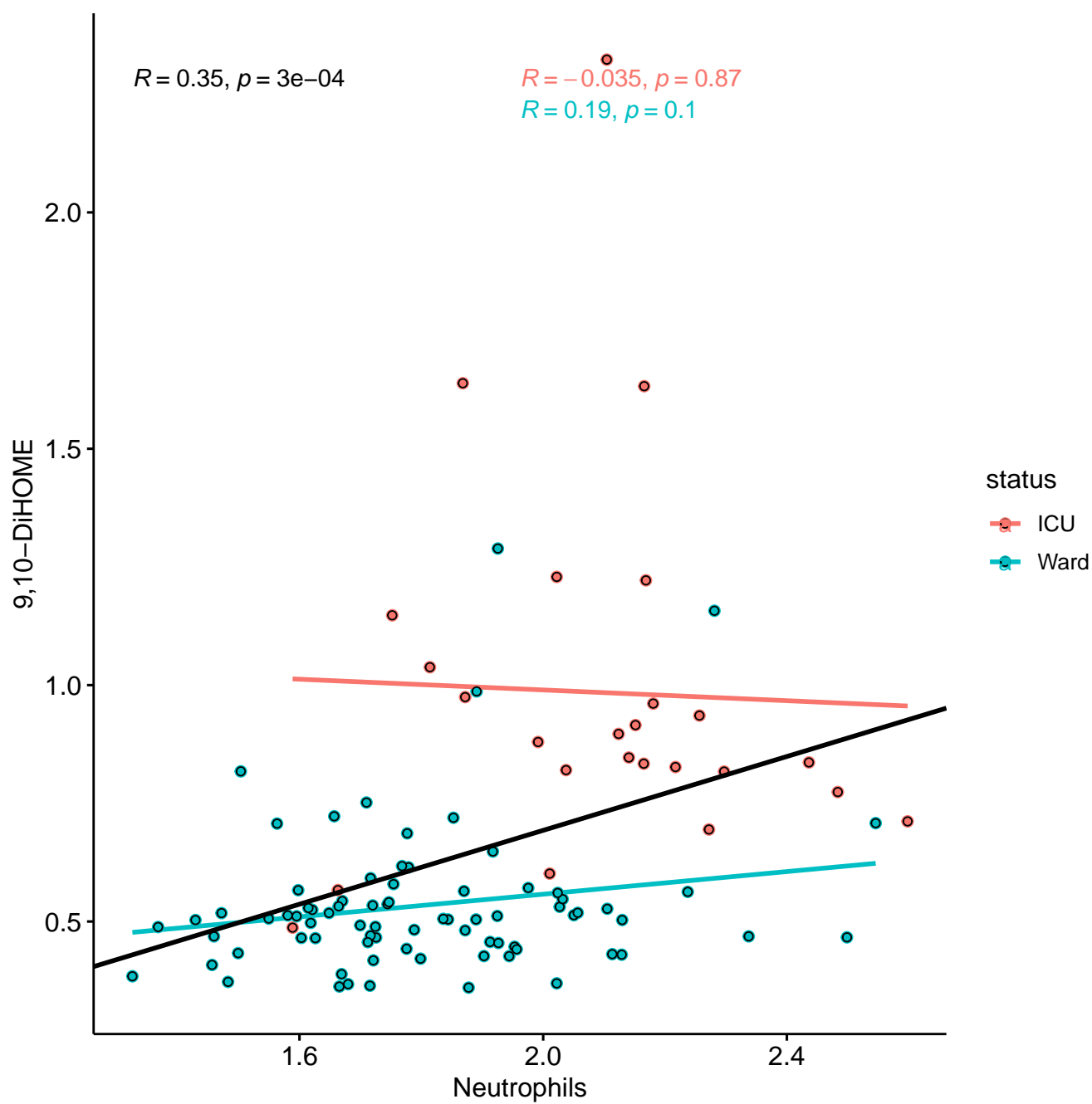

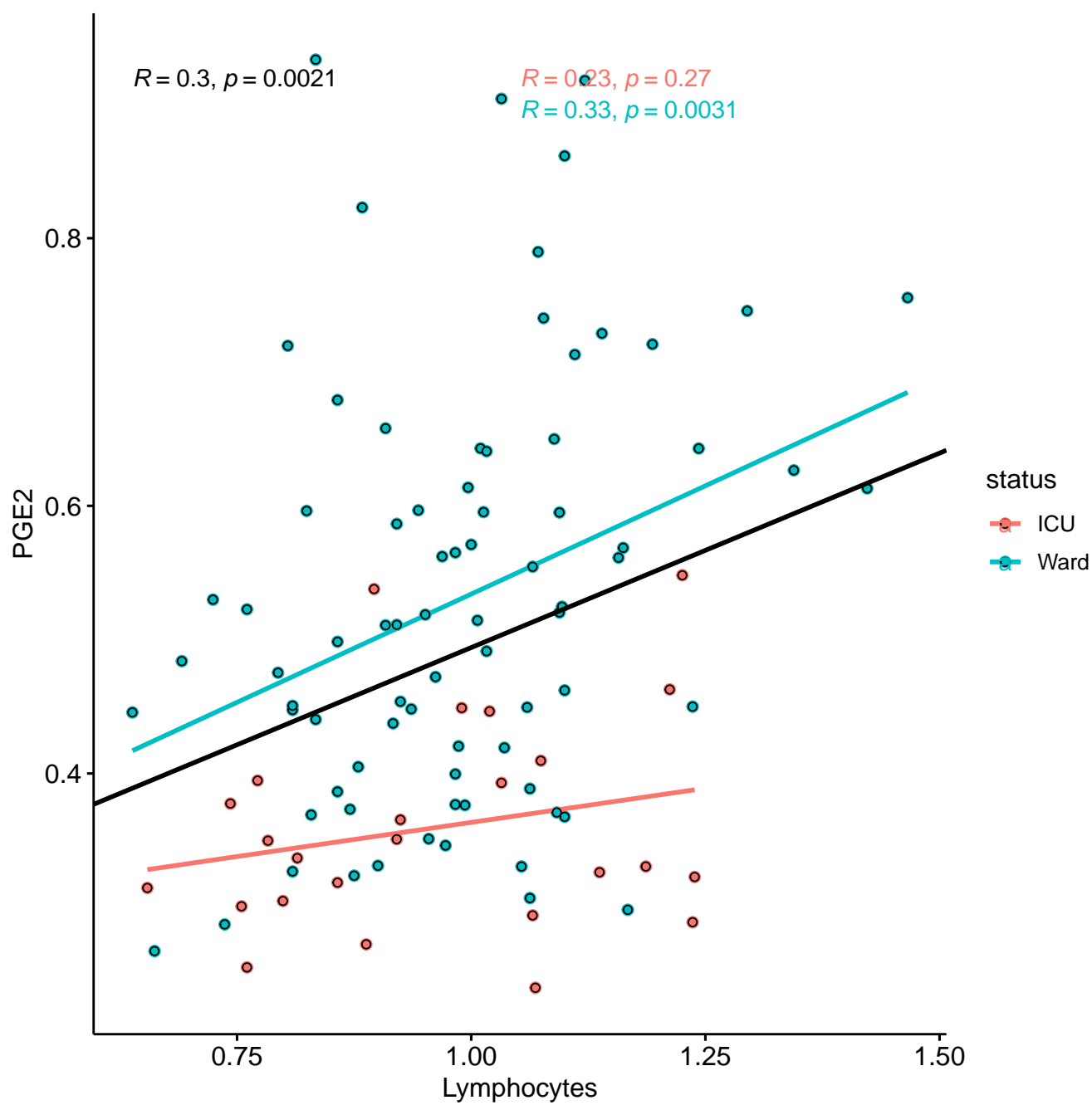

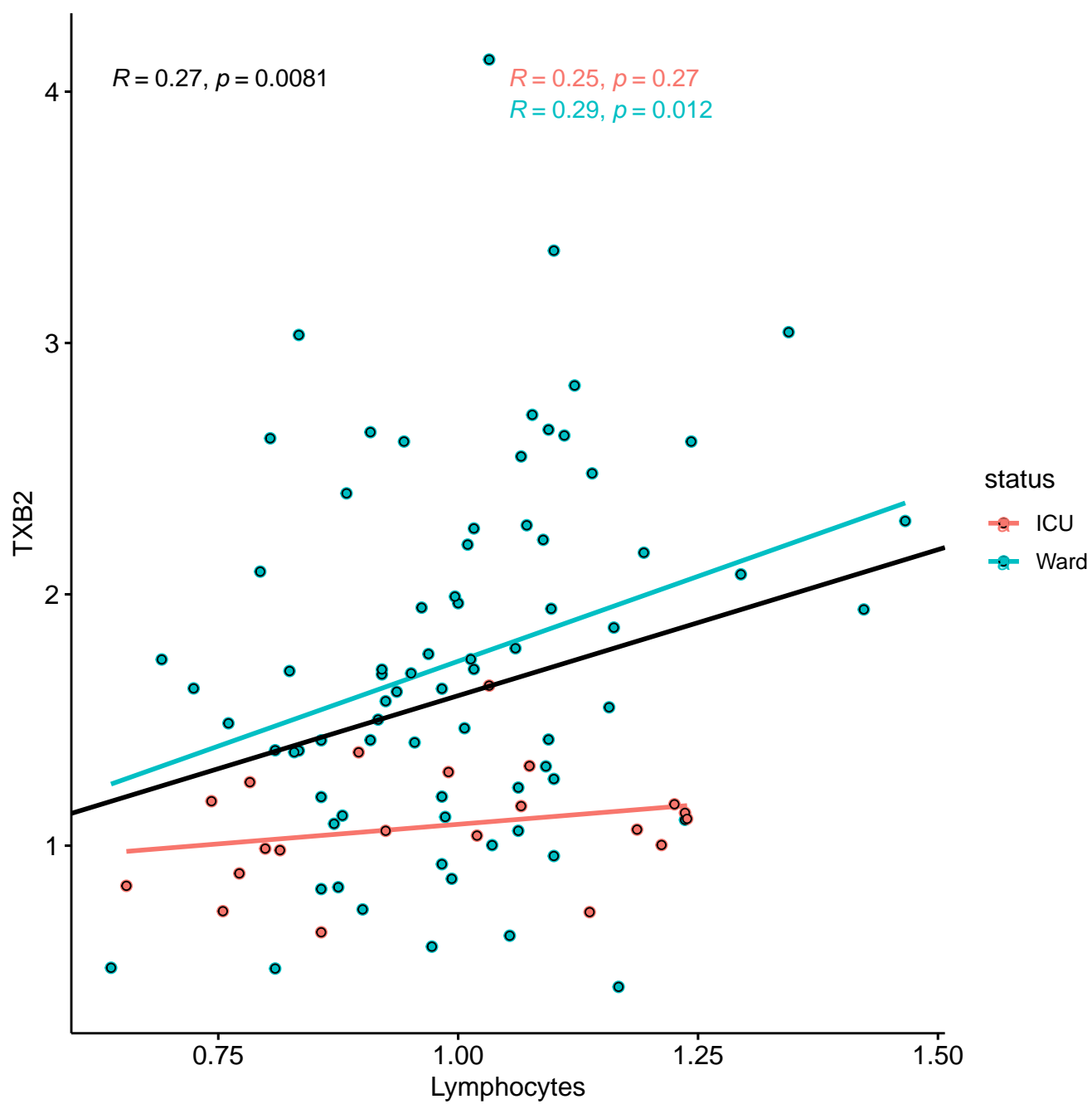

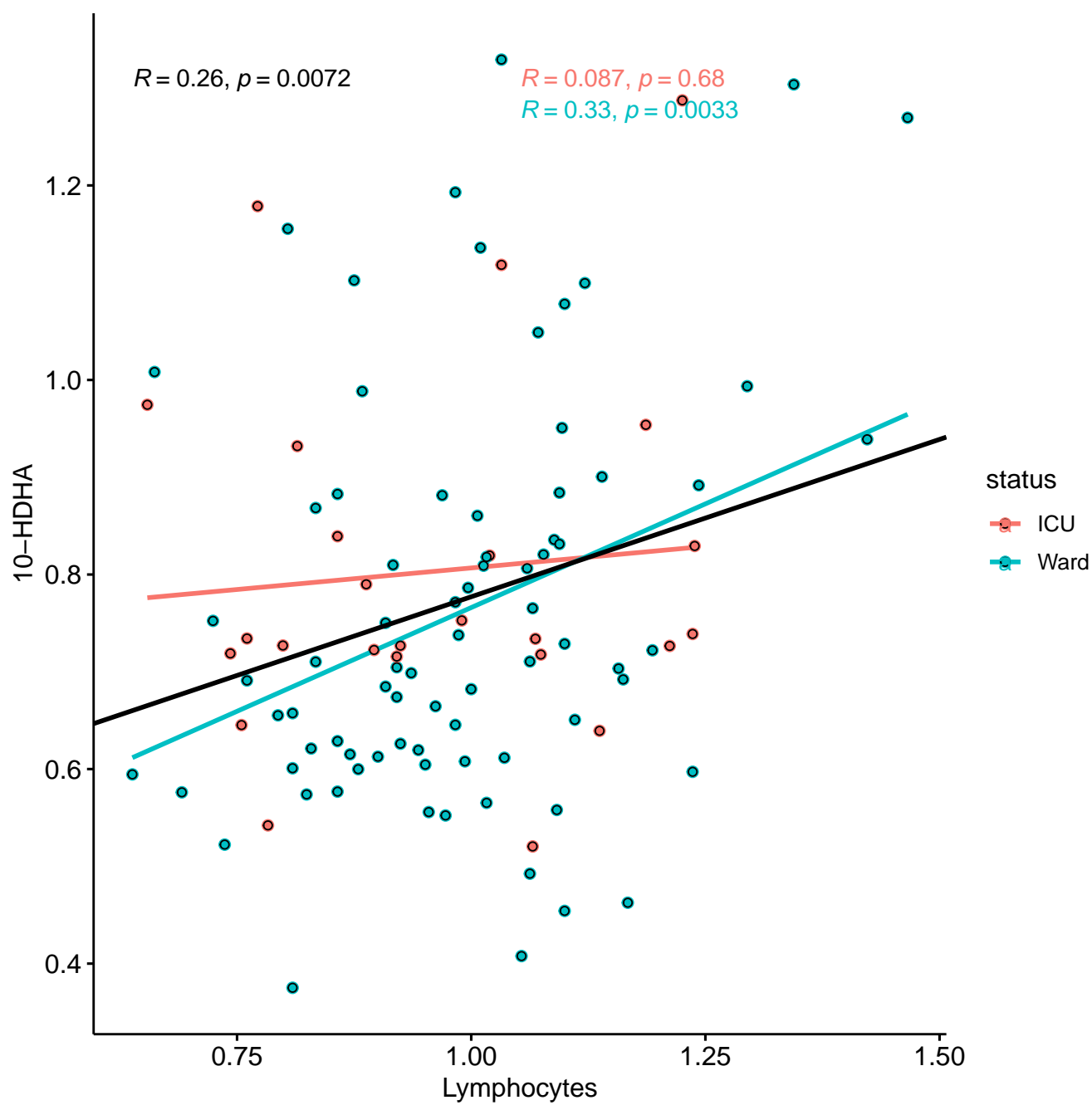

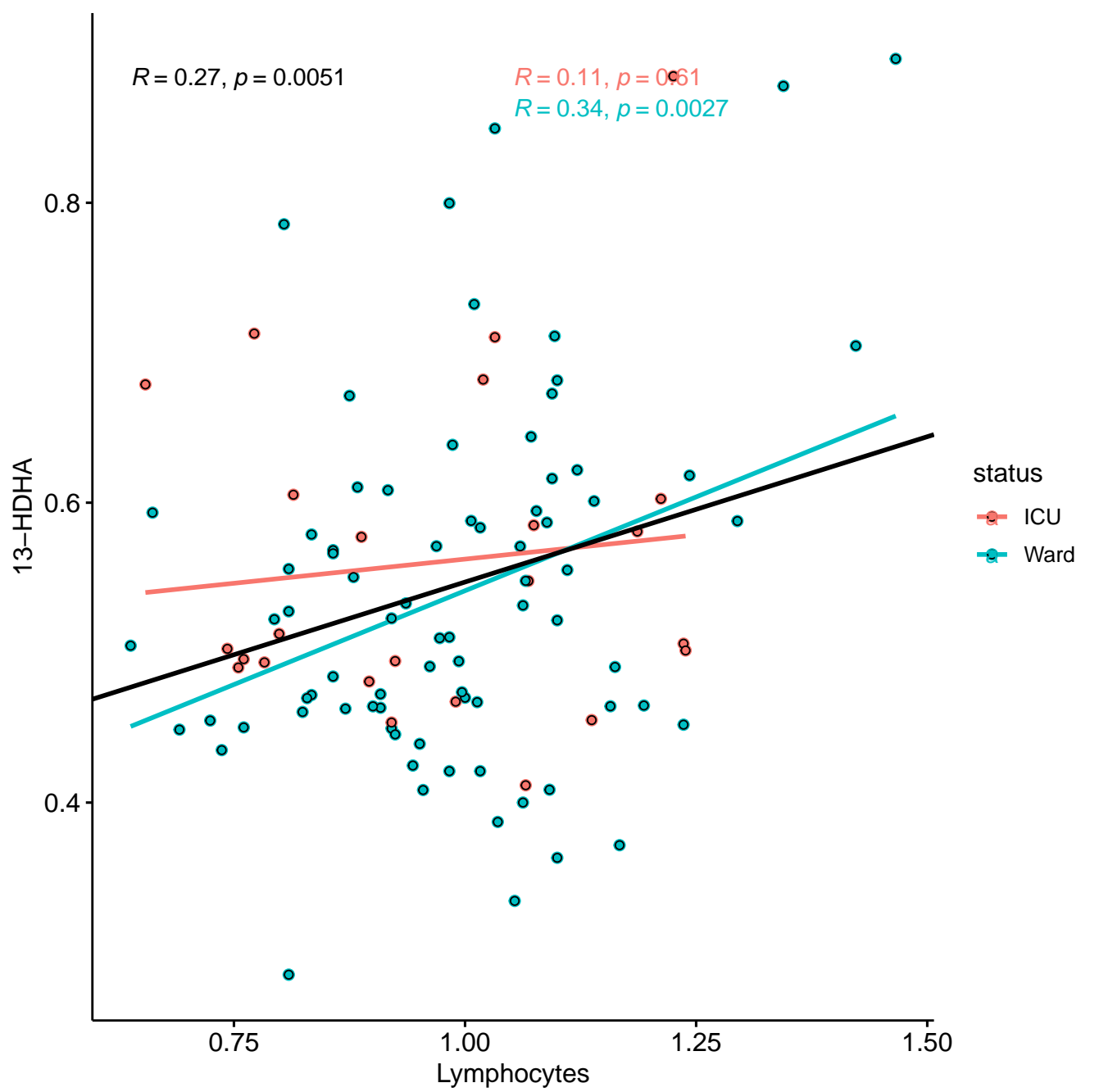

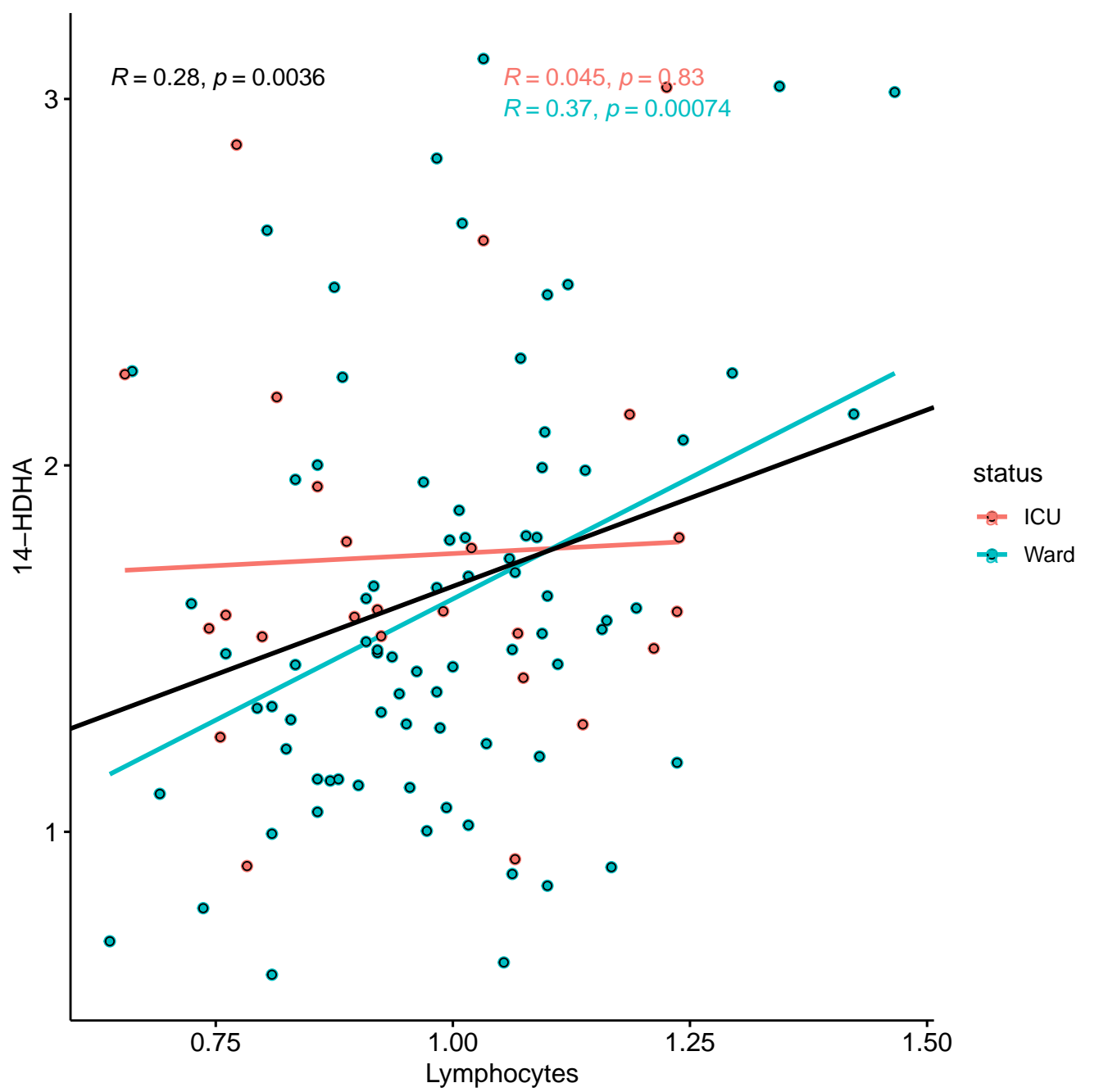

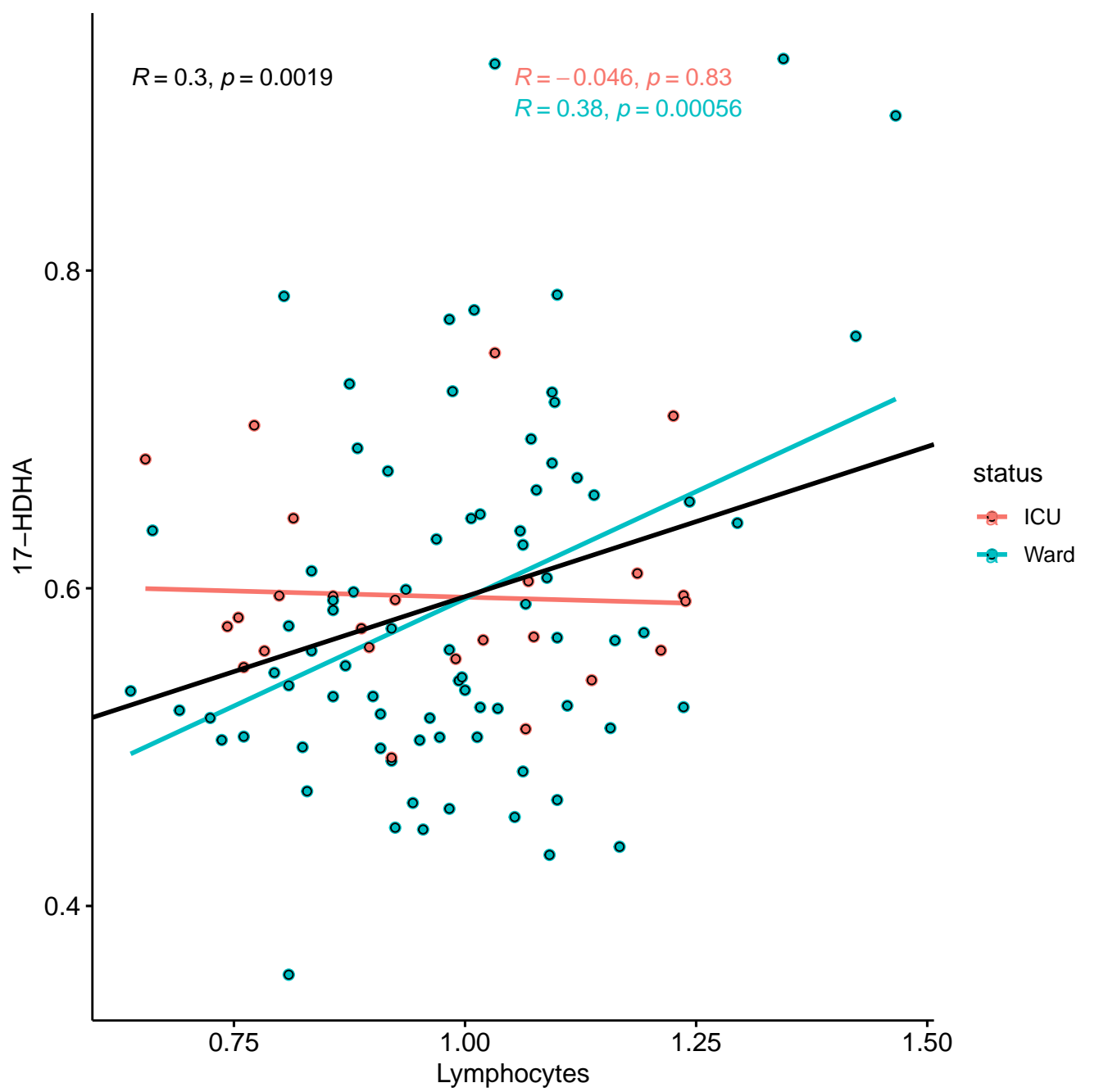

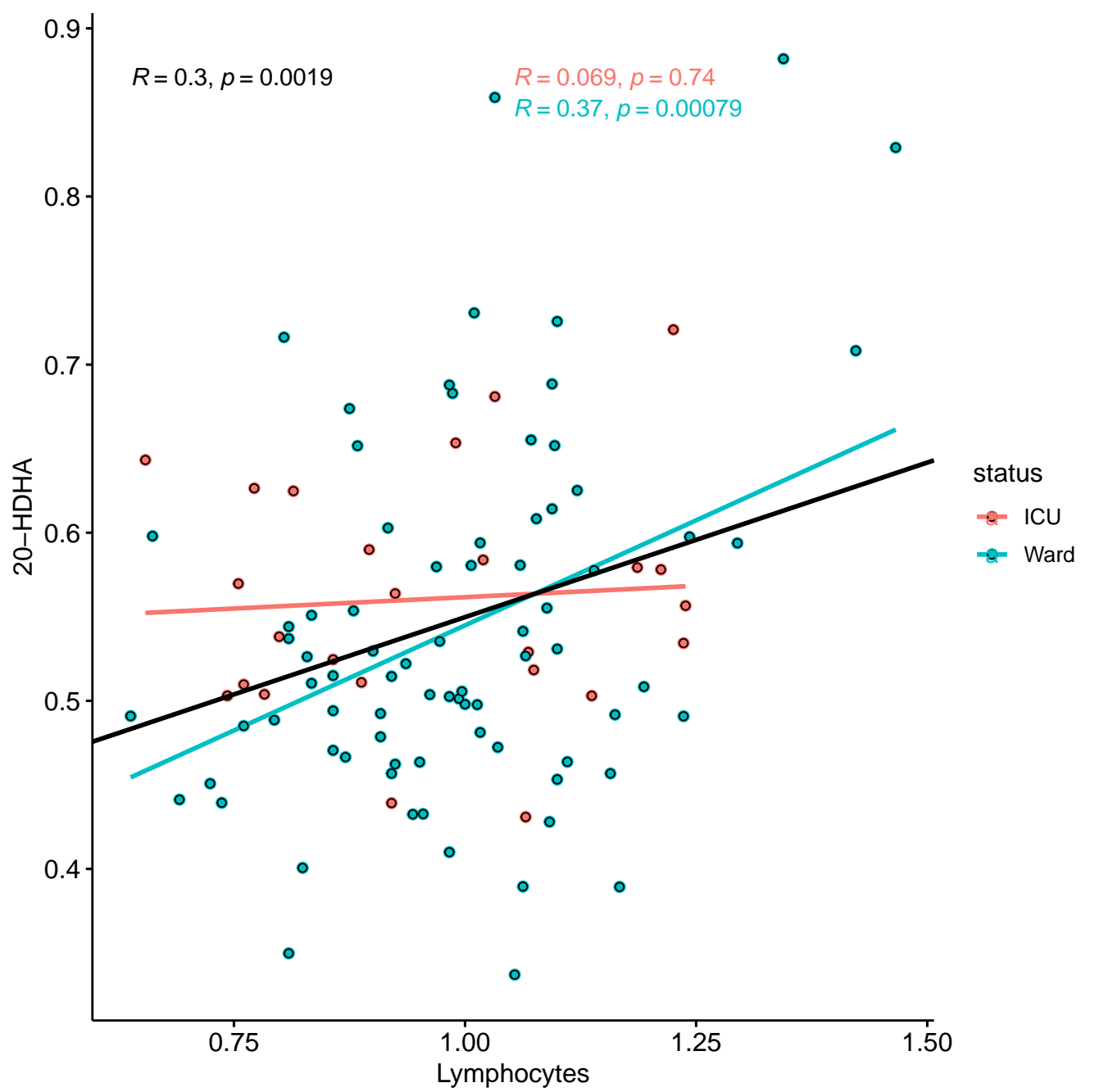

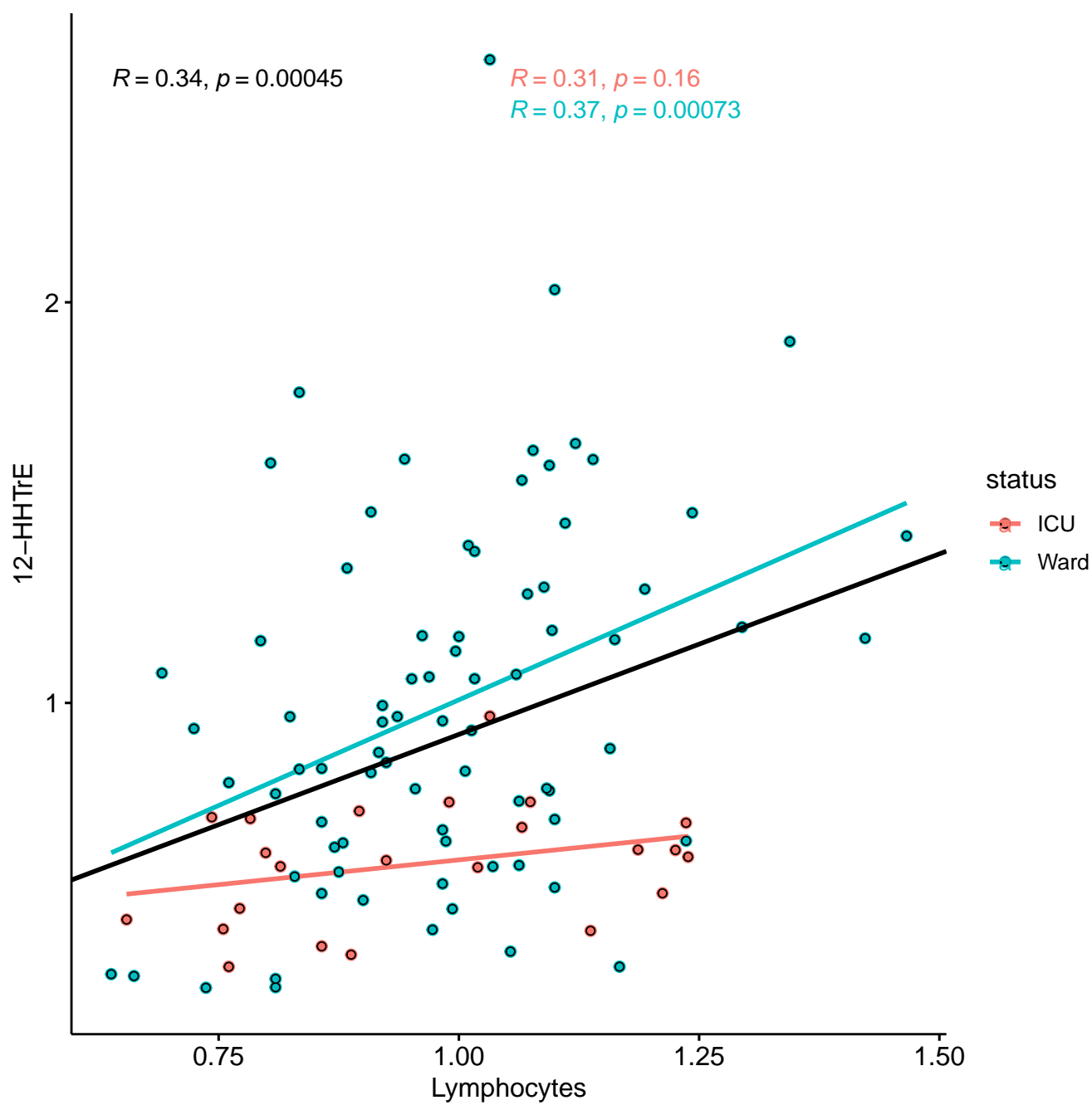

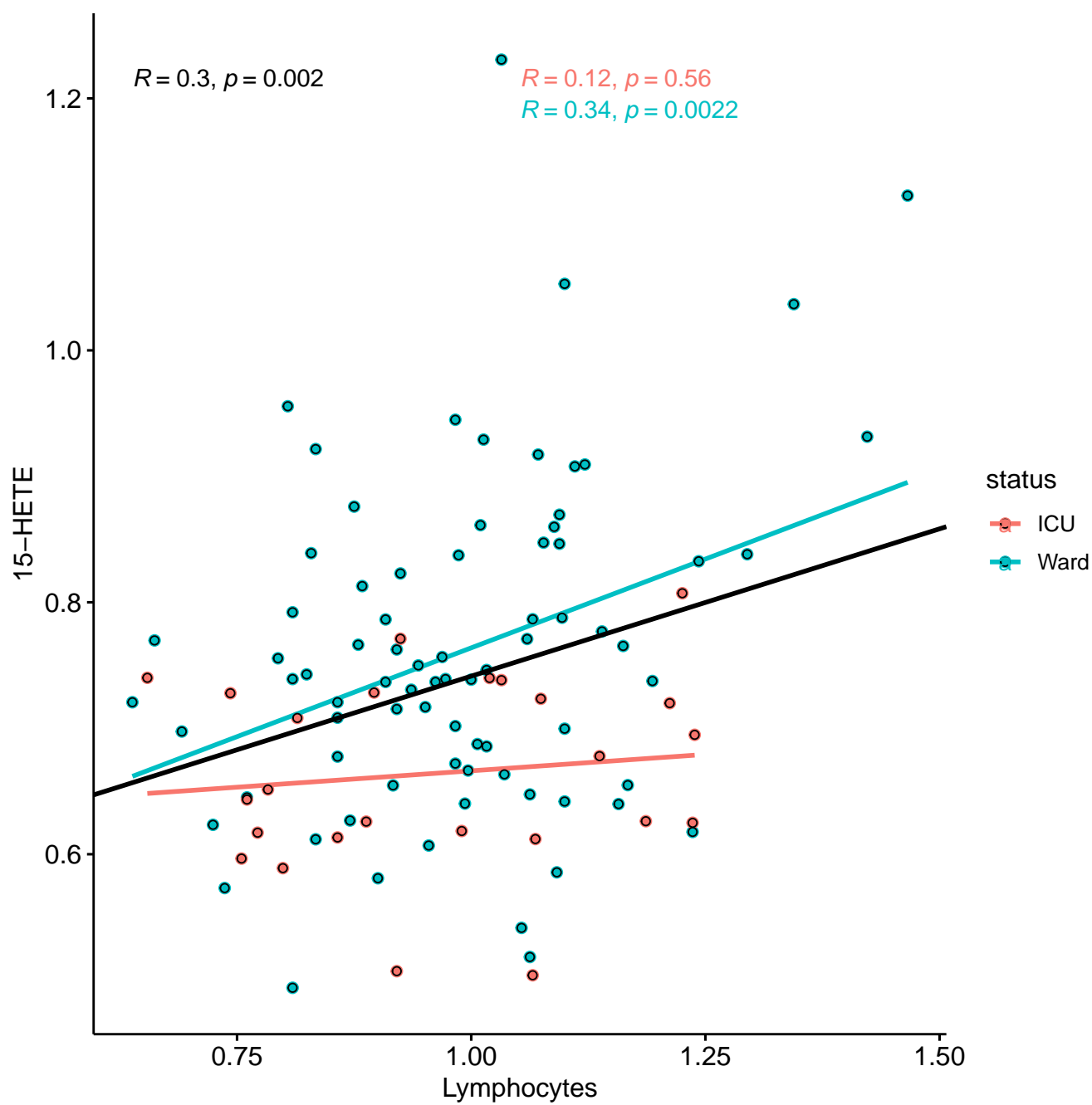

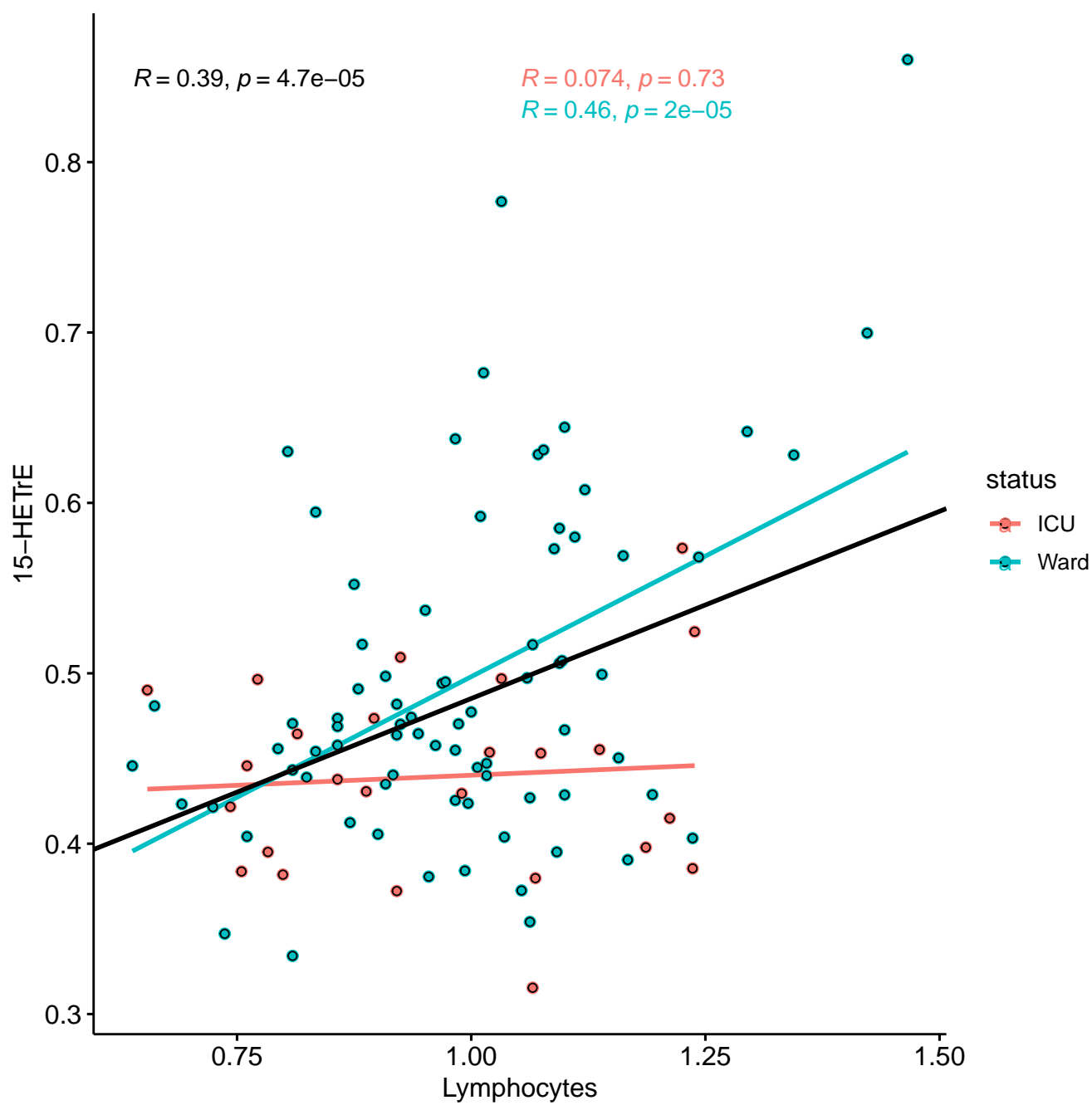

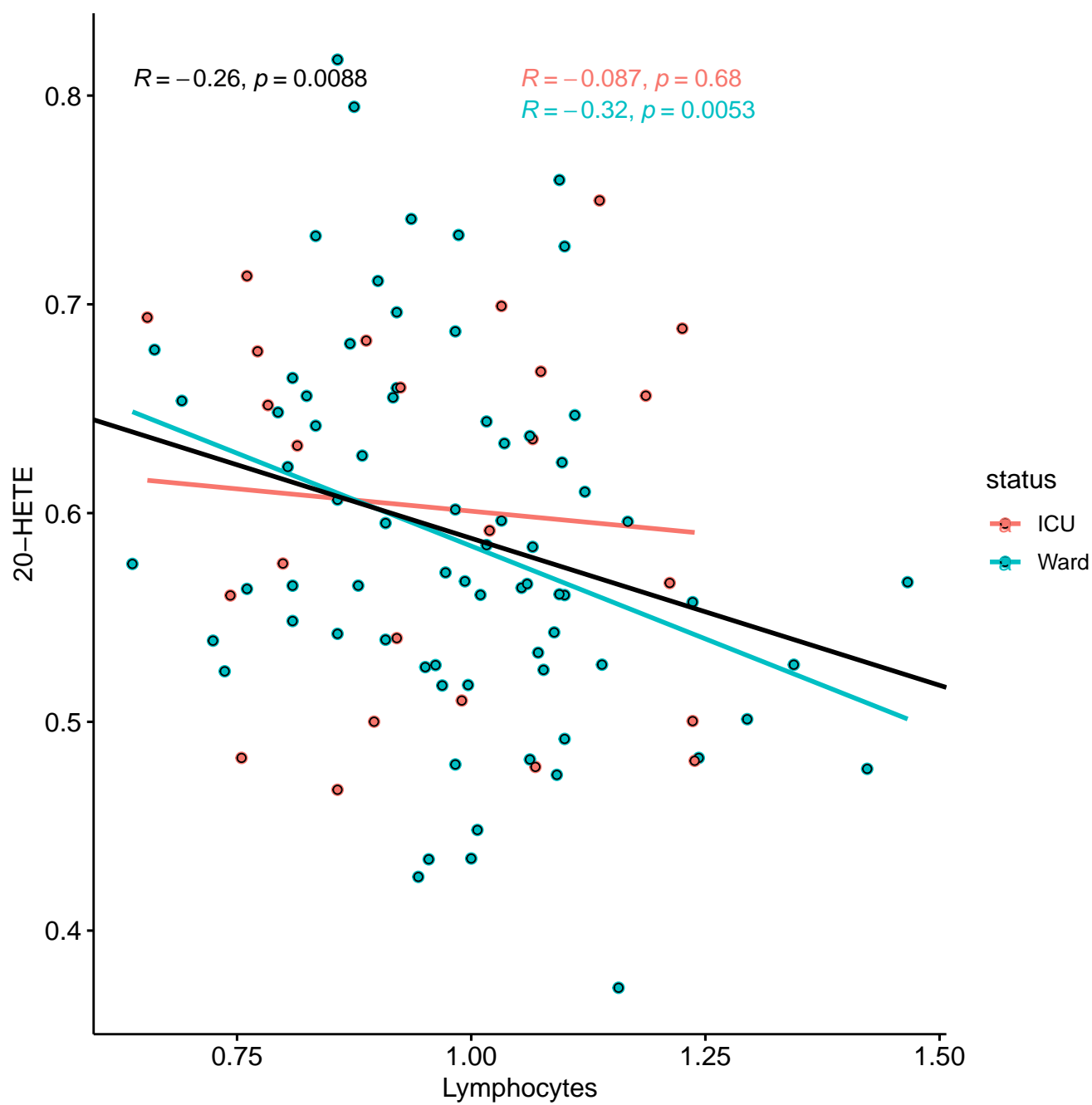

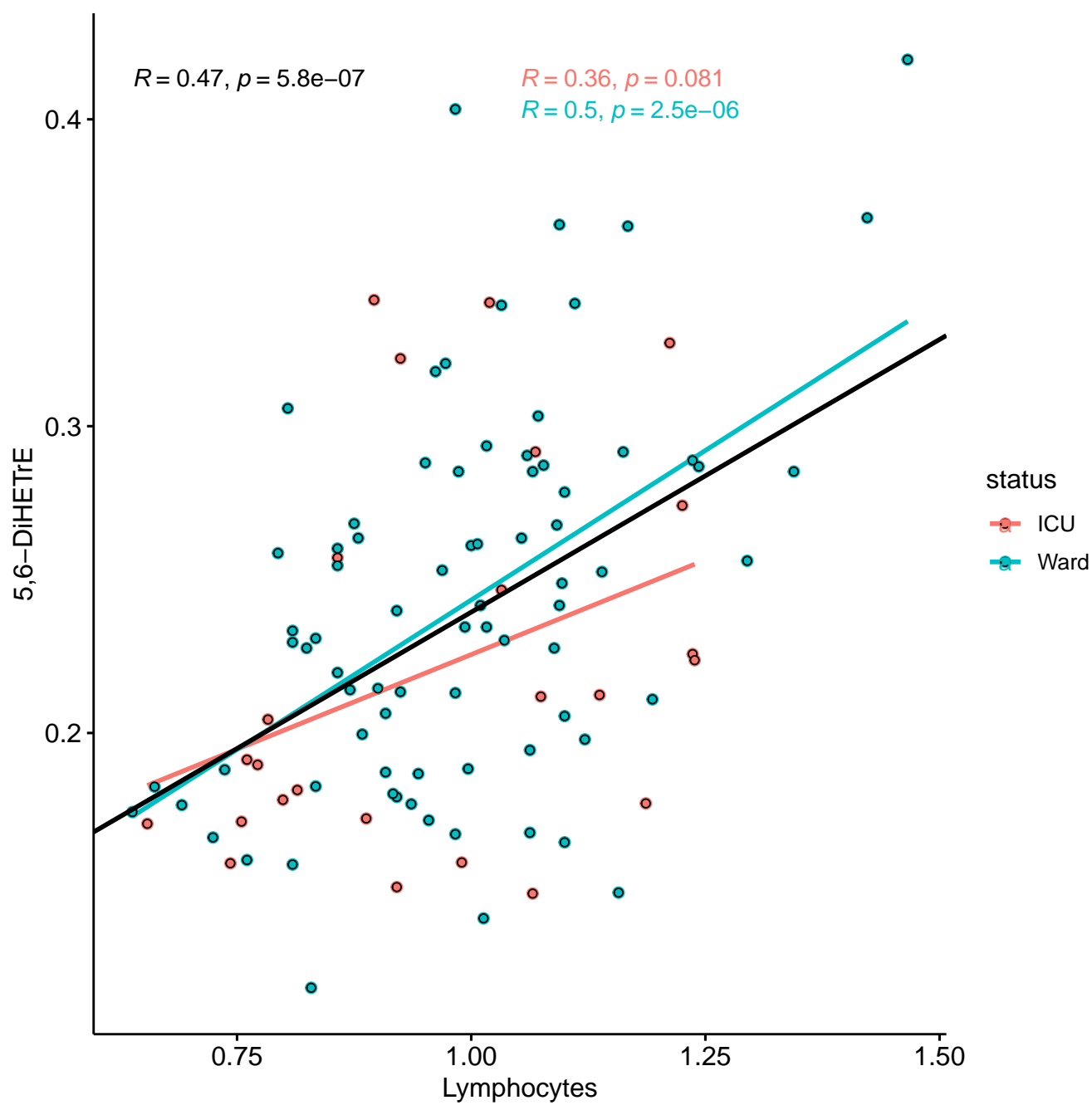

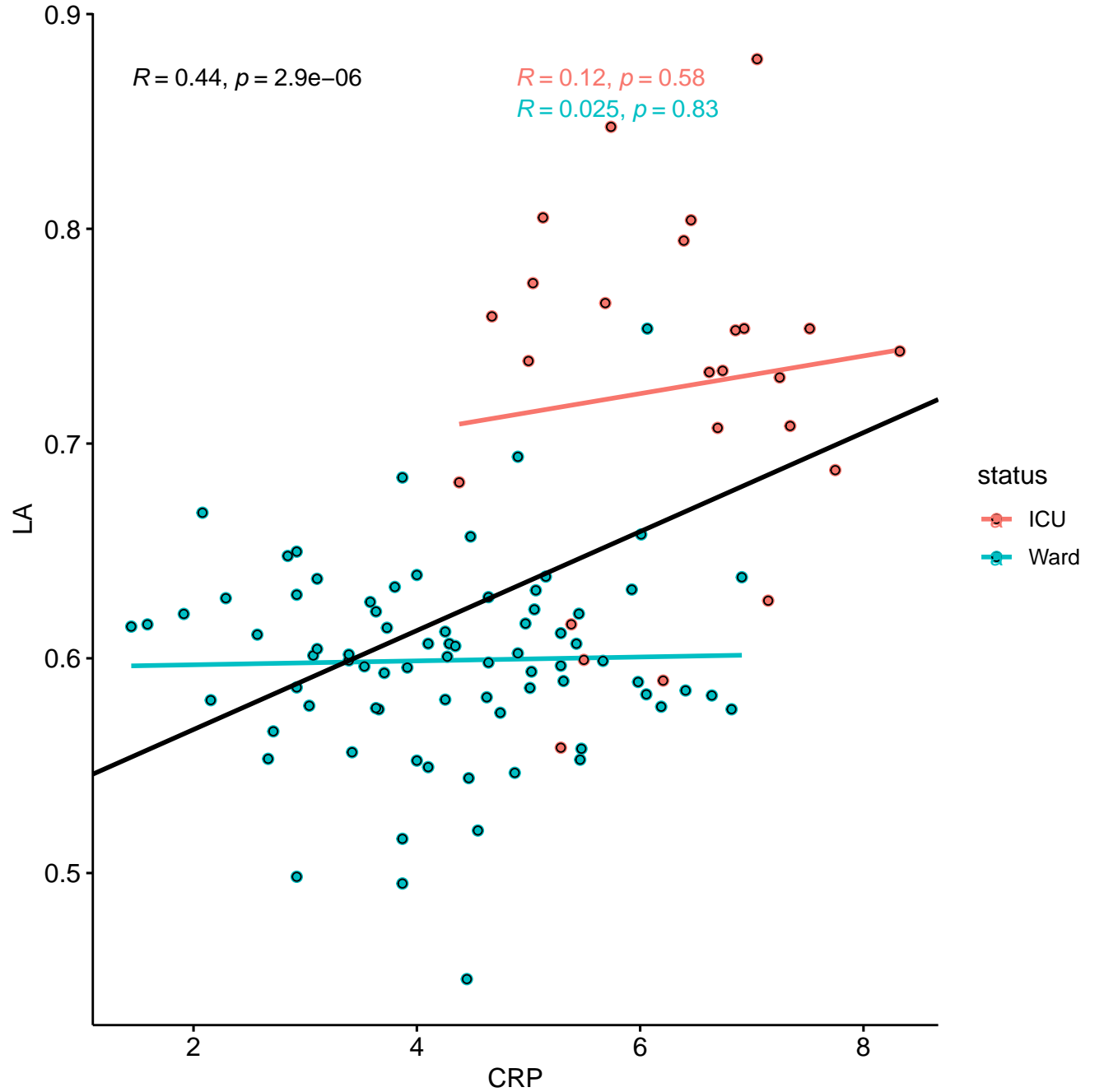

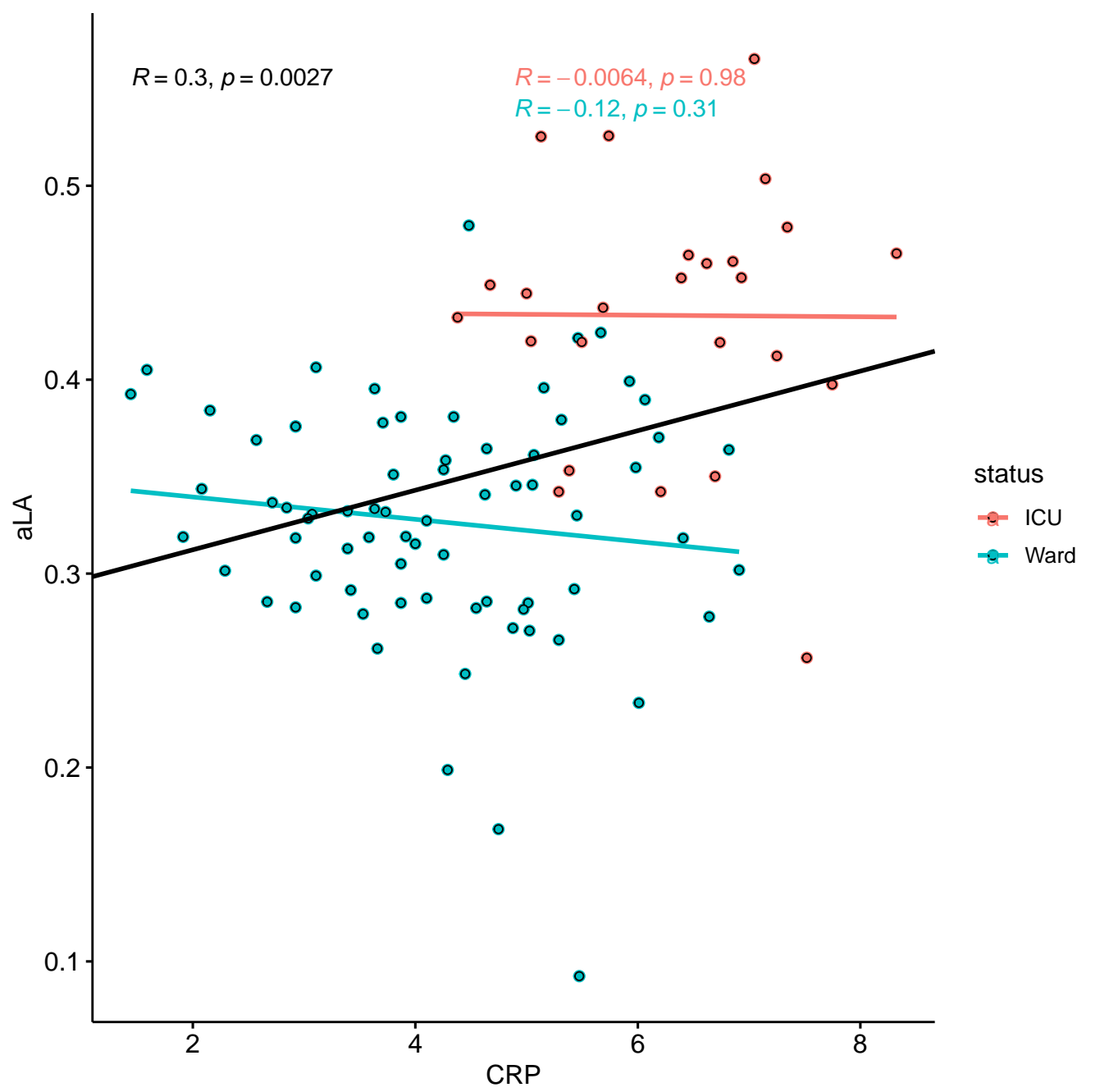

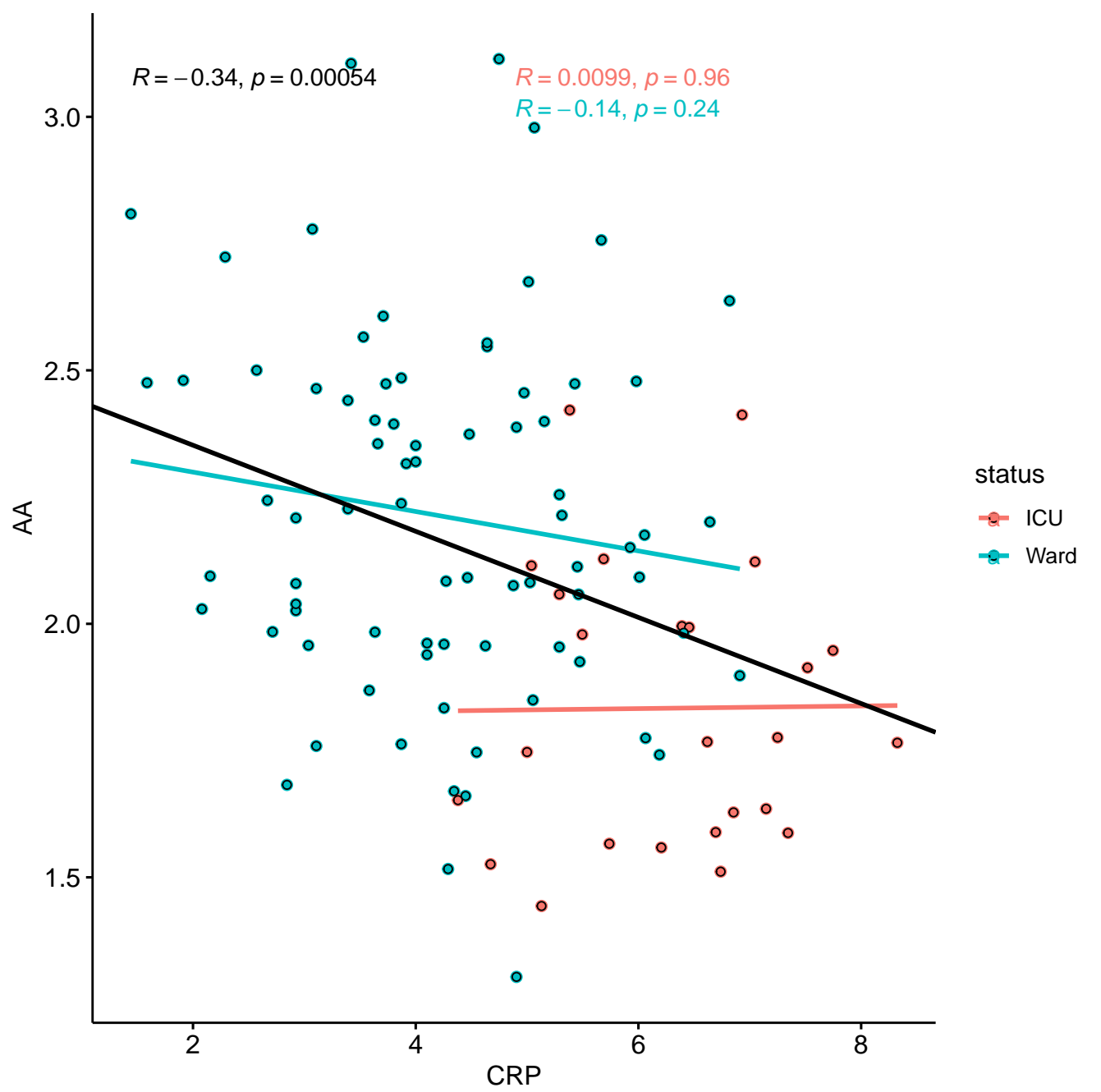

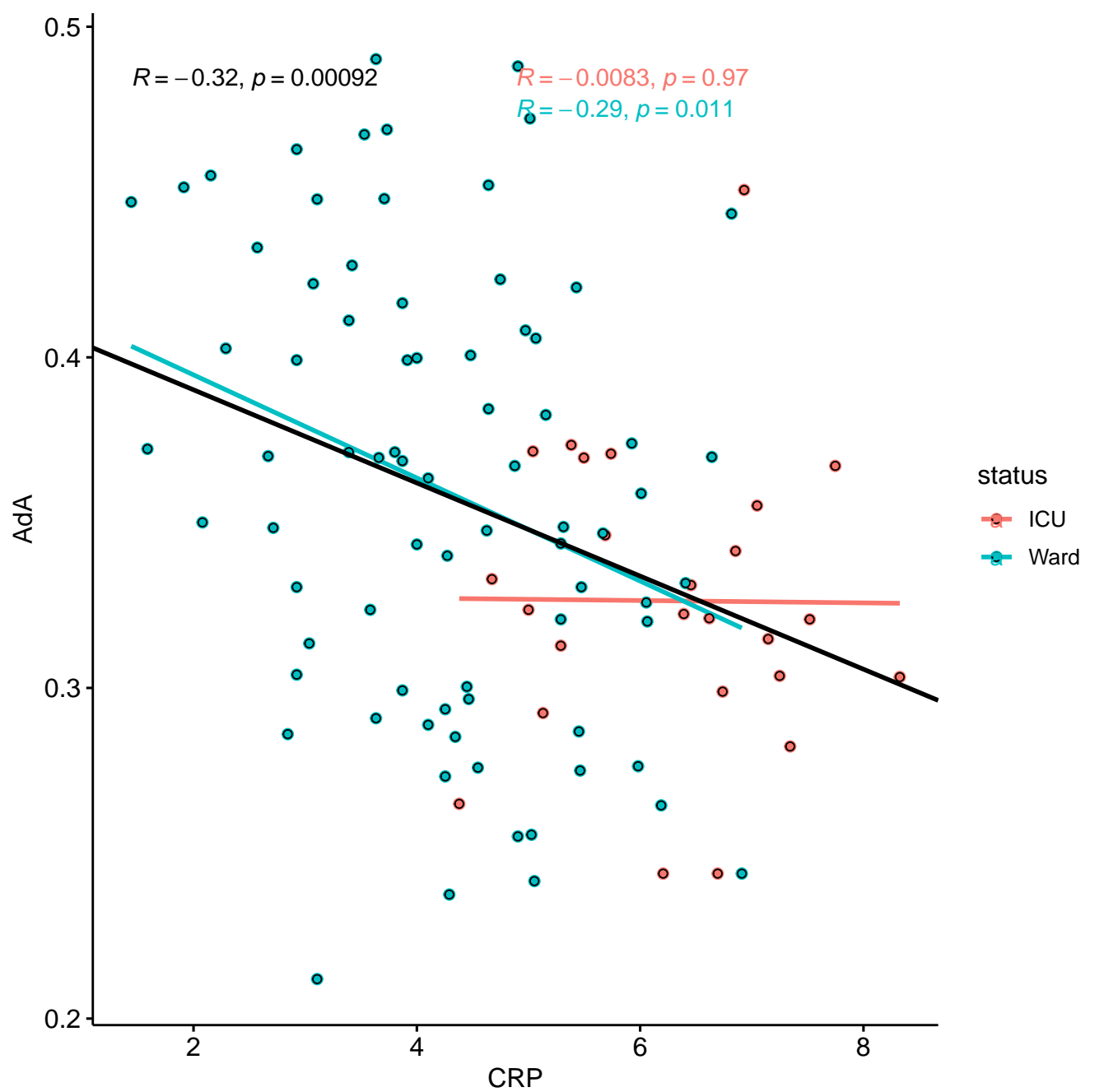

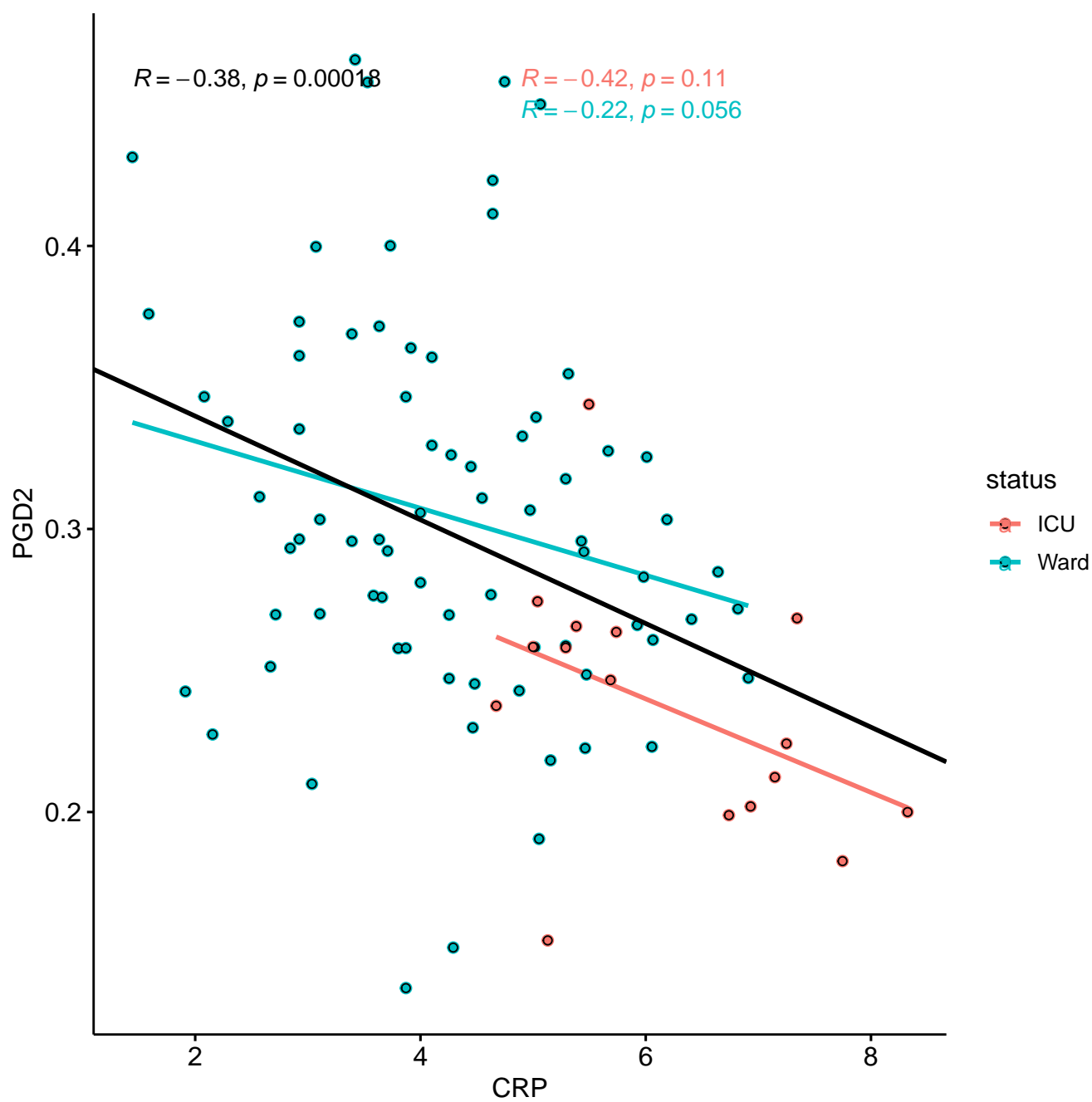

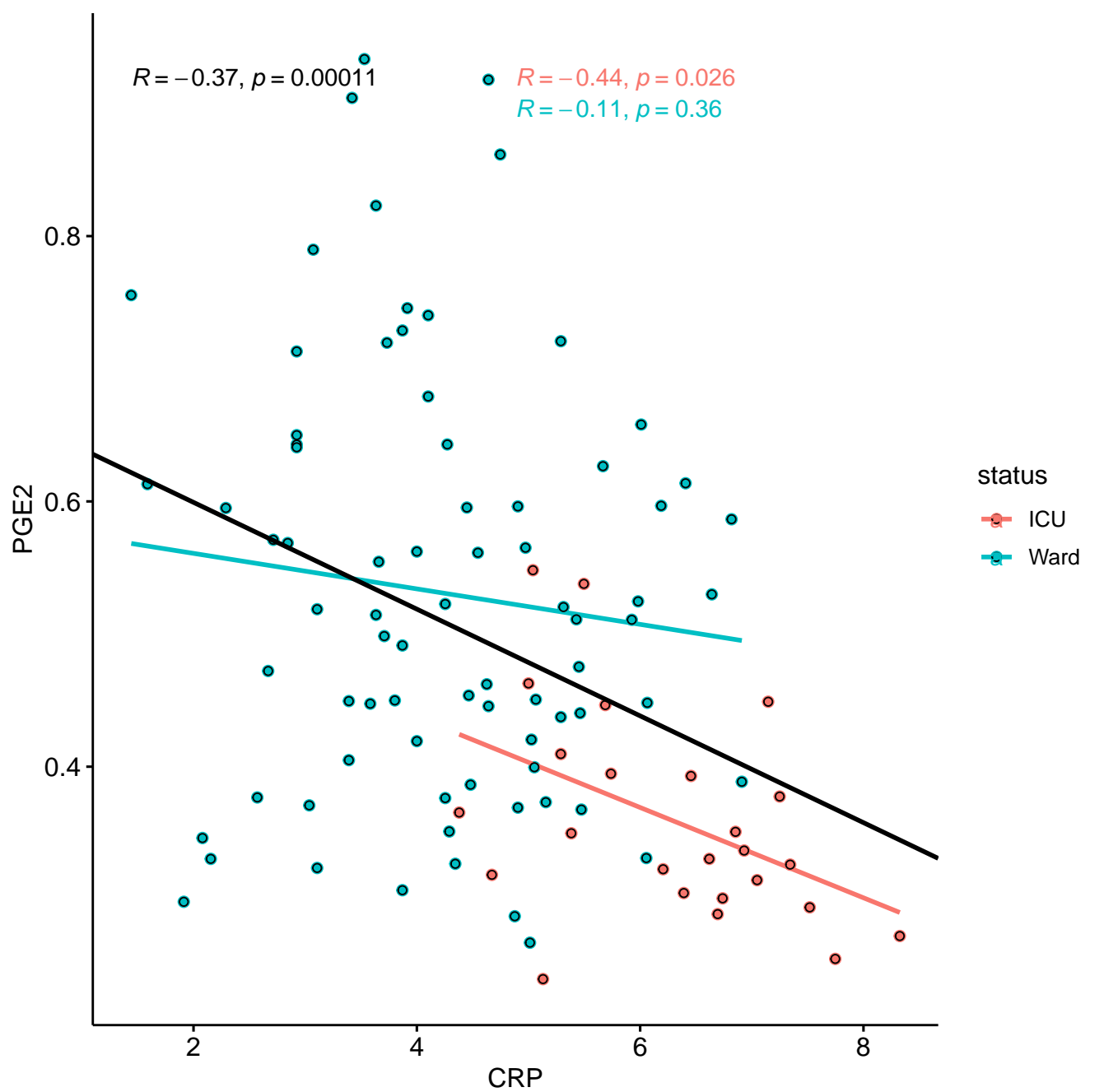

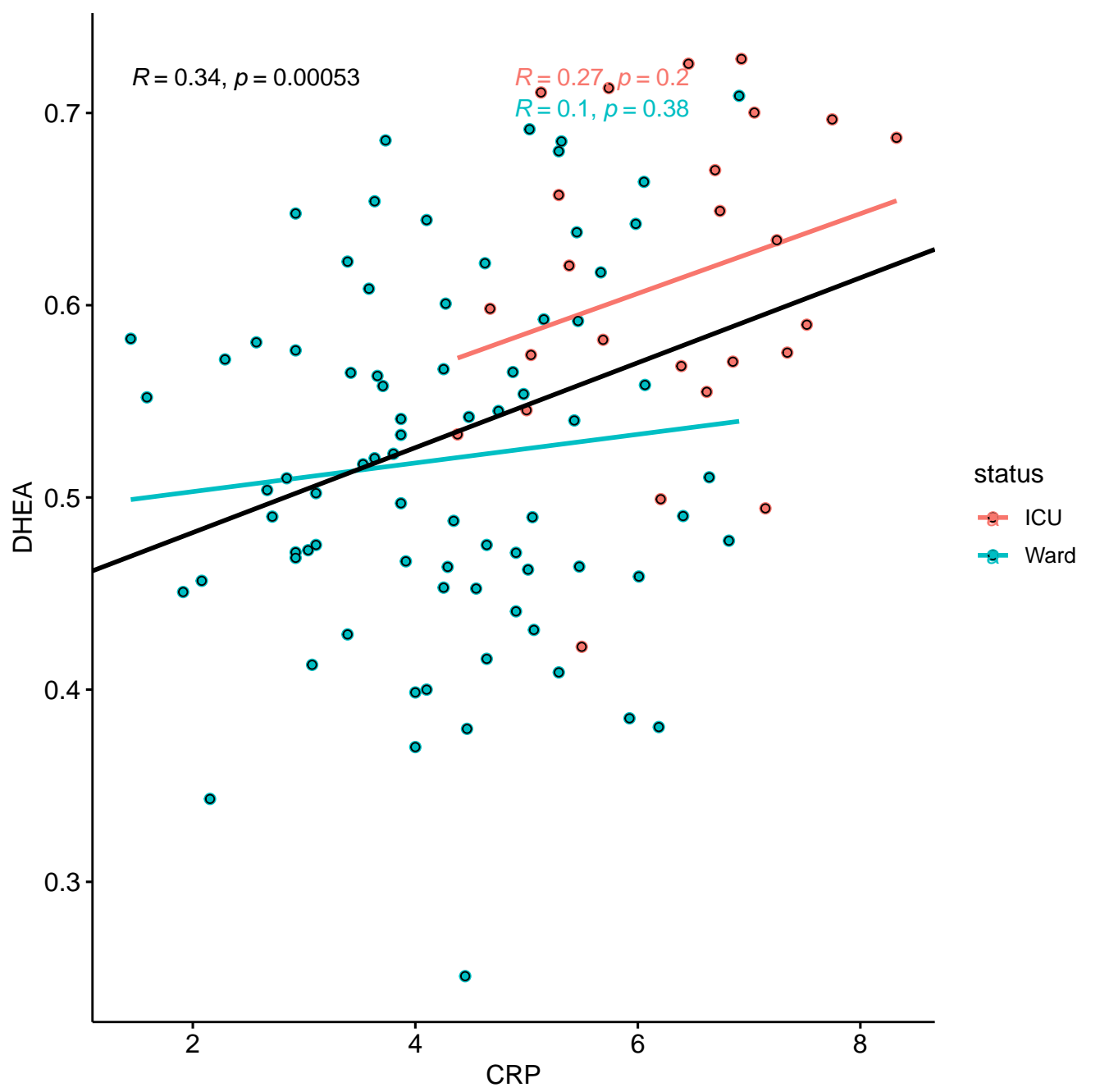

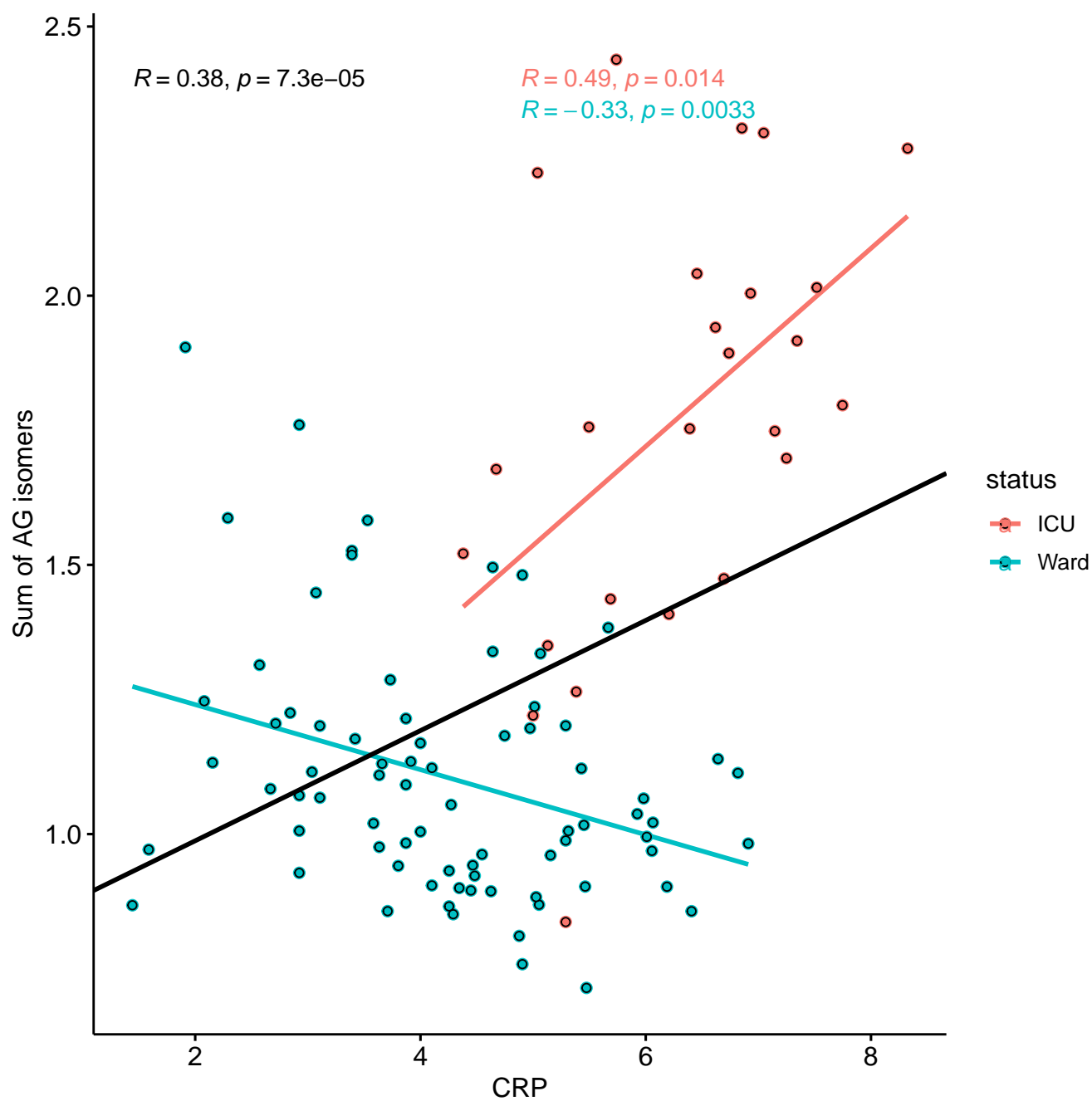

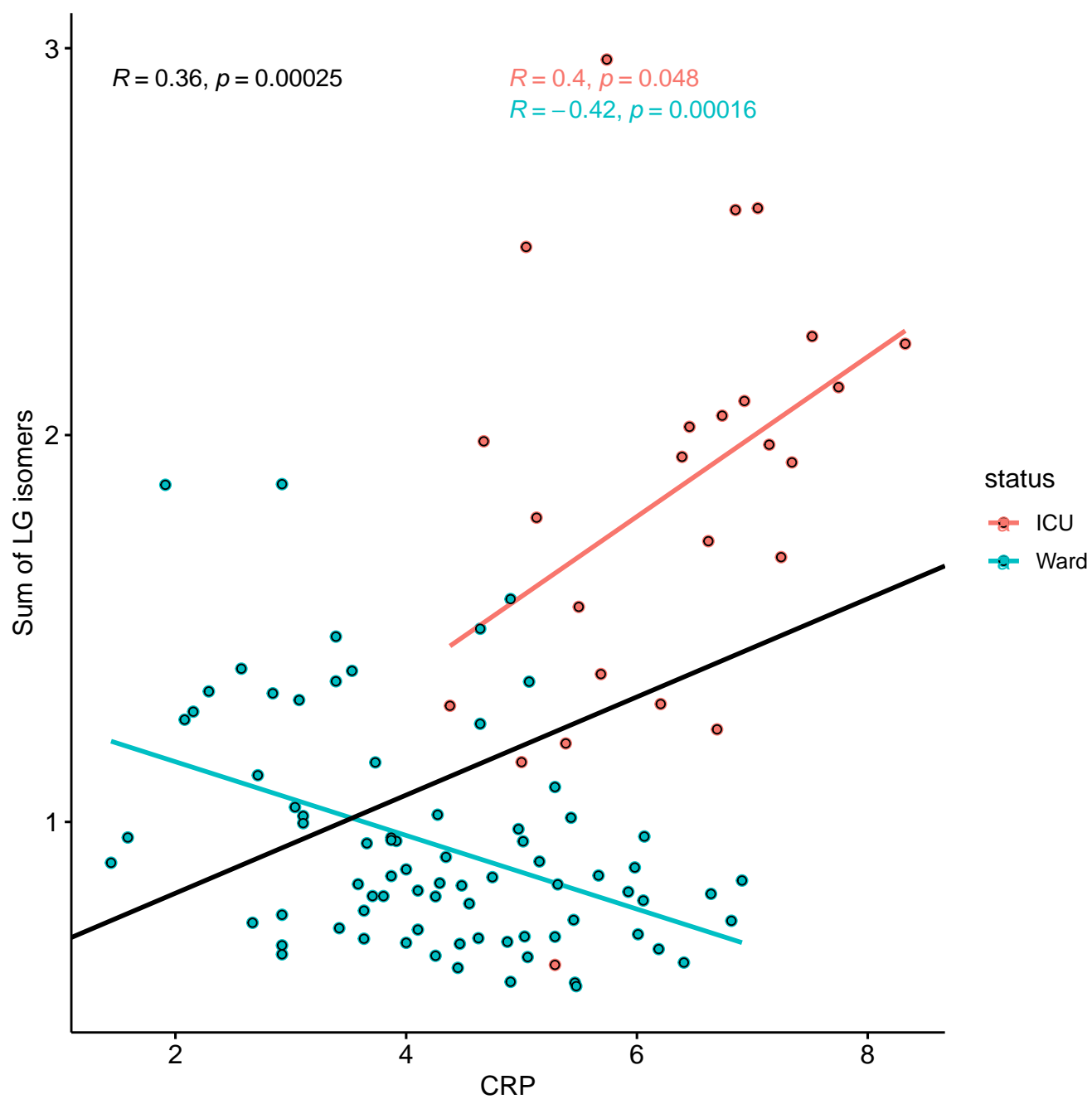

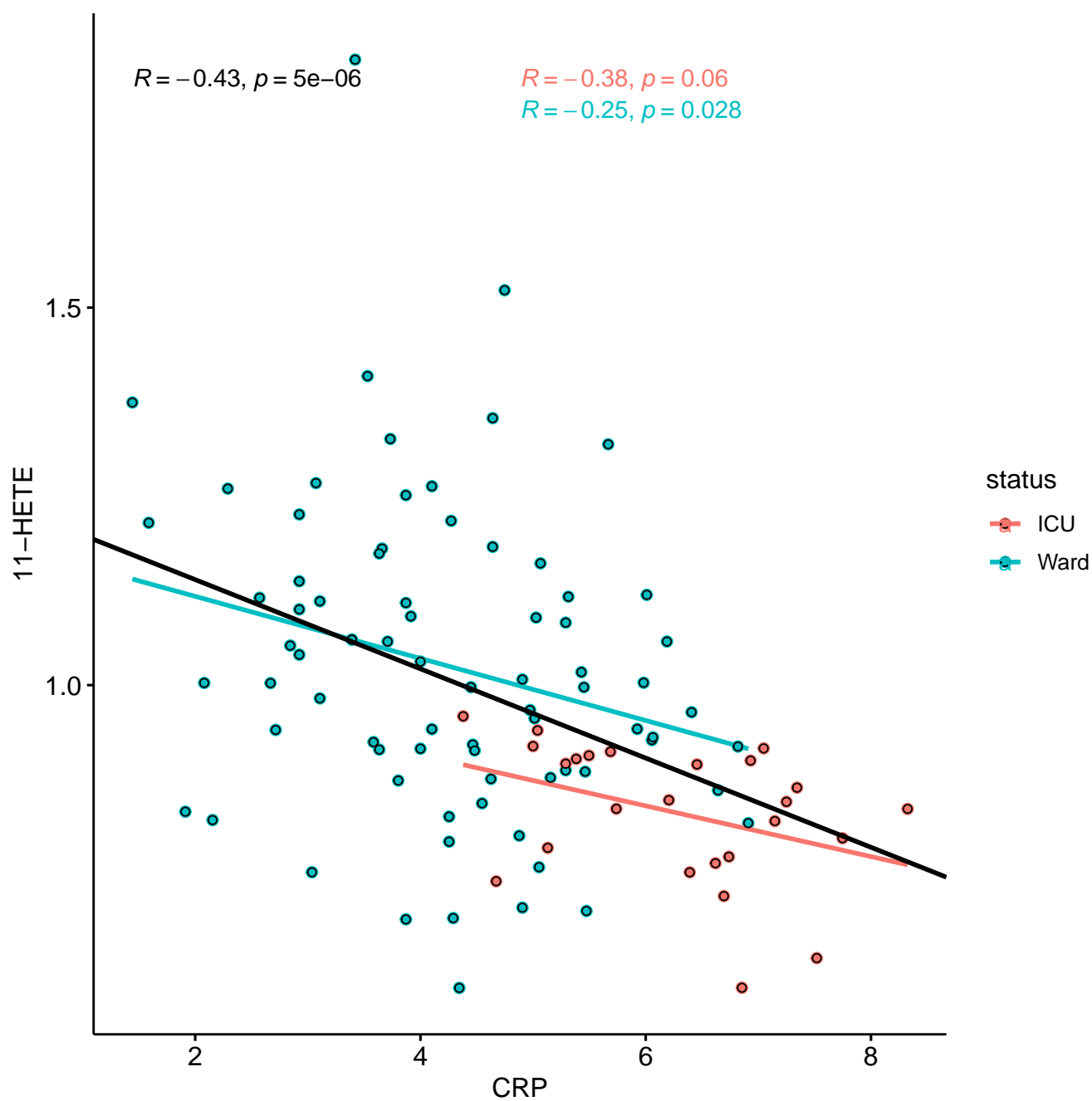

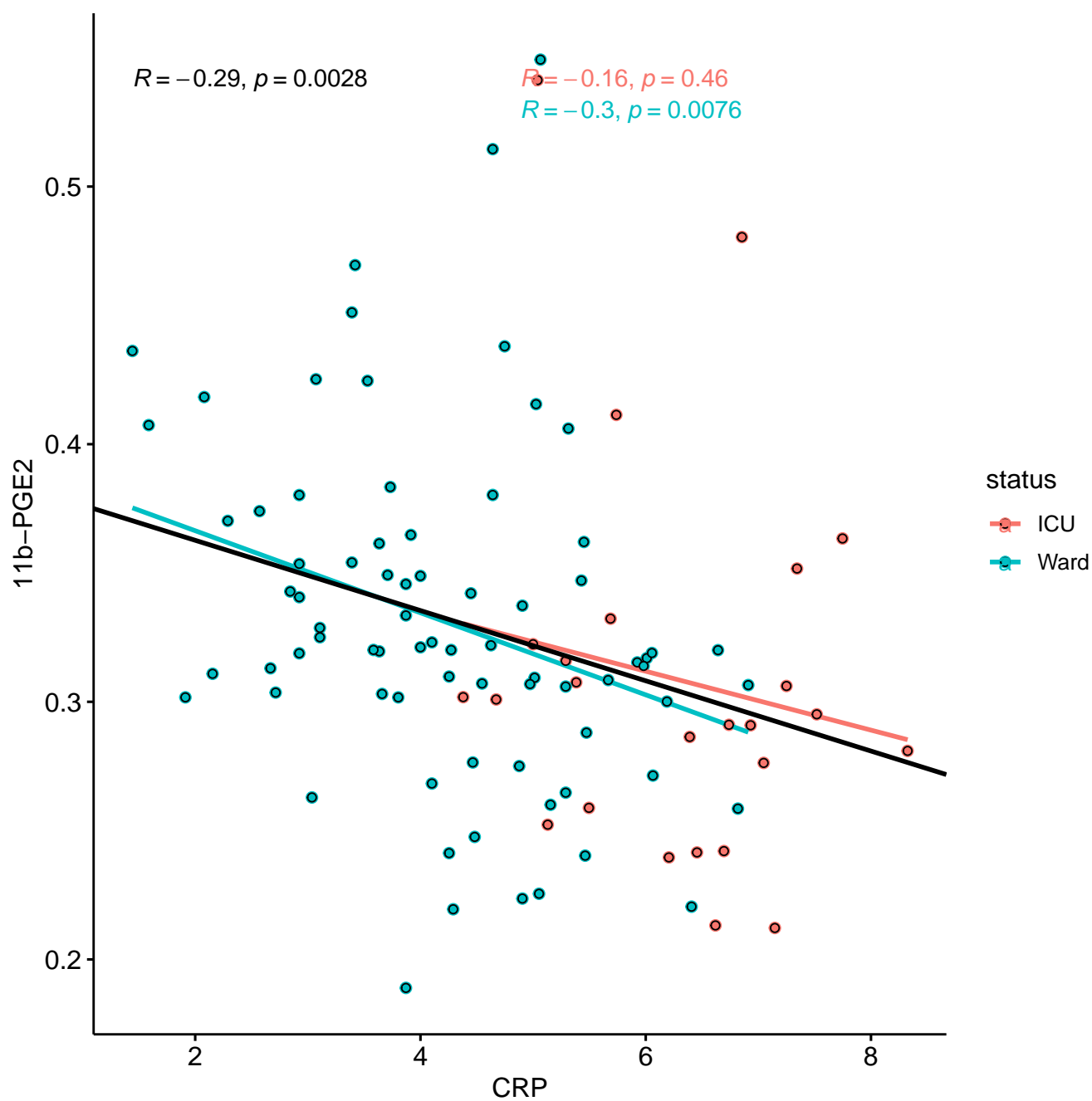

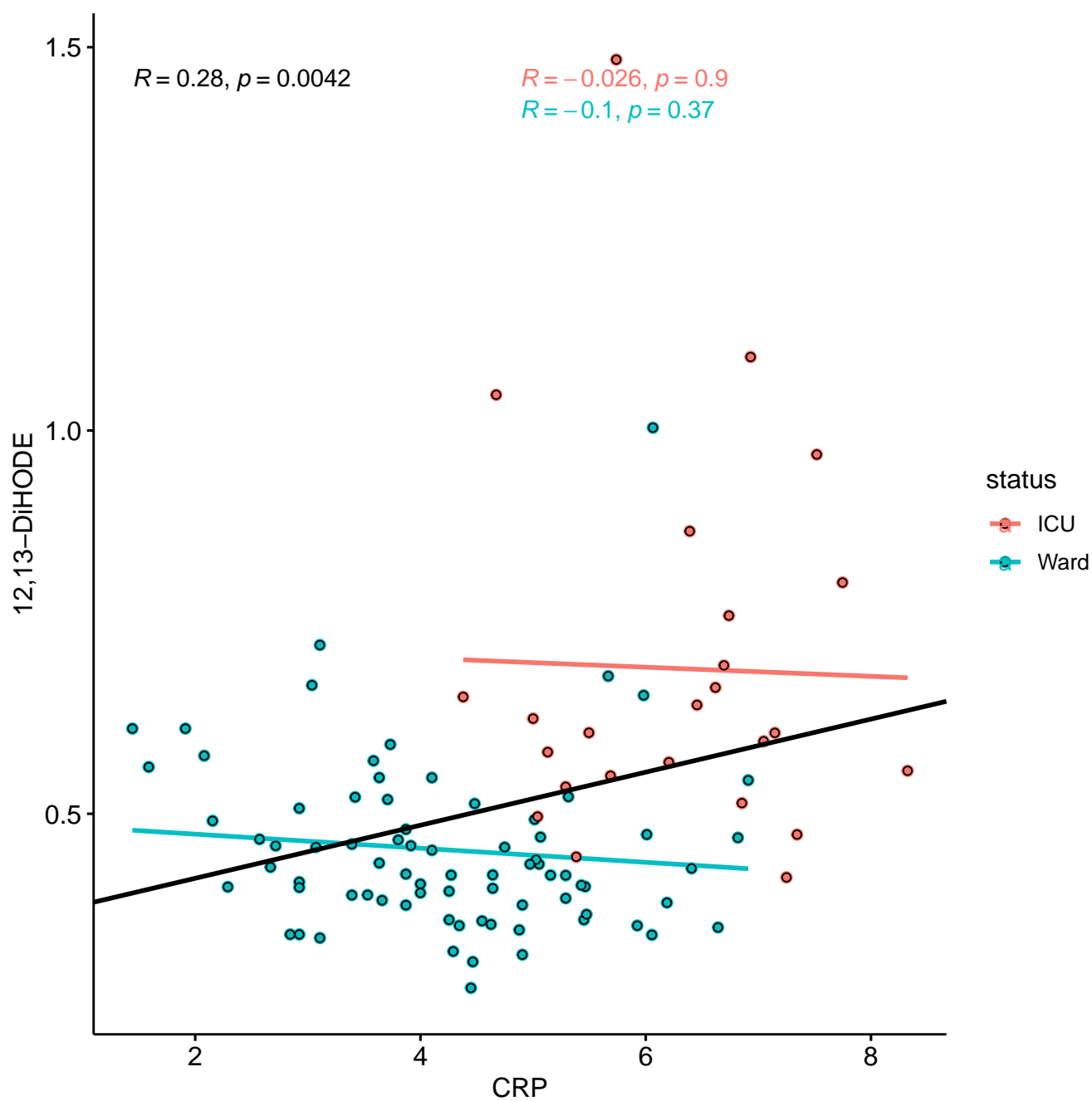

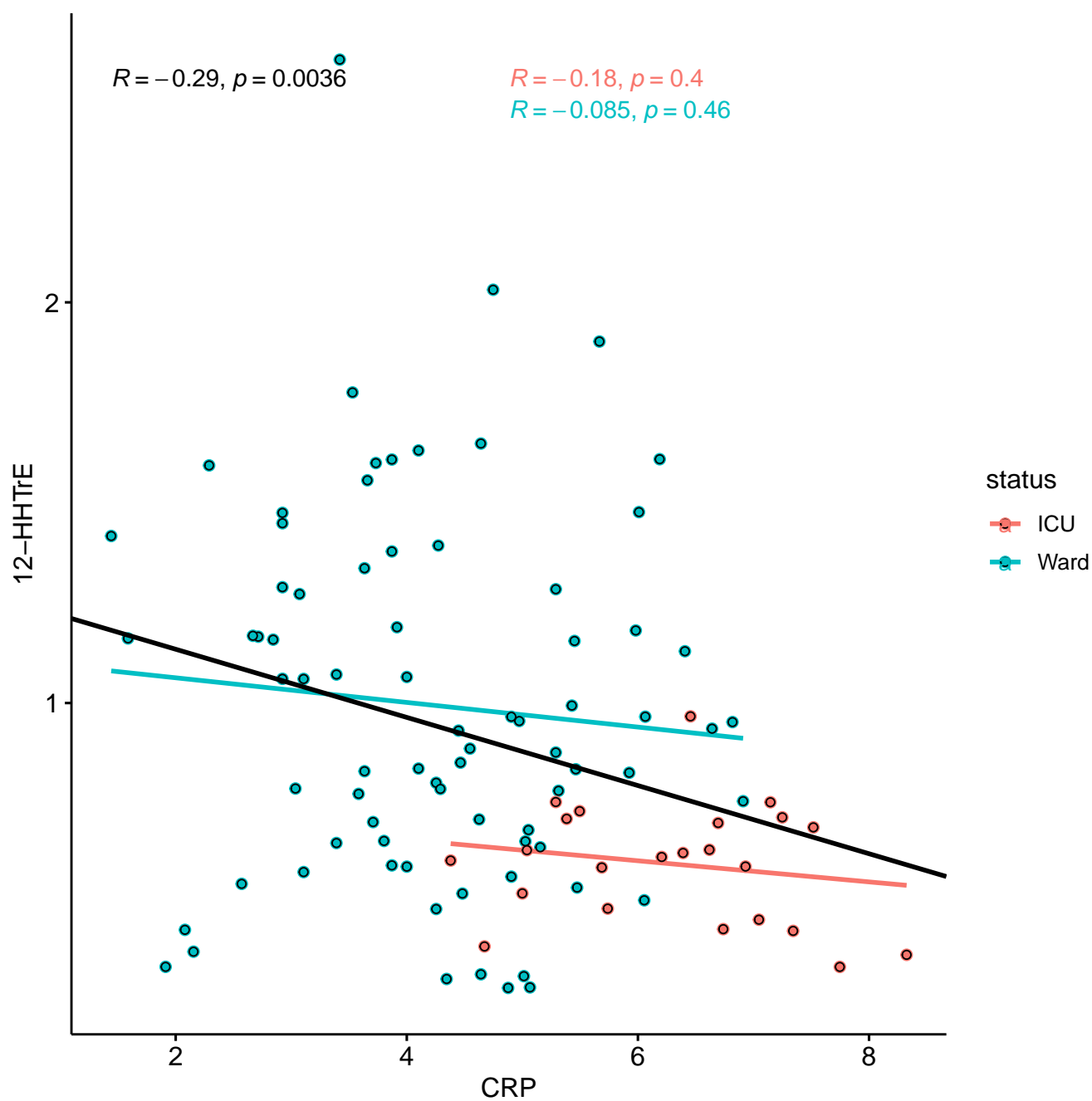

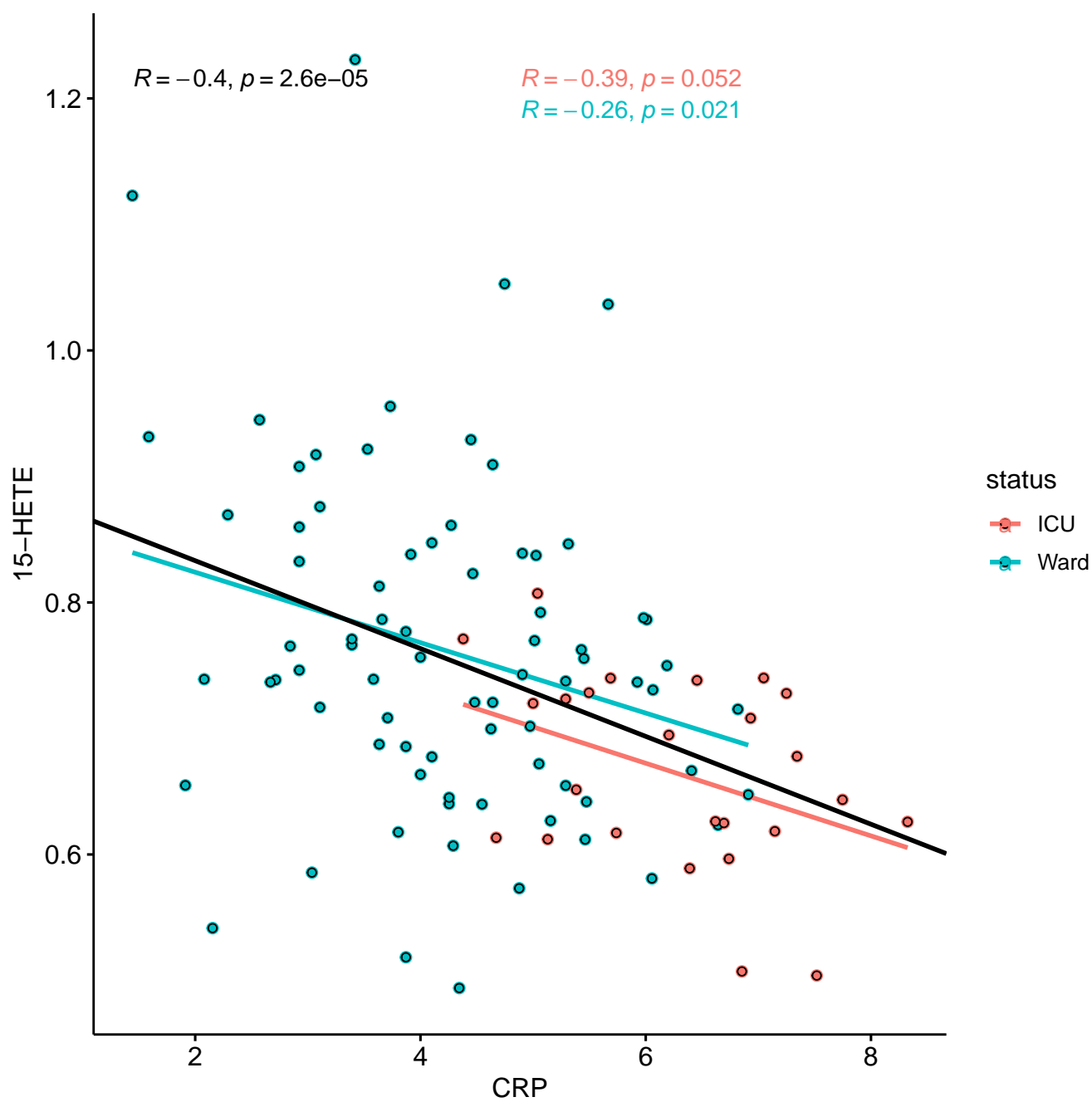

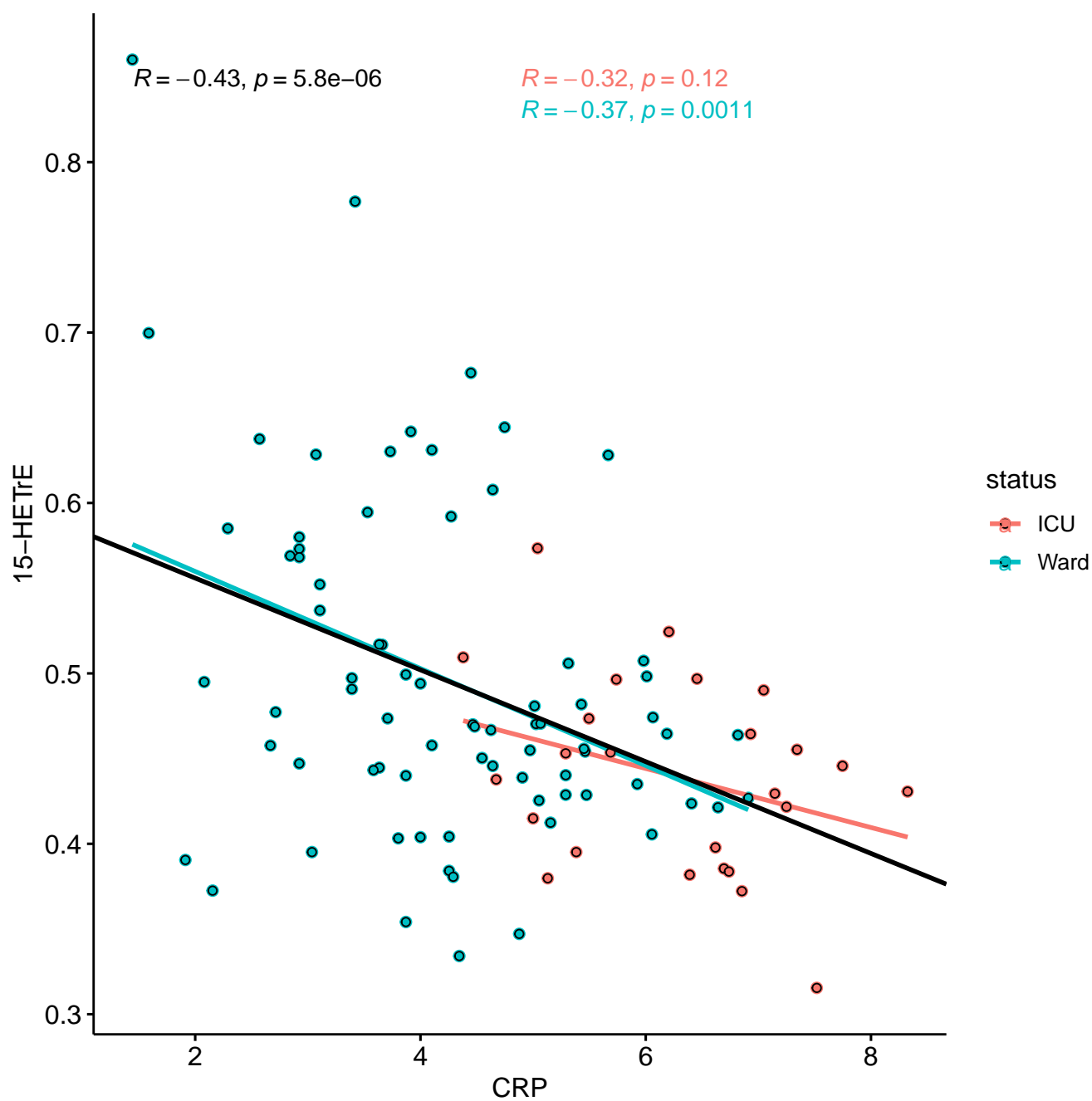

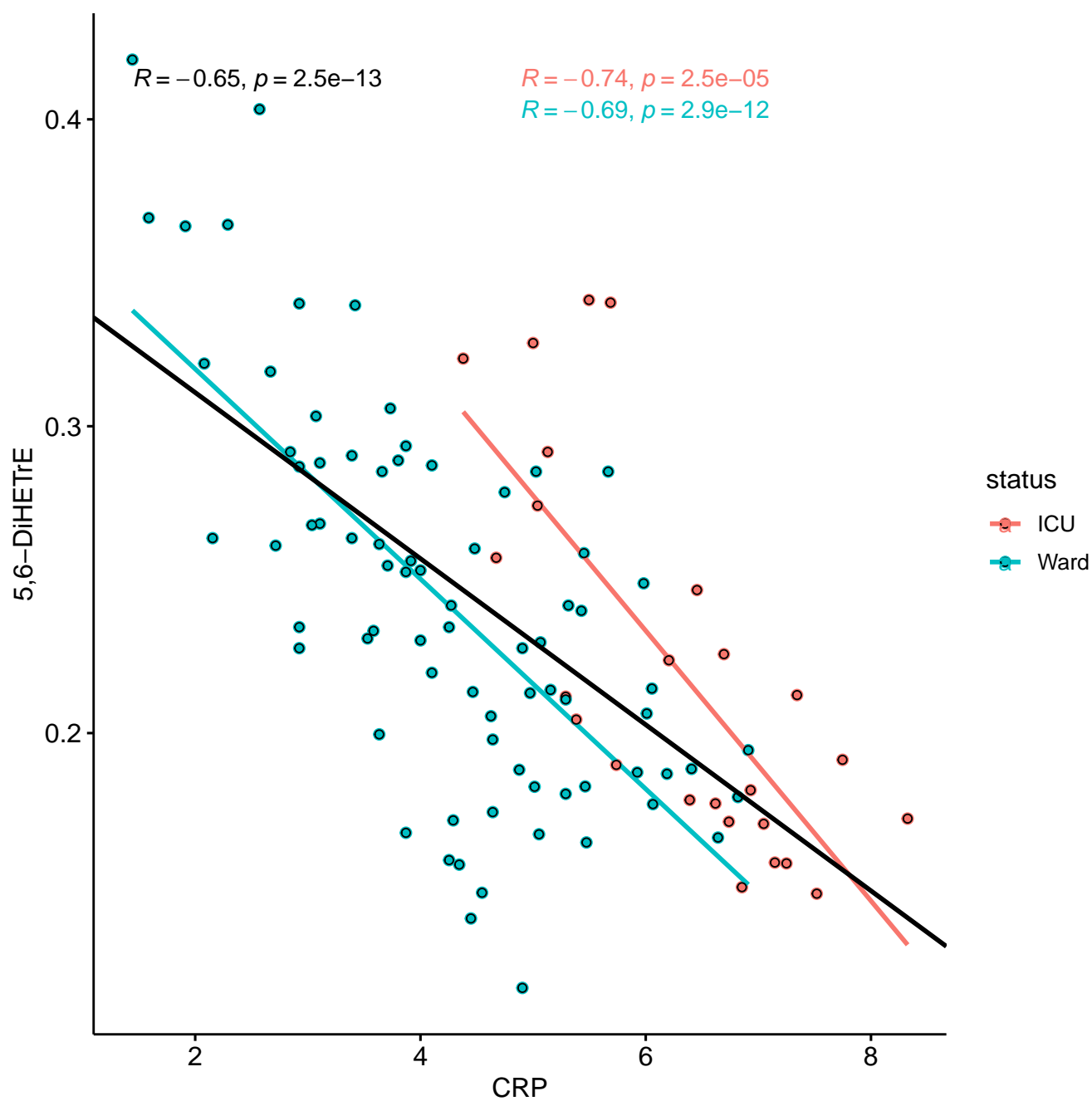

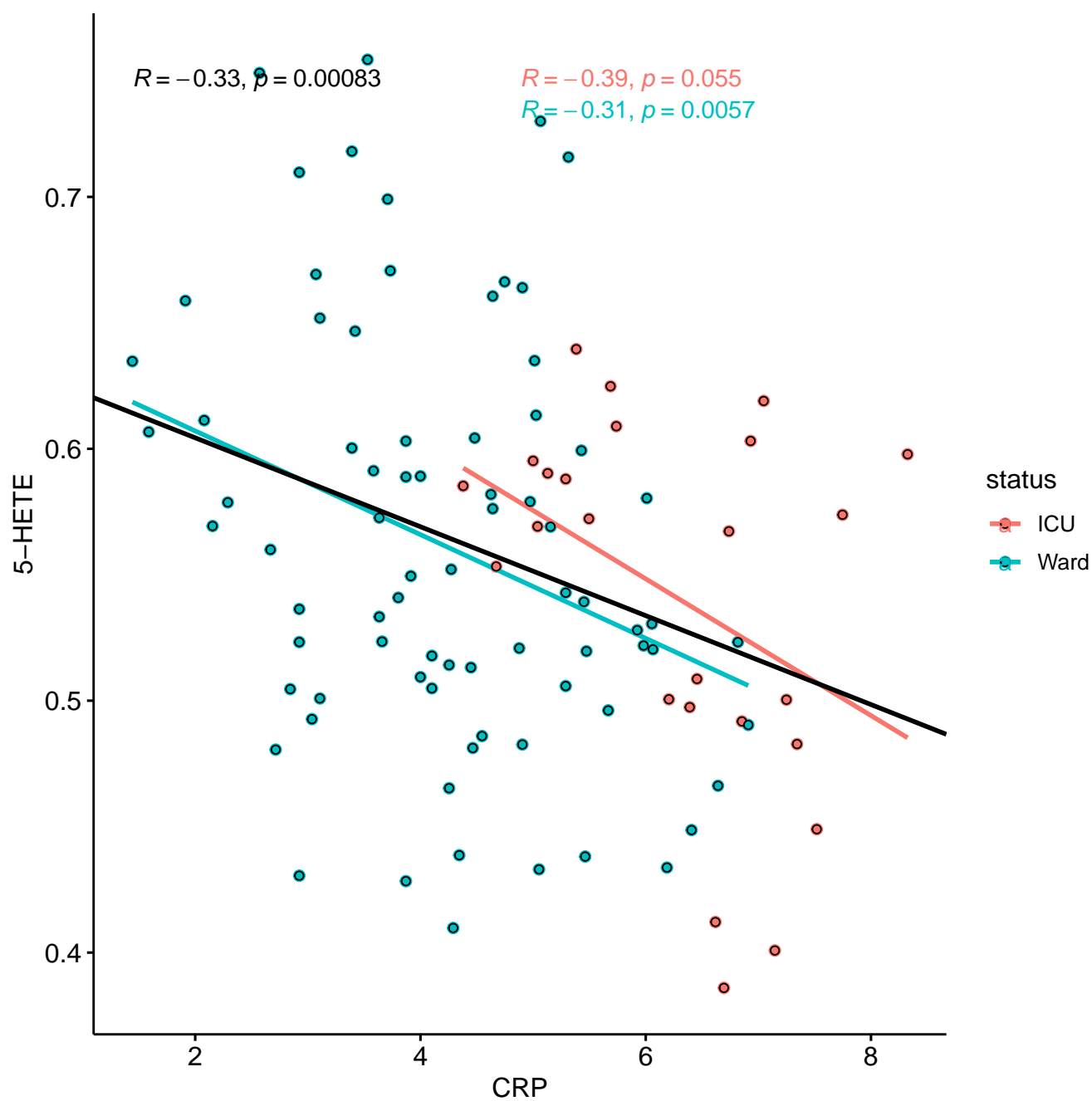

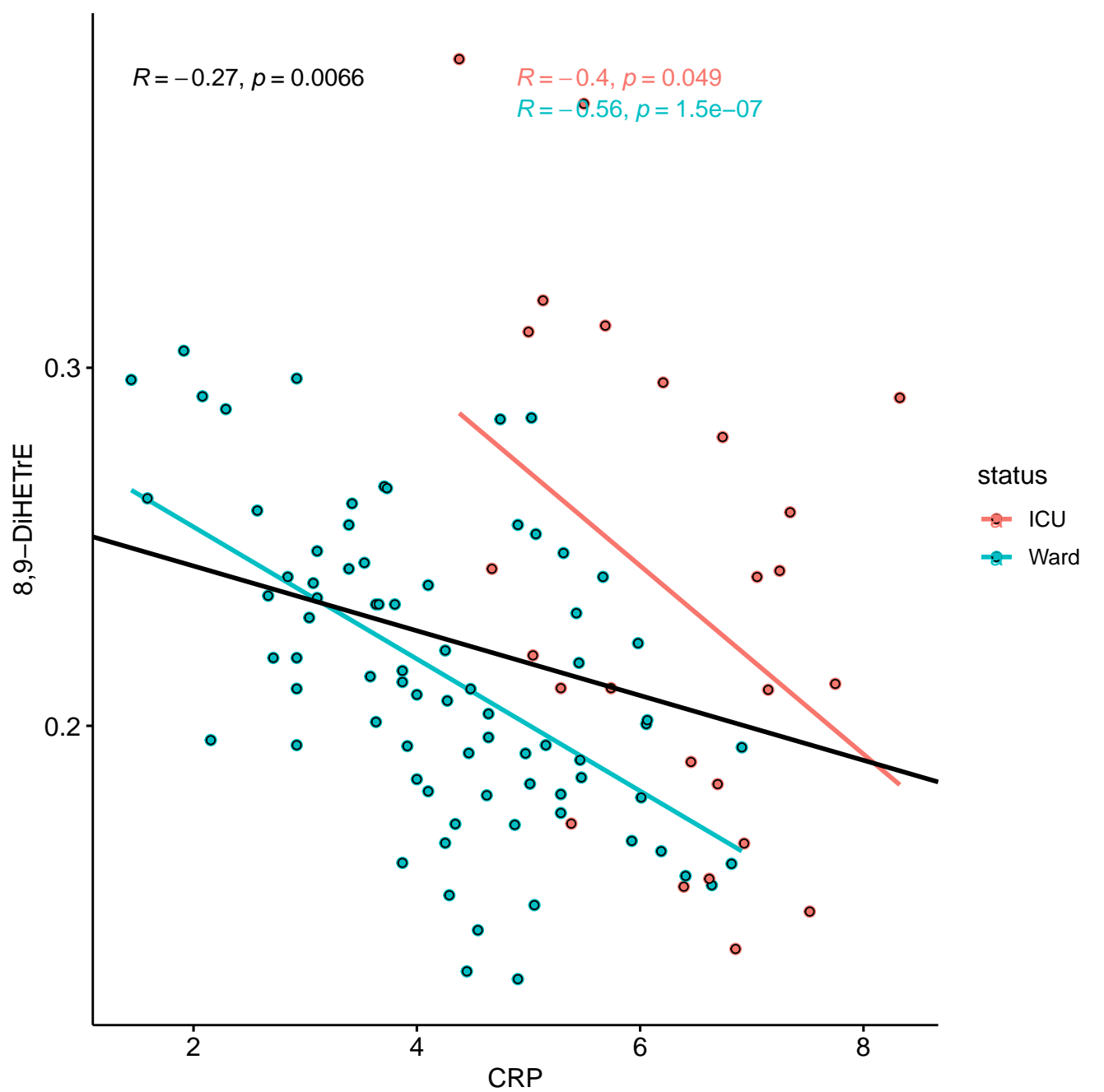

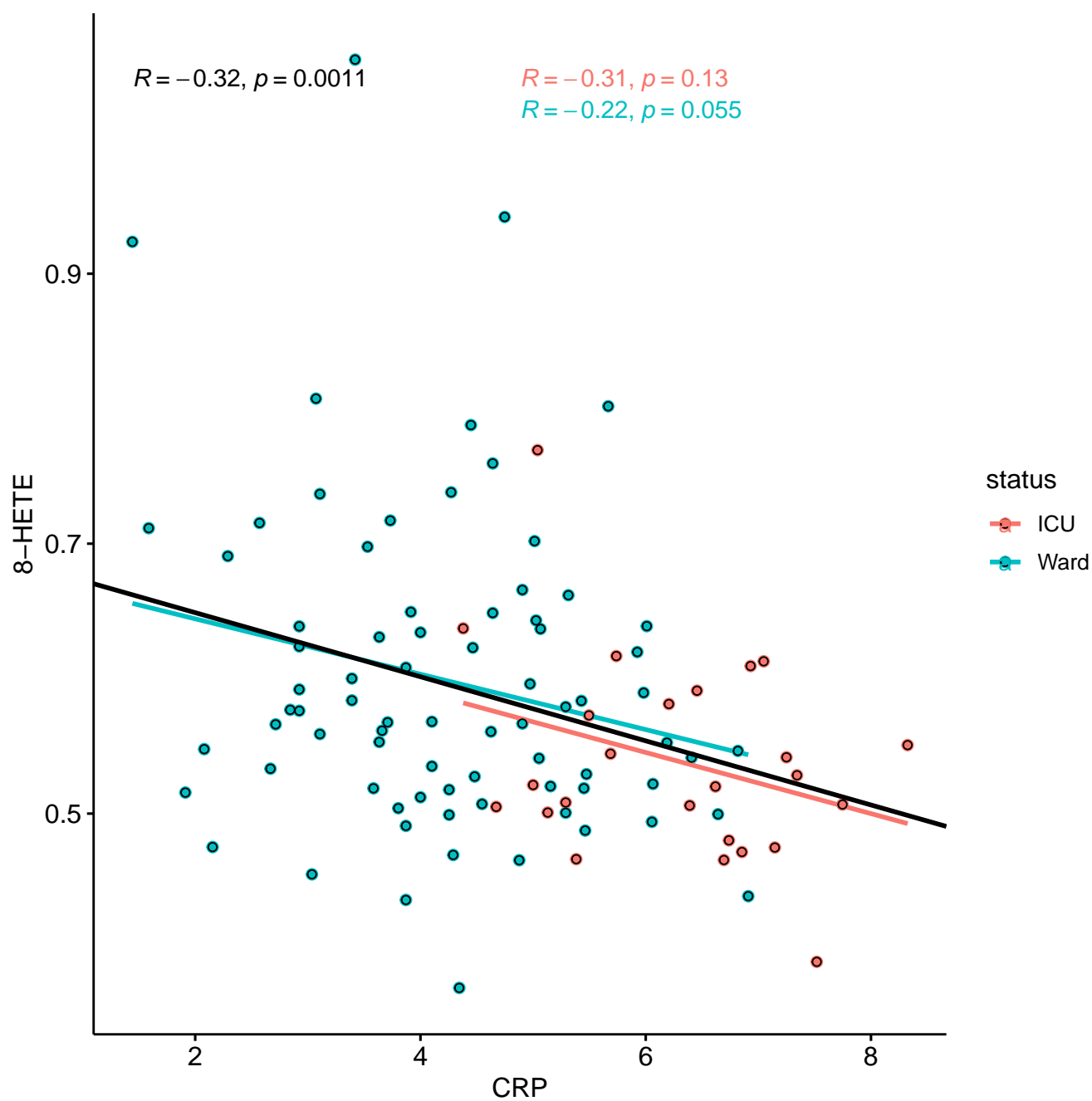

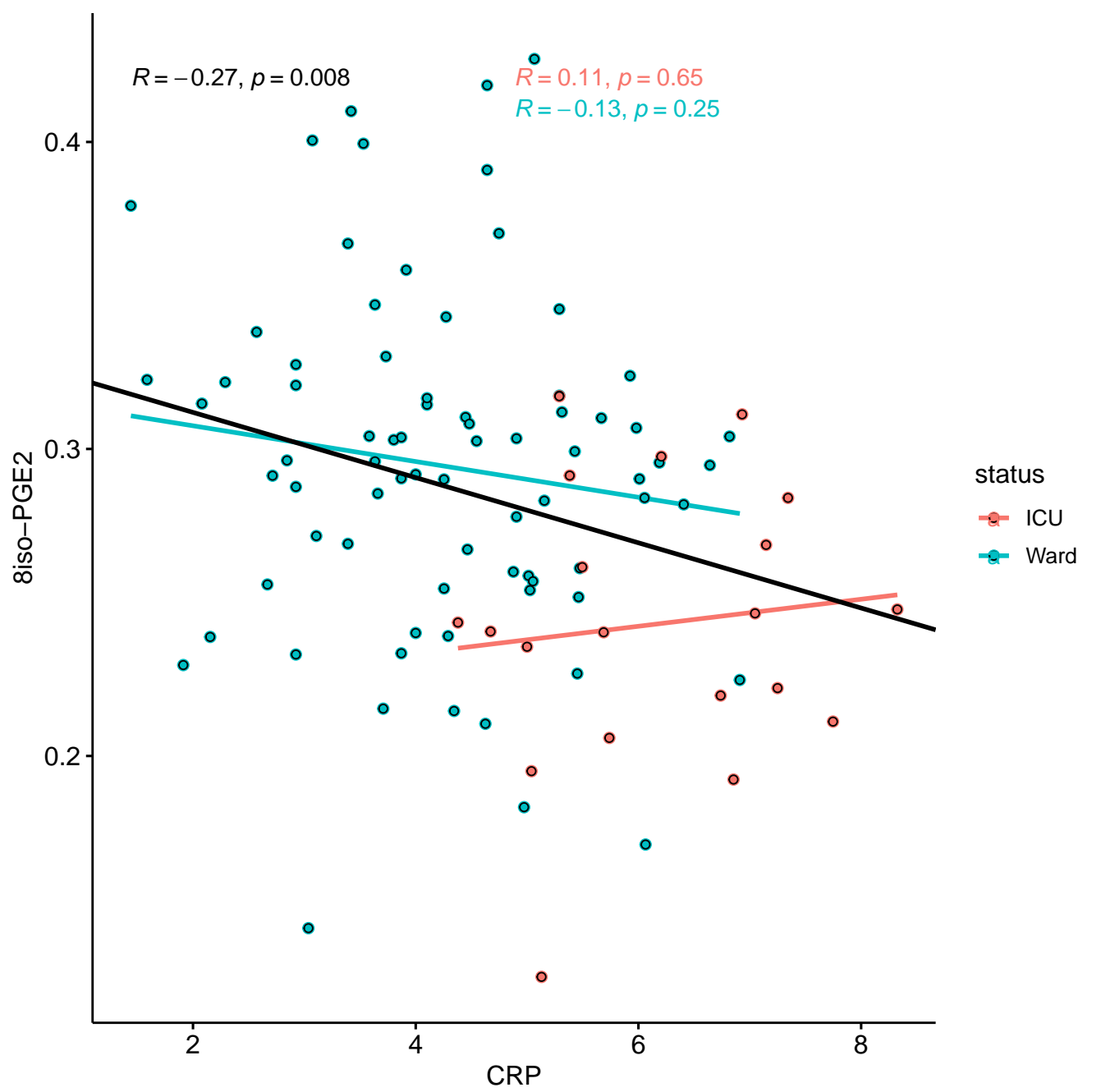

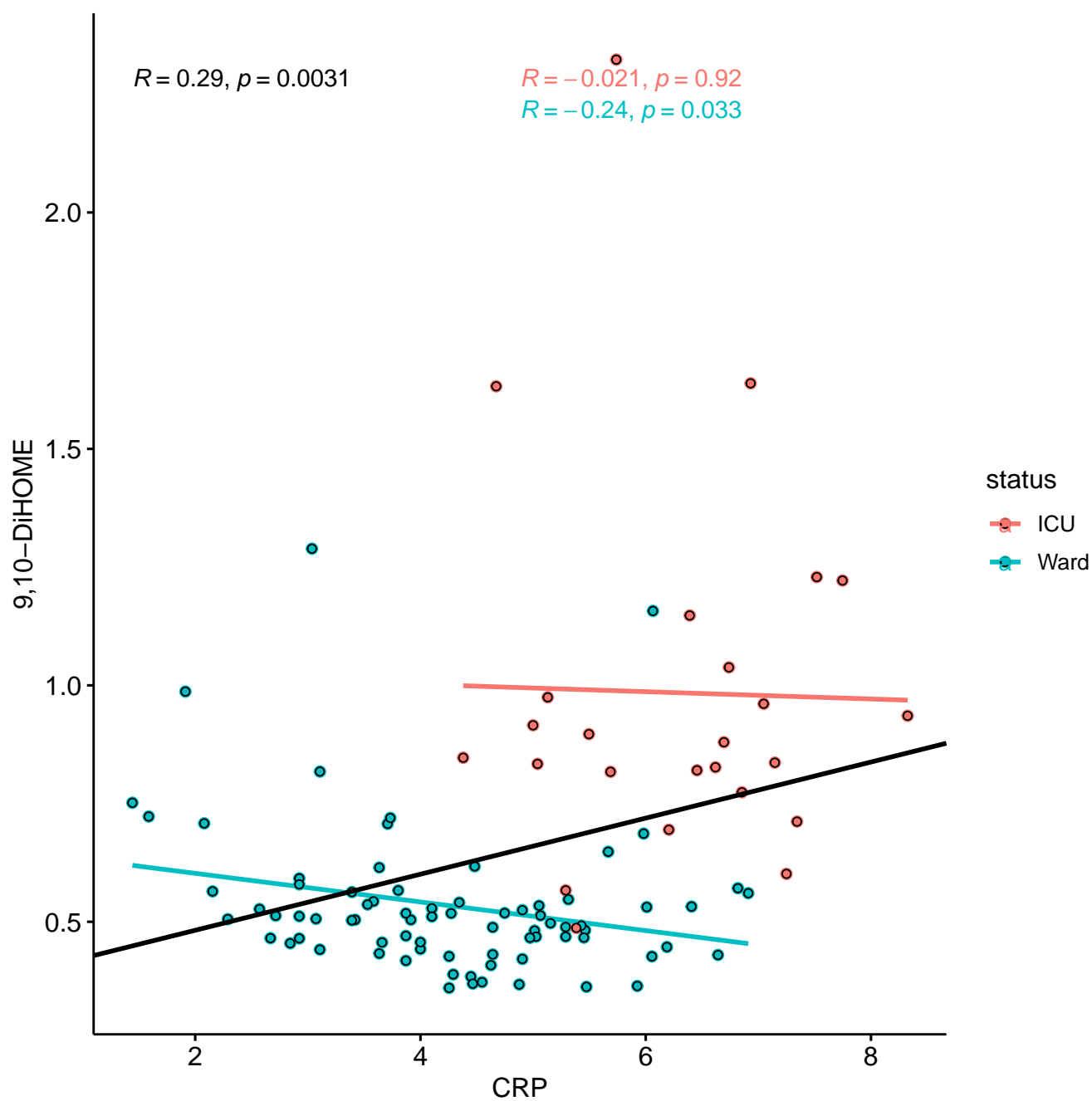

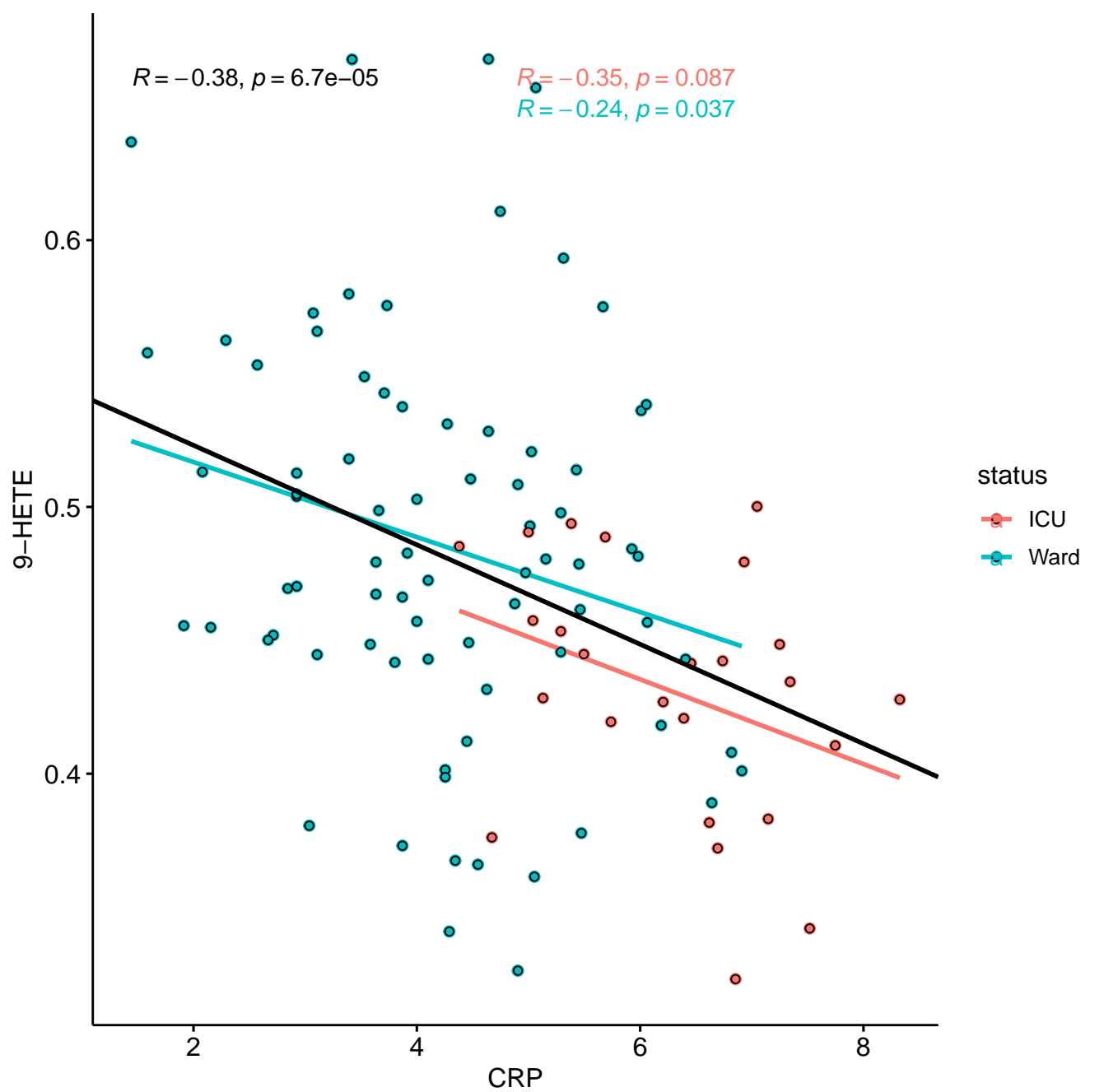

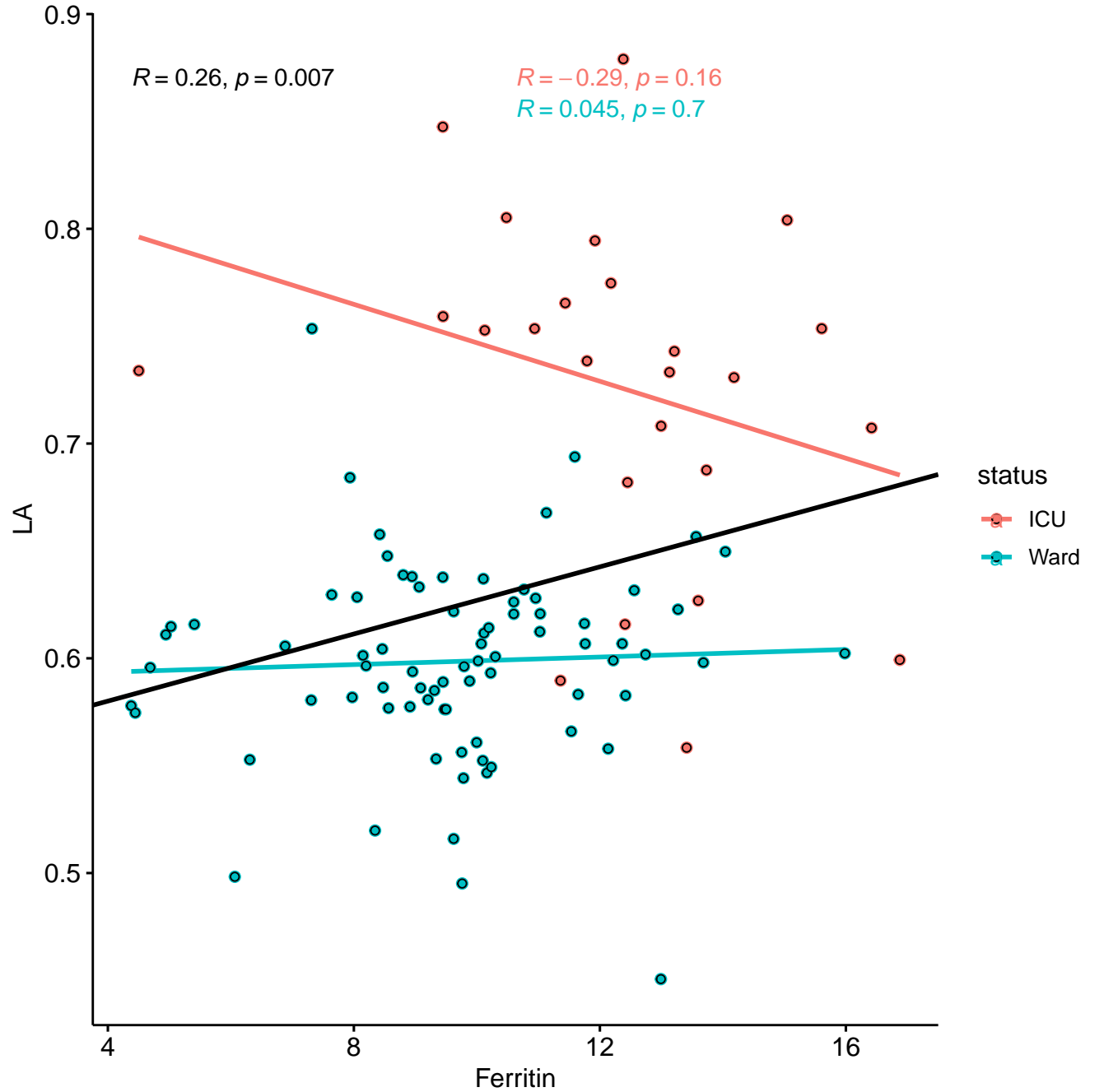

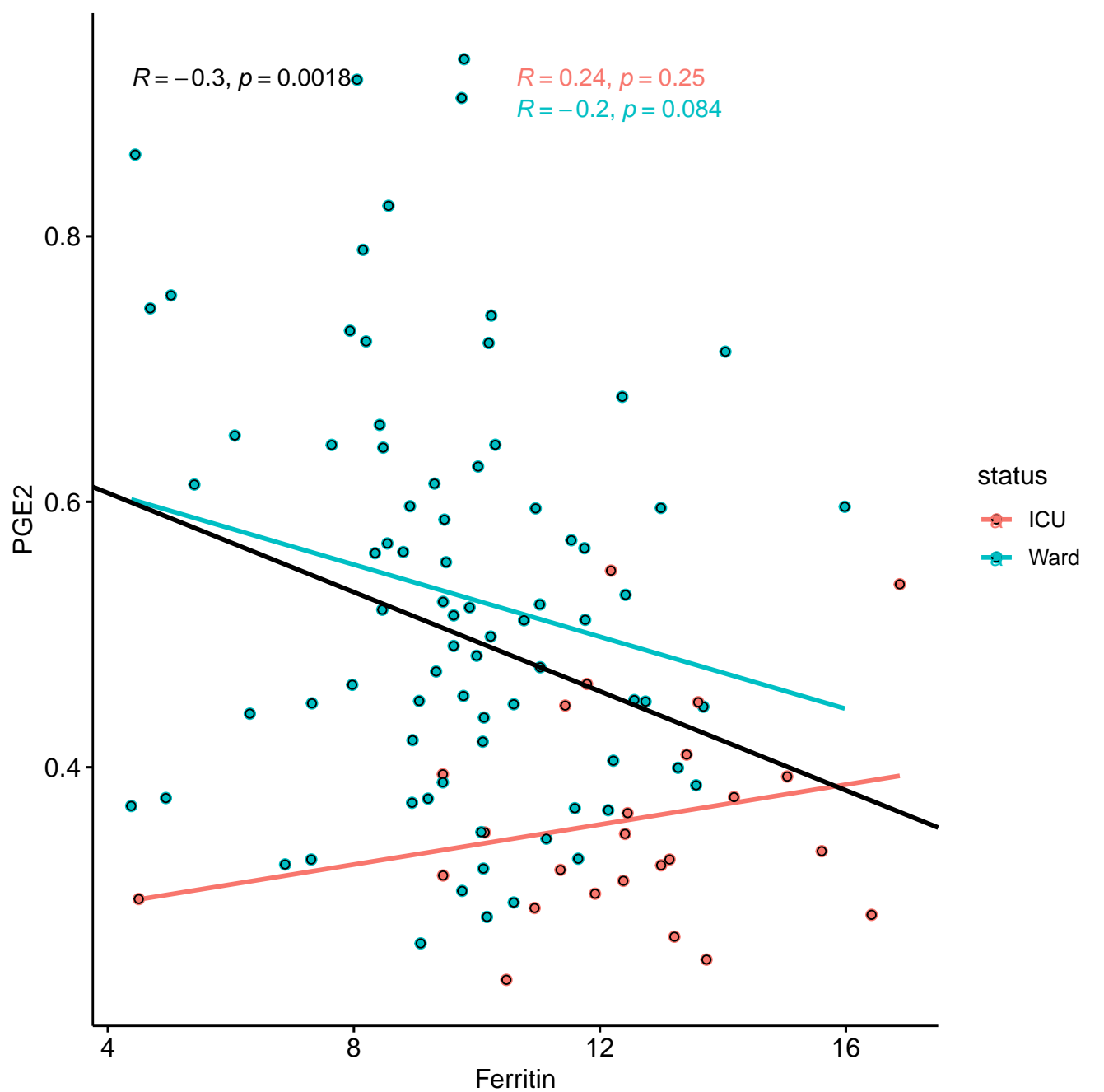

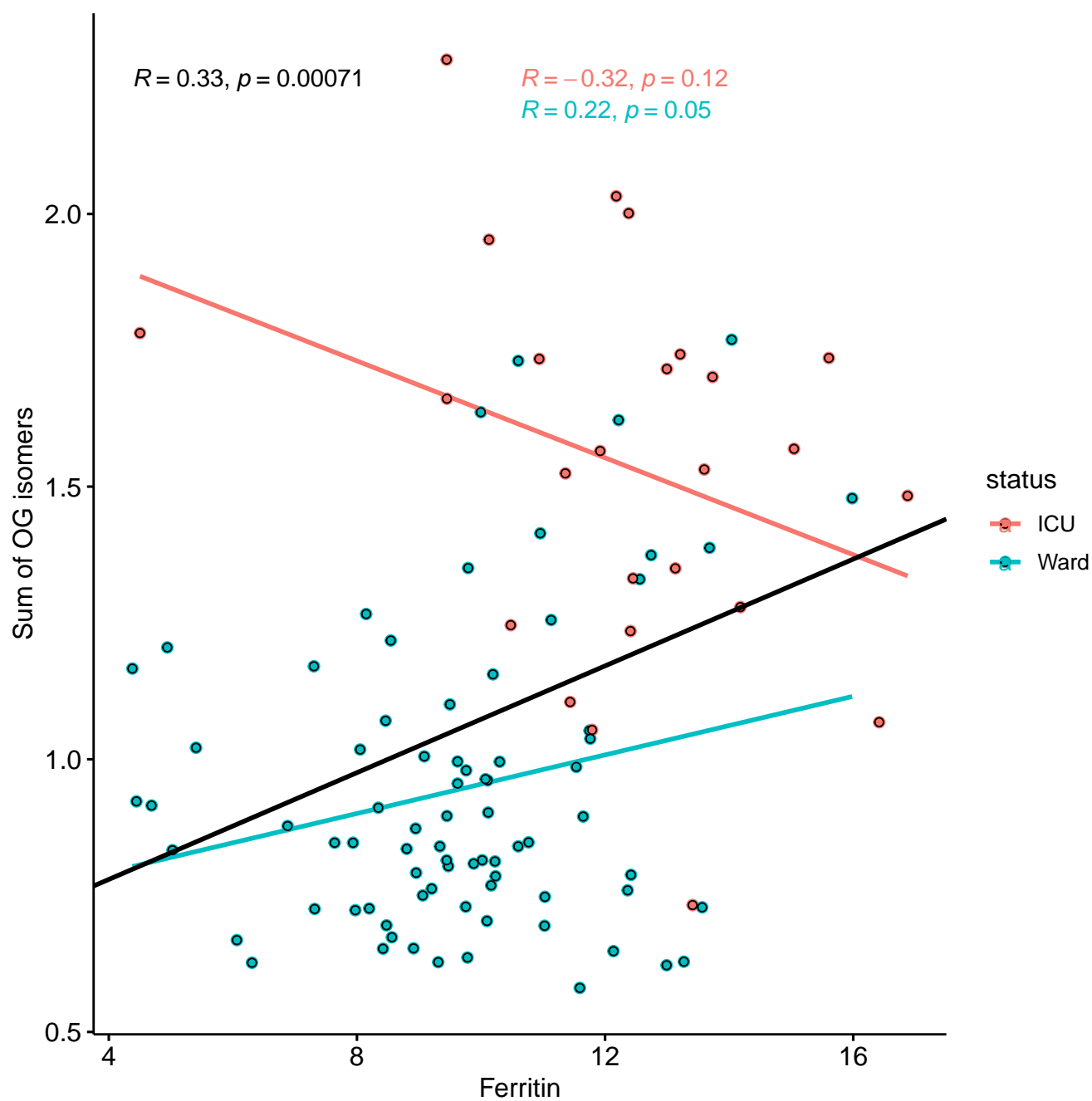

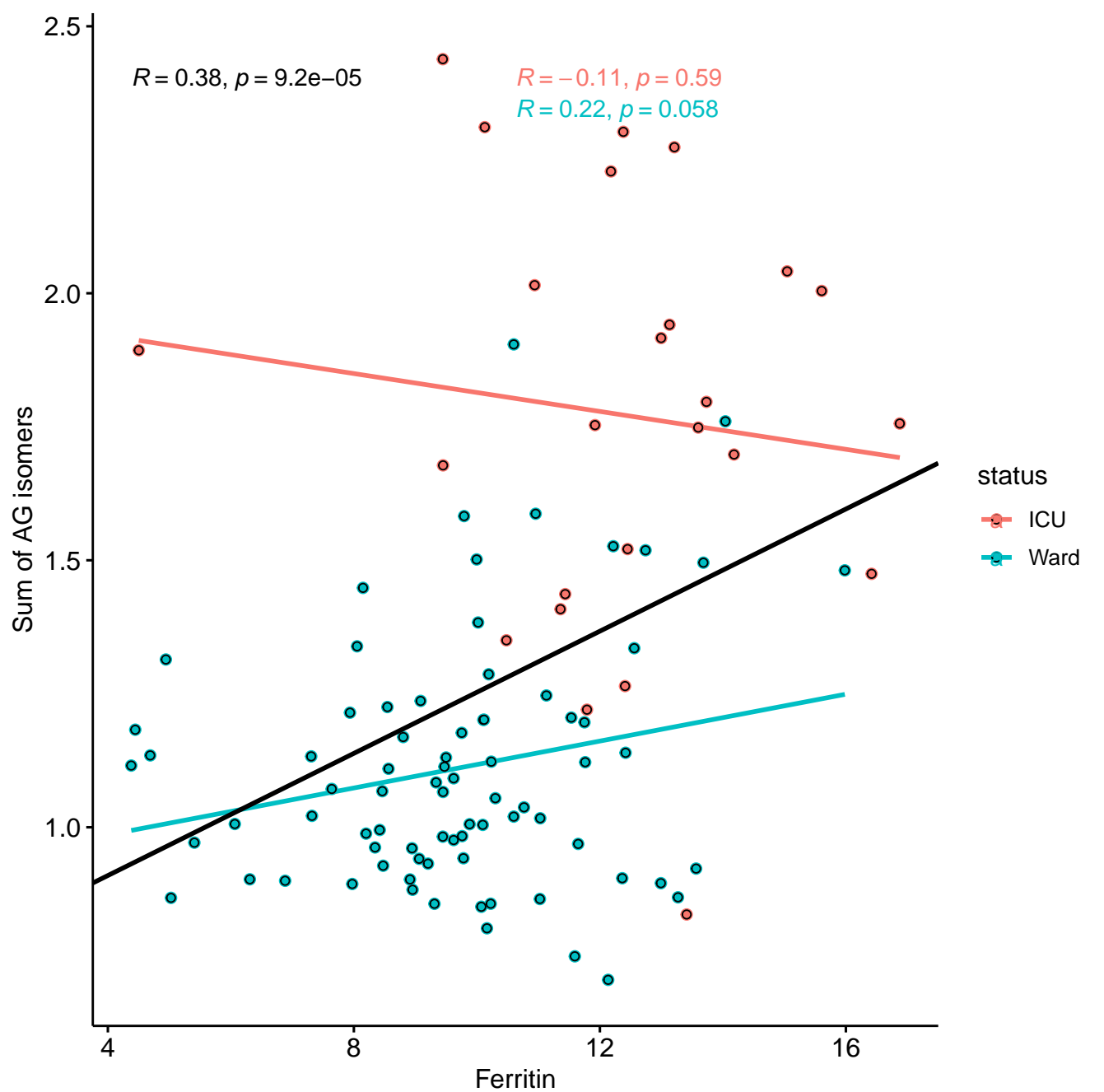

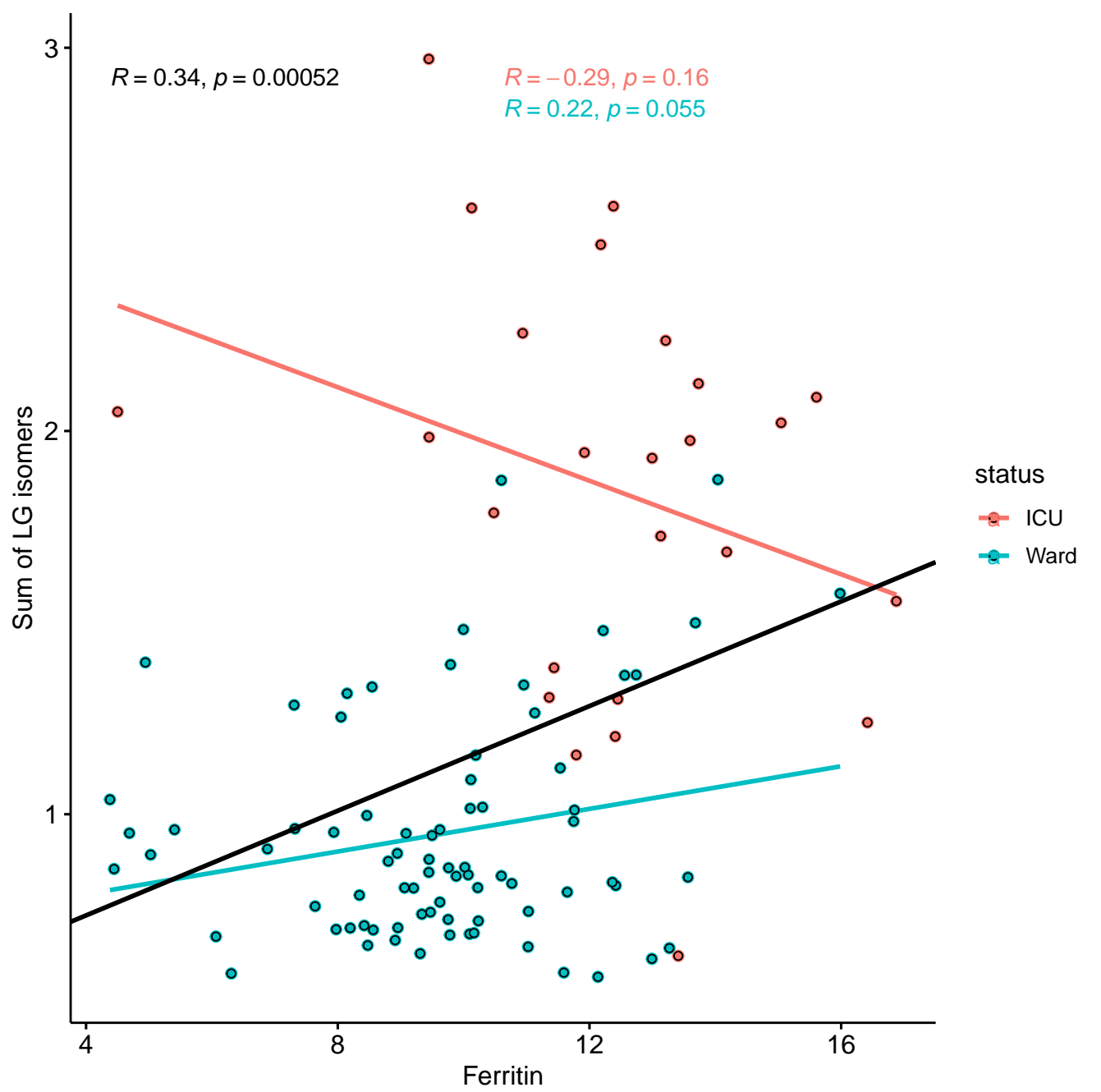

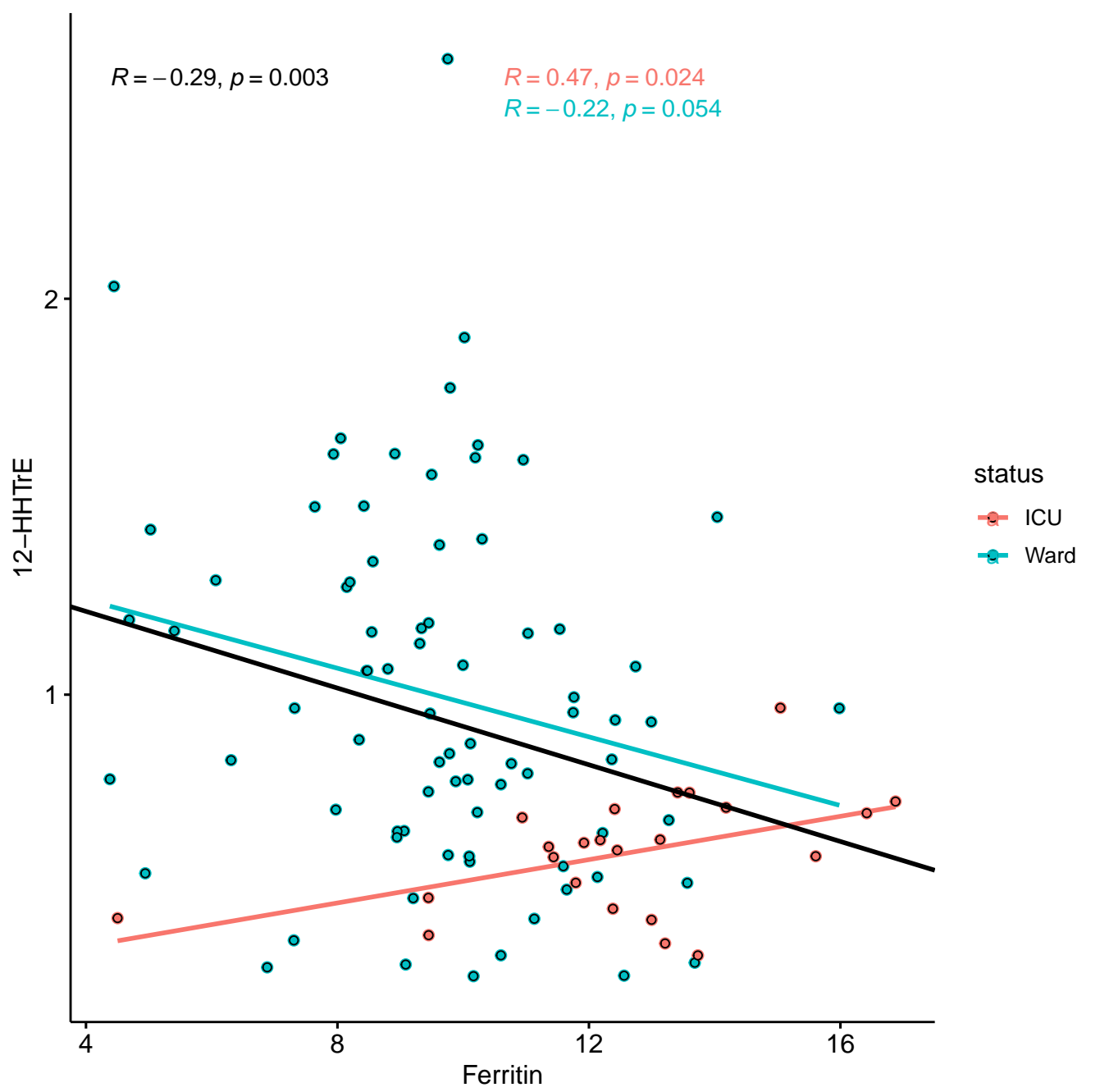

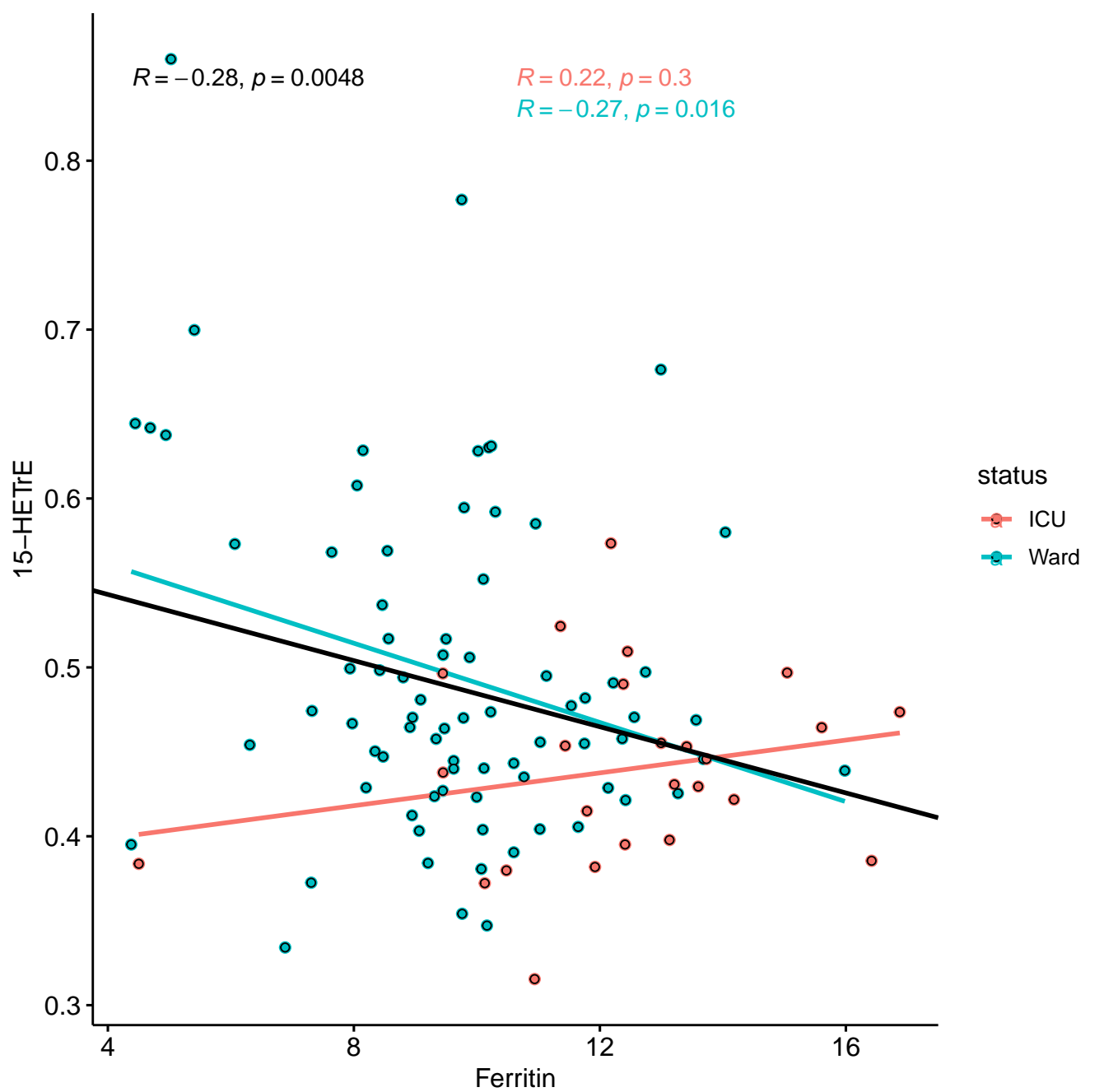

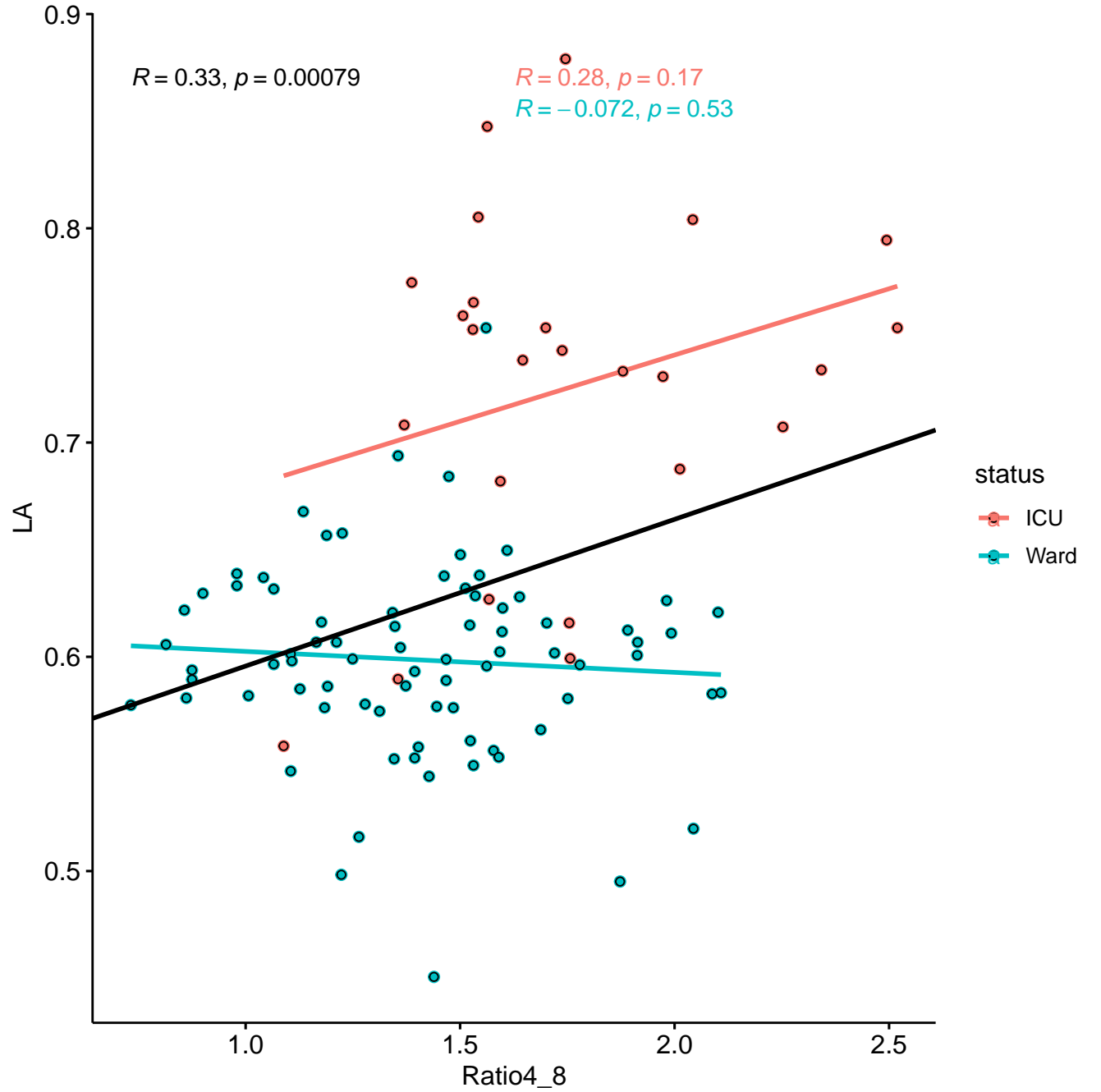

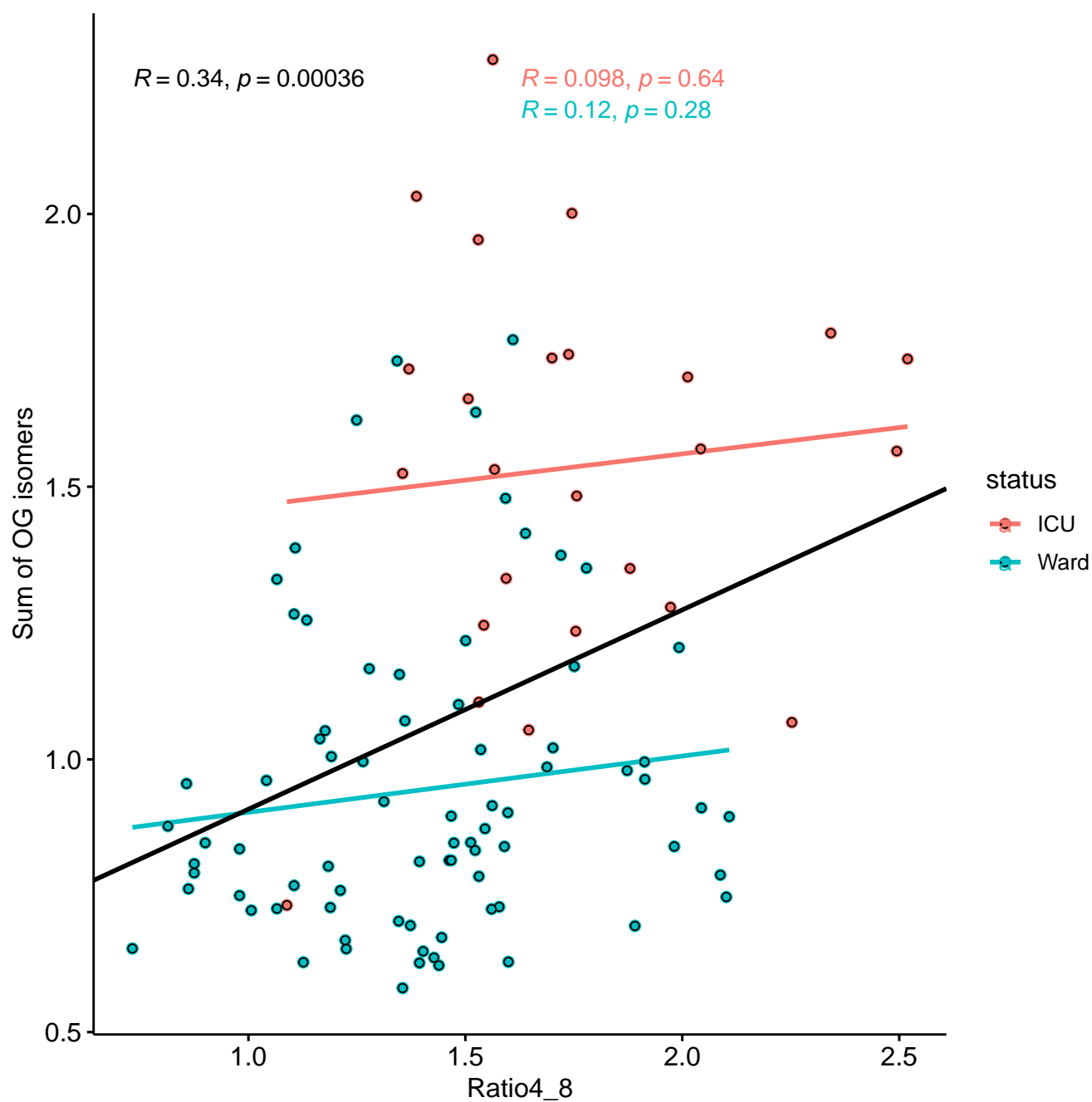

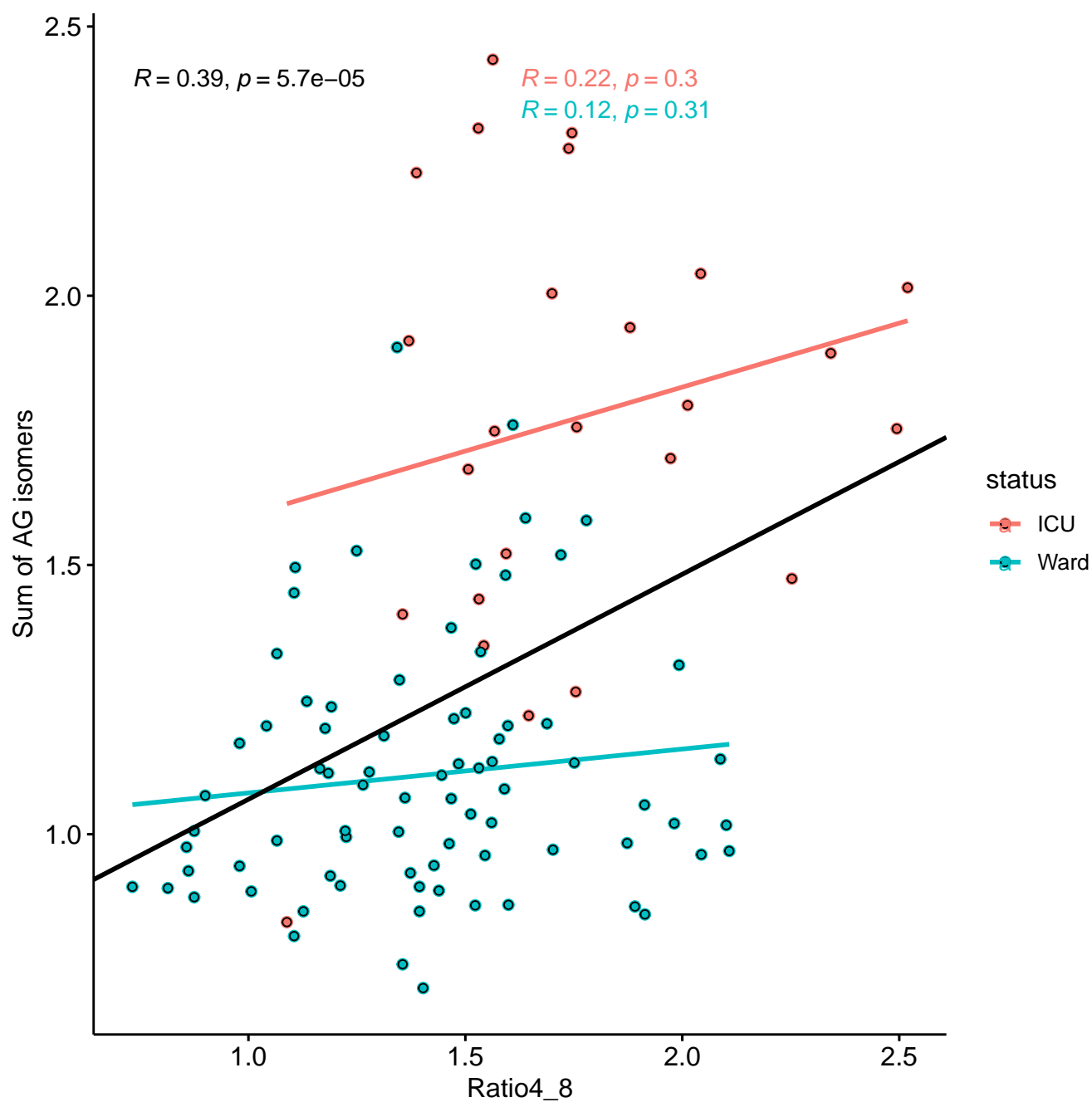

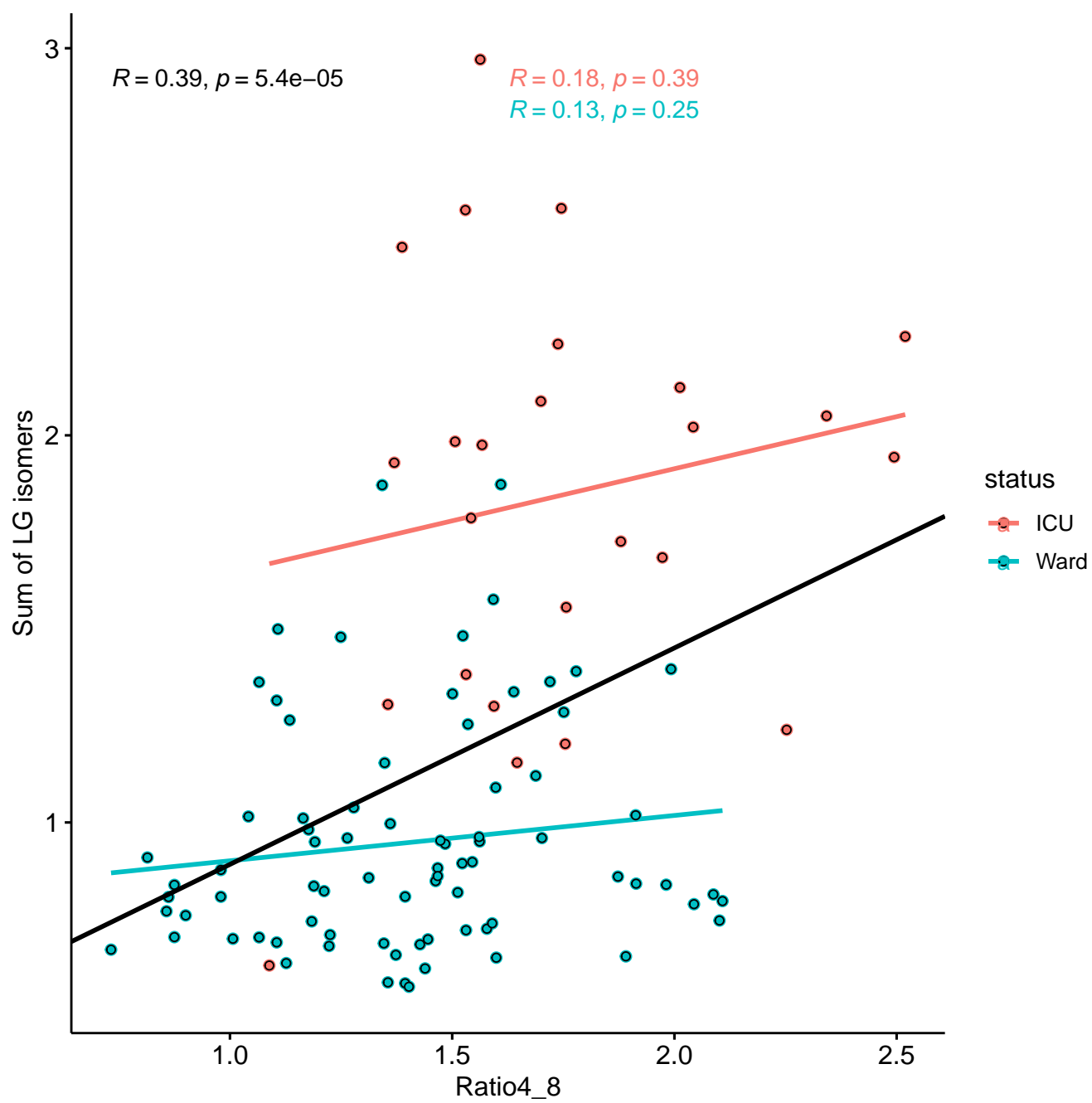

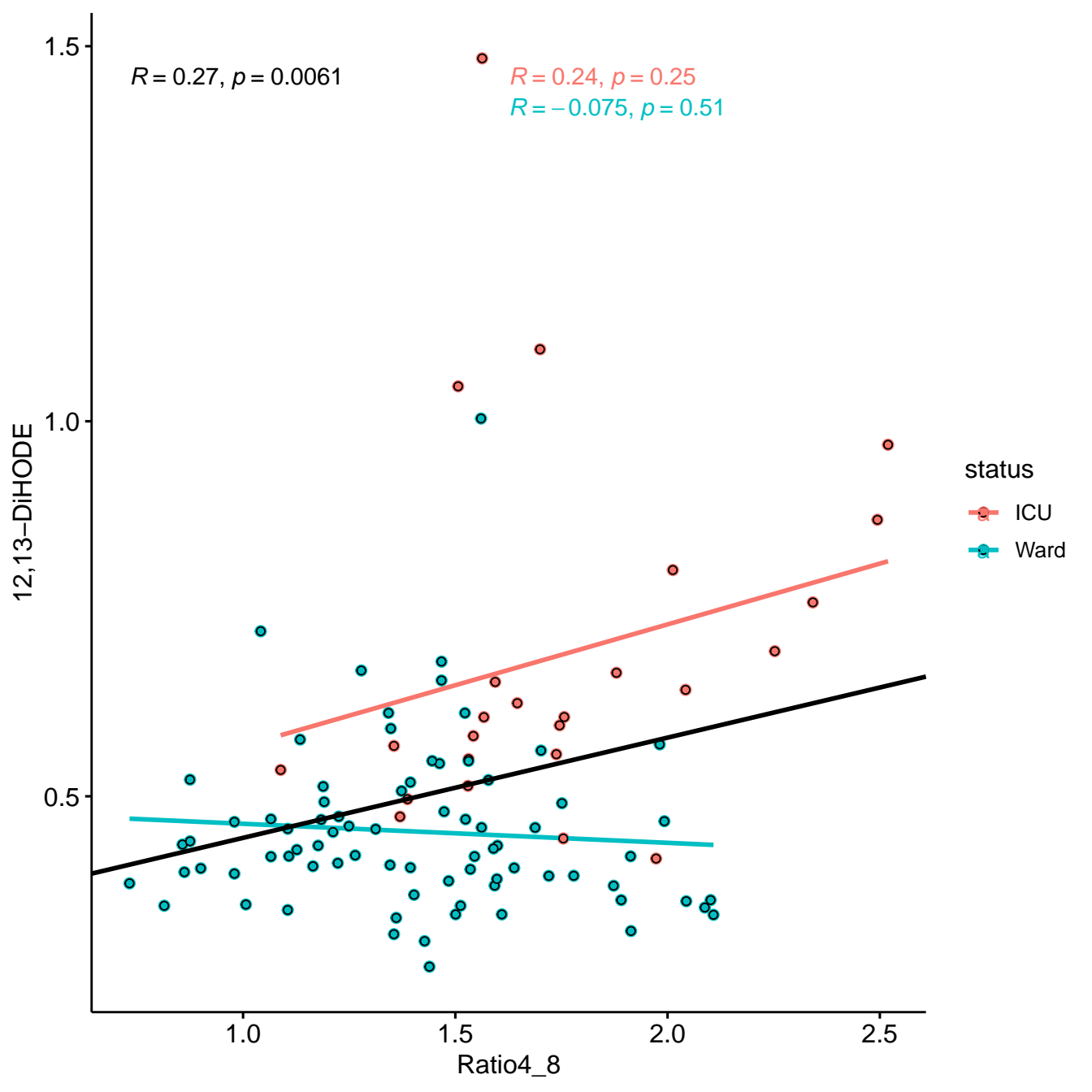

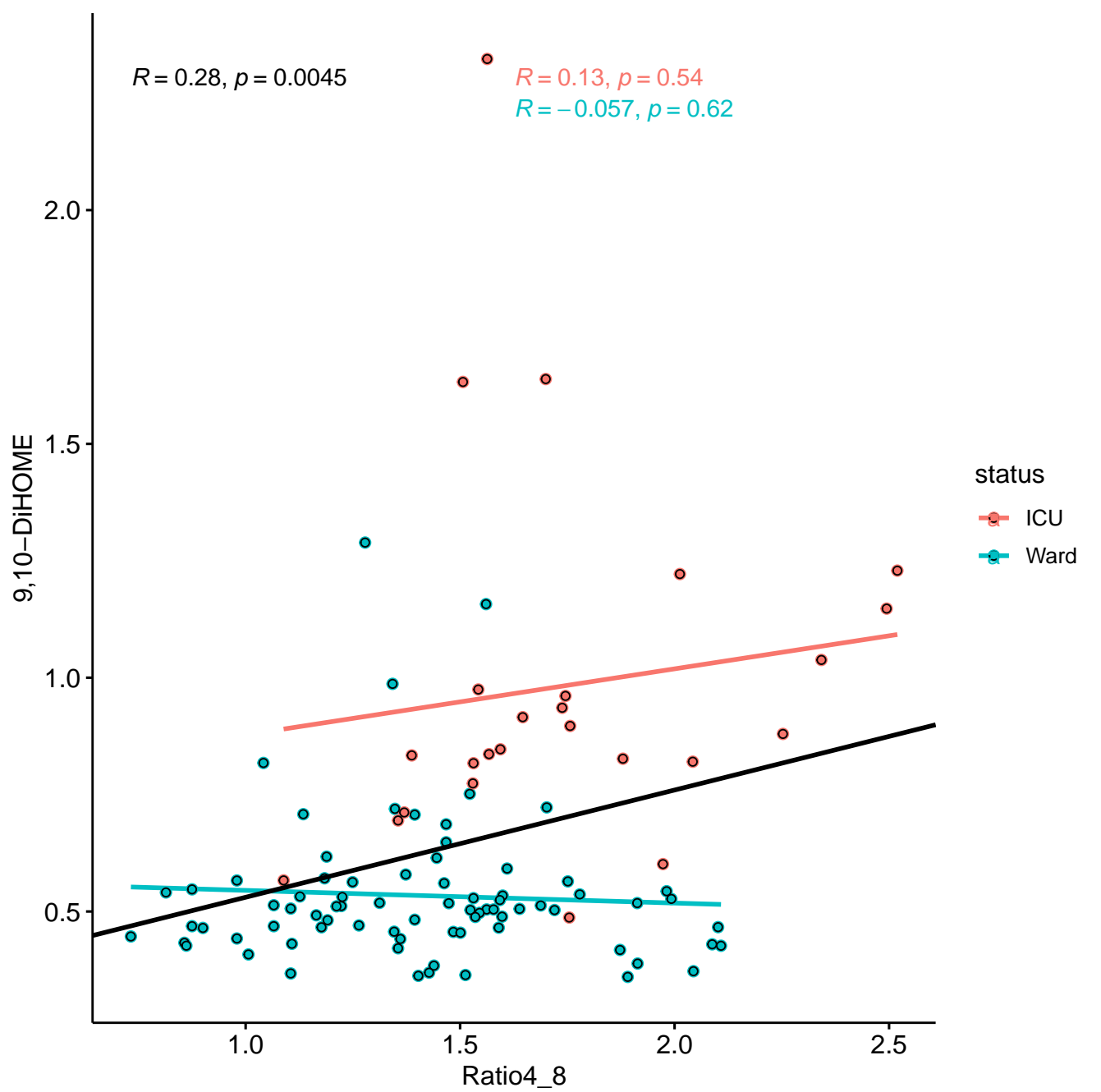

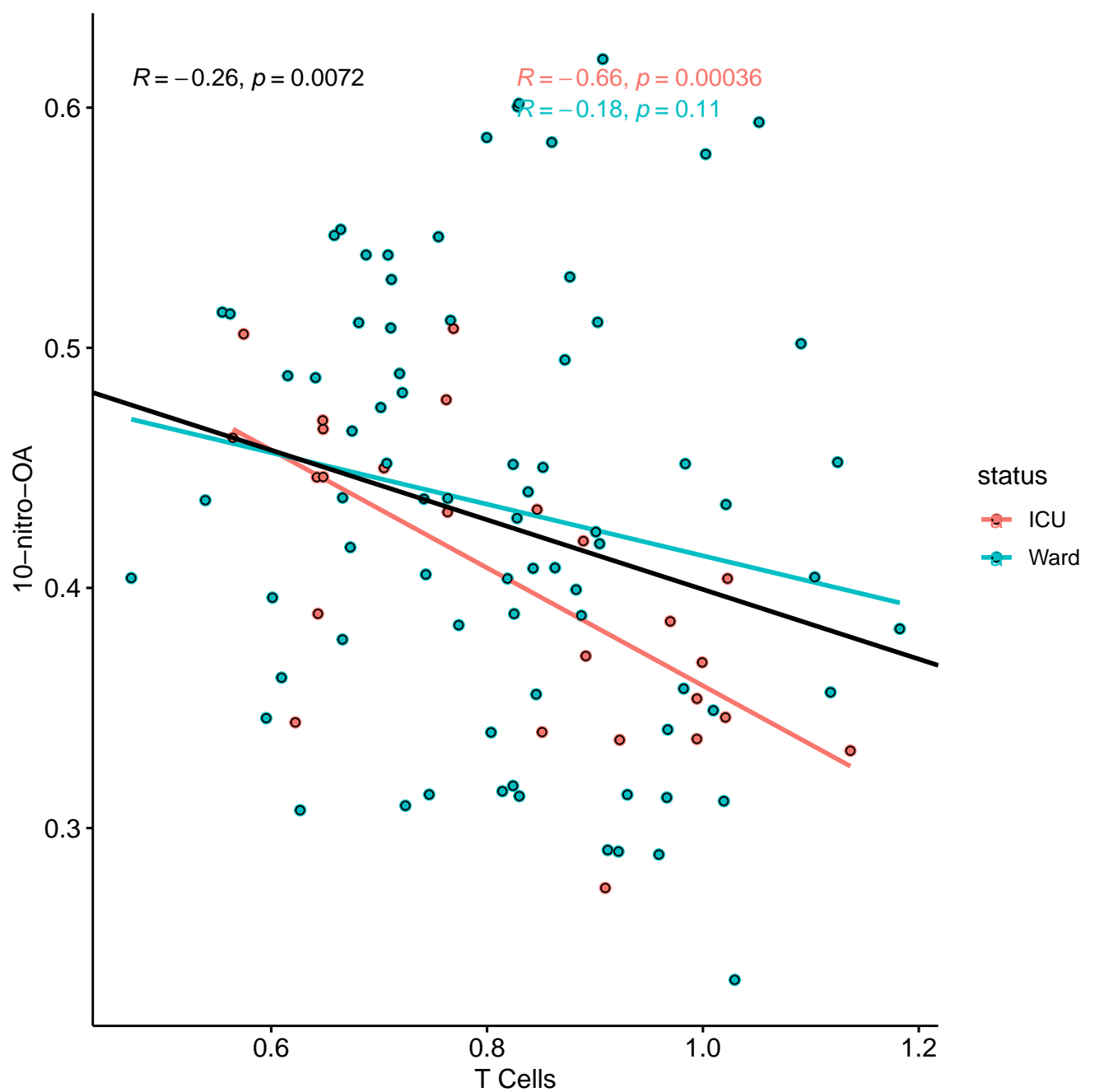

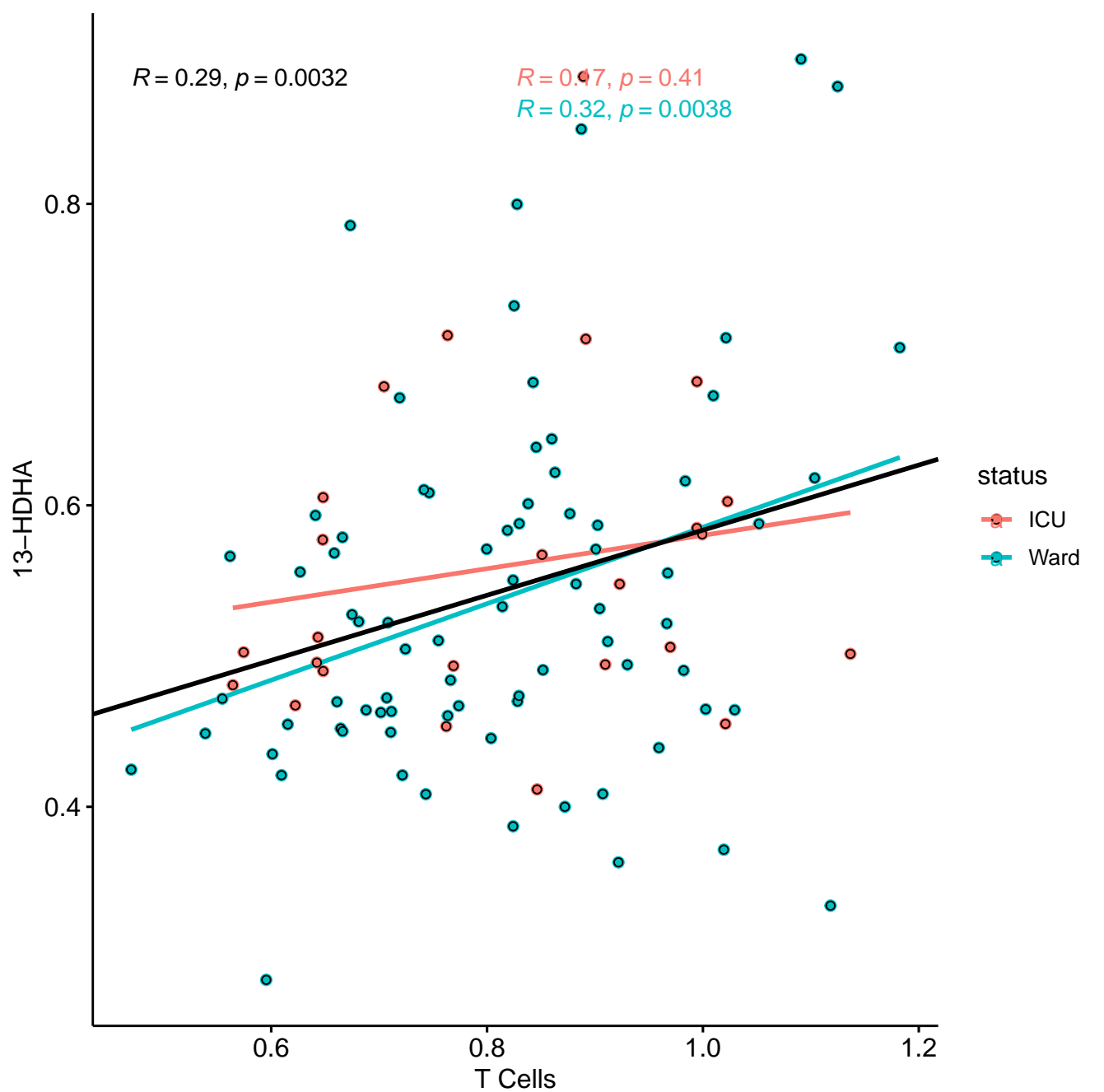

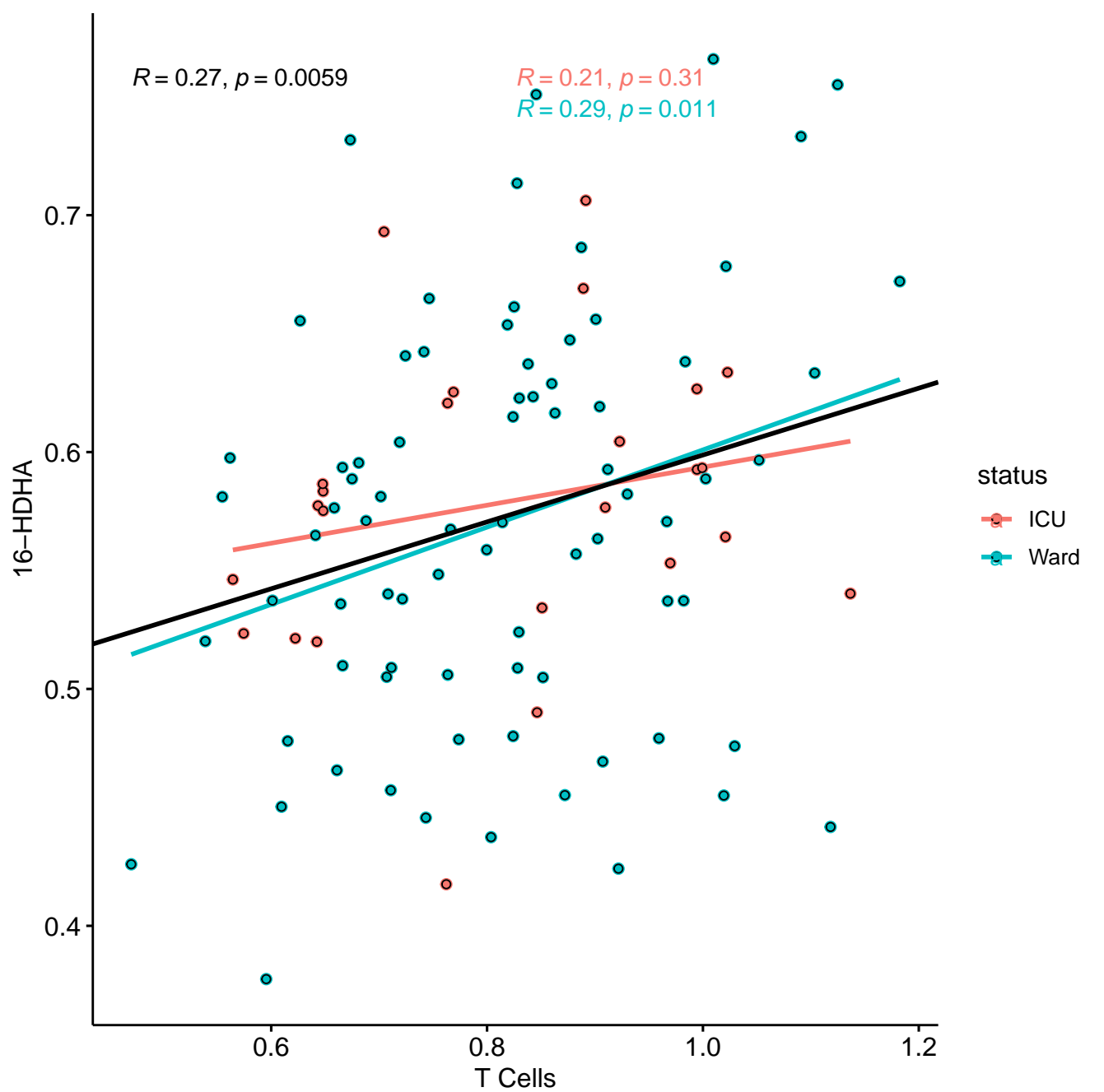

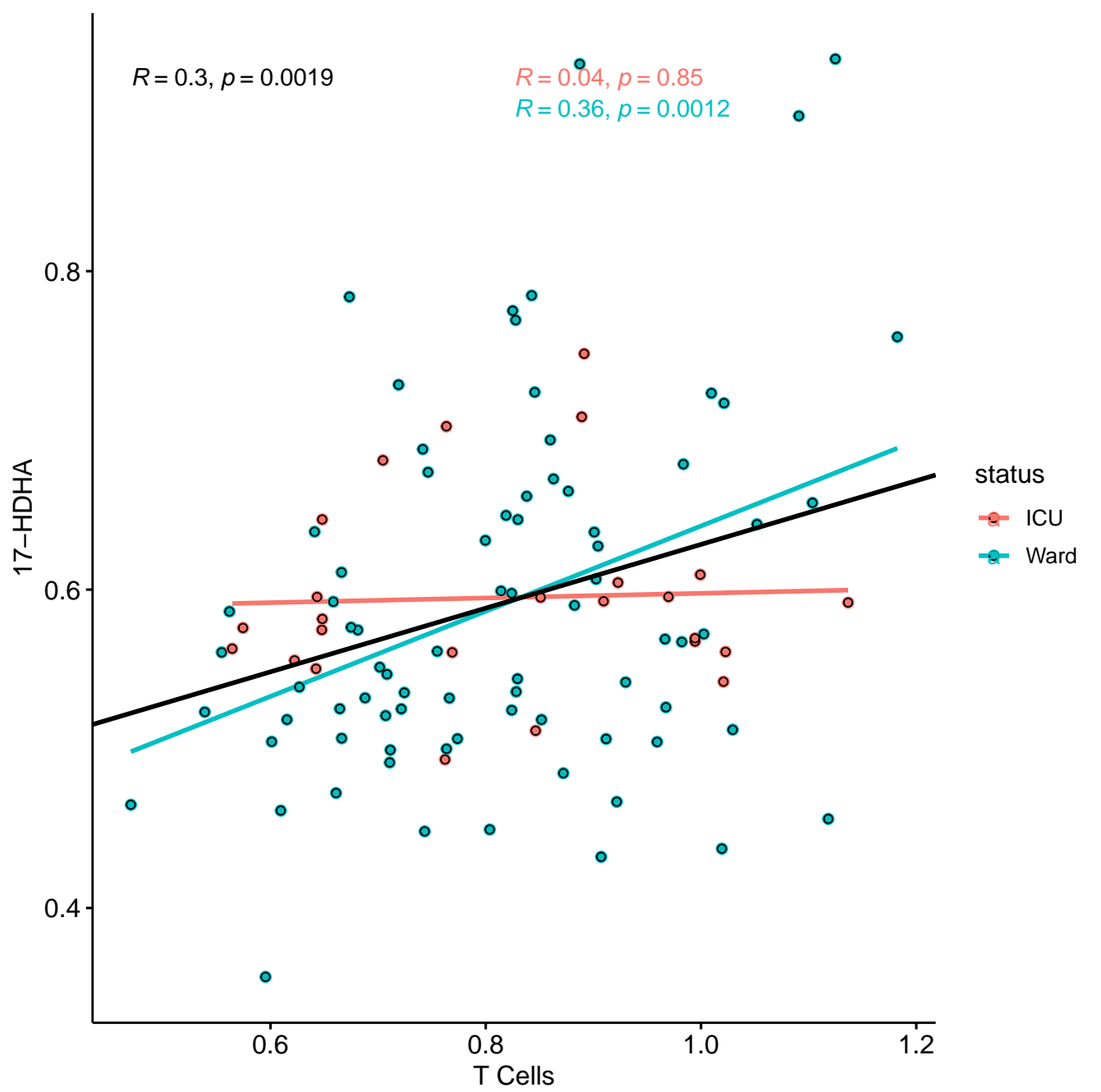

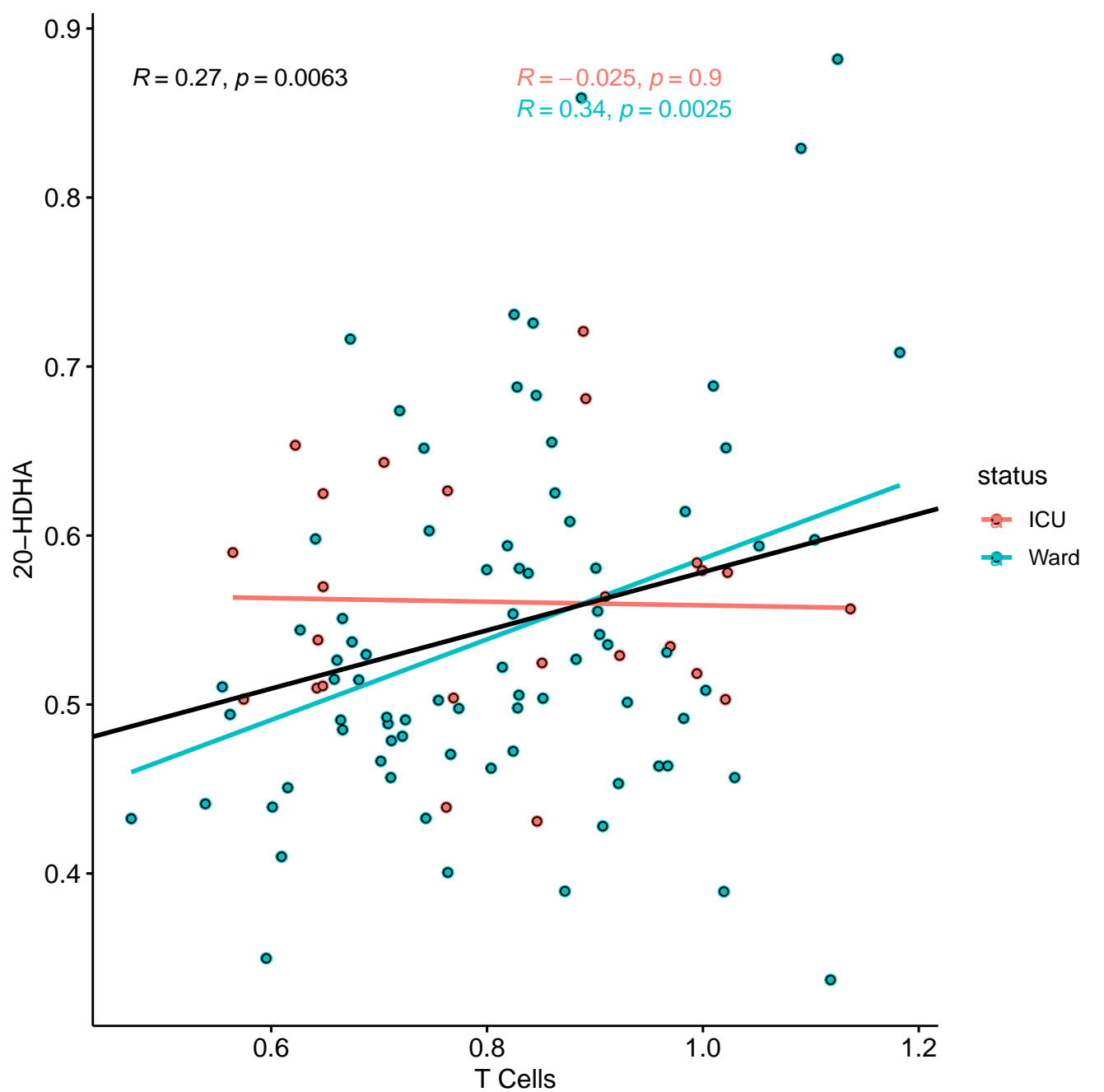

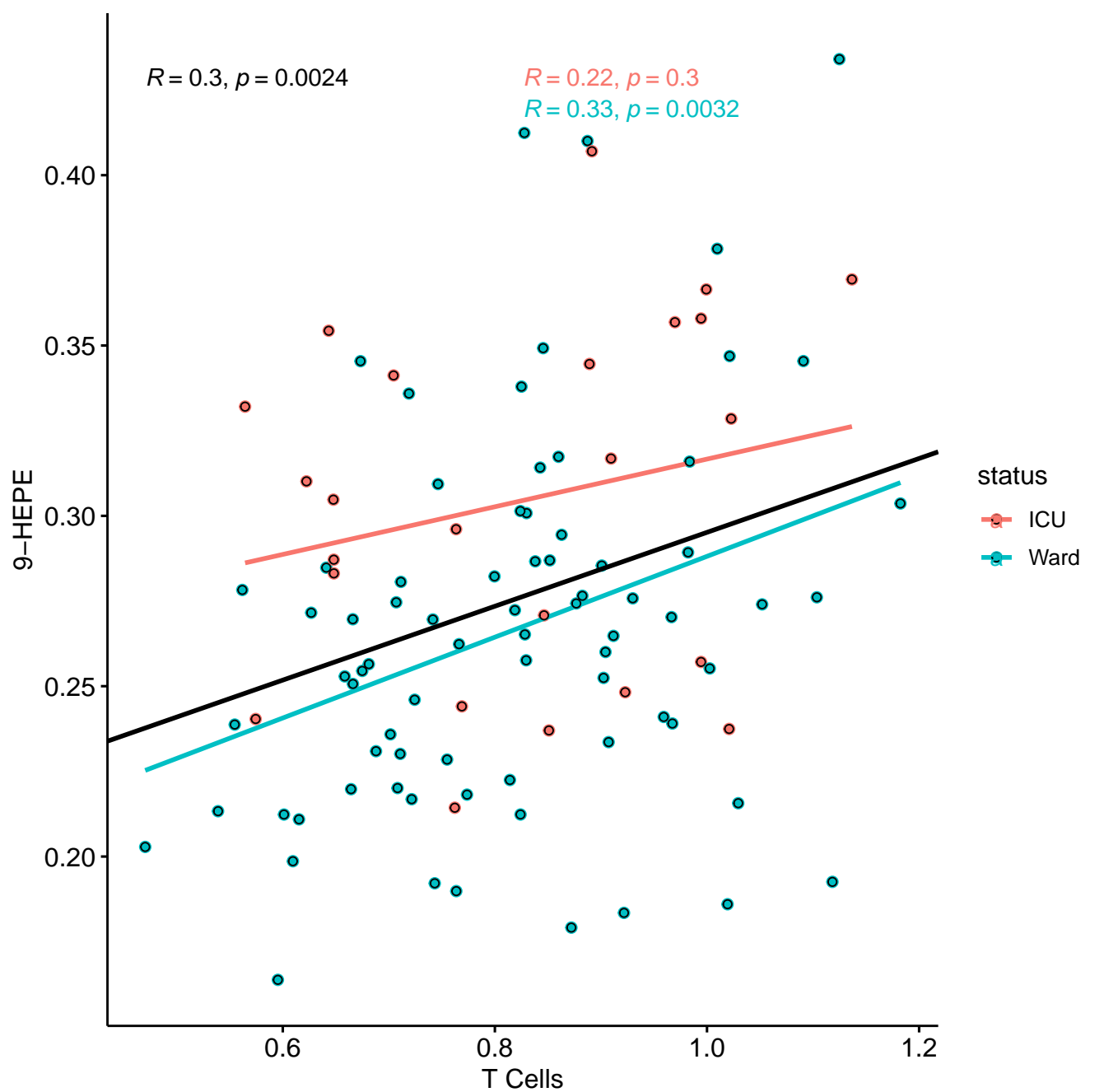

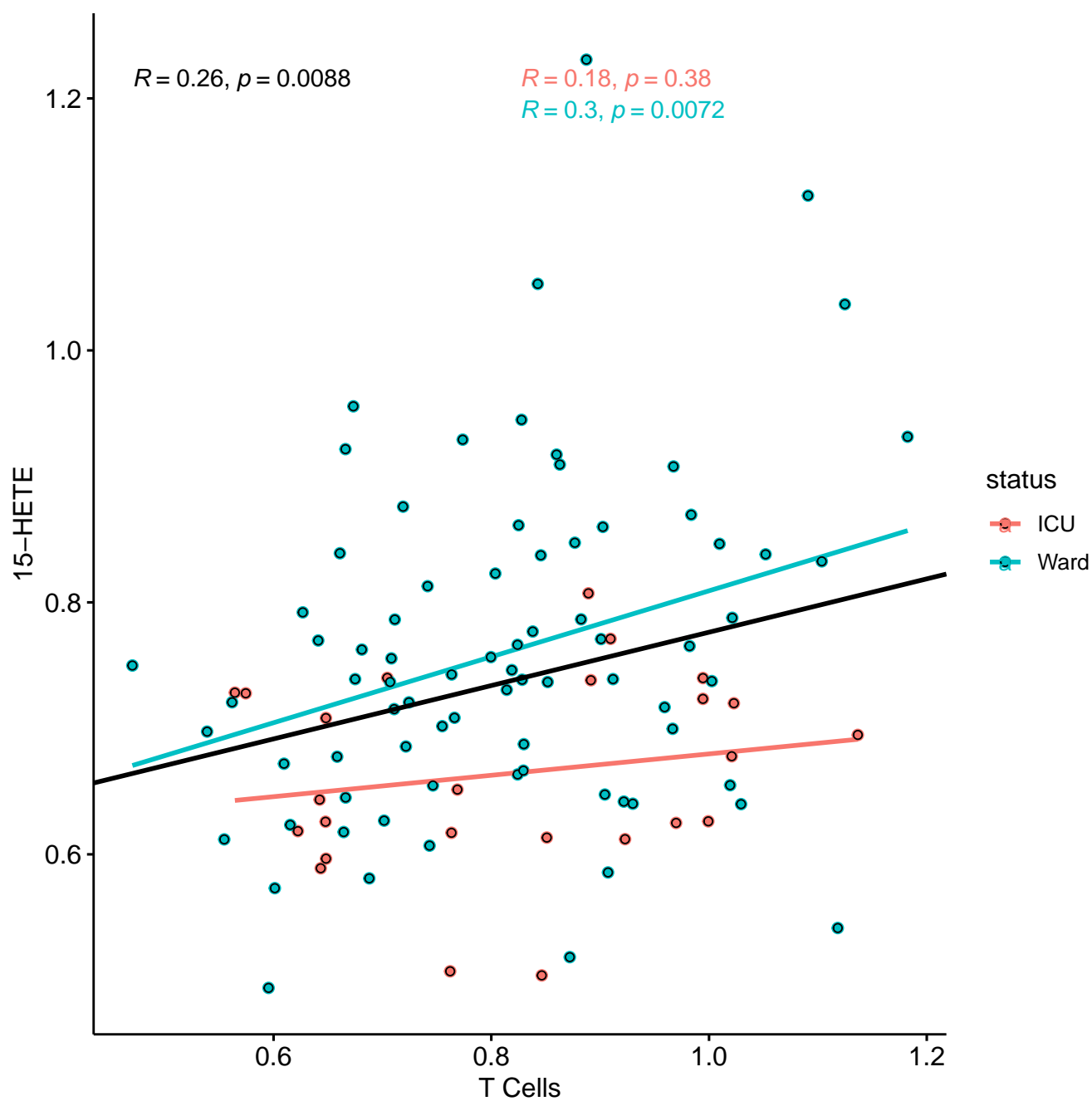

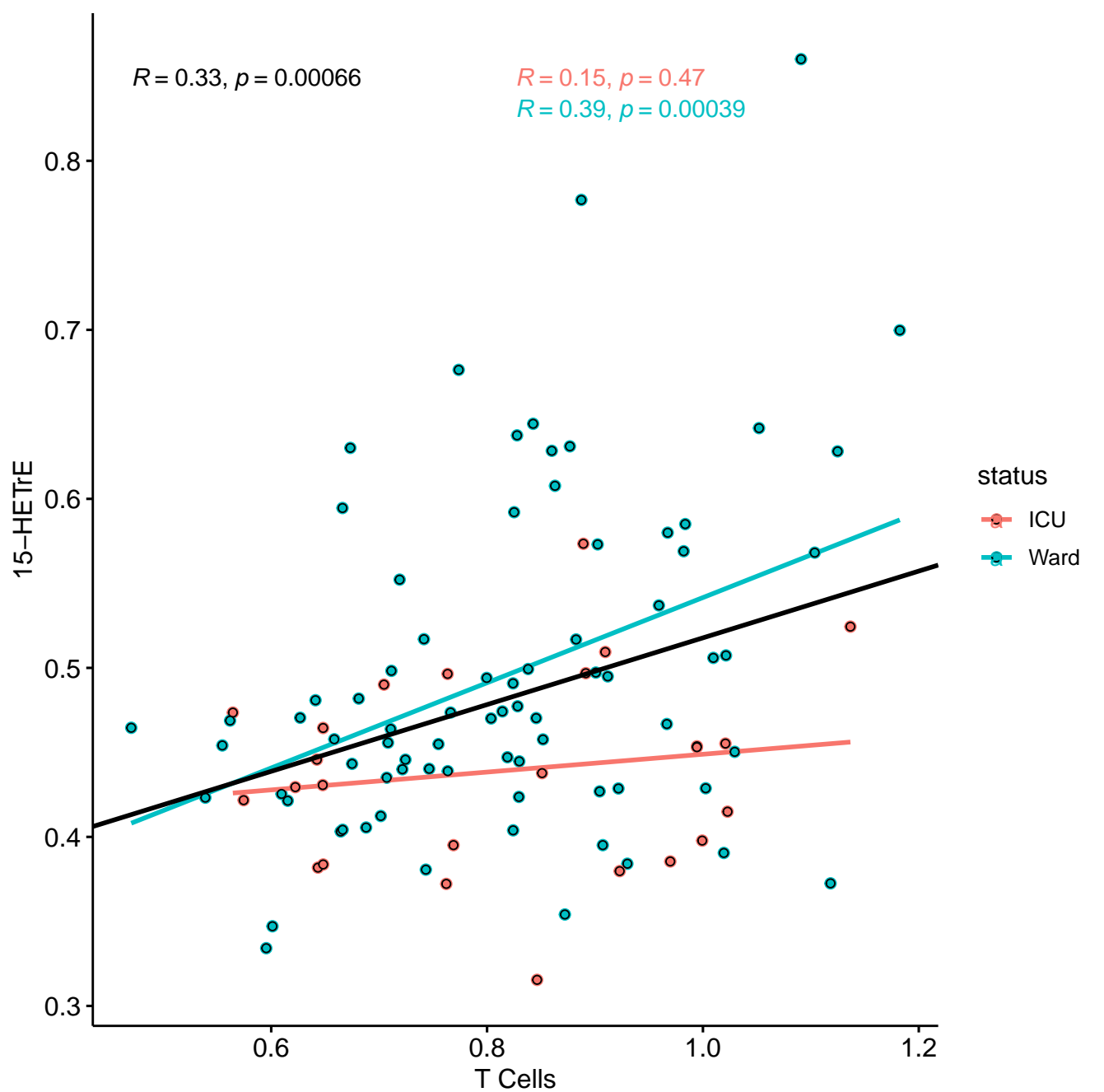

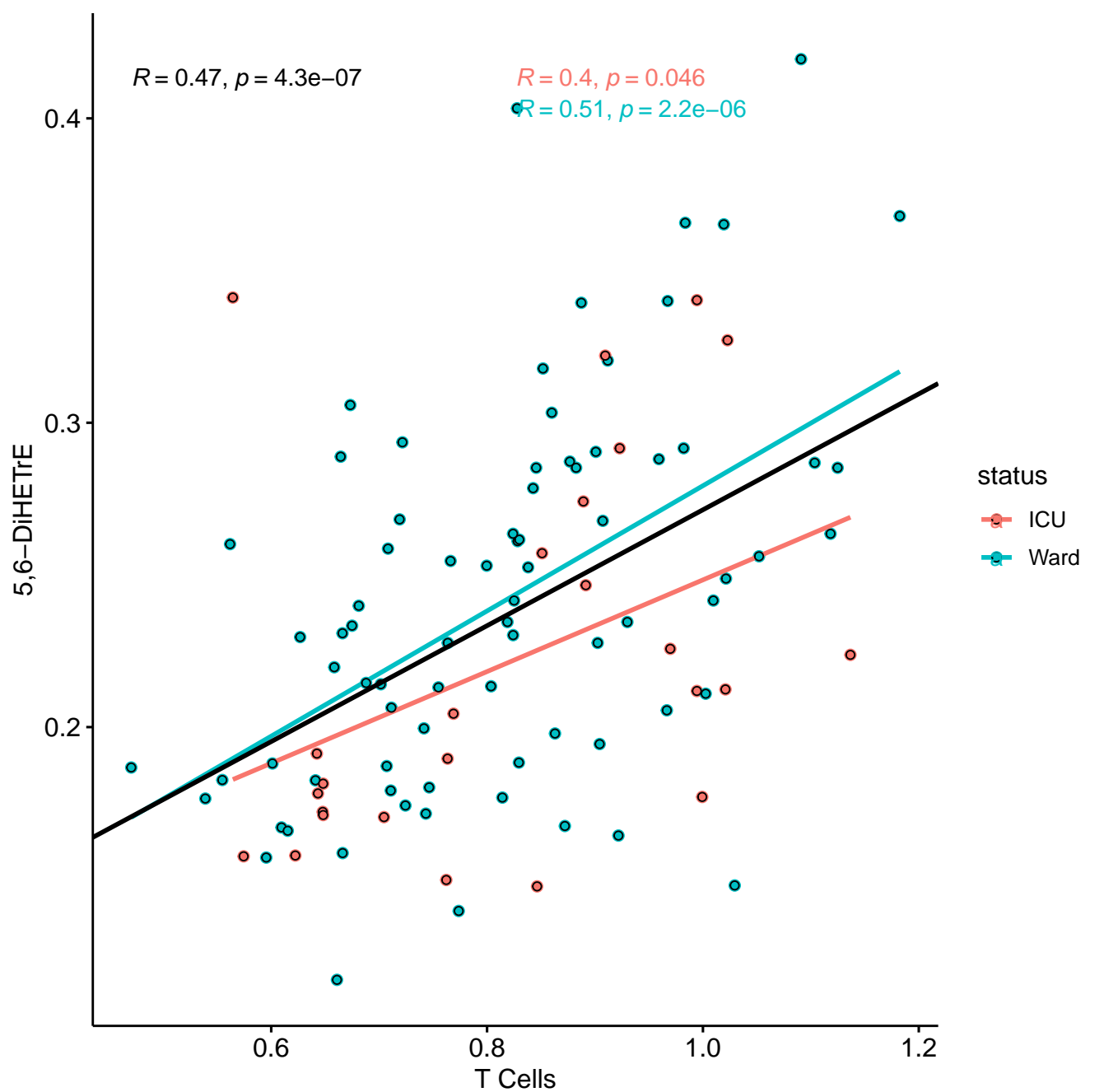

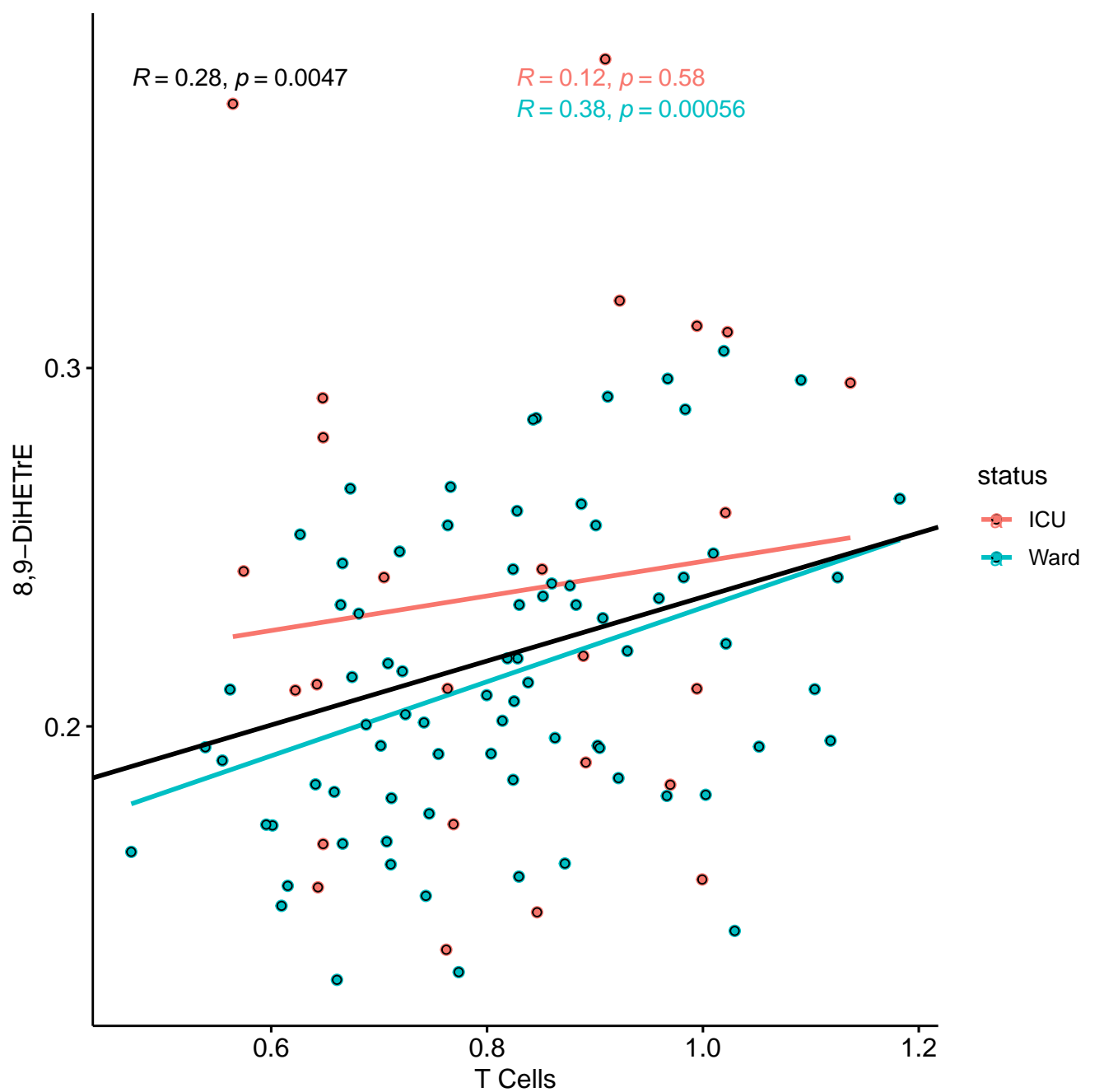

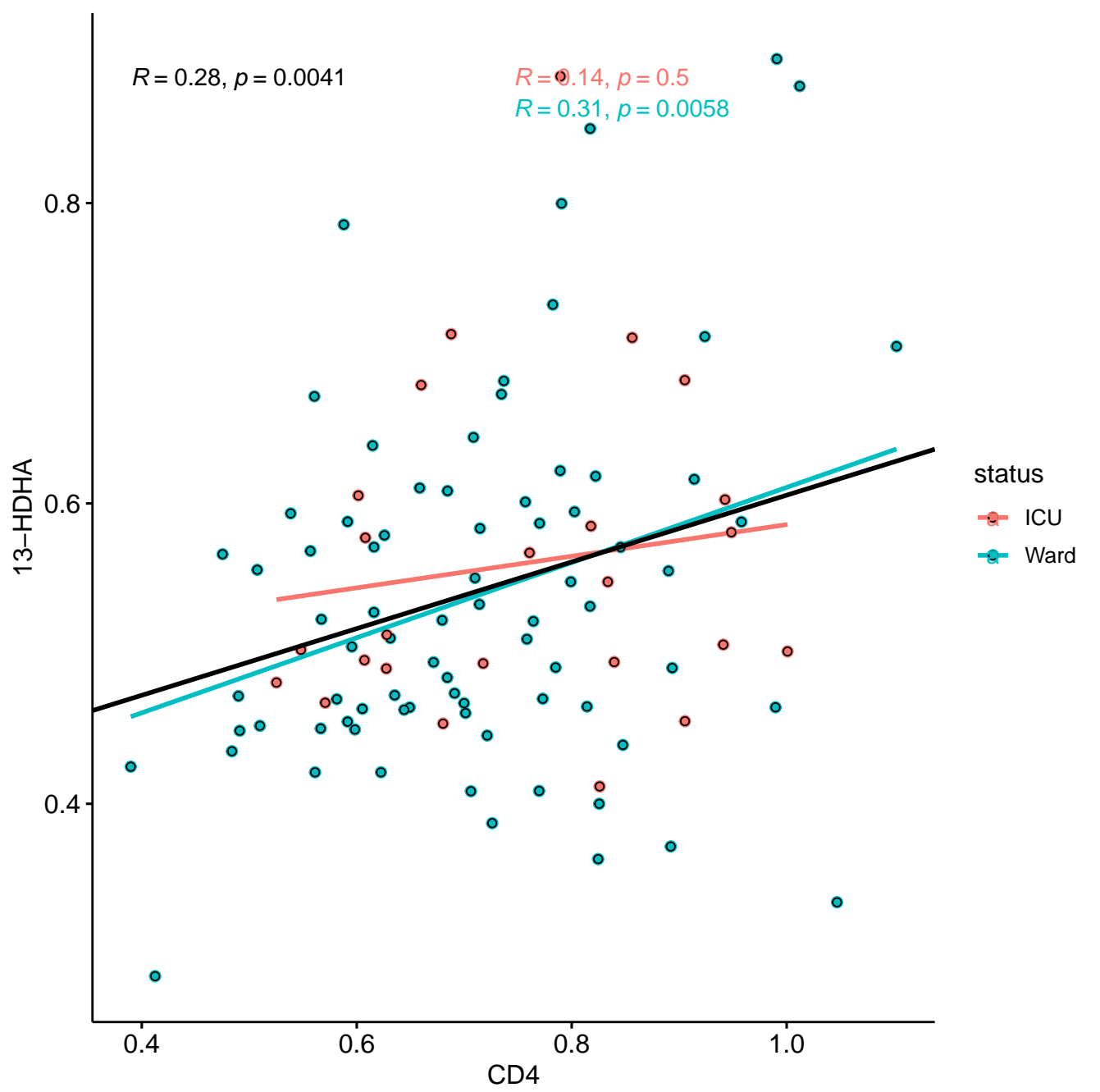

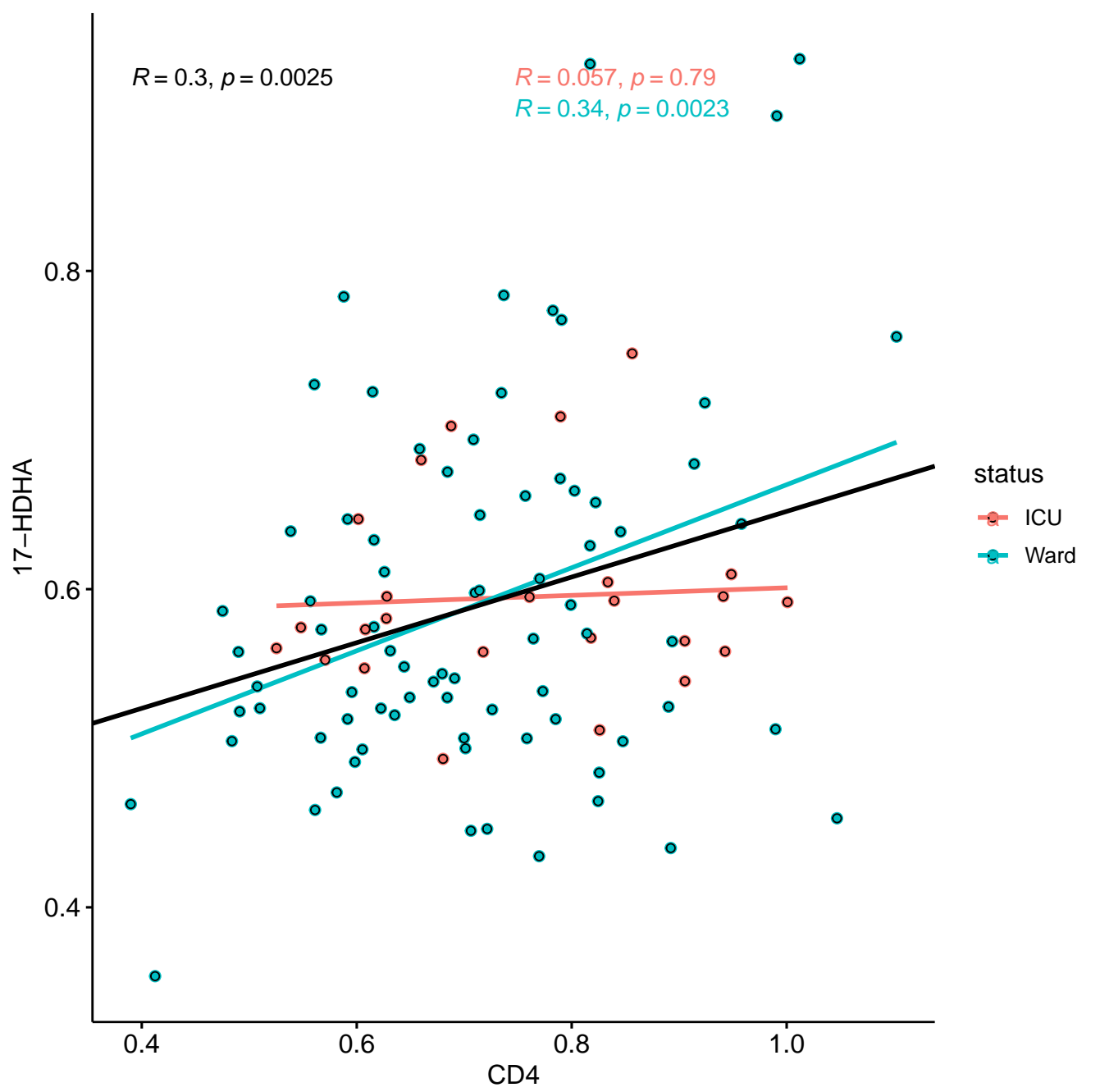

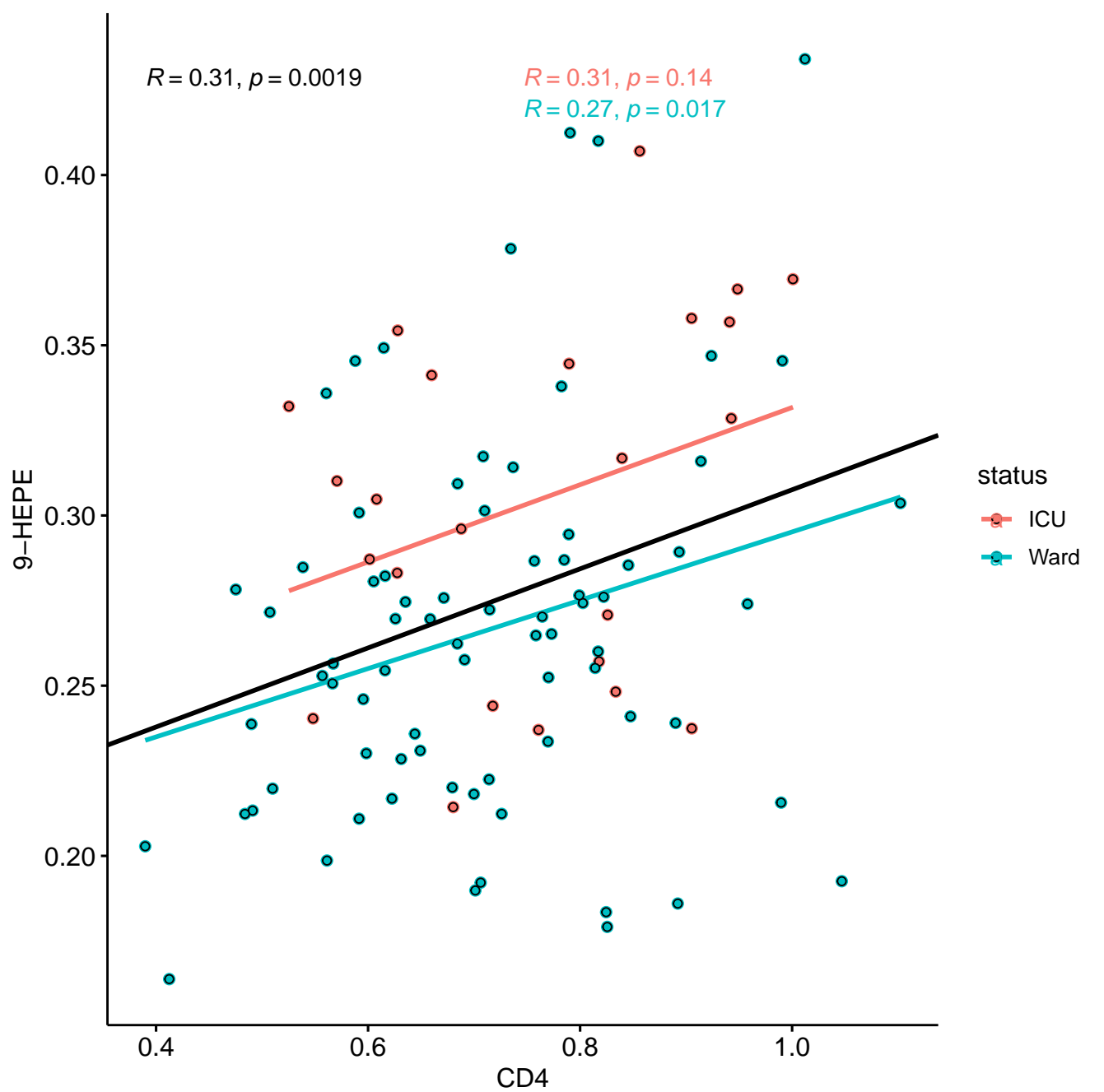

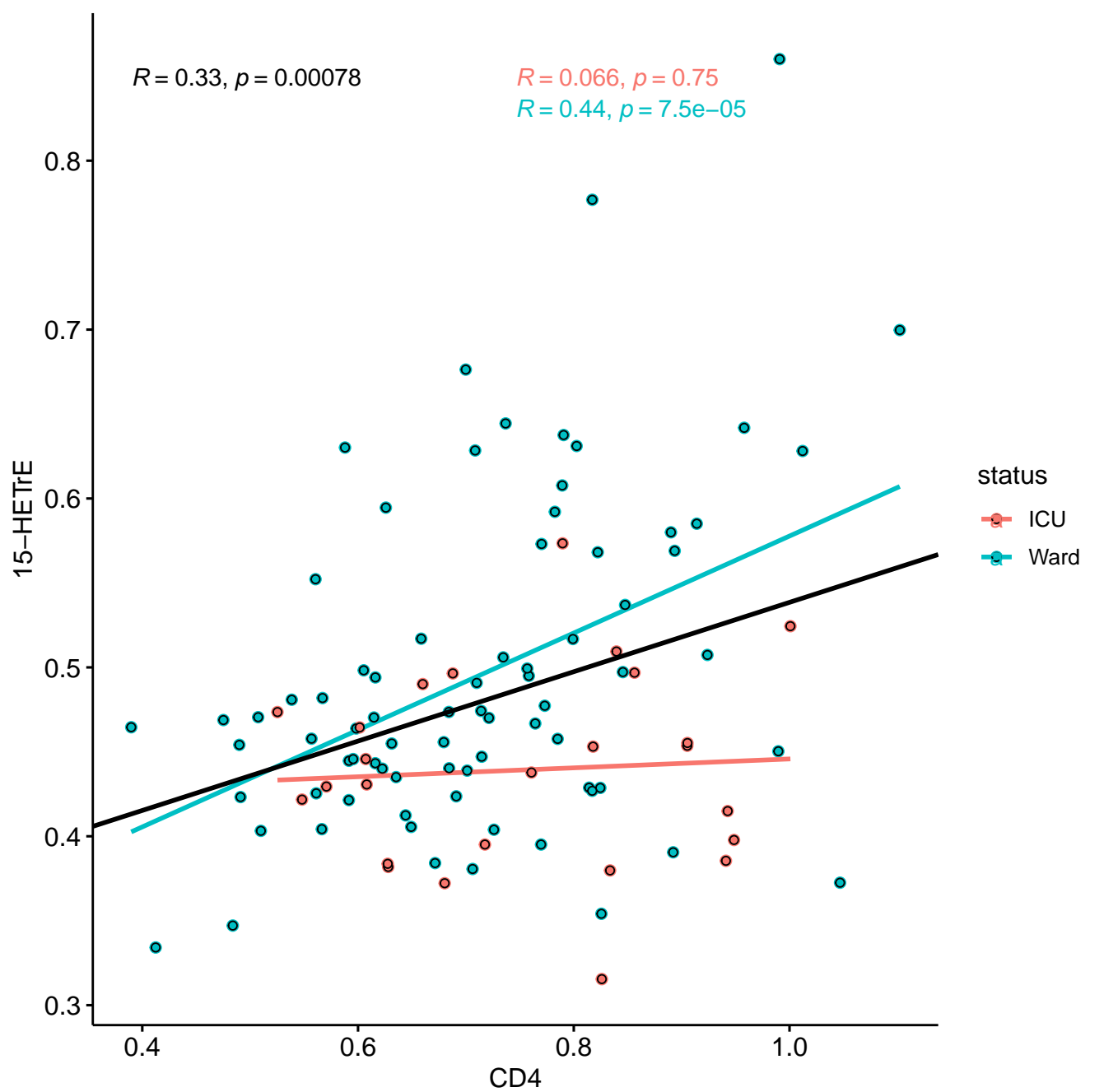

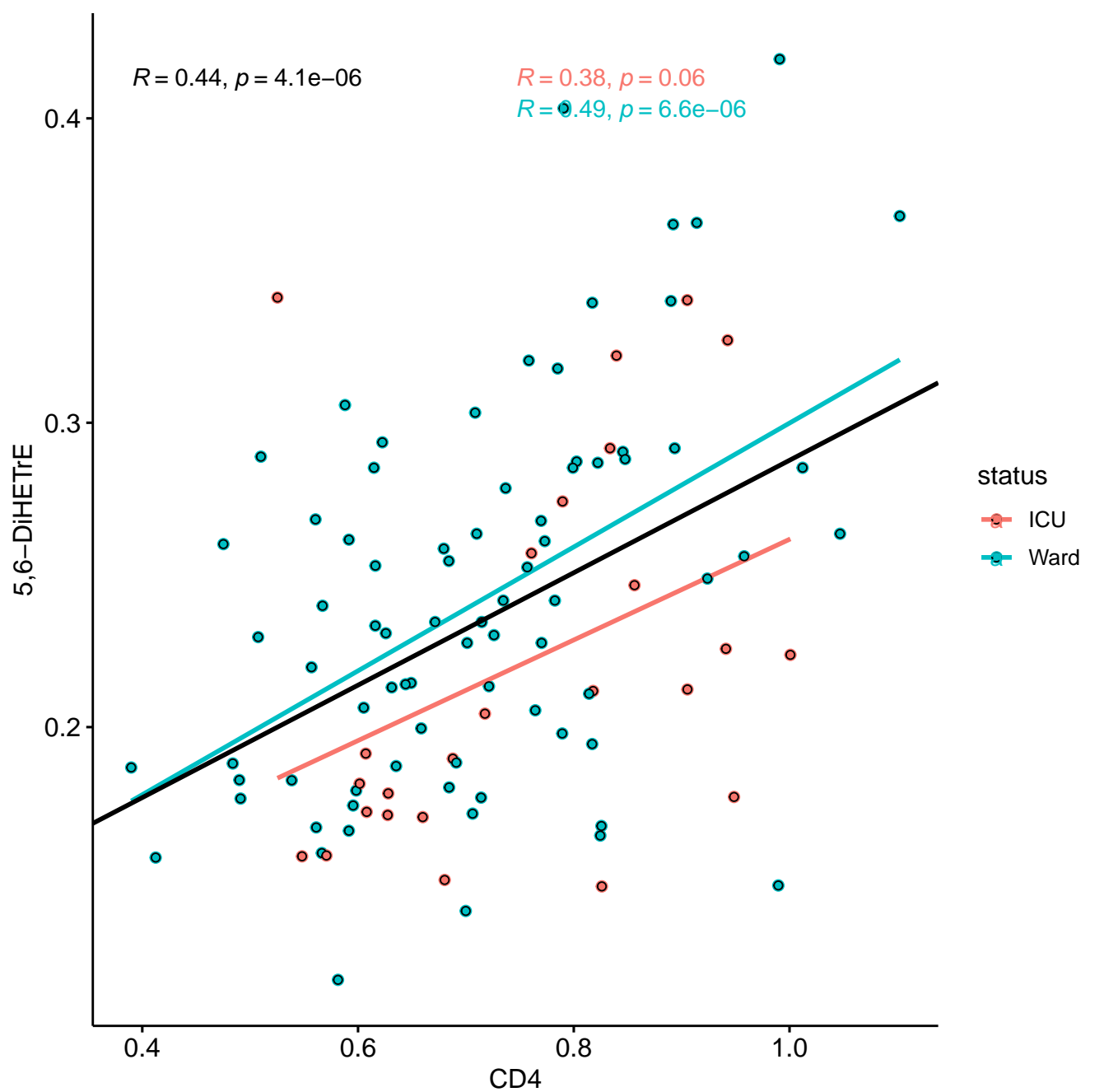

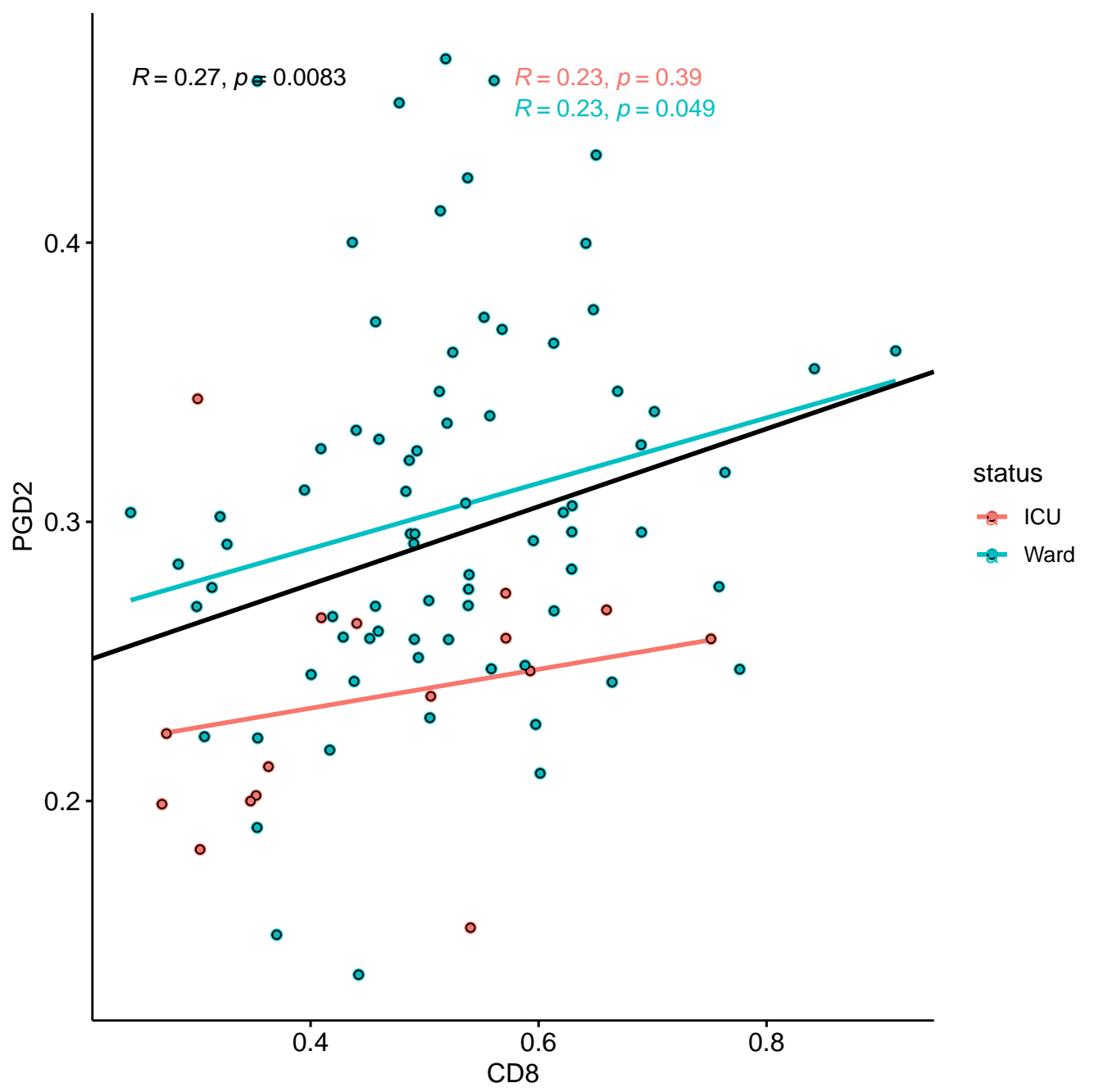

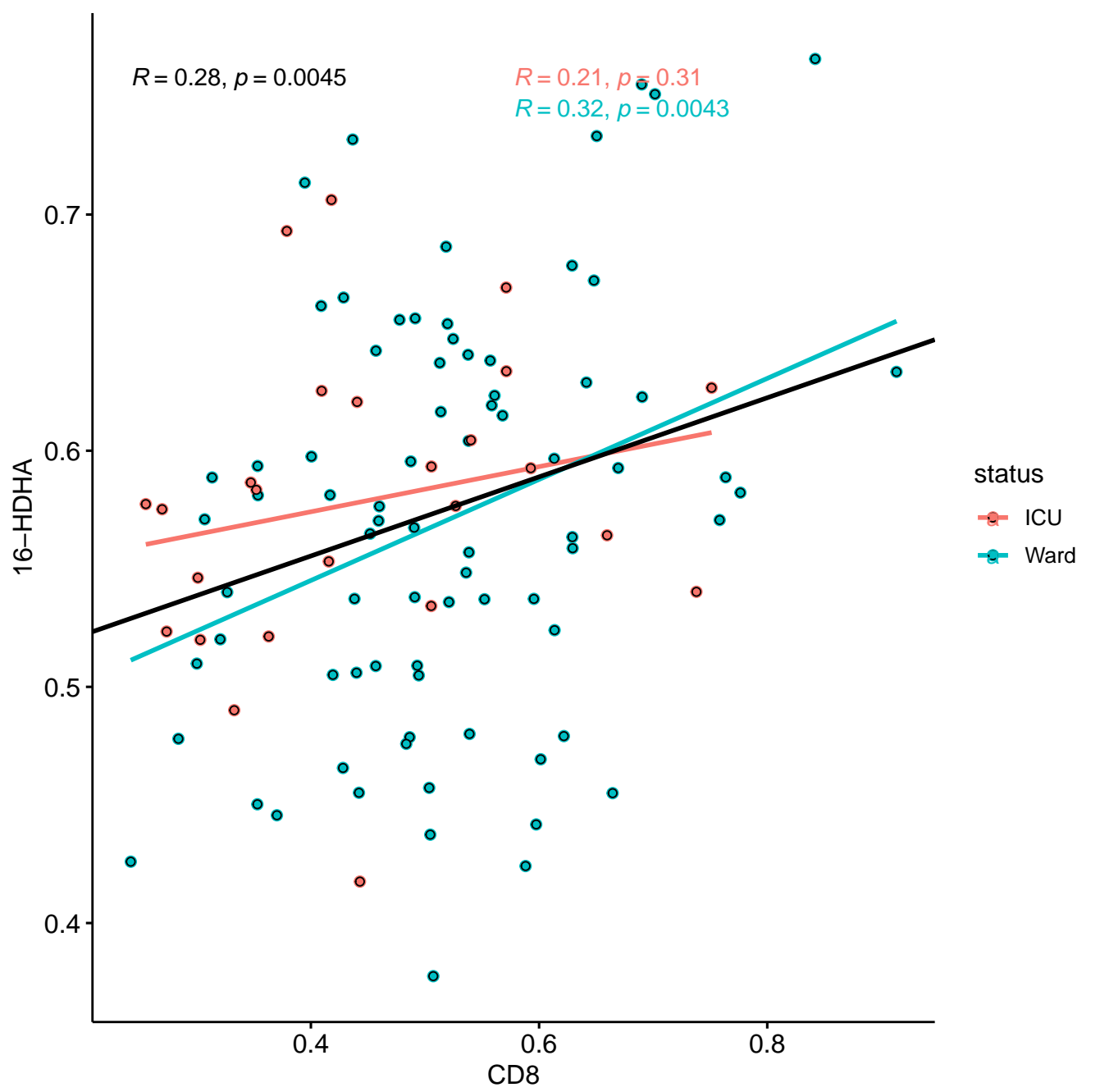

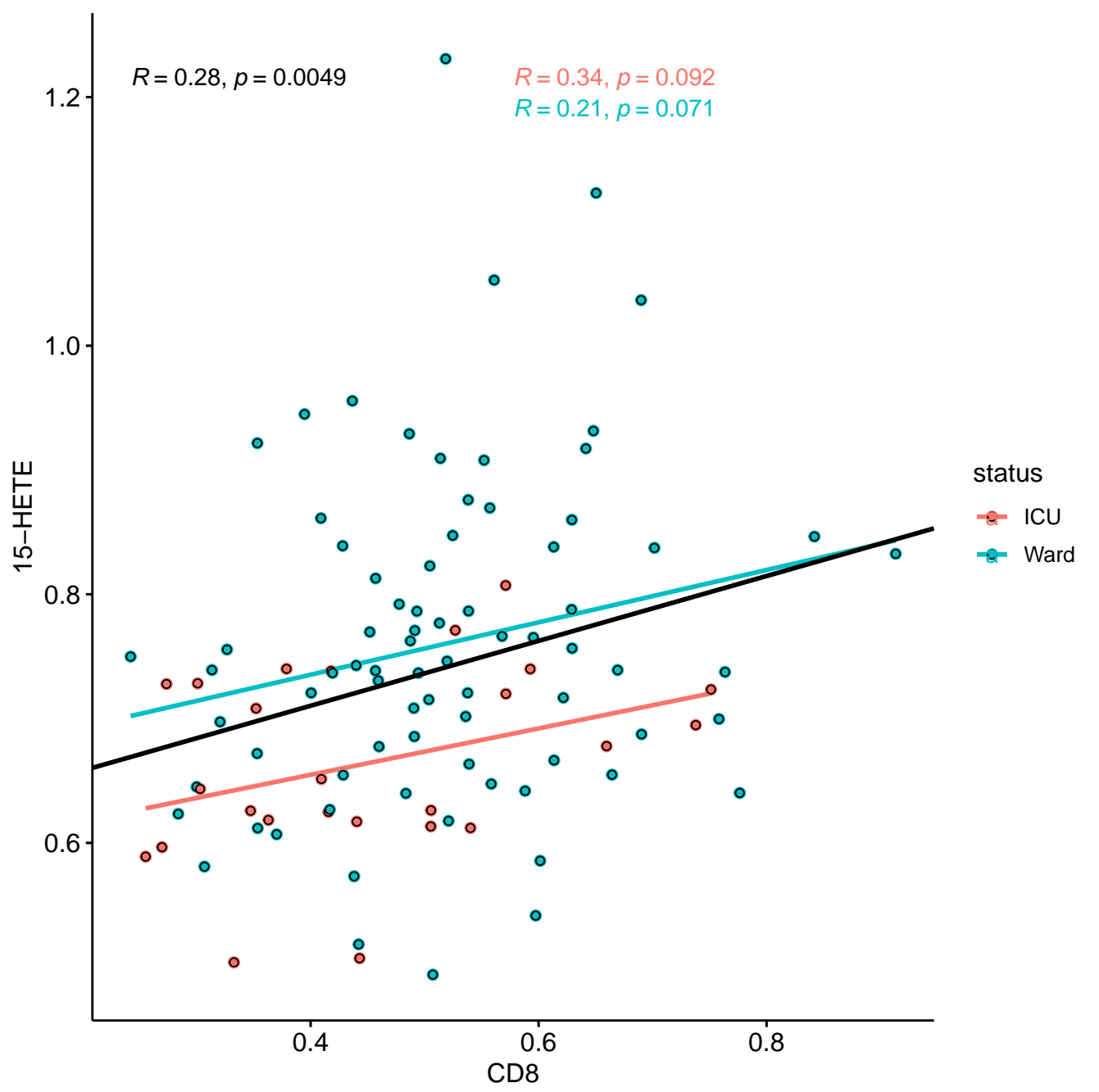

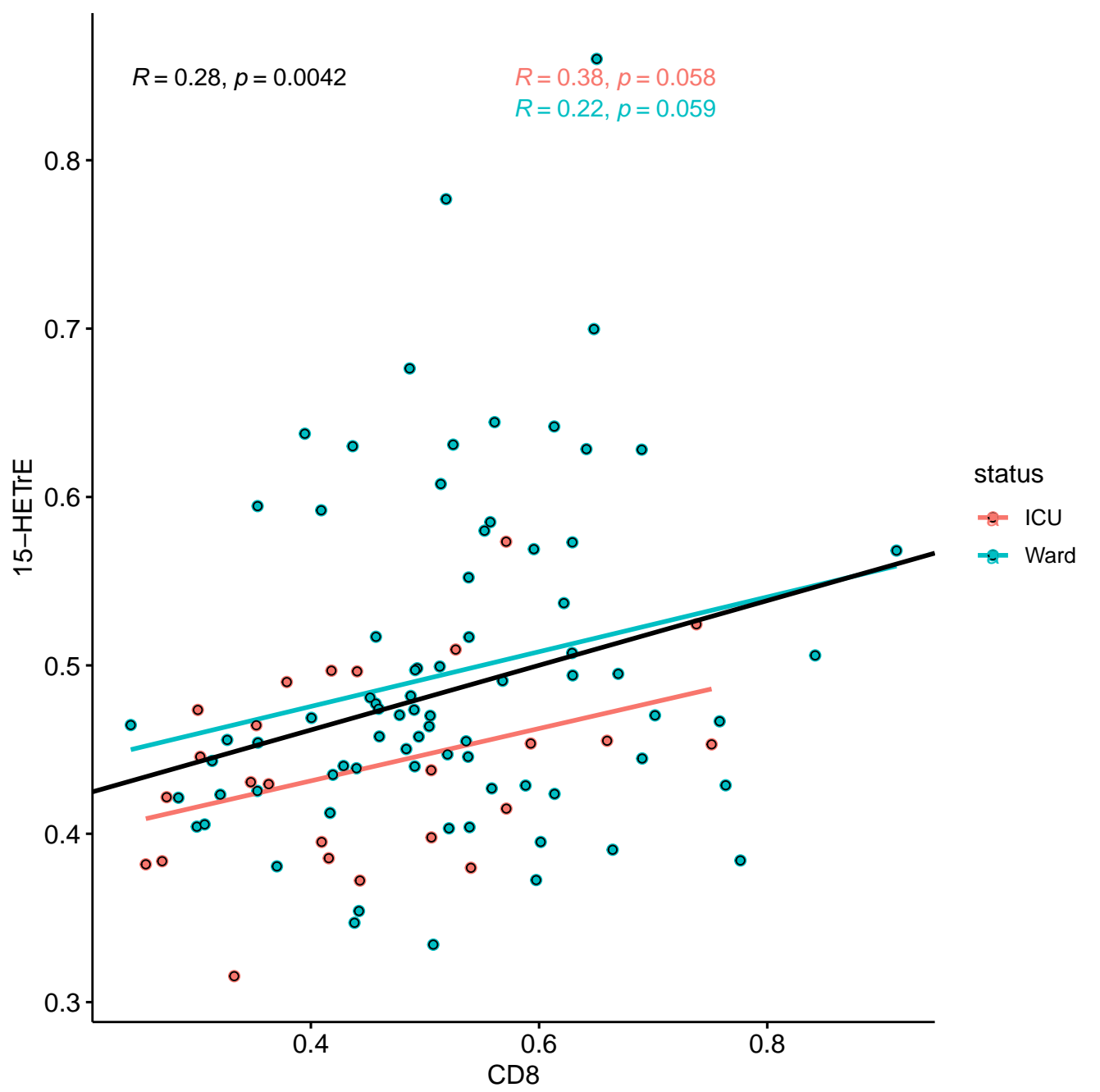

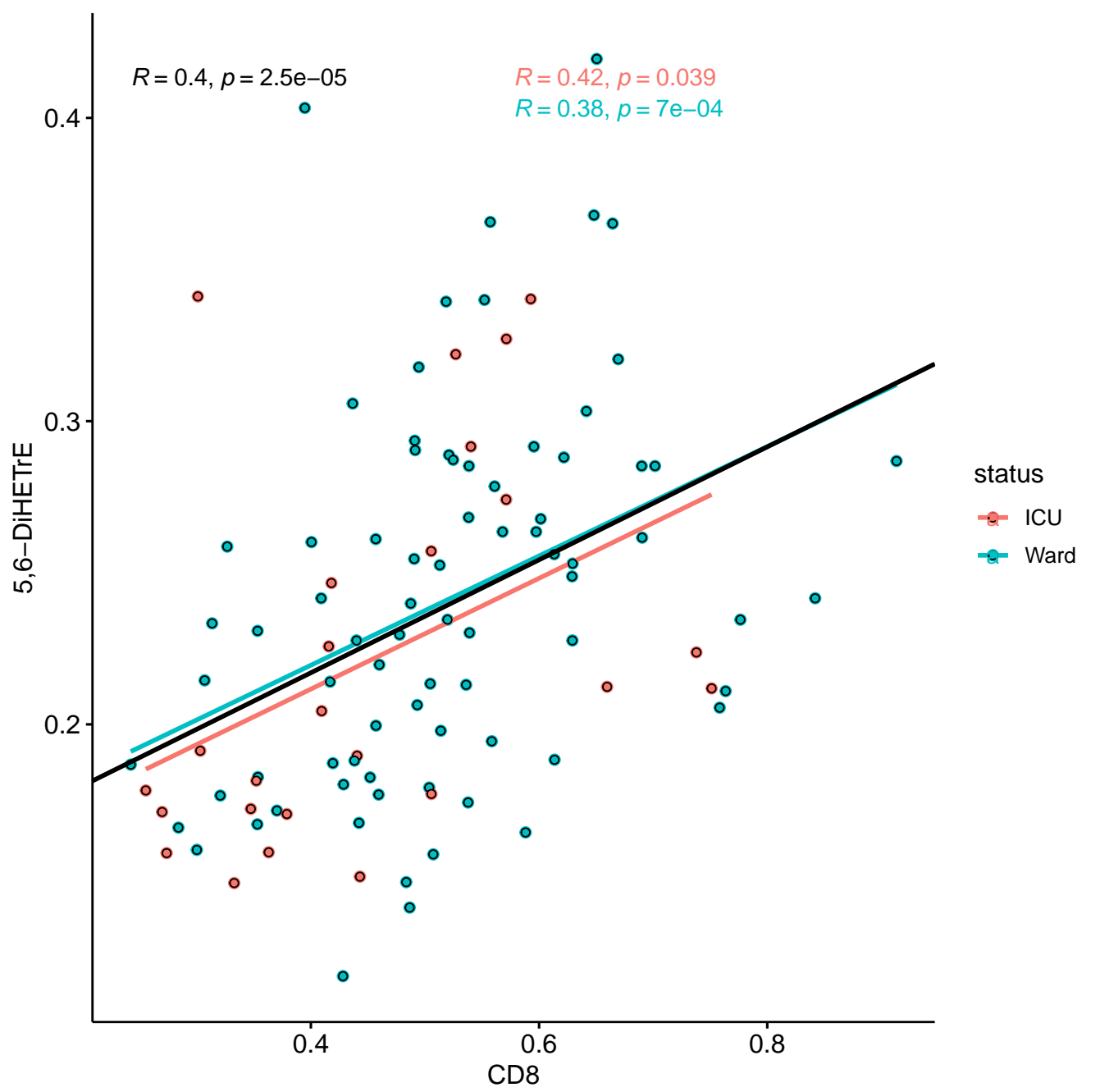

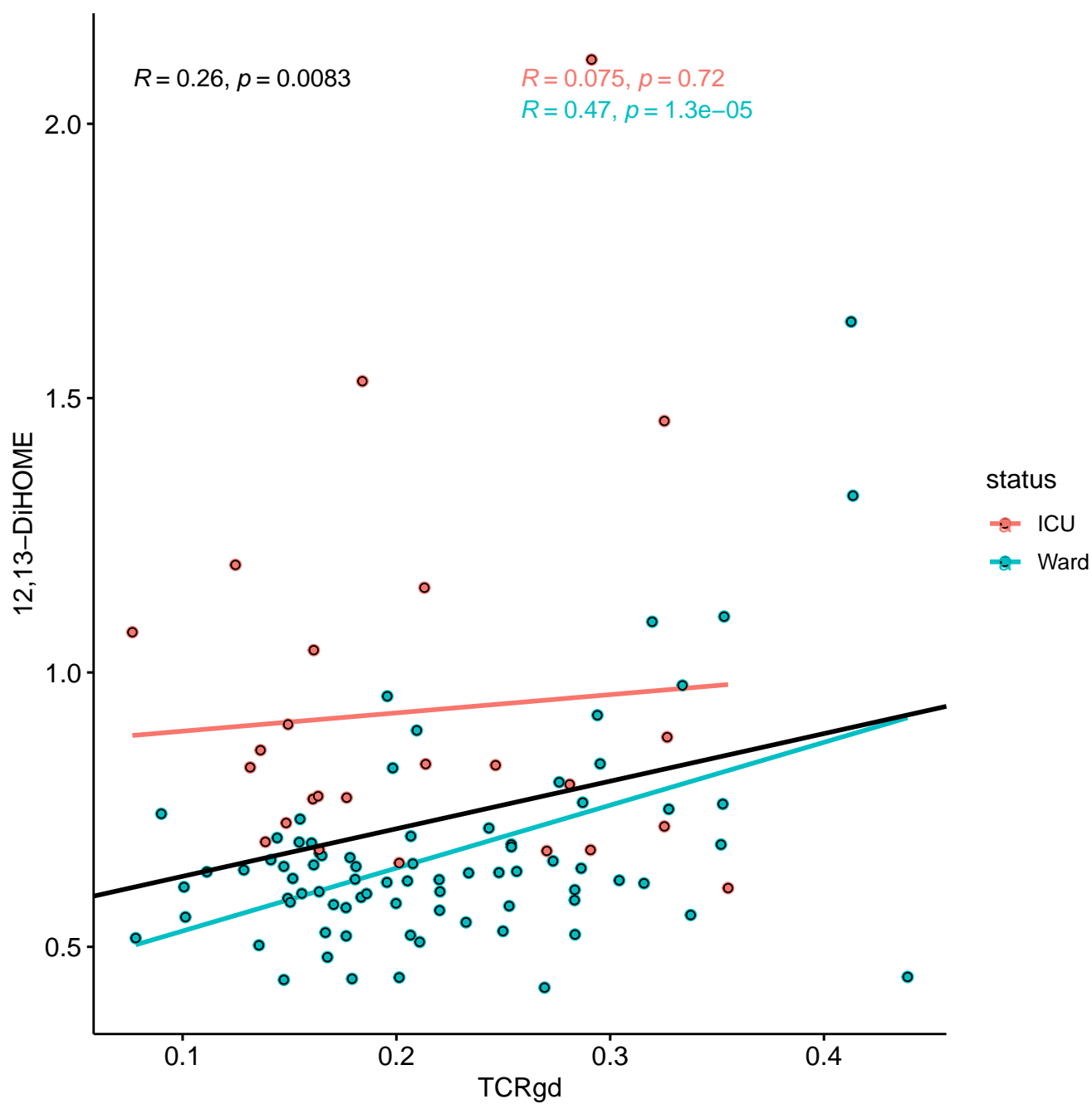

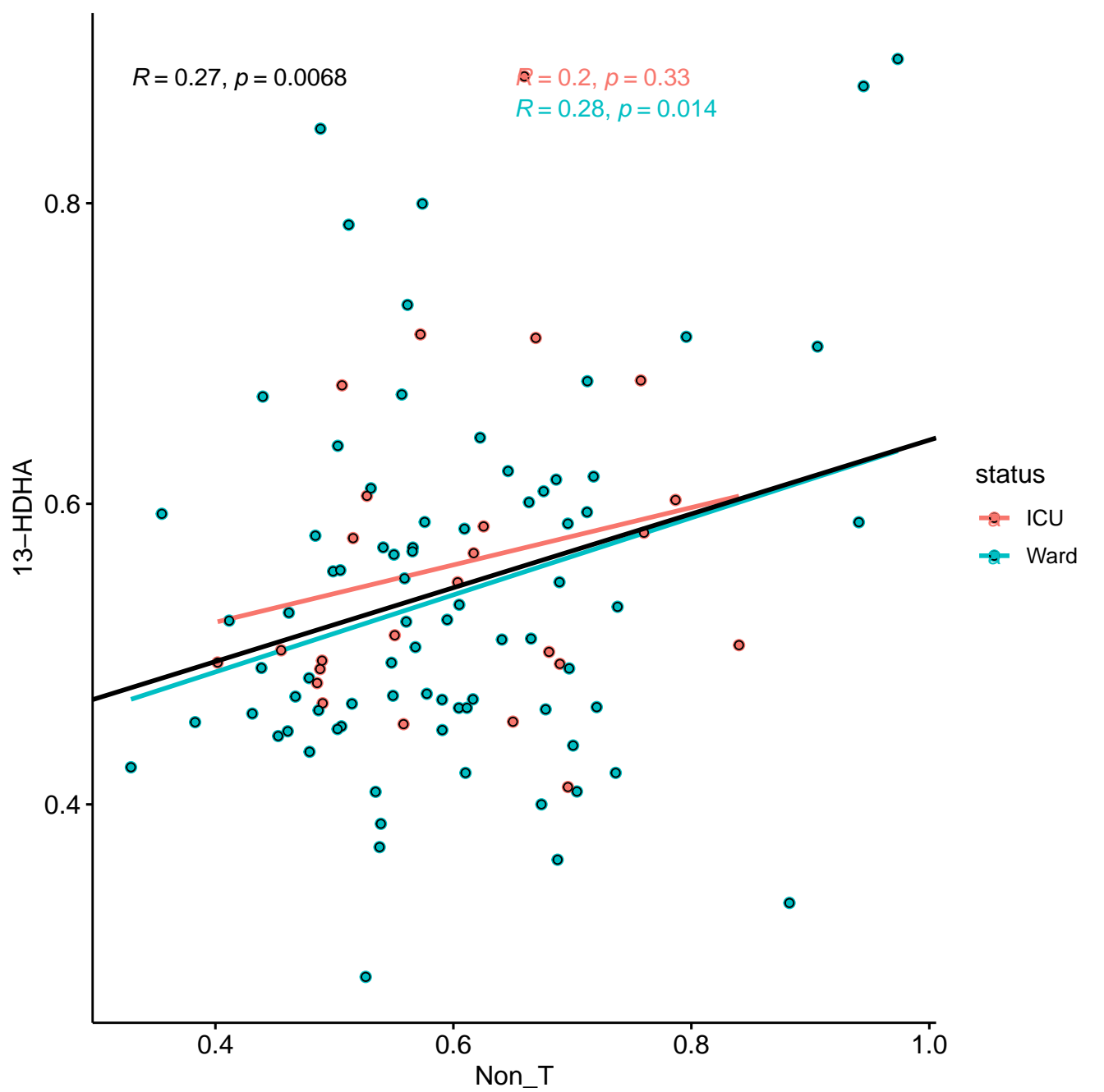

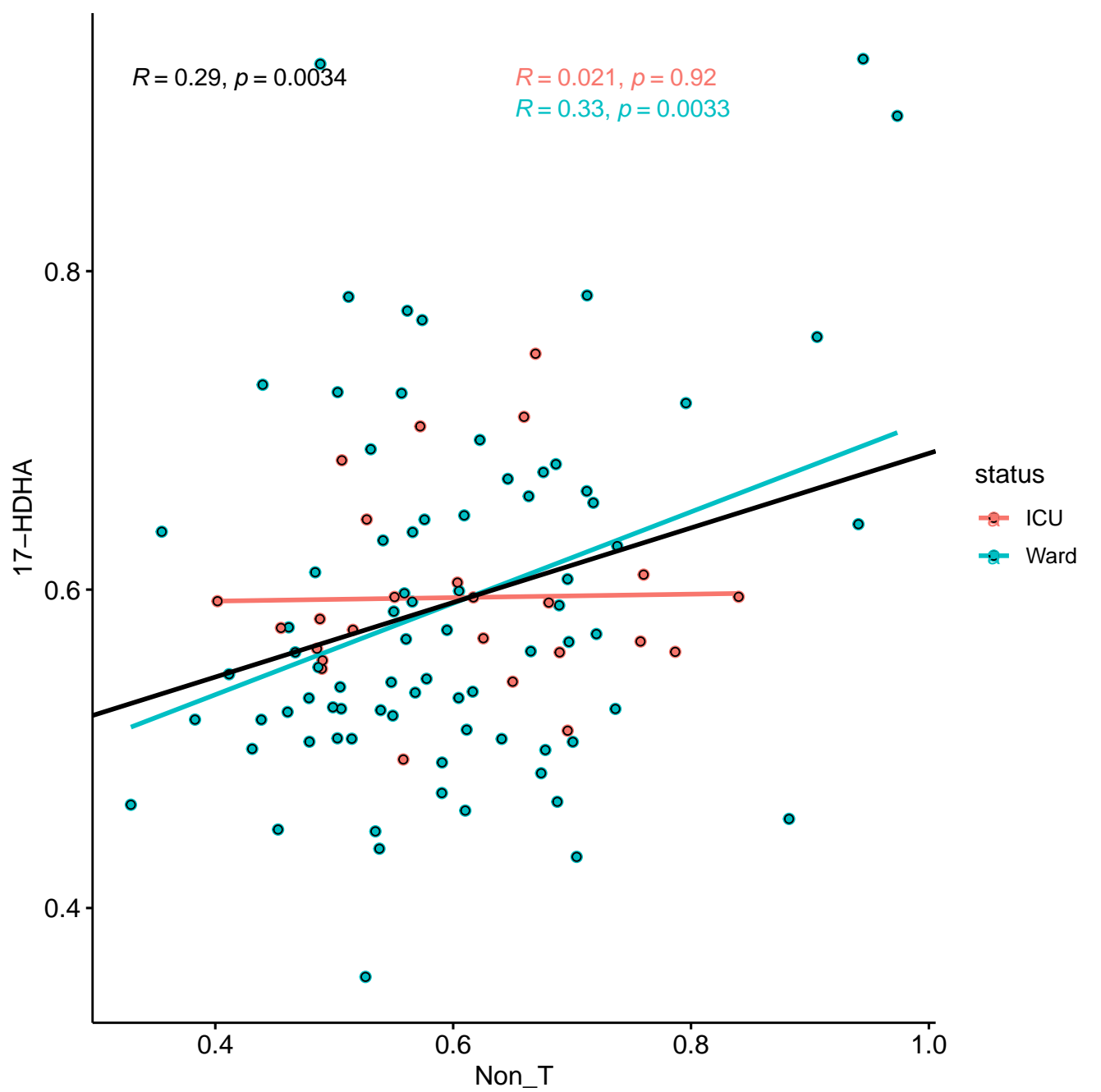

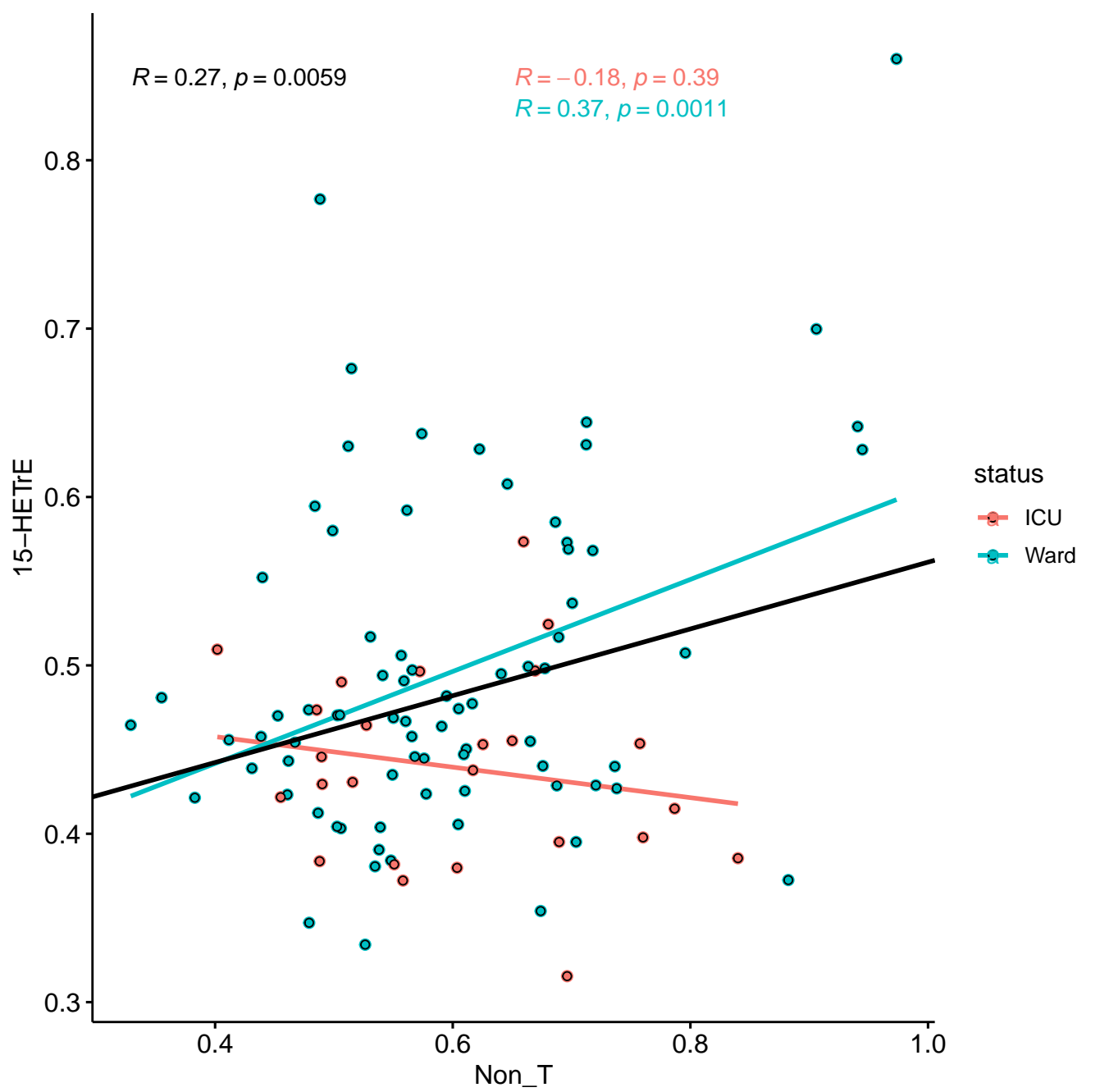

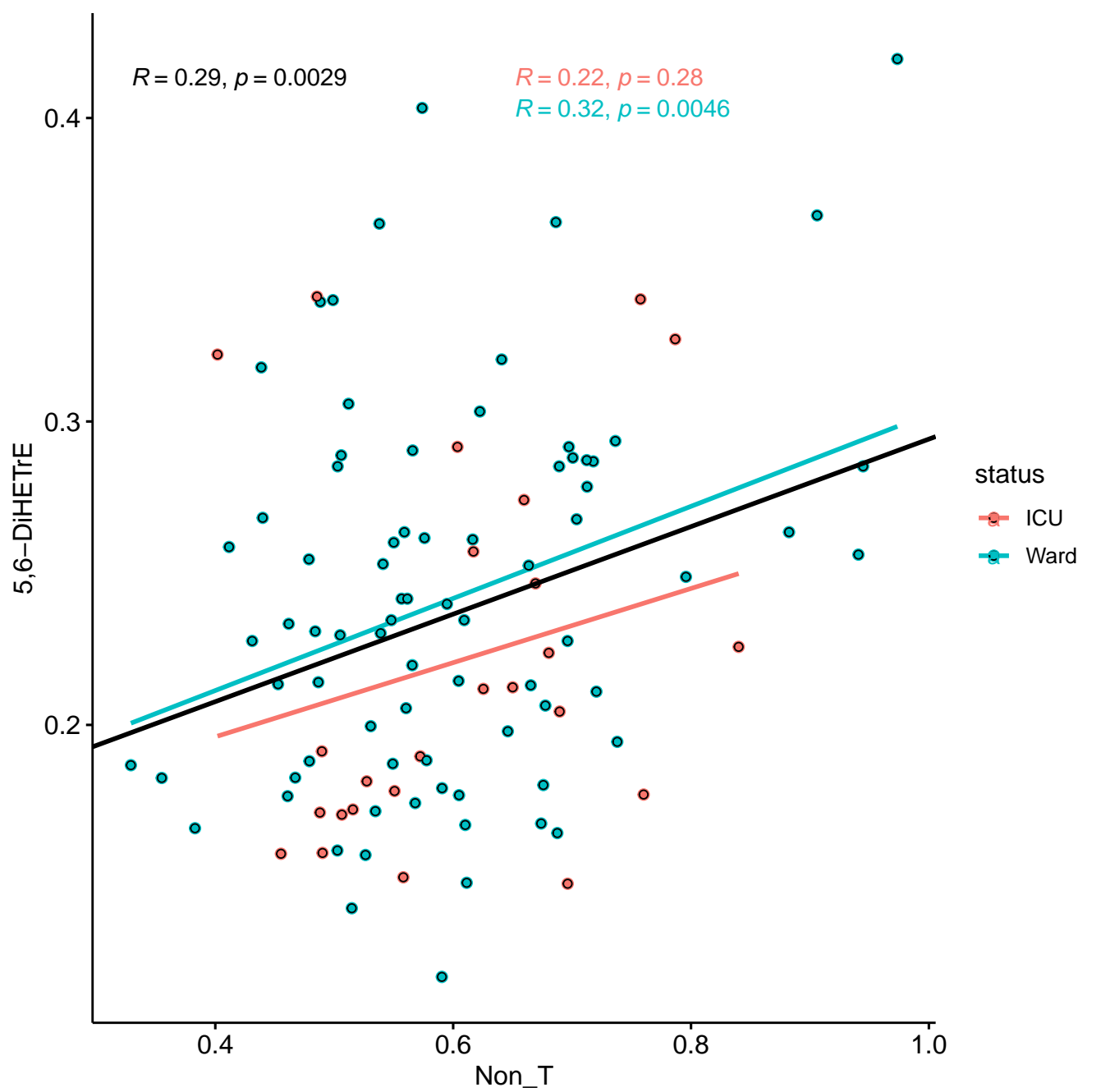

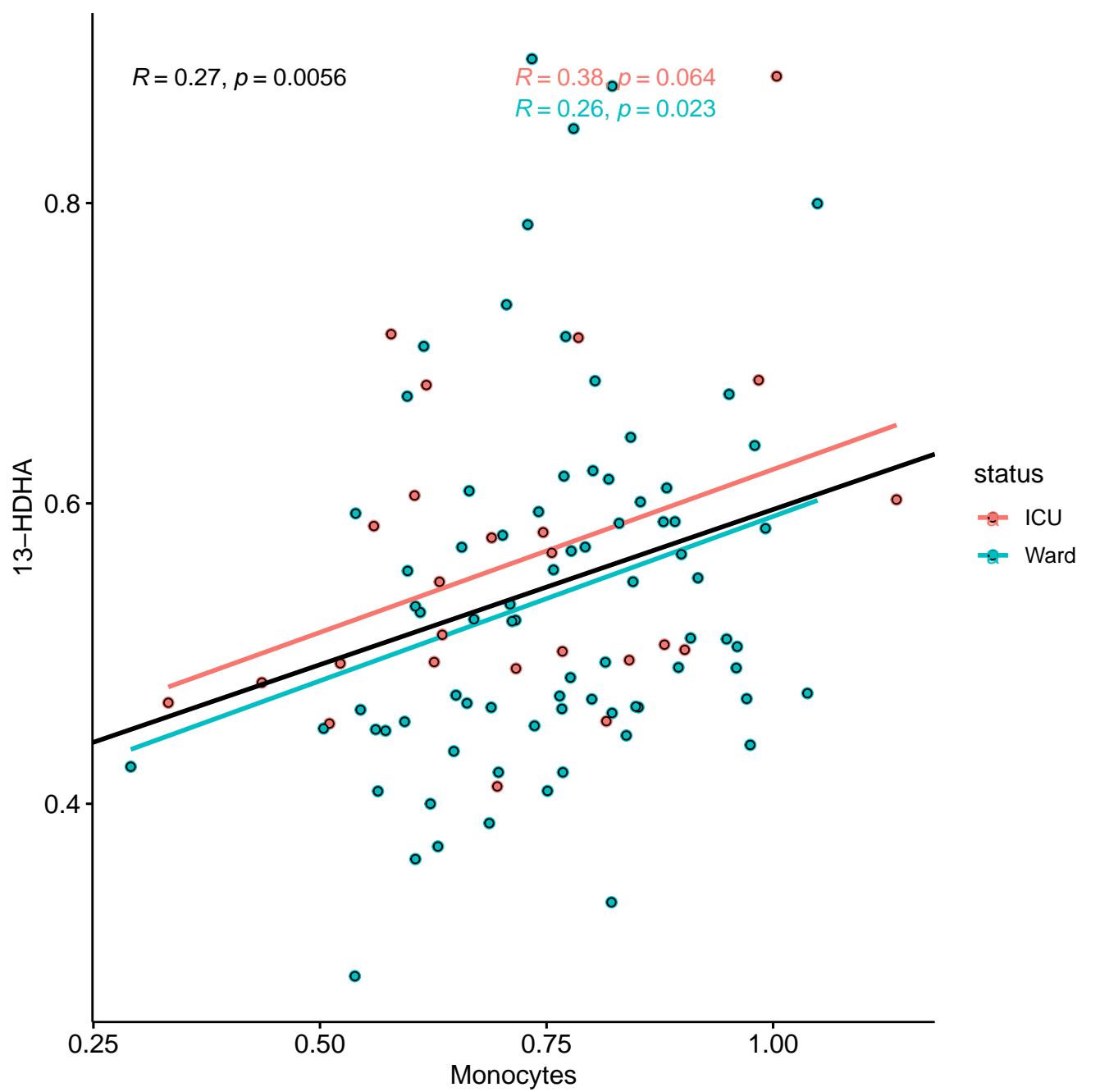

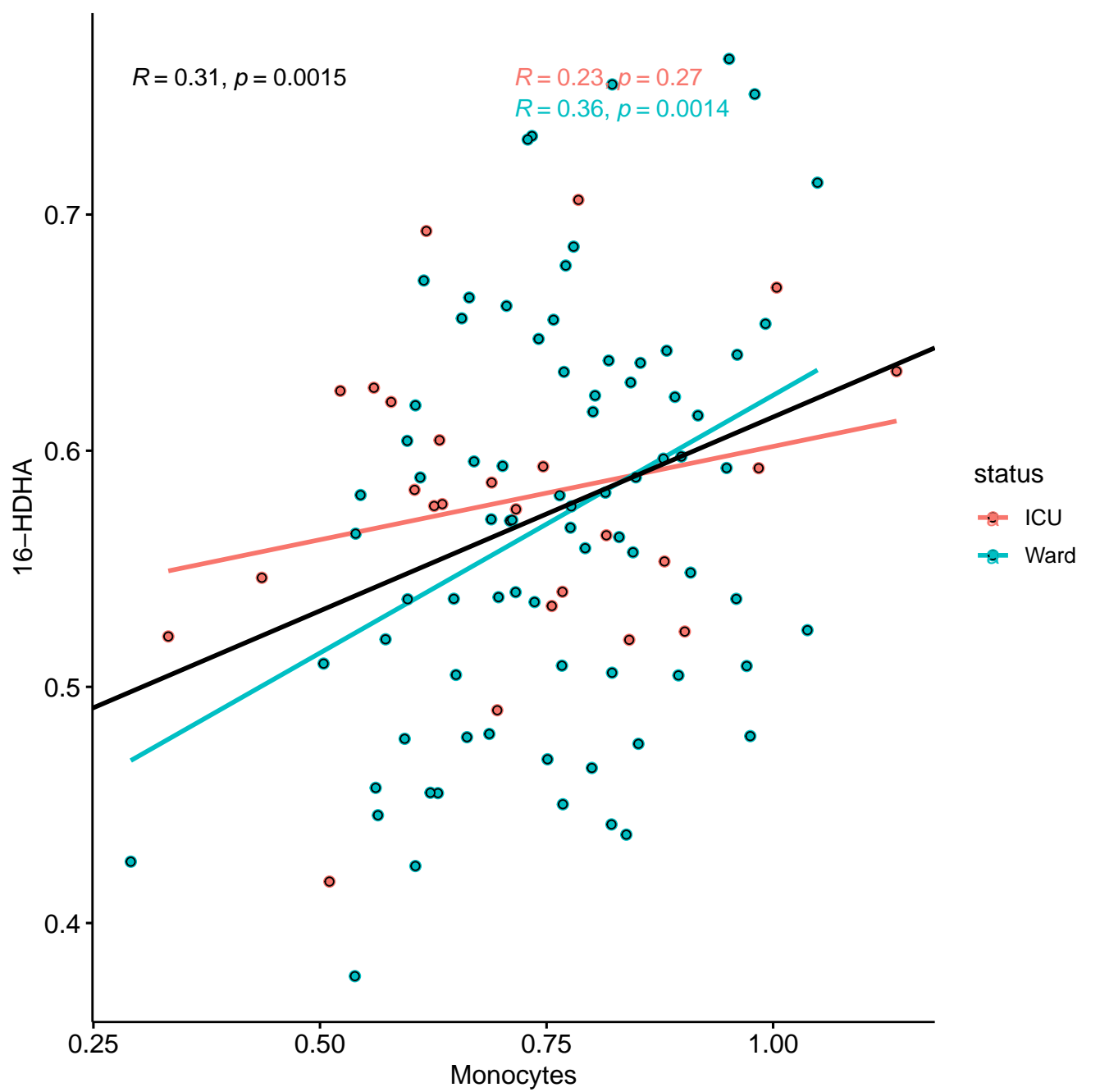

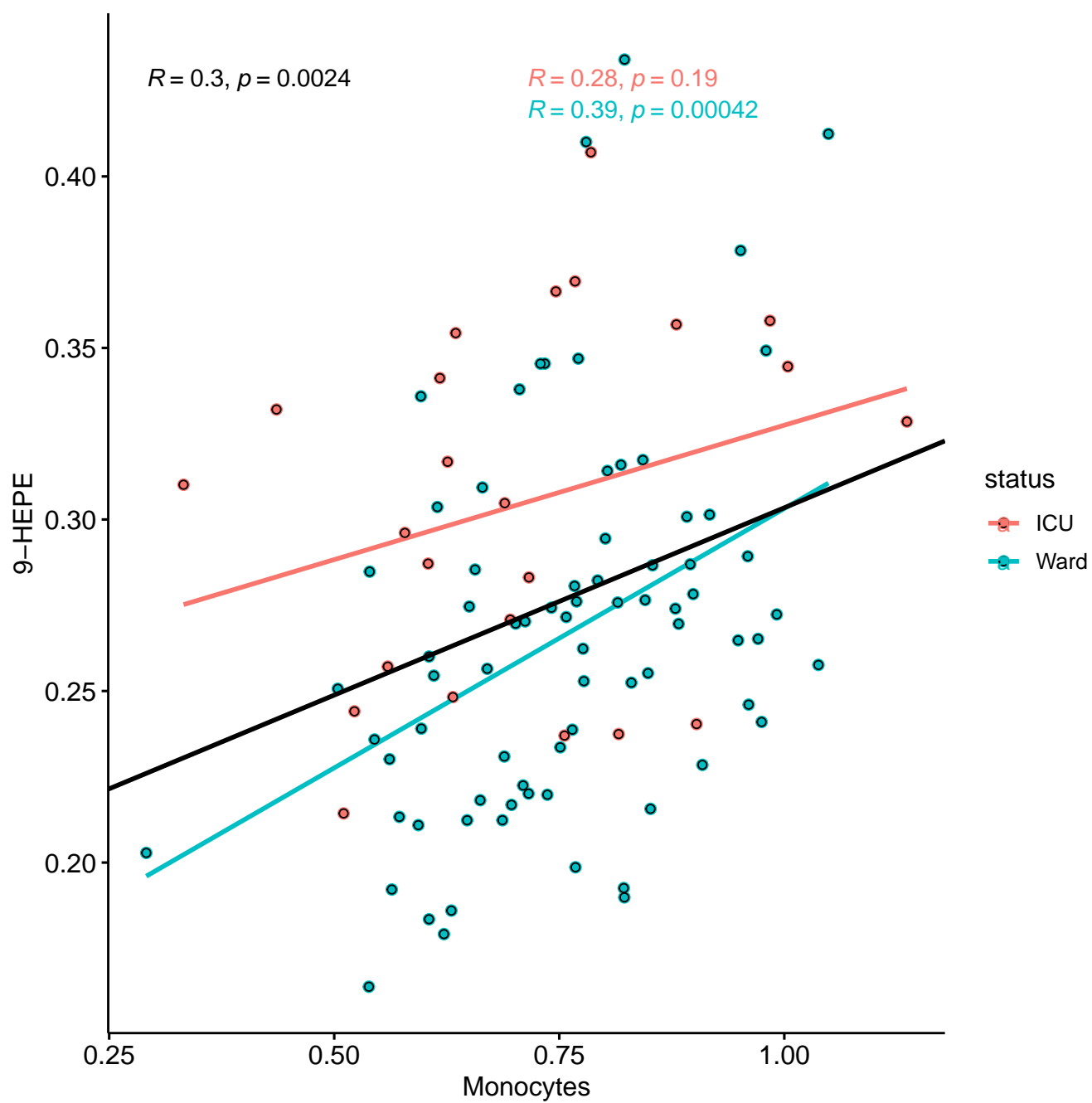

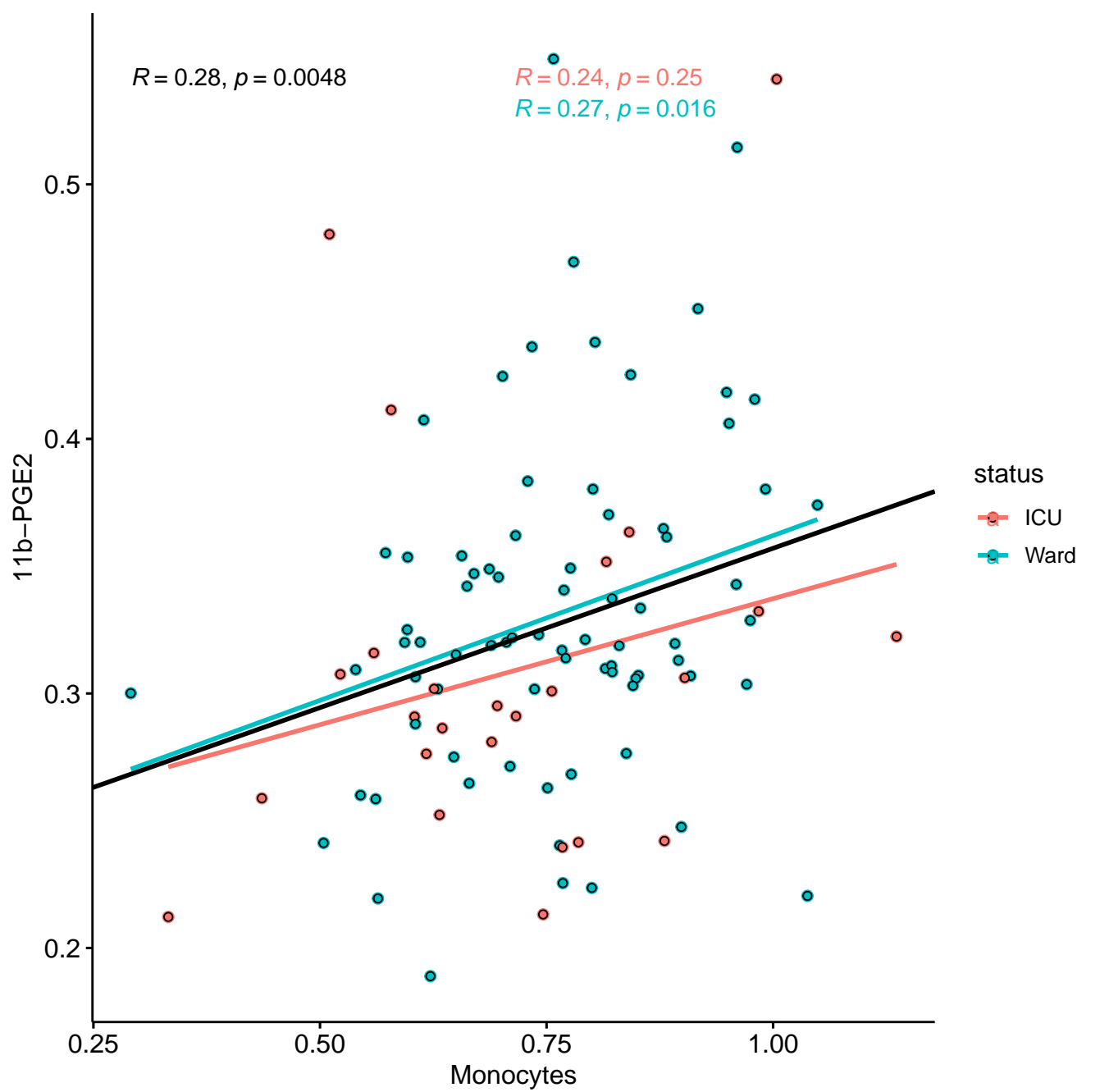

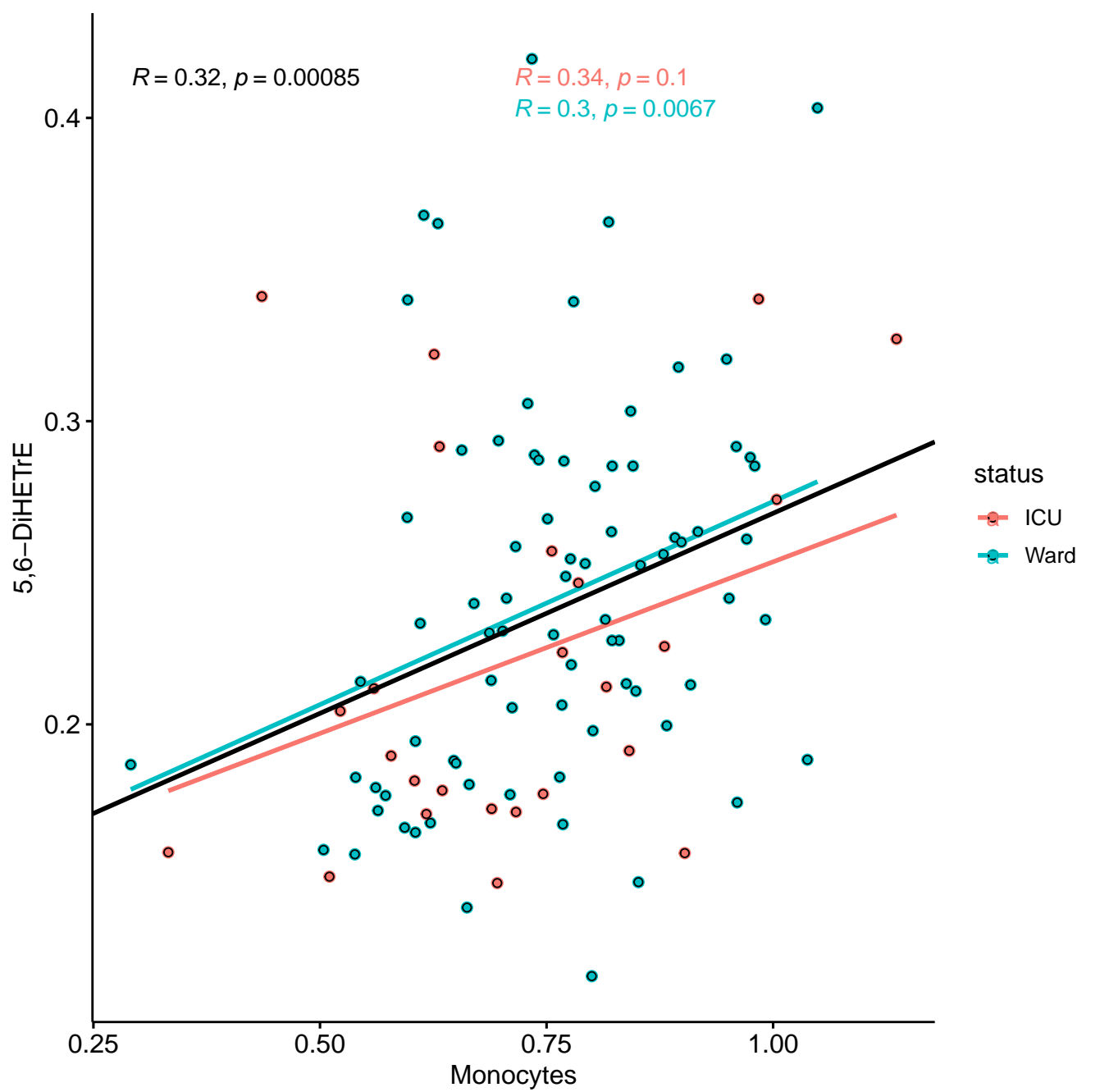

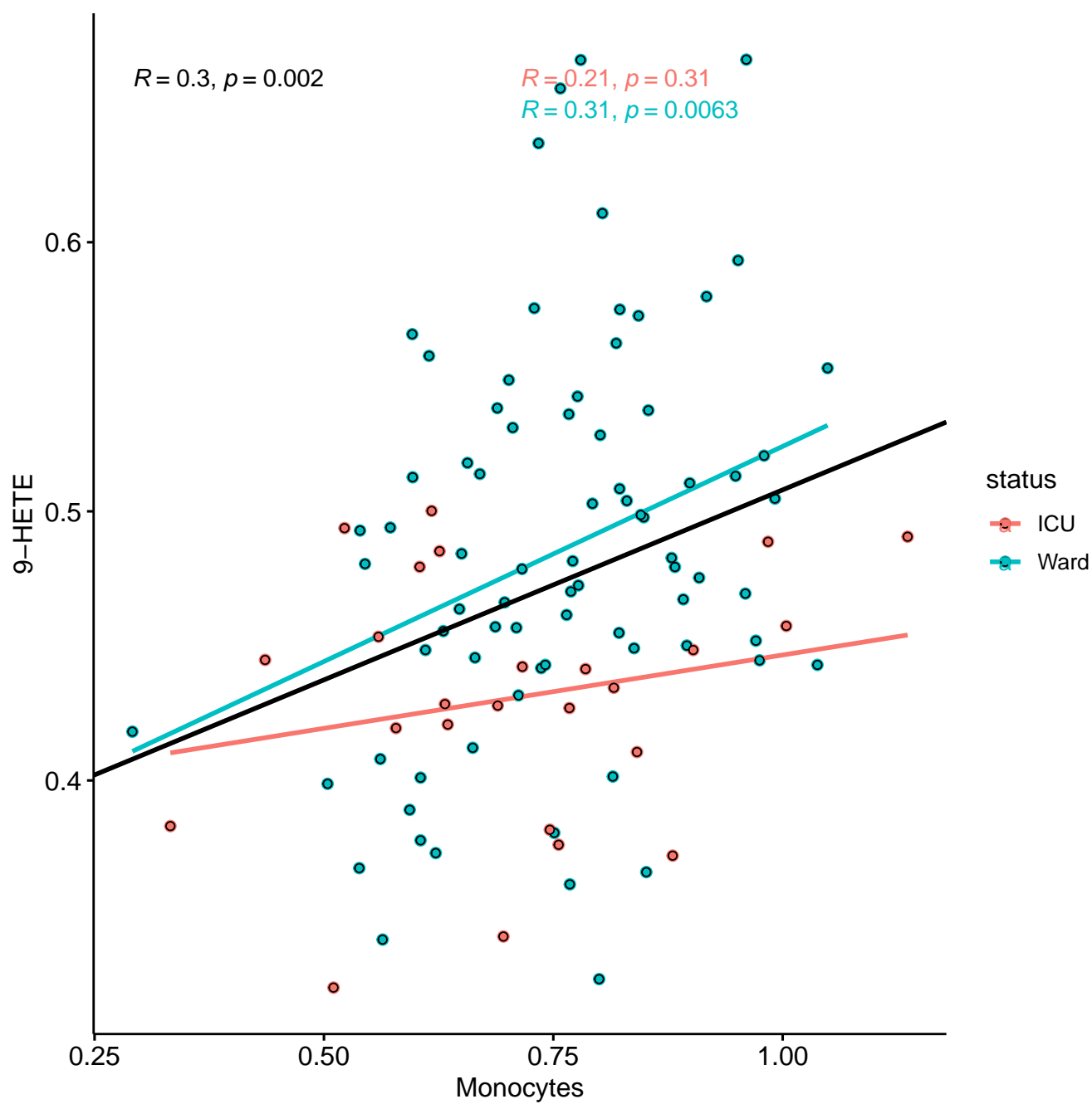

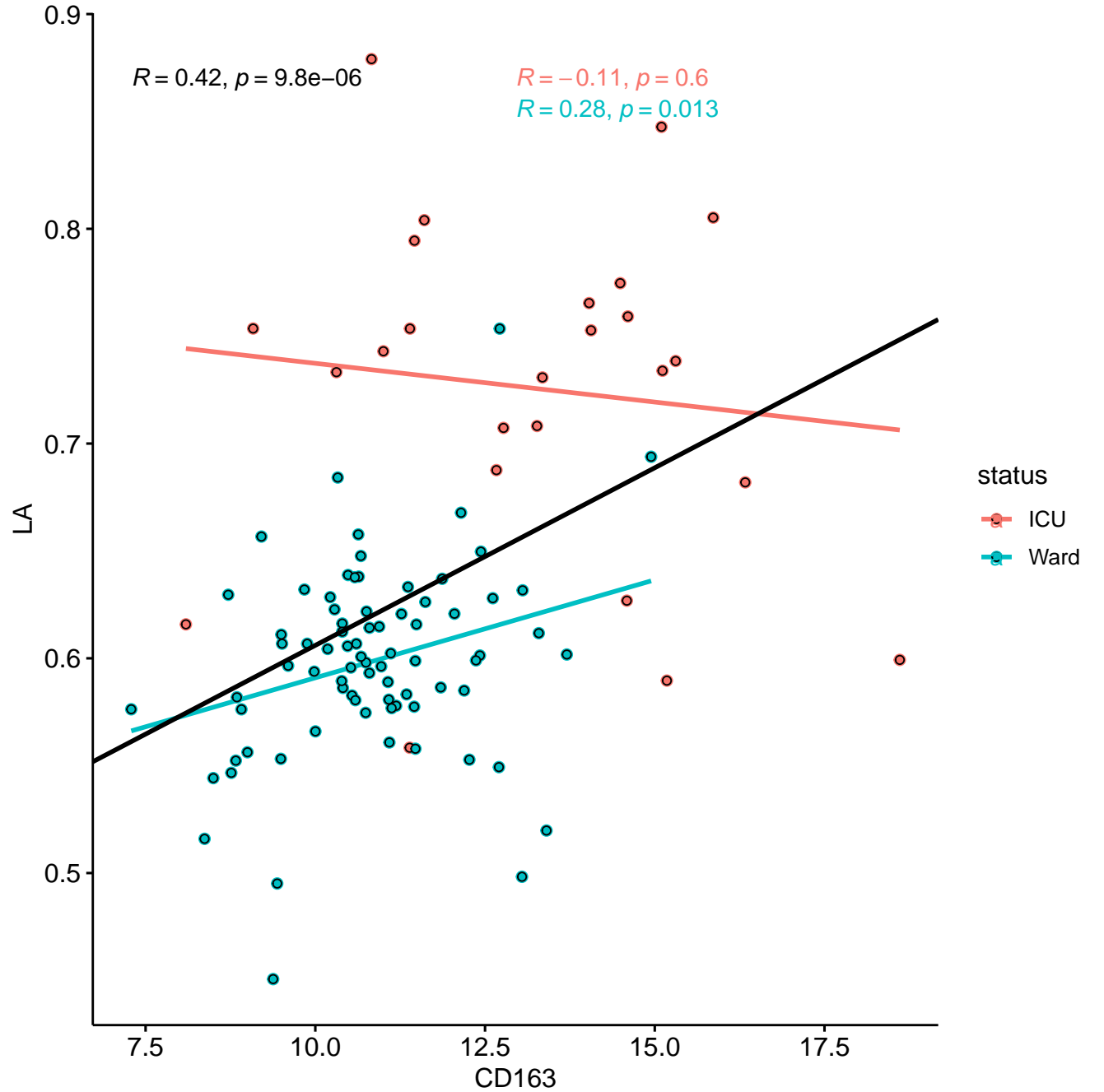

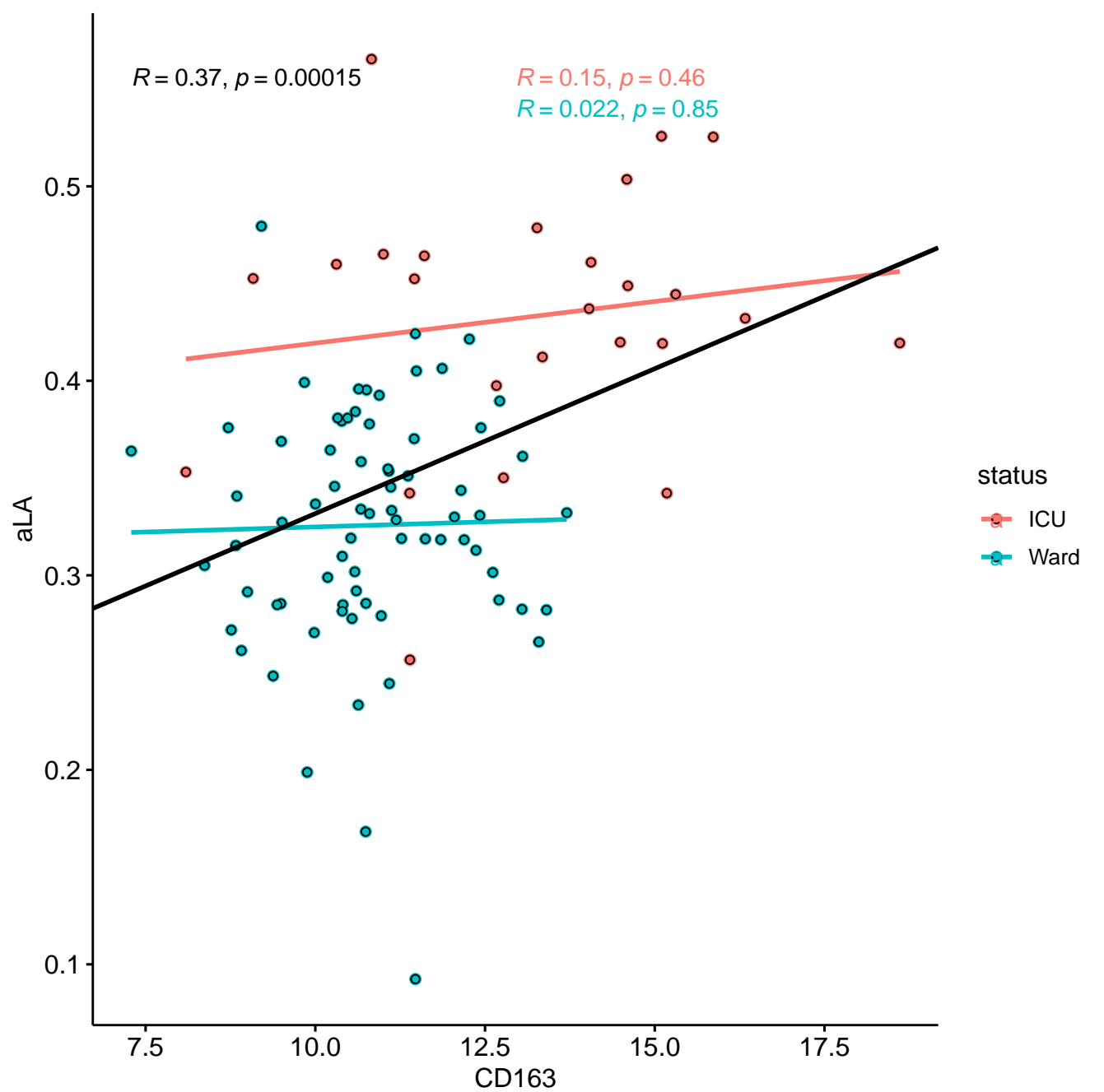

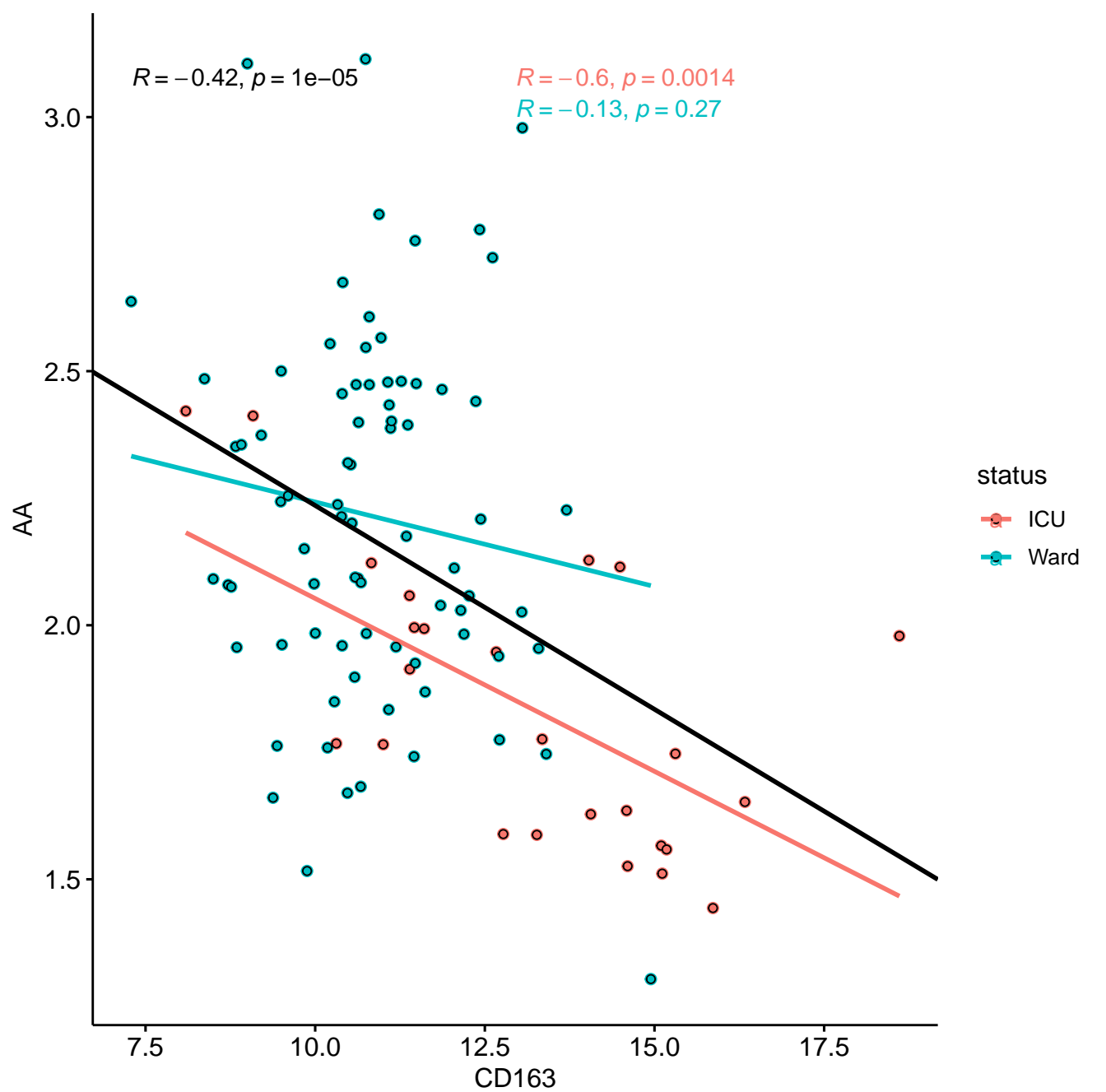

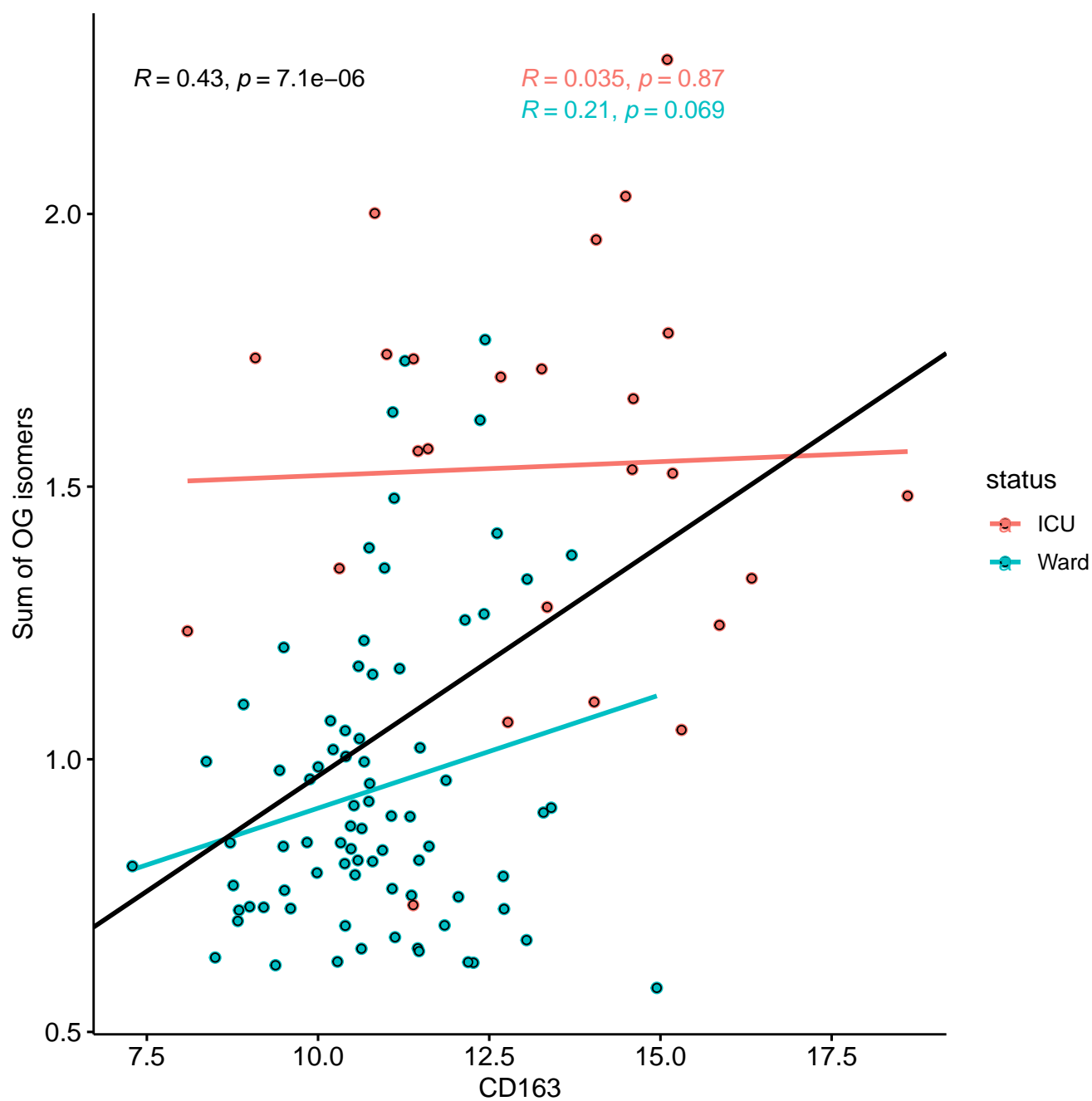

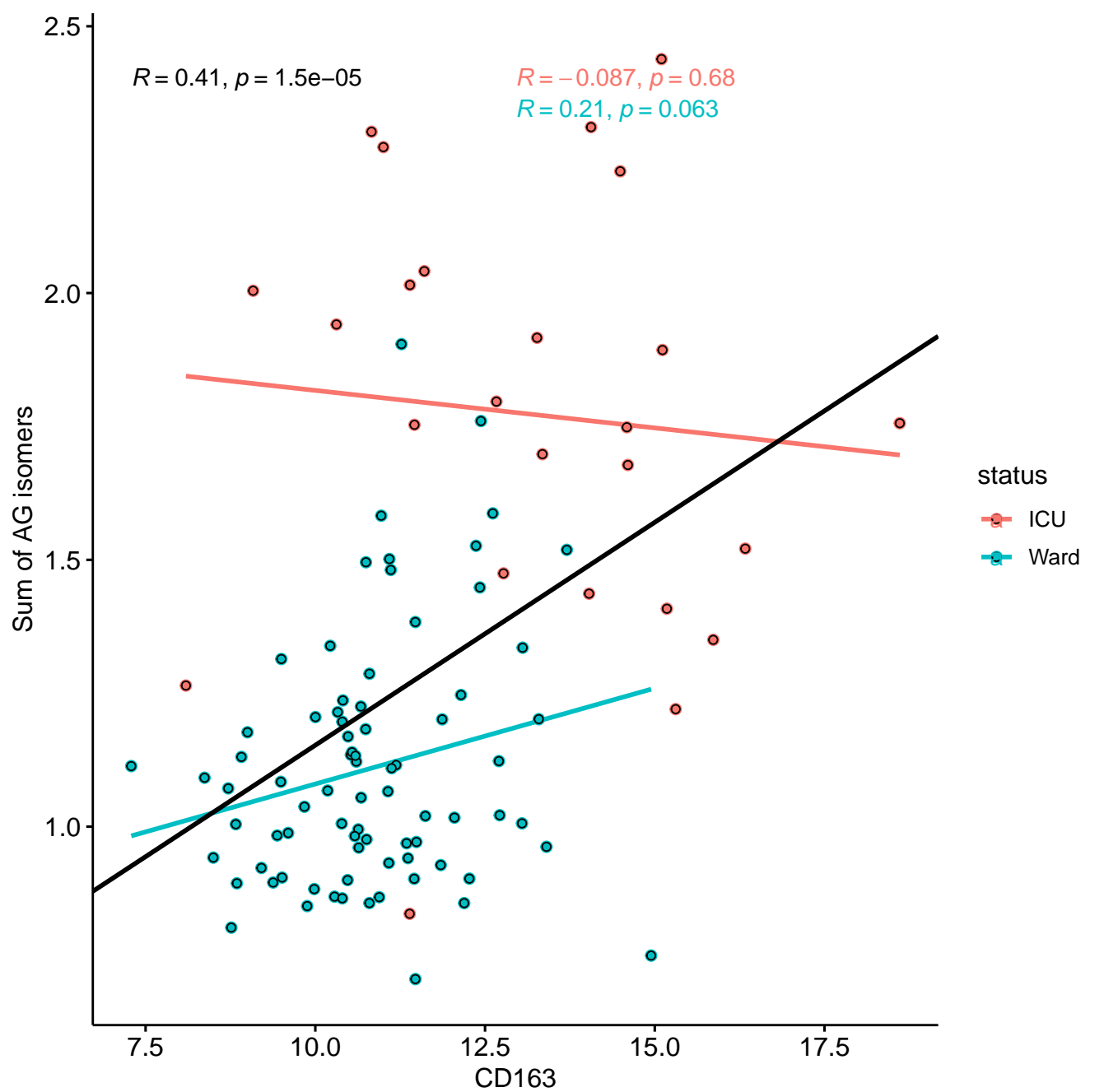

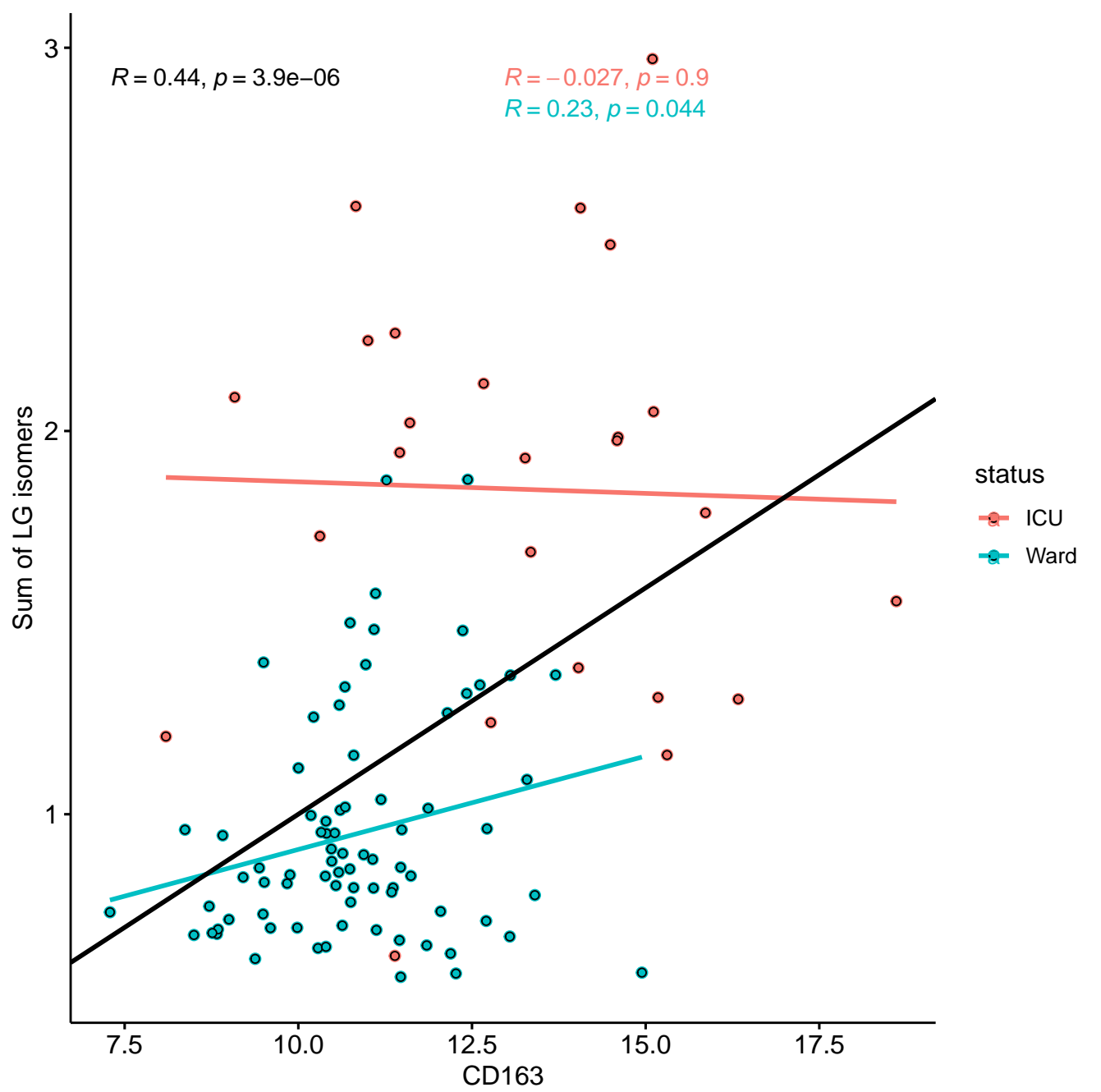

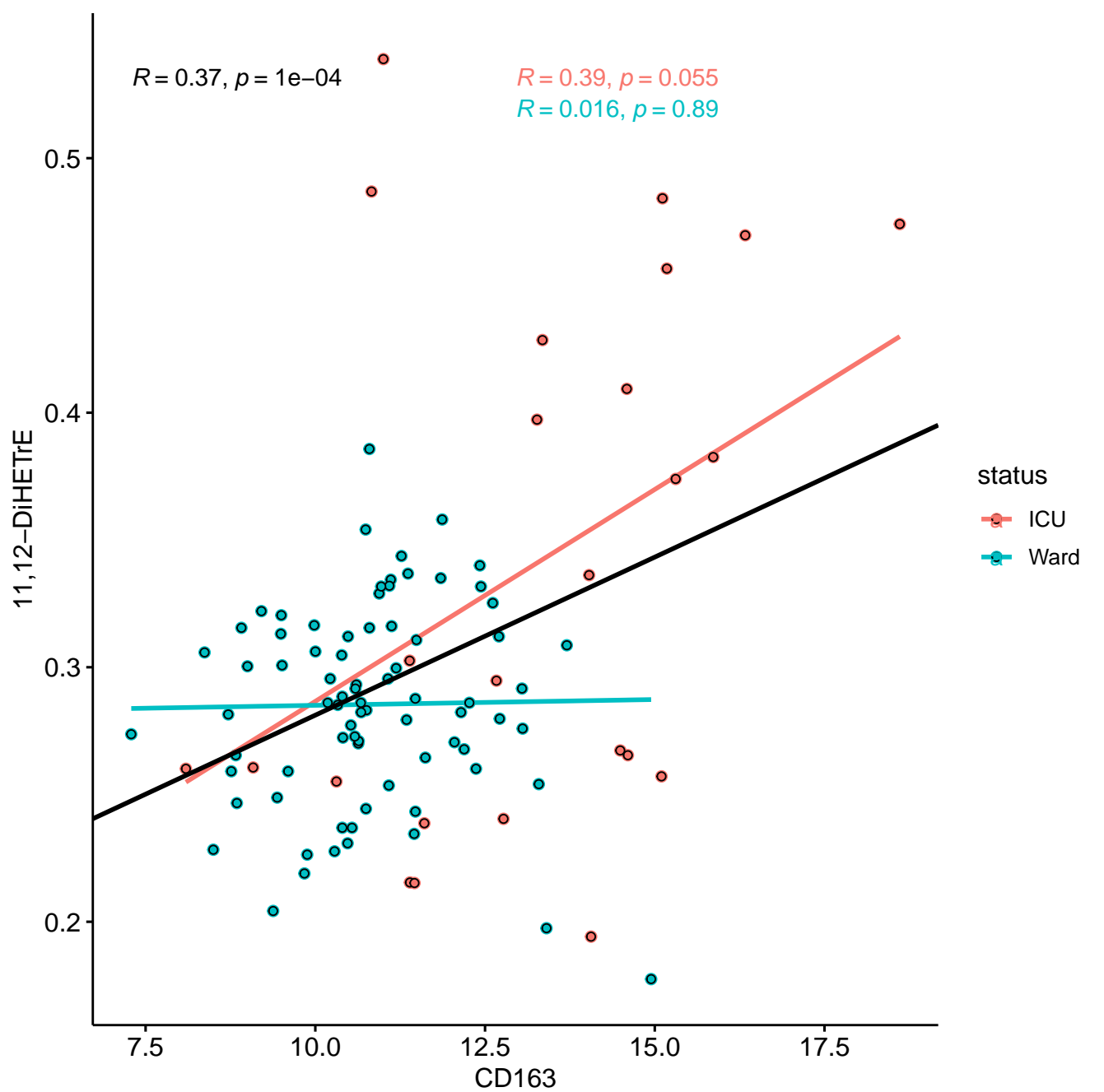

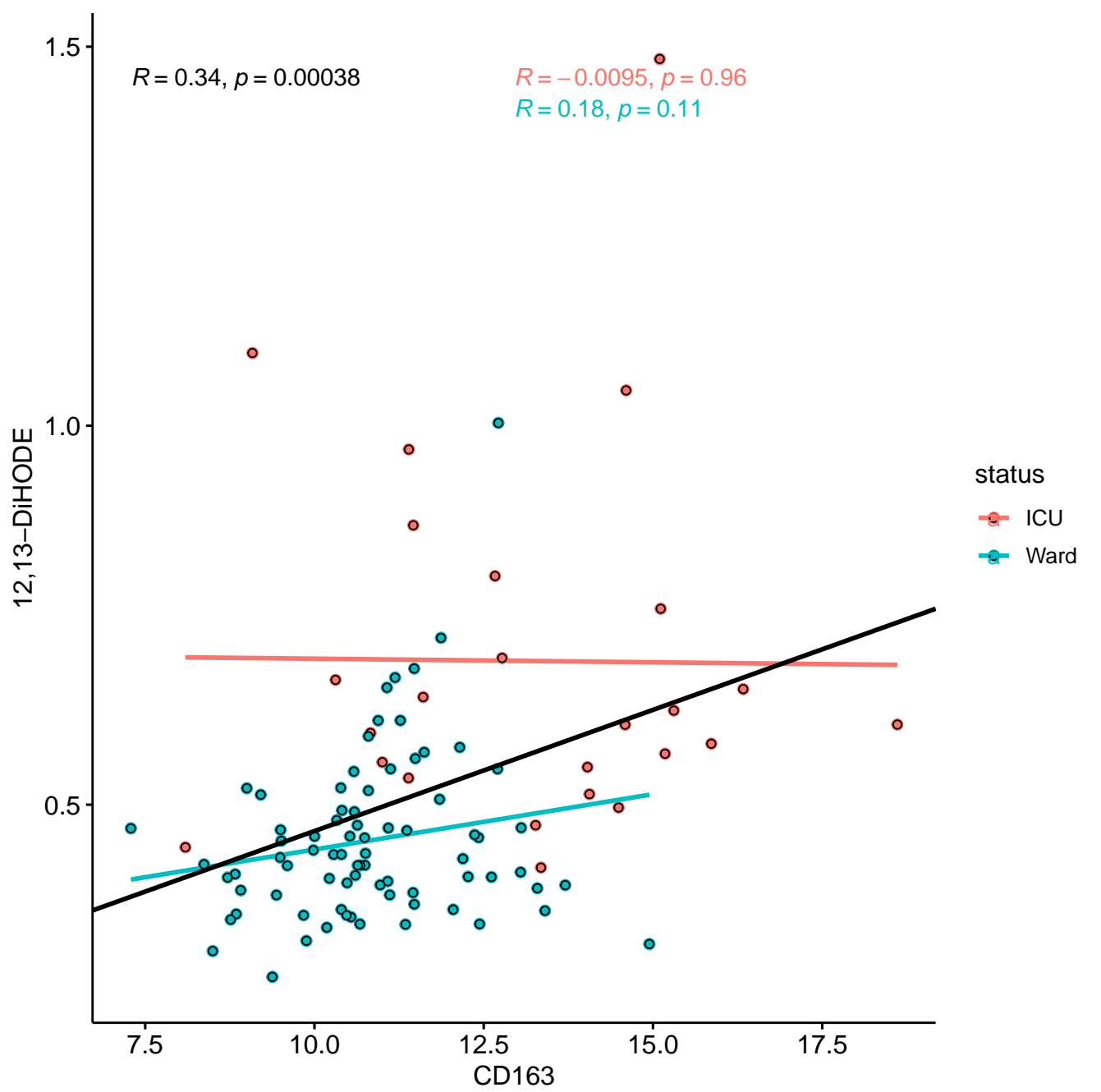

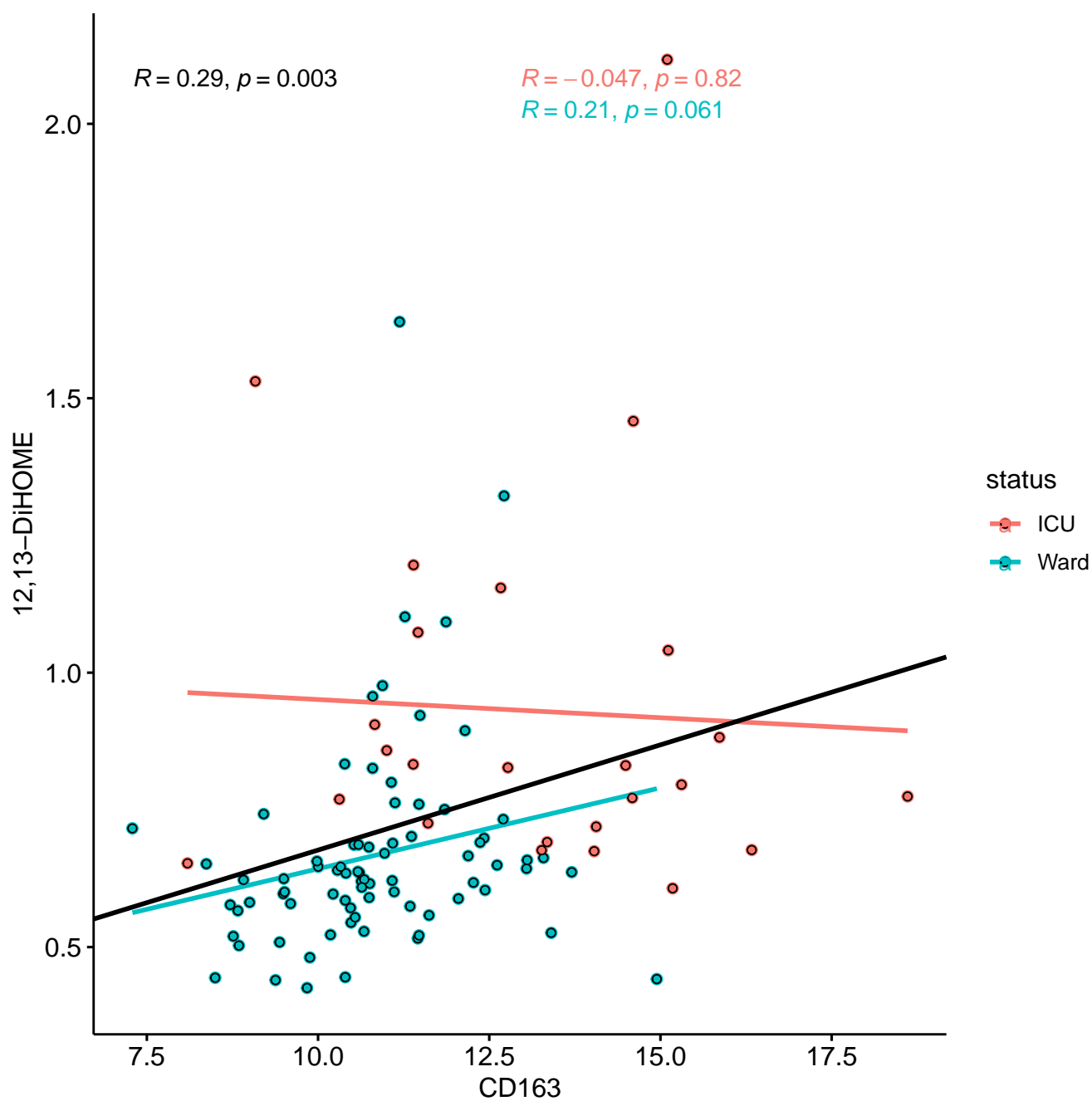

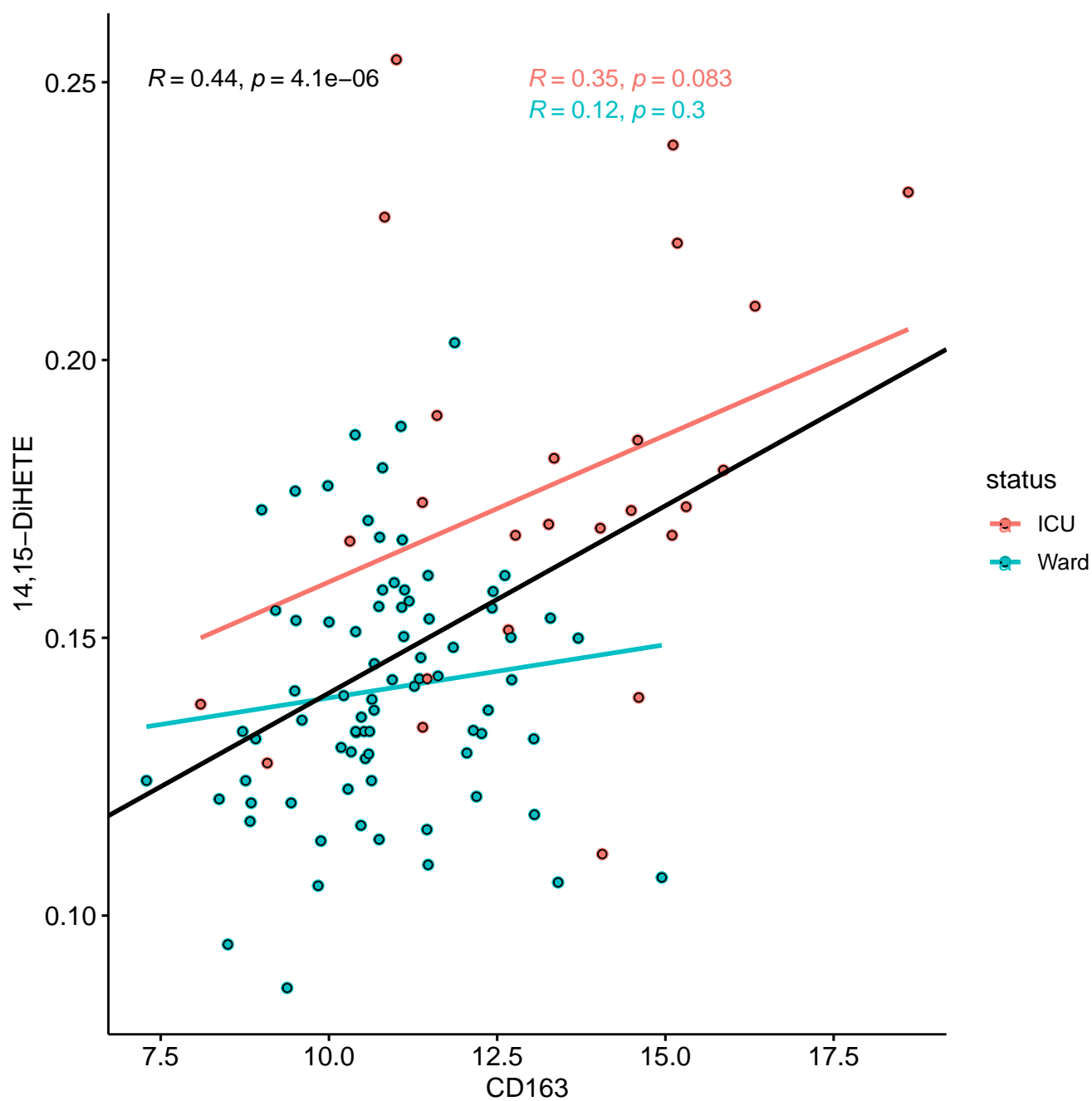

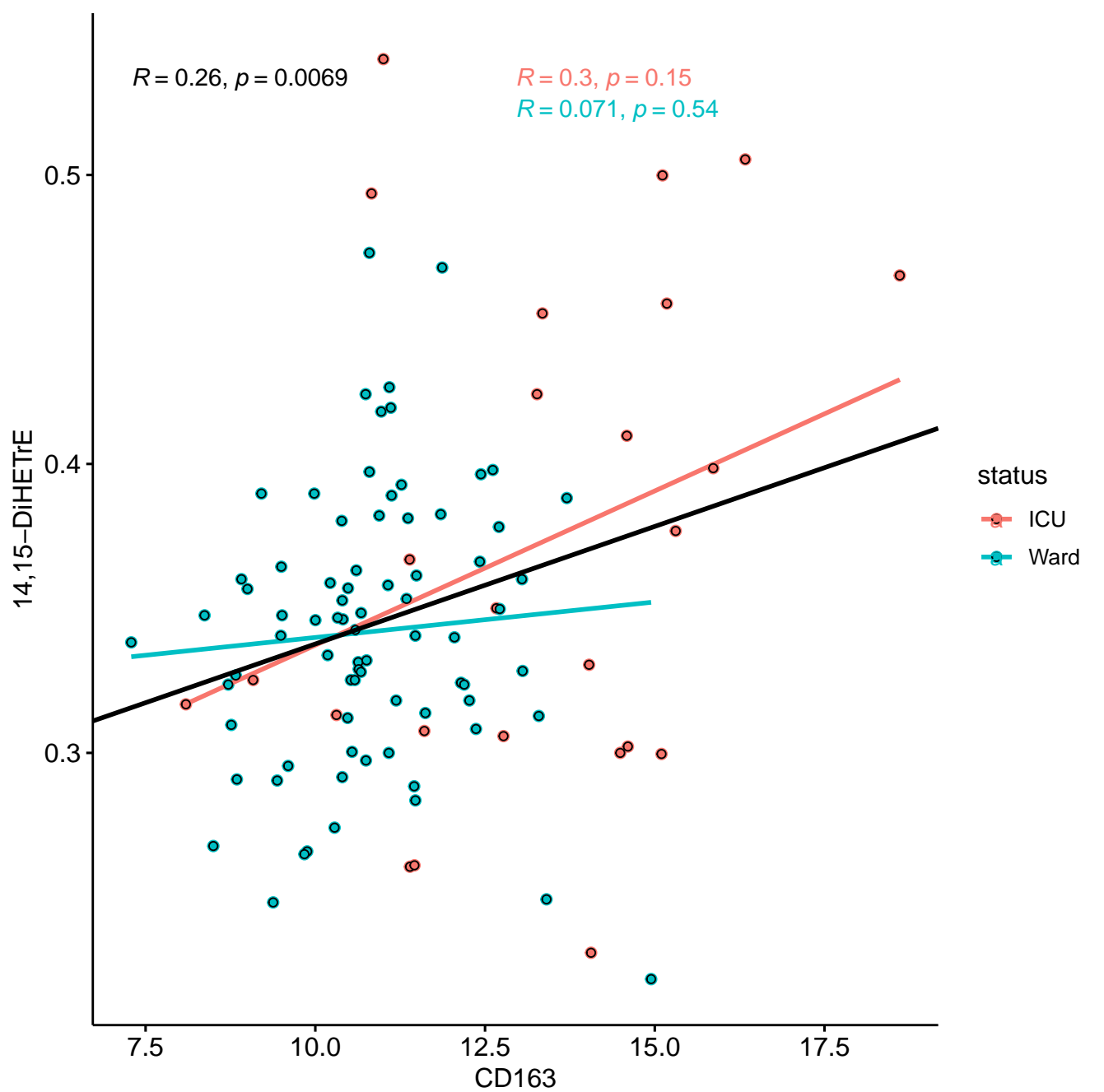

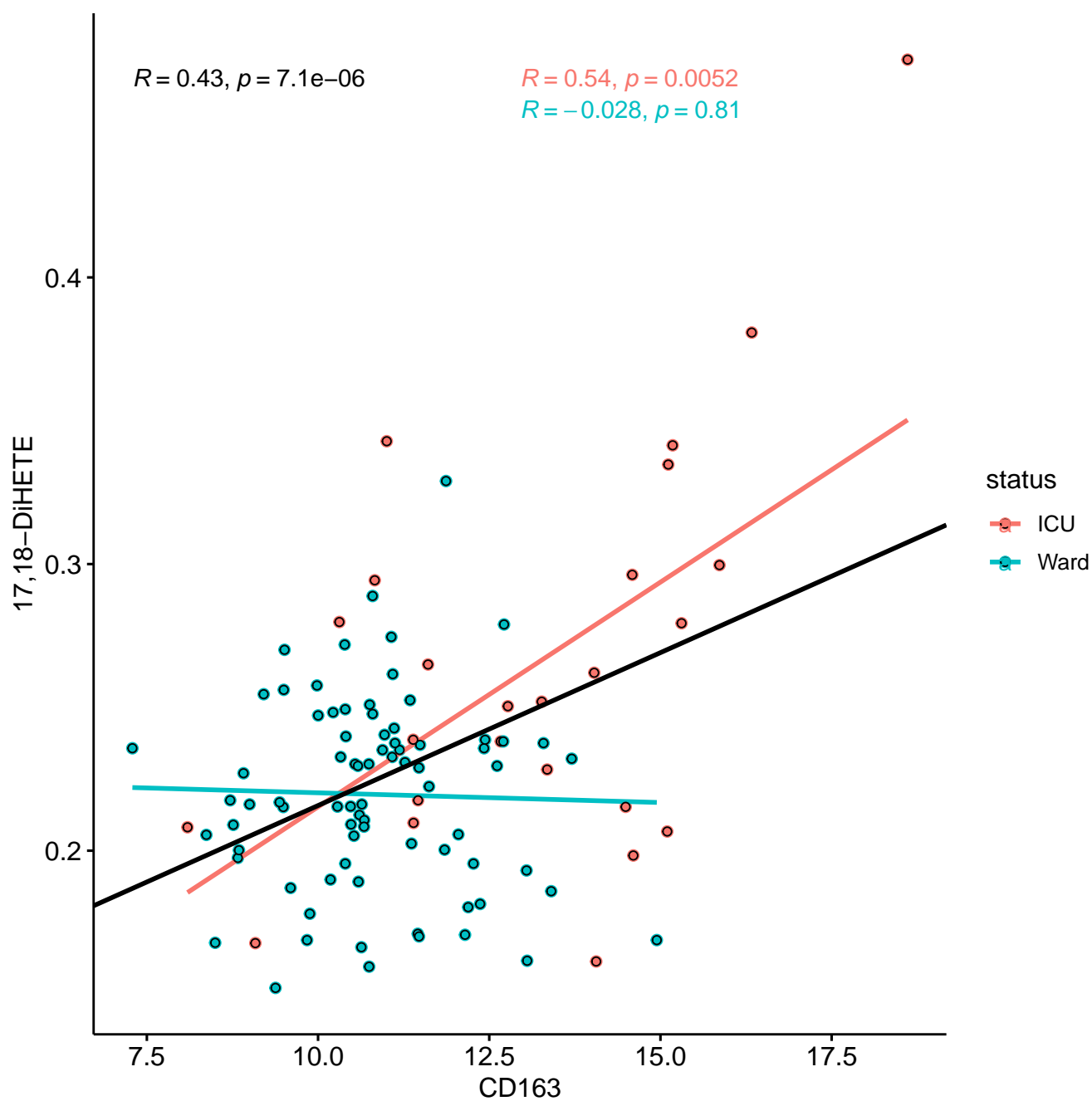

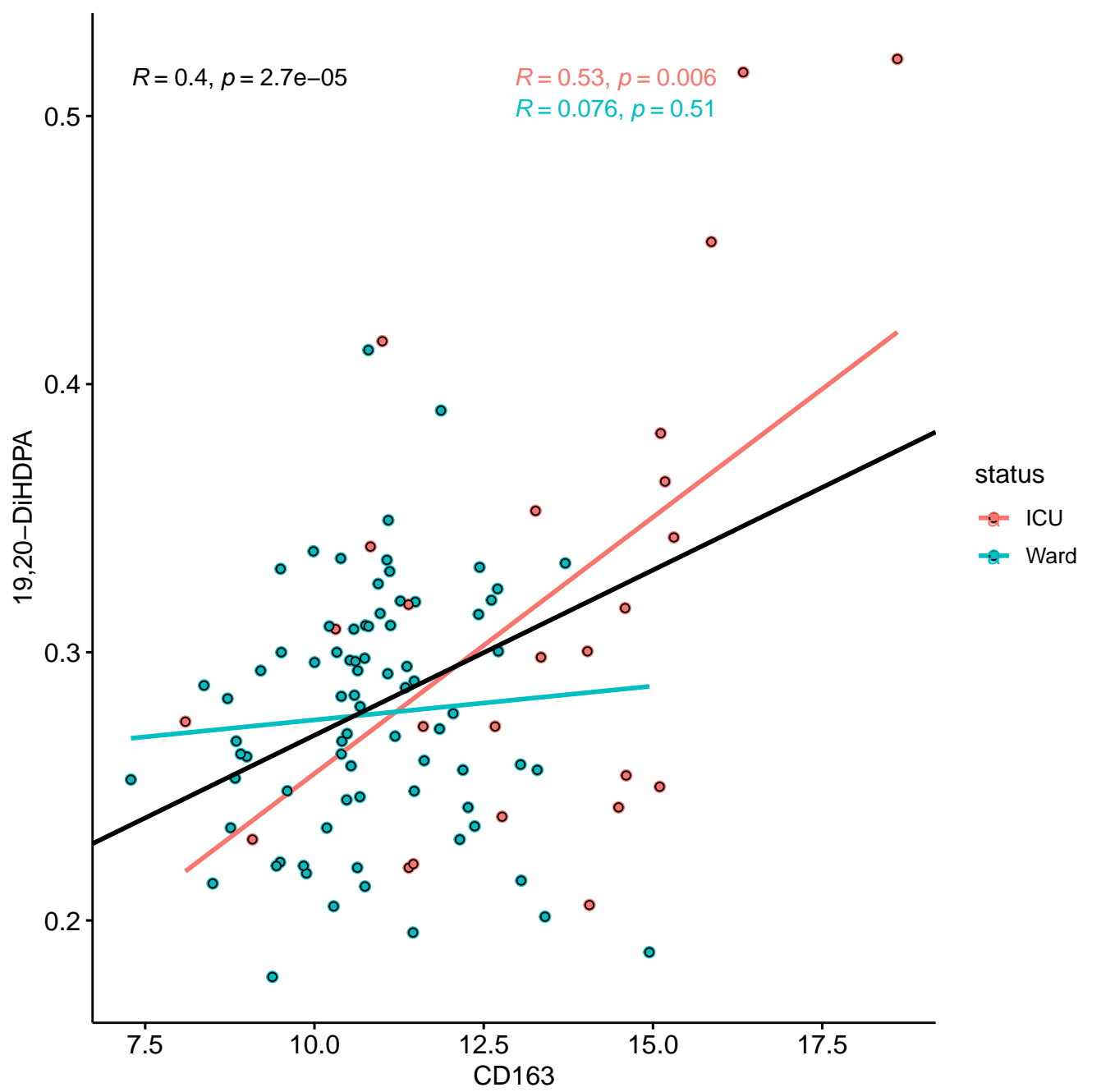

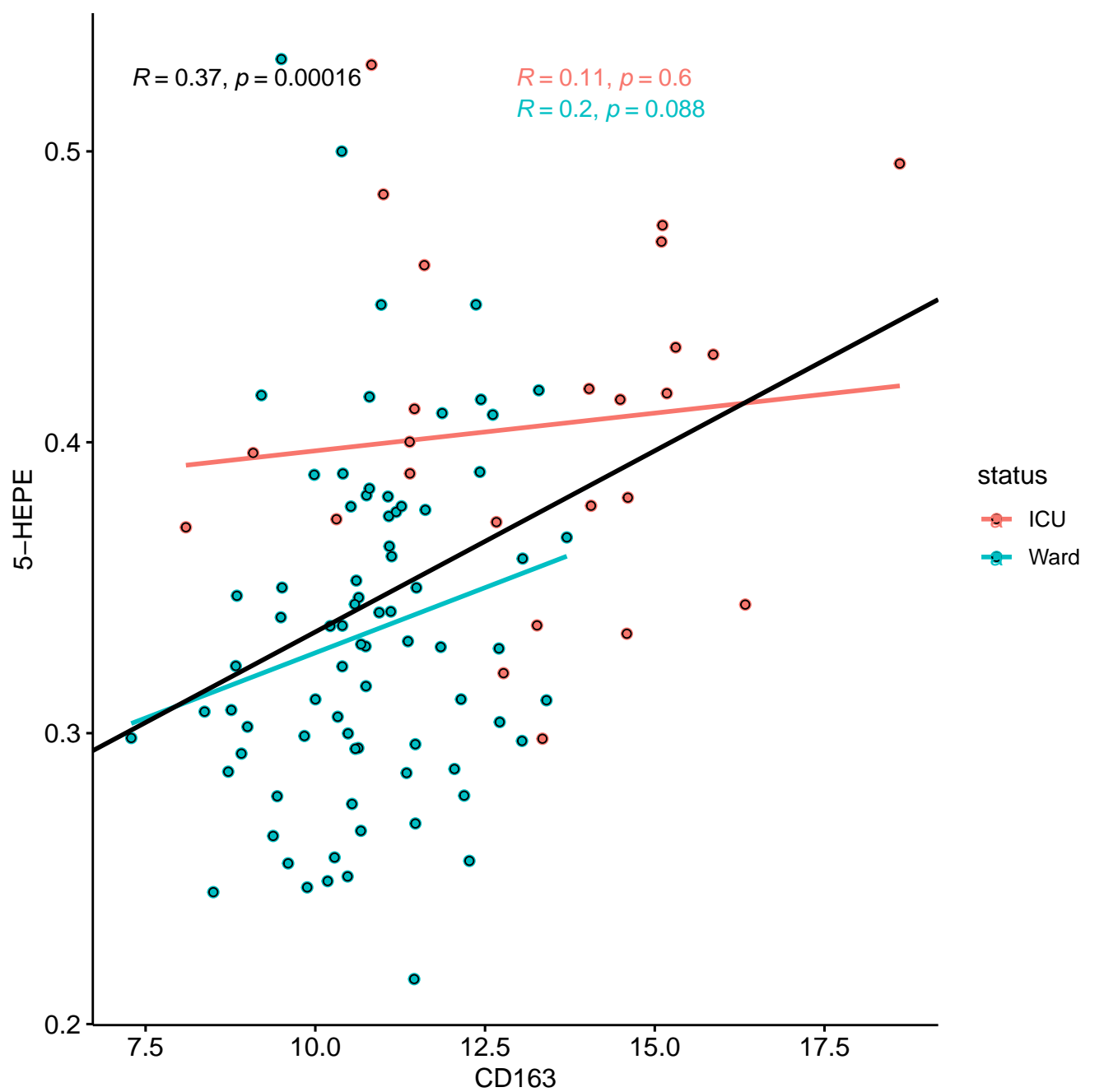

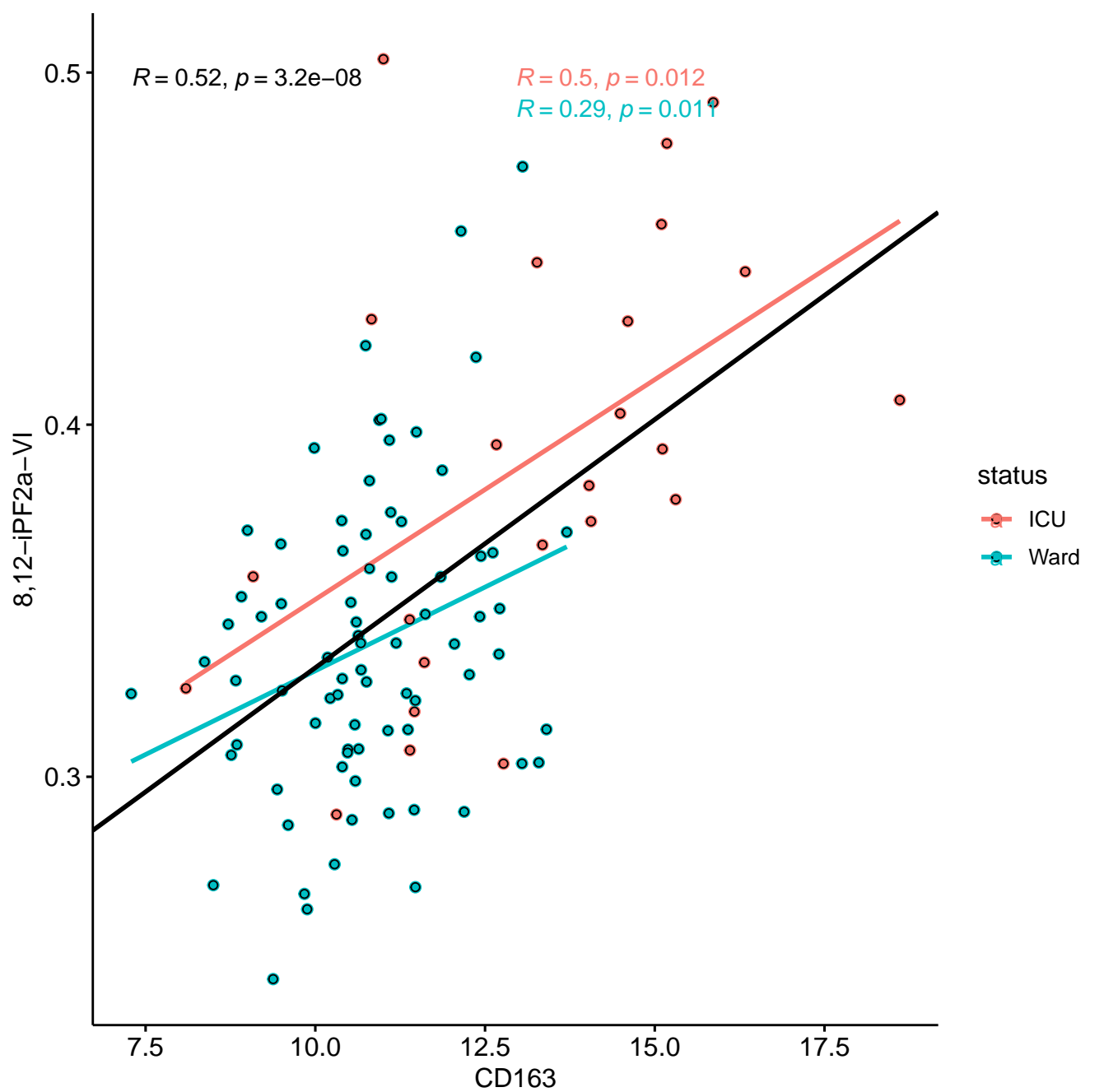

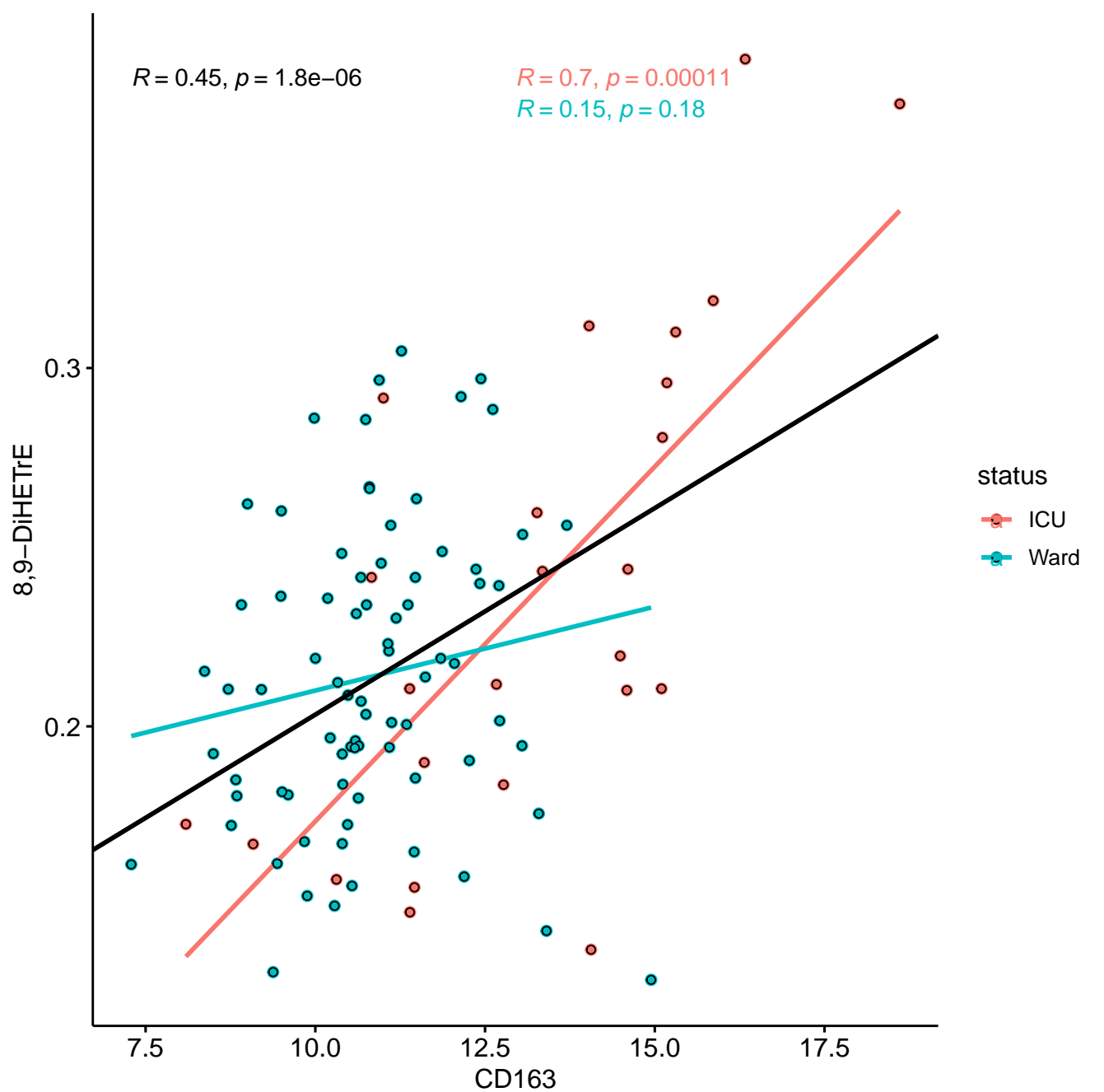

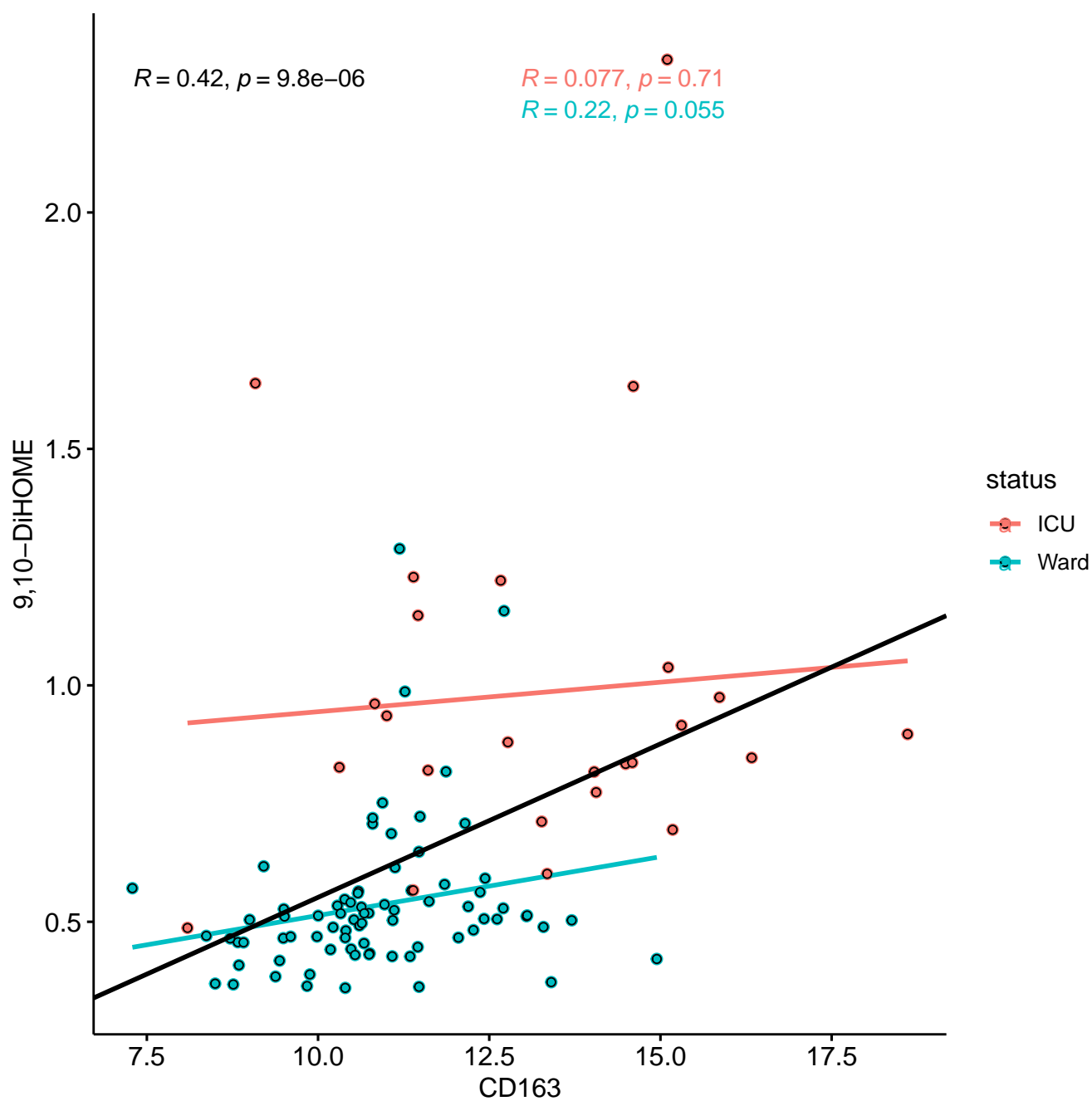

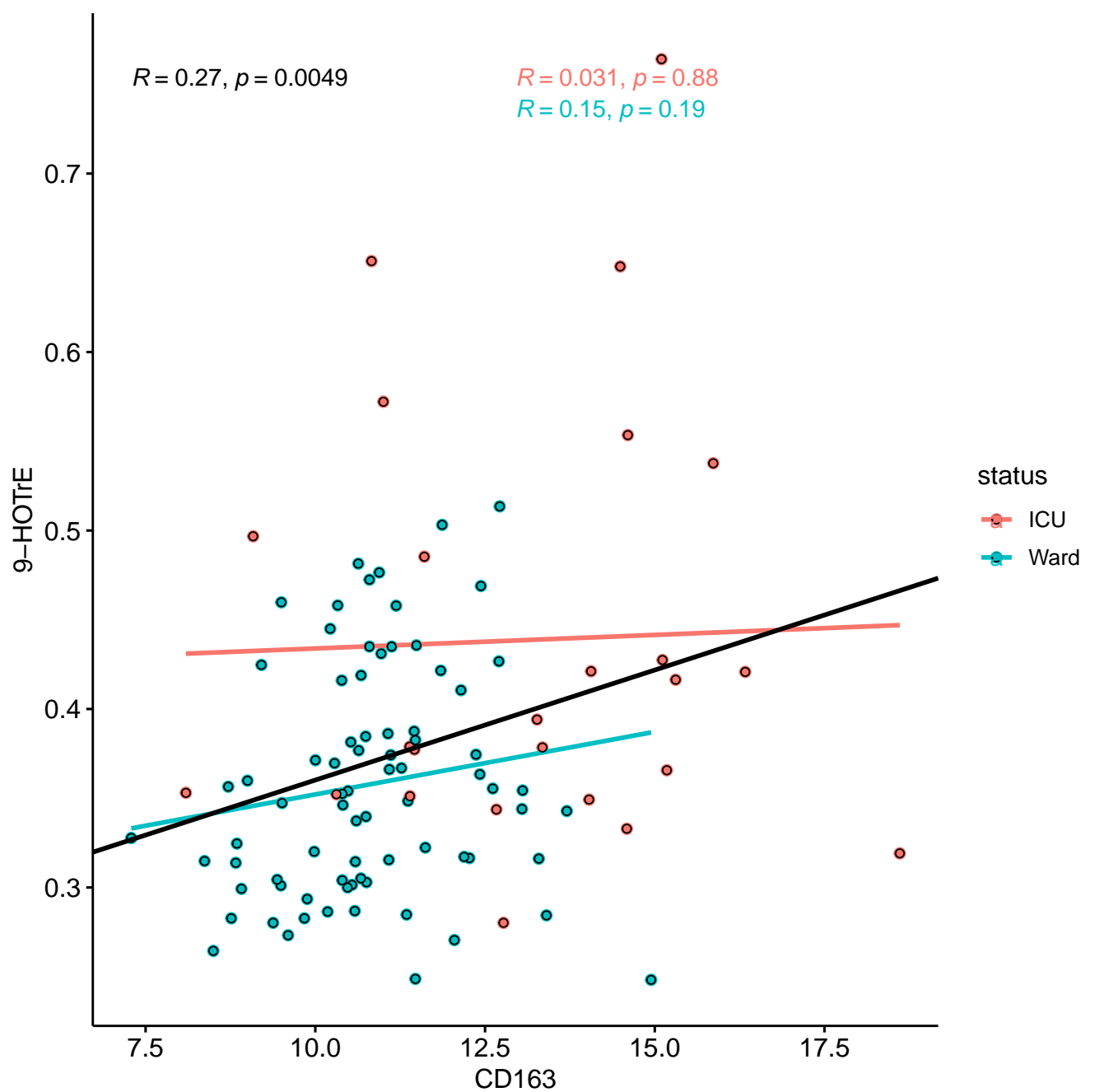

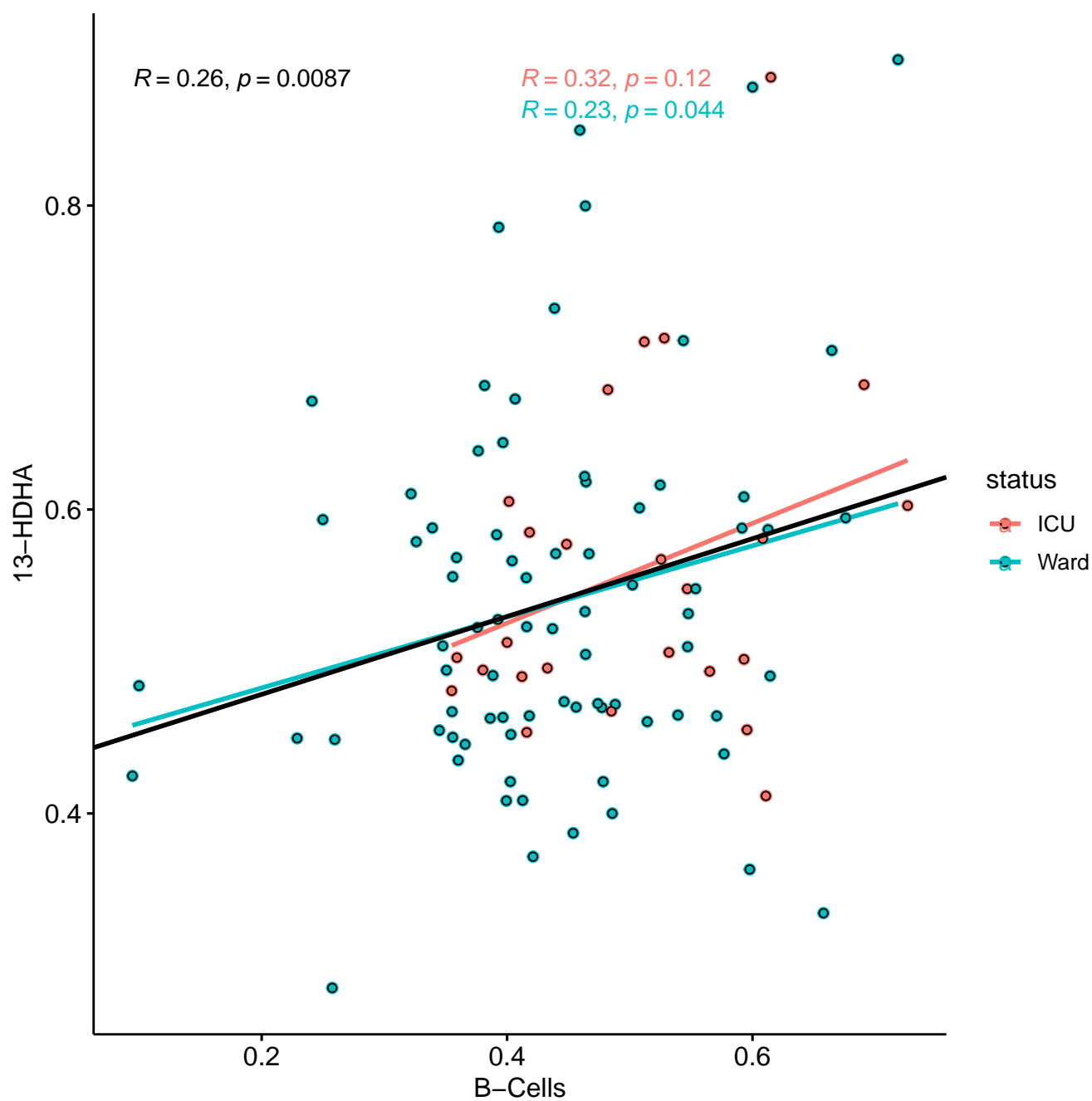

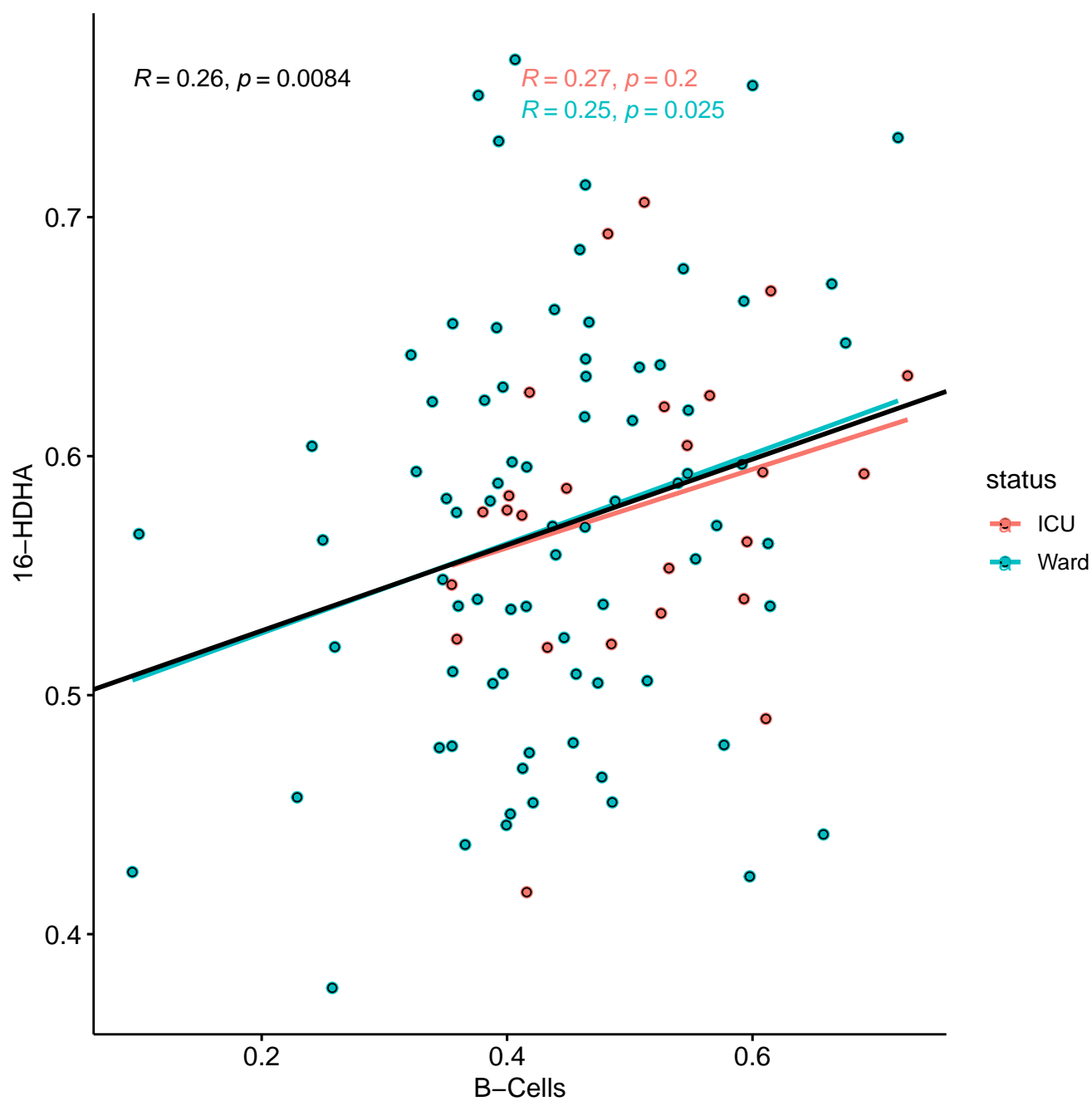

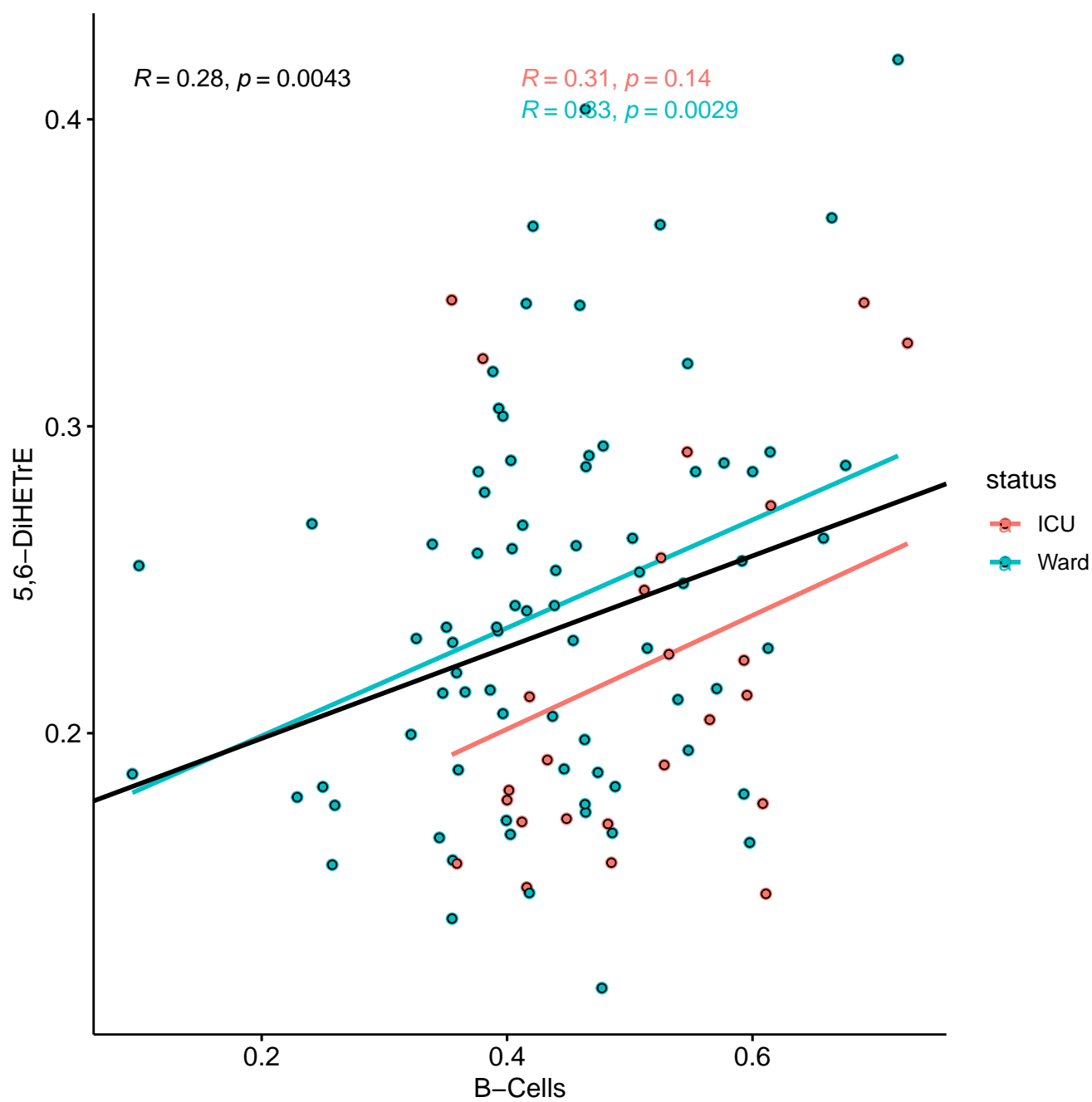

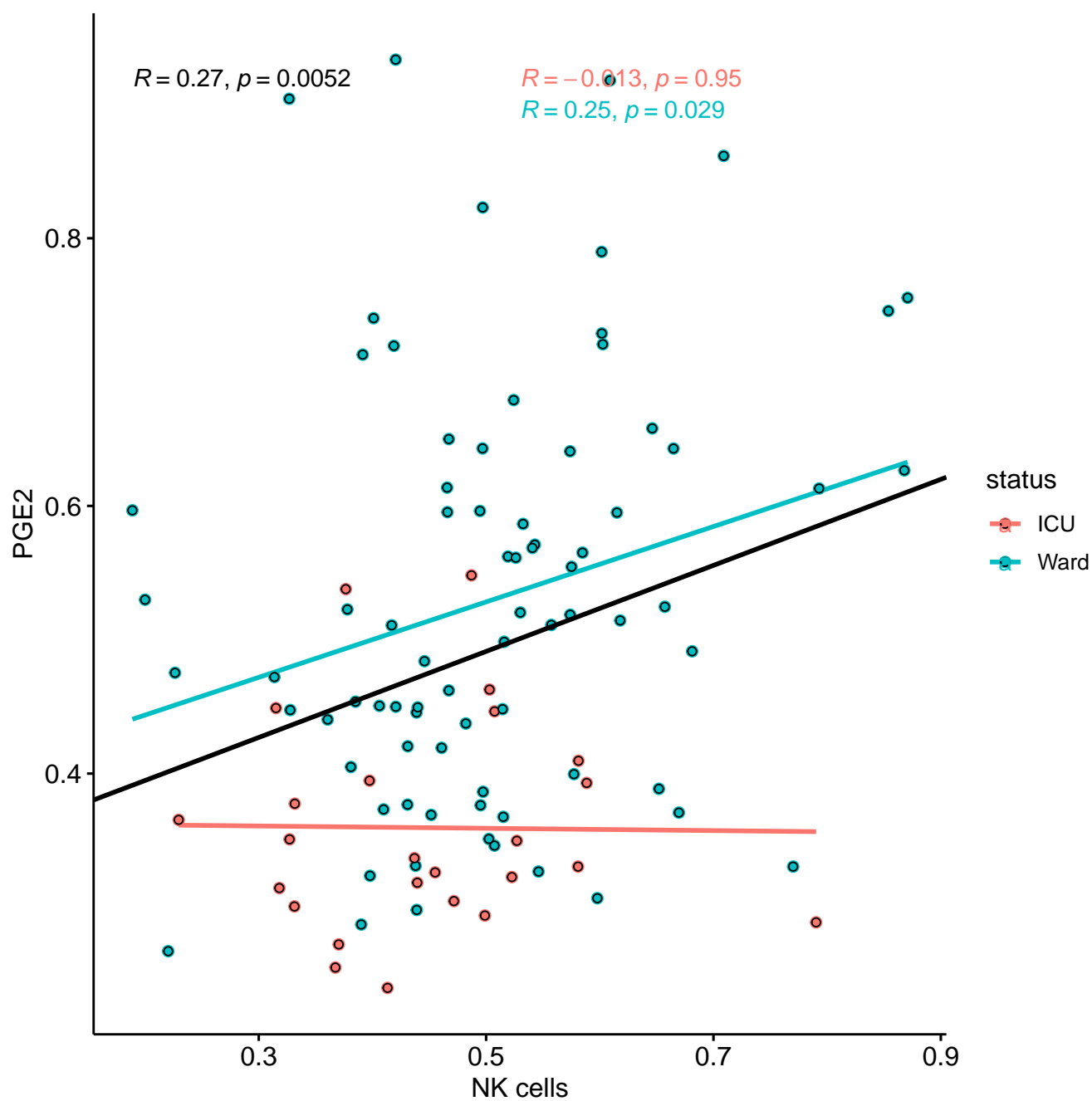

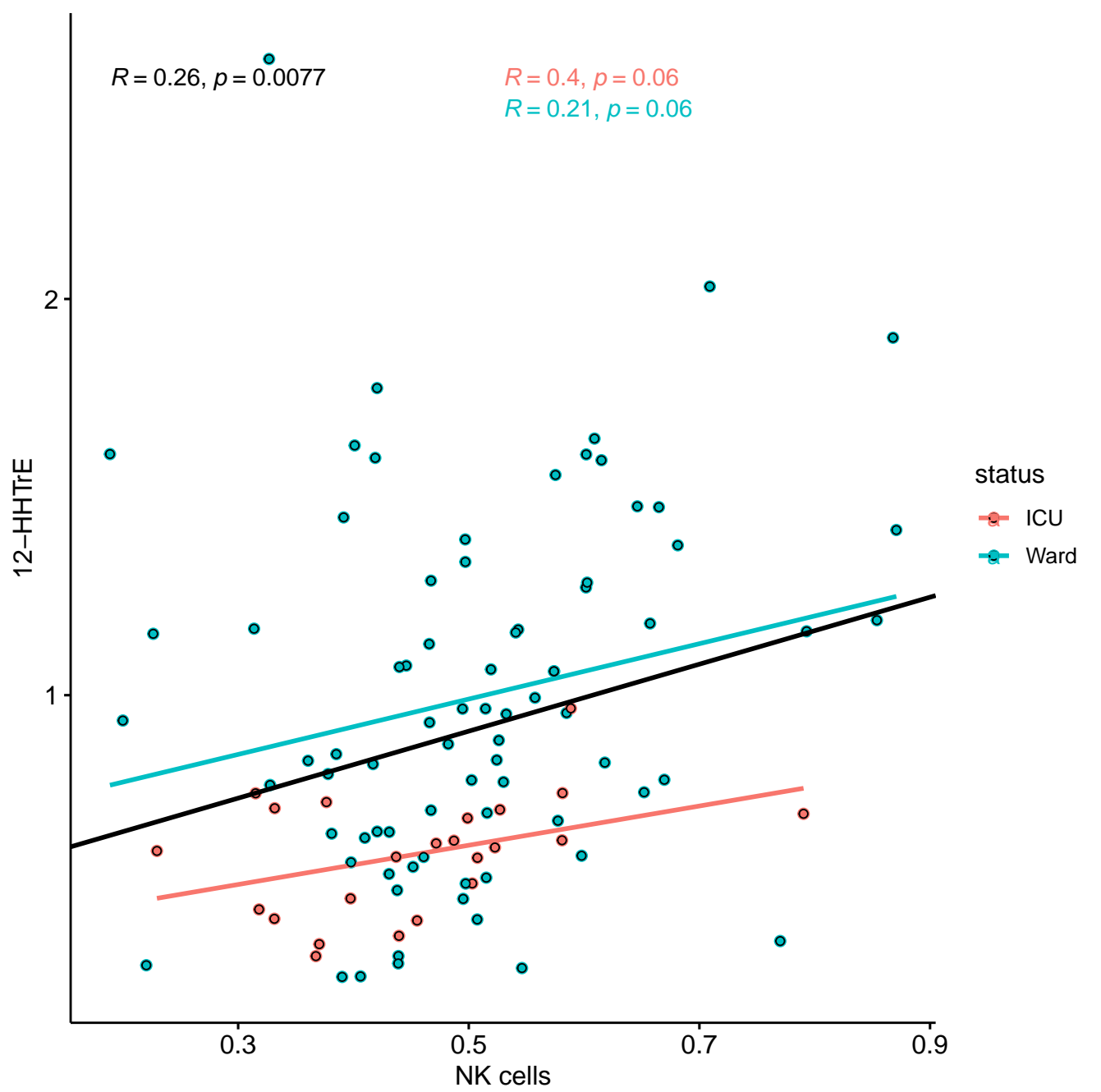

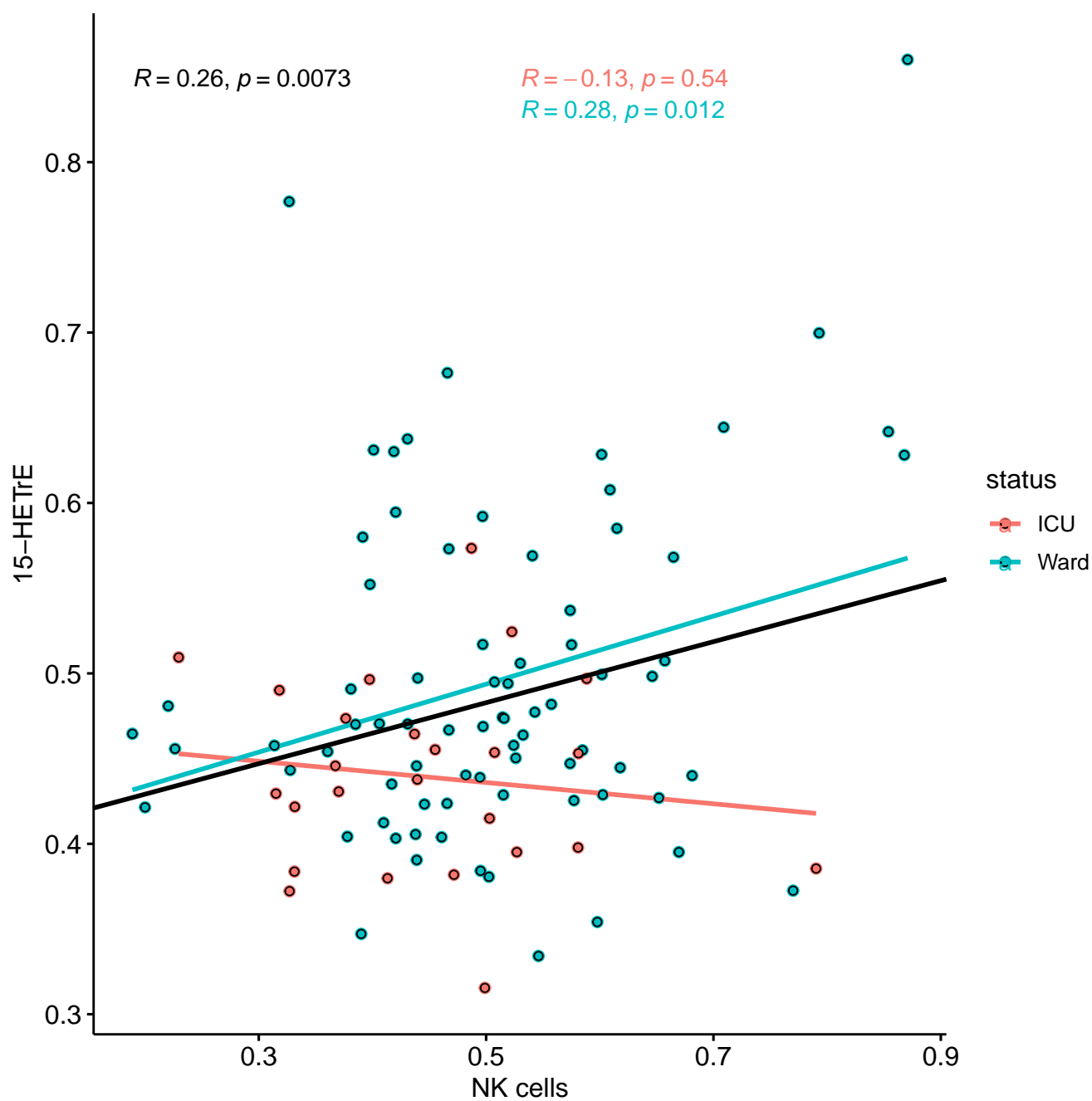

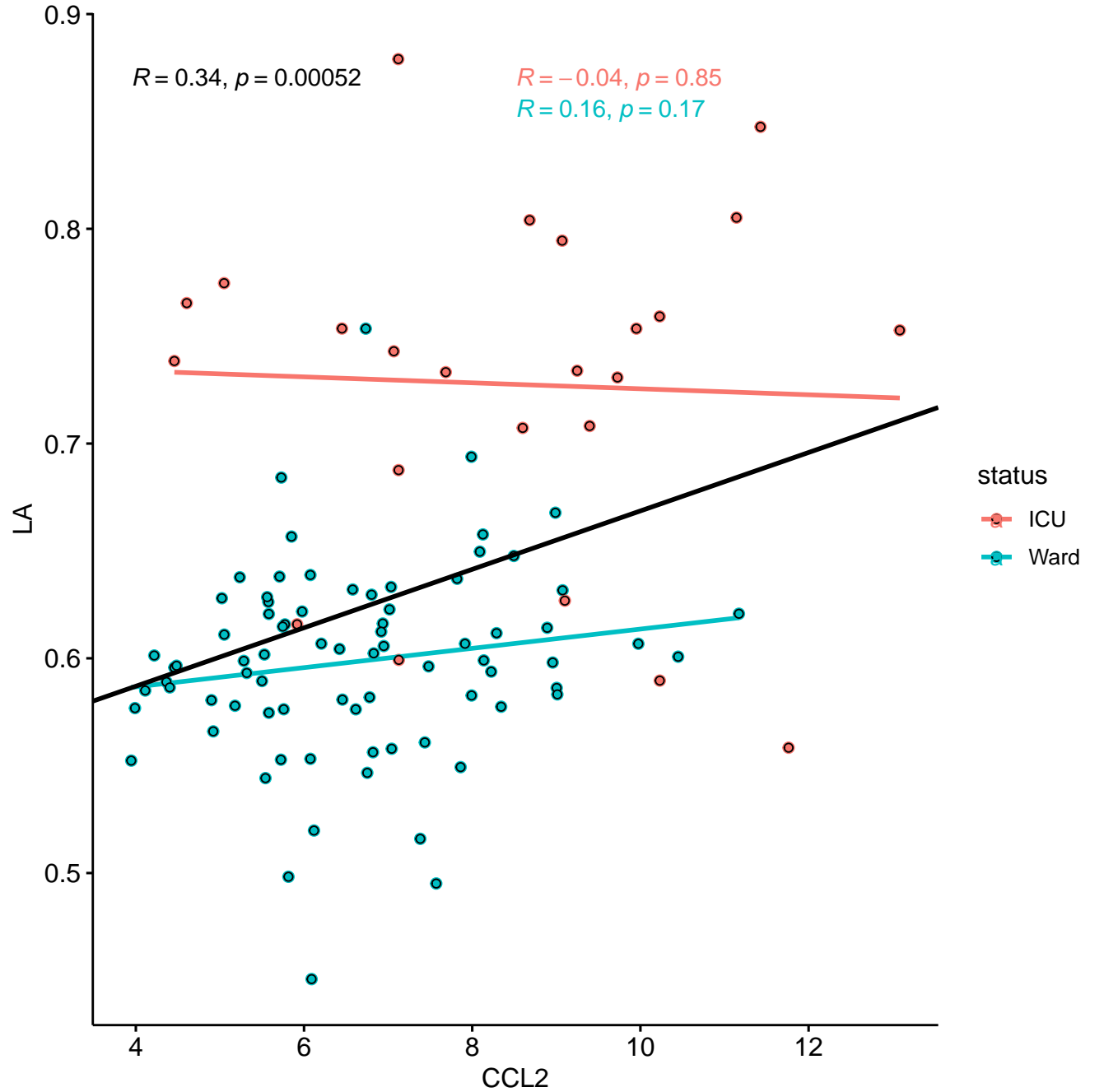

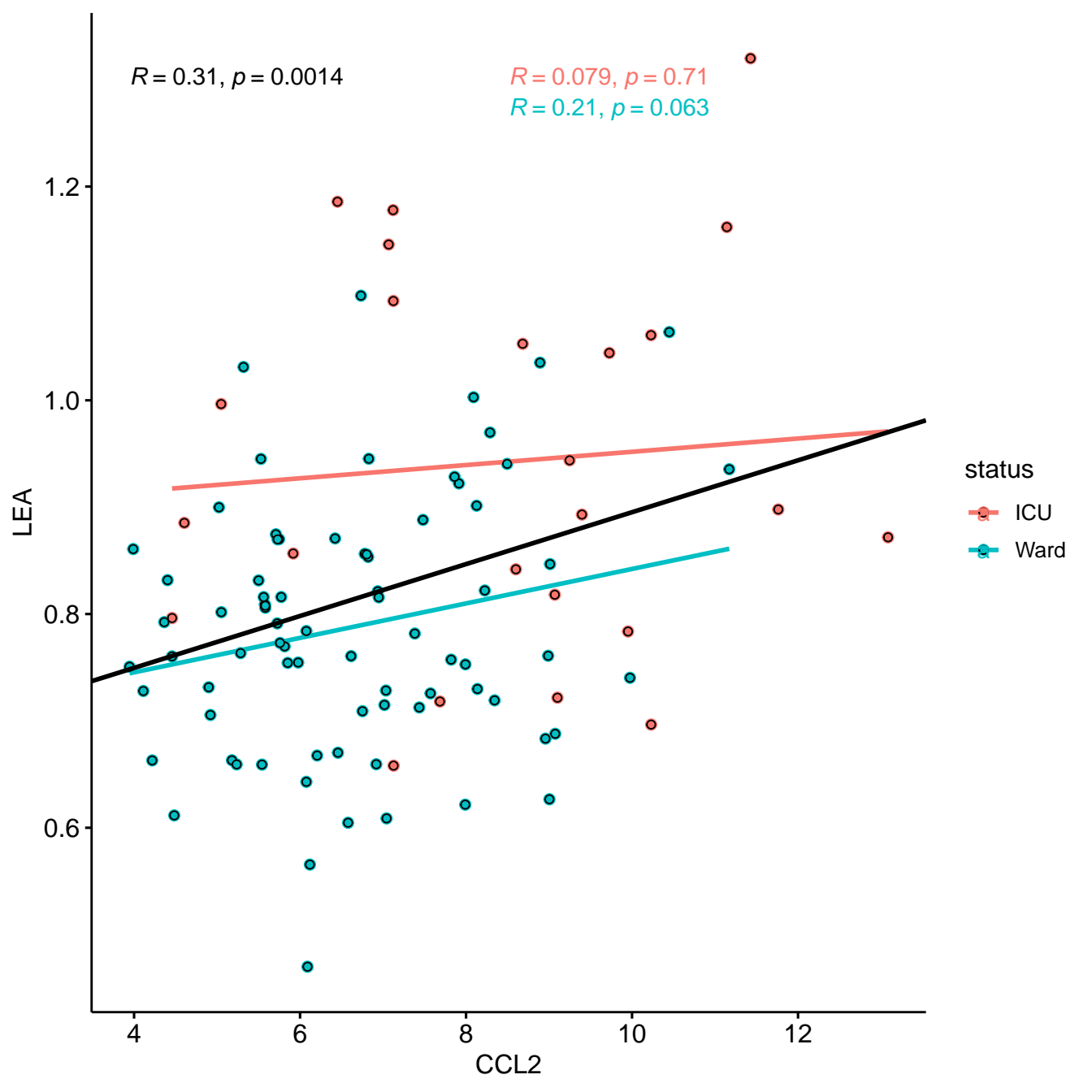

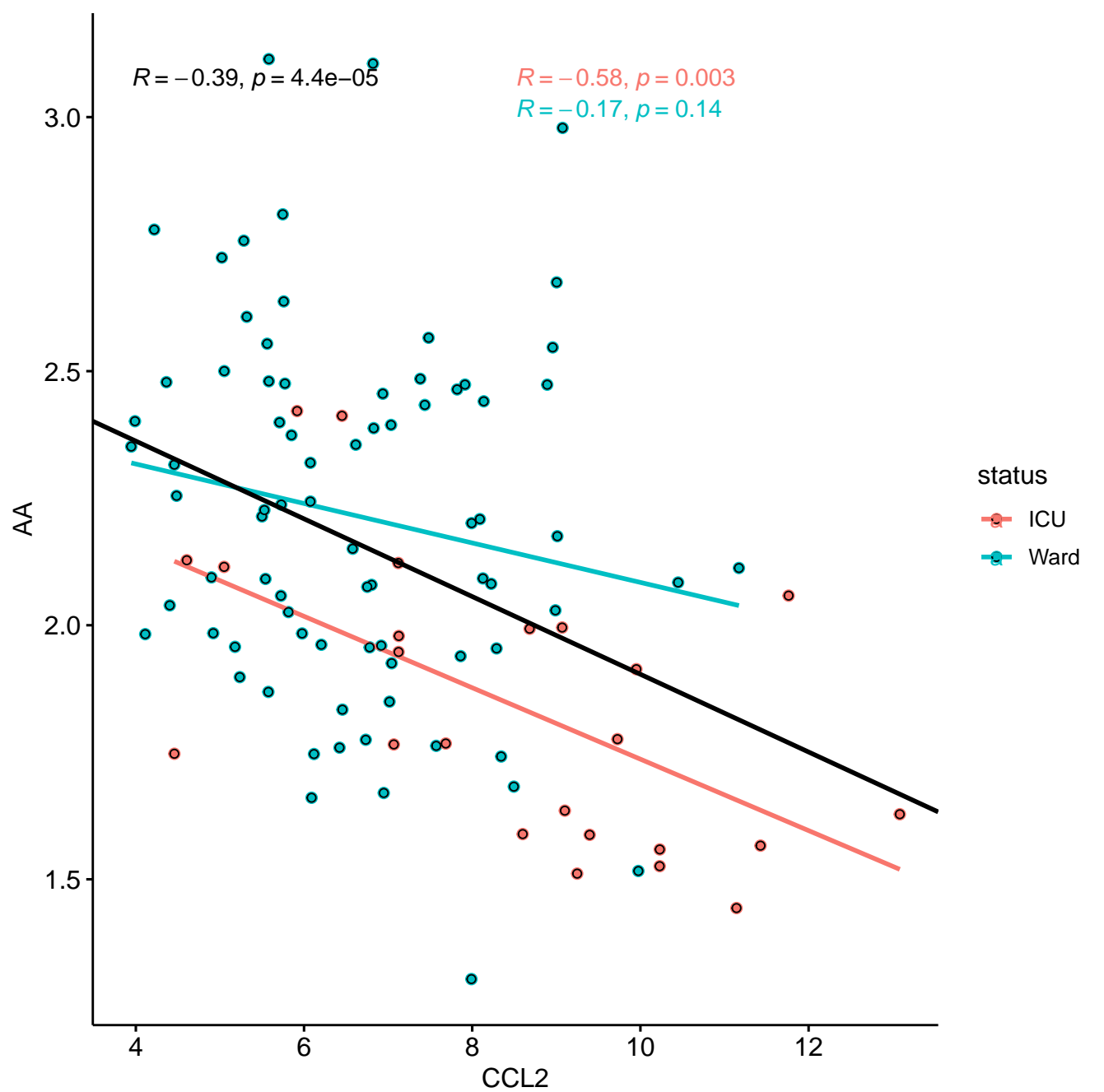

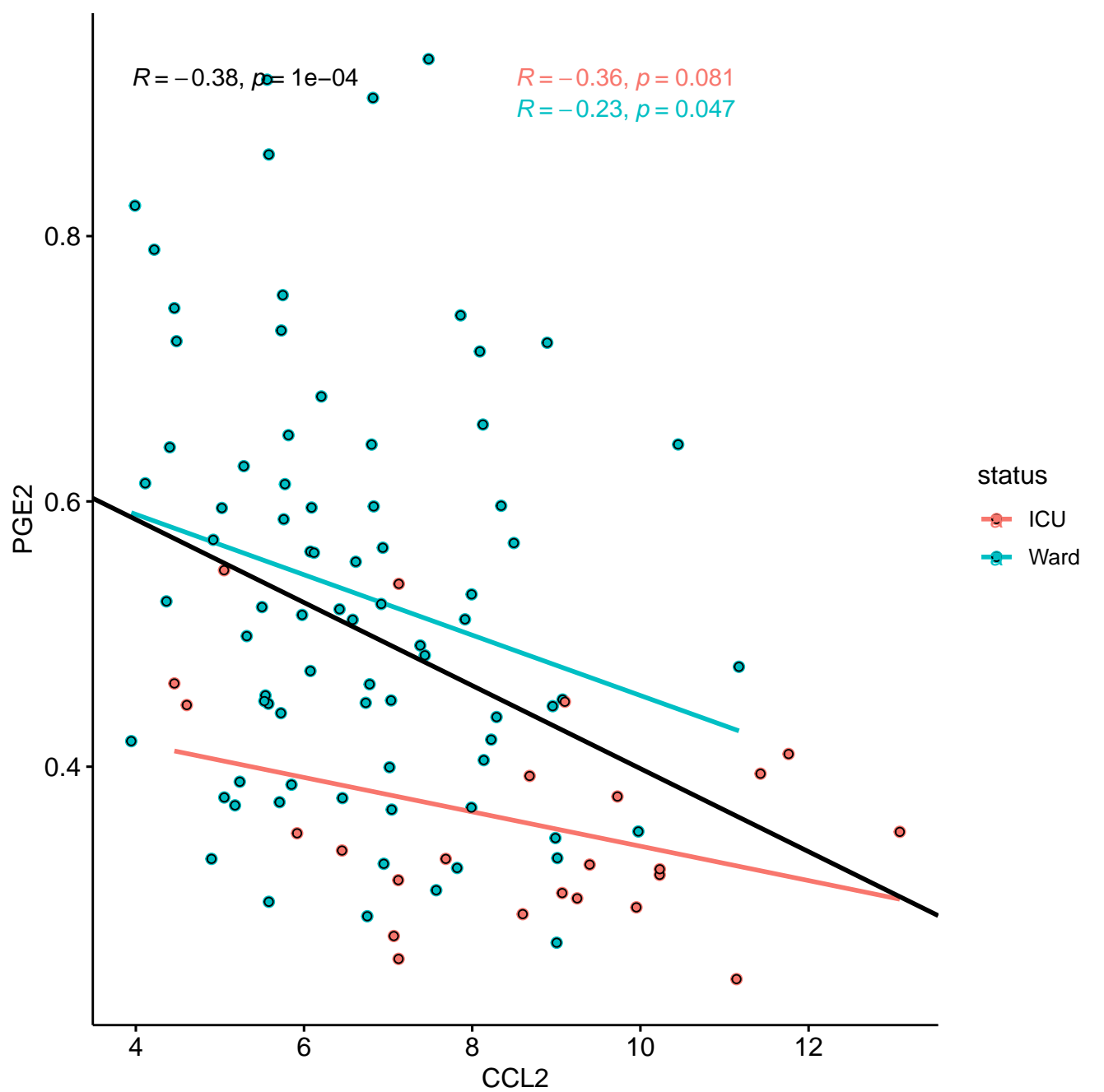

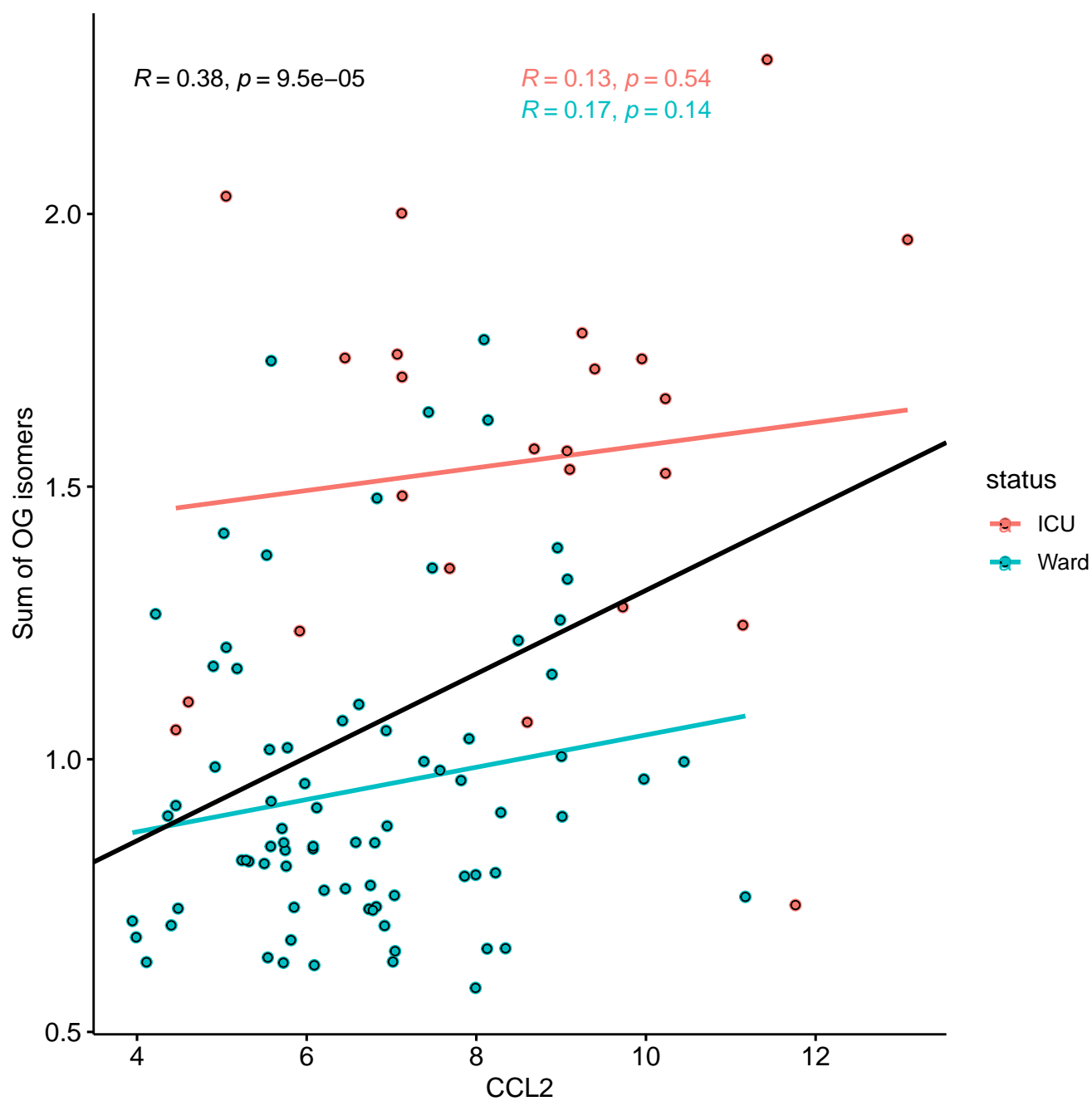

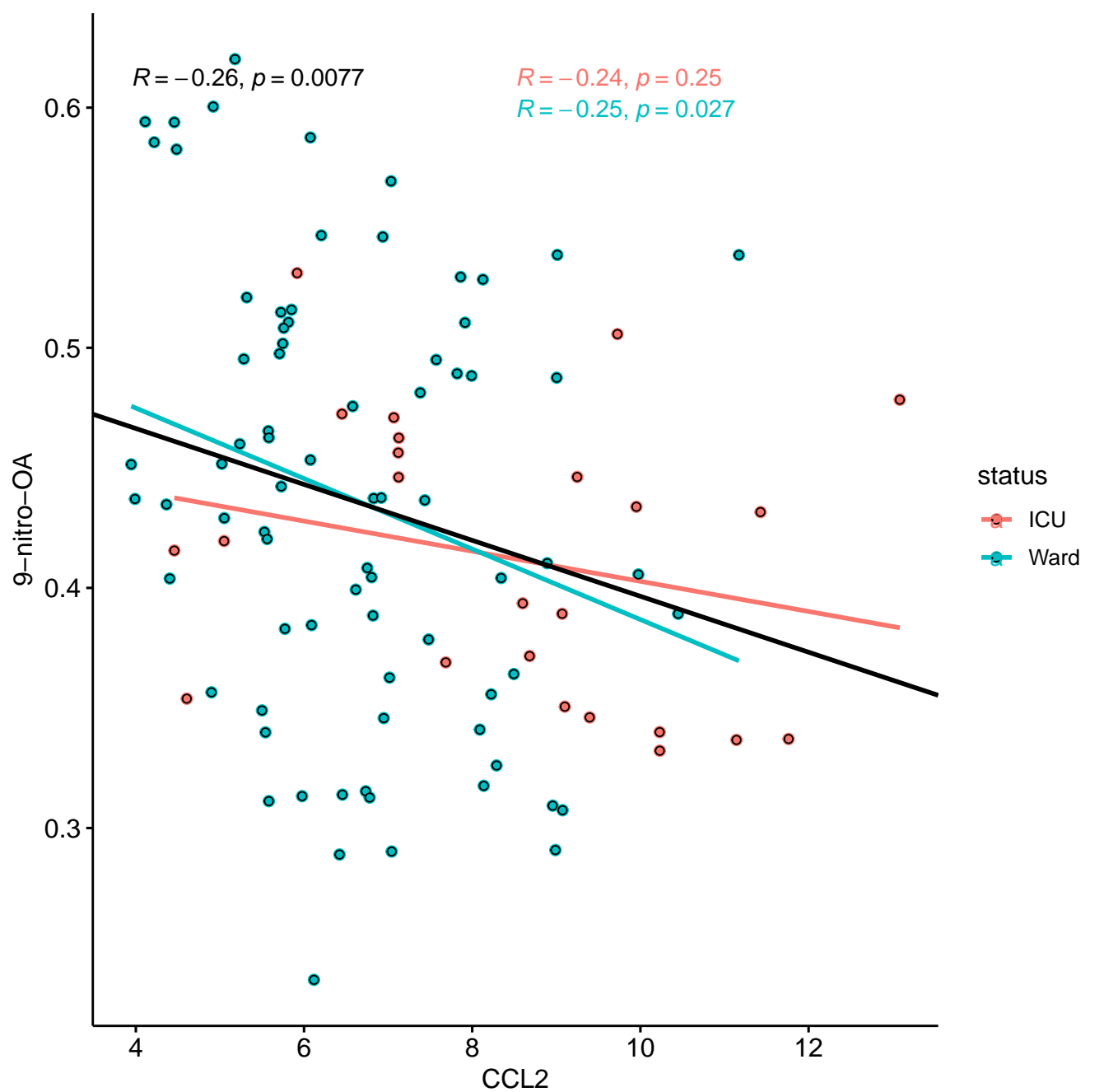

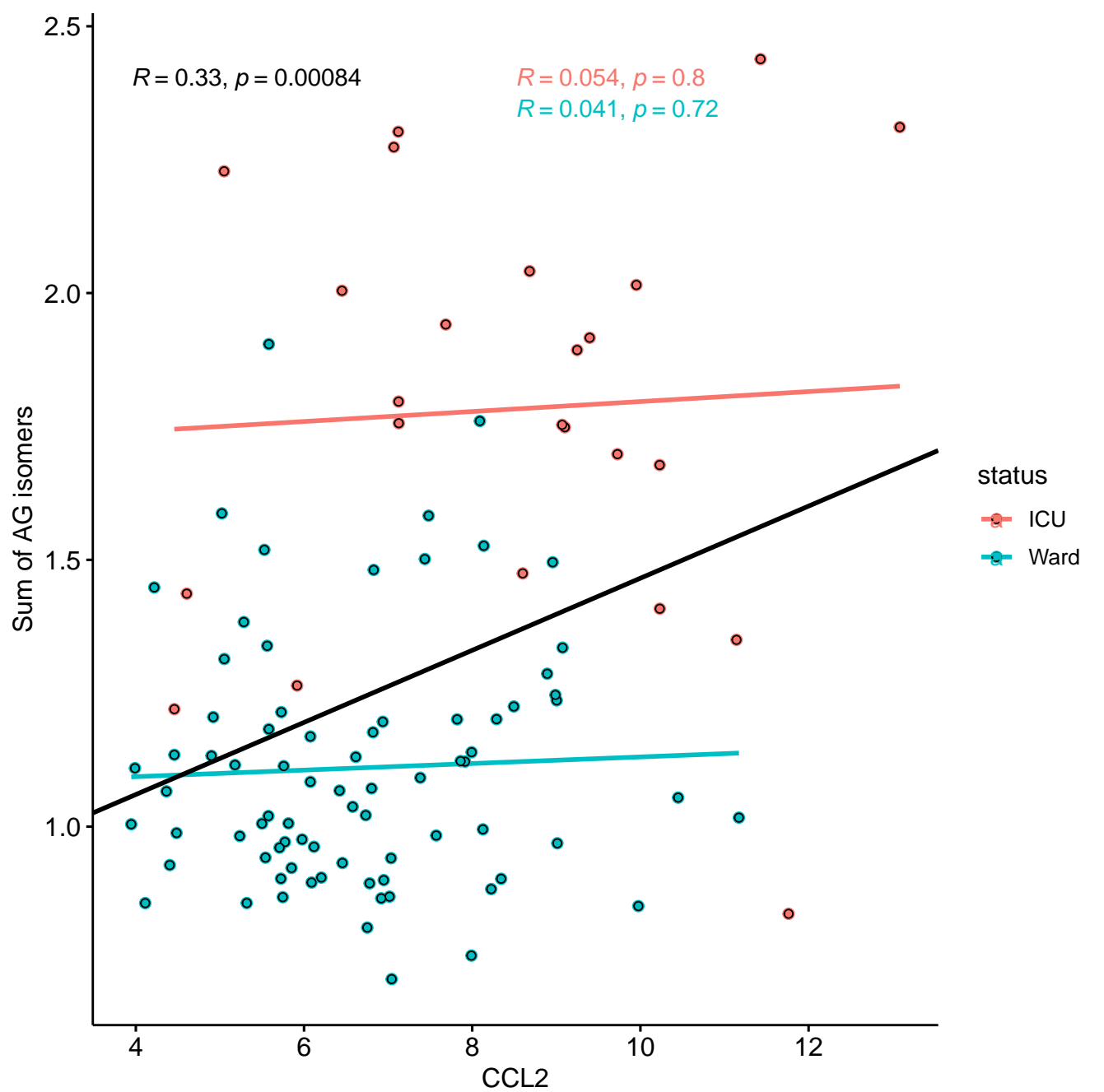

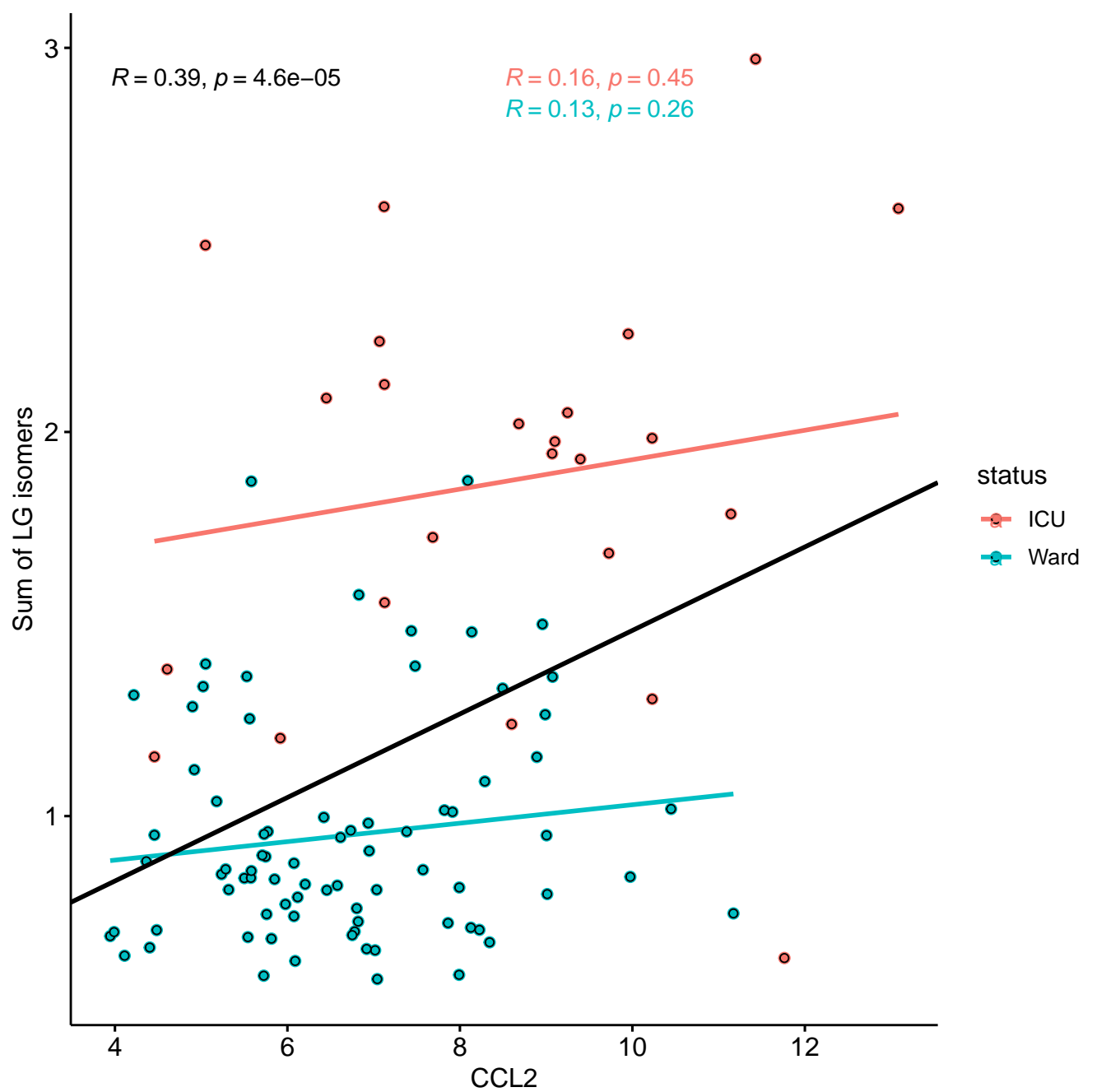

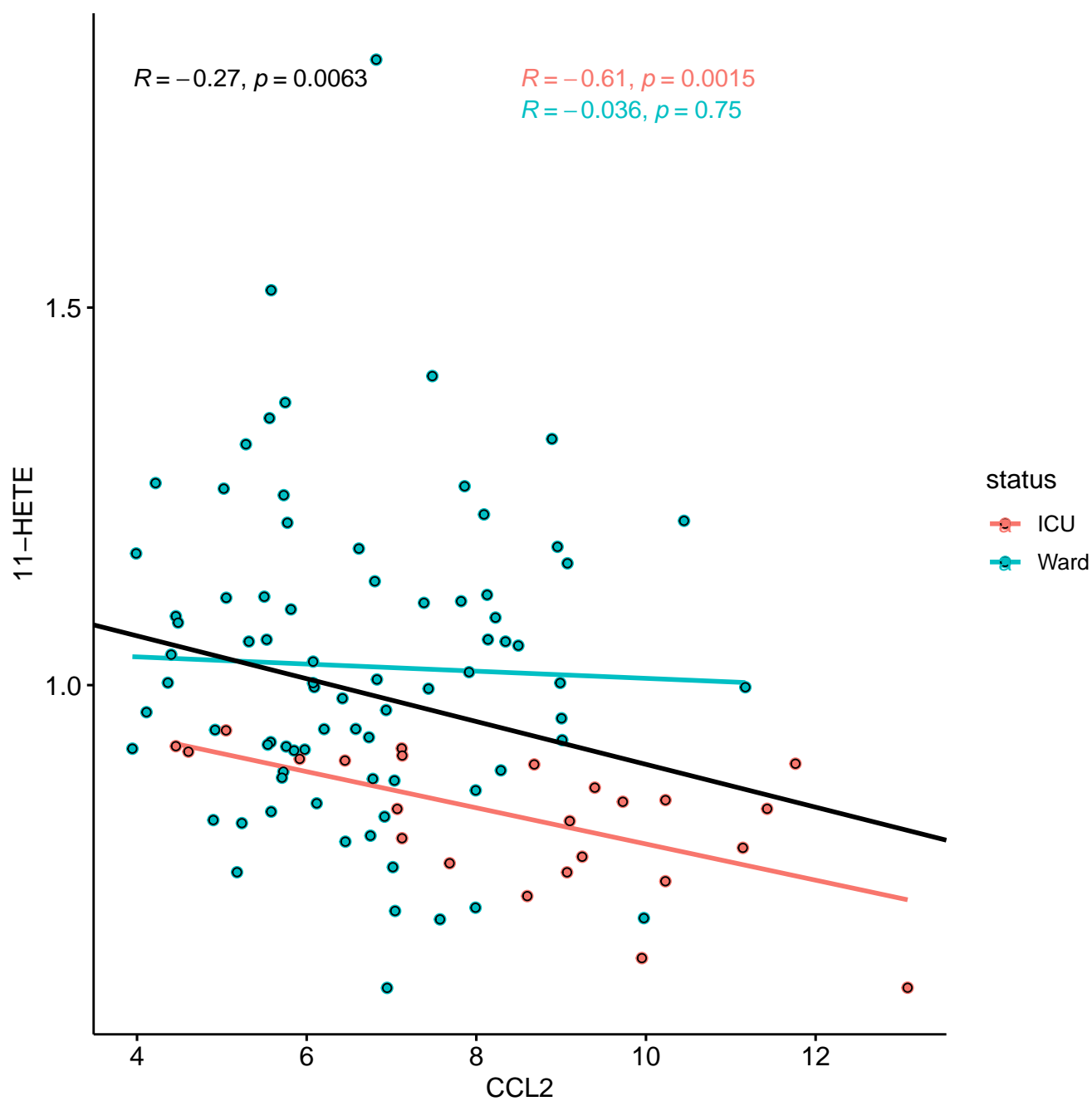

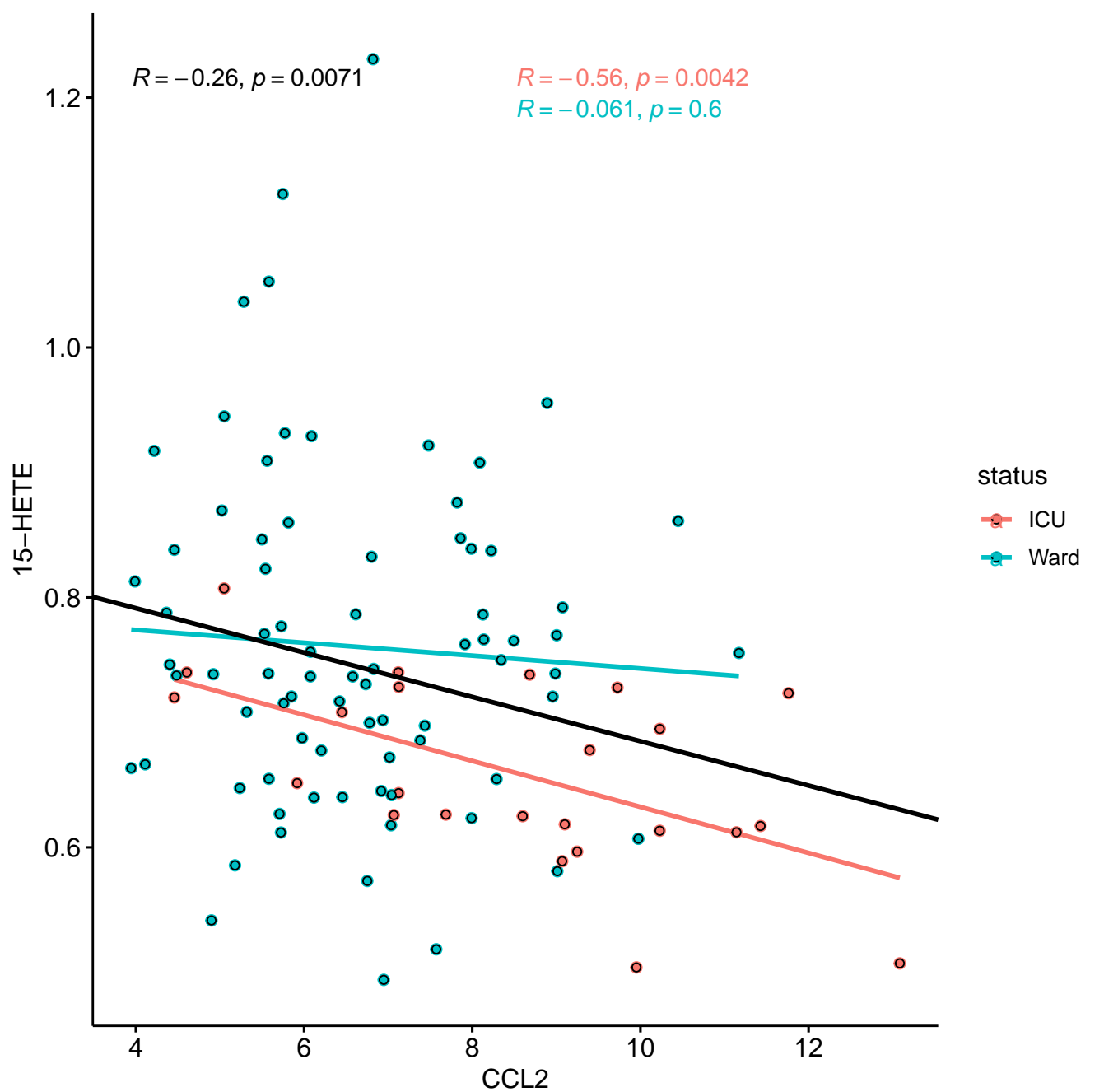

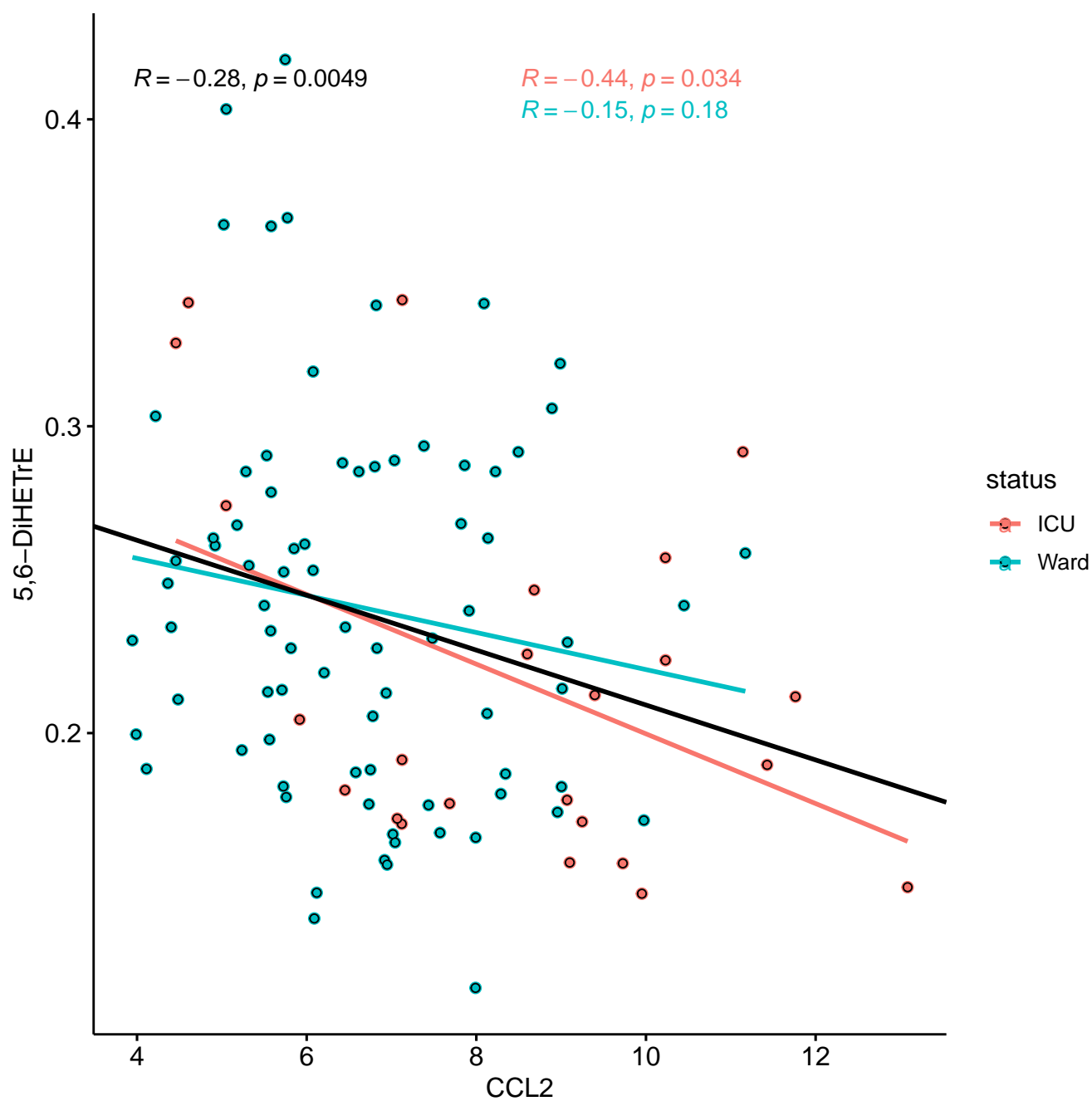

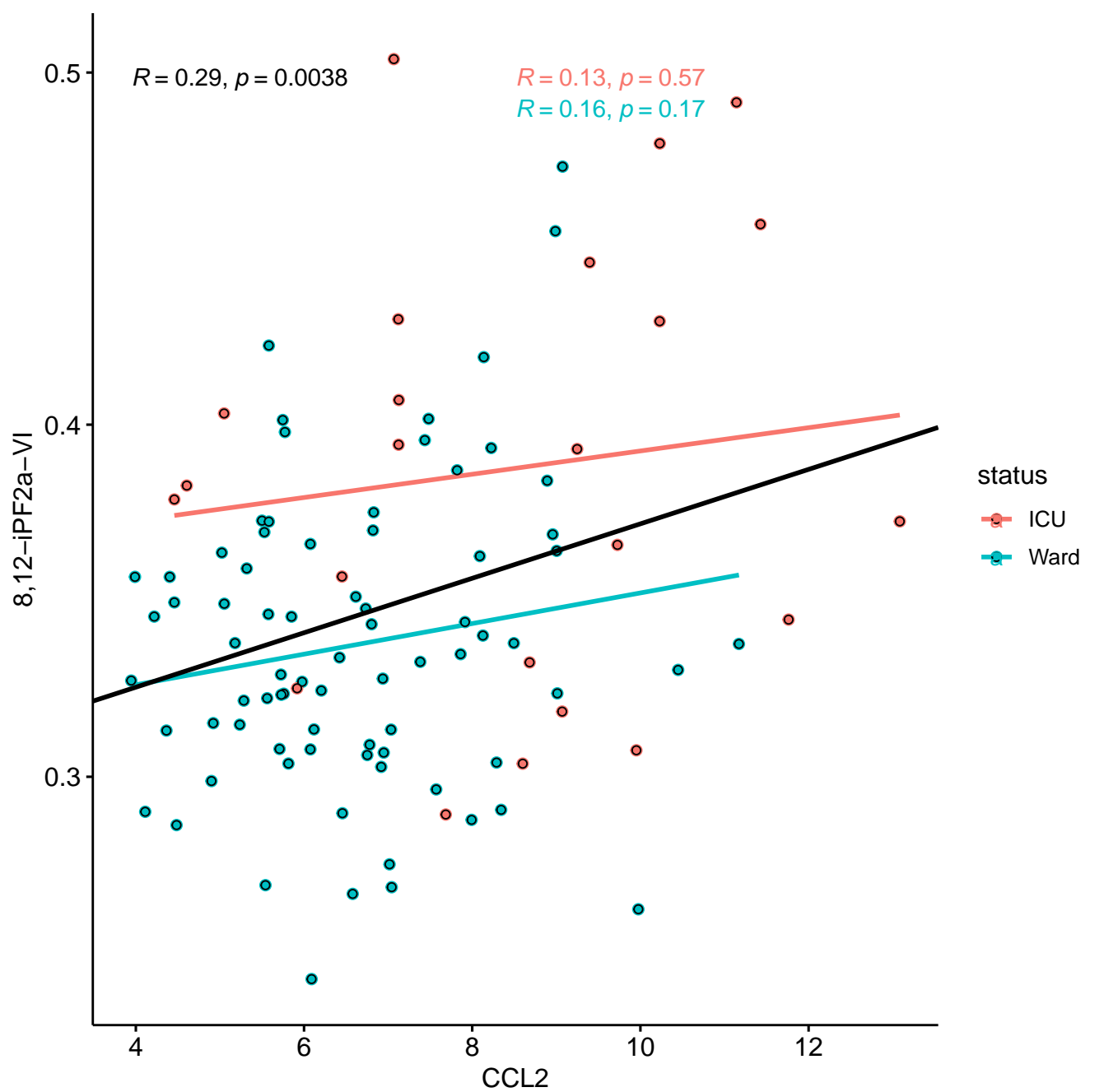

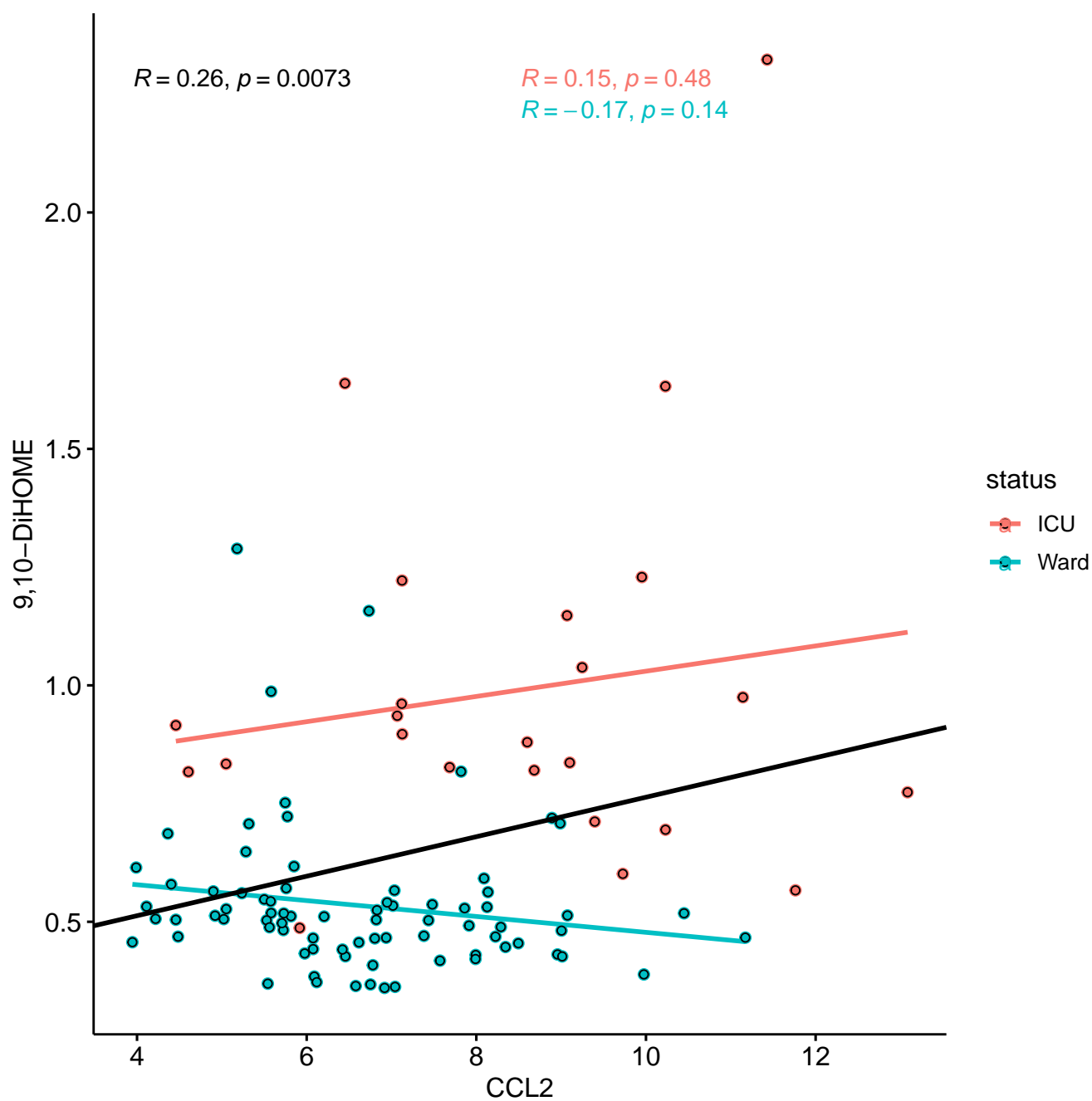

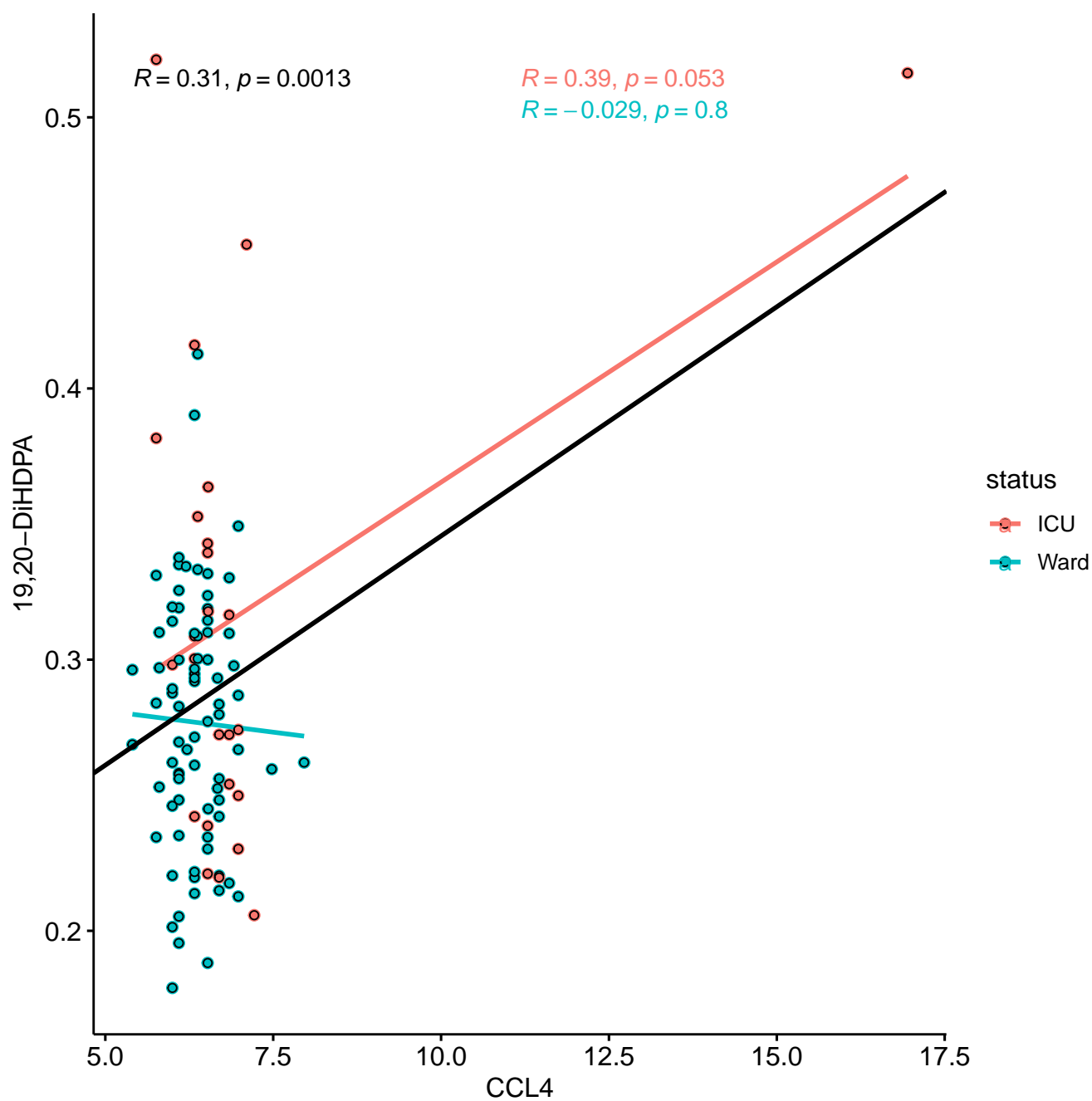

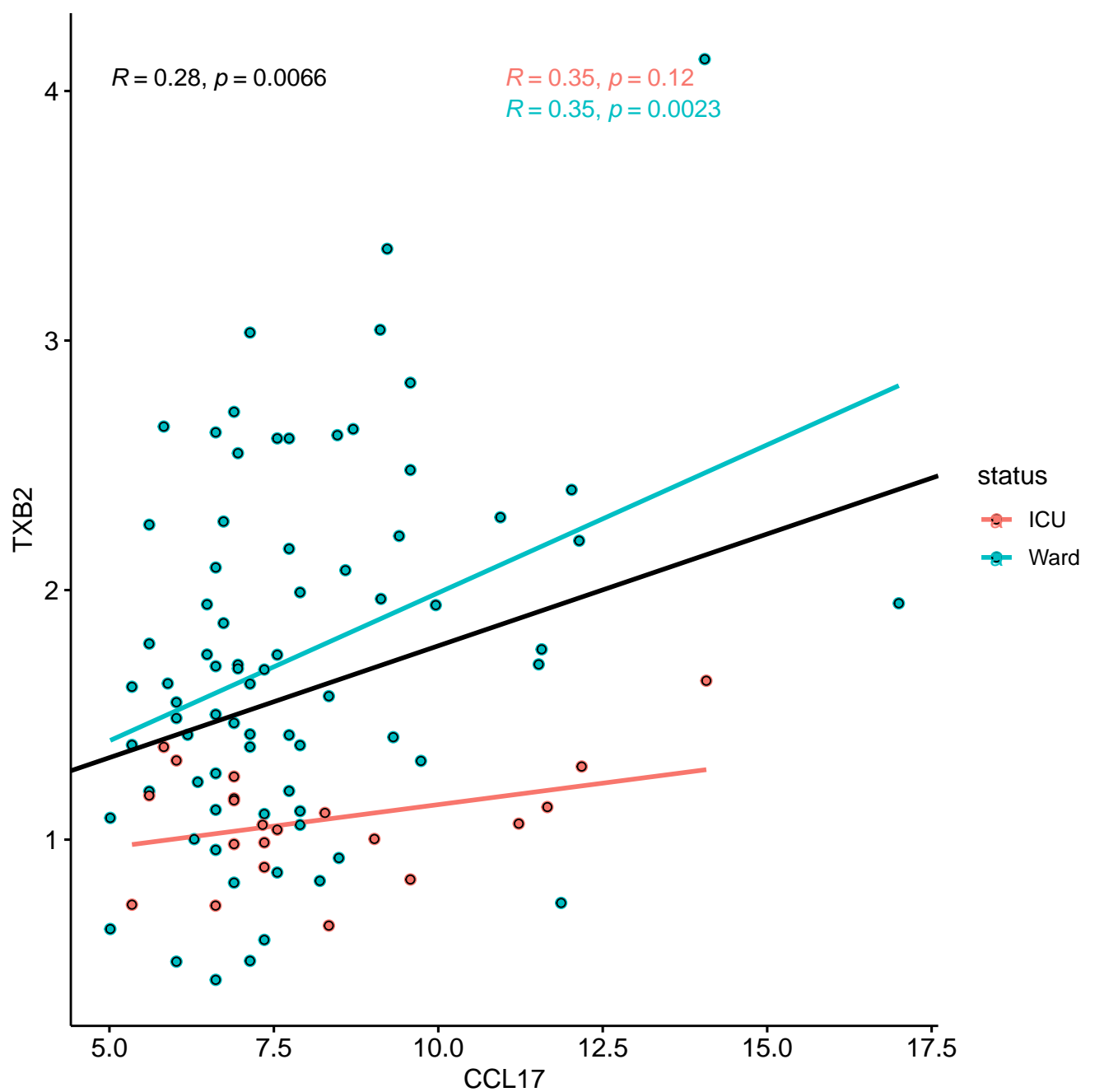

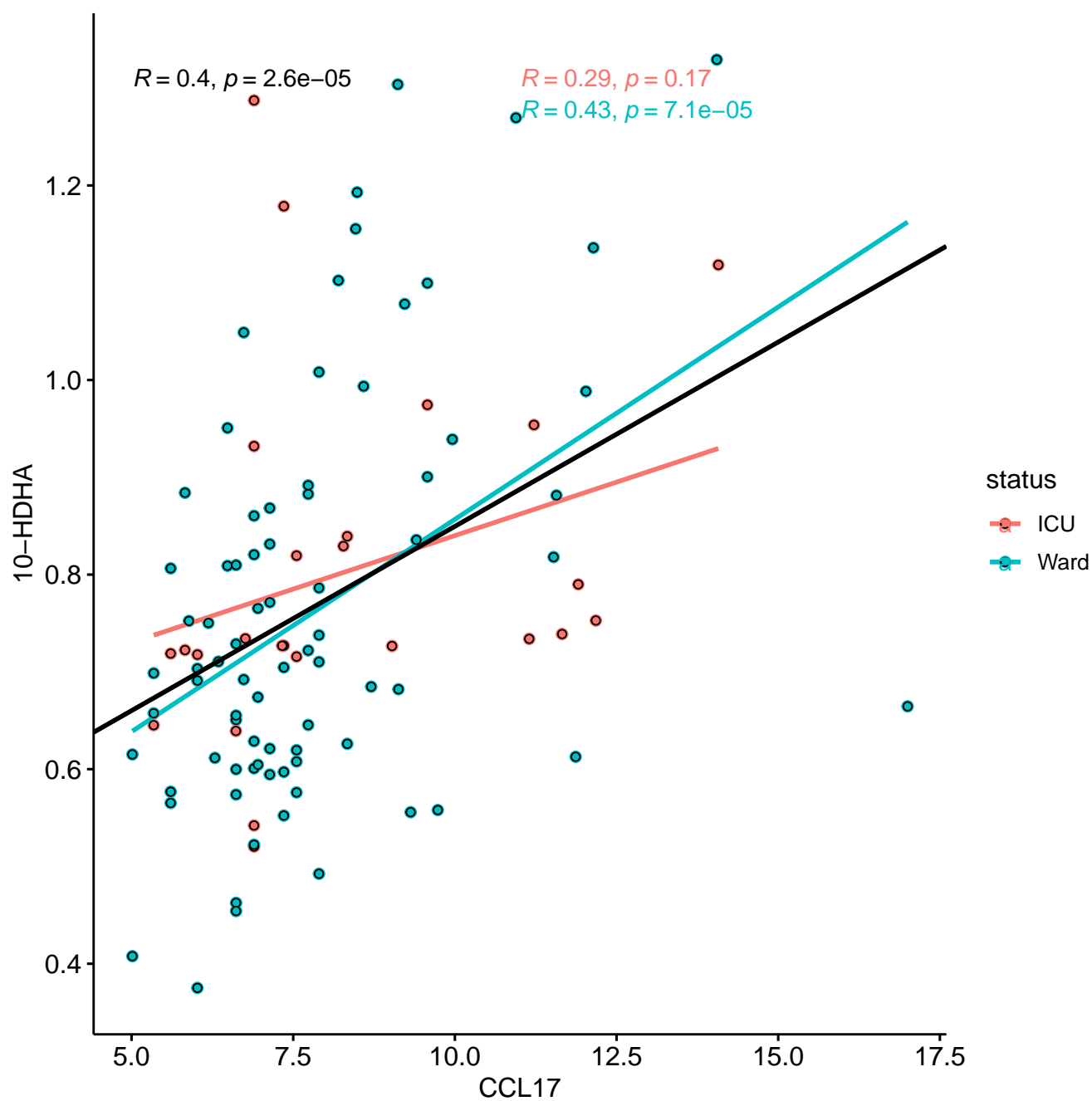

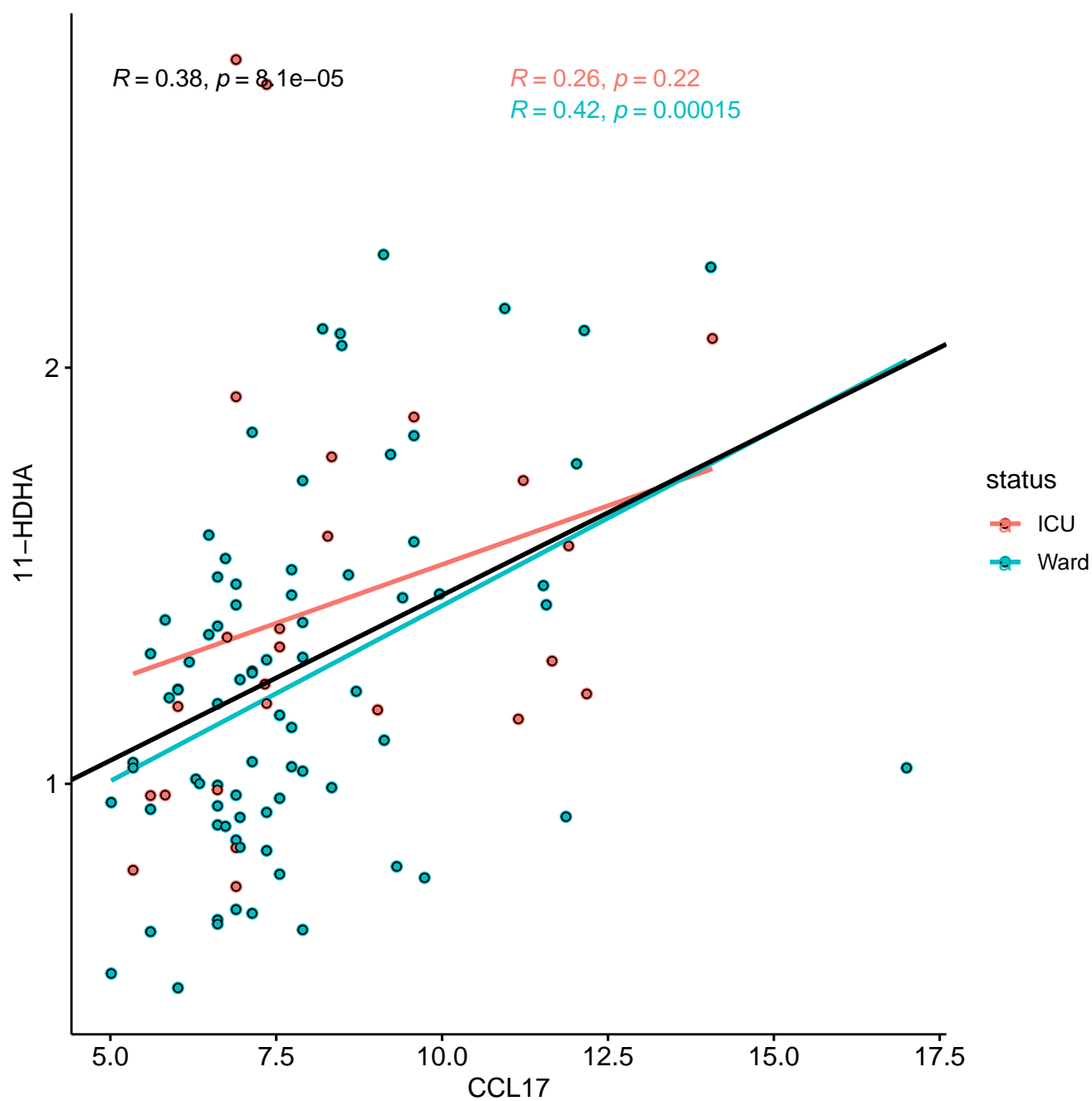

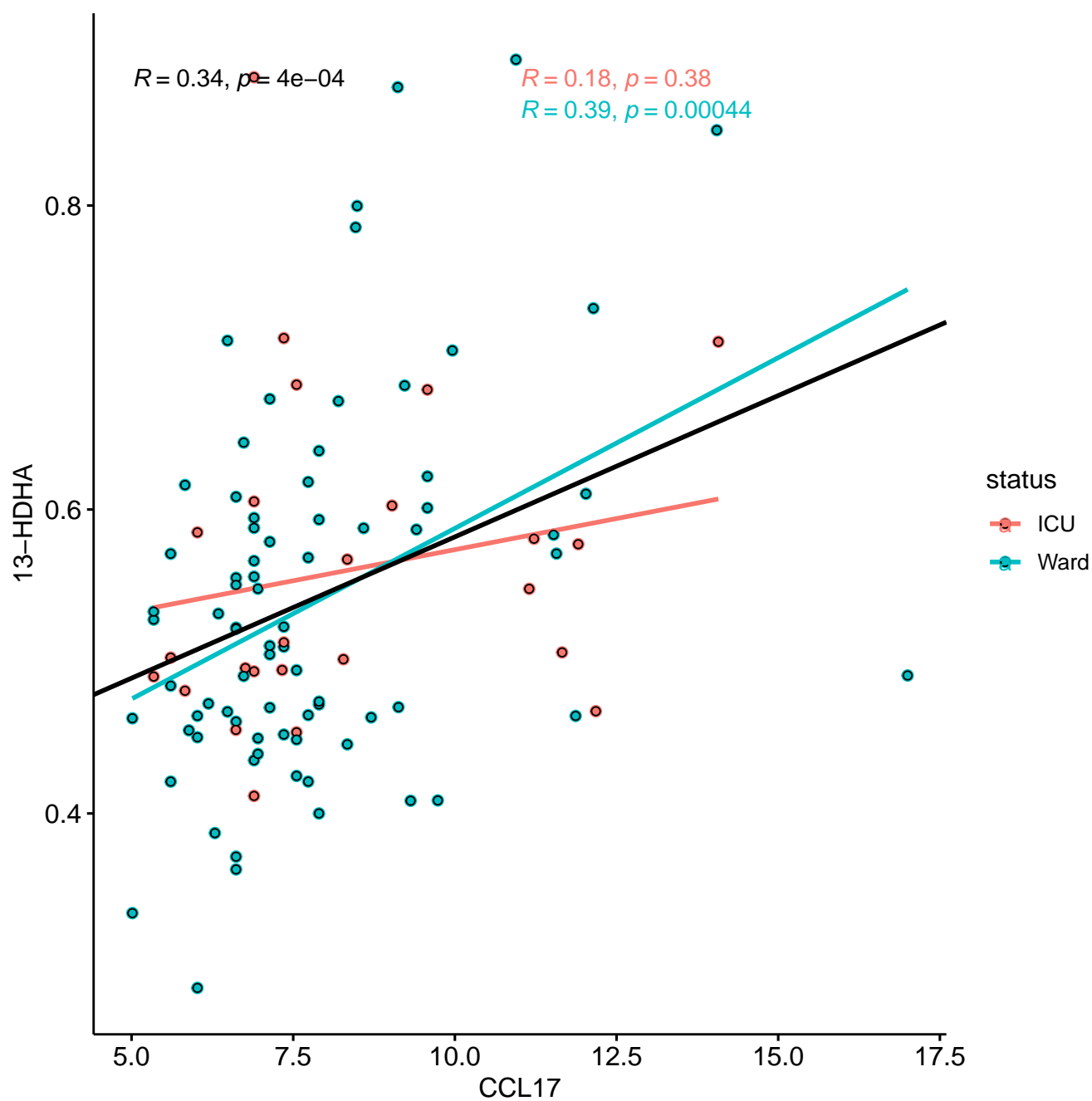

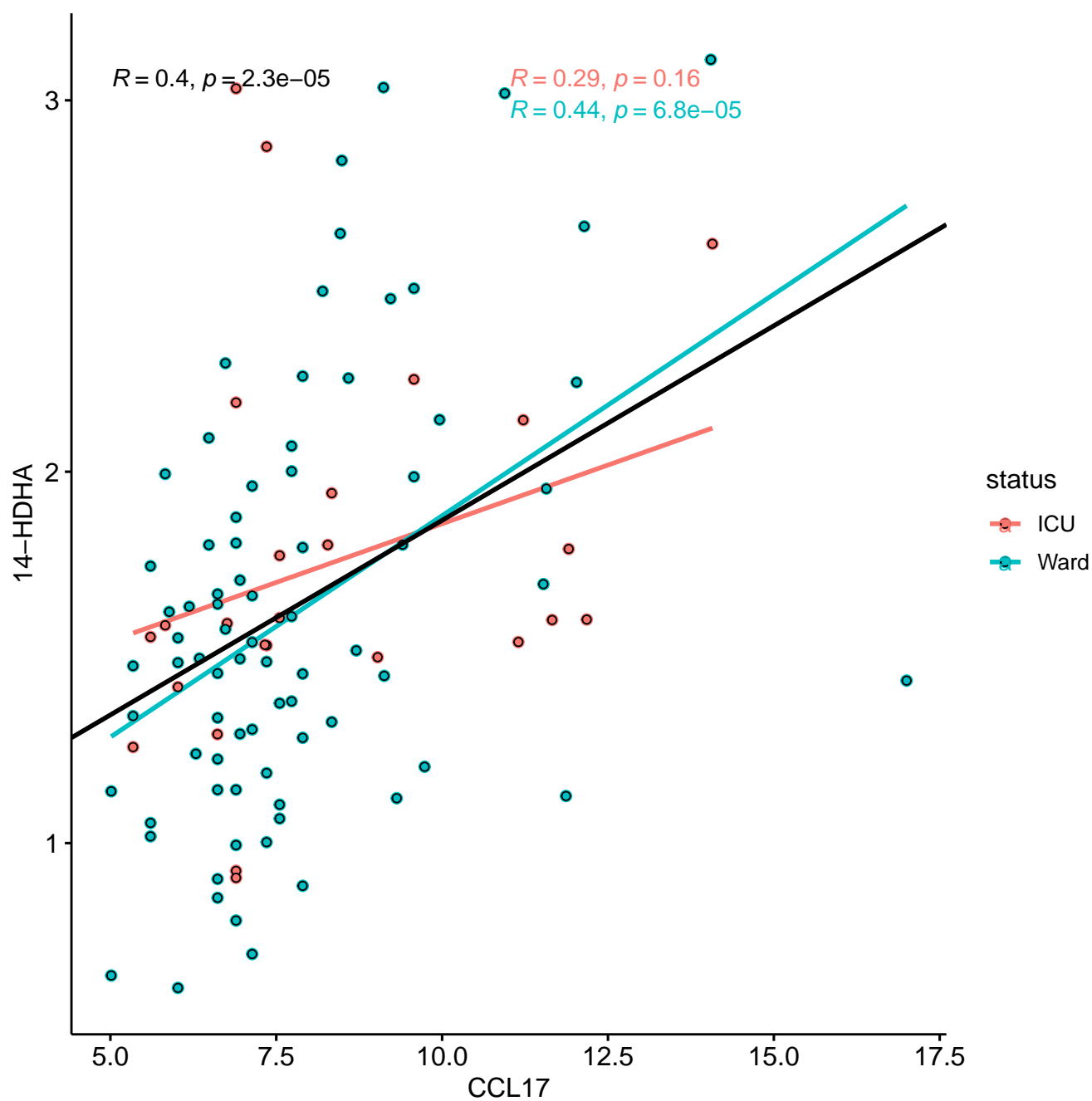

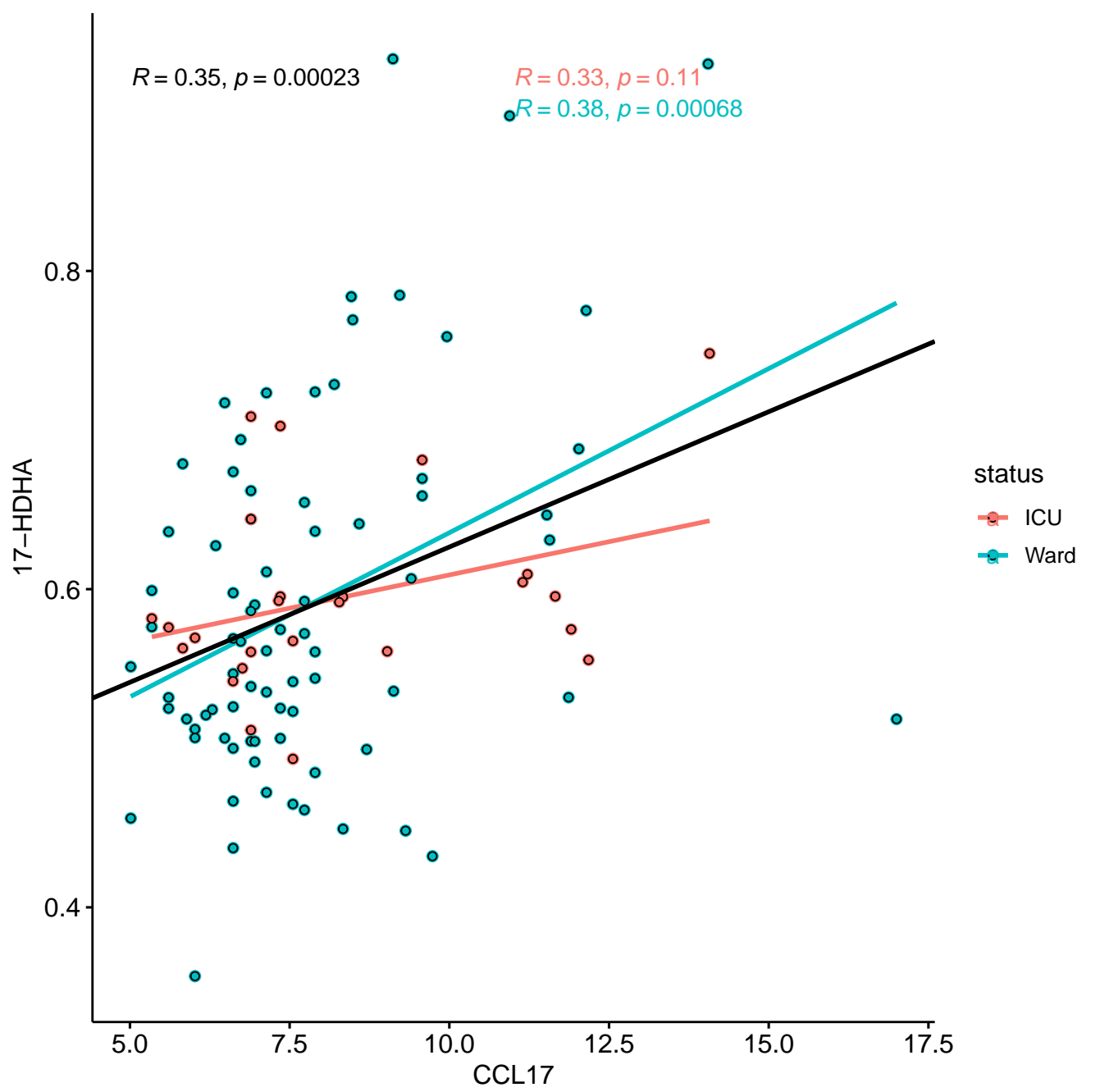

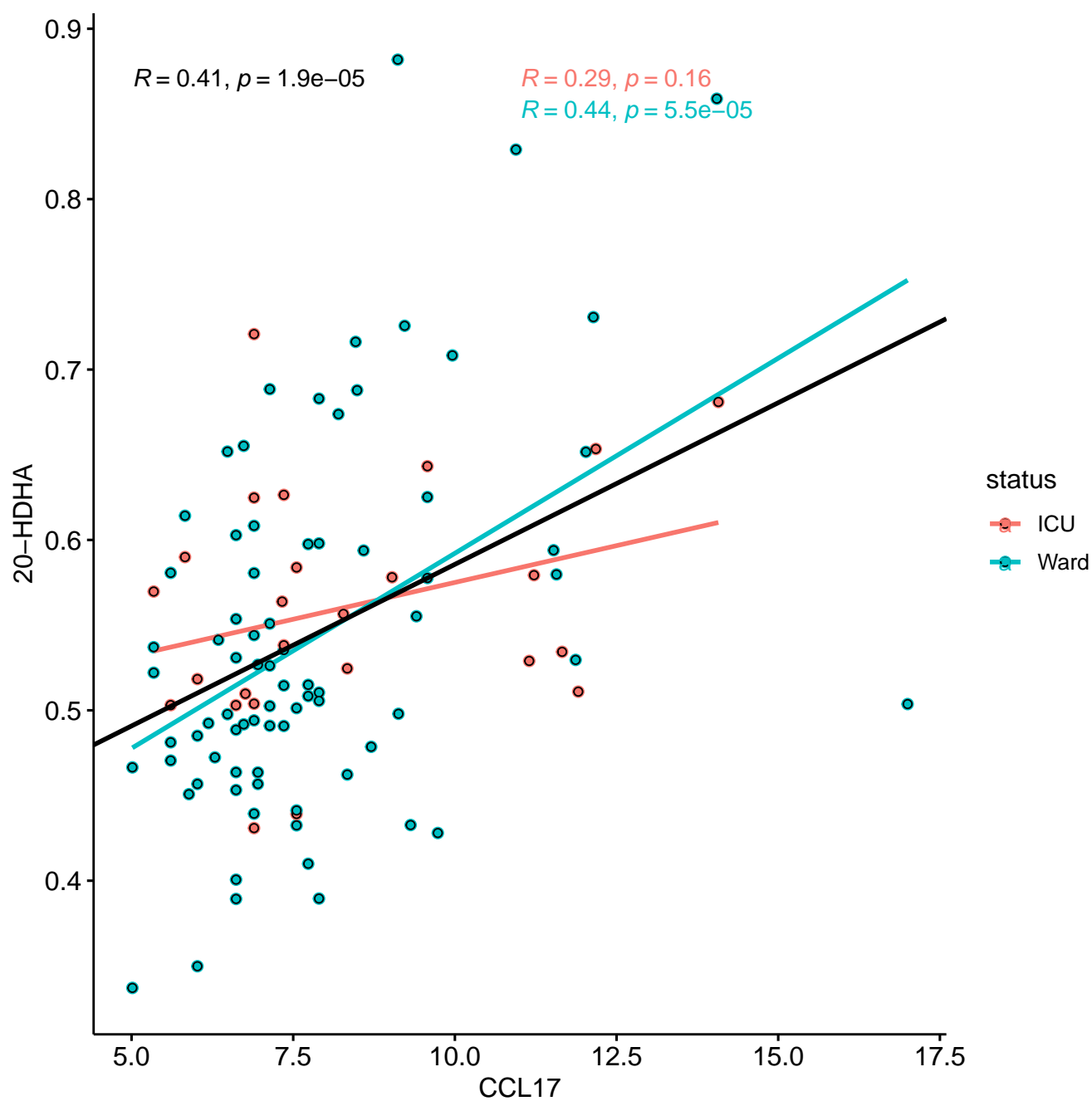

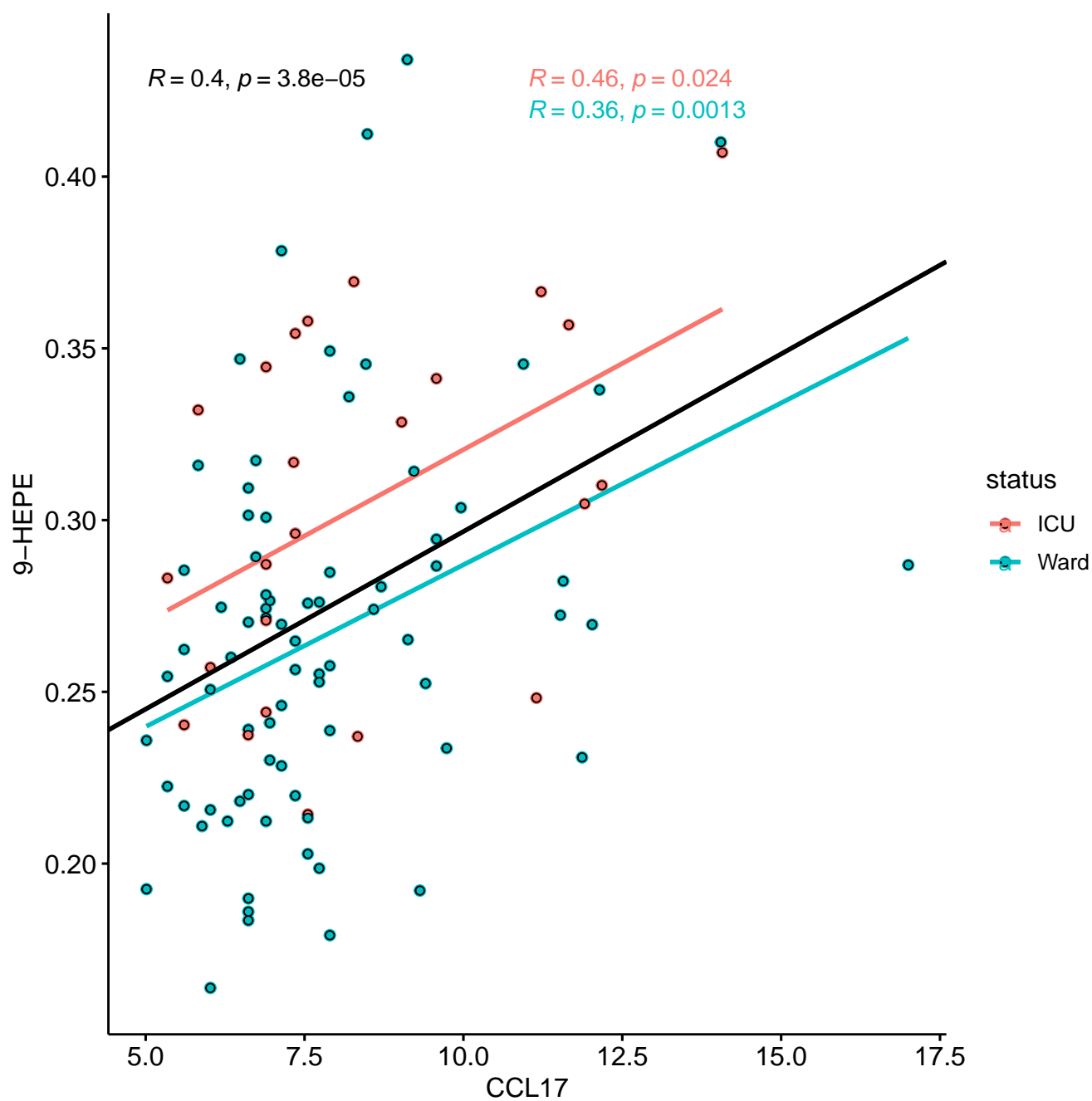

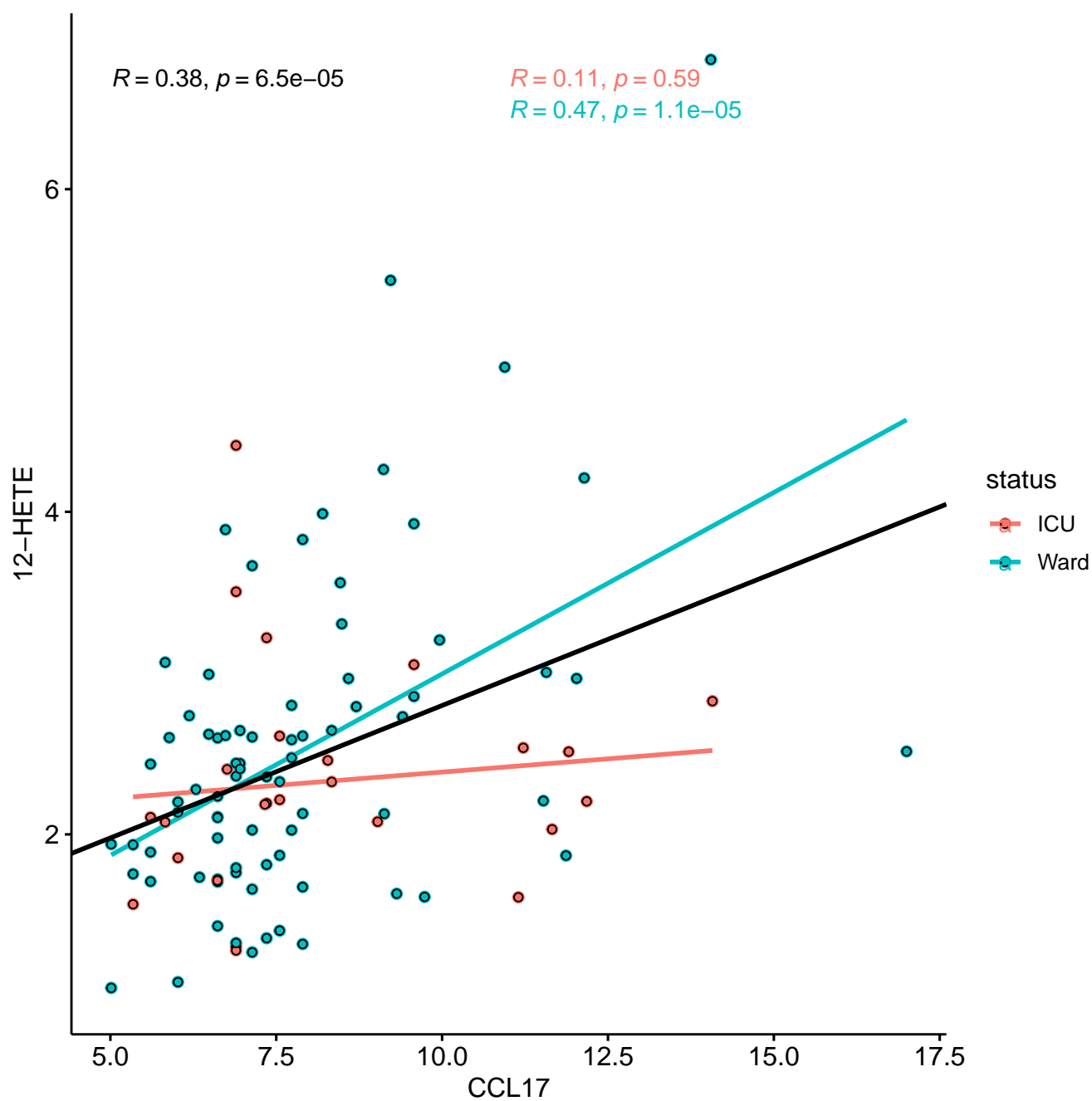

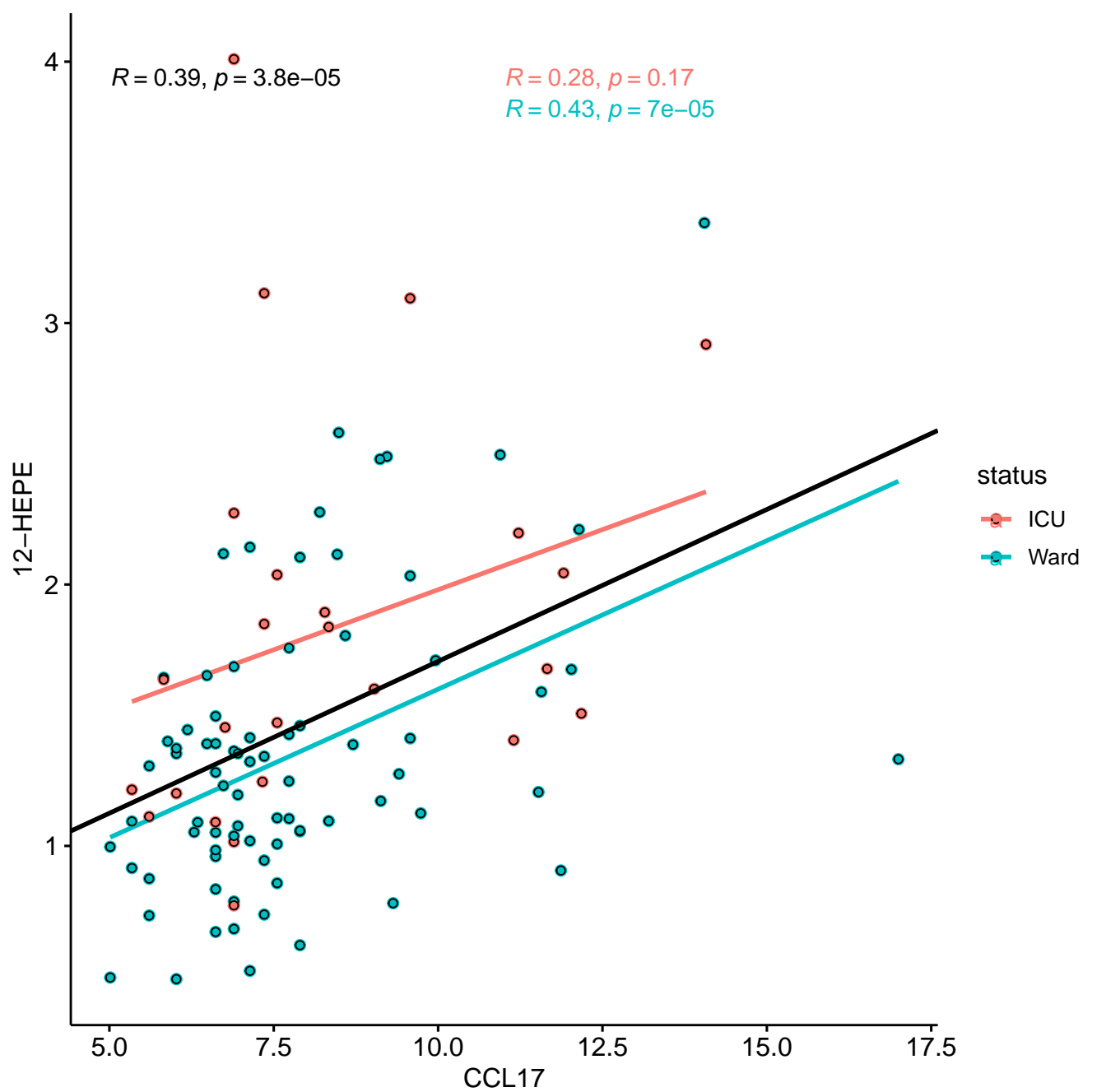

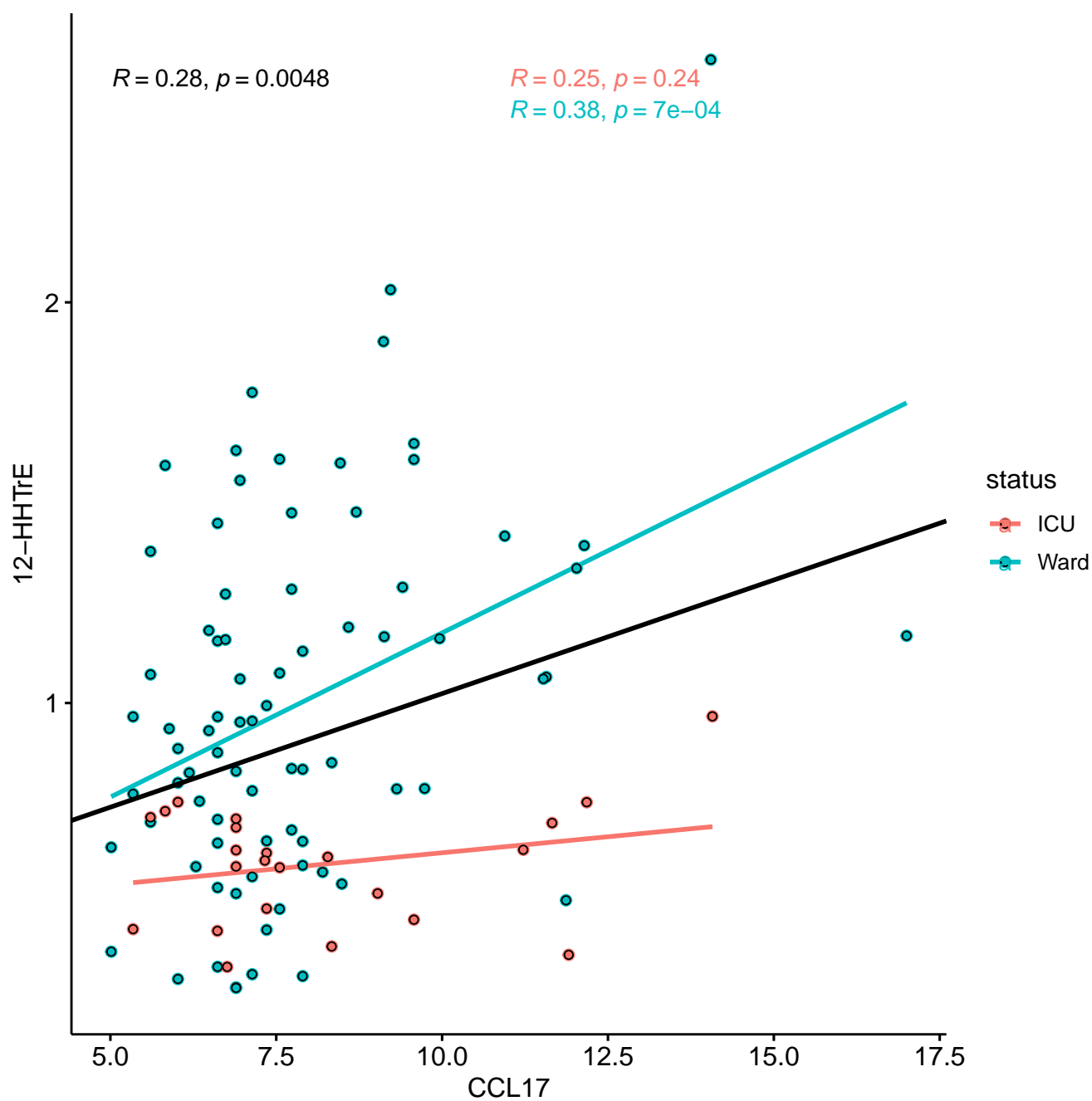

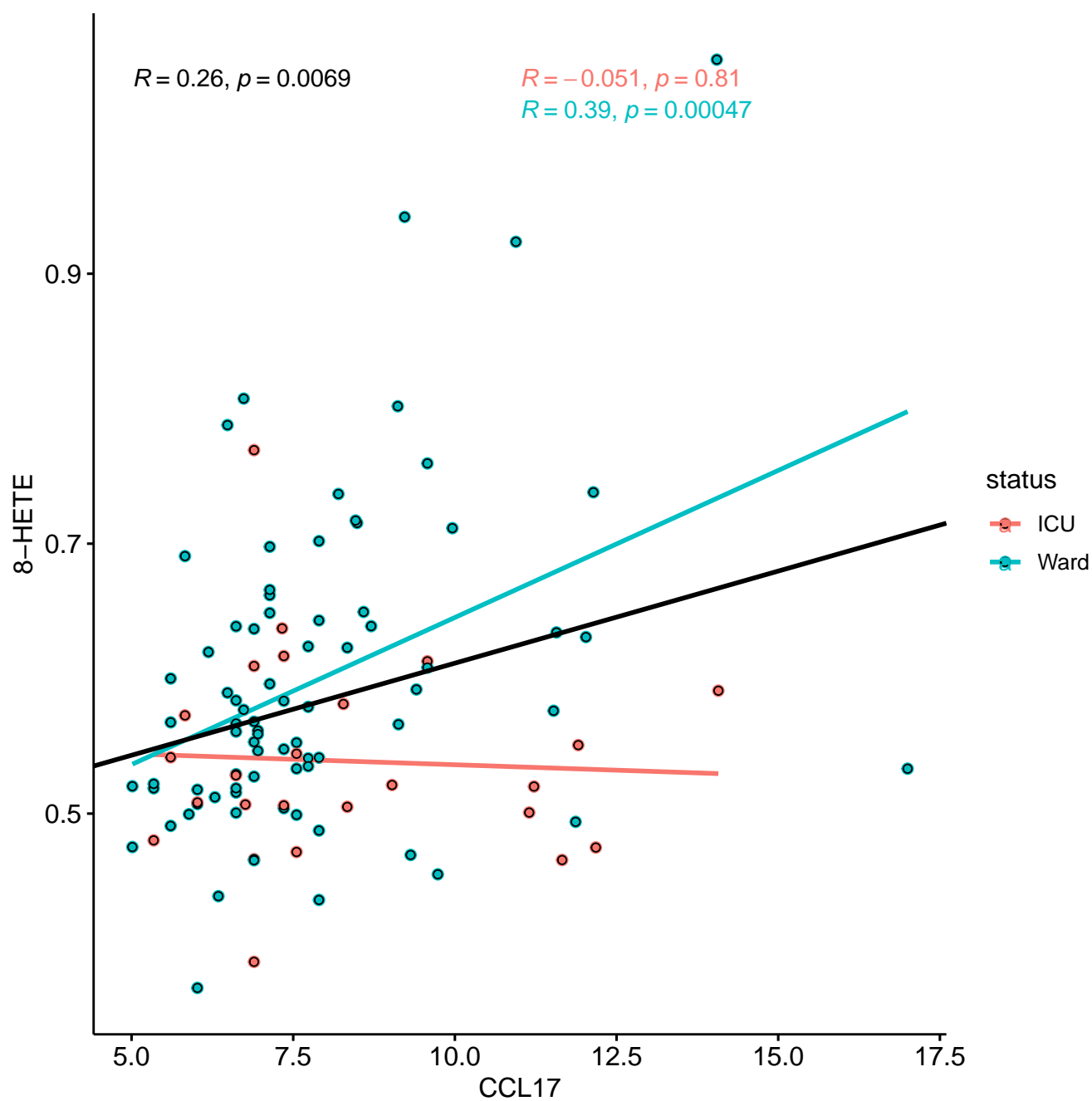

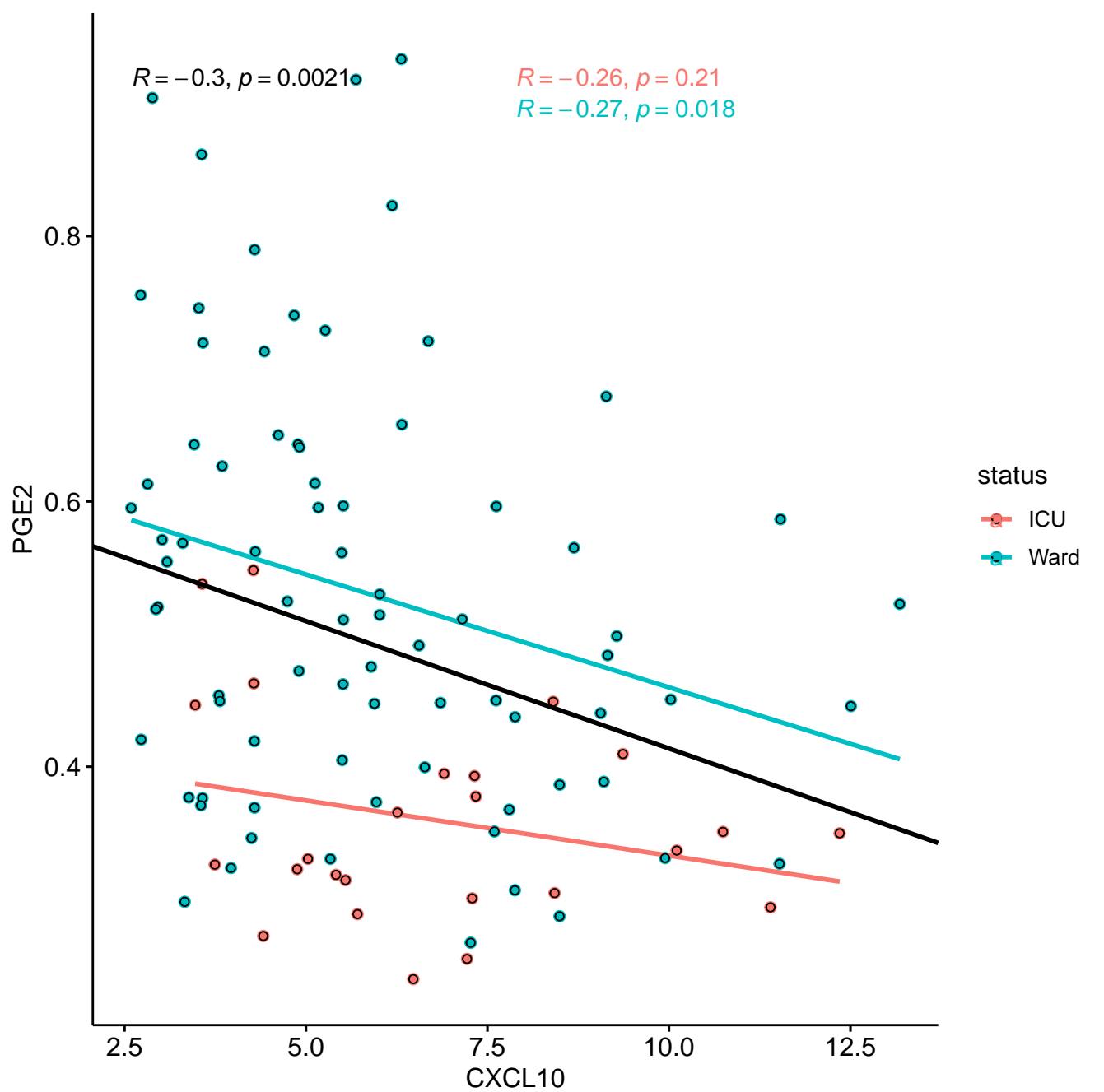

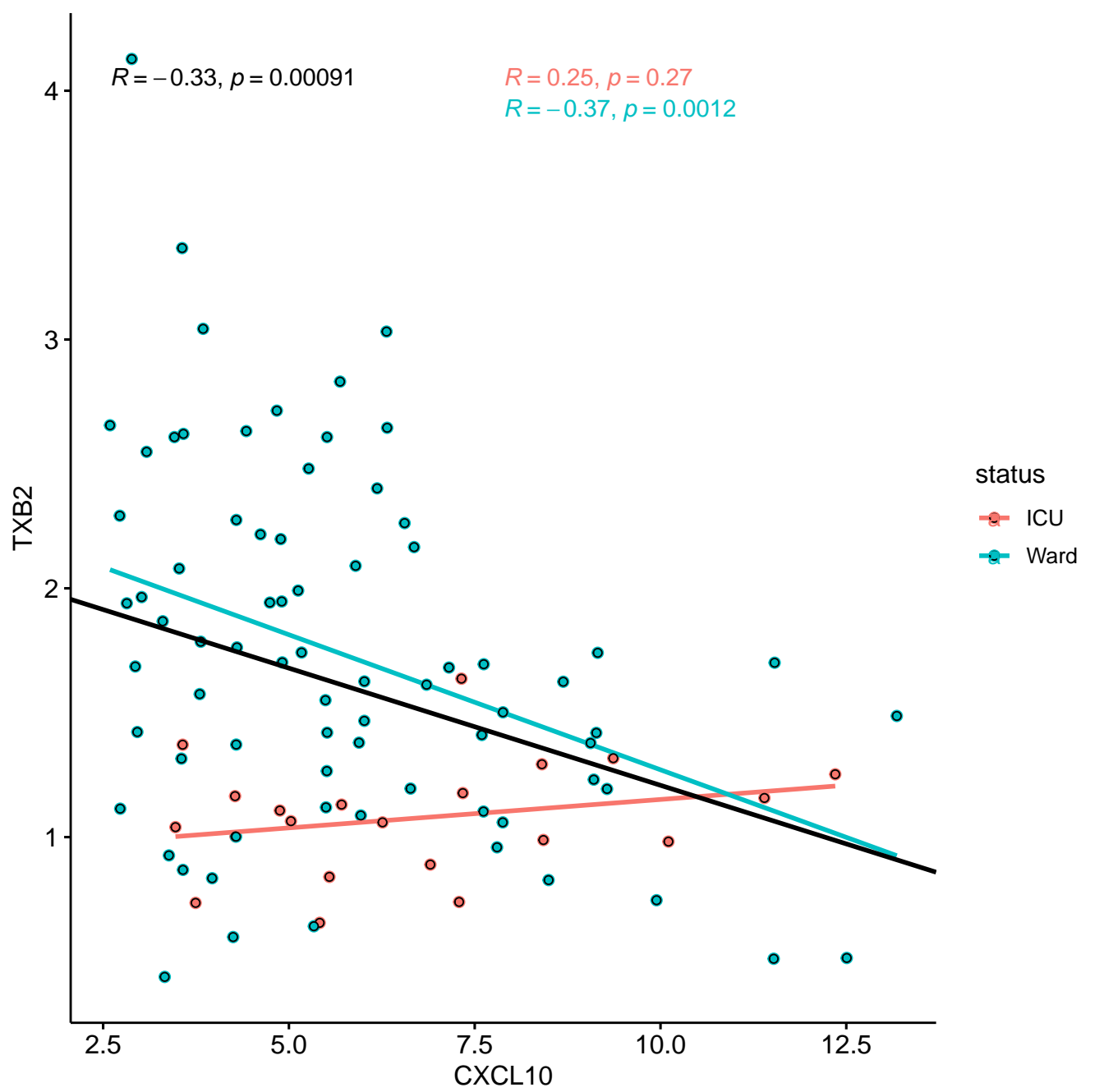

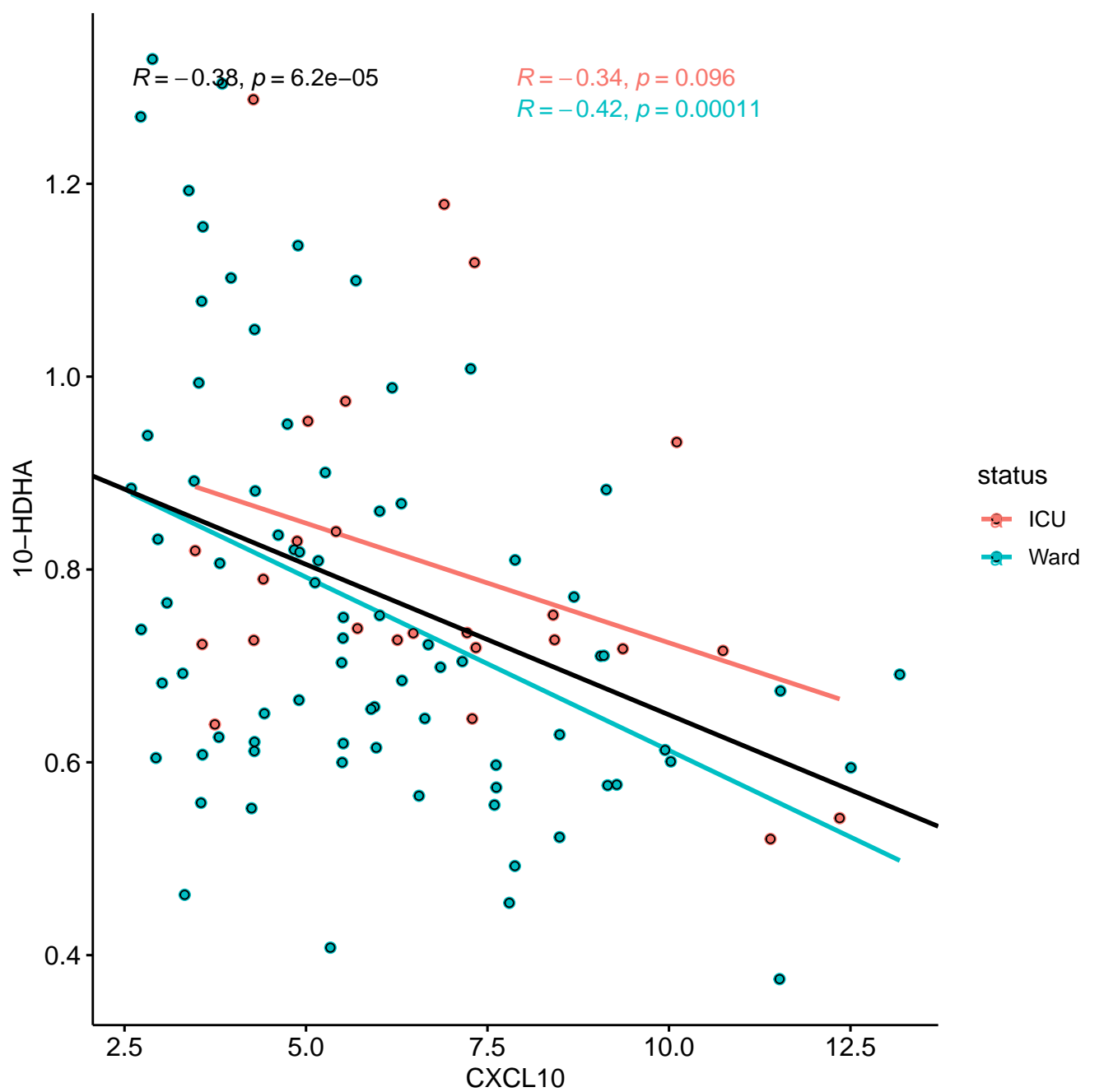

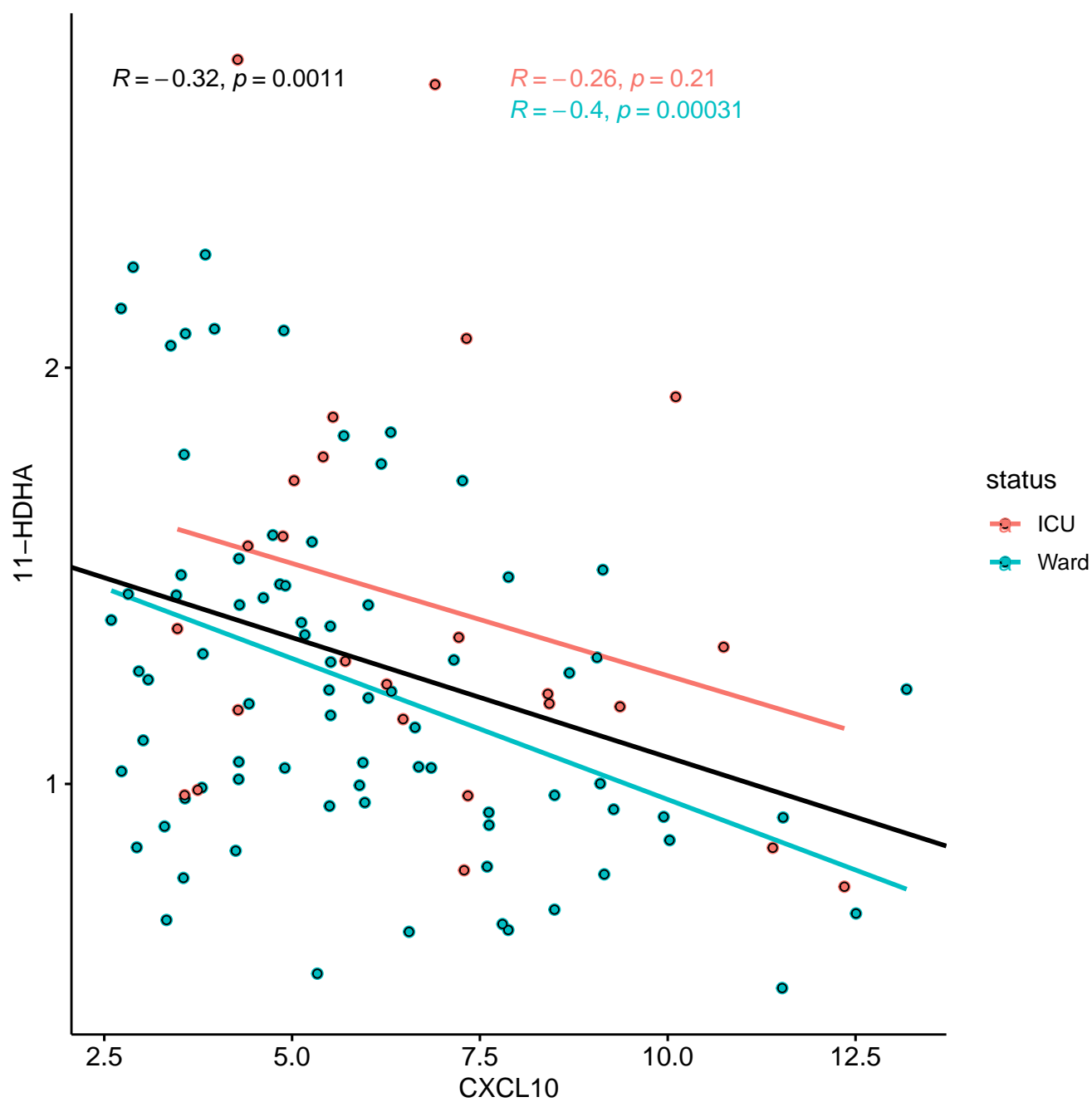

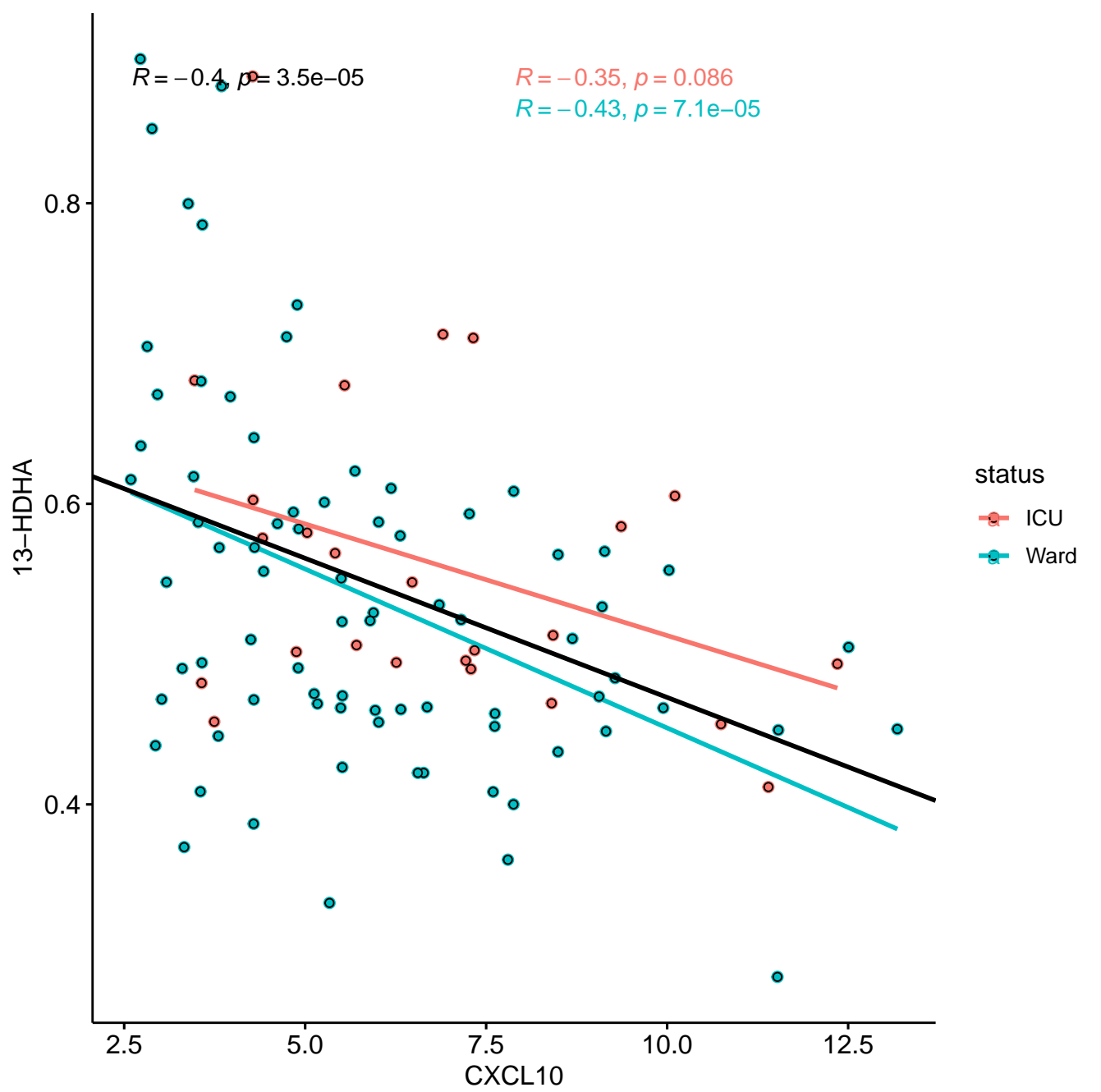

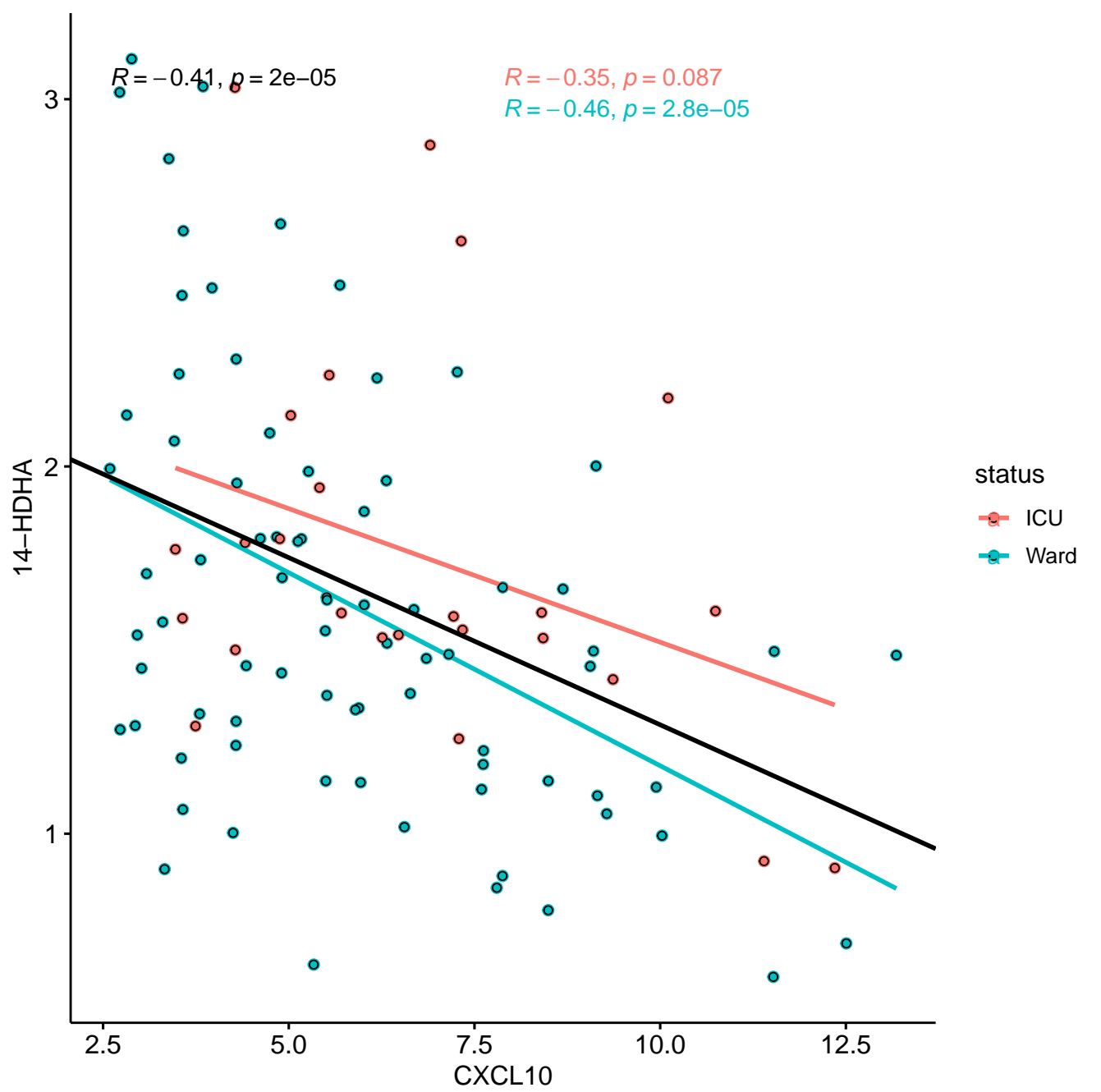

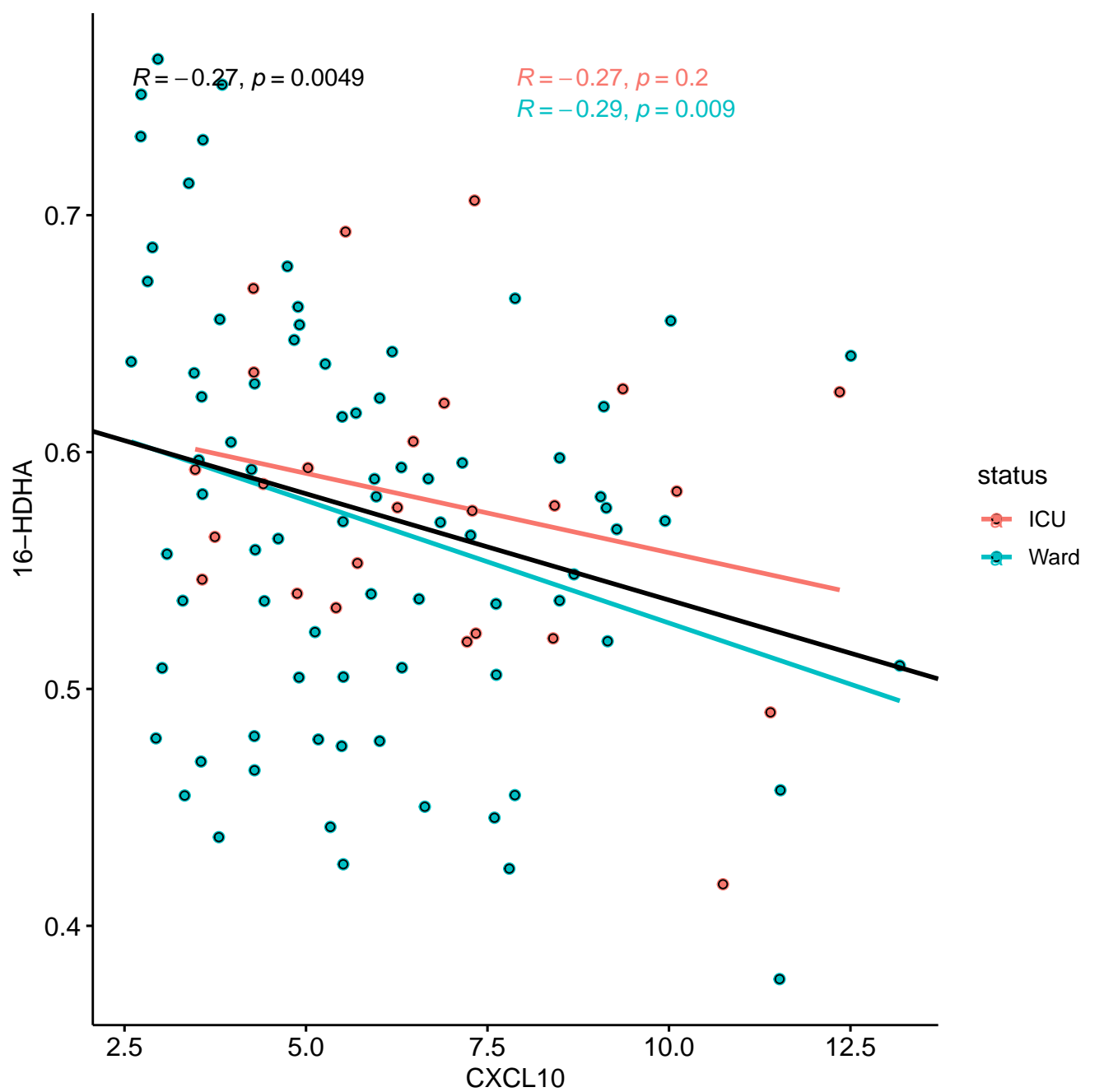

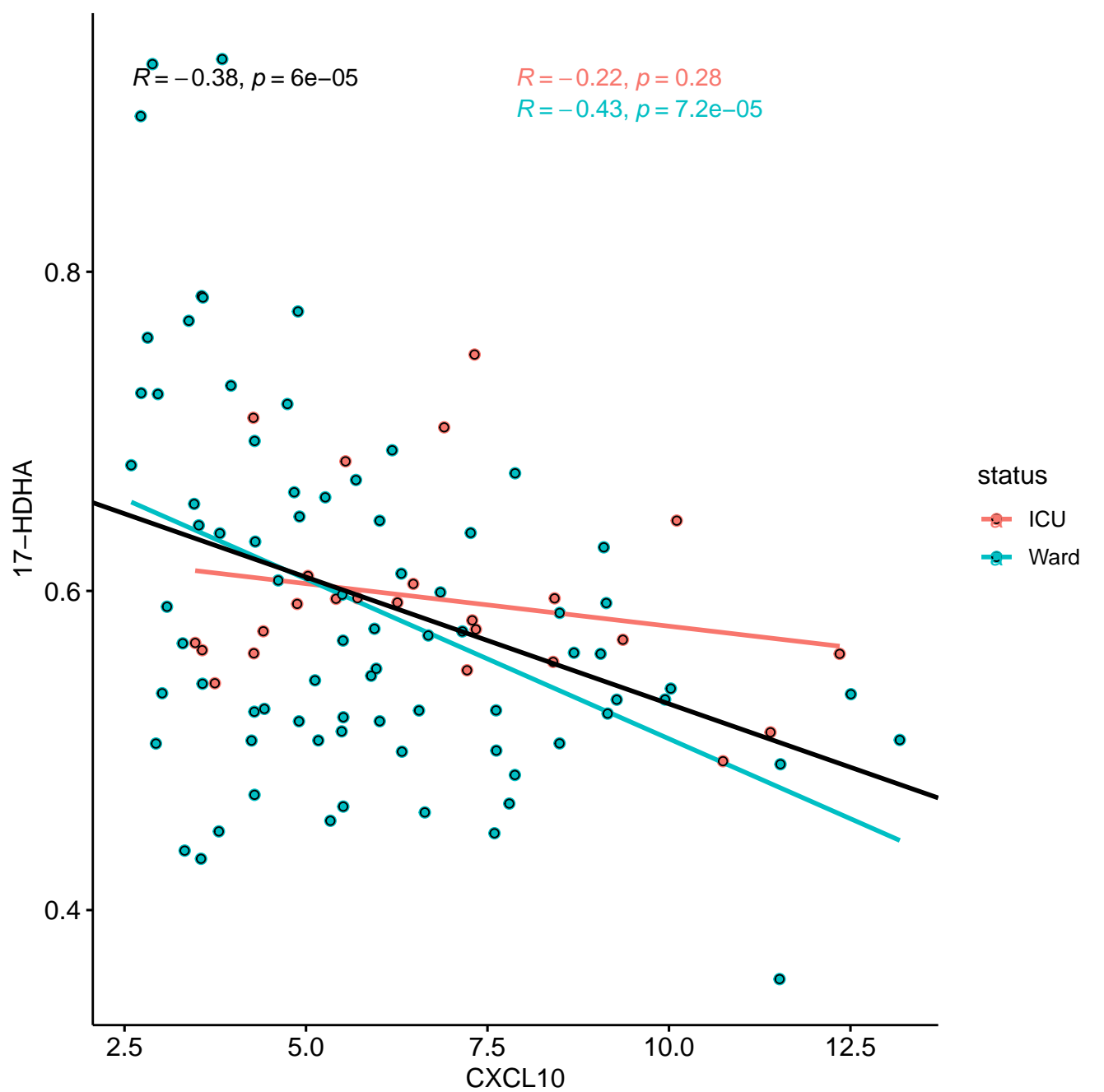

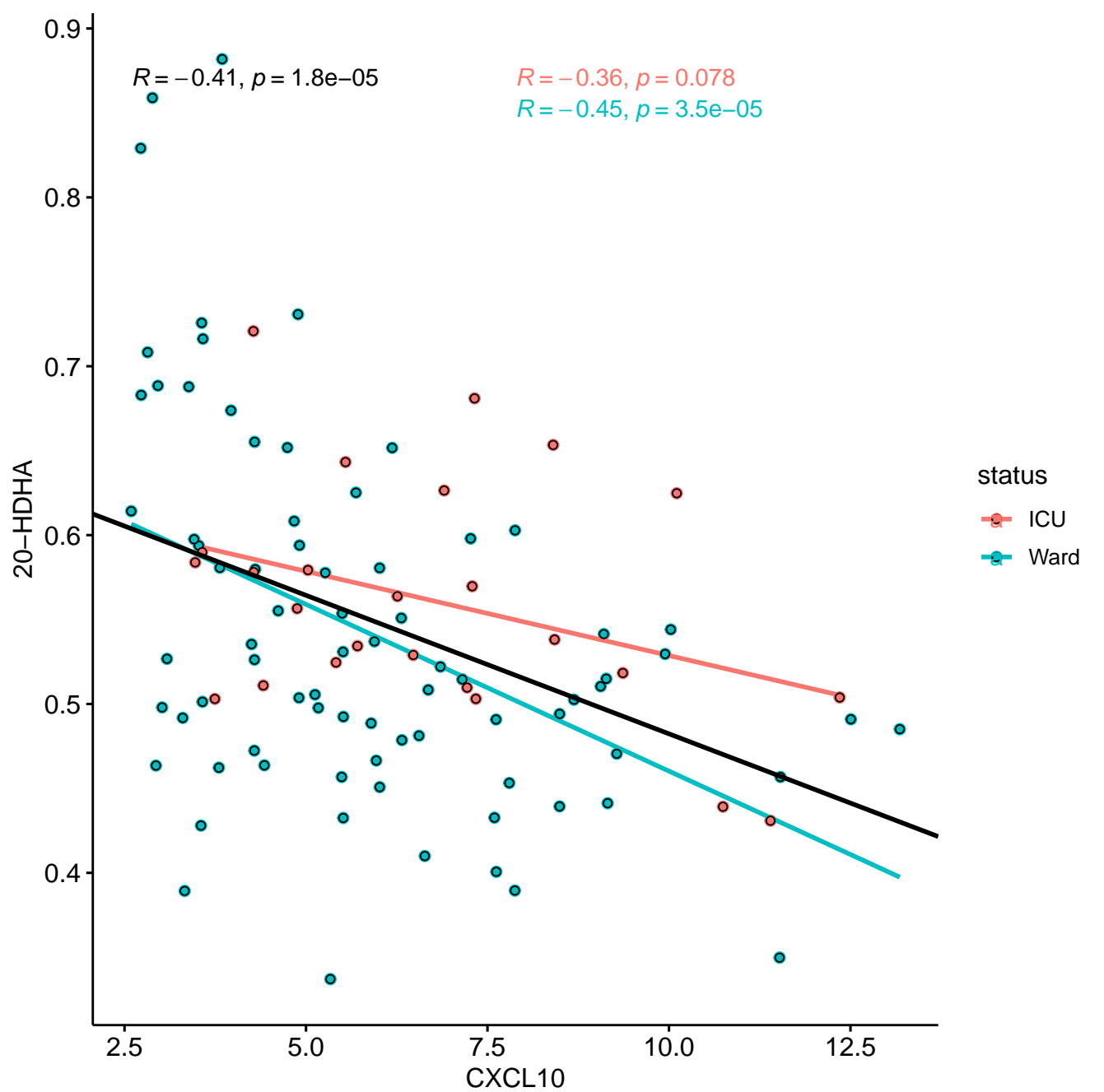

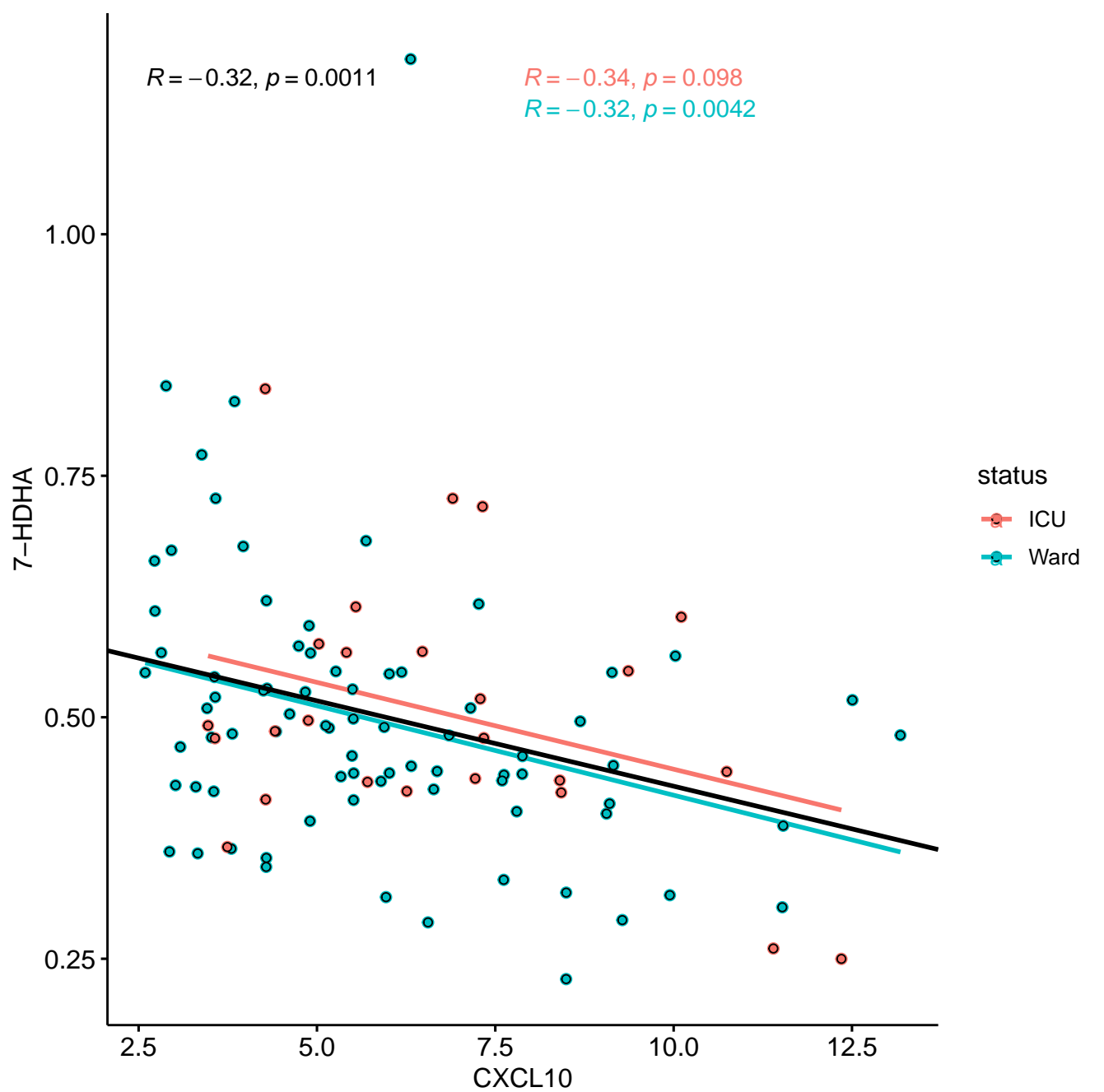

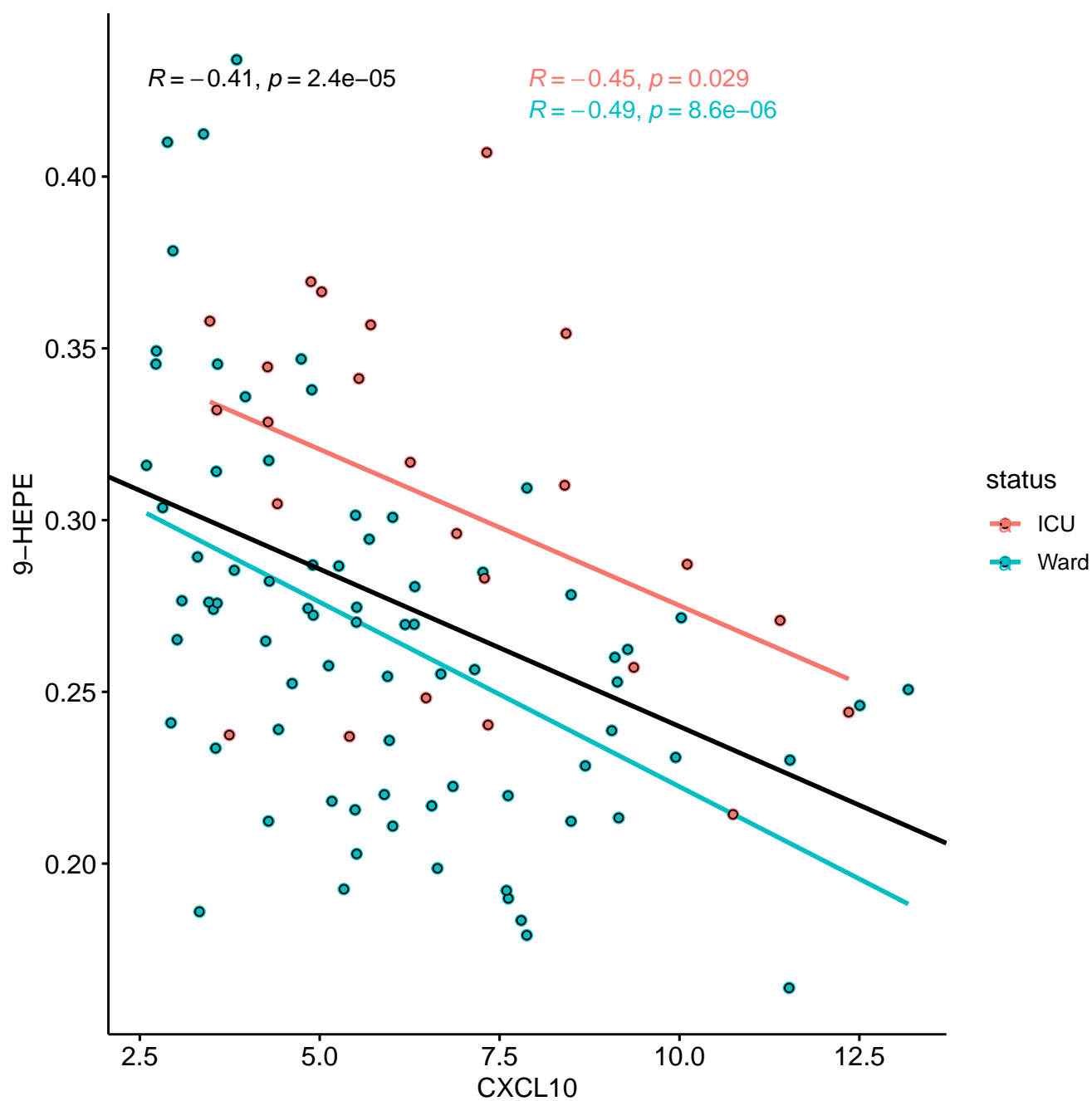

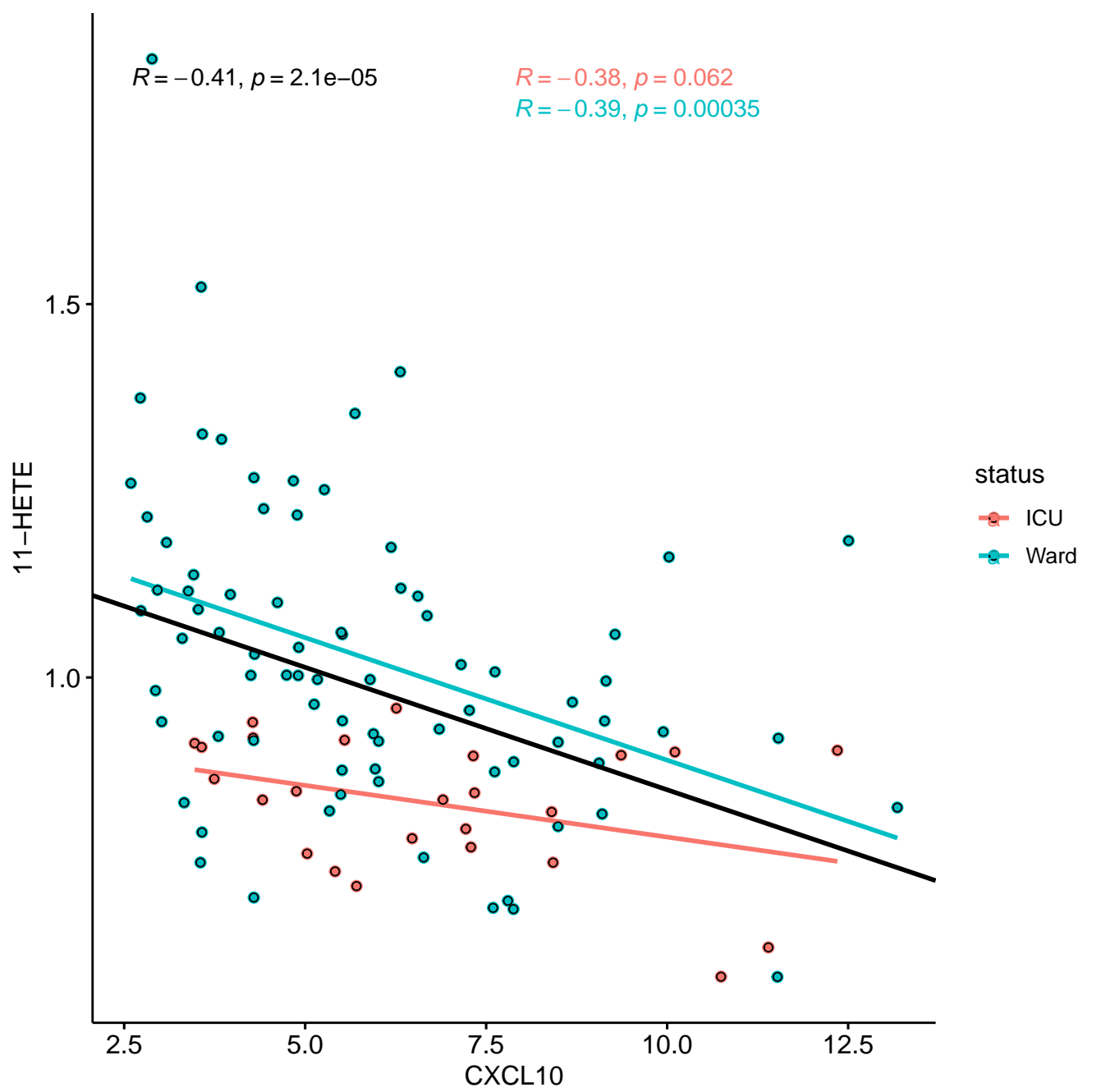

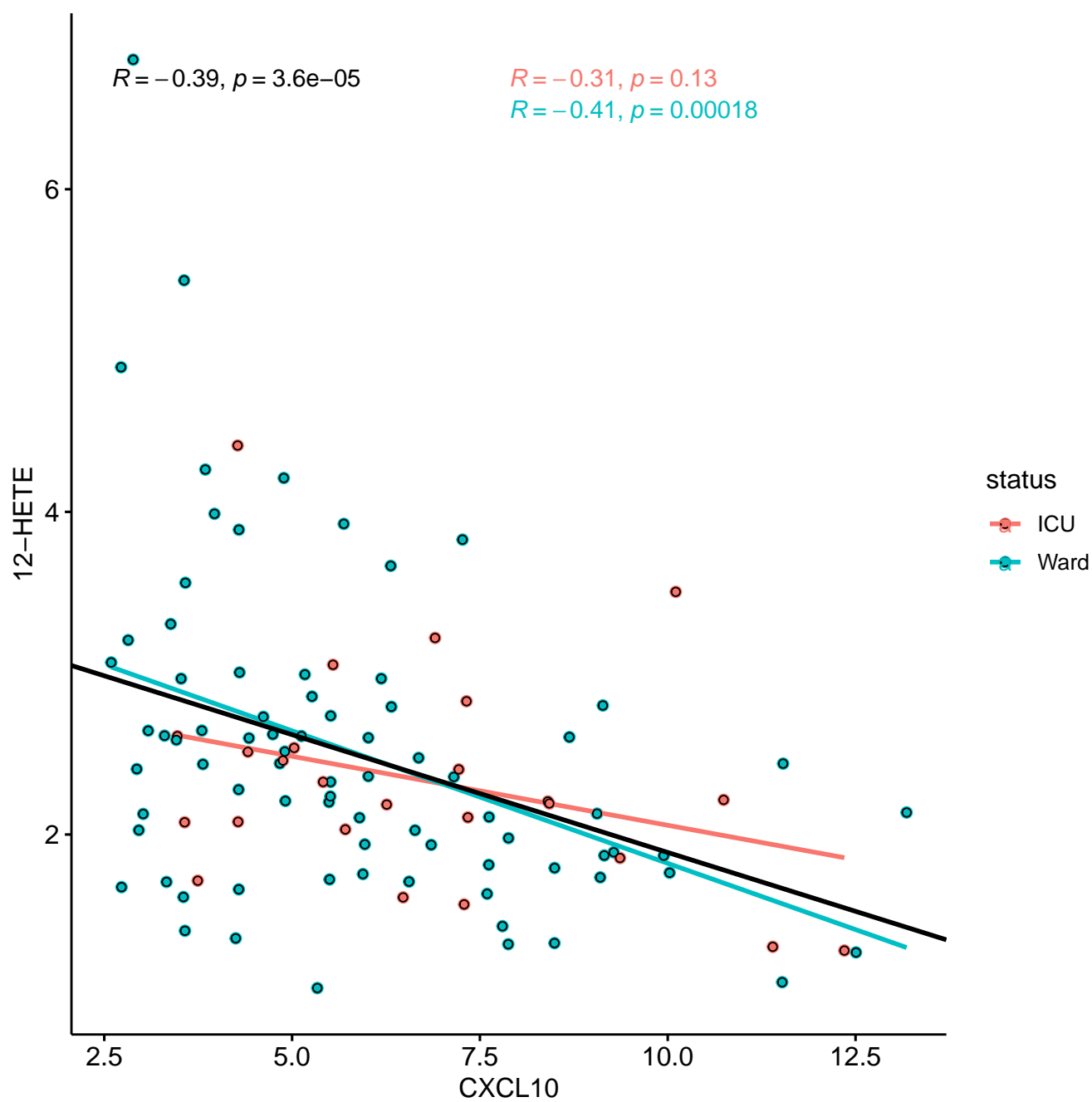

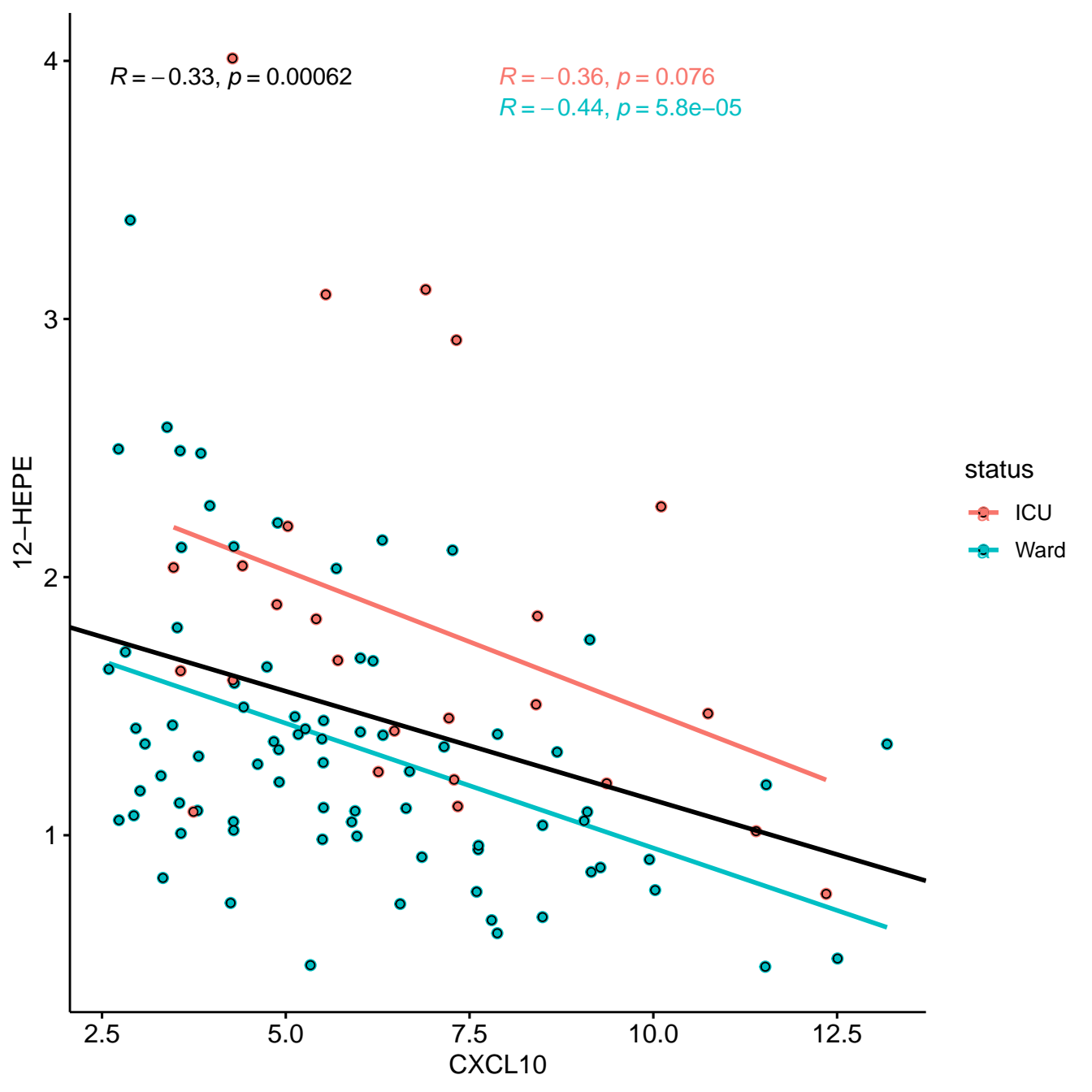

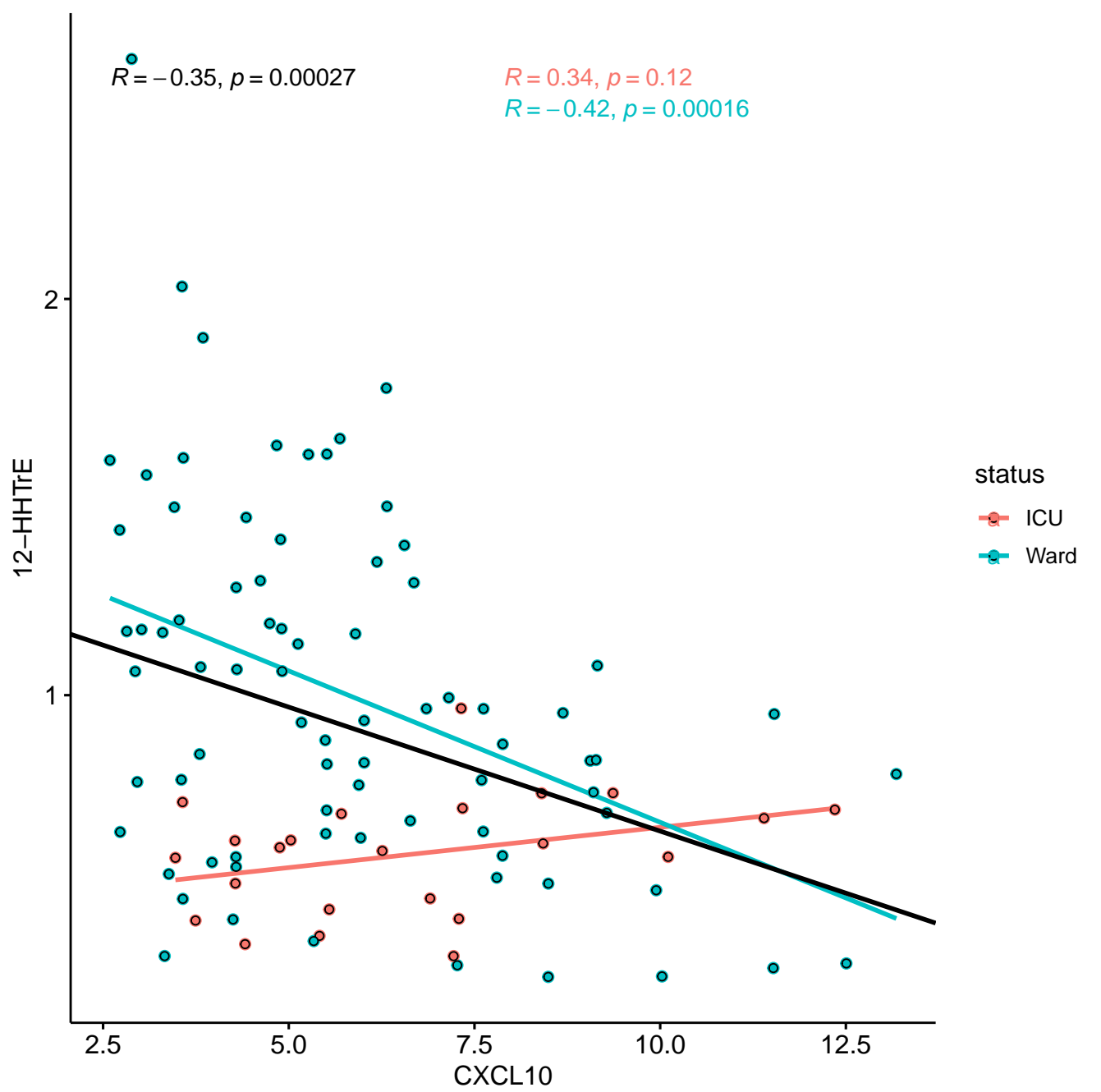

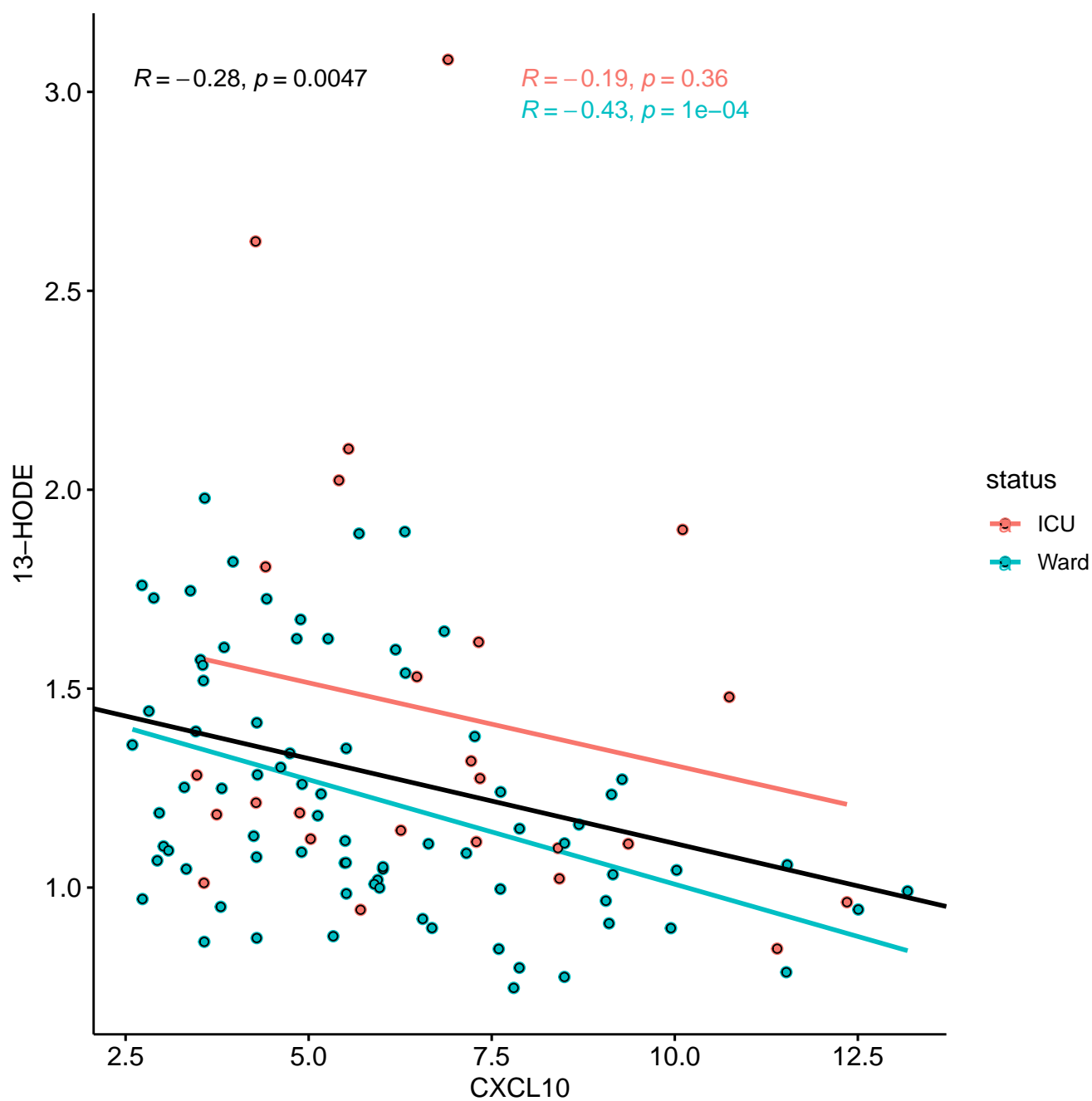

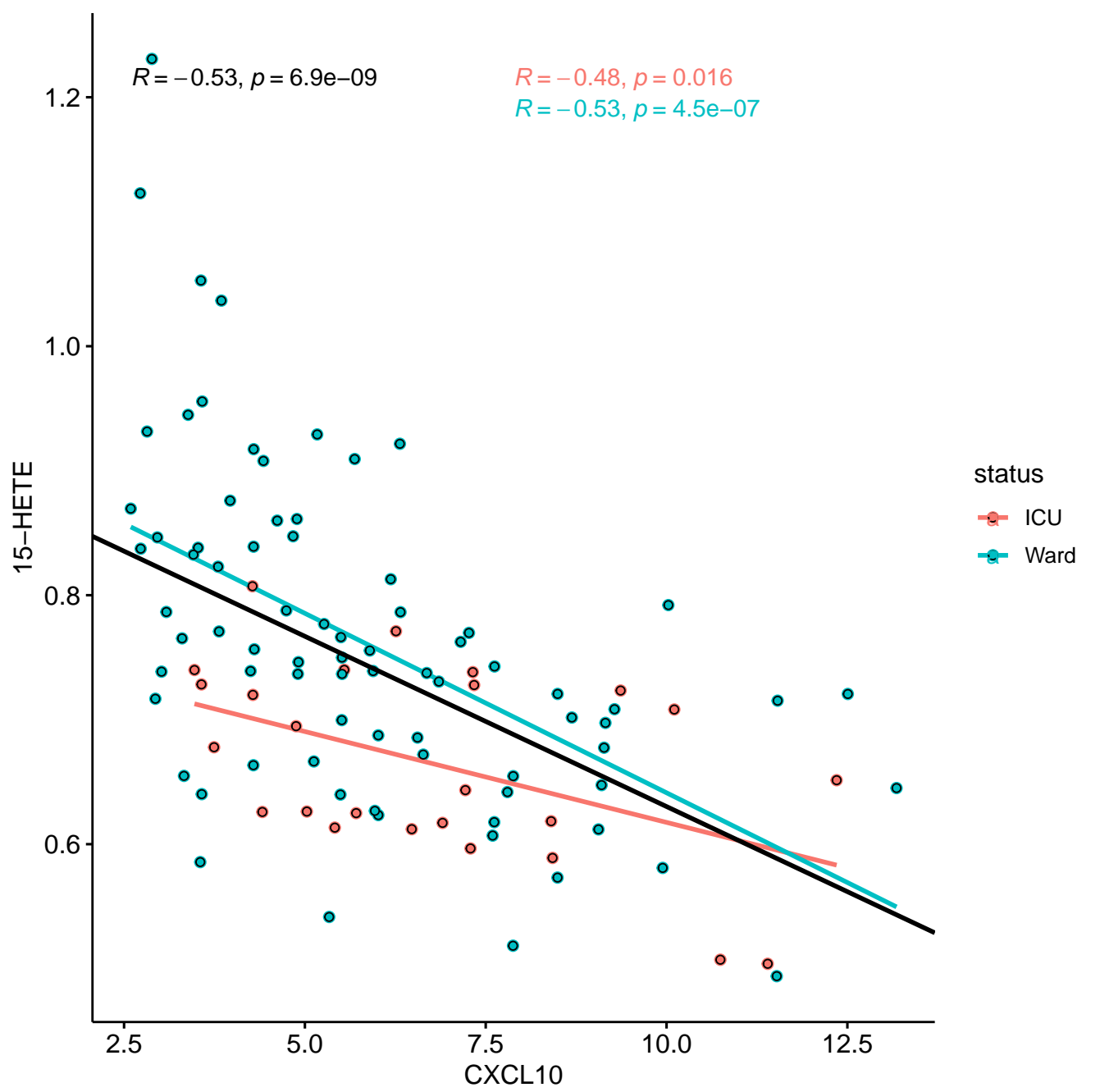

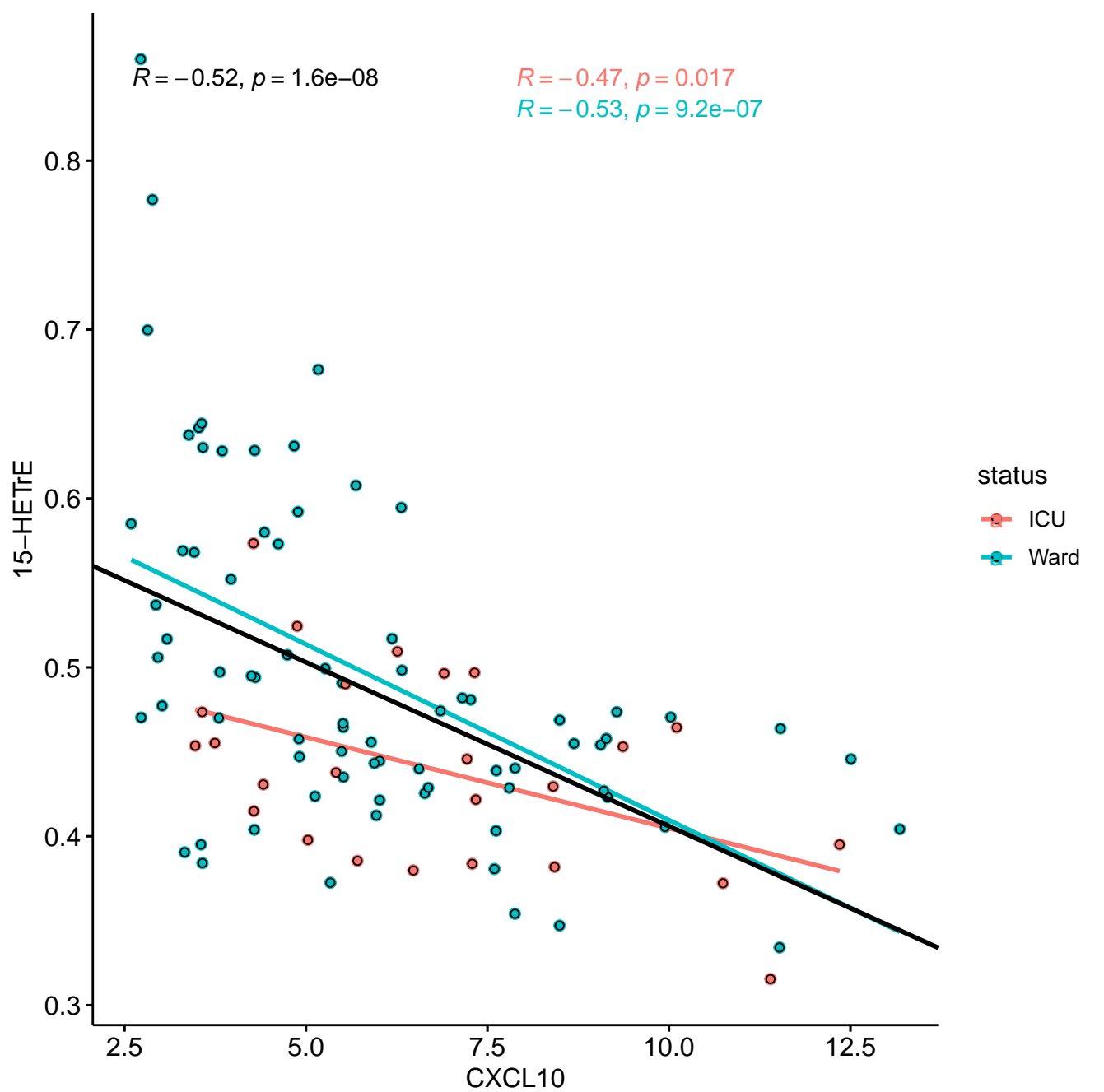

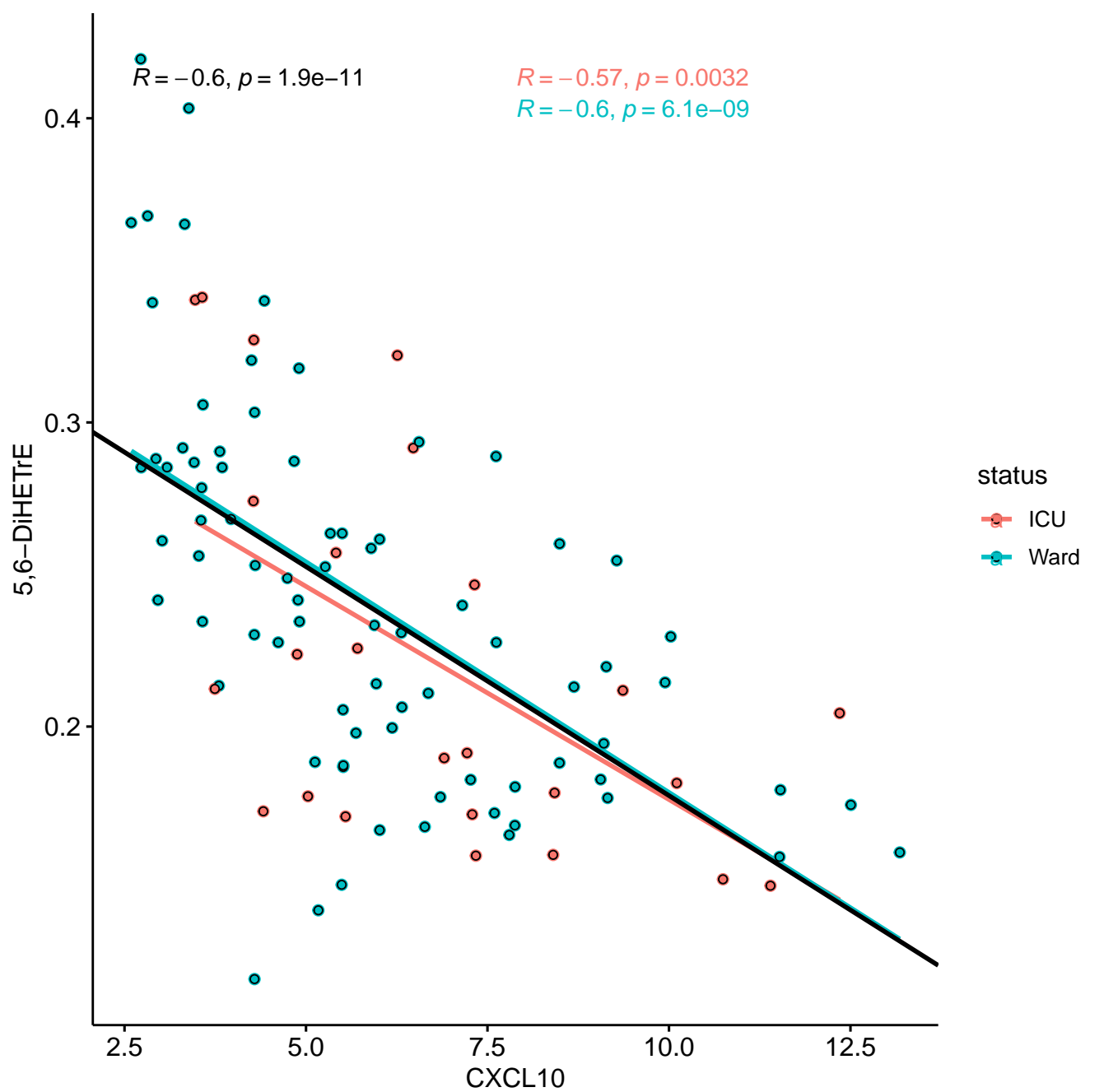

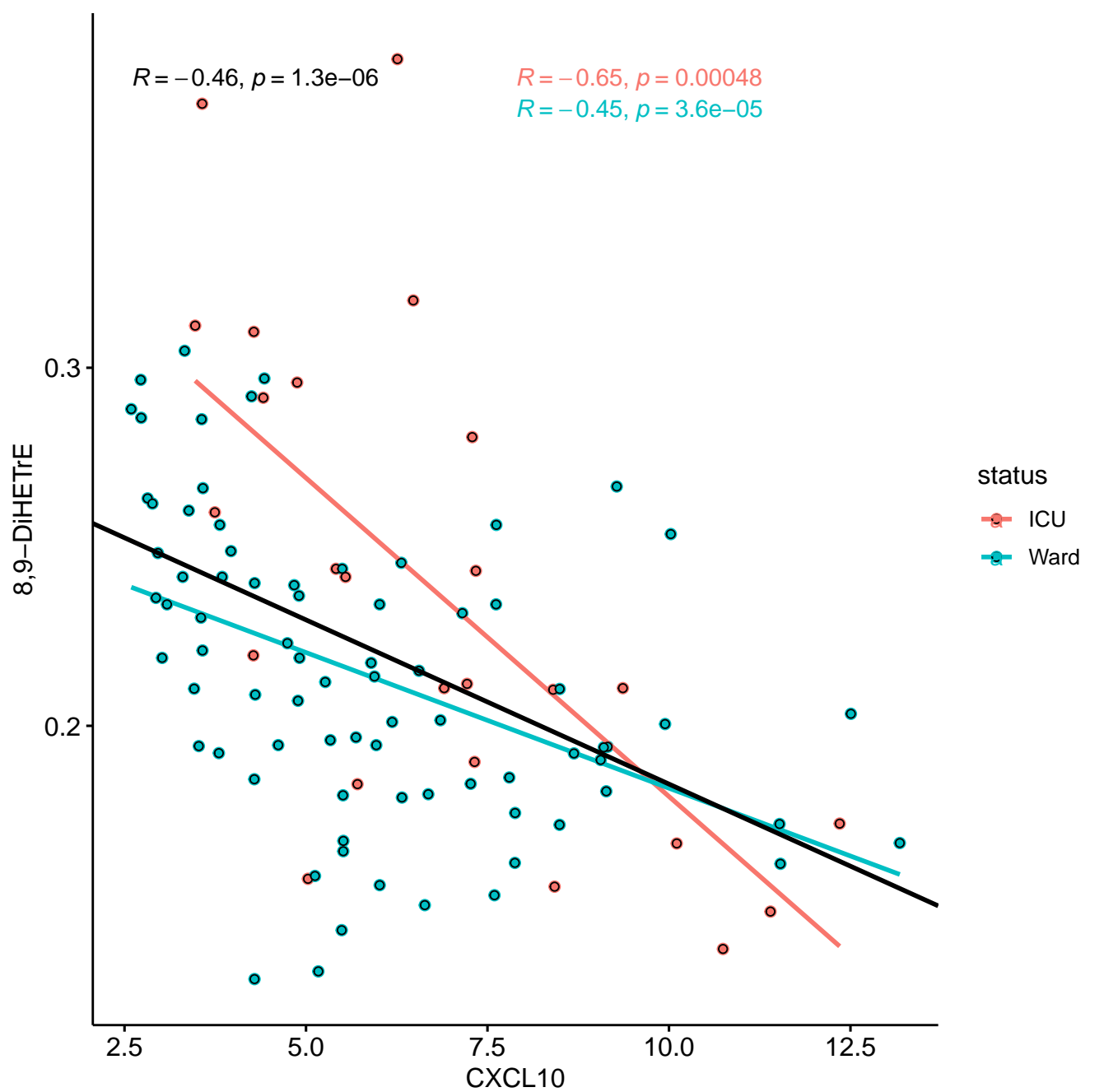

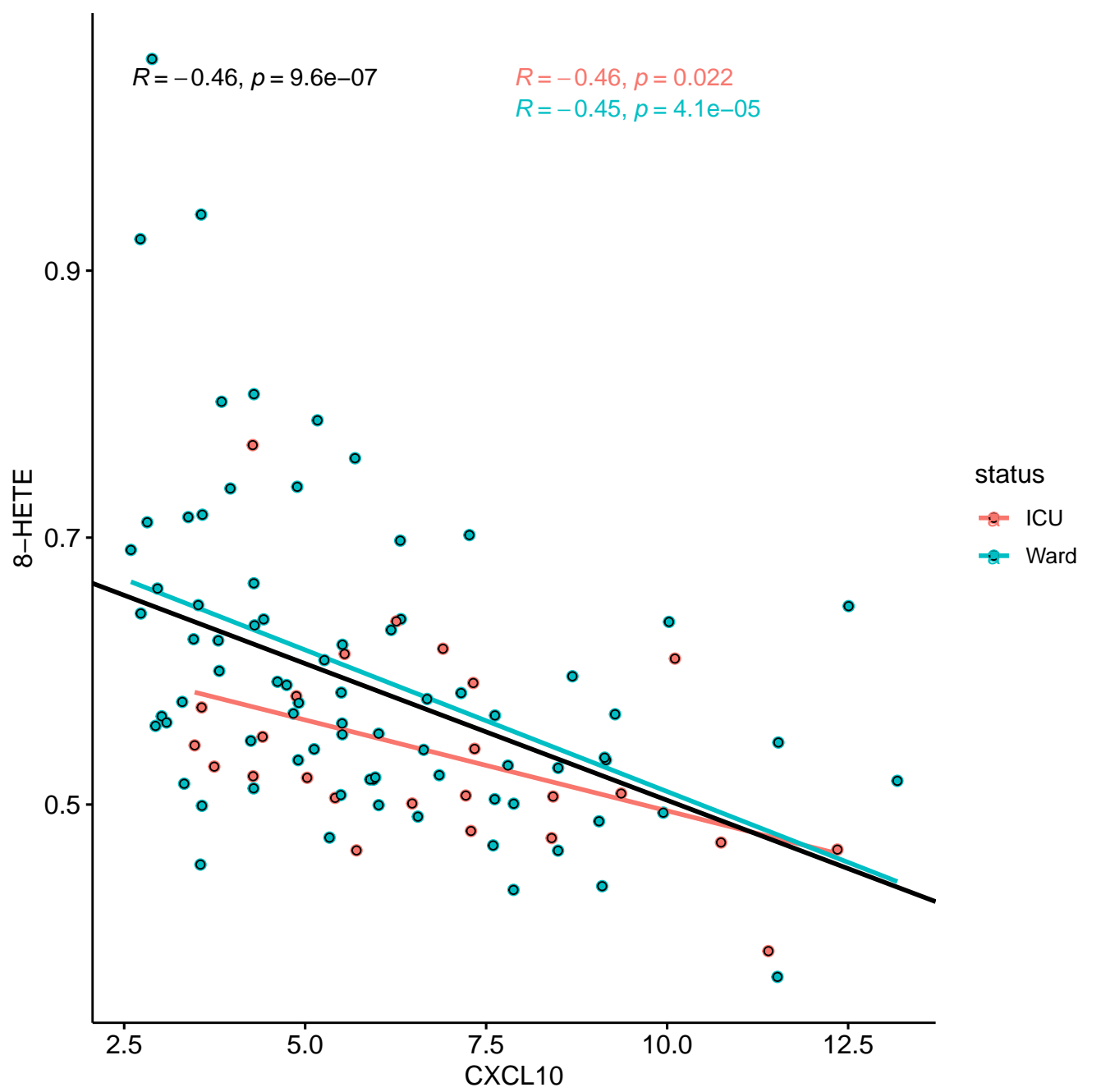

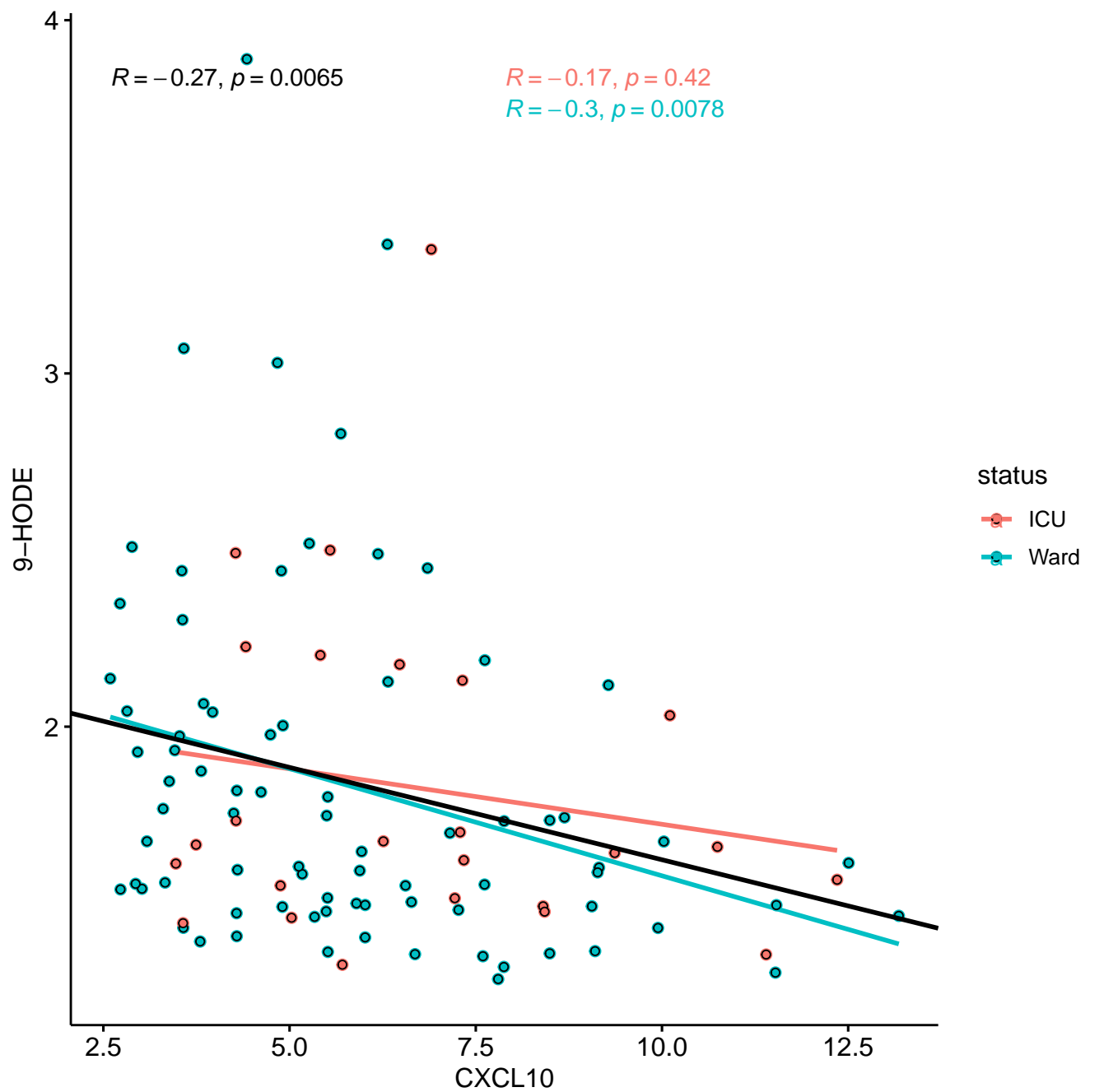

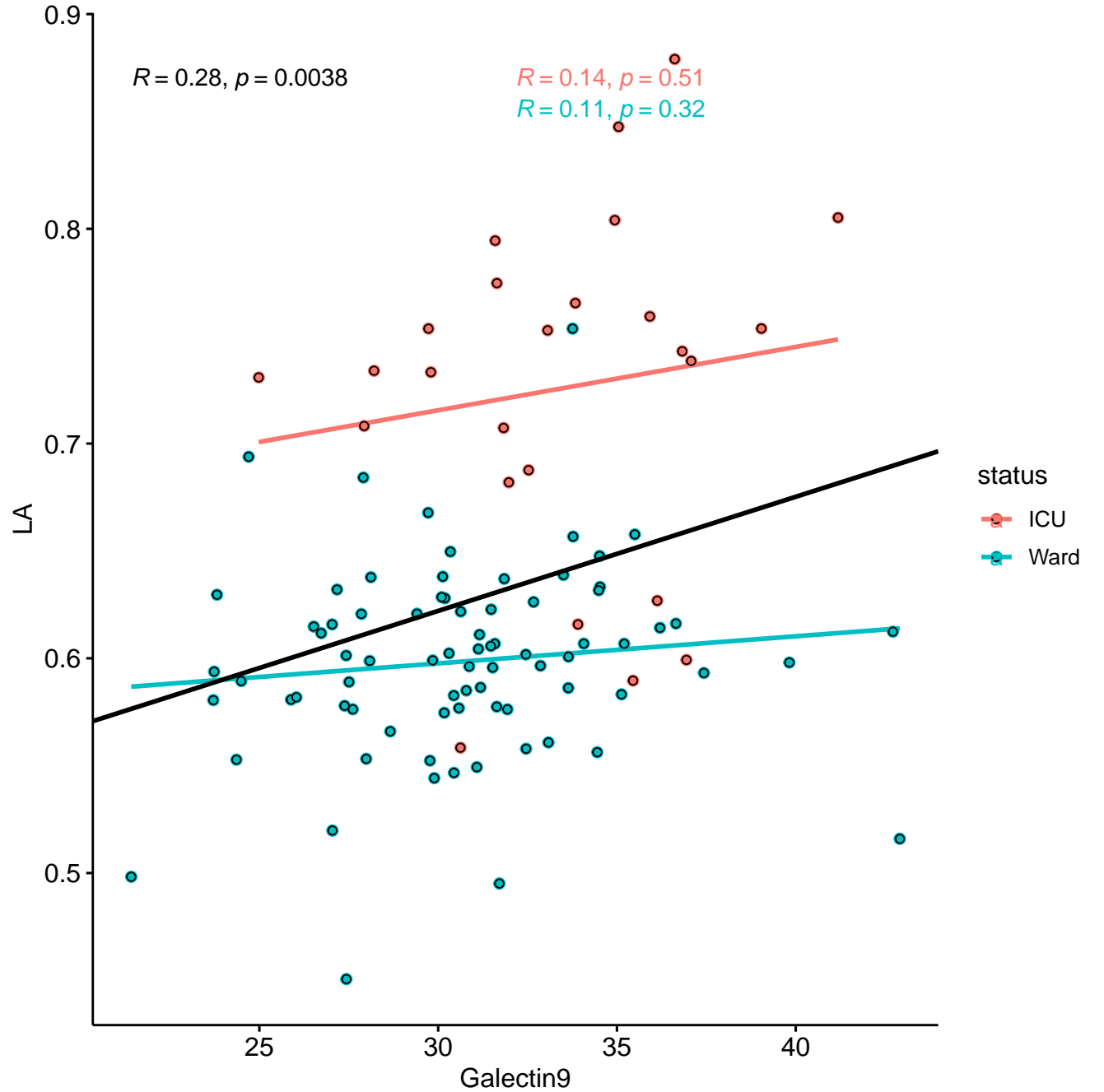

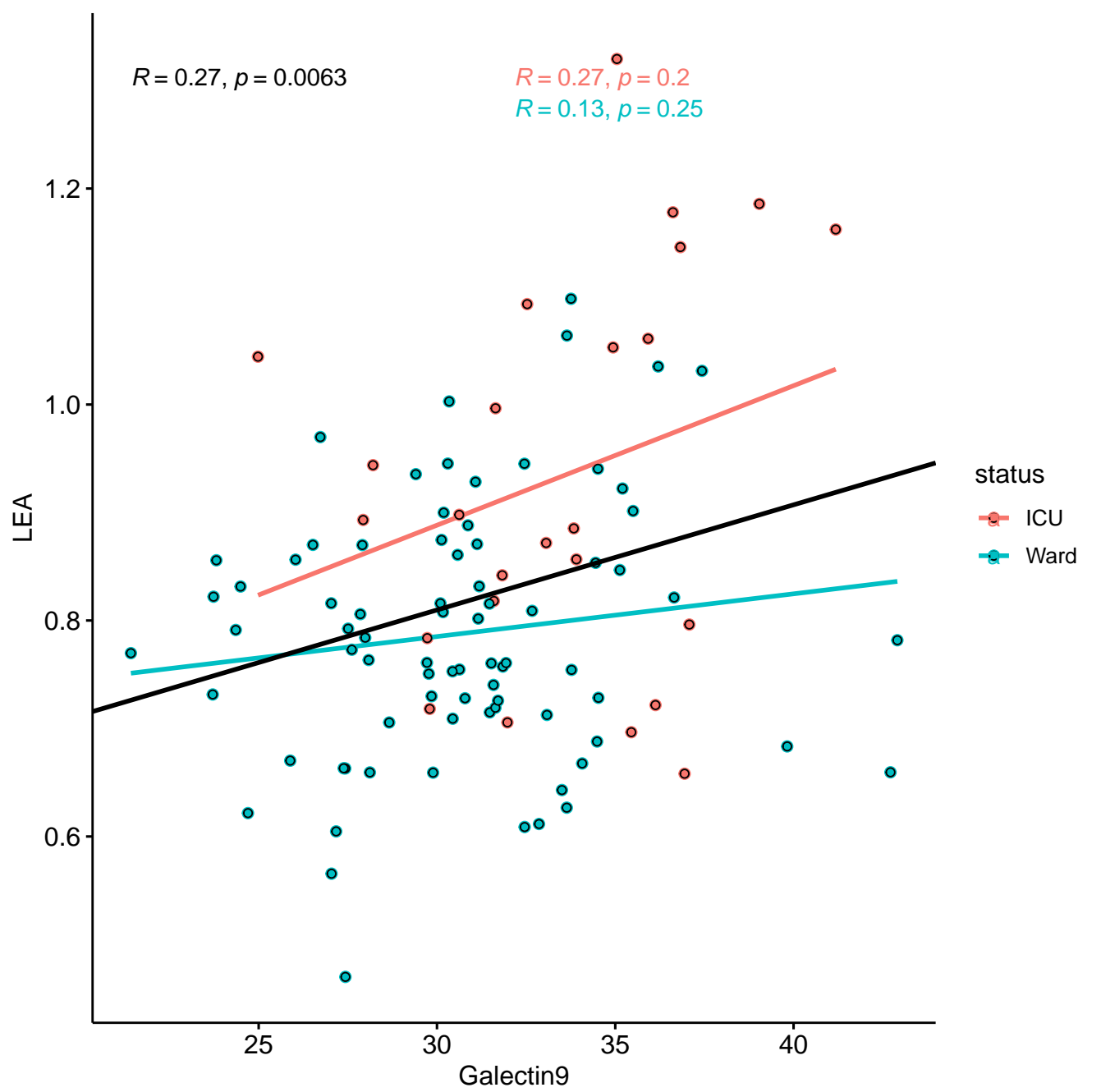

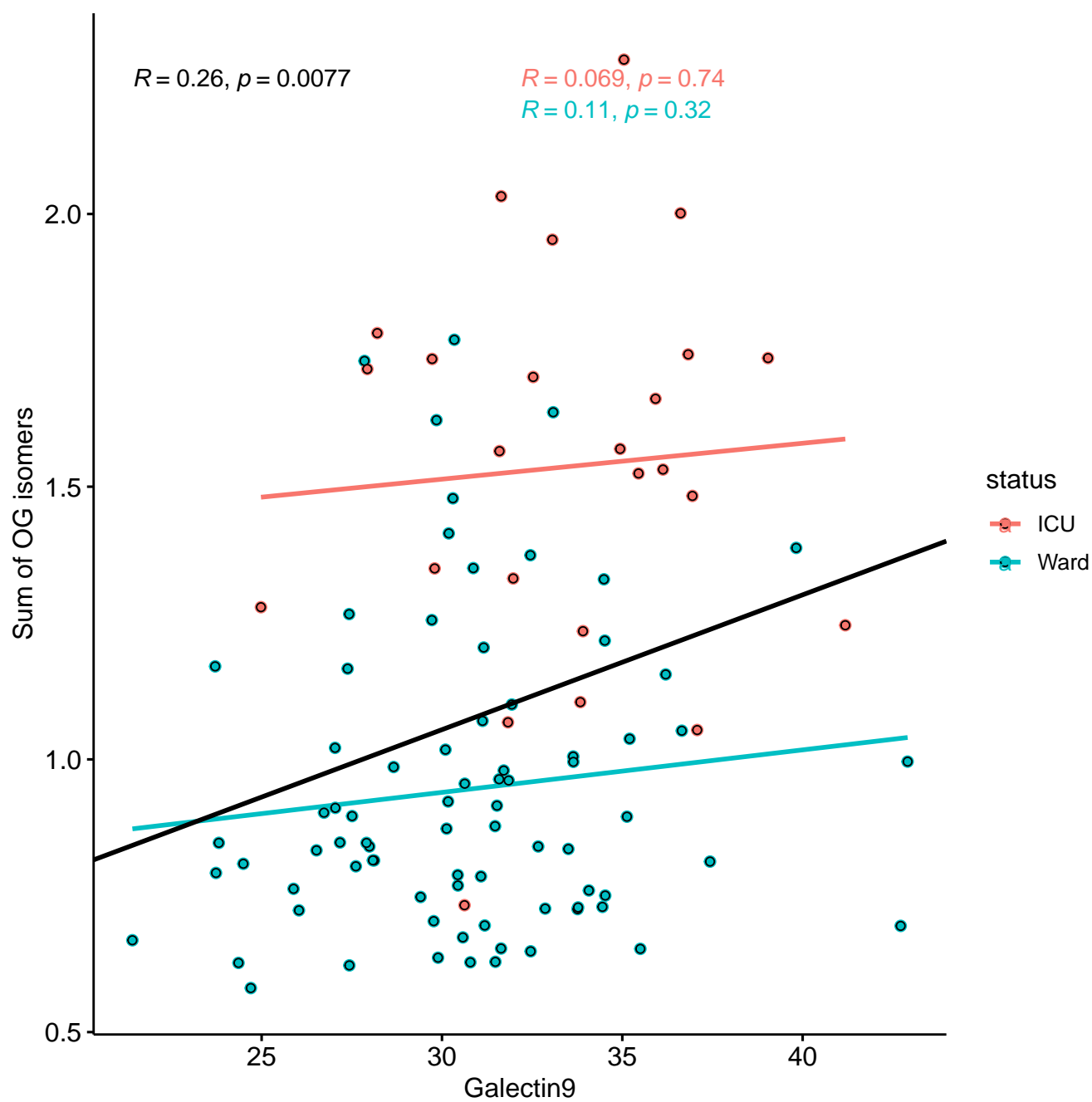

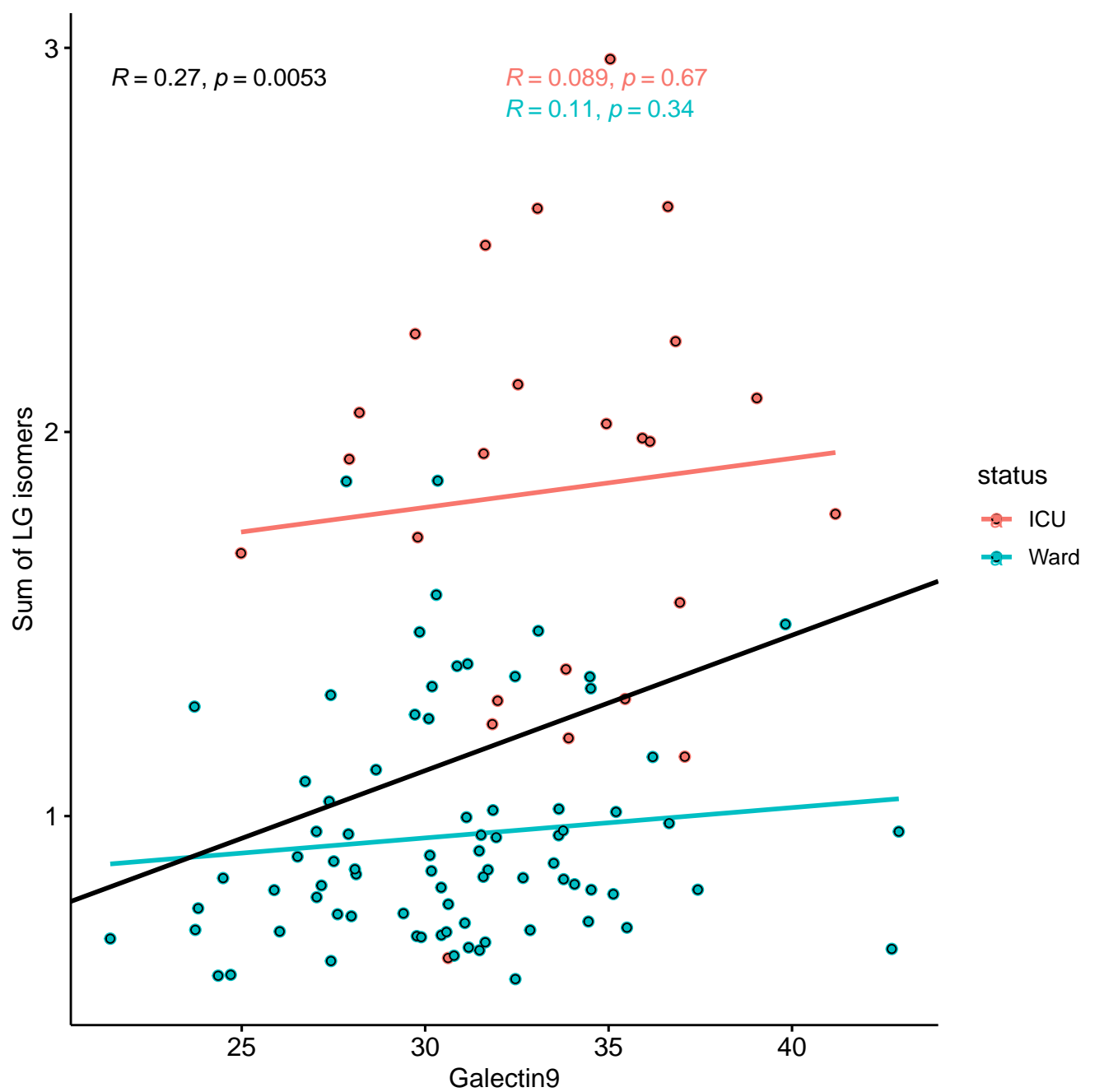

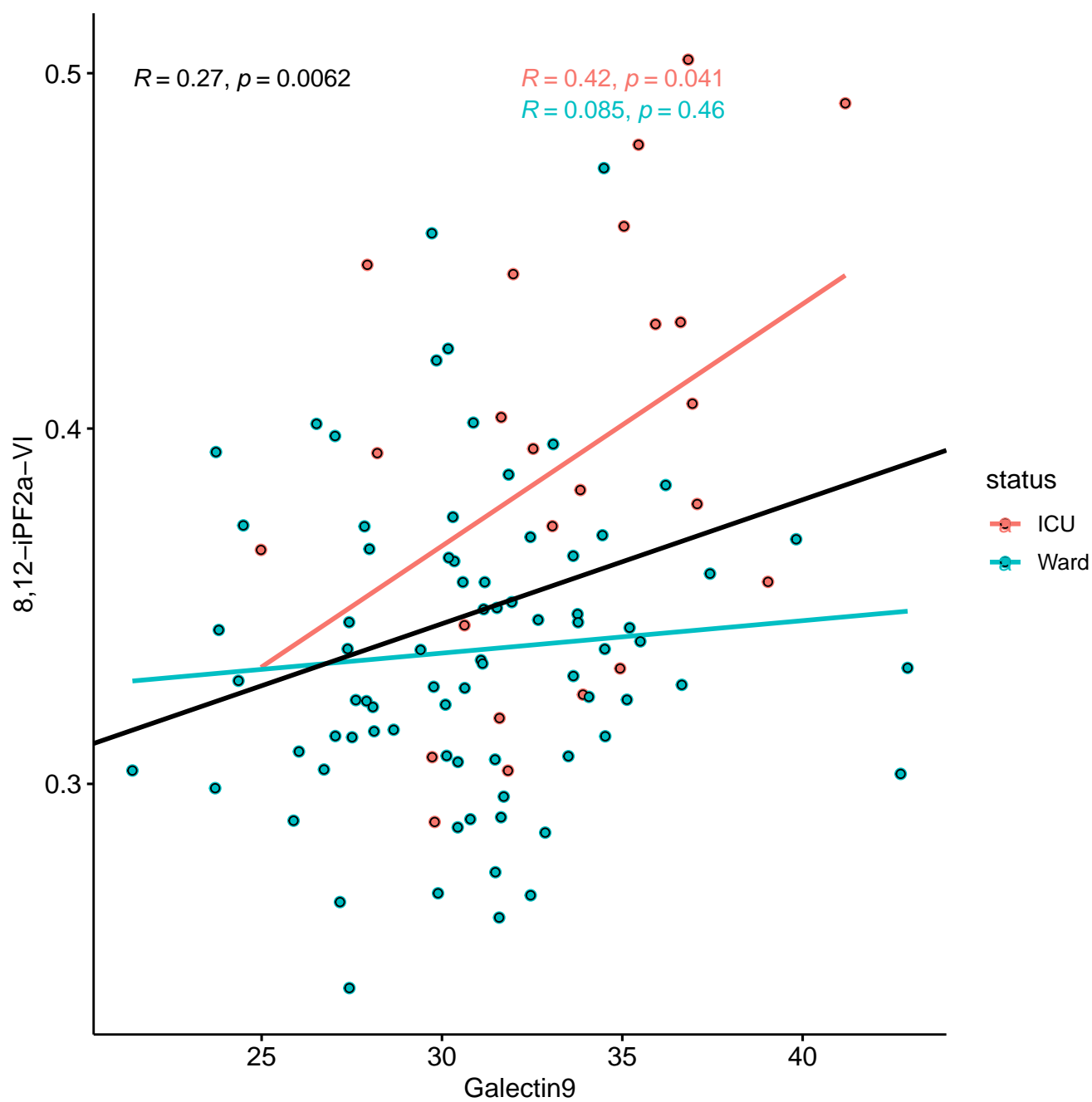

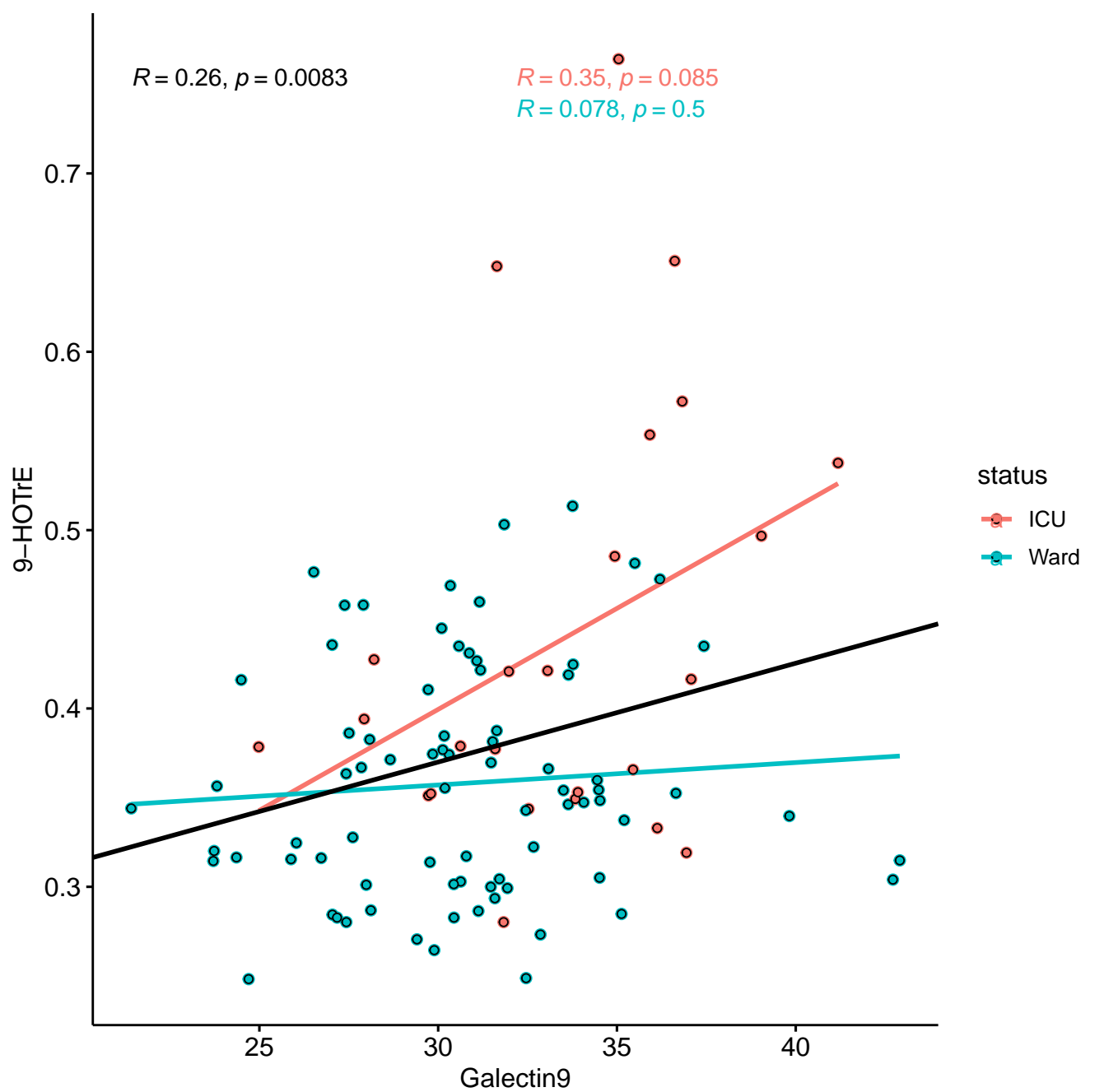

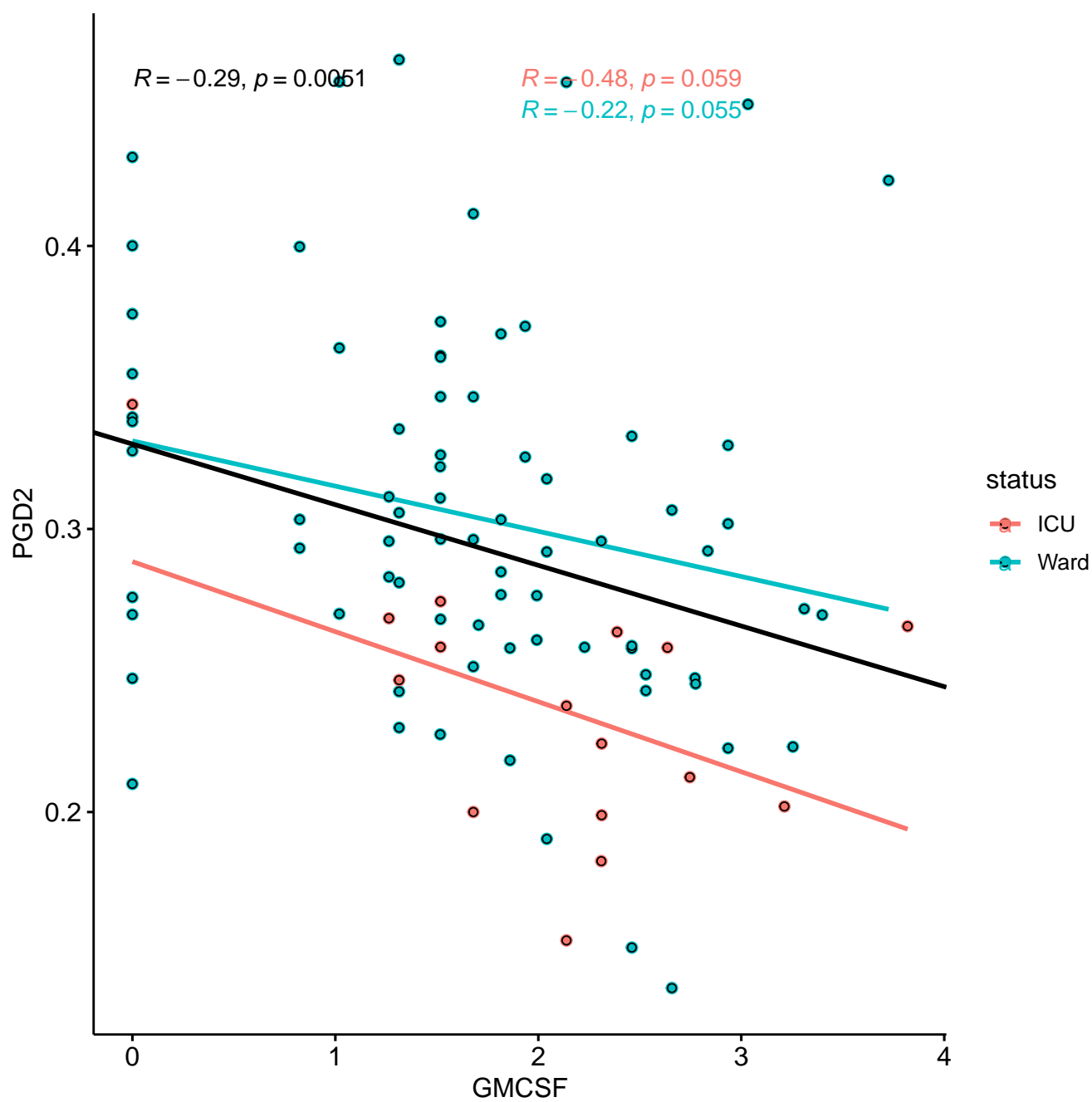

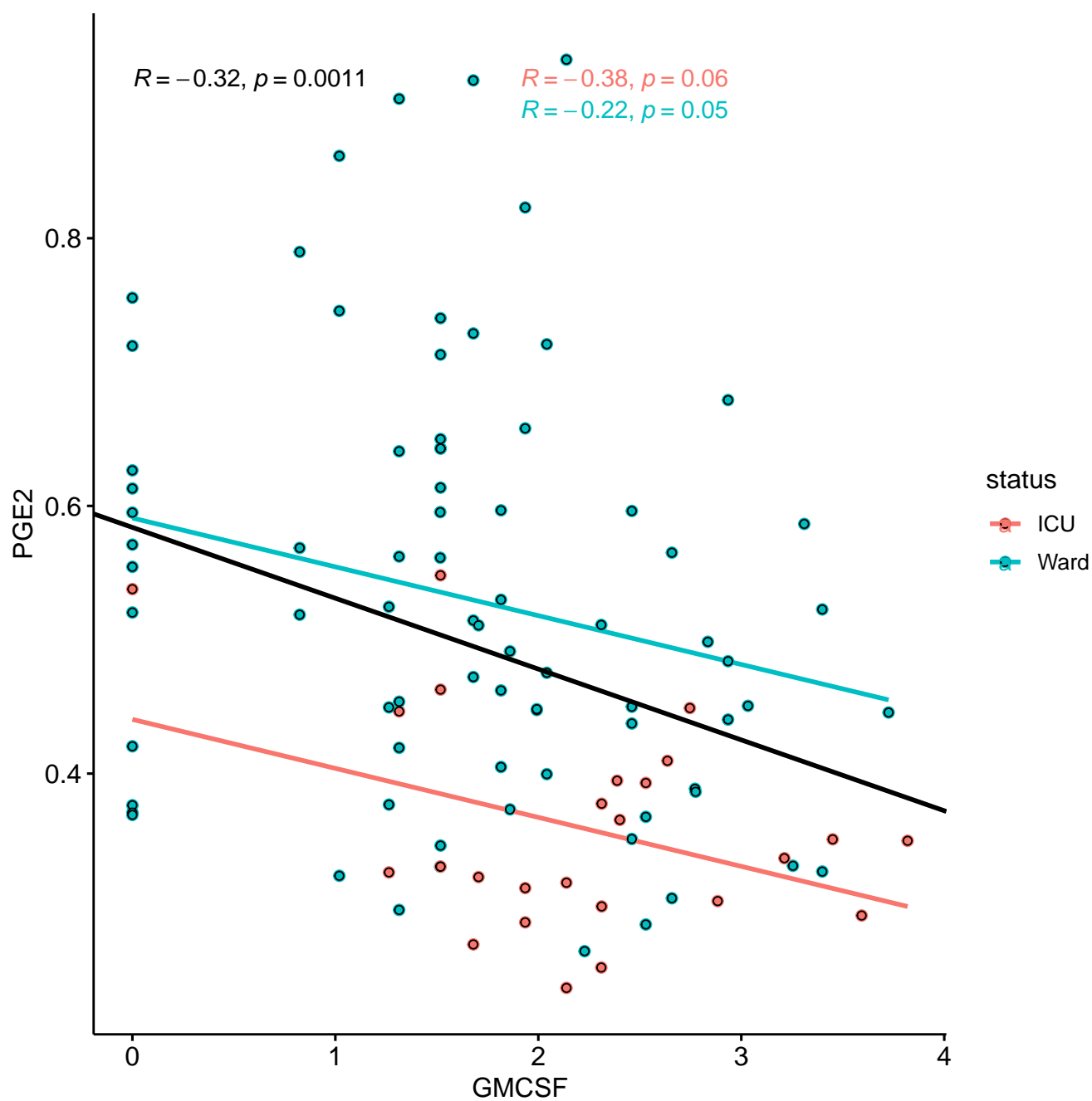

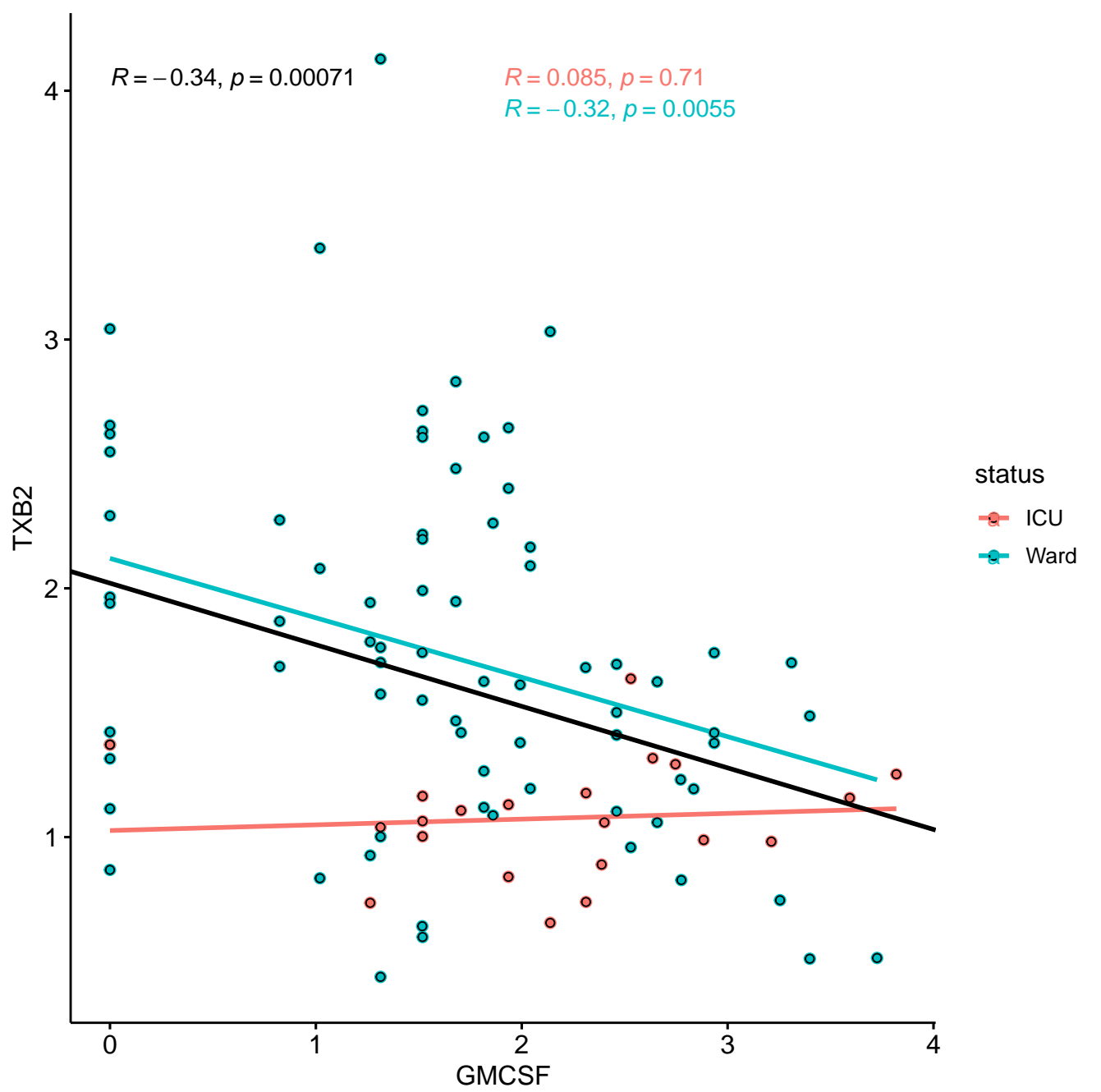

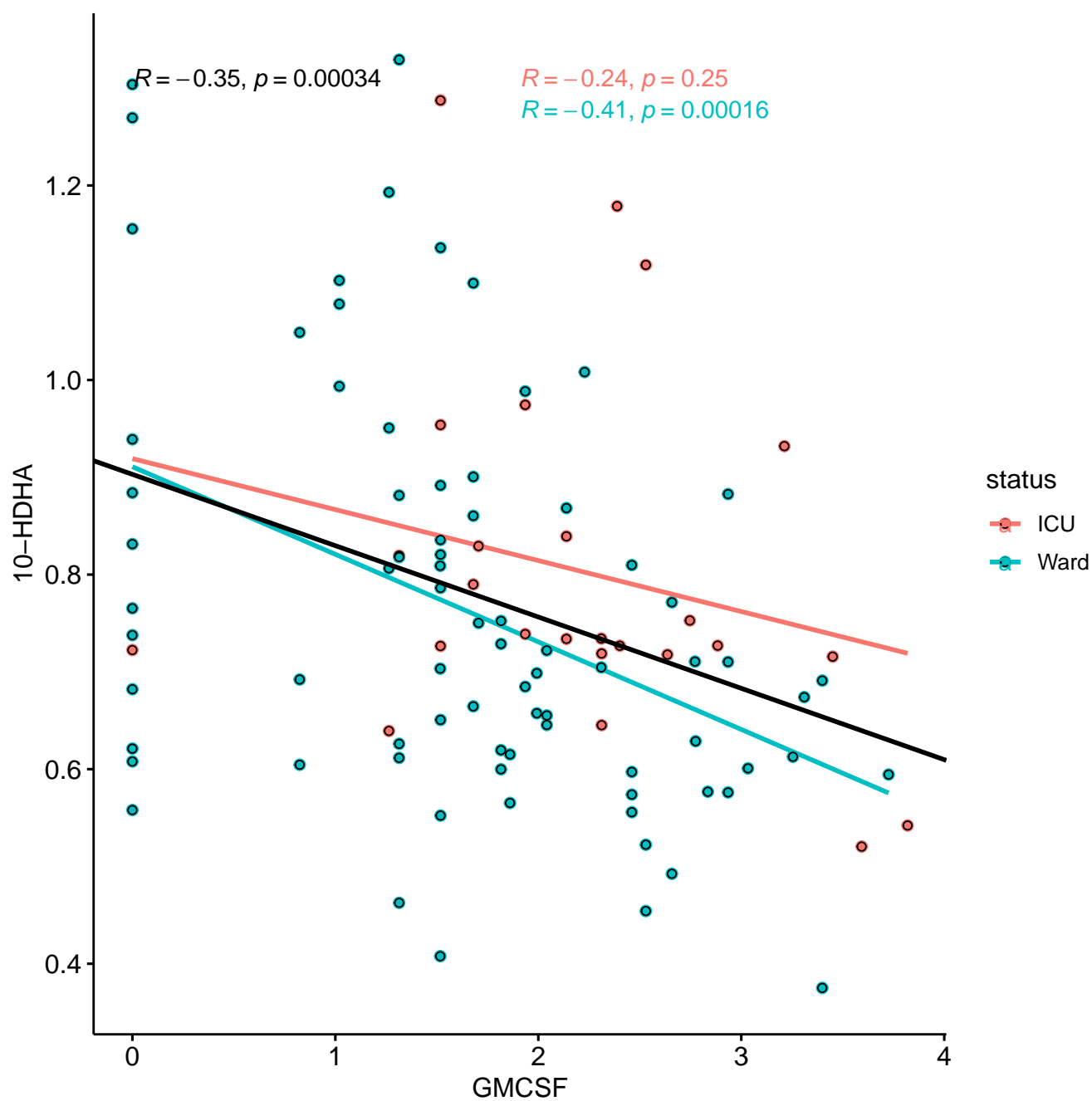

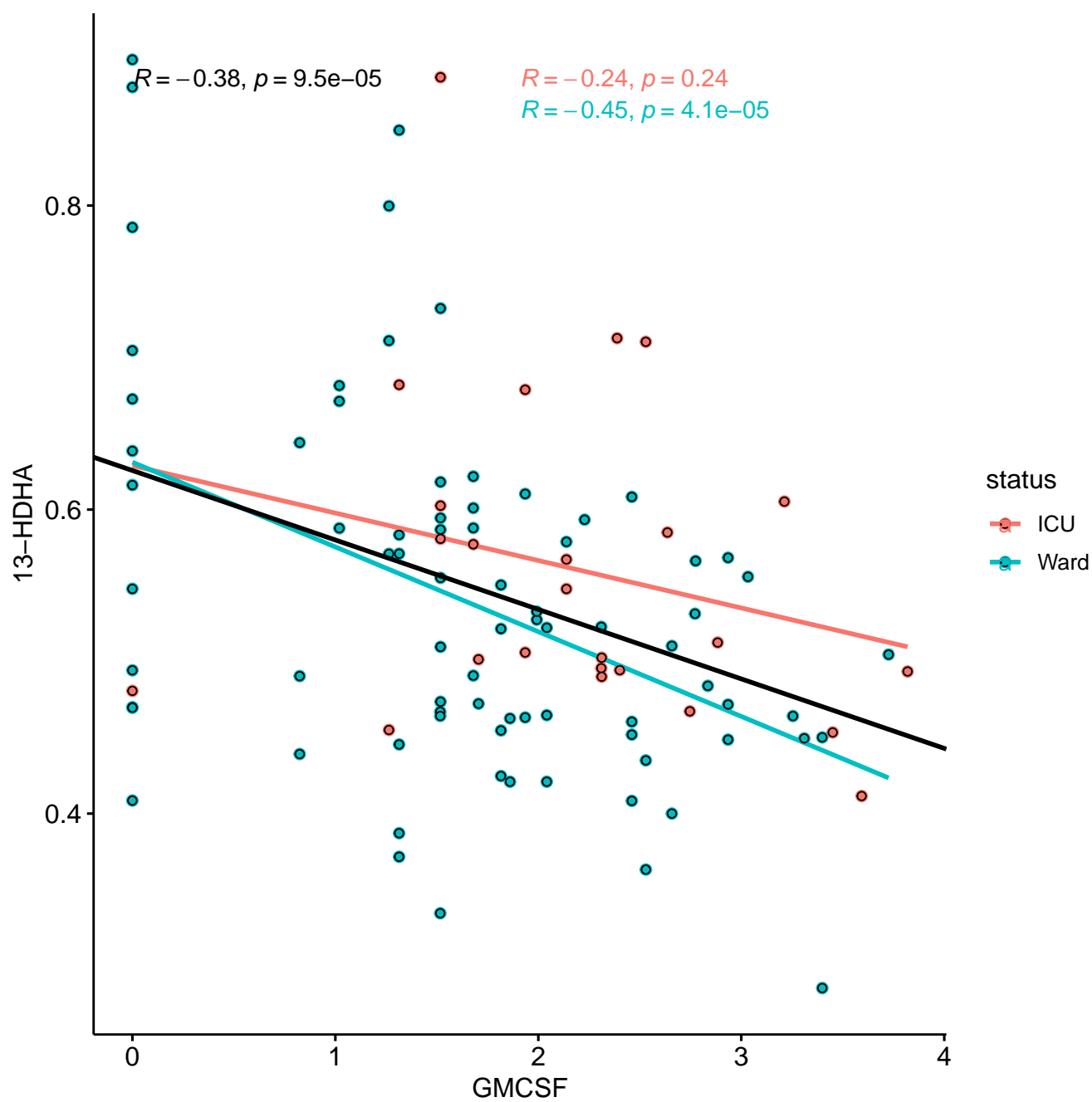

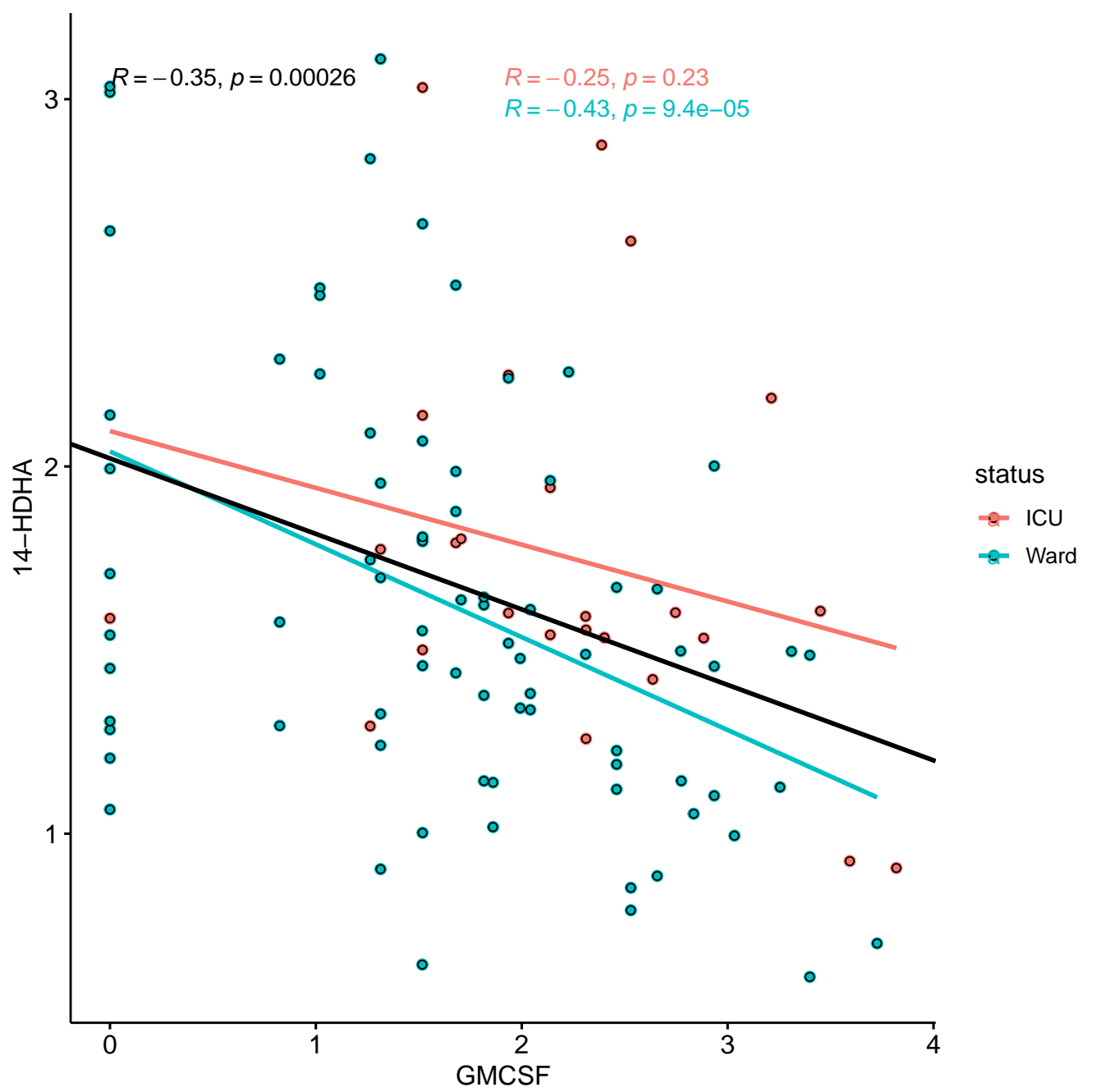

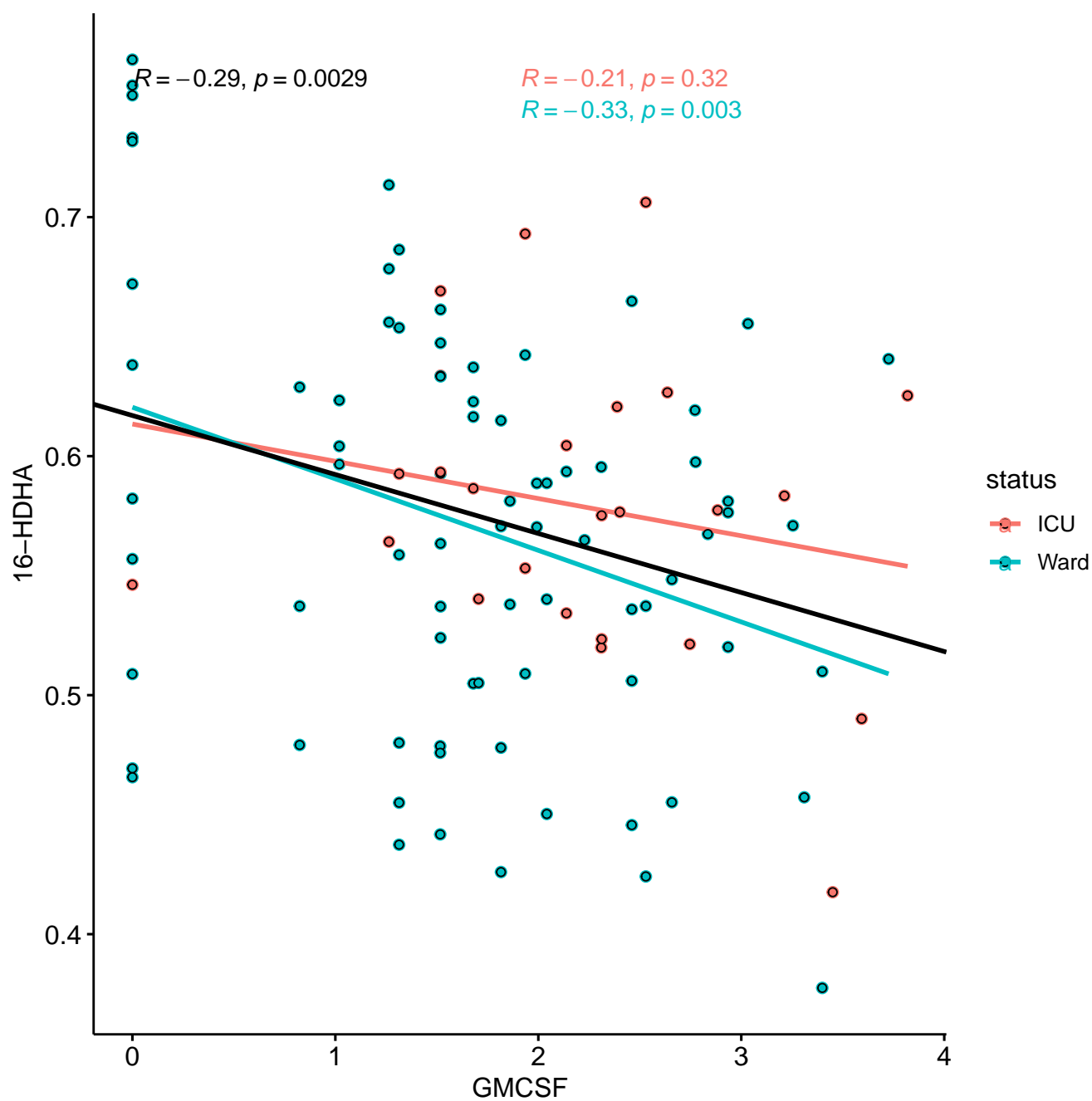

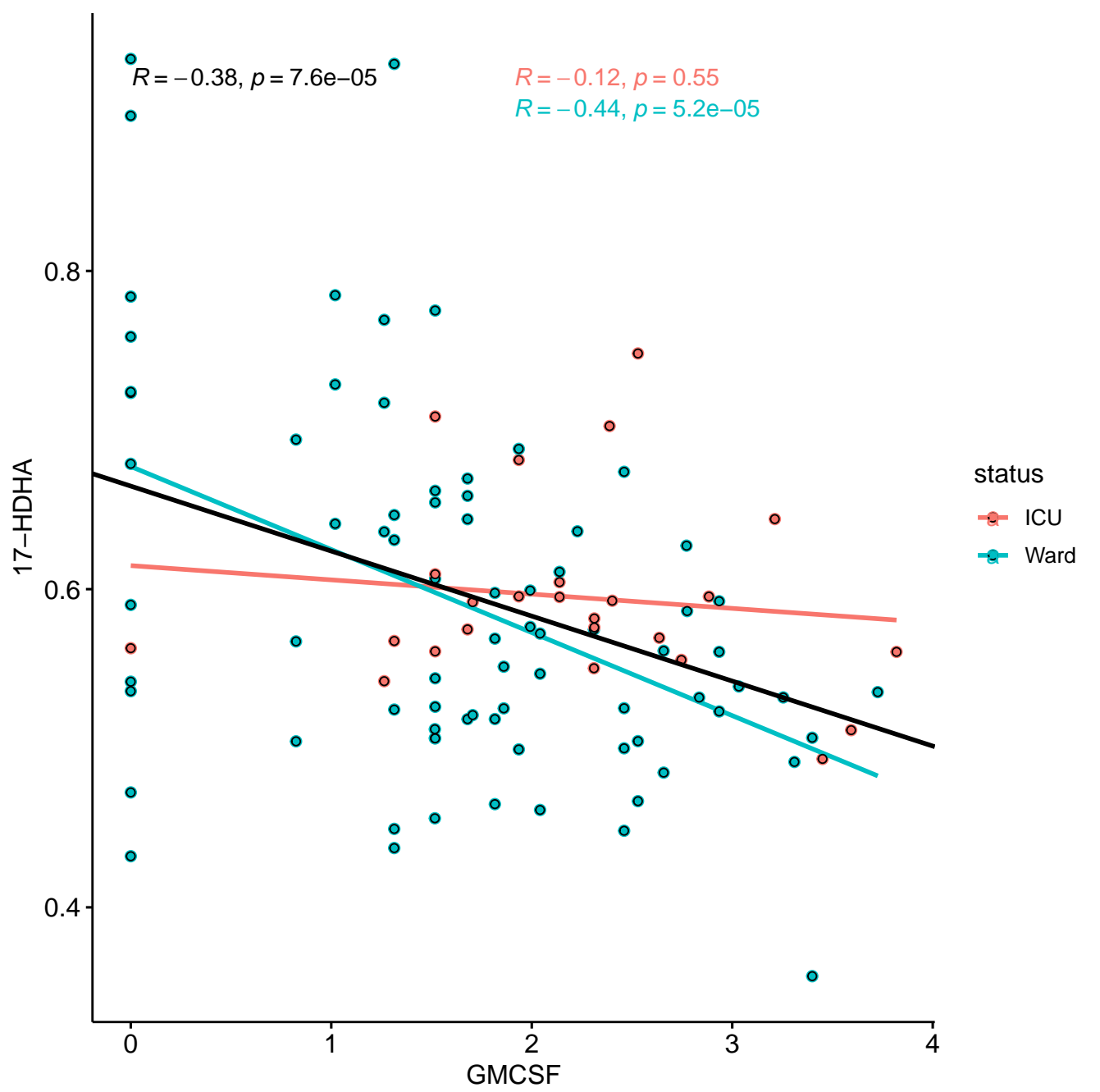

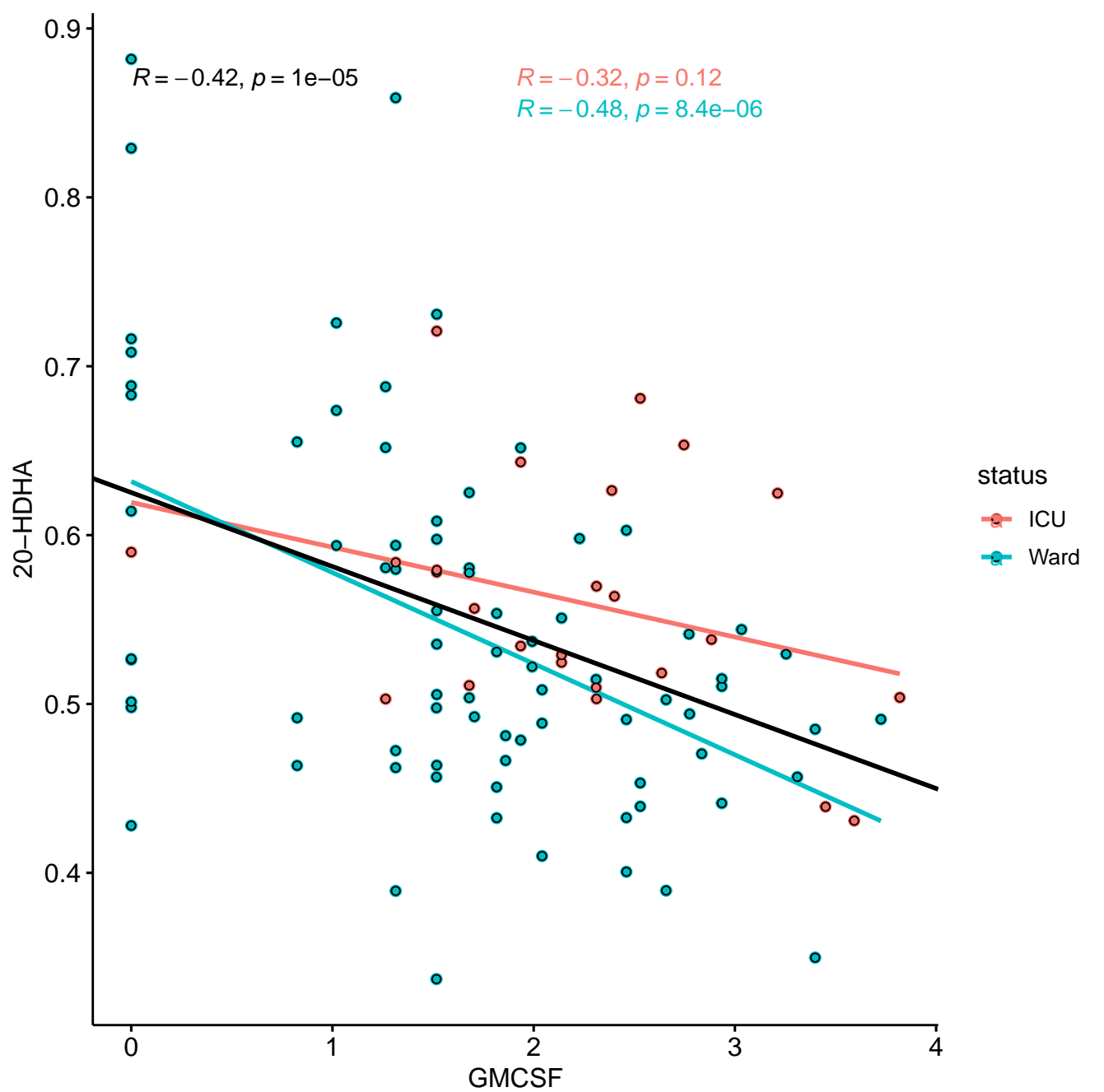

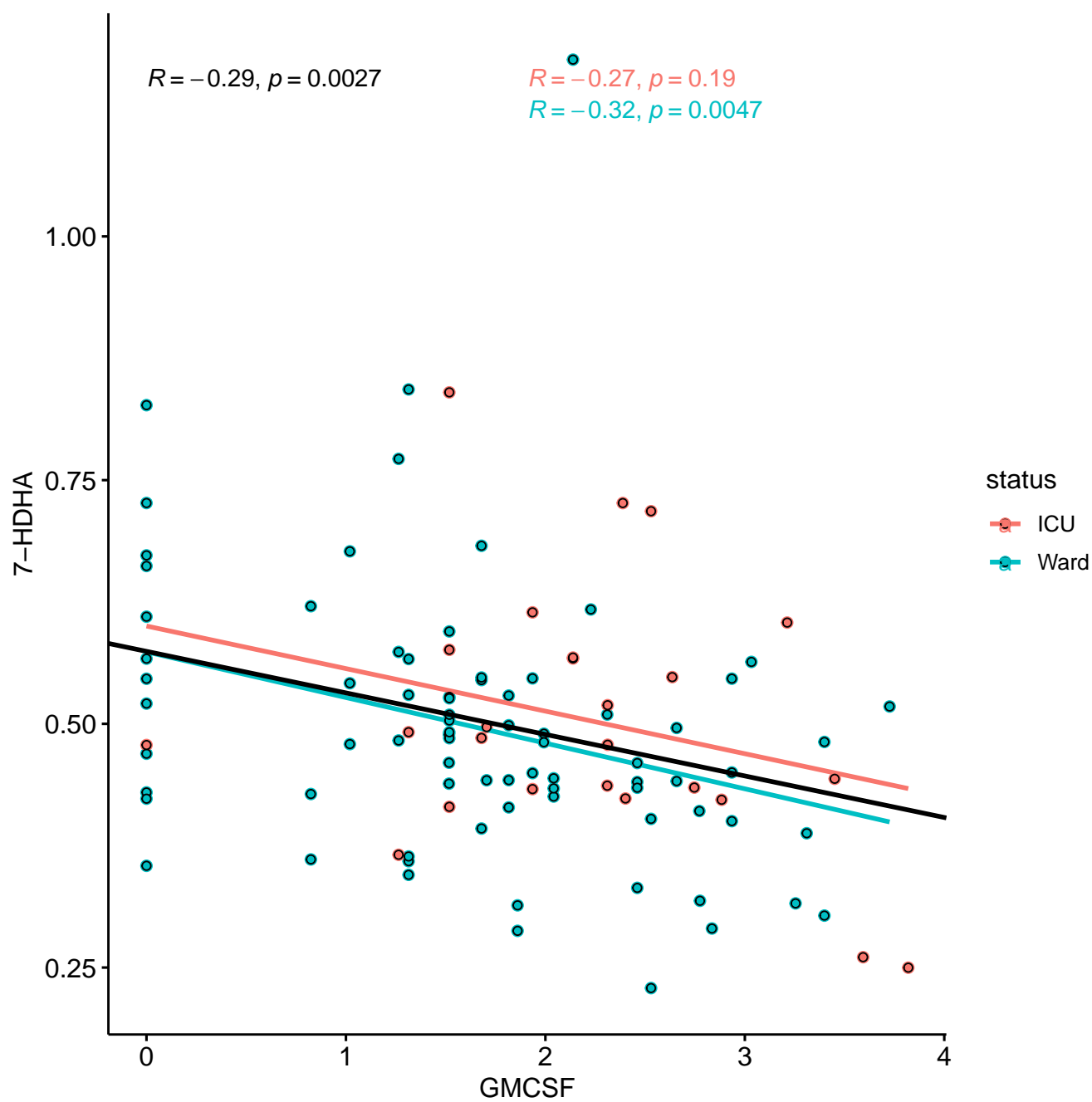

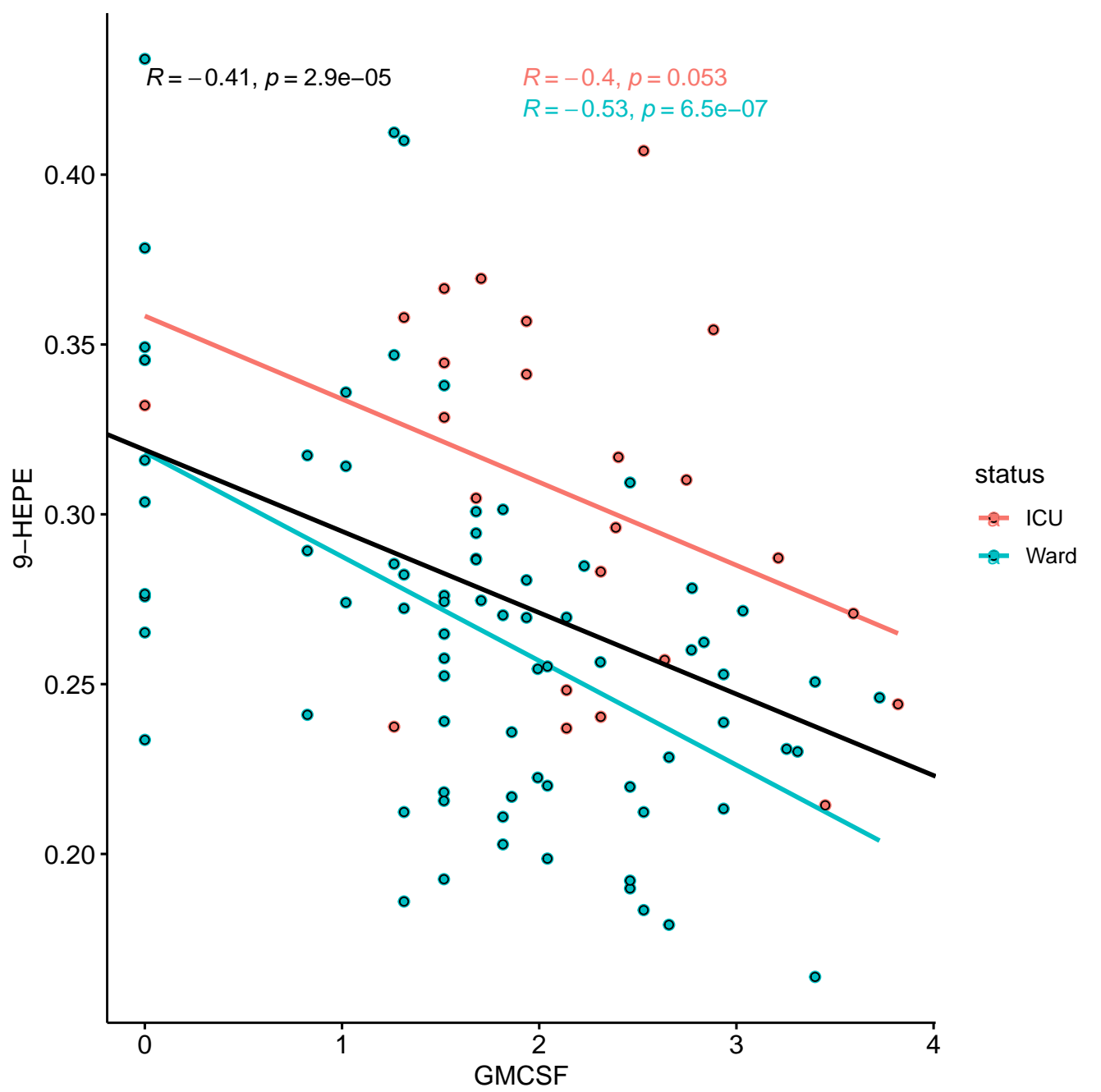

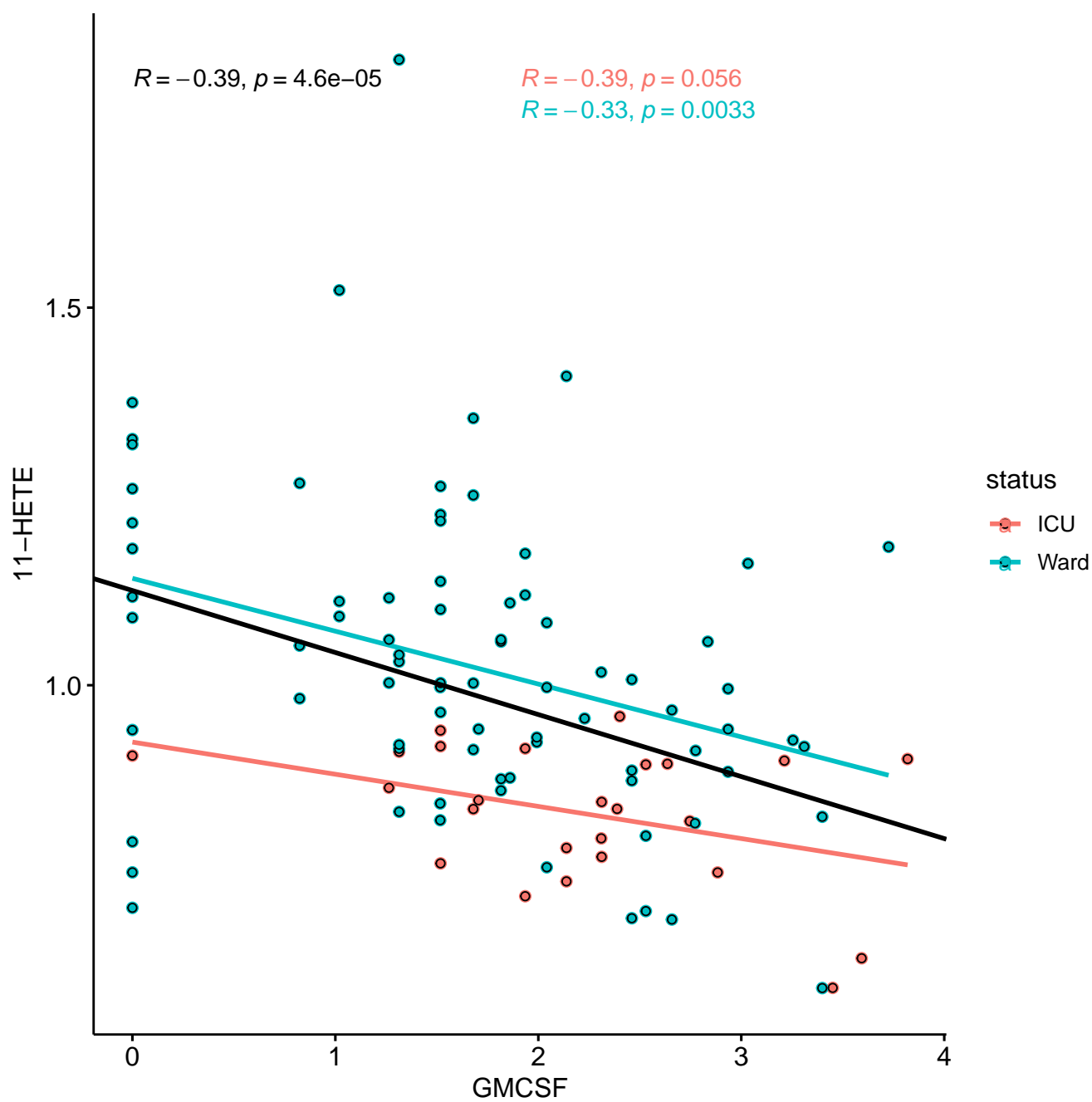

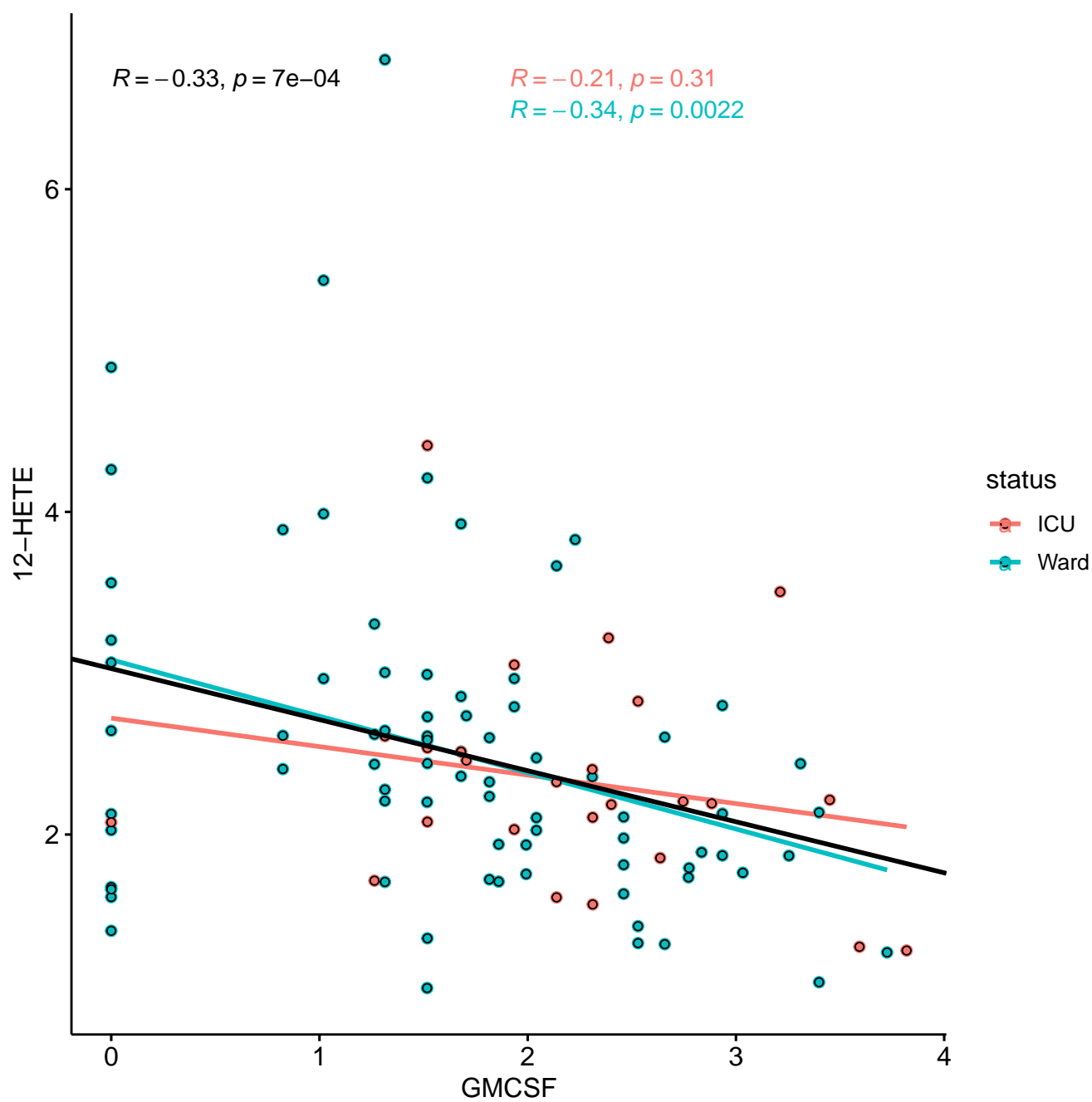

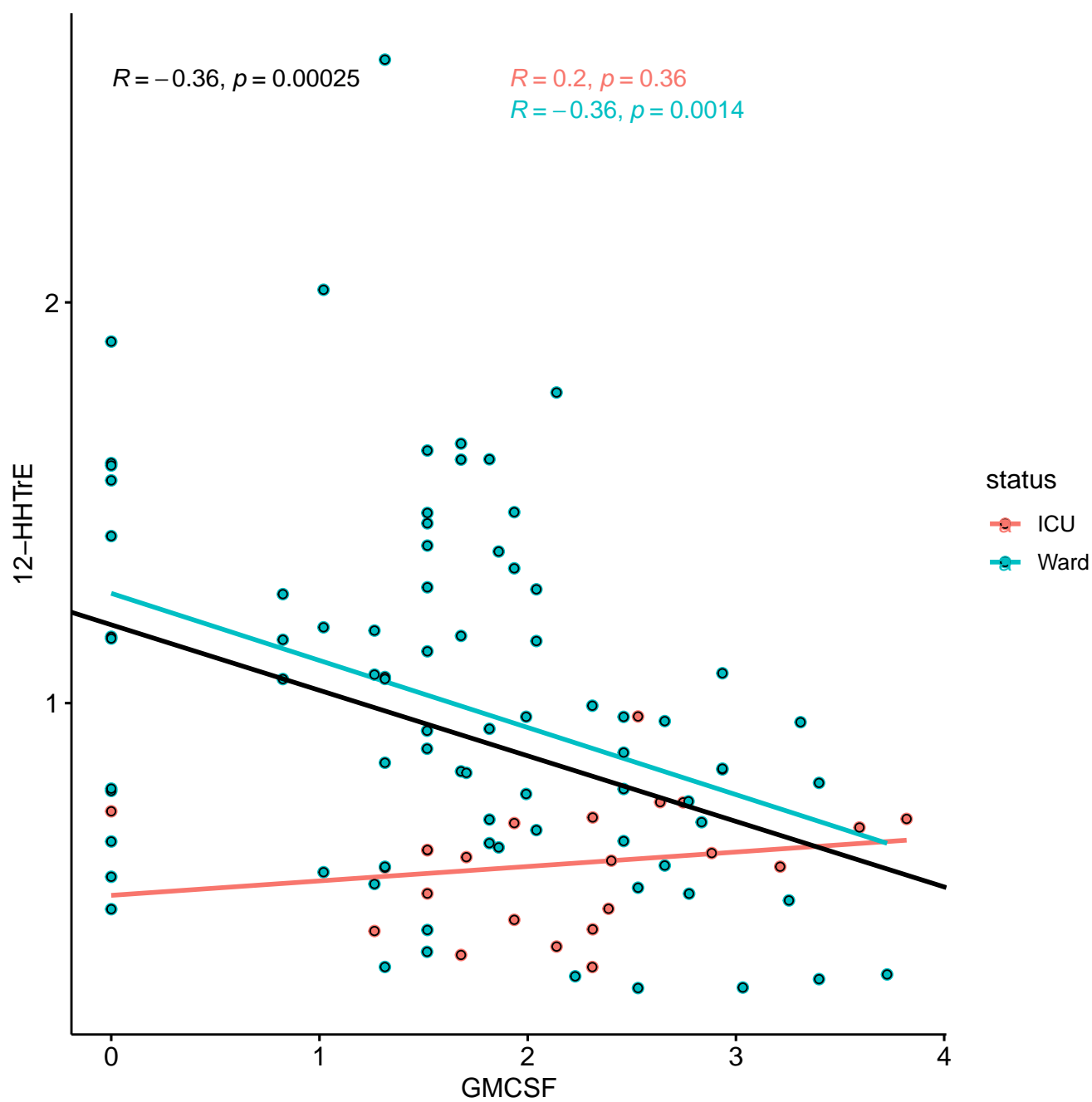

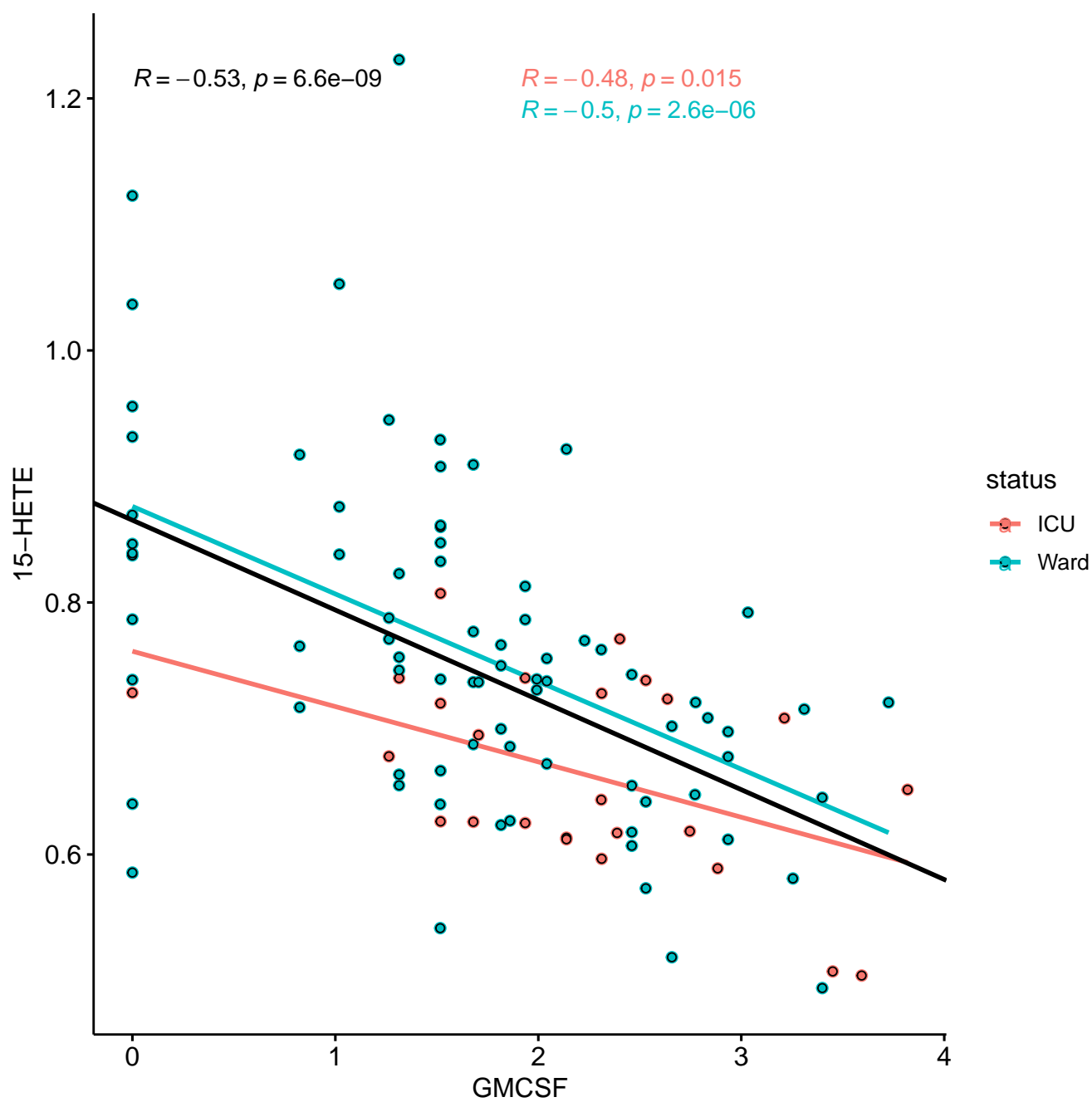

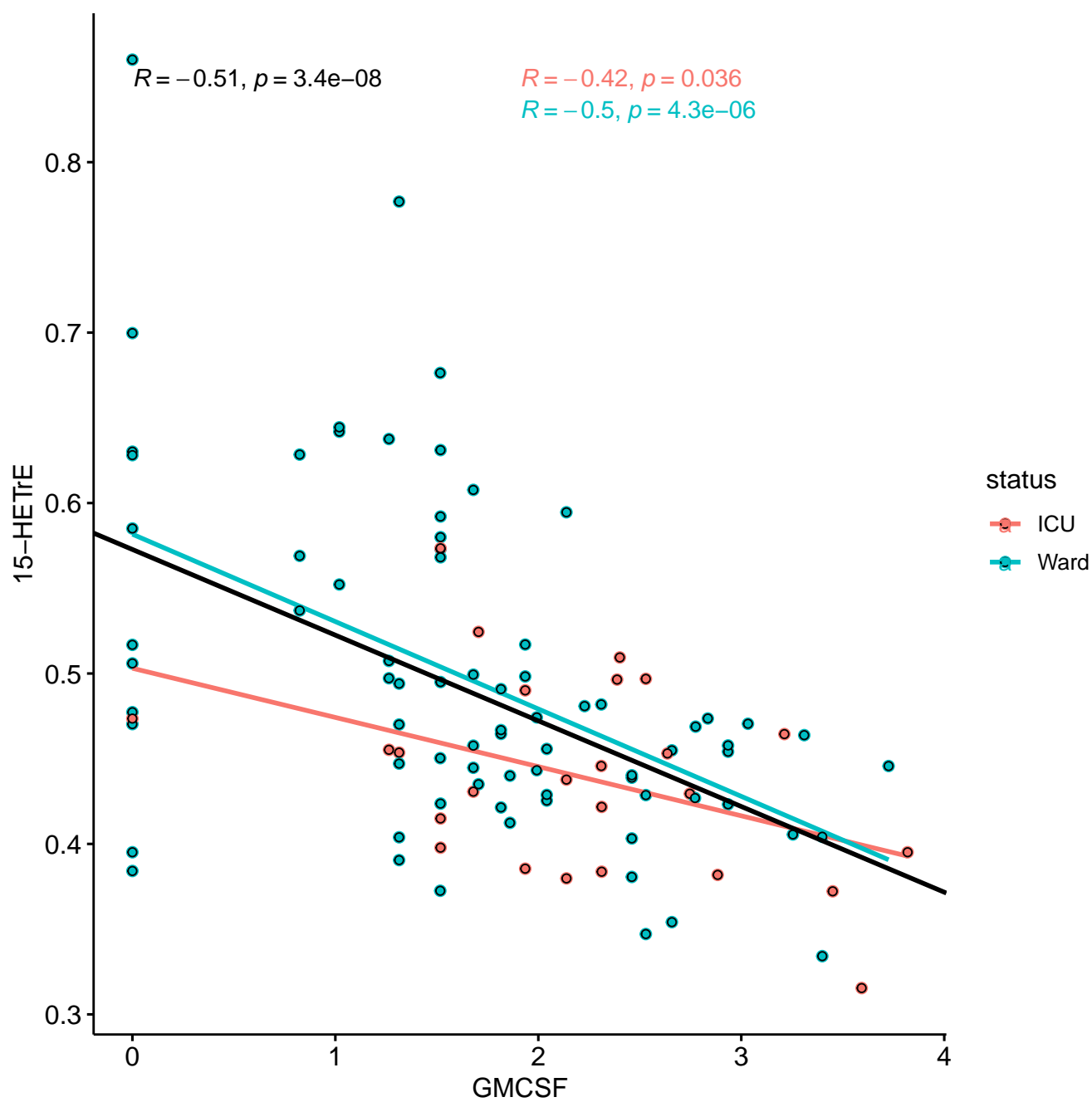

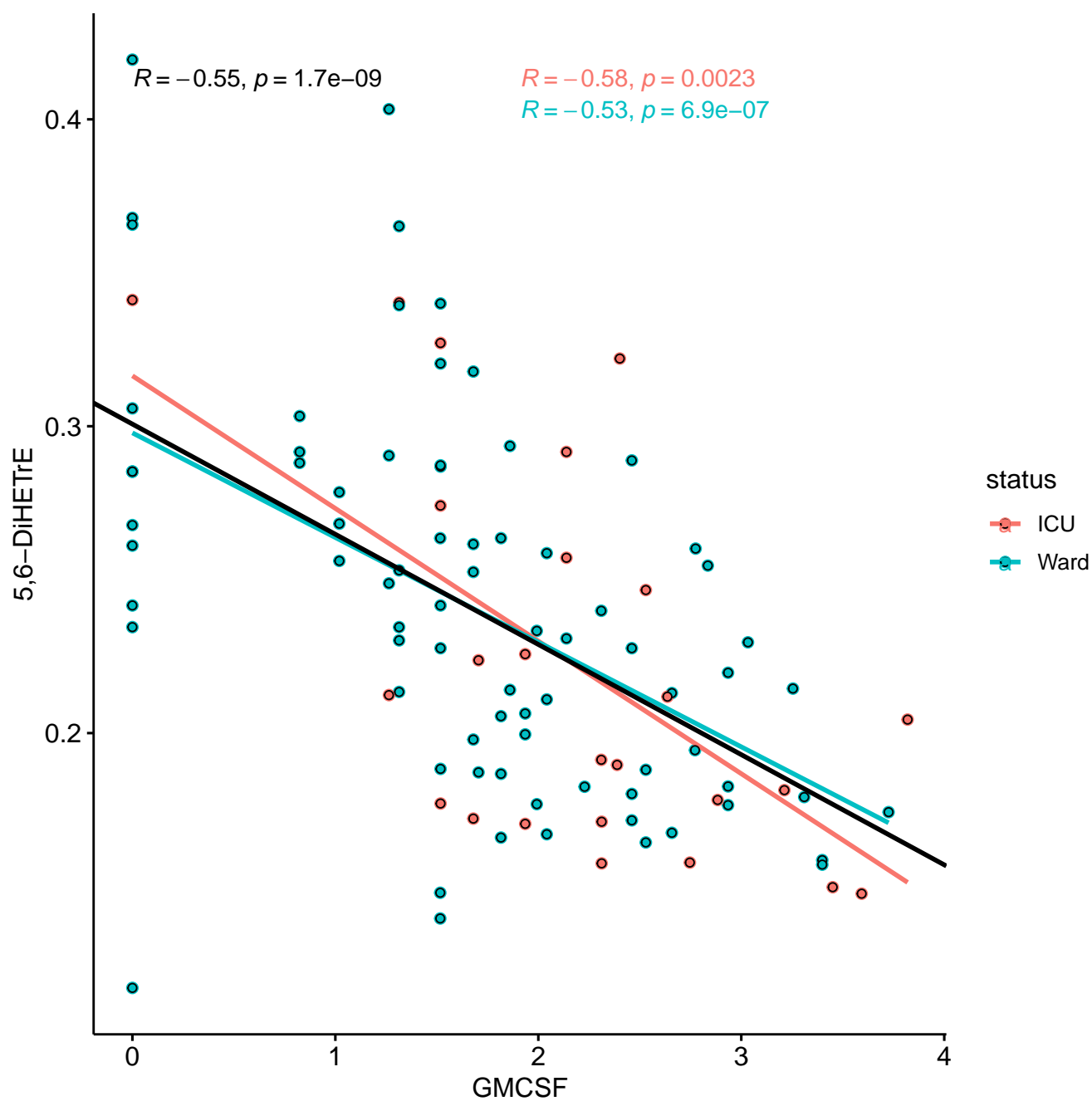

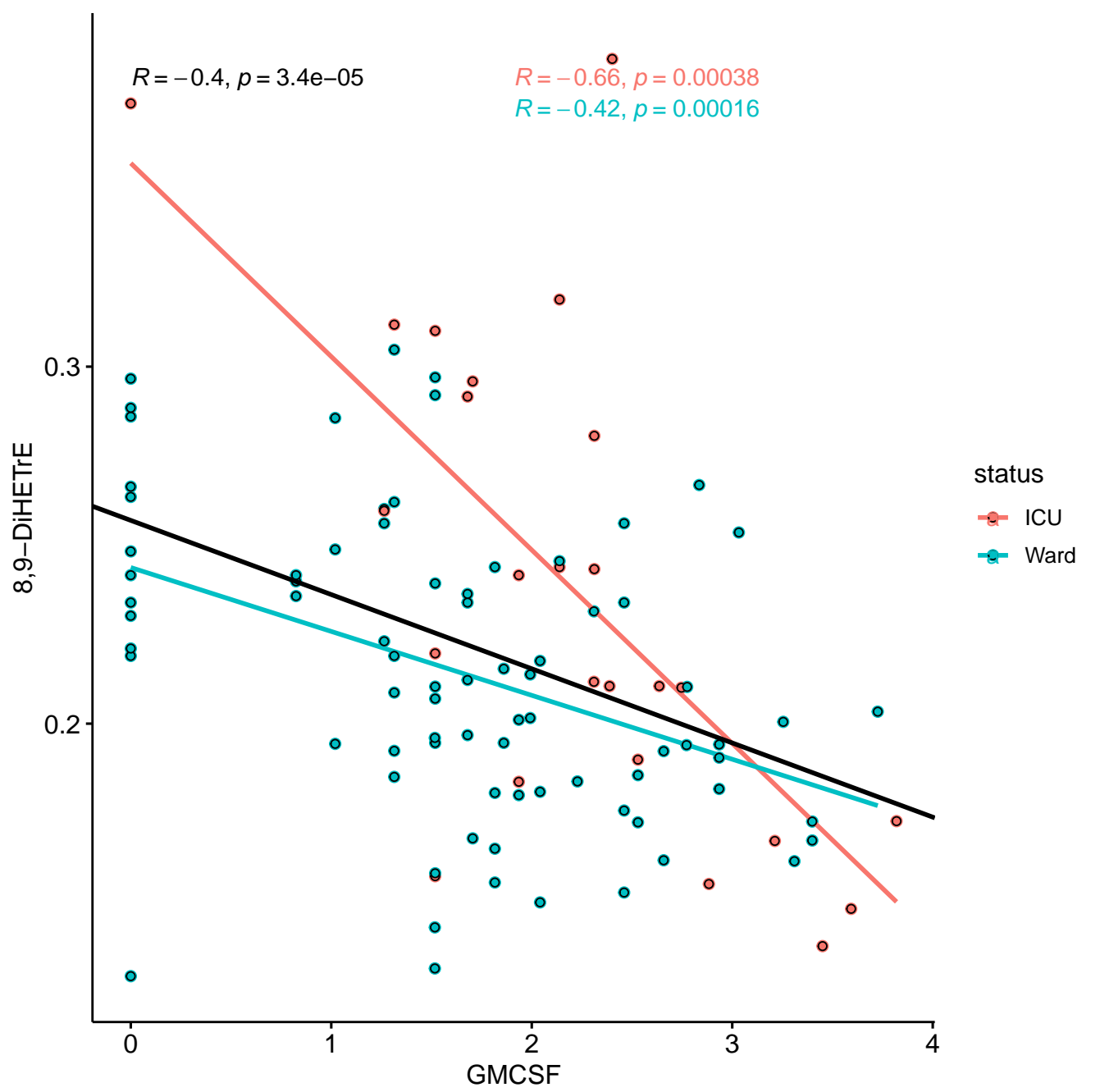

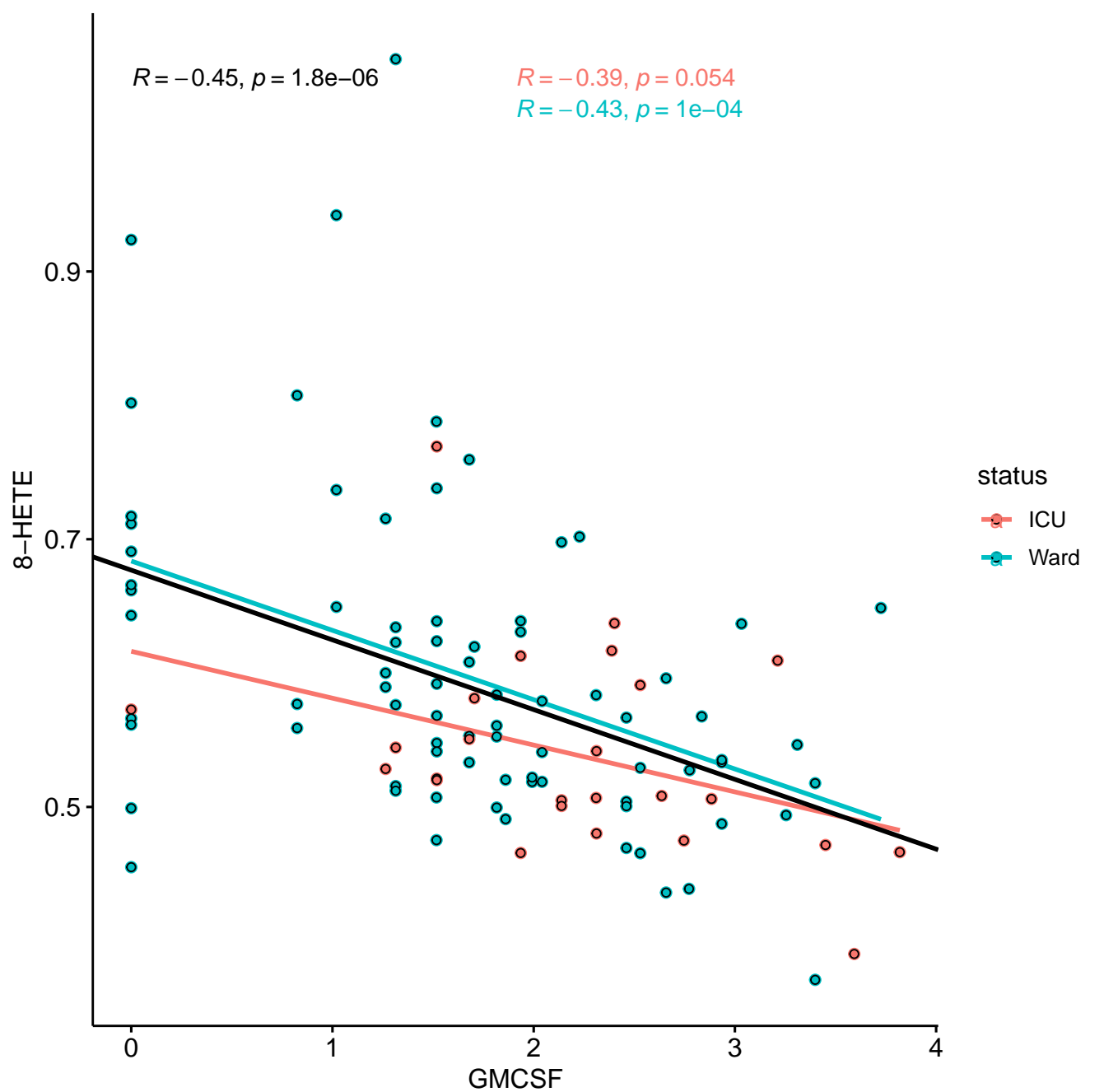

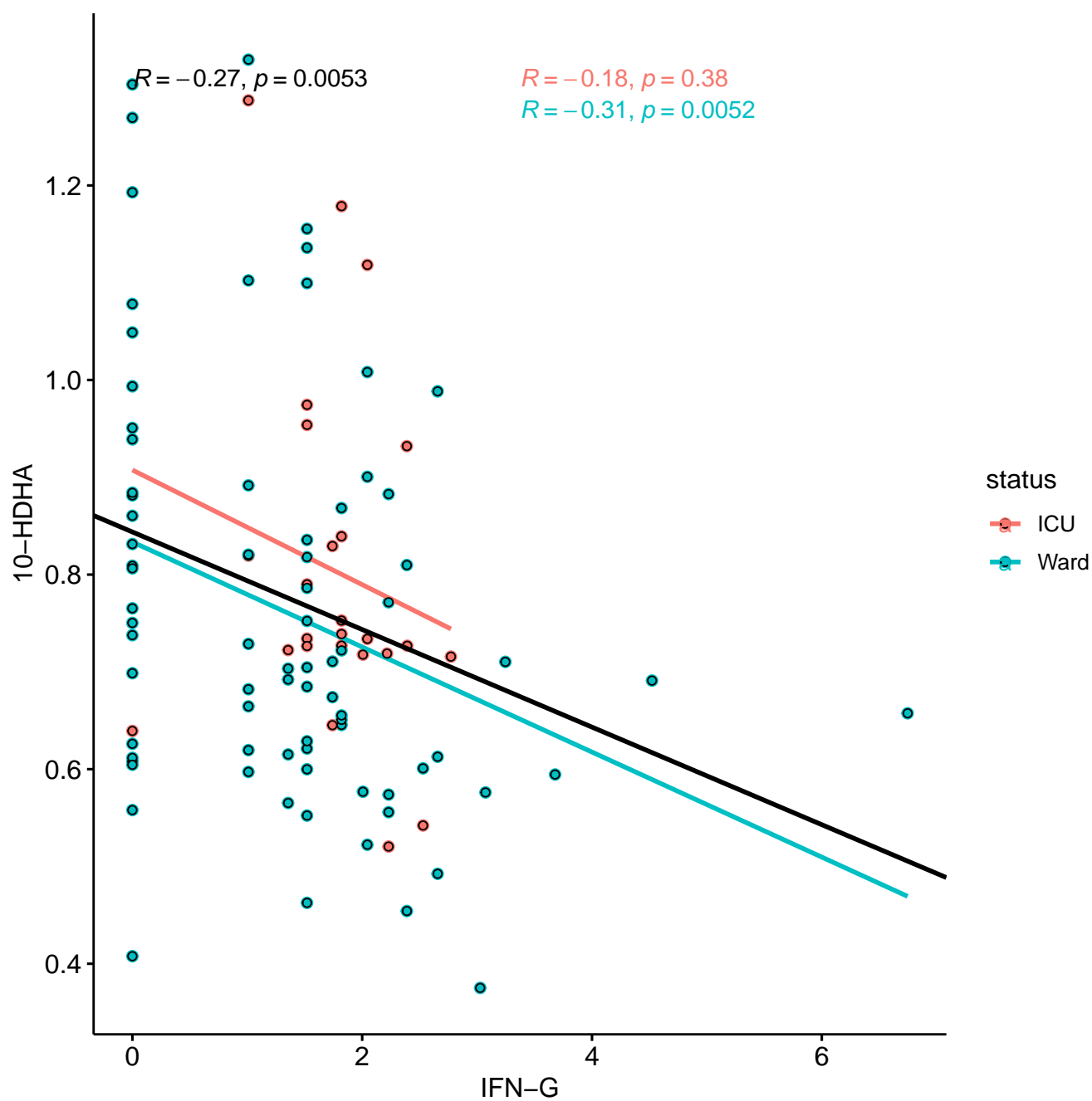

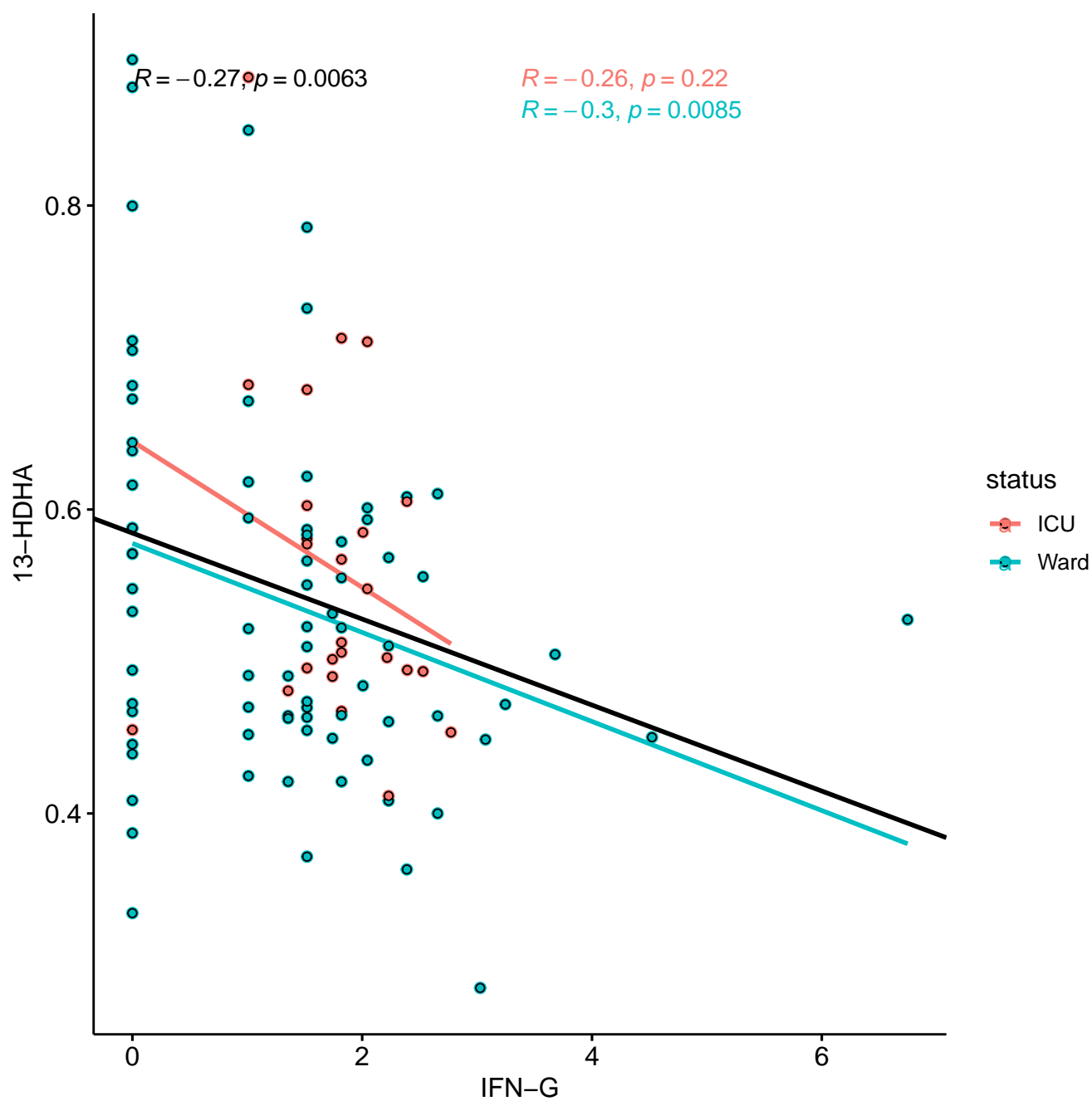

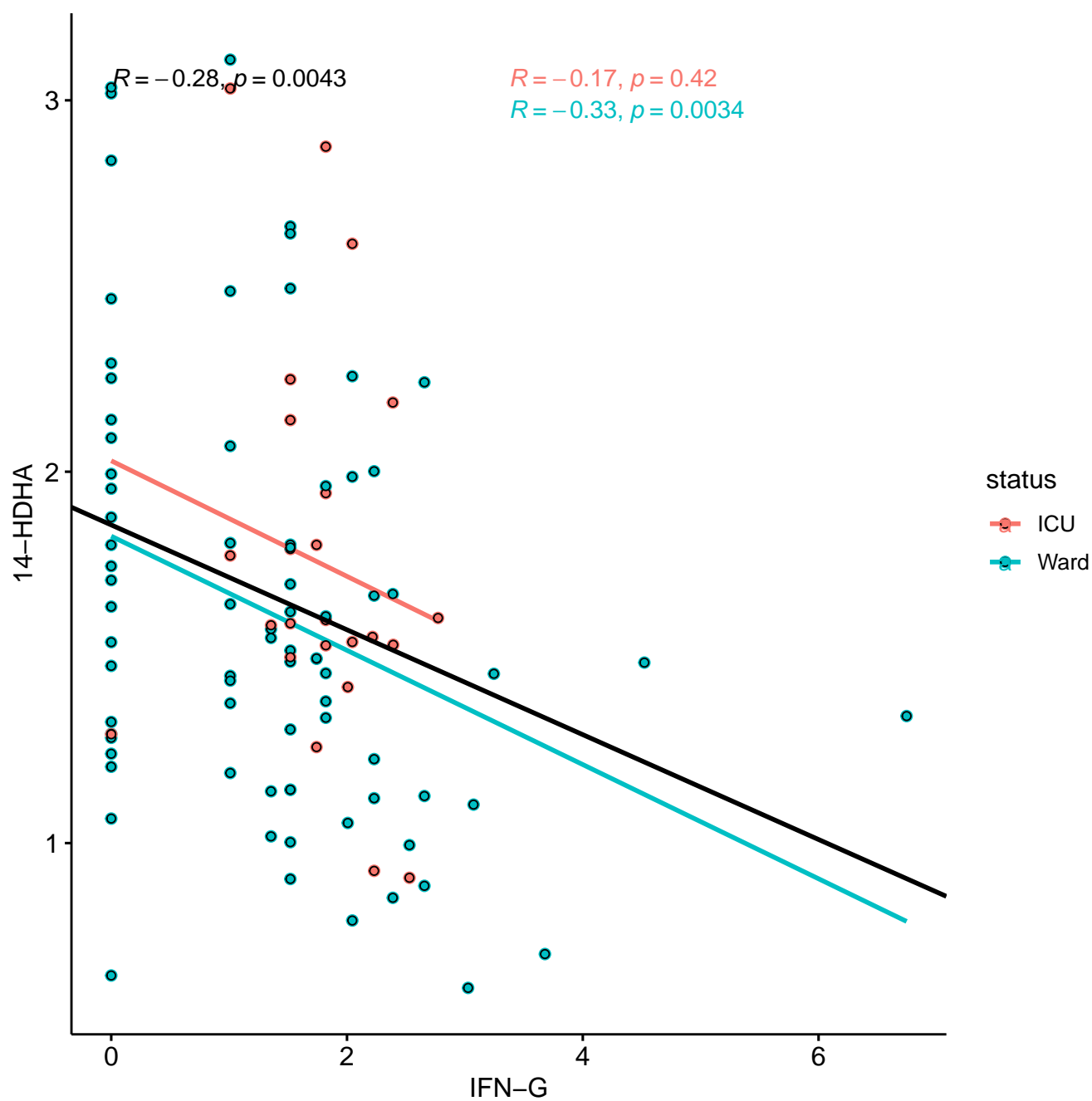

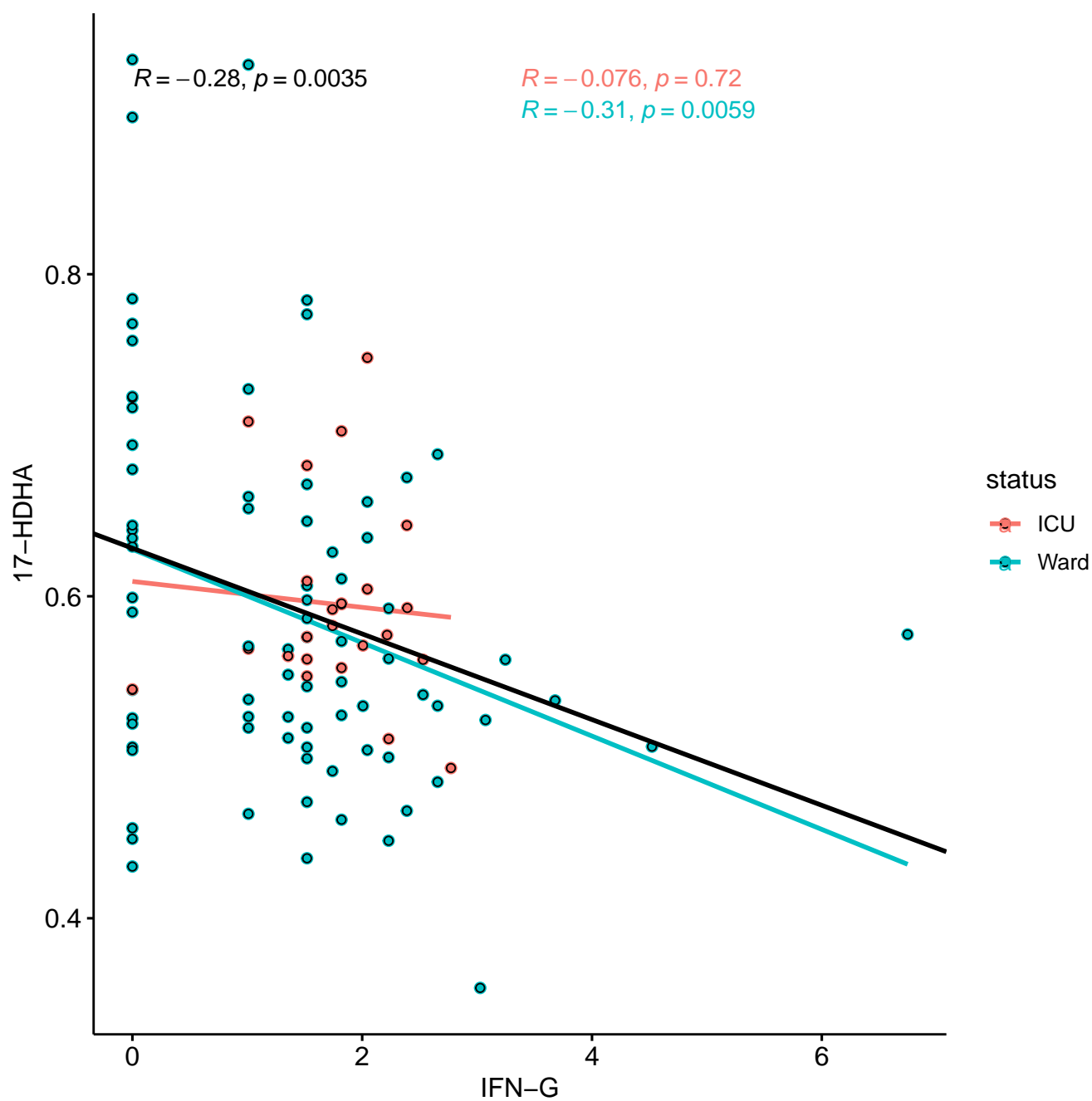

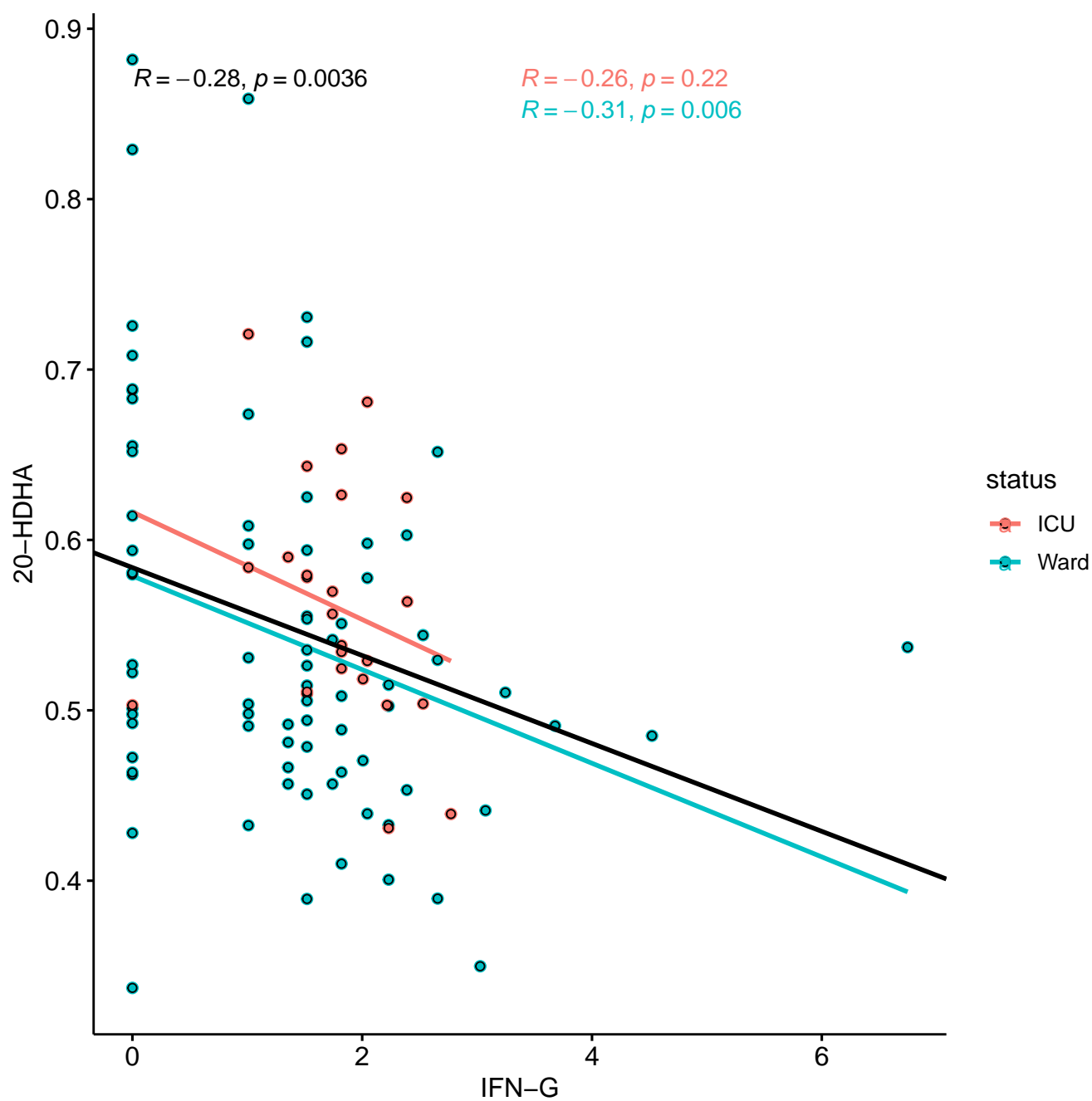

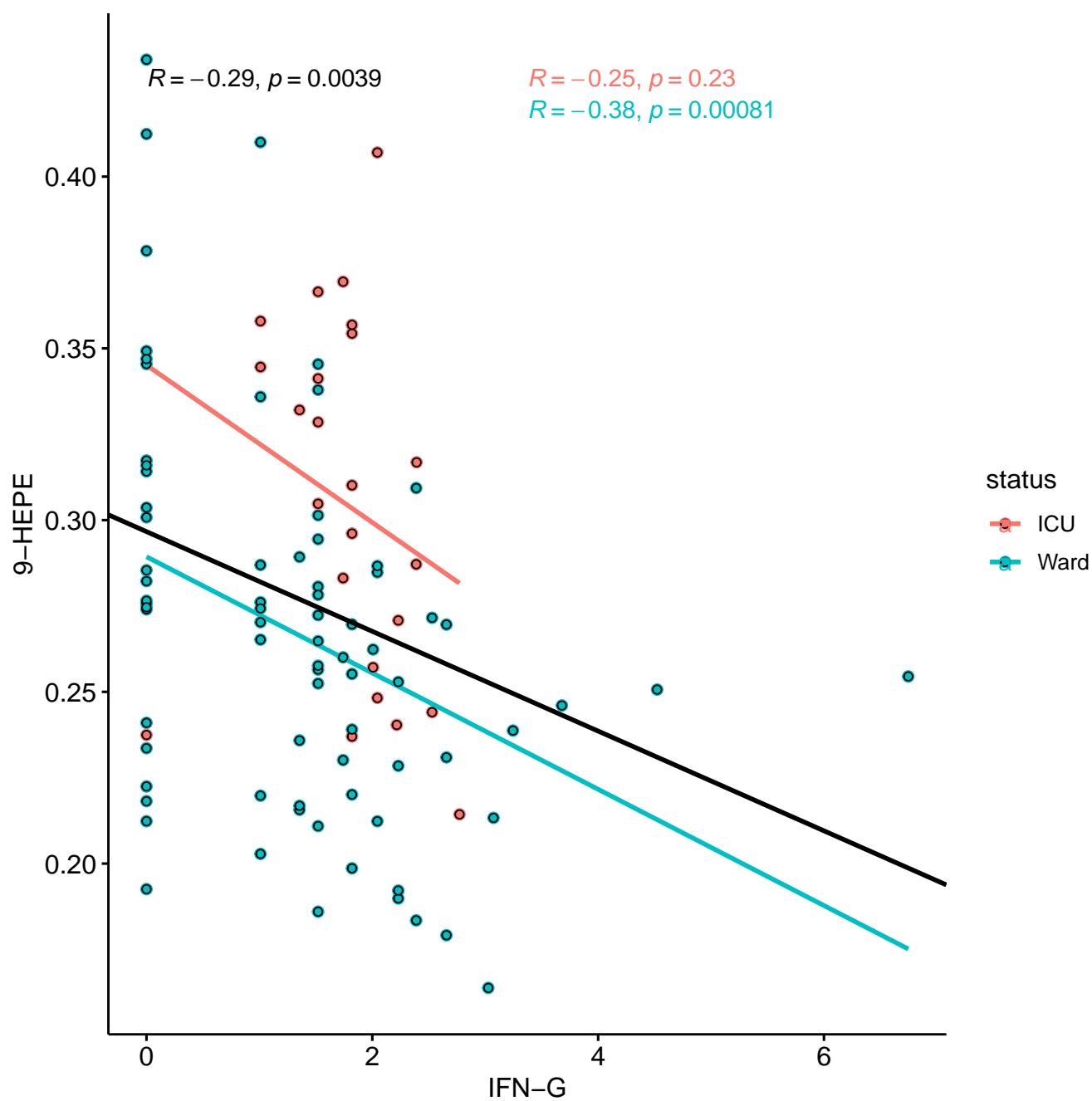

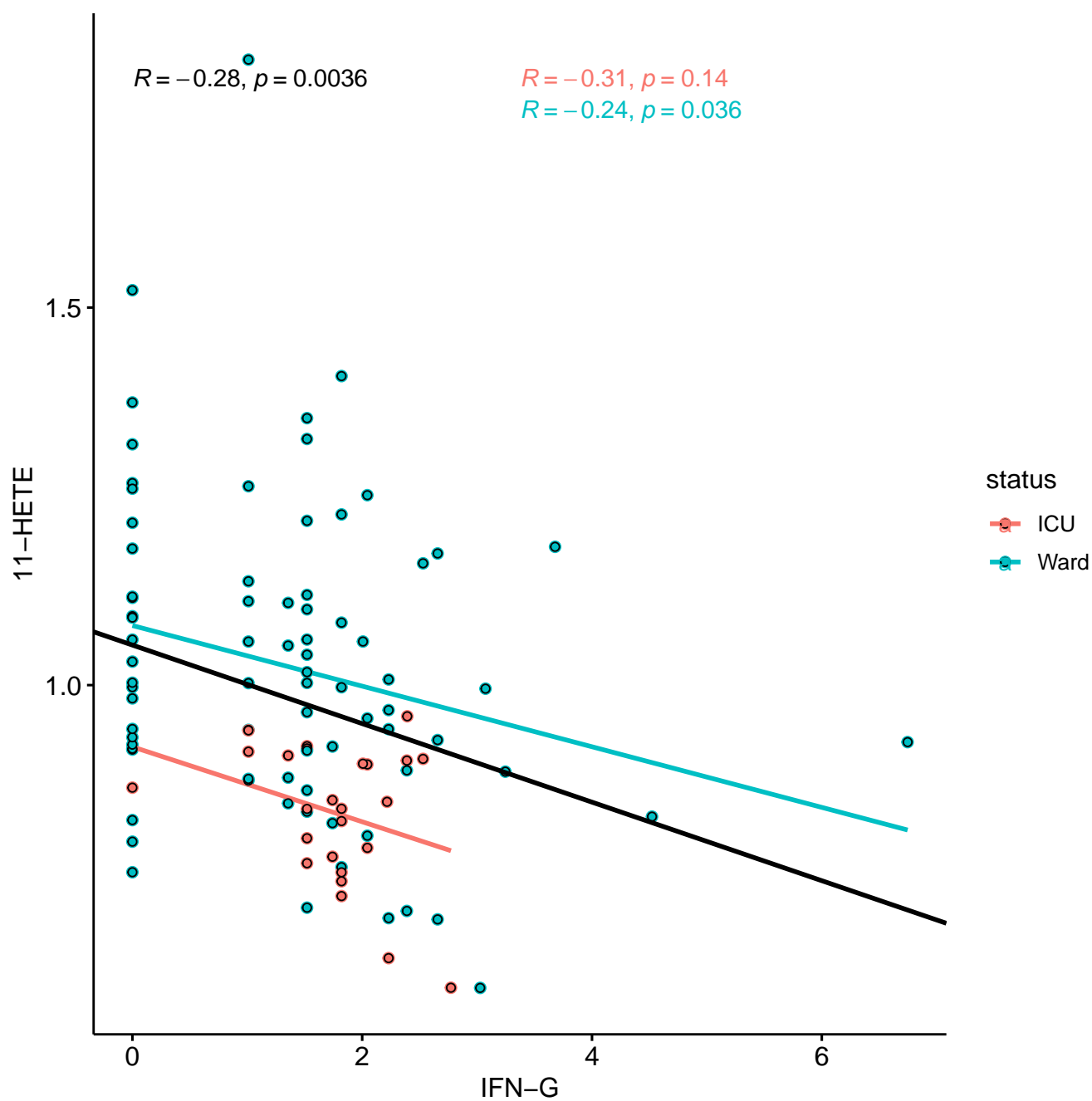

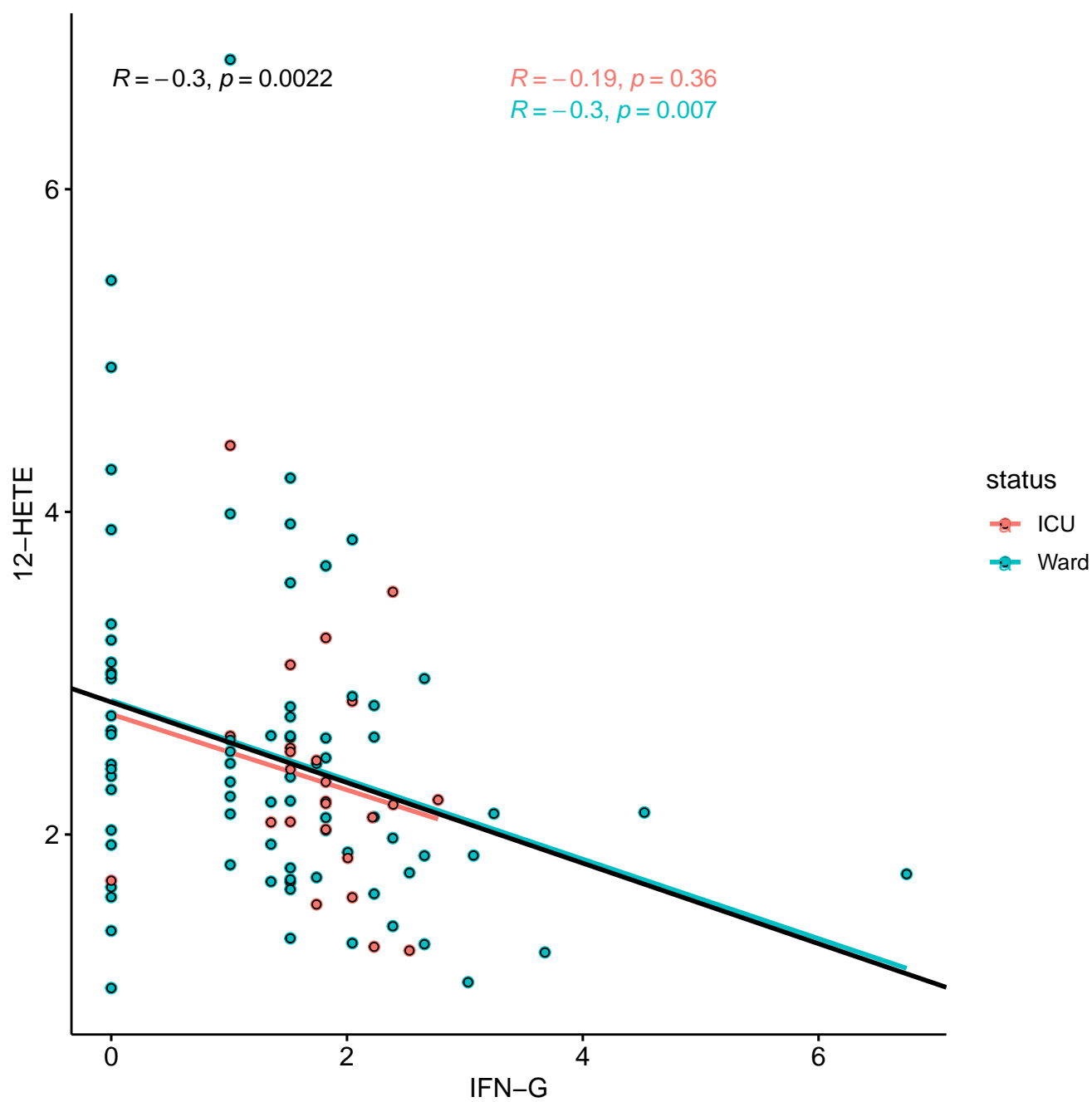

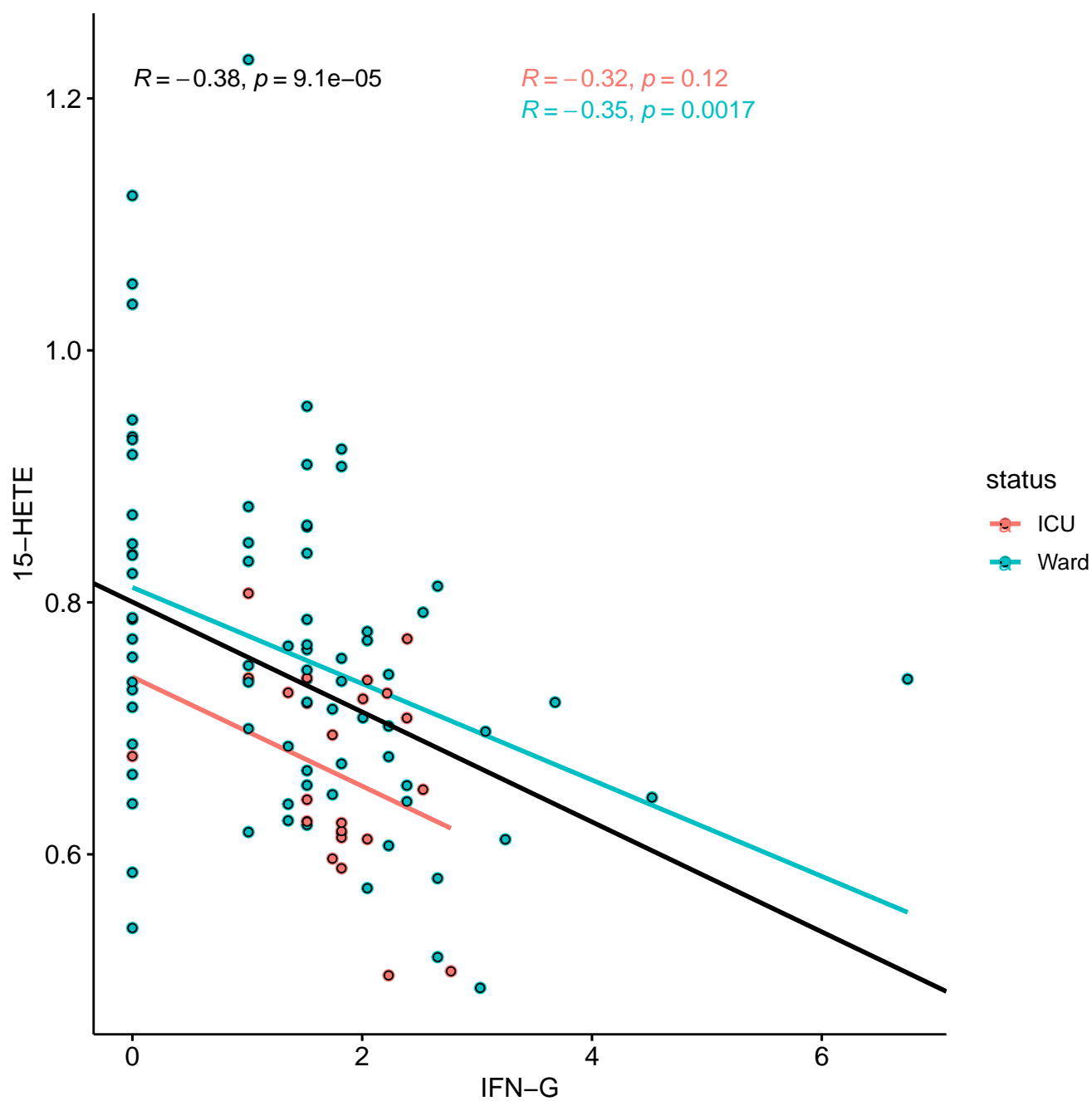

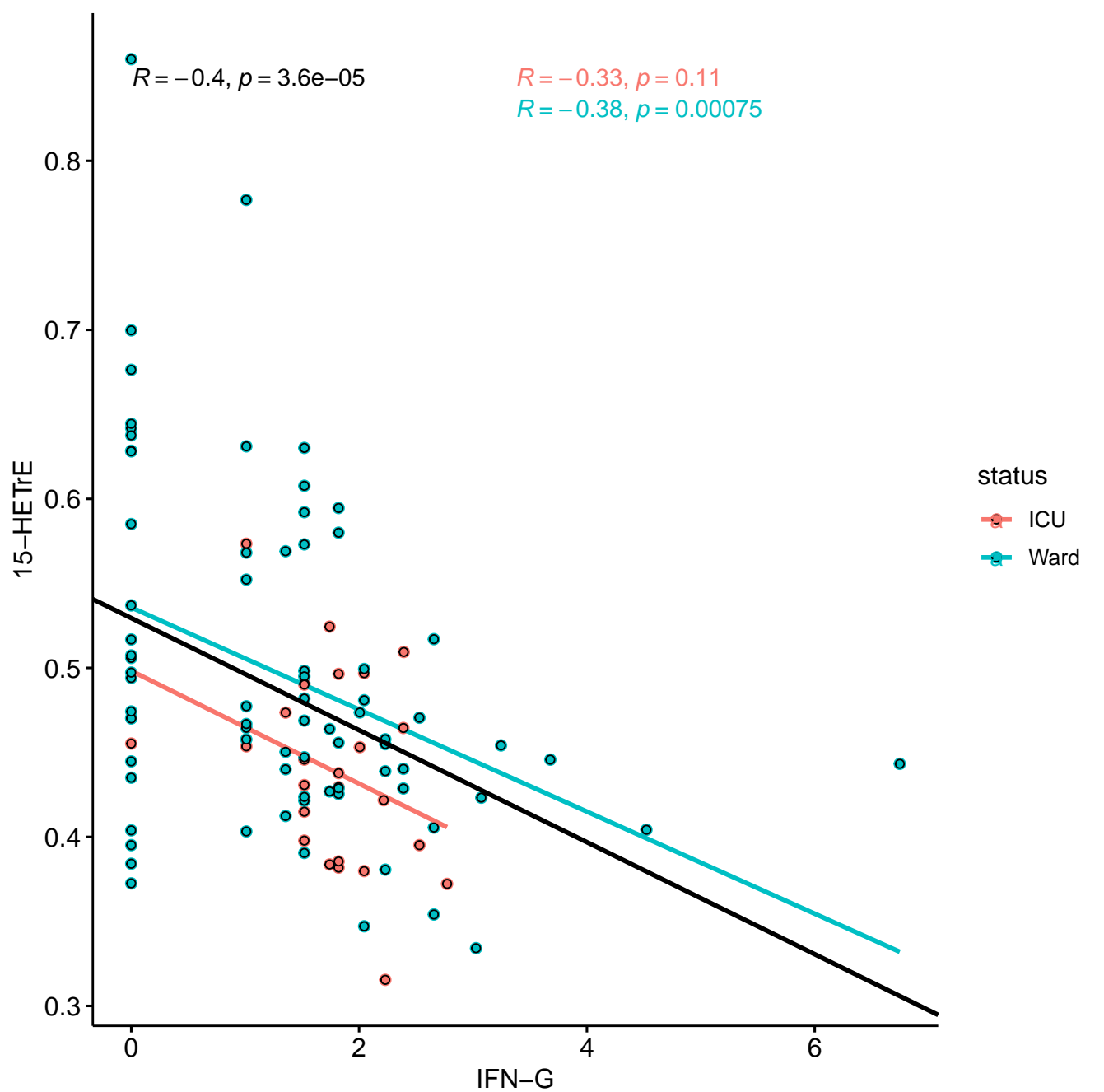

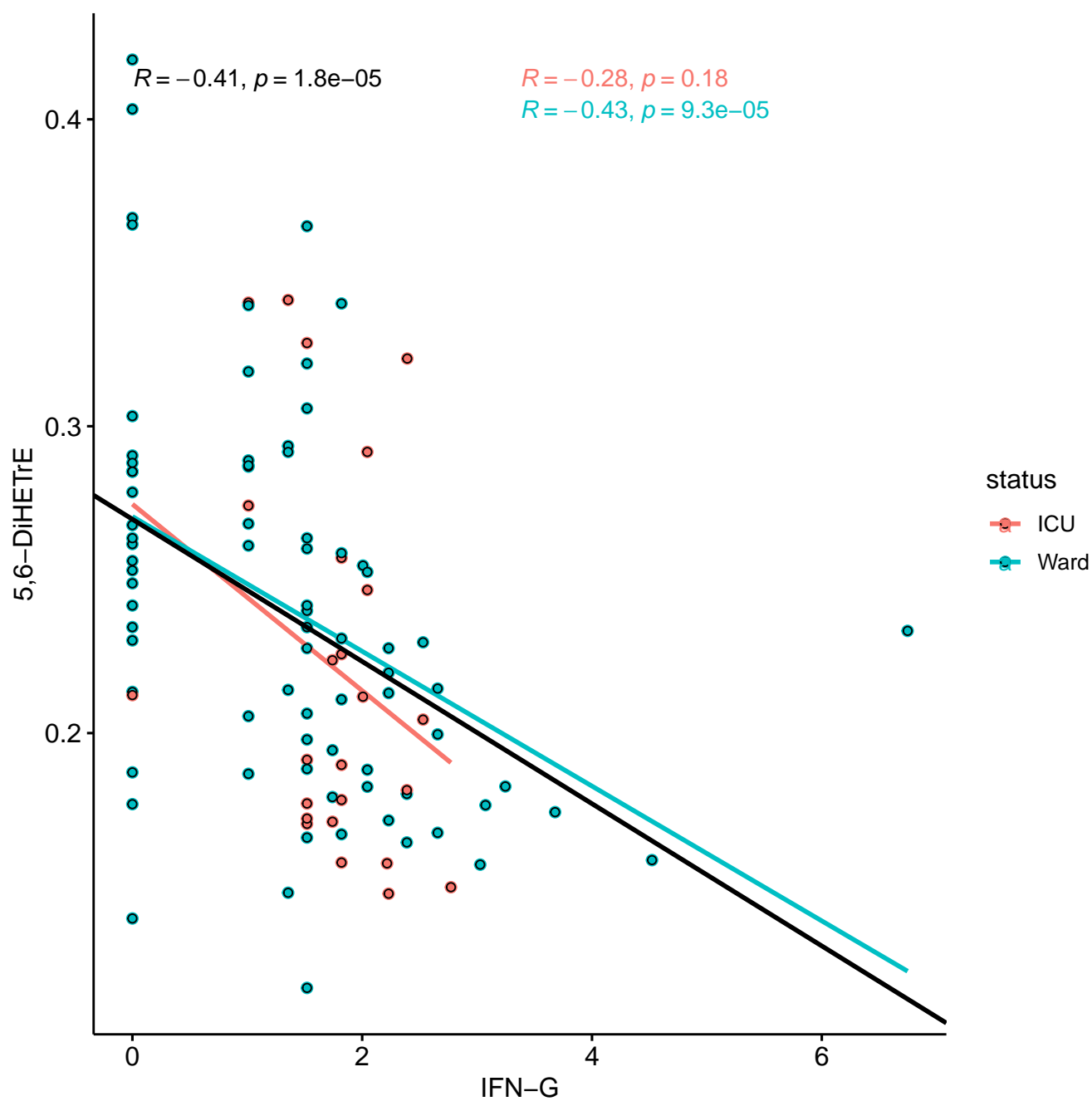

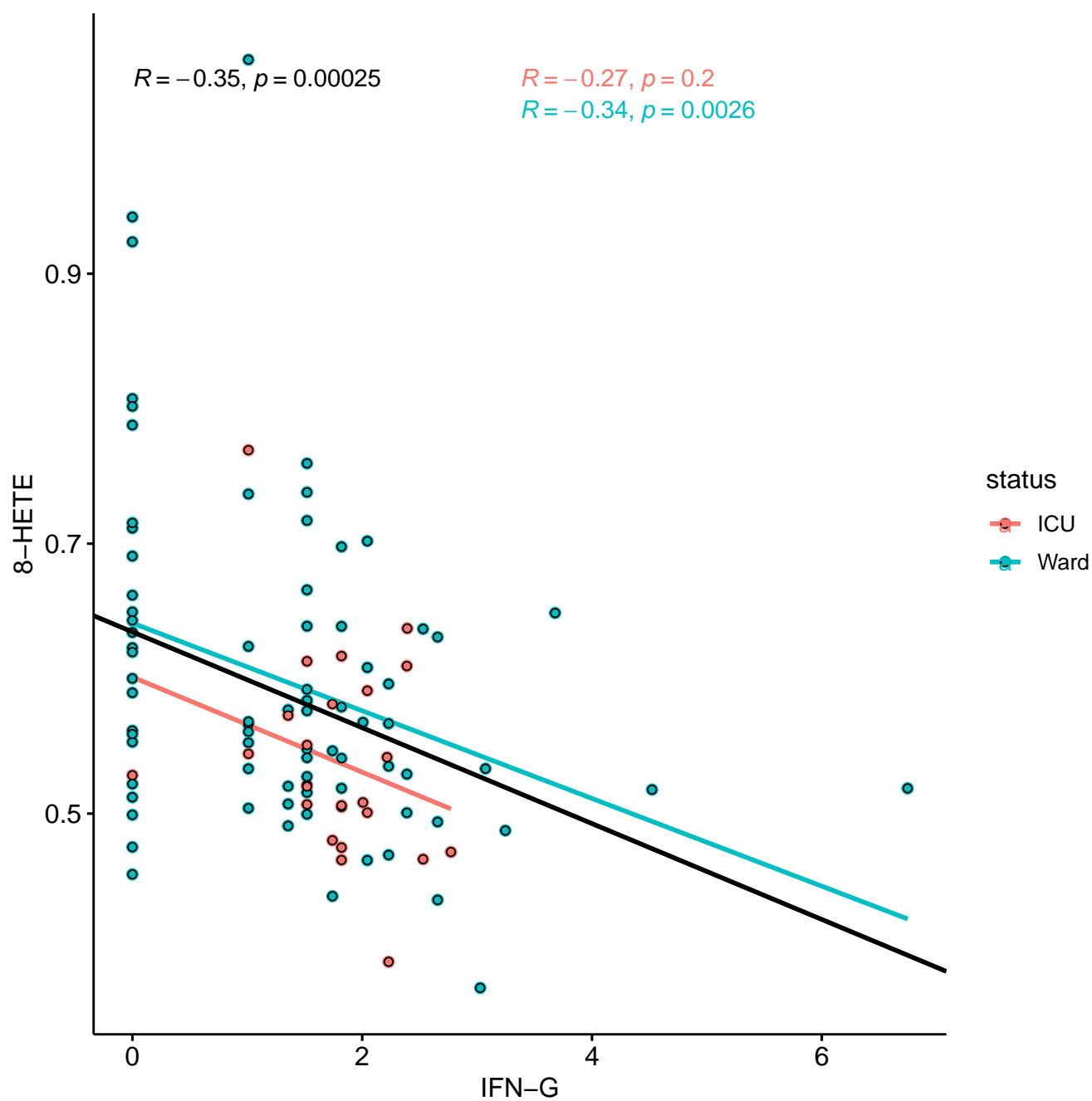

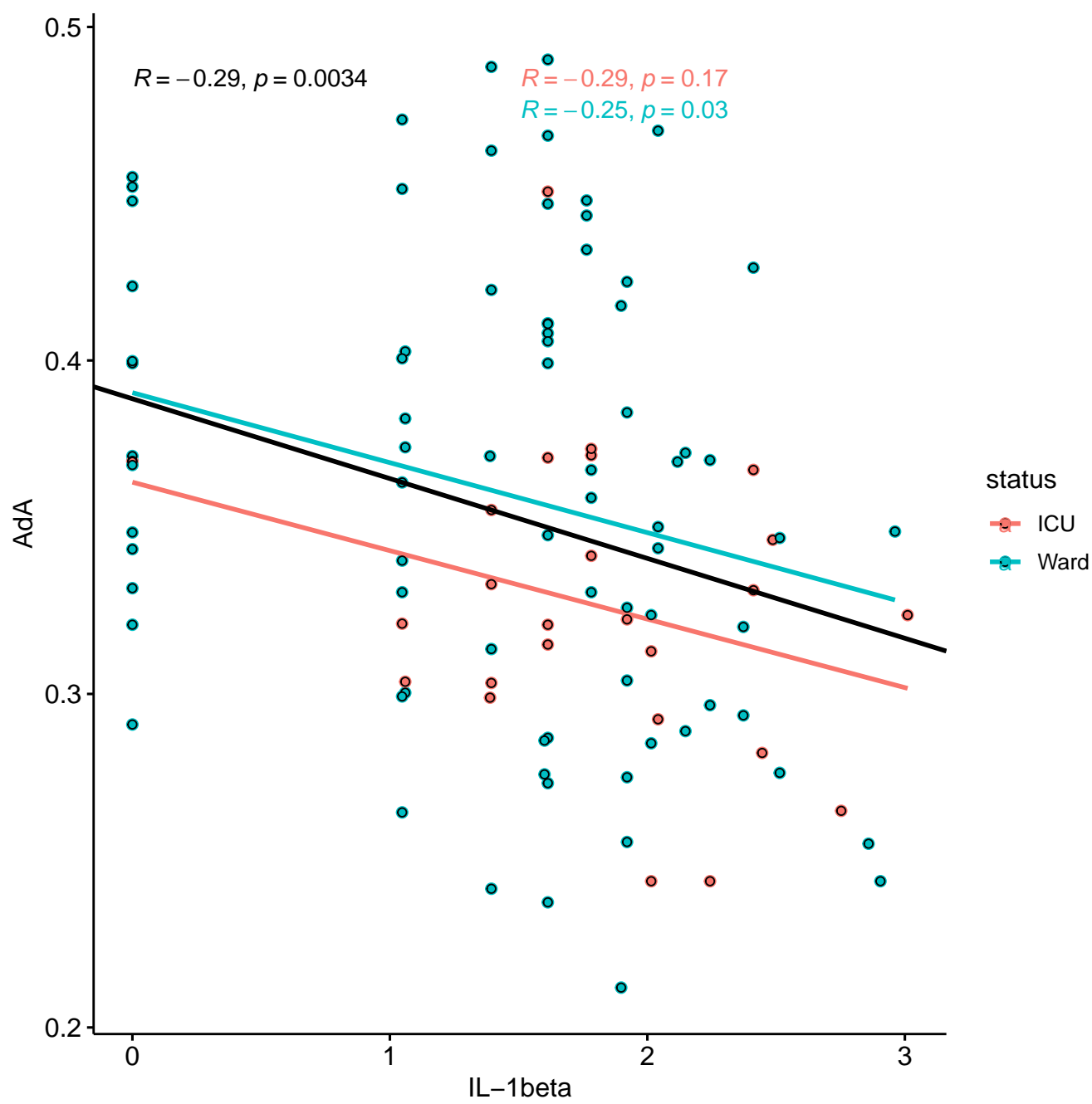

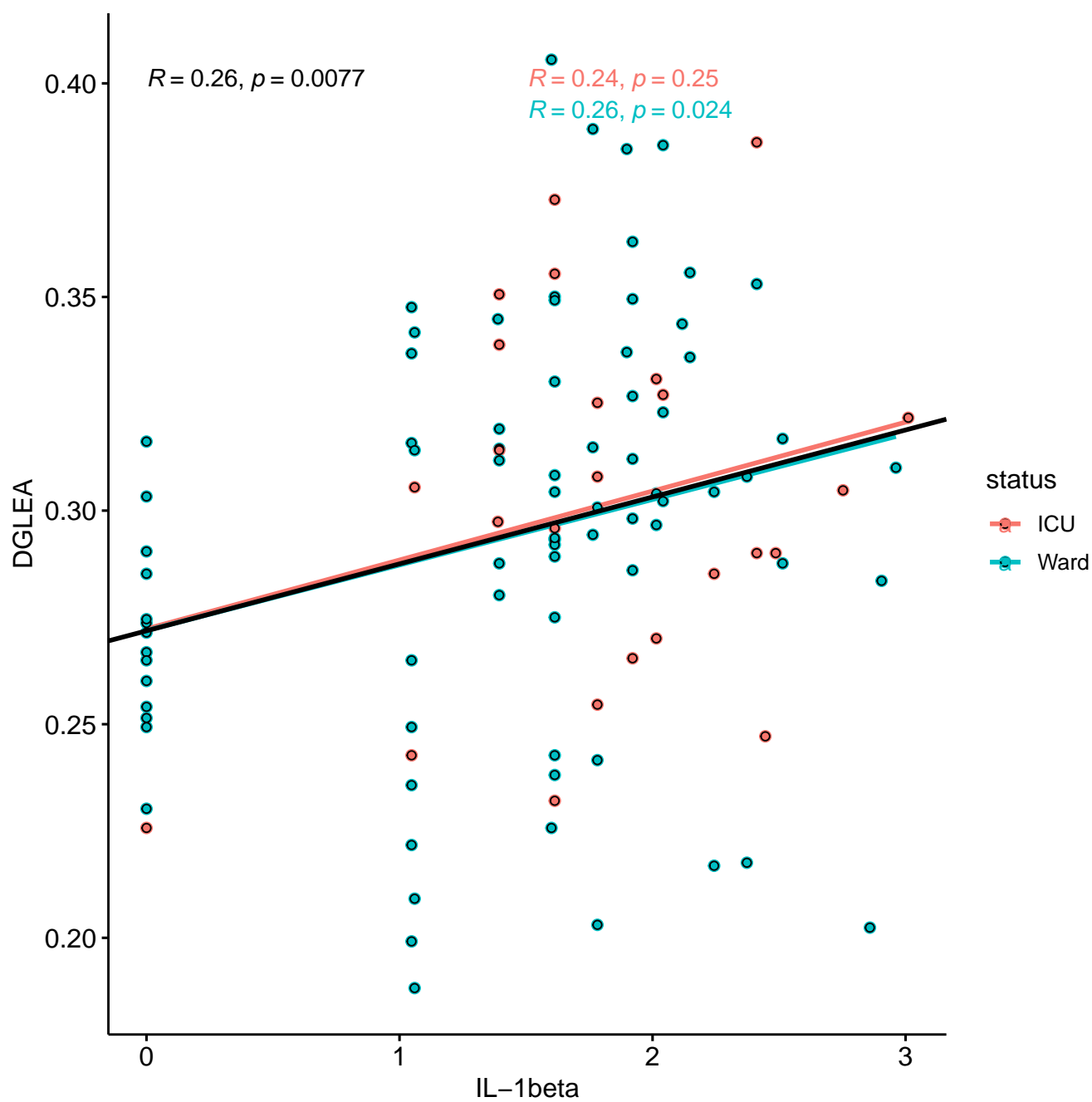

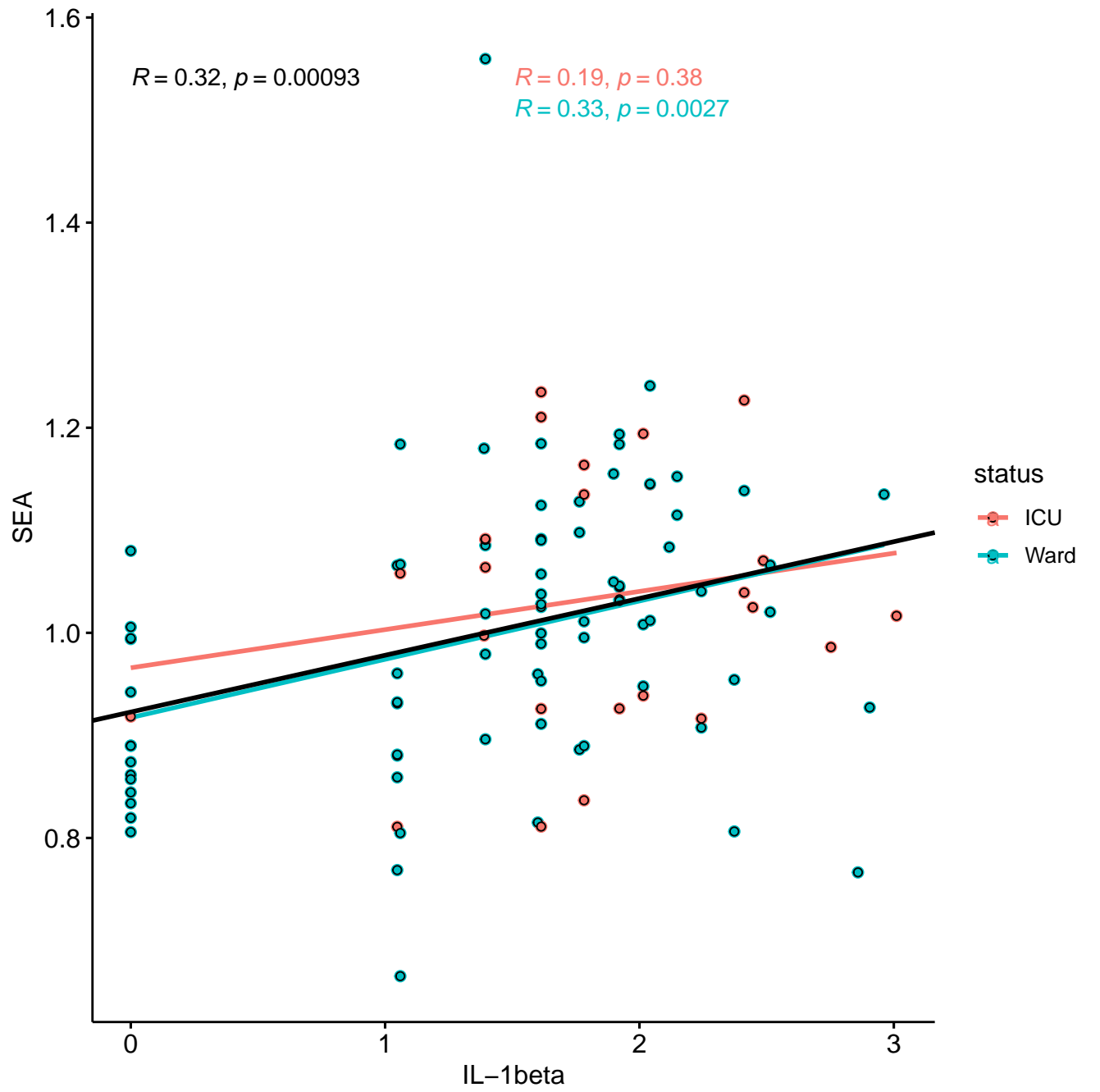

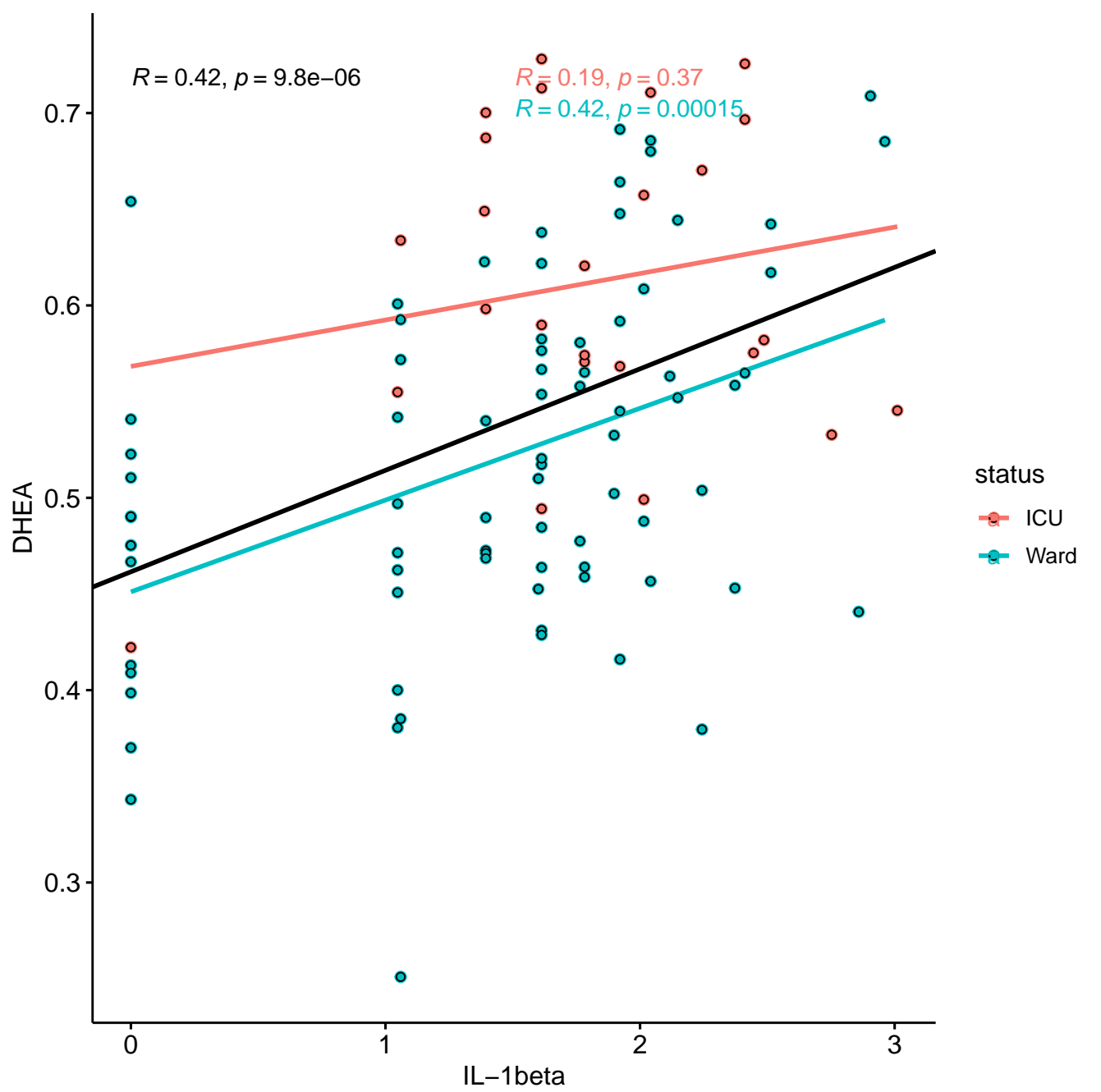

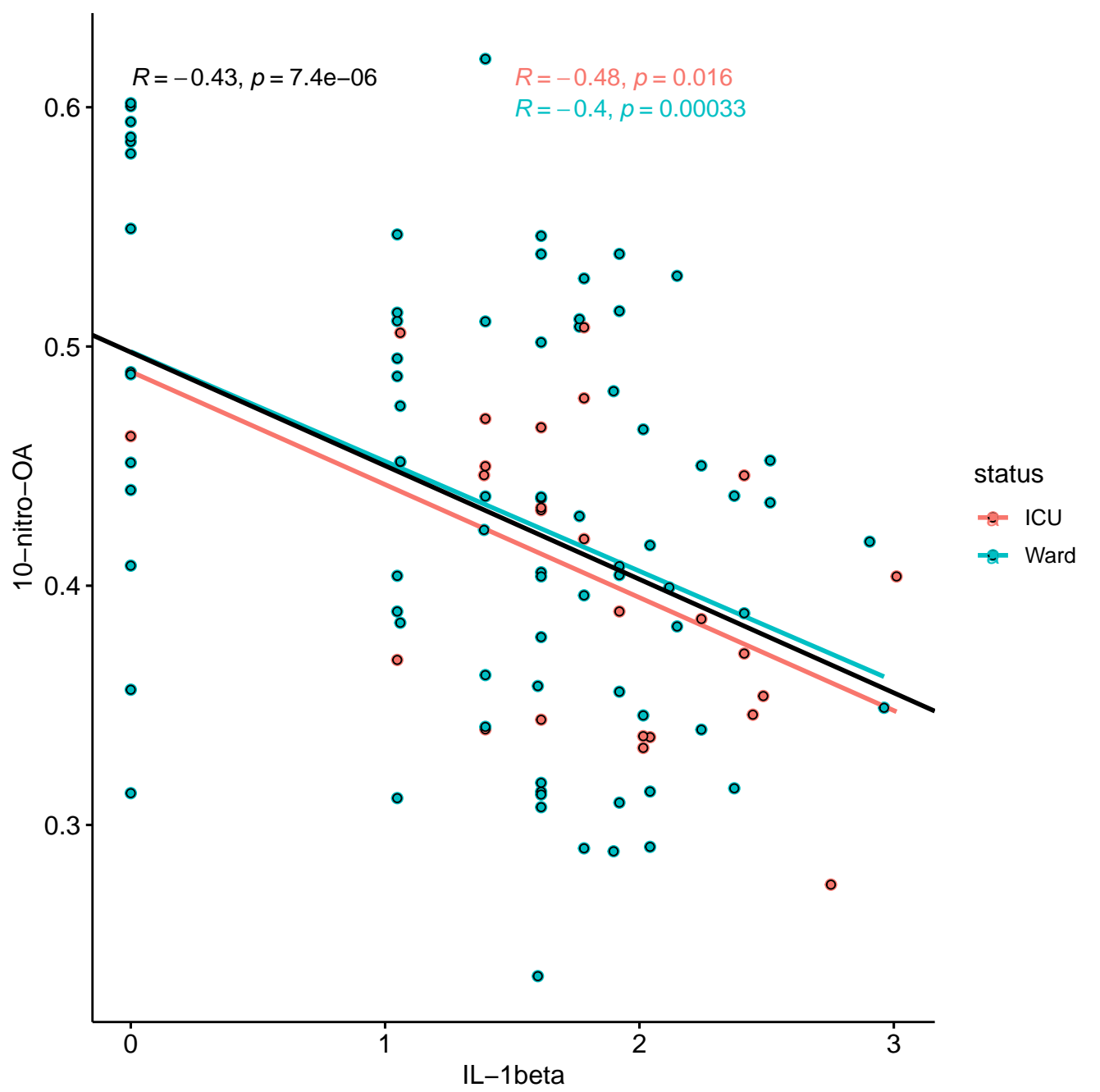

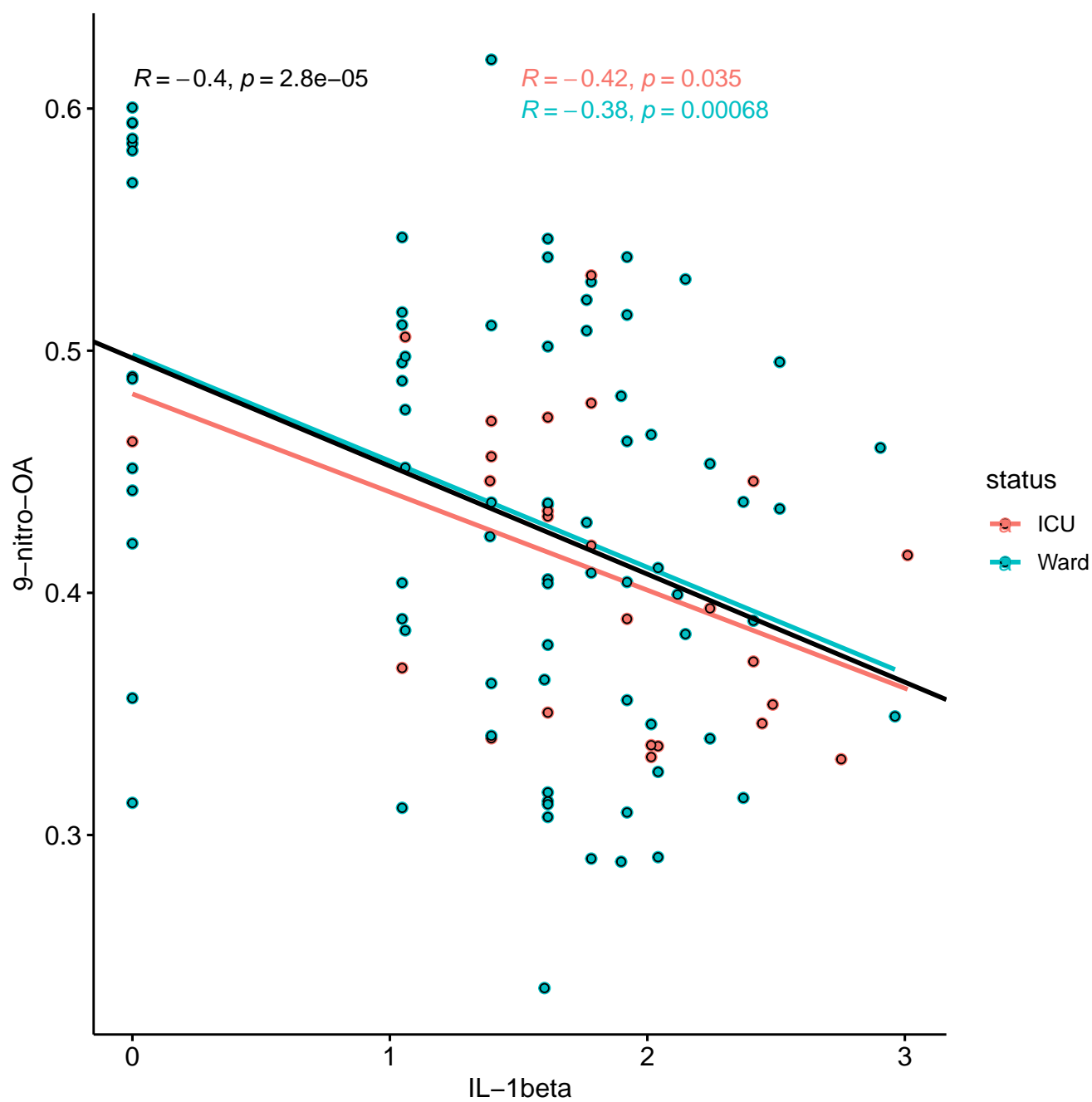

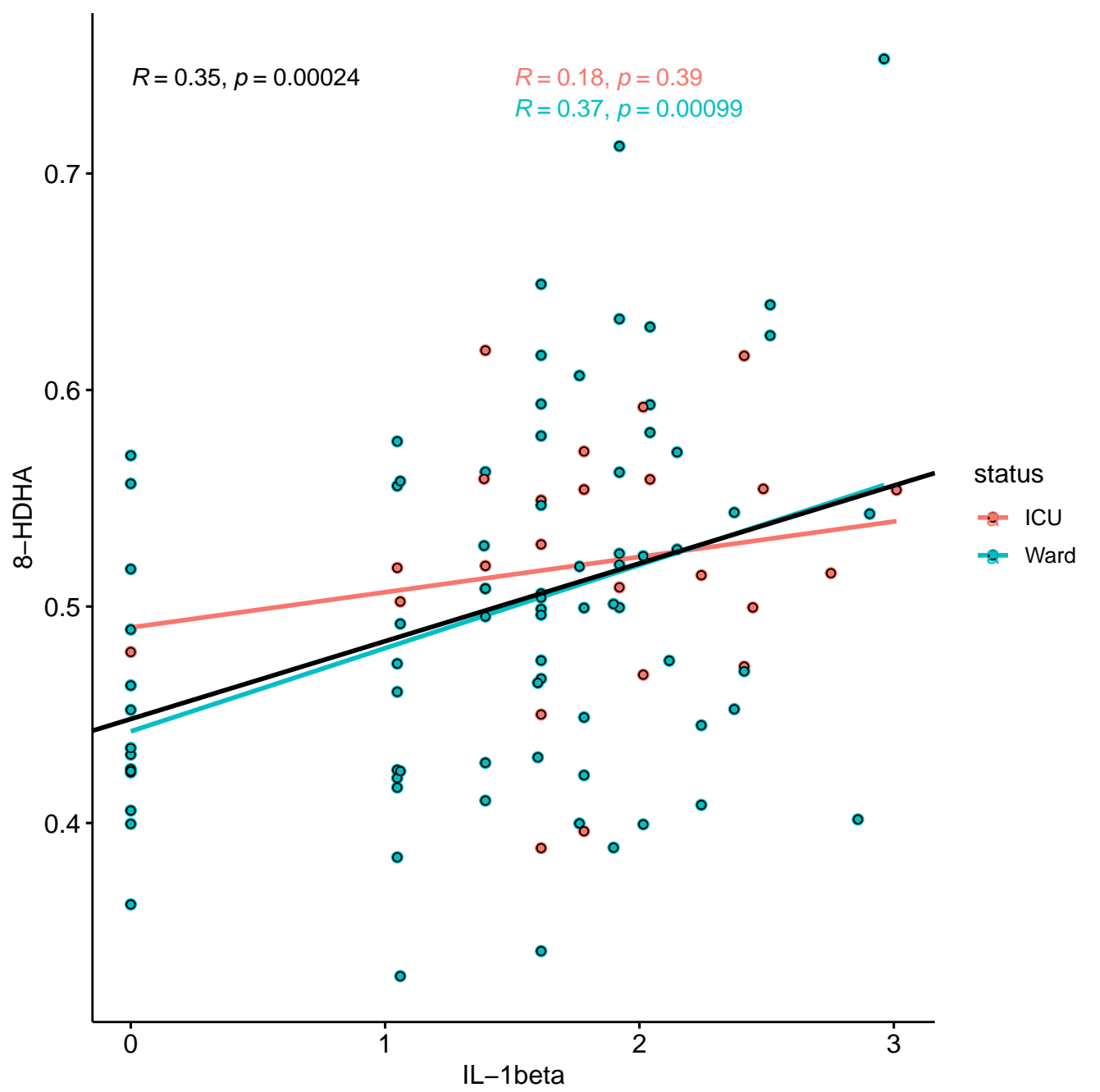

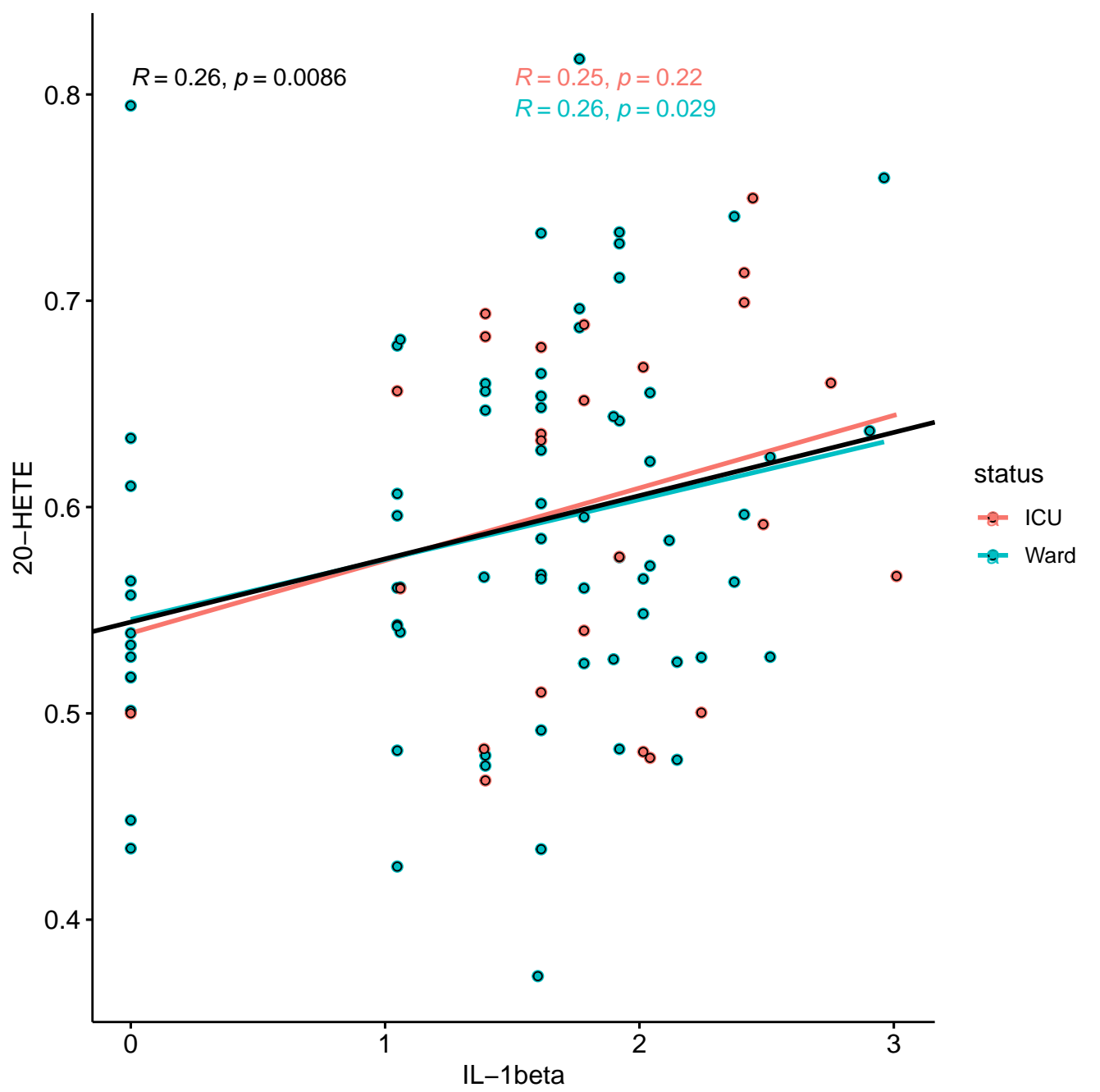

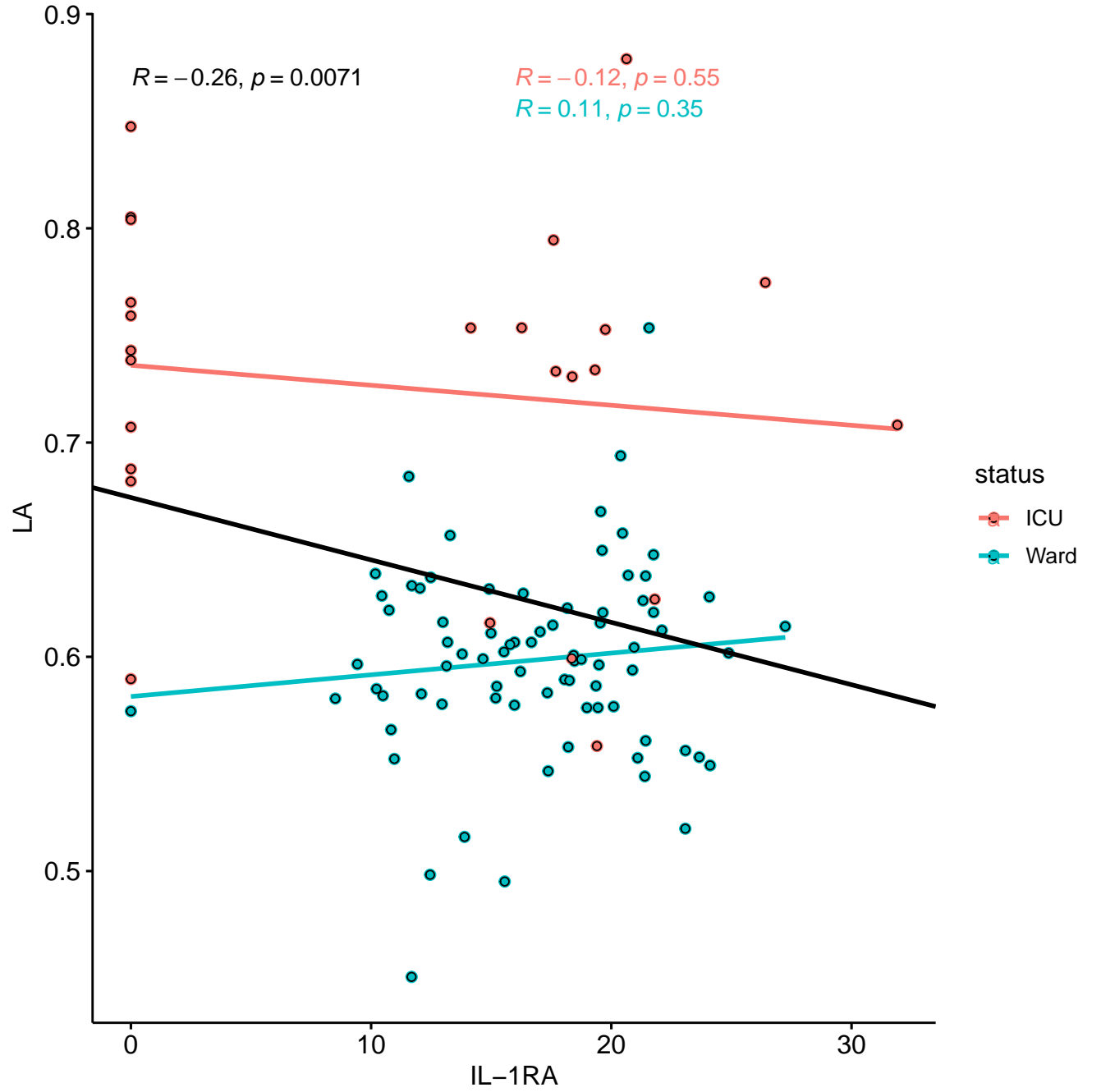

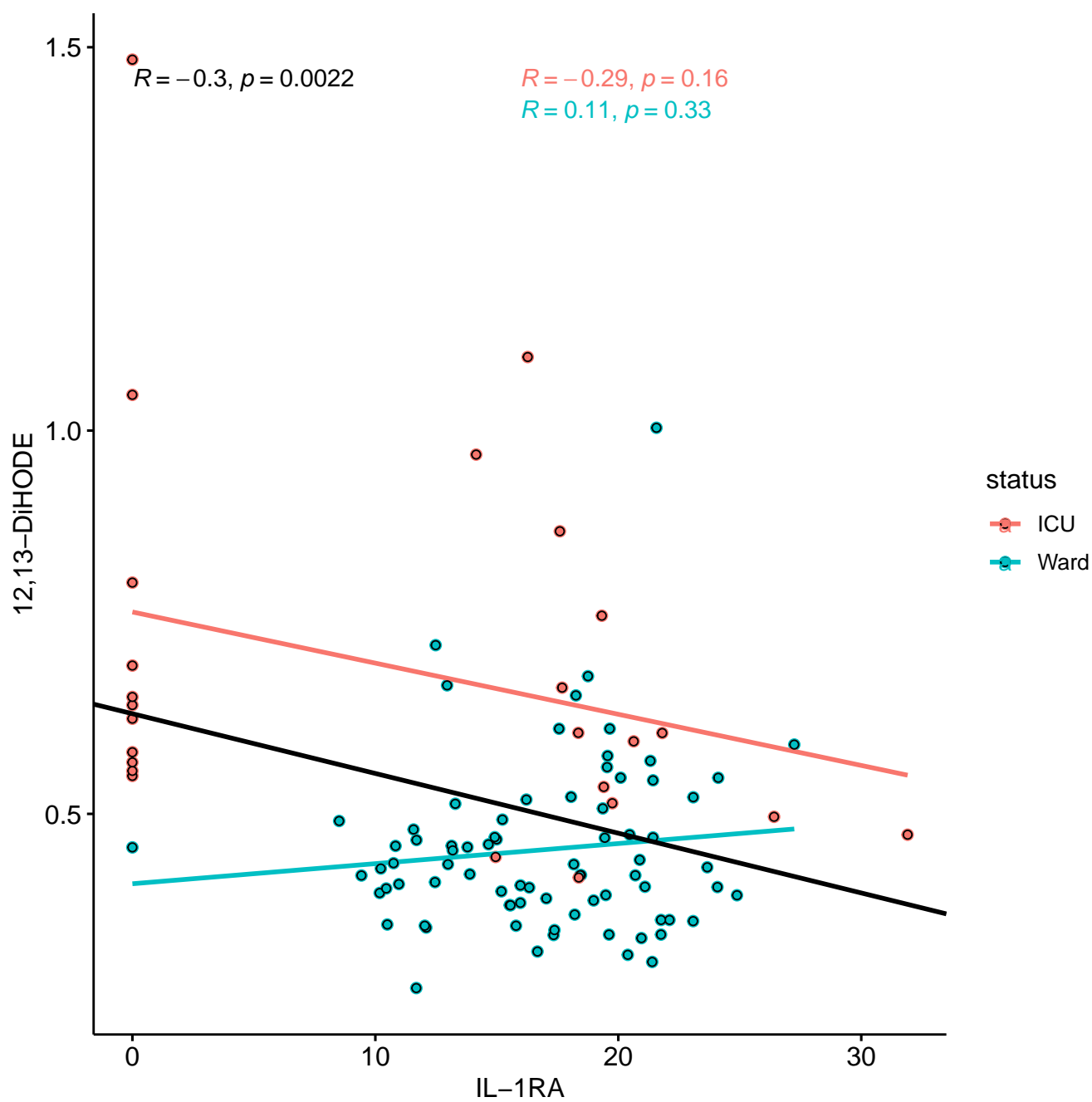

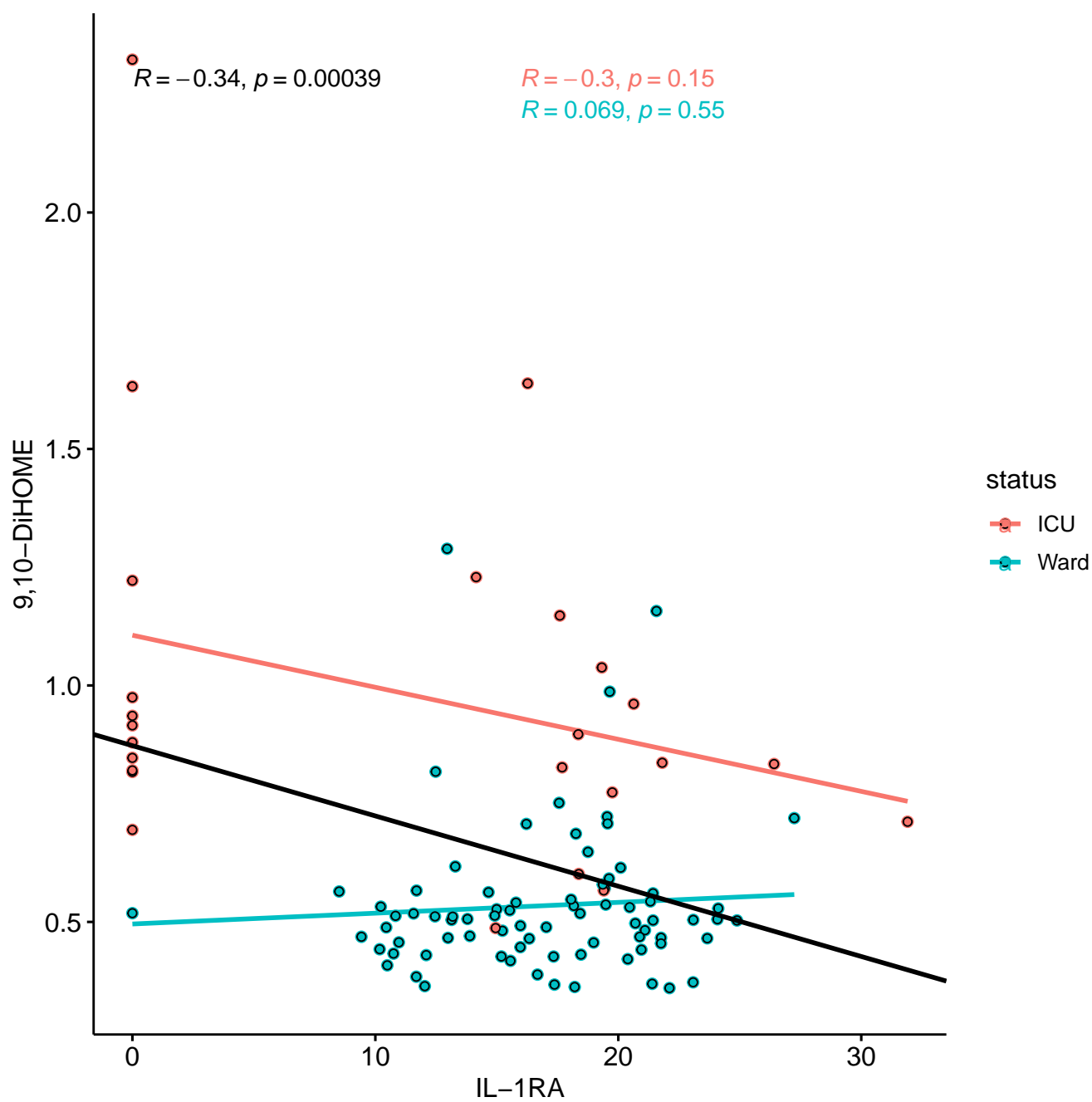

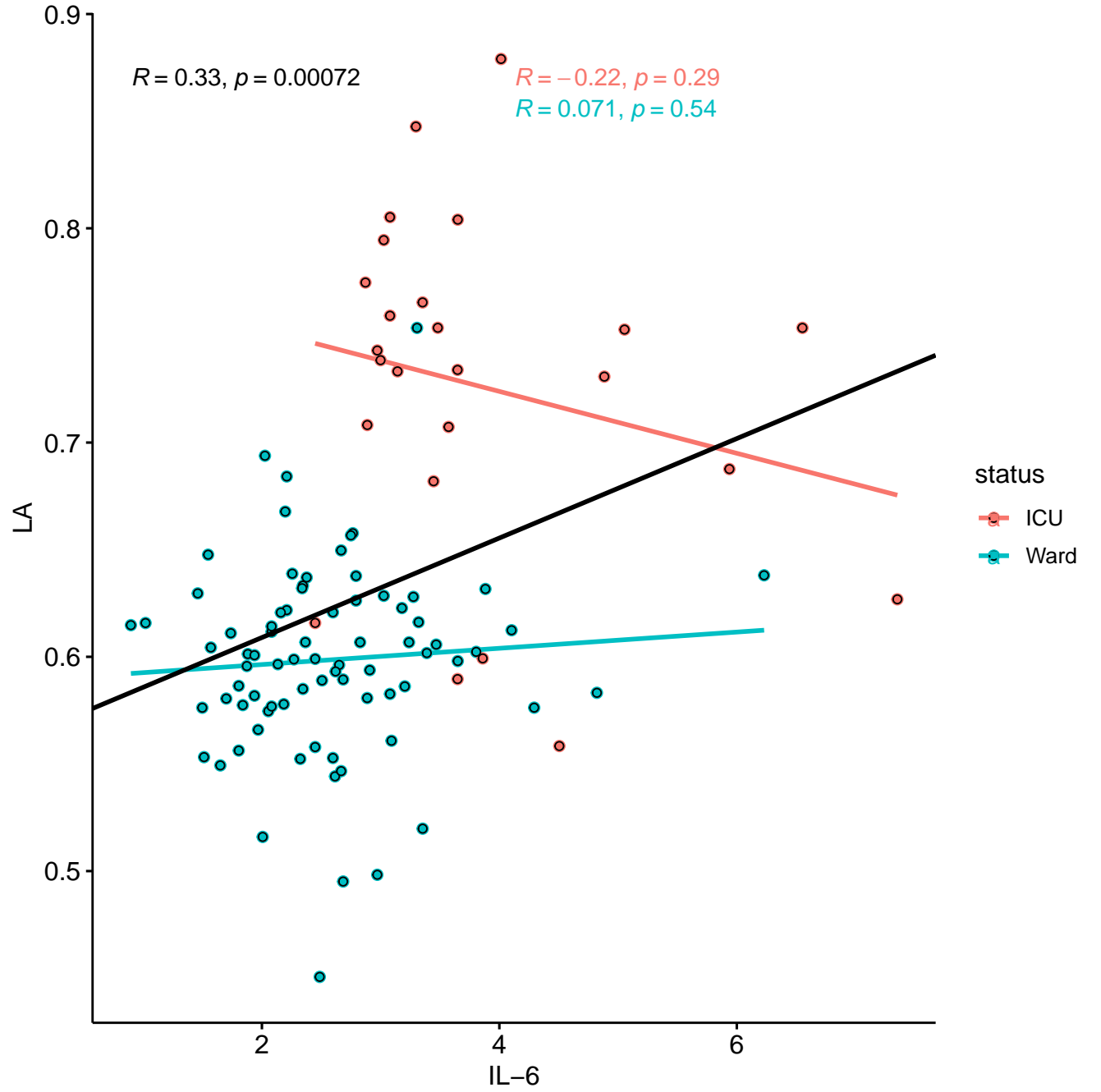

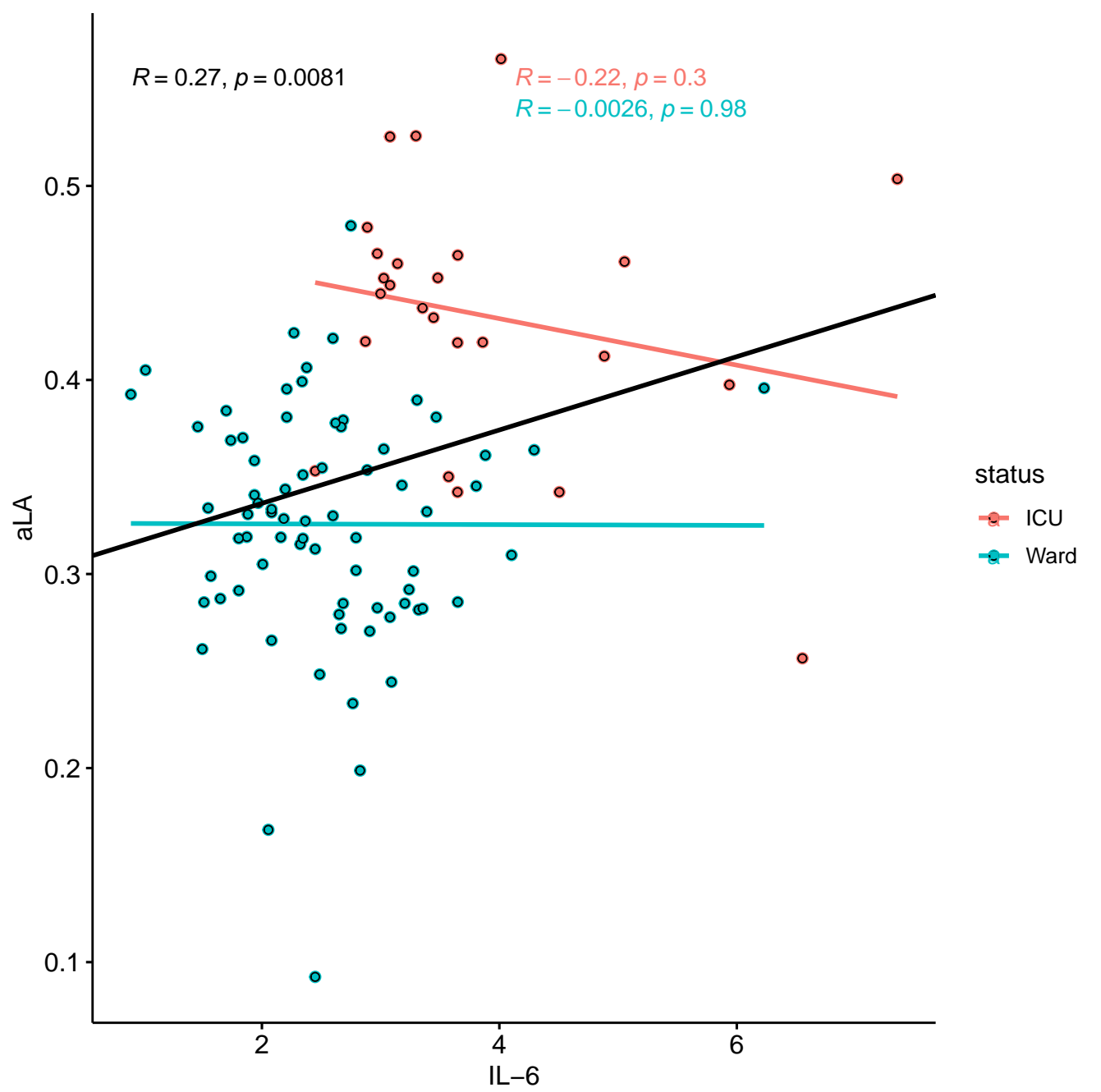

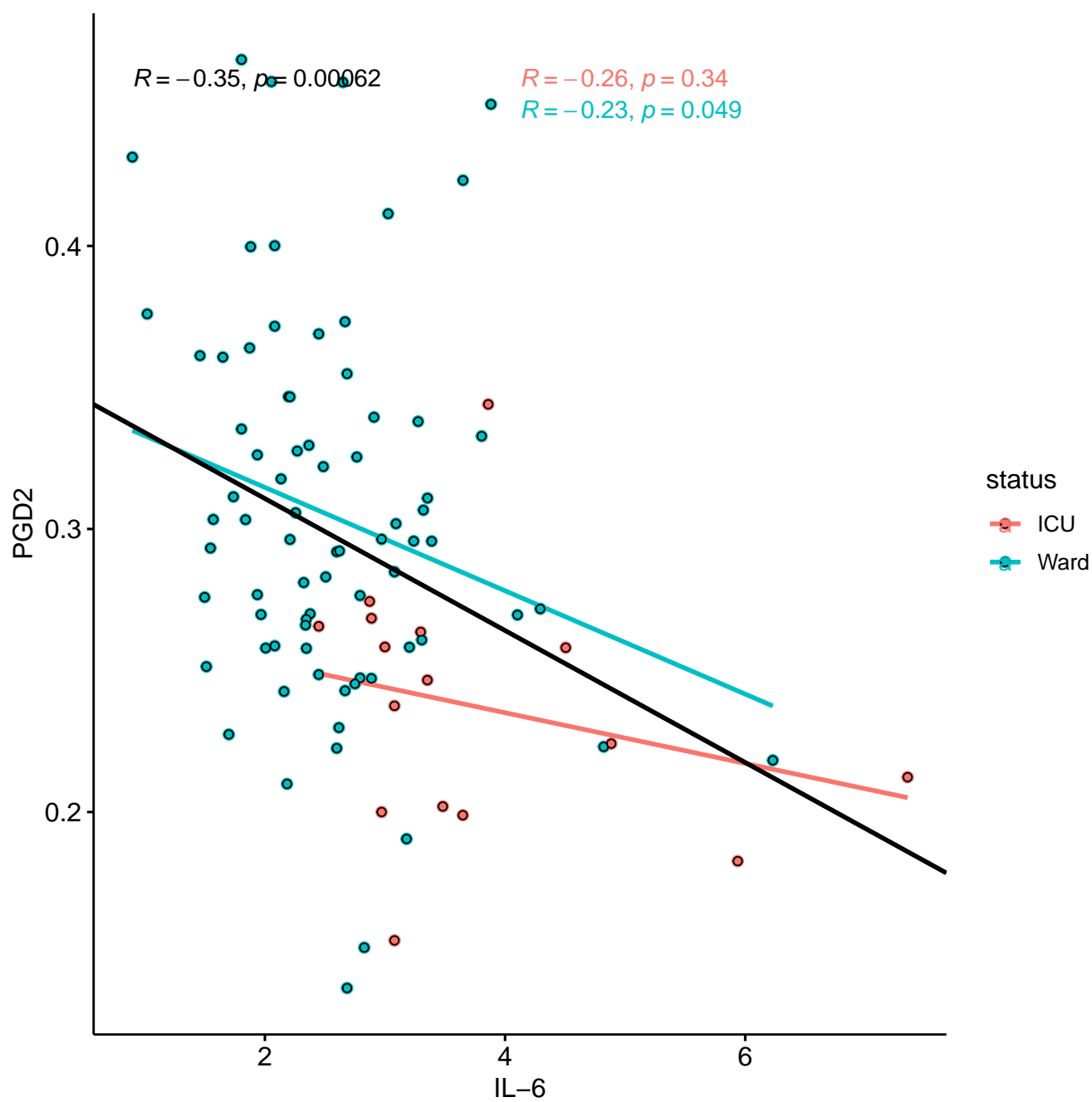

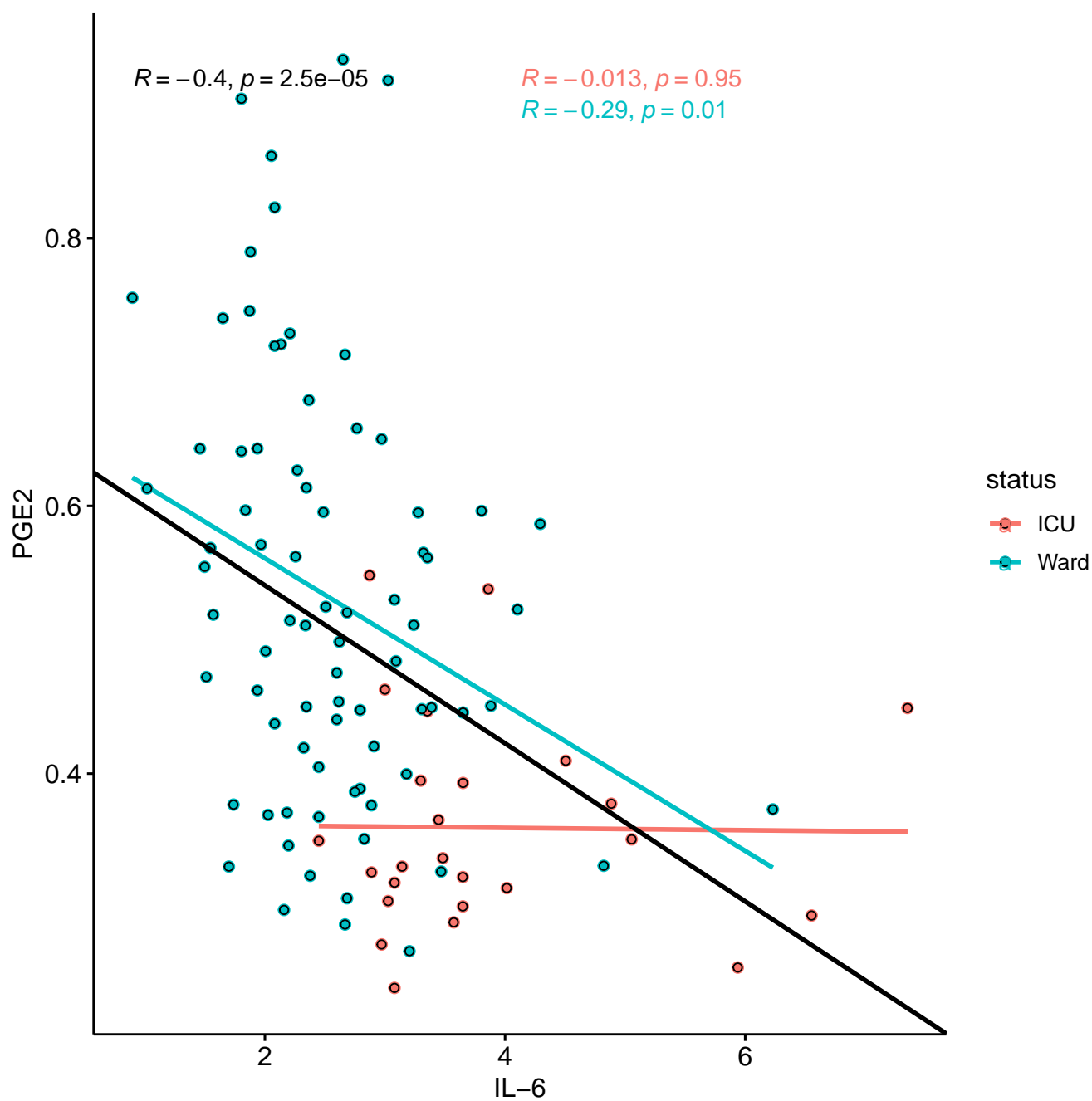

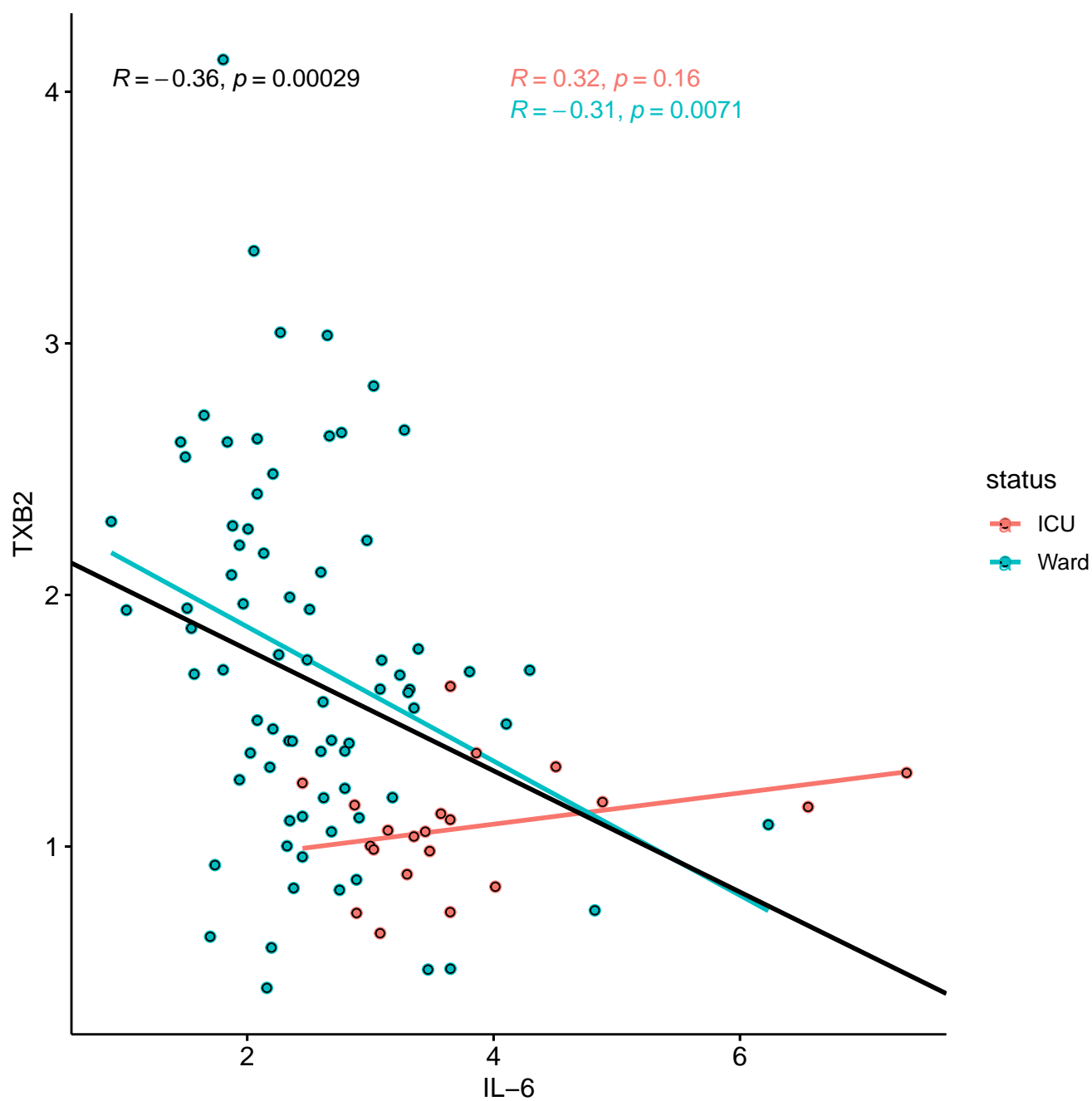

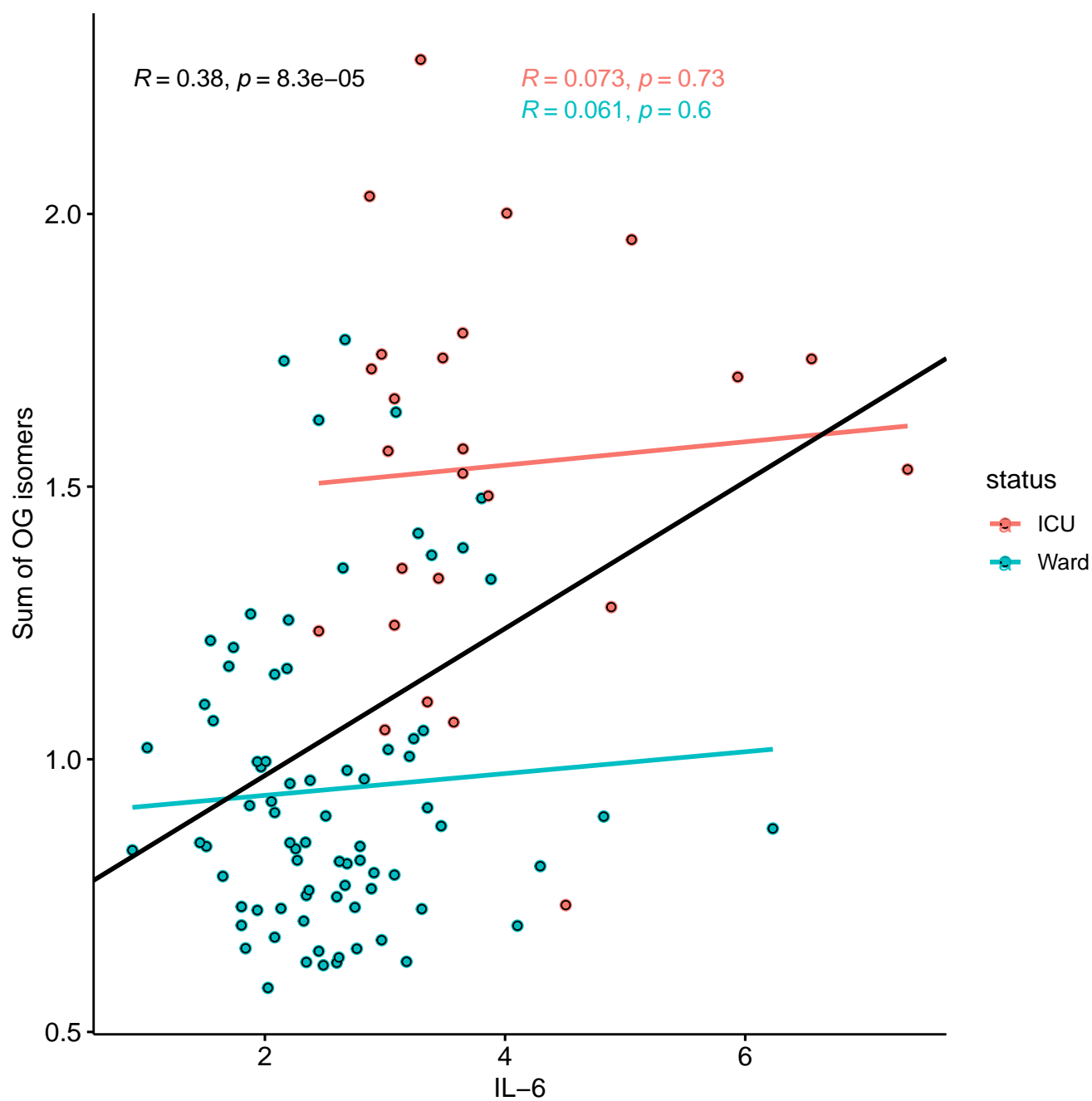

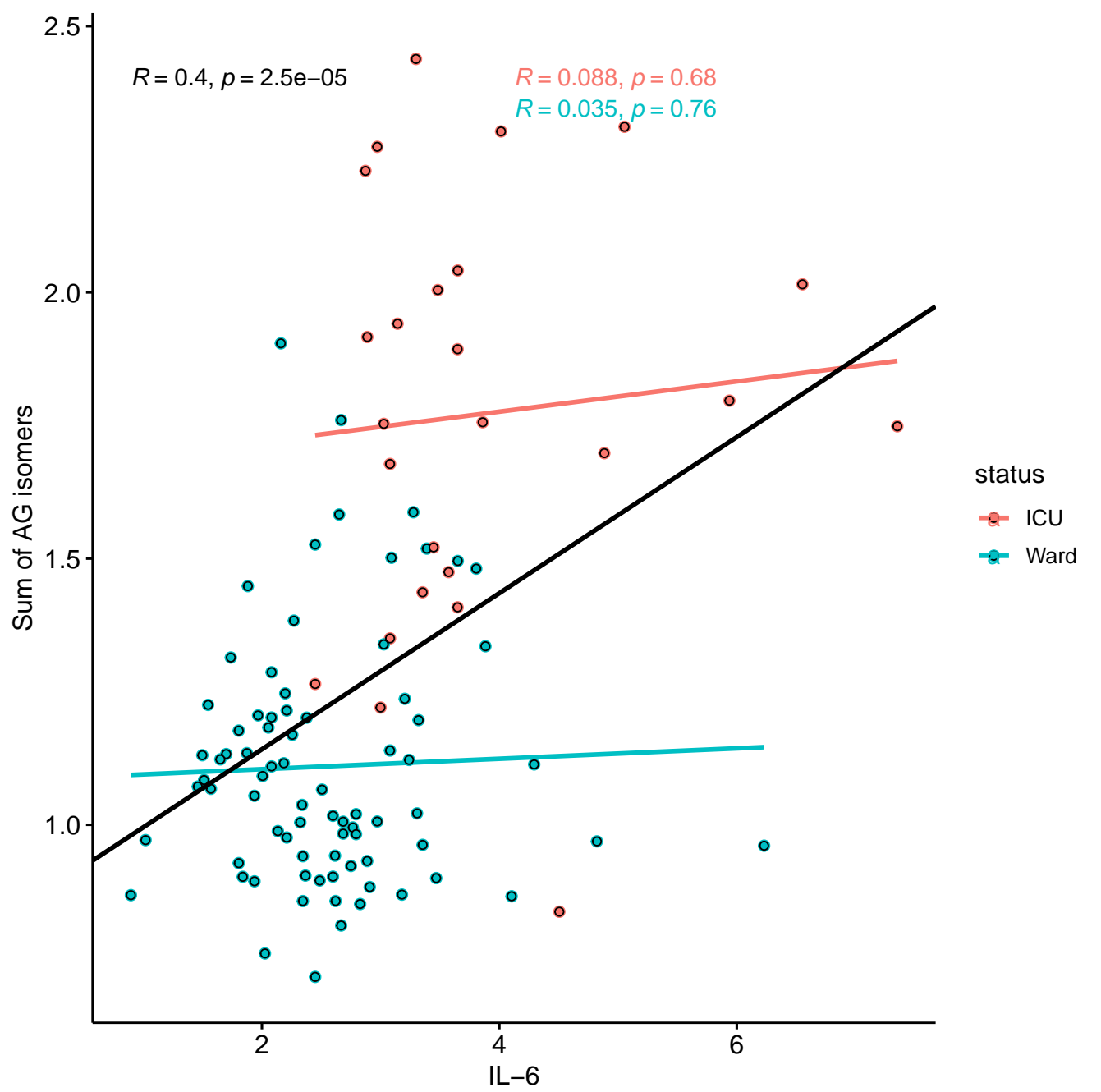

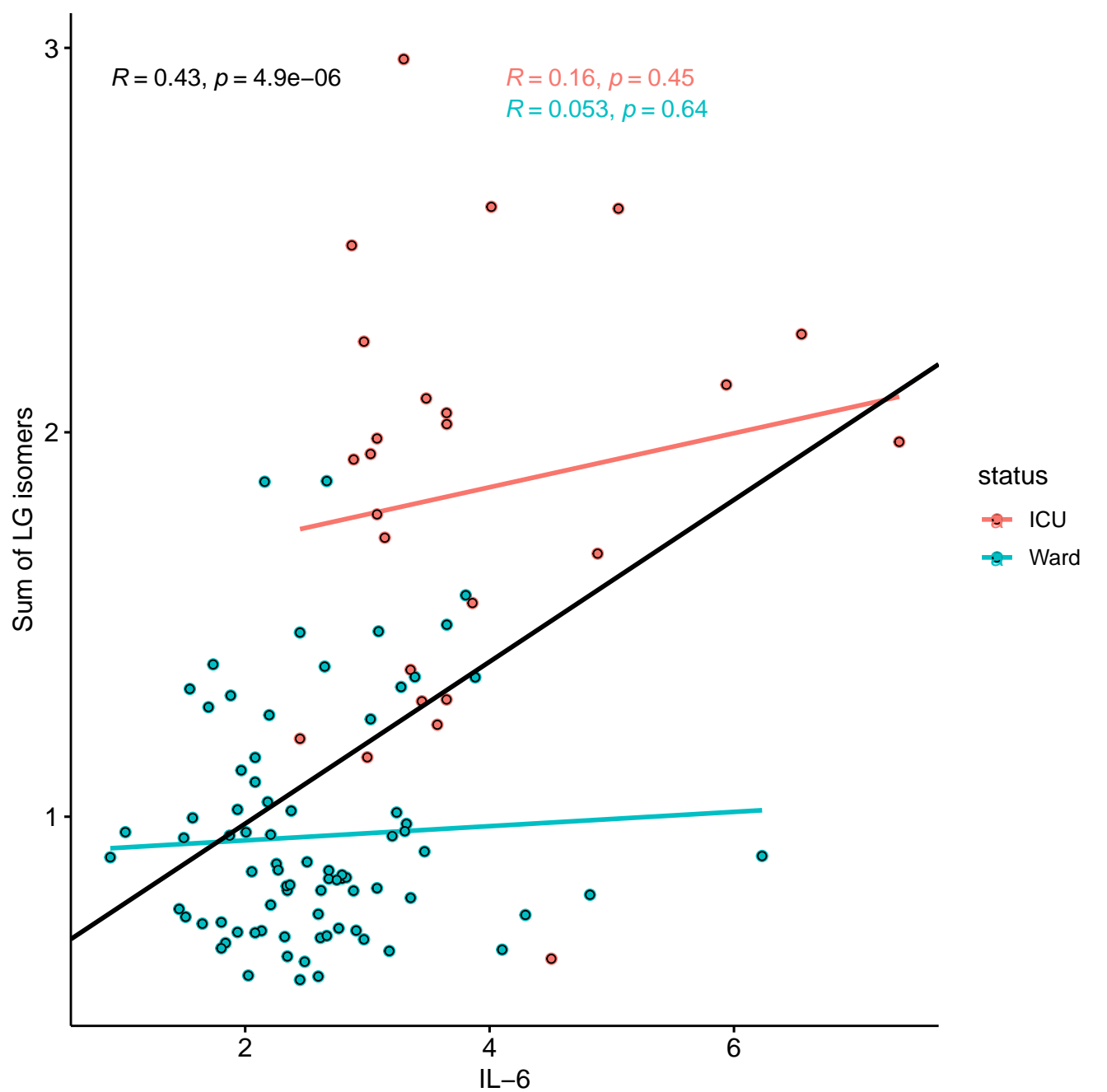

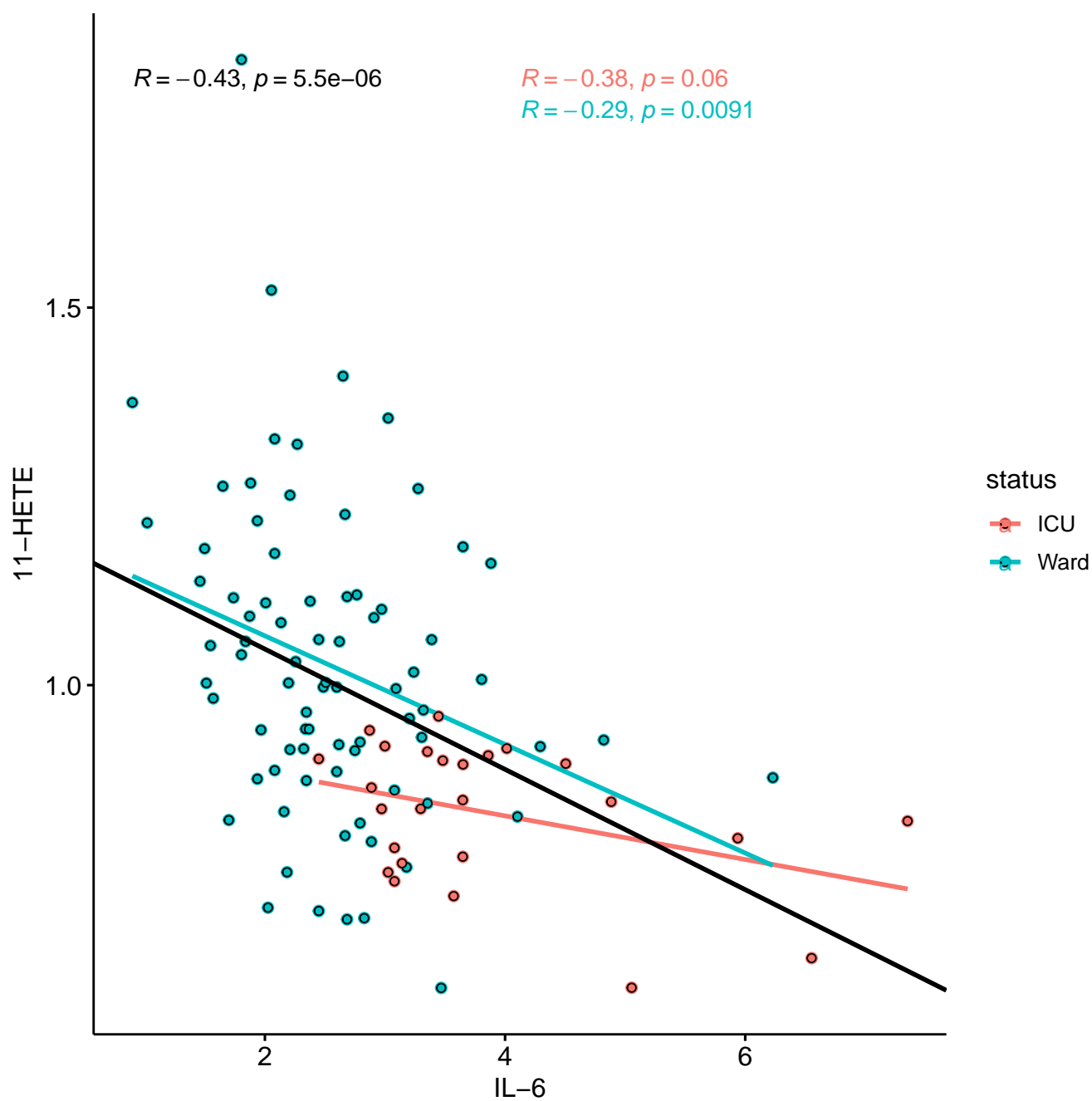

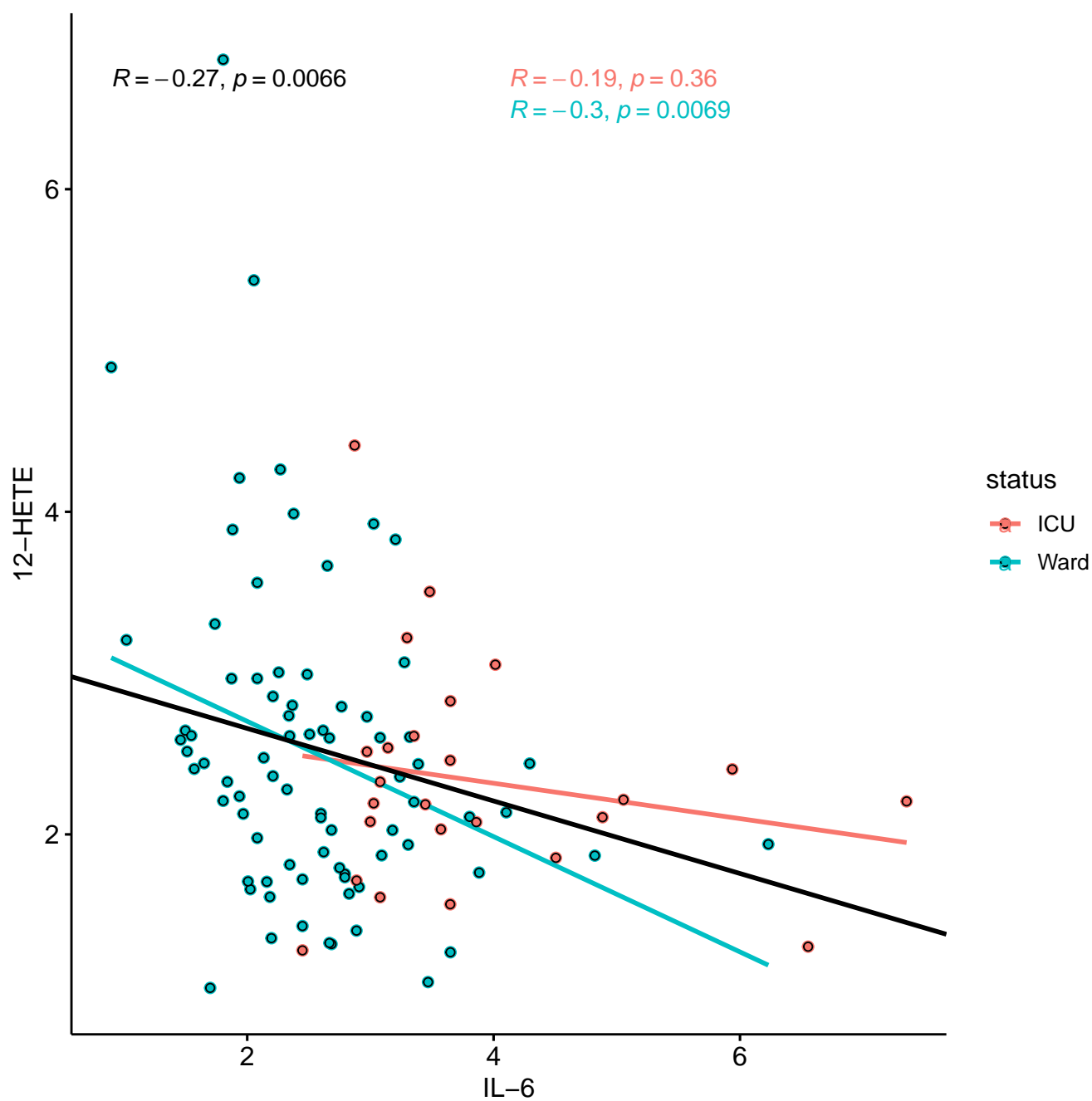

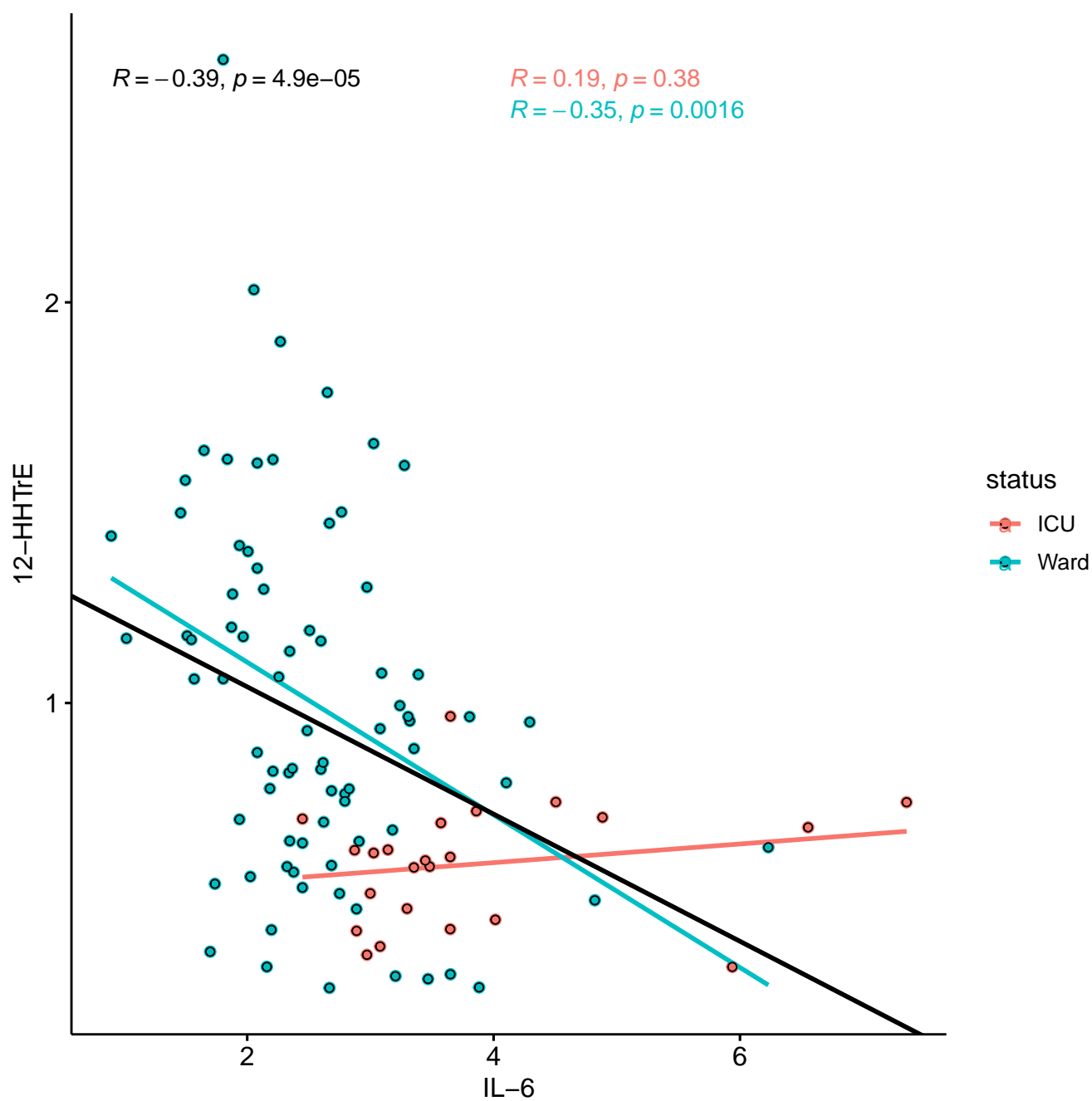

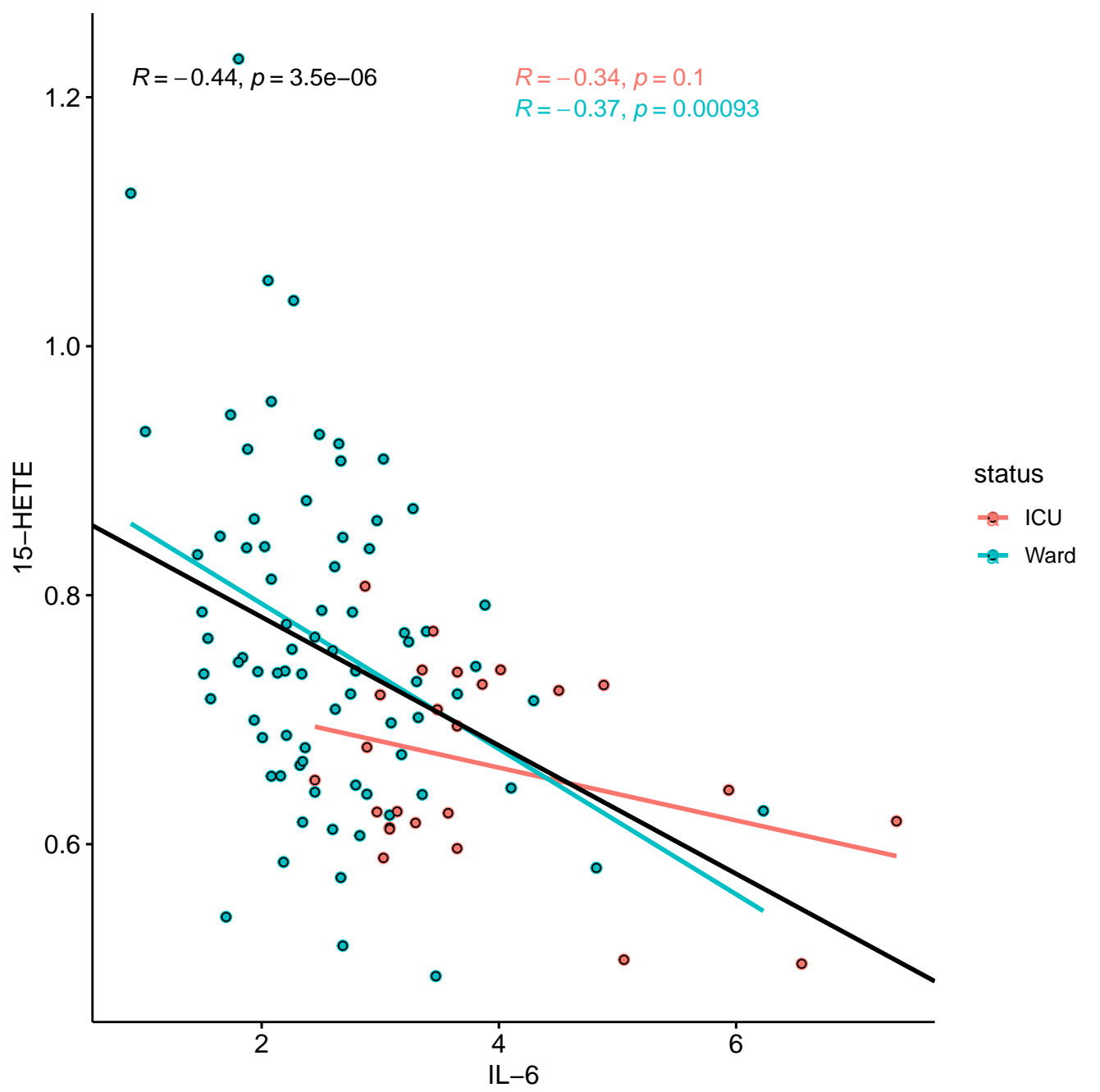

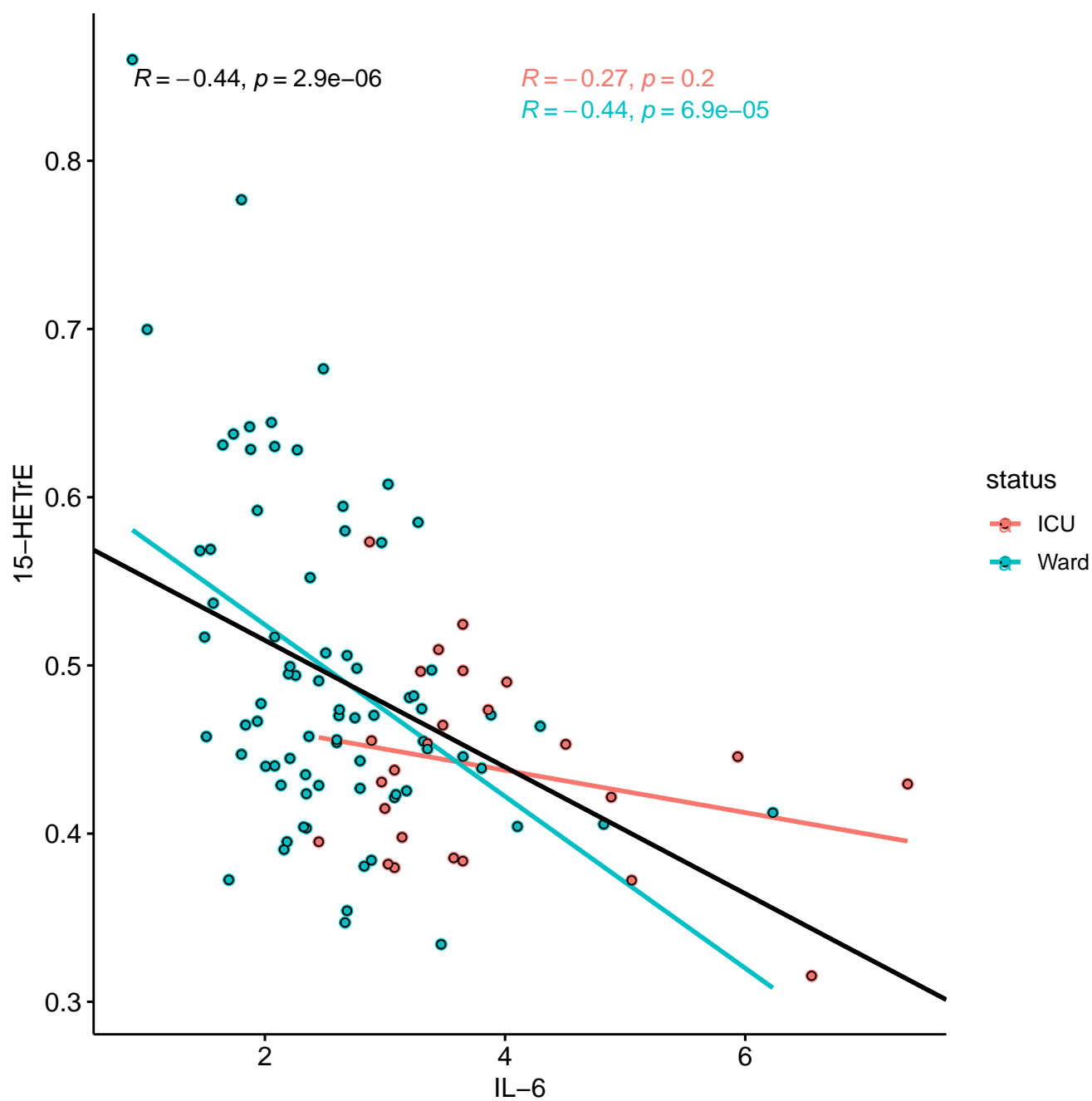

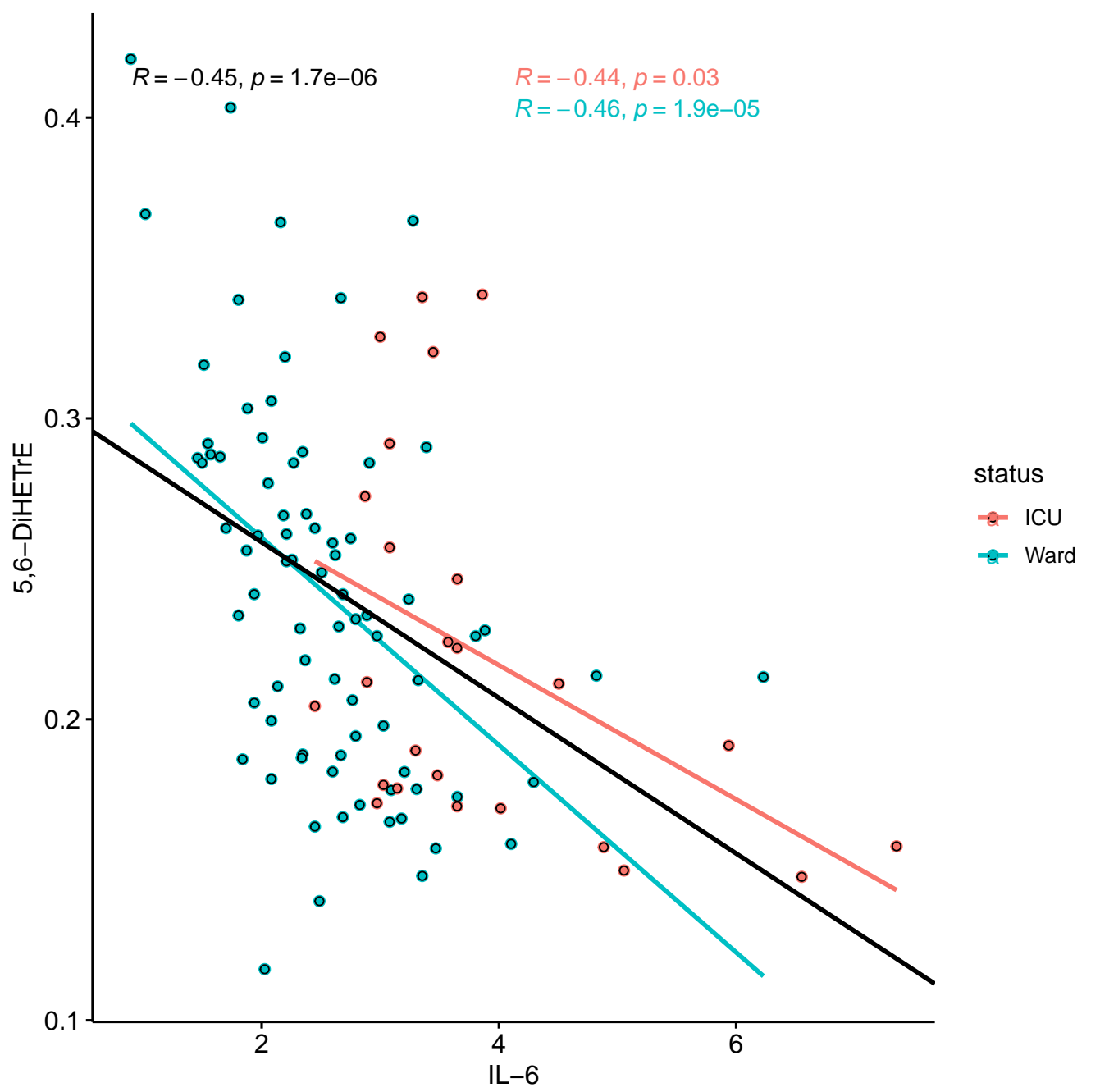

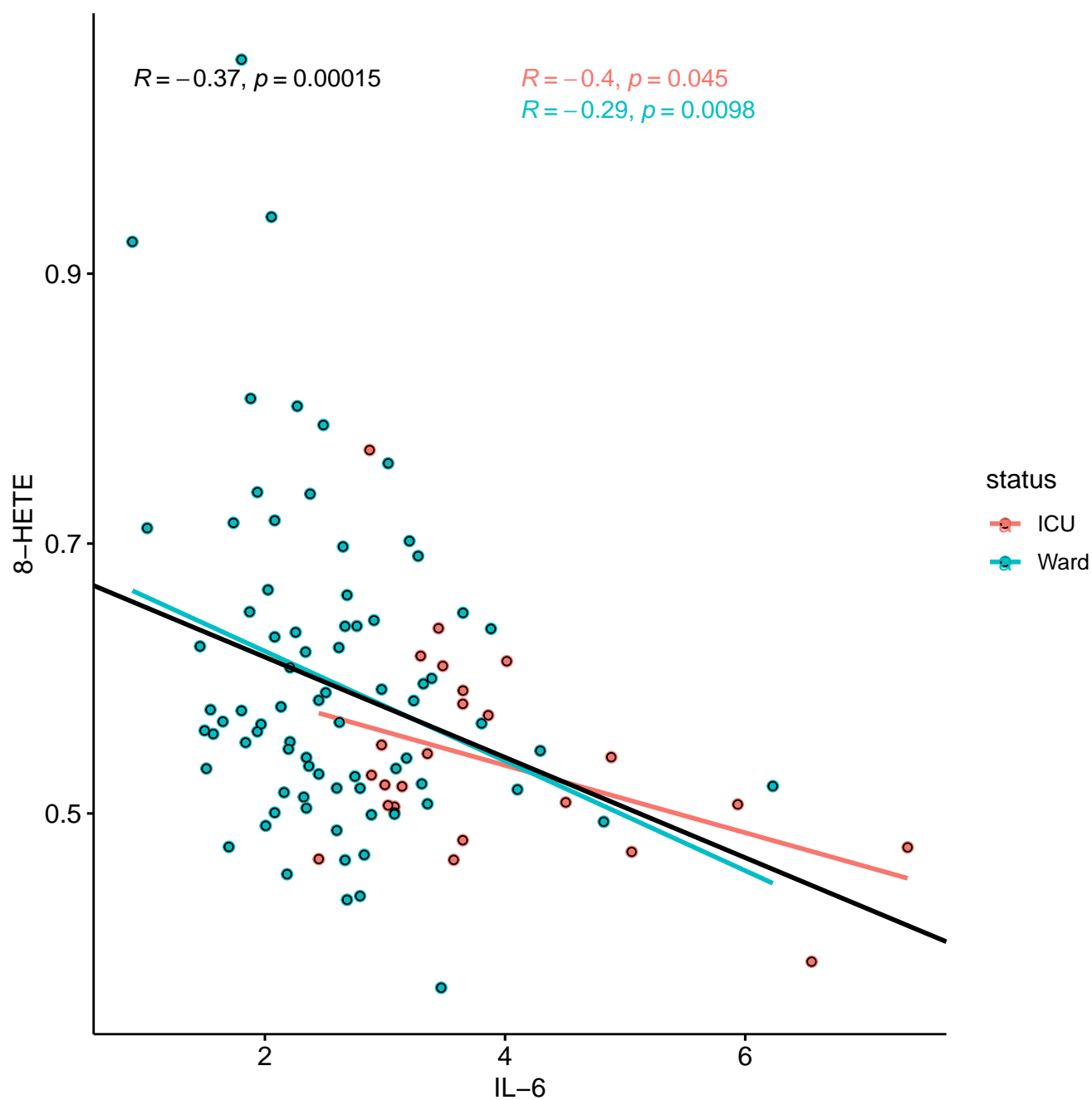

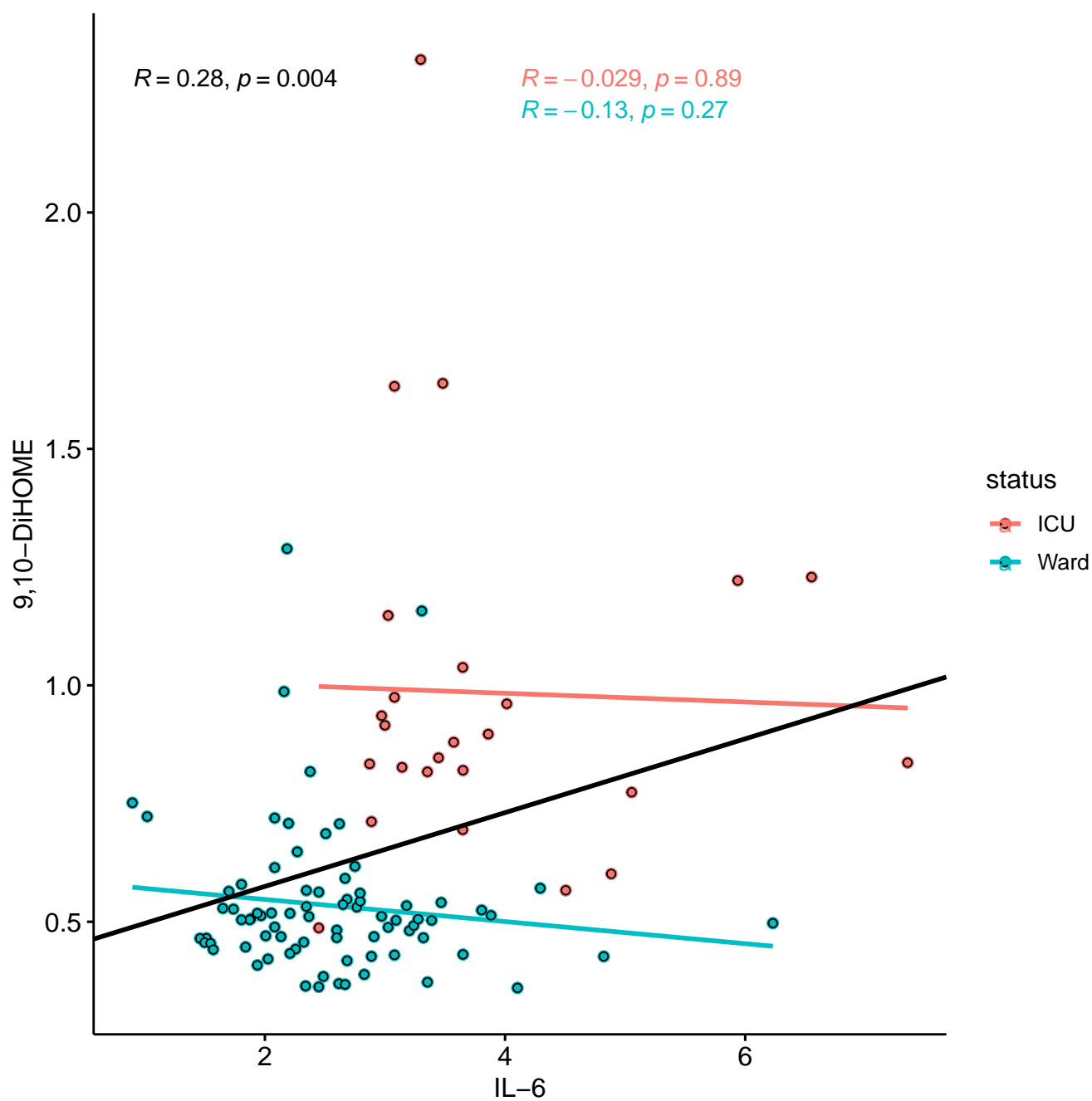

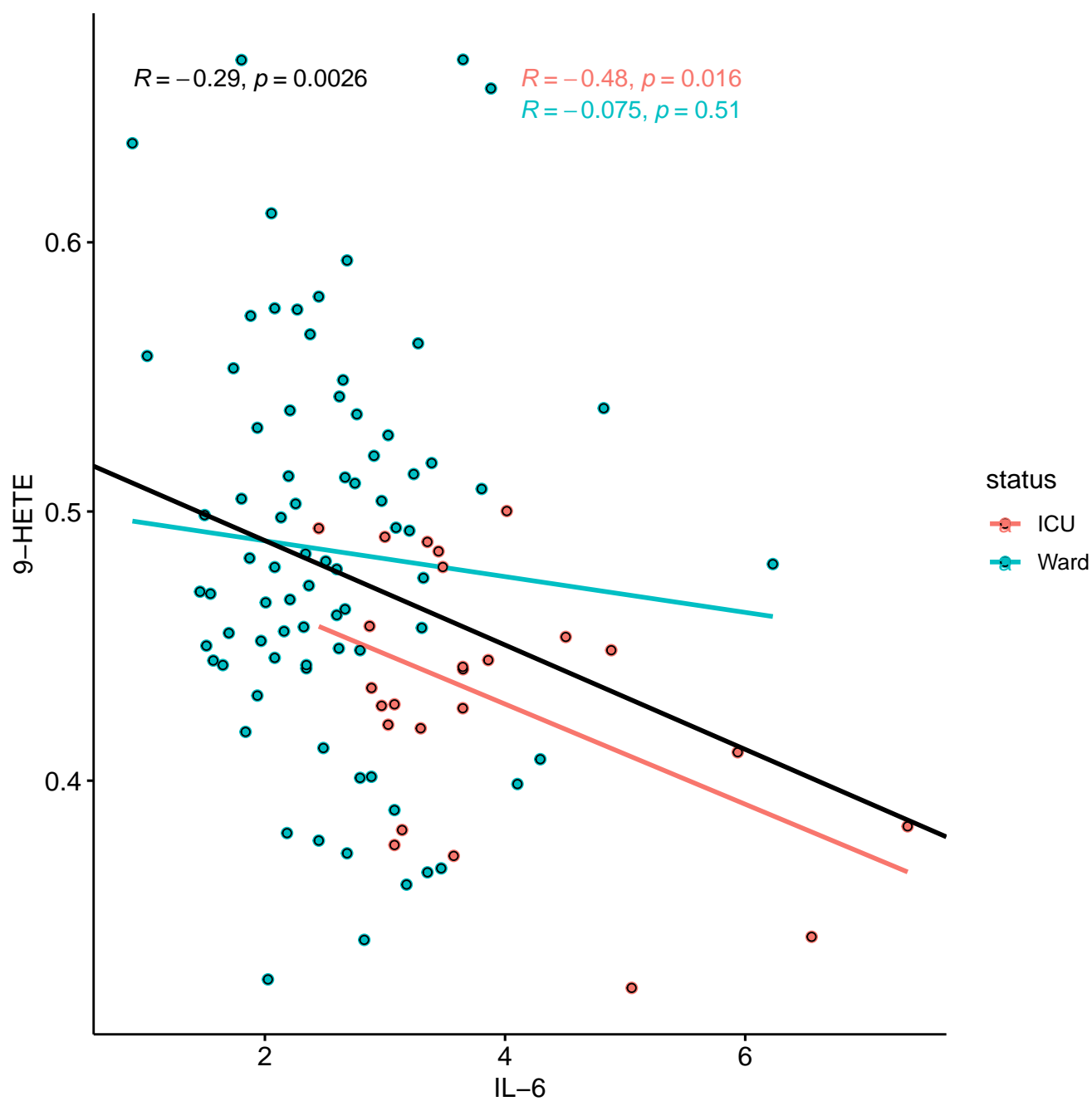

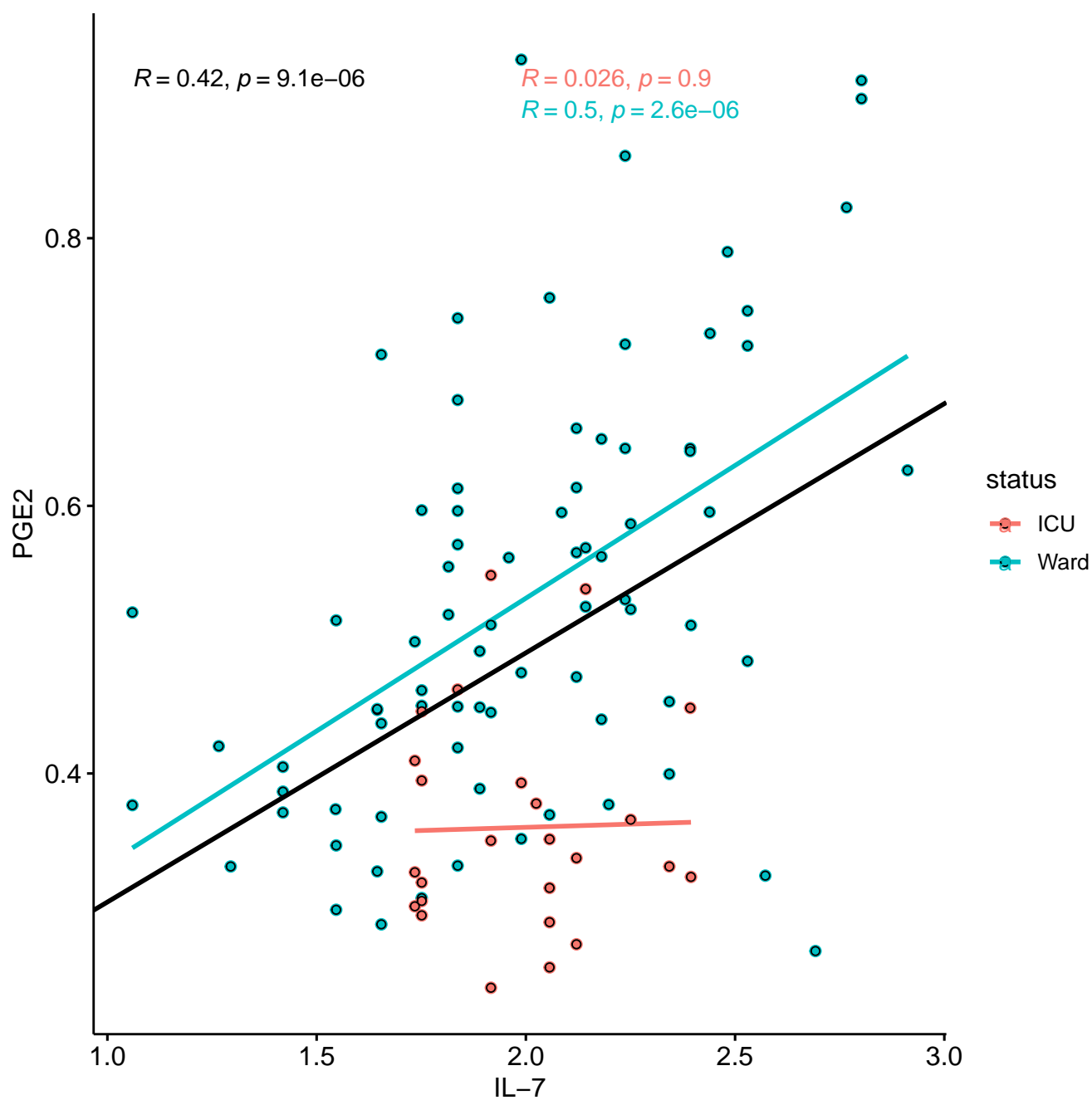

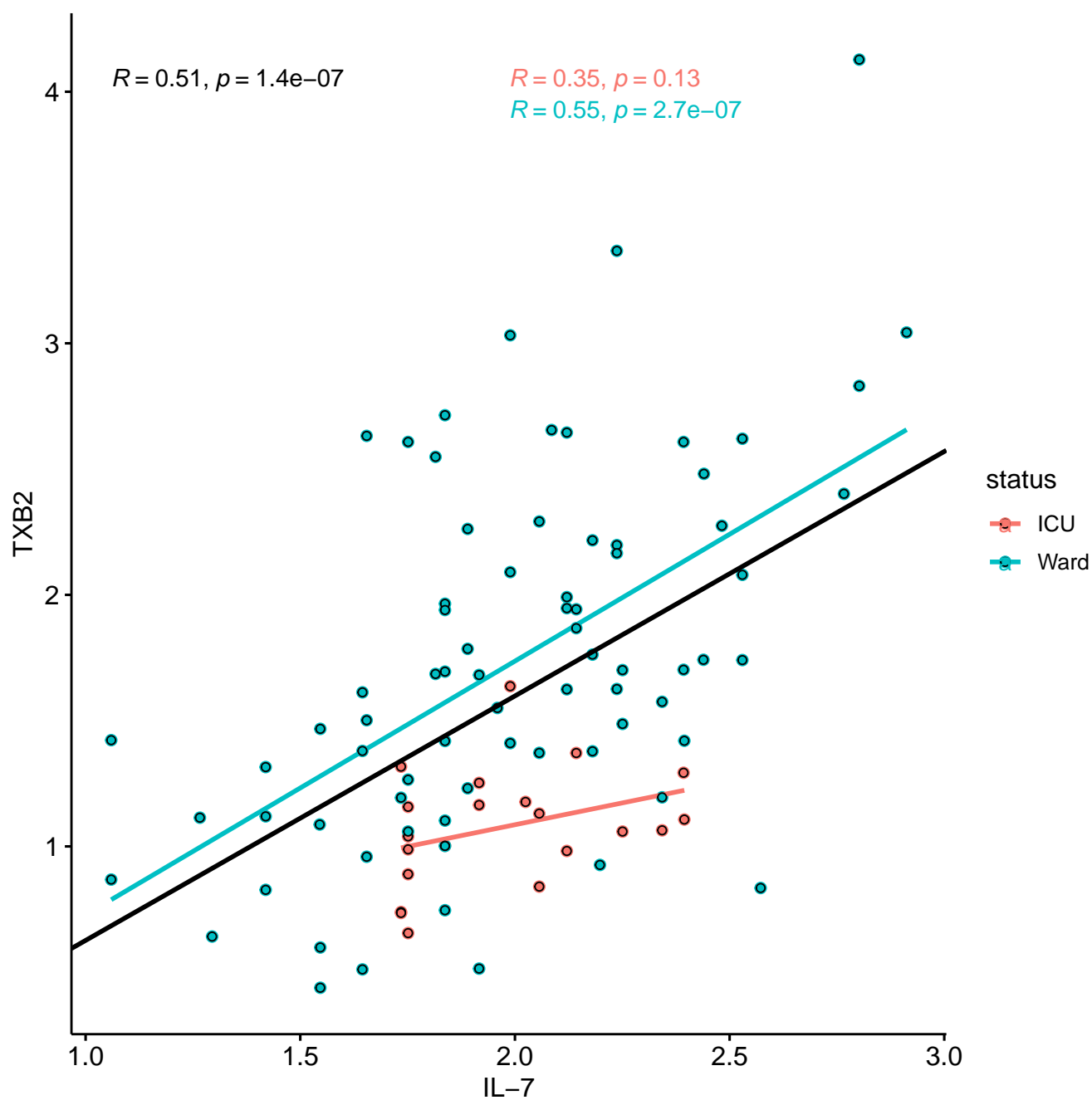

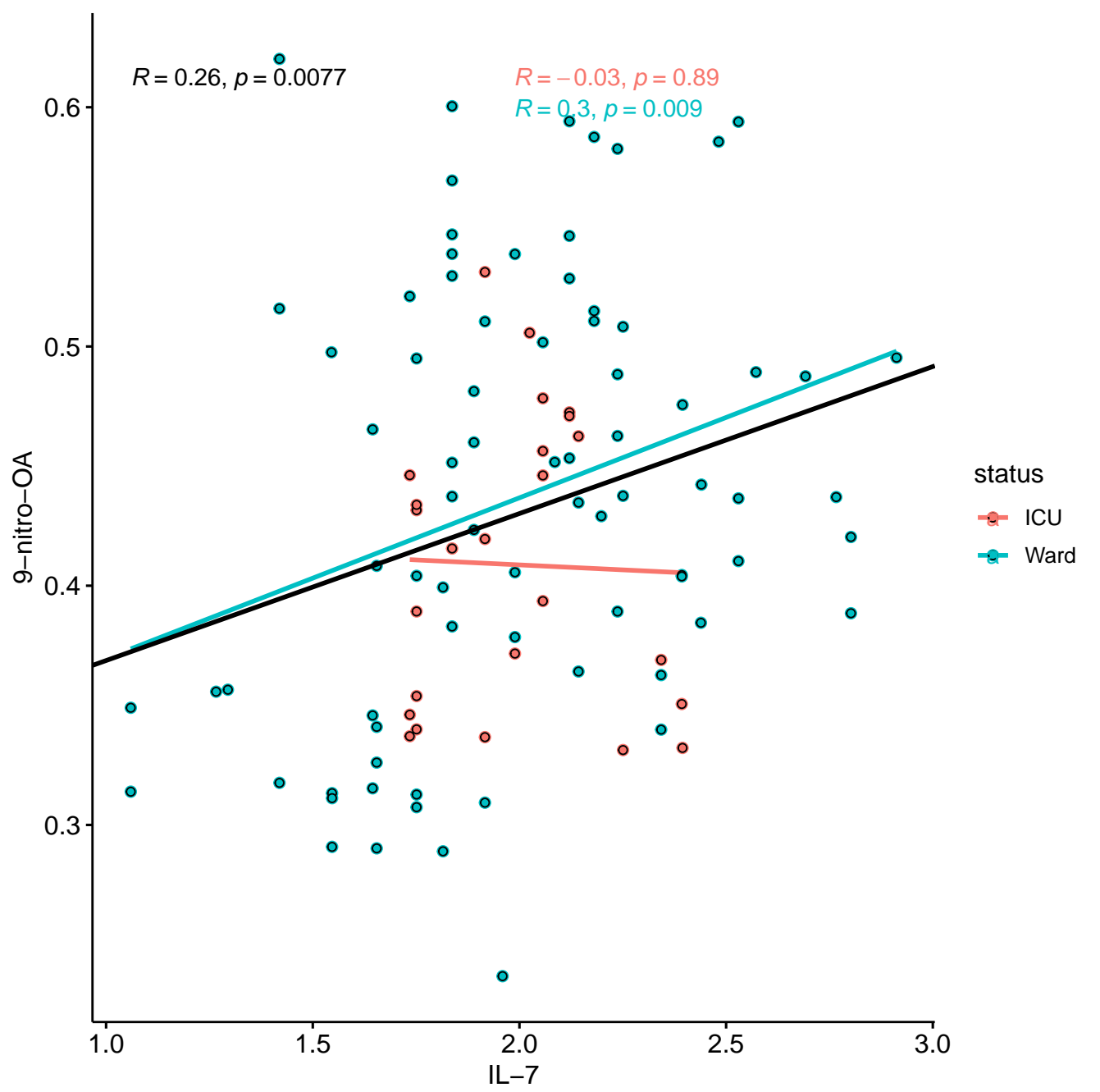

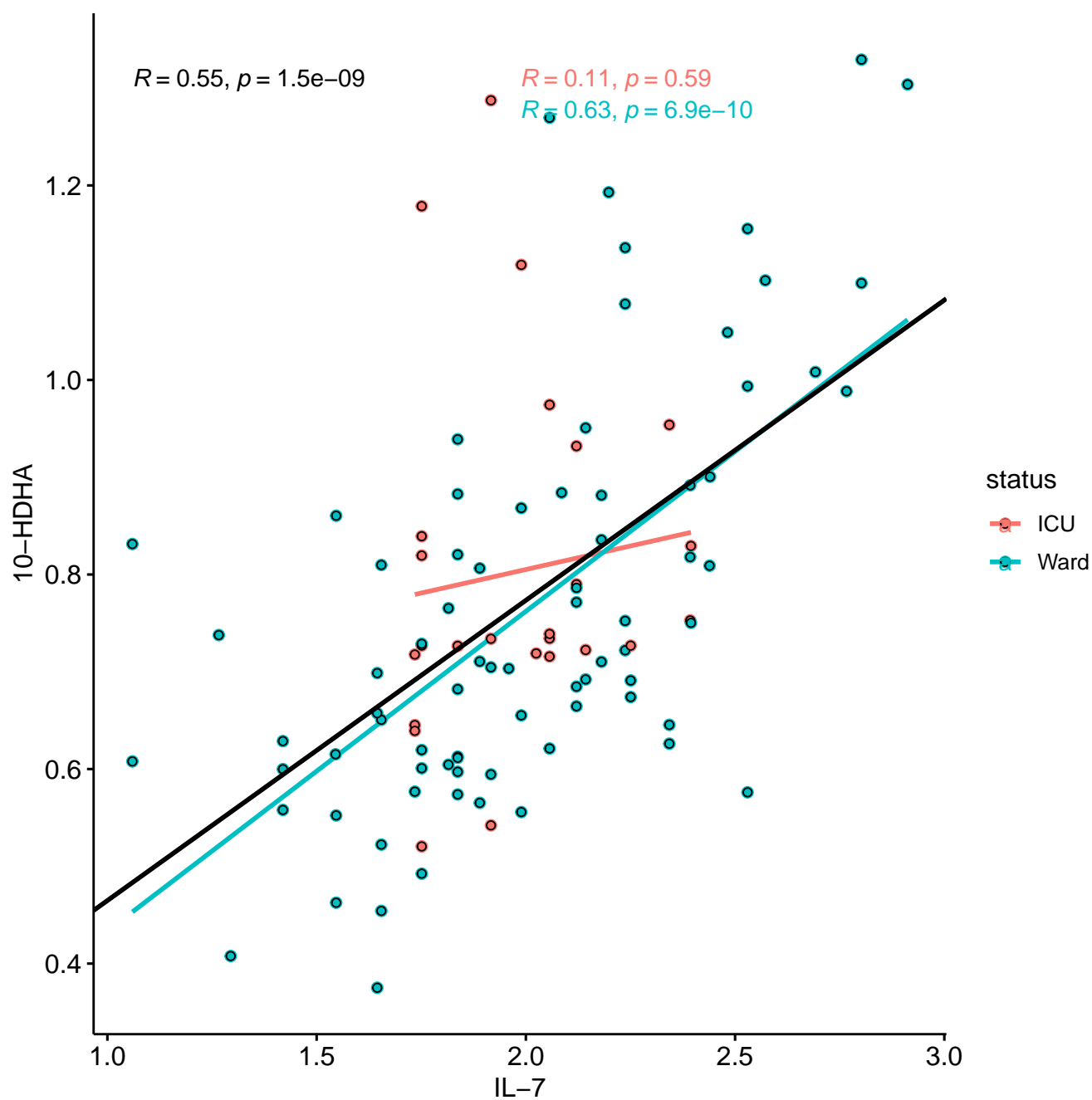

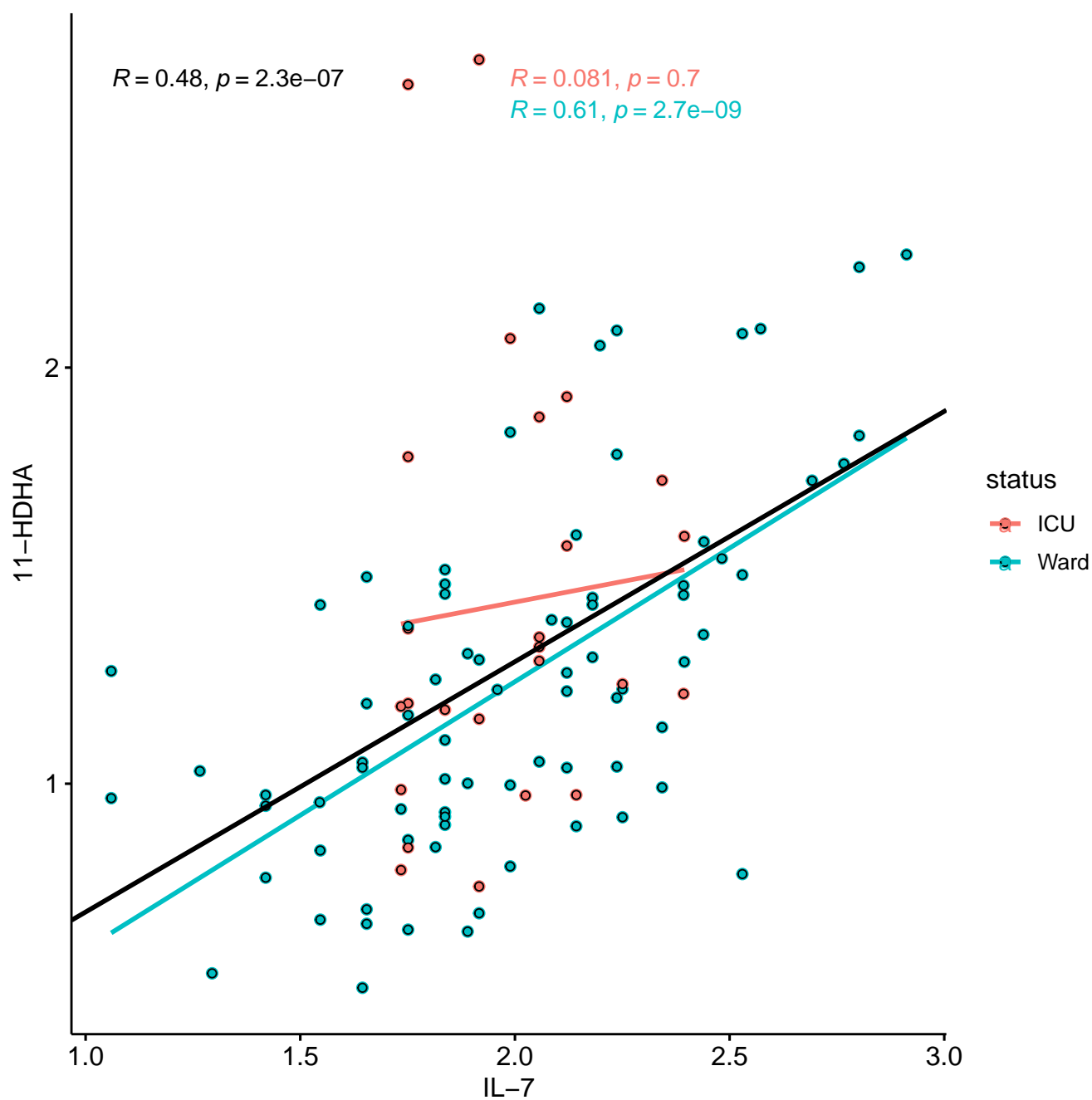

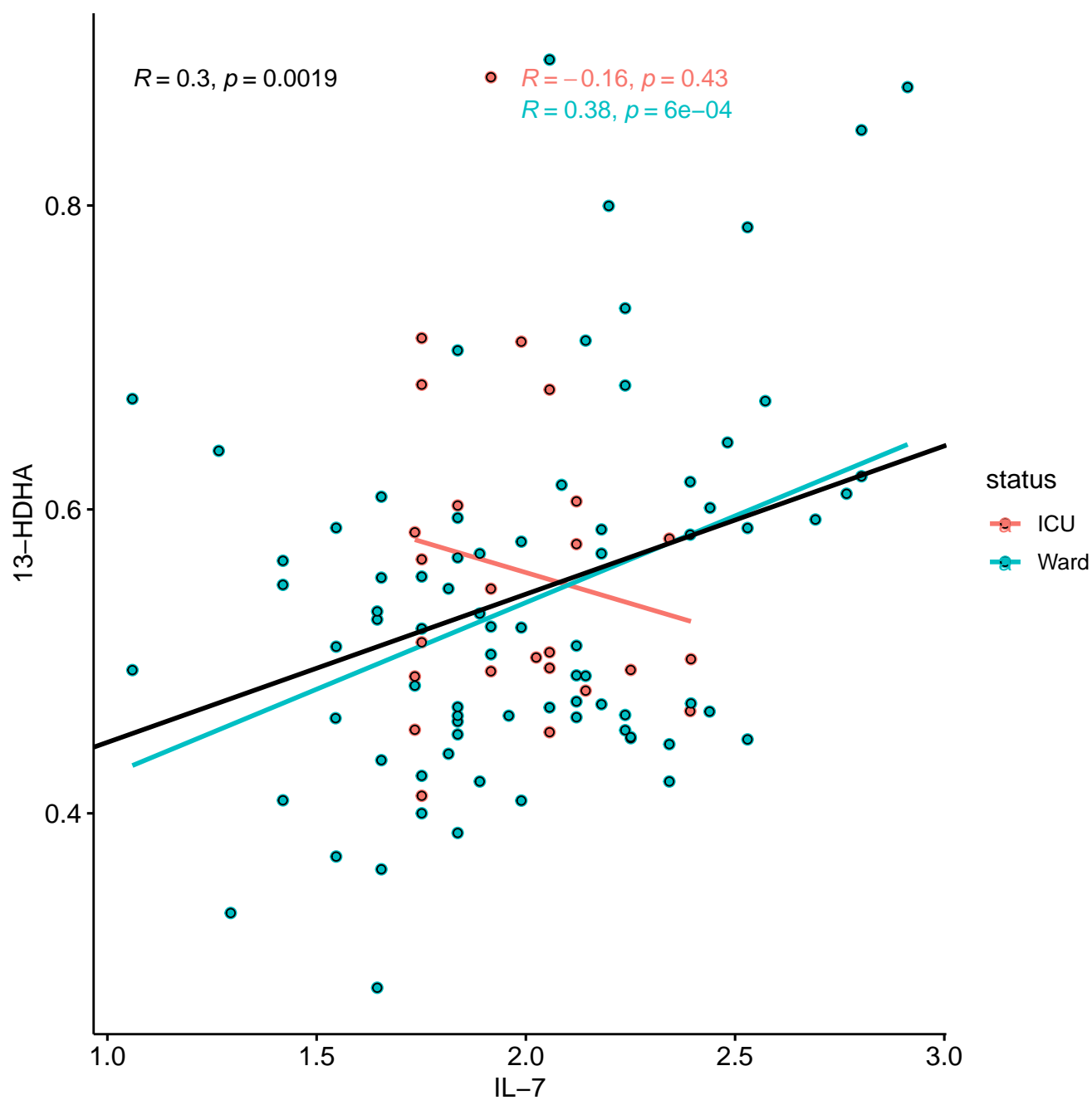

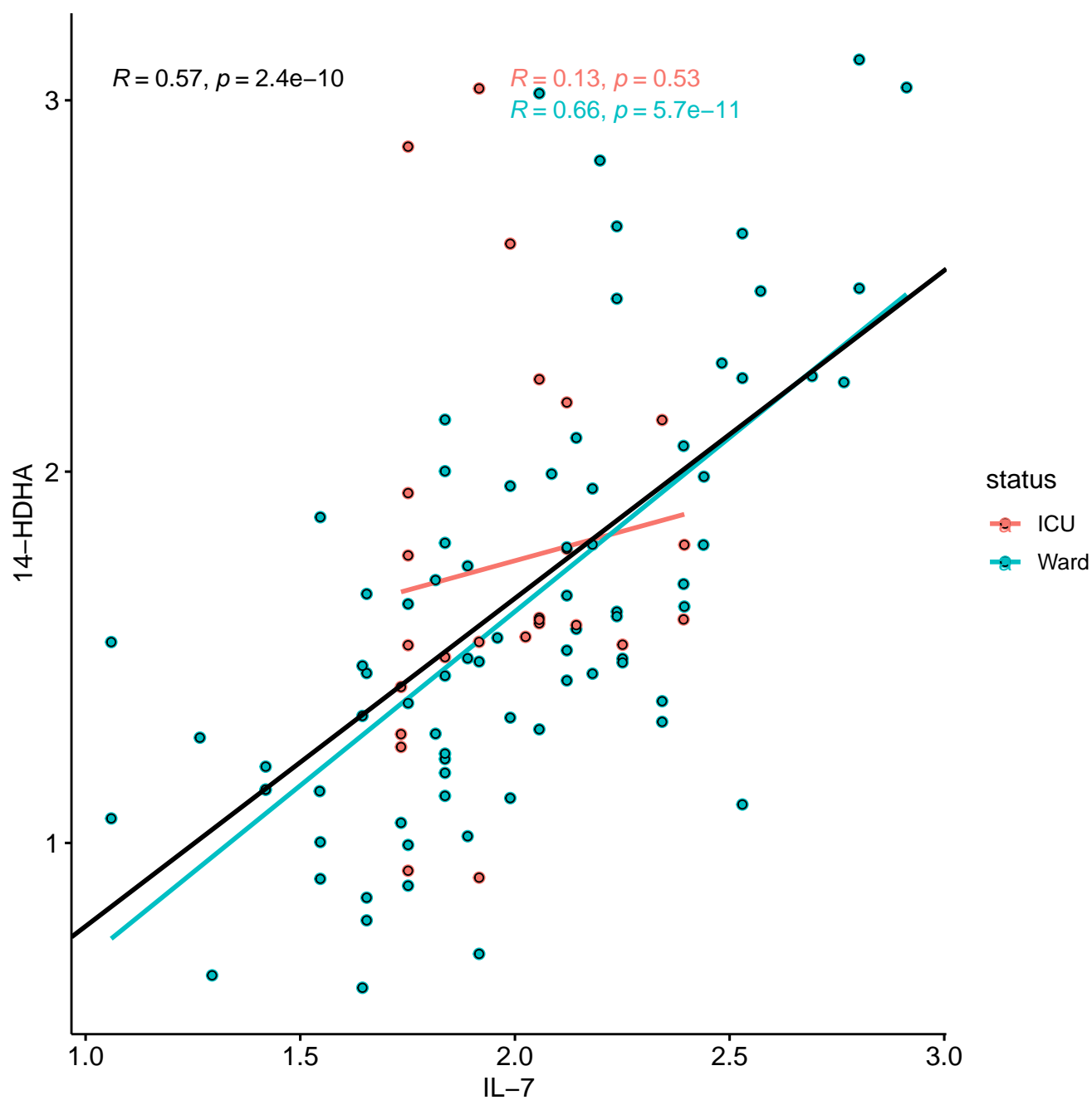

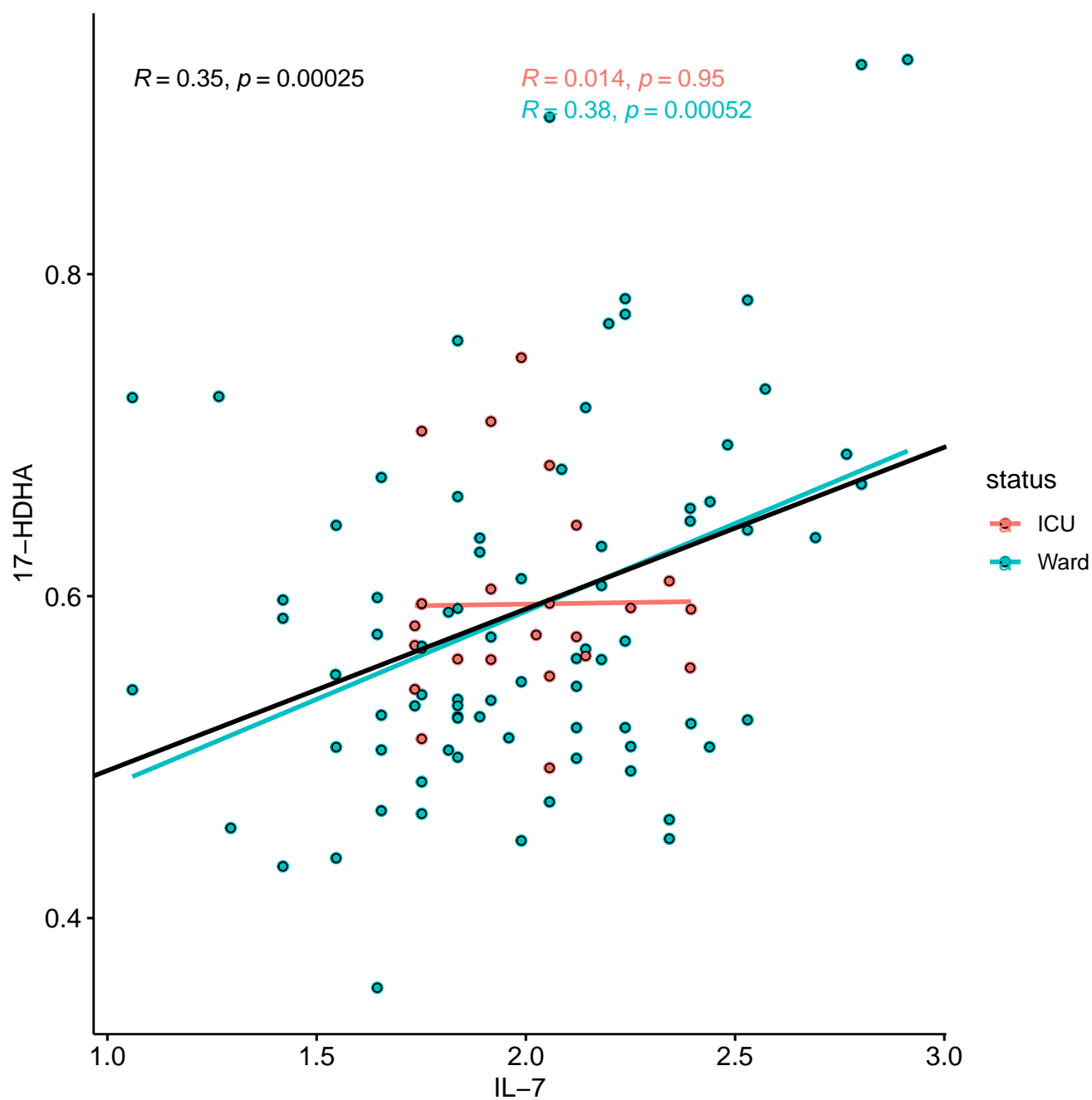

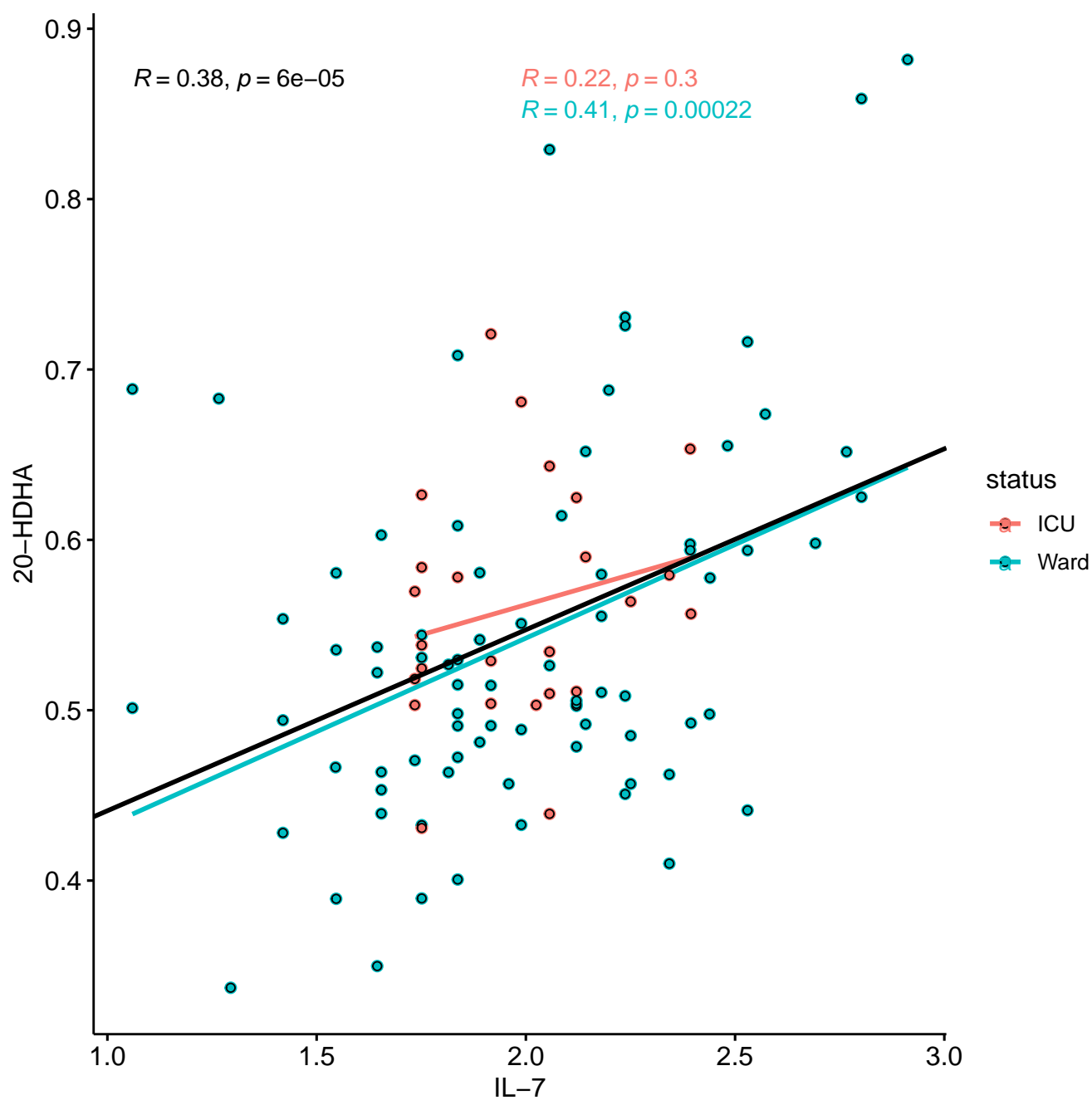

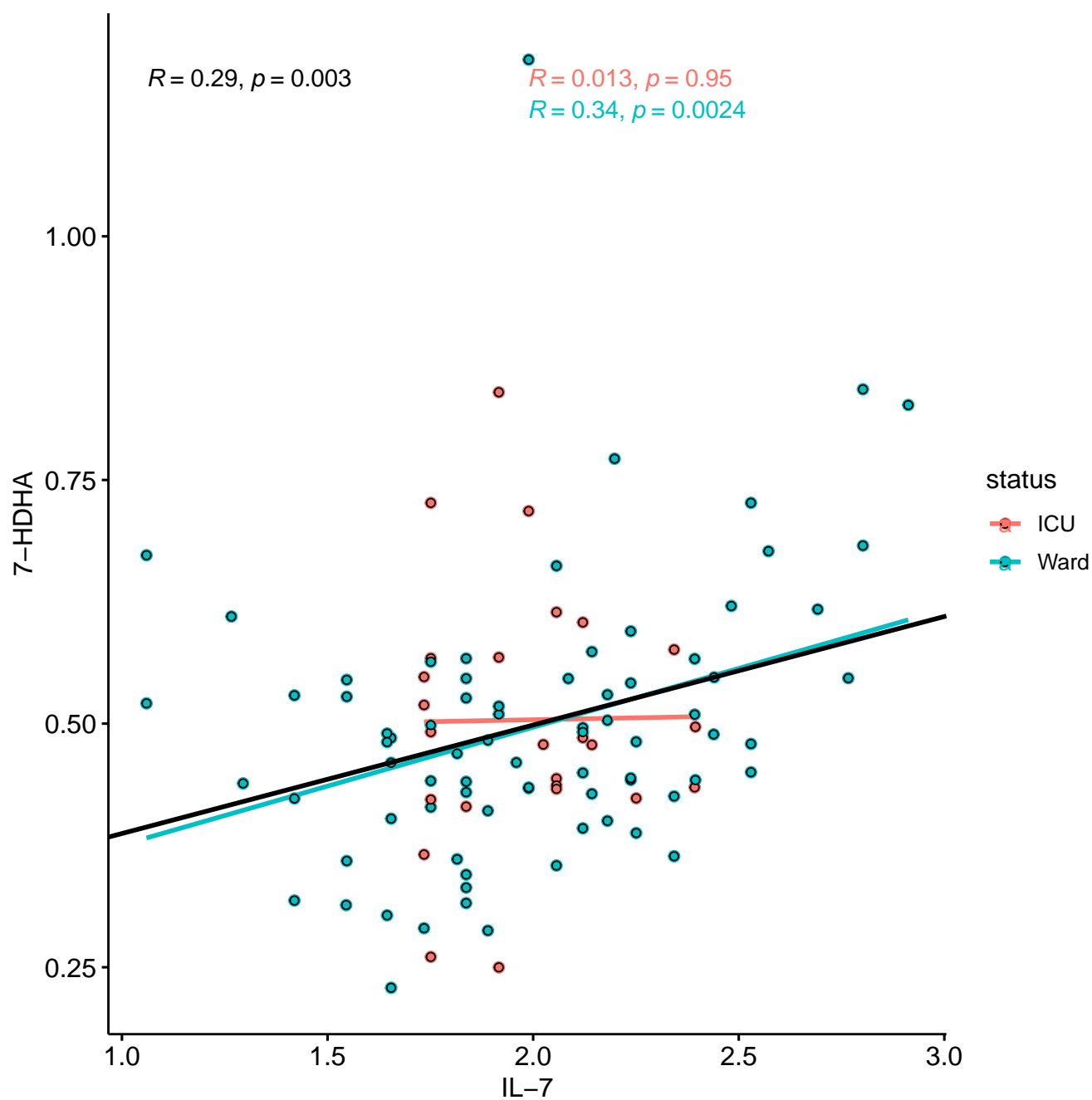

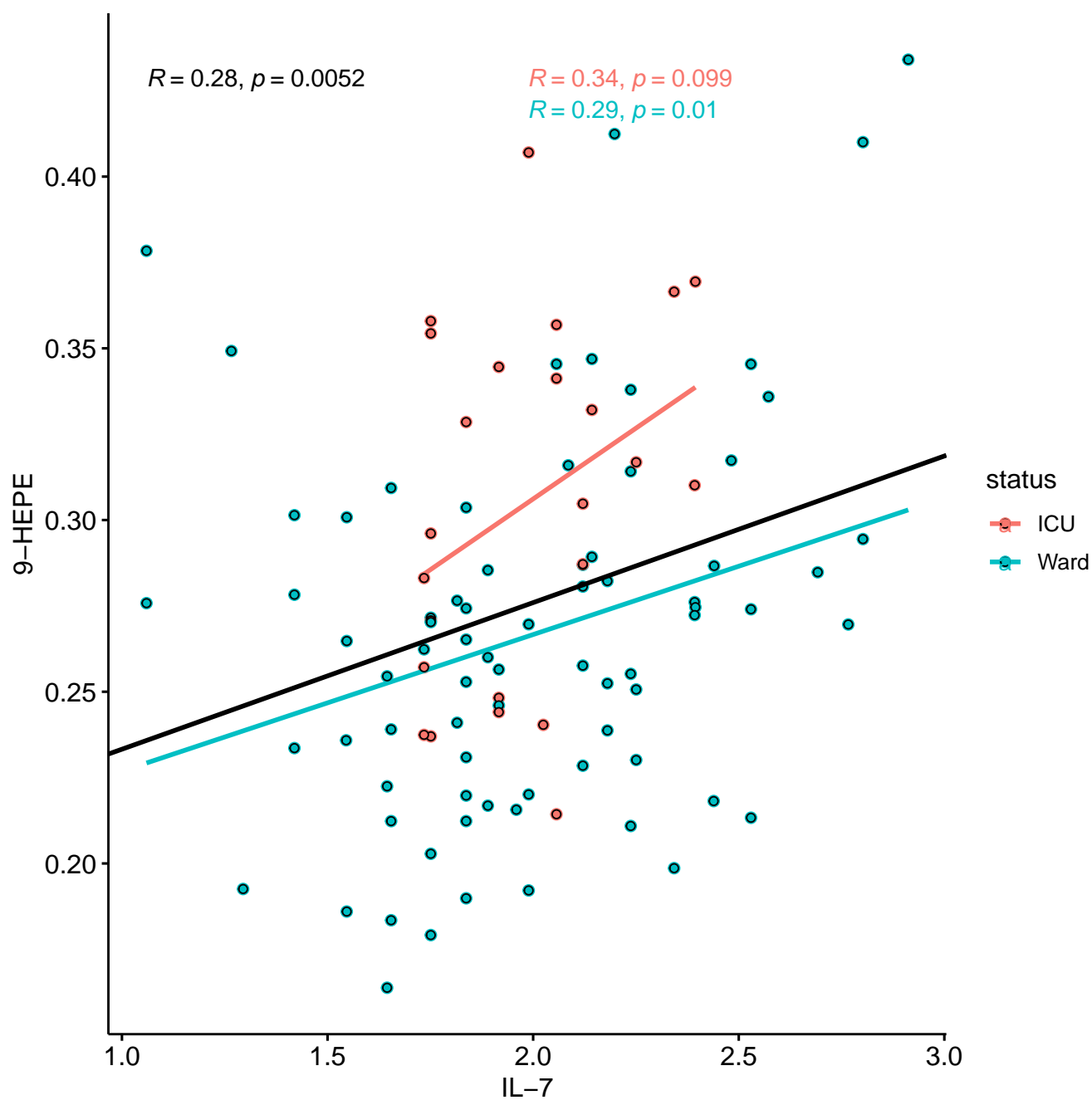

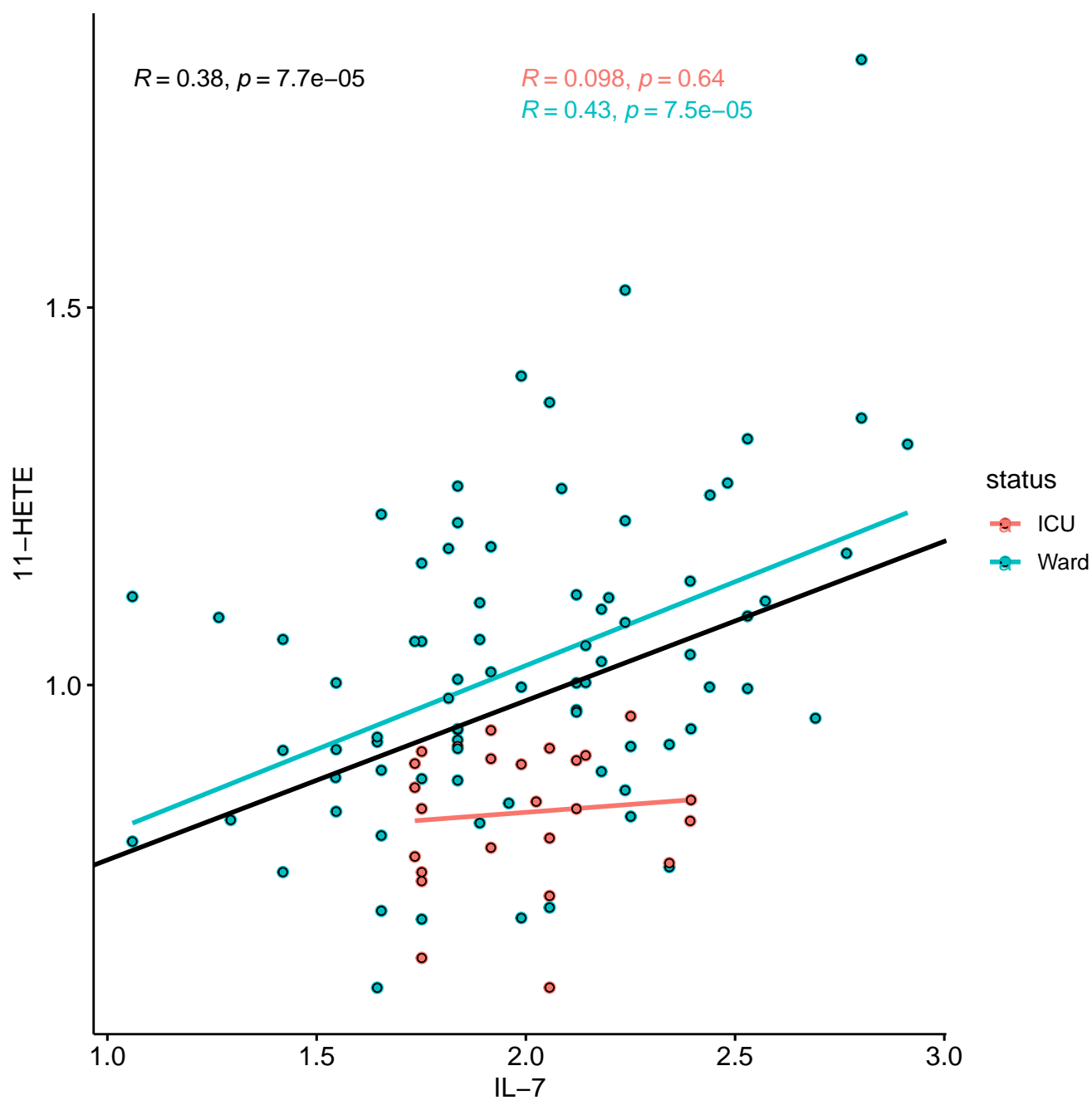

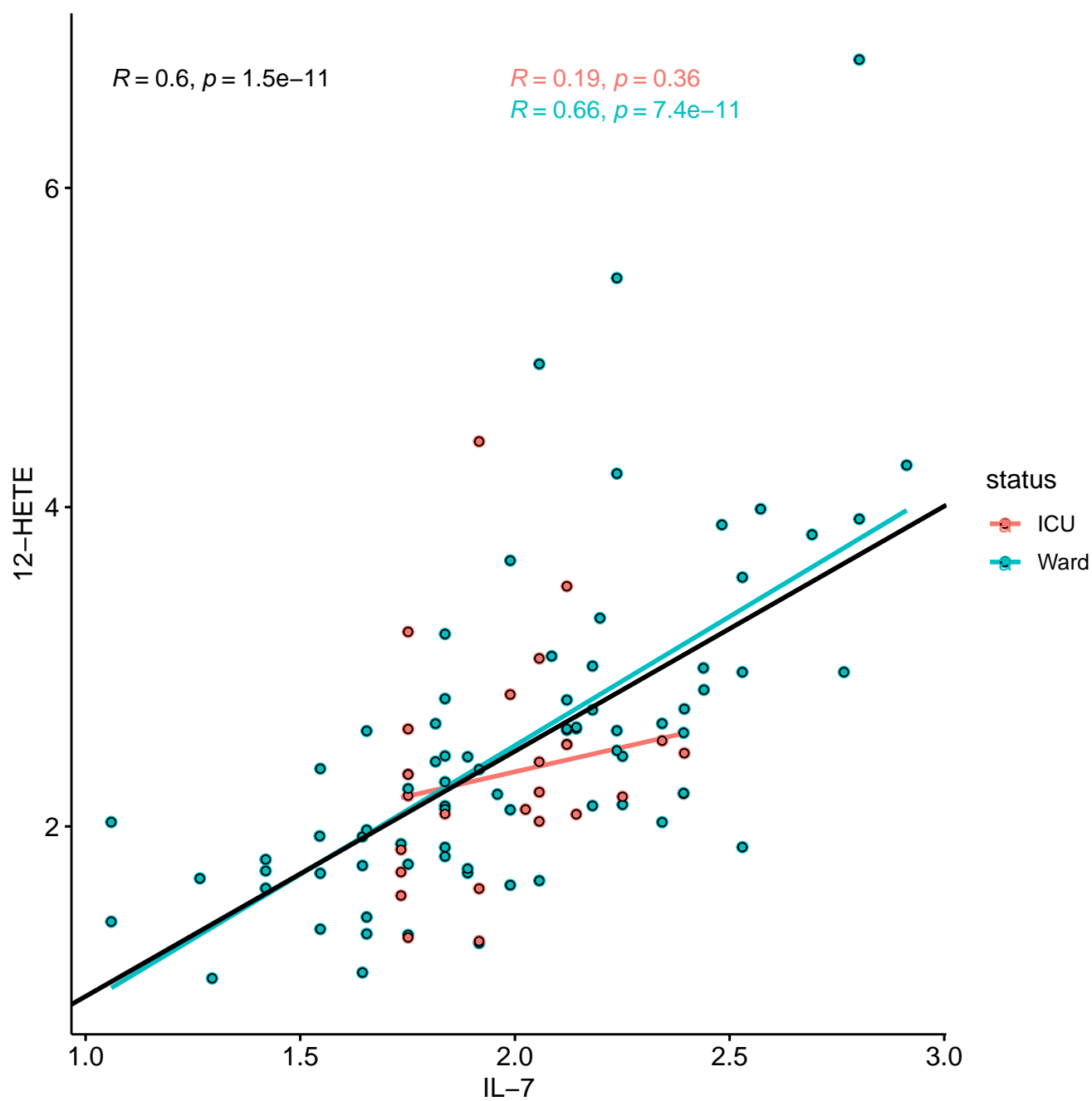

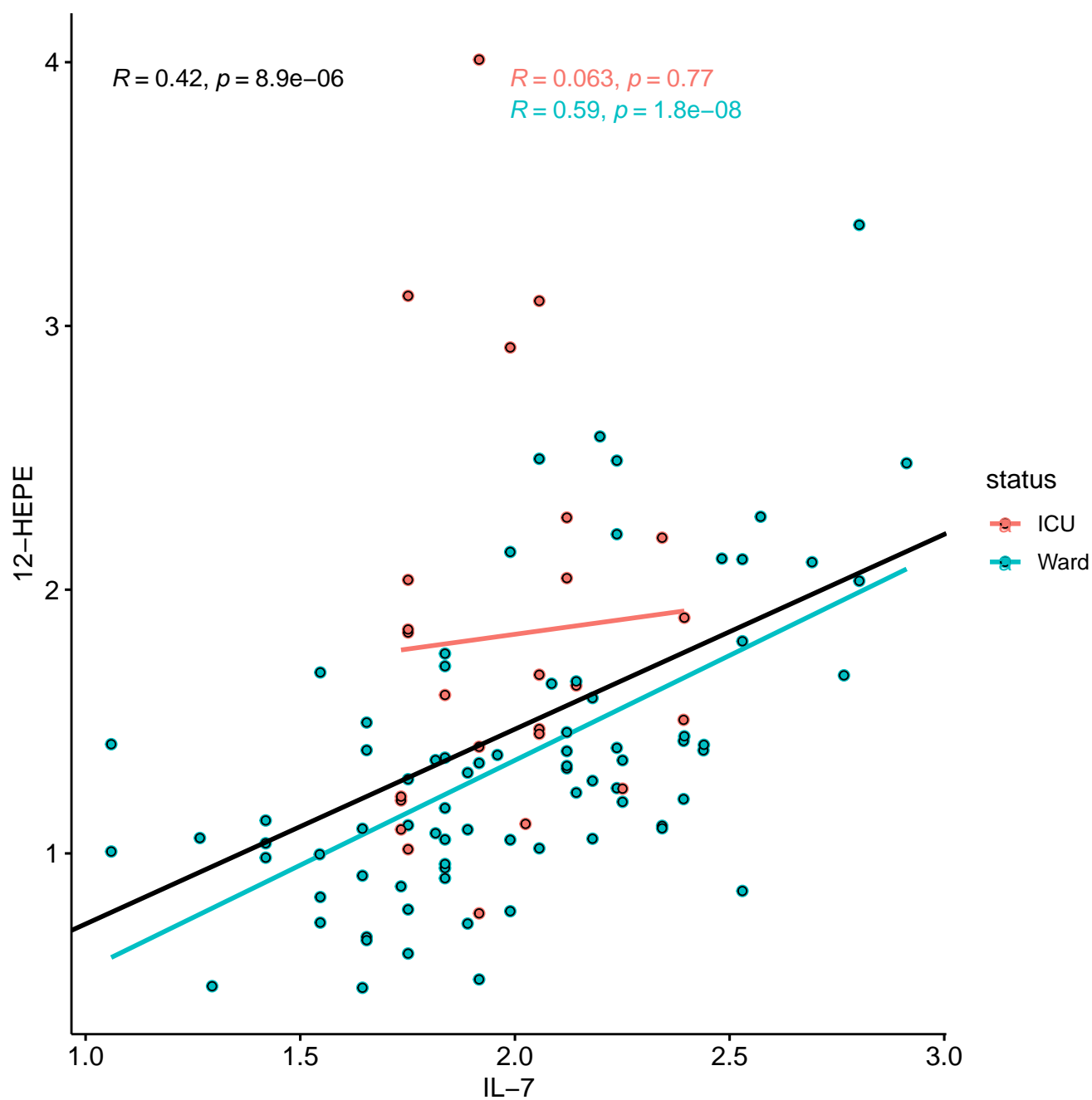

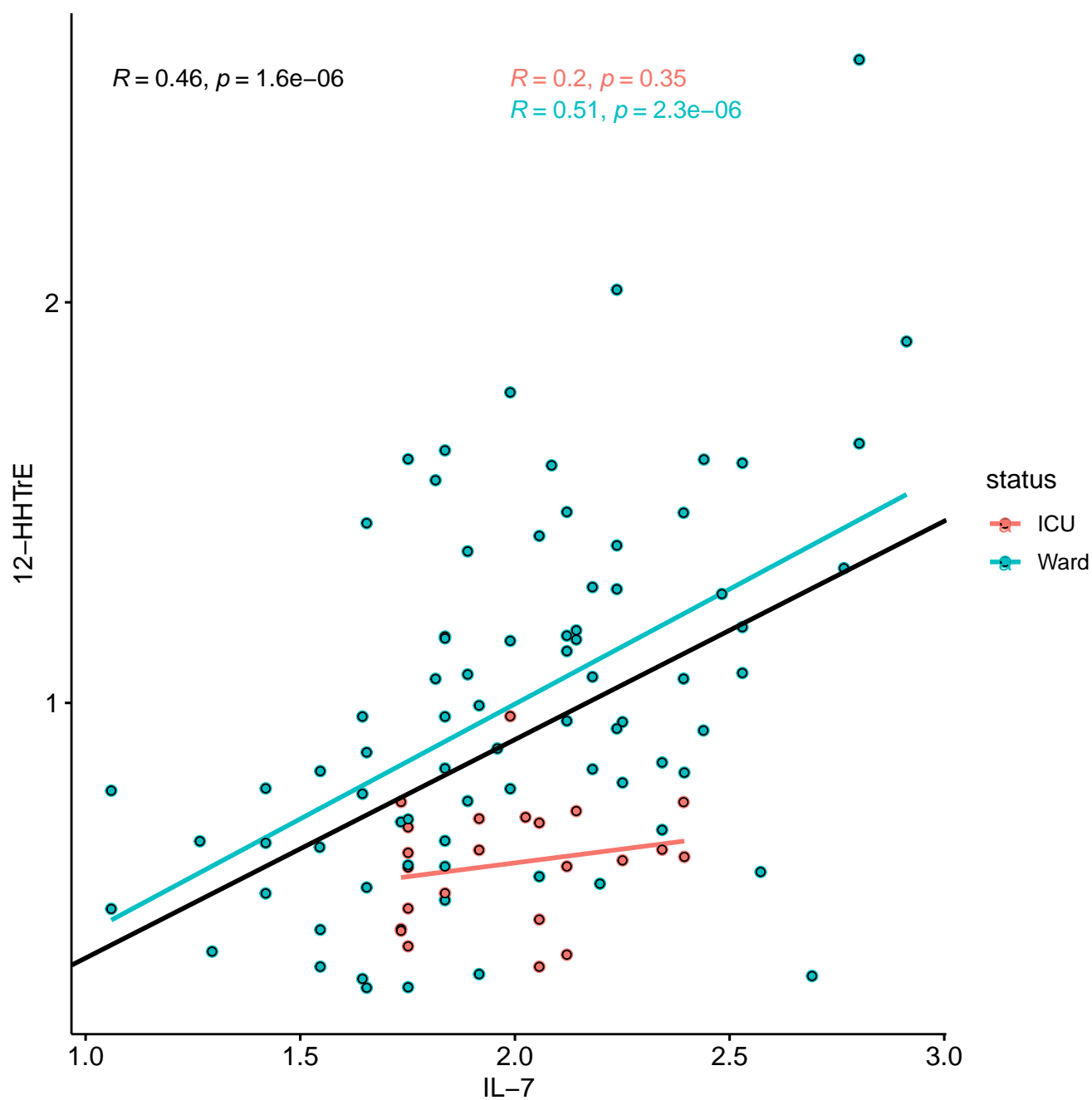

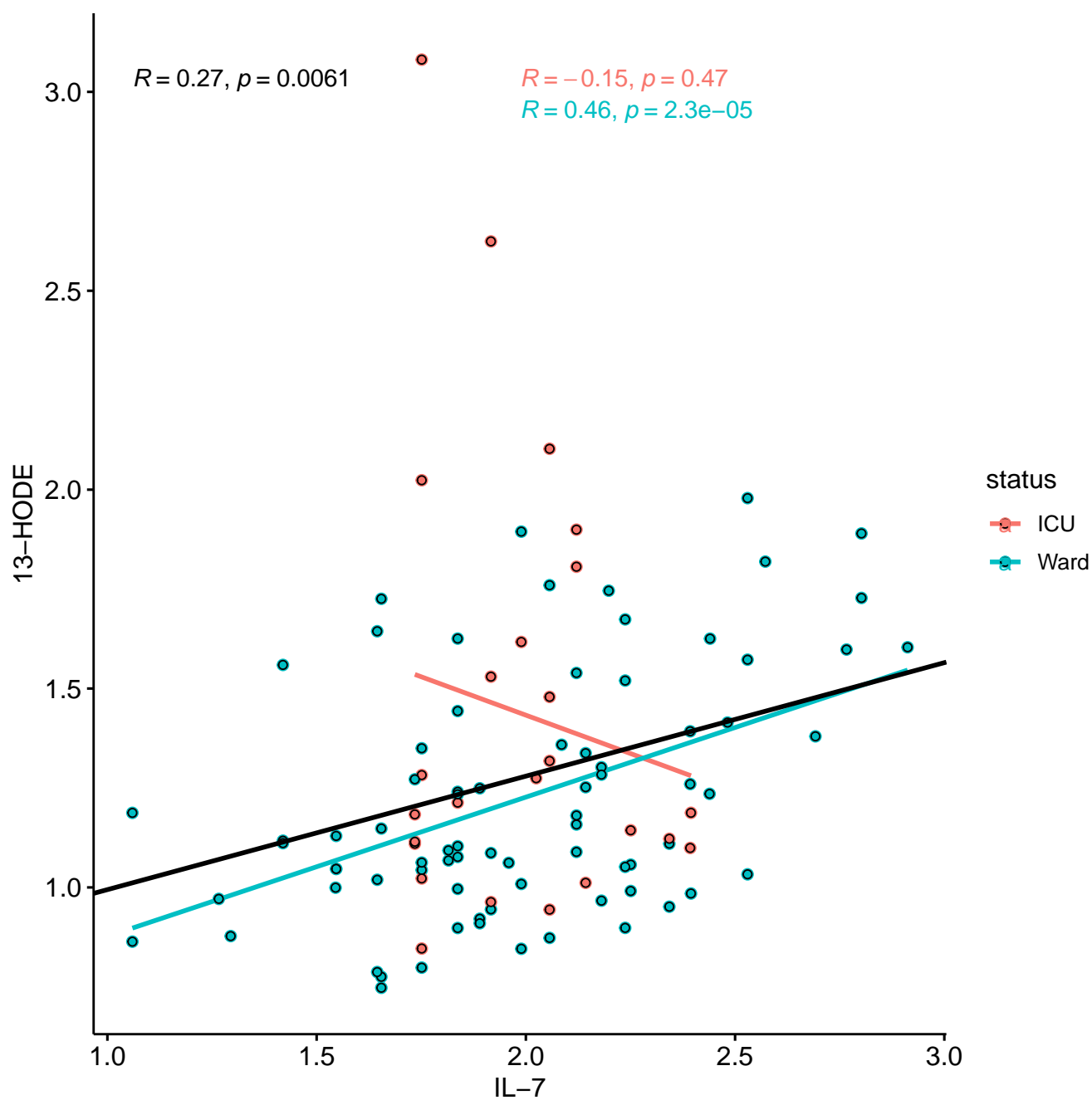

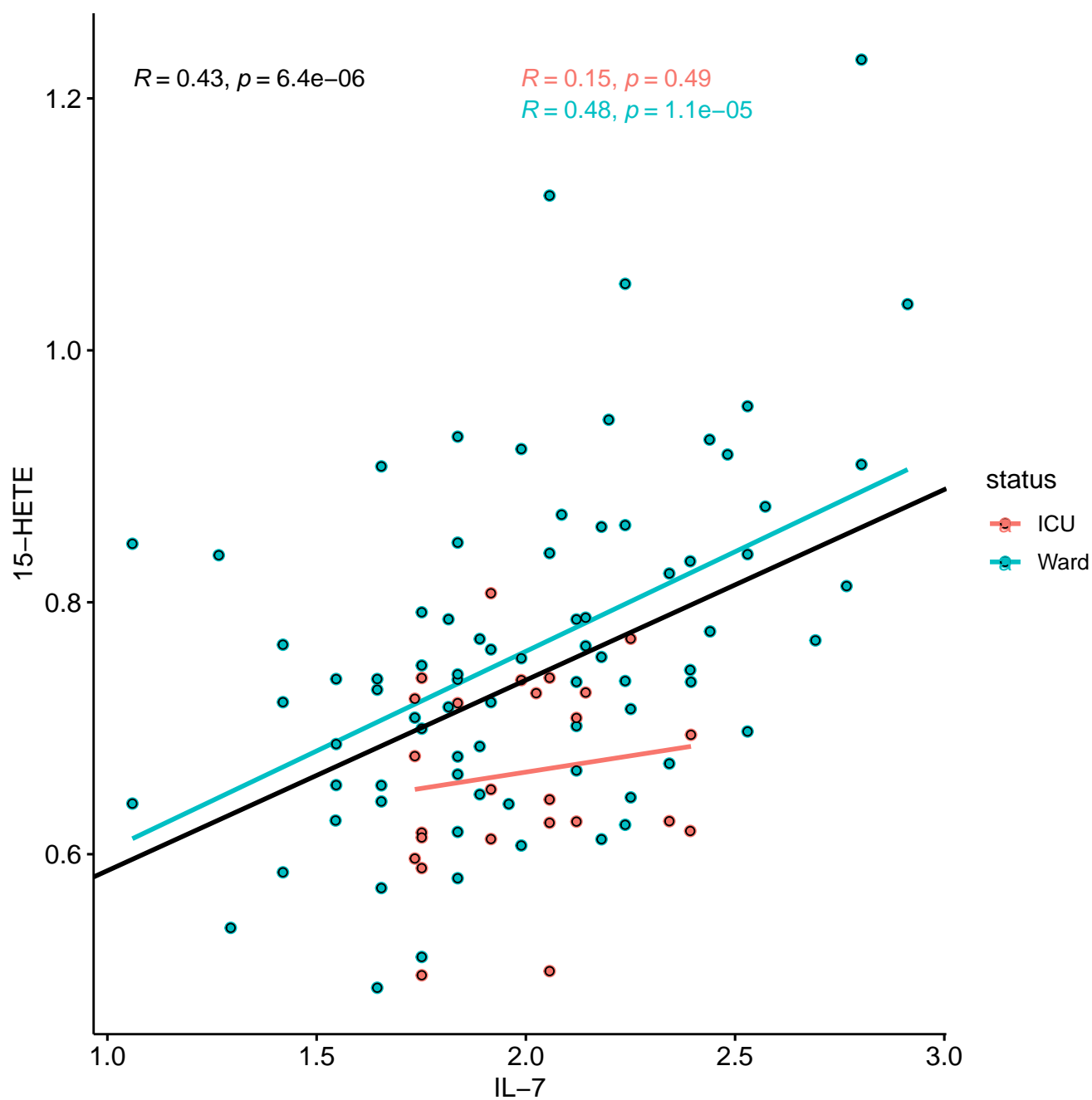

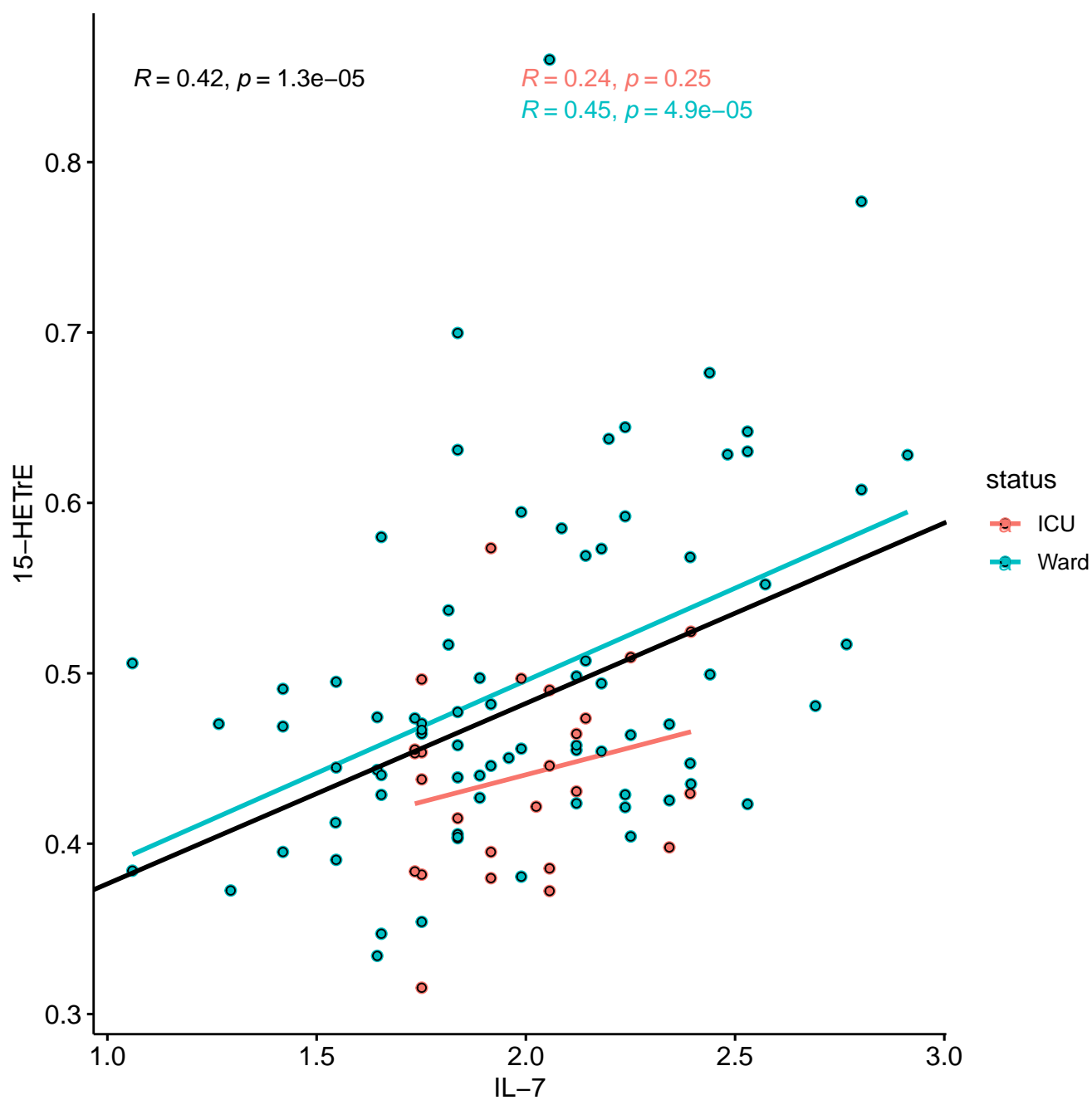

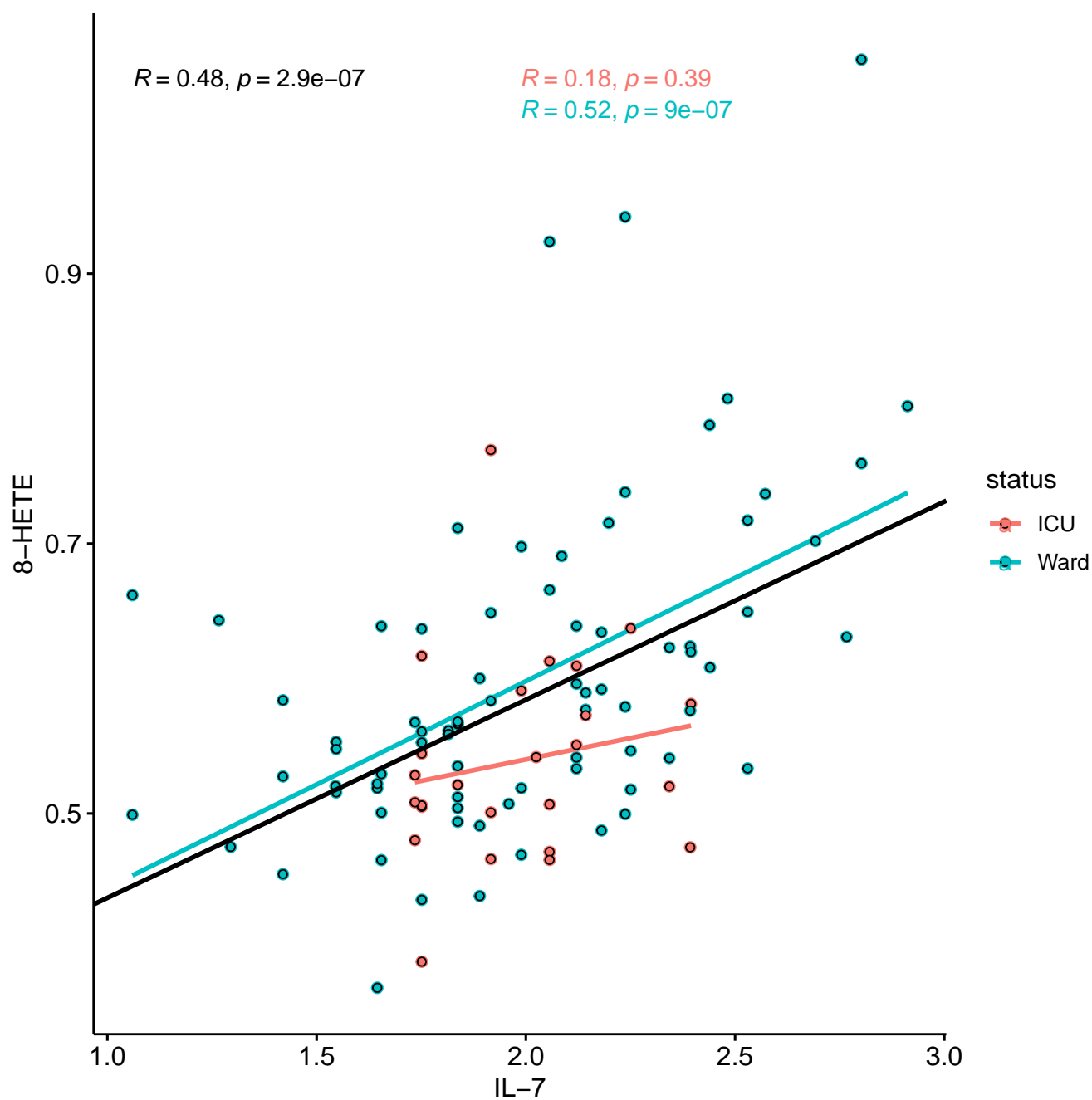

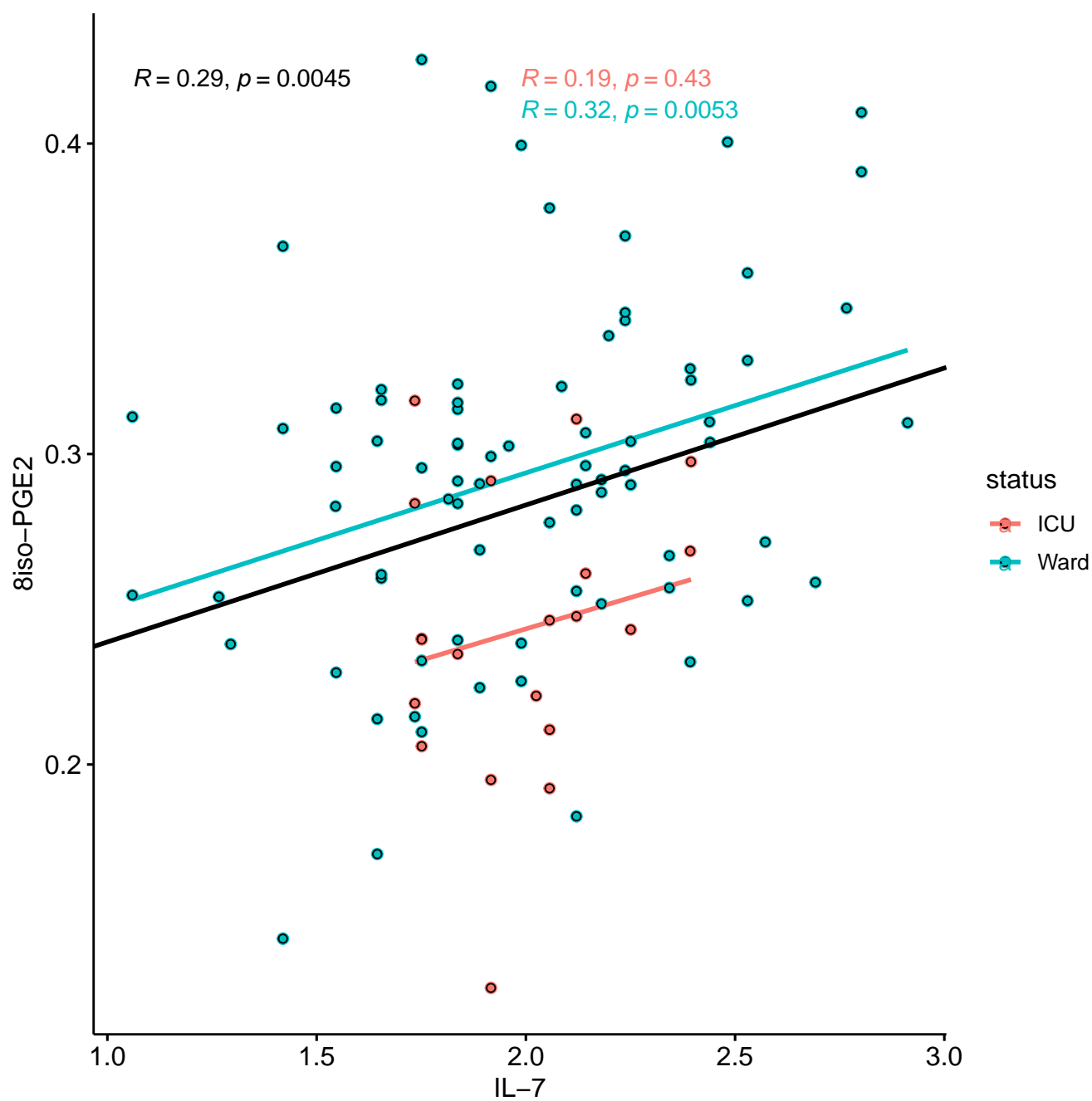

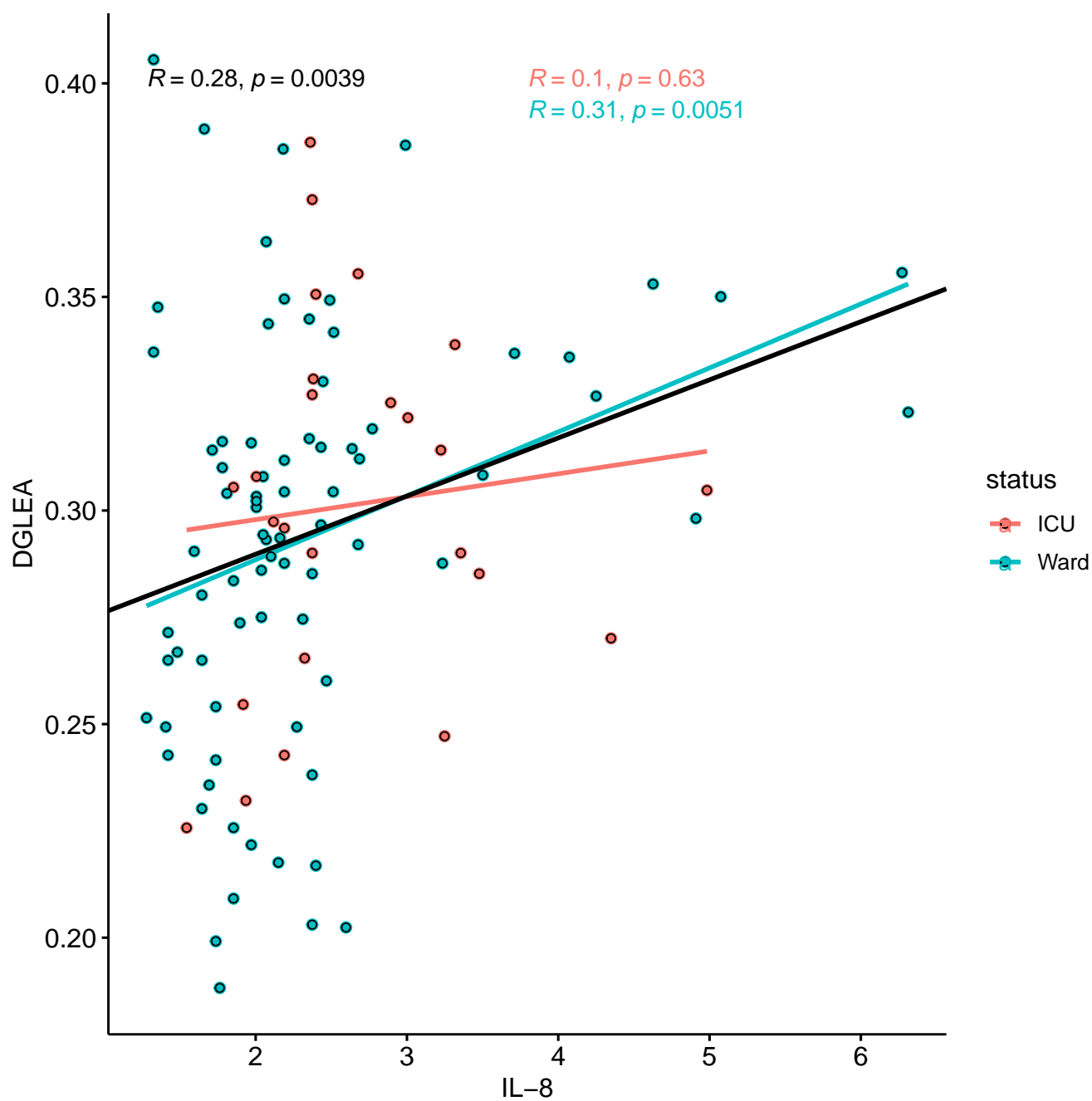

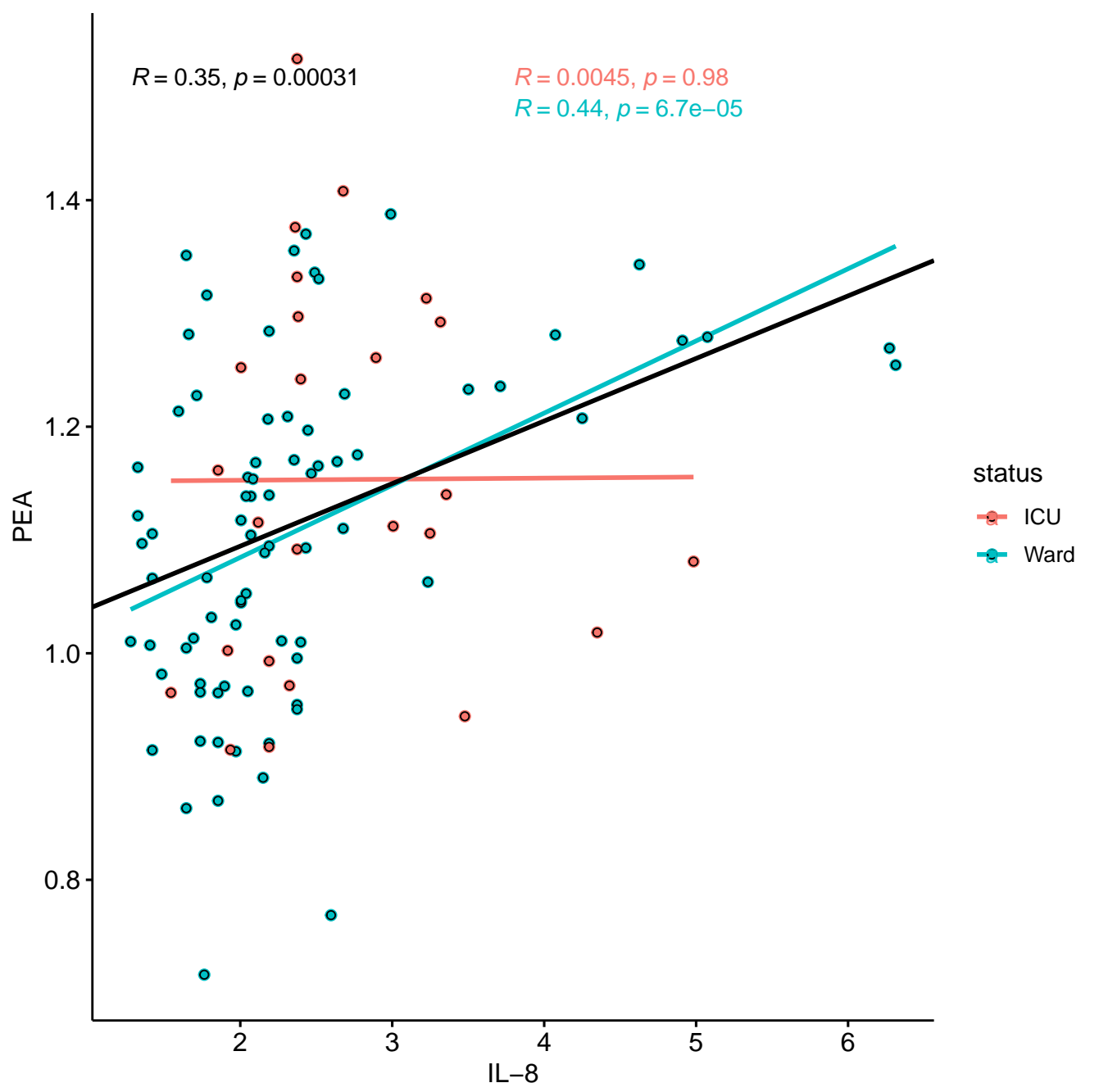

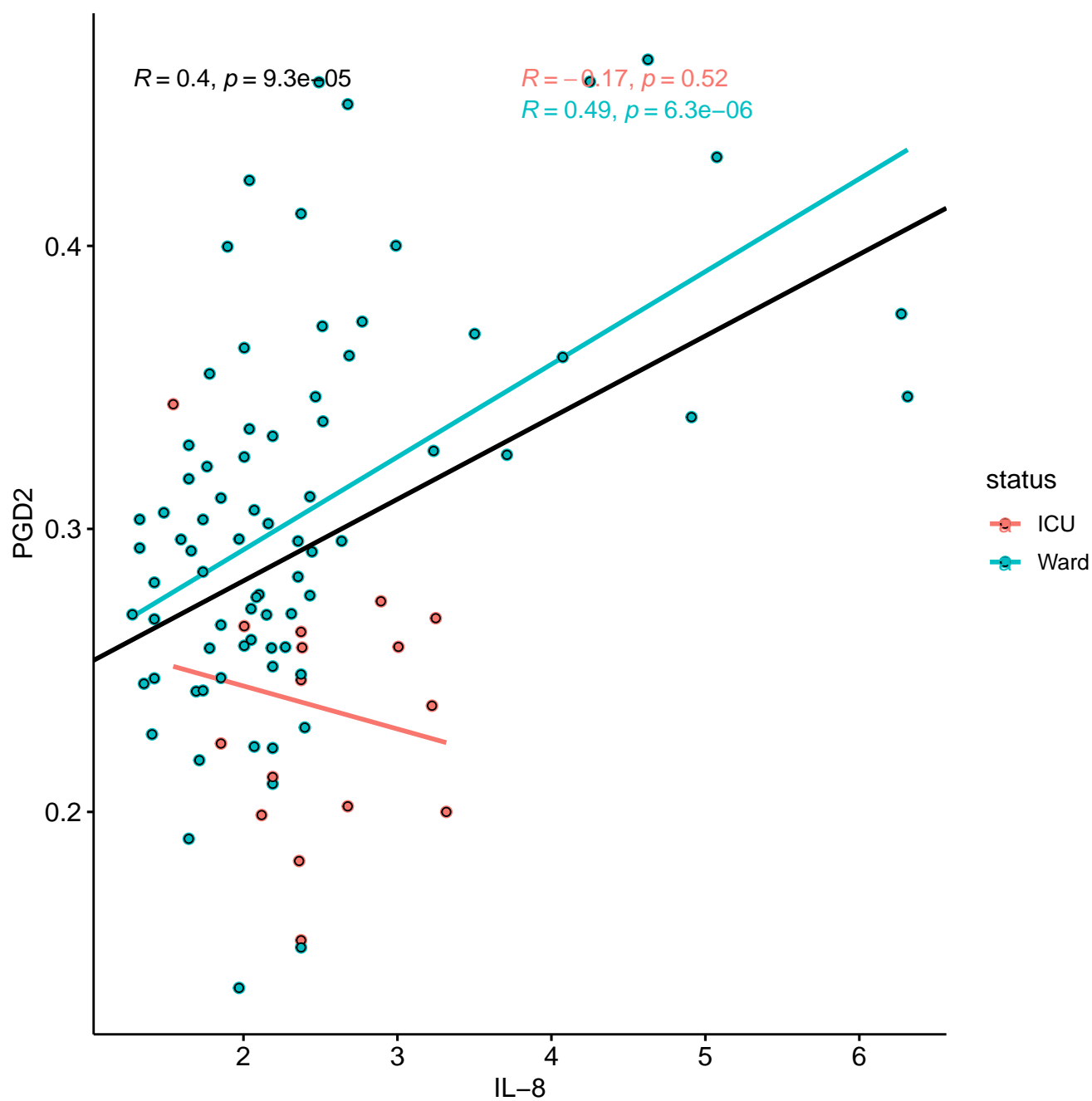

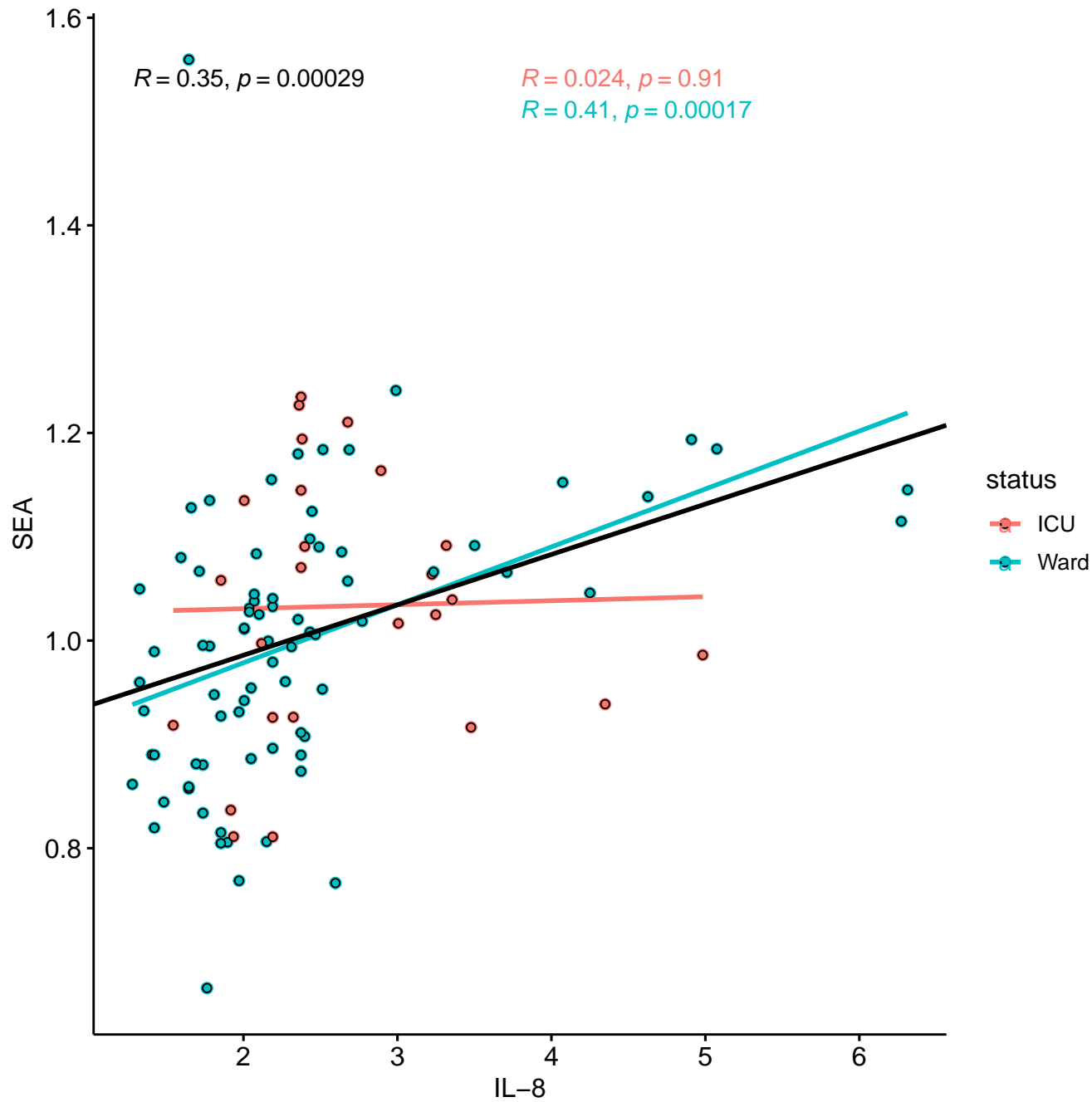

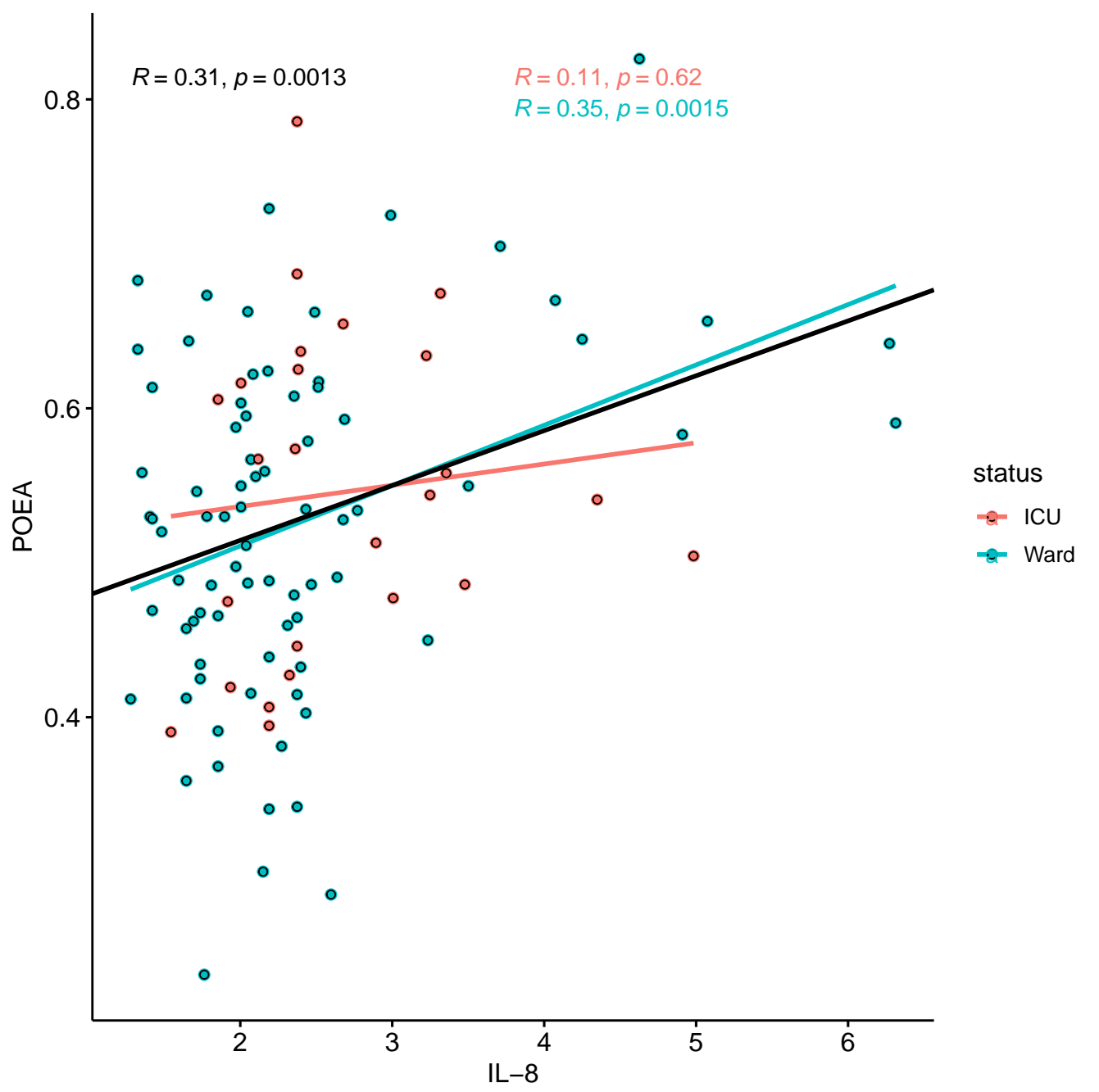

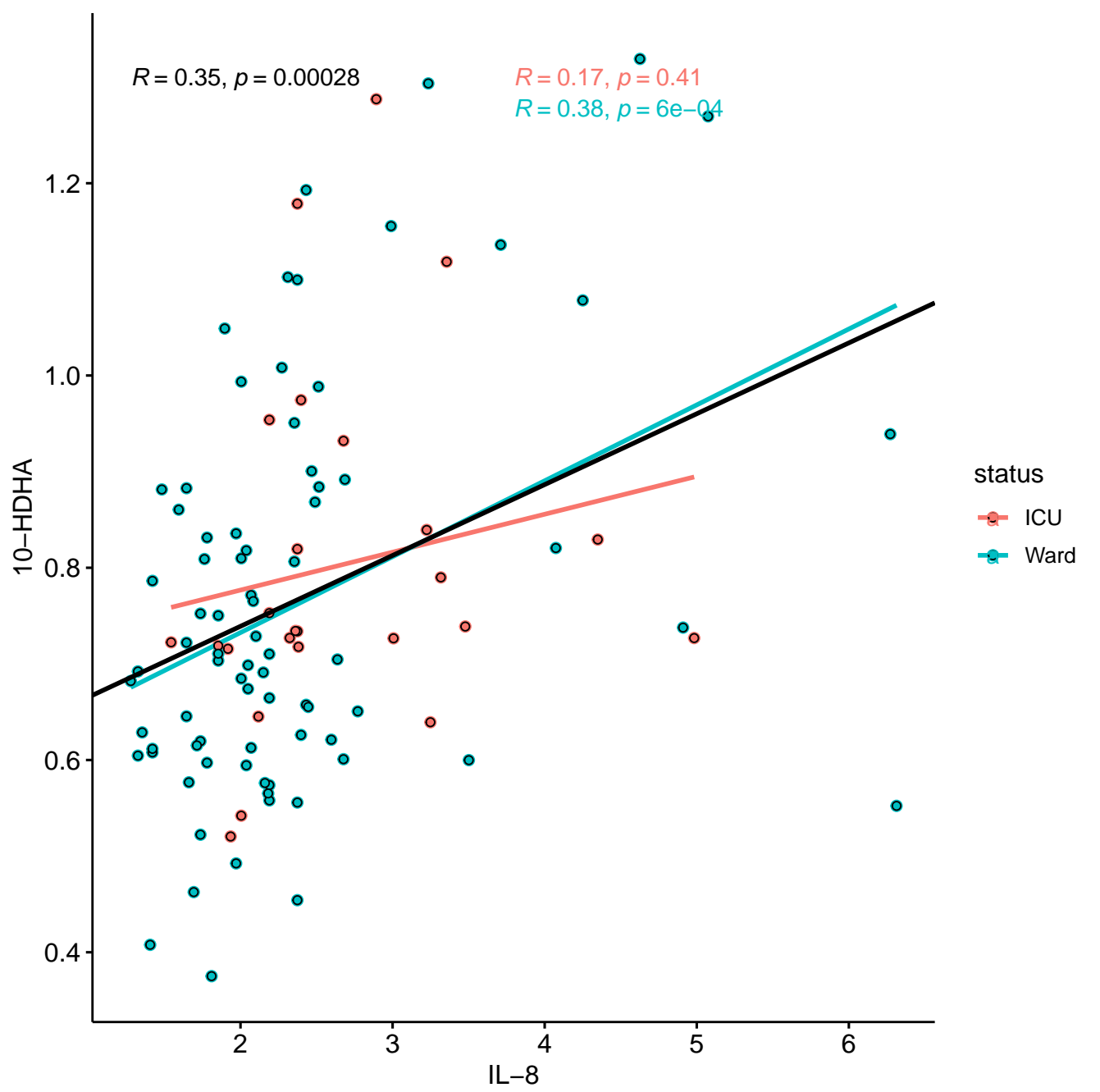

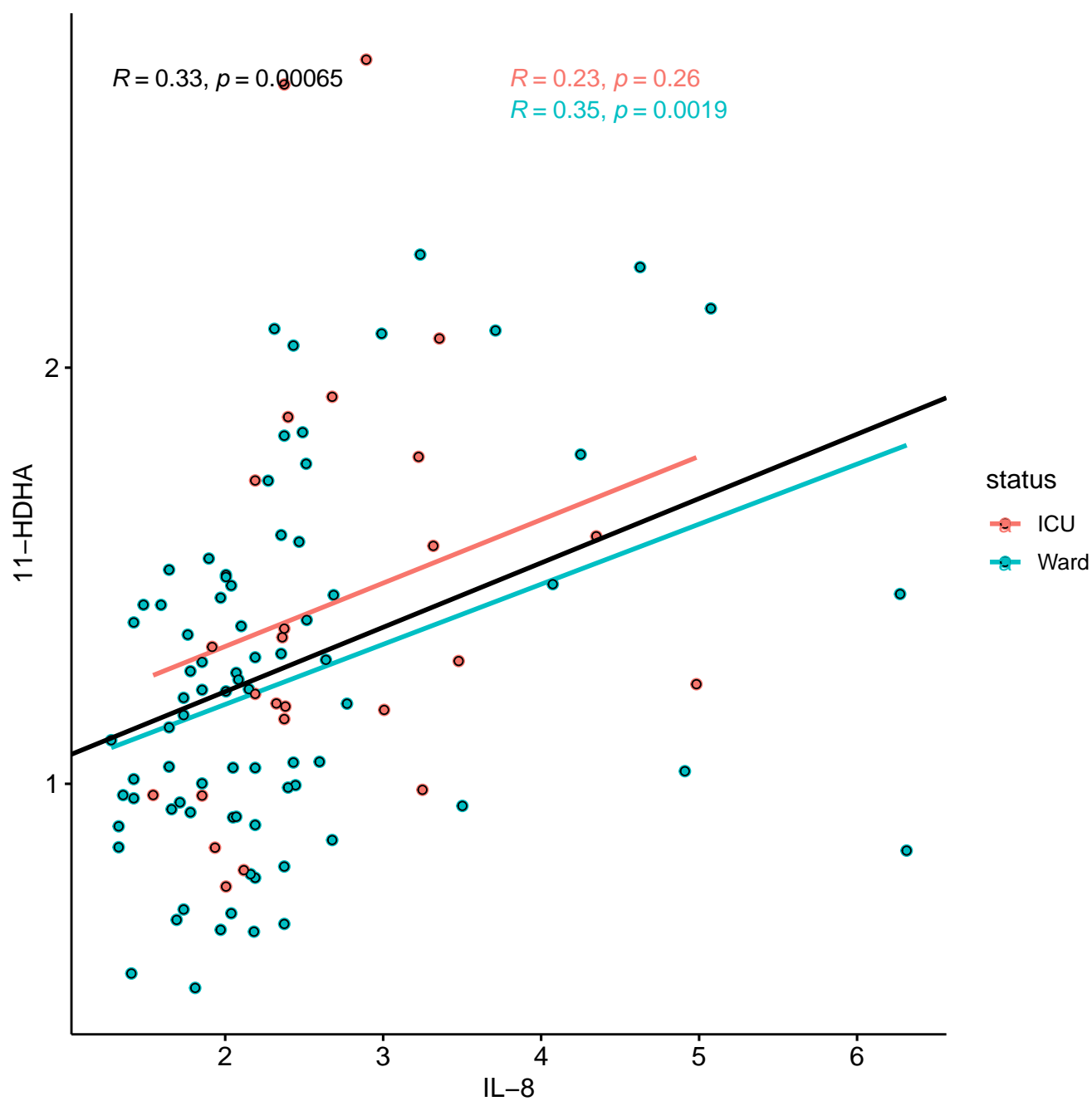

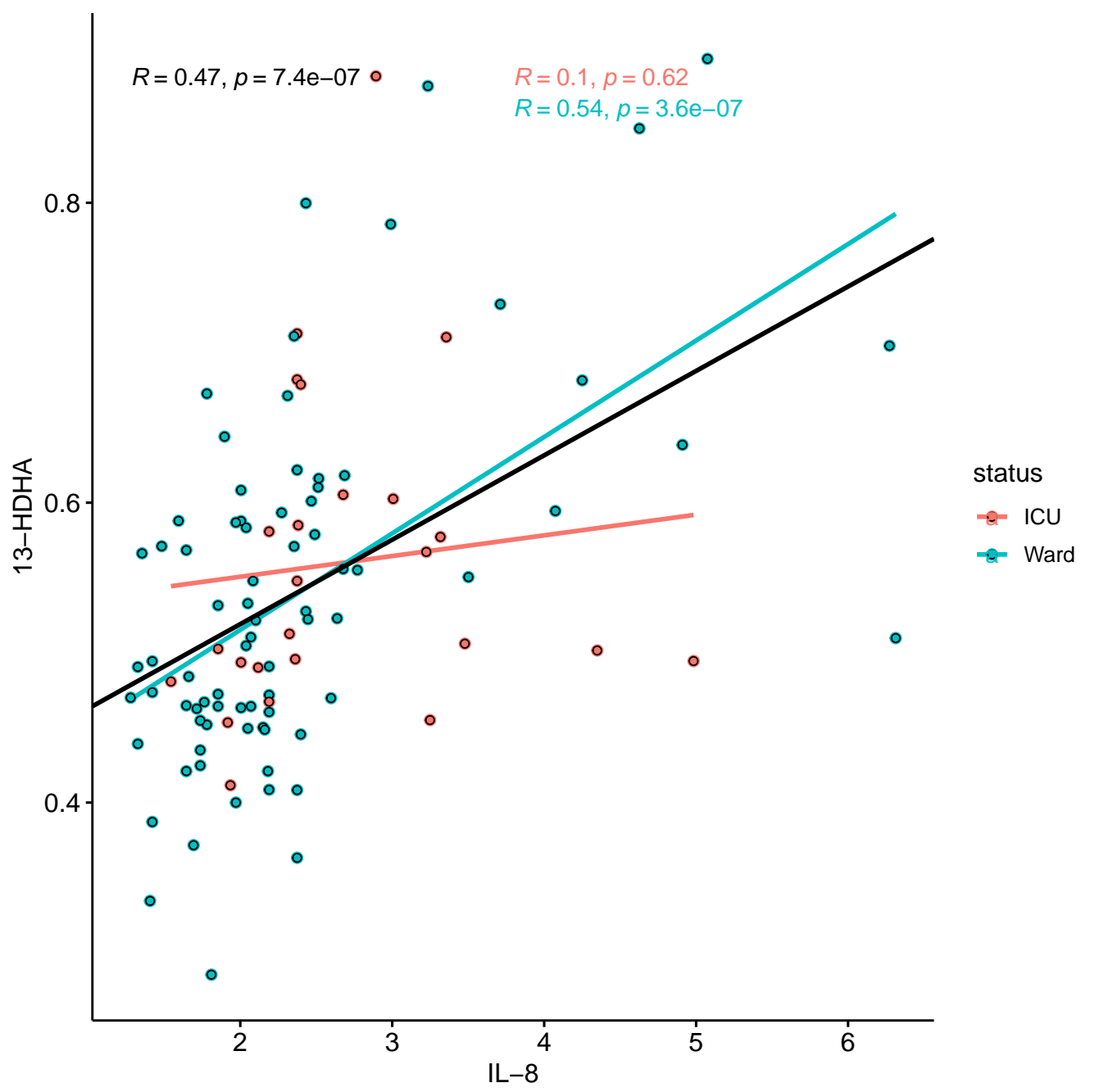

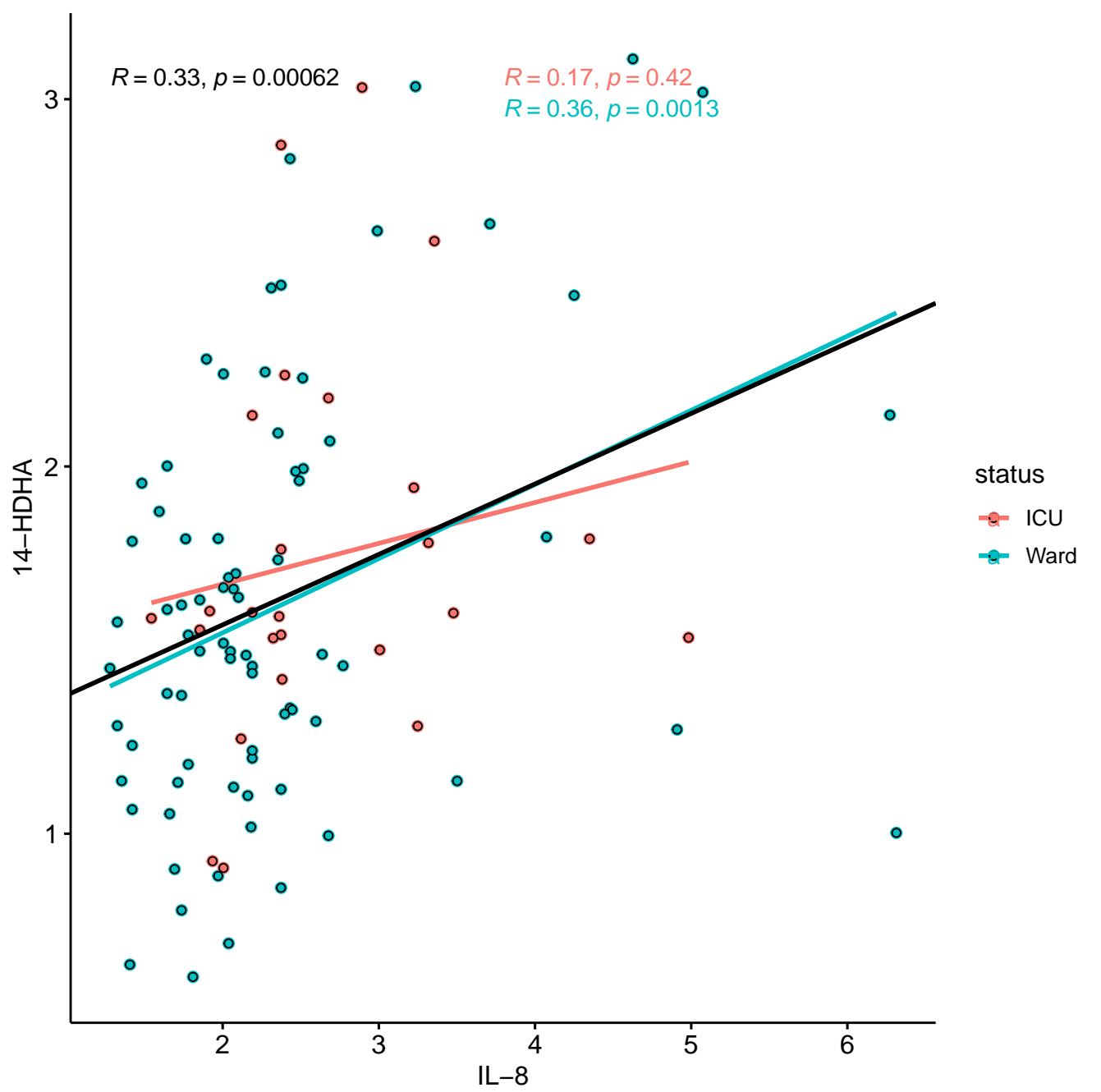

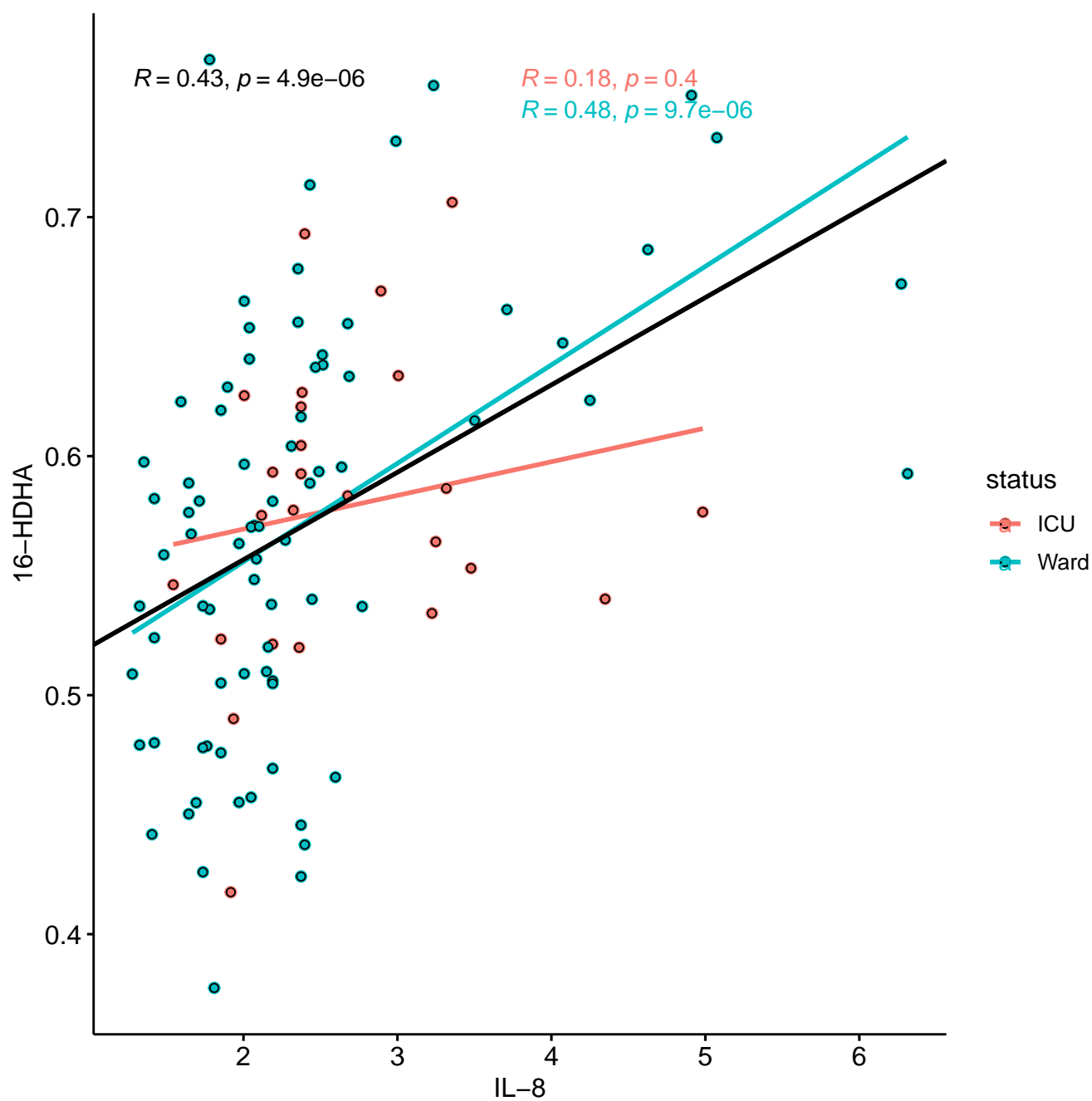

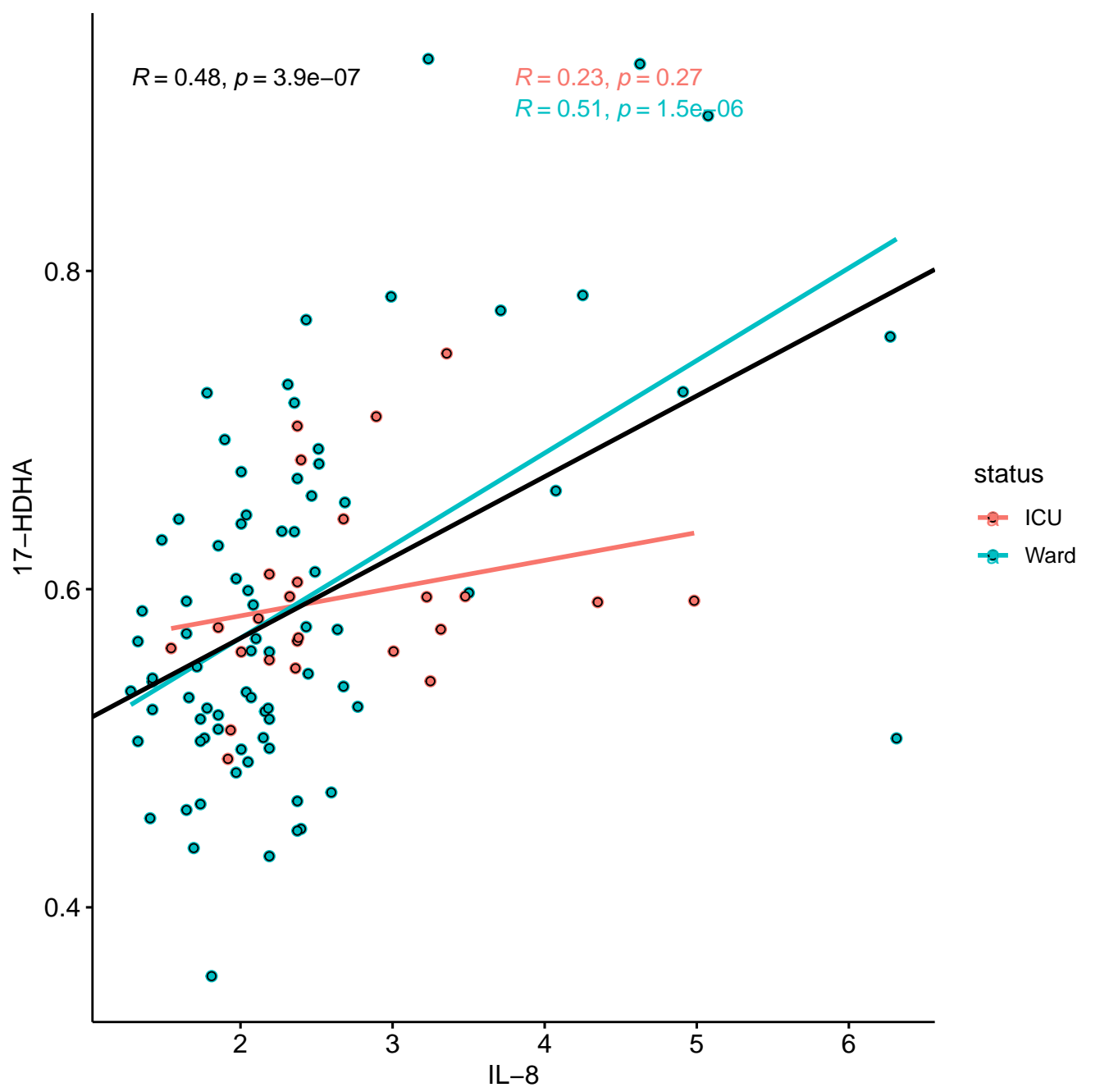

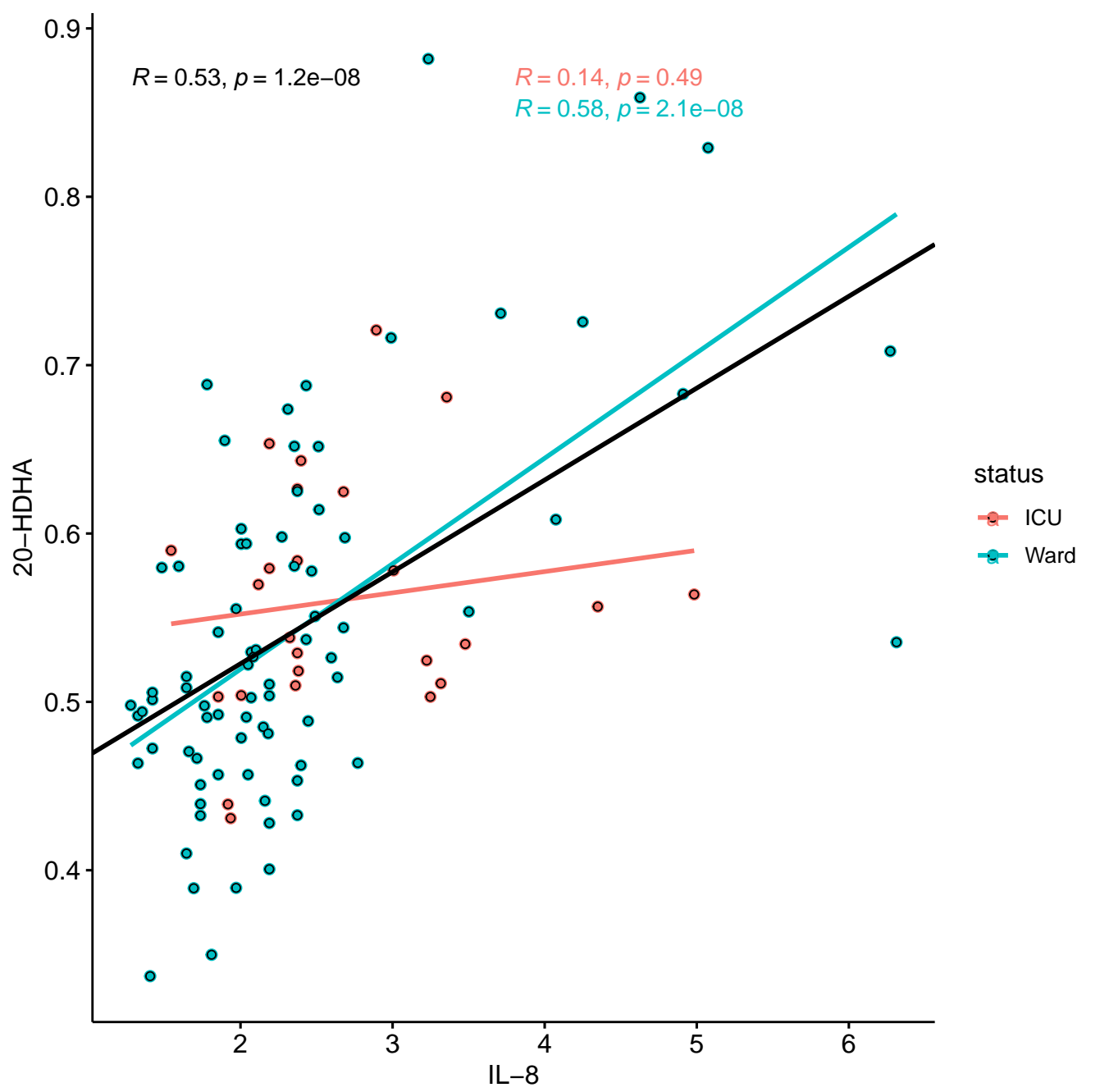

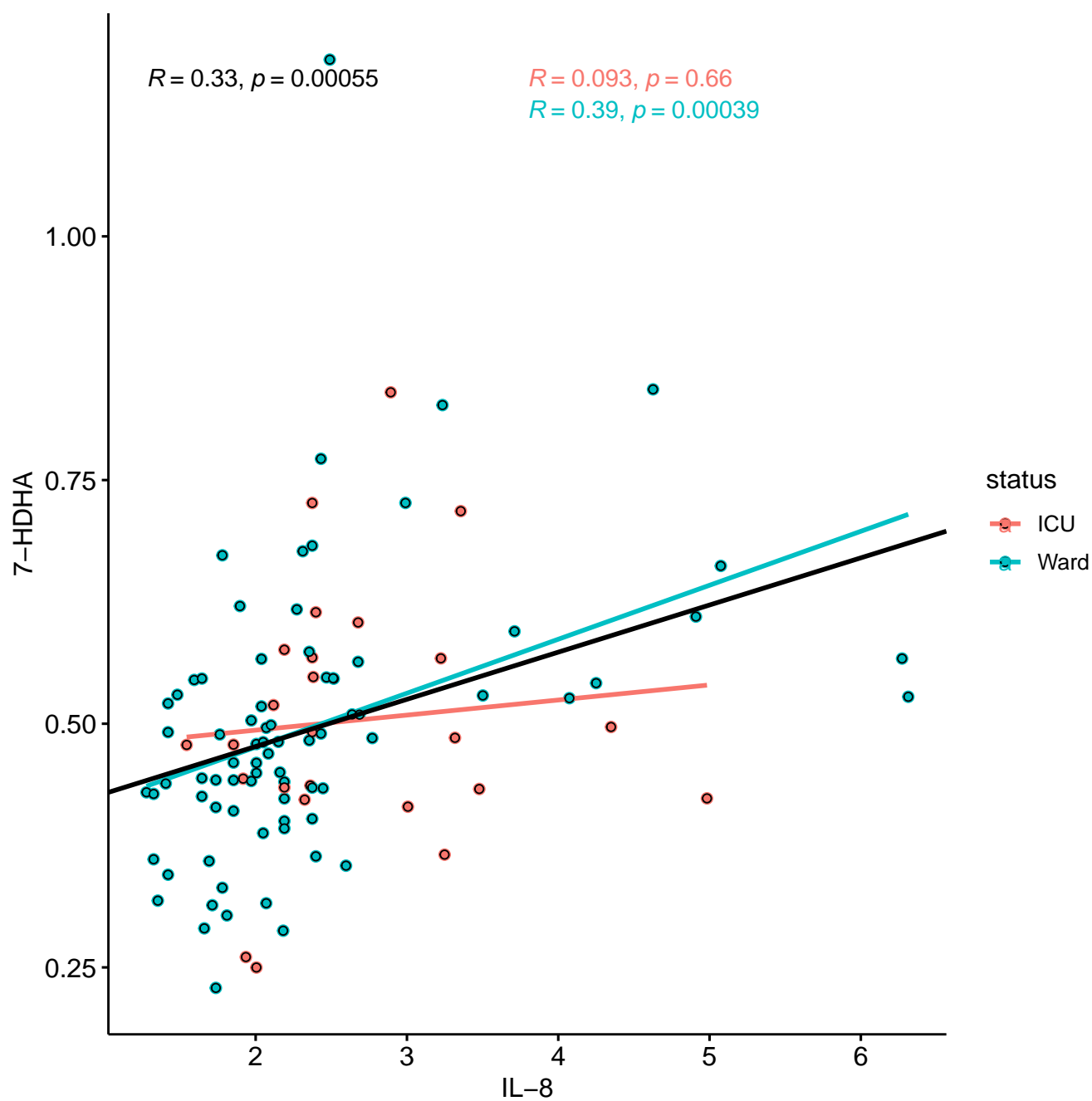

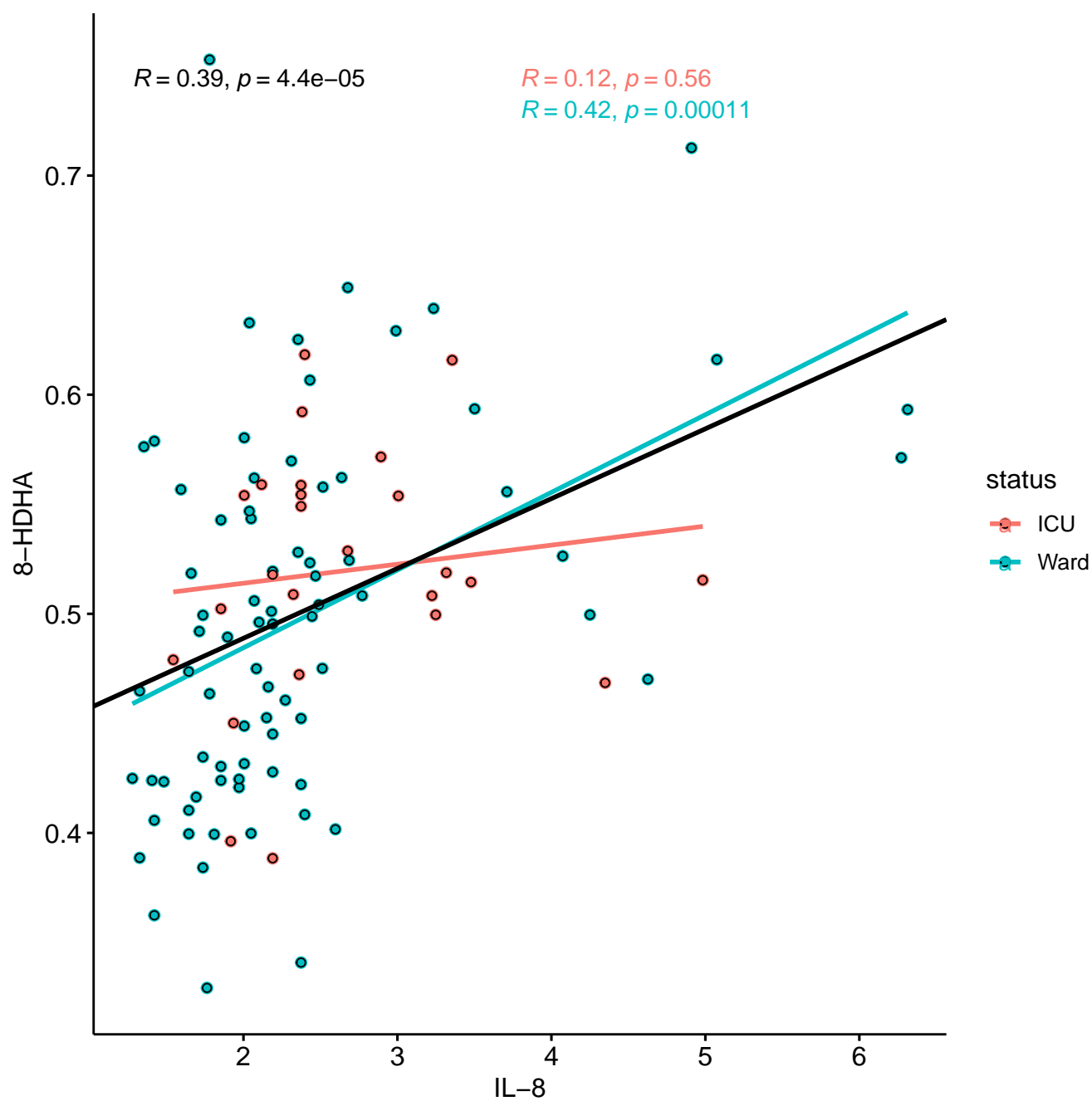

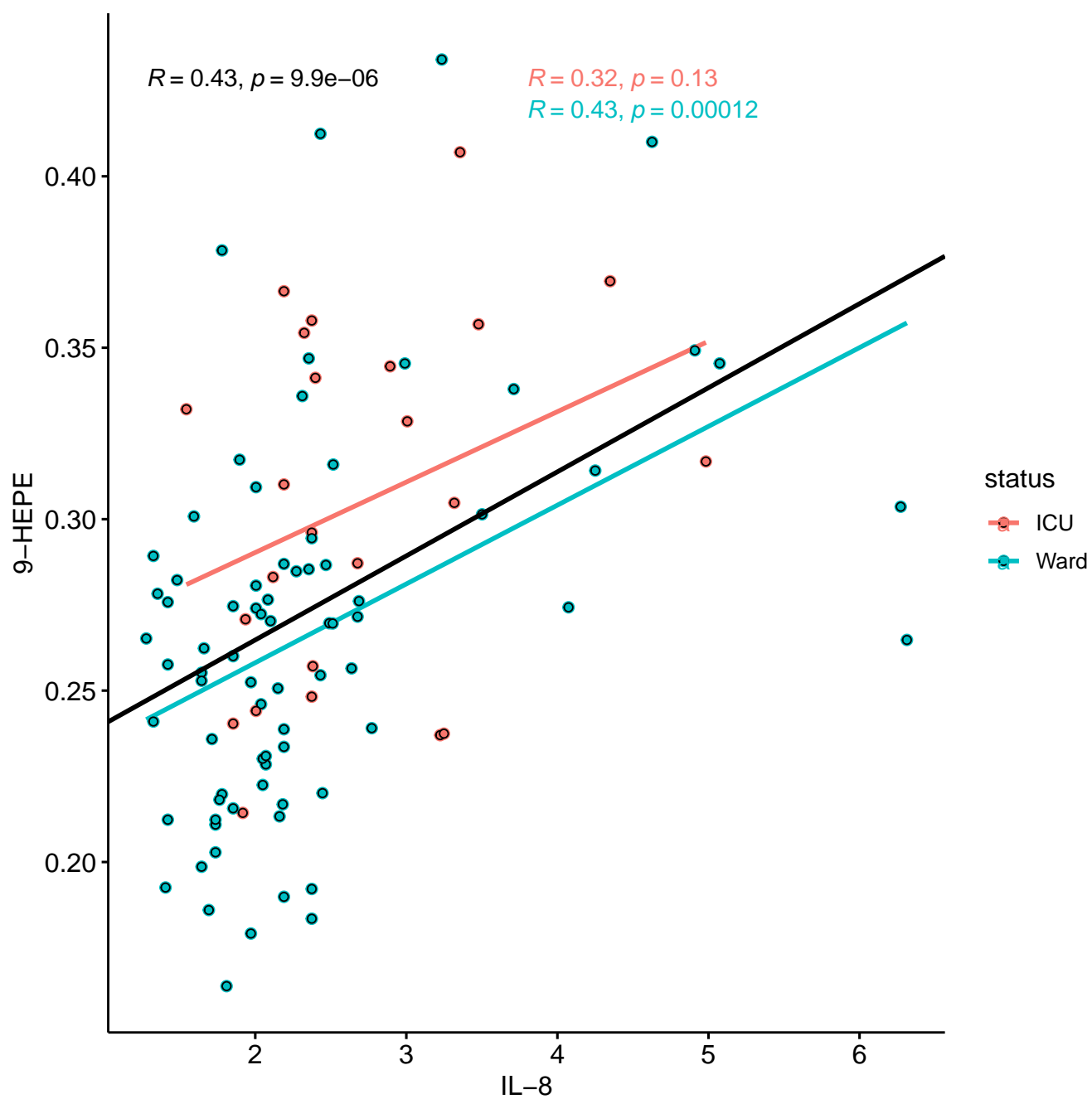

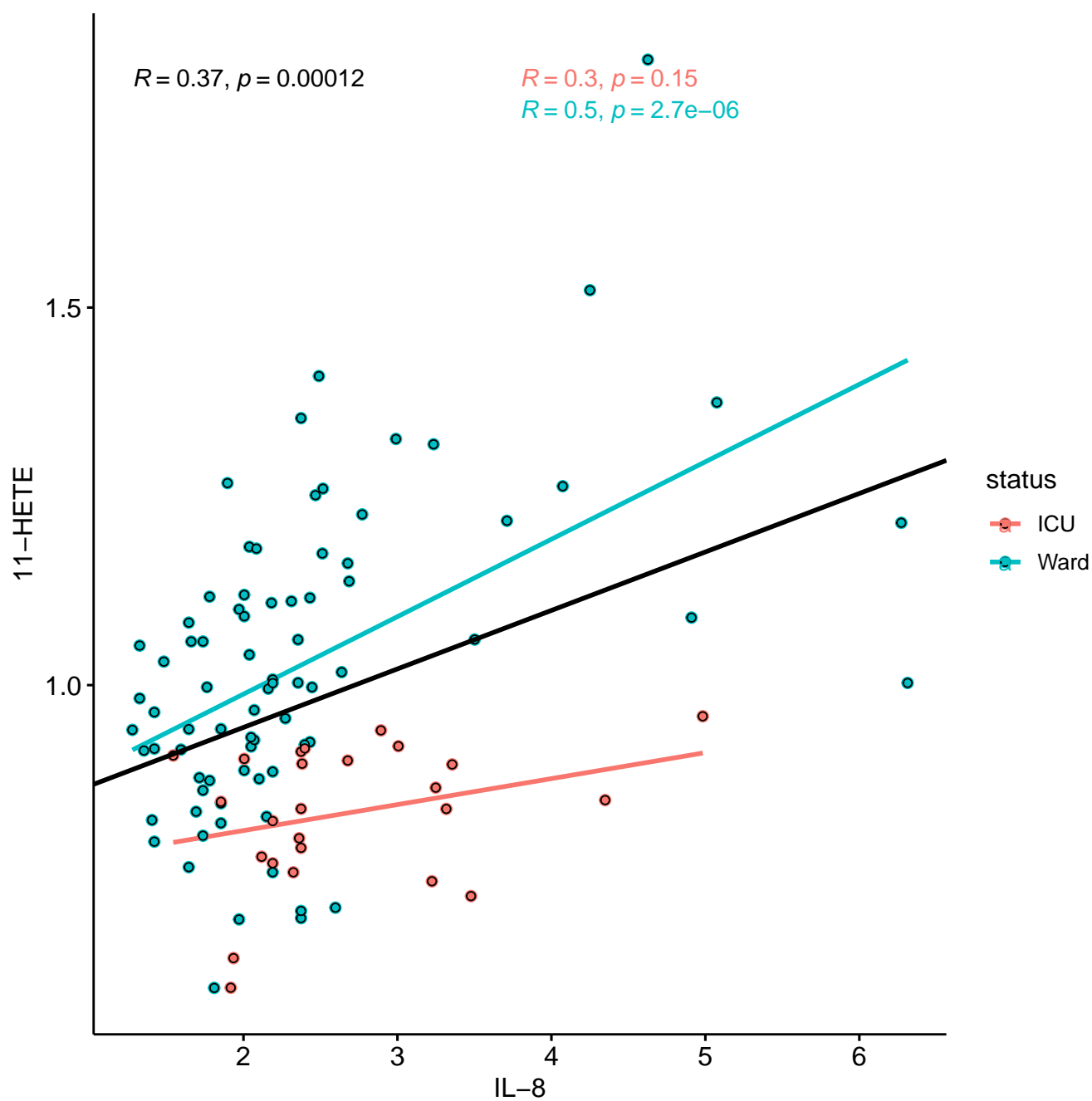

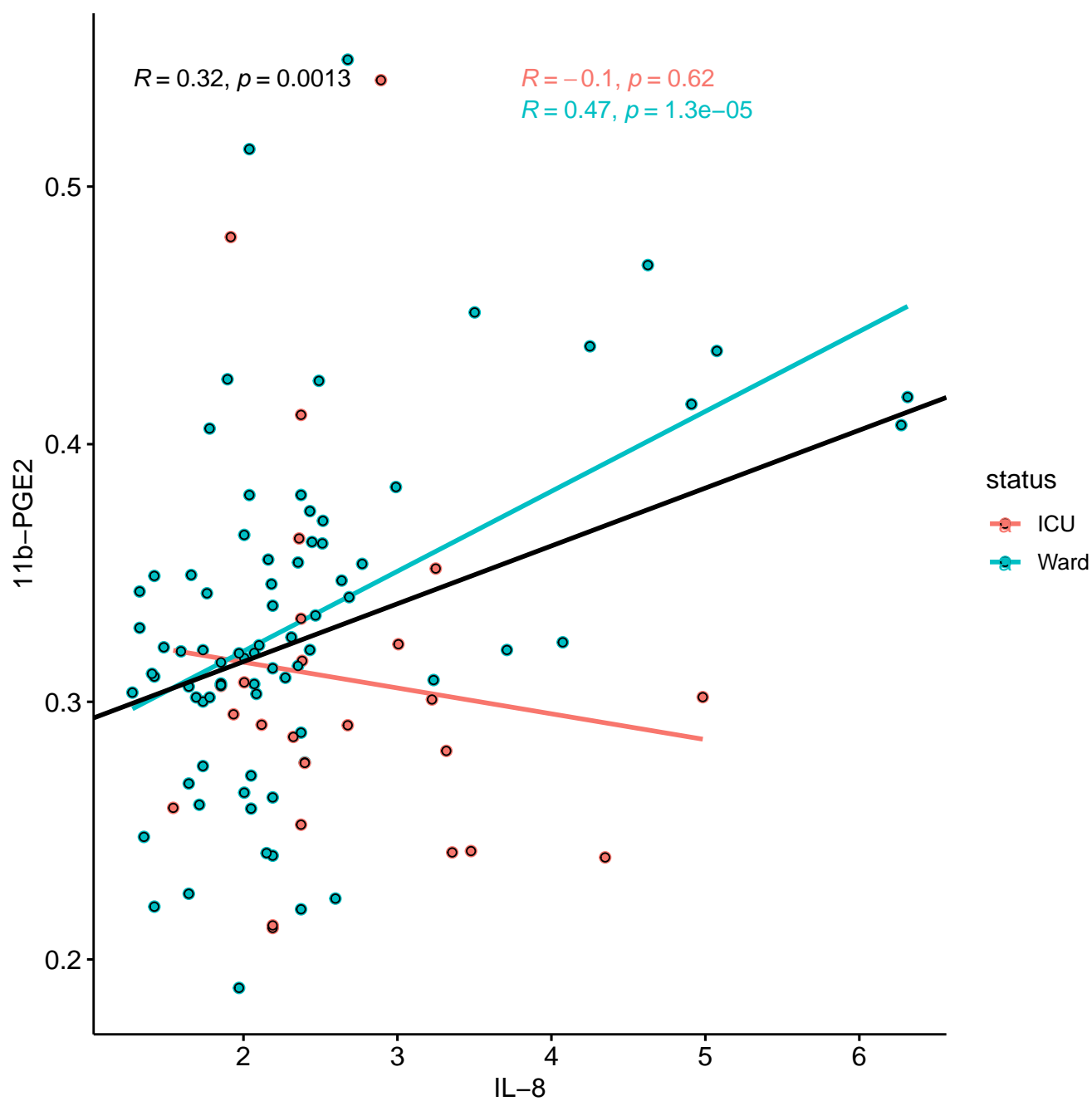

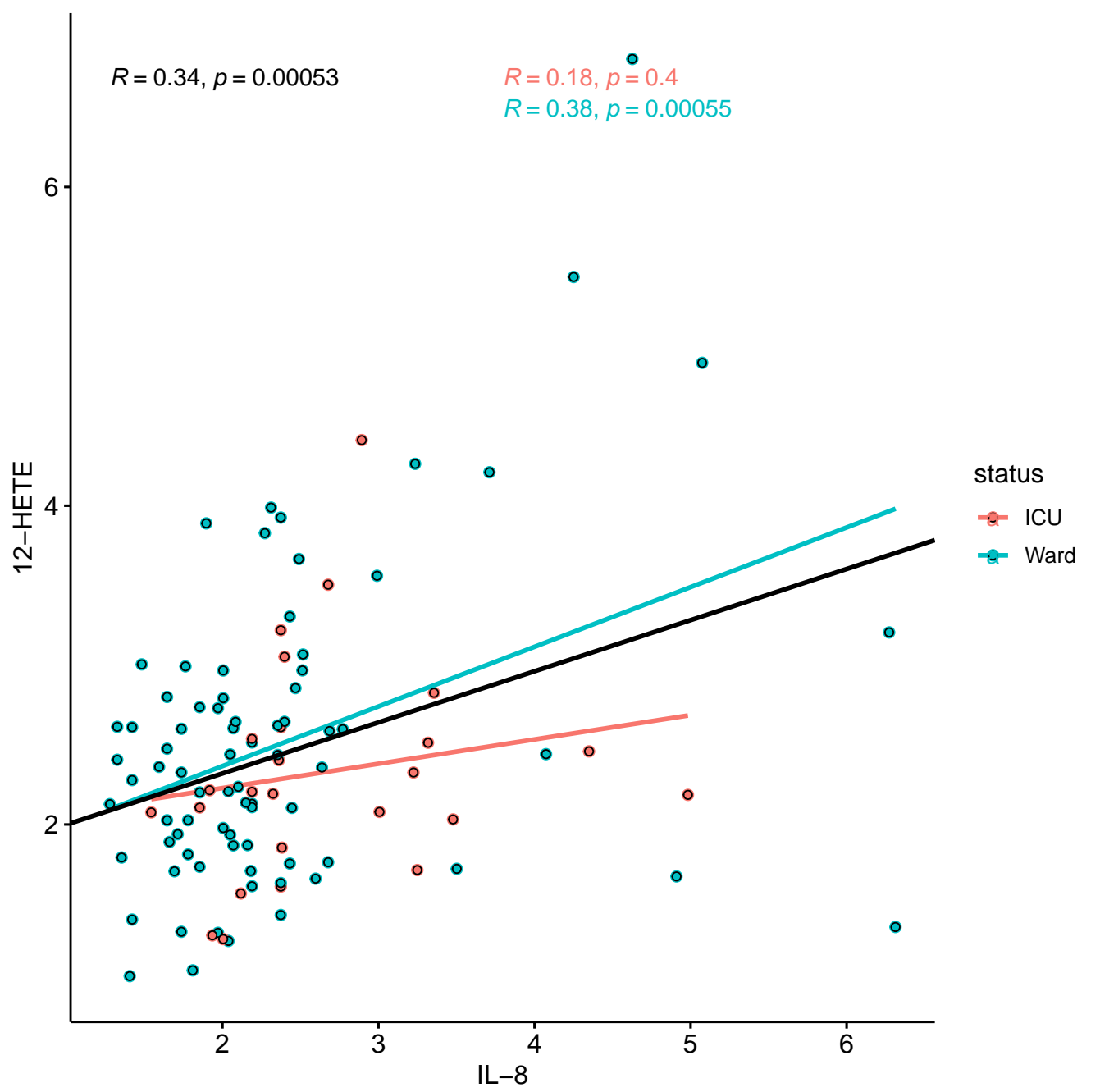

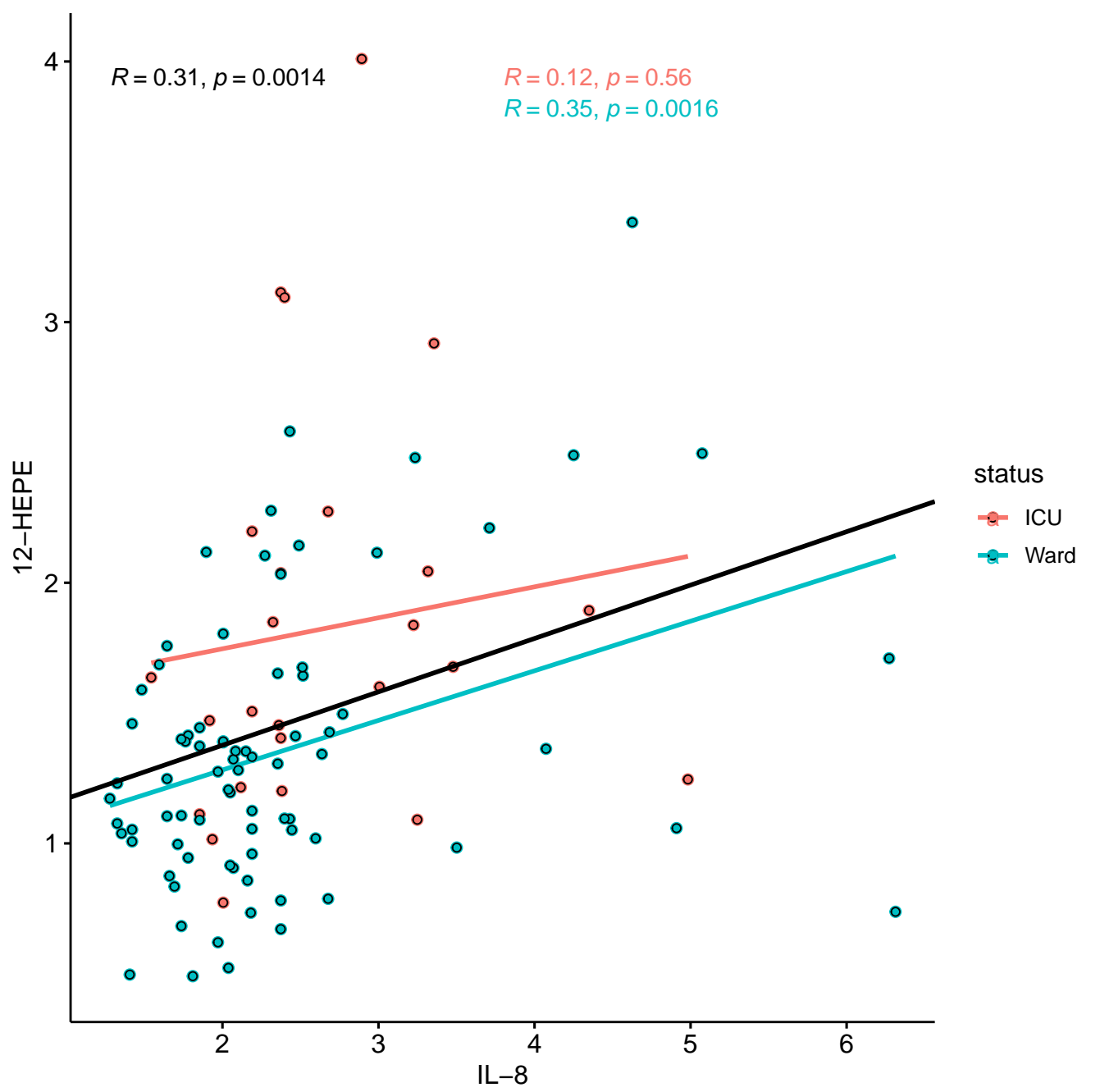

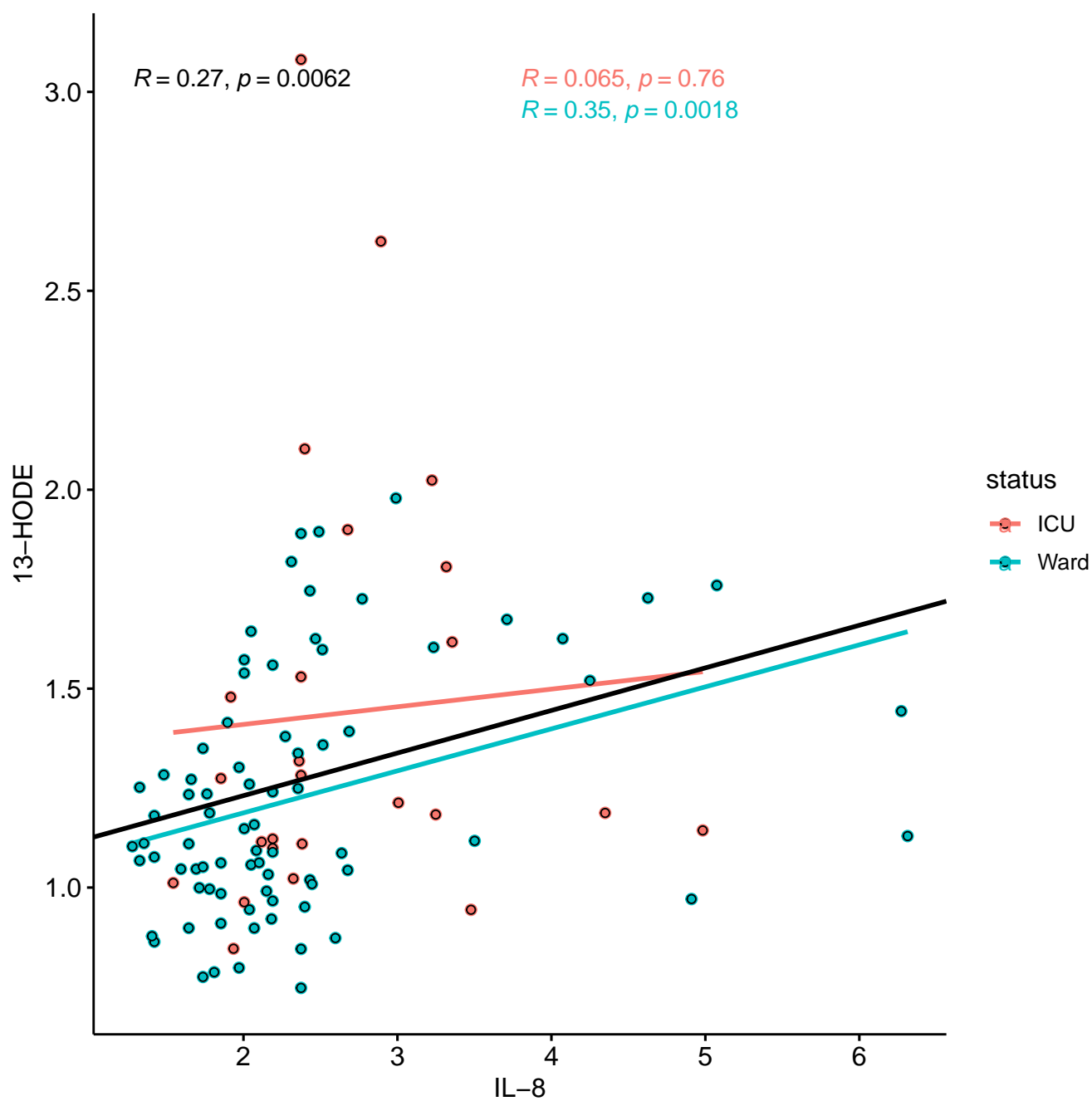

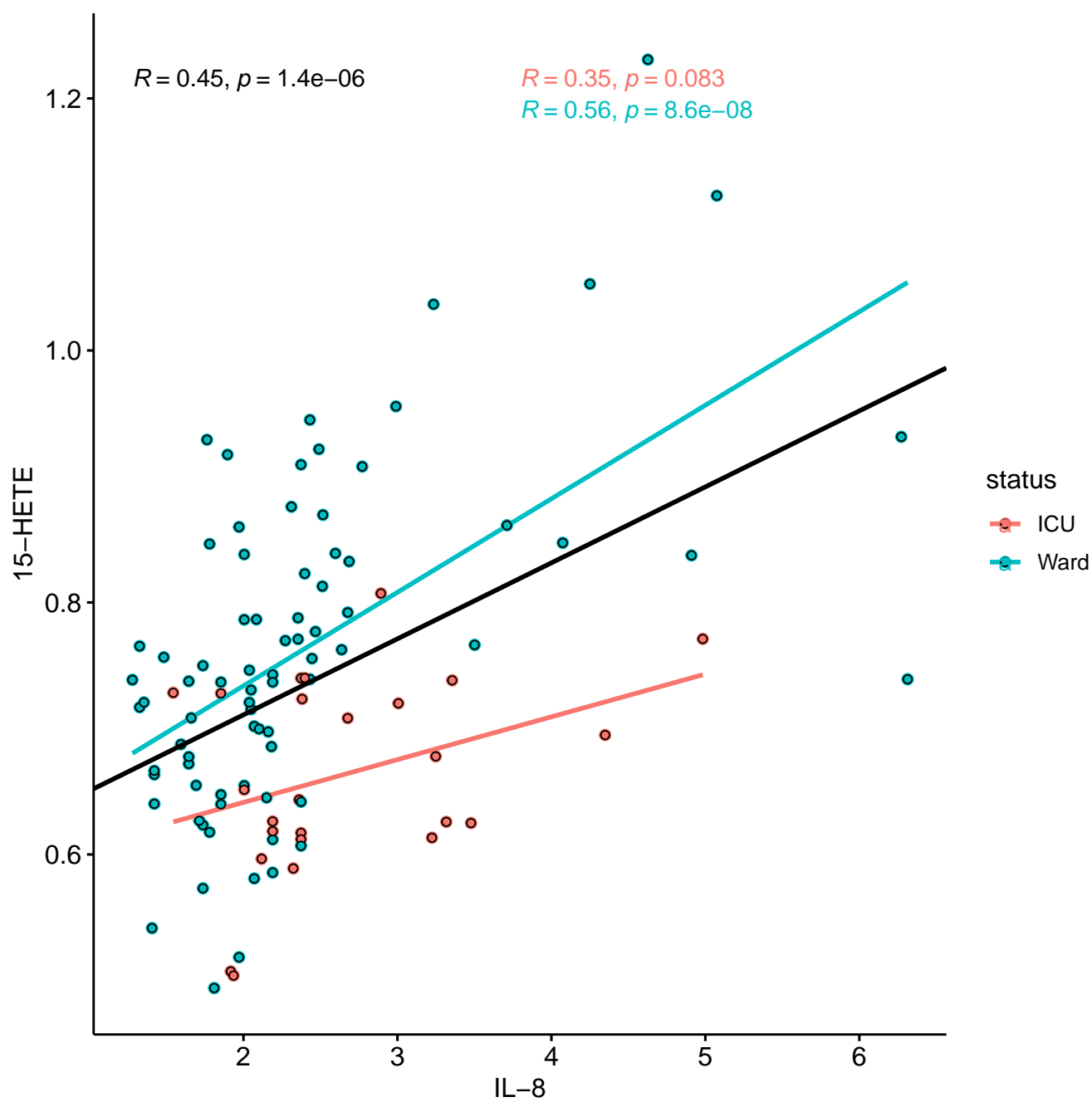

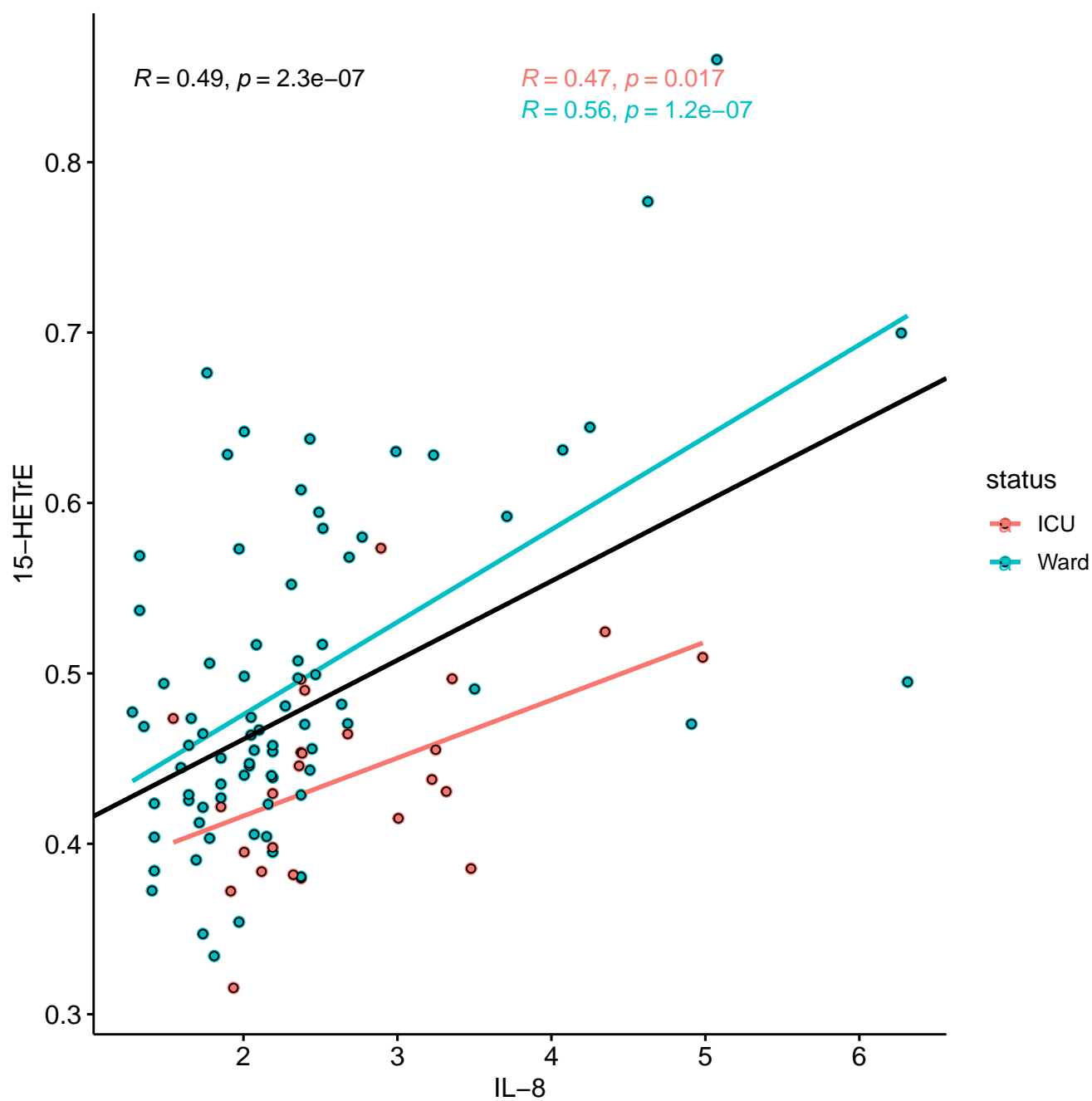

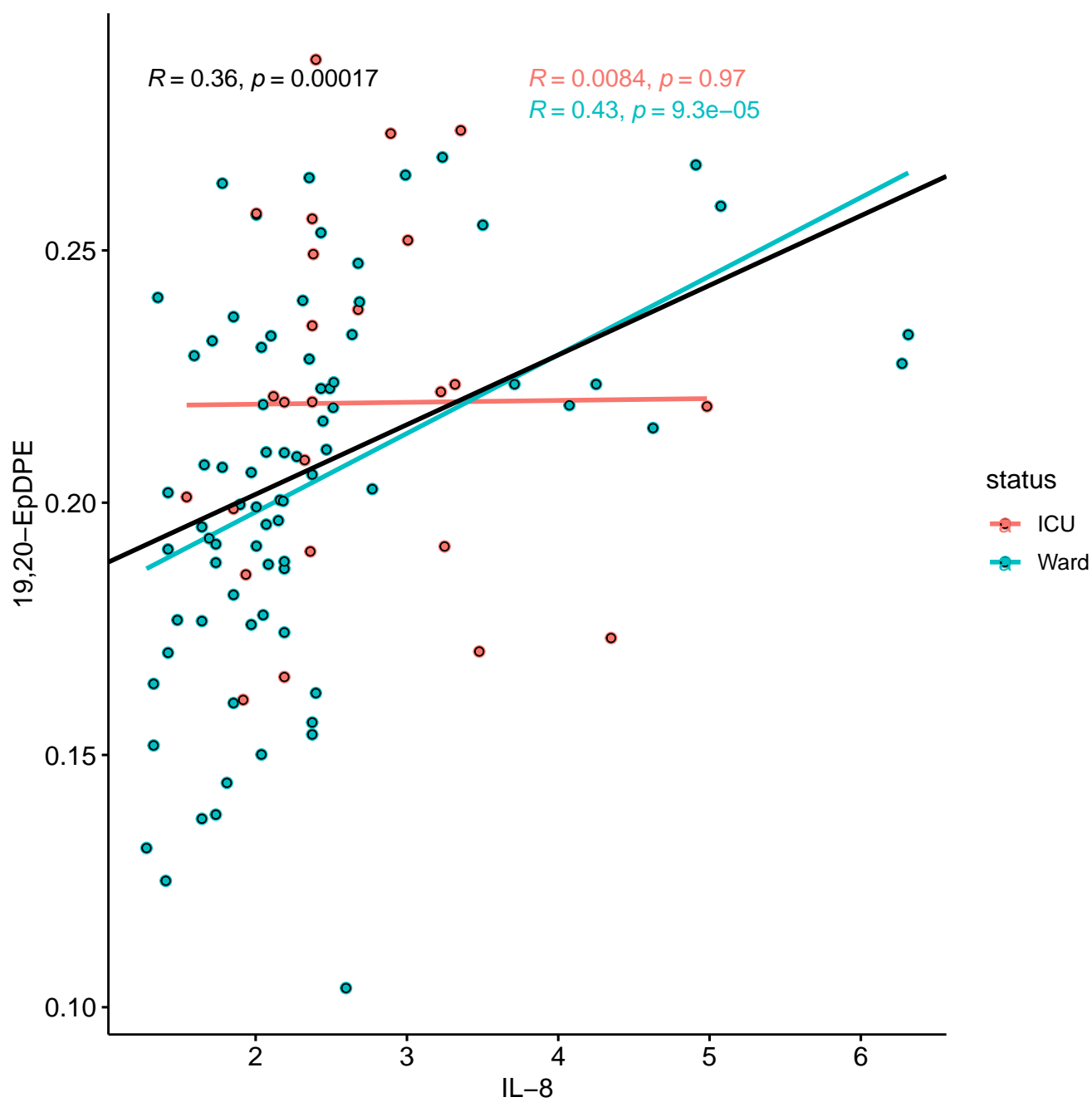

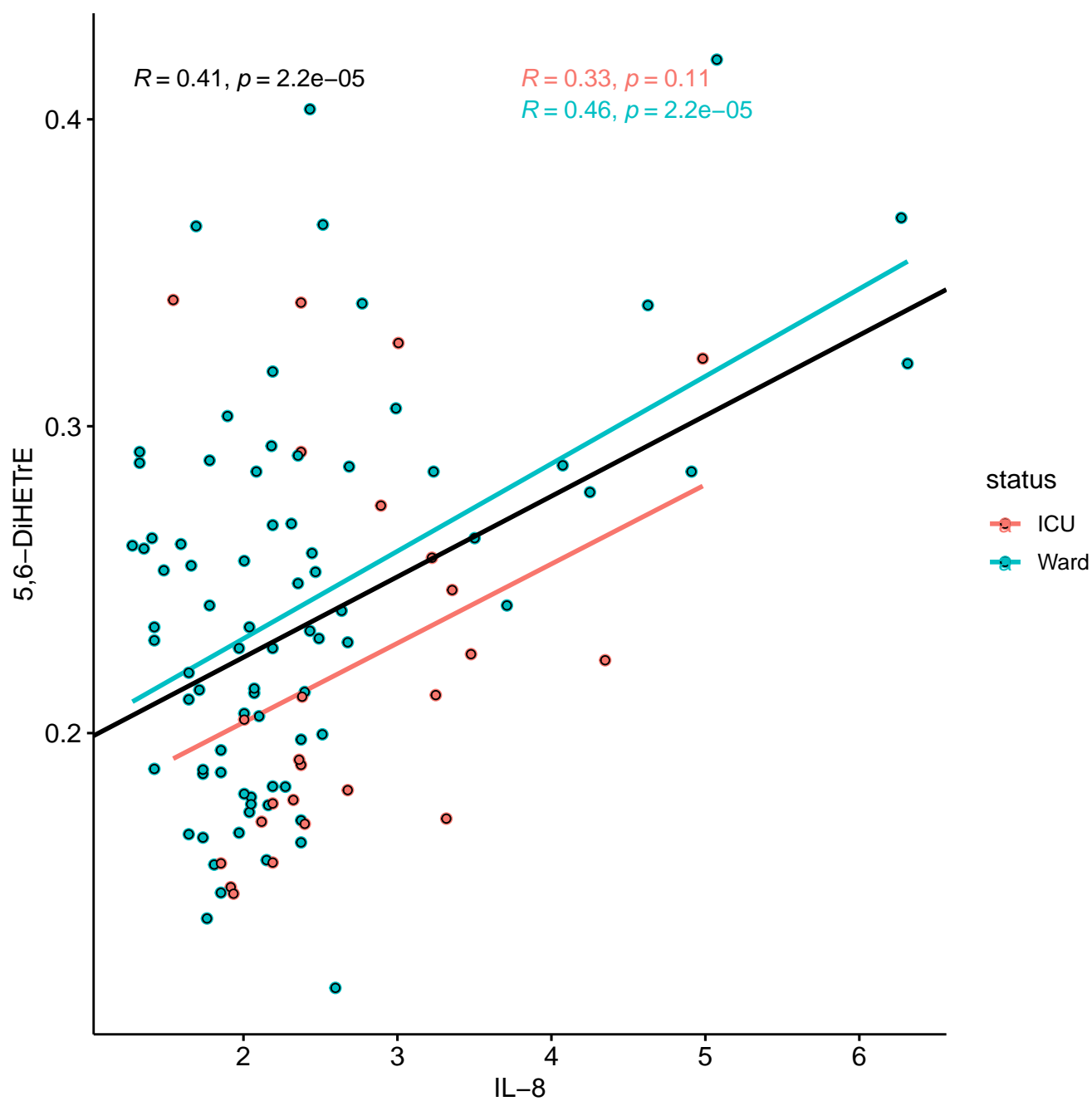

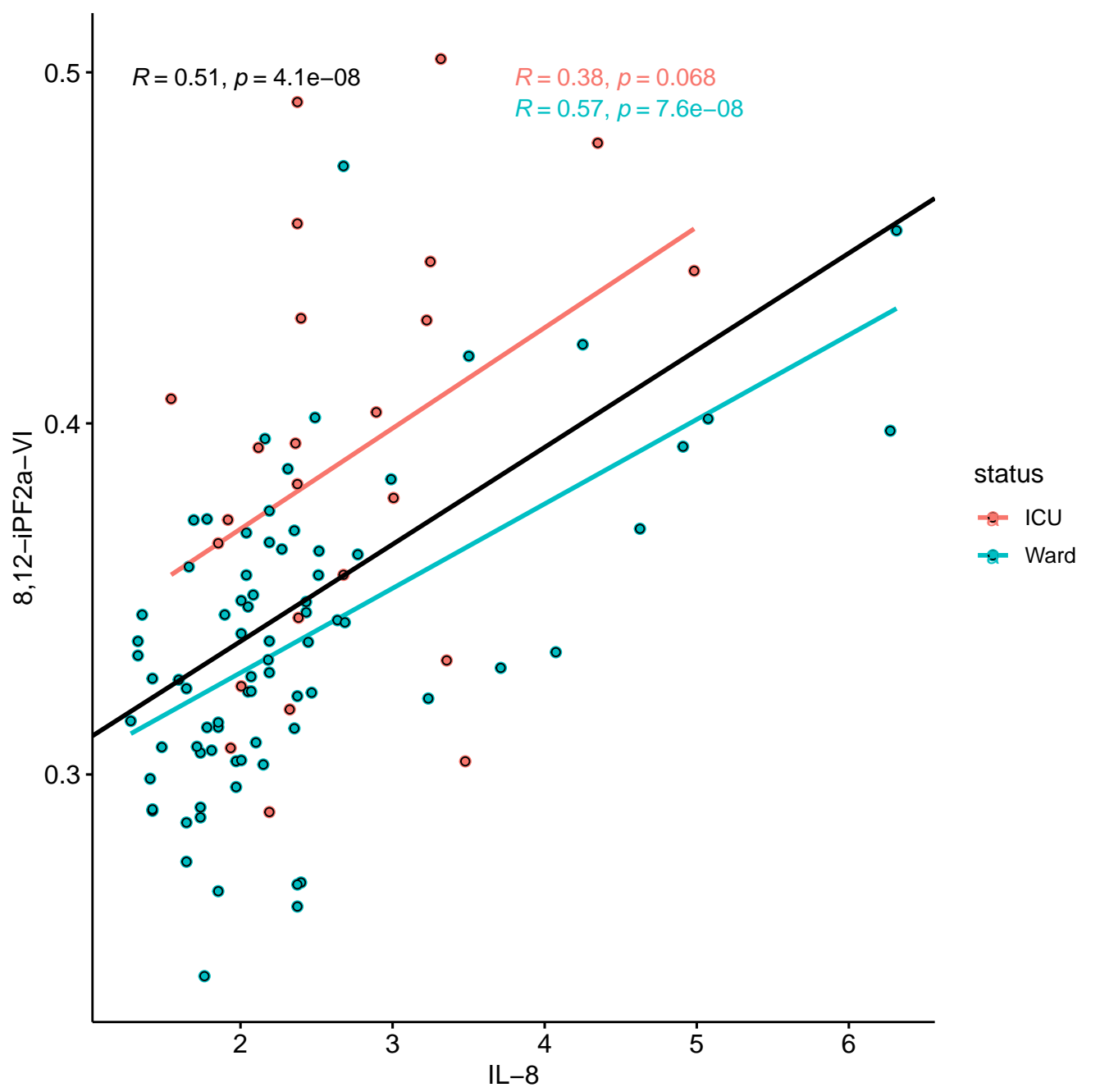

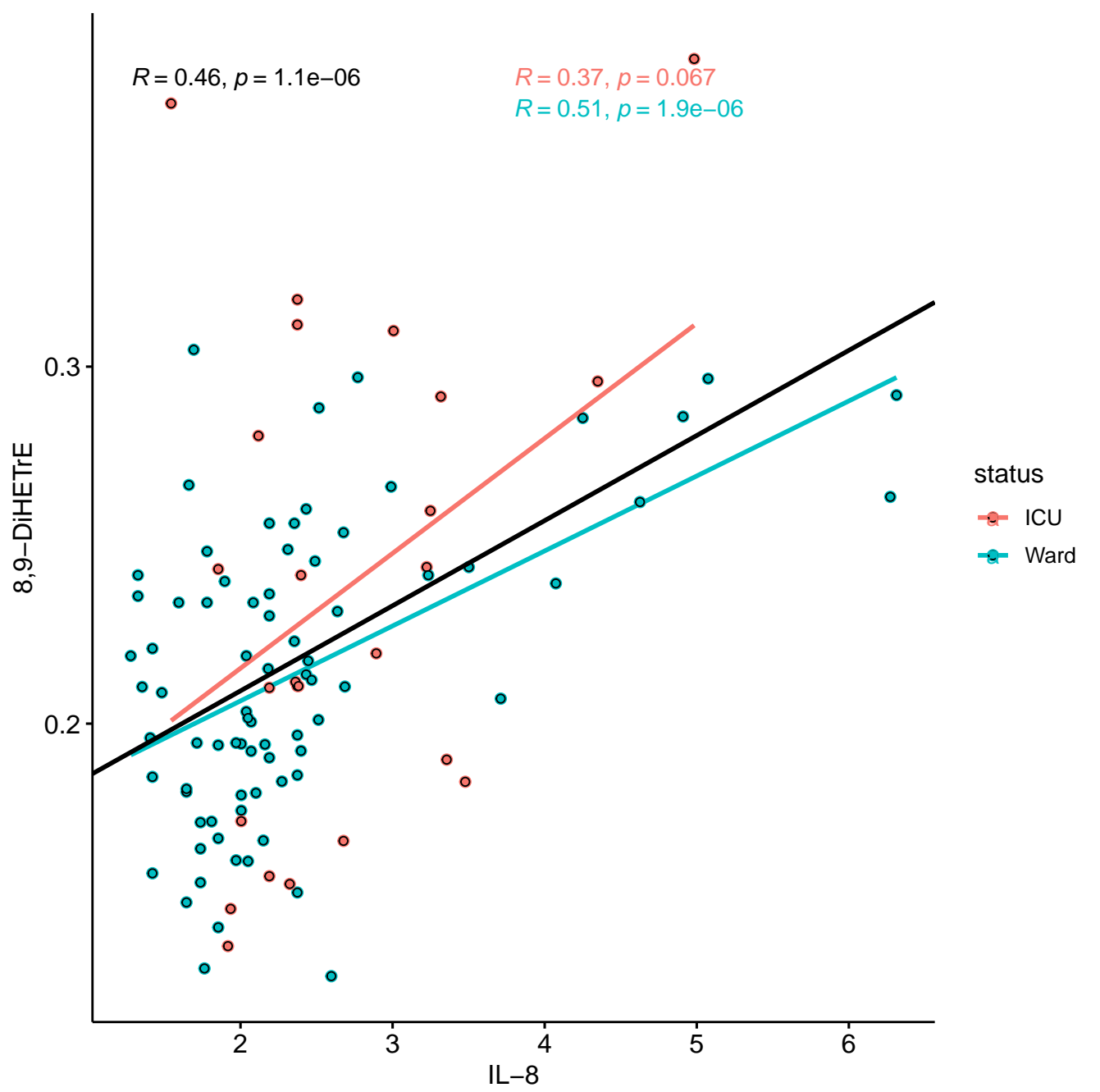

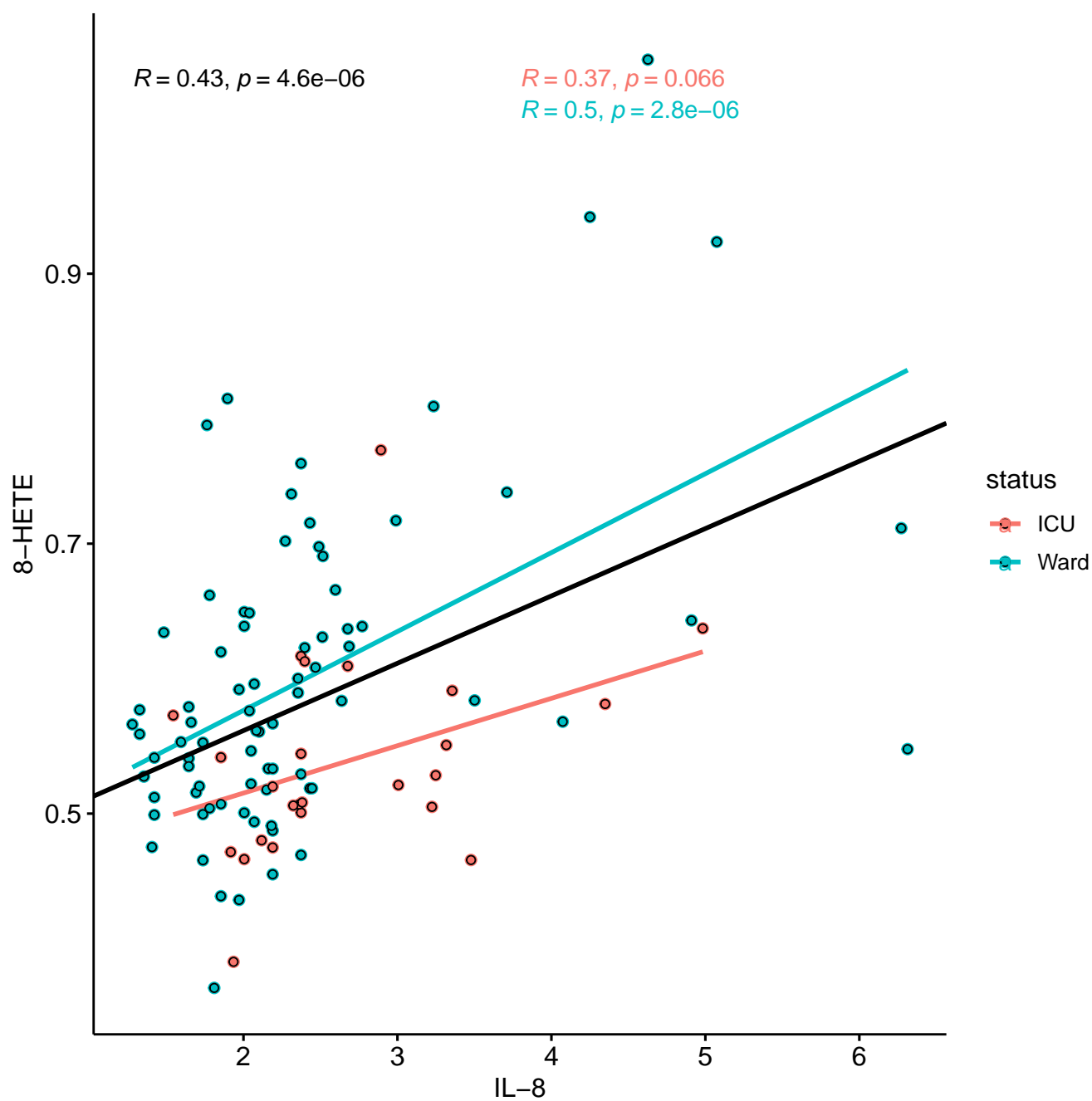

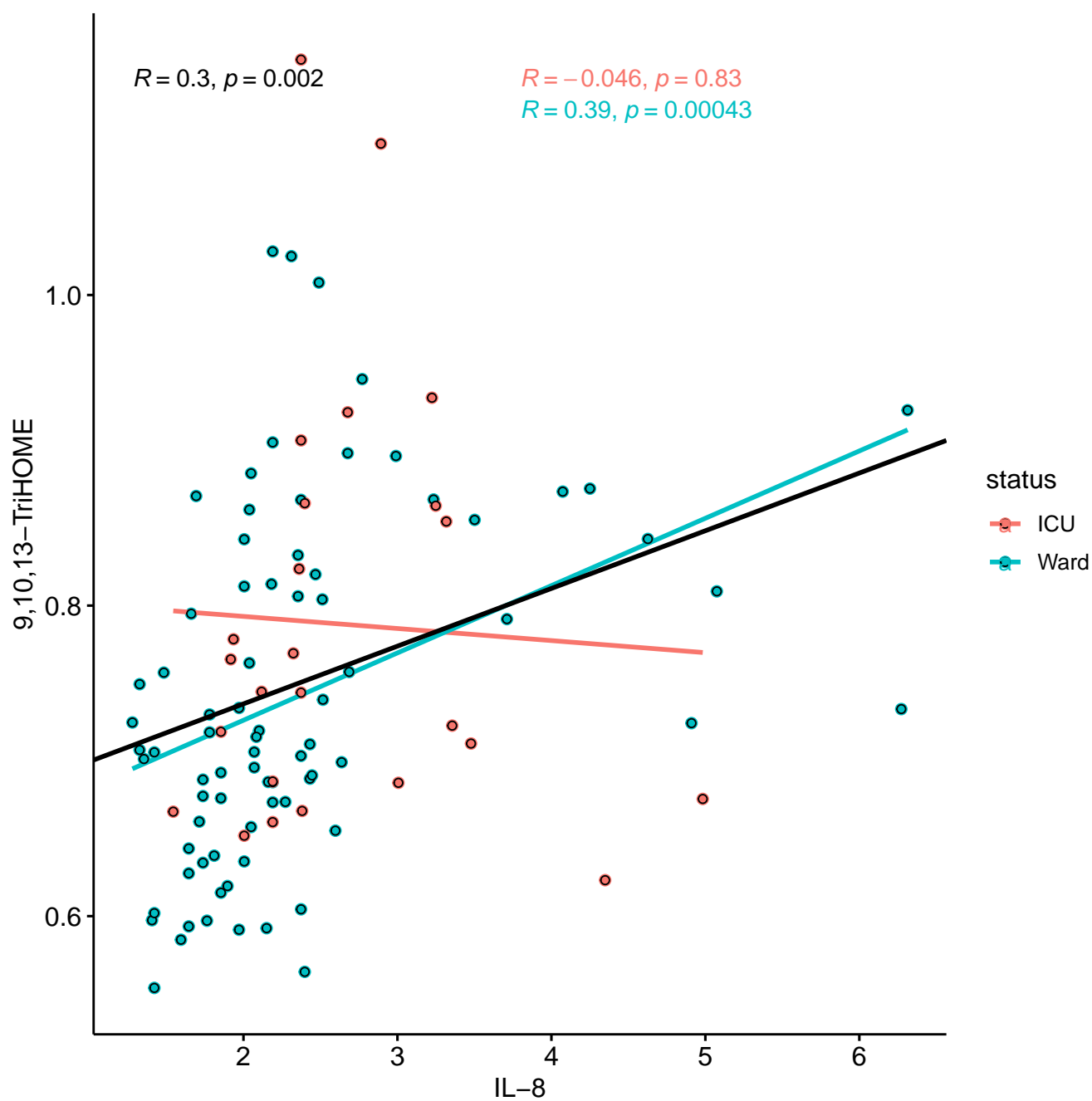

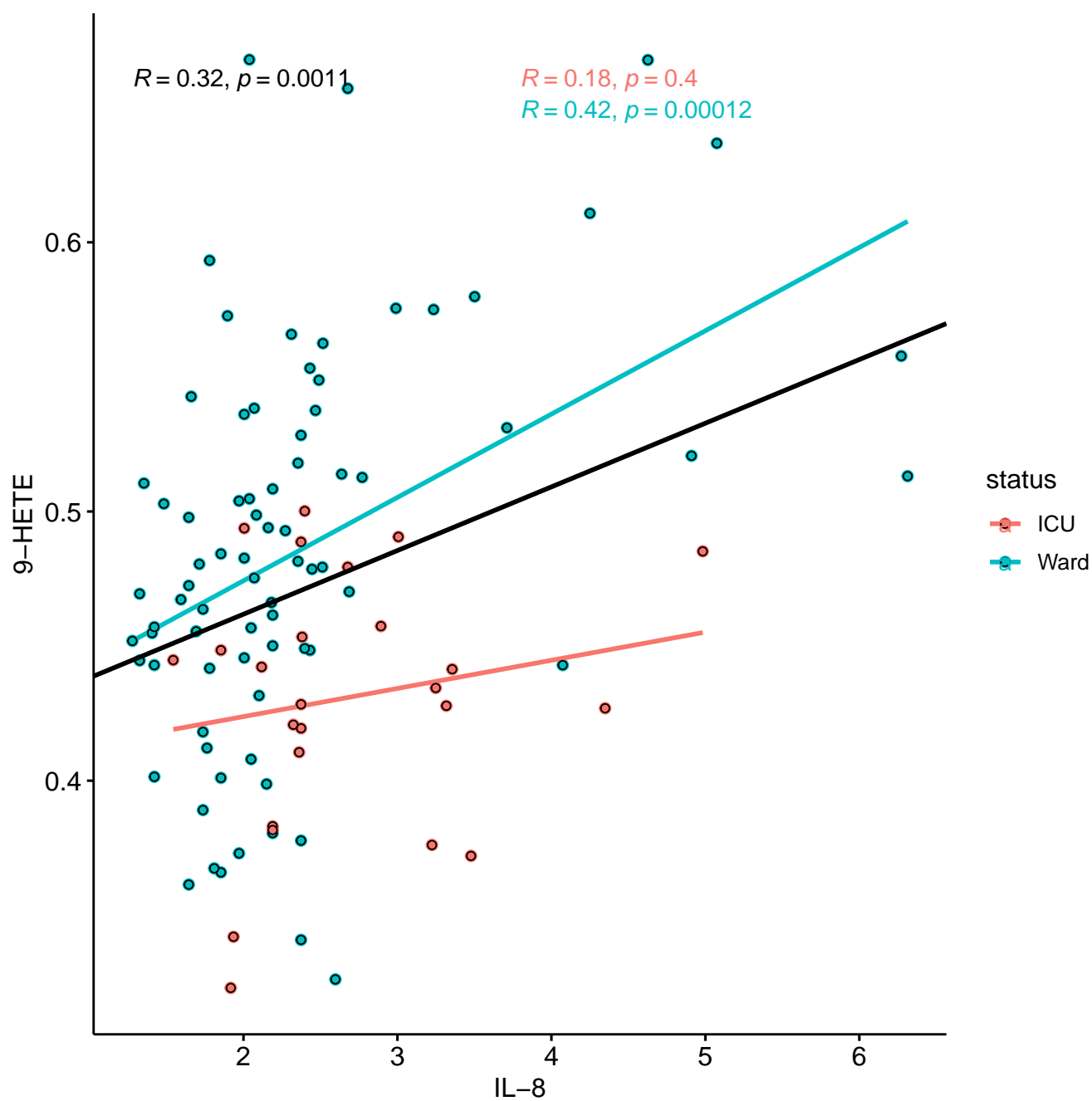

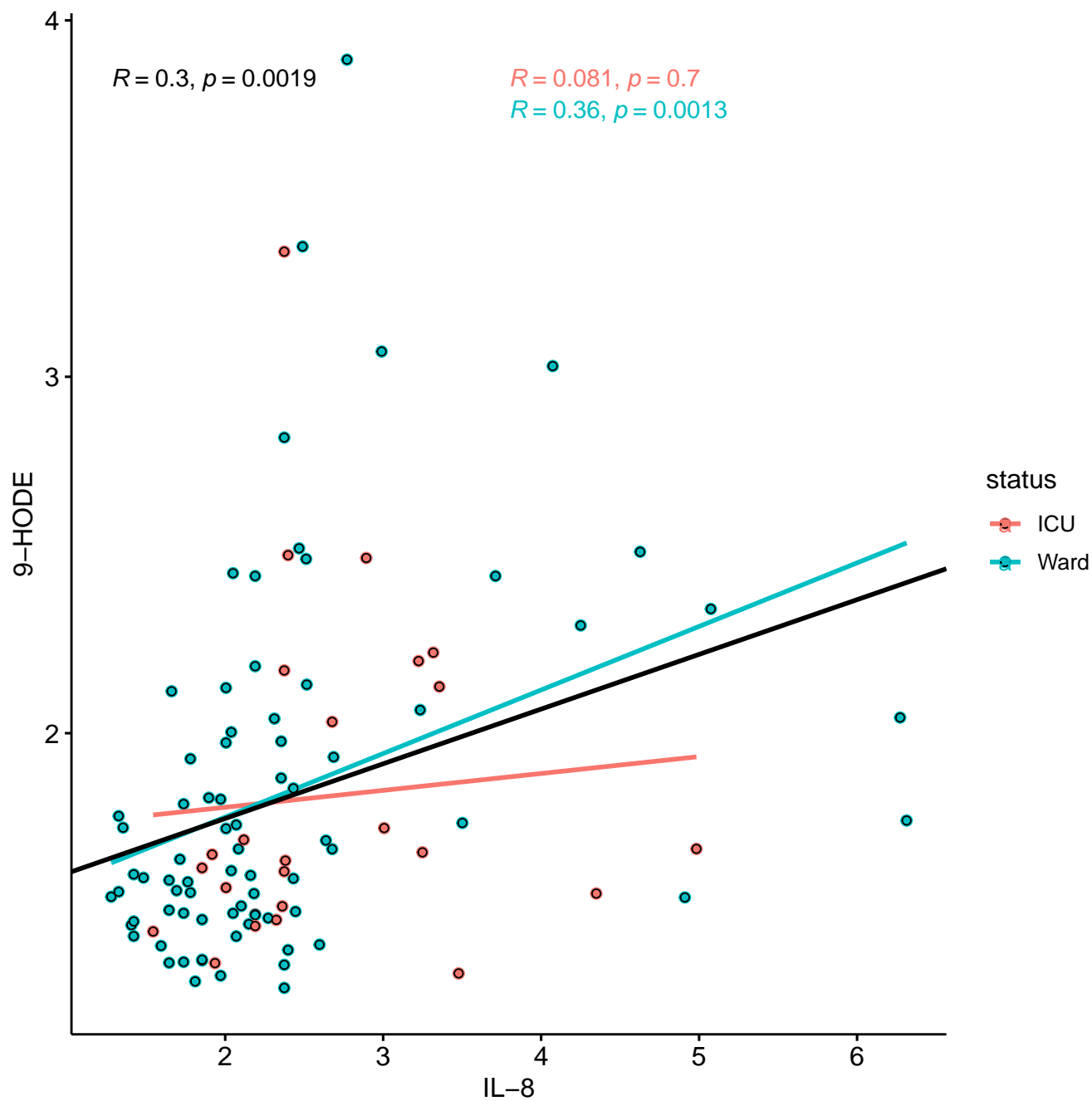

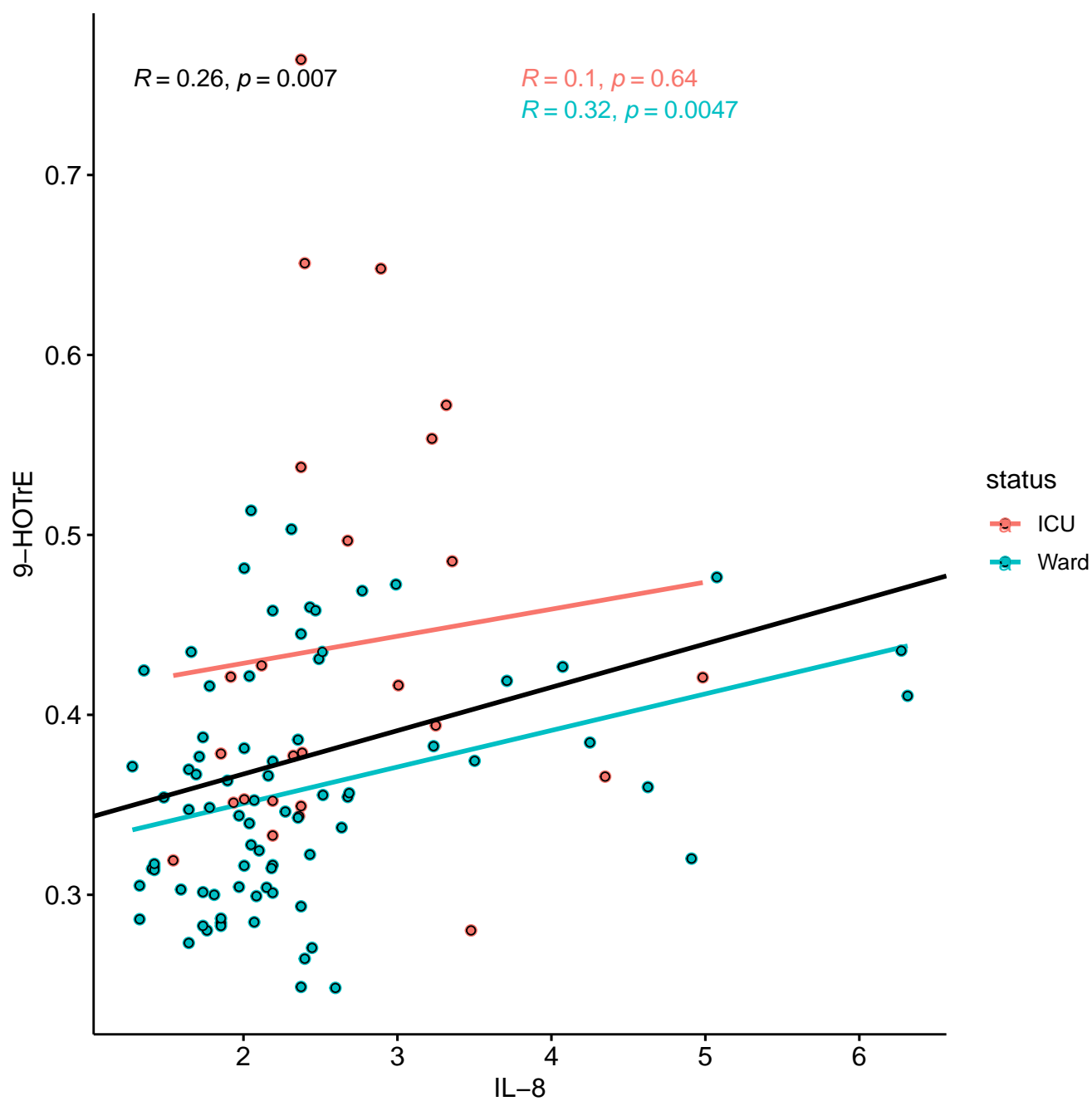

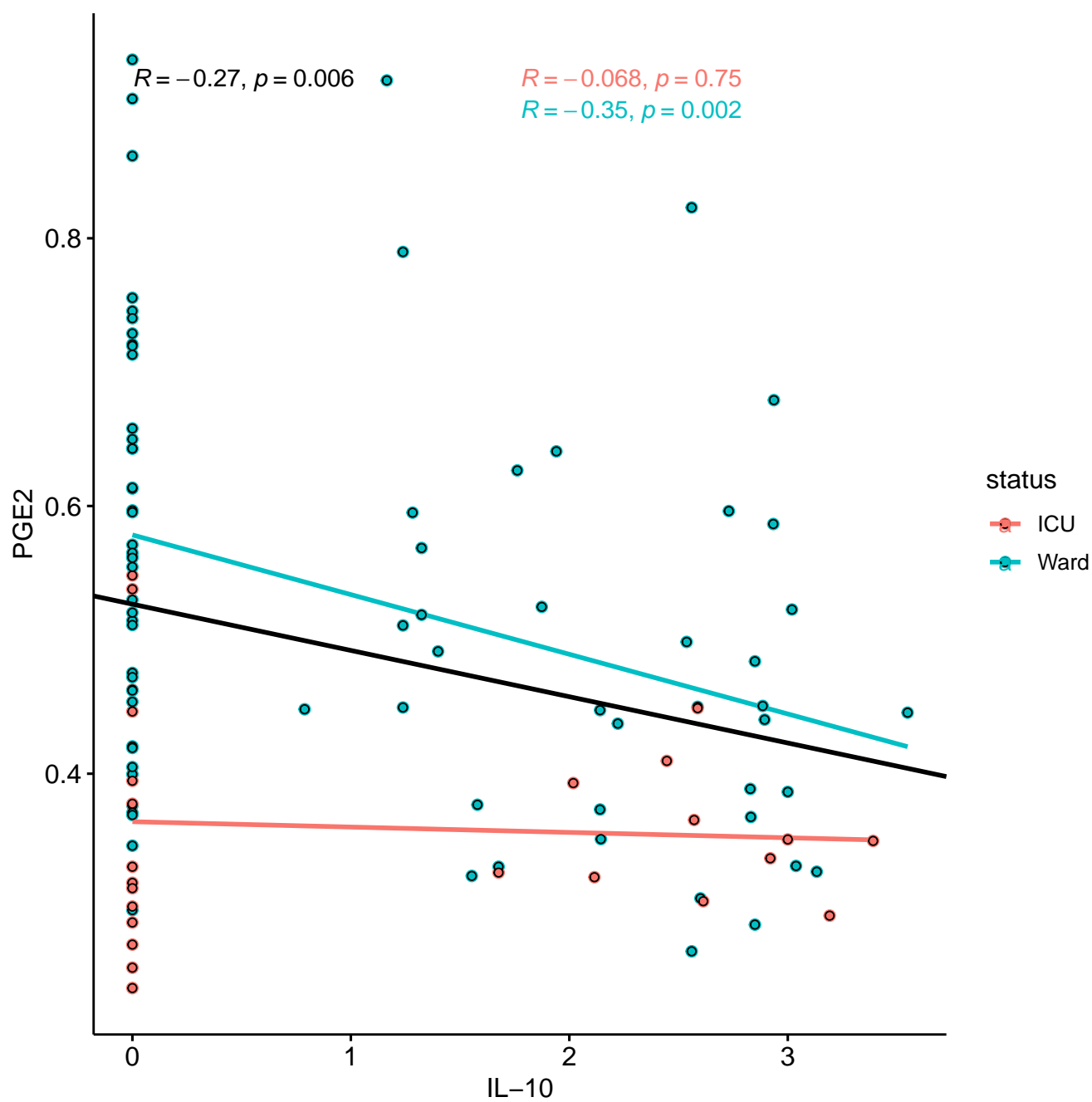

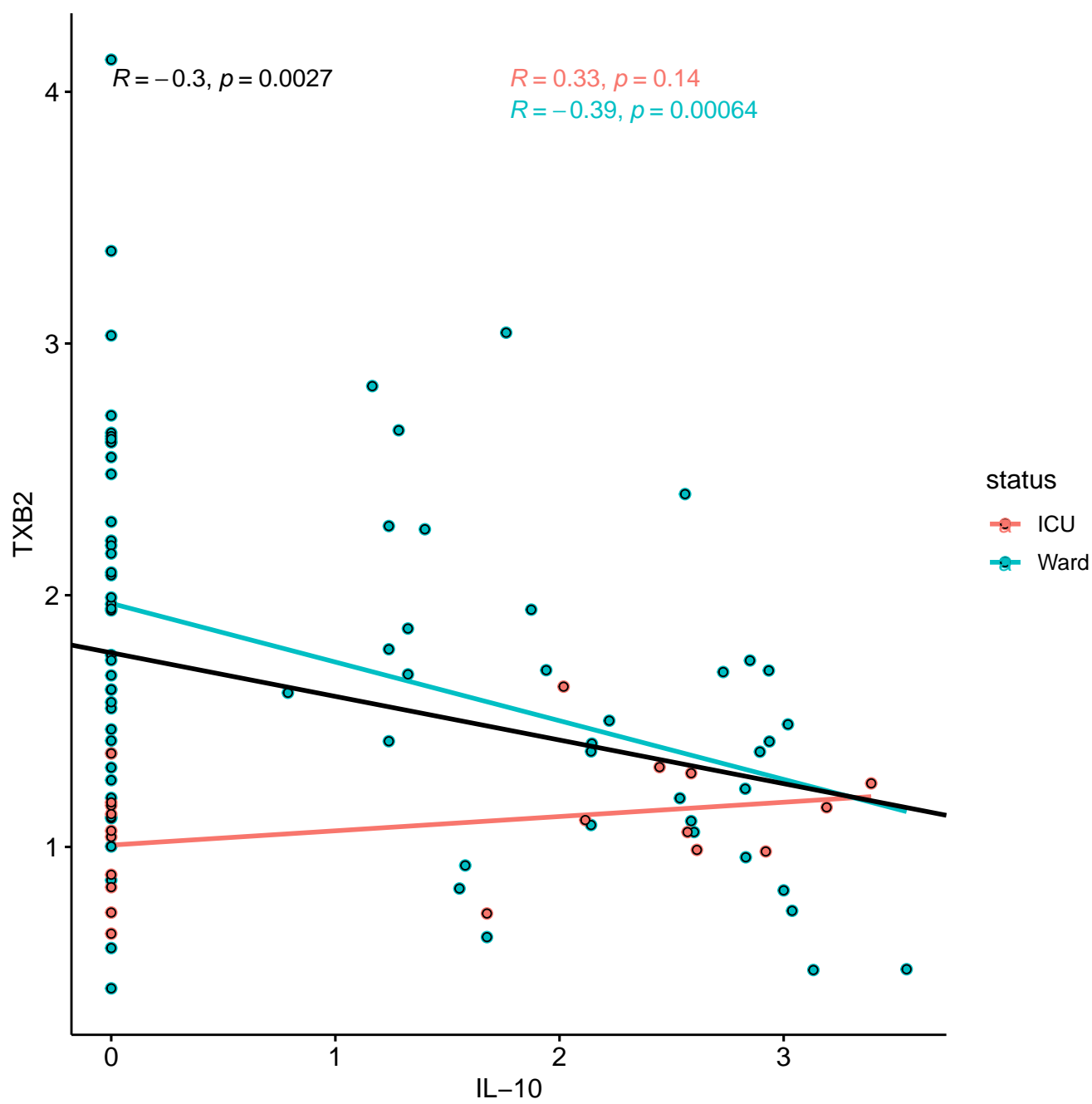

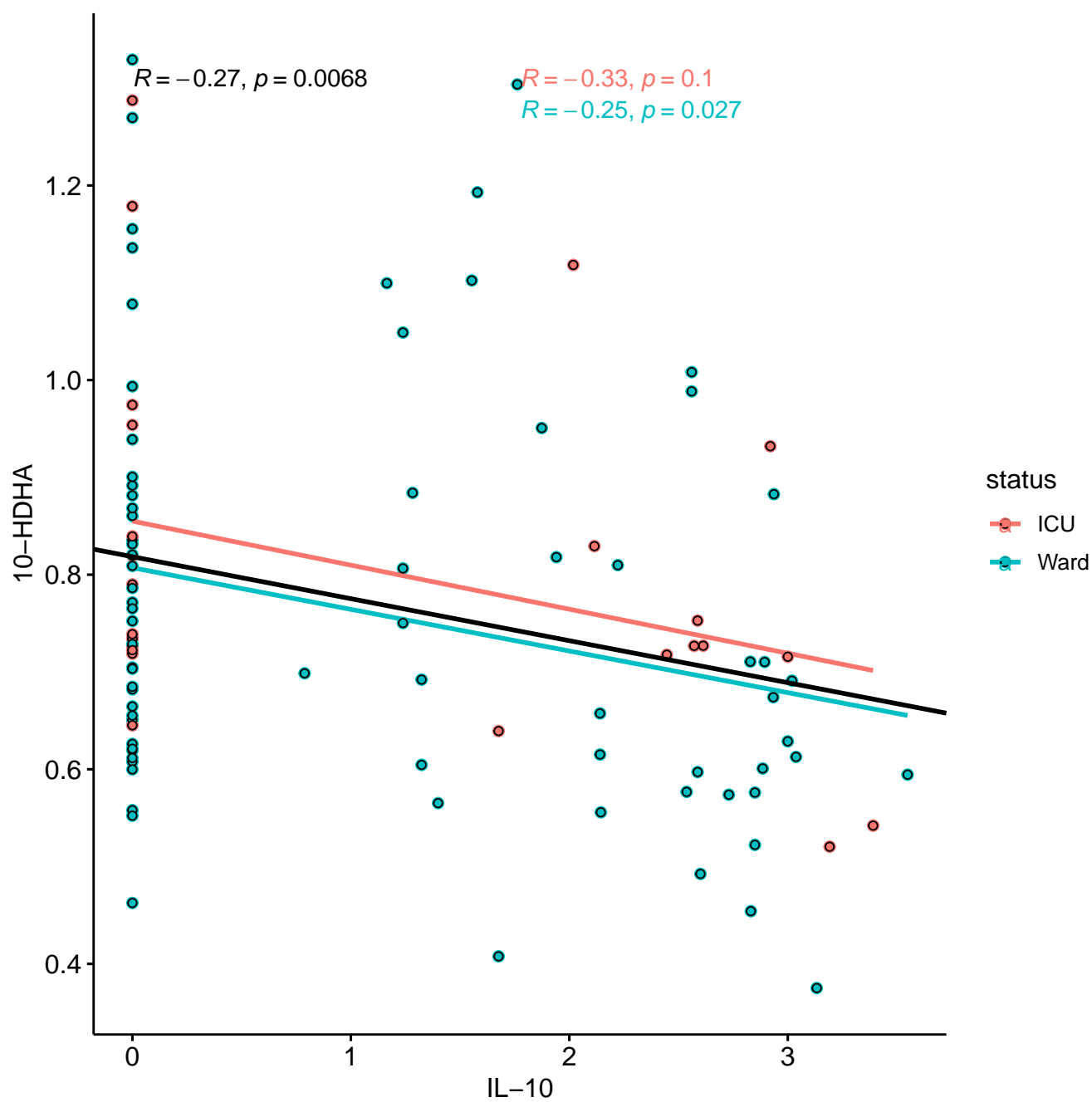

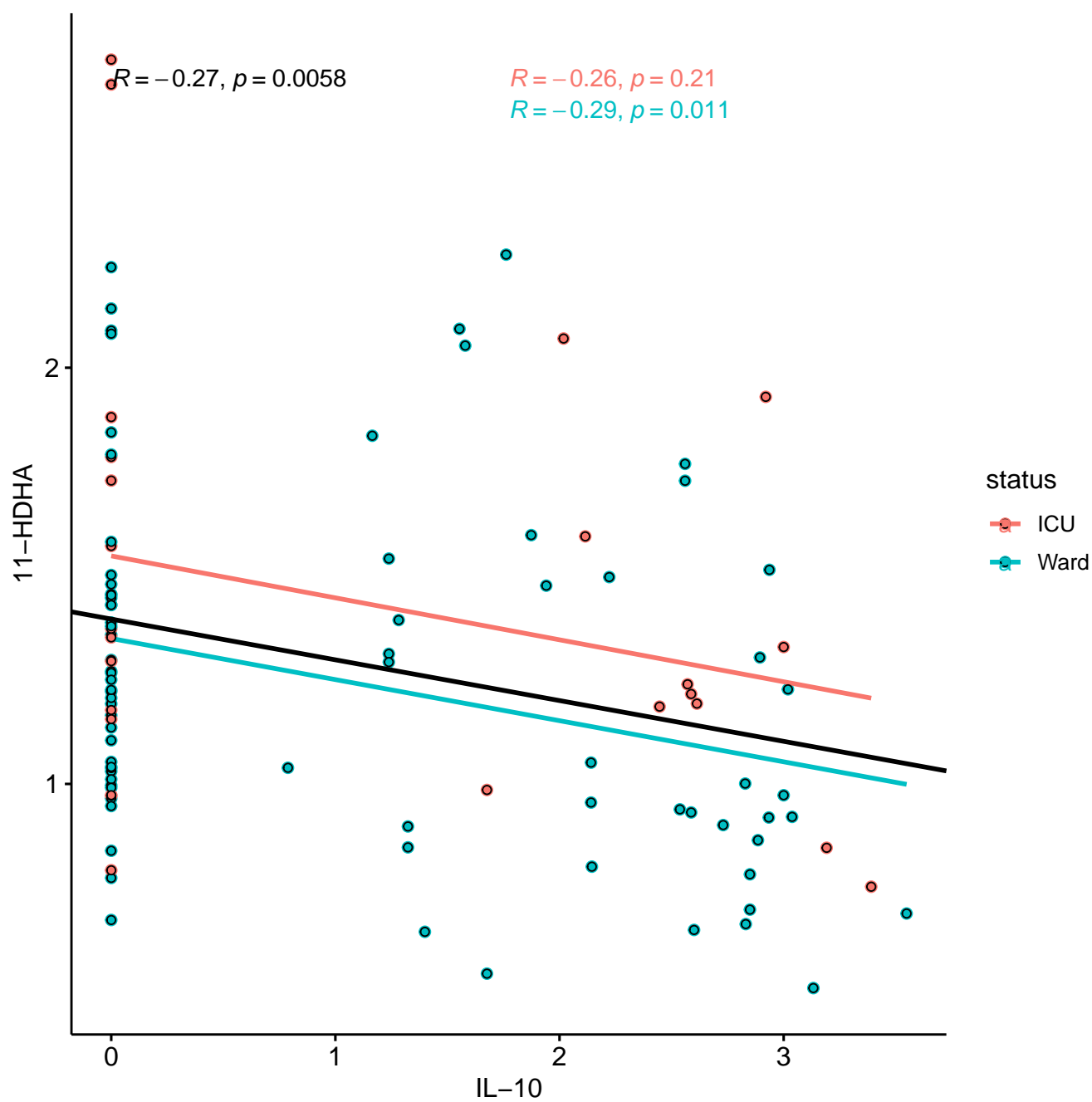

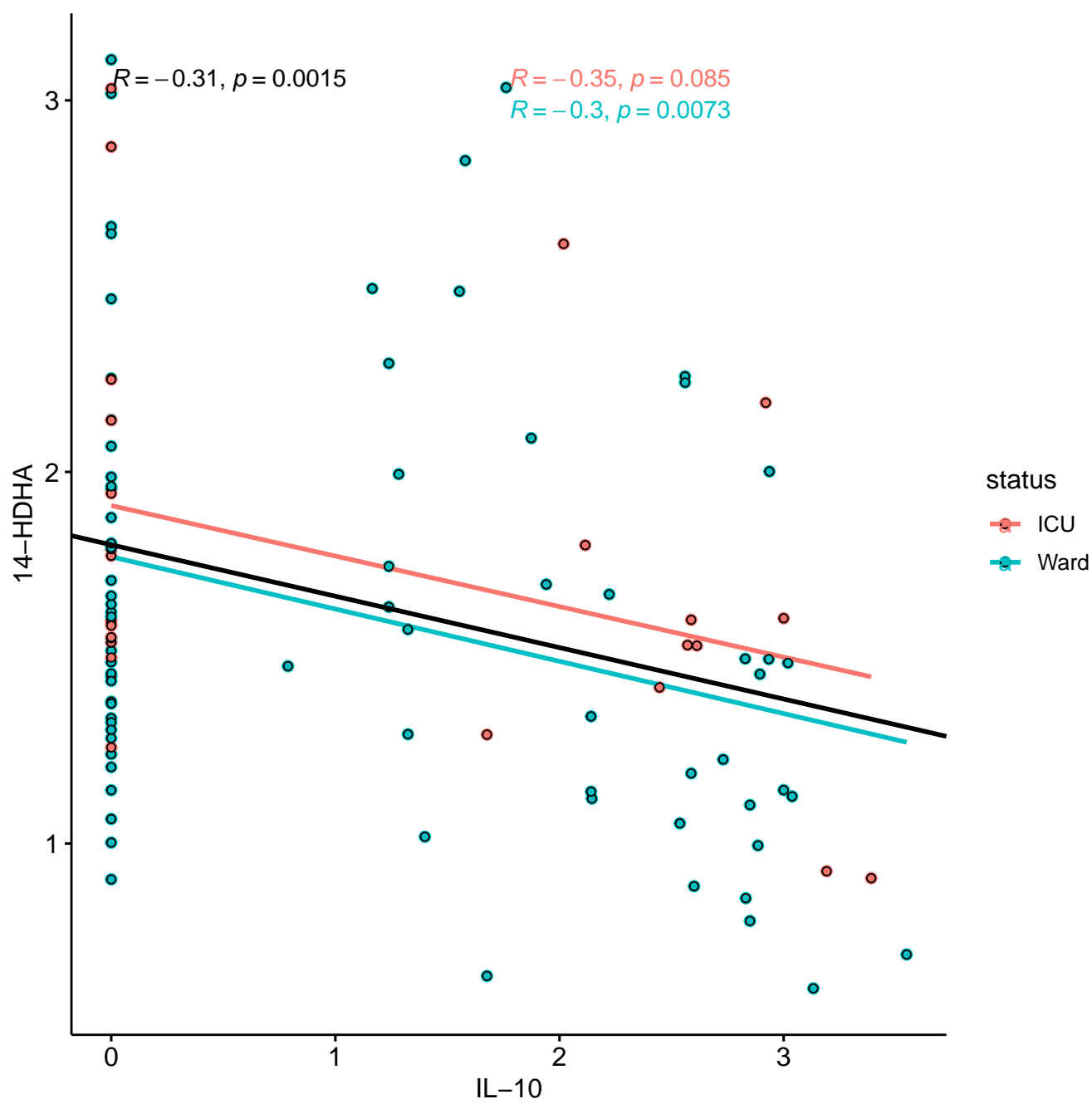

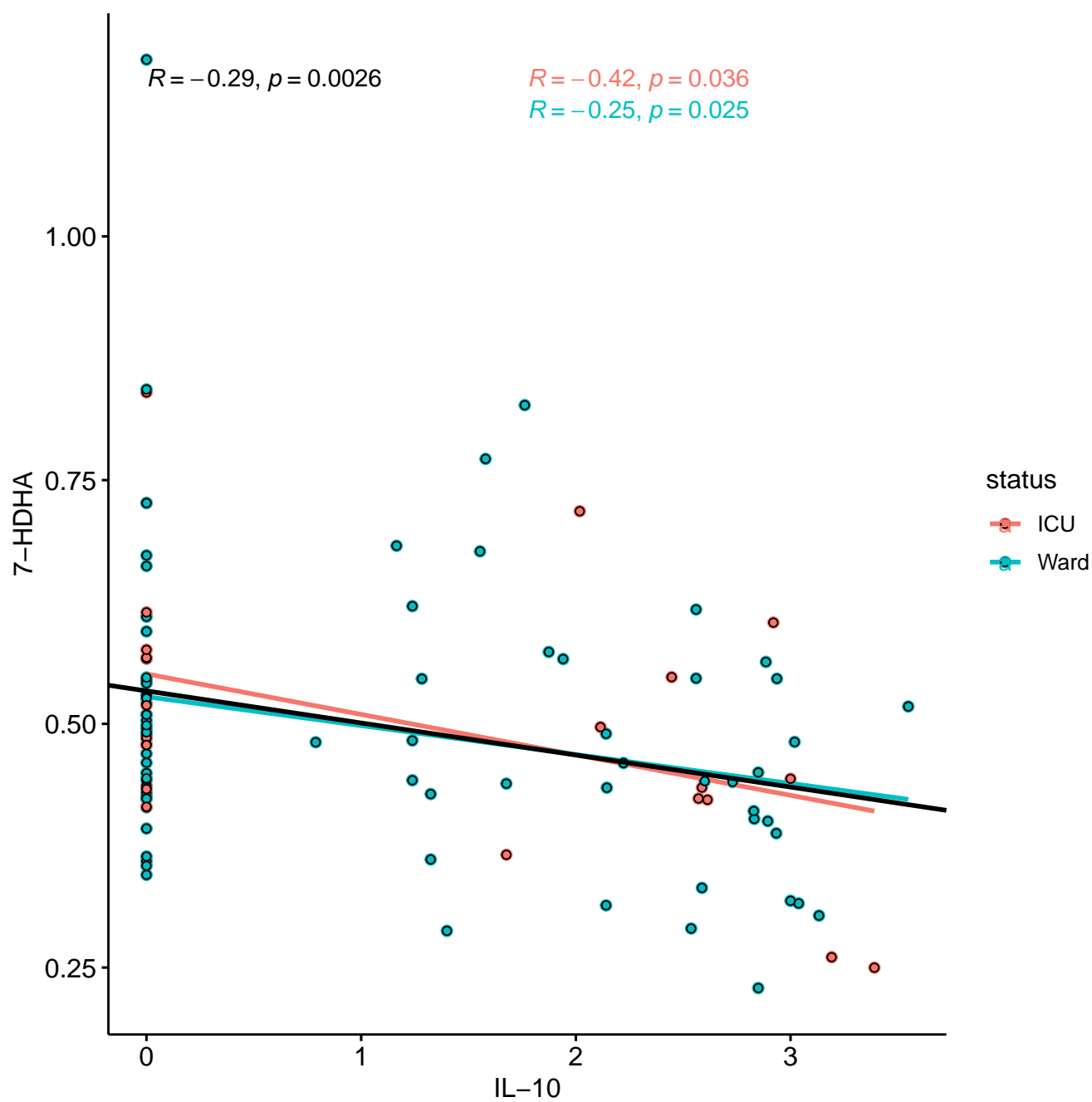

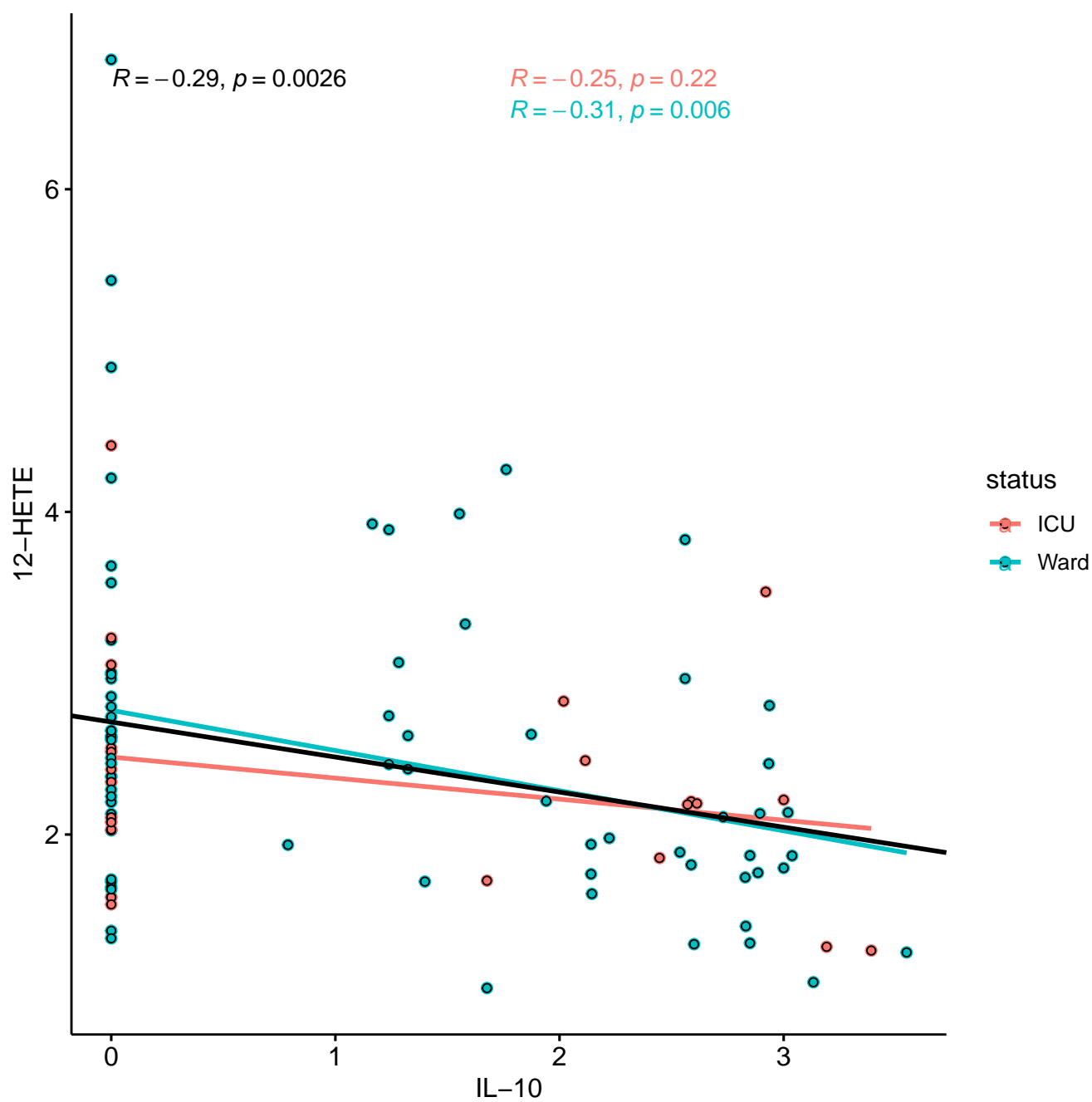

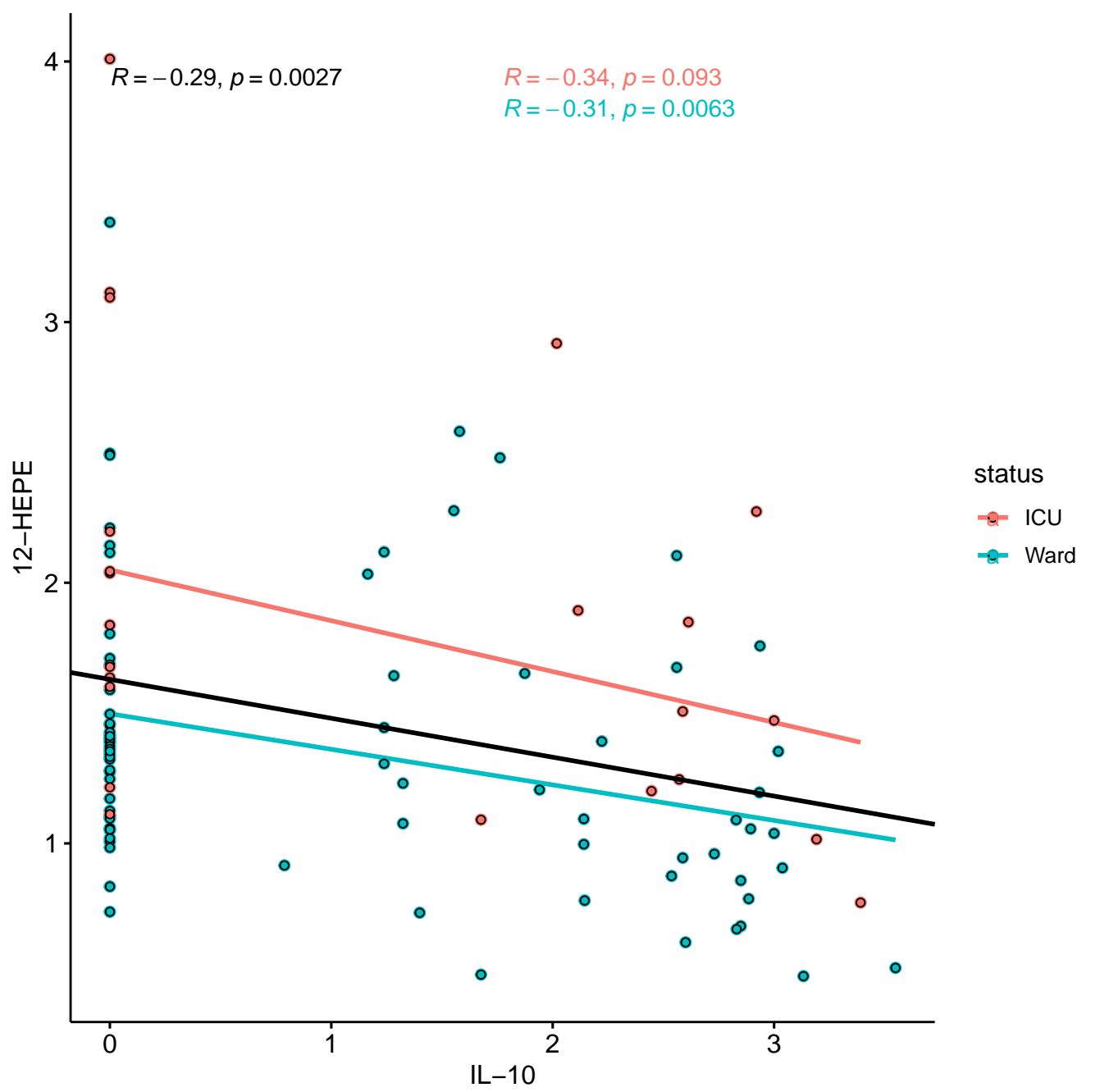

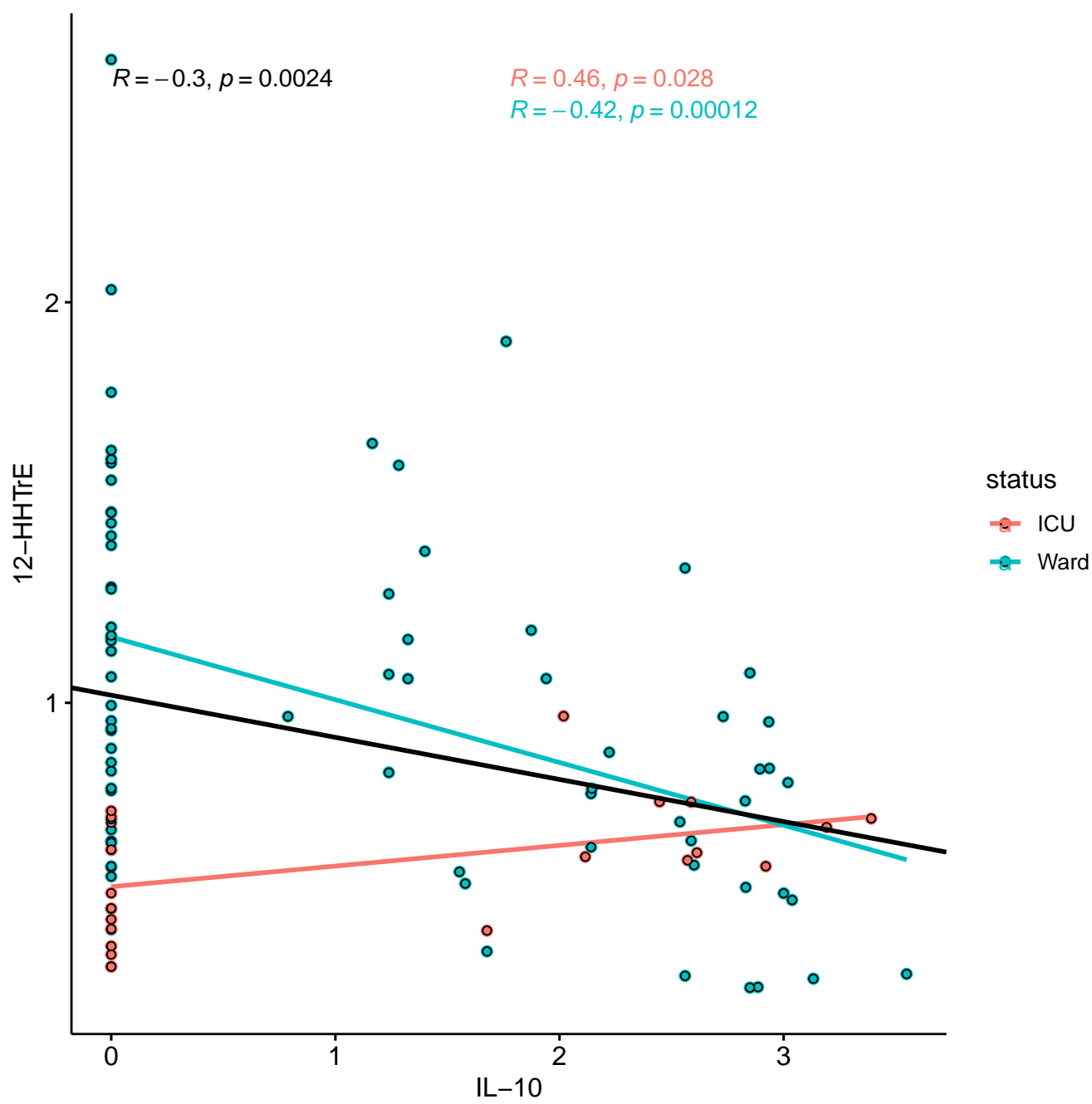

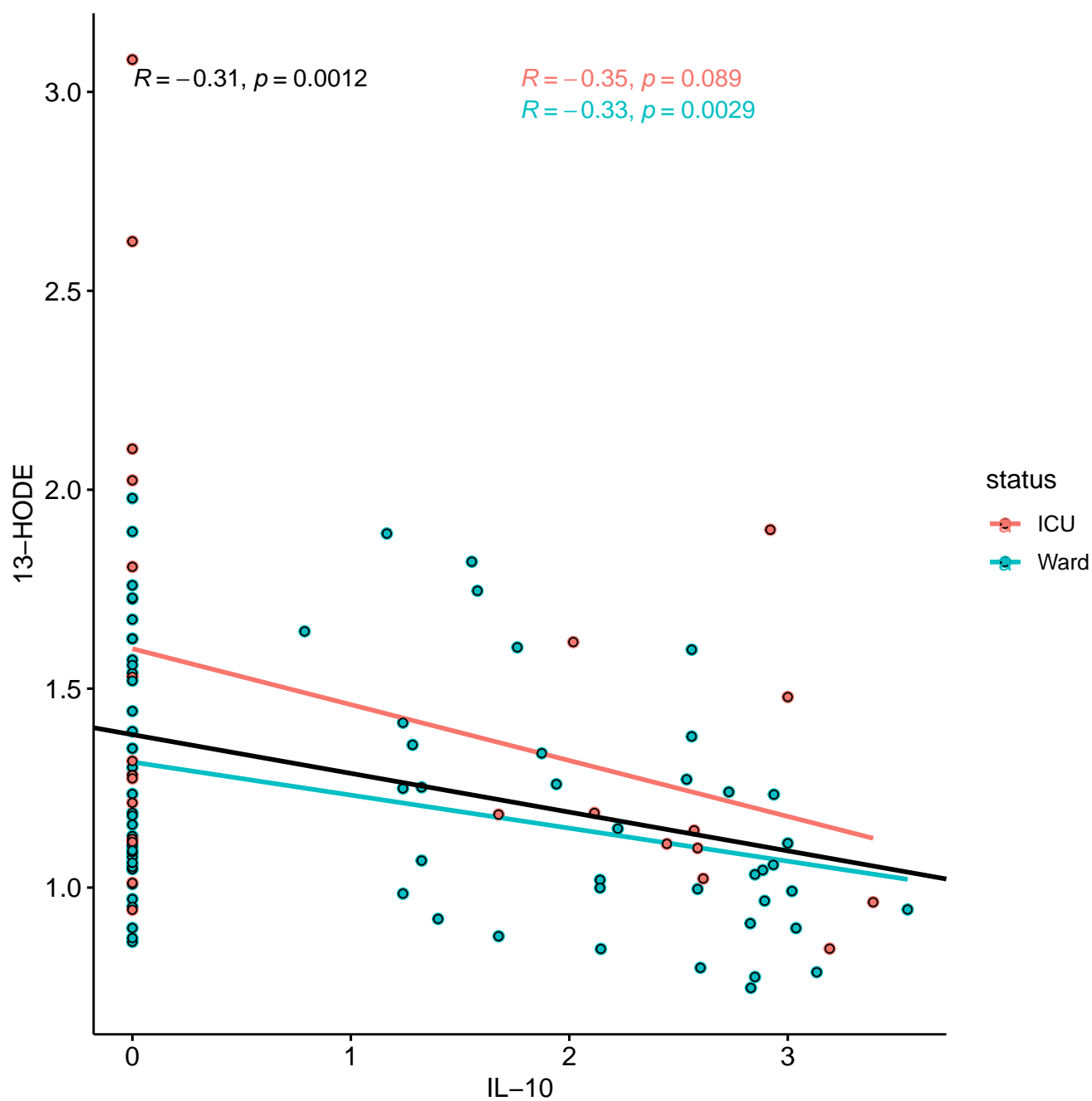

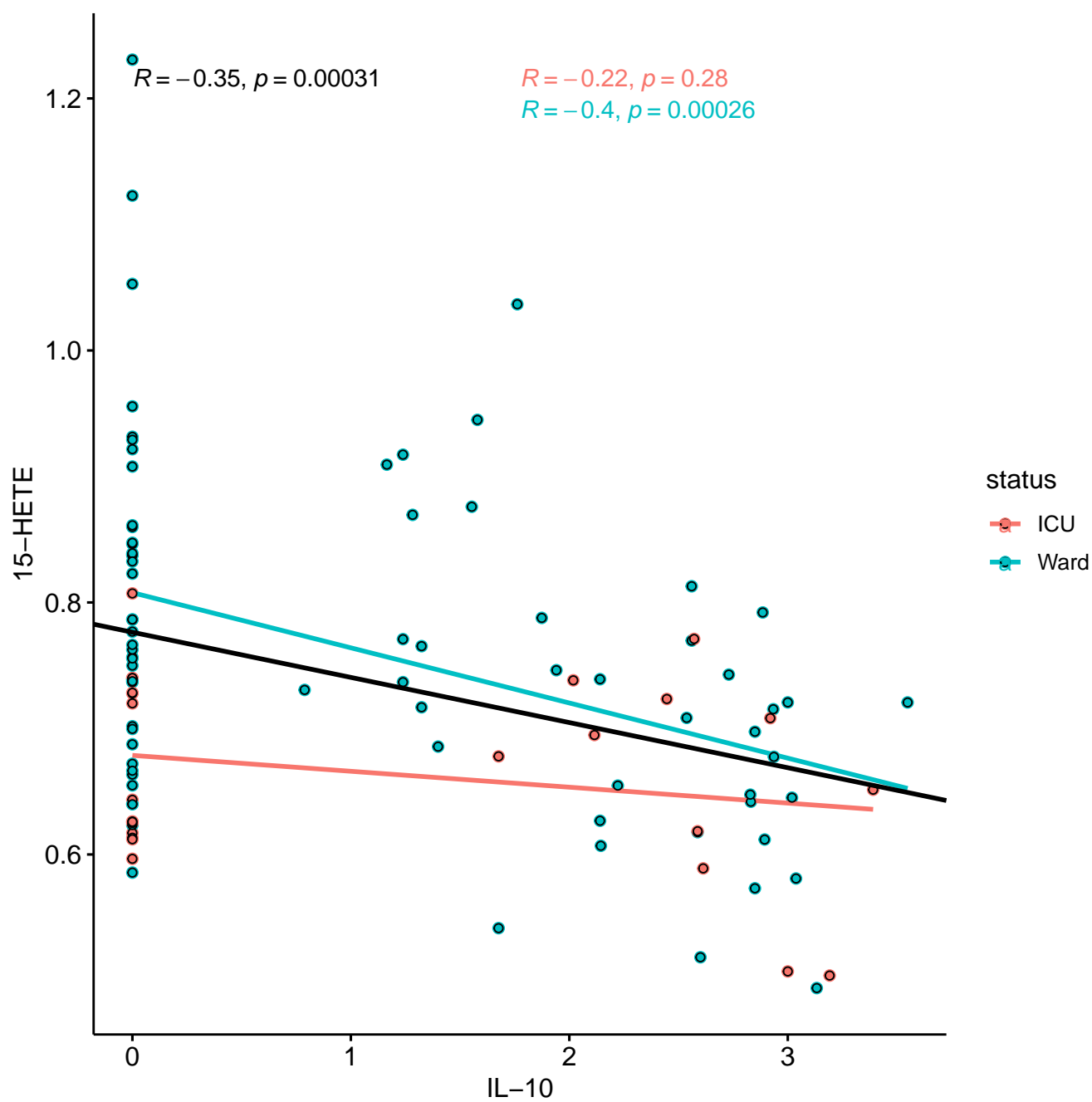

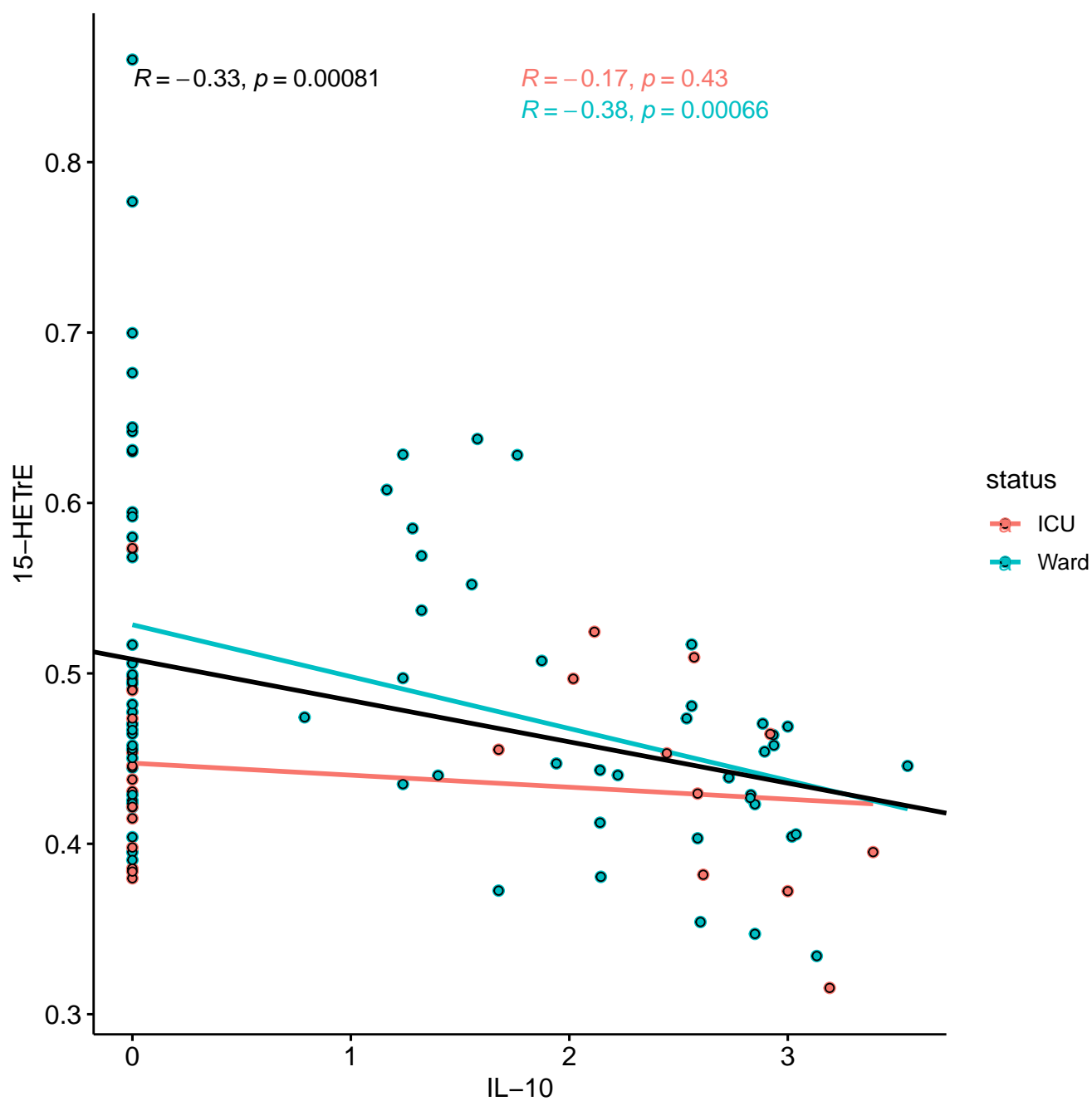

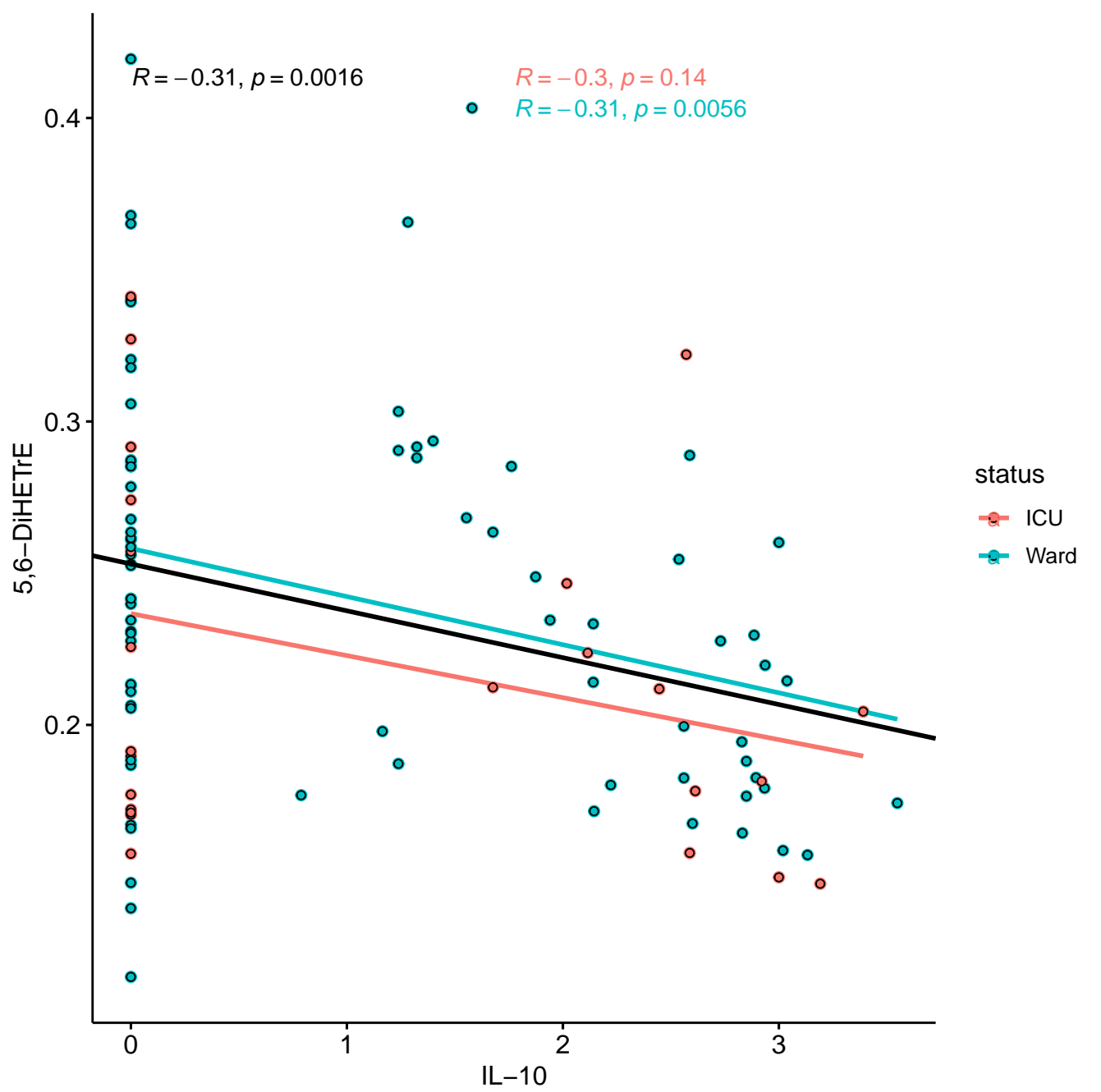

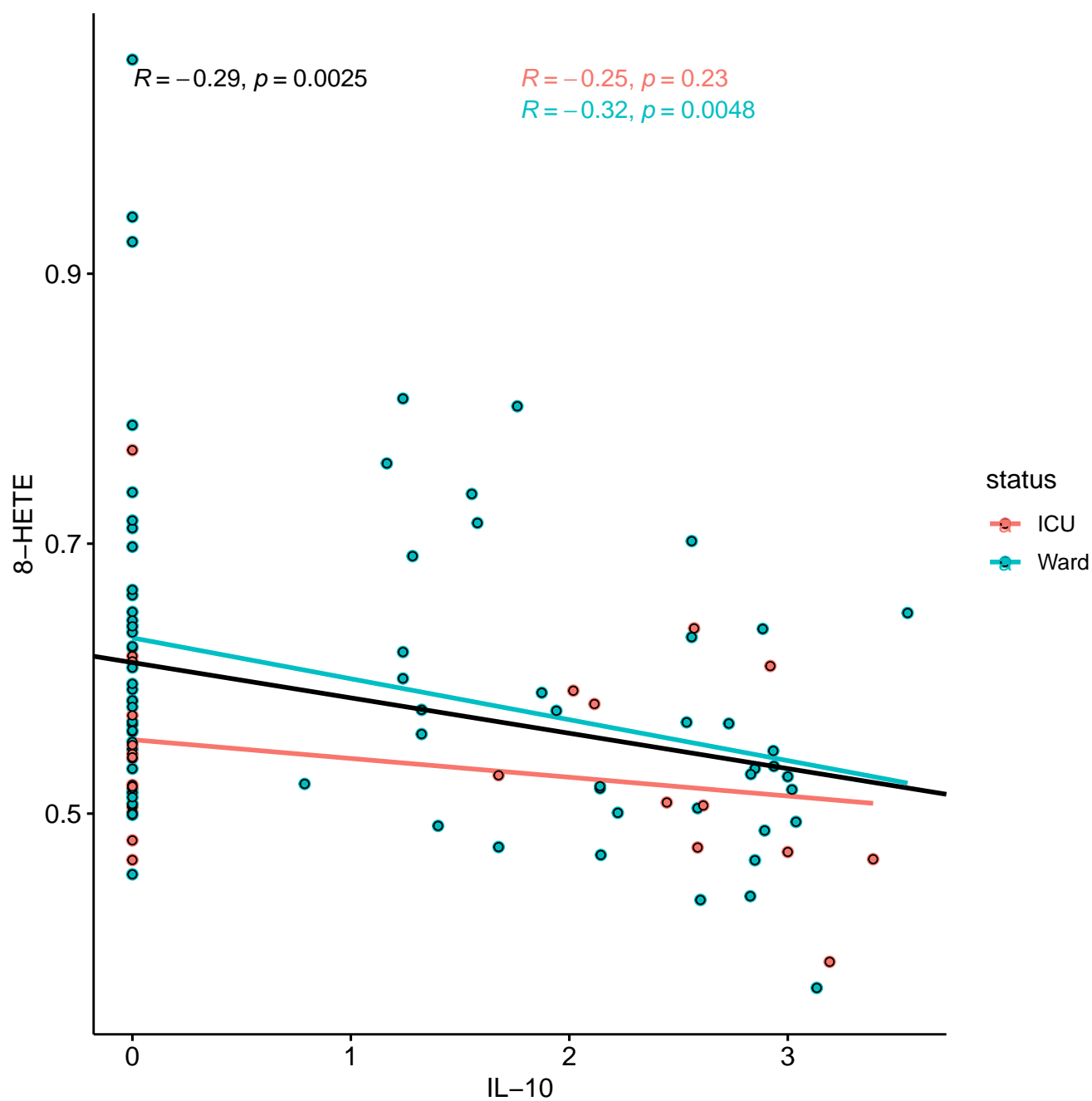

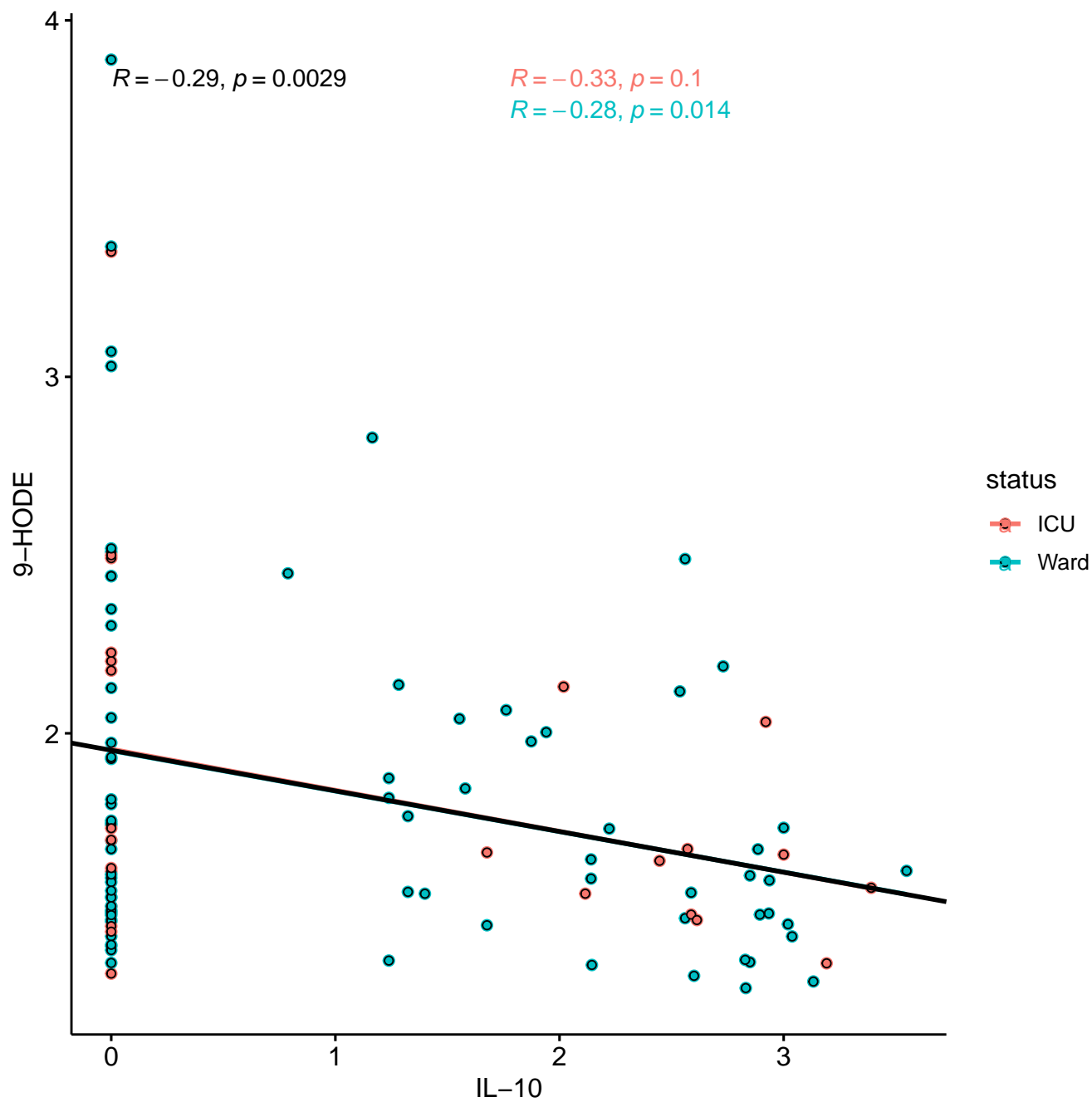

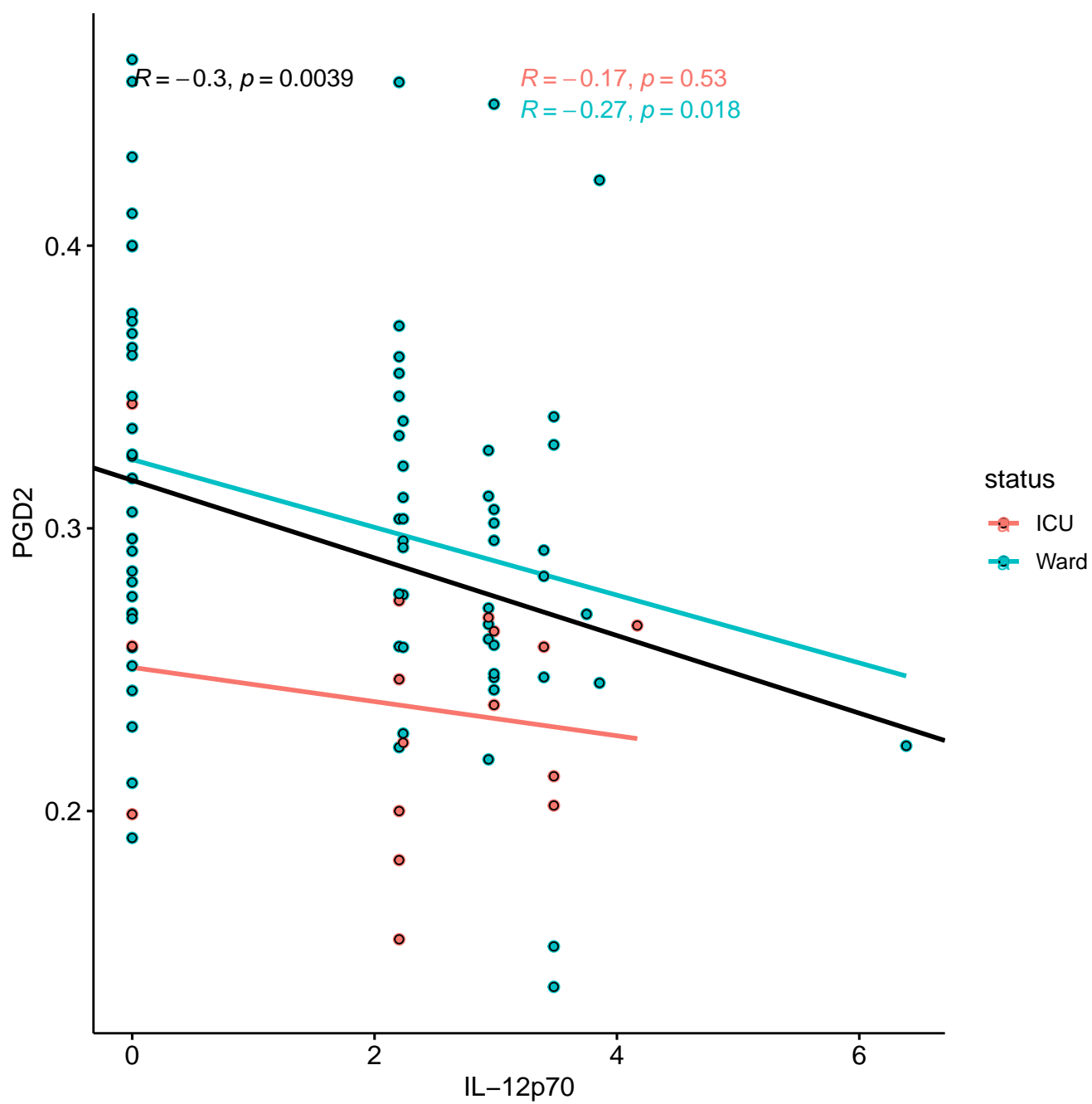

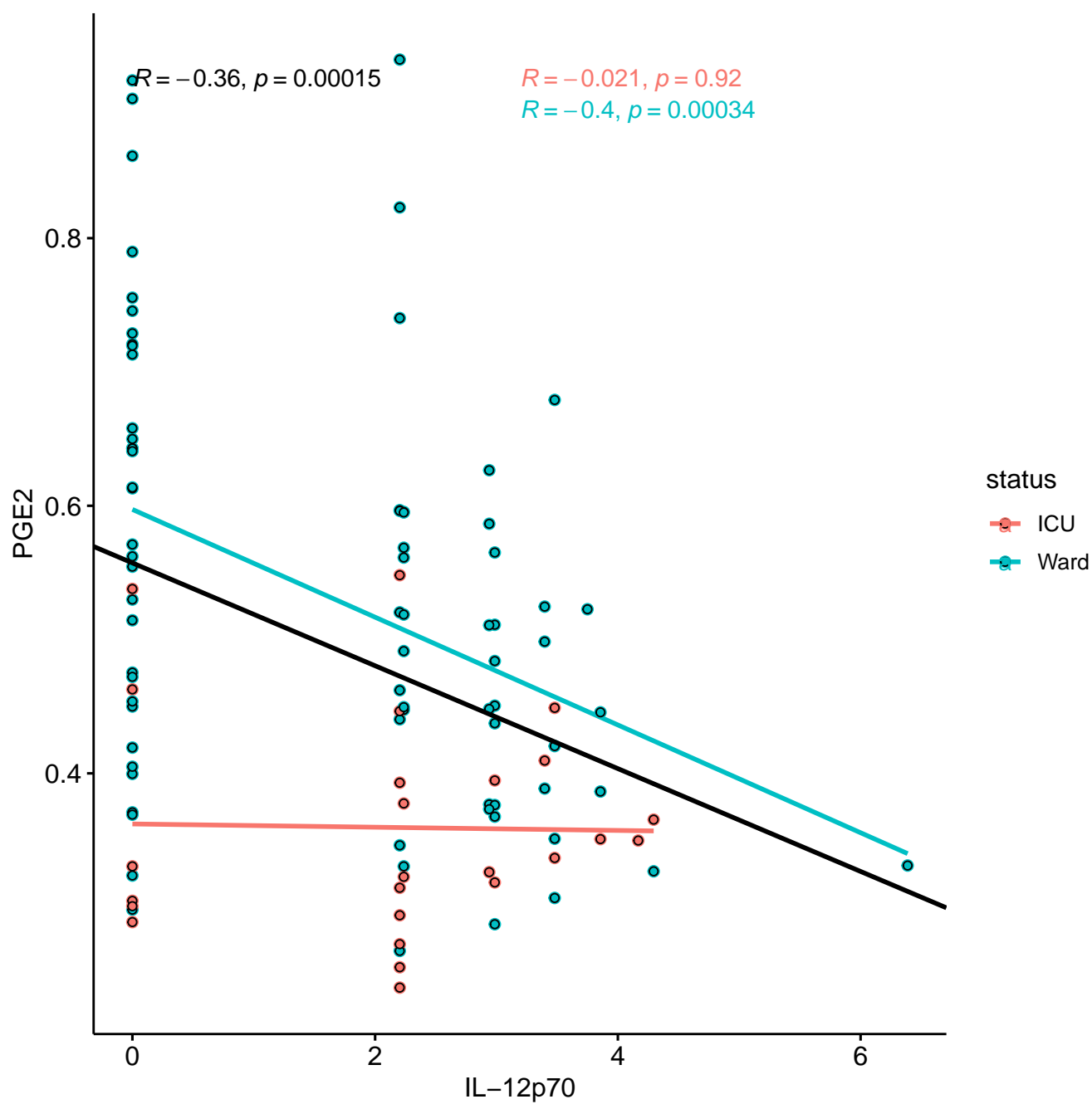

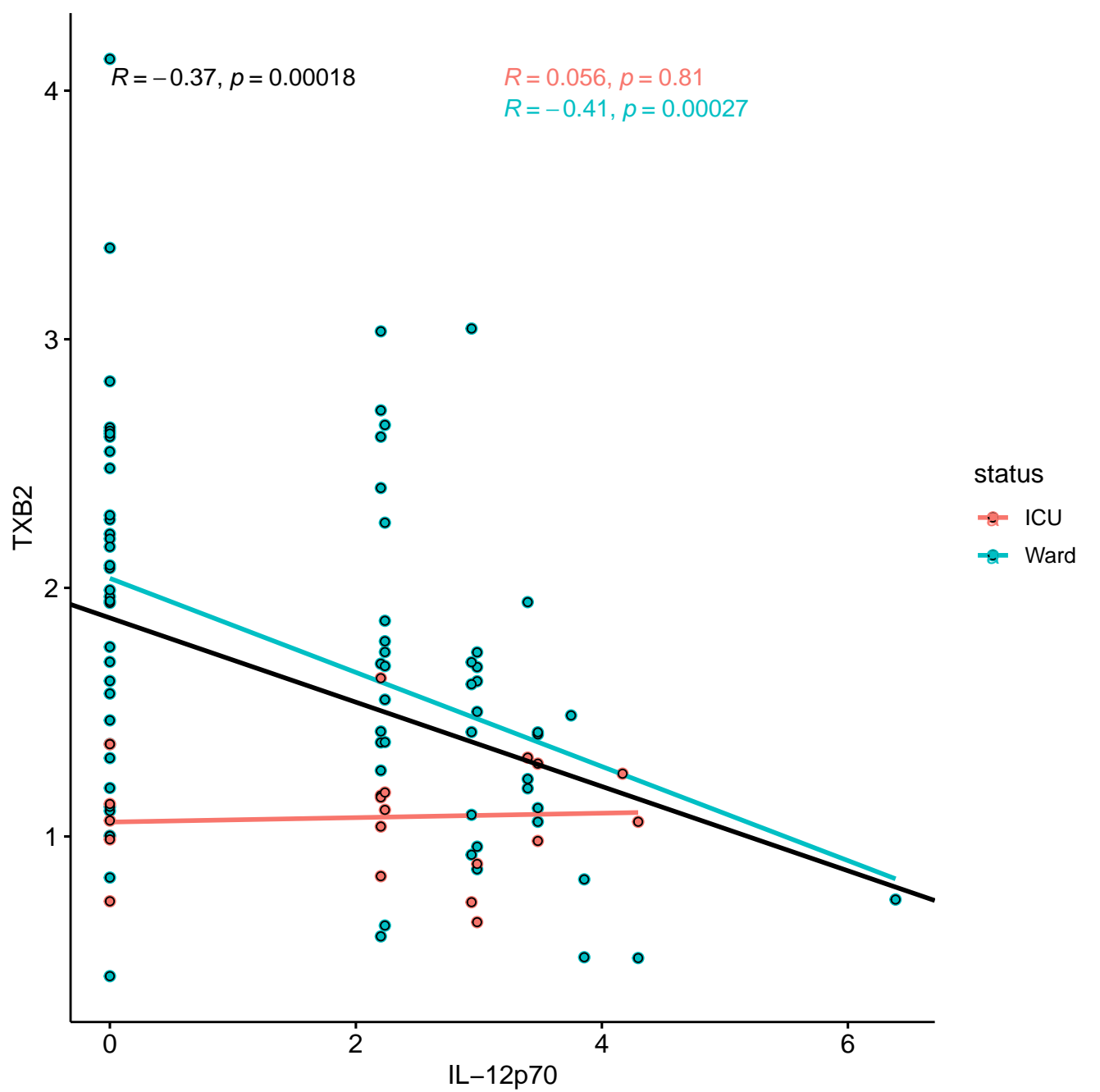

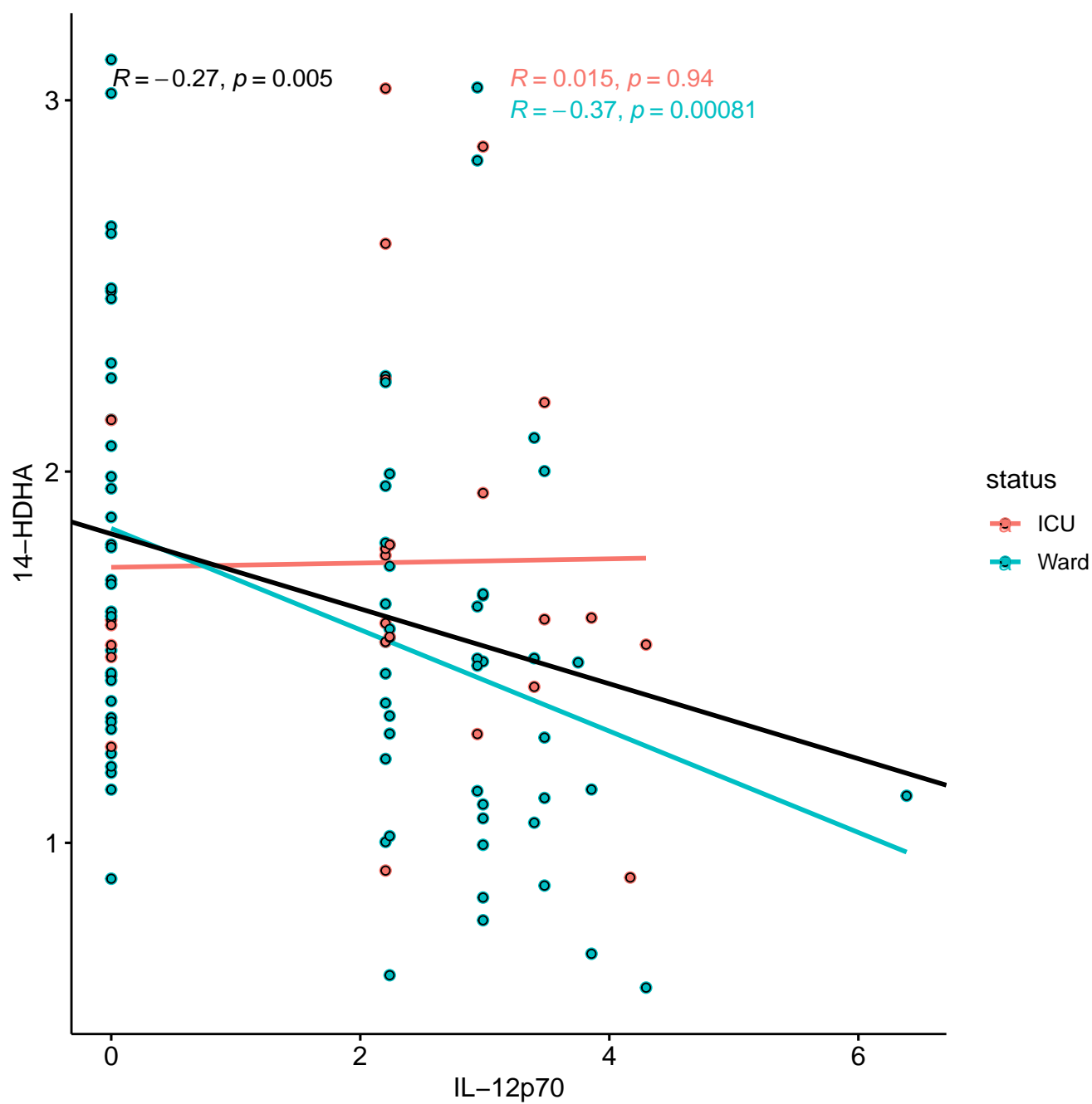

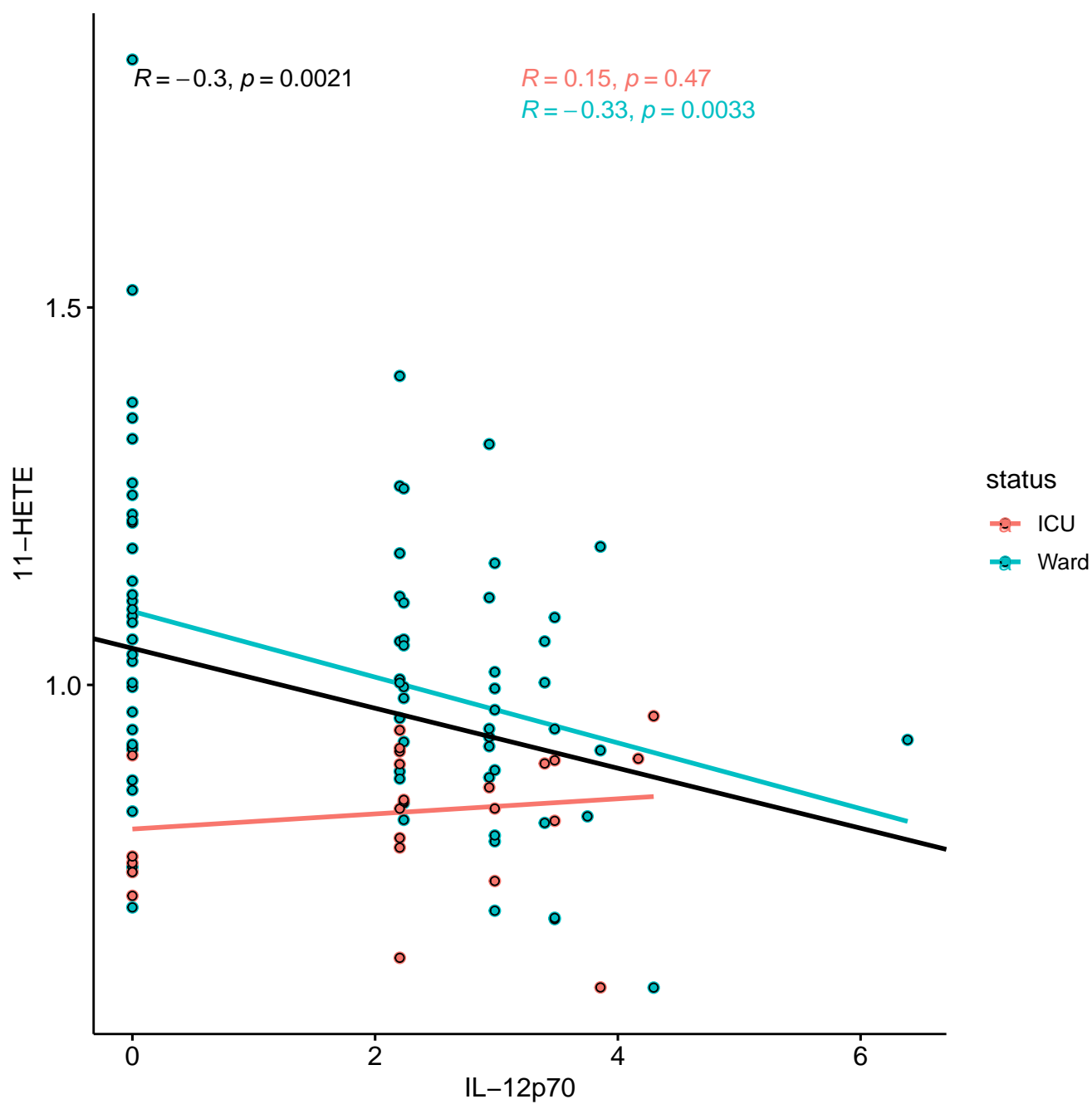

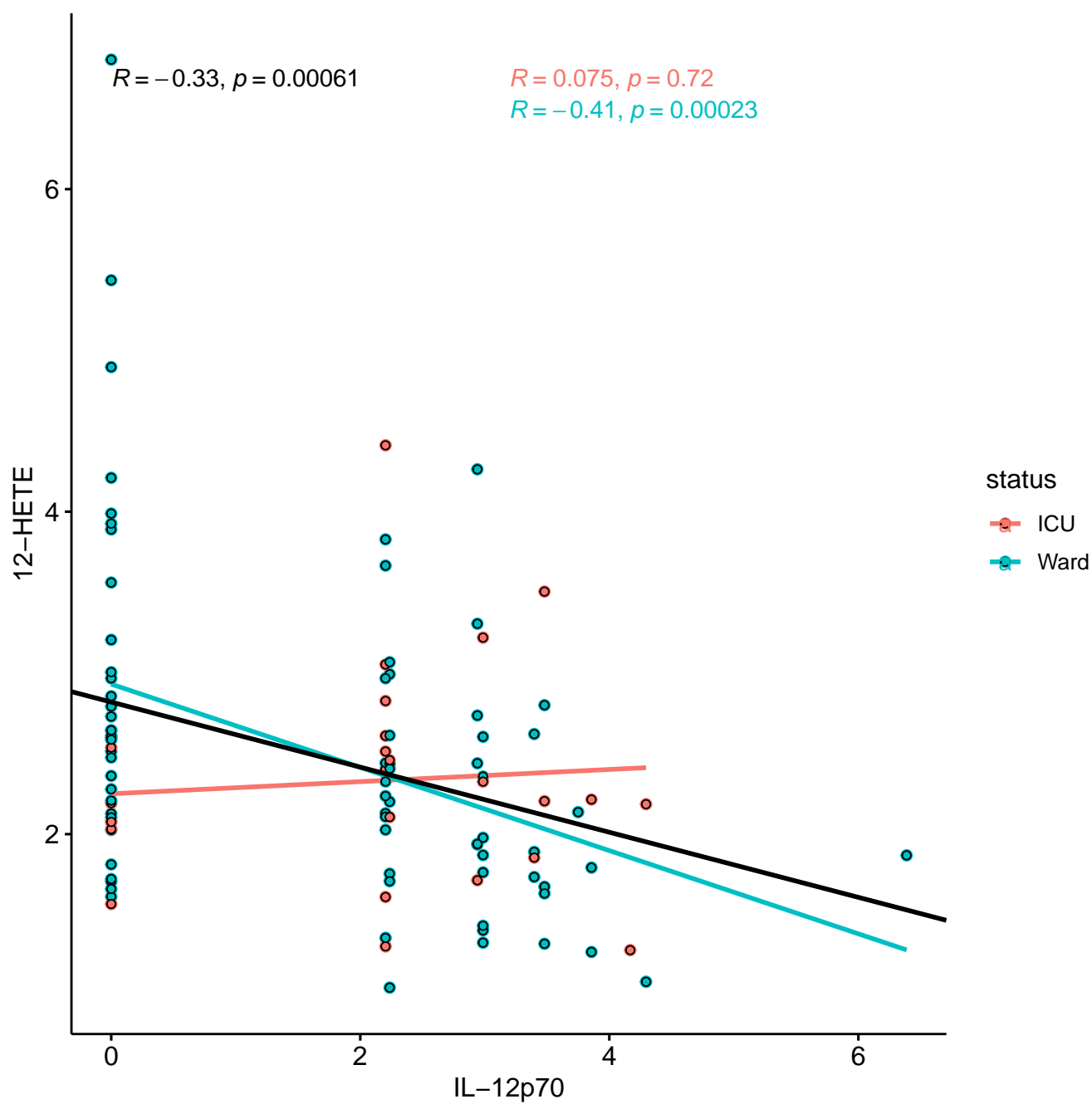

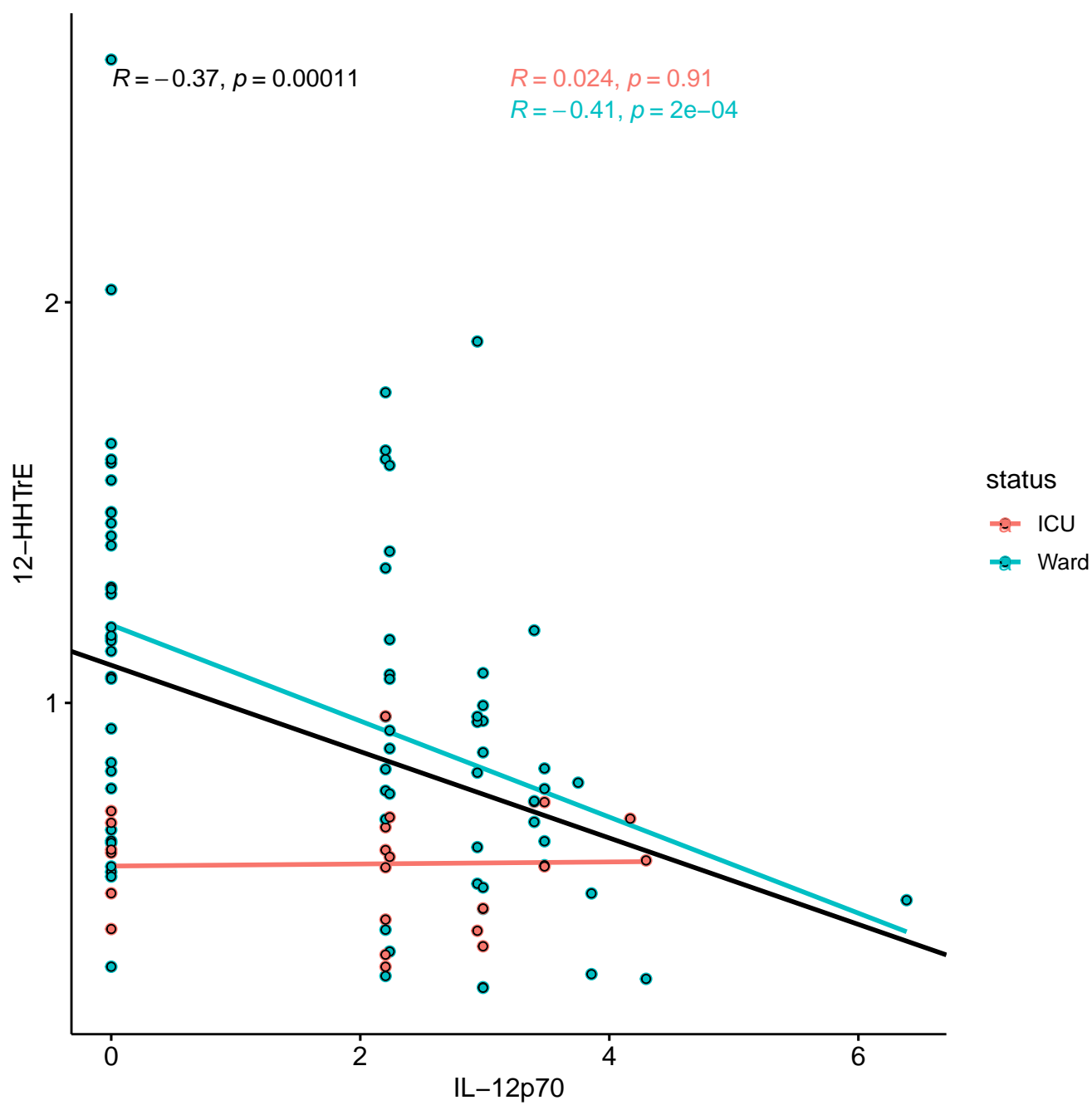

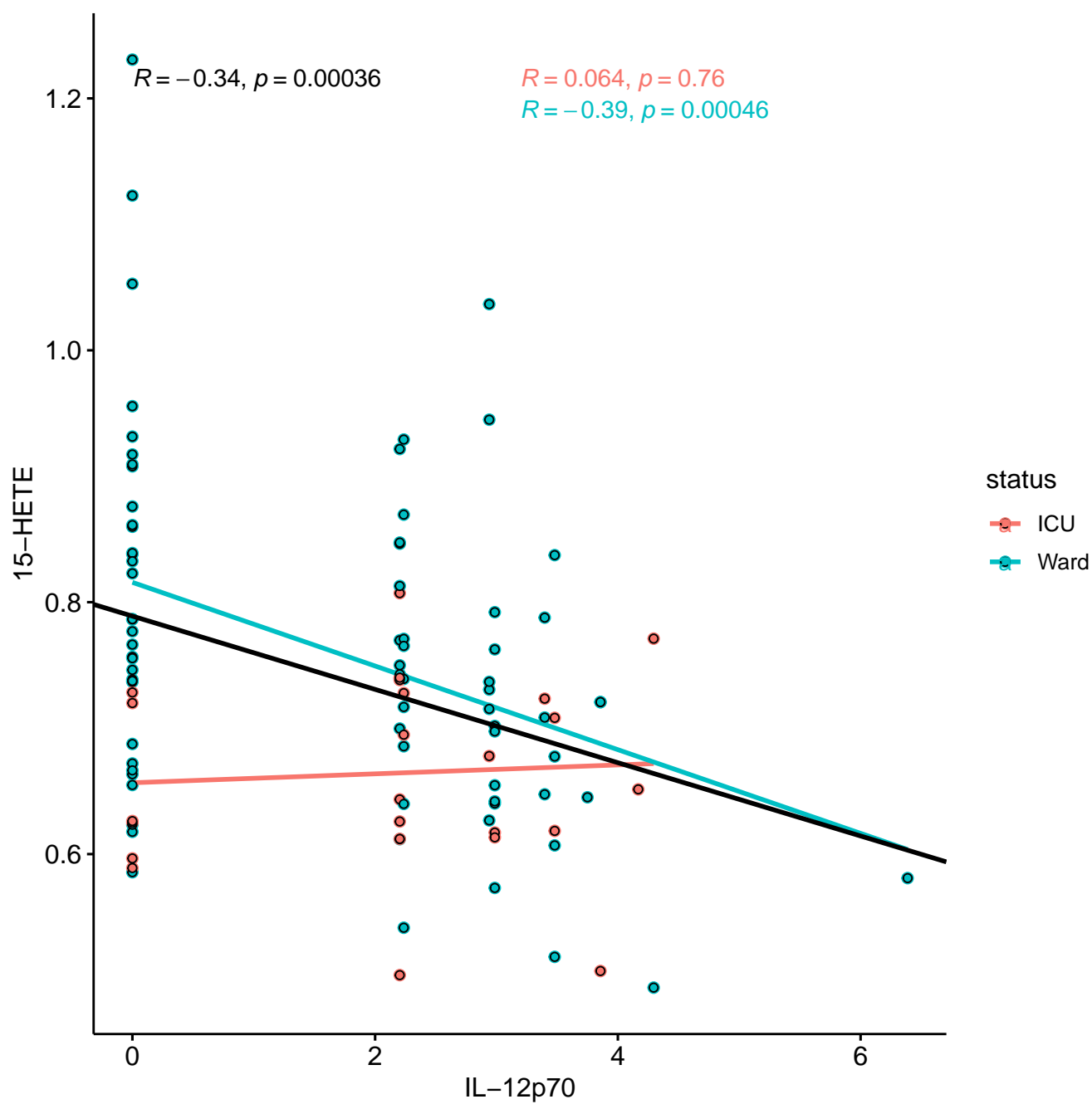

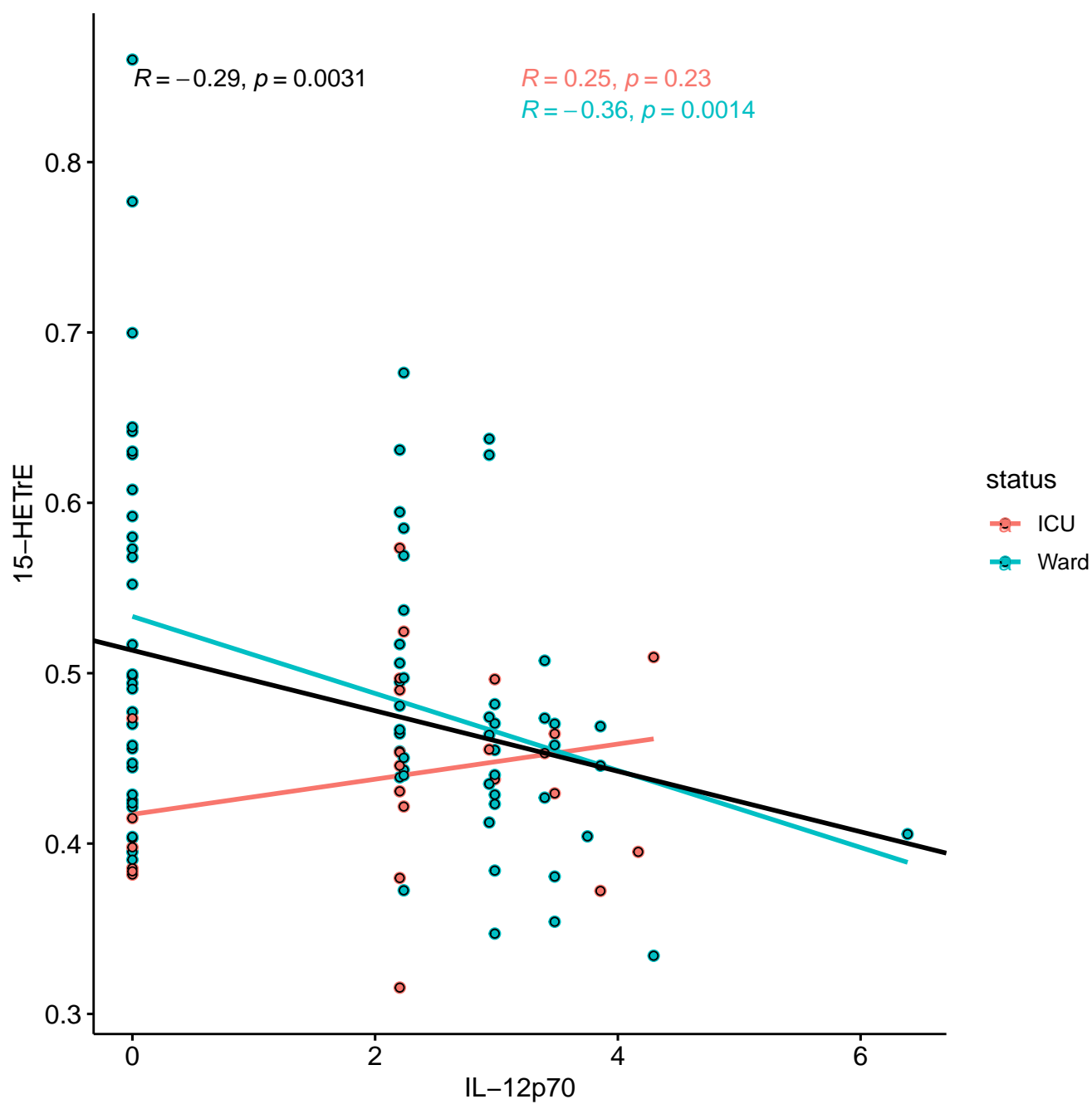

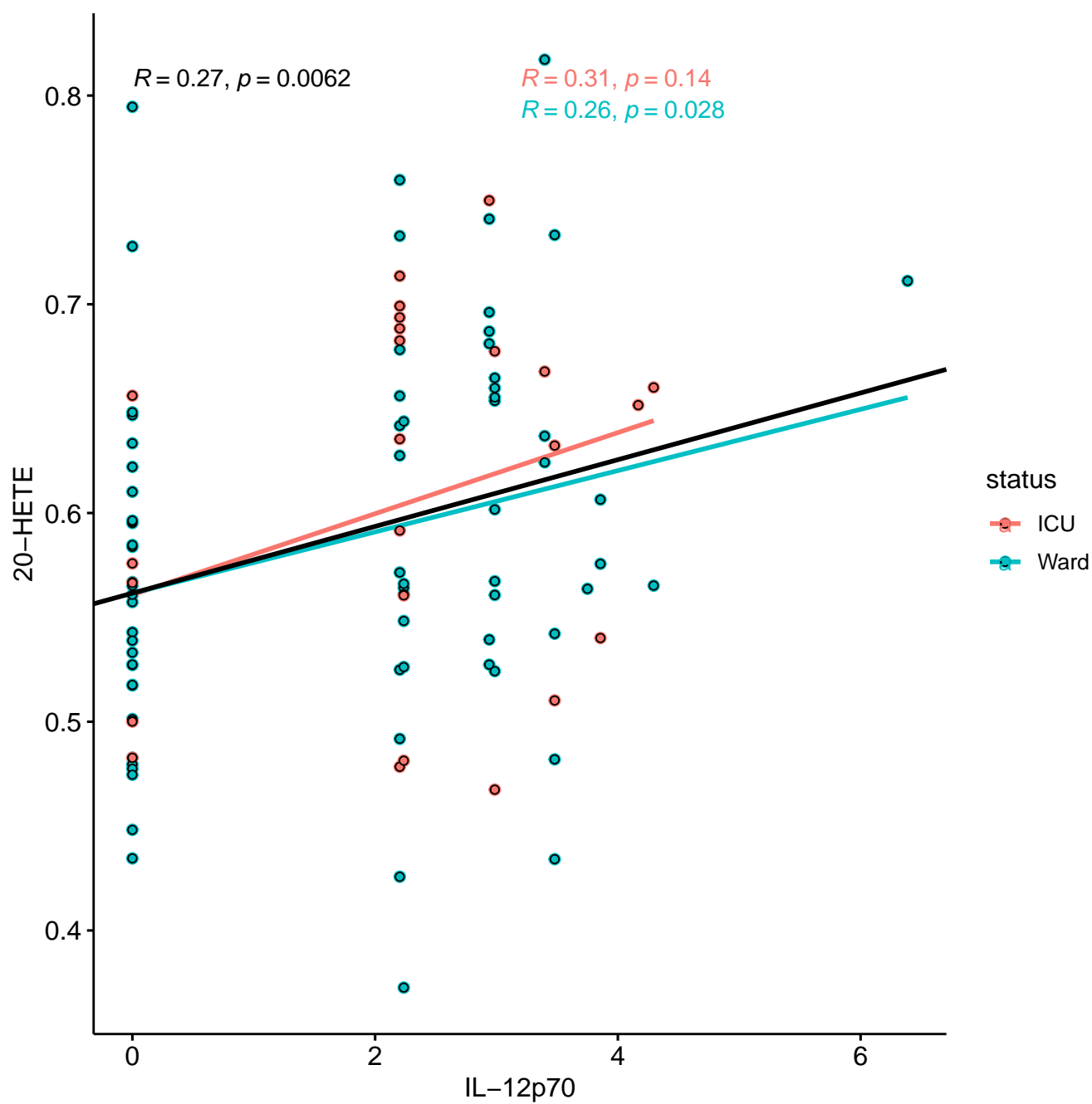

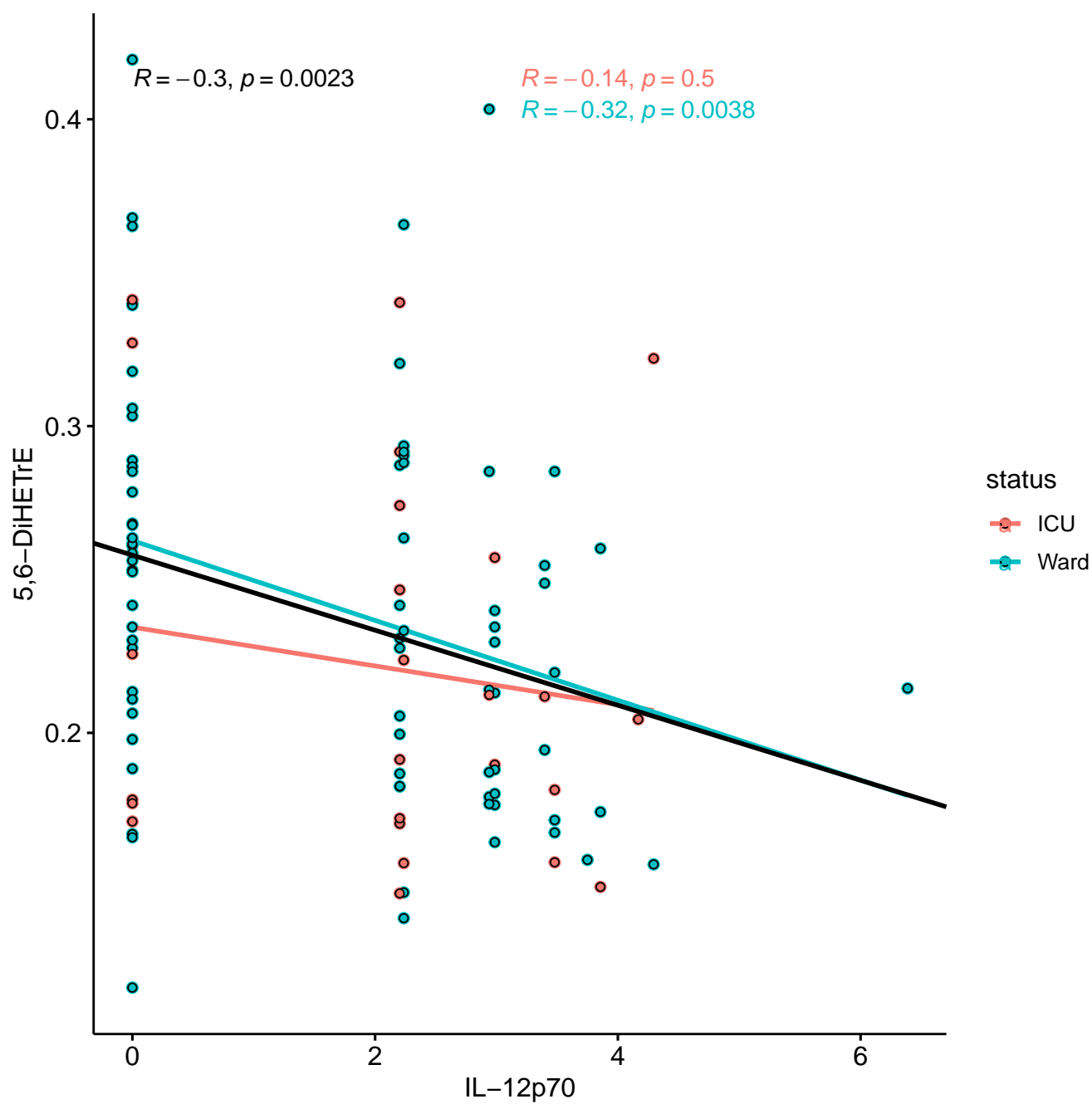

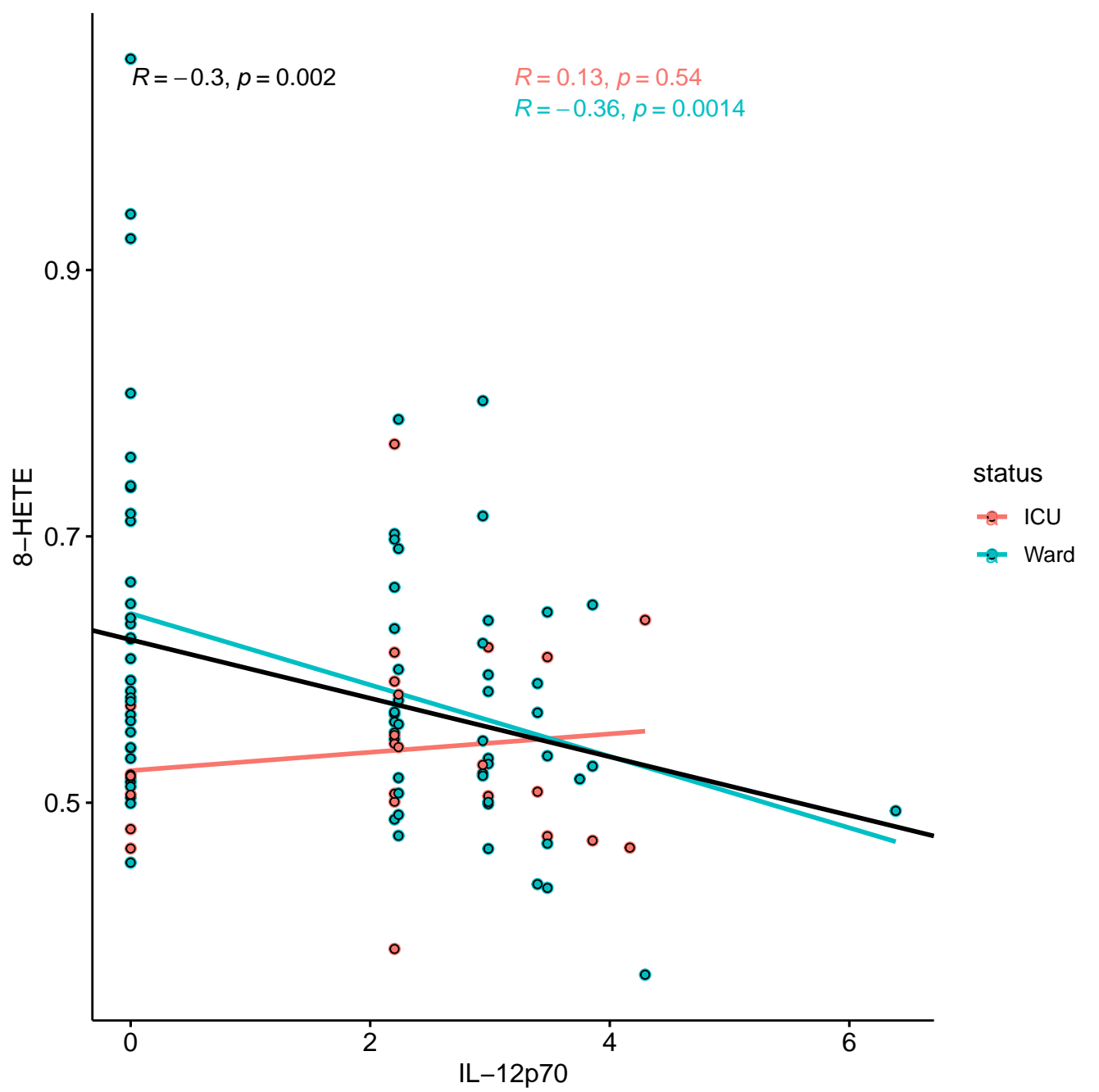

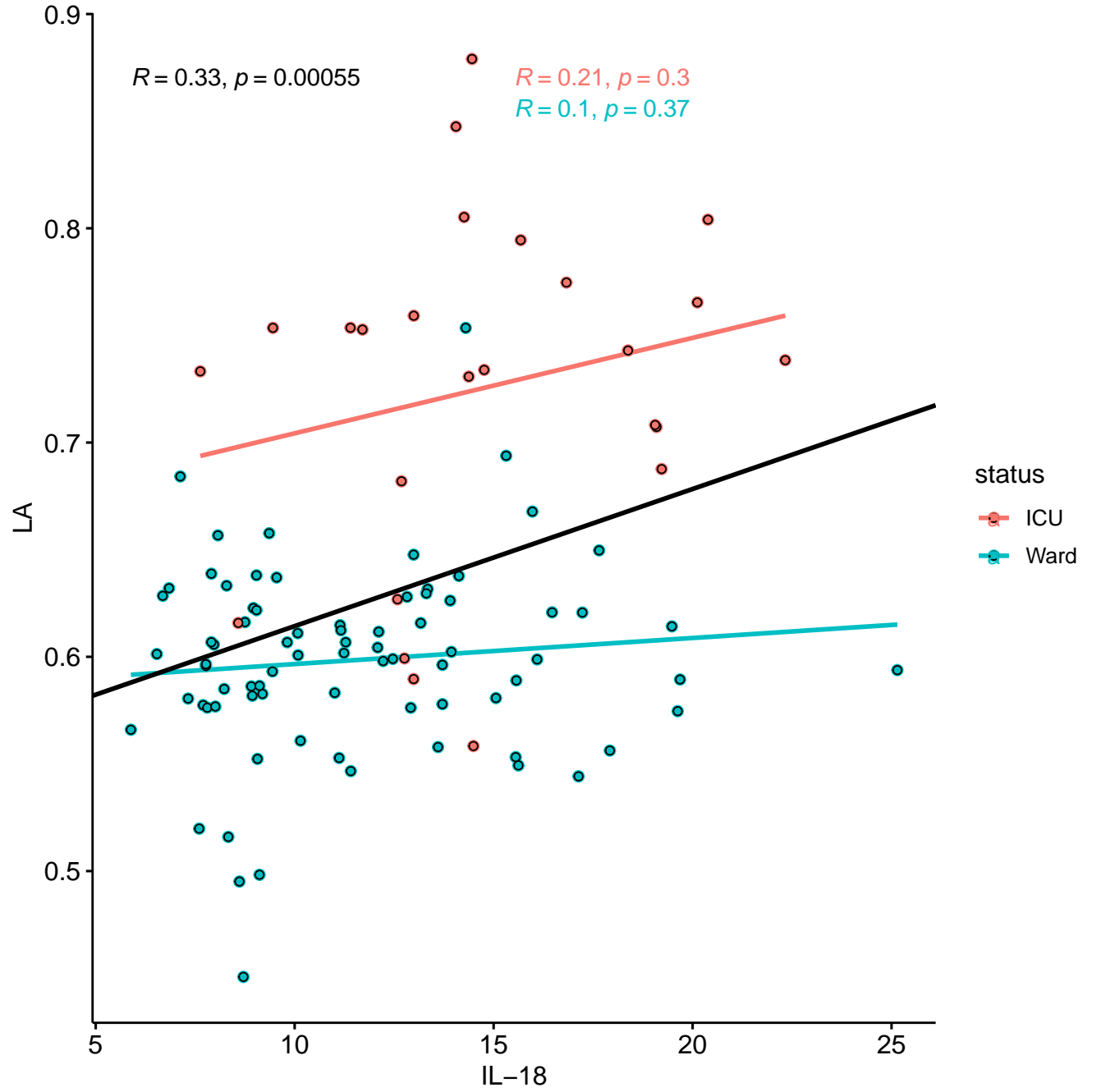

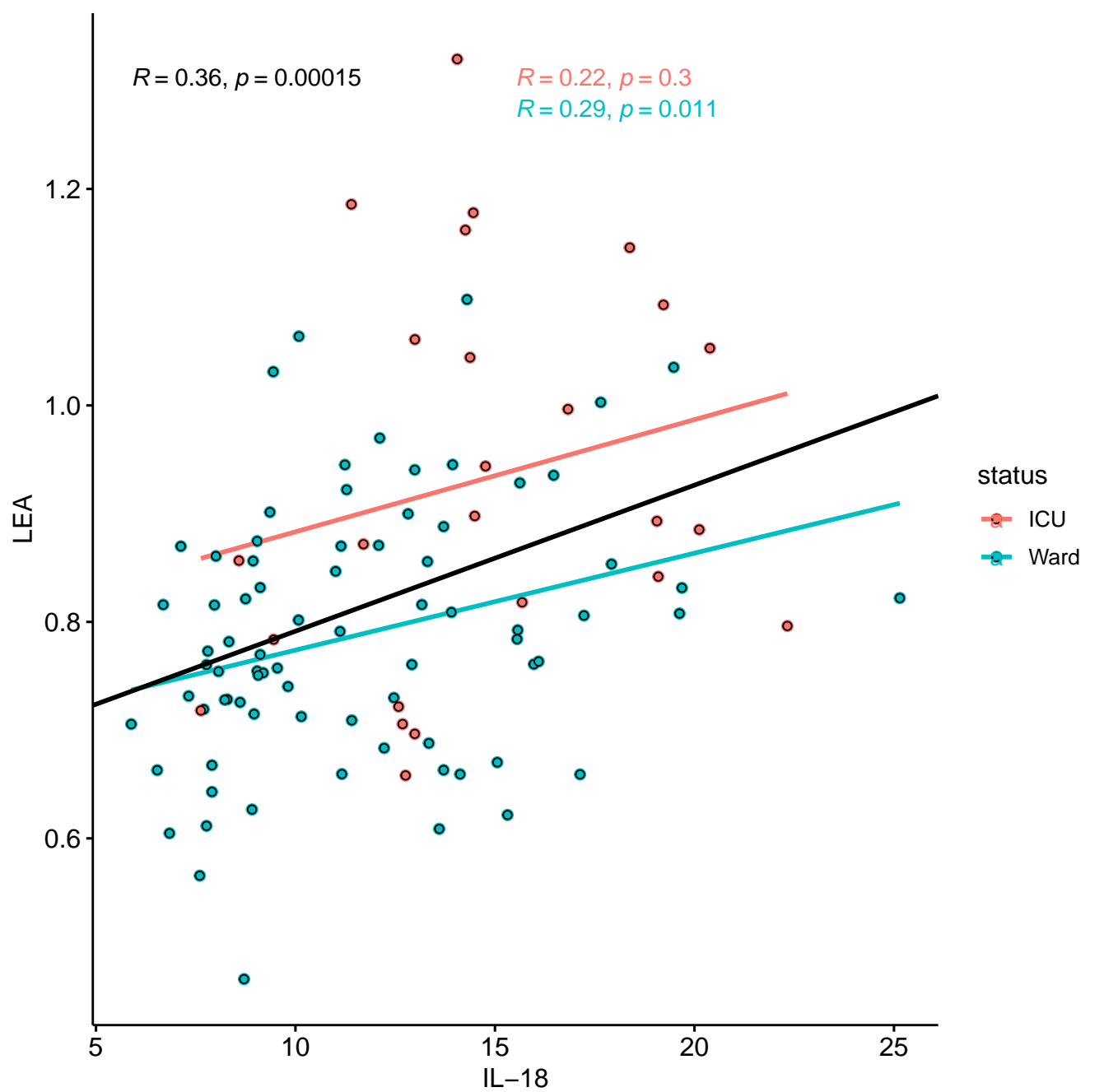

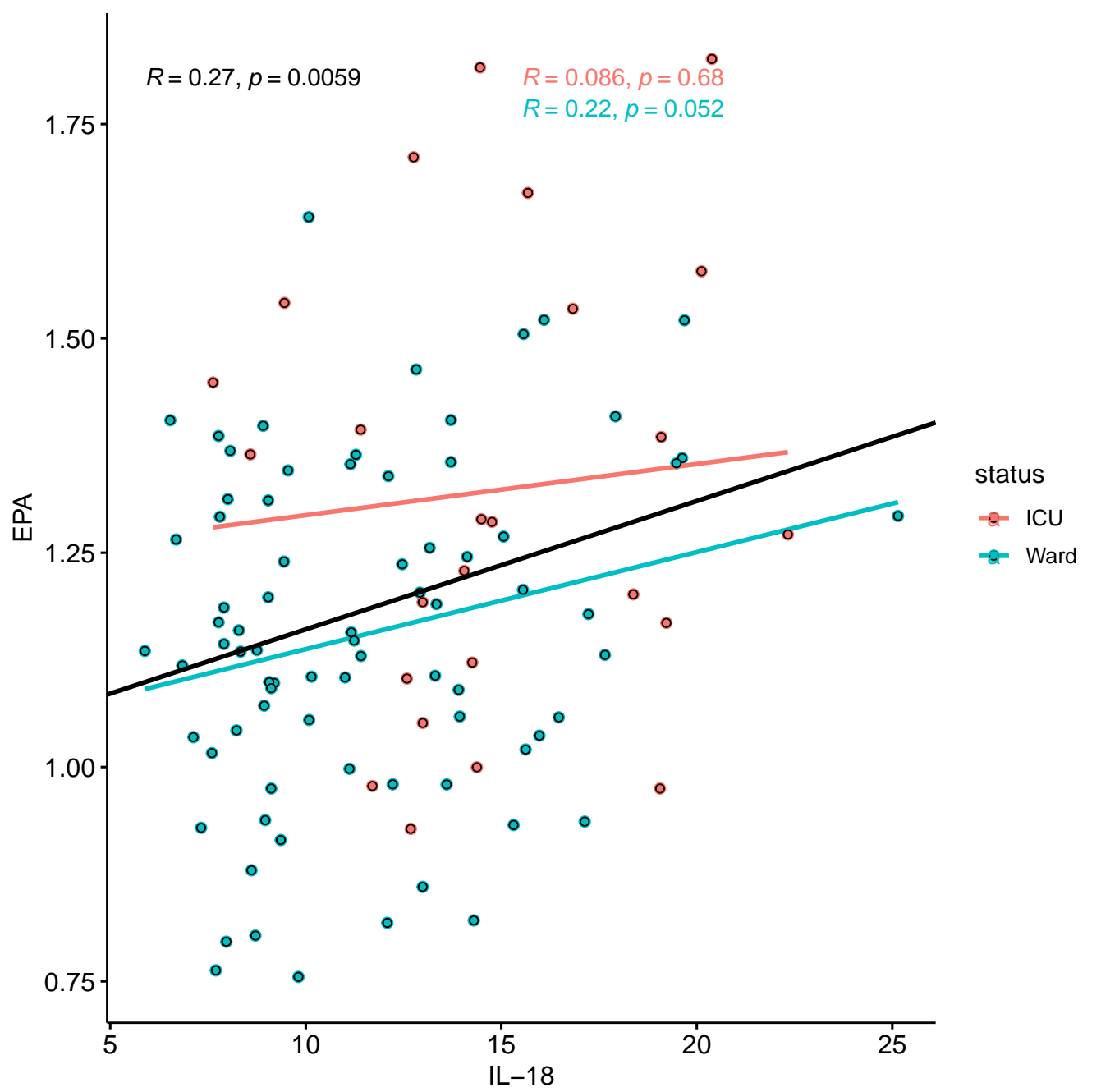

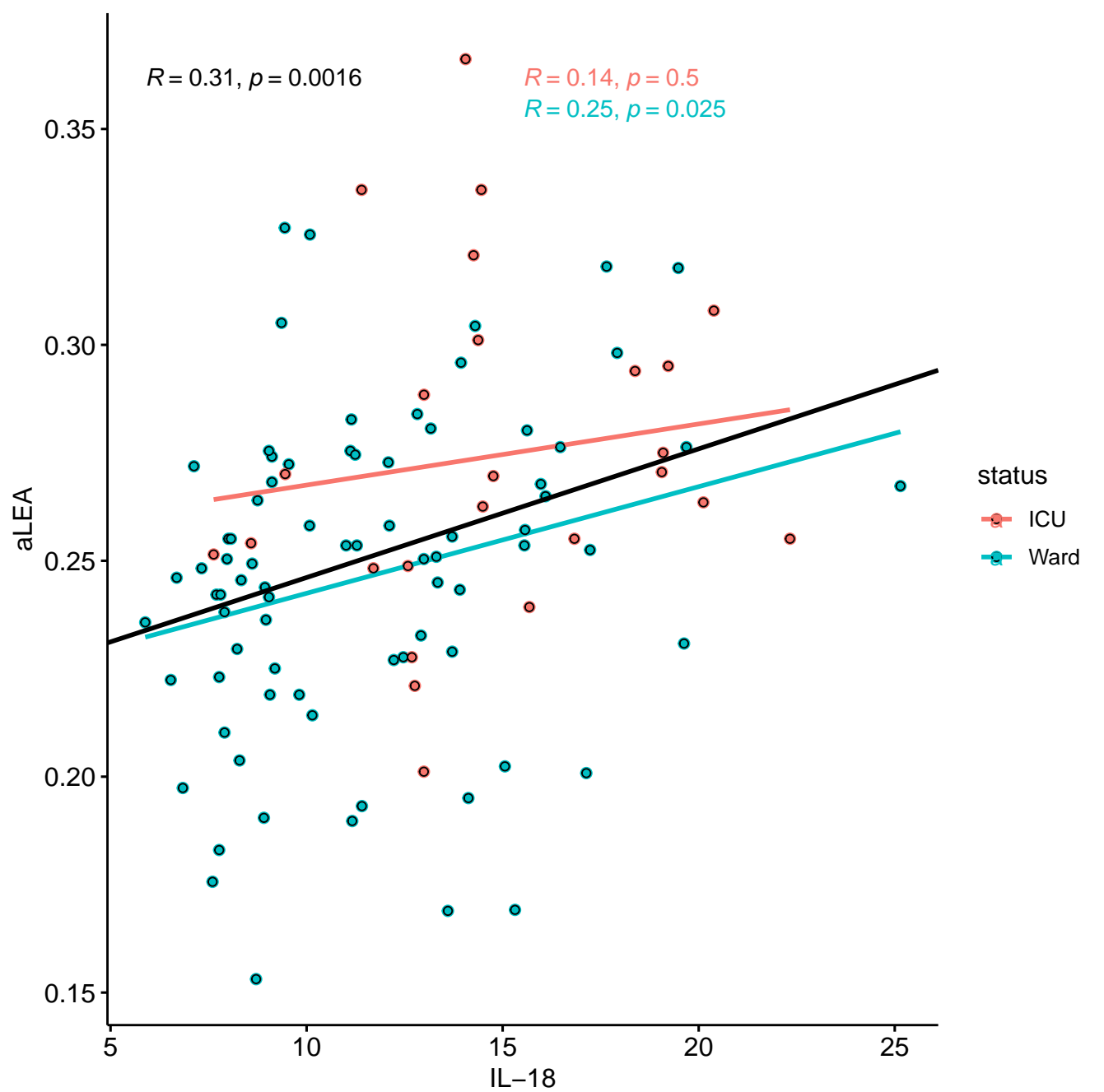

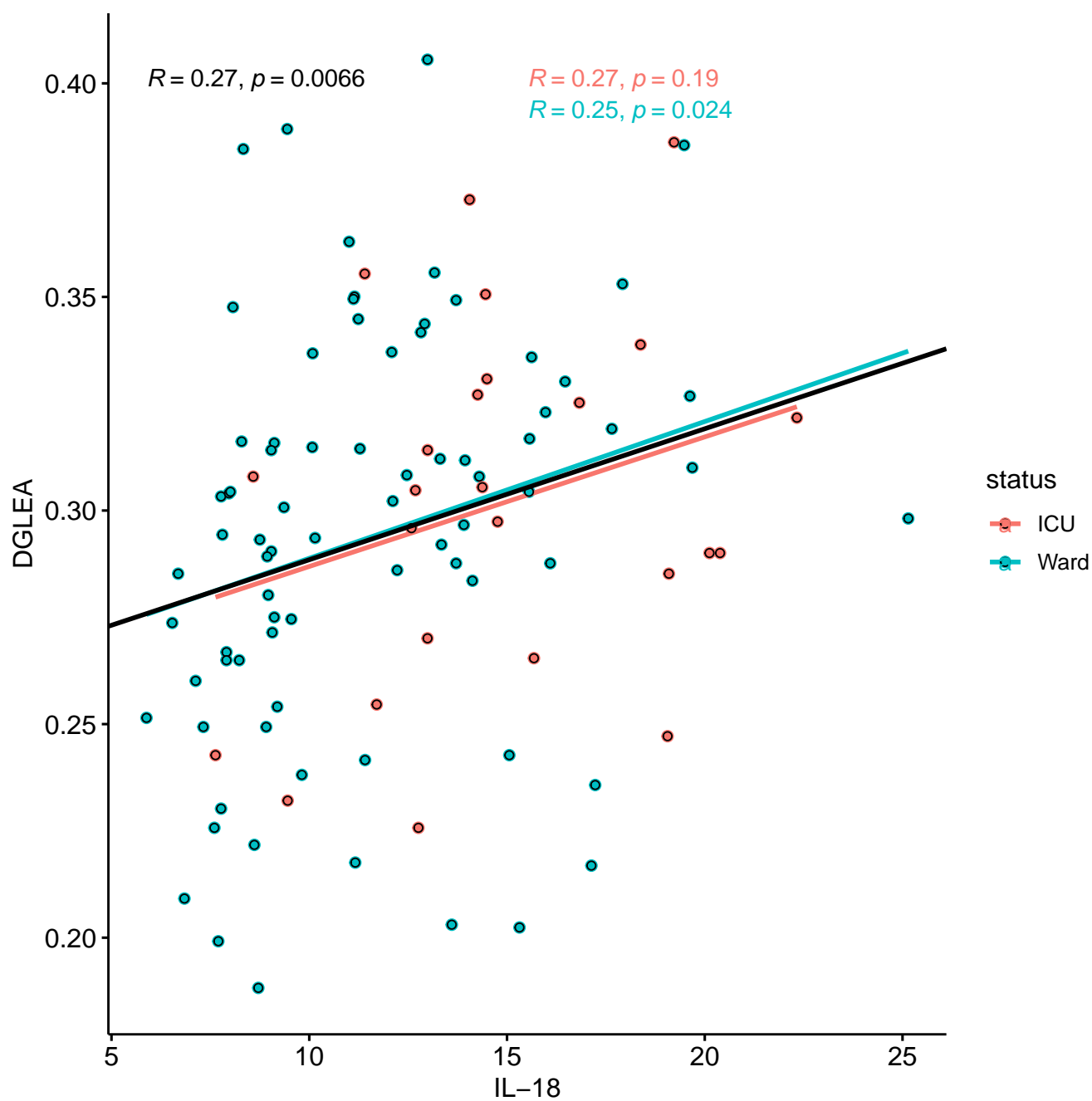

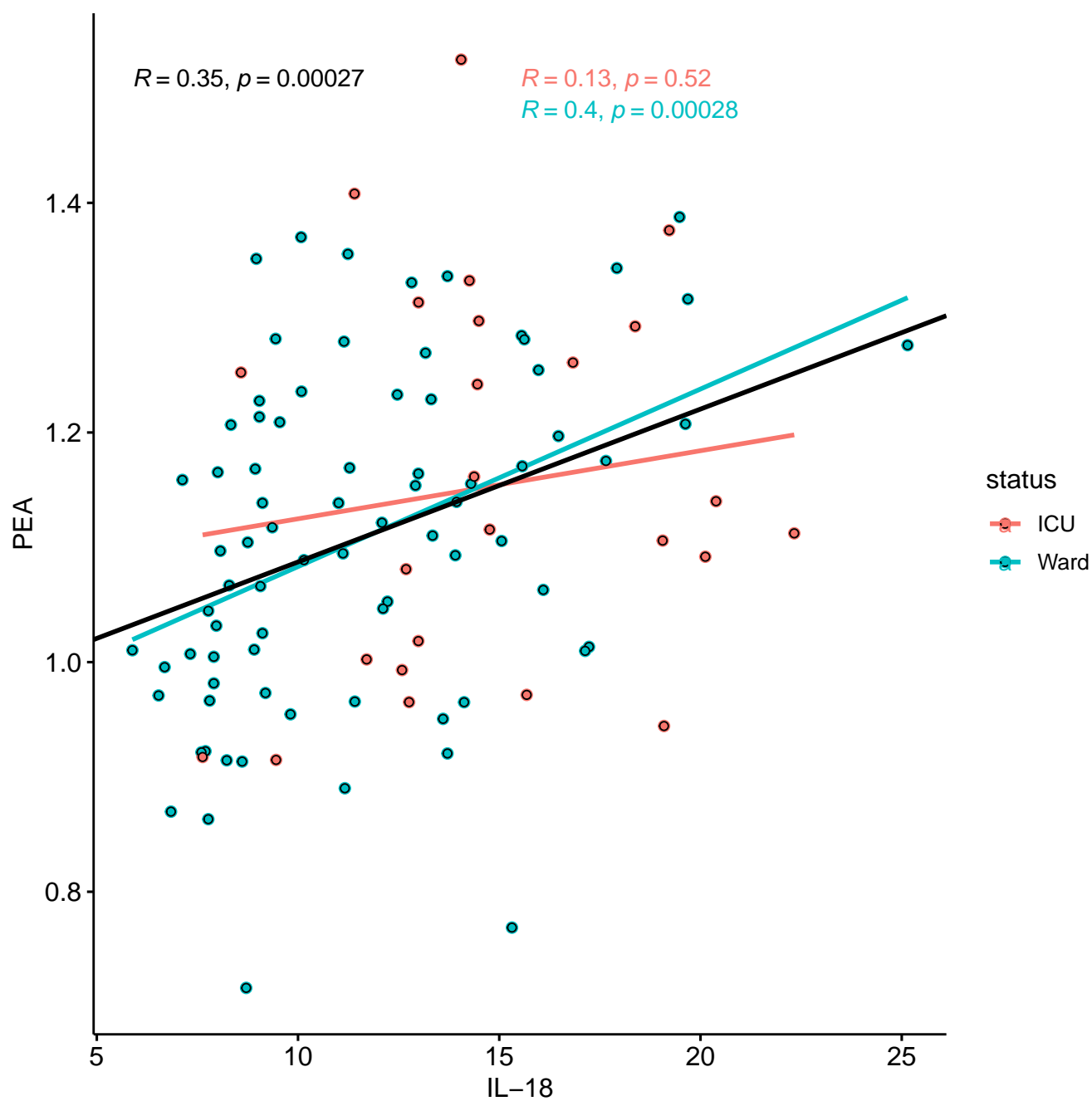

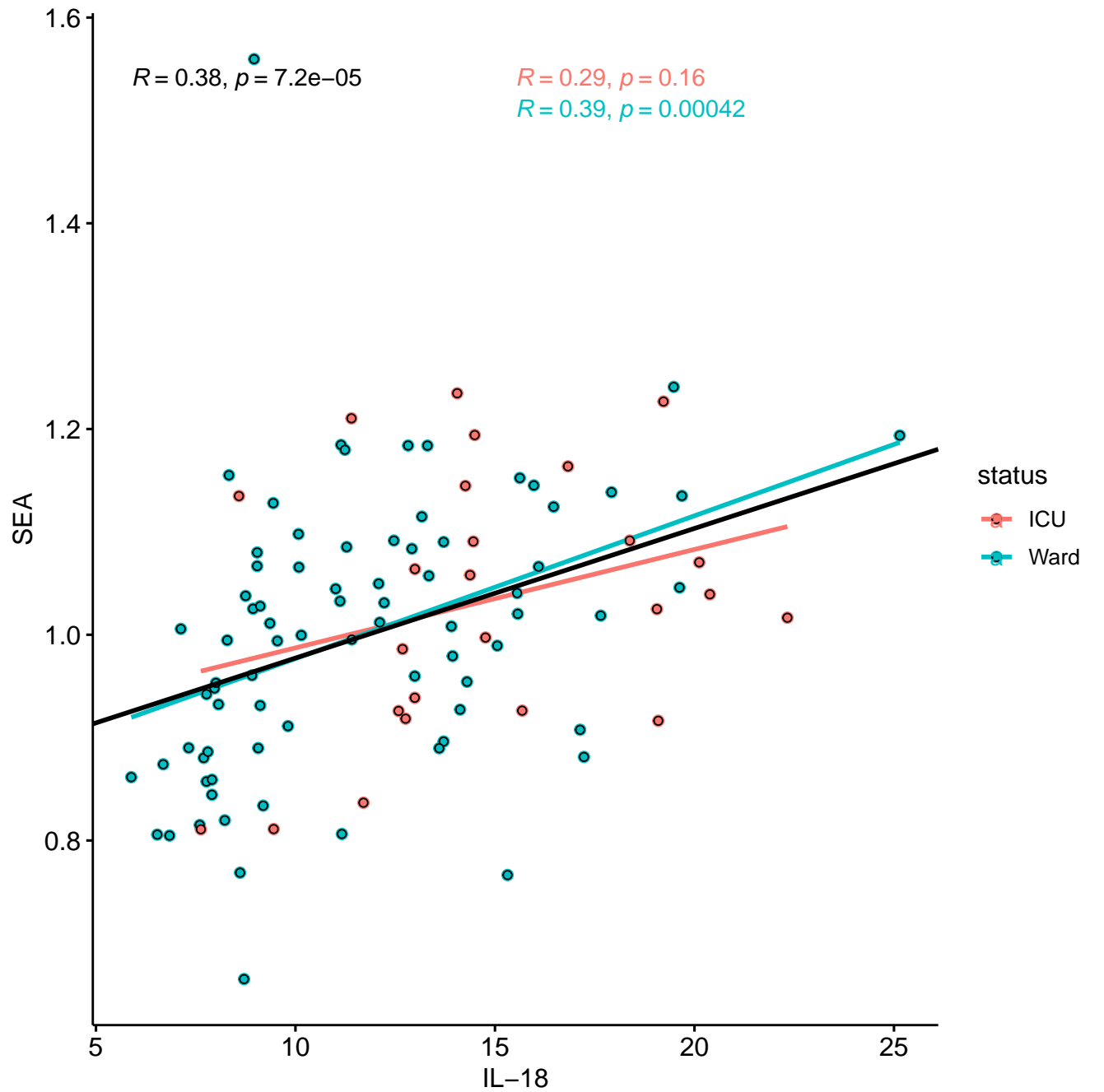

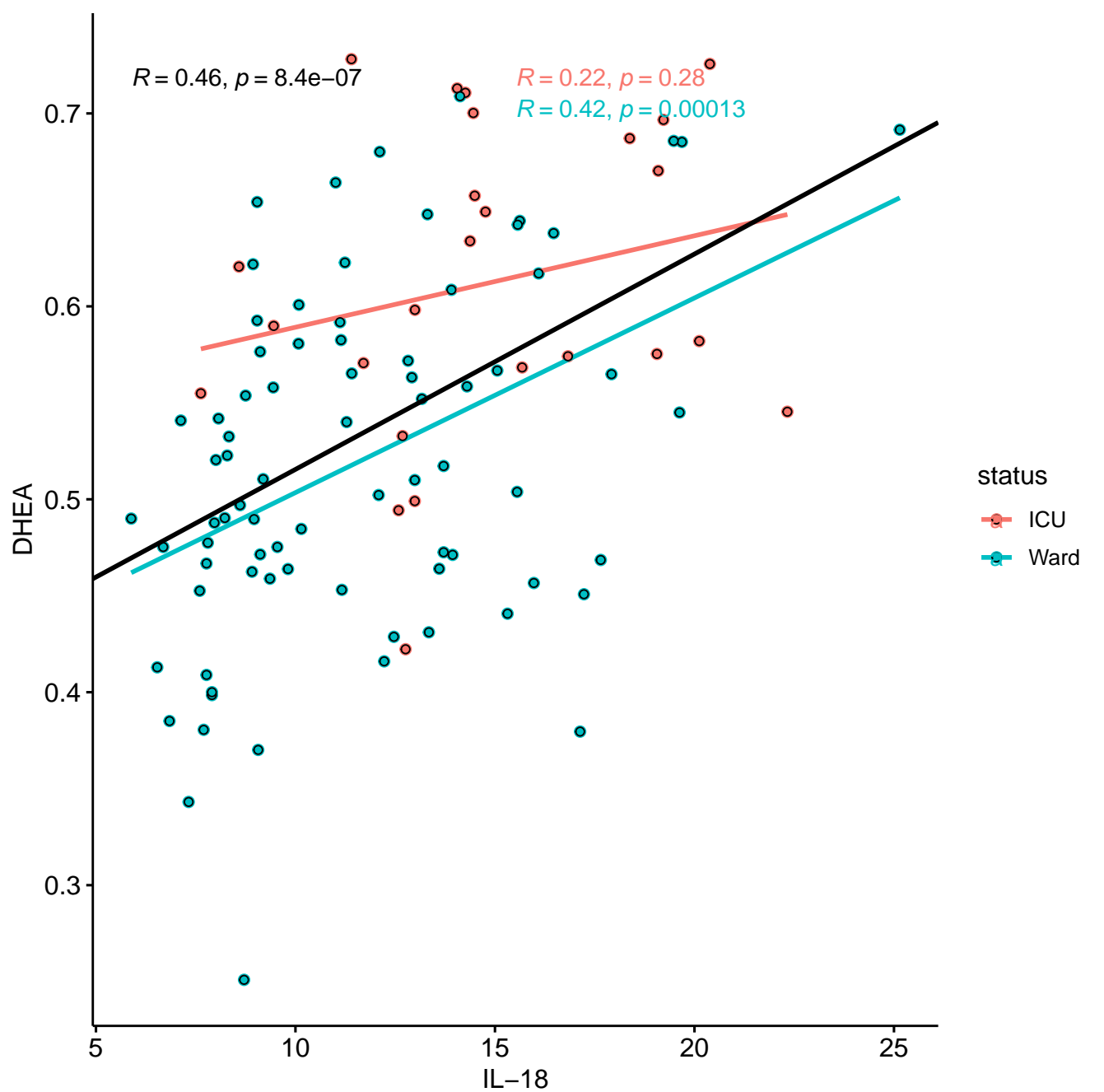

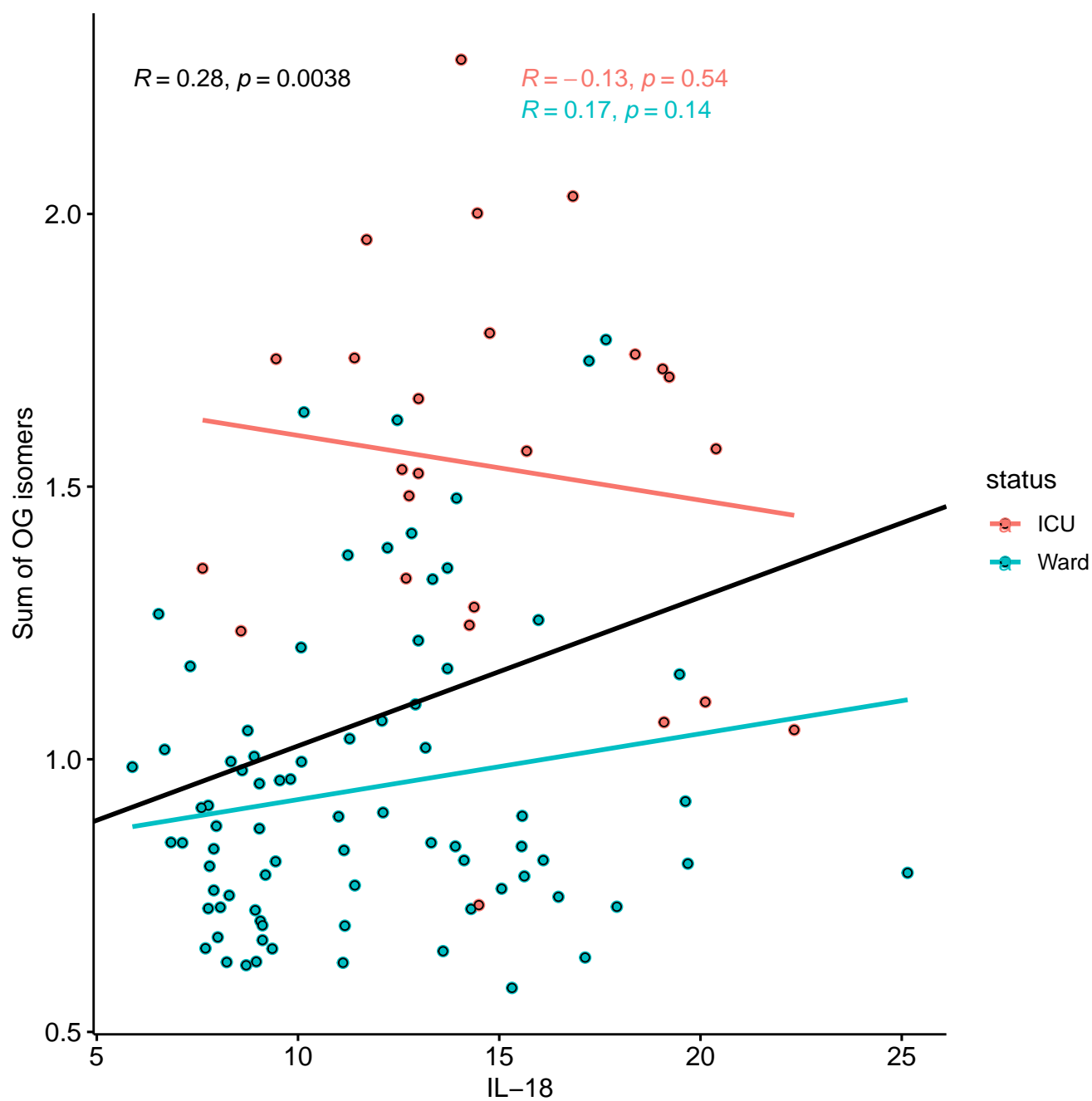

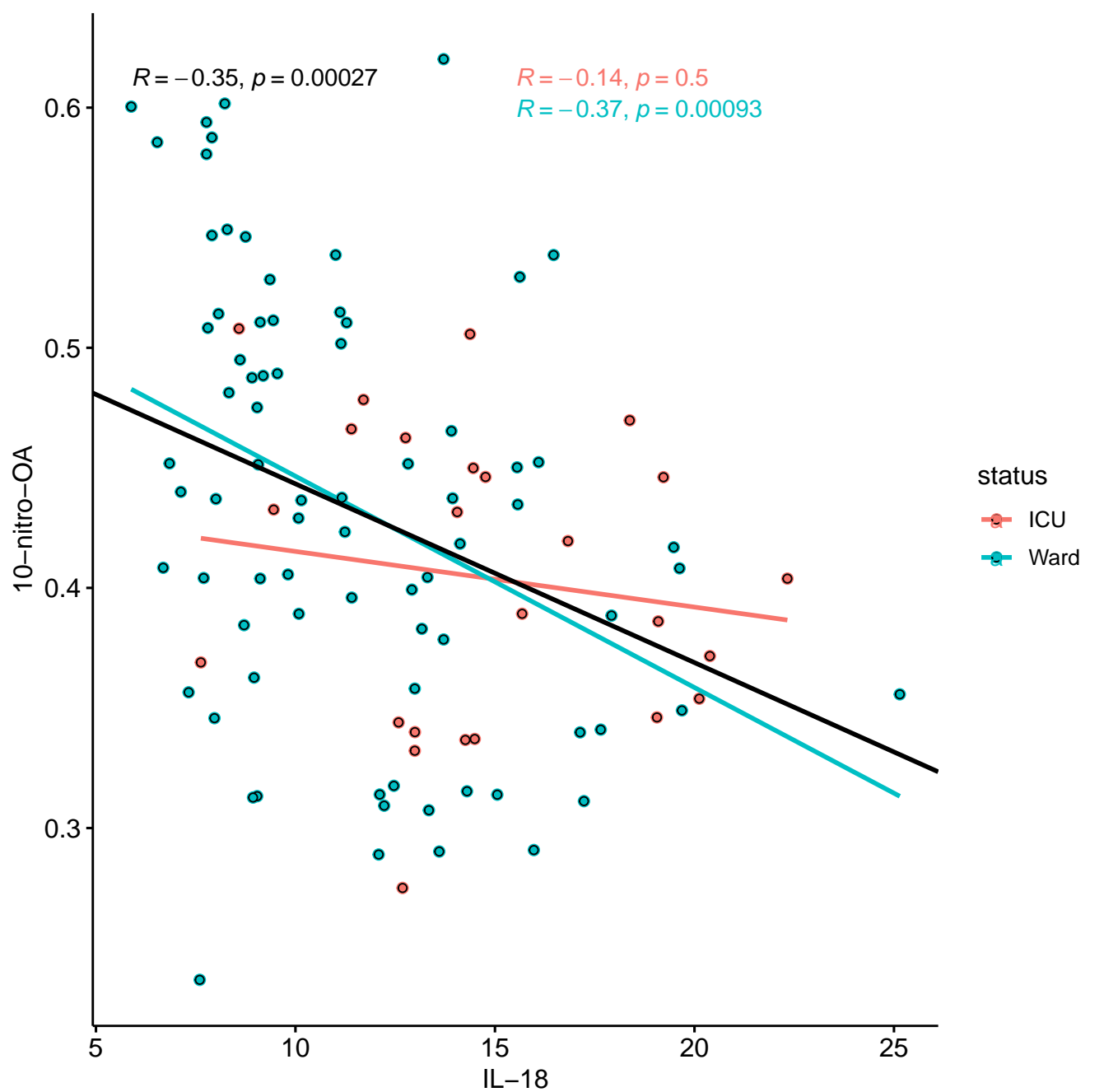

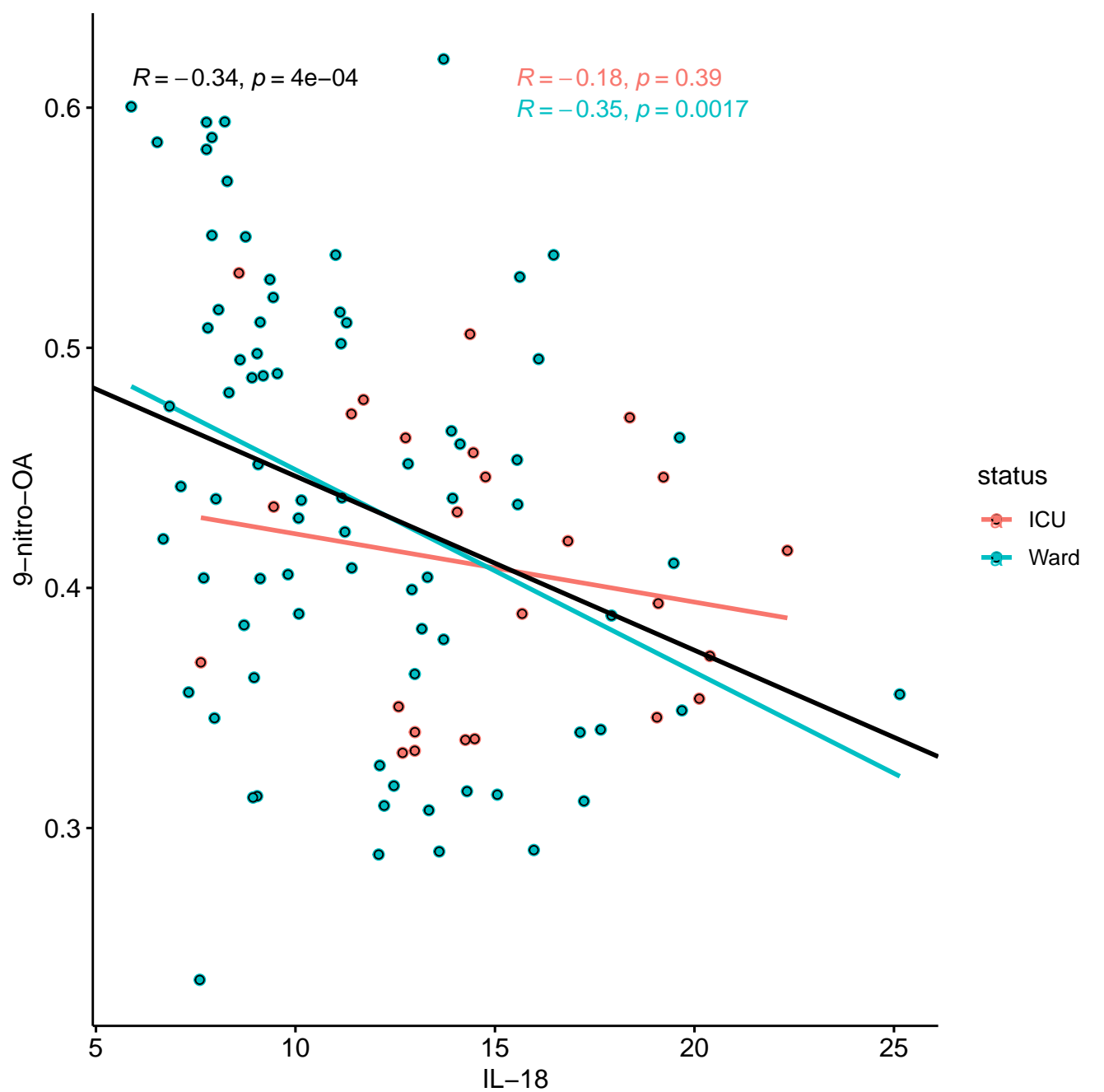

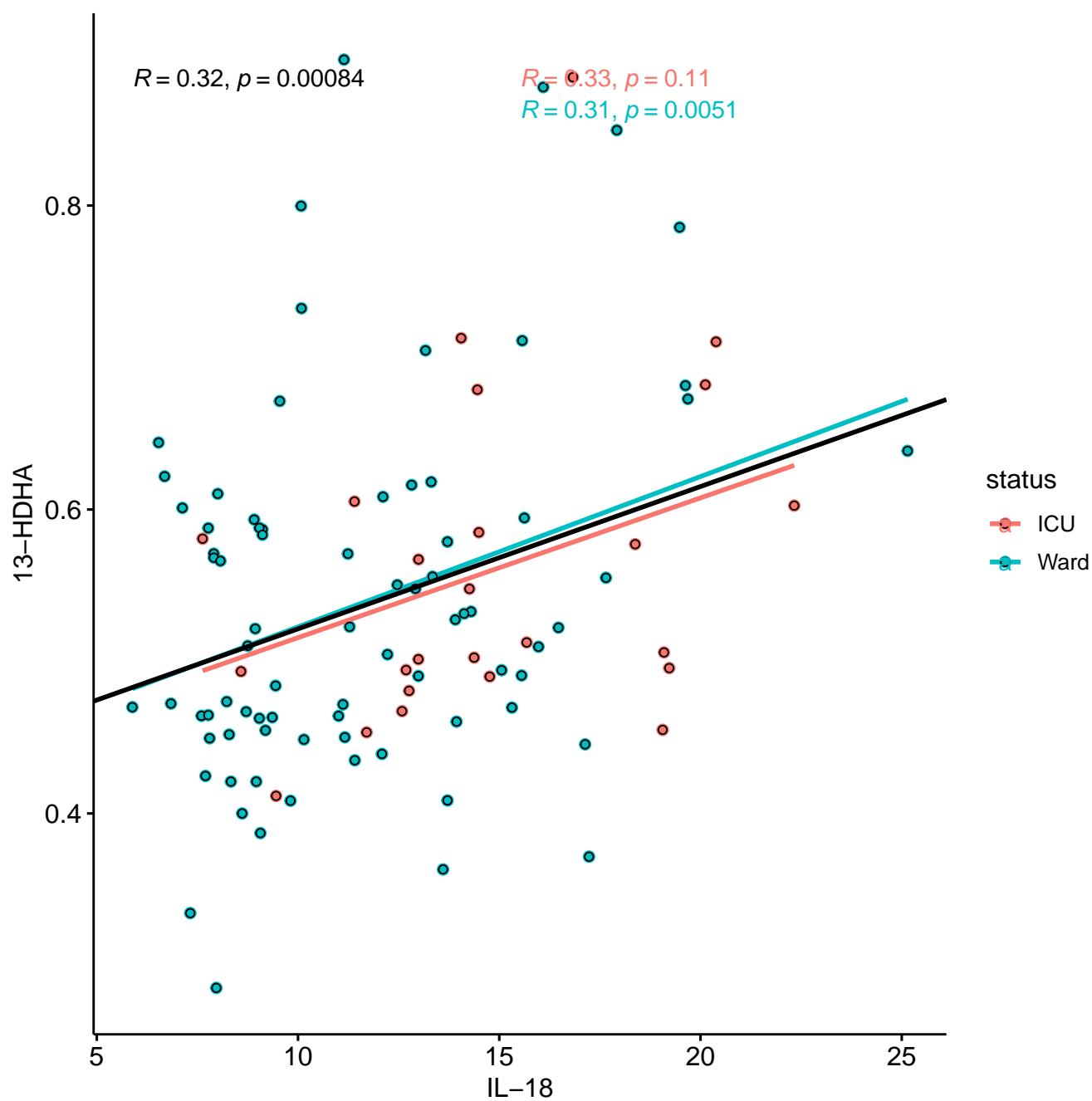

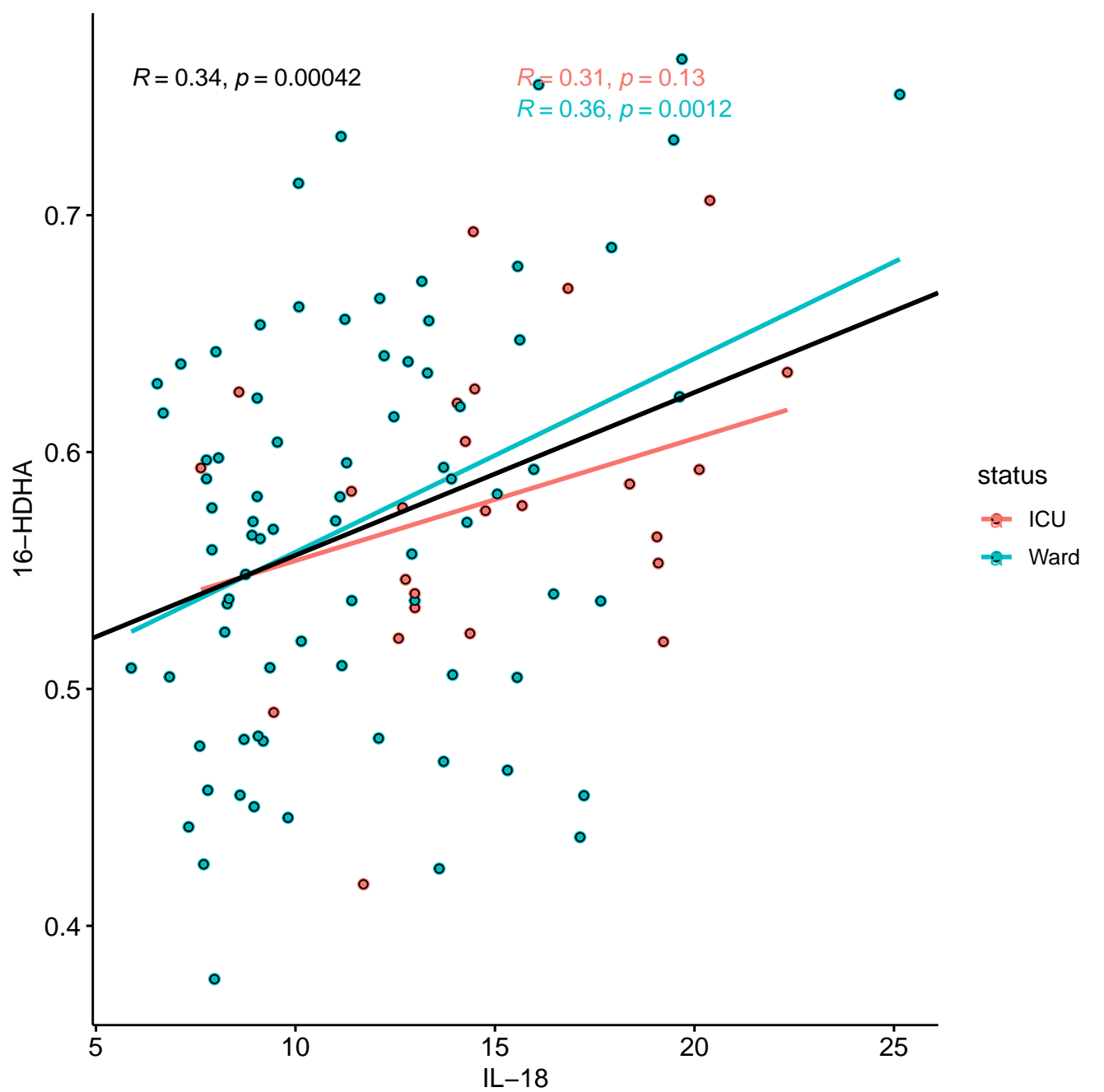

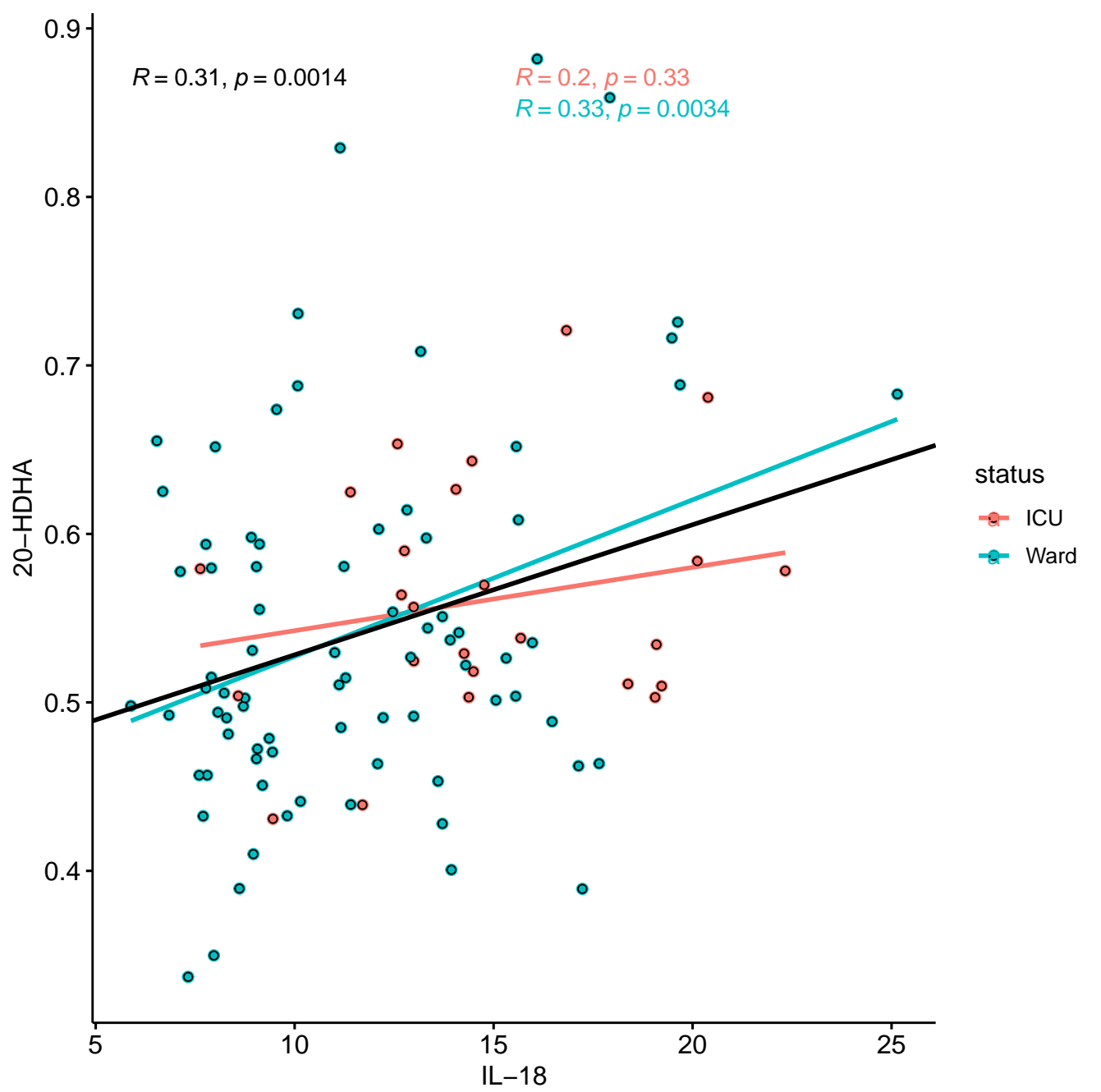

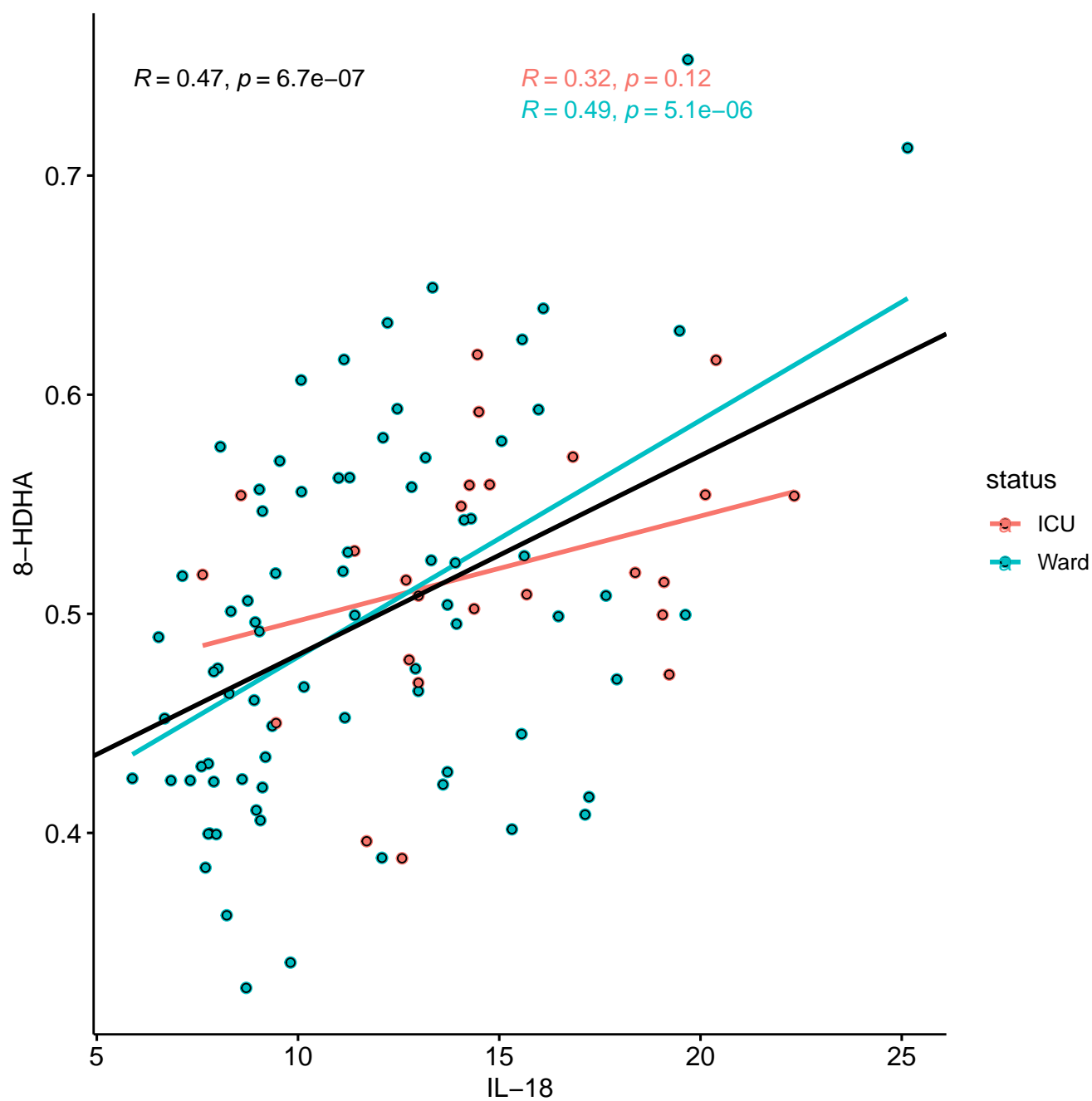

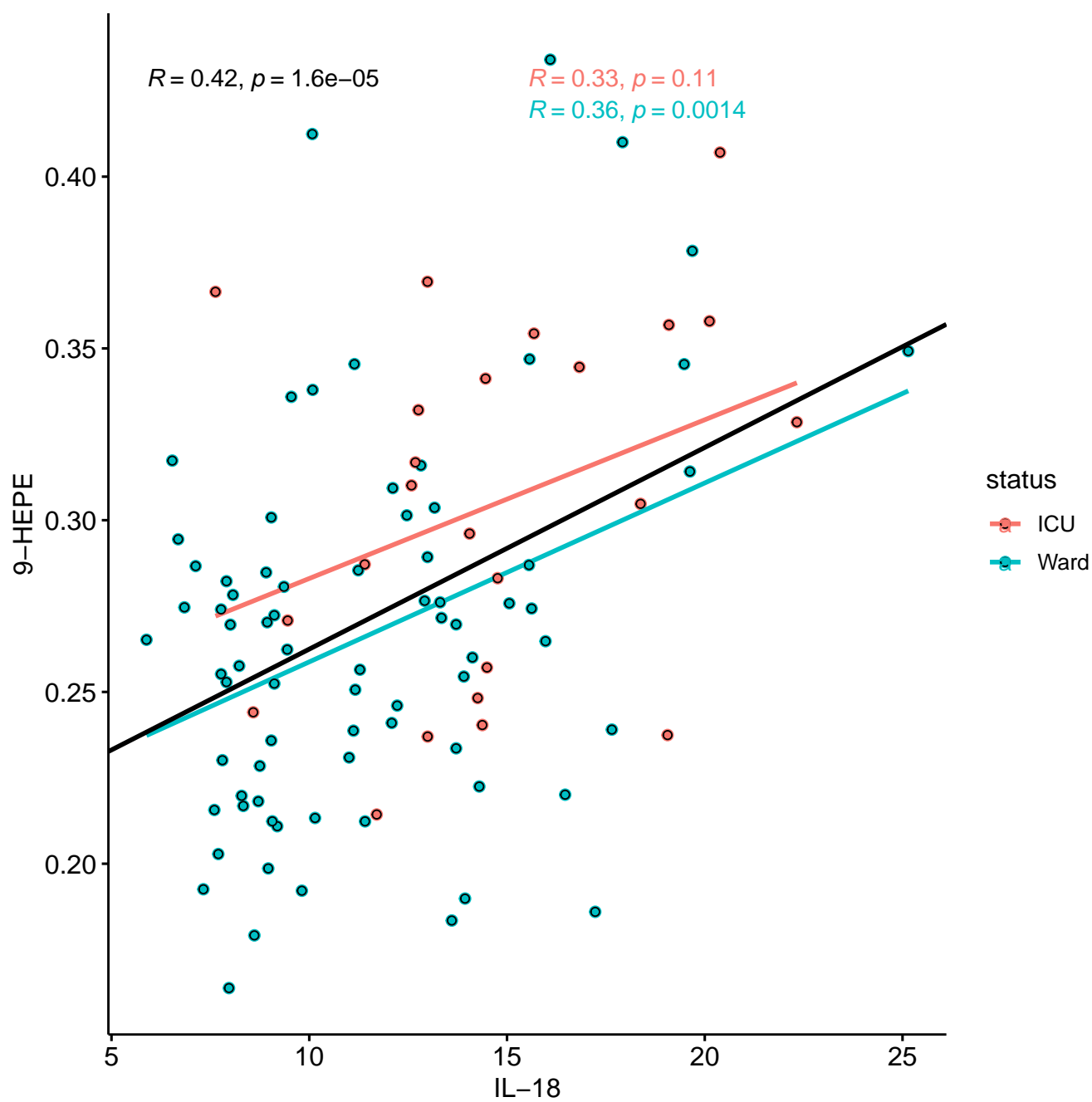

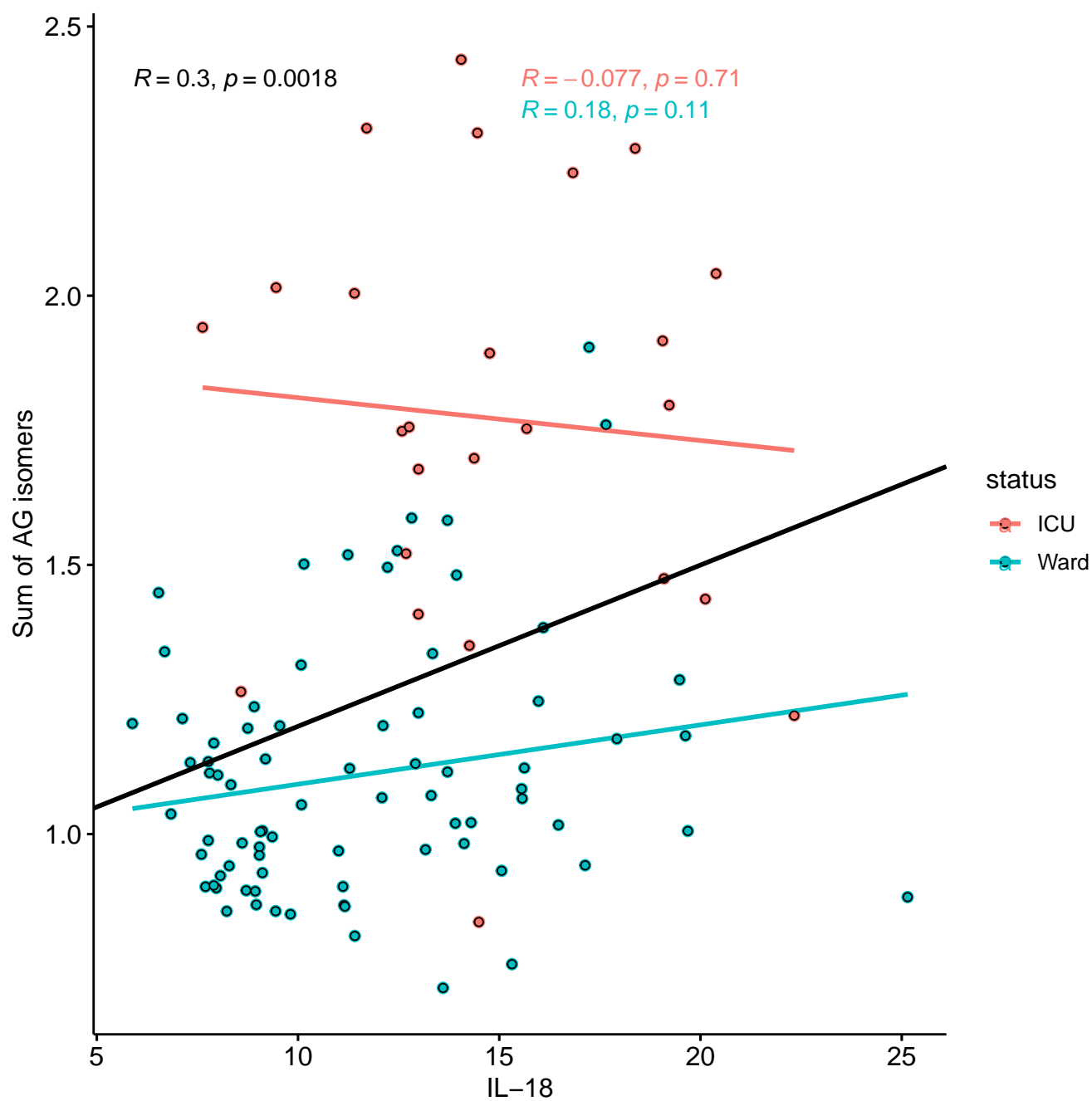

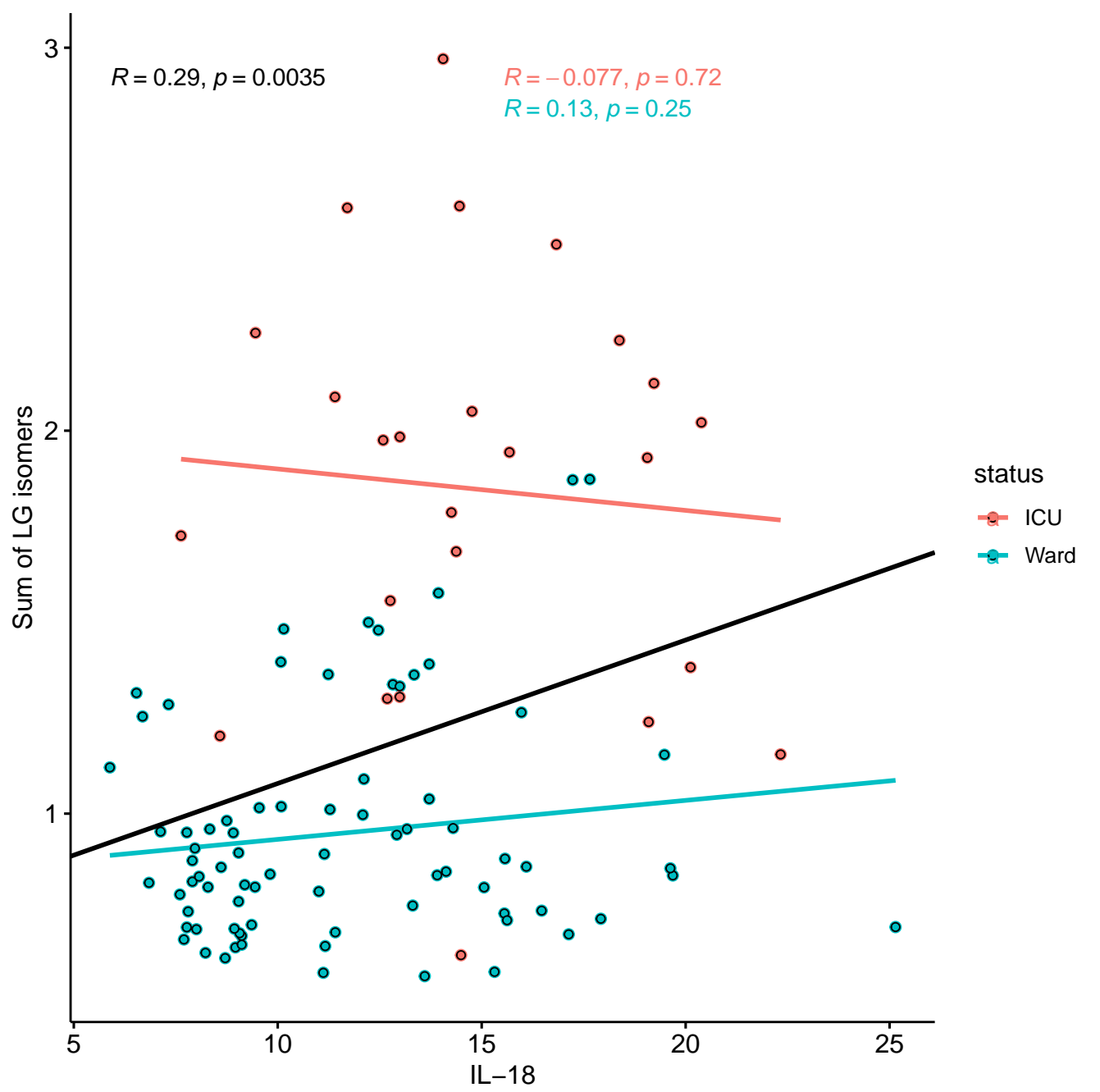

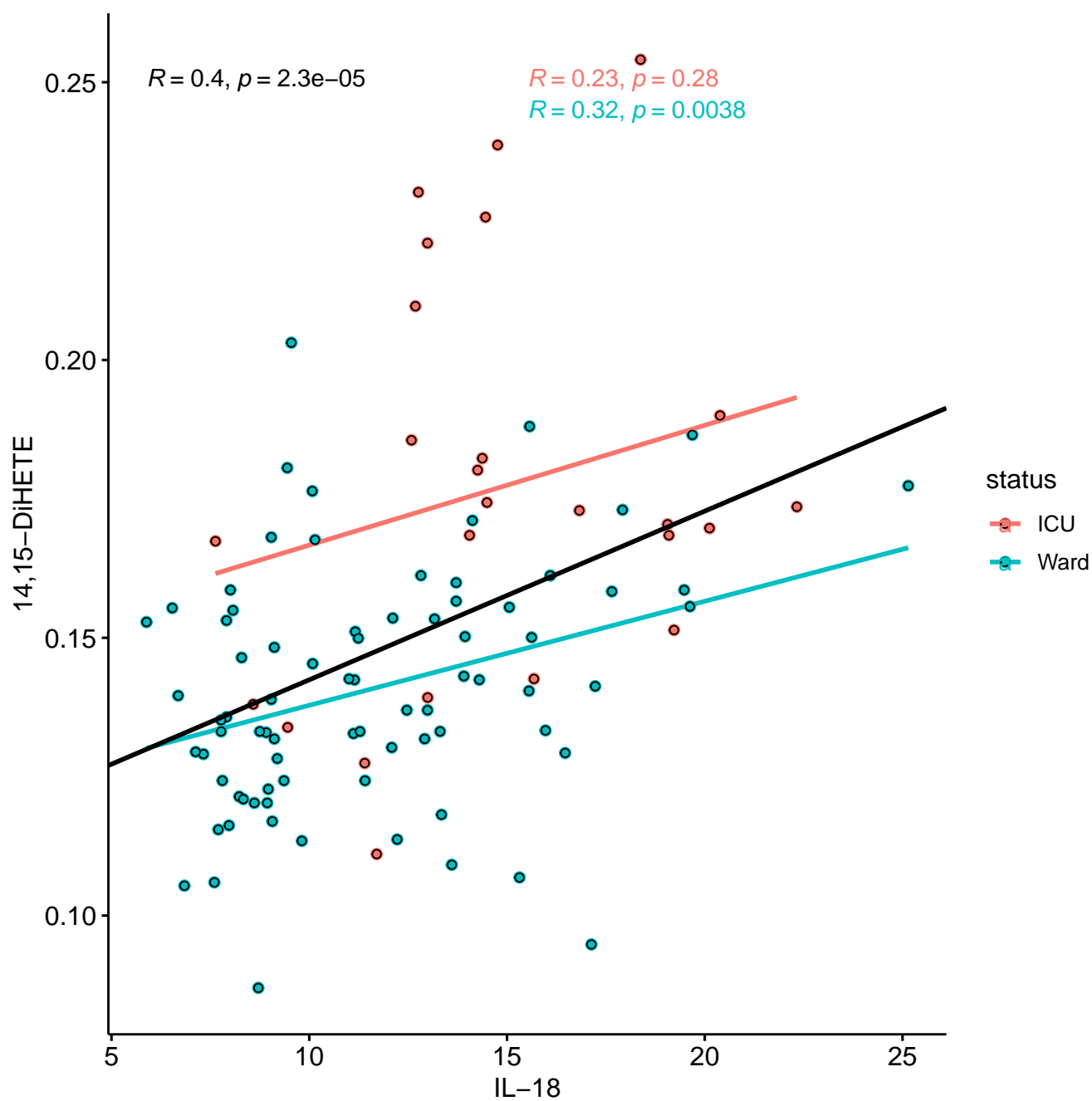

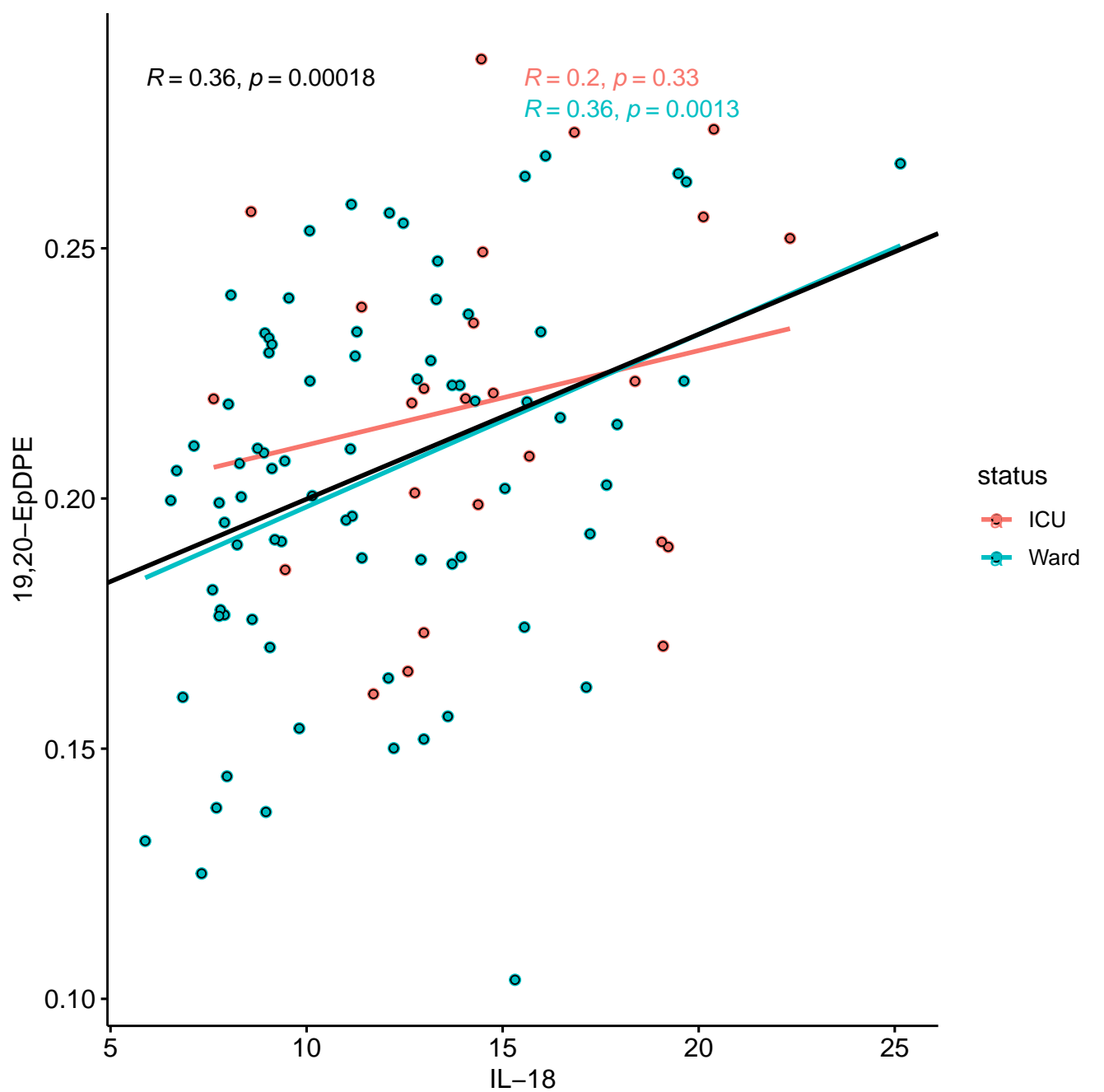

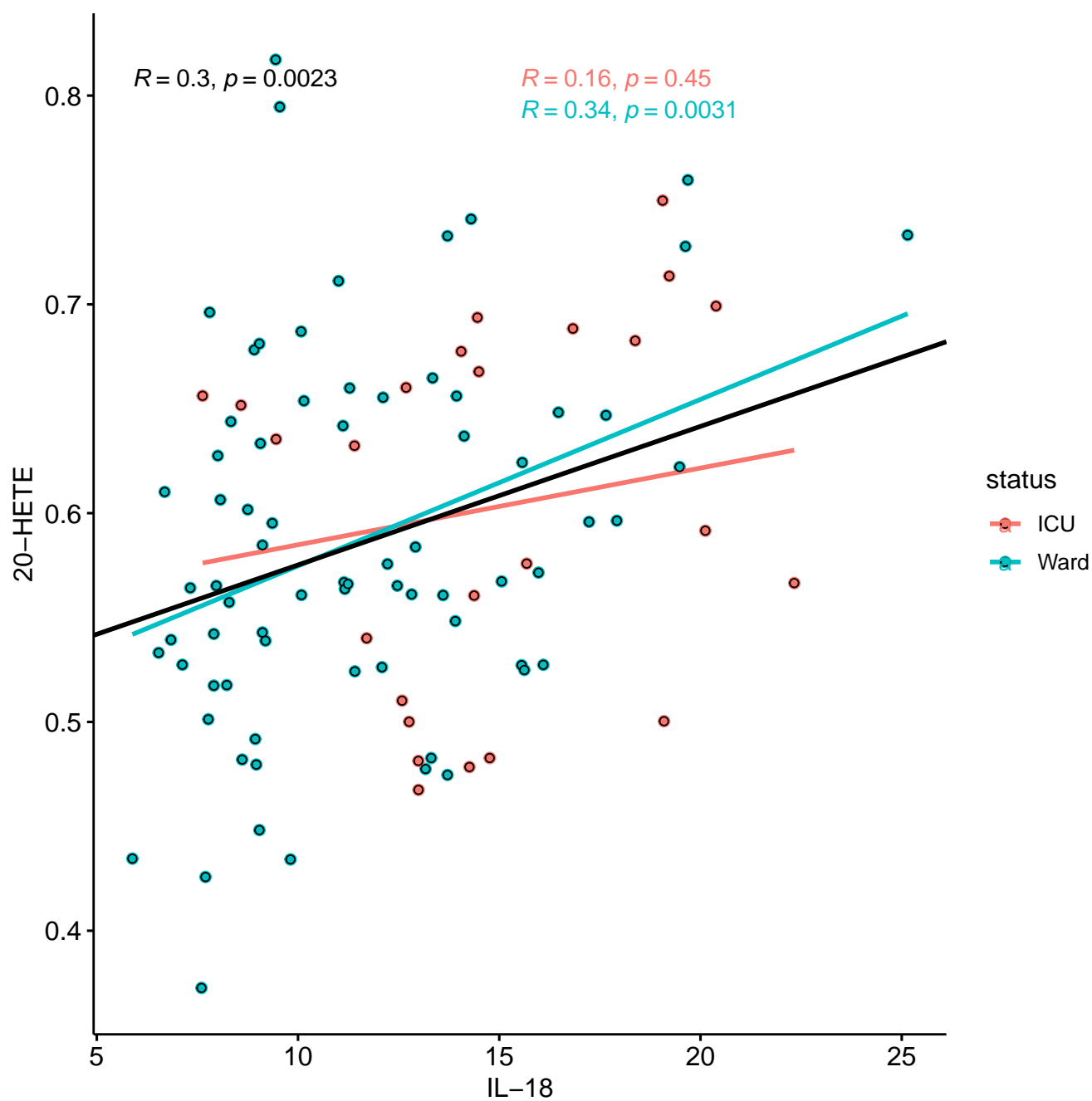

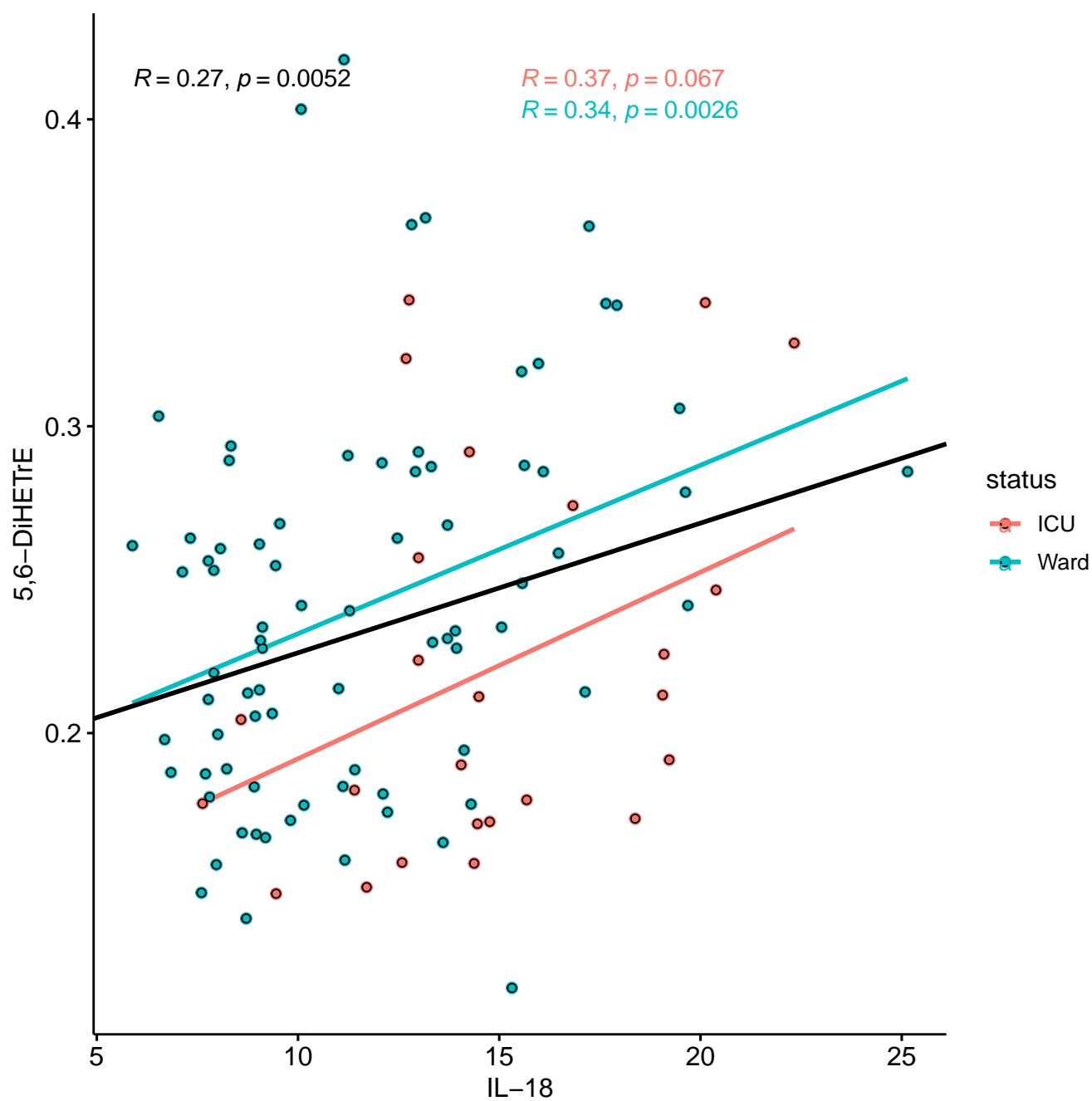

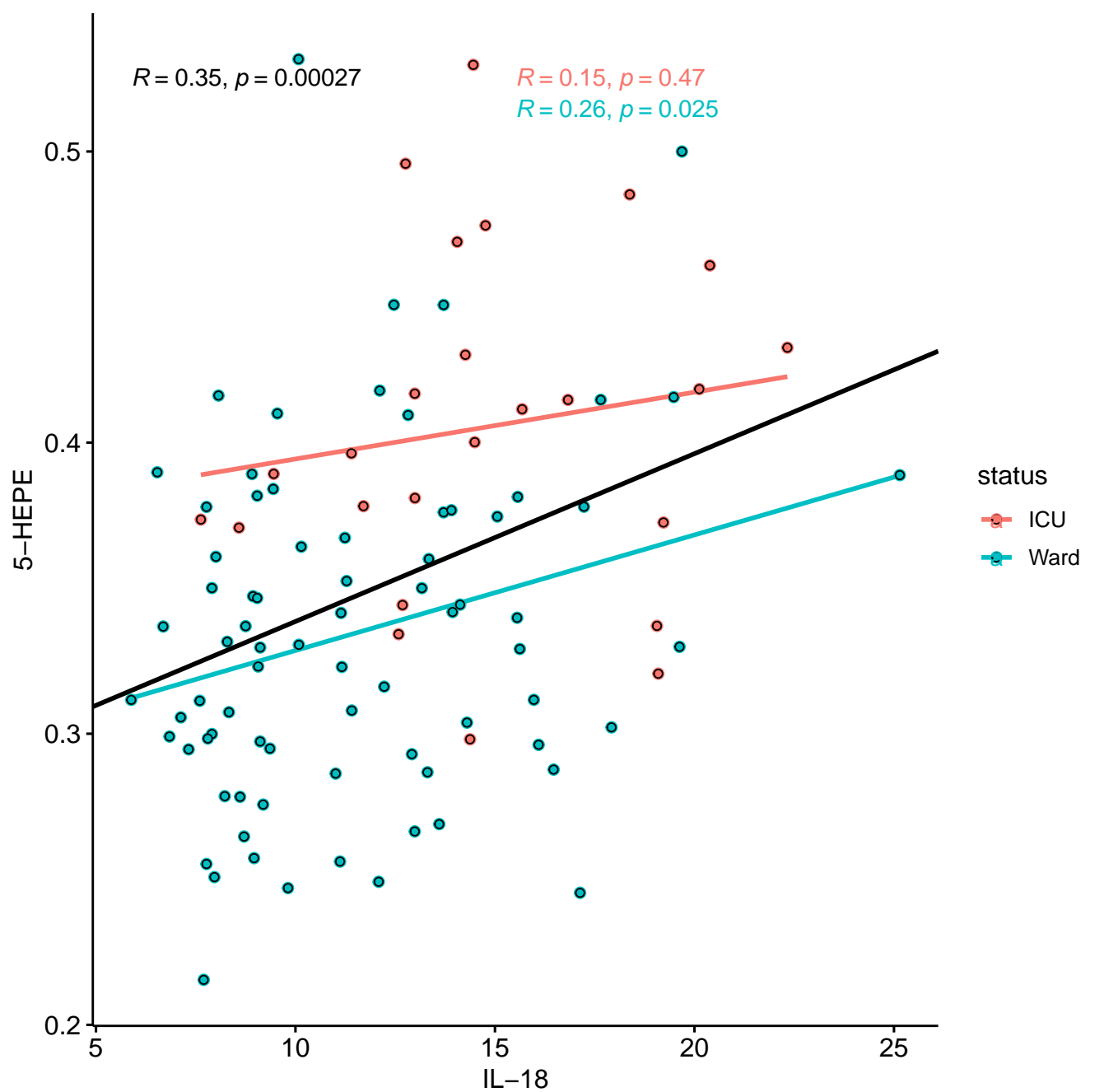

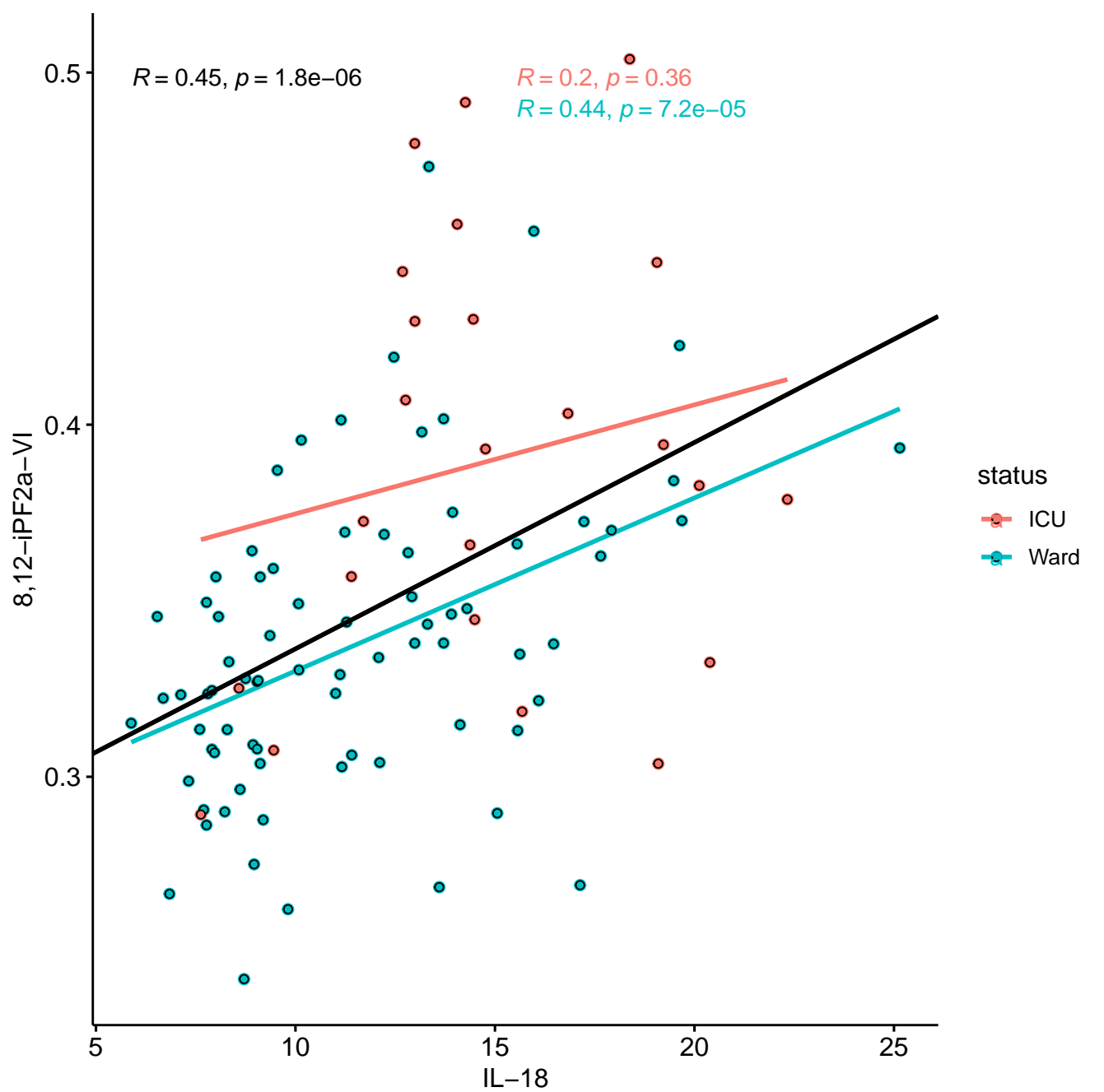

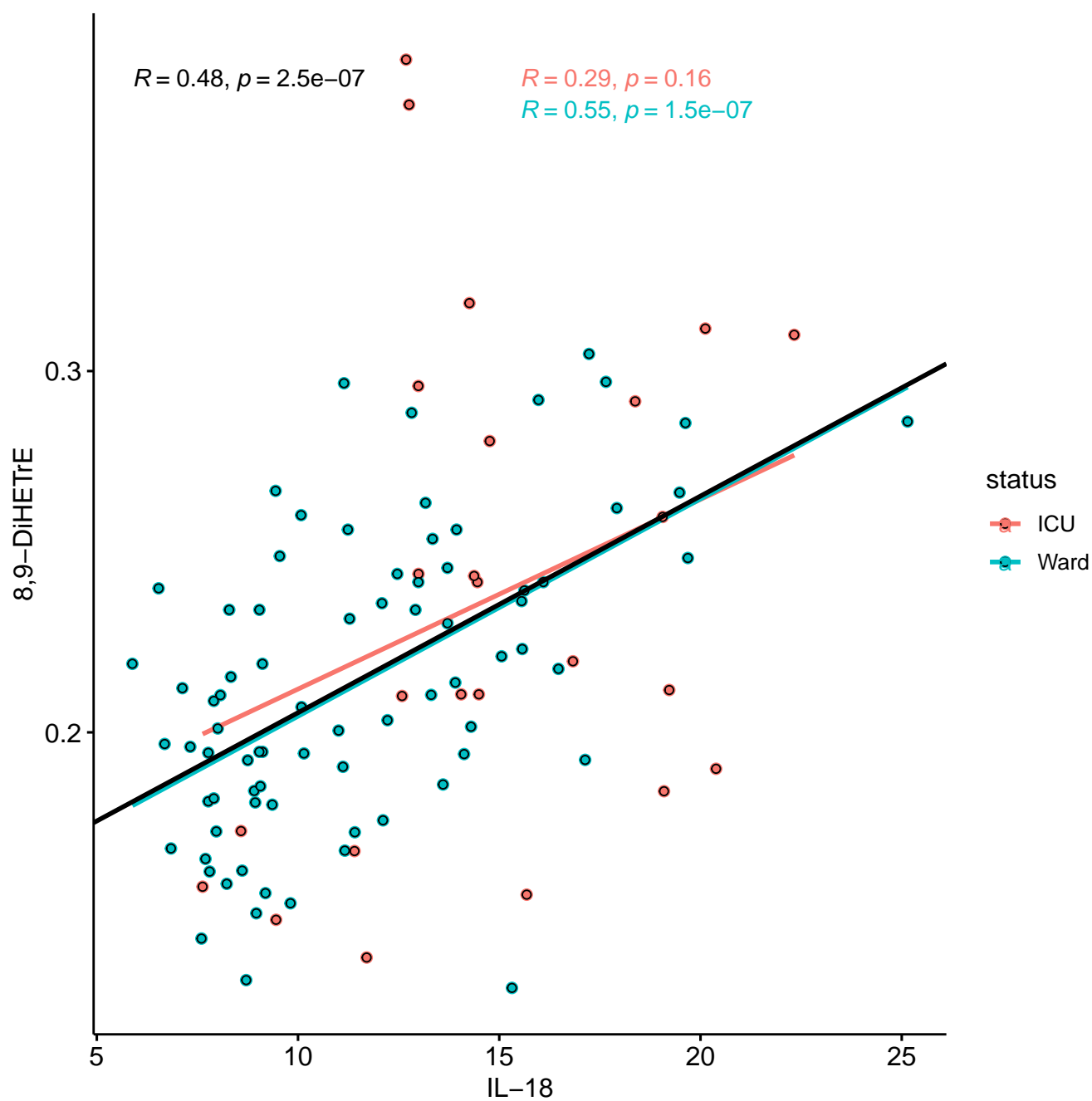

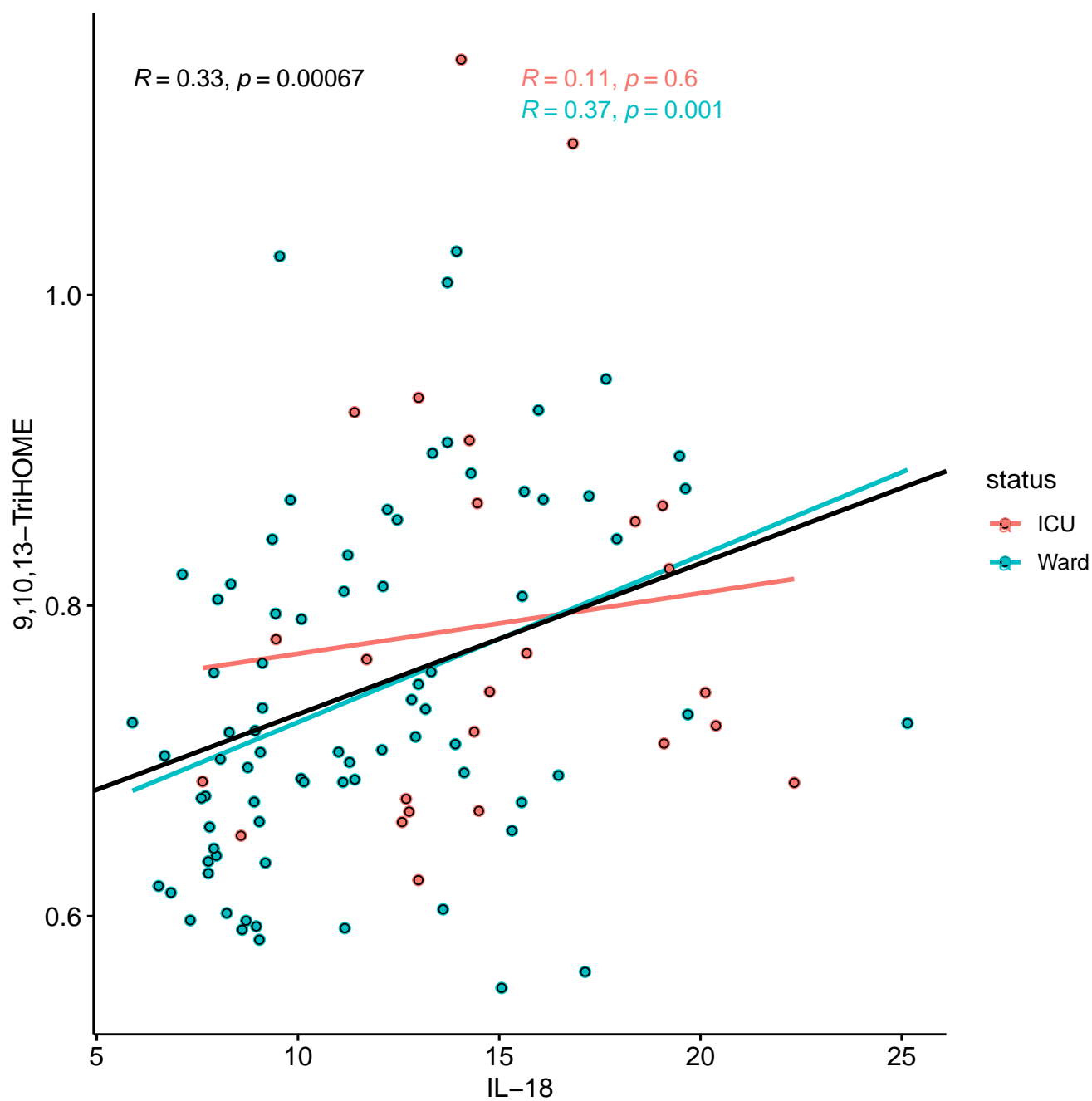

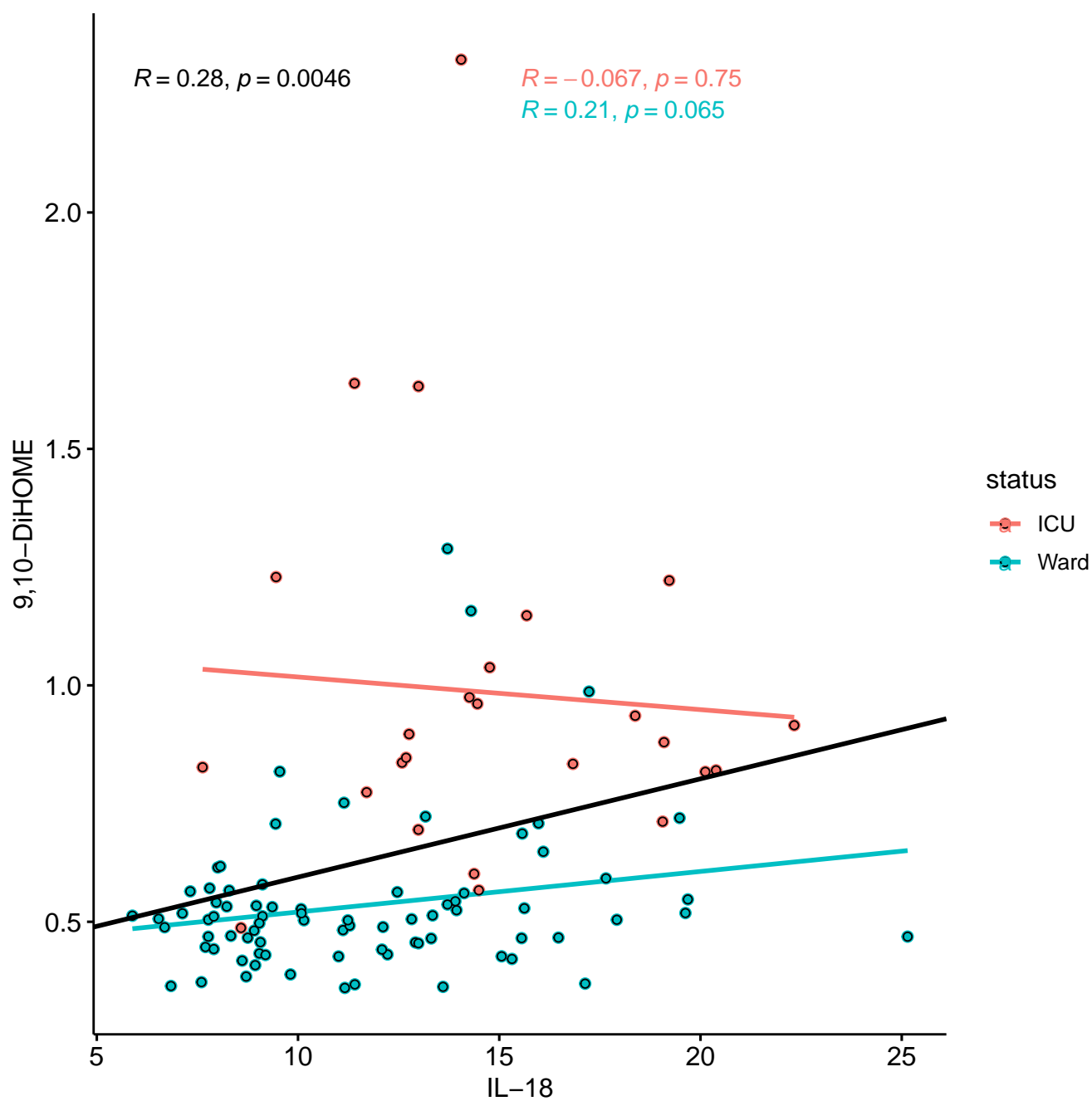

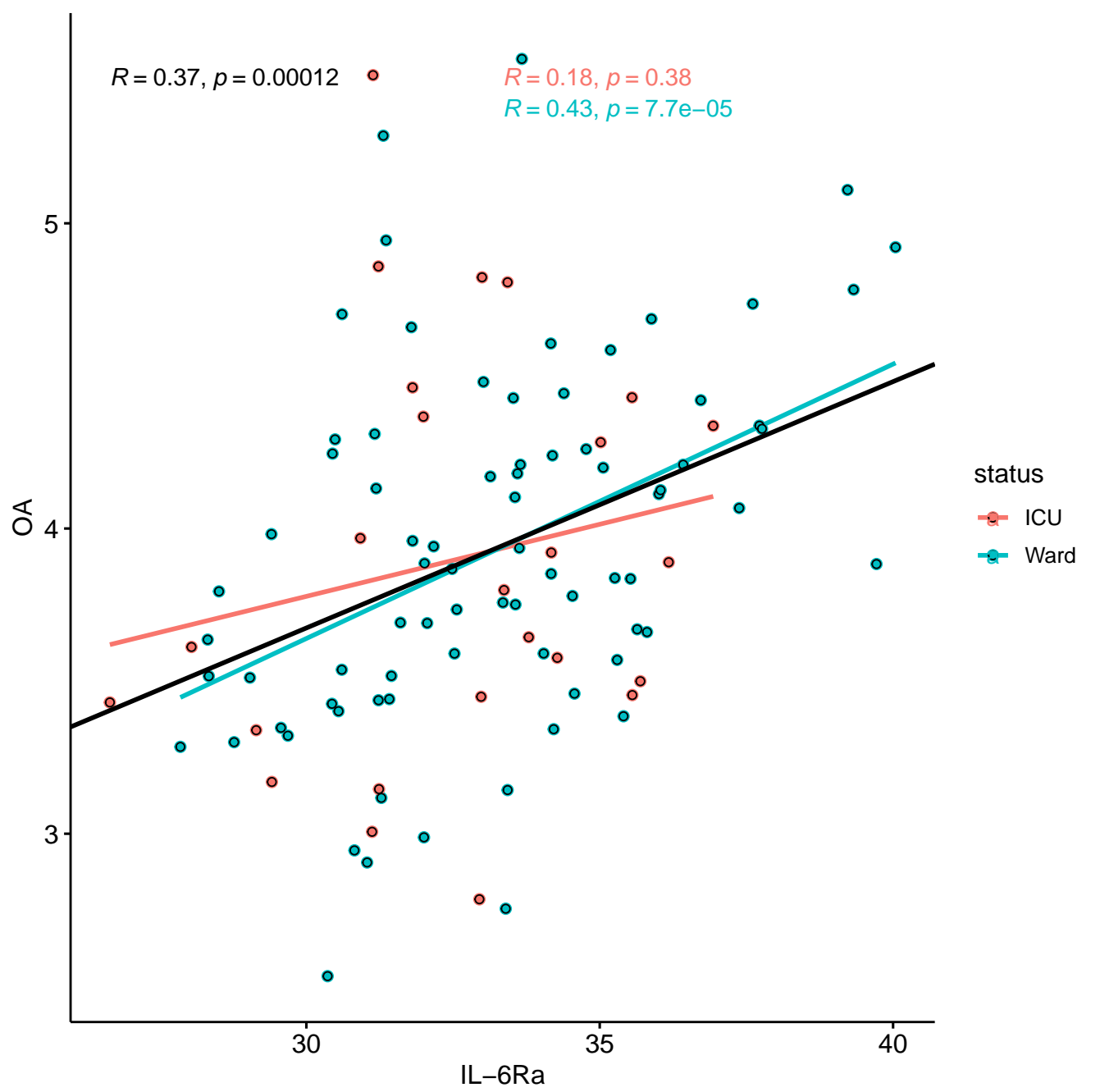

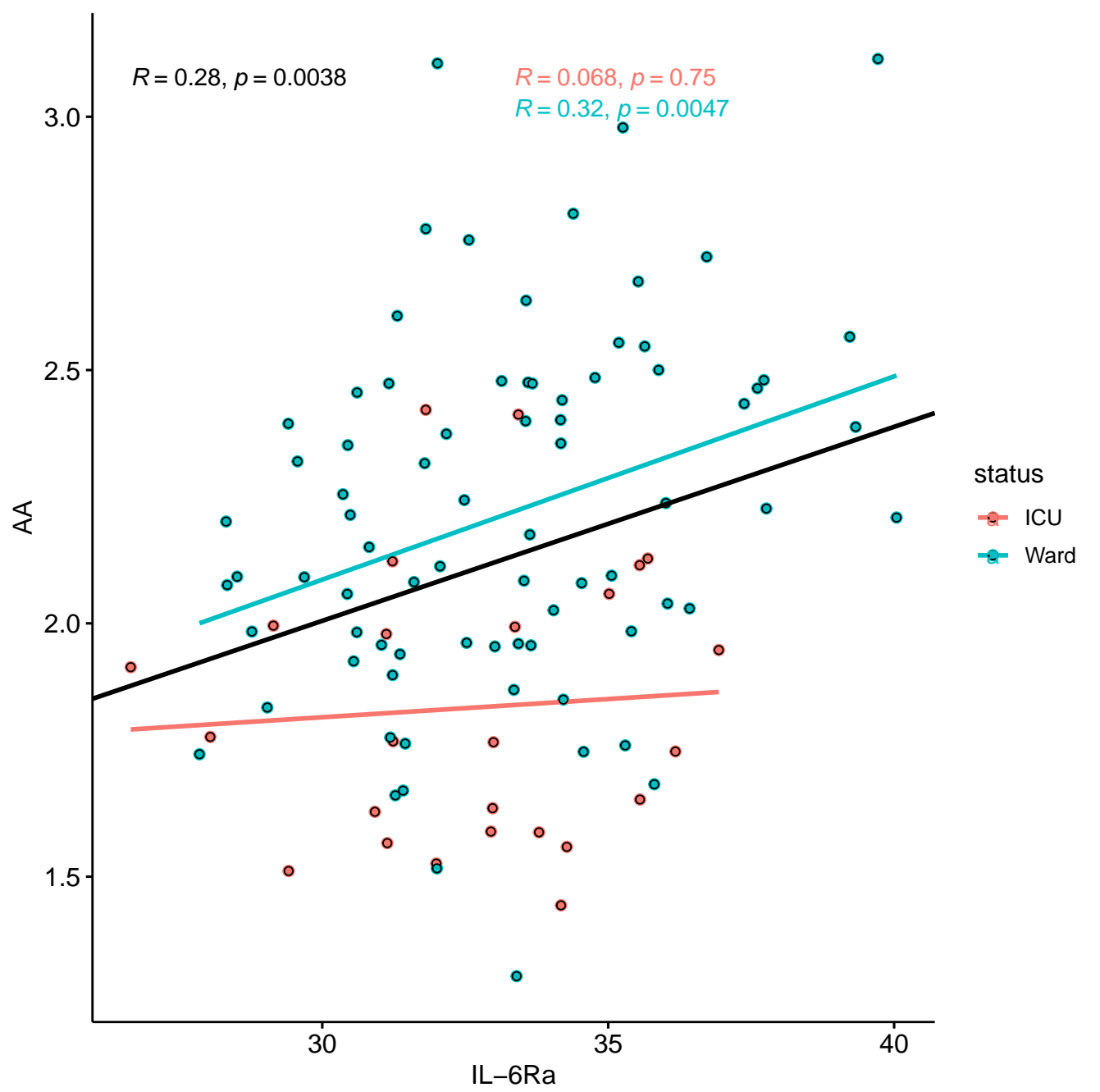

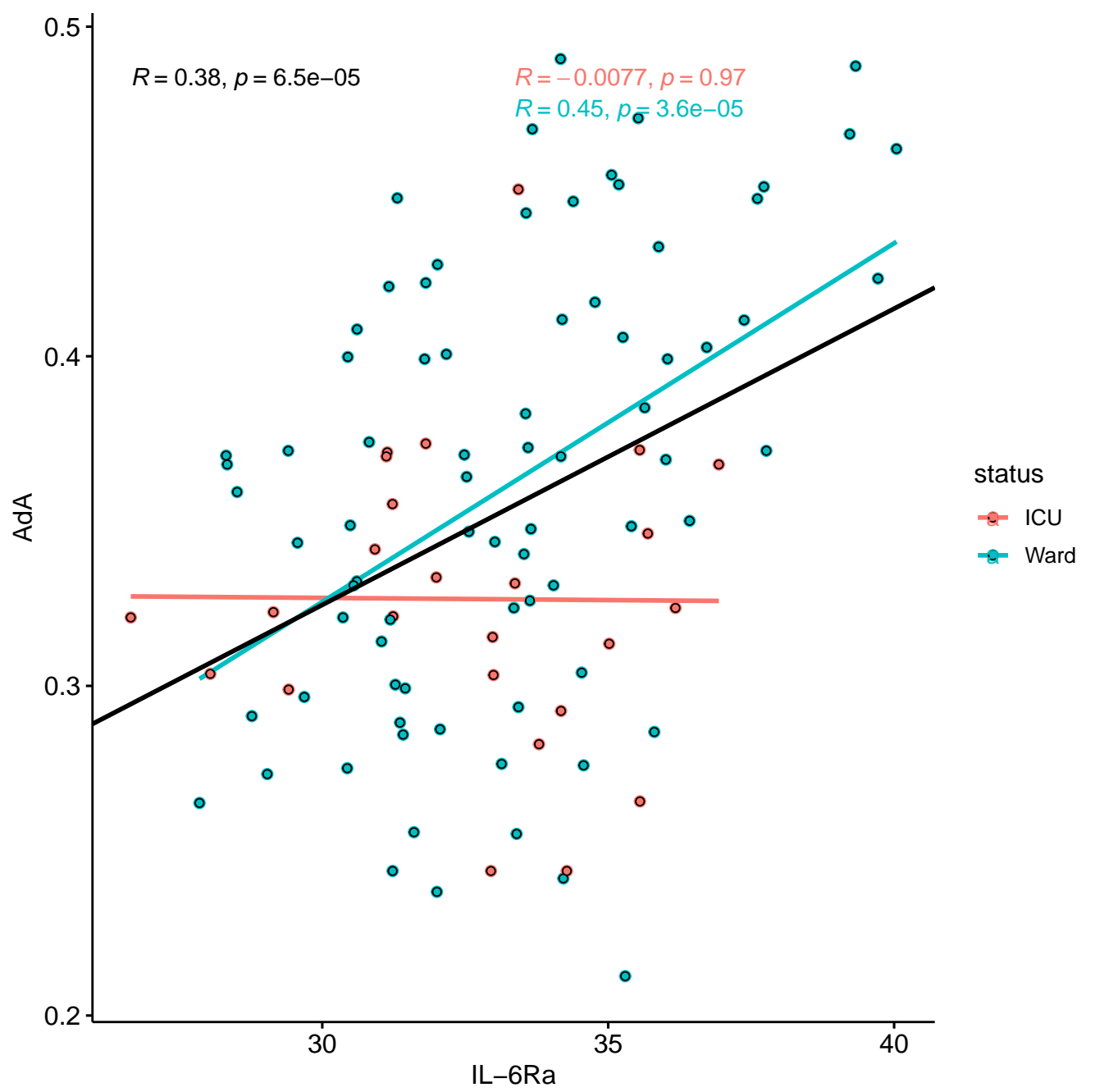

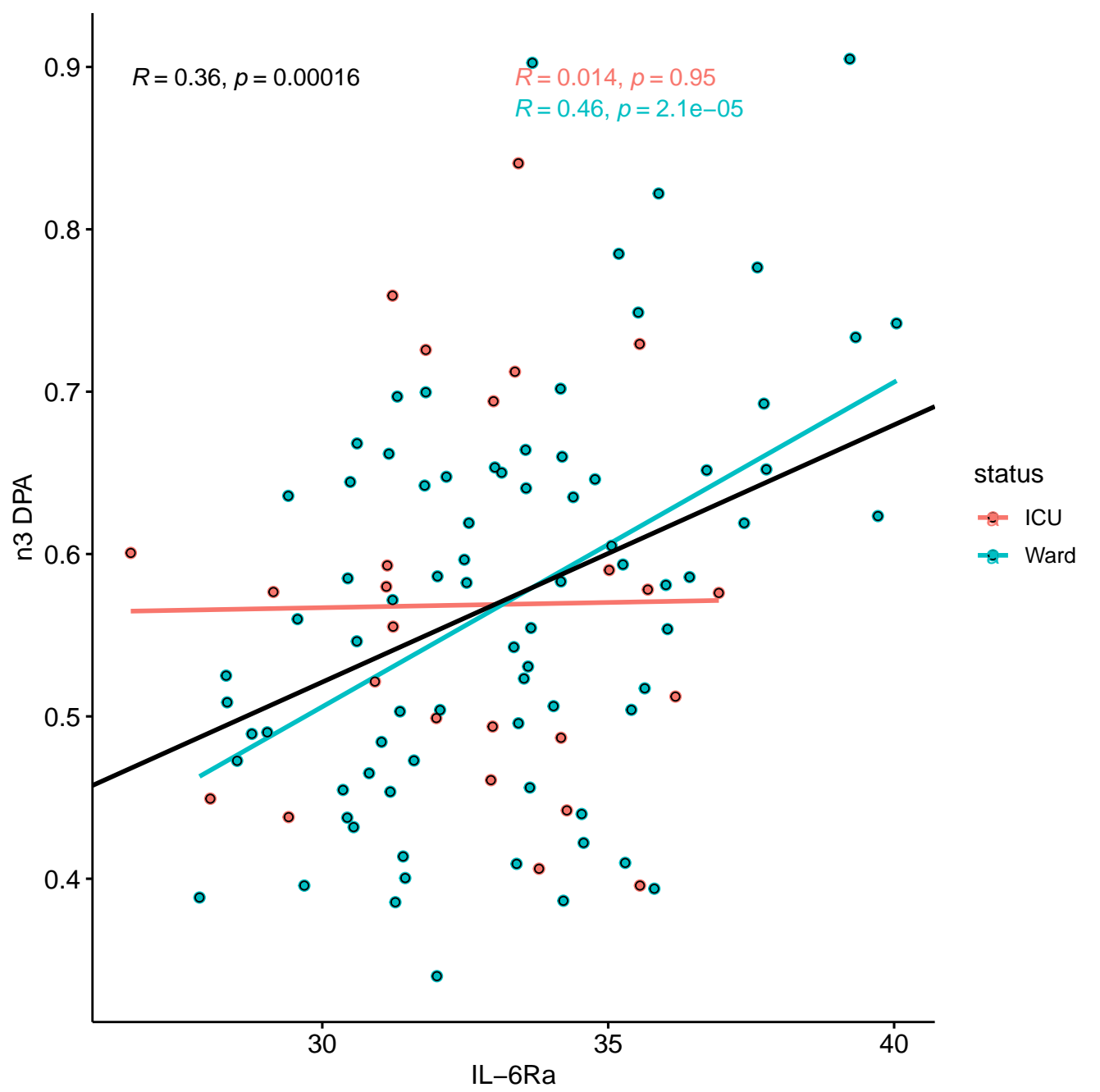

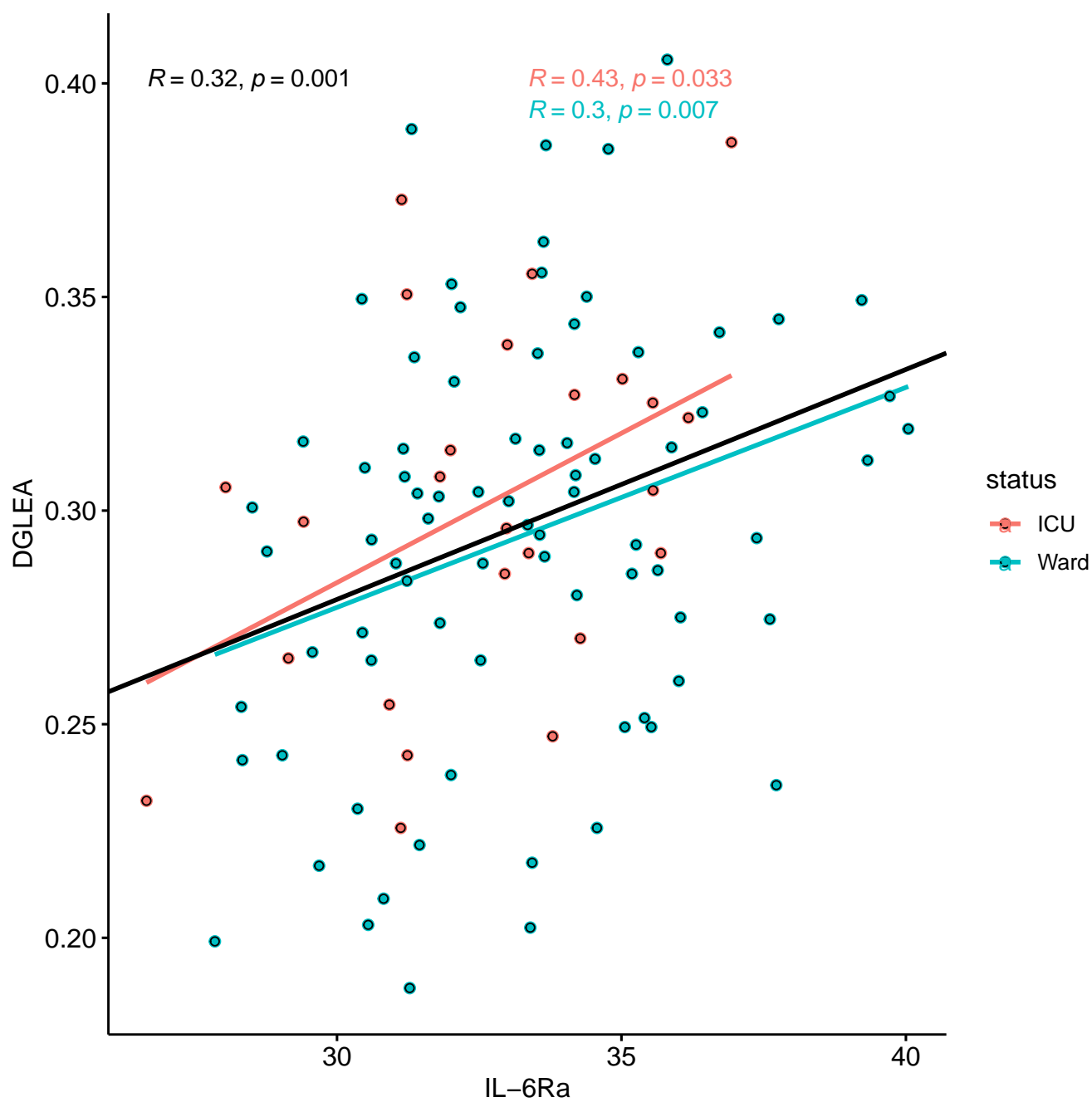

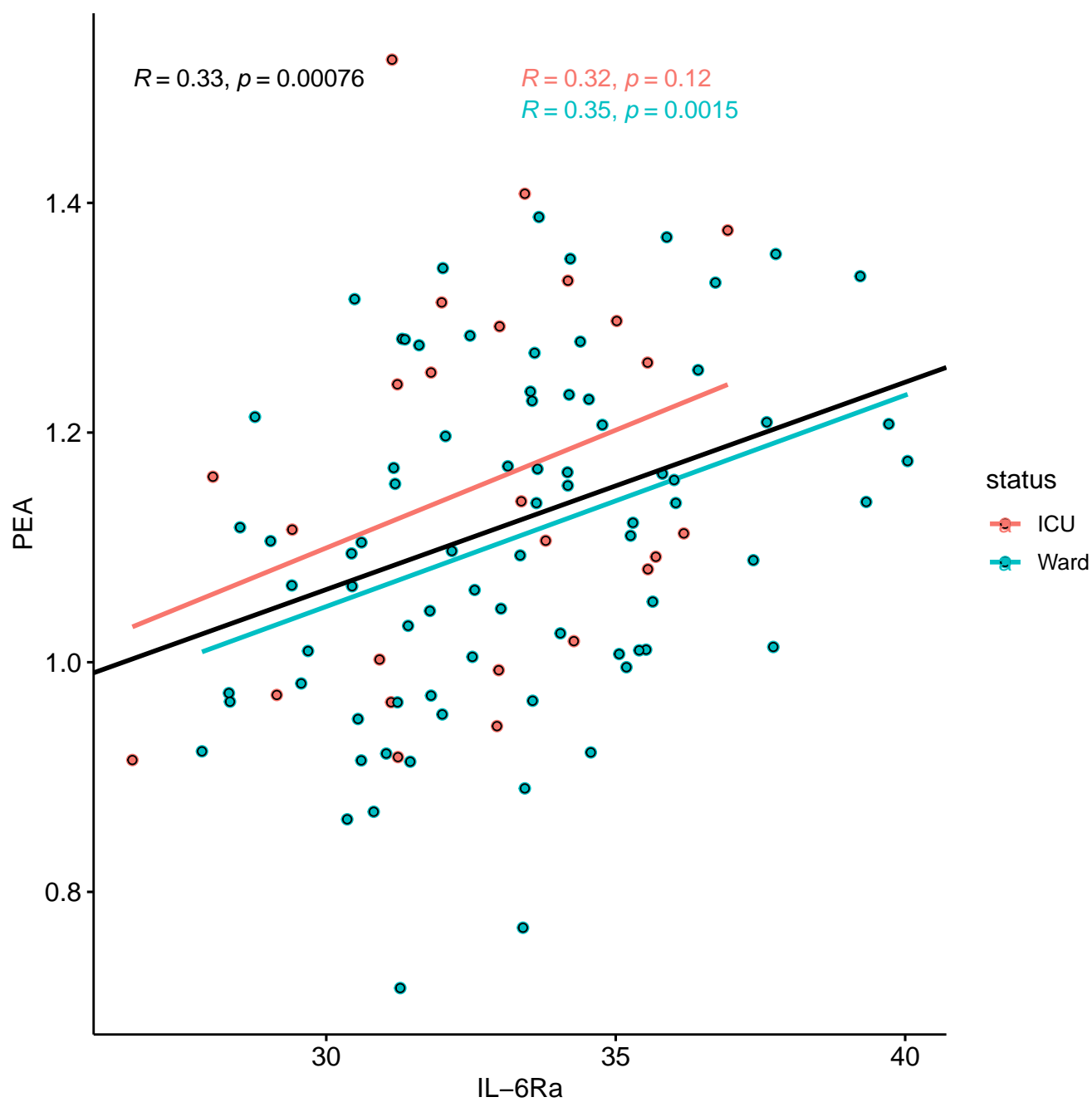

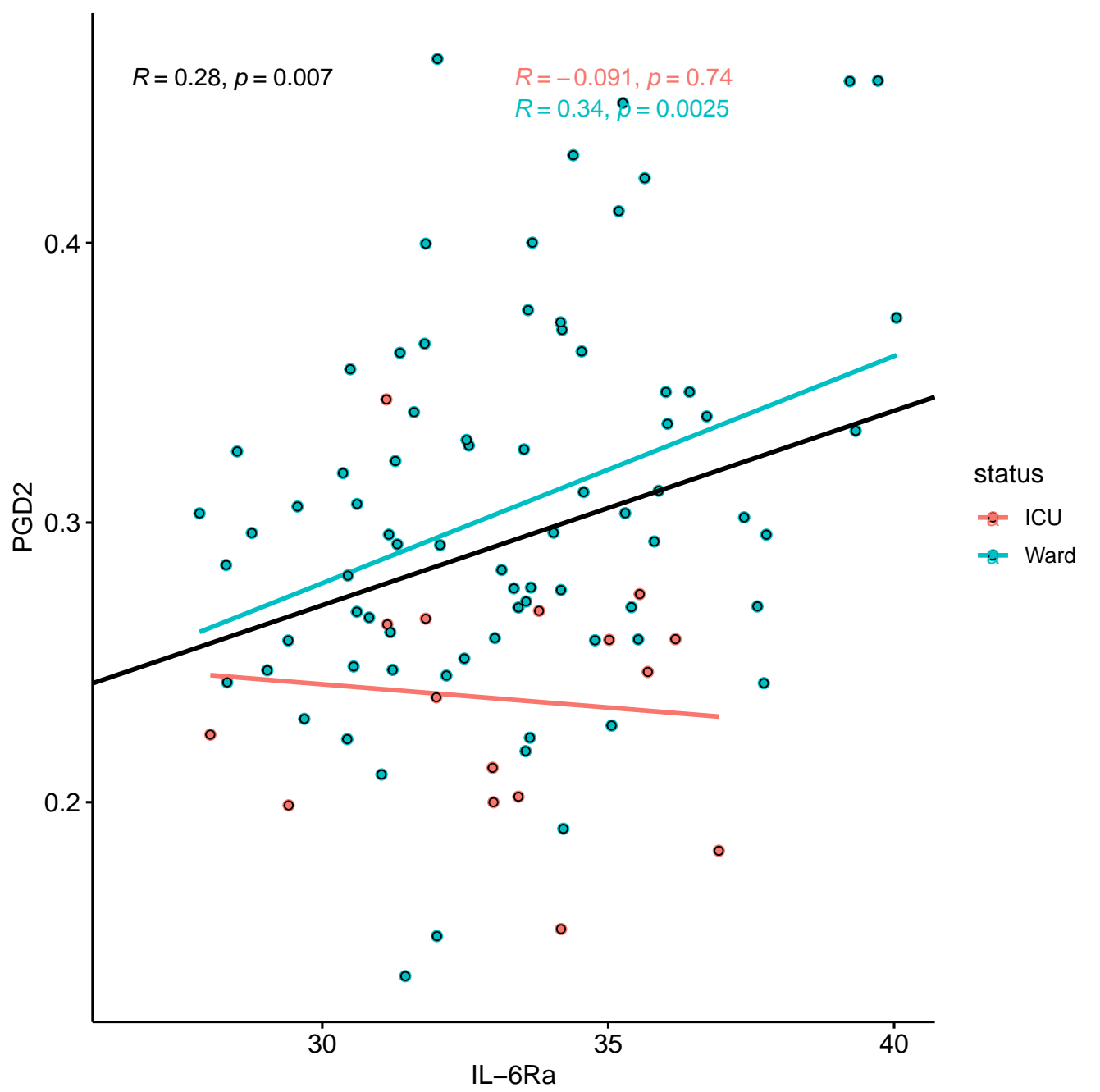

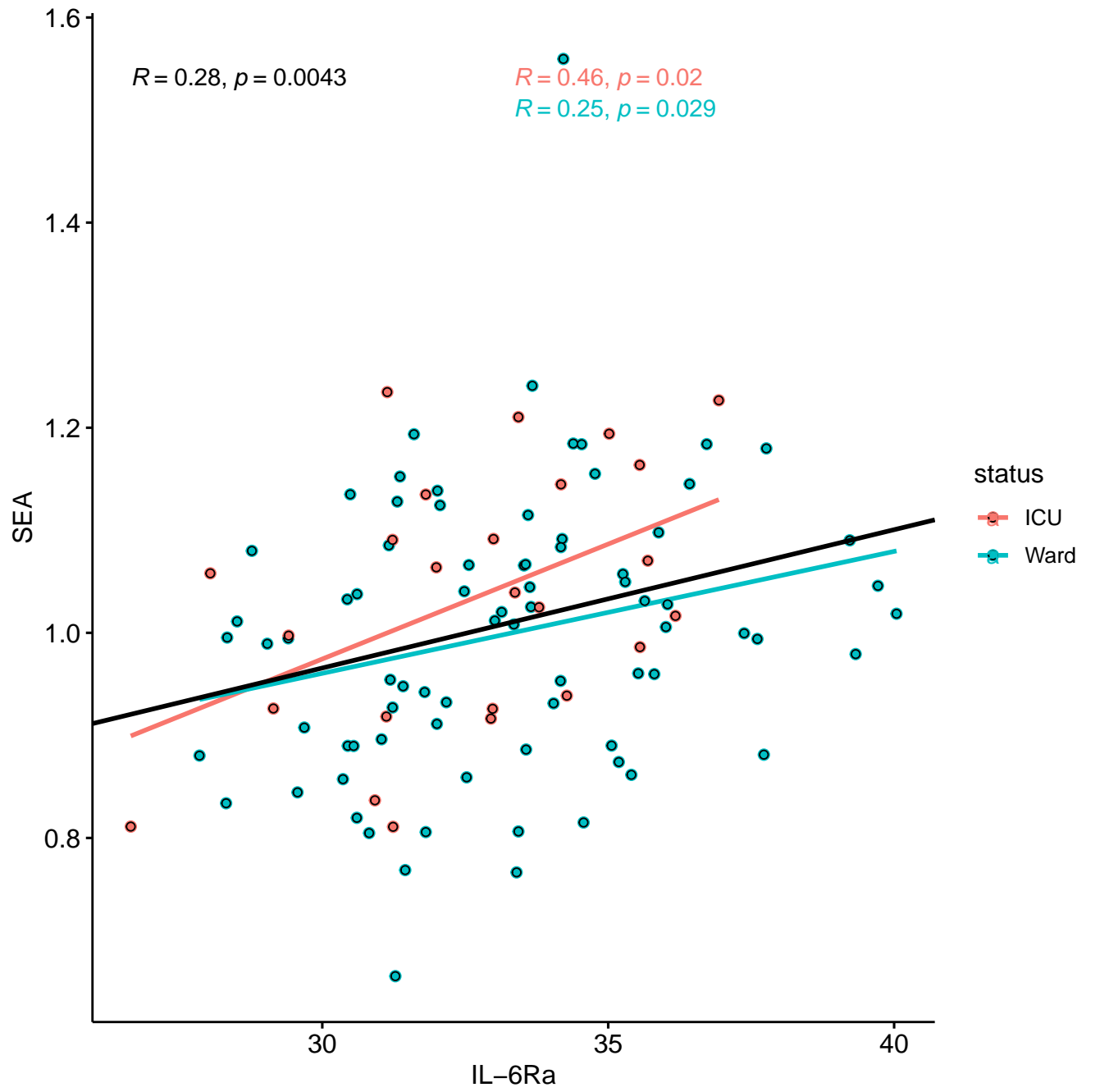

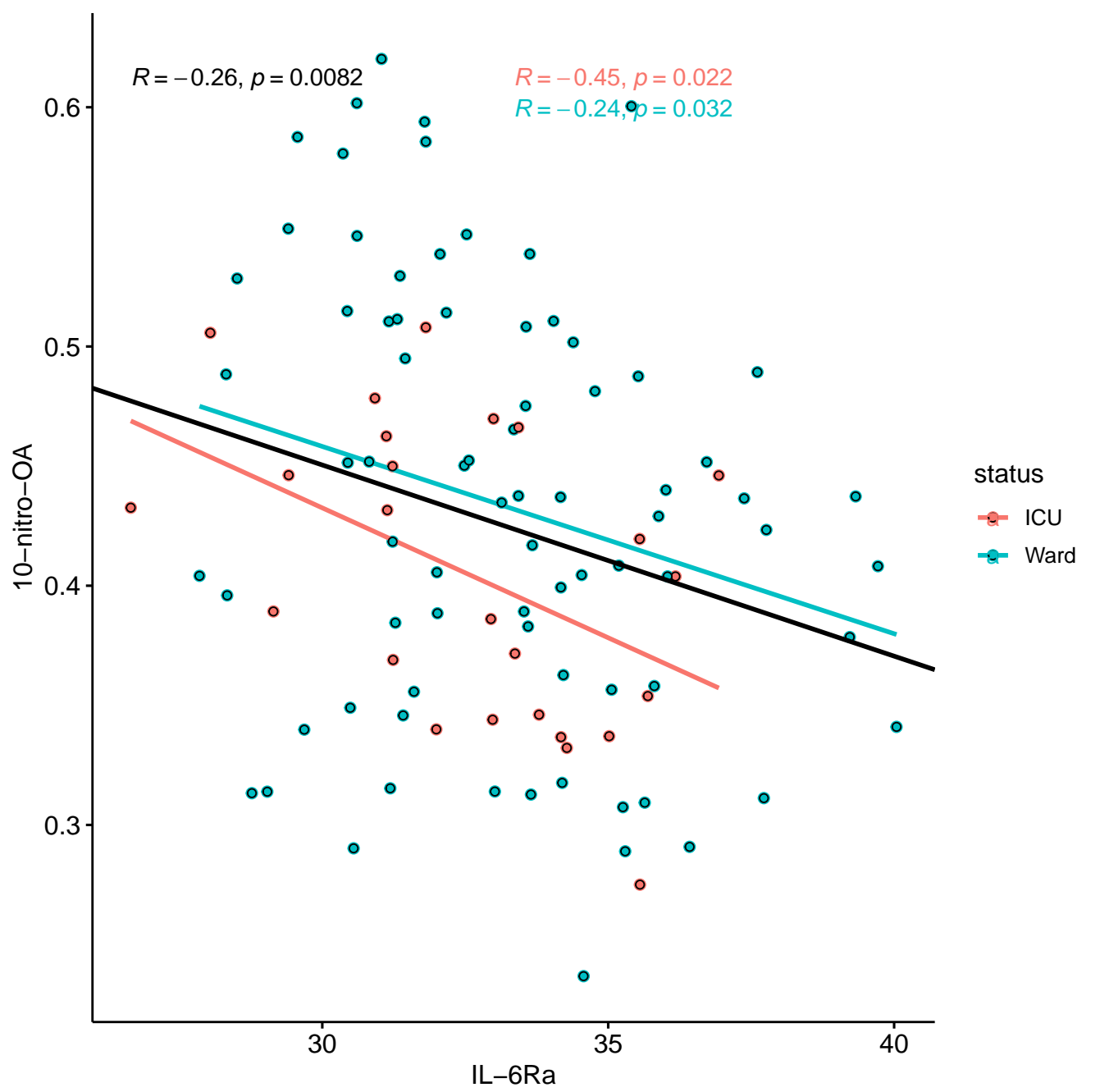

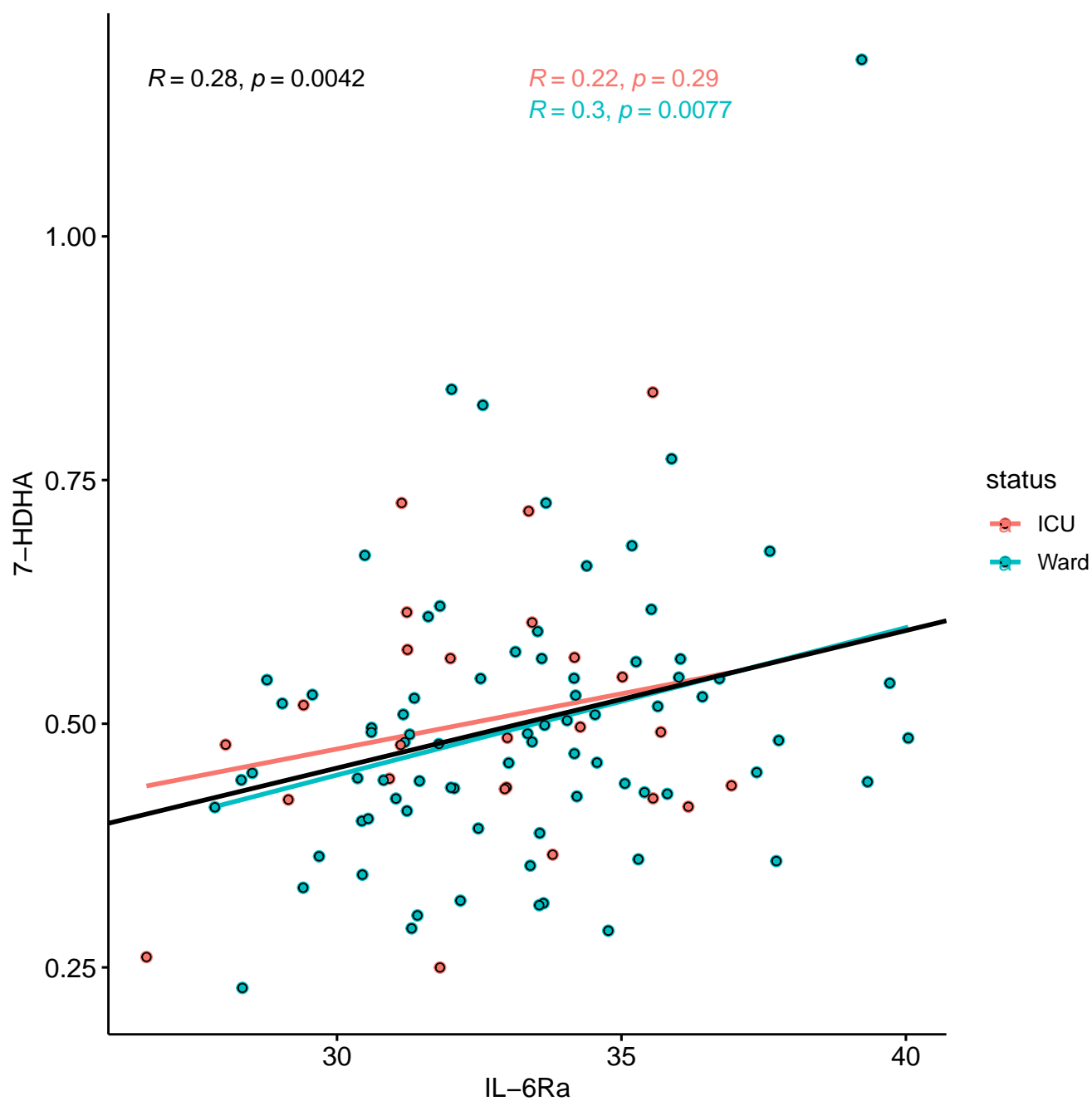

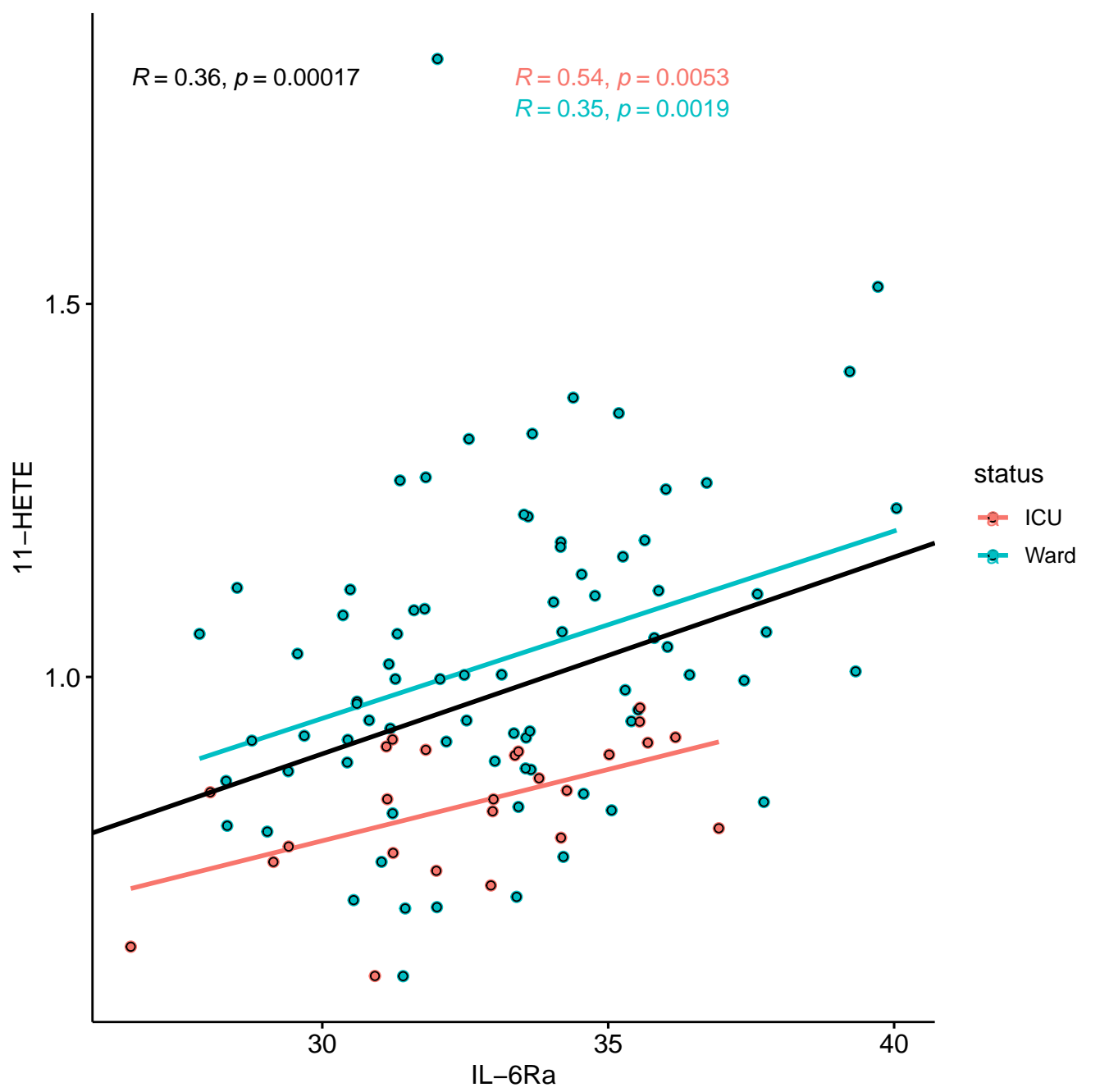

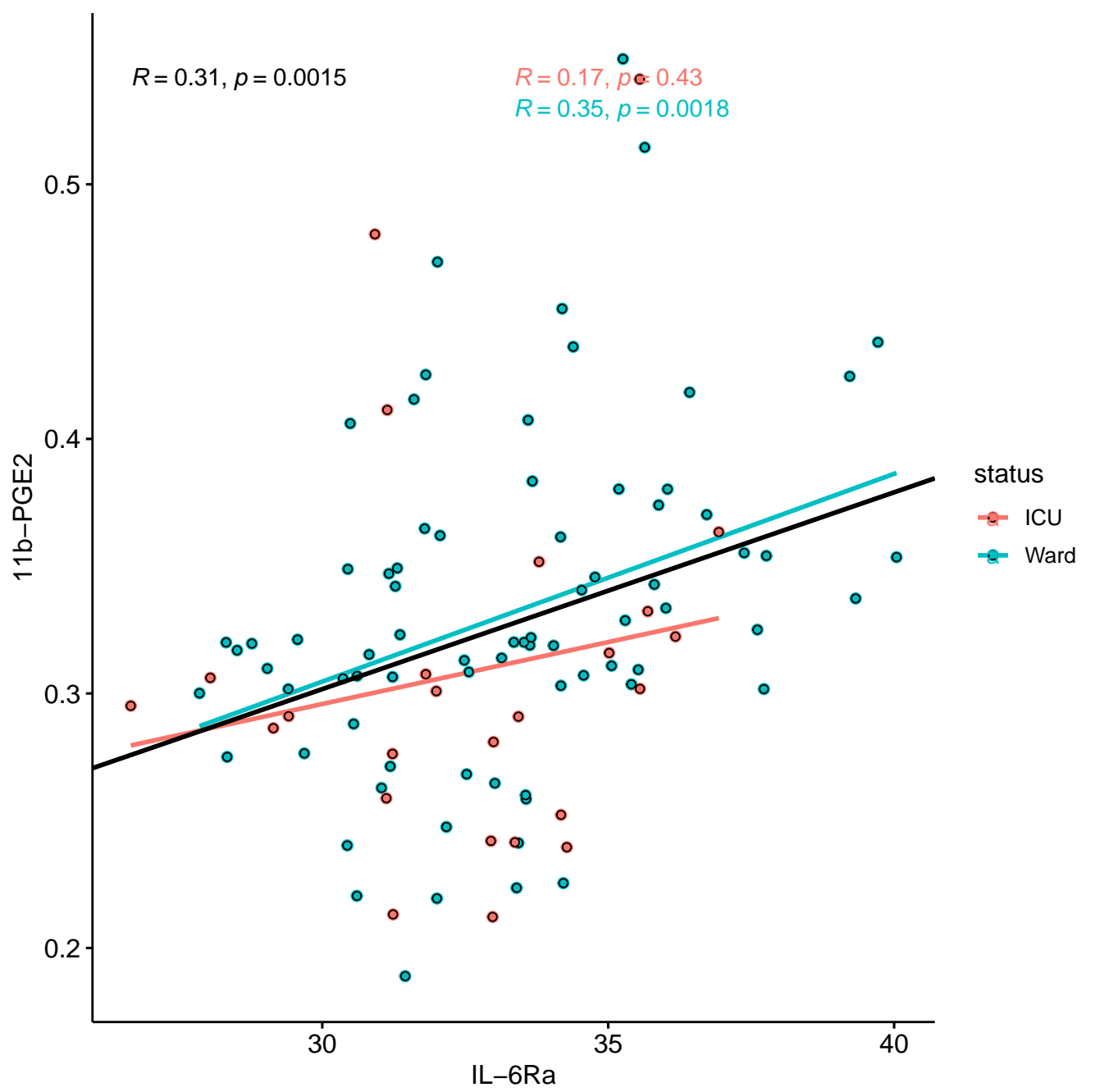

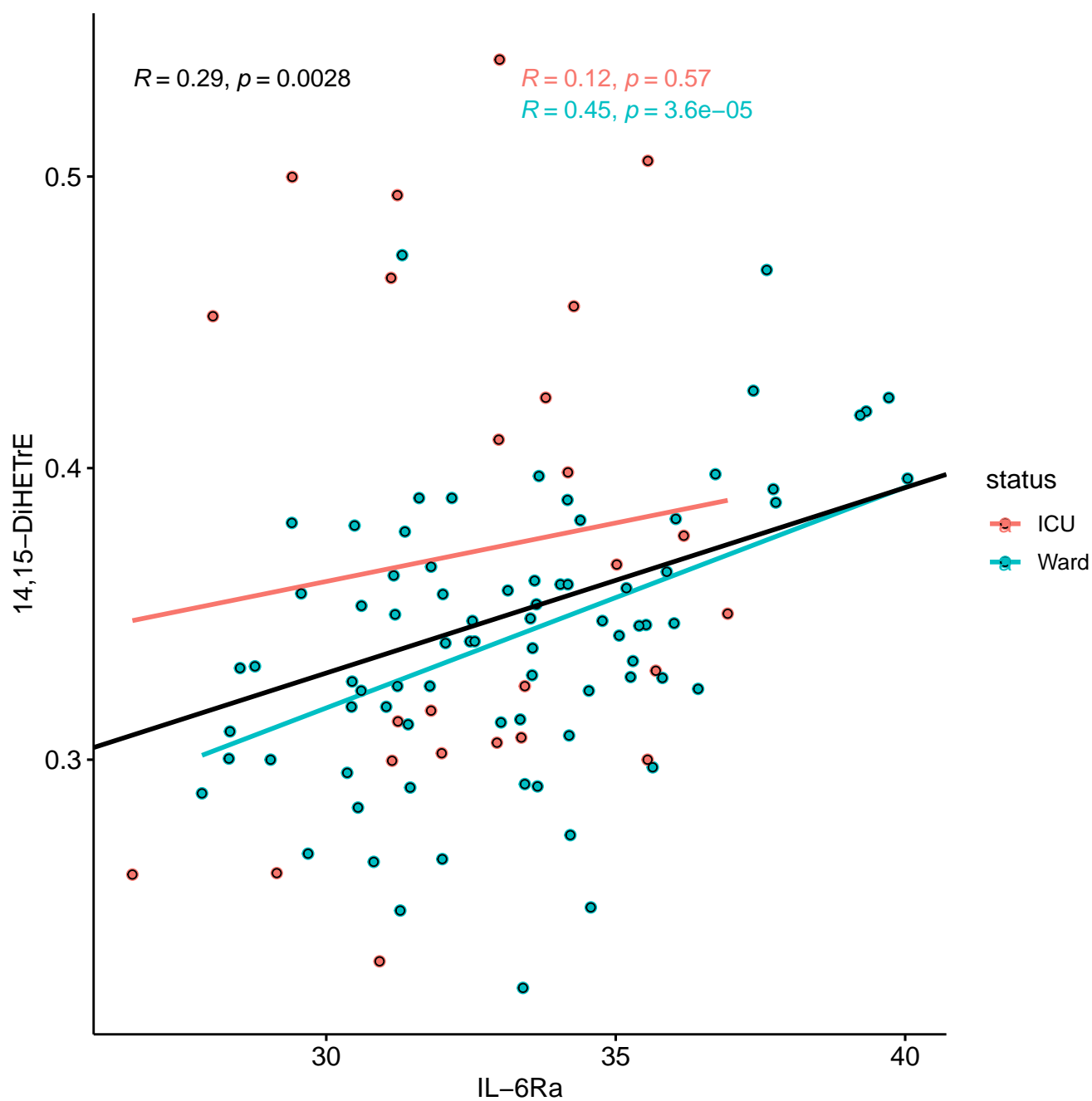

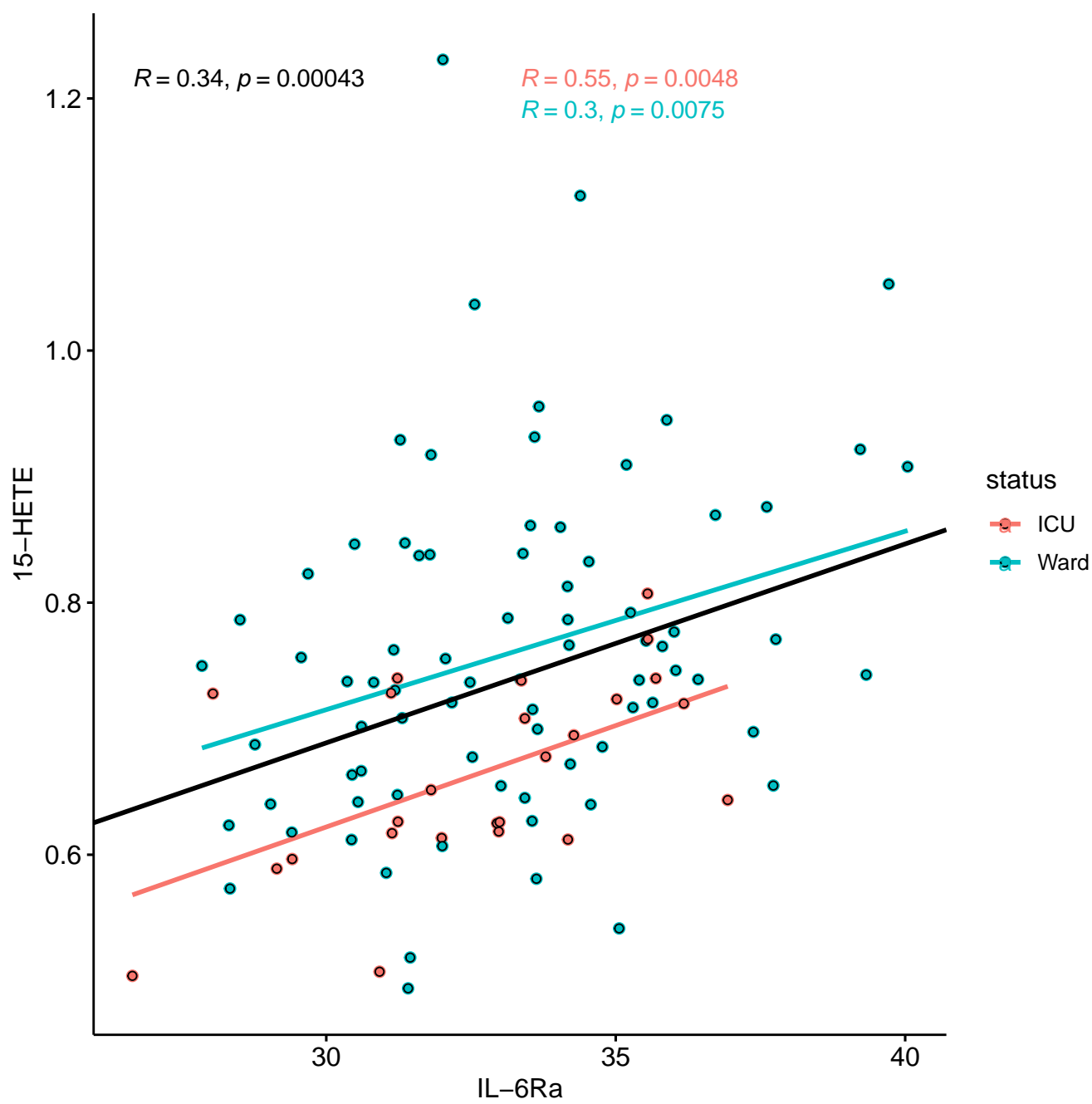

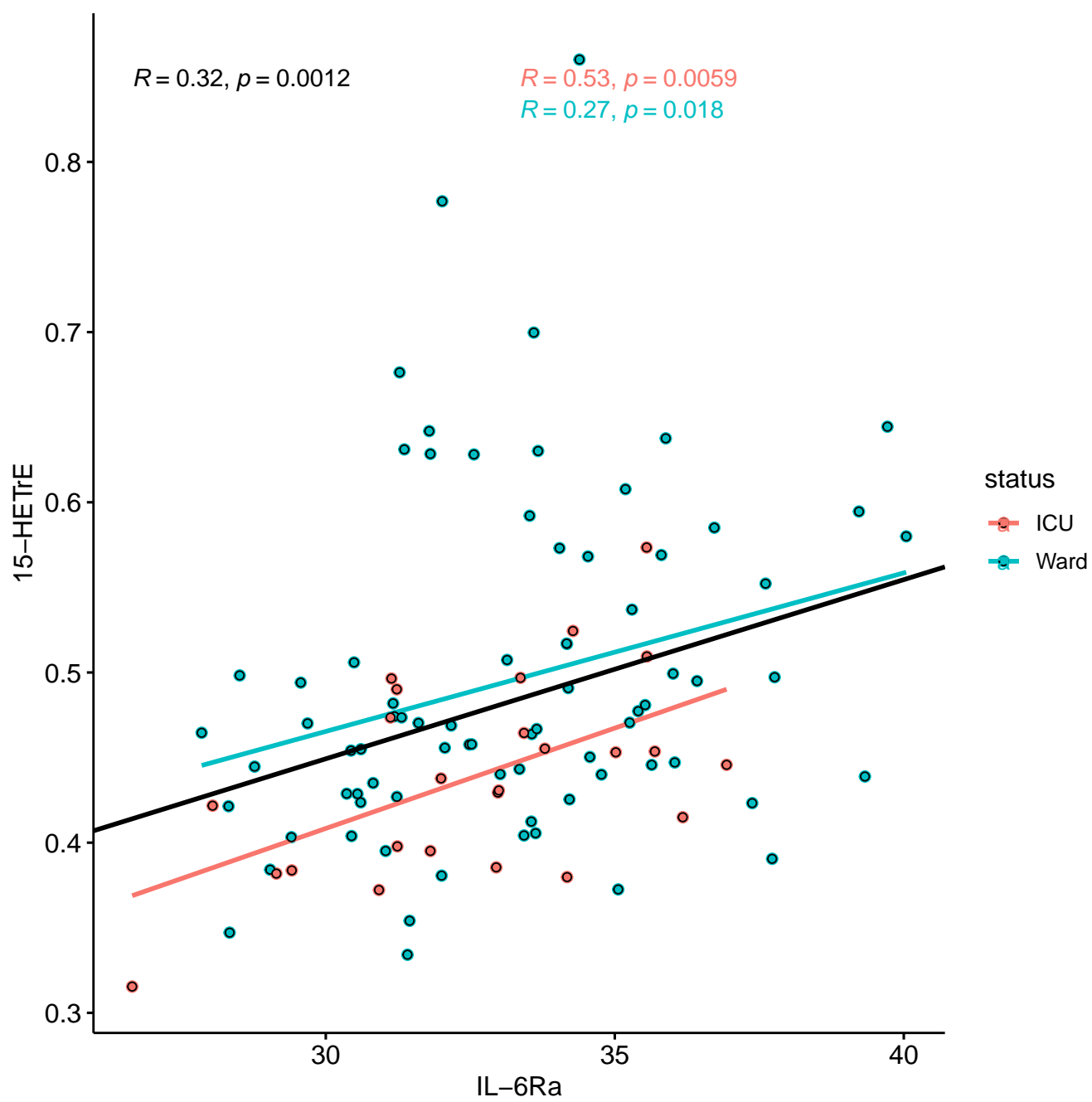

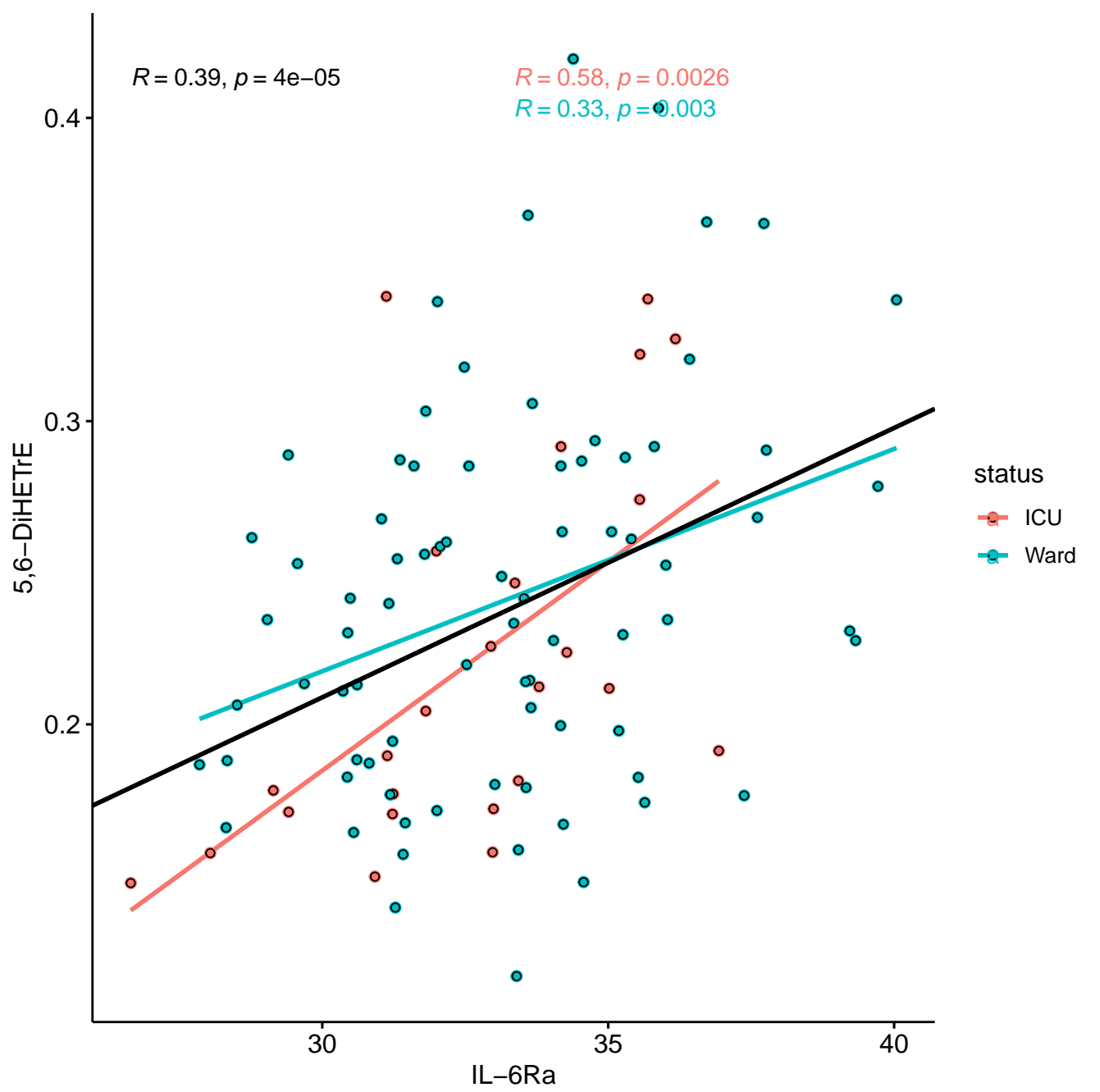

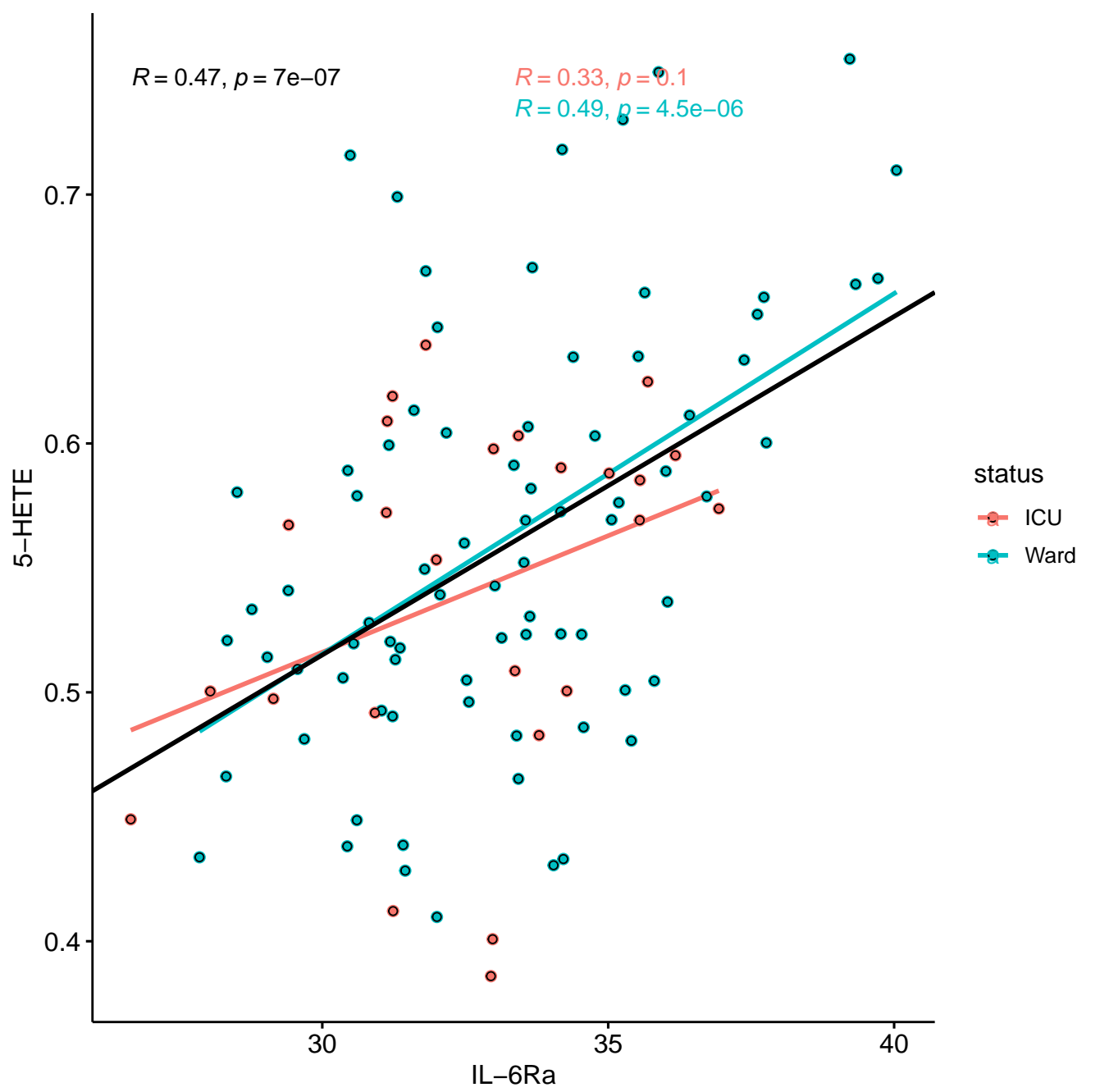

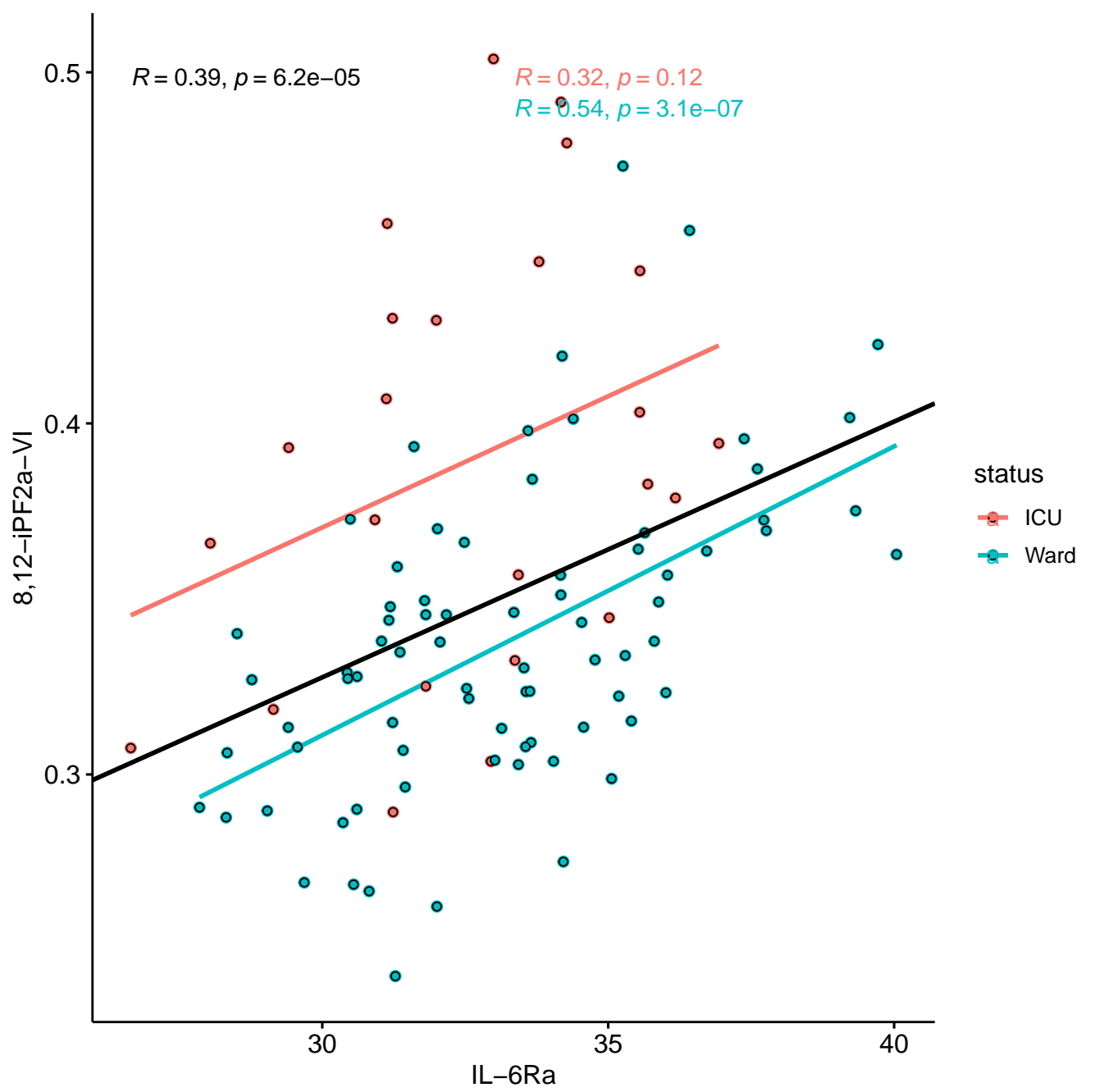

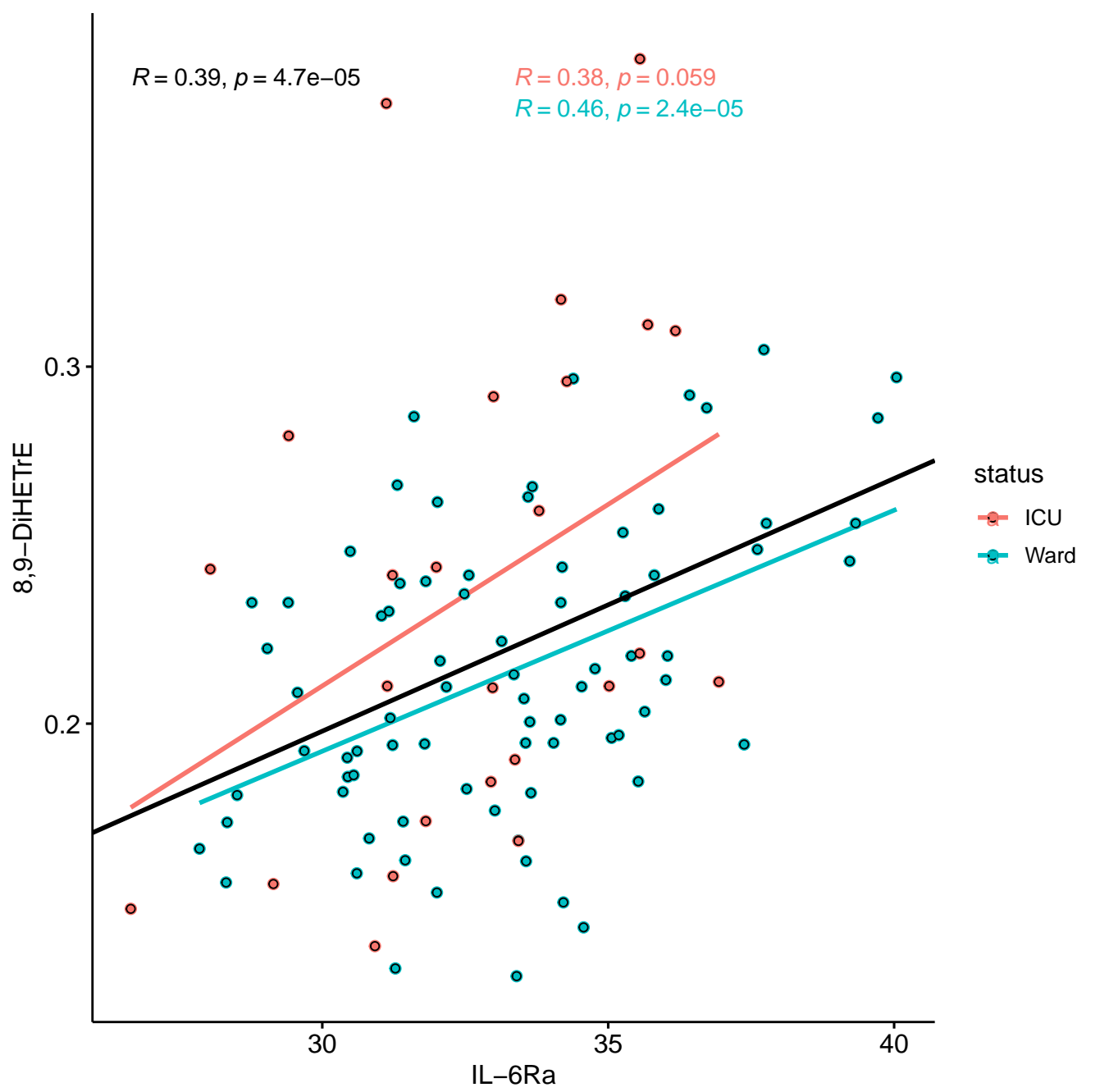

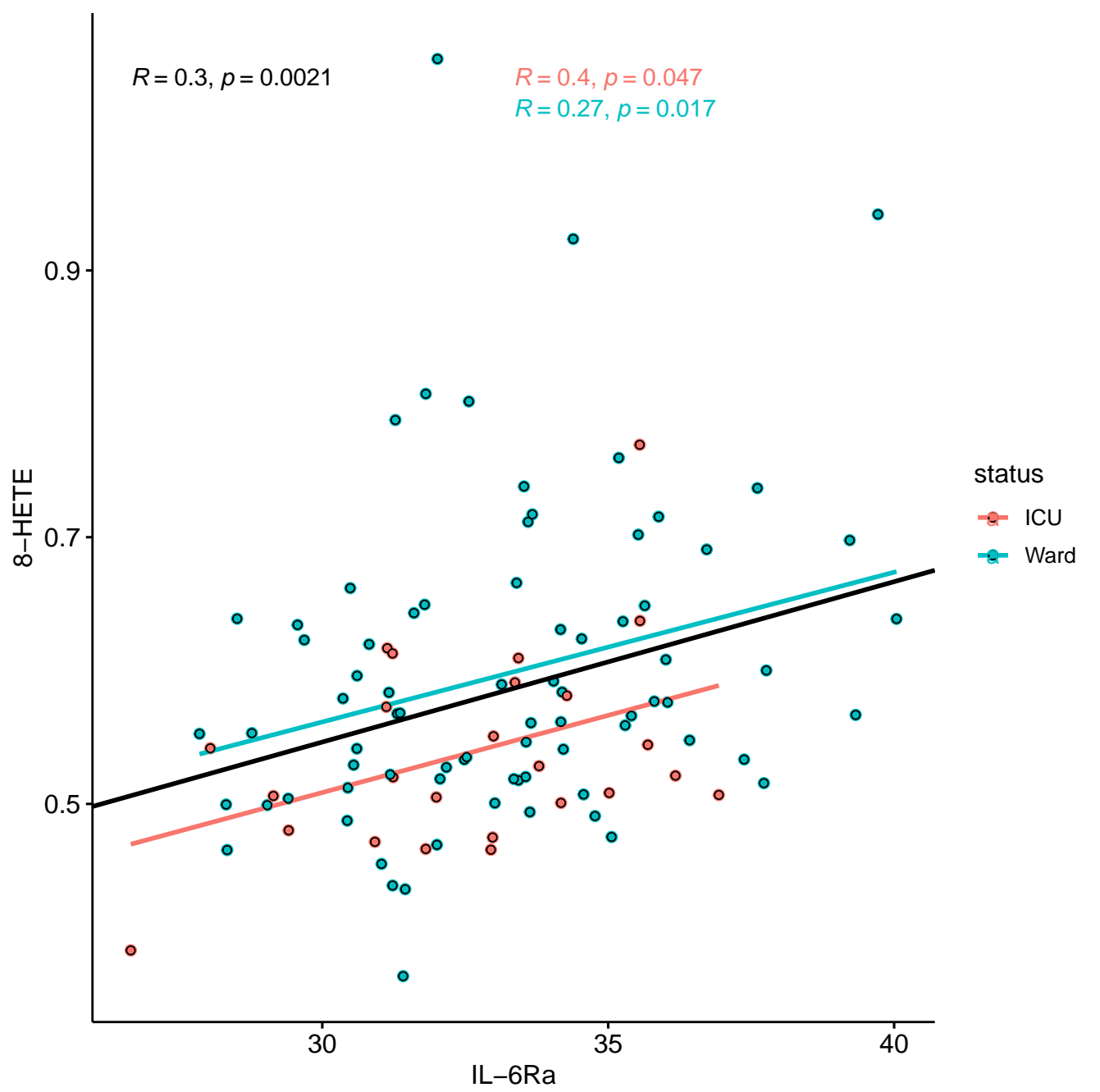

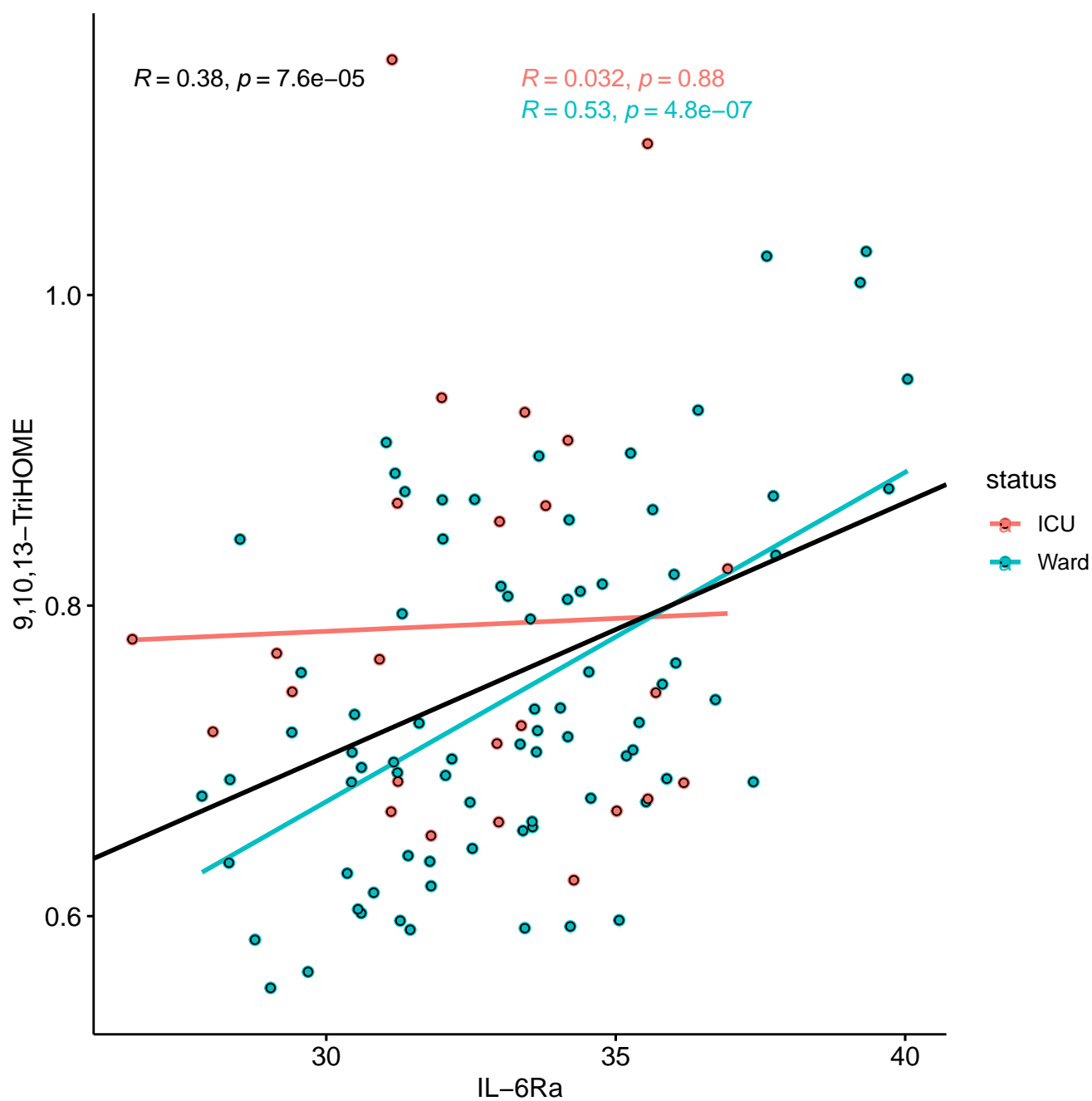

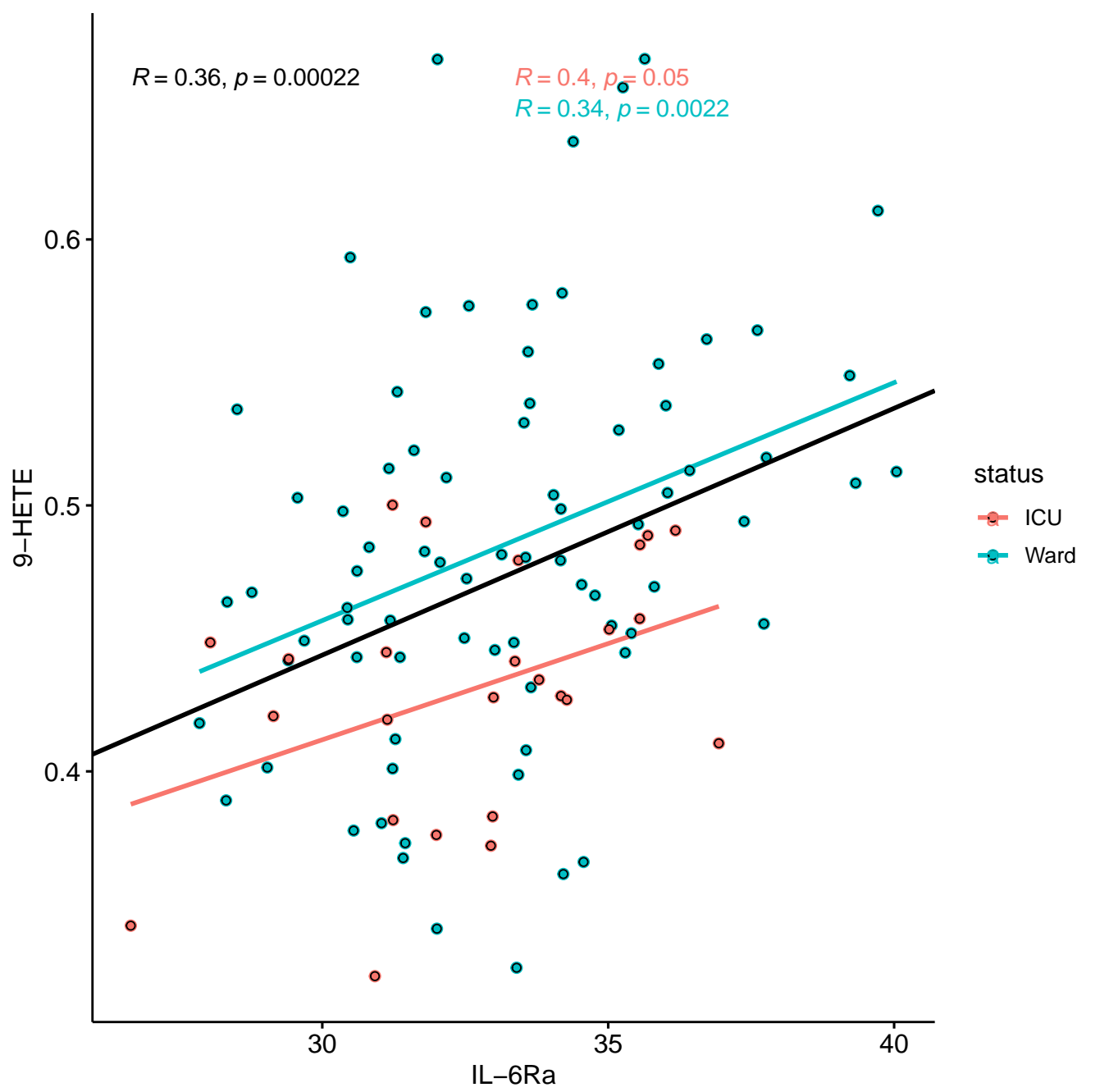

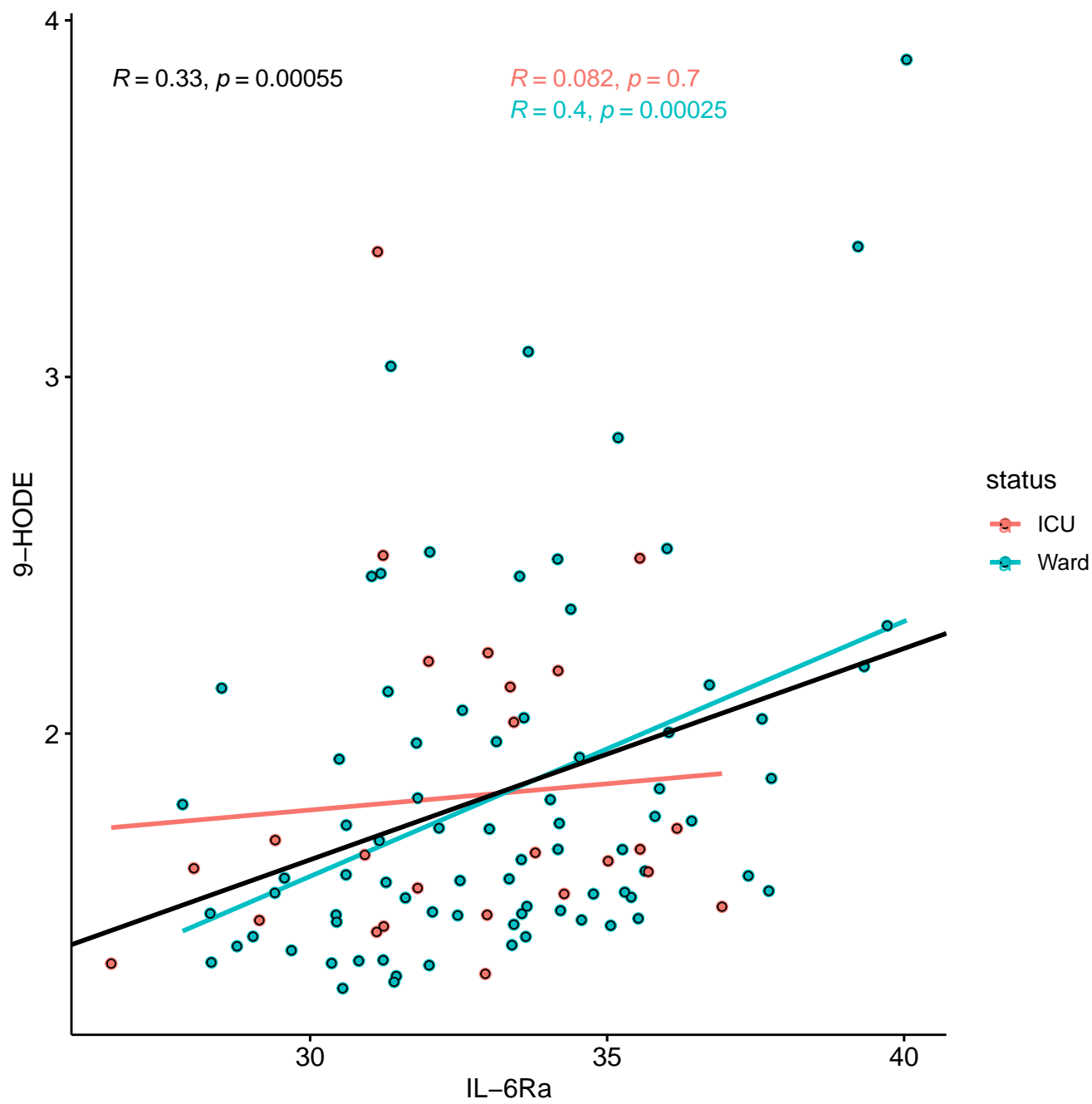

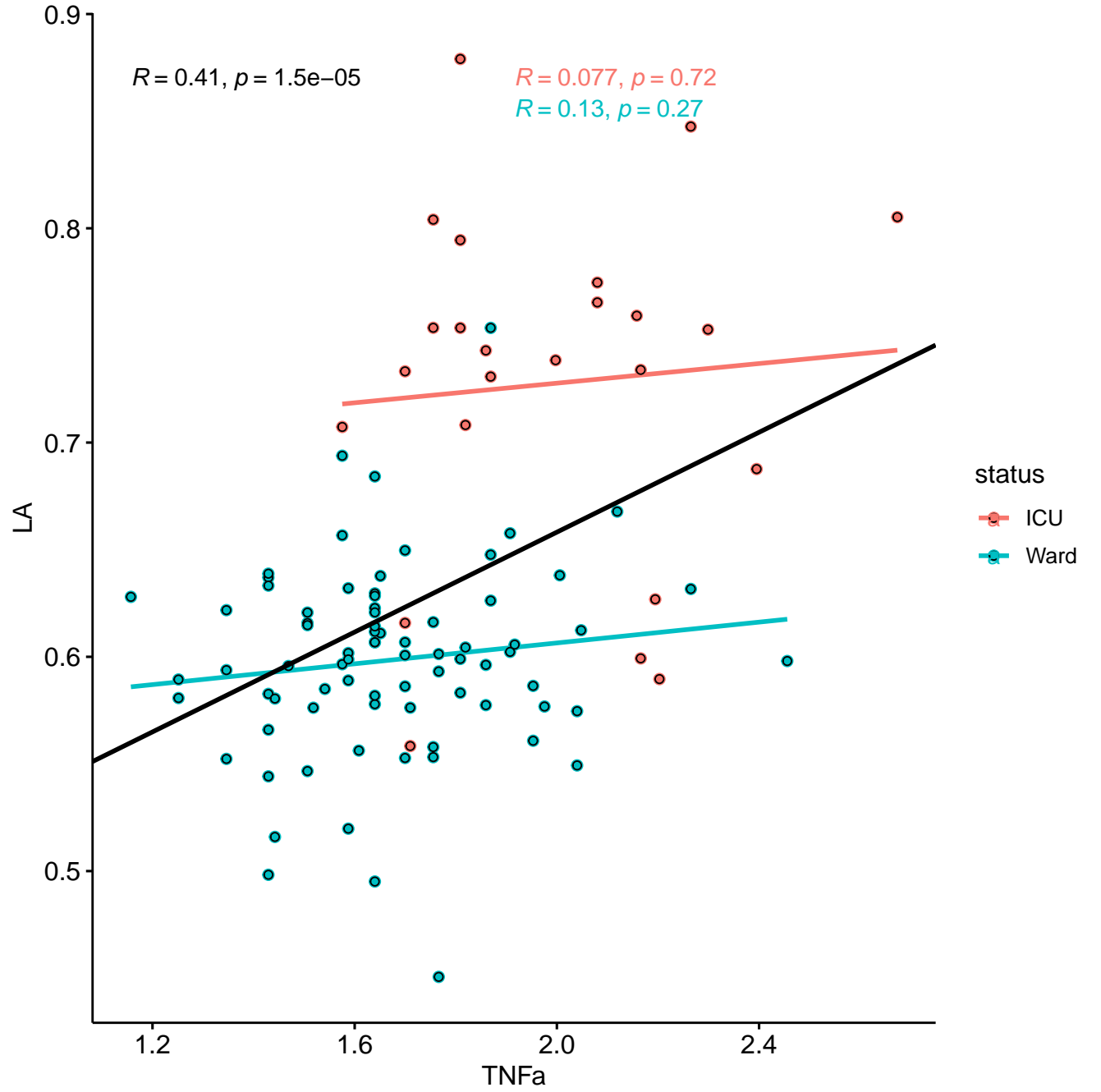

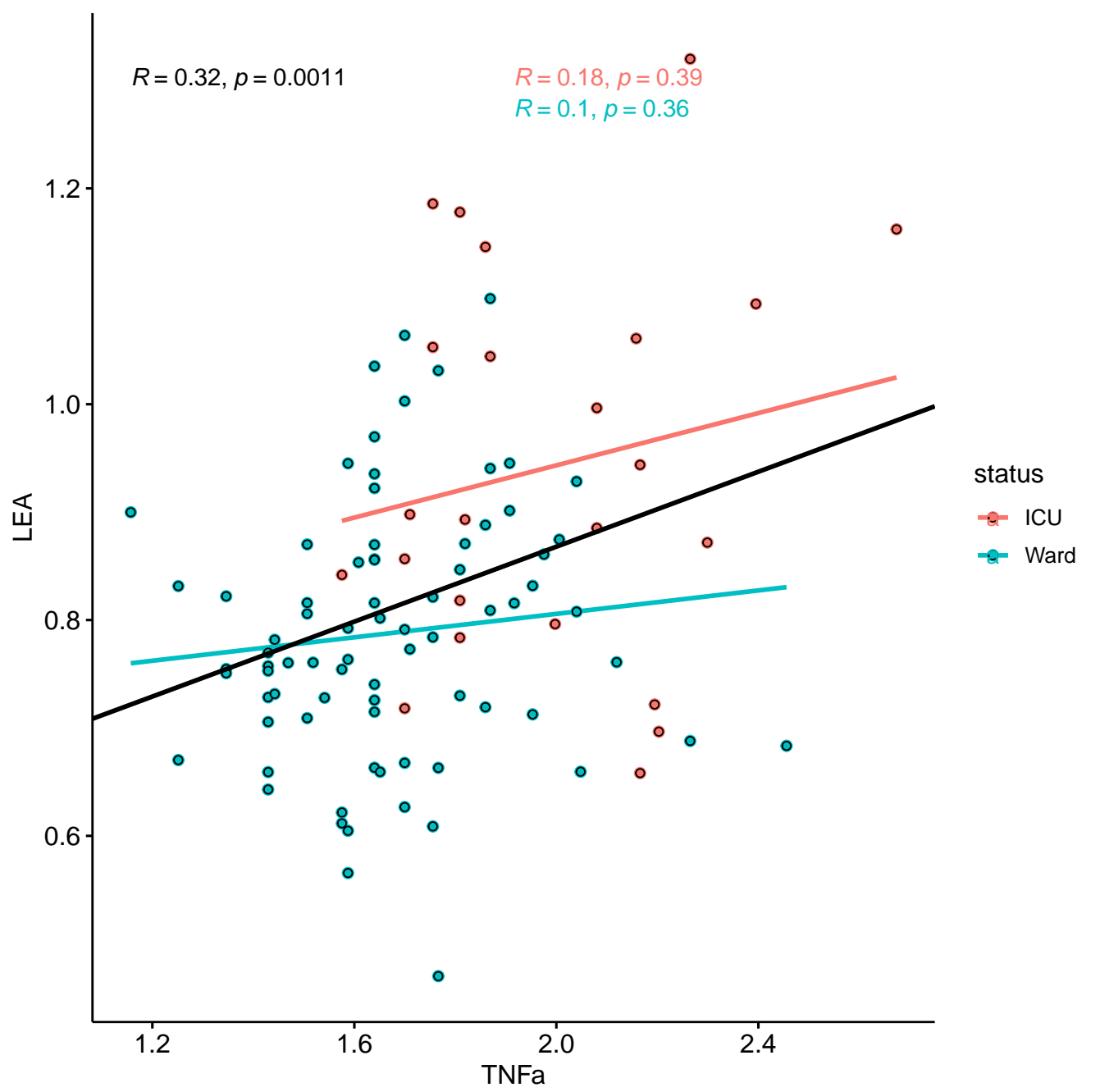

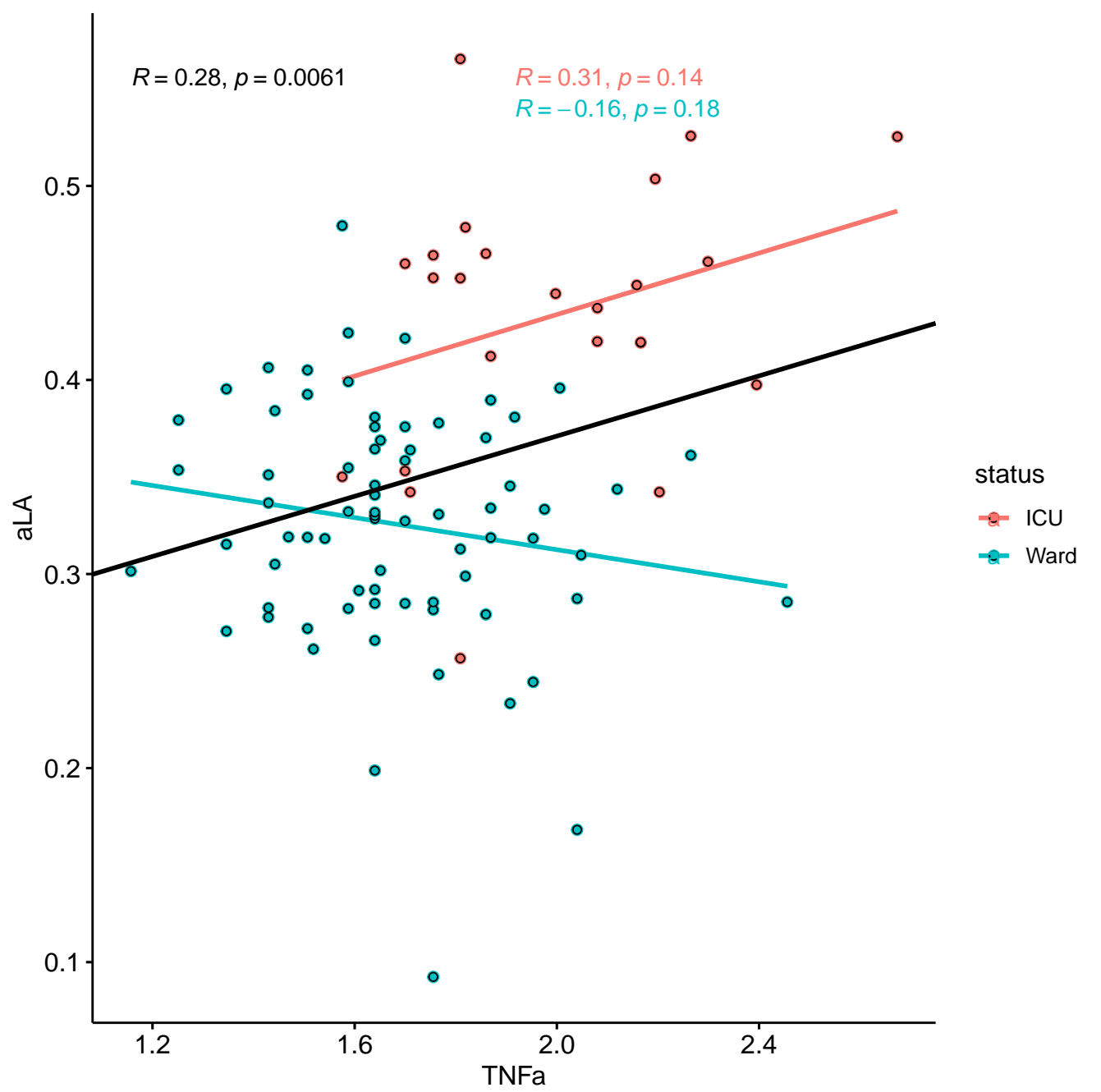

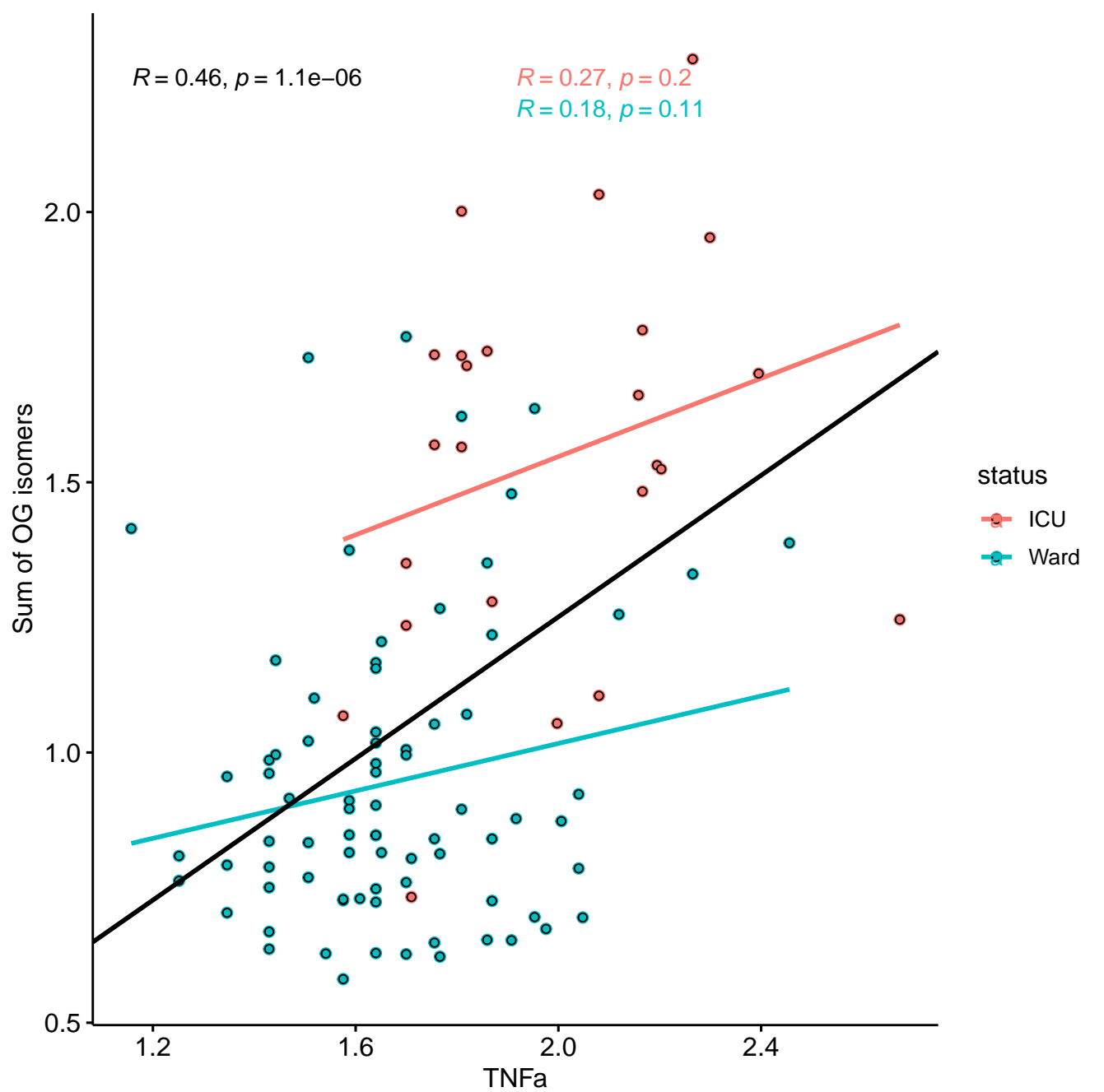

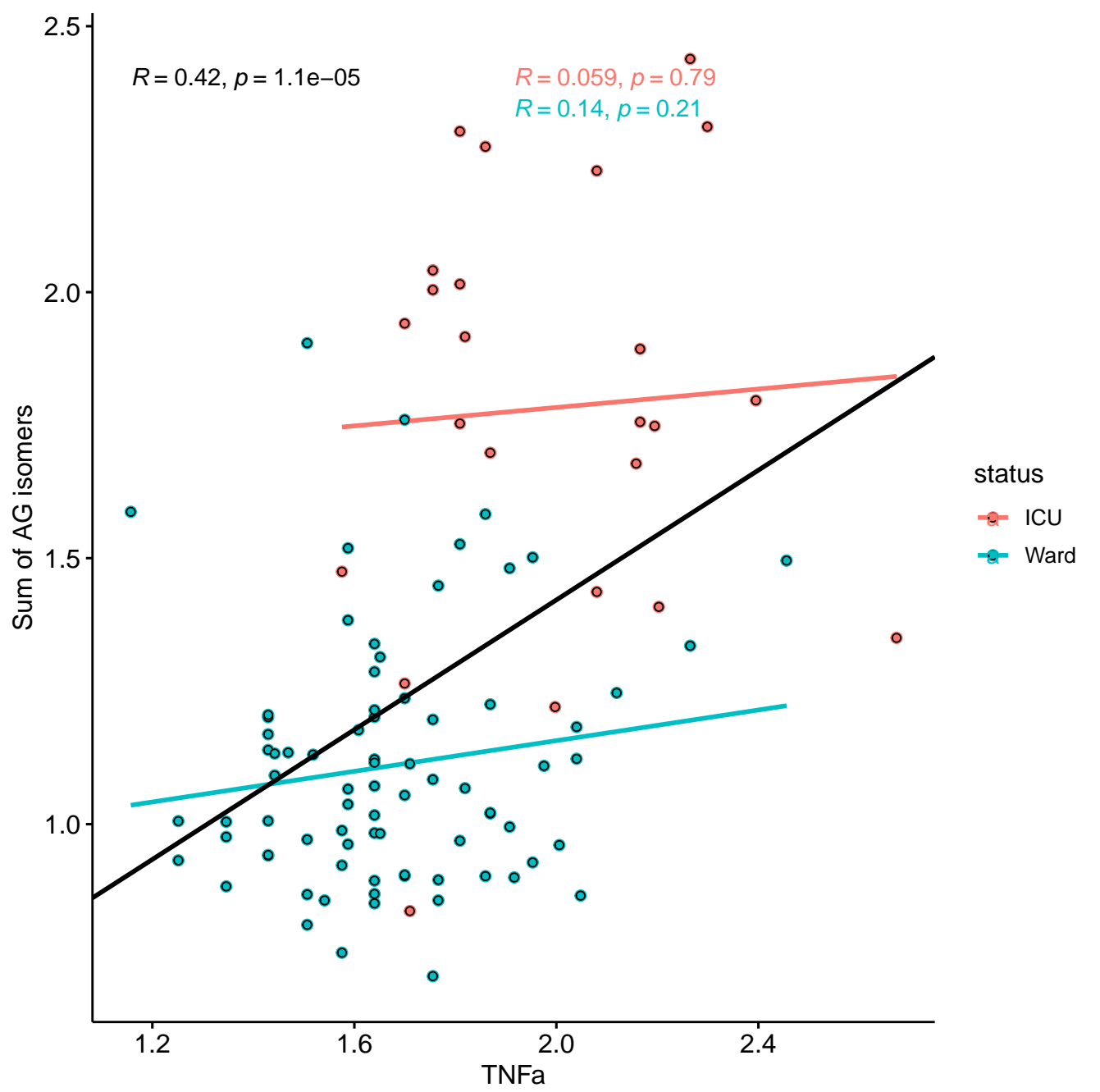

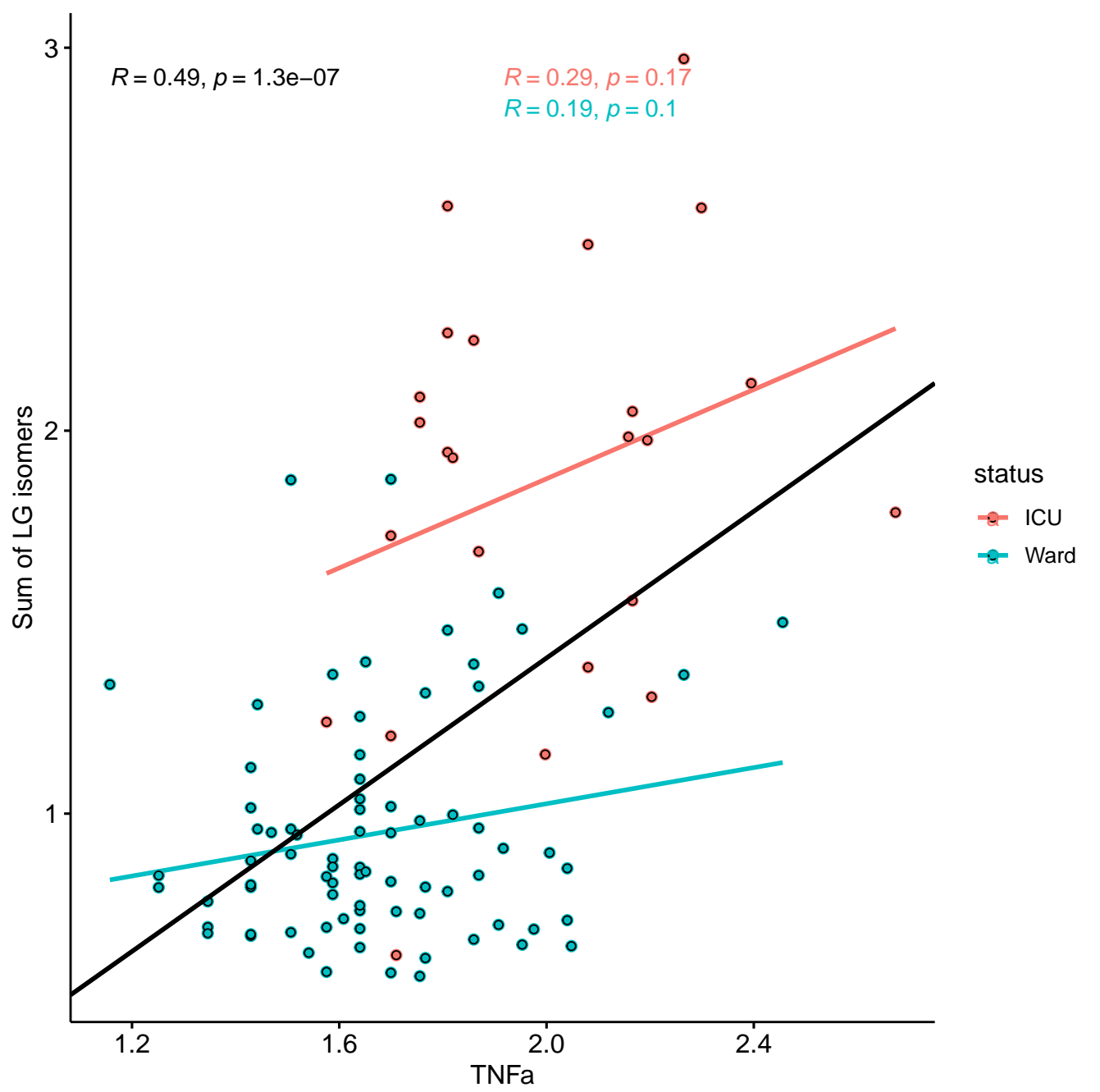

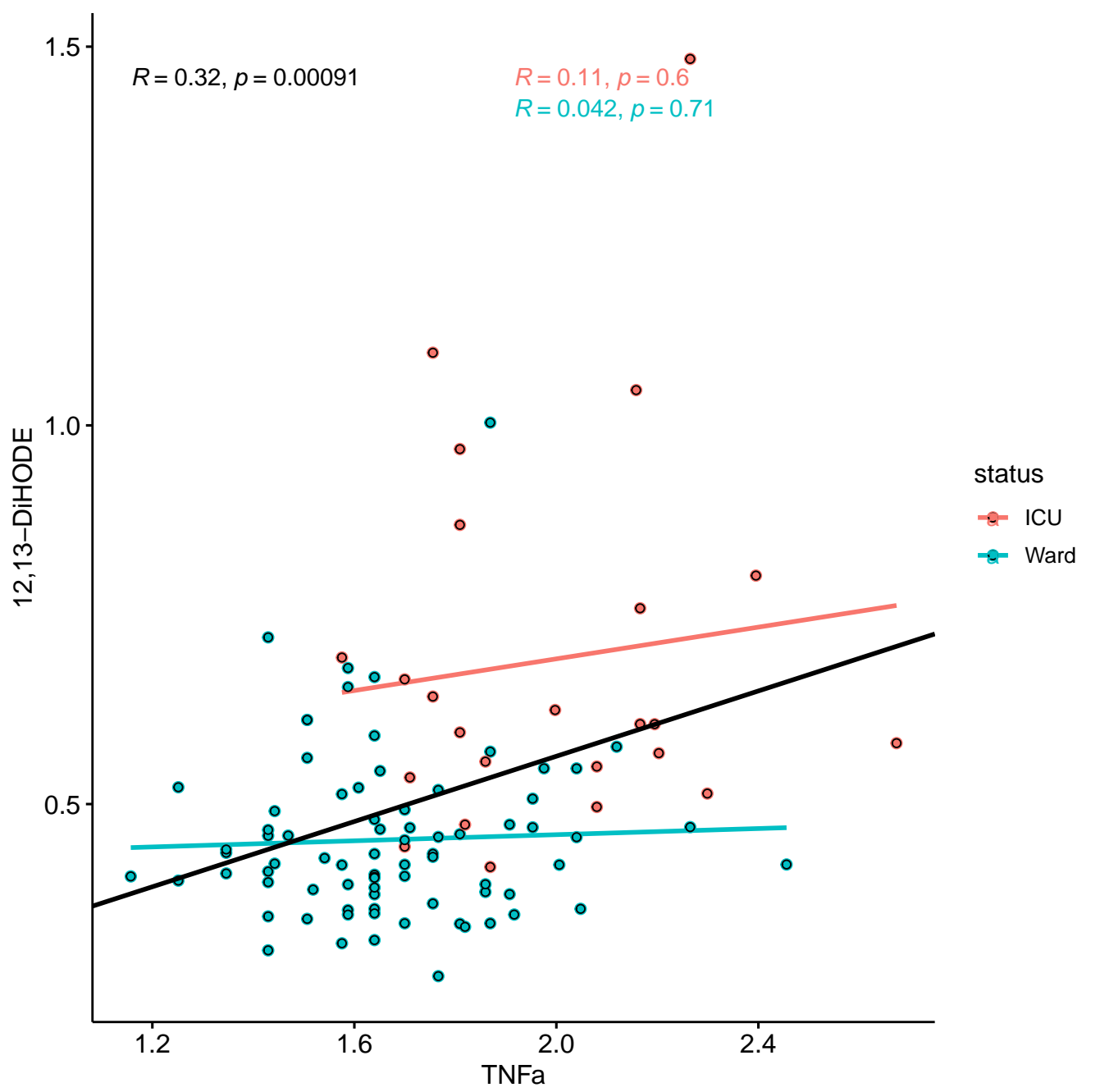

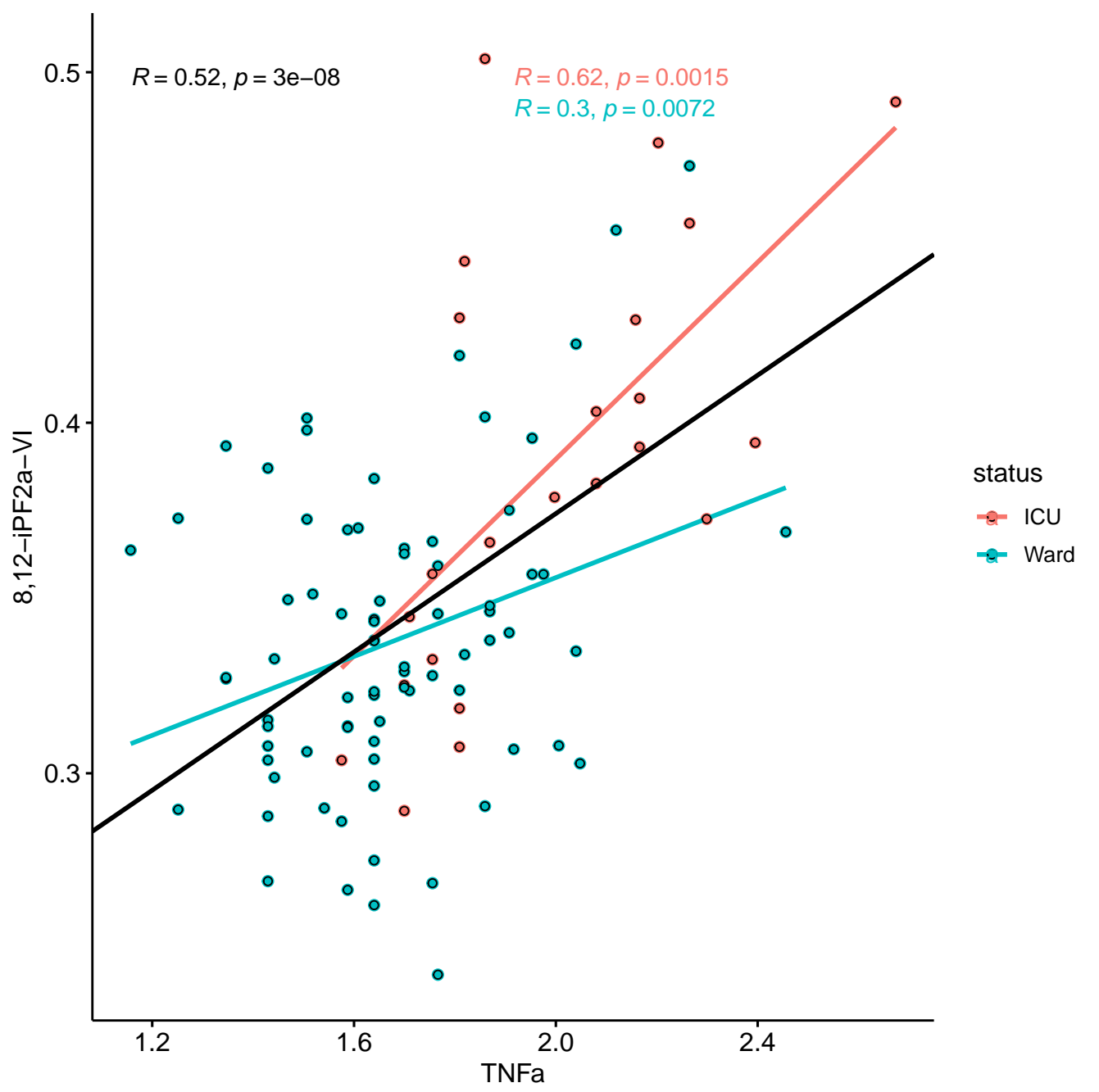

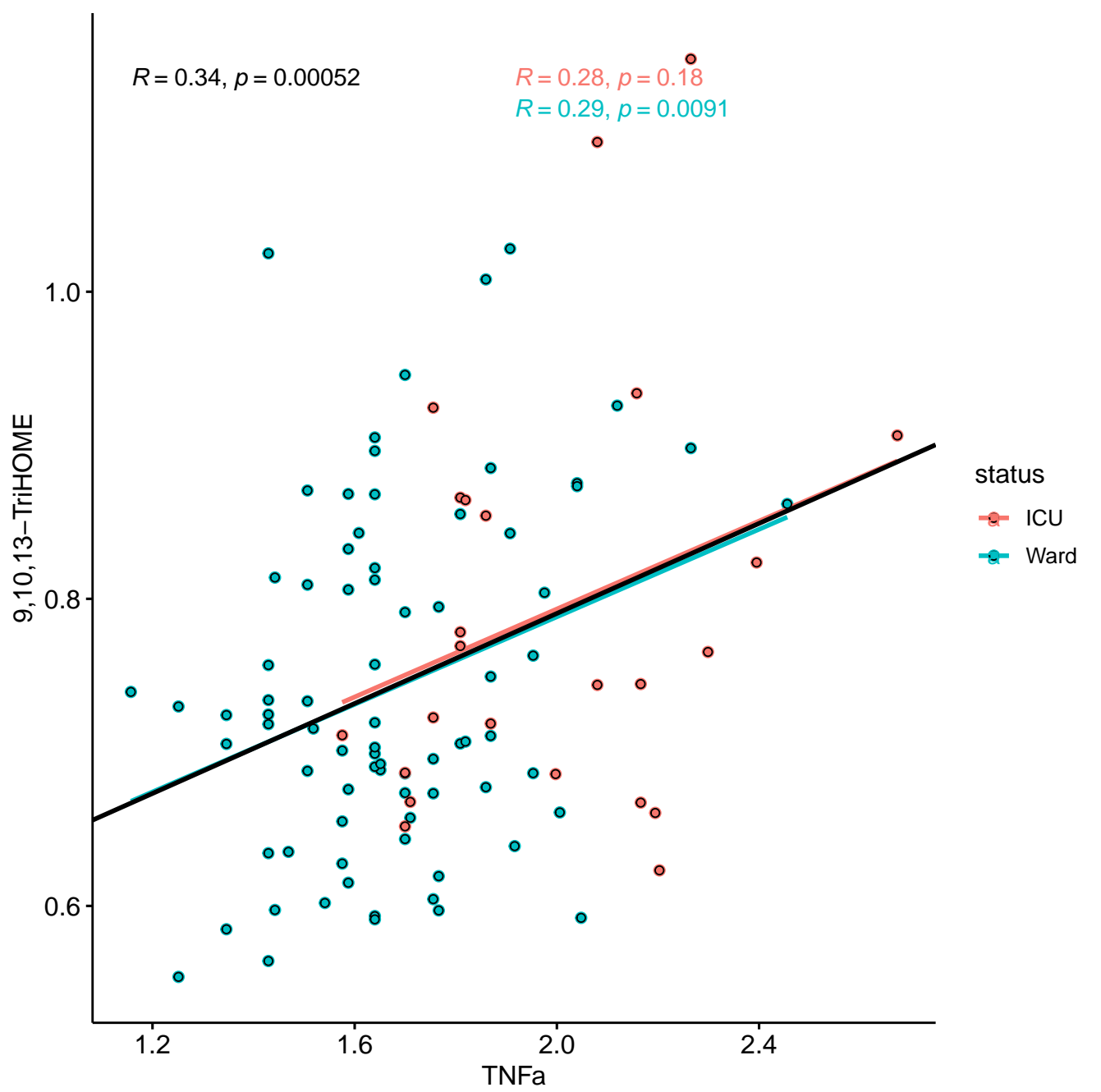

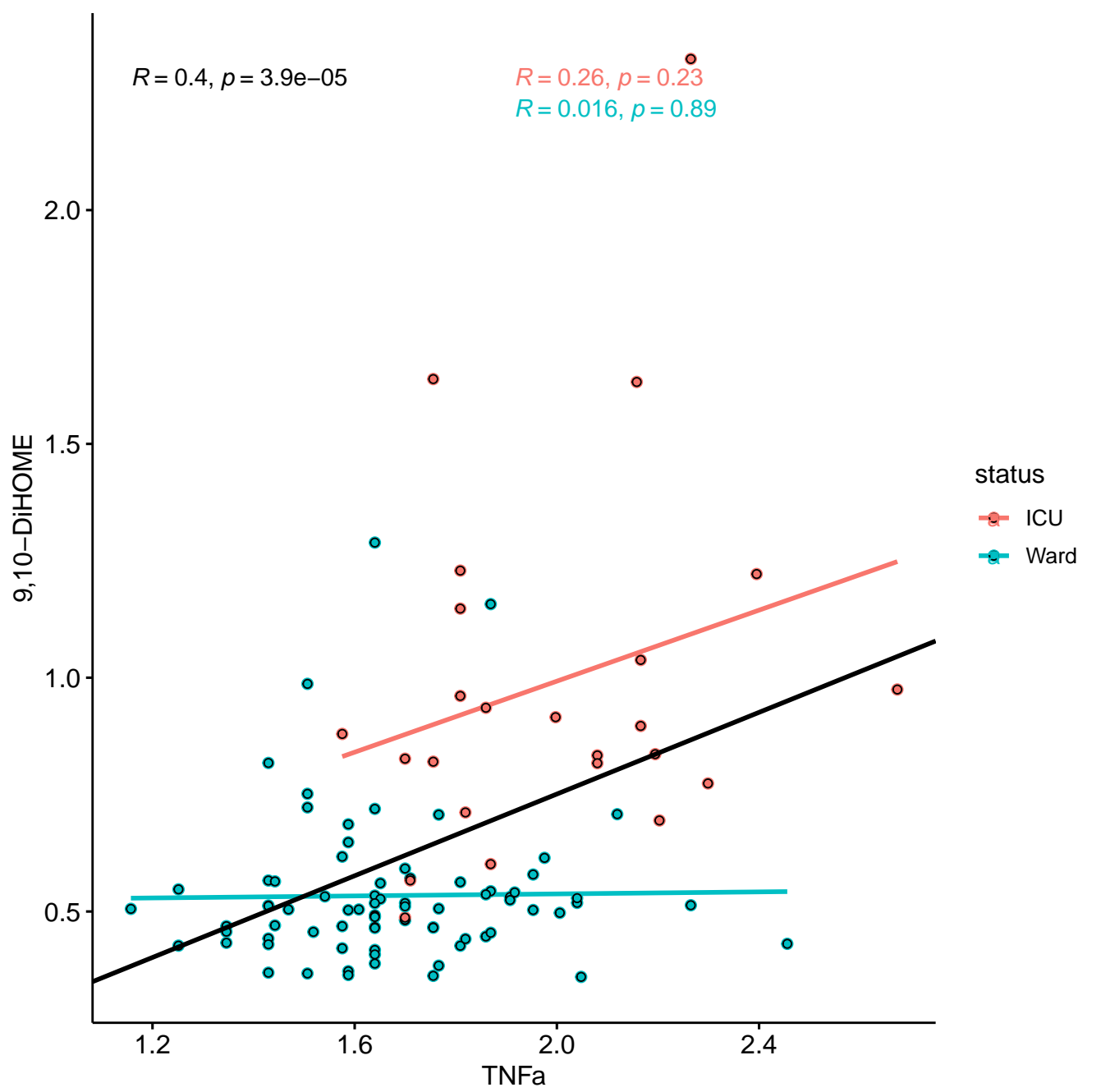

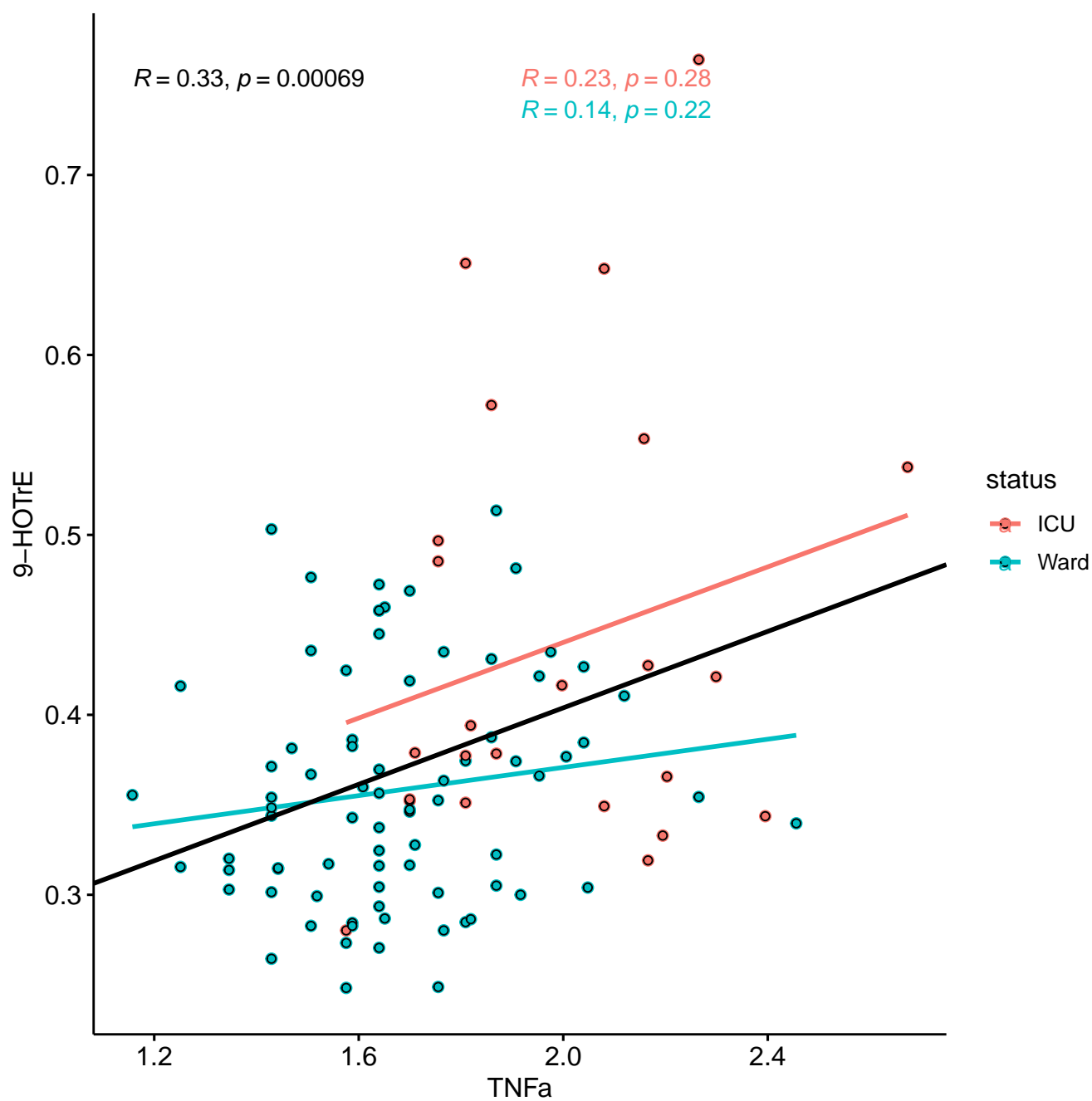

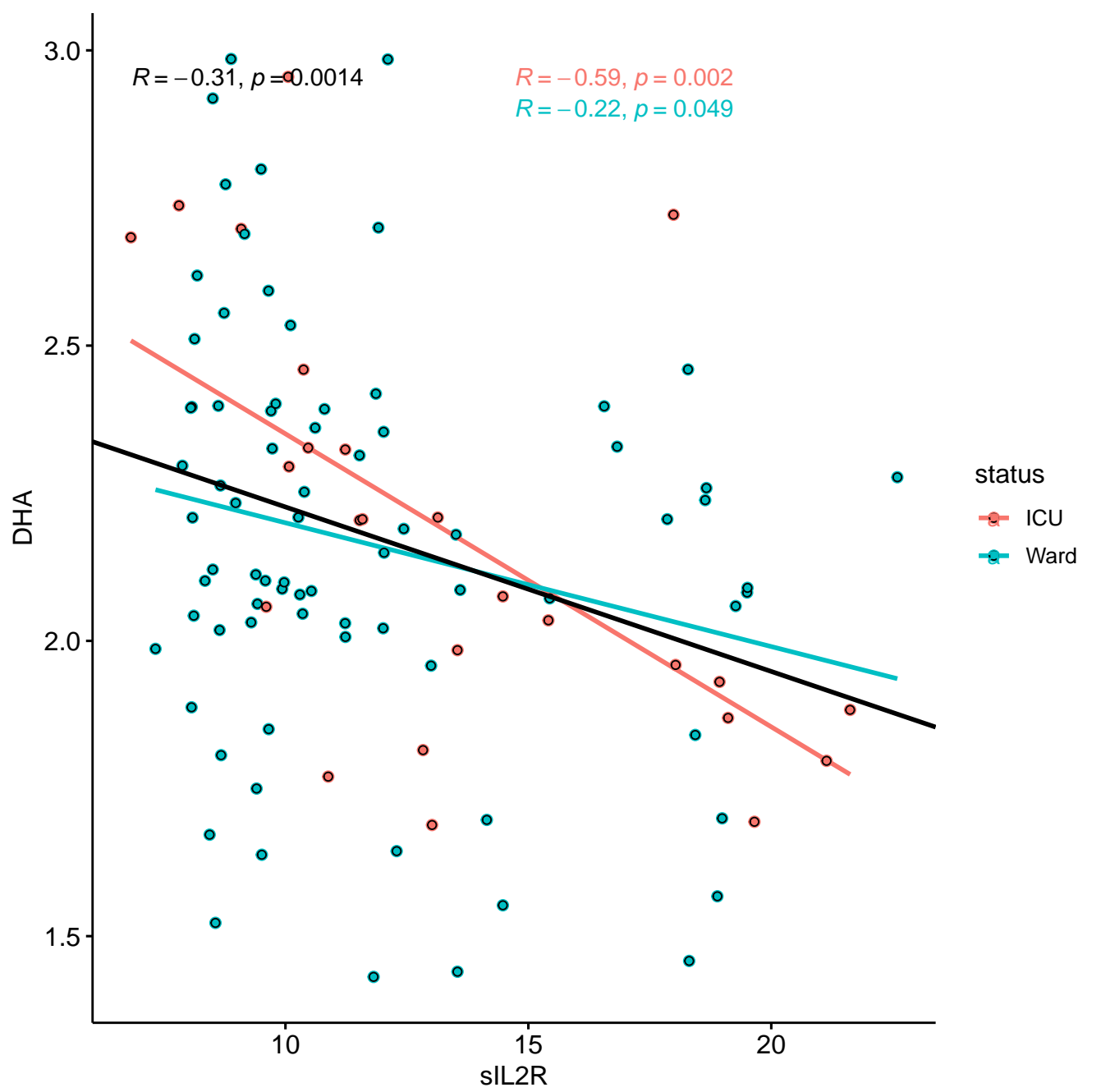

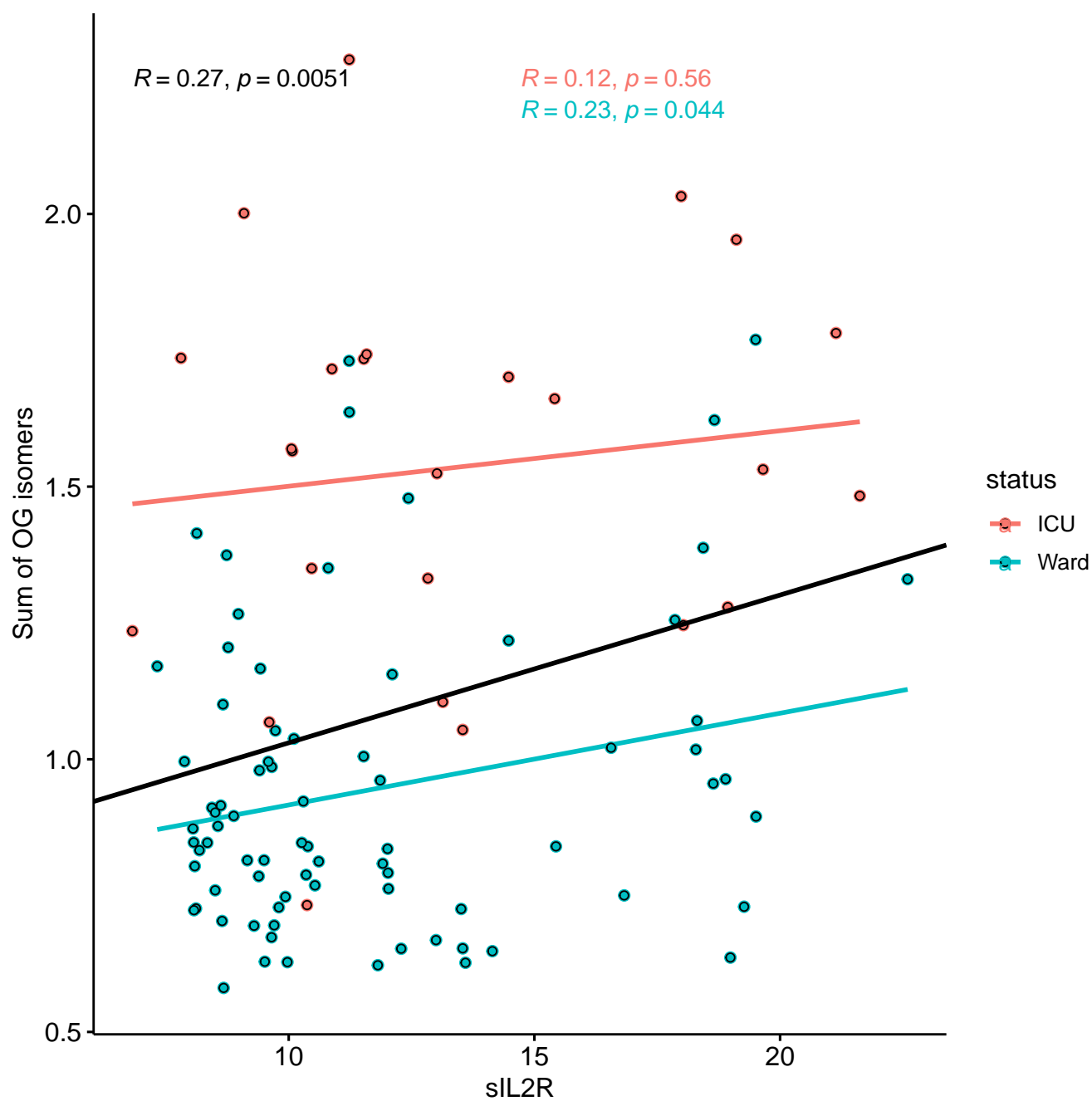

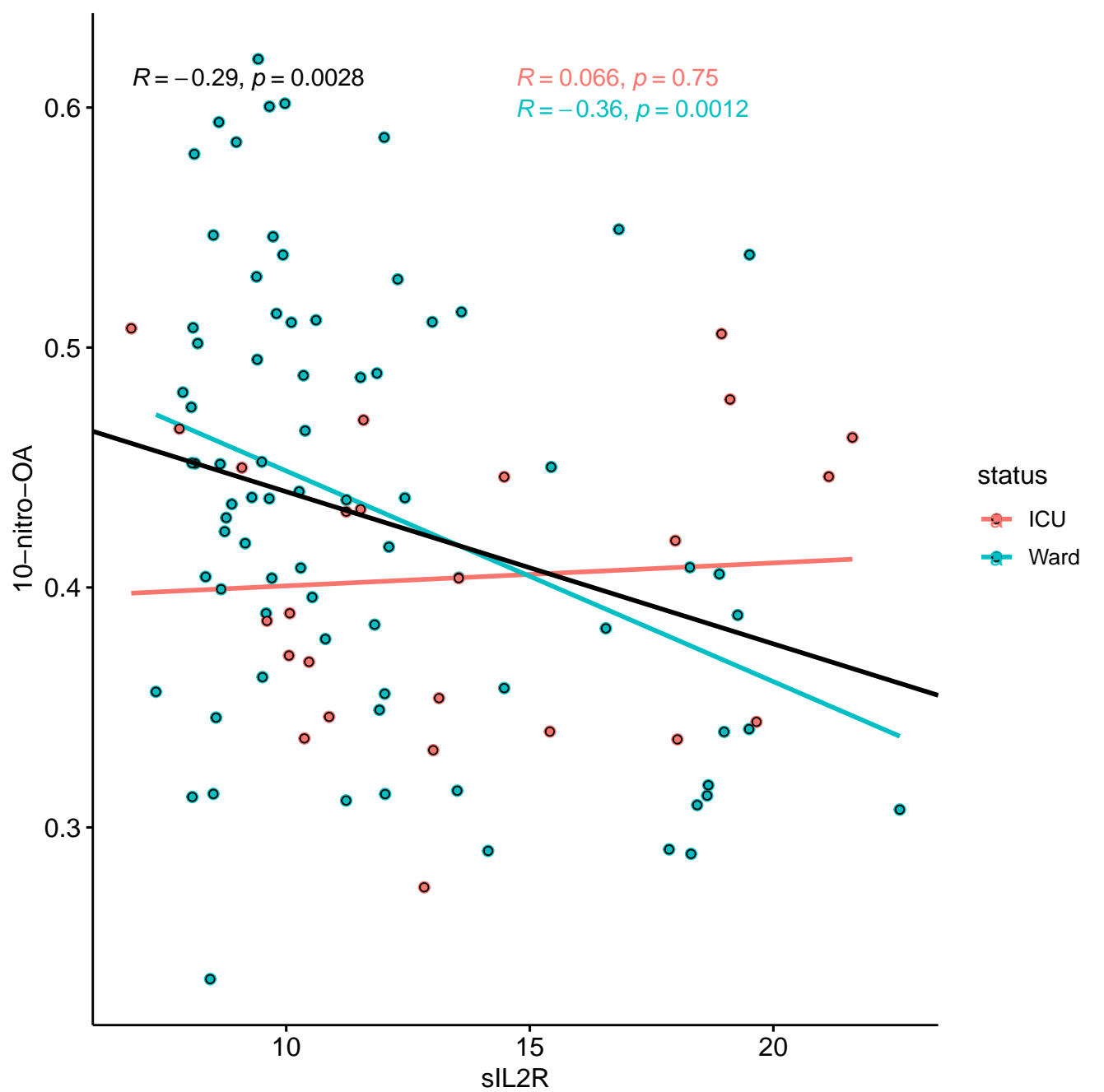

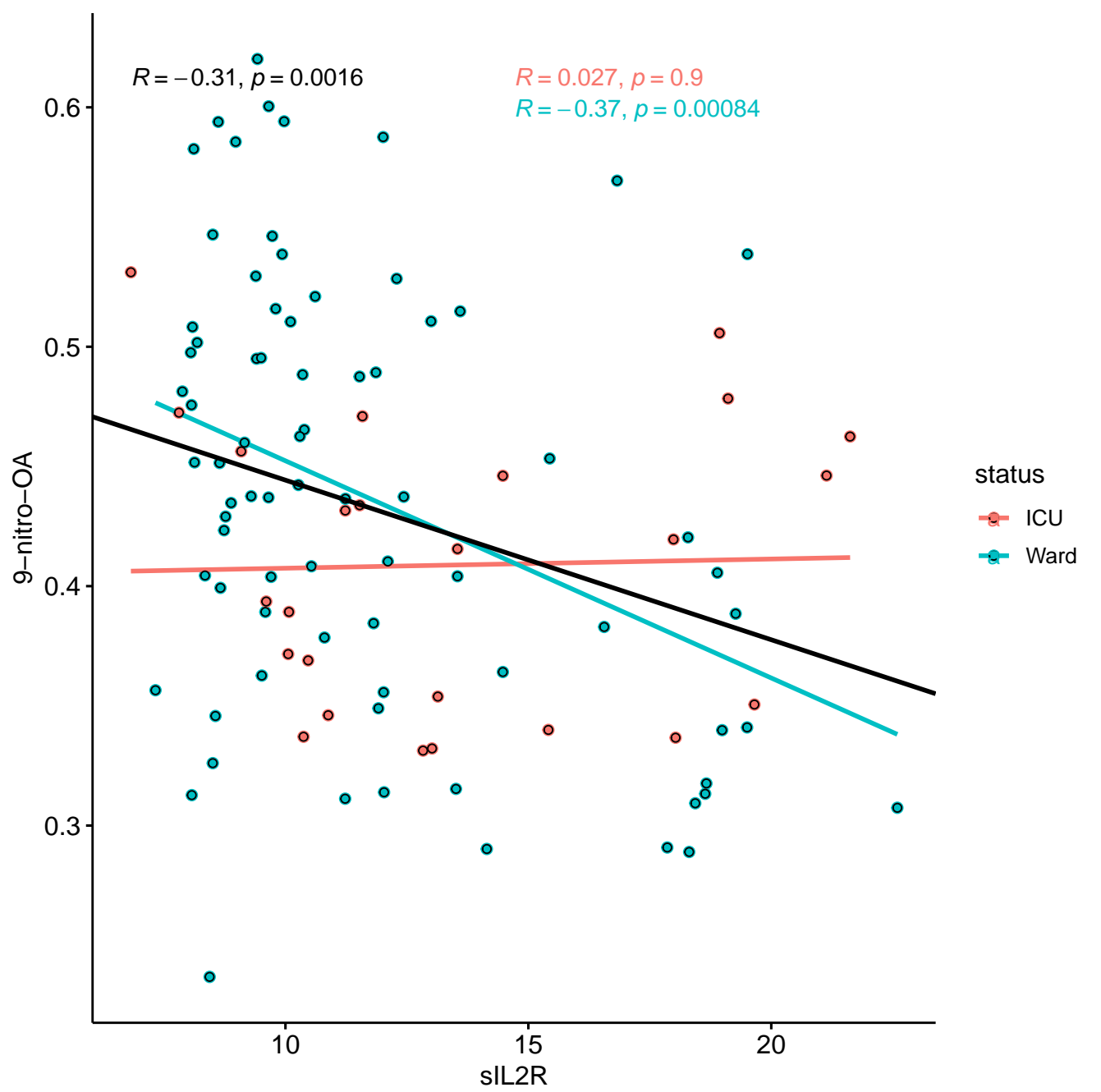

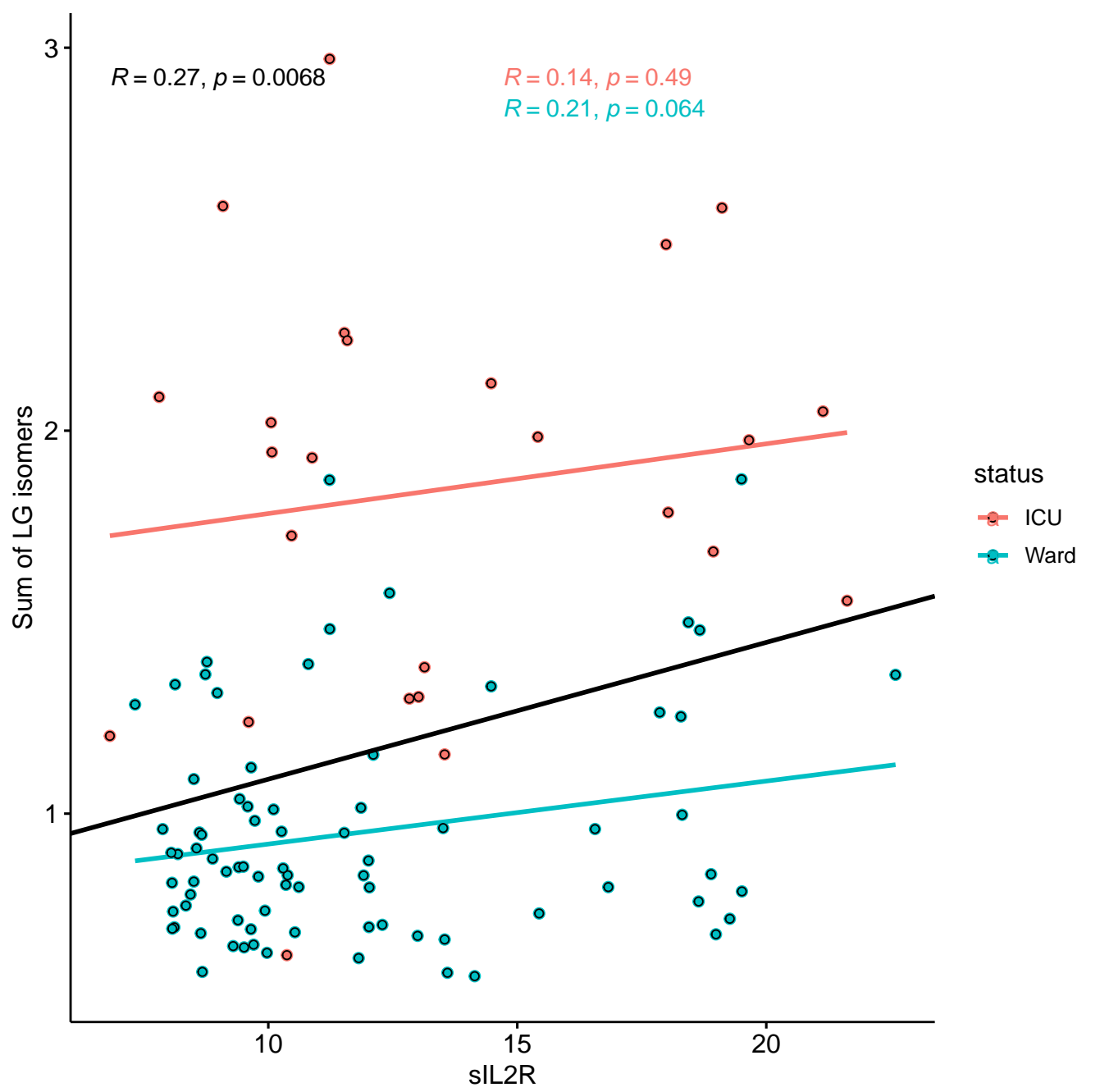

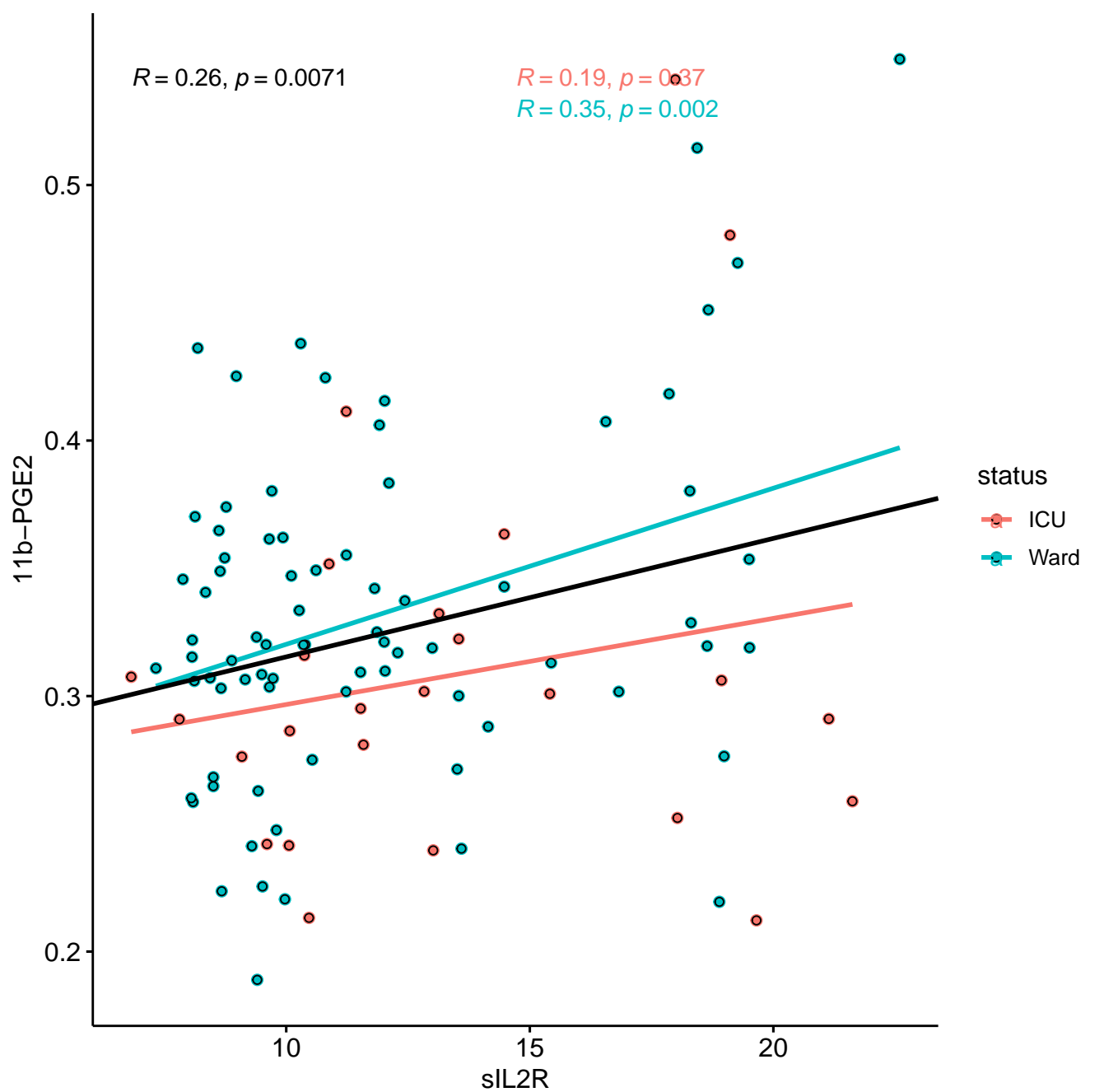

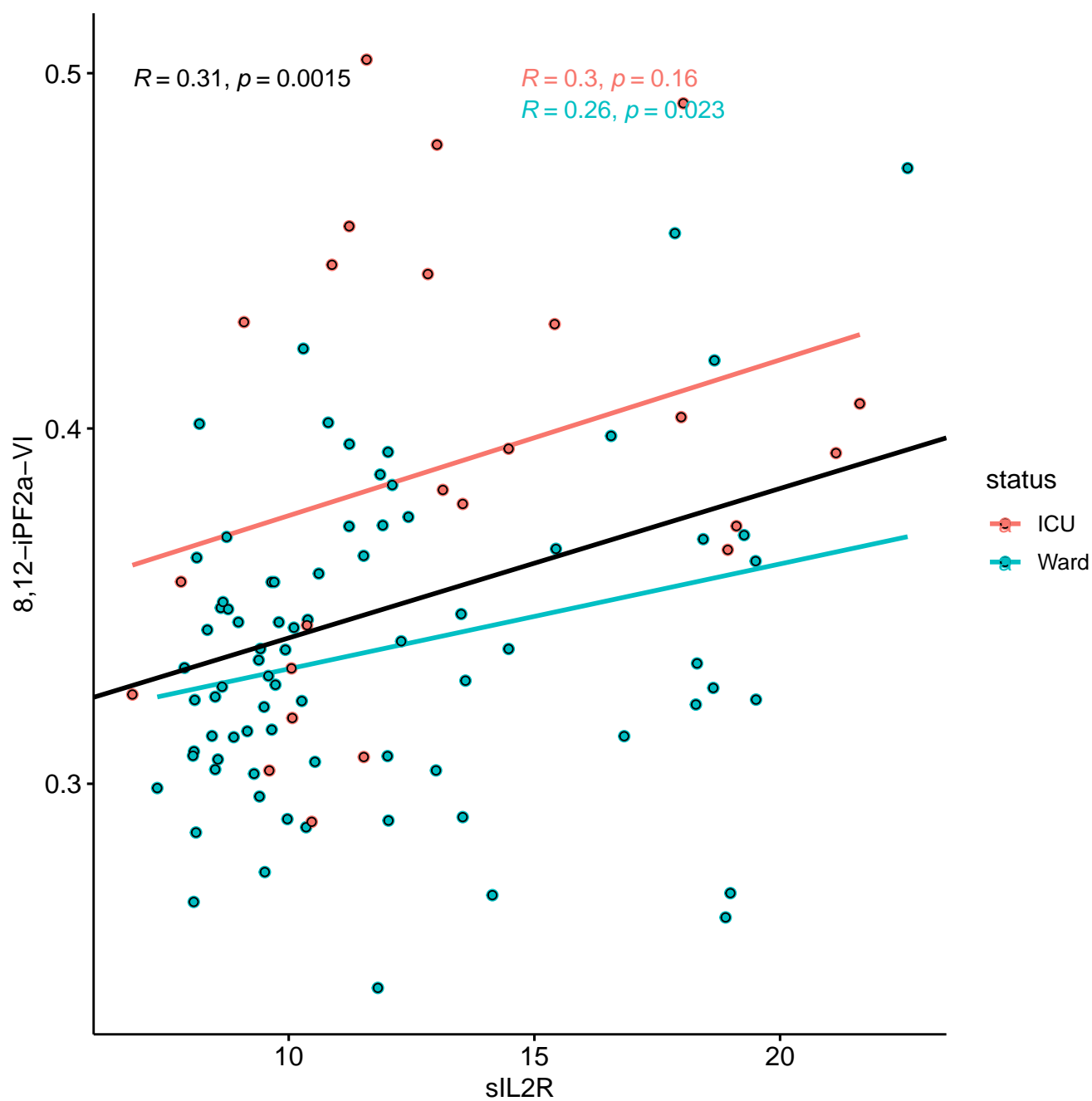

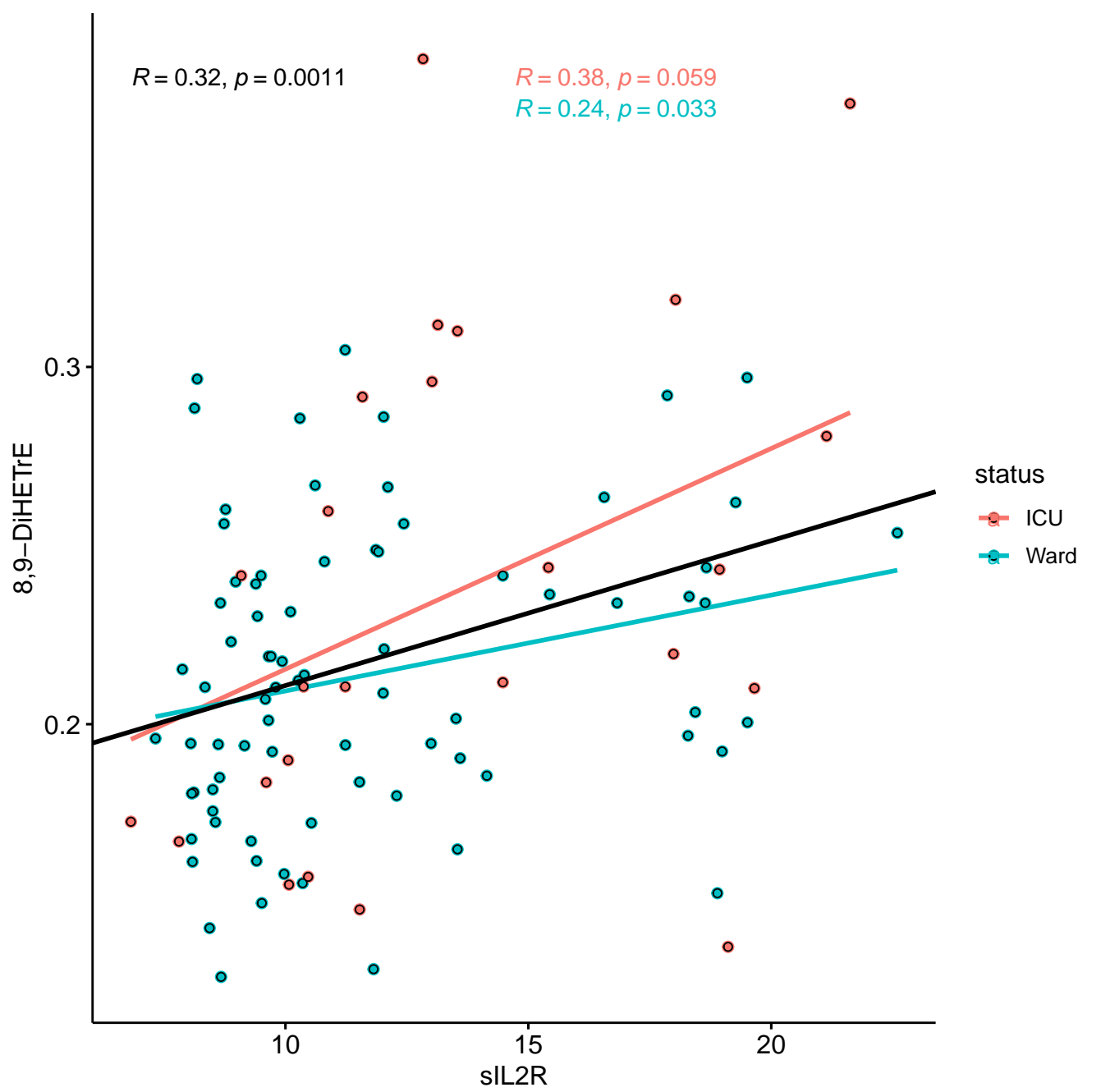

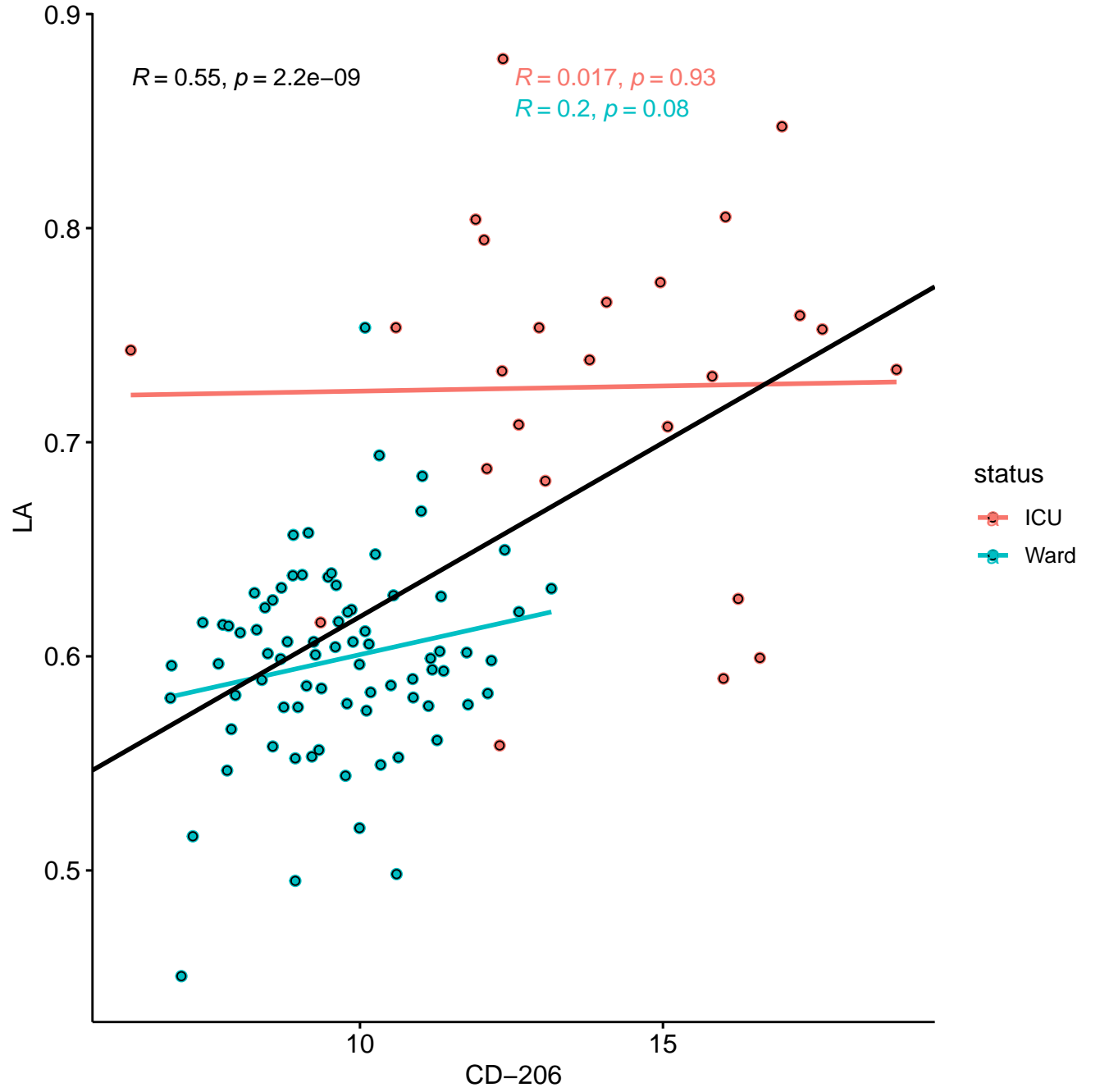

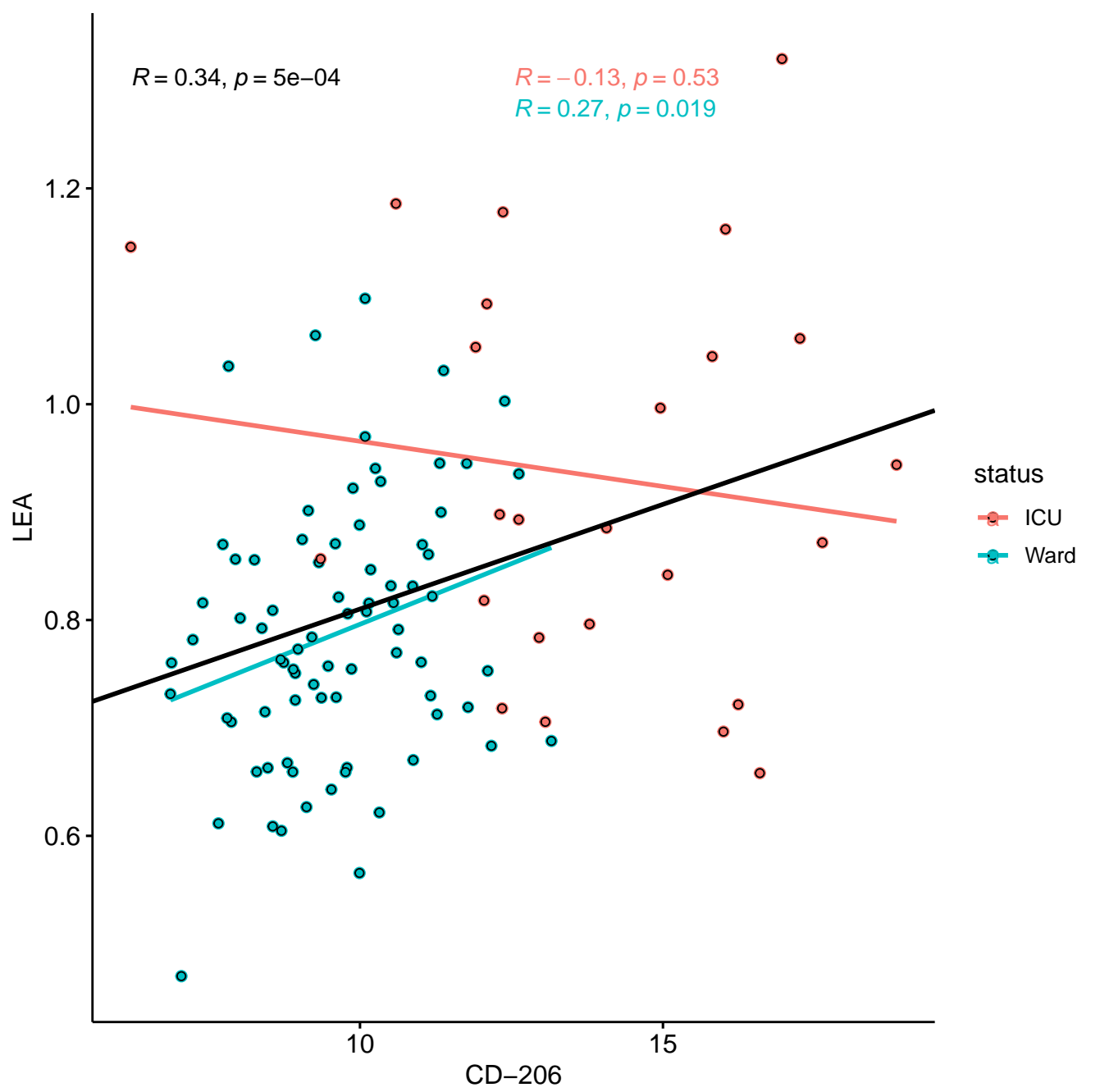

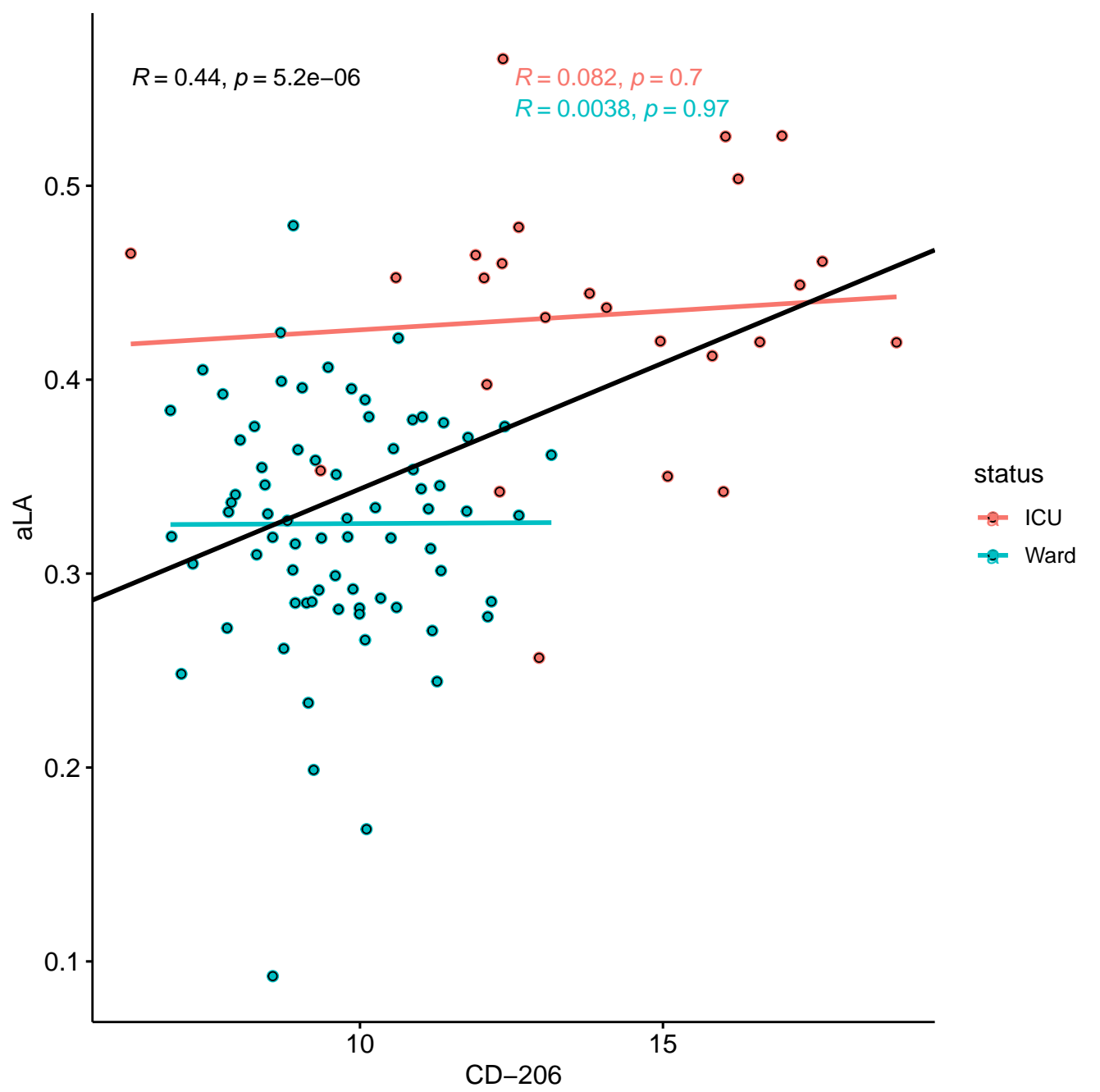

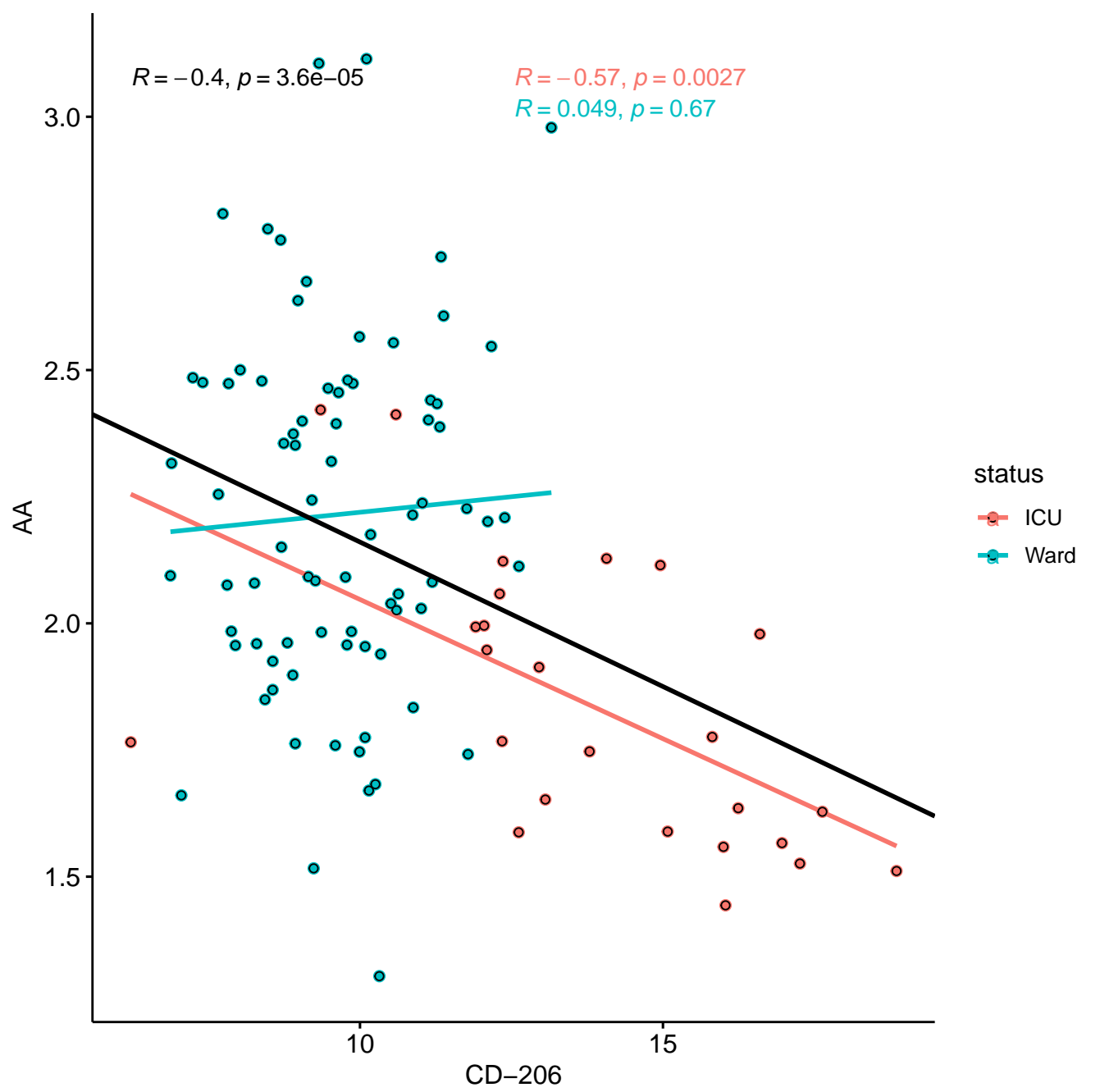

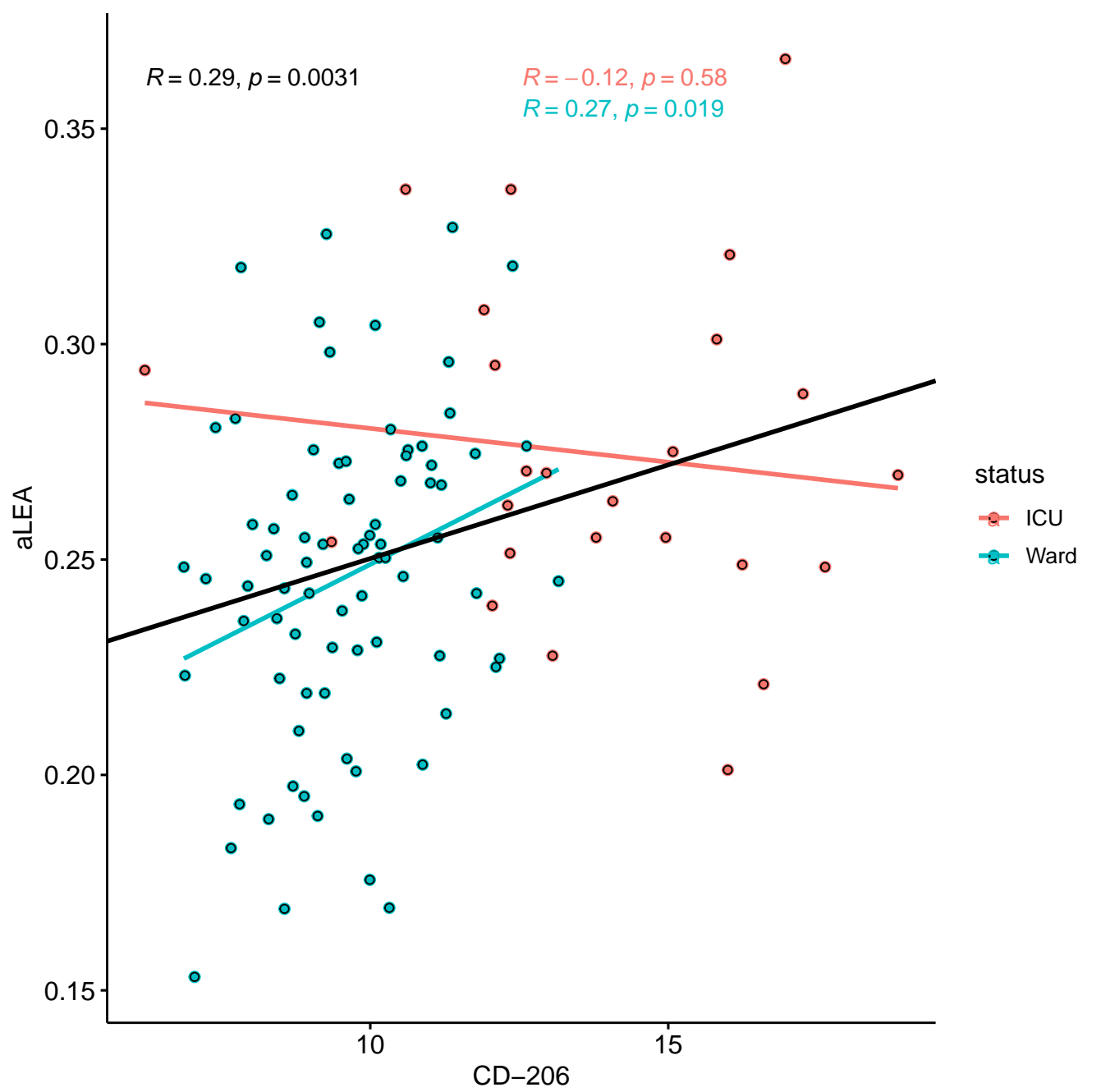

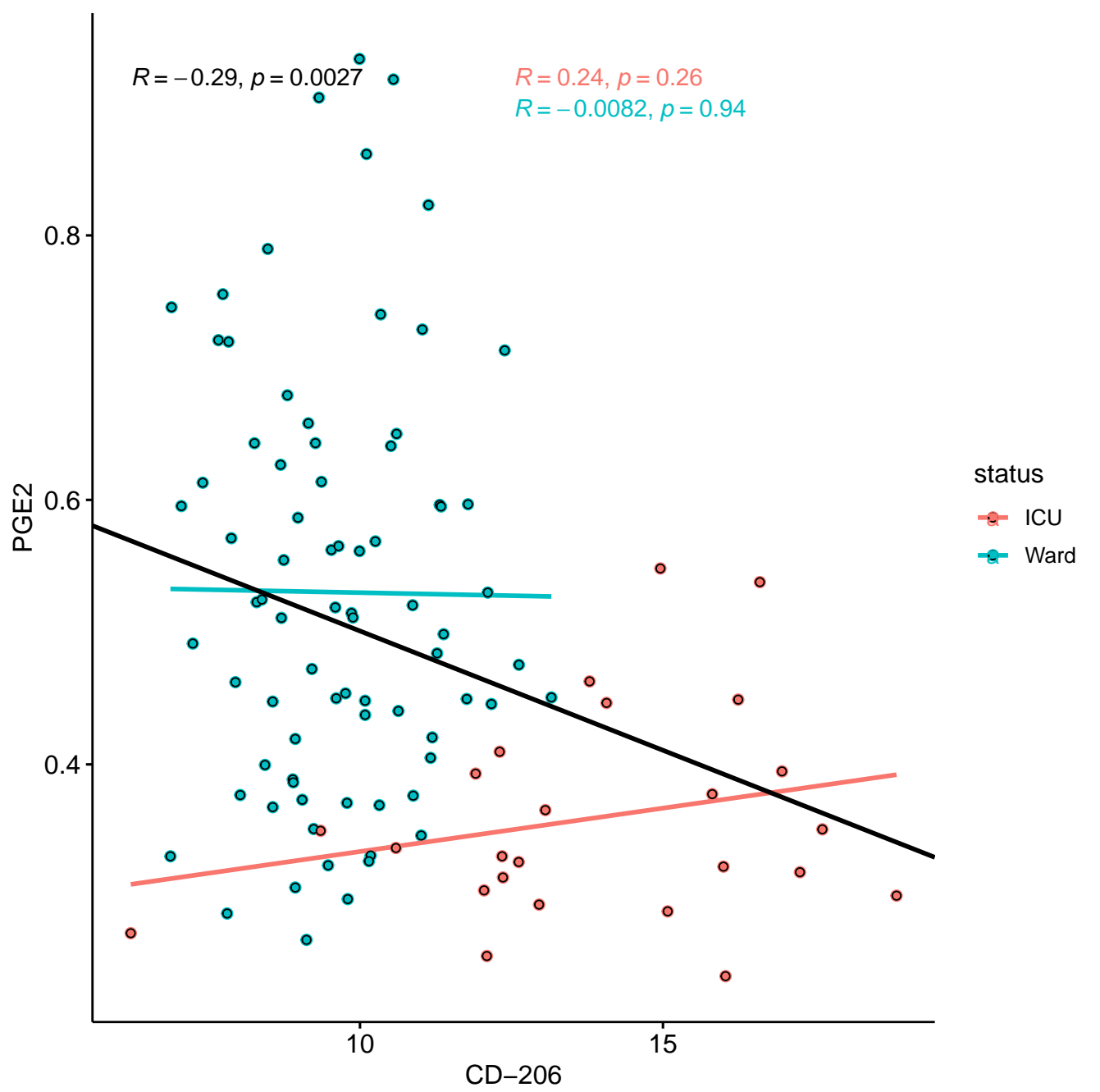

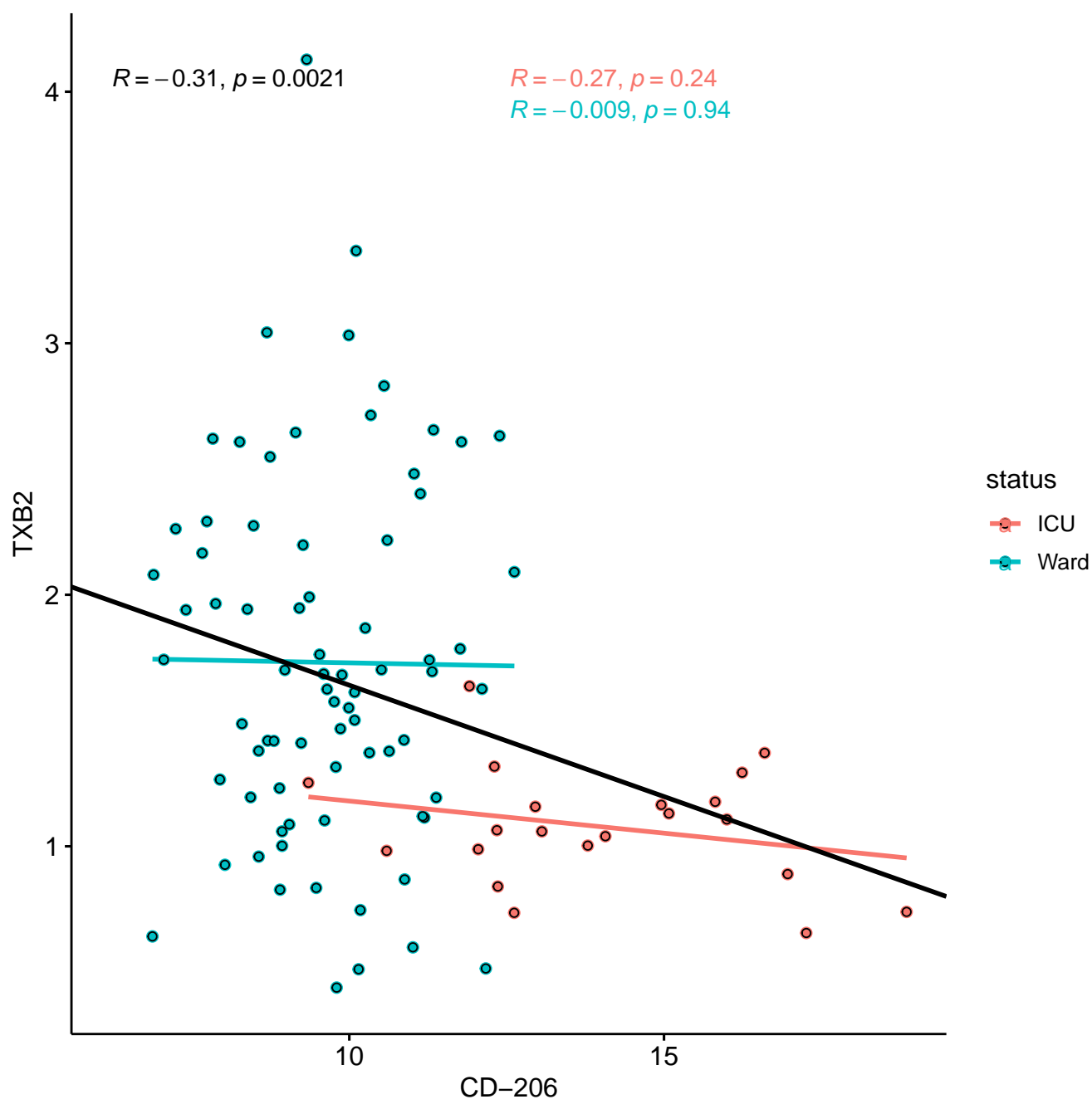

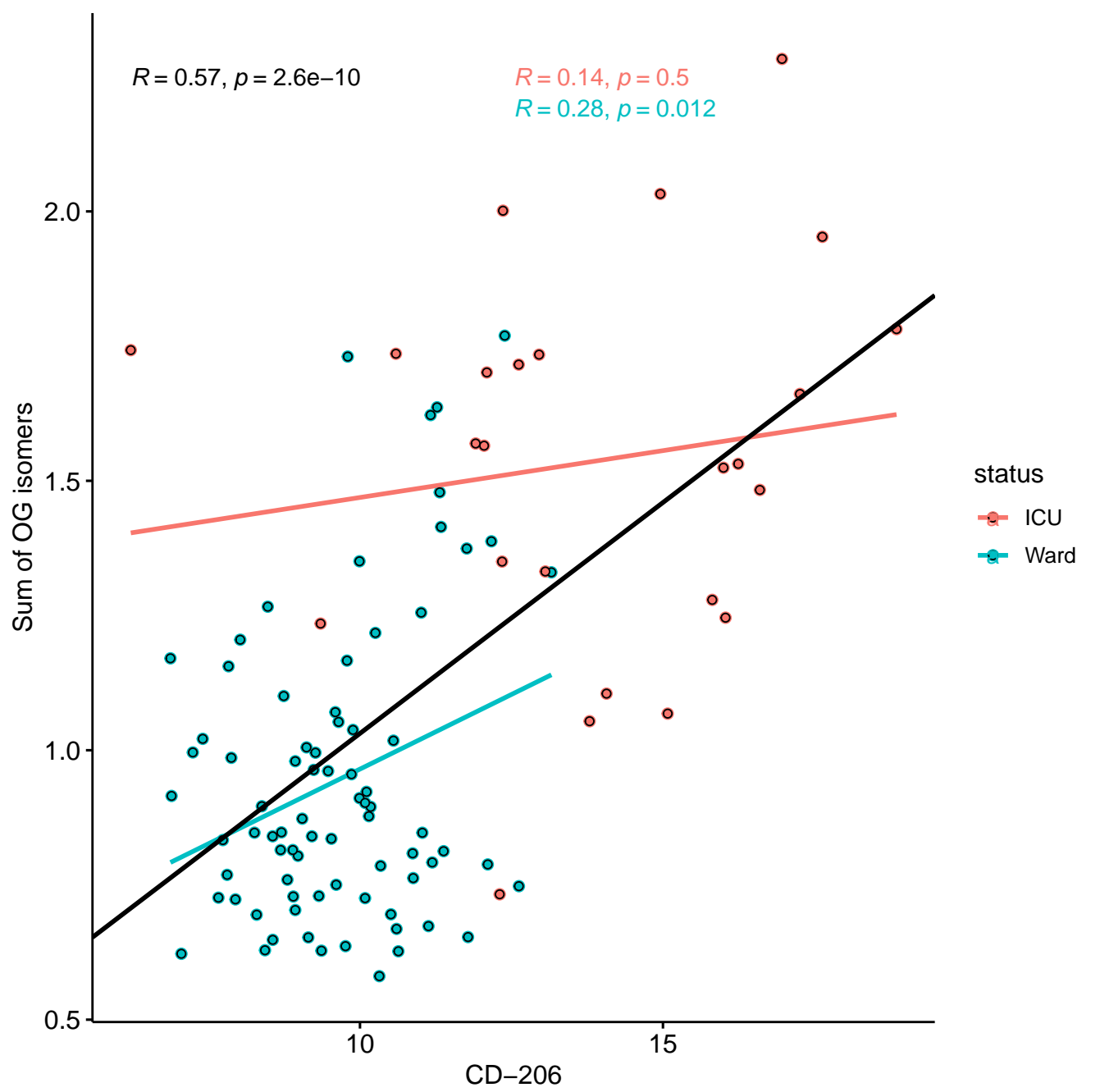

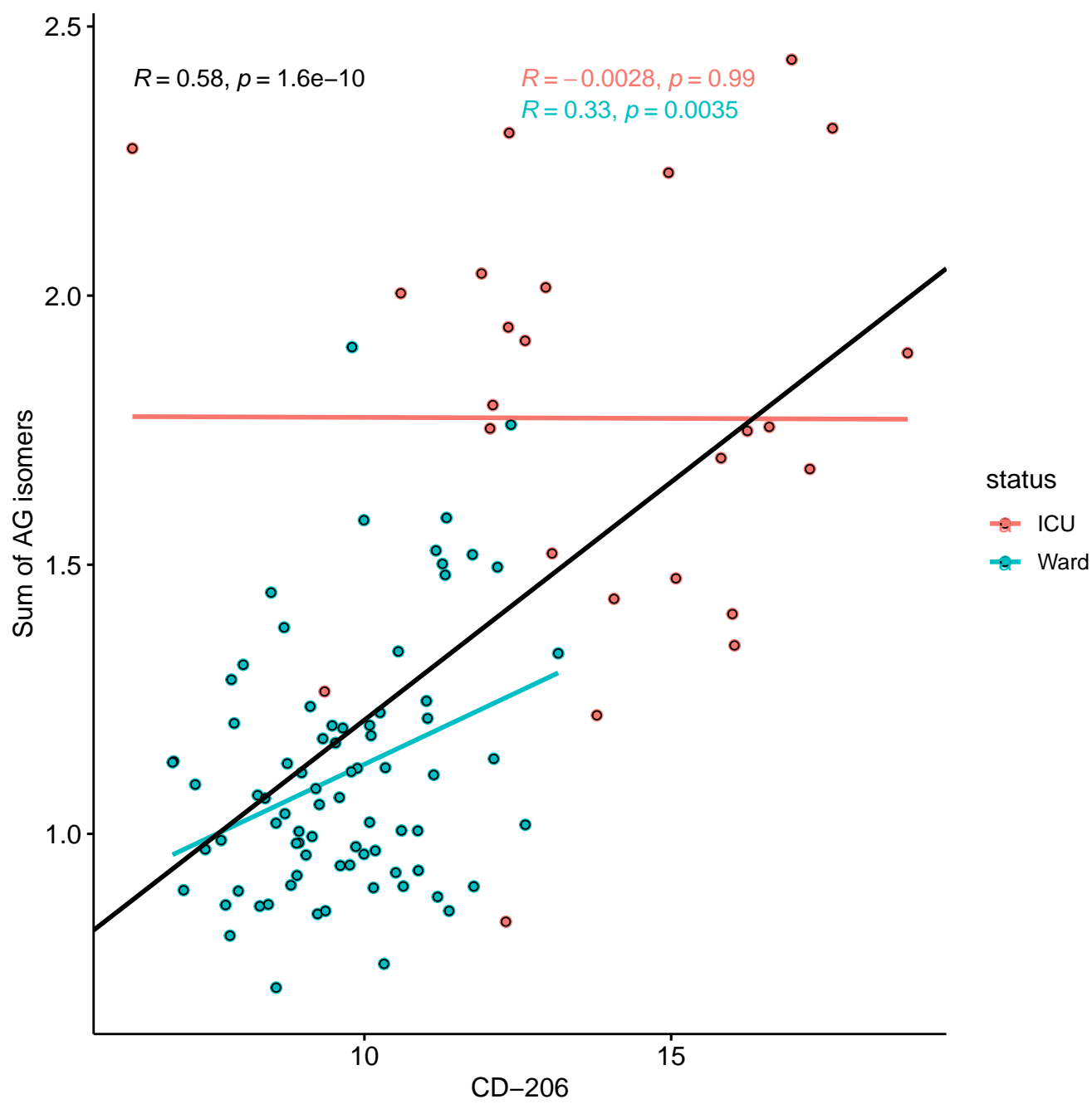

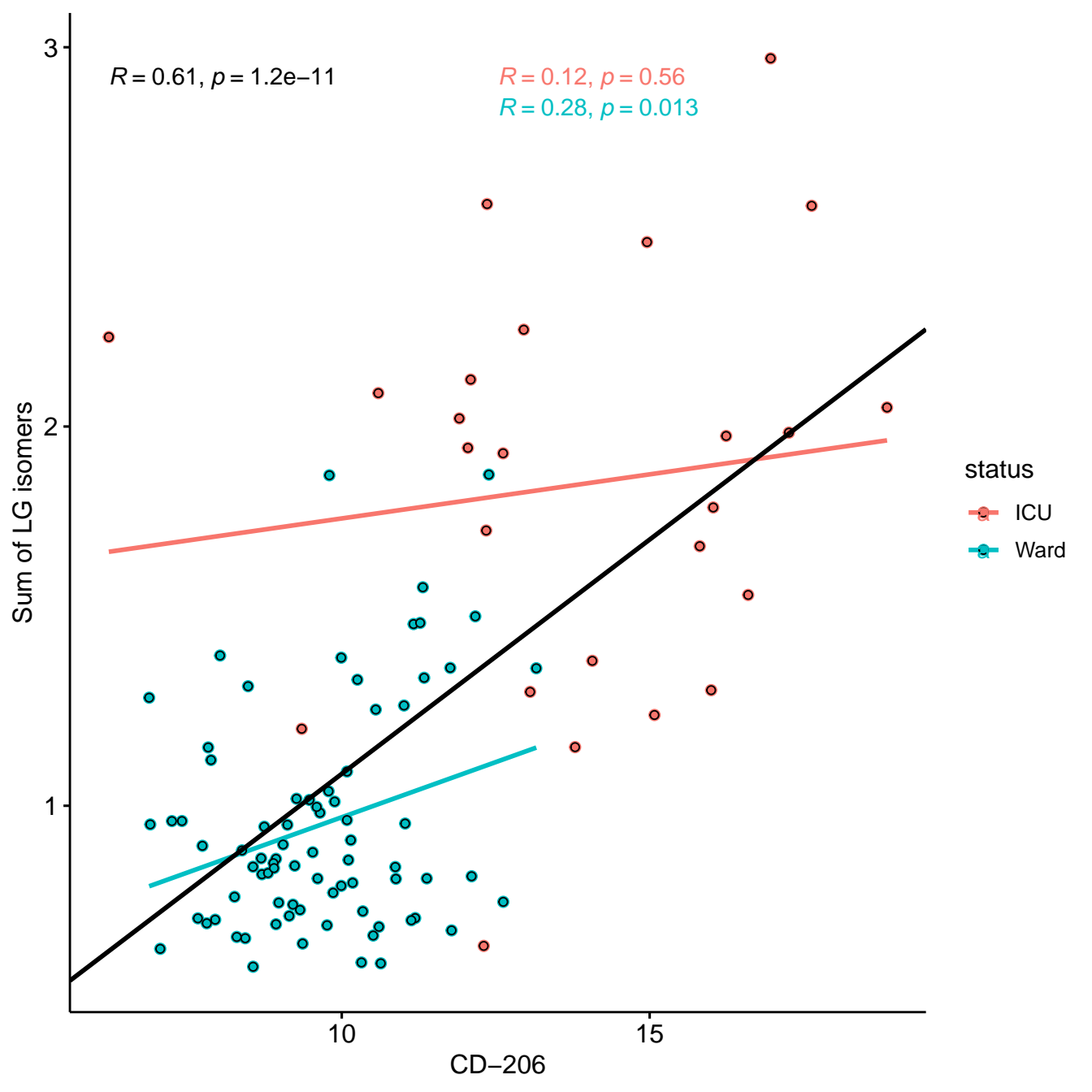

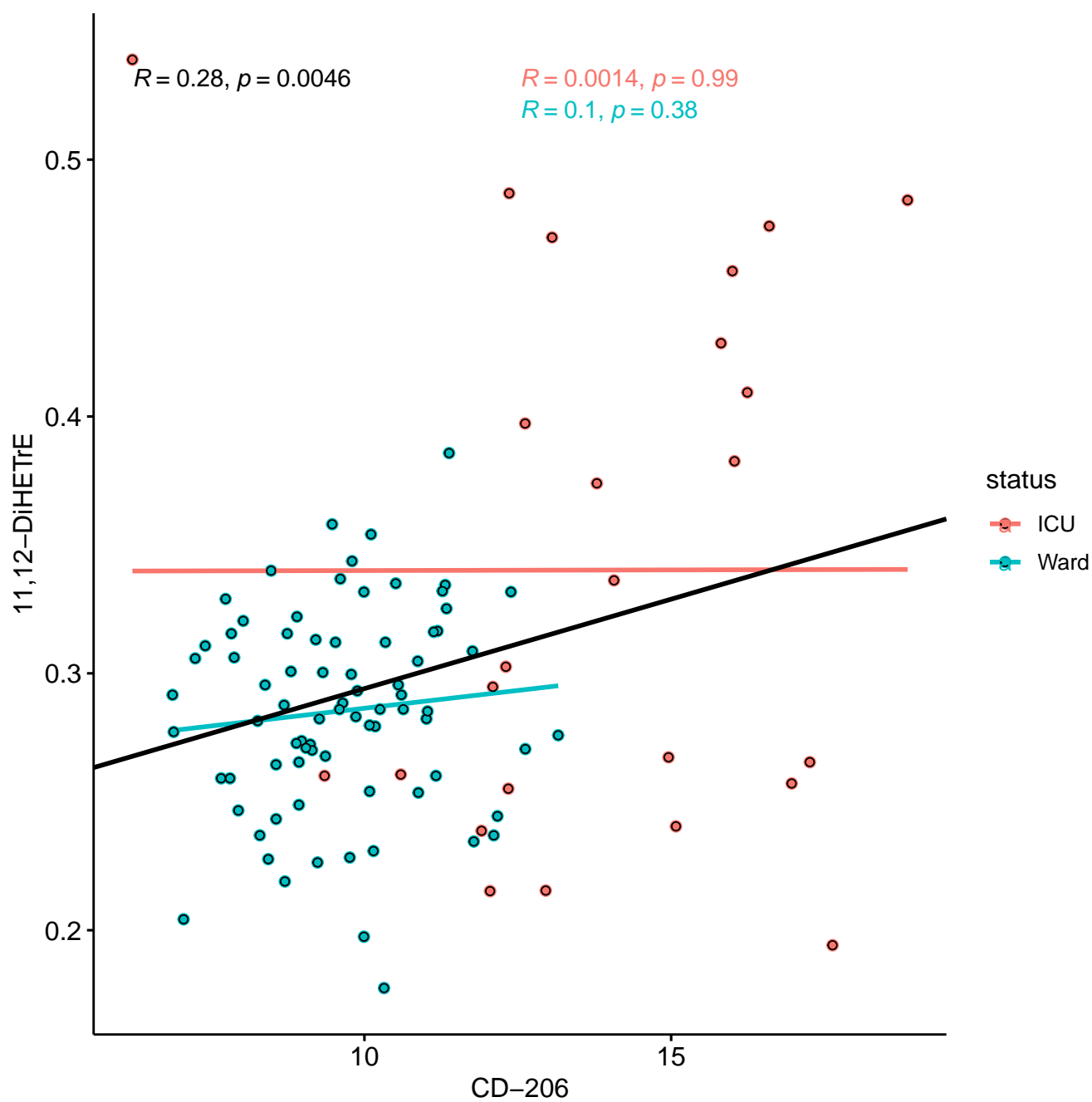

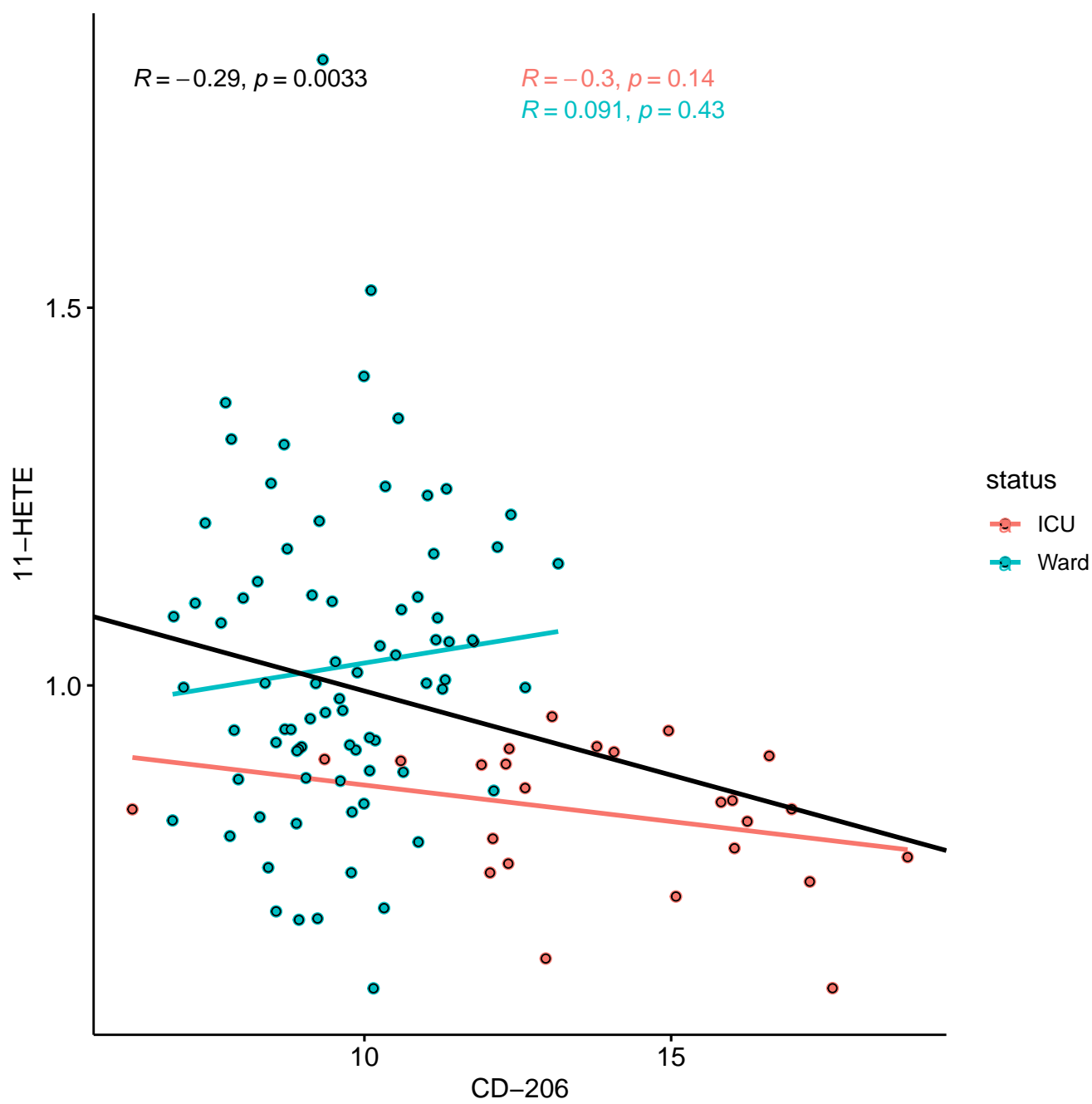

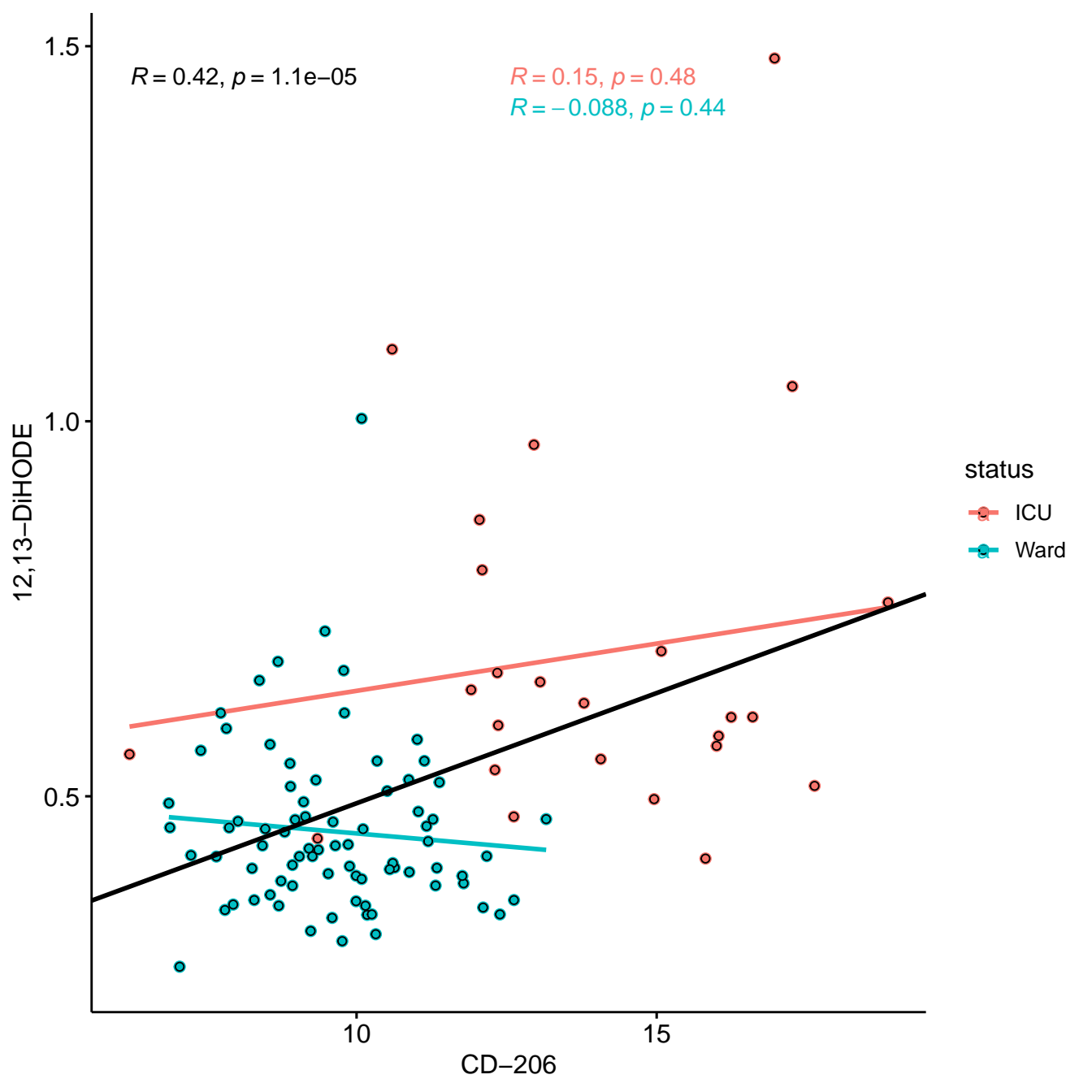

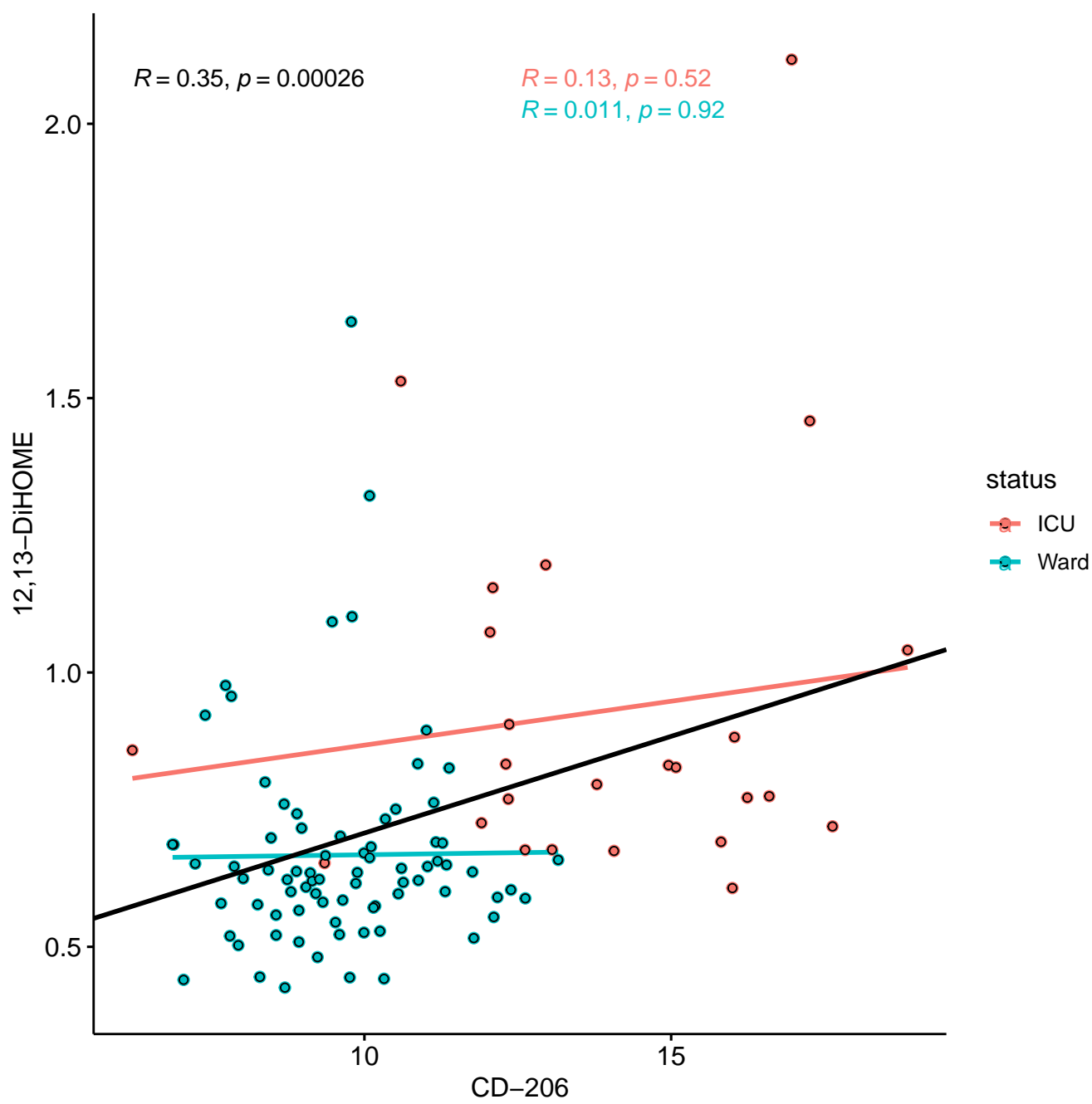

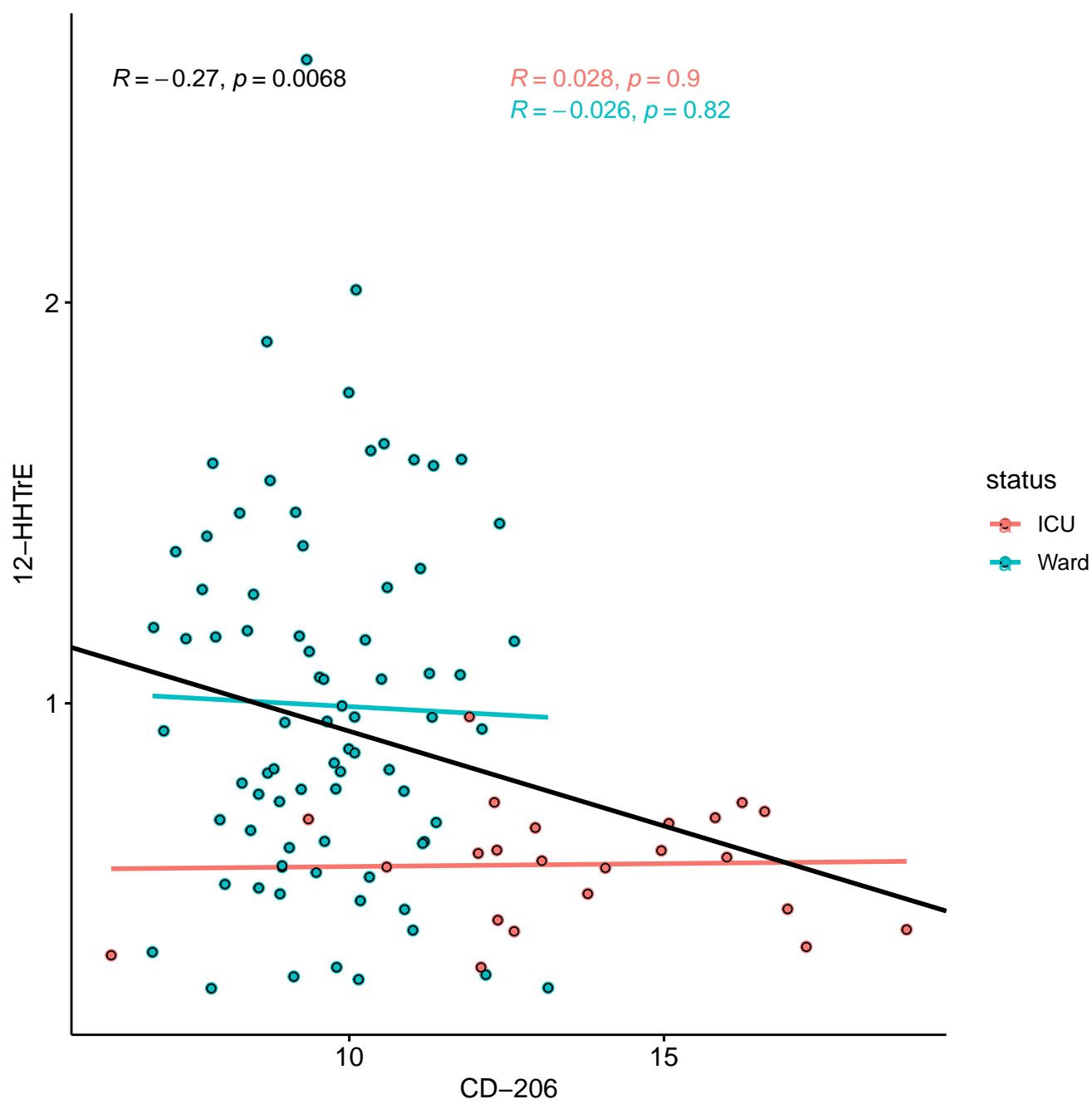

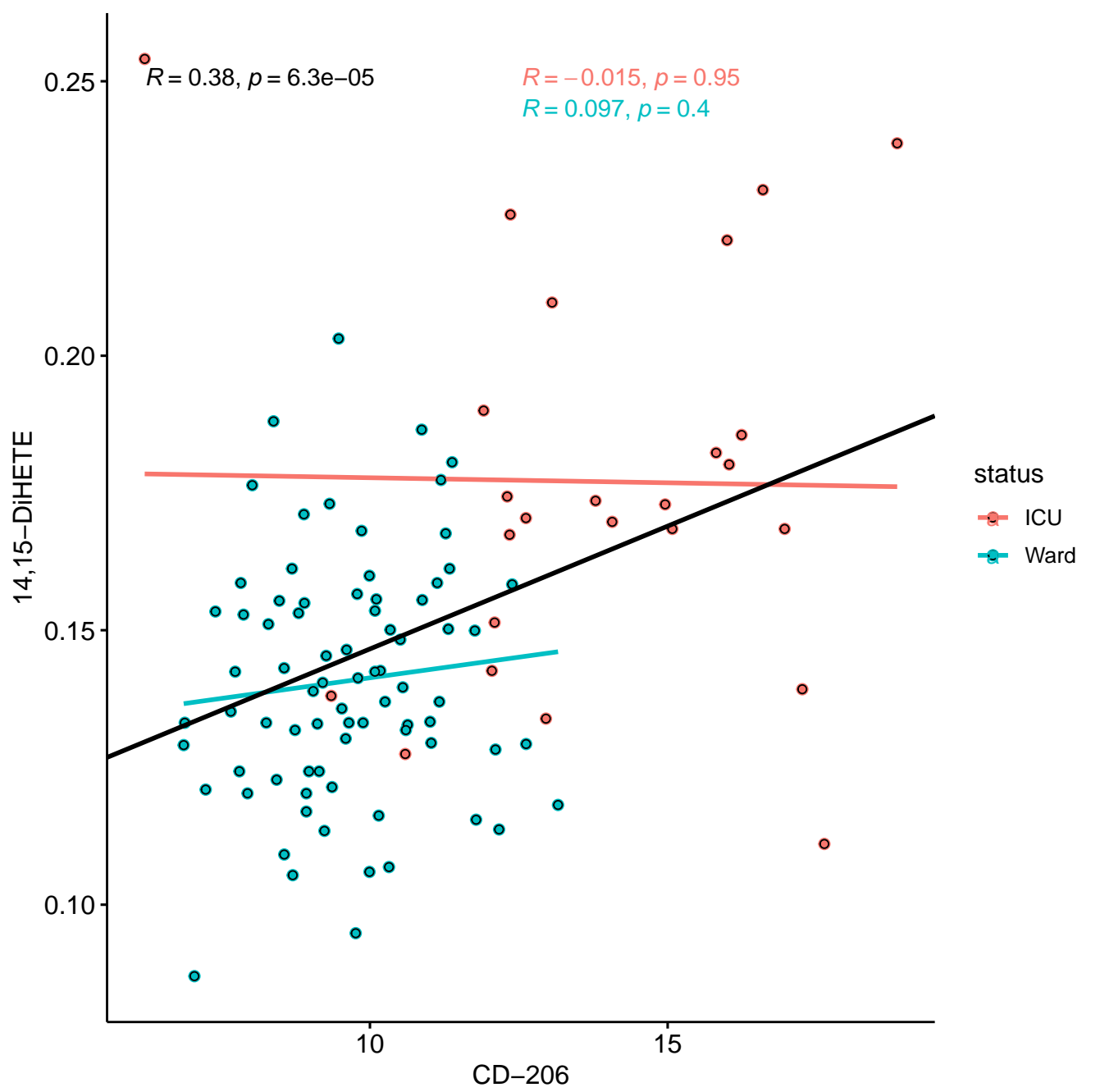

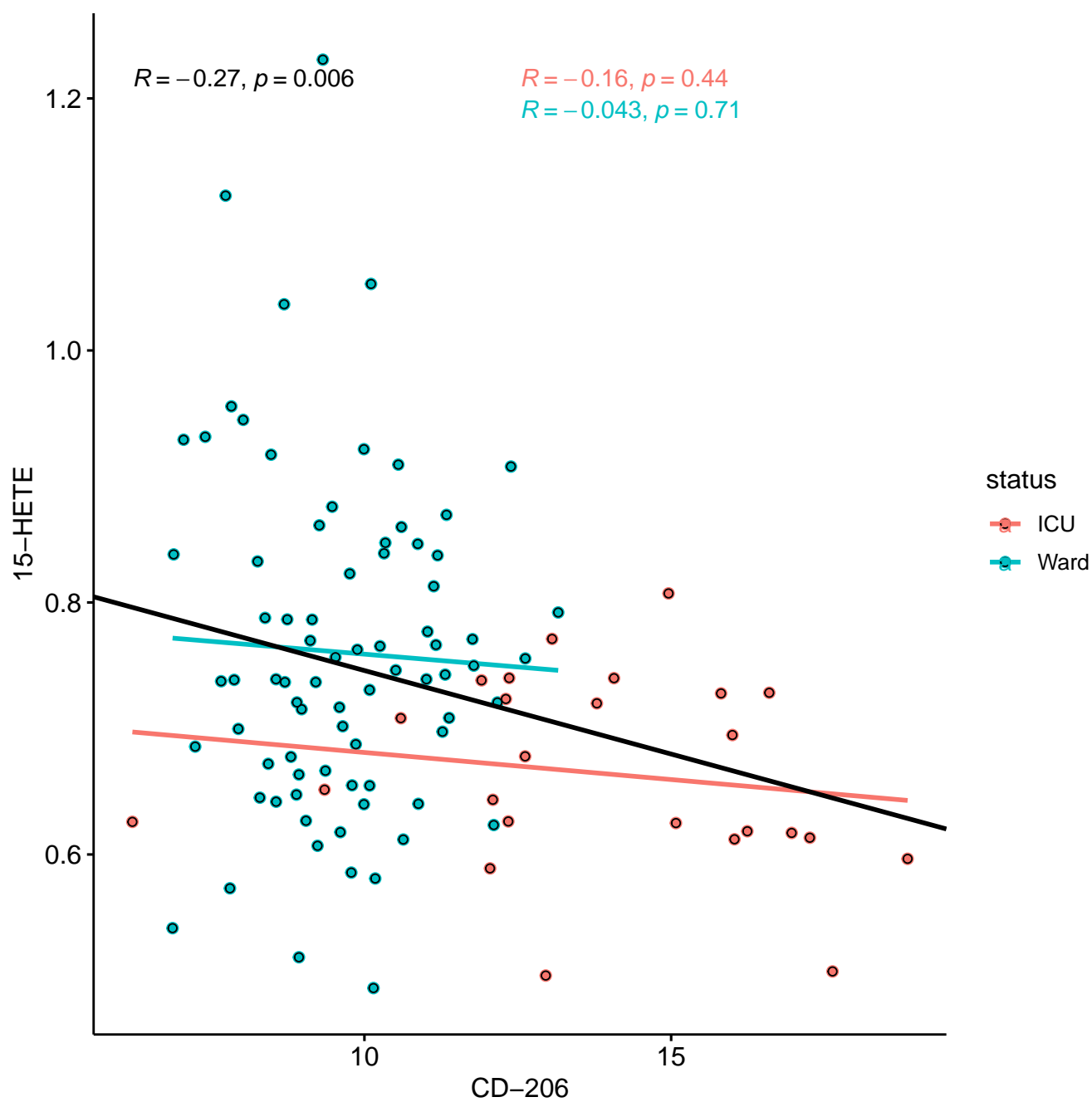

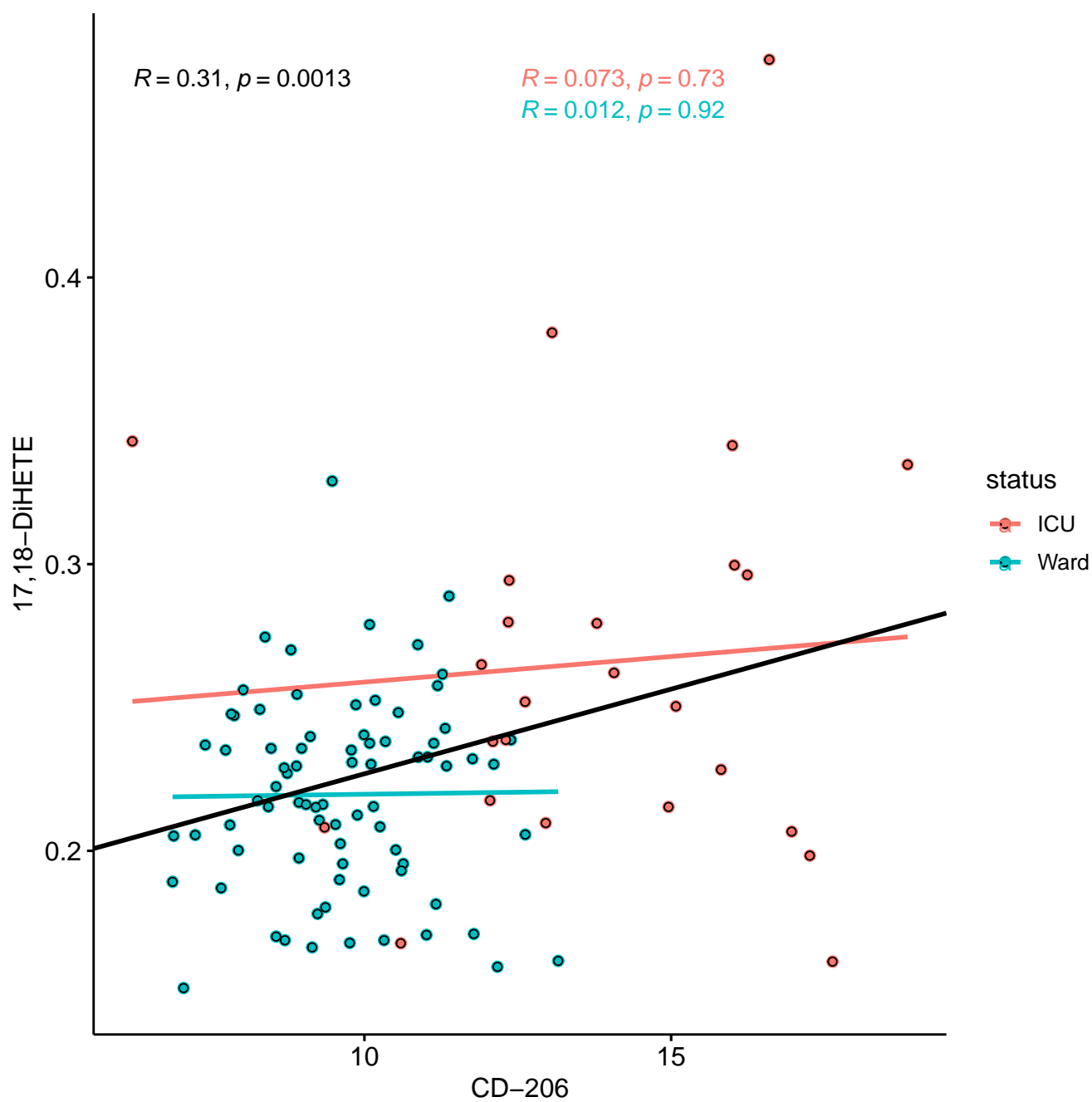

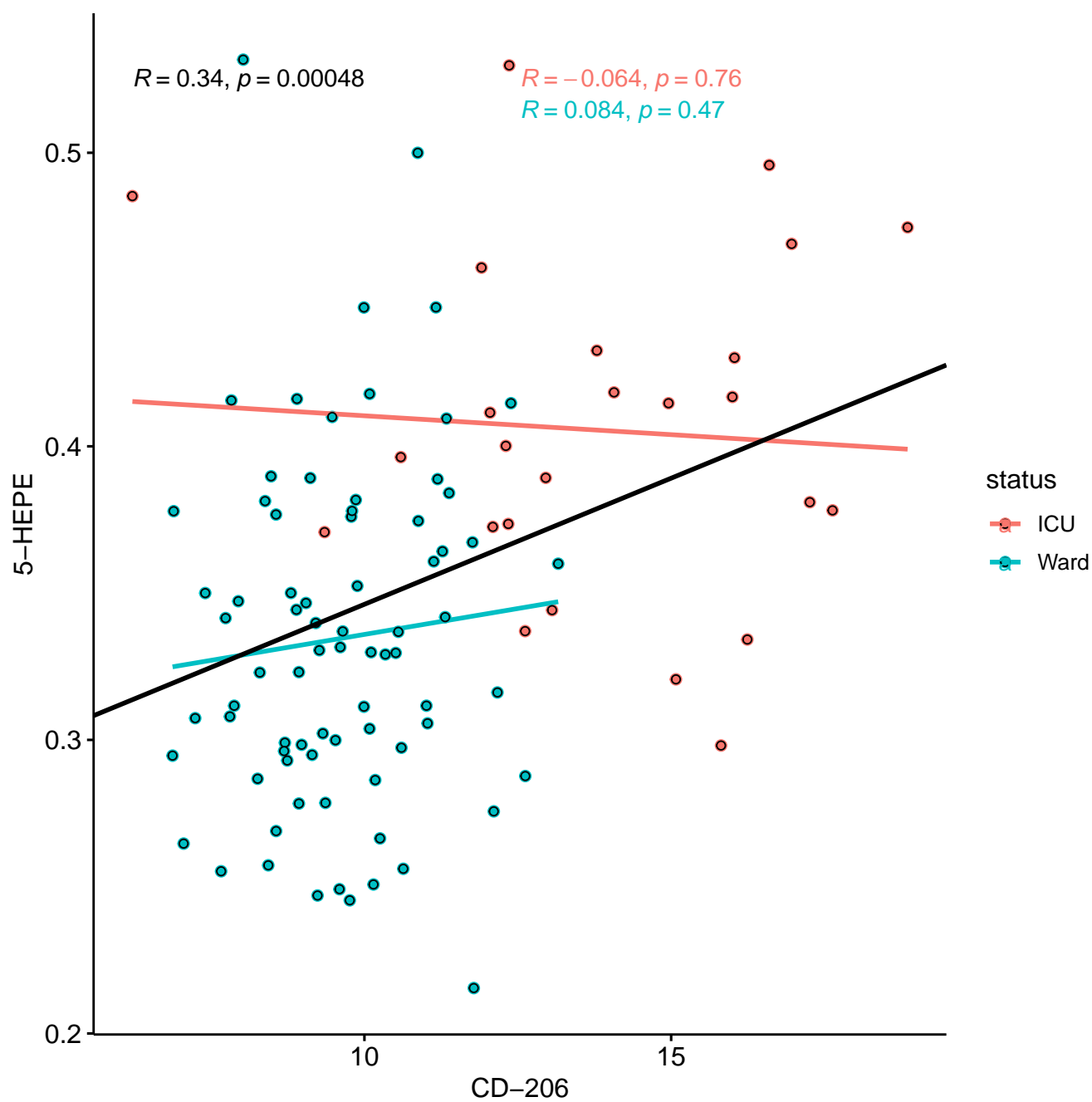

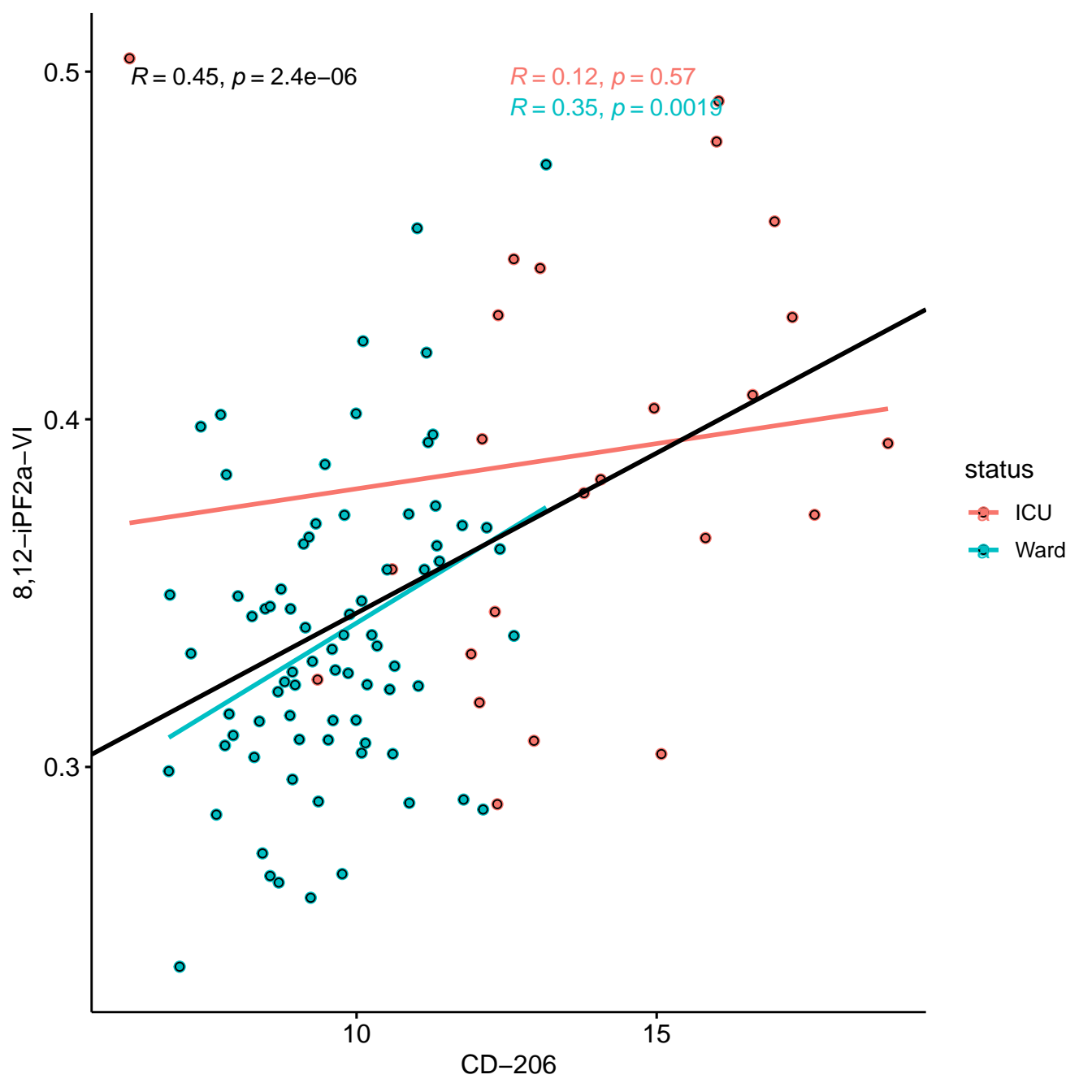

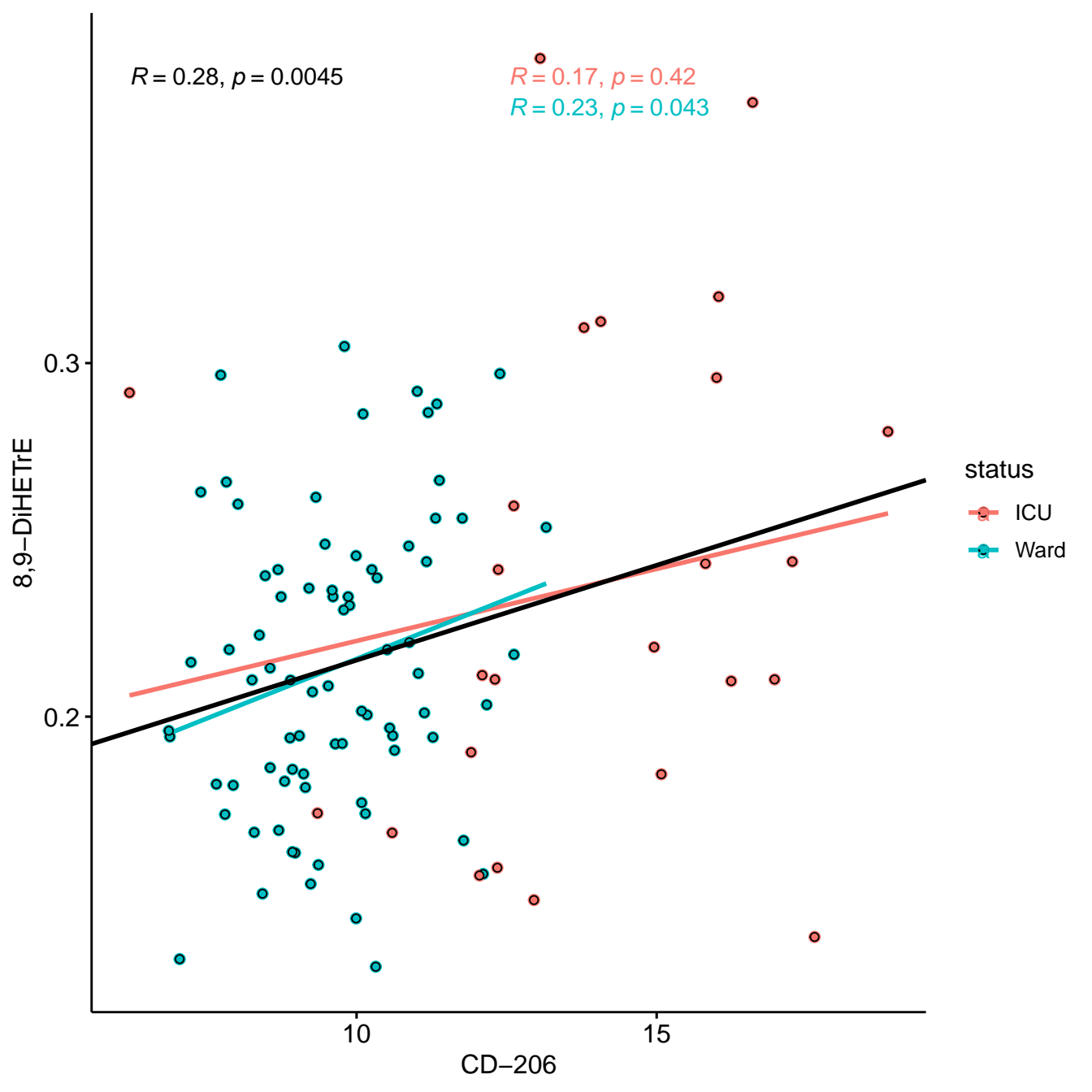

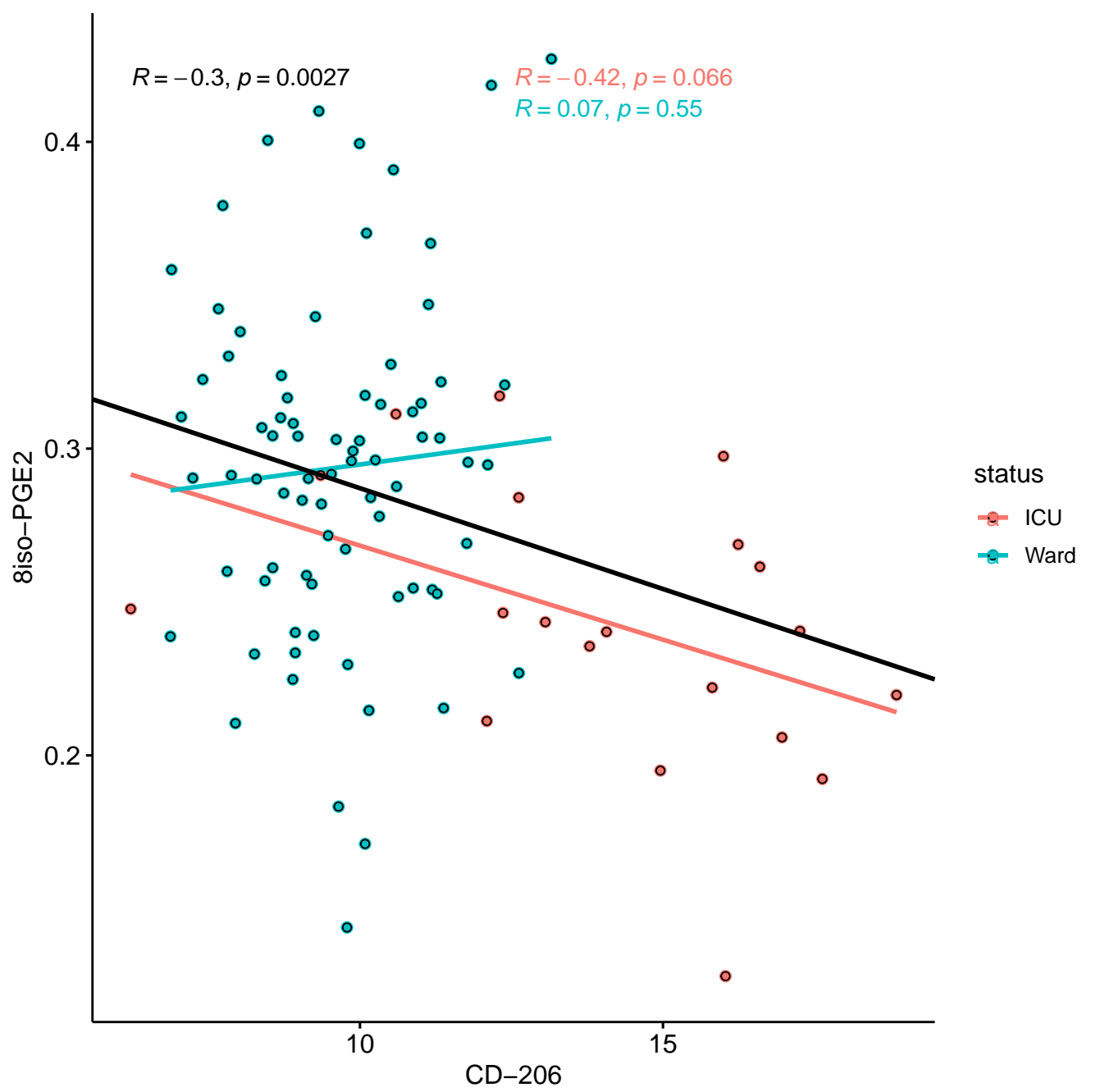

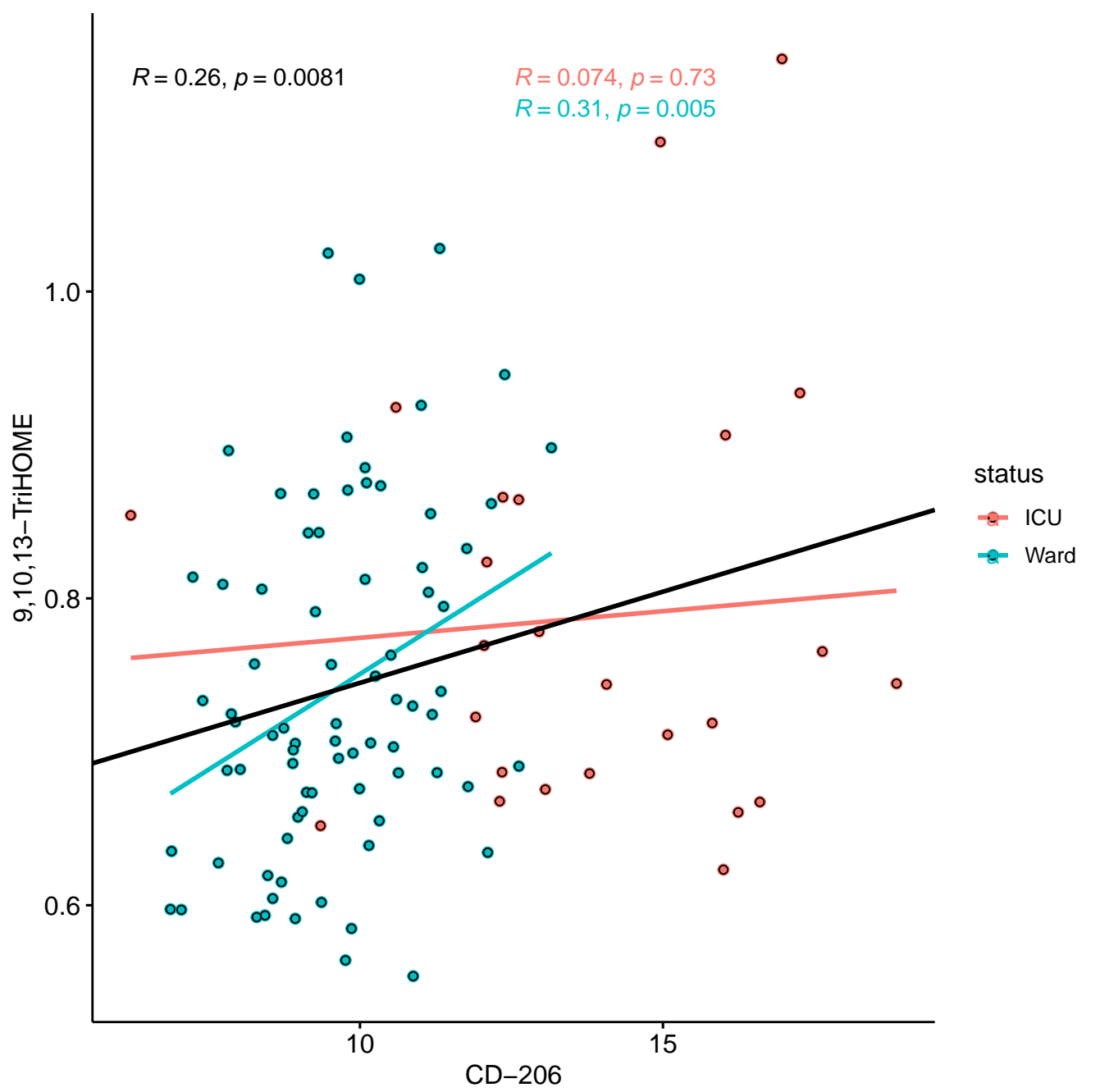

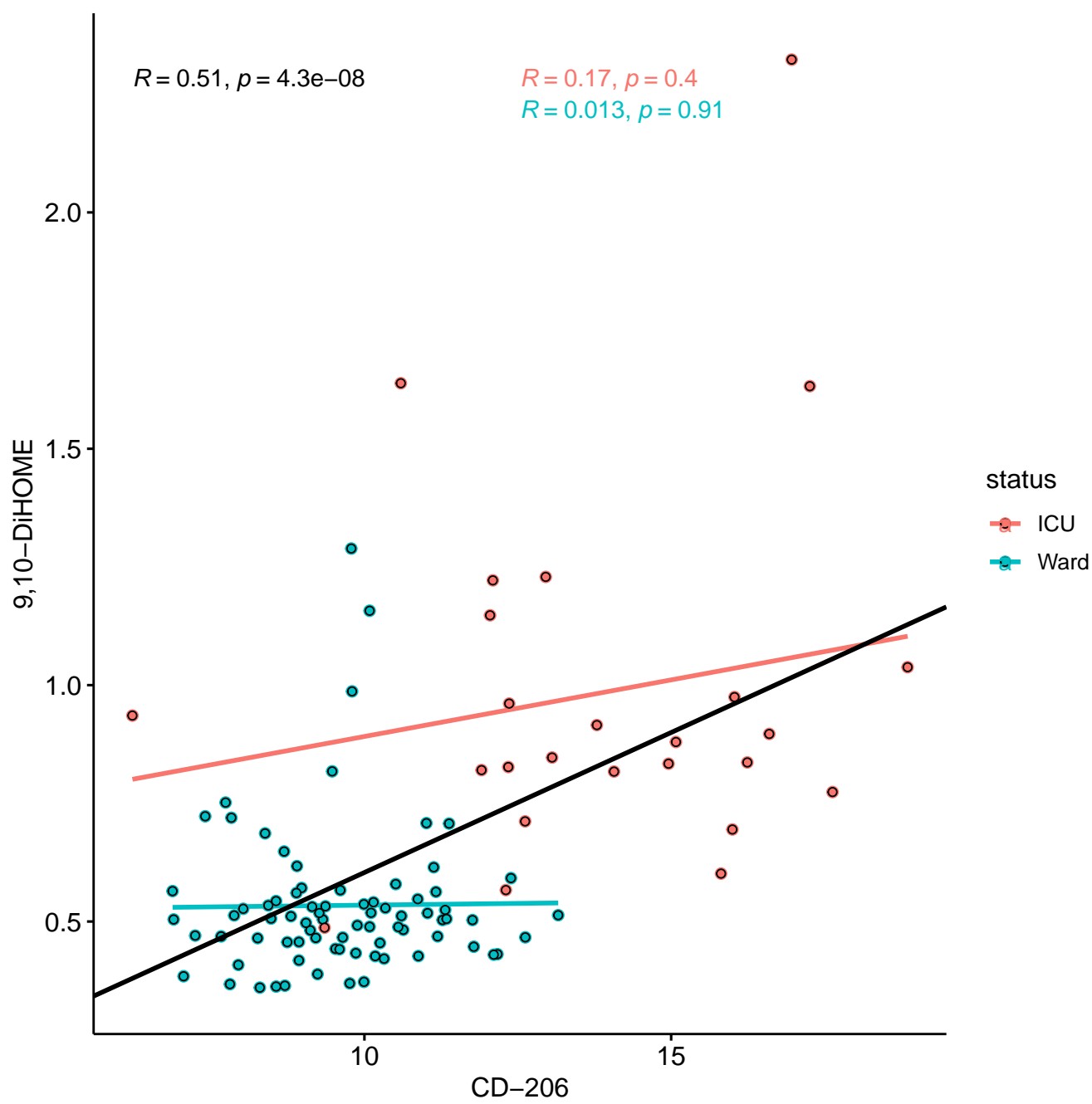

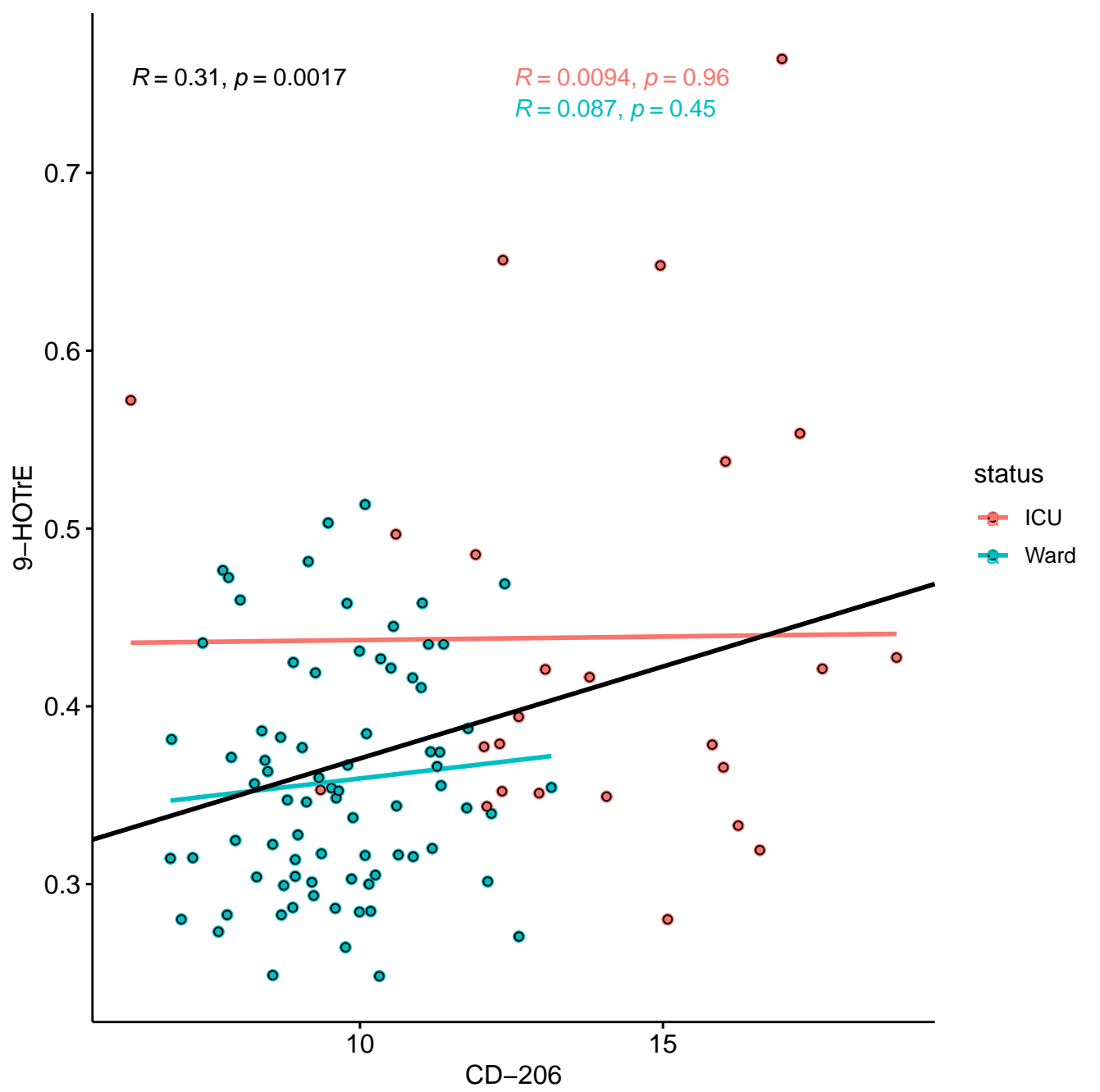

Supplement: Supplementary file 1 [file metabolites-12-00619-s001.zip › Document S4.pdf]
